# HIF-3*α*-Induced miR-630 Expression Promotes Cancer Hallmarks in Cervical Cancer Cells by Forming a Positive Feedback Loop

**DOI:** 10.1155/2022/5262963

**Published:** 2022-10-13

**Authors:** Qiaohui Gao, Zhenghua Ren, Shengyuan Jiao, Juan Guo, Xia Miao, Jin Wang, Junye Liu

**Affiliations:** Department of Radiation Medical Protection, School of Military Preventive Medicine, Air Force Medical University, Xi'an, China

## Abstract

**Purpose:**

Hypoxia has crucial functions in the development and metastasis of cervical cancer by inducing the expression of numerous genes, including microRNA genes. But we know little about how the hypoxia factors and microRNAs orchestrate to regulate hallmarks of cervical cancer cells.

**Methods:**

We conducted RNA sequencing (RNA-seq) and chromatin immunoprecipitation sequencing (ChIP-seq) experiments to investigate the targets of HIF-3*α* or miR-630. ChIP-qPCR and RT-qPCR were carried out to validate the results of ChIP-seq and RNA-seq. Cellular, molecular, and radiation experiments were conducted to explore the functions of miR-630.

**Results:**

In this study, we showed that hypoxia-induced overexpression of HIF-3*α* increased the expression of dozens of miRNAs, including miR-630. Hypoxia could also directly induce miR-630 expression. ChIP-seq data showed that HIF-3*α* activates miR-630 expression by directly binding to the promoter of its host gene. Meanwhile, stable overexpression of miR-630 increased the expression of HIF-3*α*, but repressed the expression of HIF-1*α*, indicating a positive feedback loop between HIF-3*α* and miR-630. Consequently, stable overexpression of miR-630 in HeLa cells promotes cancer hallmarks, including radioresistance, inhibition of apoptosis, increased migration and invasion, and EMT-mediated metastasis. Meanwhile, inhibition of miR-630 showed opposite features.

**Conclusion:**

Taken together, our findings indicate a novel hypoxia-induced HIF-3*α* and miR-630 regulatory feedback loop contributing to metastasis and progression of cervical cancer cells and suggest that HIF-3*α* and miR-630 might act as potential biomarkers and therapeutic targets for cervical cancer in the future.

## 1. Introduction

Cervical cancer (CC) is a common gynecological cancer that seriously threatens women's health worldwide. An estimated half a million newly diagnosed cases and over 300 000 deaths occur by cervical cancer each year [1, 2]. CC is mainly caused by the infection of high-risk subtypes of the human papilloma virus (HPV). Advances in radiotherapy technology have resulted in less treatment-related toxicity for women with cervical cancer [3]. Meanwhile, tumor recurrence and metastasis may occur to approximately one-third of cervical cancer patients within the first 2 years after radiotherapy [4]. Extrinsic abnormalities of tumor microenvironment, particularly tumor hypoxia, reduce the efficacy of radiotherapy, shorten survival time, and increase recurrences [5]. A recent review article summarized hypoxia-targeted strategies and identified further research and new treatment paradigms needed to improve patient outcomes [6]. Hypoxia-inducible factor (HIF) is a transcriptional activator of various genes related to cellular adaptive responses to hypoxia. To date, three HIF family members have been identified in mammals (HIF-1*α*, HIF-2*α*, and HIF-3*α*). HIF-1*α*-targeted genes are most significantly associated with metabolism of carbohydrates, diabetes pathways, pathways in cancer, and integration of energy metabolism [7]. HIF-2*α* overexpression in CC mouse model promoted tumor growth and reduced cisplatin sensitivity by inducing excessive autophagy [8]. Upregulation of HIF-3*α* can accelerate the progression of ovarian cancer and promote metastatic phenotypes in pancreatic cancer [9]. However, it remains largely unknown how HIF-3*α* functions in cervical cancer.

Recent studies demonstrate that miRNAs play an important role in modulating the process of epithelial mesenchymal transformation (EMT) [10]. For example, miR-27b could induce EMT and promote cervical cancer metastasis [11]. Our previous data indicated that a specific miRNA signature, including miR-630, miR-1246, miR-1290, and miR-3138, could promote radioresistance of human cervical cancer cells [12]. miR-630, identified from the miRNA cluster at chromosome 15q24.1, has been found to be deregulated and involved in several human malignancies [13]. Further study is needed to clarify the mechanisms of miR-630 in cancer development and progression.

In the current study, we investigated the regulatory loop among HIFs (HIF-1*α* and HIF-3*α*) and miR-630 to identify their functions in cervical cancer progression. Firstly, we confirmed that miR-630 is upregulated by HIF-3*α* instead of by HIF-1*α*. Then, we explored the relationship between hypoxia and radiation through the overexpression and repression of miR-630. At last, we found that HIF-1*α* can be regulated by HIF-3*α* and that miR-630 could increase HIF-3*α* expression but repress HIF-1*α* expression. Cellular phenotype experiments also demonstrated the important functions of miR-630 in the cancer hallmarks of HeLa cells. Our results identified a positive feedback loop between hypoxia factor HIF-3*α* and miRNA miR-630 and illustrated the underlying mechanisms. We also identified the functional role of miR-630 in regulating radioresistance and cancer hallmarks in cervical cancer cells. The discovery highlights the regulatory functions of HIF-3*α* and miR-630 and identifies new therapeutic candidates for hypoxia and radiotherapy strategies to treat cervical cancer.

## 2. Methods

### 2.1. Cell Culture

HeLa cells were purchased from the Institute of Biochemistry and Cell Biology, Chinese Academy of Sciences, in 2018. HeLa cells were maintained as a monolayer in Dulbecco's modified Eagle's medium (DMEM, Invitrogen, Carlsbad, CA) supplemented with 10% fetal bovine serum (FBS, Sijiqing Biological Engineering Materials Co., Hangzhou, China) at 37°C in the presence of 5% CO_2_-balanced air. The other three stable transformants were maintained as monolayers in DMEM with 10% FBS and 2 *μ*g/ml puromycin (Sigma, German).

### 2.2. Hypoxia

To induce hypoxia, HeLa cell was rendered in a chamber with a gas mixture of 1% O_2_- and 5% CO_2_-balanced N_2_ at 37°C. The level of oxygen in the chamber was verified using a gas monitor (SKC, Inc., Eighty Four, PA). To mimic hypoxia using chemicals, cells were cultured under 20% oxygen in the presence or absence of 100 *μ*M CoCl_2_ for a specified time period.

### 2.3. Plasmid Construction and Transfection

Full-length human *HIF3A* cDNA was cloned into a Pcmv-ORF-flag-his expression vector and *HIF1A* was cloned into a pBabe-puro-HA expression vector (TranSheepBio, Shanghai, China). Transfection was performed with Lipofectamine 2000 (Invitrogen, Carlsbad, CA, USA) 24 h after cell seeding. Lentiviral constructs containing upregulating miR-630 (LV-hsa-mir-630), inhibiting miR-630 (LV-hsa-mir-630-inhibitor), and the negative control lenti-vector (LV-negative control) were designed and provided by Genechem Inc. (Shanghai, China). HeLa cell at 60–70% confluence was infected with three lentiviruses at a multiplicity of infection (MOI) of 10 with enhanced infection solution (ENI.S) according to the manufacturer's protocol. At 10 hours postinfection, viruses were replaced by complete DMEM, and at 48 hours postinfection, three stably infected cells (LV-hsa-mir-630, LV-hsa-mir-630-inhibitor, and LV-negative control) were selected by DMEM with 2 *μ*g/ml puromycin (Sigma, Germany).

### 2.4. Real-Time Quantitative PCR

Total RNA was extracted using TRIzol reagent (Invitrogen, Carlsbad, CA), and reverse transcription was performed according to the manufacturer's instructions (638315, Clontech Laboratories, Inc. A Takara Biocompany, USA). U6 was used as an internal control, and each primer contains one cDNA sample as a correct sample in each plate. Primers are shown in Table S1.

### 2.5. Transwell Chamber Assay

The invasive ability of cells was assessed in 24-well transwell chambers (Corning, NY). The polycarbonate filters containing 8 *μ*m pores were coated on ice with 80 *μ*l of Matrigel (Sigma-Aldrich) at 5 mg/l. After blocking with 1% BSA for 1 h at 37°C, the cells (5 × 10^5^/ml) were suspended in serum-free culture medium, and then, 100 *μ*l of medium was added to the upper compartments of chamber. In each lower chamber, 500 *μ*l of medium (10% FBS) was added. After 24 h incubation, the cells from the upper compartment were removed, and the cells on the lower surface were fixed in ethanol and stained with hematoxylin-eosin. Cells on the lower surface were quantified in 10 random microscopic fields per filter at a magnification of 200x (DMI4000B, LEICA, Germany).

### 2.6. Western Blot Analysis

When 80% confluence in 25 cm^2^ flasks (Nunc, Roskilde, Denmark) was reached, the cells were lysed with RIPA lysis buffer (Beyotime, Shanghai, China) on ice and then centrifuged at 12000 rpm for 20 min. Supernatants were collected and protein was determined using bicinchoninic acid (BCA) kit (Boster, Wuhan, China). The extracts (20 *μ*g per lane) were fractionated by 10% SDS-PAGE and then transferred onto PVDF membranes (0.45 *μ*m; Millipore, Bedford, MA). After blocking with TBST buffer (20 mM Tris-buffered saline and 0.1% Tween-20) containing 5% nonfat milk for 1 h at 25°C, the membranes were incubated with primary antibodies against E-cadherin, N-cadherin (1 : 5000, Epitomics, USA), cytokeratin (1 : 400, Boster, China), EP300 (1 : 1000, Abnova, USA), and *β*-actin (1 : 5000, CMCTAG, USA) overnight at 4°C. Membranes were washed three times for 5 min with TBST before incubation with horseradish peroxidase-conjugated secondary antibody (1 : 3000, CST, USA) for 60 min at 25°C. The membranes were exposed by chemiluminescence (Millipore, Billerica, MA), and images were acquired by ChemiDoc XRS (Bio-Rad, Hercules, CA). Semiquantification of scanned films was performed using Quantity One-4.6.2 (Bio-Rad).

### 2.7. Immunofluorescence

Cells were grown on 24-well m-Slides (NEST Biotechnology Co. Ltd. Wuxi, China) and fixed with 4% paraformaldehyde for 30 min at 4°C, followed by treatment with 0.1% Triton for 10 min. The samples were blocked with PBST buffer (0.1% Tween-20) containing 10% goat serum at room temperature for 1 h and incubated with primary antibodies E-cadherin, N-cadherin (1 : 500, Epitomics, USA), and cytokeratin (1 : 200, Boster, China), overnight at 4°C. The cells then were washed in PBST and incubated with DyLight 488- and DyLight 594-conjugated secondary antibodies (ZS-Bio) at room temperature in the dark for 1 h. Nuclei were counterstained with DAPI for 5 min. After being washed three times, the cells were maintained with 50% glycerin in PBS and observed by laser confocal microscopy (Fluoview FV1000; Olympus, Tokyo, Japan). Photographs were taken with a digital camera (Olympus Fluoview FV1000) attached to a microscope. Ten images (approximately 30 cells per field) were acquired in each group, and the quantification of gray value was analyzed with Olympus Fluoview software FV10-ASW 1.7.

### 2.8. RNA-seq Library Construction and Sequencing

Total RNA (5 *μ*g) was used for RNA-seq library preparation. Polyadenylated mRNAs were purified and concentrated with oligo (dT)-conjugated magnetic beads (Invitrogen) before directional RNA-seq library preparation. Purified mRNAs were iron-fragmented at 95°C followed by end repair and 5′ adaptor ligation. Then, reverse transcription was performed with RT primer harboring 3′ adaptor sequence and randomized hexamer. PCR products corresponding to 200-500 bps were purified, quantified, and stored at -80°C for sequencing. The libraries were prepared and applied to Illumina NextSeq 500 system with 150 × 2 paired-end type (ABLife Inc., Wuhan, China).

### 2.9. miRNA-seq Library Construction and Sequencing

Total RNA (3 *μ*g) was used for small RNA cDNA library preparation with Balancer NGS Library Preparation Kit for small/microRNA (Genome Gen). Briefly, RNAs were ligated to 3′ and 5′ adaptor sequentially and reversely transcribed to cDNA and then amplified by PCR. Whole library was applied to 10% native PAGE gel electrophoresis, and bands corresponding to microRNA insertion were cut and eluted. The purified small RNA libraries were quantified and stored at -80°C. The libraries were prepared following the manufacturer's instructions and applied to Illumina NextSeq 500 system with 150 × 2 paired-end type (ABLife Inc., Wuhan, China).

### 2.10. Chromatin Immunoprecipitation (ChIP) Library Construction and Sequencing

Total cell extracts were prepared from 2 × 10^7^ formaldehyde-fixed cells resuspended in 1 ml lysis buffer containing 50 mM Tris 7.4, 150 mM NaCl, 2 mM EDTA, 0.1% SDS, 0.5% NP-40, and 0.5% deoxycholate. The suspension was sonicated to generate DNA fragments of 200-500 bp and centrifuged for 10 min at 12000 g. Then, 1000 *μ*l cell extracts were incubated with HIF-3*α* antibody (orb101652, Biorbyt, China) overnight at 4°C. The detailed steps of ChIP experiment were according to the published method [14]. Purified DNA fragments were end-repaired, adenylated, ligated to adaptors, and amplified by PCR for 12 cycles. The PCR products corresponding to 300-500 bps were gel purified, quantified, and stored at -80°C. The libraries were prepared and applied to Illumina X-Ten system with 150 × 2 paired-end type by Novogene.

### 2.11. RNA-seq Data Processing and Alignment

Raw reads containing more than 2-N bases were first discarded. Then, adaptors and low-quality bases were trimmed from raw sequencing reads using FASTX-Toolkit (Version 0.0.13). The short reads less than 16 nt were also dropped. Subsequently, clean reads were aligned to the GRCh38 genome by TopHat2 [15], allowing 4 mismatches. Uniquely mapped reads were used to calculate reads number and FPKM values (fragments per kilobase of transcript per million fragments mapped) [16] for each gene.

### 2.12. Differentially Expressed Gene Analysis

The R Bioconductor package edgeR [17] was utilized to screen the differentially expressed genes (DEGs). A false discovery rate < 0.05 and fold change > 2 or <0.5 were set as the cut-off criteria.

To predict the gene function and calculate the functional category distribution frequency, Gene Ontology (GO) analysis and enriched KEGG pathways were conducted using KOBAS 2.0 server [18]. Hypergeometric test and Benjamini-Hochberg FDR controlling procedure were used to define the enrichment of each pathway (corrected *p* value < 0.05).

### 2.13. Statistical Analysis

All the statistics were expressed as mean ± standard deviation (SD) and processed using SPSS 16.0 statistical software (Chicago, IL). All experiments were performed in duplicate, and *p* < 0.05 was considered significant.

## 3. Results

### 3.1. HIF-3*α* Participates in the Hypoxia-Induced Activation of miR-630 Transcription

**Figure 1:**
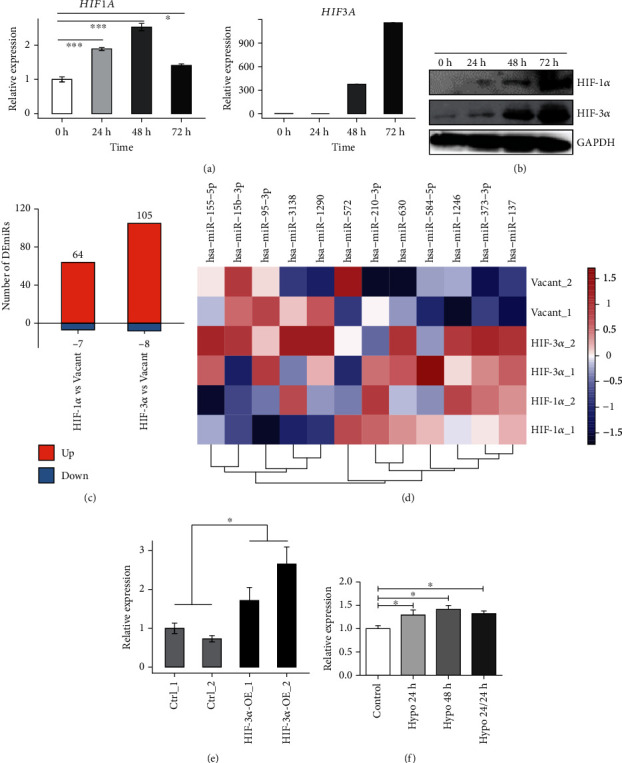
HIF-1*α* and HIF-3*α* promote miR-630 expression under hypoxia. (a) Bar plot showed the increased RNA level of HIF1A and HIF3A under hypoxia by RT-qPCR experiment. (b) Western blot result showed the increased protein level of HIF1A and HIF3A under hypoxia. (c) Bar plot showed the DEmiR number in HIF-1*α* vs. in the vacant group and in HIF-3*α* vs. in the vacant group. (d) Hierarchical clustering heat map showed the elevated expression level of selected miRNAs in HIF-1*α* and HIF-3*α* OE samples. (e) Bar plot showed the increased expression level of miR-630 under hypoxia by RT-qPCR experiment. (f) Bar plot showed the expression level of miR-630 under hypoxia by RT-qPCR experiment.

Previous study has demonstrated that the expression pattern of miRNAs was regulated by hypoxia [19]. According to the established work, the hypoxia-inducible factors, including HIF-1*α*, HIF-2*α*, and HIF-3*α*, are regulators of oxygen homeostasis [20]. We selected the well-studied HIF-1*α* and poorly studied HIF-3*α* and confirmed their elevated expression level in HeLa cells cultured under hypoxia (Figures 1(a) and 1(b)). Consistent with previous study [9], the expression of HIF-3*α* was stimulated to a higher level than that of HIF-1*α* (Figures 1(a) and 1(b)). Reanalysis of TCGA cervical cancer revealed their converse expression pattern between normal and tumor samples (Figure S1A). Higher expression of *HIF1A* and *HIF3A* could shorten the survival time of cervical cancer patients (Figure S1B), indicating their oncogenic functions.

We then investigated if the expression of miRNAs was also regulated by HIF-3*α*, as was the case with HIF-1*α*. We utilized the HIF-1*α* or HIF-3*α* overexpression (OE) and vacant HeLa cells to generate miRNA expression profile by miRNA-seq. Differentially expressed miRNA (DEmiR) analysis revealed that the upregulated miRNAs were dominant in both HIF-1*α* and HIF-3*α* overexpression samples (Figure 1(c)). Meanwhile, we analyzed the expression of miRNAs that are involved in radioresistance in cervical cancer cells [12]. The expression of miR-630 was increased in both HIF-1*α* and HIF-3*α* OE samples, especially in the HIF-3*α* OE samples (Figure 1(d)). In addition to miR-630, miR-137 was significantly upregulated in HIF-3*α* overexpression HeLa cells. miR-1246 and miR-137 were significantly upregulated in HIF-1*α* overexpression cells, whereas miR-15b-3p was significantly downregulated in HIF-1*α* overexpression cells (Figure 1(d)). RT-qPCR also showed that HIF-3*α* increased the expression level of miR-630, which was consistent with RNA-seq results (Figure 1(e)).

We then measured miR-630 expression level in HeLa cells which were cultured under hypoxia for 24 h and 48 h, respectively. We found that miR-630 was upregulated in response to hypoxia. After 24 h exposure to hypoxia, HeLa cells were incubated in normoxia for another 24 h. We found that the miR-630 level was still higher than that of the control group (Figure 1(f)). These results show that the activation of miR-630 induced by hypoxia is fast and long-lasting in HeLa cells. Together with the induced expression of HIF-3*α* under hypoxia, these results demonstrate that hypoxia promotes miR-630 expression by activating HIF-3*α* in HeLa cells.

### 3.2. The Cancer Cell Transcriptome Changes upon HIF-3*α* Overexpression

**Figure 2:**
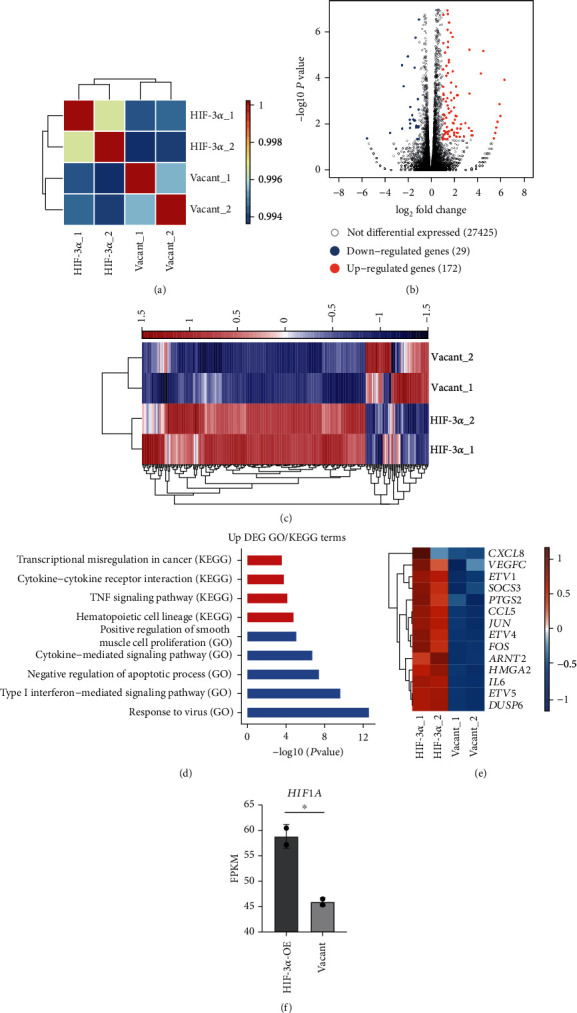
HIF-3*α* overexpression globally regulates gene expression in HeLa cells. (a) Sample correlation results showed a clear separation between HIF-3*α* OE and vacant samples. (b) Volcano plot showed the DEG results of HIF-3*α* OE and vacant samples. (c) Hierarchical clustering heat map showed the dominant upregulated genes after HIF-3*α* OE. (d) Top functionally enriched GO BP terms and KEGG pathways for HIF-3*α* upregulated genes. (e) Heat map showed the upregulated genes by HIF-3*α* OE. (f) Bar plot showed the expression levels of *HIF1A* transcripts upon HIF3A overexpression and repression.

To further explore the functions of targets regulated by HIF-3*α*, we performed HIF-3*α* overexpression and silencing experiments and utilized transcriptome profiling of HIF-3*α* OE and negative control samples by RNA-seq in HeLa cells. Sample correlation analysis revealed the global alteration of transcriptome by HIF-3*α* overexpression (Figure 2(a)). We finally obtained a total of 201 DEGs, including 172 up- and 29 downregulated genes (Figures 2(b) and 2(c)). GO and KEGG pathway analysis for the upregulated genes by HIF-3*α* OE was conducted. The upregulated target genes are enriched in terms including responses to virus, type I interferon-mediated signaling pathway, and negative regulation of apoptosis. Enriched KEGG pathways included hematopoietic cell lineage, TNF signaling pathway, cytokine-cytokine receptor interaction, and transcriptional misregulation in cancer (Figure 2(d)). These data indicated that HIF-3*α* can upregulate the transcriptional levels of cancer-related genes, including *ETV1*, *ETV5*, *ETV4*, *CXCL8*, *HMGA2*, *IL6*, *DUSP6*, *ARNT2*, *PTGS2*, *SOCS3*, *CCL5*, *FOS*, *JUN*, *IL6*, and *VEGFC* (Figure 2(e)). Immune- and inflammatory-related terms were also significantly enriched (Figure 2(d)). HIF-3*α* overexpression significantly increased the RNA level of *HIF1A* (Figure 2(f)), indicating that HIF-3*α* positively regulated *HIF1A* expression.

### 3.3. HIF-3*α* Globally Binds to the Promoters of miR-630 and HIF1A and Partial miRNAs, mRNAs, and lncRNAs

**Figure 3:**
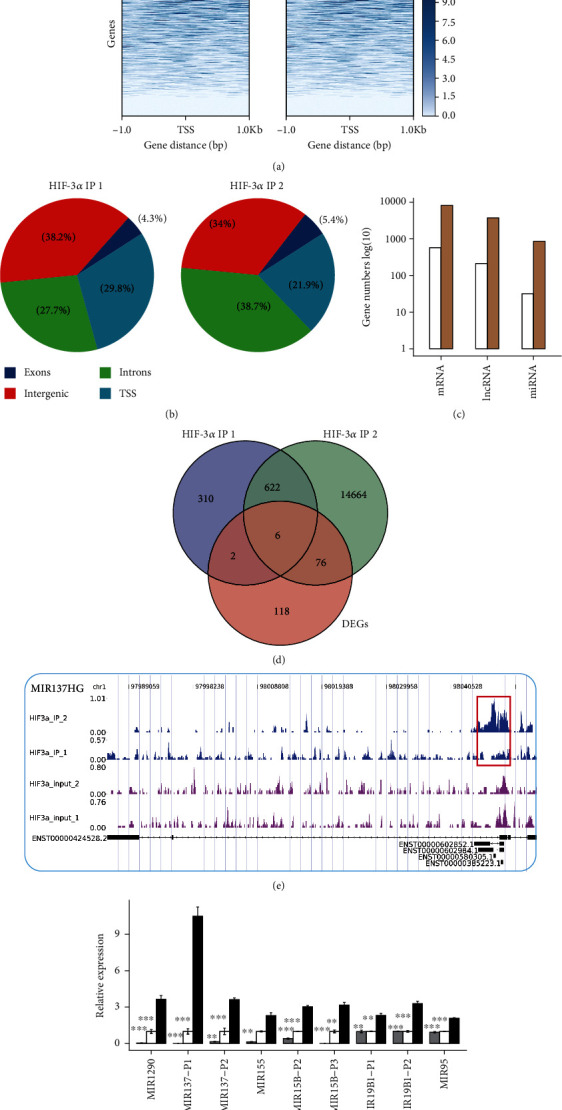
ChIP-seq results of HIF-3*α* showed its binding preference at promoter region. (a) Read density heat map plot showed the enriched read distribution around gene TSS sites. (b) Pie chart showed the percentage of peaks from four genomic regions. (c) Bar plot showed the percentage of bound genes classified by their coding types. The white and brown bars represent two biological replicates of HIF-3*α* ChIP-seq data. (d) Venn diagram showed HIF-3*α*-bound genes and HIF-3*α*-regulated DEGs. (e) Read distribution of bound miRNA miR-137. Red rectangle represents the bound region of HIF-3*α*. (f) Bar plot showed the validation results of ChIP-qPCR experiments.

To decipher how HIF-3*α* regulated the expression of their target genes, we performed ChIP-seq experiment. After aligning the quality-filtered reads to the human genome, we detected the binding peaks using MACS2 software [21]. We then assessed the read distribution around the transcription start sites (TSS) of all genes. We observed that ChIP-seq tags were obviously enriched in the TSS region of HIF3A (Figure 3(a)), suggesting that HIF-3*α* binds to the promoter region to regulate gene expression. By classifying the HIF3A-bound peaks according to their genomic distribution, approximately 21~30% peaks were located at the TSS region of genes (within 2 kb to TSS, Figure 3(b)). We also found that HIF3A globally bound to the promoters of miRNAs, mRNAs, and lncRNAs (Figure 3(c)). The bound miRNAs of the first and second replicates of HIF-3*α* accounted for a proportion of 9% and 12%, respectively. We then assessed how HIF-3*α* regulated gene expression by directly binding to their genomic locus. A total of 41.8% (84) HIF-3*α*-regulated DEGs were bound by HIF-3*α* (Figure 3(d)), among which, miR-630 and miR-137 were bound by HIF-3*α* at the promoter region of their host genes, with miR-137 shown in Figure 3(e) as an example. To verify the ChIP-seq results, ChIP-qPCR experiment was conducted for selected miRNAs (Figure 3(f)), which confirmed that miR-155, miR-158, miR-95, miR-1290, and miR-137 were directly bound by HIF-3*α*.

### 3.4. miR-630 Overexpression Increased the HIF-3*α* Activation and Suppressed HIF-1*α* Activation

**Figure 4:**
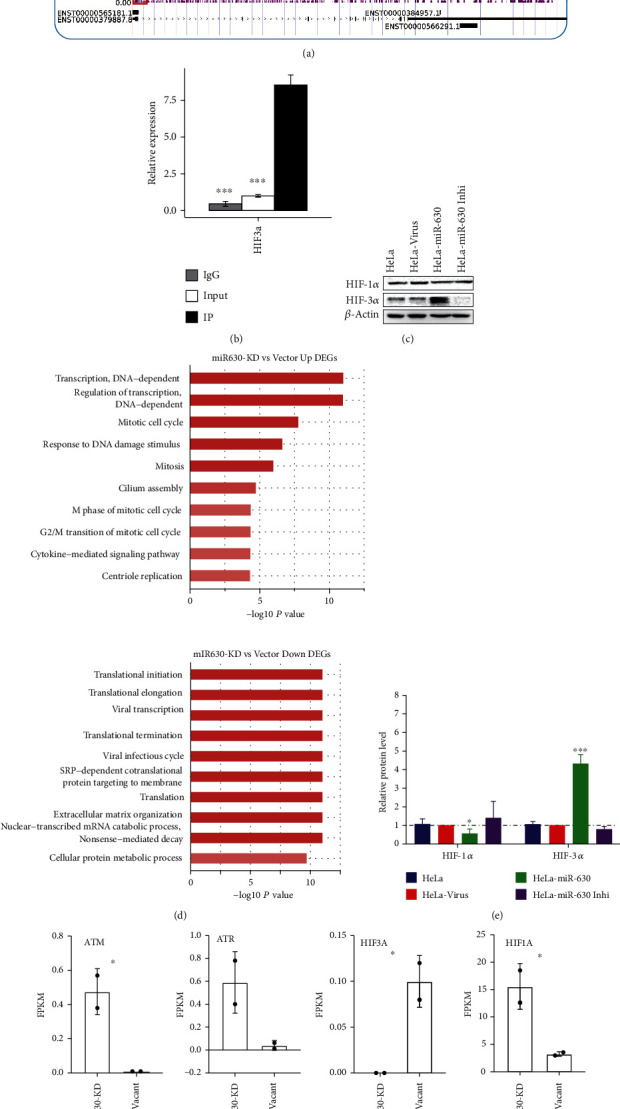
RNA-seq revealed the functions of miR-630 targets in HeLa cells. (a) Read distribution plot showed the significant HIF3A binding density in the promoter region of miR-630 host gene. Red frame represented the HIF3A binding peak. (b) Bar plot showed the ChIP-qPCR results of the HIF-3a binding density in the promoter region of miR-630 host gene. (c) Western blot showed the expression changes of HIF1A and HIF3A upon miR-630 overexpression and knockdown. (d) Bar plot showed the quantified levels of WB results shown in (c). Three biological replicates were included in this panel. (e) Bar plot showed the enriched GO BP terms for upregulated genes (left) and downregulated genes (right) by miR-630 inhibition. (f) Bar plot showed the increased expression levels of ATM and ATR by miR-630 inhibition. (g) Bar plot showed the increased expression level of HIF1A and the decreased expression level of HIF3A by miR-630 inhibition.

miR-630 is an intronic miRNA that shares the same promoter with its host gene *ARIH1*. Sequencing reads with the HRE binding motif were enriched in the promoter of the *ARIH1* gene in HIF3A IP ChIP-seq samples (Figure 4(a)), which was validated by ChIP-qPCR (Figure 4(b)). Taken together, these results demonstrated that expression of miR-630 was activated by HIF-3*α* binding to its host gene promoter. Since radiation and hypoxia both induced the expression of miR-630 in cervical cancer cells, we investigated the potential relationships between miR-630 and HIFs. By checking the protein levels of HIF-1*α* and HIF-3*α*, we found that miR-630 could significantly reduce the expression of HIF-1*α* and increase the expression of HIF-3*α* (Figures 4(c) and 4(d)).

It is clear that miR-630 can promote cell proliferation and invasion, whereas the associated regulatory mechanism is unclear. We performed RNA-seq for miR-630 knockdown and vacant HeLa cells. A total of 8413 DEGs were detected, of which 5107 were upregulated and 3306 were downregulated (Table S2). Functional enrichment analysis of the DEGs (Figure 4(e)) showed that the upregulated genes were enriched in DNA-dependent transcription, regulation of DNA-dependent transcription, response to DNA damage stimulus, mitotic cell cycle, and homologous recombination-mediated double-strand break repair. The downregulated genes are enriched in mRNA metabolic process pathways, translation, and extracellular matrix organization (Figure 4(e)). We also found that the expressions of *ATM* and *ATR* genes, the master regulators of cell cycle checkpoint signaling pathways [22], were upregulated by silencing miR-630 in HeLa cells (Figure 4(f)). RNA-seq data also revealed miR-630 knockdown-induced downregulation of *HIF1A* (Figure 4(g)) and upregulation of *HIF-3α* in HeLa cells (Figure 4(g)).

### 3.5. miR-630 Promotes HeLa Cell Survival and Proliferation by Its Radioresistance Activity

**Figure 5:**
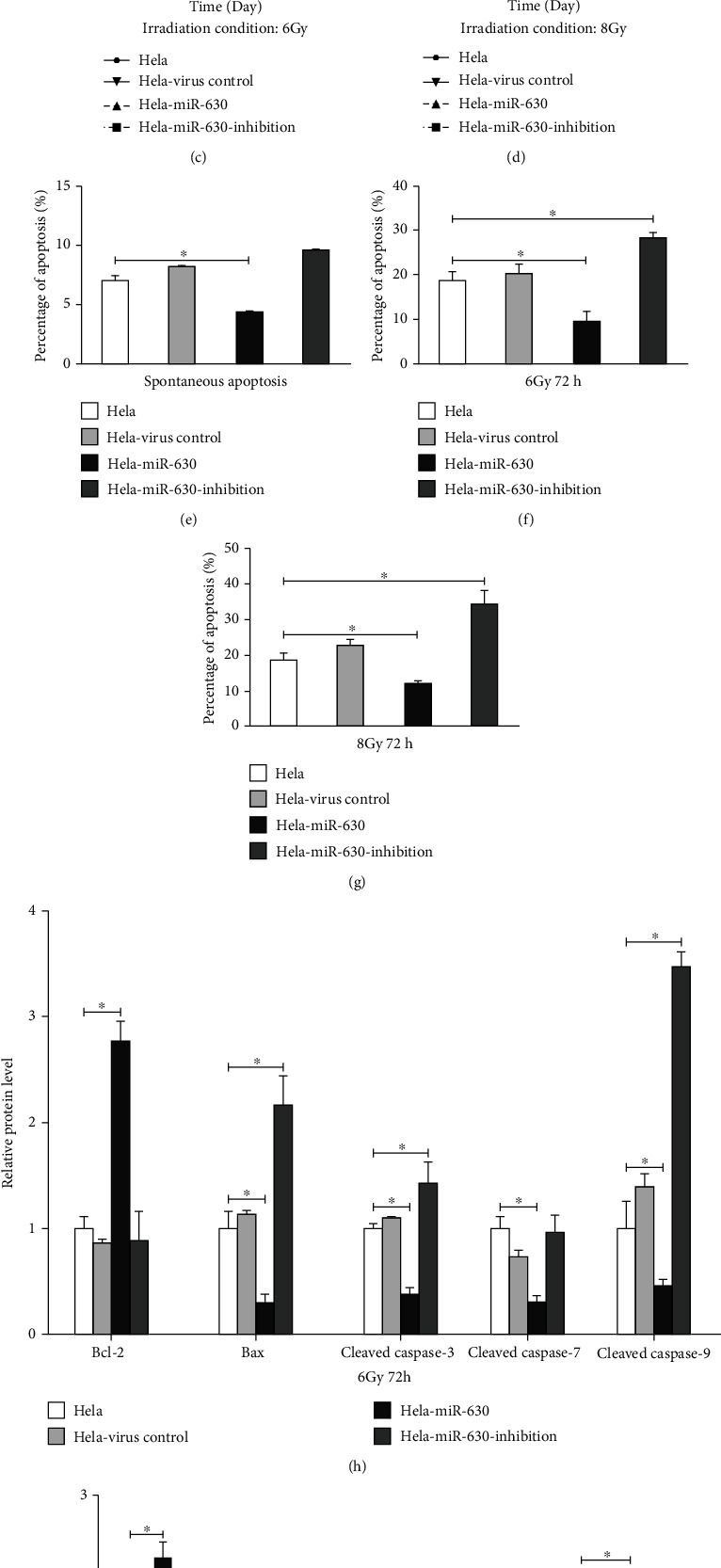
miR-630 increases the radioresistance and significantly reduces the apoptosis level of HeLa cells under spontaneous and radiation-treated conditions. (a) RT-qPCR analysis of lentiviral overexpression and inhibition of miR-630 in HeLa cell lines. (b) Colony formation assay showing the higher radioresistance by miR-630 overexpression and opposite phenotype by miR-630 inhibition. ^∗^*p* < 0.05. (c, d) Overexpression of miR-630 increased the cell proliferation rates of HeLa cells under irradiation conditions. (e) miR-630 overexpression reduced the spontaneous apoptosis levels of HeLa cells. (f, g) miR-630 overexpression reduced the radiation-induced apoptosis levels of HeLa cells. (h) Western blotting showing the expression changes of several apoptosis markers in HeLa cells with miR-630 overexpression or inhibition under radiation-treated conditions. (i) Western blotting showing the expression changes of several apoptosis markers in HeLa cells with miR-630 overexpression or inhibition under spontaneous conditions.

To explore the cellular influence of miR-630 on cervical cancer cells, we established cervical cancer HeLa cells with stably overexpressed and inhibited miR-630 level by lentivirus transfection (Figure 5(a)). Our previous study showed that radiation induced the expression of miR-630 in a time- and dose-dependent manner in cervical cancer cells [12]. Colony formation assay was used to analyze the survival rate of HeLa cells in single-dose radiotherapy (0 Gy, 4 Gy, 8 Gy, and 10 Gy). We found that overexpression of miR-630 attenuated radiotherapy-induced apoptosis of HeLa cells, whereas silencing of miR-630 accelerated radiotherapy-induced apoptosis (Figure 5(b)), indicating the radioresistant function of miR-630. To further explore the functions of miR-630, we measured cell proliferation after 6 Gy and 8 Gy radiotherapy. Overexpression of miR-630 had a positive effect on the proliferation capacity of HeLa cells. By contrast, silencing of miR-630 resulted in decreased proliferation (Figures 5(c) and 5(d)). Taken together, these results demonstrated that miR-630 enhanced the radioresistance and increased proliferation of HeLa cells.

### 3.6. miR-630 Inhibits Both Spontaneous and Radiation-Induced Apoptosis of HeLa Cells

We then conducted flow cytometry analysis to investigate the effect of miR-630 on apoptosis level in HeLa cells in cervical cancer. We found that overexpressing miR-630 inhibited spontaneous apoptosis of HeLa cells (*p* < 0.05) and that silencing miR-630 had no influence on spontaneous apoptosis of HeLa cells (*p* > 0.05) (Figure 5(e)). Furthermore, when HeLa cells were treated with 6 Gy and 8 Gy doses of radiotherapy, we found that overexpressing miR-630 suppressed the apoptosis of HeLa cells *in vitro* (*p* < 0.05) (Figures 5(f) and 5(g)) and that silencing miR-630 promoted the apoptosis of HeLa cells *in vitro* (*p* < 0.05) (Figures 5(f) and 5(g)). Subsequently, we detected the apoptosis proteins such as BCL2, BAX, Caspase 3, Caspase 7, and Caspase 9 in 6 Gy HeLa cells. We found that the expression of BCL-2 was increased, whereas the expression of BAX was decreased (Figure 5(h)). Caspase 3, Caspase 7, and Caspase 9 were all significantly decreased upon miR-630 overexpression (Figure 5(i)). Taken together, these results demonstrated that miR-630 inhibited spontaneous and radiation-induced apoptosis of HeLa cells *in vitro*.

### 3.7. miR-630 Enhances HeLa Cell Migration and Invasion In Vitro

**Figure 6:**
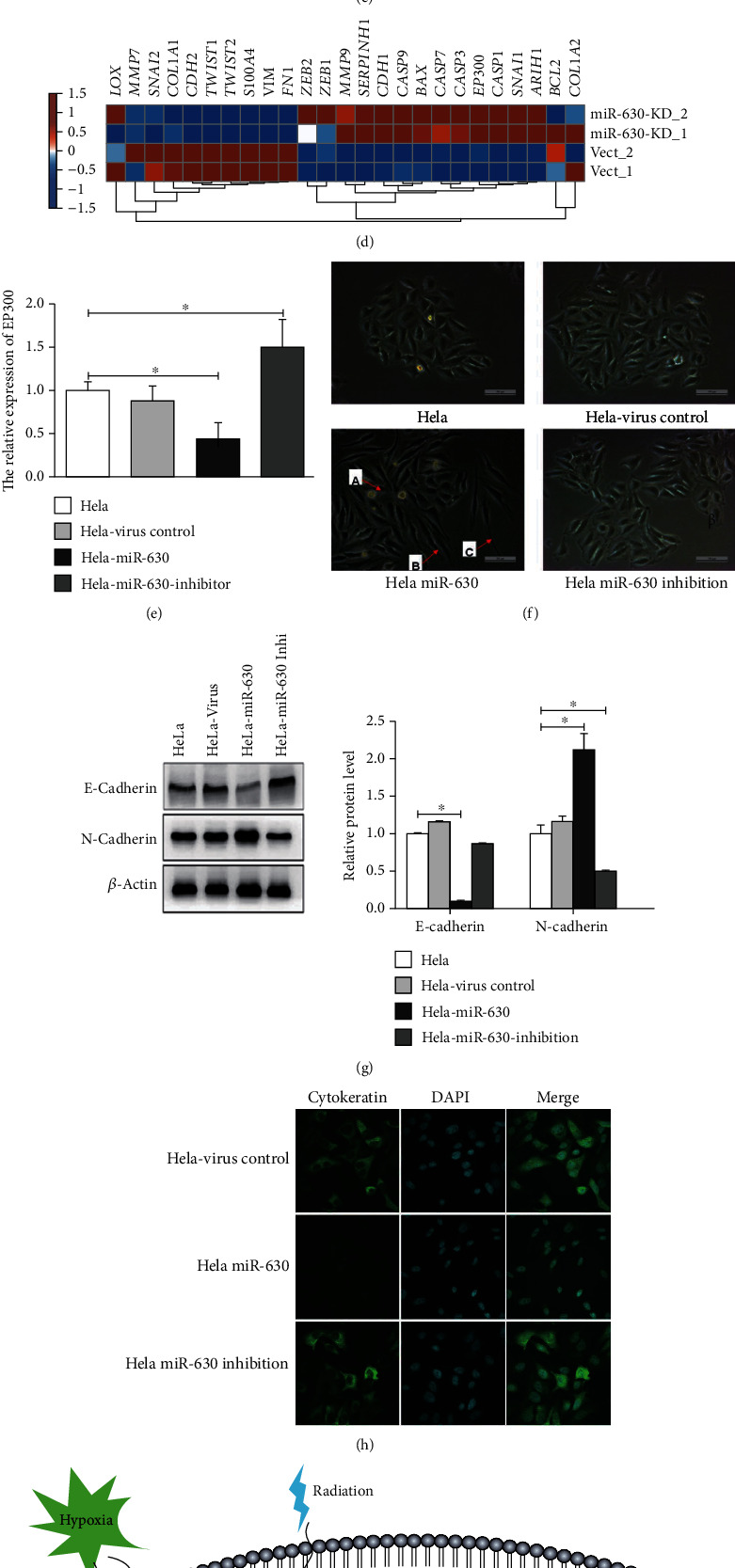
Effects of miR-630 on migration and invasion of HeLa cell line and miR-630 promoted HeLa metastasis is mediated by the EMT. (a) Wound-healing assay was performed to compare the migratory capabilities of HeLa, HeLa-virus control, and HeLa-miR-630-inhibitor cells. ^∗^*p* < 0.05. The graph shows the quantification of migration rates analyzed in HeLa, HeLa-virus control, HeLa-miR-630, and HeLa-miR-630-inhibitor cells, respectively. (b, c) Representative invasion images of HeLa, HeLa-virus control, HeLa-miR-630, and HeLa-miR-630-inhibitor cells by transwell invasion assay. The right graph shows the quantification of invasion numbers analyzed in four cells. (d) Heat map plot showed the expression level changes of miR-630 targets that are transcription factors related to EMT. (e) Bar plot showed the RT-qPCR results of transcription factor EP300 upon miR-630 overexpression and knockdown. (f) The morphology of HeLa, HeLa-virus control, HeLa-miR-630, and HeLa-miR-630-inhibitor cells. Note: (A) cell space became wide; (B) spindle-cell morphology formed; (C) pseudopodia were stretched out. Scale bar = 100. (g) WB was performed to analyze the expression of EMT makers. (h) Cellular staining showed the cytokeratin distribution in the HeLa cells. (i) Working model showed the HIF3A-miR-630 regulatory loop in HeLa cells.

To determine the role of miR-630 in regulating migration and invasion of HeLa cells, we carried out wound-healing assay and transwell chamber assay (Figures 6(a) and 6(c), Figure S2A). Wound-healing assay indicated that overexpression of miR-630 enhanced HeLa cell migration (*p* < 0.05), while the relative migration distance of miR-630-inhibited cells was significantly decreased (*p* < 0.05) (Figure 6(a) and Figure S2A). Furthermore, the transwell assay showed that the invasion rate was significantly increased (*p* < 0.05) (Figures 6(b) and 6(c)). In contrast, silencing miR-630 significantly inhibited the invasion (*p* < 0.05) (Figure 6(c)). Real-time cellular analysis (RTCA) revealed the significantly higher motility of HeLa cells upon miR-630 overexpression (Figure S2B, *p* = 0, K-S test). In conclusion, overexpression of miR-630 significantly enhanced the migration and invasion capacity of cervical cancer *in vitro*.

### 3.8. miR-630 Could Promote HeLa Cell Metastasis and EMT

Based on the above-mentioned RNA-seq results, we noted that the EMT-related transcription factors SNAI1, ZEB1, and ZEB2 were upregulated, whereas TWIST1 and SMAI2 were downregulated (Figure 6(d)). We also found that *EP300*, a metastasis suppressor gene and a direct target of miR-630 [23], was one of the upregulated DEGs by miR-630 inhibition (Figure 6(d)). *EP300* was upregulated upon miR-630 silencing and downregulated upon miR-630 overexpression (Figure 6(e)). These results suggested that miR-630 may promote EMT in HeLa cells. To further confirm the functions of miR-630 in EMT, we performed cellular morphological change experiment. We found that miR-630-induced morphological changes in HeLa cells were consistent with EMT treatment (Figure 6(f)). We checked the expression changes of canonical EMT markers, including E-cadherin and N-cadherin. We found that overexpression of miR-630 increased the expression of N-cadherin and decreased the expression of E-cadherin compared with the control group (*p* < 0.05) (Figures 6(g) and 6(h)). These results suggest that miR-630 can promote HeLa metastasis by EMT in cervical cancer cells. The induction of HIF-3*α* under hypoxia increases the transcription of miR-630, and miR-630 overexpression also has positive impact on HIF-3*α* expression. In conclusion, we described a positive regulatory loop between miR-630 and HIF-3*α*. Our results also showed that overexpression of miR-630 could significantly reduce the apoptosis and increase the proliferation, invasion, metastasis, and EMT in HeLa cells (Figure 6(i)).

## 4. Discussion

In our study, we described a regulatory loop between HIF-3*α* and miR-630 (Figure 6(i)). Overexpression of miR-630 increases the expression of HIF-3*α*, but decreases the expression of HIF-1*α*. Overexpression of HIF-3*α* increases the expression of miR-630. The cellular functions of miR-630 were extensively investigated to support its carcinogenic function. We also found that HIF-3*α* cooperates in the expression of cancer cell transcriptome. These results demonstrated that the novel HIF-3*α*-miR-630 axis promotes the development of HeLa cell at multiple aspects. Several miRNAs have been found to be related to radioresistance in cancers, such as miRNA-668 in breast cancer [24] and miR-125 in cervical cancer [25]. Our previous study demonstrated that overexpression of miR-630 in HeLa cells resulted in radioresistance [12]. In this study, we further showed that overexpression of miR-630 significantly promoted the migration, invasion, and EMT-mediated metastasis of HeLa cells and inhibited the apoptosis. Several studies have demonstrated the bidirectional functions of miR-630. On the bright side, miR-630, as a tumor suppressor, inhibited tumor progression [26]. Simultaneously, miR-630, as an oncogene, promoted tumor progression, consequently resulting in poor prognosis [27]. Yuan-Yuan Lyu reported that miR-630 acts as a tumor suppressor and inhibits EMT in cervical cancer [28]. We conjectured that one miRNA may target a set of mRNAs and affect radiosensitivity [29].

The mechanisms of HIF-1*α* have been extensively studied in multiple cancer types [30]. However, the functional studies focusing on HIF-3*α* only emerged in recent years. HIF-3*α* was considered to play a negative role in gene expression by competing with HIF-1*α* and HIF-2*α* via binding to transcriptional elements in target genes during hypoxia [31]. HIF-3*α* acts as a transcription activator in zebrafish, the expression of which is oxygen-dependent [32]. According to our study, HIF-3*α*, induced by hypoxia, increased the expression of multiple miRNAs, consistent with the master regulator of hypoxia in microRNA biogenesis and activity [19]. We found that HIF-3*α* activates the expression of miRNA by directly binding to their promoter region. Transcriptome sequencing suggested the transcriptional activation role of HIF-3*α*. Upregulating HIF-3*α* can greatly activate the cancer development-related genes. Bioinformatics analysis of genes indicated that HIF-3*α* and HIF-1*α* regulate the common biological processes.

We focused on miR-630, a HIF-3*α*-activated miRNA, to explore its cellular functions. miR-630, upregulated under hypoxia, increased tumor growth and metastasis by being delivered into a model of ovarian cancer in mouse [33]. Overexpression of miR-630 could also enhance HIF-3*α* expression, forming a positive feedback loop. Several reports have indicated how hypoxia-induced miRNAs regulated the switch between HIF-1*α* and HIF-3*α* in human endothelial cells [34] and how their regulatory feedback circuit enhanced tumor metastasis in hepatocellular carcinoma [35]. We found that HIF-3*α* binds to miR-630 promoter region and activates its expression. Meanwhile, miR-630 overexpression enhances the expression level of HIF-3*α*, but represses HIF-1*α* level. Given that HIF-3*α* and miR-630 were both consistently upregulated by hypoxia, we proposed that they may cofunctionally regulate the cellular processes of HeLa cells under hypoxia. Our results also highlighted the prognosis effects of HIF-1*α* and HIF-3*α* on CC patients. Studies are needed to further explore the contribution of miR-630 to prognosis by regulating the expression of HIF-1*α* and HIF-3*α* and by modulating cancer hallmarks of HeLa cells.

Taken together, our results for the first time revealed the mechanisms of HIF-3*α*. We extensively investigated the cellular functions of HIF-3*α*-induced microRNA miR-630 and detected the HIF-3*α*/miR-630-positive loop to regulate multiple cellular processes. The potential miR-630-mediated radioresistance can provide useful information on how to treat miR-630-mediated resistance to radiotherapy and hypoxia in the near future. The newly identified functions of HIF-3*α* and its regulatory loop with miR-630 in HeLa cells also provide theoretical basis for future clinical prognosis and treatment of cervical cancer.

## Data Availability

The datasets used and/or analyzed during the current study are available from the corresponding author upon reasonable request.

## Abbreviations

ChIP: Chromatin immunoprecipitation
DEG: Differentially expressed gene
EMT: Epithelial-mesenchymal transition
HIF: Hypoxia-inducible factor
RT-qPCR: Reverse transcription quantitative polymerase chain reaction.

## Supplementary Materials

Supplementary Materials Figure S1: the expression level of HIF1A and HIF3A and their related survival time in cervical cancer patients and normal specimens. Figure S2: miR-630 enhances HeLa cell migration and invasion *in vitro*. Table S1: PCR primer table in this study. Table S2: differentially expressed genes (DEGs) between miR-630 and the control group.
