# Supplementary material for: HIF-3α-Induced miR-630 Expression Promotes Cancer Hallmarks in Cervical Cancer Cells by Forming a Positive Feedback Loop
Source: J Immunol Res. 2022 Oct 13;2022:5262963. doi: 10.1155/2022/5262963 (PMC9584697; doi:10.1155/2022/5262963)
Supplement: Supplementary Materials — Figure S1: the expression level of HIF1A and HIF3A and their related survival time in cervical cancer patients and normal specimens. Figure S2: miR-630 enhances HeLa cell migration and invasion in vitro. Table S1: PCR primer table in this study. Table S2: differentially expressed genes (DEGs) between miR-630 and the control group. [file 5262963.f1.zip › Table S1.docx]

Table S1. PCR Primer table in this study.

| **Gene Name** | **Forward** | **Reverse** |
| --- | --- | --- |
| ***HIF1A*** | GAAGTGTACCCTAACTAGCCG | GTTCACAAATCAGCACCAAGC |
| ***HIF3A*** | CTCATCTGCGAAGCCATCCCC | TCTGCAATCCTGTCGTCACAGTAGG |
| **miR-630** | ACACTCCAGCTGGGAGTATTCTGTACCA | TGGTGTCGTGGAGTCG |
| **miR-1290** | GCACTCAACAGCAGACACC | TGGCAAGCCCTCAGGAACT |
| **miR-137P1** | CTCTCTTGCTCCCACGCCTT | GTCTTCCCTGCCTCTGCCTTTA |
| **miR-137P2** | ACACAGGACATCCATAGACAA | AAACTCGGGAGGACAGCA |
| **miR-155** | CGTCATTTGAAGGCGTTTCC | GTGGGTCATTGCGTGTGT |
| **miR-15b-P2** | CGACTTGAAGTTGCTAGTGGTT | GTGAAGACTAACTGGCGACTC |
| **miR-15b-P3** | CCACAGCACGGAAACACAG | CGGTCTGGGCACCTGAAG |
| **miR-19b1-P1** | CGCCTTCGCGCCACTTCG | AGGAGAGCTTCGCGGAGGA |
| **miR-19b1-P2** | CCCTCTGGGCCGGGCTCG | CCGAGTGCGGCGGGGACG |
| **miR-95** | CACCCACCTGCACTCATCTCA | CGGGCGGGCGGAGGATAG |
| ***EP300*** | GGCGAATTTGTGCTCTTGTG | CAGTGGCTGGAGAGGGATG |
| ***U6*** | CTCGCTTCGGCAGCACA | AACGCTTCACGAATTTGCGT |
