# Supplementary material for: HIF-3α-Induced miR-630 Expression Promotes Cancer Hallmarks in Cervical Cancer Cells by Forming a Positive Feedback Loop
Source: J Immunol Res. 2022 Oct 13;2022:5262963. doi: 10.1155/2022/5262963 (PMC9584697; doi:10.1155/2022/5262963)
Supplement: Supplementary Materials — Figure S1: the expression level of HIF1A and HIF3A and their related survival time in cervical cancer patients and normal specimens. Figure S2: miR-630 enhances HeLa cell migration and invasion in vitro. Table S1: PCR primer table in this study. Table S2: differentially expressed genes (DEGs) between miR-630 and the control group. [file 5262963.f1.zip › Table S2.pdf]

Table S2. Differentially expressed genes (DEGs) between miR-630 and the control group

Gene	logFC	logCPM	PValue	FDR	Vect_1	Vect_2	miR630_1	miR630_2	Symbol	Description	Chromosome	Genetype
ENSG00000257527.1	-1.6063261	0.66845222	9.49E-06	4.63E-05	3.2	3.81	1.24	1.81	MIR3179-3	microRNA 3179-3	chr16	lincRNA
ENSG00000101310.13	1.10234956	5.20131879	0.00014732	0.00057331	5.23	4.96	12.99	16.26	SEC23B	Sec23 homolog B (S. cerevisiae)	chr20	protein_coding
ENSG00000108309.11	1.73178534	-0.4213583	0.00020204	0.00076235	0.04	0.16	0.59	0.32	RUNDC3A	RUN domain containing 3A	chr17	protein_coding
ENSG00000057704.9	-2.0733457	-1.8928472	0.00303791	0.00892022	0.04	0.06	0.02	0.01	TMCC3	transmembrane and coiled-coil domain family 3	chr12	protein_coding
ENSG00000185963.12	-1.0175381	5.86513658	0.00042081	0.00149293	10.38	9.26	5.88	7.08	BICD2	bicaudal D homolog 2 (Drosophila)	chr9	protein_coding
ENSG00000114698.13	2.46415426	-0.2891022	2.04E-07	1.31E-06	0.05	0.05	0.23	0.5	PLSCR4	phospholipid scramblase 4	chr3	protein_coding
ENSG00000249199.1	-5.837457	-0.918486	2.49E-14	3.72E-13	2.06	3.58	0	0.1	CTD-2139B1	--	chr5	lincRNA
ENSG00000173480.9	1.78940751	0.10587979	1.71E-05	7.97E-05	0.08	0.12	0.45	0.47	ZNF417	zinc finger protein 417	chr19	protein_coding
ENSG00000129515.17	1.30220869	2.91197562	2.12E-05	9.68E-05	0.96	1.05	2.31	4.31	SNX6	sorting nexin 6	chr14	protein_coding
ENSG00000248787.1	-2.949478	-2.0750183	0.00016274	0.00062431	1	0.27	0	0.19	RP11-666A2	--	chr3	lincRNA
ENSG00000052795.11	1.64930962	-0.1535694	0.00011663	0.00046266	0.02	0.07	0.12	0.26	FNIP2	folliculin interacting protein 2	chr4	protein_coding
ENSG00000124702.16	-1.0755368	6.24271305	0.00019245	0.0007287	45.33	36.93	26.31	25.73	KLHDC3	kelch domain containing 3	chr6	protein_coding
ENSG00000005810.16	1.34114429	3.23277999	9.44E-06	4.61E-05	0.3	0.28	1	0.95	MYCBP2	MYC binding protein 2, E3 ubiquitin protein ligase	chr13	protein_coding
ENSG00000181085.13	-8.0070223	-0.9638217	7.69E-14	1.09E-12	0.4	0.36	0	0	MAPK15	mitogen-activated protein kinase 15	chr8	protein_coding
ENSG00000279713.1	2.33140554	-2.1108519	0.00923116	0.02429359	0	0.04	0.15	0.15	CTD-3035K2	--	chr17	TEC
ENSG00000104221.11	-1.0698958	4.51728639	0.00024846	0.00092498	9.75	8.6	6.01	5.64	BRF2	BRF2, RNA polymerase III transcription initiation factor 50 kDa subunit	chr8	protein_coding
ENSG00000101443.16	1.90333886	2.40088238	2.94E-09	2.42E-08	2.01	1.14	9.03	6.73	WFDC2	WAP four-disulfide core domain 2	chr20	protein_coding
ENSG00000268975.1	1.11882677	2.93870309	0.00022913	0.00085797	3.05	3.25	9.57	8.64	MIA-RAB4B	MIA-RAB4B readthrough (NMD candidate)	chr19	protein_coding
ENSG00000266967.5	-1.6895367	6.53070035	7.78E-09	6.05E-08	89.66	83.13	36.95	34.38	AARSD1	alanyl-tRNA synthetase domain containing 1	chr17	protein_coding
ENSG00000278272.1	9.24136049	0.31654077	9.98E-24	3.16E-22	0	0	7.48	4.51	HIST1H3C	histone cluster 1, H3c	chr6	protein_coding
ENSG00000142453.10	-1.2275527	7.34789595	2.10E-05	9.60E-05	55.93	55.06	34.09	28.92	CARM1	coactivator-associated arginine methyltransferase 1	chr19	protein_coding
ENSG00000103942.11	2.69545931	3.93980122	1.09E-17	2.19E-16	1.45	1.87	13.79	14.83	HOMER2	homer scaffolding protein 2	chr15	protein_coding
ENSG00000162869.14	2.86847962	2.93415073	3.40E-18	7.10E-17	0.33	0.6	3.42	5.62	PPP1R21	protein phosphatase 1, regulatory subunit 21	chr2	protein_coding
ENSG00000167157.10	-2.9711692	4.90146821	1.70E-21	4.62E-20	31.97	33.53	6.5	4.59	PRRX2	paired related homeobox 2	chr9	protein_coding
ENSG00000138002.13	-2.4819347	5.2909012	3.06E-16	5.45E-15	9.01	8.64	2.08	2.13	IFT172	intraflagellar transport 172	chr2	protein_coding
ENSG00000196233.10	3.33052813	-0.3807586	7.88E-10	6.96E-09	0	0.02	0.12	0.14	LCOR	ligand dependent nuclear receptor corepressor	chr10	protein_coding
ENSG00000233871.1	1.6047608	1.59147586	1.99E-06	1.09E-05	2.64	2.15	8.88	10.62	DLG5-AS1	DLG5 antisense RNA 1	chr10	antisense
ENSG00000180279.5	-5.2109276	-3.0346387	0.00538165	0.01488851	0.06	0.18	0	0	MGC45922	--	chr19	lincRNA
ENSG00000102053.12	4.87917439	-3.0678699	0.01889873	0.04519557	0	0	0	0.03	ZC3H12B	zinc finger CCCH-type containing 12B	chrX	protein_coding
ENSG00000205212.3	3.77844945	-1.5015709	3.56E-06	1.88E-05	0.01	0.01	0.17	0.22	CCDC144NL	coiled-coil domain containing 144 family, N-terminal like	chr17	protein_coding
ENSG00000179091.4	-1.1061515	8.34574255	0.00011894	0.00047064	313.7	265.93	191.78	166.55	CYC1	cytochrome c-1	chr8	protein_coding
ENSG00000151692.13	8.43149953	-0.419548	3.10E-16	5.50E-15	0	0	0.22	0.27	RNF144A	ring finger protein 144A	chr2	protein_coding
ENSG00000177119.14	1.04658892	3.75022266	0.00041951	0.00148875	0.98	1	2.47	3.01	ANO6	anoctamin 6	chr12	protein_coding
ENSG00000050327.13	3.55646736	4.76825541	3.56E-28	1.50E-26	0.62	0.58	9.64	9.05	ARHGEF5	Rho guanine nucleotide exchange factor (GEF) 5	chr7	protein_coding
ENSG00000136930.11	-1.5014581	8.08323614	2.30E-07	1.46E-06	356.81	304.94	151.89	159.94	PSMB7	proteasome (prosome, macropain) subunit, beta type, 7	chr9	protein_coding
ENSG00000179163.11	3.01458053	5.2097363	4.35E-22	1.23E-20	2.93	3.5	30.23	39.18	FUCA1	fucosidase, alpha-L- 1, tissue	chr1	protein_coding
ENSG00000099812.7	-1.1923484	7.23272342	3.56E-05	0.00015638	58.92	59.04	35.12	33.6	MISP	mitotic spindle positioning	chr19	protein_coding
ENSG00000164715.5	1.52230163	4.41403895	2.81E-07	1.76E-06	1.1	0.88	3.56	4.05	LMTK2	lemur tyrosine kinase 2	chr7	protein_coding
ENSG00000244122.2	4.86951858	4.14271174	2.82E-42	2.43E-40	0.4	0.4	13.17	18.3	UGT1A7	UDP glucuronosyltransferase 1 family, polypeptide A7	chr2	protein_coding
ENSG00000039319.15	2.72802943	0.73549615	1.63E-11	1.79E-10	0.08	0.02	0.25	0.6	ZFYVE16	zinc finger, FYVE domain containing 16	chr5	protein_coding
ENSG00000182585.8	3.82634103	0.04481804	1.72E-13	2.34E-12	0.07	0.05	0.61	1.7	EPGN	epithelial mitogen	chr4	protein_coding
ENSG00000053501.11	-2.2477877	6.53737192	4.68E-14	6.75E-13	60.8	53.1	14.22	17.84	USE1	unconventional SNARE in the ER 1 homolog (S. cerevisiae)	chr19	protein_coding
ENSG00000166922.7	-4.6772828	1.64791985	4.61E-29	2.04E-27	3.78	4.12	0.23	0.18	SCG5	secretogranin V	chr15	protein_coding
ENSG00000202198.1	1.38044871	3.99611505	3.53E-06	1.87E-05	16.48	26.71	110.13	37.6	RN7SK	RNA, 7SK small nuclear	chr6	misc_RNA
ENSG00000143318.11	5.6781718	-2.6083292	0.00047761	0.00167191	0	0	0.08	0.12	CASQ1	calsequestrin 1 (fast-twitch, skeletal muscle)	chr1	protein_coding
ENSG00000178425.12	1.87158799	1.26199708	1.24E-07	8.21E-07	0.14	0.09	0.3	0.83	NT5DC1	5'-nucleotidase domain containing 1	chr6	protein_coding
ENSG00000012983.10	1.99554662	2.32993085	7.19E-10	6.37E-09	0.39	0.34	1.24	2.69	MAP4K5	mitogen-activated protein kinase kinase kinase kinase 5	chr14	protein_coding
ENSG00000099864.16	-1.4606082	5.87879051	5.42E-07	3.26E-06	26.18	24.27	12.63	11.77	PALM	paralemmin	chr19	protein_coding
ENSG00000261783.1	2.4765608	-2.4103938	0.00737015	0.01977584	0.14	0	0.86	0.36	RP11-252K2	--	chr16	sense_intronic
ENSG00000230797.2	-1.0026652	-0.3758787	0.01890111	0.04519676	0.3	0.27	0.11	0.27	YY2	YY2 transcription factor	chrX	protein_coding
ENSG00000161328.10	1.49055151	1.78089431	5.74E-06	2.92E-05	0.43	0.59	2.02	1.82	LRRC56	leucine rich repeat containing 56	chr11	protein_coding
ENSG00000102743.13	-1.0198704	2.5740277	0.0009147	0.00303221	4.32	3.77	3.01	2.3	SLC25A15	solute carrier family 25 (mitochondrial carrier; ornithine transporter) member 15	chr13	protein_coding
ENSG00000187595.13	2.26050432	-1.4015611	0.00030738	0.0011156	0	0.07	0.21	0.21	ZNF385C	zinc finger protein 385C	chr17	protein_coding
ENSG00000261396.1	5.28996391	-0.2327792	9.96E-16	1.69E-14	0.1	0	3.02	2.98	CTD-2012K1	--	chr16	antisense
ENSG00000185904.10	6.74420988	2.54708552	3.16E-45	3.31E-43	0.05	0.03	5.63	5.21	LINC00839	long intergenic non-protein coding RNA 839	chr10	lincRNA
ENSG00000130826.14	-2.1747109	7.69104494	2.29E-13	3.06E-12	80.95	97.1	23.59	29.04	DKC1	dyskeratosis congenita 1, dyskerin	chrX	protein_coding
ENSG00000243572.1	5.45974547	-2.7440718	0.00158008	0.00495521	0	0	0.27	0.46	RP11-889D3	--	chr3	lincRNA
ENSG00000107223.11	-1.0664197	9.81381815	0.00020225	0.00076281	838.49	805.1	530.33	515.04	EDF1	endothelial differentiation-related factor 1	chr9	protein_coding
ENSG00000183734.4	5.02752229	-1.2230318	7.16E-09	5.62E-08	0	0.02	0.47	0.32	ASCL2	achaete-scute family bHLH transcription factor 2	chr11	protein_coding
ENSG00000274225.1	-5.2091082	-3.0354822	0.00538165	0.01488851	0.17	0.18	0	0	KB-68A7.1	--	chr21	lincRNA
ENSG00000168481.7	-3.4636989	-2.1134182	0.00016274	0.00062431	0.1	0.07	0.02	0	LGI3	leucine-rich repeat LGI family, member 3	chr8	protein_coding
ENSG00000122970.14	2.40459959	1.23677449	6.20E-11	6.35E-10	0.17	0.08	0.47	1.37	IFT81	intraflagellar transport 81	chr12	protein_coding
ENSG00000041357.14	1.3422459	5.73299328	3.98E-06	2.08E-05	4.74	5.69	13.8	21.62	PSMA4	proteasome (prosome, macropain) subunit, alpha type, 4	chr15	protein_coding
ENSG00000204314.9	-2.1684011	1.62222973	2.13E-10	2.04E-09	2.09	1.77	0.62	0.52	PRRT1	proline-rich transmembrane protein 1	chr6	protein_coding
ENSG00000133740.9	-2.0686822	-0.6261143	1.76E-05	8.15E-05	0.36	0.44	0.14	0.11	E2F5	E2F transcription factor 5, p130-binding	chr8	protein_coding
ENSG00000260274.1	2.22511902	1.12131794	1.30E-09	1.11E-08	0.52	0.3	2.35	2.82	RP11-817O1	--	chr15	lincRNA
ENSG00000066933.14	4.11816536	1.64367538	5.98E-24	1.91E-22	0.01	0.03	0.39	0.46	MYO9A	myosin IXA	chr15	protein_coding
ENSG00000133433.9	-1.6080403	0.82877542	8.80E-06	4.32E-05	2.38	1.47	1.08	0.62	GSTT2B	glutathione S-transferase theta 2B (gene/pseudogene)	chr22	protein_coding
ENSG00000155926.12	-4.7350496	-1.8397013	1.01E-06	5.80E-06	0.06	0.15	0.01	0	SLA	Src-like-adaptor	chr8	protein_coding
ENSG00000167131.15	-1.1190109	1.87775056	0.00048418	0.00169391	1.33	0.96	0.81	0.6	CCDC103	coiled-coil domain containing 103	chr17	protein_coding
ENSG00000215788.8	-4.0977401	3.63135733	8.03E-33	4.24E-31	12.39	10.57	0.91	0.87	TNFRSF25	tumor necrosis factor receptor superfamily, member 25	chr1	protein_coding
ENSG00000196584.2	2.14635229	0.47548924	7.39E-08	5.05E-07	0.14	0.11	0.65	0.84	XRCC2	X-ray repair complementing defective repair in Chinese hamster cells 2	chr7	protein_coding
ENSG00000231074.7	-1.2702017	3.38358012	2.29E-05	0.00010408	0.86	0.85	0.47	0.47	HCG18	HLA complex group 18 (non-protein coding)	chr6	antisense
ENSG00000145736.13	2.15981553	0.9029394	7.66E-09	5.97E-08	0.13	0.11	0.56	0.83	GTF2H2	general transcription factor IIH, polypeptide 2, 44kDa	chr5	protein_coding
ENSG00000124839.11	1.34129475	1.60067015	5.21E-05	0.00022168	0.69	0.68	2.54	2.09	RAB17	RAB17, member RAS oncogene family	chr2	protein_coding
ENSG00000130818.9	1.02693349	2.21066213	0.001119	0.00364367	0.33	0.32	0.83	0.92	ZNF426	zinc finger protein 426	chr19	protein_coding
ENSG00000144591.16	-1.0403998	5.57883458	0.00032138	0.00116429	26.41	25.07	18.1	15.19	GMPPA	GDP-mannose pyrophosphorylase A	chr2	protein_coding
ENSG00000246763.5	3.07388642	-2.0211877	0.00094481	0.00312013	0	0.02	0.16	0.13	RGMB-AS1	RGMB antisense RNA 1	chr5	antisense
ENSG00000151116.15	3.54715601	0.98177649	2.86E-17	5.52E-16	0.02	0.08	0.45	1.14	UEVLD	UEV and lactate/malate dehyrogenase domains	chr11	protein_coding
ENSG00000115844.9	3.49269136	3.52666103	1.62E-25	5.79E-24	0.41	0.6	7.69	7.52	DLX2	distal-less homeobox 2	chr2	protein_coding
ENSG00000196517.10	-3.0928071	5.91431388	2.26E-23	6.93E-22	20.18	20.34	3.11	3.22	SLC6A9	solute carrier family 6 (neurotransmitter transporter, glycine), member 9	chr1	protein_coding
ENSG00000261801.4	-2.4110799	2.19583827	2.97E-13	3.92E-12	1.43	1.52	0.27	0.46	LOXL1-AS1	LOXL1 antisense RNA 1	chr15	antisense
ENSG00000165644.9	-1.2410662	4.84316361	2.13E-05	9.73E-05	36.83	29.97	18.77	18.92	COMTD1	catechol-O-methyltransferase domain containing 1	chr10	protein_coding
ENSG00000120690.12	2.18333916	2.24153218	3.19E-11	3.38E-10	0.38	0.33	1.46	2.88	ELF1	E74-like factor 1 (ets domain transcription factor)	chr13	protein_coding
ENSG00000237489.2	1.96029974	-2.0544475	0.01397446	0.03477946	0.02	0.02	0.15	0.07	LINC00959	long intergenic non-protein coding RNA 959	chr10	lincRNA
ENSG00000117143.12	1.85168094	4.44616712	6.54E-10	5.85E-09	2.67	3.76	12.94	18.02	UAP1	UDP-N-acetylglucosamine pyrophosphorylase 1	chr1	protein_coding
ENSG00000106367.12	-1.3770897	6.00035187	2.16E-06	1.18E-05	60.88	53.62	27.64	31.21	AP1S1	adaptor-related protein complex 1, sigma 1 subunit	chr7	protein_coding
ENSG00000100027.13	3.73320799	0.13350755	9.36E-14	1.31E-12	0.01	0.04	0.42	0.49	YPEL1	yippee-like 1 (Drosophila)	chr22	protein_coding
ENSG00000204713.9	-1.0800489	6.59750933	0.00017786	0.00067785	36.28	35.45	22.39	22.83	TRIM27	tripartite motif containing 27	chr6	protein_coding
ENSG00000129521.12	-3.9480889	3.90972113	1.15E-31	5.74E-30	5.5	3.87	0.48	0.33	EGLN3	egl-9 family hypoxia-inducible factor 3	chr14	protein_coding
ENSG00000256050.2	-2.7281736	-0.5603523	9.37E-08	6.30E-07	0.76	0.63	0.25	0.04	RP11-982M1	--	chr14	lincRNA
ENSG00000170456.13	2.29253041	2.16577442	6.92E-12	7.91E-11	0.09	0.14	0.7	0.78	DENND5B	DENN/MADD domain containing 5B	chr12	protein_coding
ENSG00000272335.1	2.94234222	-0.0015208	3.10E-10	2.91E-09	0.02	0.11	0.38	0.98	RP11-53O19	--	chr5	lincRNA
ENSG00000116984.11	1.25322445	2.71884136	4.72E-05	0.00020301	0.25	0.34	0.54	1.34	MTR	5-methyltetrahydrofolate-homocysteine methyltransferase	chr1	protein_coding
ENSG00000204228.3	-2.6336577	2.21623835	2.96E-15	4.83E-14	7.7	5.35	1.04	1.76	HSD17B8	hydroxysteroid (17-beta) dehydrogenase 8	chr6	protein_coding
ENSG00000260193.1	-3.1033285	0.04112597	9.99E-12	1.12E-10	1.13	1.16	0.25	0.11	RP11-83N9.5	--	chr9	lincRNA
ENSG00000135596.16	-3.2134317	5.59698009	1.10E-24	3.71E-23	19.18	16.62	2.84	2.3	MICAL1	microtubule associated monooxygenase, calponin and LIM domain containing 1	chr6	protein_coding
ENSG00000167842.14	1.41013538	2.01848804	1.26E-05	5.99E-05	0.55	0.69	1.65	2.78	MIS12	MIS12 kinetochore complex component	chr17	protein_coding
ENSG00000261717.4	2.73046651	-1.5310955	0.00013983	0.00054609	0.2	0	0.73	1.18	RP11-77K12.	--	chr16	protein_coding
ENSG00000189266.10	1.69936225	3.29825144	2.74E-08	1.97E-07	1.72	1.35	4.61	8.77	PNRC2	proline-rich nuclear receptor coactivator 2	chr1	protein_coding
ENSG00000155666.10	-1.414567	1.58854257	1.62E-05	7.58E-05	0.99	1.65	0.59	0.73	KDM8	lysine (K)-specific demethylase 8	chr16	protein_coding
ENSG00000103356.14	-1.0619061	4.88523484	0.0002598	0.0009623	7.27	6.96	4.4	4.69	EARS2	glutamyl-tRNA synthetase 2, mitochondrial	chr16	protein_coding
ENSG00000266202.1	3.16037301	-1.2025217	2.24E-06	1.22E-05	0.18	0	0.55	1.78	RP1-66C13.4	--	chr17	protein_coding
ENSG00000005379.14	-7.4155397	1.94242975	3.61E-43	3.30E-41	0.84	0.78	0.01	0	BZRAP1	benzodiazepine receptor (peripheral) associated protein 1	chr17	protein_coding
ENSG00000145012.11	1.61230287	2.8282975	2.04E-07	1.31E-06	0.12	0.12	0.44	0.56	LPP	LIM domain containing preferred translocation partner in lipoma	chr3	protein_coding
ENSG00000274012.1	3.9356861	3.7466254	6.42E-31	3.08E-29	4.9	3.87	126.31	51.56	RN7SL2	RNA, 7SL, cytoplasmic 2	chr14	misc_RNA
ENSG00000234336.5	-5.2091082	-3.0354822	0.00538165	0.01488851	0.03	0.03	0	0	JAZF1-AS1	JAZF1 antisense RNA 1	chr7	antisense
ENSG00000258289.6	1.9593068	4.13461879	1.00E-10	1.01E-09	1.58	1.62	8.31	8.26	CHURC1	churchill domain containing 1	chr14	protein_coding
ENSG00000159593.13	2.23770607	4.52541726	1.87E-13	2.52E-12	3.14	3.54	15.13	27.13	NAE1	NEDD8 activating enzyme E1 subunit 1	chr16	protein_coding
ENSG00000204531.14	-1.3113936	0.72400019	0.00026653	0.00097861	0.39	0.91	0.27	0.43	POU5F1	POU class 5 homeobox 1	chr6	protein_coding
ENSG00000198315.9	1.33443247	1.44927945	7.14E-05	0.00029569	0.14	0.19	0.49	0.6	ZKSCAN8	zinc finger with KRAB and SCAN domains 8	chr6	protein_coding
ENSG00000069431.9	1.67784461	-1.1962515	0.00368477	0.01064105	0	0.02	0.04	0.06	ABCC9	ATP-binding cassette, sub-family C (CFTR/MRP), member 9	chr12	protein_coding
ENSG00000154529.13	-5.5071225	1.68315117	1.48E-34	8.39E-33	0.53	0.5	0.01	0.02	CNTNAP3B	contactin associated protein-like 3B	chr9	protein_coding
ENSG00000157873.16	2.55753376	0.28337735	2.15E-09	1.80E-08	0.12	0.15	0.96	1.19	TNFRSF14	tumor necrosis factor receptor superfamily, member 14	chr1	protein_coding
ENSG00000149346.13	1.69592653	0.5468877	9.58E-06	4.67E-05	0.09	0.08	0.34	0.4	SLX4IP	SLX4 interacting protein	chr20	protein_coding
ENSG00000118402.5	2.82955674	1.37331477	2.97E-14	4.39E-13	0.27	0.05	1.02	2.08	ELOVL4	ELOVL fatty acid elongase 4	chr6	protein_coding
ENSG00000279088.1	-1.0704796	1.18549564	0.00158886	0.0049756	1.84	2.13	1.53	0.99	RP11-574K1	--	chr10	TEC
ENSG00000115255.9	1.48390711	4.74124952	4.61E-07	2.80E-06	7.69	4.94	23.71	23.5	REEP6	receptor accessory protein 6	chr19	protein_coding
ENSG00000124486.11	2.45643072	5.50188781	4.32E-16	7.58E-15	0.88	0.94	6.27	7.05	USP9X	ubiquitin specific peptidase 9, X-linked	chrX	protein_coding
ENSG00000139517.7	1.99503352	0.24979207	7.83E-07	4.58E-06	0.13	0.02	0.47	0.35	LNX2	ligand of numb-protein X 2	chr13	protein_coding
ENSG00000223711.1	4.91020659	-3.0511096	0.0100455	0.02582742	0	0	0.18	0.05	AC091633.3	--	chr3	antisense
ENSG00000205143.2	-2.1006923	-0.1506585	1.50E-06	8.40E-06	0.83	0.78	0.23	0.27	ARID3C	AT rich interactive domain 3C (BRIGHT-like)	chr9	protein_coding
ENSG00000142197.11	1.43488852	2.45387731	5.27E-06	2.69E-05	0.31	0.3	1	1.22	DOPEY2	dopey family member 2	chr21	protein_coding
ENSG00000150551.10	-5.1470956	-1.5087027	1.07E-08	8.18E-08	0.14	0.3	0.01	0	LYPD1	LY6/PLAUR domain containing 1	chr2	protein_coding
ENSG00000140396.11	1.89393417	1.07321518	1.62E-07	1.05E-06	0.08	0.08	0.37	0.44	NCOA2	nuclear receptor coactivator 2	chr8	protein_coding
ENSG00000030582.15	1.42527239	9.69198714	8.43E-07	4.92E-06	176.73	161.44	635	573.84	GRN	granulin	chr17	protein_coding
ENSG00000111331.11	1.91976206	5.84605437	8.51E-11	8.57E-10	2.54	2.41	12.54	12.4	OAS3	2'-5'-oligoadenylate synthetase 3, 100kDa	chr12	protein_coding
ENSG00000086619.12	5.439154	0.40457014	4.16E-21	1.09E-19	0.01	0.01	0.36	0.76	ERO1LB	ERO1-like beta (S. cerevisiae)	chr1	protein_coding
ENSG00000213023.8	11.5807074	2.56482028	5.90E-53	9.82E-51	0	0	4.03	3.94	SYT3	synaptotagmin III	chr19	protein_coding
ENSG00000260339.1	2.16345574	-0.857826	3.53E-05	0.00015509	0.1	0	0.18	0.44	HEXA-AS1	HEXA antisense RNA 1	chr15	antisense
ENSG00000038002.7	1.18451331	1.64365954	0.00030692	0.00111459	0.69	0.74	1.43	2.91	AGA	aspartylglucosaminidase	chr4	protein_coding
ENSG00000160716.4	6.51968671	-2.0237618	1.78E-06	9.85E-06	0	0	0.08	0.05	CHRNB2	cholinergic receptor, nicotinic, beta 2 (neuronal)	chr1	protein_coding
ENSG00000119125.15	1.99802163	4.42746465	3.44E-11	3.62E-10	1.12	1.18	4.96	7.38	GDA	guanine deaminase	chr9	protein_coding
ENSG00000146555.17	-8.9699908	2.11889278	1.11E-48	1.40E-46	0.62	0.74	0	0	SDK1	sidekick cell adhesion molecule 1	chr7	protein_coding
ENSG00000203950.6	-1.3331211	5.24816375	4.87E-06	2.51E-05	34.98	35.81	20.11	17.25	FAM127B	family with sequence similarity 127, member B	chrX	protein_coding
ENSG00000116690.10	9.63063682	4.45169858	2.62E-76	1.43E-73	0.01	0.01	8.91	9.69	PRG4	proteoglycan 4	chr1	protein_coding
ENSG00000101194.16	-8.5991214	1.74899213	1.23E-43	1.16E-41	1.56	1.43	0	0.01	SLC17A9	solute carrier family 17 (vesicular nucleotide transporter), member 9	chr20	protein_coding
ENSG00000272667.1	1.10518675	1.10631322	0.00132706	0.00426552	1.15	2.25	5.8	3.89	RP11-395A1	--	chr2	lincRNA
ENSG00000223813.2	6.72803071	-1.8641315	2.24E-07	1.42E-06	0	0	0.33	0.26	AC007255.8	--	chr7	antisense
ENSG00000132603.12	1.13667212	4.81672205	9.73E-05	0.00039288	4.97	4.65	13.1	15.13	NIP7	NIP7, nucleolar pre-rRNA processing protein	chr16	protein_coding
ENSG00000090924.13	-1.9576235	4.89258636	5.65E-11	5.83E-10	4.43	4.72	1.79	1.34	PLEKHG2	pleckstrin homology domain containing, family G (with RhoGef domain) member 2	chr19	protein_coding
ENSG00000135069.12	-1.9643963	6.62884385	2.79E-11	2.98E-10	56.27	60.37	17.58	22.34	PSAT1	phosphoserine aminotransferase 1	chr9	protein_coding
ENSG00000135482.5	-1.4747293	3.33354245	1.10E-06	6.26E-06	1.51	1.81	0.82	0.77	ZC3H10	zinc finger CCCH-type containing 10	chr12	protein_coding
ENSG00000028203.16	1.60437937	2.07431342	7.84E-07	4.59E-06	0.18	0.41	0.92	1.46	VEZT	vezatin, adherens junctions transmembrane protein	chr12	protein_coding
ENSG00000120526.9	1.05617888	0.83208691	0.003444	0.01000844	0.28	0.13	0.21	0.93	NUDCD1	NudC domain containing 1	chr8	protein_coding
ENSG00000141179.12	-1.8897505	2.43056314	2.58E-09	2.14E-08	2.06	2.26	0.57	0.98	PCTP	phosphatidylcholine transfer protein	chr17	protein_coding
ENSG00000088854.12	1.0003517	2.26070933	0.00137477	0.00440875	0.31	0.43	0.7	1.28	C20orf194	chromosome 20 open reading frame 194	chr20	protein_coding
ENSG00000103222.17	-1.6156277	8.21835343	2.87E-08	2.06E-07	53.75	56.56	24	23.94	ABCC1	ATP-binding cassette, sub-family C (CFTR/MRP), member 1	chr16	protein_coding
ENSG00000242687.2	-1.0386415	0.4513654	0.00599764	0.01642959	0.43	0.25	0.37	0.08	AC004893.11	--	chr7	antisense
ENSG00000274897.2	1.82582413	0.32220992	4.60E-06	2.38E-05	0.29	0.2	1.07	1.26	PANO1	proapoptotic nucleolar protein 1	chr11	protein_coding
ENSG00000185728.15	1.72915324	1.43855921	4.55E-07	2.77E-06	0.15	0.21	0.58	1.02	YTHDF3	YTH N(6)-methyladenosine RNA binding protein 3	chr8	protein_coding
ENSG00000221953.5	2.79254626	-2.209175	0.00550654	0.01520944	0	0.03	0.11	0.13	C1orf229	chromosome 1 open reading frame 229	chr1	lincRNA
ENSG00000177576.9	-1.0370387	8.08625319	0.00030354	0.001104	44.56	49	27.73	33.13	C18orf32	chromosome 18 open reading frame 32	chr18	protein_coding
ENSG00000137801.10	1.87593911	7.10263646	1.77E-10	1.71E-09	5.26	7	26.52	33.56	THBS1	thrombospondin 1	chr15	protein_coding
ENSG00000269038.1	2.24418087	-1.4157076	0.00043455	0.00153757	0.3	0.06	0.78	1.57	AP001462.6	--	chr11	lincRNA
ENSG00000148700.12	1.68640318	2.11416465	1.84E-07	1.19E-06	0.37	0.3	1.02	1.9	ADD3	adducin 3 (gamma)	chr10	protein_coding
ENSG00000196754.9	-2.8357695	3.63841484	5.76E-19	1.29E-17	11.78	10.78	2.53	1.67	S100A2	S100 calcium binding protein A2	chr1	protein_coding
ENSG00000196924.13	-2.785093	12.0664445	4.15E-20	1.01E-18	734.83	689.16	142.87	132.15	FLNA	filamin A, alpha	chrX	protein_coding
ENSG00000167380.15	1.1320391	3.0546326	0.00018194	0.00069218	0.68	0.65	1.5	2.41	ZNF226	zinc finger protein 226	chr19	protein_coding
ENSG00000051382.7	2.6199868	2.12586419	1.65E-14	2.51E-13	0.2	0.12	1.05	1.59	PIK3CB	phosphatidylinositol-4,5-bisphosphate 3-kinase, catalytic subunit beta	chr3	protein_coding
ENSG00000149212.9	-3.6485301	-1.5489762	1.56E-06	8.69E-06	0.06	0.03	0.01	0	SESN3	sestrin 3	chr11	protein_coding
ENSG00000203814.6	2.24953234	1.62977341	1.12E-10	1.12E-09	0.25	0.41	2.91	1.28	HIST2H2BF	histone cluster 2, H2bf	chr1	protein_coding
ENSG00000148773.11	1.42634838	7.16287697	8.82E-07	5.12E-06	4.17	5.76	16.67	18.91	MKI67	marker of proliferation Ki-67	chr10	protein_coding
ENSG00000176597.10	1.38358216	0.97529521	0.00010747	0.00042972	0.15	0.2	0.4	0.83	B3GNT5	UDP-GlcNAc:betaGal beta-1,3-N-acetylglucosaminyltransferase 5	chr3	protein_coding
ENSG00000141759.13	-1.5748182	6.57403228	6.67E-08	4.59E-07	27.57	28.19	12.75	12.17	TXNL4A	thioredoxin-like 4A	chr18	protein_coding
ENSG00000044459.13	3.15808507	0.86409647	2.52E-14	3.76E-13	0.01	0.05	0.22	0.49	CNTLN	centlein, centrosomal protein	chr9	protein_coding
ENSG00000159899.13	-1.2540257	1.61925424	0.00012859	0.0005061	0.92	0.96	0.5	0.55	NPR2	natriuretic peptide receptor 2	chr9	protein_coding
ENSG00000182118.5	1.68594805	1.15167479	1.79E-06	9.89E-06	0.32	0.78	2.04	2.66	FAM89A	family with sequence similarity 89, member A	chr1	protein_coding
ENSG00000124614.12	-1.8312954	8.83941973	7.56E-10	6.70E-09	38.56	35.22	13.96	13.67	RPS10	ribosomal protein S10	chr6	protein_coding
ENSG00000118707.8	-2.8458698	3.95255598	2.06E-19	4.73E-18	6.03	6.18	1.26	1	TGIF2	TGFB-induced factor homeobox 2	chr20	protein_coding
ENSG00000186815.11	-2.2866964	6.75574751	1.81E-14	2.73E-13	26.84	26.27	7.17	7.34	TPCN1	two pore segment channel 1	chr12	protein_coding
ENSG00000166471.9	1.35746298	2.88976917	9.64E-06	4.69E-05	0.68	0.92	2.24	3.26	TMEM41B	transmembrane protein 41B	chr11	protein_coding
ENSG00000105472.11	6.7720806	2.57185328	1.42E-45	1.50E-43	0	0	9.18	7.73	CLEC11A	C-type lectin domain family 11, member A	chr19	protein_coding
ENSG00000136630.12	-2.4589745	3.61054175	3.49E-15	5.65E-14	7.73	6.94	1.73	1.83	HLX	H2.0-like homeobox	chr1	protein_coding
ENSG00000223714.1	1.37278788	-1.0814184	0.01224023	0.03086062	0.22	0.2	0.54	0.95	RP5-1172N1	--	chrX	lincRNA
ENSG00000278002.1	5.66878161	-2.6135853	0.00086564	0.00288128	0	0	0	0.23	RP11-596C2	--	chr14	lincRNA
ENSG00000119685.18	1.27688753	4.14024631	1.60E-05	7.47E-05	0.82	1.43	3.48	3.75	TTLL5	tubulin tyrosine ligase-like family member 5	chr14	protein_coding
ENSG00000274265.3	-1.1772814	2.85911999	0.00011305	0.00044935	10.28	9.06	6.92	4.45	CH17-189H2	--	chr1	lincRNA
ENSG00000119684.14	1.67967388	1.6314049	6.52E-07	3.86E-06	0.16	0.14	0.56	0.69	MLH3	mutL homolog 3	chr14	protein_coding
ENSG00000171540.7	5.20747907	-2.8915189	0.00538165	0.01488851	0	0	0.03	0.04	OTP	orthopedia homeobox	chr5	protein_coding

ENSG00000197172.9	-12.854239	3.8041332	3.49E-73	1.67E-70	13.76	11.33	0	0	MAGEA6	melanoma antigen family A6	chrX	protein_coding
ENSG00000184194.5	-10.166699	1.11601571	1.99E-35	1.19E-33	0.72	0.85	0	0	GPR173	G protein-coupled receptor 173	chrX	protein_coding
ENSG00000138160.5	2.40274532	2.60718937	1.86E-13	2.52E-12	0.38	0.25	1.53	2.96	KIF11	kinesin family member 11	chr10	protein_coding
ENSG00000272764.1	1.64192656	-0.0014711	0.00011764	0.00046621	1.36	1.23	3.71	7.1	RP11-318E3.	--	chr10	lincRNA
ENSG00000124459.10	1.01985819	2.34403501	0.00108779	0.00354652	0.48	0.87	1.33	2.32	ZNF45	zinc finger protein 45	chr19	protein_coding
ENSG00000164241.12	1.34283011	1.00485318	0.00015171	0.00058571	0.17	0.1	0.41	0.5	C5orf63	chromosome 5 open reading frame 63	chr5	protein_coding
ENSG00000169962.4	1.55989025	0.21772149	0.00013052	0.00051325	0.11	0.15	0.47	0.55	TAS1R3	taste receptor, type 1, member 3	chr1	protein_coding
ENSG00000198925.9	-1.4202703	7.53708372	9.58E-07	5.53E-06	52.49	51.5	28.69	22.92	ATG9A	autophagy related 9A	chr2	protein_coding
ENSG00000008517.15	-6.8940438	6.03341797	2.74E-72	1.26E-69	67.61	57.23	0.82	0.58	IL32	interleukin 32	chr16	protein_coding
ENSG00000279978.1	1.86676151	4.83472677	3.87E-10	3.59E-09	0.04	0.04	0.2	0.16	chr22-38_28	--	chr22	lincRNA
ENSG00000229950.1	1.83033092	0.57008394	2.00E-06	1.10E-05	0.36	0.22	1.5	1.29	TFAP2A-AS1	TFAP2A antisense RNA 1	chr6	antisense
ENSG00000148303.15	-1.755152	11.0329101	1.85E-09	1.56E-08	3117.06	2754.53	1167.26	1151.04	RPL7A	ribosomal protein L7a	chr9	protein_coding
ENSG00000226237.1	-3.1883349	0.41016368	3.74E-14	5.44E-13	0.35	0.53	0.04	0.08	RP11-276H1	--	chr9	lincRNA
ENSG00000214595.10	2.37321142	-0.0110525	1.22E-07	8.08E-07	0.04	0.02	0.12	0.27	EML6	echinoderm microtubule associated protein like 6	chr2	protein_coding
ENSG00000254093.7	-1.0263581	4.56699603	0.00043208	0.00152996	16	17.3	11.28	10.47	PINX1	PIN2/TERF1 interacting, telomerase inhibitor 1	chr8	protein_coding
ENSG00000231327.1	-1.2357888	-0.0915486	0.00287234	0.00855245	1.74	1.33	1.35	0.41	AC016700.5	--	chr2	lincRNA
ENSG00000163347.5	-2.3570192	1.39411659	1.52E-11	1.67E-10	0.83	1.21	0.16	0.37	CLDN1	claudin 1	chr3	protein_coding
ENSG00000176383.8	2.40673939	0.63096251	1.36E-09	1.16E-08	0.09	0.07	0.58	0.55	B3GNT4	UDP-GlcNAc:betaGal beta-1,3-N-acetylglucosaminyltransferase 4	chr12	protein_coding
ENSG00000198718.11	5.55634758	-0.7694664	3.90E-12	4.58E-11	0	0	0.15	0.18	FAM179B	family with sequence similarity 179, member B	chr14	protein_coding
ENSG00000243725.5	-1.0377549	0.3277798	0.00692577	0.01870545	0.57	0.53	0.37	0.34	TTC4	tetratricopeptide repeat domain 4	chr1	protein_coding
ENSG00000116649.8	-1.5577969	7.49246056	8.58E-08	5.80E-07	170.78	173.06	83.6	71.67	SRM	spermidine synthase	chr1	protein_coding
ENSG00000169594.11	3.31884968	-0.392259	1.04E-09	9.04E-09	0.03	0.01	0.21	0.34	BNC1	basonuclin 1	chr15	protein_coding
ENSG00000128849.10	-4.2559946	0.47294033	6.64E-20	1.59E-18	0.22	0.37	0	0.04	CGNL1	cingulin-like 1	chr15	protein_coding
ENSG00000112118.16	-1.1496348	8.94275727	6.40E-05	0.00026805	156.12	186.05	102.48	102.82	MCM3	minichromosome maintenance complex component 3	chr6	protein_coding
ENSG00000171462.13	-2.6277077	-0.1252746	3.70E-09	3.01E-08	0.45	0.66	0.11	0.12	DLK2	delta-like 2 homolog (Drosophila)	chr6	protein_coding
ENSG00000234678.1	-8.9541236	-0.0746533	6.39E-22	1.78E-20	1.39	1.47	0	0	RP11-465N4	--	chr1	antisense
ENSG00000224596.6	-4.8937477	3.10553551	4.68E-39	3.42E-37	3.14	2.96	0.13	0.14	ZMIZ1-AS1	ZMIZ1 antisense RNA 1	chr10	antisense
ENSG00000165606.7	-7.0872298	-1.7605703	1.16E-08	8.76E-08	0.14	0.15	0	0	DRGX	dorsal root ganglia homeobox	chr10	protein_coding
ENSG00000205362.9	-8.1748869	1.32728289	4.22E-38	2.89E-36	12.03	7.32	0	0.07	MT1A	metallothionein 1A	chr16	protein_coding
ENSG00000090621.12	-1.1123721	8.10800522	0.00010939	0.00043639	93.53	103.44	57.54	63.94	PABPC4	poly(A) binding protein, cytoplasmic 4 (inducible form)	chr1	protein_coding
ENSG00000145604.14	1.24102787	4.73758984	2.22E-05	0.00010109	3.55	3.12	8.36	12.76	SKP2	S-phase kinase-associated protein 2, E3 ubiquitin protein ligase	chr5	protein_coding
ENSG00000265121.1	6.04467832	-2.3656018	4.74E-05	0.00020301	0	0	0.48	0.41	RP11-285E9.	--	chr17	lincRNA
ENSG00000204922.4	-1.1141626	7.05255573	0.00010953	0.00043673	67.19	55.3	39.15	36.23	UQCC3	ubiquinol-cytochrome c reductase complex assembly factor 3	chr11	protein_coding
ENSG00000230753.4	2.08655688	-1.9731797	0.00956893	0.02497848	0.04	0.12	0.55	0.39	ZNF341-AS1	ZNF341 antisense RNA 1	chr20	antisense
ENSG00000173918.13	-4.7773872	5.66086565	7.43E-45	7.52E-43	18.8	17.51	0.95	0.81	C1QTNF1	C1q and tumor necrosis factor related protein 1	chr17	protein_coding
ENSG00000174136.10	-1.2596813	3.71943153	2.31E-05	0.0001049	2.27	3.88	1.81	1.6	RGMB	repulsive guidance molecule family member b	chr5	protein_coding
ENSG00000113248.5	-4.1843497	0.30555316	1.78E-17	3.51E-16	0.41	0.53	0.06	0.01	PCDHB15	protocadherin beta 15	chr5	protein_coding
ENSG00000258486.3	3.62671506	3.59335487	3.77E-27	1.51E-25	4.19	5.48	112.65	44.84	RN7SL1	RNA, 7SL, cytoplasmic 1	chr14	non_coding
ENSG00000105559.10	-1.9849274	1.63357042	3.70E-09	3.01E-08	1.39	1.19	0.44	0.43	PLEKHA4	pleckstrin homology domain containing, family A (phosphoinositide binding specific) member 4	chr19	protein_coding
ENSG00000245105.2	2.69320711	-1.086078	7.10E-06	3.54E-05	0.05	0.03	0.32	0.35	A2M-AS1	A2M antisense RNA 1 (head to head)	chr12	antisense
ENSG00000275221.1	4.92065931	-3.045493	0.0100455	0.02582742	0	0	0.65	0	HIST1H2AK	histone cluster 1, H2ak	chr6	protein_coding
ENSG00000111897.6	1.08947719	3.81710335	0.00024079	0.00089826	2.14	2.48	4.1	9.11	SERINC1	serine incorporator 1	chr6	protein_coding
ENSG00000235034.5	4.00074754	-2.0228487	3.06E-05	0.00013563	0.03	0	0.54	0.39	C19orf81	chromosome 19 open reading frame 81	chr19	protein_coding
ENSG00000156482.9	-1.2642675	7.91844656	1.18E-05	5.64E-05	462.19	386.74	220.44	251.65	RPL30	ribosomal protein L30	chr8	protein_coding
ENSG00000175591.10	-4.7462948	2.90455602	5.73E-37	3.67E-35	4.52	3.67	0.12	0.28	P2RY2	purinergic receptor P2Y, G-protein coupled, 2	chr11	protein_coding
ENSG00000239264.7	-1.4866764	0.25041353	0.00011986	0.00047423	0.36	0.5	0.15	0.25	TXNDC5	thioredoxin domain containing 5 (endoplasmic reticulum)	chr6	protein_coding
ENSG00000185404.15	-1.3874785	2.85247101	5.88E-06	2.98E-05	1.63	2.35	0.85	1.17	SP140L	SP140 nuclear body protein-like	chr2	protein_coding
ENSG00000170873.17	1.07275405	2.1698464	0.00066239	0.0022642	0.39	0.47	0.96	1.44	MTSS1	metastasis suppressor 1	chr8	protein_coding
ENSG00000271997.1	4.56974895	-1.5844101	2.48E-07	1.57E-06	0.01	0	0.04	0.23	RP11-97O12	--	chr15	sense_intronic
ENSG00000137331.11	-3.3682502	7.94598086	2.95E-27	1.19E-25	291.1	245.4	35.53	33.7	IER3	immediate early response 3	chr6	protein_coding
ENSG00000165244.6	2.19966214	1.66071157	2.26E-10	2.15E-09	0.21	0.28	1.02	1.96	ZNF367	zinc finger protein 367	chr9	protein_coding
ENSG00000232956.7	-1.6426486	6.11284197	1.99E-08	1.46E-07	28.57	25.87	11.83	11.4	SNHG15	small nucleolar RNA host gene 15	chr7	lincRNA
ENSG00000196843.14	-1.3289008	2.79324616	1.50E-05	7.08E-05	2.72	2.33	1.3	1.39	ARID5A	AT rich interactive domain 5A (MRF1-like)	chr2	protein_coding
ENSG00000101236.15	1.49047777	4.83295216	4.04E-07	2.48E-06	1.55	1.6	5.92	5.87	RNF24	ring finger protein 24	chr20	protein_coding
ENSG00000134262.11	-1.0439255	4.2672188	0.0003673	0.00131928	7.64	6.85	4.46	4.93	AP4B1	adaptor-related protein complex 4, beta 1 subunit	chr1	protein_coding
ENSG00000072840.11	-11.668473	2.61645485	1.05E-55	2.11E-53	1.32	1.67	0	0	EVC	Ellis van Creveld protein	chr4	protein_coding
ENSG00000196914.7	2.3097544	3.3865728	1.77E-13	2.40E-12	0.26	0.3	1.72	2	ARHGEF12	Rho guanine nucleotide exchange factor (GEF) 12	chr11	protein_coding
ENSG00000163131.9	-3.40452	0.84837222	2.31E-16	4.13E-15	0.63	0.66	0.12	0.04	CTSS	cathepsin S	chr1	protein_coding
ENSG00000183598.3	3.02063418	-2.6615909	0.00943894	0.02469931	0.07	0	0.62	0.27	HIST2H3D	histone cluster 2, H3d	chr1	protein_coding
ENSG00000104313.16	-5.2824194	-0.6364806	4.08E-15	6.58E-14	0.2	0.23	0.01	0	EYA1	EYA transcriptional coactivator and phosphatase 1	chr8	protein_coding
ENSG00000153363.11	1.48283059	1.52058298	1.10E-05	5.31E-05	0.37	0.31	0.75	1.78	LINC00467	long intergenic non-protein coding RNA 467	chr1	lincRNA
ENSG00000131386.16	-6.2014387	2.36567268	8.29E-44	7.88E-42	1.83	1.55	0.01	0.04	GALNT15	polypeptide N-acetylgalactosaminyltransferase 15	chr3	protein_coding
ENSG00000184254.15	2.2440183	3.90171283	3.08E-13	4.06E-12	0.76	0.78	4.22	5.55	ALDH1A3	aldehyde dehydrogenase 1 family, member A3	chr15	protein_coding
ENSG00000139697.10	2.00797382	3.63972075	6.15E-11	6.31E-10	0.26	0.46	1.54	2.32	SBNO1	strawberry notch homolog 1 (Drosophila)	chr12	protein_coding
ENSG00000256196.1	-4.806998	-3.2310942	0.01889873	0.04519557	0.09	0.1	0	0	RP11-881M1	--	chr11	antisense
ENSG00000254911.3	3.44063908	4.15249206	4.68E-26	1.72E-24	6.59	7.06	110.98	86.09	SCARNA9	small Cajal body-specific RNA 9	chr11	antisense
ENSG00000119139.15	-3.9541121	3.72992015	1.36E-31	6.75E-30	2.87	3.78	0.22	0.35	TJP2	tight junction protein 2	chr9	protein_coding
ENSG00000107130.8	-1.3472945	5.78192002	3.60E-06	1.90E-05	13.33	11.38	6.71	6.22	NCS1	neuronal calcium sensor 1	chr9	protein_coding
ENSG00000120675.5	-7.6787823	1.66327241	1.81E-42	1.58E-40	0.64	0.66	0	0.01	DNAJC15	DnaJ (Hsp40) homolog, subfamily C, member 15	chr13	protein_coding
ENSG00000184922.12	-1.5258346	6.71329853	1.60E-07	1.04E-06	31.14	29.35	14.17	13.81	FMNL1	formin-like 1	chr17	protein_coding
ENSG00000250451.4	1.40761406	1.70200129	1.79E-05	8.28E-05	2.67	2.06	8.93	7.82	HOXC-AS1	HOXC cluster antisense RNA 1	chr12	antisense
ENSG00000183287.12	4.1463881	-1.9121319	1.13E-05	5.42E-05	0	0	0.05	0.08	CCBE1	collagen and calcium binding EGF domains 1	chr18	protein_coding
ENSG00000102172.14	-1.4855505	7.12846313	3.16E-07	1.97E-06	85.43	96.42	36.08	50.77	SMS	spermine synthase	chrX	protein_coding
ENSG00000258102.2	2.64631443	-1.5894058	0.00020516	0.00077306	0.04	0.13	0.58	0.87	MAP1LC3B2	microtubule-associated protein 1 light chain 3 beta 2	chr12	protein_coding
ENSG00000155542.10	2.36879893	-1.1464505	4.93E-05	0.0002108	0.09	0.02	0.41	0.34	SETD9	SET domain containing 9	chr5	protein_coding
ENSG00000228624.6	6.66024074	-1.913388	6.25E-07	3.72E-06	0	0	0.04	0.06	RP3-399L15.	--	chr6	antisense
ENSG00000198804.2	1.49703484	13.2216269	2.40E-07	1.52E-06	2047.94	3211.87	10935.89	8739.66	MT-CO1	mitochondrially encoded cytochrome c oxidase I	chrM	protein_coding
ENSG00000160551.8	3.3602908	2.58461135	5.90E-22	1.64E-20	0.05	0.08	0.79	1.08	TAOK1	TAO kinase 1	chr17	protein_coding
ENSG00000270165.1	2.03862009	-1.2141602	0.00040219	0.00143131	0.05	0.35	0.51	1.63	RP11-167P1	--	chr16	sense_intronic
ENSG00000071243.14	1.51238944	0.41800029	8.90E-05	0.00036046	0.1	0.1	0.19	0.58	ING3	inhibitor of growth family, member 3	chr7	protein_coding
ENSG00000168116.12	4.5790902	-0.8476207	1.64E-10	1.59E-09	0.02	0	0.24	0.41	KIAA1586	KIAA1586	chr6	protein_coding
ENSG00000102935.10	-4.4391802	-2.0626482	1.13E-05	5.42E-05	0.03	0.04	0	0	ZNF423	zinc finger protein 423	chr16	protein_coding
ENSG00000230873.7	5.21752153	-2.8860363	0.00538165	0.01488851	0	0	0.15	0.08	STMND1	stathmin domain containing 1	chr6	protein_coding
ENSG00000167384.9	2.24252326	-0.0341444	4.29E-07	2.62E-06	0.04	0.08	0.24	0.47	ZNF180	zinc finger protein 180	chr19	protein_coding
ENSG00000152409.8	1.65583176	2.0956432	3.23E-07	2.01E-06	0.15	0.21	0.66	0.86	JMY	junction mediating and regulatory protein, p53 cofactor	chr5	protein_coding
ENSG00000186104.9	1.11834206	2.85625963	0.00025412	0.00094374	1.37	1.04	3.64	3.34	CYP2R1	cytochrome P450, family 2, subfamily R, polypeptide 1	chr11	protein_coding
ENSG00000102098.16	1.4946715	0.85386155	4.08E-05	0.00017771	0.2	0.14	0.63	0.68	SCML2	sex comb on midleg-like 2 (Drosophila)	chrX	protein_coding
ENSG00000011083.7	-9.0032495	-0.0267182	2.24E-22	6.45E-21	0.42	0.35	0	0	SLC6A7	solute carrier family 6 (neurotransmitter transporter), member 7	chr5	protein_coding
ENSG00000041982.13	-1.0897987	4.87913291	0.00017868	0.00068065	3.35	3.83	1.98	2.53	TNC	tenascin C	chr9	protein_coding
ENSG00000260466.1	2.33743678	-1.558914	0.0007657	0.00258598	0.15	0.11	0.68	1.15	RP4-536B24.	--	chr16	antisense
ENSG00000163818.15	1.54422267	0.24818786	0.00013484	0.00052841	0.09	0.11	0.33	0.45	LZTFL1	leucine zipper transcription factor-like 1	chr3	protein_coding
ENSG00000115464.13	2.70016104	2.79115627	1.99E-16	3.58E-15	0.16	0.1	1.1	1.15	USP34	ubiquitin specific peptidase 34	chr2	protein_coding
ENSG00000198513.10	4.27860072	1.79016922	8.60E-26	3.13E-24	0.08	0.09	1.63	2.97	ATL1	atlastin GTPase 1	chr14	protein_coding
ENSG00000171116.7	-1.8282859	-1.7489723	0.00328145	0.0095697	0.15	0.17	0	0.11	HSFX1	heat shock transcription factor family, X linked 1	chrX	protein_coding
ENSG00000230641.1	-5.0197065	-3.1311226	0.0100455	0.02582742	0.58	0.1	0	0	USP12-AS2	USP12 antisense RNA 2 (head to head)	chr13	antisense
ENSG00000179988.12	1.35341011	0.22809506	0.0007142	0.00242503	0.35	0.23	0.75	1.23	PSTK	phosphoseryl-tRNA kinase	chr10	protein_coding
ENSG00000075461.5	-1.4762997	1.14932399	1.47E-05	6.96E-05	0.58	0.97	0.23	0.51	CACNG4	calcium channel, voltage-dependent, gamma subunit 4	chr17	protein_coding
ENSG00000268714.3	6.24527241	-2.2216849	1.55E-05	7.26E-05	0	0	0.46	0.97	CTD-2287O1	--	chr5	protein_coding
ENSG00000167005.12	1.68969081	5.60237838	9.07E-09	6.99E-08	4.38	3.99	15.5	20.63	NUDT21	nudix (nucleoside diphosphate linked moiety X)-type motif 21	chr16	protein_coding
ENSG00000151117.7	4.95767292	1.66259582	1.67E-29	7.54E-28	0.07	0.02	2.04	1.5	TMEM86A	transmembrane protein 86A	chr11	protein_coding
ENSG00000185681.11	4.89980841	-3.0567113	0.01889873	0.04519557	0	0	0.18	0.15	MORN5	MORN repeat containing 5	chr9	protein_coding
ENSG00000159224.4	-5.3733592	-2.9472019	0.00290522	0.00856312	0.22	0.12	0	0	GIP	gastric inhibitory polypeptide	chr17	protein_coding
ENSG00000223501.7	-1.4263861	5.9982296	9.56E-07	5.52E-06	21.97	22.28	11.07	10.86	VPS52	vacuolar protein sorting 52 homolog (S. cerevisiae)	chr6	protein_coding
ENSG00000204941.12	6.75509957	-1.847914	2.24E-07	1.42E-06	0	0	0.2	0.05	PSG5	pregnancy specific beta-1-glycoprotein 5	chr19	protein_coding
ENSG00000135776.4	2.56439273	0.49321615	6.87E-10	6.13E-09	0.03	0.12	0.4	0.85	ABCB10	ATP-binding cassette, sub-family B (MDR/TAP), member 10	chr1	protein_coding
ENSG00000161921.13	2.76407136	4.67692471	5.75E-19	1.29E-17	2.08	2.28	19.78	19.66	CXCL16	chemokine (C-X-C motif) ligand 16	chr17	protein_coding
ENSG00000095002.11	2.61750039	2.06721348	2.39E-14	3.57E-13	0.23	0.21	0.93	2.67	MSH2	mutS homolog 2	chr2	protein_coding
ENSG00000259424.1	3.13606823	-2.5941487	0.00552196	0.01522223	0.08	0	0.86	0.33	RP11-35O15	--	chr15	antisense
ENSG00000279571.1	-1.1529155	-0.2447659	0.0058754	0.01613345	1.04	1.37	0.43	0.99	RP11-56D16	--	chr9	lincRNA
ENSG00000135363.10	2.86874491	-0.0704458	1.23E-09	1.06E-08	0.07	0.07	0.6	0.75	LMO2	LIM domain only 2 (rhombotin-like 1)	chr11	protein_coding
ENSG00000143575.13	-1.072181	7.88635495	0.00019076	0.00072275	208.88	206.15	139.65	122.88	HAX1	HCLS1 associated protein X-1	chr1	protein_coding
ENSG00000150403.16	-1.1796906	5.75596976	4.63E-05	0.00020005	14.18	15.21	7.48	9.86	TMCO3	transmembrane and coiled-coil domains 3	chr13	protein_coding
ENSG00000217128.10	3.0008856	1.07466907	2.06E-14	3.10E-13	0.07	0.04	0.52	0.62	FNIP1	folliculin interacting protein 1	chr5	protein_coding
ENSG00000163001.10	1.83893735	3.28282754	2.47E-09	2.06E-08	1.03	1.17	3.46	7.08	CFAP36	cilia and flagella associated protein 36	chr2	protein_coding
ENSG00000124159.14	-1.8220802	0.02929753	6.24E-06	3.15E-05	0.68	0.32	0.16	0.22	MATN4	matrilin 4	chr20	protein_coding
ENSG00000110619.15	-1.3650799	8.19553131	2.36E-06	1.28E-05	97.87	122.62	60.28	53.45	CARS	cysteinyl-tRNA synthetase	chr11	protein_coding
ENSG00000261079.1	5.89090986	-2.4715586	0.00014842	0.00057423	0	0	0.15	0.06	RP11-252A2	--	chr16	lincRNA
ENSG00000197961.10	3.0112783	1.53612099	7.54E-16	1.29E-14	0.07	0.07	0.61	0.88	ZNF121	zinc finger protein 121	chr19	protein_coding
ENSG00000255717.5	-1.7193627	5.53914764	5.06E-09	4.03E-08	35.85	41.87	15.98	15.42	SNHG1	small nucleolar RNA host gene 1	chr11	processed_transcript
ENSG00000162438.10	2.99046512	-2.67716	0.00943894	0.02469931	0	0.01	0.02	0.08	CTRC	chymotrypsin C (caldecrin)	chr1	protein_coding
ENSG00000279281.1	1.40855936	-1.058981	0.00959833	0.02503712	0.1	0.07	0.23	0.38	RP11-226M1	--	chr17	TEC
ENSG00000172296.11	-2.3223869	-1.732254	0.00065065	0.00222818	0.04	0.07	0.02	0.01	SPTLC3	serine palmitoyltransferase, long chain base subunit 3	chr20	protein_coding
ENSG00000249590.6	-1.2504017	5.98738878	1.60E-05	7.49E-05	53.93	44.34	29.07	25.93	RP4-539M6.	--	chr22	protein_coding
ENSG00000239542.3	4.91020659	-3.0511096	0.0100455	0.02582742	0	0	0.69	0.2	RN7SL399P	RNA, 7SL, cytoplasmic 399, pseudogene	chr17	misc_RNA
ENSG00000055917.14	1.94366467	2.92232503	5.66E-10	5.11E-09	0.35	0.42	1.48	2.48	PUM2	pumilio RNA-binding family member 2	chr2	protein_coding
ENSG00000100852.11	5.41746522	1.66798571	1.57E-31	7.76E-30	0.02	0.01	0.47	0.84	ARHGAP5	Rho GTPase activating protein 5	chr14	protein_coding
ENSG00000186472.18	-2.4348858	-1.1739331	2.56E-05	0.00011557	0.02	0.03	0.01	0	PCLO	piccolo presynaptic cytomatrix protein	chr7	protein_coding
ENSG00000175084.10	-10.391748	1.33998079	2.87E-38	1.97E-36	1.65	2	0	0	DES	desmin	chr2	protein_coding
ENSG00000164252.11	1.20733387	2.49027762	0.00010817	0.00043231	0.48	0.64	1.16	2.31	AGGF1	angiogenic factor with G patch and FHA domains 1	chr5	protein_coding
ENSG00000164825.3	3.52150748	-0.2229685	4.15E-11	4.33E-10	0.11	0.3	3.28	3.08	DEFB1	defensin, beta 1	chr8	protein_coding
ENSG00000163466.14	-1.2987591	7.10875749	7.06E-06	3.53E-05	89.08	95.62	45.28	54.95	ARPC2	actin related protein 2/3 complex, subunit 2, 34kDa	chr2	protein_coding
ENSG00000144152.11	-2.7034292	0.93212677	2.62E-12	3.15E-11	1.28	1.02	0.33	0.14	FBLN7	fibulin 7	chr2	protein_coding
ENSG00000266473.1	3.16098632	-0.0695818	1.16E-10	1.15E-09	0.39	0	1.95	2.89	RP11-401F2.	--	chr17	antisense
ENSG00000275367.1	2.14889956	-1.9337541	0.00653791	0.01776746	0.02	0.02	0.11	0.09	RP11-266K4.	--	chr12	lincRNA
ENSG00000171954.11	9.31006949	0.38193425	1.69E-24	5.60E-23	0	0	1.15	0.8	CYP4F22	cytochrome P450, family 4, subfamily F, polypeptide 22	chr19	protein_coding
ENSG00000065154.10	2.33741221	3.84640604	4.28E-14	6.21E-13	2.14	1.5	11.13	13.5	OAT	ornithine aminotransferase	chr10	protein_coding
ENSG00000237021.2	-3.1767501	-2.8660743	0.01615662	0.03958166	0.04	0.08	0	0.01	RP3-486I3.7	--	chr6	lincRNA
ENSG00000125967.15	1.3713856	5.20248027	2.69E-06	1.45E-05	9.3	6.89	28.32	27.58	NECAB3	N-terminal EF-hand calcium binding protein 3	chr20	protein_coding
ENSG00000237819.4	-6.2754363	-2.3796349	2.70E-05	0.00012094	0.36	0.34	0	0	AC002454.1	--	chr7	antisense
ENSG00000258768.2	3.71972769	-1.5427366	5.39E-06	2.75E-05	0	0.01	0.08	0.2	CTD-2292M	--	chr14	lincRNA
ENSG00000123096.10	1.92969284	-1.6346762	0.00345484	0.01003143	0.02	0.01	0.11	0.07	SSPN	sarcospan	chr12	protein_coding
ENSG00000103175.9	-10.021144	4.93098077	6.40E-86	5.90E-83	24.73	26.98	0.07	0	WFDC1	WAP four-disulfide core domain 1	chr16	protein_coding
ENSG00000251381.5	-7.382432	2.8341824	2.30E-53	3.91E-51	7.76	7	0.04	0.07	LINC00958	long intergenic non-protein coding RNA 958	chr11	lincRNA
ENSG00000026559.12	-1.1202018	4.94091834	0.00011836	0.0004685	15.93	14.84	9.75	9.1	KCNG1	potassium channel, voltage gated modifier subfamily G, member 1	chr20	protein_coding
ENSG00000136603.12	4.85201228	2.16997783	3.08E-32	1.58E-30	0.04	0.03	0.86	1.68	SKIL	SKI-like proto-oncogene	chr3	protein_coding
ENSG00000169139.10	1.30852744	3.25918534	1.50E-05	7.07E-05	0.65	0.83	1.59	3.34	UBE2V2	ubiquitin-conjugating enzyme E2 variant 2	chr8	protein_coding
ENSG00000196504.14	3.01277566	3.44096127	2.24E-20	5.53E-19	0.24	0.24	1.58	3.59	PRPF40A	PRP40 pre-mRNA processing factor 40 homolog A (S. cerevisiae)	chr2	protein_coding
ENSG00000131828.12	-1.5624651	8.55923516	7.60E-08	5.18E-07	119.93	122.61	49.09	60.61	PDHA1	pyruvate dehydrogenase (lipoamide) alpha 1	chrX	protein_coding
ENSG00000150051.12	-1.4937034	0.76006246	3.15E-05	0.00013935	0.39	0.65	0.17	0.32	MKX	mohawk homeobox	chr10	protein_coding
ENSG00000184613.9	-3.4919462	-0.8419799	2.06E-09	1.73E-08	0.23	0.11	0.04	0.01	NELL2	NEL-like 2 (chicken)	chr12	protein_coding
ENSG00000171109.17	2.00032142	1.77051875	3.34E-09	2.74E-08	0.26	0.14	0.92	1.24	MFN1	mitofusin 1	chr3	protein_coding
ENSG00000253368.3	1.11611817	5.23246955	0.00012171	0.00048099	8.55	11.06	28.4	28.15	TRNP1	TMF1-regulated nuclear protein 1	chr1	protein_coding
ENSG00000129493.13	2.25985885	2.78385235	2.03E-12	2.47E-11	0.17	0.31	1.28	1.81	HEATR5A	HEAT repeat containing 5A	chr14	protein_coding
ENSG00000204852.14	1.1514495	3.32925854	0.0001241	0.00048971	1.09	1.42	3.57	3.86	TCTN1	tectonic family member 1	chr12	protein_coding
ENSG00000183837.9	9.62221136	0.67610395	7.68E-28	3.17E-26	0	0	0.95	0.77	PNMA3	paraneoplastic Ma antigen 3	chrX	protein_coding
ENSG00000118292.7	2.64459752	-1.5922696	0.00030126	0.00109685	0.06	0.02	0.26	0.44	C1orf54	chromosome 1 open reading frame 54	chr1	protein_coding
ENSG00000182287.12	2.27783948	1.14352847	5.40E-10	4.89E-09	0.07	0.15	0.33	1.1	AP1S2	adaptor-related protein complex 1, sigma 2 subunit	chrX	protein_coding
ENSG00000172000.6	-4.5671608	1.53278462	1.27E-27	5.17E-26	0.68	0.69	0.03	0.04	ZNF556	zinc finger protein 556	chr19	protein_coding
ENSG00000168658.17	5.06713725	-2.9688687	0.0100455	0.02582742	0	0	0.03	0.02	VWA3B	von Willebrand factor A domain containing 3B	chr2	protein_coding
ENSG00000101255.9	-2.3198084	5.64287198	1.07E-14	1.66E-13	23.48	23.05	7.01	5.37	TRIB3	tribbles pseudokinase 3	chr20	protein_coding
ENSG00000230074.1	-2.1070133	-0.4114229	2.28E-06	1.24E-05	2.01	0.81	0.19	0.66	RP11-195F1	--	chr9	antisense
ENSG00000115421.11	1.41485825	0.75400808	0.00011804	0.00046741	0.1	0.23	0.41	0.77	PAPOLG	poly(A) polymerase gamma	chr2	protein_coding
ENSG00000112200.15	2.80307764	1.19900823	1.82E-13	2.46E-12	0.02	0.04	0.18	0.37	ZNF451	zinc finger protein 451	chr6	protein_coding
ENSG00000123415.13	-1.139242	4.30737009	0.00010631	0.0004256	6.31	4.47	3.5	3.03	SMUG1	single-strand-selective monofunctional uracil-DNA glycosylase 1	chr12	protein_coding
ENSG00000231652.2	2.27579239	-0.7722233	1.80E-05	8.34E-05	0.03	0.11	0.54	0.39	RP11-553A2	--	chr6	antisense
ENSG00000187634.9	4.20862549	7.37917014	1.62E-38	1.14E-36	6.22	4.56	135.35	130.49	SAMD11	sterile alpha motif domain containing 11	chr1	protein_coding
ENSG00000104320.12	2.48180167	0.92918473	1.06E-10	1.06E-09	0.08	0.1	0.51	0.85	NBN	nibrin	chr8	protein_coding
ENSG00000210164.1	3.07860311	-0.5938605	3.07E-08	2.19E-07	1.18	1.67	19.31	13.62	MT-TG	mitochondrially encoded tRNA glycine	chrM	Mt_tRNA
ENSG00000116747.11	2.25539538	0.6574555	1.09E-08	8.31E-08	0.04	0.05	0.25	0.35	TROVE2	TROVE domain family, member 2	chr1	protein_coding
ENSG00000120075.5	-1.7979843	1.35016664	1.20E-07	7.96E-07	1.45	1.67	0.35	0.84	HOXB5	homeobox B5	chr17	protein_coding
ENSG00000182326.13	-1.5354108	6.78410571	1.33E-07	8.82E-07	35.07	37.29	15.4	17.91	C1S	complement component 1, s subcomponent	chr12	protein_coding
ENSG00000011677.11	-10.307839	1.25697965	3.47E-37	2.24E-35	1.08	0.9	0	0	GABRA3	gamma-aminobutyric acid (GABA) A receptor, alpha 3	chrX	protein_coding
ENSG00000150594.6	-8.6949756	-0.3225641	1.70E-19	3.95E-18	0.36	0.31	0	0	ADRA2A	adrenoceptor alpha 2A	chr10	protein_coding
ENSG00000263002.6	2.60483251	0.51810806	4.13E-10	3.82E-09	0.08	0.06	0.42	0.65	ZNF234	zinc finger protein 234	chr19	protein_coding
ENSG00000273373.1	1.2669571	-0.8118157	0.0099917	0.02582742	0.08	0.09	0.11	0.41	RP5-1074L1.	--	chr1	antisense
ENSG00000005812.9	1.19986696	0.97211376	0.00063945	0.00219267	0.28	0.24	0.76	0.86	FBXL3	F-box and leucine-rich repeat protein 3	chr13	protein_coding
ENSG00000156697.11	-1.9748863	6.45429019	2.28E-11	2.45E-10	40.6	47.93	13.36	16.71	UTP14A	UTP14, U3 small nucleolar ribonucleoprotein, homolog A (yeast)	chrX	protein_coding
ENSG00000168291.11	-1.125032	5.91714716	9.96E-05	0.00040127	41.34	40.03	23.62	26.16	PDHB	pyruvate dehydrogenase (lipoamide) beta	chr3	protein_coding
ENSG00000259672.1	10.6901233	1.69741731	8.58E-41	6.88E-39	0	0	10.76	9.68	RP11-69G7.1	--	chr15	lincRNA
ENSG00000272949.1	2.54657281	-0.8472331	6.55E-06	3.29E-05	0.02	0.04	0.3	0.2	RP11-514P8.	--	chr7	processed_transcript
ENSG00000128487.15	1.20264974	6.32699408	3.21E-05	0.00014205	3.32	3.74	10.5	11.16	SPECC1	sperm antigen with calponin homology and coiled-coil domains 1	chr17	protein_coding

ENSG00000164342.11	6.77858562	-1.8168276	2.24E-07	1.42E-06	0	0	0.02	0.12	TLR3	toll-like receptor 3	chr4	protein_coding
ENSG00000104419.13	-1.7363579	8.77889559	2.76E-09	2.29E-08	176.11	114.26	57.64	58.74	NDRG1	N-myc downstream regulated 1	chr8	protein_coding
ENSG00000133657.13	2.63381347	2.51588451	2.43E-15	4.01E-14	0.1	0.16	0.71	1.42	ATP13A3	ATPase type 13A3	chr3	protein_coding
ENSG00000182575.7	1.35127207	-0.762703	0.00520073	0.01456448	0.1	0.06	0.32	0.23	NXPH3	neurexophilin 3	chr17	protein_coding
ENSG00000160336.13	1.61353389	2.18512478	5.41E-07	3.25E-06	0.41	0.34	1.15	1.94	ZNF761	zinc finger protein 761	chr19	protein_coding
ENSG00000269068.1	-4.2389387	-1.547913	2.58E-08	1.86E-07	0.59	1.15	0	0.1	RP11-256I23	--	chr2	antisense
ENSG00000171227.6	-4.3675588	2.54937859	2.57E-31	1.26E-29	5.29	4.93	0.42	0.25	TMEM37	transmembrane protein 37	chr2	protein_coding
ENSG00000164694.15	3.1264535	-2.599436	0.00552196	0.01522223	0	0	0.03	0.02	FNDC1	fibronectin type III domain containing 1	chr6	protein_coding
ENSG00000076716.8	8.02498052	3.55444086	7.87E-61	2.05E-58	0.01	0.02	4.17	5.93	GPC4	glypican 4	chrX	protein_coding
ENSG00000159251.6	2.18258471	0.67872345	1.32E-08	9.91E-08	0.14	0.07	0.8	0.5	ACTC1	actin, alpha, cardiac muscle 1	chr15	protein_coding
ENSG00000135842.15	-1.4995389	3.14545146	8.08E-07	4.72E-06	1.27	1.79	0.72	0.72	FAM129A	family with sequence similarity 129, member A	chr1	protein_coding
ENSG00000261864.1	2.47962798	-1.4569846	0.00017847	0.00068008	0	0.26	1.05	0.89	RP11-67A1.2	--	chr16	processed_transcript
ENSG00000136286.13	-4.9771048	3.5833552	3.56E-42	3.03E-40	5.15	6.4	0.16	0.33	MYO1G	myosin IG	chr7	protein_coding
ENSG00000112851.13	3.54763253	2.19521396	1.86E-22	5.38E-21	0.05	0.08	0.77	1.25	ERBB2IP	erbb2 interacting protein	chr5	protein_coding
ENSG00000026508.15	-2.5094034	8.22362001	6.38E-17	1.20E-15	64.96	69.44	14.22	17.3	CD44	CD44 molecule (Indian blood group)	chr11	protein_coding
ENSG00000172345.12	2.11136883	1.49916872	1.33E-09	1.14E-08	0.13	0.21	0.94	1.02	STARD5	StAR-related lipid transfer (START) domain containing 5	chr15	protein_coding
ENSG00000119599.15	-1.1945509	3.91162176	5.48E-05	0.00023179	4.33	3.69	2.25	2.43	DCAF4	DDB1 and CUL4 associated factor 4	chr14	protein_coding
ENSG00000188549.11	-1.1182496	6.09929626	0.00010825	0.00043256	12.72	15.57	8.74	8.6	C15orf52	chromosome 15 open reading frame 52	chr15	protein_coding
ENSG00000164651.15	3.50986807	1.15572697	5.48E-18	1.13E-16	0.12	0.03	1.17	1.09	SP8	Sp8 transcription factor	chr7	protein_coding
ENSG00000102057.8	-2.3898882	0.81839716	1.68E-10	1.62E-09	0.34	0.49	0.1	0.11	KCND1	potassium channel, voltage gated Shal related subfamily D, member 1	chrX	protein_coding
ENSG00000198589.9	1.23797475	2.61207706	6.49E-05	0.00027159	0.27	0.26	0.74	0.93	LRBA	LPS-responsive vesicle trafficking, beach and anchor containing	chr4	protein_coding
ENSG00000174279.4	5.19748351	-2.8969879	0.00538165	0.01488851	0	0	0.02	0.05	EVX2	even-skipped homeobox 2	chr2	protein_coding
ENSG00000204528.3	1.52664237	-1.5660914	0.01254665	0.03155686	0.18	0.19	0.92	0.55	PSORS1C3	psoriasis susceptibility 1 candidate 3 (non-protein coding)	chr6	sense_intronic
ENSG00000196636.7	1.48650946	-0.3928962	0.00113746	0.00369419	0.15	0.12	0.27	0.74	ACN9	--	chr7	protein_coding
ENSG00000042429.9	1.44693971	3.4036666	1.72E-06	9.58E-06	0.7	0.7	2.17	2.94	MED17	mediator complex subunit 17	chr11	protein_coding
ENSG00000126768.11	-1.1323041	5.53219988	9.39E-05	0.00037948	46.56	46.94	34.01	22.53	TIMM17B	translocase of inner mitochondrial membrane 17 homolog B (yeast)	chrX	protein_coding
ENSG00000116539.9	1.48803691	3.40129156	9.13E-07	5.29E-06	0.33	0.41	1.23	1.53	ASH1L	ash1 (absent, small, or homeotic)-like (Drosophila)	chr1	protein_coding
ENSG00000256223.4	3.43587304	0.16938999	7.48E-13	9.50E-12	0.03	0.02	0.26	0.51	ZNF10	zinc finger protein 10	chr12	protein_coding
ENSG00000156860.14	-1.6328766	6.63702588	2.26E-08	1.64E-07	22.82	24.06	9.86	10.28	FBRS	fibrosin	chr16	protein_coding
ENSG00000183479.11	-2.1876187	5.36255778	2.56E-13	3.41E-12	21.57	18.63	5.84	5.93	TREX2	three prime repair exonuclease 2	chrX	protein_coding
ENSG00000136859.8	-6.9372636	2.61391745	3.66E-49	4.77E-47	3.05	2.26	0.03	0.02	ANGPTL2	angiopoietin-like 2	chr9	protein_coding
ENSG00000128714.5	10.9568763	1.95770539	2.53E-44	2.50E-42	0	0	3.17	4.07	HOXD13	homeobox D13	chr2	protein_coding
ENSG00000196074.11	11.4415678	2.43339741	5.42E-51	7.60E-49	0	0	0.89	3.23	SYCP2	synaptonemal complex protein 2	chr20	protein_coding
ENSG00000150510.14	1.18132276	0.67990553	0.00098837	0.00325143	0.18	0.06	0.42	0.3	FAM124A	family with sequence similarity 124A	chr13	protein_coding
ENSG00000075239.12	1.14873772	5.63624846	7.40E-05	0.00030587	8.99	10.02	23.25	33.11	ACAT1	acetyl-CoA acetyltransferase 1	chr11	protein_coding
ENSG00000152292.15	-4.0964868	-1.6584283	1.57E-07	1.02E-06	0.3	0.06	0	0.02	SH2D6	SH2 domain containing 6	chr2	protein_coding
ENSG00000178502.5	2.74793855	-1.0417457	3.62E-06	1.91E-05	0.07	0	0.17	0.46	KLHL11	kelch-like family member 11	chr17	protein_coding
ENSG00000065559.13	2.52925604	1.81286516	3.90E-13	5.10E-12	0.22	0.2	1.33	1.92	MAP2K4	mitogen-activated protein kinase kinase 4	chr17	protein_coding
ENSG00000135541.19	1.77557306	0.56357812	3.85E-06	2.02E-05	0.11	0.03	0.32	0.32	AHI1	Abelson helper integration site 1	chr6	protein_coding
ENSG00000111530.11	1.61962117	2.95705403	1.66E-07	1.08E-06	0.25	0.29	0.74	1.5	CAND1	cullin-associated and neddylation-dissociated 1	chr12	protein_coding
ENSG00000066455.11	1.63491238	2.56345538	2.23E-07	1.42E-06	0.65	0.9	2.38	4.04	GOLGA5	golgin A5	chr14	protein_coding
ENSG00000114942.12	-1.4663376	9.44565945	4.16E-07	2.56E-06	739.16	744.03	322.5	394.52	EEF1B2	eukaryotic translation elongation factor 1 beta 2	chr2	protein_coding
ENSG00000189280.3	-6.880294	-1.9264137	1.35E-07	8.87E-07	0.32	0.21	0	0	GJB5	gap junction protein, beta 5, 31.1kDa	chr1	protein_coding
ENSG00000147224.9	-1.1151892	6.44013	0.00011057	0.00044008	45.4	38.89	24.18	27.79	PRPS1	phosphoribosyl pyrophosphate synthetase 1	chrX	protein_coding
ENSG00000135678.10	1.08891344	4.46852423	0.00019845	0.00075024	1.5	1.86	3.91	5.65	CPM	carboxypeptidase M	chr12	protein_coding
ENSG00000162971.9	1.63609866	1.0119707	4.56E-06	2.37E-05	0.16	0.19	0.68	0.78	TYW5	tRNA-yW synthesizing protein 5	chr2	protein_coding
ENSG00000154556.16	-7.4643633	2.66728095	4.54E-52	7.03E-50	1.04	1.27	0.01	0.01	SORBS2	sorbin and SH3 domain containing 2	chr4	protein_coding
ENSG00000164778.4	5.27951584	2.7312449	1.42E-38	1.01E-36	0.07	0.08	3.67	4.41	EN2	engrailed homeobox 2	chr7	protein_coding
ENSG00000156304.13	1.18009277	6.01388869	4.57E-05	0.00019745	6.75	8.53	22.93	23.14	SCAF4	SR-related CTD-associated factor 4	chr21	protein_coding
ENSG00000111196.8	-1.2914649	3.16437851	1.82E-05	8.41E-05	2.85	4.58	1.58	2.46	MAGOHB	mago-nashi homolog B (Drosophila)	chr12	protein_coding
ENSG00000115525.15	-1.2008315	0.20376478	0.00163639	0.00511642	0.38	0.61	0.21	0.35	ST3GAL5	ST3 beta-galactoside alpha-2,3-sialyltransferase 5	chr2	protein_coding
ENSG00000172171.9	1.86186066	1.08095243	2.23E-07	1.42E-06	0.19	0.34	1.05	1.49	TEFM	transcription elongation factor, mitochondrial	chr17	protein_coding
ENSG00000271425.5	-1.140937	0.4568563	0.00232121	0.00703197	0.12	0.1	0.07	0.07	NBPF10	neuroblastoma breakpoint family, member 10	chr1	protein_coding
ENSG00000134030.12	-2.5433424	5.28744867	6.96E-17	1.30E-15	9.79	8.72	2.29	1.94	CTIF	CBP80/20-dependent translation initiation factor	chr18	protein_coding
ENSG00000146535.12	-1.5000632	5.71561052	2.80E-07	1.76E-06	12.06	11.26	5.74	5.23	GNA12	guanine nucleotide binding protein (G protein) alpha 12	chr7	protein_coding
ENSG00000083750.11	1.0918254	2.75351021	0.00035207	0.00126837	1.6	1.61	4.03	5.1	RRAGB	Ras-related GTP binding B	chrX	protein_coding
ENSG00000128394.15	5.33137857	1.74453235	6.77E-32	3.40E-30	0.04	0.04	2.35	2.13	APOBEC3F	apolipoprotein B mRNA editing enzyme, catalytic polypeptide-like 3F	chr22	protein_coding
ENSG00000174951.9	-1.534889	2.12823254	1.73E-06	9.60E-06	1.21	1.25	0.66	0.47	FUT1	fucosyltransferase 1 (galactoside 2-alpha-L-fucosyltransferase, H blood group)	chr19	protein_coding
ENSG00000010671.14	4.89980841	-3.0567113	0.01889873	0.04519557	0	0	0.04	0.04	BTK	Bruton agammaglobulinemia tyrosine kinase	chrX	protein_coding
ENSG00000129993.13	2.46987255	-2.4121796	0.00737015	0.01977584	0	0.01	0.06	0.04	CBFA2T3	core-binding factor, runt domain, alpha subunit 2; translocated to, 3	chr16	protein_coding
ENSG00000163739.4	-6.2711019	5.01449677	2.19E-61	5.91E-59	48.86	45.36	0.91	0.72	CXCL1	chemokine (C-X-C motif) ligand 1 (melanoma growth stimulating activity, alpha)	chr4	protein_coding
ENSG00000105245.8	-1.0753194	6.29125205	0.00019251	0.00072881	24.8	22.36	15.13	14.69	NUMBL	numb homolog (Drosophila)-like	chr19	protein_coding
ENSG00000170577.7	-1.9153164	5.21758448	1.06E-10	1.06E-09	21.57	21.62	7.9	7.34	SIX2	SIX homeobox 2	chr2	protein_coding
ENSG00000185585.18	-3.628061	6.35102904	3.40E-30	1.57E-28	18.6	18.27	2.18	1.78	OLFML2A	olfactomedin-like 2A	chr9	protein_coding
ENSG00000103313.10	-5.1885693	2.0827372	8.52E-35	4.91E-33	1.66	1.89	0.12	0.01	MEFV	Mediterranean fever	chr16	protein_coding
ENSG00000124279.10	1.41707021	1.8308152	1.37E-05	6.48E-05	0.69	0.58	1.27	3.29	FASTKD3	FAST kinase domains 3	chr5	protein_coding
ENSG00000261183.4	3.90913678	1.29974692	4.42E-21	1.16E-19	0.06	0.32	4.11	3.56	RP11-532F12	--	chr15	antisense
ENSG00000225914.1	2.16094946	-2.2185025	0.02071392	0.04894569	0.02	0.01	0.03	0.12	XXbac-BPG1	--	chr6	antisense
ENSG00000171517.5	-3.2676308	0.79882133	3.19E-16	5.65E-15	0.85	0.58	0.05	0.14	LPAR3	lysophosphatidic acid receptor 3	chr1	protein_coding
ENSG00000137875.4	8.85305975	-0.0357538	5.29E-20	1.27E-18	0	0	1.26	1.59	BCL2L10	BCL2-like 10 (apoptosis facilitator)	chr15	protein_coding
ENSG00000279838.1	5.56116133	-2.6812008	0.00086564	0.00288128	0	0	0.03	0.15	RP4-635A23	--	chr1	TEC
ENSG00000103061.10	1.17018383	5.0731188	5.85E-05	0.00024637	3.92	4.05	11.18	12.77	SLC7A6OS	solute carrier family 7, member 6 opposite strand	chr16	protein_coding
ENSG00000170153.9	-1.8421834	0.61282935	1.39E-06	7.82E-06	0.19	0.17	0.09	0.05	RNF150	ring finger protein 150	chr4	protein_coding
ENSG00000100314.3	-3.5744564	-1.6255668	7.13E-07	4.20E-06	0.21	0.05	0	0.03	CABP7	calcium binding protein 7	chr22	protein_coding
ENSG00000260630.5	1.67911797	1.49795472	7.90E-07	4.62E-06	0.13	0.29	0.86	0.92	SNAI3-AS1	SNAI3 antisense RNA 1	chr16	antisense
ENSG00000160323.17	1.62668431	0.69124462	1.08E-05	5.19E-05	0.17	0.06	0.41	0.53	ADAMTS13	ADAM metallopeptidase with thrombospondin type 1 motif, 13	chr9	protein_coding
ENSG00000180346.3	1.64194144	0.93718627	5.92E-06	3.00E-05	0.22	0.23	0.98	0.88	TIGD2	tigger transposable element derived 2	chr4	protein_coding
ENSG00000112782.14	2.54777751	-1.657755	0.00044263	0.00156293	0	0.02	0.04	0.08	CLIC5	chloride intracellular channel 5	chr6	protein_coding
ENSG00000100360.13	1.65713615	3.81606508	3.67E-08	2.60E-07	5.56	4.19	20.84	20.2	IFT27	intraflagellar transport 27	chr22	protein_coding
ENSG00000265933.4	-3.8683194	-1.3952246	3.66E-08	2.59E-07	0.3	0.15	0	0.03	LINC00668	long intergenic non-protein coding RNA 668	chr18	lincRNA
ENSG00000262292.2	5.34914687	-2.8103904	0.00290522	0.00856312	0	0	0.13	0.09	RP11-160E2.	--	chr17	lincRNA
ENSG00000275379.1	6.75122422	-1.8502353	2.24E-07	1.42E-06	0	0	1.64	0.53	HIST1H3I	histone cluster 1, H3i	chr6	protein_coding
ENSG00000089157.14	-1.1059935	10.7460935	0.00011725	0.00046483	1119.76	971.19	633.18	662.7	RPLP0	ribosomal protein, large, P0	chr12	protein_coding
ENSG00000111335.11	3.84326481	-0.9974004	1.50E-08	1.12E-07	0	0.01	0.15	0.12	OAS2	2'-5'-oligoadenylate synthetase 2, 69/71kDa	chr12	protein_coding
ENSG00000005001.8	7.18783171	-1.4901423	2.80E-09	2.31E-08	0	0	0.23	0.61	PRSS22	protease, serine, 22	chr16	protein_coding
ENSG00000160284.13	-2.8325435	2.80313188	8.18E-18	1.65E-16	4.36	4.13	0.93	0.65	SPATC1L	spermatogenesis and centriole associated 1-like	chr21	protein_coding
ENSG00000126653.14	1.12851035	3.85044231	0.00014257	0.00055554	1.61	3.34	5.58	8.88	NSRP1	nuclear speckle splicing regulatory protein 1	chr17	protein_coding
ENSG00000160113.5	-1.397999	6.95049913	1.43E-06	8.03E-06	71.34	49.21	31.5	29.5	NR2F6	nuclear receptor subfamily 2, group F, member 6	chr19	protein_coding
ENSG00000062725.8	1.8401536	1.65920555	6.34E-08	4.38E-07	0.09	0.24	0.57	1.03	APPBP2	amyloid beta precursor protein (cytoplasmic tail) binding protein 2	chr17	protein_coding
ENSG00000169570.8	4.37118618	-1.0176981	4.33E-09	3.50E-08	0.01	0	0.07	0.29	DTWD2	DTW domain containing 2	chr5	protein_coding
ENSG00000163694.13	3.00236548	2.01569939	2.17E-17	4.26E-16	0.11	0.14	0.99	1.7	RBM47	RNA binding motif protein 47	chr4	protein_coding
ENSG00000173209.21	1.46273416	4.0215284	9.48E-07	5.48E-06	0.74	1.34	3.01	4.64	AHSA2	AHA1, activator of heat shock 90kDa protein ATPase homolog 2 (yeast)	chr2	protein_coding
ENSG00000165832.5	1.96970366	0.34791619	9.97E-07	5.74E-06	0.05	0.17	0.38	0.82	TRUB1	TruB pseudouridine (psi) synthase family member 1	chr10	protein_coding
ENSG00000084073.7	1.36704266	3.52304937	5.60E-06	2.85E-05	1.24	2.09	4.32	7.15	ZMPSTE24	zinc metallopeptidase STE24	chr1	protein_coding
ENSG00000088826.16	2.43962754	6.47663825	4.75E-16	8.29E-15	9.81	7.97	65.79	62.76	SMOX	spermine oxidase	chr20	protein_coding
ENSG00000094975.12	2.54279926	1.21820233	8.90E-12	1.01E-10	0.06	0.12	0.54	0.8	SUCO	SUN domain containing ossification factor	chr1	protein_coding
ENSG00000184305.13	-5.2127486	-3.0337949	0.00538165	0.01488851	0	0.03	0	0	CCSER1	coiled-coil serine-rich protein 1	chr4	protein_coding
ENSG00000183196.7	-4.2569684	0.75630291	6.20E-21	1.60E-19	0.29	0.32	0.02	0.02	CHST6	carbohydrate (N-acetylglucosamine 6-O) sulfotransferase 6	chr16	protein_coding
ENSG00000203722.6	-3.4755394	3.12945028	5.46E-25	1.89E-23	8.08	9.44	1.14	0.95	RAET1G	retinoic acid early transcript 1G	chr6	protein_coding
ENSG00000038382.16	-1.0659827	6.88895184	0.00021249	0.00079964	10	11.27	6.59	6.95	TRIO	trio Rho guanine nucleotide exchange factor	chr5	protein_coding
ENSG00000184517.10	1.09160447	-0.594109	0.01915356	0.04574946	0.06	0.11	0.16	0.32	ZFP1	ZFP1 zinc finger protein	chr16	protein_coding
ENSG00000101000.4	-1.3206681	3.7469624	9.23E-06	4.51E-05	13.61	9.33	5.54	6.75	PROCR	protein C receptor, endothelial	chr20	protein_coding
ENSG00000187164.16	1.57394177	2.72572124	4.59E-07	2.80E-06	0.27	0.41	0.86	1.84	KIAA1598	KIAA1598	chr10	protein_coding
ENSG00000064300.7	-1.3519378	-0.6272473	0.00387471	0.01114258	0.15	0.24	0.08	0.12	NGFR	nerve growth factor receptor	chr17	protein_coding
ENSG00000156858.10	-1.2300585	5.65330421	2.24E-05	0.00010209	22.67	21.18	12.53	12.37	PRR14	proline rich 14	chr16	protein_coding
ENSG00000184908.16	-7.0925601	-1.7576052	1.16E-08	8.76E-08	0.06	0.22	0	0	CLCNKB	chloride channel, voltage-sensitive Kb	chr1	protein_coding
ENSG00000111615.11	2.37758923	2.78466581	1.99E-13	2.68E-12	0.13	0.22	0.64	1.8	KRR1	KRR1, small subunit (SSU) processome component, homolog (yeast)	chr12	protein_coding
ENSG00000135424.14	3.2782597	2.05029095	1.32E-19	3.07E-18	0.11	0.16	1.75	1.74	ITGA7	integrin, alpha 7	chr12	protein_coding
ENSG00000132749.9	-4.0948487	1.37178663	6.87E-23	2.04E-21	0.83	0.7	0.12	0	MTL5	metallothionein-like 5, testis-specific (tesmin)	chr11	protein_coding
ENSG00000112972.13	2.87613986	4.79857717	2.80E-20	6.86E-19	1.39	1.66	10.32	19.76	HMGCS1	3-hydroxy-3-methylglutaryl-CoA synthase 1 (soluble)	chr5	protein_coding
ENSG00000170340.10	2.96368511	2.25519766	1.50E-17	2.99E-16	0.3	0.3	1.81	4.45	B3GNT2	UDP-GlcNAc:betaGal beta-1,3-N-acetylglucosaminyltransferase 2	chr2	protein_coding
ENSG00000160781.14	1.17277098	1.9661764	0.00027575	0.0010109	0.8	0.78	1.8	2.97	PAQR6	progestin and adipoQ receptor family member VI	chr1	protein_coding
ENSG00000169598.14	-1.1202105	3.61797702	0.00016305	0.00062507	3.73	4.81	2.41	2.82	DFFB	DNA fragmentation factor, 40kDa, beta polypeptide (caspase-activated DNase)	chr1	protein_coding
ENSG00000176390.11	1.21419131	1.78828903	0.00020068	0.00075796	0.49	0.62	1.45	1.99	CRLF3	cytokine receptor-like factor 3	chr17	protein_coding
ENSG00000169129.13	7.76256032	-1.011338	2.22E-11	2.38E-10	0	0	0.22	0.23	AFAP1L2	actin filament associated protein 1-like 2	chr10	protein_coding
ENSG00000239552.2	5.80179215	-2.5306812	0.00026535	0.0009747	0	0	0.42	0.13	HOXB-AS2	HOXB cluster antisense RNA 2	chr17	antisense
ENSG00000250264.1	-1.6009487	5.49802807	4.75E-08	3.32E-07	19.73	20.96	8.86	9.01	XXbac-BPG2	--	chr6	protein_coding
ENSG00000115556.12	2.48862498	-0.8877582	1.19E-05	5.68E-05	0	0.06	0.27	0.21	PLCD4	phospholipase C, delta 4	chr2	protein_coding
ENSG00000276900.1	-1.2288869	1.08695702	0.00034519	0.00124528	0.96	1.32	0.6	0.69	RP11-467L13	--	chr12	antisense
ENSG00000117724.11	2.21731608	4.61419541	2.69E-13	3.57E-12	0.64	0.71	3.12	5.31	CENPF	centromere protein F, 350/400kDa	chr1	protein_coding
ENSG00000058272.14	3.71892168	2.91263509	2.62E-26	9.84E-25	0.09	0.2	1.93	3.08	PPP1R12A	protein phosphatase 1, regulatory subunit 12A	chr12	protein_coding
ENSG00000260836.1	5.67970084	0.11950243	3.73E-19	8.46E-18	0	0.06	2.55	2.2	RP11-152F13	--	chr15	protein_coding
ENSG00000259712.1	-5.3760424	-2.9459444	0.00290522	0.00856312	0.13	0.27	0	0	CTD-2184D3	--	chr15	antisense
ENSG00000069849.9	1.35315273	7.48655053	2.93E-06	1.57E-05	45.93	44.22	129.23	179.01	ATP1B3	ATPase, Na+/K+ transporting, beta 3 polypeptide	chr3	protein_coding
ENSG00000111224.12	2.80132654	-2.2042217	0.00550654	0.01520944	0	0.01	0.07	0.05	PARP11	poly (ADP-ribose) polymerase family, member 11	chr12	protein_coding
ENSG00000130340.13	1.05803708	4.5519936	0.00029311	0.00106971	2.36	3.49	6.54	9.73	SNX9	sorting nexin 9	chr6	protein_coding
ENSG00000187764.10	3.33801382	3.69017239	2.85E-24	9.37E-23	0.18	0.23	3.19	2.41	SEMA4D	sema domain, immunoglobulin domain (Ig), transmembrane domain (TM) and short cytoplasmic domain, (semaphorin) 4D	chr9	protein_coding
ENSG00000278588.1	7.46223261	-1.2715593	7.65E-11	7.77E-10	0	0	2.35	1.43	HIST1H2BI	histone cluster 1, H2bi	chr6	protein_coding
ENSG00000177551.5	8.69196979	-0.1793342	1.90E-18	4.07E-17	0	0	0.15	0.29	NHLH2	nescient helix loop helix 2	chr1	protein_coding
ENSG00000188529.13	1.06005828	4.3568347	0.00030276	0.00110165	1.63	1.89	3.64	6.17	SRSF10	serine/arginine-rich splicing factor 10	chr1	protein_coding
ENSG00000261135.1	1.69849079	0.03635062	5.83E-05	0.00024582	0.36	0.28	1.36	1.45	RP4-798A10	--	chr1	lincRNA
ENSG00000233006.5	-1.7047076	0.04719231	2.89E-05	0.00012907	0.56	0.44	0.23	0.18	AC034220.3	--	chr5	processed_transcript
ENSG00000165507.8	-3.8337954	-1.84623	1.02E-05	4.95E-05	0.28	0.05	0.03	0	C10orf10	chromosome 10 open reading frame 10	chr10	protein_coding
ENSG00000128591.14	-6.2296355	3.44283296	2.91E-52	4.68E-50	1.7	2.1	0.02	0.04	FLNC	filamin C, gamma	chr7	protein_coding
ENSG00000142675.16	-4.2402141	1.37562219	4.90E-25	1.71E-23	1.32	1.4	0.08	0.11	CNKSR1	connector enhancer of kinase suppressor of Ras 1	chr1	protein_coding
ENSG00000271430.1	1.90356765	0.01772089	6.72E-06	3.38E-05	0.13	0.03	0.49	0.32	RP3-368A4.5	--	chrX	sense_intronic
ENSG00000277075.1	1.59496369	1.91109416	1.15E-06	6.53E-06	3.06	4.47	17.93	12.26	HIST1H2AE	histone cluster 1, H2ae	chr6	protein_coding
ENSG00000278862.1	3.03431301	-2.6531591	0.00552196	0.01522223	0	0.01	0.1	0.01	RP11-571O6	--	chr16	TEC
ENSG00000164631.17	1.10893683	0.20756395	0.00521719	0.01460886	0.11	0.1	0.14	0.47	ZNF12	zinc finger protein 12	chr7	protein_coding
ENSG00000236255.1	1.40798179	-0.5752327	0.00315915	0.0092513	0.05	0.12	0.26	0.34	AC009404.2	--	chr2	lincRNA
ENSG00000242516.1	-6.7081884	-2.0613188	6.25E-07	3.72E-06	0.13	0.17	0	0	LINC00960	long intergenic non-protein coding RNA 960	chr3	lincRNA
ENSG00000123146.18	-2.6617069	8.22935636	1.24E-18	2.69E-17	122.79	118.34	26.45	24.27	CD97	--	chr19	protein_coding
ENSG00000214413.6	1.29104299	0.14956871	0.00146751	0.00467247	0.21	0.19	0.42	0.91	BBIP1	BBSome interacting protein 1	chr10	protein_coding
ENSG00000177108.5	6.11073974	-2.3182927	4.74E-05	0.00020301	0	0	0.07	0.1	ZDHHC22	zinc finger, DHHC-type containing 22	chr14	protein_coding
ENSG00000177679.15	-1.1446344	1.58185422	0.00041832	0.00148473	1.18	0.64	0.4	0.7	SRRM3	serine/arginine repetitive matrix 3	chr7	protein_coding
ENSG00000122550.16	2.192734	2.44063212	1.54E-11	1.69E-10	0.2	0.27	1.22	1.67	KLHL7	kelch-like family member 7	chr7	protein_coding
ENSG00000085276.16	-7.1777335	-1.6856402	4.48E-09	3.59E-08	0.08	0.06	0	0	MECOM	MDS1 and EVI1 complex locus	chr3	protein_coding
ENSG00000104915.13	-1.5554126	7.73643356	8.86E-08	5.97E-07	189.33	188.14	83.59	87.62	STX10	syntaxin 10	chr19	protein_coding
ENSG00000178202.11	4.89648025	4.43567859	1.94E-43	1.83E-41	0.25	0.23	6.62	12.84	KDELC2	KDEL (Lys-Asp-Glu-Leu) containing 2	chr11	protein_coding
ENSG00000149289.9	1.71880962	0.10325384	3.69E-05	0.00016155	0.05	0.03	0.11	0.22	ZC3H12C	zinc finger CCCH-type containing 12C	chr11	protein_coding
ENSG00000167578.15	1.12296185	4.38074345	0.00013104	0.00051512	6.81	6.85	20.67	18.91	RAB4B	RAB4B, member RAS oncogene family	chr19	protein_coding
ENSG00000272948.2	-2.1074317	-1.3988694	0.0004955	0.00173152	0.6	0.64	0.27	0.11	AP001412.1	--	chr21	antisense
ENSG00000256546.1	8.47400405	-0.3836556	1.22E-16	2.23E-15	0	0	1.69	1.56	AC156455.1	--	chr12	processed_transcript
ENSG00000122733.12	2.65660885	-2.2979643	0.00281991	0.00840887	0	0.01	0.07	0.02	KIAA1045	KIAA1045	chr9	protein_coding
ENSG00000176273.13	2.25672396	0.86867289	3.00E-09	2.47E-08	0.15	0.12	0.56	1.21	SLC35G1	solute carrier family 35, member G1	chr10	protein_coding
ENSG00000174718.10	4.69148059	0.37498852	3.16E-18	6.61E-17	0.02	0	0.25	0.55	KIAA1551	KIAA1551	chr12	protein_coding
ENSG00000272886.4	1.46057349	3.97834604	9.57E-07	5.52E-06	1.1	1.15	4.21	4.06	DCP1A	decapping mRNA 1A	chr3	protein_coding
ENSG00000076641.4	1.21819792	-0.6406202	0.01010345	0.025949	0.03	0.02	0.09	0.07	PAG1	phosphoprotein membrane anchor with glycosphingolipid microdomains 1	chr8	protein_coding
ENSG00000164163.9	1.18531592	2.61056307	0.00013156	0.00051708	0.75	0.77	1.24	3.4	ABCE1	ATP-binding cassette, sub-family E (OABP), member 1	chr4	protein_coding
ENSG00000181016.8	1.47511803	-1.0110878	0.00587472	0.01613343	0.04	0.14	0.29	0.38	LSMEM1	leucine-rich single-pass membrane protein 1	chr7	protein_coding
ENSG00000205403.11	7.24773016	-1.4458174	1.11E-09	9.61E-09	0	0	0.24	0.28	CFI	complement factor I	chr4	protein_coding
ENSG00000179528.14	2.40783803	1.20091876	7.03E-11	7.19E-10	0.42	0.19	2.22	2.14	LBX2	ladybird homeobox 2	chr2	protein_coding
ENSG00000077585.12	2.00398316	1.500893	6.47E-09	5.11E-08	0.43	0.43	2.21	2.41	GPR137B	G protein-coupled receptor 137B	chr1	protein_coding
ENSG00000072163.17	-3.2092253	3.33345349	1.26E-22	3.65E-21	3.51	3.54	0.47	0.55	LIMS2	LIM and senescent cell antigen-like domains 2	chr2	protein_coding
ENSG00000079335.16	3.53749611	-0.9050176	2.19E-08	1.59E-07	0.01	0.01	0.18	0.2	CDC14A	cell division cycle 14A	chr1	protein_coding
ENSG00000166510.12	1.64191197	-1.4864297	0.0092368	0.02430566	0.02	0.03	0	0.21	CCDC68	coiled-coil domain containing 68	chr18	protein_coding
ENSG00000196562.13	-4.9517613	6.51707202	4.27E-48	5.33E-46	29.38	28.73	1.31	1.18	SULF2	sulfatase 2	chr20	protein_coding
ENSG00000078747.11	2.74805698	3.01635891	3.87E-17	7.40E-16	0.2	0.31	1.71	2.89	ITCH	itchy E3 ubiquitin protein ligase	chr20	protein_coding
ENSG00000262660.1	-1.0563302	4.66007559	0.00028998	0.00105871	15.27	13.45	9.62	8.77	SLC25A10	solute carrier family 25 (mitochondrial carrier; dicarboxylate transporter), member 10	chr17	protein_coding
ENSG00000151304.5	2.4998434	-0.362697	2.51E-07	1.58E-06	0.04	0.08	0.29	0.59	SRFBP1	serum response factor binding protein 1	chr5	protein_coding
ENSG00000196345.11	-7.3153474	-1.5731293	1.11E-09	9.61E-09	0.04	0.21	0	0	ZKSCAN7	zinc finger with KRAB and SCAN domains 7	chr3	protein_coding
ENSG00000198056.12	1.33278993	2.59082525	1.87E-05	8.62E-05	1.82	2.1	5.27	7.94	PRIM1	primase, DNA, polypeptide 1 (49kDa)	chr12	protein_coding
ENSG00000272696.1	-1.2297317	-1.4572541	0.02009733	0.04771922	0.47	0.79	0	0.67	RP11-339B2	--	chr9	antisense
ENSG00000116731.21	1.81061325	3.6648423	2.64E-09	2.19E-08	0.45	0.49	2.02	2.37	PRDM2	PR domain containing 2, with ZNF domain	chr1	protein_coding
ENSG00000204186.6	3.64123023	-0.5506248	2.56E-10	2.42E-09	0.01	0.01	0.14	0.09	ZDBF2	zinc finger, DBF-type containing 2	chr2	protein_coding
ENSG00000188266.12	1.58400413	1.68961182	2.12E-06	1.16E-05	0.24	0.27	1	1.03	HYKK	hydroxylysine kinase	chr15	protein_coding
ENSG00000272986.1	5.88171008	-2.4767444	0.00014842	0.00057423	0	0	0.39	0.26	RP11-46J23.	--	chr4	antisense
ENSG00000081059.18	-4.1143239	5.25493136	9.09E-36	5.59E-34	13.1	15.31	1.03	1.15	TCF7	transcription factor 7 (T-cell specific, HMG-box)	chr5	protein_coding
ENSG00000132874.12	3.24322299	-2.5296985	0.003237	0.00945156	0.01	0	0.06	0.02	SLC14A2	solute carrier family 14 (urea transporter), member 2	chr18	protein_coding
ENSG00000166963.11	2.92876715	2.78991762	1.78E-18	3.81E-17	0.15	0.09	1.36	1.07	MAP1A	microtubule-associated protein 1A	chr15	protein_coding
ENSG00000163625.14	1.35721684	2.39410385	1.57E-05	7.36E-05	0.13	0.2	0.55	0.58	WDFY3	WD repeat and FYVE domain containing 3	chr4	protein_coding

ENSG00000259065.1	-2.6125167	0.21631821	5.19E-10	4.71E-09	2.33	2.27	0.65	0.36	RP5-1021I20	--	chr14	antisense
ENSG00000183691.4	-3.1945347	2.88444595	2.03E-21	5.45E-20	6.12	5.26	1.08	0.58	NOG	noggin	chr17	protein_coding
ENSG00000145687.14	-1.7375918	3.44781891	1.18E-08	8.90E-08	1.61	1.47	0.65	0.58	SSBP2	single-stranded DNA binding protein 2	chr5	protein_coding
ENSG00000278869.1	2.2389086	1.70853109	9.58E-11	9.61E-10	1.01	1.03	7.2	5.65	CITF22-49E9	--	chr22	lincRNA
ENSG00000021762.18	-2.2639252	7.20403621	2.95E-14	4.36E-13	47.57	40.44	12.63	11.78	OSBPL5	oxysterol binding protein-like 5	chr11	protein_coding
ENSG00000271533.1	6.32244873	-2.1673809	8.95E-06	4.38E-05	0	0	0.08	0.1	RP3-368A4.6	--	chrX	sense_intronic
ENSG00000154957.12	1.38315168	2.05027513	1.65E-05	7.67E-05	0.65	0.62	2.02	2.41	ZNF18	zinc finger protein 18	chr17	protein_coding
ENSG00000137124.6	-2.3414267	5.58858175	6.31E-15	1.00E-13	20.87	21.58	5.22	5.95	ALDH1B1	aldehyde dehydrogenase 1 family, member B1	chr9	protein_coding
ENSG00000261594.3	3.74794731	0.67688303	1.63E-16	2.97E-15	0.06	0.06	1.28	0.87	TPBGL	trophoblast glycoprotein-like	chr11	protein_coding
ENSG00000114107.7	2.58892262	1.76717416	2.15E-13	2.88E-12	0.24	0.23	1.06	2.69	CEP70	centrosomal protein 70kDa	chr3	protein_coding
ENSG00000121769.6	1.90906809	1.33385801	5.66E-08	3.92E-07	1.36	0.9	5.7	5.71	FABP3	fatty acid binding protein 3, muscle and heart	chr1	protein_coding
ENSG00000222033.1	2.72783277	1.71624449	2.21E-14	3.30E-13	0.42	0.21	2.59	3.05	LINC01124	long intergenic non-protein coding RNA 1124	chr2	lincRNA
ENSG00000164306.9	1.10180247	0.55889492	0.00332263	0.00968508	0.31	0.31	0.68	1.1	PRIMPOL	primase and polymerase (DNA-directed)	chr4	protein_coding
ENSG00000172403.9	-5.6565858	-2.7839092	0.00086564	0.00288128	0.01	0.01	0	0	SYNPO2	synaptopodin 2	chr4	protein_coding
ENSG00000106348.15	-2.0260563	7.50541747	6.74E-12	7.72E-11	81.04	77.56	27.93	23.85	IMPDH1	IMP (inosine 5'-monophosphate) dehydrogenase 1	chr7	protein_coding
ENSG00000136114.14	6.72961289	1.09478477	2.48E-30	1.16E-28	0.02	0	1.26	1.32	THSD1	thrombospondin, type I, domain containing 1	chr13	protein_coding
ENSG00000262514.1	5.04680446	-2.9799228	0.0100455	0.02582742	0	0	0.03	0.18	RP11-96D1.9	--	chr16	sense_intronic
ENSG00000166266.12	3.7399004	1.62213116	1.76E-21	4.78E-20	0.05	0.05	0.53	1.35	CUL5	cullin 5	chr11	protein_coding
ENSG00000136999.4	-1.3520917	2.38902067	1.44E-05	6.83E-05	2.16	2.65	1.05	1.46	NOV	nephroblastoma overexpressed	chr8	protein_coding
ENSG00000169567.10	-1.5343835	6.83887309	1.35E-07	8.90E-07	88.36	86.35	37.29	43.22	HINT1	histidine triad nucleotide binding protein 1	chr5	protein_coding
ENSG00000122068.11	3.10696464	1.9302798	8.23E-18	1.66E-16	0.09	0.07	0.57	1.33	FYTTD1	forty-two-three domain containing 1	chr3	protein_coding
ENSG00000163993.6	4.3369288	7.27370165	2.68E-40	2.08E-38	9.43	9.02	258.54	237.87	S100P	S100 calcium binding protein P	chr4	protein_coding
ENSG00000170264.11	1.5748519	0.56702198	3.53E-05	0.00015536	0.1	0.19	0.5	0.67	FAM161A	family with sequence similarity 161, member A	chr2	protein_coding
ENSG00000214212.7	2.38200669	-0.1715884	2.77E-07	1.73E-06	0.04	0.26	1.47	0.59	C19orf38	chromosome 19 open reading frame 38	chr19	protein_coding
ENSG00000104903.4	8.99244885	2.43932117	9.12E-50	1.24E-47	0.02	0	8.43	6.85	LYL1	lymphoblastic leukemia associated hematopoiesis regulator 1	chr19	protein_coding
ENSG00000164332.7	1.69560089	2.42906249	1.07E-07	7.18E-07	0.64	1.05	2.6	4.72	UBLCP1	ubiquitin-like domain containing CTD phosphatase 1	chr5	protein_coding
ENSG00000092445.10	-1.173776	6.89250657	4.73E-05	0.00020301	15.17	14.42	8.99	8.48	TYRO3	TYRO3 protein tyrosine kinase	chr15	protein_coding
ENSG00000123200.15	1.77307912	4.13754395	3.77E-09	3.07E-08	0.51	0.81	2.29	3.77	ZC3H13	zinc finger CCCH-type containing 13	chr13	protein_coding
ENSG00000173068.16	-1.5073168	0.38734331	8.90E-05	0.00036046	0.13	0.09	0.06	0.04	BNC2	basonuclin 2	chr9	protein_coding
ENSG00000203644.3	5.66409976	-2.616209	0.00086564	0.00288128	0	0	0.07	0.36	RP11-332M2	--	chr3	sense_intronic
ENSG00000131437.14	3.19531619	1.13664989	9.26E-16	1.58E-14	0.06	0.04	0.4	0.87	KIF3A	kinesin family member 3A	chr5	protein_coding
ENSG00000250105.1	5.78313448	-2.5411454	0.00026535	0.0009747	0	0	0.39	0.38	CTD-3074O7	--	chr11	lincRNA
ENSG00000131459.11	-2.2774617	5.46140261	3.05E-14	4.49E-13	19.35	18.88	5.21	5.3	GFPT2	glutamine-fructose-6-phosphate transaminase 2	chr5	protein_coding
ENSG00000213213.12	1.17711416	0.70882343	0.00120728	0.00390302	0.5	0.42	1.4	1.39	CCDC183	coiled-coil domain containing 183	chr9	protein_coding
ENSG00000144749.12	-2.3705185	4.74306936	6.29E-15	1.00E-13	6.63	6.77	1.64	1.81	LRIG1	leucine-rich repeats and immunoglobulin-like domains 1	chr3	protein_coding
ENSG00000077684.14	-1.1592459	4.44404242	7.54E-05	0.00031148	2.83	2.69	1.37	1.94	JADE1	jade family PHD finger 1	chr4	protein_coding
ENSG00000068120.13	-1.0488608	7.51466405	0.00026254	0.00097122	84.45	76.57	53.05	50.62	COASY	CoA synthase	chr17	protein_coding
ENSG00000122042.9	1.2882415	-0.2498987	0.00275034	0.008227	0.09	0.07	0.19	0.34	UBL3	ubiquitin-like 3	chr13	protein_coding
ENSG00000139174.9	-1.9817137	-0.8265991	0.0001213	0.00047961	0.09	0.12	0.07	0.01	PRICKLE1	prickle homolog 1 (Drosophila)	chr12	protein_coding
ENSG00000210112.1	3.86810358	1.17551407	4.12E-20	9.98E-19	2.75	3.75	71.13	56.47	MT-TM	mitochondrially encoded tRNA methionine	chrM	Mt_tRNA
ENSG00000261578.1	-1.1900474	1.32605697	0.0003995	0.00142279	0.61	0.72	0.4	0.37	RP11-21L23.	--	chr11	sense_overlapping
ENSG00000102024.16	1.30050304	4.1336024	1.12E-05	5.40E-05	2.26	2.26	5.1	9.84	PLS3	plastin 3	chrX	protein_coding
ENSG00000227543.4	1.69850871	-0.9650194	0.00166616	0.00519662	0.07	0.04	0.24	0.21	SPAG5-AS1	SPAG5 antisense RNA 1	chr17	processed_transcript
ENSG00000254806.4	-2.4260093	4.05485804	3.53E-15	5.71E-14	35.08	32.21	7.89	8.81	SYS1-DBNDI	SYS1-DBNDD2 readthrough (NMD candidate)	chr20	protein_coding
ENSG00000198265.10	2.92417357	2.43663787	9.86E-18	1.99E-16	0.04	0.1	0.59	0.87	HELZ	helicase with zinc finger	chr17	protein_coding
ENSG00000128590.4	2.55143016	0.79980108	7.51E-11	7.65E-10	0.16	0.13	1.04	1.19	DNAJB9	DnaJ (Hsp40) homolog, subfamily B, member 9	chr7	protein_coding
ENSG00000102181.18	-1.0768811	6.35962194	0.00018815	0.00071331	25.93	22.2	16.62	13.73	CD99L2	CD99 molecule-like 2	chrX	protein_coding
ENSG00000084207.14	-1.2176386	8.44089418	2.38E-05	0.0001077	441.06	373.76	245.11	221.41	GSTP1	glutathione S-transferase pi 1	chr11	protein_coding
ENSG00000272172.1	1.42076122	1.28655538	3.57E-05	0.00015683	3.72	5.59	18.96	14.17	RP13-582O9	--	chr8	antisense
ENSG00000163596.15	2.83946394	-0.2084508	5.49E-09	4.37E-08	0.02	0.01	0.13	0.19	ICA1L	islet cell autoantigen 1,69kDa-like	chr2	protein_coding
ENSG00000179119.13	1.21043648	2.0731851	0.00015014	0.00058036	0.33	0.37	0.81	1.36	SPTY2D1	SPT2, Suppressor of Ty, domain containing 1 (S. cerevisiae)	chr11	protein_coding
ENSG00000149591.15	-4.0515999	3.96436347	4.97E-33	2.67E-31	10.08	9.78	0.88	0.71	TAGLN	transgelin	chr11	protein_coding
ENSG00000165806.18	1.03826841	3.49083653	0.00050862	0.00177434	1.73	2.1	4.27	6.25	CASP7	caspase 7, apoptosis-related cysteine peptidase	chr10	protein_coding
ENSG00000142046.13	1.65318399	1.2926326	1.65E-06	9.19E-06	0.43	0.25	1.4	1.47	TMEM91	transmembrane protein 91	chr19	protein_coding
ENSG00000122035.6	3.51256314	1.41830209	4.34E-19	9.80E-18	0.1	0.33	3.32	3.26	RASL11A	RAS-like, family 11, member A	chr13	protein_coding
ENSG00000198768.9	-6.1863178	-2.4412039	4.74E-05	0.00020301	0.09	0.03	0	0	APCDD1L	adenomatosis polyposis coli down-regulated 1-like	chr20	protein_coding
ENSG00000163825.3	8.58026618	-0.2838411	1.46E-17	2.91E-16	0	0	0.99	1.36	RTP3	receptor (chemosensory) transporter protein 3	chr3	protein_coding
ENSG00000198535.5	6.75188369	-1.8412616	2.24E-07	1.42E-06	0	0	0.08	0.17	C2CD4A	C2 calcium-dependent domain containing 4A	chr15	protein_coding
ENSG00000196968.9	1.65589596	3.43038405	5.22E-08	3.63E-07	2.05	1.77	7.62	8.46	FUT11	fucosyltransferase 11 (alpha (1,3) fucosyltransferase)	chr10	protein_coding
ENSG00000092148.11	1.95837886	4.09487193	1.03E-10	1.03E-09	0.53	0.66	2.81	3.33	HECTD1	HECT domain containing E3 ubiquitin protein ligase 1	chr14	protein_coding
ENSG00000173898.10	-2.8931668	8.05932093	2.43E-21	6.52E-20	45.29	45.06	7.99	8.21	SPTBN2	spectrin, beta, non-erythrocytic 2	chr11	protein_coding
ENSG00000198740.7	2.26623673	1.47295989	1.24E-10	1.23E-09	0.12	0.12	0.74	0.8	ZNF652	zinc finger protein 652	chr17	protein_coding
ENSG00000254142.2	-6.0973213	-2.5024874	8.36E-05	0.00034052	0.27	0.25	0	0	RP11-53M11	--	chr8	lincRNA
ENSG00000183092.14	-1.7841769	4.44636084	2.33E-09	1.94E-08	4.8	4.26	1.93	1.57	BEGAIN	brain-enriched guanylate kinase-associated	chr14	protein_coding
ENSG00000157654.16	-2.776427	4.53243492	4.13E-19	9.35E-18	4.01	4.71	0.9	0.79	PALM2-AKAI	PALM2-AKAP2 readthrough	chr9	protein_coding
ENSG00000134256.11	-1.918161	-0.1459321	6.95E-06	3.48E-05	0.26	0.38	0.06	0.16	CD101	CD101 molecule	chr1	protein_coding
ENSG00000273199.1	2.24206911	-0.0388459	4.29E-07	2.62E-06	0.12	0.45	2.15	1.46	AP000692.10	--	chr21	antisense
ENSG00000186399.10	6.02248524	-2.3782733	8.36E-05	0.00034052	0	0	0.07	0.2	GOLGA8R	golgin A8 family, member R	chr15	protein_coding
ENSG00000137872.14	2.44096527	-1.4836086	0.00036944	0.00132576	0.01	0	0.03	0.11	SEMA6D	sema domain, transmembrane domain (TM), and cytoplasmic domain, (semaphorin) 6D	chr15	protein_coding
ENSG00000135250.15	1.15941844	3.87401976	9.23E-05	0.00037325	1.59	2.05	4.8	6.04	SRPK2	SRSF protein kinase 2	chr7	protein_coding
ENSG00000175354.17	1.00841712	3.62682046	0.00069193	0.00235542	1.32	1.73	3.18	5.02	PTPN2	protein tyrosine phosphatase, non-receptor type 2	chr18	protein_coding
ENSG00000104267.8	-7.3059275	-1.5785321	1.11E-09	9.61E-09	0.33	0.23	0	0	CA2	carbonic anhydrase II	chr8	protein_coding
ENSG00000142552.6	-5.1203676	4.60192693	6.15E-47	7.23E-45	22.65	18.46	0.83	0.75	RCN3	reticulocalbin 3, EF-hand calcium binding domain	chr19	protein_coding
ENSG00000152348.14	-1.6067757	1.80643069	7.84E-07	4.59E-06	0.95	0.96	0.28	0.55	ATG10	autophagy related 10	chr5	protein_coding
ENSG00000135436.7	1.75871785	-0.5609444	0.00021339	0.0008028	0.09	0.01	0.23	0.23	FAM186B	family with sequence similarity 186, member B	chr12	protein_coding
ENSG00000153140.7	1.47073509	1.39218957	1.74E-05	8.08E-05	0.41	0.28	0.66	1.94	CETN3	centrin, EF-hand protein, 3	chr5	protein_coding
ENSG00000169136.7	1.95623338	7.4945783	3.12E-11	3.32E-10	21.59	24.88	119.77	120.36	ATF5	activating transcription factor 5	chr19	protein_coding
ENSG00000114812.11	-3.9448273	1.31412037	1.15E-22	3.35E-21	0.98	1.27	0.1	0.1	VIPR1	vasoactive intestinal peptide receptor 1	chr3	protein_coding
ENSG00000258938.1	2.06374041	1.86237614	1.03E-09	9.01E-09	0.35	0.43	1.89	2.41	RP11-317N8	--	chr14	antisense
ENSG00000148297.14	-1.1040917	6.02532368	0.00013255	0.00052072	10.18	10.17	6.85	5.74	MED22	mediator complex subunit 22	chr9	protein_coding
ENSG00000261455.1	1.74445258	1.32169	4.76E-07	2.89E-06	0.61	0.4	1.58	2.96	LINC01003	long intergenic non-protein coding RNA 1003	chr7	lincRNA
ENSG00000111790.12	1.06931657	2.3444424	0.00064347	0.00220517	0.69	0.92	1.4	3.13	FGFR1OP2	FGFR1 oncogene partner 2	chr12	protein_coding
ENSG00000181350.10	2.98995831	1.4356477	1.52E-15	2.55E-14	0.2	0.13	1.51	2.08	LRRC75A	leucine rich repeat containing 75A	chr17	protein_coding
ENSG00000115109.12	2.21681123	1.14347109	1.37E-09	1.18E-08	0.11	0.03	0.37	0.55	EPB41L5	erythrocyte membrane protein band 4.1 like 5	chr2	protein_coding
ENSG00000248712.6	2.13912511	0.19845106	2.82E-07	1.77E-06	0.28	0.06	1.13	0.96	CCDC153	coiled-coil domain containing 153	chr11	protein_coding
ENSG00000066379.13	-1.7163787	4.10492688	1.02E-08	7.82E-08	28.17	21.16	9.97	10.05	ZNRD1	zinc ribbon domain containing 1	chr6	protein_coding
ENSG00000174080.9	1.57353584	6.80470301	6.76E-08	4.65E-07	23.59	21.08	88.87	88.37	CTSF	cathepsin F	chr11	protein_coding
ENSG00000212283.1	6.07152863	-2.3503156	4.74E-05	0.00020301	0	0	4.21	0.72	SNORD89	small nucleolar RNA, C/D box 89	chr2	snoRNA
ENSG00000142227.9	-1.7379389	6.91448781	2.92E-09	2.41E-08	166.43	153.5	66.56	61.13	EMP3	epithelial membrane protein 3	chr19	protein_coding
ENSG00000164961.14	1.02837384	3.12615956	0.00066321	0.0022654	1	1.2	2.52	3.47	KIAA0196	KIAA0196	chr8	protein_coding
ENSG00000152953.11	-8.8642873	2.84615682	5.39E-59	1.21E-56	3.12	2.94	0	0.01	STK32B	serine/threonine kinase 32B	chr4	protein_coding
ENSG00000179542.14	-6.9346651	-1.8833141	8.18E-08	5.55E-07	0.05	0.03	0	0	SLITRK4	SLIT and NTRK-like family, member 4	chrX	protein_coding
ENSG00000184164.13	1.13973146	7.50424696	7.56E-05	0.00031215	64.01	52.2	176.21	164.98	CRELD2	cysteine-rich with EGF-like domains 2	chr22	protein_coding
ENSG00000243279.3	-1.324709	6.74234334	4.78E-06	2.47E-05	63.58	79.29	40.59	35.17	PRAF2	PRA1 domain family, member 2	chrX	protein_coding
ENSG00000136274.8	-6.4613173	0.46547662	4.69E-26	1.72E-24	0.68	0.23	0	0.01	NACAD	NAC alpha domain containing	chr7	protein_coding
ENSG00000144029.10	-1.2601513	7.7377453	1.26E-05	6.01E-05	132.4	163.07	78.39	85.98	MRPS5	mitochondrial ribosomal protein S5	chr2	protein_coding
ENSG00000095596.10	5.74383254	-0.6068919	1.81E-13	2.45E-12	0.01	0	0.43	0.54	CYP26A1	cytochrome P450, family 26, subfamily A, polypeptide 1	chr10	protein_coding
ENSG00000152402.9	5.81452118	2.20236674	4.45E-38	3.03E-36	0.01	0	0.53	0.63	GUCY1A2	guanylate cyclase 1, soluble, alpha 2	chr11	protein_coding
ENSG00000180818.4	-3.1900656	6.08908489	1.33E-24	4.47E-23	46.54	56.2	8.31	6.64	HOXC10	homeobox C10	chr12	protein_coding
ENSG00000166796.10	6.97966347	-1.6625683	1.87E-08	1.38E-07	0	0	0.2	0.29	LDHC	lactate dehydrogenase C	chr11	protein_coding
ENSG00000163710.6	-2.1553733	2.99416647	5.75E-12	6.65E-11	4.67	5.76	0.97	2.16	PCOLCE2	procollagen C-endopeptidase enhancer 2	chr3	protein_coding
ENSG00000261253.2	-1.2923528	-0.7828076	0.00520073	0.01456448	0.61	0.29	0.14	0.34	AC137932.6	--	chr16	antisense
ENSG00000245571.5	2.39045294	-0.6835125	5.94E-06	3.01E-05	0.04	0.09	0.43	0.5	AP001258.4	--	chr11	lincRNA
ENSG00000176542.8	1.78254082	0.33357274	6.94E-06	3.47E-05	0.04	0.02	0.14	0.15	KIAA2018	KIAA2018	chr3	protein_coding
ENSG00000177096.7	-4.1118658	4.49204574	1.03E-34	5.86E-33	15.72	13.79	1.08	1.19	FAM109B	family with sequence similarity 109, member B	chr22	protein_coding
ENSG00000152082.12	-1.0579458	7.05501172	0.00023559	0.0008798	148.94	112.01	87.84	79.15	MZT2B	mitotic spindle organizing protein 2B	chr2	protein_coding
ENSG00000137868.17	-7.8395576	5.58332745	6.27E-80	4.17E-77	13.16	15.07	0.11	0.06	STRA6	stimulated by retinoic acid 6	chr15	protein_coding
ENSG00000168495.11	-2.0457968	6.47467498	4.85E-12	5.64E-11	20.19	21.41	6.83	6.58	POLR3D	polymerase (RNA) III (DNA directed) polypeptide D, 44kDa	chr8	protein_coding
ENSG00000065534.17	5.87132694	4.64850586	6.38E-55	1.19E-52	0.04	0.05	3.37	3.62	MYLK	myosin light chain kinase	chr3	protein_coding
ENSG00000125968.8	2.24234673	9.45384483	4.37E-14	6.33E-13	184.46	136.37	1069.28	953.04	ID1	inhibitor of DNA binding 1, dominant negative helix-loop-helix protein	chr20	protein_coding
ENSG00000274245.1	-2.080304	-1.3251795	0.00025593	0.00094973	0.58	0.78	0	0.39	RP11-357P18	--	chr1	lincRNA
ENSG00000223382.3	4.91020659	-3.0511096	0.0100455	0.02582742	0	0	0.17	0.05	RP1-65J11.1	--	chr1	lincRNA
ENSG00000270171.1	5.33927207	-2.8158158	0.00290522	0.00856312	0	0	0.07	0.11	RP11-338N1	--	chr1	lincRNA
ENSG00000046653.13	-3.9419989	-1.3356177	1.61E-08	1.20E-07	0.14	0.07	0	0.02	GPM6B	glycoprotein M6B	chrX	protein_coding
ENSG00000179981.9	-1.804838	3.73809996	2.37E-09	1.97E-08	3.02	3.22	1.04	1.34	TSHZ1	teashirt zinc finger homeobox 1	chr18	protein_coding
ENSG00000227896.2	1.00930856	1.4054402	0.00250915	0.00755266	0.39	0.51	1.08	1.34	RP11-77P6.2	--	chr10	antisense
ENSG00000271303.1	-1.1133644	5.8361891	0.00011776	0.00046659	23.51	23.36	13.56	15.35	SRXN1	sulfiredoxin 1	chr20	protein_coding
ENSG00000279187.1	2.56115546	-2.3585964	0.00455824	0.01291303	0.02	0	0.12	0.08	RP11-455O6	--	chr17	TEC
ENSG00000243701.4	1.92890791	-0.8986304	0.00032282	0.00116916	0	0.11	0.11	0.46	LINC00883	long intergenic non-protein coding RNA 883	chr3	lincRNA
ENSG00000108021.18	1.91804142	1.71866598	1.57E-08	1.17E-07	0.07	0.19	0.6	0.67	FAM208B	family with sequence similarity 208, member B	chr10	protein_coding
ENSG00000188994.11	2.82349709	0.58124103	2.01E-11	2.18E-10	0	0.04	0.14	0.32	ZNF292	zinc finger protein 292	chr6	protein_coding
ENSG00000121068.12	-2.1228299	2.95775337	1.24E-11	1.38E-10	3.32	2.79	0.7	1.17	TBX2	T-box 2	chr17	protein_coding
ENSG00000179532.11	-2.1360037	2.50058655	2.90E-11	3.09E-10	0.42	0.53	0.17	0.12	DNHD1	dynein heavy chain domain 1	chr11	protein_coding
ENSG00000114933.14	1.03026689	-0.1499514	0.01457126	0.03612598	0.05	0.02	0.08	0.09	INO80D	INO80 complex subunit D	chr2	protein_coding
ENSG00000152932.7	4.87917439	-3.0678699	0.01889873	0.04519557	0	0	0	0.02	RAB3C	RAB3C, member RAS oncogene family	chr5	protein_coding
ENSG00000152377.11	-1.6638517	3.94278297	2.89E-08	2.08E-07	3.88	3.92	1.46	1.83	SPOCK1	sparc/osteonectin, cwcv and kazal-like domains proteoglycan (testican) 1	chr5	protein_coding
ENSG00000256742.1	2.1373167	-1.3117521	0.0003561	0.00128174	0.15	0.21	1.3	0.85	RP13-941N1	--	chr12	lincRNA
ENSG00000138756.16	1.4500511	0.99379588	4.71E-05	0.00020301	0.19	0.14	0.56	0.66	BMP2K	BMP2 inducible kinase	chr4	protein_coding
ENSG00000081026.17	1.77050899	0.37219815	9.57E-06	4.66E-05	0.06	0.07	0.2	0.38	MAGI3	membrane associated guanylate kinase, WW and PDZ domain containing 3	chr1	protein_coding
ENSG00000126814.6	1.12418959	1.3816472	0.00088745	0.00294859	0.2	0.26	0.51	0.84	TRMT5	tRNA methyltransferase 5	chr14	protein_coding
ENSG00000239697.9	1.86332625	1.9941819	1.62E-08	1.20E-07	1.22	0.51	4.74	3.72	TNFSF12	tumor necrosis factor (ligand) superfamily, member 12	chr17	protein_coding
ENSG00000136152.13	1.04976171	1.25470908	0.00204268	0.00625139	0.19	0.3	0.64	0.72	COG3	component of oligomeric golgi complex 3	chr13	protein_coding
ENSG00000137574.9	1.25654033	2.27901362	6.70E-05	0.00027966	0.55	0.65	1.37	2.48	TGS1	trimethylguanosine synthase 1	chr8	protein_coding
ENSG00000142621.18	3.97133628	-2.048278	3.06E-05	0.00013563	0	0	0.11	0.01	FHAD1	forkhead-associated (FHA) phosphopeptide binding domain 1	chr1	protein_coding
ENSG00000232519.2	-5.2091082	-3.0354822	0.00538165	0.01488851	0.49	0.52	0	0	RP11-29H23	--	chr1	antisense
ENSG00000187676.7	1.69424912	1.78439187	3.56E-07	2.21E-06	0.33	0.27	1.2	1.4	B3GALTL	beta 1,3-galactosyltransferase-like	chr13	protein_coding
ENSG00000117569.17	2.07402165	0.15196079	7.10E-07	4.18E-06	0.04	0.01	0.12	0.18	PTBP2	polypyrimidine tract binding protein 2	chr1	protein_coding
ENSG00000115866.9	1.16268212	4.33863219	7.64E-05	0.00031541	3.41	5.14	9.17	16.45	DARS	aspartyl-tRNA synthetase	chr2	protein_coding
ENSG00000280777.1	5.45007274	-2.7494288	0.00158008	0.00495521	0	0	0.02	0.08	HYMAI	hydatidiform mole associated and imprinted (non-protein coding)	chr6	non_coding
ENSG00000254004.5	2.41508349	0.17446711	2.16E-08	1.57E-07	0.03	0.06	0.18	0.46	ZNF260	zinc finger protein 260	chr19	protein_coding
ENSG00000196683.9	-1.8621033	7.2854029	2.30E-10	2.19E-09	188.05	164.51	65.49	63.75	TOMM7	translocase of outer mitochondrial membrane 7 homolog (yeast)	chr7	protein_coding
ENSG00000108599.13	1.92124442	2.45988791	2.16E-09	1.80E-08	0.33	0.57	1.83	2.71	AKAP10	A kinase (PRKA) anchor protein 10	chr17	protein_coding
ENSG00000156508.16	-1.1029949	12.0343456	0.00012196	0.00048183	927.74	872.28	486.33	634.45	EEF1A1	eukaryotic translation elongation factor 1 alpha 1	chr6	protein_coding
ENSG00000271826.4	2.20793876	-0.3910813	2.90E-06	1.56E-05	0.03	0.16	0.57	0.59	PLS3-AS1	PLS3 antisense RNA 1	chrX	antisense
ENSG00000117650.11	1.27270595	3.32644969	2.42E-05	0.00010948	1.02	2.27	3.97	6.64	NEK2	NIMA-related kinase 2	chr1	protein_coding
ENSG00000156218.11	2.45594129	2.65792881	6.02E-14	8.61E-13	0.17	0.25	1.28	1.81	ADAMTSL3	ADAMTS-like 3	chr15	protein_coding
ENSG00000170837.2	12.2499461	3.22357375	1.33E-62	3.89E-60	0	0	7.02	9.26	GPR27	G protein-coupled receptor 27	chr3	protein_coding
ENSG00000164142.14	2.11032982	2.58569203	5.55E-11	5.73E-10	0.31	0.47	2.4	2.07	FAM160A1	family with sequence similarity 160, member A1	chr4	protein_coding
ENSG00000148225.14	1.25783678	2.75566359	4.53E-05	0.00019597	0.63	0.67	2.13	2.02	WDR31	WD repeat domain 31	chr9	protein_coding
ENSG00000116990.10	1.30651181	0.82471565	0.00027779	0.00101776	0.15	0.17	0.5	0.55	MYCL	v-myc avian myelocytomatosis viral oncogene lung carcinoma derived homolog	chr1	protein_coding
ENSG00000235097.1	-4.1474433	-2.269462	0.00013996	0.00054609	0.2	0.04	0.02	0	LINC00330	long intergenic non-protein coding RNA 330	chr13	lincRNA
ENSG00000169752.15	1.56554624	0.5228303	3.69E-05	0.00016155	0.51	0.44	1.41	2.35	NRG4	neuregulin 4	chr15	protein_coding
ENSG00000162702.7	2.00415411	1.44068676	1.07E-08	8.16E-08	0.11	0.24	0.79	1.06	ZNF281	zinc finger protein 281	chr1	protein_coding
ENSG00000253250.2	-6.8837939	-1.9245183	1.35E-07	8.87E-07	0.33	0.48	0	0	C8orf88	chromosome 8 open reading frame 88	chr8	protein_coding
ENSG00000181274.6	-1.0421292	5.94083306	0.00030512	0.00110909	30.37	30.11	19.41	19.73	FRAT2	frequently rearranged in advanced T-cell lymphomas 2	chr10	protein_coding
ENSG00000275056.1	2.51714644	-1.2214213	3.89E-05	0.00016973	0.15	0	0.62	0.57	RP11-127I20	--	chr16	sense_intronic
ENSG00000196739.13	-4.55225	4.4989303	3.75E-40	2.90E-38	4.39	4.63	0.22	0.29	COL27A1	collagen, type XXVII, alpha 1	chr9	protein_coding
ENSG00000159733.12	-1.2712824	3.18783981	2.54E-05	0.00011476	1.2	1.36	0.78	0.63	ZFYVE28	zinc finger, FYVE domain containing 28	chr4	protein_coding
ENSG00000129450.7	-5.2072905	-3.0363253	0.00538165	0.01488851	0.07	0.02	0	0	SIGLEC9	sialic acid binding Ig-like lectin 9	chr19	protein_coding
ENSG00000165169.9	1.79620236	1.60453564	1.20E-07	7.99E-07	0.28	0.39	0.74	2.37	DYNLT3	dynein, light chain, Tctex-type 3	chrX	protein_coding
ENSG00000231500.5	-2.1162204	10.3826822	8.10E-13	1.02E-11	2829.95	2539.16	867.91	780.43	RPS18	ribosomal protein S18	chr6	protein_coding
ENSG00000261051.1	4.73235456	-3.1388833	0.01889873	0.04519557	0	0	0.11	0	RP11-274H2	--	chr3	sense_overlapping
ENSG00000186326.3	1.34720514	0.39764831	0.000371	0.00133079	0.21	0.18	0.82	0.51	RGS9BP	regulator of G protein signaling 9 binding protein	chr19	protein_coding
ENSG00000115738.8	5.64774396	3.53273167	1.30E-46	1.49E-44	0.07	0.26	9.65	13.02	ID2	inhibitor of DNA binding 2, dominant negative helix-loop-helix protein	chr2	protein_coding
ENSG00000086827.7	1.25497339	2.21649125	7.10E-05	0.00029435	0.66	0.8	1.81	2.84	ZW10	zw10 kinetochore protein	chr11	protein_coding
ENSG00000101639.17	2.31296946	2.00045908	6.32E-12	7.27E-11	0.19	0.07	0.77	0.98	CEP192	centrosomal protein 192kDa	chr18	protein_coding
ENSG00000104368.16	-5.589448	2.24518468	5.08E-39	3.70E-37	3.04	2.41	0.06	0.09	PLAT	plasminogen activator, tissue	chr8	protein_coding
ENSG00000197021.7	-1.8647232	3.96163282	6.85E-10	6.12E-09	9.42	8.06	3.61	2.77	CXorf40B	chromosome X open reading frame 40B	chrX	protein_coding
ENSG00000237512.5	5.08767213	-2.9577572	0.00538165	0.01488851	0	0	0.41	0	UNC5B-AS1	UNC5B antisense RNA 1	chr10	antisense
ENSG00000076770.13	3.31954832	-0.3882281	1.04E-09	9.04E-09	0	0.01	0.09	0.13	MBNL3	muscleblind-like splicing regulator 3	chrX	protein_coding
ENSG00000114166.7	1.40911911	-0.3836409	0.0016763	0.00522485	0.06	0.06	0.09	0.35	KAT2B	K(lysine) acetyltransferase 2B	chr3	protein_coding
ENSG00000145332.12	1.82172468	0.36696063	4.32E-06	2.25E-05	0.08	0.07	0.22	0.47	KLHL8	kelch-like family member 8	chr4	protein_coding
ENSG00000281026.1	4.02962997	-2.006058	1.86E-05	8.57E-05	0.01	0	0.13	0.02	N4BP2L2-IT2	N4BPL2 intronic transcript 2	chr13	sense_intronic
ENSG00000183688.4	-1.33685	4.15181887	6.17E-06	3.12E-05	6.21	5.15	3.07	2.93	FAM101B	family with sequence similarity 101, member B	chr17	protein_coding
ENSG00000272267.2	1.73933544	-0.9329451	0.00098911	0.00325251	0.04	0.21	0.7	0.4	RP11-375N1	--	chr8	antisense
ENSG00000138593.7	3.45781576	1.2490771	7.35E-18	1.50E-16	0.03	0.05	0.52	0.73	SECISBP2L	SECIS binding protein 2-like	chr15	protein_coding
ENSG00000223784.1	-4.4622013	0.81336868	5.92E-23	1.76E-21	2.48	3.46	0.1	0.24	RP11-554I8.2	--	chr10	lincRNA
ENSG00000128342.4	-3.8218027	5.20120397	5.86E-32	2.96E-30	13.4	14.65	1.52	1.12	LIF	leukemia inhibitory factor	chr22	protein_coding
ENSG00000100644.15	1.83240248	5.29446788	5.96E-10	5.35E-09	2.98	3.55	12.61	18.52	HIF1A	hypoxia inducible factor 1, alpha subunit (basic helix-loop-helix transcription factor)	chr14	protein_coding
ENSG00000160360.10	-1.3148864	5.00513062	6.71E-06	3.37E-05	8.13	6.83	3.88	4.14	GPSM1	G-protein signaling modulator 1	chr9	protein_coding
ENSG00000196338.11	1.84983978	0.04203125	1.44E-05	6.81E-05	0.09	0.08	0.38	0.42	NLGN3	neuroligin 3	chrX	protein_coding

ENSG00000260220.4	-5.5205905	-2.8639386	0.00158008	0.00495521	0.05	0.01	0	0	MGC50722	--		chr9	protein_coding
ENSG00000180881.18	5.04175262	-2.9826774	0.0100455	0.02582742	0	0	0	0.06	CAPS2	calcyphosine 2		chr12	protein_coding
ENSG00000246465.1	-1.5768883	0.79051065	8.83E-06	4.34E-05	0.83	0.83	0.22	0.52	RP11-57A19	--		chr16	lincRNA
ENSG00000197933.11	3.63379307	-1.1606035	1.97E-07	1.27E-06	0.03	0	0.13	0.45	ZNF823	zinc finger protein 823		chr19	protein_coding
ENSG00000104907.11	-1.6360527	7.14328195	2.05E-08	1.50E-07	63.76	59.67	27.17	25.72	TRMT1	tRNA methyltransferase 1 homolog (S. cerevisiae)		chr19	protein_coding
ENSG00000145934.14	-7.5225296	3.70787525	2.01E-62	5.75E-60	1.78	2.45	0.03	0.01	TENM2	teneurin transmembrane protein 2		chr5	protein_coding
ENSG00000263069.4	-1.6431532	-1.983427	0.01967235	0.04677036	0.04	0.09	0.03	0.02	CTD-2047H1	--		chr17	antisense
ENSG00000167165.17	5.06713725	-2.9688687	0.0100455	0.02582742	0	0	0.06	0.04	UGT1A6	UDP glucuronosyltransferase 1 family, polypeptide A6		chr2	protein_coding
ENSG00000095059.14	-1.1530285	6.34675699	6.54E-05	0.00027331	62.19	55.3	35.04	35.4	DHPS	deoxyhypusine synthase		chr19	protein_coding
ENSG00000214575.8	4.54085528	2.57440042	3.69E-32	1.88E-30	0.04	0.08	2.07	1.75	CPEB1	cytoplasmic polyadenylation element binding protein 1		chr15	protein_coding
ENSG00000157077.13	1.39996718	2.93588612	5.07E-06	2.60E-05	0.77	0.52	1.85	2.72	ZFYVE9	zinc finger, FYVE domain containing 9		chr1	protein_coding
ENSG00000261101.2	-8.1344129	-0.8492437	8.27E-15	1.30E-13	0.82	1.81	0	0	RP4-545K15.	--		chrX	sense_overlapping
ENSG00000158716.7	-1.3934551	5.47670276	1.77E-06	9.82E-06	70.71	55.68	33.31	30.8	DUSP23	dual specificity phosphatase 23		chr1	protein_coding
ENSG00000137103.15	-1.2905056	2.80464655	2.48E-05	0.00011214	1.51	1.63	0.79	0.92	TMEM8B	transmembrane protein 8B		chr9	protein_coding
ENSG00000132849.17	1.37587736	2.43197157	1.13E-05	5.41E-05	0.23	0.33	0.89	1.02	INADL	InaD-like (Drosophila)		chr1	protein_coding
ENSG00000186684.11	2.46885112	0.95621641	1.04E-10	1.04E-09	0.13	0.08	0.62	0.88	CYP27C1	cytochrome P450, family 27, subfamily C, polypeptide 1		chr2	protein_coding
ENSG00000187391.16	3.32593741	0.60946556	2.97E-14	4.39E-13	0.03	0.02	0.31	0.36	MAGI2	membrane associated guanylate kinase, WW and PDZ domain containing 2		chr7	protein_coding
ENSG00000132026.12	-2.4670207	-1.8497186	0.00052507	0.0018287	0.02	0.25	0.03	0.03	RTBDN	retbindin		chr19	protein_coding
ENSG00000153246.10	-2.2202997	4.1271188	3.38E-13	4.45E-12	2.55	2.34	0.58	0.82	PLA2R1	phospholipase A2 receptor 1, 180kDa		chr2	protein_coding
ENSG00000215301.8	2.15527064	6.35730913	4.34E-13	5.64E-12	3.65	4.72	20.03	29.83	DDX3X	DEAD (Asp-Glu-Ala-Asp) box helicase 3, X-linked		chrX	protein_coding
ENSG00000109046.13	2.07549615	4.5197438	6.34E-12	7.29E-11	0.9	1.1	4.33	6.94	WSB1	WD repeat and SOCS box containing 1		chr17	protein_coding
ENSG00000196912.11	2.9319571	-0.25515	3.59E-09	2.93E-08	0.02	0.02	0.19	0.26	ANKRD36B	ankyrin repeat domain 36B		chr2	protein_coding
ENSG00000068354.14	-1.2229809	4.5567671	2.95E-05	0.00013132	6.45	6.74	3.84	3.68	TBC1D25	TBC1 domain family, member 25		chrX	protein_coding
ENSG00000254693.1	-5.8966026	-2.6351464	0.00026535	0.0009747	0.2	0.47	0	0	RP11-58K22.	--		chr11	antisense
ENSG00000086300.14	5.12543578	-0.3718216	2.47E-14	3.69E-13	0.02	0	0.28	0.64	SNX10	sorting nexin 10		chr7	protein_coding
ENSG00000117594.8	-6.7016407	-2.0647893	6.25E-07	3.72E-06	0.34	0.08	0	0	HSD11B1	hydroxysteroid (11-beta) dehydrogenase 1		chr1	protein_coding
ENSG00000122417.14	2.5184555	1.49167595	2.79E-12	3.33E-11	0.07	0.08	0.3	0.82	ODF2L	outer dense fiber of sperm tails 2-like		chr1	protein_coding
ENSG00000087258.12	4.18029379	6.14998152	2.78E-37	1.82E-35	0.77	0.74	20.11	16.25	GNAO1	guanine nucleotide binding protein (G protein), alpha activating activity polypeptide O		chr16	protein_coding
ENSG00000143061.16	-2.9172064	6.20961884	1.73E-21	4.71E-20	14.65	14.24	2.36	2.74	IGSF3	immunoglobulin superfamily, member 3		chr1	protein_coding
ENSG00000119906.10	1.19967551	2.65724643	9.97E-05	0.00040167	0.36	0.33	0.95	1.18	FAM178A	family with sequence similarity 178, member A		chr10	protein_coding
ENSG00000278829.1	2.28099126	-2.1473929	0.00354424	0.01027109	0.1	0	0.51	0.2	RP11-358B2:	--		chr17	sense_intronic
ENSG00000091622.14	-3.7879965	4.78572293	3.16E-31	1.54E-29	4.91	6.87	0.54	0.59	PITPNM3	PITPNM family member 3		chr17	protein_coding
ENSG00000177943.12	-1.9937311	0.68623356	1.22E-07	8.08E-07	0.58	0.47	0.21	0.15	MAMDC4	MAM domain containing 4		chr9	protein_coding
ENSG00000113810.14	3.12691196	3.87544796	3.02E-22	8.62E-21	0.29	0.45	2.16	6.55	SMC4	structural maintenance of chromosomes 4		chr3	protein_coding
ENSG00000166546.12	5.58503228	-2.6679206	0.00086564	0.00288128	0	0	0.04	0.03	BEAN1	brain expressed, associated with NEDD4, 1		chr16	protein_coding
ENSG00000140280.12	1.12125926	2.17836497	0.00039178	0.00139885	0.94	1.24	2.13	4.23	LYSMD2	LysM, putative peptidoglycan-binding, domain containing 2		chr15	protein_coding
ENSG00000115904.11	2.13918396	1.7549563	4.37E-10	4.02E-09	0.1	0.13	0.61	0.75	SOS1	son of sevenless homolog 1 (Drosophila)		chr2	protein_coding
ENSG00000196967.9	3.19462504	-1.1681605	1.55E-06	8.67E-06	0	0.01	0.01	0.14	ZNF585A	zinc finger protein 585A		chr19	protein_coding
ENSG00000145198.13	1.97497393	-1.4235217	0.00185375	0.00571918	0.04	0	0.05	0.2	VWA5B2	von Willebrand factor A domain containing 5B2		chr3	protein_coding
ENSG00000272854.1	-1.6743463	-1.1256842	0.00214312	0.00653623	1.17	0.85	0.77	0.11	RP4-593H12	--		chr7	antisense
ENSG00000124532.13	1.50505425	2.71327774	1.37E-06	7.69E-06	0.63	0.59	1.71	2.93	MRS2	MRS2 magnesium transporter		chr6	protein_coding
ENSG00000269026.2	3.47119178	-1.7389258	4.49E-05	0.00019431	0	0.09	1.05	0.44	AC003006.7	--		chr19	protein_coding
ENSG00000262484.1	1.28821343	0.65685934	0.00051224	0.00178657	0.63	0.7	2.64	1.7	CCER2	coiled-coil glutamate-rich protein 2		chr19	protein_coding
ENSG00000253103.1	4.89462967	-3.0595066	0.01889873	0.04519557	0	0	0.17	0.24	RP11-946L2(	--		chr8	lincRNA
ENSG00000254414.1	2.36030579	-2.4765989	0.01191236	0.03011315	0	0.07	0.23	0.26	RP11-182J1.:	--		chr15	antisense
ENSG00000175792.10	-1.2319663	7.83274084	1.94E-05	8.91E-05	90.37	87.6	49.95	51.04	RUVBL1	RuvB-like AAA ATPase 1		chr3	protein_coding
ENSG00000117475.12	3.24719492	0.35511576	1.21E-12	1.50E-11	0.04	0.06	0.48	0.73	BLZF1	basic leucine zipper nuclear factor 1		chr1	protein_coding
ENSG00000231274.5	-6.5060014	-2.2130378	3.03E-06	1.62E-05	0.25	0.18	0	0	SBK3	SH3 domain binding kinase family, member 3		chr19	protein_coding
ENSG00000139714.11	4.78143374	0.45330727	3.26E-19	7.42E-18	0	0.04	0.74	0.64	MORN3	MORN repeat containing 3		chr12	protein_coding
ENSG00000272512.1	1.36943993	-0.5424598	0.0030971	0.0090807	0.17	0.1	0.55	0.37	RP11-54O7.:	--		chr1	lincRNA
ENSG00000225969.1	2.70876814	-2.26271	0.00281991	0.00840887	0	0.1	0.34	0.59	ABHD11-AS:	ABHD11 antisense RNA 1 (tail to tail)		chr7	antisense
ENSG00000112531.15	1.19225475	2.60988744	0.00011788	0.00046685	0.17	0.2	0.44	0.71	QKI	QKI, KH domain containing, RNA binding		chr6	protein_coding
ENSG00000167748.9	-6.7650535	-2.0164922	3.73E-07	2.30E-06	0.49	0.26	0	0	KLK1	kallikrein 1		chr19	protein_coding
ENSG00000083814.11	-5.8266545	0.75080756	7.88E-27	3.07E-25	0.79	0.97	0.02	0.02	ZNF671	zinc finger protein 671		chr19	protein_coding
ENSG00000164976.8	-1.0077035	5.63207887	0.0004856	0.00169865	8.34	8.41	5.4	5.71	KIAA1161	KIAA1161		chr9	protein_coding
ENSG00000185250.14	1.45590083	-1.4708937	0.01609851	0.03953627	0.03	0.03	0.05	0.17	PPIL6	peptidylprolyl isomerase (cyclophilin)-like 6		chr6	protein_coding
ENSG00000175265.16	2.29842509	2.51951937	1.95E-12	2.37E-11	0.2	0.44	1.46	2.76	GOLGA8A	golgin A8 family, member A		chr15	protein_coding
ENSG00000132003.8	-1.6835204	4.9473925	1.15E-08	8.76E-08	9.19	8.35	3.45	3.84	ZSWIM4	zinc finger, SWIM-type containing 4		chr19	protein_coding
ENSG00000068394.10	-1.2739628	6.76720598	1.06E-05	5.12E-05	72.65	70.56	41.17	37.64	GPKOW	G patch domain and KOW motifs		chrX	protein_coding
ENSG00000156802.11	3.5846759	3.80981666	4.43E-27	1.76E-25	0.19	0.38	3.29	5.84	ATAD2	ATPase family, AAA domain containing 2		chr8	protein_coding
ENSG00000118922.15	-2.216517	0.47713393	8.44E-09	6.54E-08	0.1	0.24	0.03	0.07	KLF12	Kruppel-like factor 12		chr13	protein_coding
ENSG00000255234.4	2.7503082	-0.8559393	7.51E-07	4.41E-06	0.05	0.02	0.34	0.29	RP11-727A2:	--		chr11	antisense
ENSG00000259138.1	-5.022479	-3.1298504	0.0100455	0.02582742	0.22	0.31	0	0	RP11-950C1.	--		chr14	antisense
ENSG00000186222.4	-1.0296757	3.35564993	0.00056397	0.00195593	7.77	5.92	3.9	5.07	BLOC1S4	biogenesis of lysosomal organelles complex-1, subunit 4, cappuccino		chr4	protein_coding
ENSG00000231908.1	-1.89224	-1.2200977	0.00060303	0.00208087	1.14	0.35	0.1	0.42	IDH1-AS1	IDH1 antisense RNA 1		chr2	antisense
ENSG00000157020.16	-1.3208929	7.81093716	4.86E-06	2.51E-05	127.08	115.01	63.8	65.41	SEC13	SEC13 homolog (S. cerevisiae)		chr3	protein_coding
ENSG00000236682.1	1.18981479	-0.3158339	0.00550487	0.01520944	0.14	0.13	0.35	0.47	AC068282.3	--		chr2	lincRNA
ENSG00000247033.1	4.73235456	-3.1388833	0.01889873	0.04519557	0	0	0.13	0	RP11-252E2.	--		chr16	antisense
ENSG00000143067.4	-1.0042506	0.85662805	0.00390734	0.01122833	0.36	0.4	0.22	0.28	ZNF697	zinc finger protein 697		chr1	protein_coding
ENSG00000099957.15	1.204669	0.48417221	0.00147063	0.00467959	0.23	0.12	0.54	0.54	P2RX6	purinergic receptor P2X, ligand gated ion channel, 6		chr22	protein_coding
ENSG00000226386.1	5.34914687	-2.8103904	0.00290522	0.00856312	0	0	0.64	0.45	PARD3-AS1	PARD3 antisense RNA 1		chr10	antisense
ENSG00000148344.10	2.56087151	9.32859173	1.64E-17	3.25E-16	89.16	80.82	662.97	674.24	PTGES	prostaglandin E synthase		chr9	protein_coding
ENSG00000258927.1	-5.3742532	-2.9467828	0.00290522	0.00856312	0.08	0.07	0	0	RP11-1070N	--		chr14	lincRNA
ENSG00000105246.5	-2.3700178	0.45112355	1.78E-09	1.51E-08	1.89	1.17	0.32	0.46	EBI3	Epstein-Barr virus induced 3		chr19	protein_coding
ENSG00000198794.10	3.22242306	4.51060355	5.32E-24	1.70E-22	0.95	0.95	12.14	11.51	SCAMP5	secretory carrier membrane protein 5		chr15	protein_coding
ENSG00000121743.3	5.66409976	-2.616209	0.00086564	0.00288128	0	0	0.01	0.06	GJA3	gap junction protein, alpha 3, 46kDa		chr13	protein_coding
ENSG00000224272.2	-6.3541471	-2.3231927	1.55E-05	7.26E-05	0.15	0.06	0	0	AC114730.3	--		chr2	antisense
ENSG00000179195.14	1.1294996	4.71807222	0.00010993	0.00043806	1.86	3.36	5.44	9.82	ZNF664	zinc finger protein 664		chr12	protein_coding
ENSG00000170775.2	-1.7167769	0.79514318	1.91E-06	1.05E-05	0.66	0.75	0.22	0.34	GPR37	G protein-coupled receptor 37 (endothelin receptor type B-like)		chr7	protein_coding
ENSG00000204356.10	-1.1147614	7.07261262	0.00010854	0.00043353	86.95	78.43	52.81	48.87	NELFE	negative elongation factor complex member E		chr6	protein_coding
ENSG00000260578.1	4.73235456	-3.1388833	0.01889873	0.04519557	0	0	0.07	0	CTD-2541J1:	--		chr18	lincRNA
ENSG00000147679.10	1.17371892	0.49884697	0.0018393	0.00568632	0.14	0.17	0.35	0.58	UTP23	UTP23, small subunit (SSU) processome component, homolog (yeast)		chr8	protein_coding
ENSG00000197213.8	4.72173804	-3.1445547	0.01889873	0.04519557	0	0	0.08	0.03	ZSCAN5B	zinc finger and SCAN domain containing 5B		chr19	protein_coding
ENSG00000173575.17	1.77504558	5.68118084	1.70E-09	1.45E-08	1.04	1.48	5.18	6.3	CHD2	chromodomain helicase DNA binding protein 2		chr15	protein_coding
ENSG00000189275.3	5.19748351	-2.8969879	0.00538165	0.01488851	0	0	0.02	0.06	LINC01164	long intergenic non-protein coding RNA 1164		chr10	lincRNA
ENSG00000241933.1	5.7784906	-2.5437546	0.00026535	0.0009747	0	0	0.07	0.08	RP11-755B1(	--		chr3	lincRNA
ENSG00000170293.7	3.46386935	0.72998054	1.65E-15	2.76E-14	0.23	0.12	2.16	3.09	CMTM8	CKLF-like MARVEL transmembrane domain containing 8		chr3	protein_coding
ENSG00000235725.1	4.41563134	1.033709	2.75E-22	7.87E-21	0.08	0.09	2.77	2.06	AC007389.3	--		chr2	lincRNA
ENSG00000249786.6	-3.1776452	-2.8656583	0.01615662	0.03958166	0.01	0.05	0	0.01	EAF1-AS1	EAF1 antisense RNA 1		chr3	antisense
ENSG00000187094.10	3.91060631	-0.6002174	8.22E-11	8.29E-10	0.06	0	0.81	0.59	CCK	cholecystokinin		chr3	protein_coding
ENSG00000257622.1	1.17058585	-0.9559172	0.0202624	0.0480683	0.5	0.32	1.38	1.12	RP11-44N21	--		chr14	processed_transcript
ENSG00000169398.18	1.05748306	4.64104662	0.00029004	0.00105876	1.33	1.58	3.63	4.45	PTK2	protein tyrosine kinase 2		chr8	protein_coding
ENSG00000176563.8	5.20747907	-2.8915189	0.00538165	0.01488851	0	0	0.05	0.07	CNTD1	cyclin N-terminal domain containing 1		chr17	protein_coding
ENSG00000120616.14	1.39040266	1.12939199	7.13E-05	0.00029551	0.19	0.24	0.59	0.9	EPC1	enhancer of polycomb homolog 1 (Drosophila)		chr10	protein_coding
ENSG00000133706.16	-1.3736644	5.59715141	2.39E-06	1.30E-05	11.31	12.43	5.52	6.71	LARS	leucyl-tRNA synthetase		chr5	protein_coding
ENSG00000224215.1	5.35410067	-2.8076728	0.00290522	0.00856312	0	0	0.25	0.12	RP11-371A1!	--		chr10	antisense
ENSG00000185633.9	-3.3159486	1.0323441	4.75E-17	9.04E-16	1.72	2.83	0.32	0.29	NDUFA4L2	NADH dehydrogenase (ubiquinone) 1 alpha subcomplex, 4-like 2		chr12	protein_coding
ENSG00000181929.10	-1.2442017	6.28326405	1.72E-05	7.99E-05	39.68	37.08	20.5	22.74	PRKAG1	protein kinase, AMP-activated, gamma 1 non-catalytic subunit		chr12	protein_coding
ENSG00000234787.6	3.64992589	-1.5979403	1.25E-05	5.96E-05	0	0.05	0.33	0.62	LINC00458	long intergenic non-protein coding RNA 458		chr13	lincRNA
ENSG00000127995.15	2.90179131	0.06660982	2.49E-10	2.36E-09	0.04	0.05	0.26	0.66	CASD1	CAS1 domain containing 1		chr7	protein_coding
ENSG00000198677.9	2.04538712	1.5138124	3.36E-09	2.75E-08	0.14	0.16	0.68	1	TTC37	tetratricopeptide repeat domain 37		chr5	protein_coding
ENSG00000067955.12	2.41674874	3.88005706	6.92E-15	1.10E-13	1.19	1.16	6.8	10.04	CBFB	core-binding factor, beta subunit		chr16	protein_coding
ENSG00000165124.16	1.73898353	4.25448859	6.41E-09	5.06E-08	0.63	0.49	2.67	2.29	SVEP1	sushi, von Willebrand factor type A, EGF and pentraxin domain containing 1		chr9	protein_coding
ENSG00000185261.12	5.04175262	-2.9826774	0.0100455	0.02582742	0	0	0	0.04	KIAA0825	KIAA0825		chr5	protein_coding
ENSG00000260997.1	-4.7389875	-0.2660659	4.28E-16	7.51E-15	0.66	0.67	0.07	0	RP4-647J21.:	--		chr7	sense_overlapping
ENSG00000070371.14	-1.173911	4.37075935	6.35E-05	0.00026636	4.54	3.63	2.56	2.26	CLTCL1	clathrin, heavy chain-like 1		chr22	protein_coding
ENSG00000145335.14	-8.6394906	2.63330122	2.92E-54	5.35E-52	2.09	2.93	0.02	0	SNCA	synuclein, alpha (non A4 component of amyloid precursor)		chr4	protein_coding
ENSG00000170364.11	1.427981	0.86011992	7.47E-05	0.00030886	0.23	0.26	0.88	0.88	SETMAR	SET domain and mariner transposase fusion gene		chr3	protein_coding
ENSG00000105755.6	-1.2577036	5.25068979	1.56E-05	7.32E-05	35.09	32.82	20.14	17.65	ETHE1	ethylmalonic encephalopathy 1		chr19	protein_coding
ENSG00000160211.14	-1.7580217	7.74835221	1.86E-09	1.57E-08	95	91.97	38.68	34.88	G6PD	glucose-6-phosphate dehydrogenase		chrX	protein_coding
ENSG00000073712.12	2.47038381	3.67157104	2.99E-15	4.88E-14	0.91	0.86	4.95	8.17	FERMT2	fermitin family member 2		chr14	protein_coding
ENSG00000196712.15	2.21984764	2.71995996	4.49E-12	5.24E-11	0.17	0.11	0.77	0.97	NF1	neurofibromin 1		chr17	protein_coding
ENSG00000212747.4	-1.0488999	3.31395897	0.00046151	0.00162505	5.21	5.47	3.68	3.19	FAM127C	family with sequence similarity 127, member C		chrX	protein_coding
ENSG00000104518.9	3.20865772	6.84299814	4.15E-25	1.45E-23	8.47	5.12	86.15	81.58	GSDMD	gasdermin D		chr8	protein_coding
ENSG00000164603.10	2.71330502	-0.1944962	1.32E-08	9.91E-08	0.03	0.06	0.31	0.43	C7orf60	chromosome 7 open reading frame 60		chr7	protein_coding
ENSG00000125648.13	-1.7501359	5.74380667	2.66E-09	2.21E-08	16.97	21.07	7.57	7.47	SLC25A23	solute carrier family 25 (mitochondrial carrier; phosphate carrier), member 23		chr19	protein_coding
ENSG00000162444.11	4.42088073	1.39489758	2.58E-24	8.49E-23	0.38	0.18	7.71	8.61	RBP7	retinol binding protein 7, cellular		chr1	protein_coding
ENSG00000247596.7	-1.8229051	5.2960362	6.95E-10	6.19E-09	34.69	22.91	11.54	10.16	TWF2	twinfilin actin binding protein 2		chr3	protein_coding
ENSG00000111011.16	1.47193417	4.27948202	7.01E-07	4.13E-06	1.85	2.87	6.55	10.96	RSRC2	arginine/serine-rich coiled-coil 2		chr12	protein_coding
ENSG00000267374.1	-5.998864	-2.5681316	0.00014842	0.00057423	0.09	0.07	0	0	RP11-244M2	--		chr18	lincRNA
ENSG00000225973.3	-1.3339025	3.04703079	1.15E-05	5.50E-05	8.82	10.65	5.28	4.99	PIGBOS1	PIGB opposite strand 1		chr15	protein_coding
ENSG00000115290.8	-4.3023712	-2.161588	3.06E-05	0.00013563	0.11	0.12	0.01	0	GRB14	growth factor receptor-bound protein 14		chr2	protein_coding
ENSG00000162616.8	1.22544763	0.25179272	0.00185111	0.00571472	0.16	0.2	0.44	0.68	DNAJB4	DnaJ (Hsp40) homolog, subfamily B, member 4		chr1	protein_coding
ENSG00000123388.4	-3.3615593	3.73304922	7.63E-25	2.62E-23	6.06	5.96	0.77	0.79	HOXC11	homeobox C11		chr12	protein_coding
ENSG00000157601.12	1.40206972	2.21200106	1.01E-05	4.87E-05	0.6	0.39	1.58	1.95	MX1	MX dynamin-like GTPase 1		chr21	protein_coding
ENSG00000178445.8	-2.0031417	-1.7837852	0.00328145	0.0095697	0.11	0.05	0.01	0.04	GLDC	glycine dehydrogenase (decarboxylating)		chr9	protein_coding
ENSG00000204463.11	-1.0124234	7.9280484	0.00041974	0.00148934	68.68	64.16	46.31	41.31	BAG6	BCL2-associated athanogene 6		chr6	protein_coding
ENSG00000007080.9	-1.2700775	8.0719469	1.08E-05	5.19E-05	291.47	274.51	166.73	145.44	CCDC124	coiled-coil domain containing 124		chr19	protein_coding
ENSG00000180626.9	2.66474739	-0.601565	4.91E-07	2.97E-06	0.02	0.03	0.15	0.27	ZNF594	zinc finger protein 594		chr17	protein_coding
ENSG00000072736.17	2.73281231	4.56914512	1.39E-18	2.99E-17	0.81	0.63	6.19	6.61	NFATC3	nuclear factor of activated T-cells, cytoplasmic, calcineurin-dependent 3		chr16	protein_coding
ENSG00000233101.9	1.98562032	-1.5952665	0.00345484	0.01003143	0.08	0	0.12	0.34	HOXB-AS3	HOXB cluster antisense RNA 3		chr17	antisense
ENSG00000156265.14	-3.4385254	-1.1109753	1.25E-07	8.30E-07	0.18	0.24	0.05	0	MAP3K7CL	MAP3K7 C-terminal like		chr21	protein_coding
ENSG00000157542.9	-5.0706469	1.25899552	4.12E-29	1.83E-27	0.19	0.19	0	0.01	KCNJ6	potassium channel, inwardly rectifying subfamily J, member 6		chr21	protein_coding
ENSG00000135537.15	1.45579629	-0.6891826	0.002147	0.00654725	0.05	0.04	0.15	0.18	LACE1	lactation elevated 1		chr6	protein_coding
ENSG00000164400.5	-6.4301203	-2.2680998	8.95E-06	4.38E-05	0.51	0.14	0	0	CSF2	colony stimulating factor 2 (granulocyte-macrophage)		chr5	protein_coding
ENSG00000238113.5	1.74909483	-1.147078	0.0021156	0.00646136	0	0.1	0.19	0.25	LINC01410	long intergenic non-protein coding RNA 1410		chr9	lincRNA
ENSG00000106799.11	1.23940241	1.21045414	0.00032856	0.00118833	0.18	0.15	0.43	0.6	TGFBR1	transforming growth factor, beta receptor 1		chr9	protein_coding
ENSG00000233338.1	-4.8079387	-3.2306674	0.01889873	0.04519557	0.04	0.08	0	0	TLR8-AS1	TLR8 antisense RNA 1		chrX	antisense
ENSG00000142611.15	4.82123151	-0.6445769	3.84E-12	4.51E-11	0.01	0	0.13	0.13	PRDM16	PR domain containing 16		chr1	protein_coding
ENSG00000134874.16	2.38080667	2.95013664	1.07E-13	1.48E-12	0.37	0.15	1.36	2.34	DZIP1	DAZ interacting zinc finger protein 1		chr13	protein_coding
ENSG00000171448.8	1.56056276	0.41082398	5.62E-05	0.00023726	0.13	0.09	0.37	0.5	ZBTB26	zinc finger and BTB domain containing 26		chr9	protein_coding
ENSG00000049768.13	1.86531672	-0.4792689	6.67E-05	0.00027839	0.1	0.06	0.35	0.46	FOXP3	forkhead box P3		chrX	protein_coding
ENSG00000005249.11	3.34193827	-0.5638516	1.74E-09	1.48E-08	0.01	0.03	0.21	0.4	PRKAR2B	protein kinase, cAMP-dependent, regulatory, type II, beta		chr7	protein_coding
ENSG00000110811.18	-3.489024	6.79976483	9.97E-29	4.30E-27	65.65	60.18	7.59	7.34	P3H3	prolyl 3-hydroxylase 3		chr12	protein_coding
ENSG00000162779.19	5.78313448	-2.5411454	0.00026535	0.0009747	0	0	0.06	0.06	AXDND1	axonemal dynein light chain domain containing 1		chr1	protein_coding
ENSG00000198435.3	-1.2444033	3.00067444	4.04E-05	0.00017603	4.7	5.57	2.88	2.89	NRARP	NOTCH-regulated ankyrin repeat protein		chr9	protein_coding
ENSG00000122515.13	-1.2135881	6.44323722	2.70E-05	0.00012094	17.34	20.42	11.33	10.31	ZMIZ2	zinc finger, MIZ-type containing 2		chr7	protein_coding
ENSG00000171163.14	-1.7162169	5.39398219	5.59E-09	4.45E-08	21.11	24.43	10.27	8.14	ZNF692	zinc finger protein 692		chr1	protein_coding
ENSG00000164169.11	1.45961584	0.83789629	5.68E-05	0.00023981	0.14	0.28	0.55	0.97	PRMT9	protein arginine methyltransferase 9		chr4	protein_coding
ENSG00000247675.5	1.30380689	-1.1292795	0.01979609	0.04704122	0.18	0.04	0.24	0.49	LRP4-AS1	LRP4 antisense RNA 1		chr11	antisense
ENSG00000243444.6	-1.2600247	0.5960836	0.00050864	0.00177434	0.22	0.5	0.13	0.27	PALM2	paralemmin 2		chr9	protein_coding
ENSG00000170381.11	1.03743678	0.05644949	0.0097706	0.02542838	0.06	0.1	0.22	0.21	SEMA3E	sema domain, immunoglobulin domain (Ig), short basic domain, secreted, (semaphorin) 3E		chr7	protein_coding
ENSG00000148832.13	1.13602855	1.80628399	0.00043105	0.00152655	1.21	0.64	2.64	2.81	PAOX	polyamine oxidase (exo-N4-amino)		chr10	protein_coding
ENSG00000116815.14	1.24962202	0.33767607	0.00137582	0.00441102	0.24	0.41	0.82	1.23	CD58	CD58 molecule		chr1	protein_coding
ENSG00000166128.11	2.32609061	0.57553855	5.55E-09	4.42E-08	0.05	0.1	0.3	0.72	RAB8B	RAB8B, member RAS oncogene family		chr15	protein_coding
ENSG00000100031.17	-6.3844261	4.37761741	2.46E-59	5.61E-57	10.92	9.46	0.15	0.18	GGT1	gamma-glutamyltransferase 1		chr22	protein_coding
ENSG00000244255.4	-1.7211812	4.76432624	6.65E-09	5.24E-08	7.83	8.77	3.33	3.37	XXbac-BPG1	--		chr6	protein_coding
ENSG00000173486.11	-1.0598503	7.71560377	0.00022569	0.0008456	194.07	184.74	114.81	127.68	FKBP2	FK506 binding protein 2, 13kDa		chr11	protein_coding
ENSG00000100106.18	-1.1277784	4.67855357	0.00011053	0.00043999	2.45	2.61	1.47	1.62	TRIOBP	TRIO and F-actin binding protein		chr22	protein_coding
ENSG00000163501.6	3.71897221	-2.2182862	0.00023419	0.00087473	0.01	0	0.1	0.19	IHH	indian hedgehog		chr2	protein_coding
ENSG00000278185.1	-2.078436	1.80538817	6.61E-10	5.91E-09	9.4	8.36	3.38	2.23	RP11-153F5.	--		chr12	sense_intronic
ENSG00000199785.1	-1.315654	-1.3882429	0.01881929	0.04519557	2.59	3.17	0.96	2.03	SNORA52	small nucleolar RNA, H/ACA box 52		chr11	snoRNA
ENSG00000172765.15	1.05408557	2.34562468	0.00074882	0.0025347	0.52	0.27	1.07	1.13	TMCC1	transmembrane and coiled-coil domain family 1		chr3	protein_coding
ENSG00000183570.15	2.82944478	-1.7771675	0.00034876	0.00125664	0.01	0.02	0.22	0.12	PCBP3	poly(rC) binding protein 3		chr21	protein_coding
ENSG00000186188.9	5.60265295	-0.7342859	1.94E-12	2.37E-11	0	0.01	0.32	0.22	FFAR4	free fatty acid receptor 4		chr10	protein_coding
ENSG00000281468.1	4.37596321	-1.0191005	2.98E-09	2.46E-08	0.08	0	1.01	1.63	CTC-459F4.9	--		chr19	sense_intronic
ENSG00000008735.13	2.01622774	4.61138549	2.11E-11	2.27E-10	2.42	2.2	13.34	11.52	MAPK8IP2	mitogen-activated protein kinase 8 interacting protein 2		chr22	protein_coding
ENSG00000113593.10	1.47388741	2.17100759	4.12E-06	2.15E-05	0.78	0.94	2.52	3.86	PPWD1	peptidylprolyl isomerase domain and WD repeat containing 1		chr5	protein_coding
ENSG00000125378.14	-7.6639151	-1.2695891	9.11E-12	1.03E-10	0.39	0.19	0	0	BMP4	bone morphogenetic protein 4		chr14	protein_coding
ENSG00000187049.8	1.00465858	2.96203651	0.00092727	0.00307066	2.24	1.92	6.01	5.11	TMEM216	transmembrane protein 216		chr11	protein_coding
ENSG00000067992.11	1.49730173	3.49160593	7.02E-07	4.14E-06	1.84	1.92	6.91	7.24	PDK3	pyruvate dehydrogenase kinase, isozyme 3		chrX	protein_coding
ENSG00000178409.12	-1.1744164	2.70078531	0.00012097	0.00047846	1.09	1.04	0.56	0.69	BEND3	BEN domain containing 3		chr6	protein_coding
ENSG00000262903.1	2.23052765	-0.057119	5.28E-07	3.18E-06	0.43	0.13	2.27	1.31	RP11-235E1:	--		chr17	antisense
ENSG00000267508.4	-3.098313	-1.6640116	1.25E-05	5.96E-05	0.1	0.03	0	0.02	ZNF285	zinc finger protein 285		chr19	protein_coding
ENSG00000207405.1	-1.3233059	0.69568001	0.00026356	0.00097455	12.37	15.02	7.65	6.91	SNORA64	small nucleolar RNA, H/ACA box 64		chr16	snoRNA
ENSG00000257086.1	-1.2400294	2.90862102	4.70E-05	0.00020272	9.72	10.23	6.53	4.69	RP11-783K1(	--		chr11	lincRNA
ENSG00000168661.13	-7.7608299	-1.1851516	2.65E-12	3.17E-11	0.24	0.24	0	0	ZNF30	zinc finger protein 30		chr19	protein_coding
ENSG00000206799.1	2.26928029	-2.5313377	0.01923175	0.0458365	0.44	0	2.12	1.13	SNORA32	small nucleolar RNA, H/ACA box 32		chr11	snoRNA
ENSG00000165916.7	-1.7147272	9.03013998	4.21E-09	3.42E-08	416.57	404.66	170.7	162.44	PSMC3	proteasome (prosome, macropain) 26S subunit, ATPase, 3		chr11	protein_coding
ENSG00000115966.15	2.54530588	1.56241476	8.18E-13	1.03E-11	0.2	0.08	0.93	1.3	ATF2	activating transcription factor 2		chr2	protein_coding
ENSG00000176678.5	-5.6362872	4.50070238	1.65E-52	2.70E-50	10.52	9.15	0.28	0.25	FOXL1	forkhead box L1		chr16	protein_coding

ENSG00000165669.12	1.45912845	2.49147764	3.32E-06	1.76E-05	0.15	0.19	0.43	0.84	FAM204A	family with sequence similarity 204, member A	chr10	protein_coding
ENSG00000163291.13	2.5209967	0.96692825	4.78E-11	4.97E-10	0.03	0.06	0.19	0.5	PAQR3	progestin and adipoQ receptor family member III	chr4	protein_coding
ENSG00000260260.1	-1.5145173	3.37046409	5.60E-07	3.36E-06	39.26	33.06	16.48	17.28	SNHG19	small nucleolar RNA host gene 19	chr16	lincRNA
ENSG00000166886.11	1.02689161	6.20018574	0.00036798	0.00132135	16.05	12.8	38.87	39.55	NAB2	NGFI-A binding protein 2 (EGR1 binding protein 2)	chr12	protein_coding
ENSG00000123143.11	-1.5029421	8.80951064	2.21E-07	1.41E-06	176.63	164.65	82.18	78.19	PKN1	protein kinase N1	chr19	protein_coding
ENSG00000117226.10	3.19112251	0.81054983	1.51E-14	2.31E-13	0.15	0.02	0.7	1.39	GBP3	guanylate binding protein 3	chr1	protein_coding
ENSG00000255398.2	8.41378089	-0.4378572	3.10E-16	5.50E-15	0	0	0.67	0.62	HCAR3	hydroxycarboxylic acid receptor 3	chr12	protein_coding
ENSG00000118507.14	1.20475455	-0.1527914	0.00412997	0.01179879	0.02	0.2	0.29	0.39	AKAP7	A kinase (PRKA) anchor protein 7	chr6	protein_coding
ENSG00000163482.10	-1.2000611	3.84359975	5.16E-05	0.00021959	3.43	3.02	2.07	1.67	STK36	serine/threonine kinase 36	chr2	protein_coding
ENSG00000175309.13	-2.0871581	3.44317282	1.32E-11	1.46E-10	6.26	5.11	1.74	1.83	PHYKPL	5-phosphohydroxy-L-lysine phospho-lyase	chr5	protein_coding
ENSG00000106683.13	-1.5138074	6.24462216	2.07E-07	1.33E-06	26.4	22.88	12.66	10.29	LIMK1	LIM domain kinase 1	chr7	protein_coding
ENSG00000168778.10	1.96923626	3.68002266	1.22E-10	1.20E-09	1.4	1.48	7.23	7.79	TCTN2	tectonic family member 2	chr12	protein_coding
ENSG00000274600.1	3.32911425	-0.3784277	7.88E-10	6.96E-09	0	0.03	0.16	0.27	RIMBP3B	RIMS binding protein 3B	chr22	protein_coding
ENSG00000143376.11	1.00532192	4.14554057	0.00063011	0.00216312	1.2	1.43	3.74	3.27	SNX27	sorting nexin family member 27	chr1	protein_coding
ENSG00000090447.10	-1.0680984	6.70404932	0.00020806	0.00078358	49.61	53.61	32.86	32.7	TFAP4	transcription factor AP-4 (activating enhancer binding protein 4)	chr16	protein_coding
ENSG00000130635.14	-2.1119734	10.6024562	8.90E-13	1.12E-11	239.61	239.21	77.35	70.02	COL5A1	collagen, type V, alpha 1	chr9	protein_coding
ENSG00000181007.8	-6.9857141	-1.8421603	4.98E-08	3.47E-07	0.18	0.11	0	0	ZFP82	ZFP82 zinc finger protein	chr19	protein_coding
ENSG00000089876.10	1.4991829	4.40754727	4.28E-07	2.62E-06	2.32	2.48	7.1	11.06	DHX32	DEAH (Asp-Glu-Ala-His) box polypeptide 32	chr10	protein_coding
ENSG00000128309.15	-1.2487187	5.54504707	1.72E-05	8.00E-05	24.37	18.44	12.38	11.63	MPST	mercaptopyruvate sulfurtransferase	chr22	protein_coding
ENSG00000182472.7	-2.1207263	-1.7033196	0.00158671	0.00496952	0.11	0.12	0.02	0.05	CAPN12	calpain 12	chr19	protein_coding
ENSG00000010810.16	-3.3086836	4.55539516	3.37E-25	1.18E-23	8.29	8.43	1.04	1.2	FYN	FYN proto-oncogene, Src family tyrosine kinase	chr6	protein_coding
ENSG00000173825.6	1.54413069	1.12794397	1.08E-05	5.19E-05	0.48	0.38	1.94	1.41	TIGD3	tigger transposable element derived 3	chr11	protein_coding
ENSG00000198862.12	3.10966231	0.23981688	1.64E-11	1.80E-10	0.02	0.02	0.14	0.37	LTN1	listerin E3 ubiquitin protein ligase 1	chr21	protein_coding
ENSG00000114115.8	7.51419236	-1.2206565	3.23E-11	3.41E-10	0	0	0.17	0.27	RBP1	retinol binding protein 1, cellular	chr3	protein_coding
ENSG00000277895.1	4.41659089	-0.9907332	1.43E-09	1.22E-08	0	0.04	0.86	0.48	RP11-114F3.	--	chr12	antisense
ENSG00000261189.1	1.53268788	-0.8031162	0.00216733	0.00660335	0.42	0.03	1.05	0.77	RP3-512B11.	--	chr6	antisense
ENSG00000185186.7	-1.2247624	1.57453433	0.00020049	0.00075733	2.85	4.07	2.22	1.72	LINC00313	long intergenic non-protein coding RNA 313	chr21	lincRNA
ENSG00000036549.11	1.40924794	0.45038319	0.00025313	0.00094108	0.08	0.18	0.35	0.55	ZZZ3	zinc finger, ZZ-type containing 3	chr1	protein_coding
ENSG00000186205.11	1.60523496	5.72547071	4.32E-08	3.03E-07	2.43	3.26	10.11	13.02	1-Mar	mitochondrial amidoxime reducing component 1	chr1	protein_coding
ENSG00000127980.14	1.37293174	1.07275695	8.86E-05	0.00035907	0.25	0.16	0.29	1.14	PEX1	peroxisomal biogenesis factor 1	chr7	protein_coding
ENSG00000255389.1	4.91020659	-3.0511096	0.0100455	0.02582742	0	0	0.08	0.02	C6orf3	--	chr6	antisense
ENSG00000163624.5	2.94958087	-0.3808938	1.09E-08	8.29E-08	0.04	0.02	0.32	0.27	CDS1	CDP-diacylglycerol synthase (phosphatidate cytidylyltransferase) 1	chr4	protein_coding
ENSG00000270010.1	3.0442683	-1.2987936	6.79E-06	3.41E-05	0.21	0	1.34	1.19	CTD-2132N1	--	chr17	sense_intronic
ENSG00000110195.10	-9.956573	9.35024374	1.50E-120	7.20E-117	736.41	601.29	1.3	0.5	FOLR1	folate receptor 1 (adult)	chr11	protein_coding
ENSG00000126070.17	1.28540473	2.46737058	3.69E-05	0.00016155	0.12	0.13	0.36	0.45	AGO3	argonaute RISC catalytic component 3	chr1	protein_coding
ENSG00000103510.18	-1.5187715	5.45389624	2.10E-07	1.35E-06	25.61	23.52	11.94	10.88	KAT8	K(lysine) acetyltransferase 8	chr16	protein_coding
ENSG00000272219.1	2.27115128	-1.8525306	0.00303791	0.00892022	0.02	0.05	0.25	0.17	CTB-181H17	--	chr7	sense_intronic
ENSG00000227619.1	-3.1986442	2.72385093	2.11E-21	5.67E-20	4.76	3.1	0.48	0.66	RP11-492E3.	--	chr9	antisense
ENSG00000163746.10	-5.3715723	-2.9480397	0.00290522	0.00856312	0.12	0.02	0	0	PLSCR2	phospholipid scramblase 2	chr3	protein_coding
ENSG00000105372.5	-1.1077208	9.48751722	0.00011509	0.00045684	353.36	305.58	210.83	196.42	RPS19	ribosomal protein S19	chr19	protein_coding
ENSG00000055609.16	2.51320317	2.64938264	1.90E-14	2.87E-13	0.09	0.08	0.67	0.67	KMT2C	lysine (K)-specific methyltransferase 2C	chr7	protein_coding
ENSG00000172824.13	4.74089537	2.35728928	1.93E-32	1.00E-30	0.11	0.11	4.12	3.93	CES4A	carboxylesterase 4A	chr16	protein_coding
ENSG00000256824.1	-3.3207244	-2.7832753	0.00943894	0.02469931	0.06	0.26	0.04	0	RP11-21A7A	--	chr11	antisense
ENSG00000185480.10	1.22146347	-0.2931734	0.00463261	0.01310977	0.1	0.09	0.17	0.41	PARPBP	PARP1 binding protein	chr12	protein_coding
ENSG00000159176.12	-1.1871114	8.01369073	3.76E-05	0.00016473	122.6	110.16	70.62	65.5	CSRP1	cysteine and glycine-rich protein 1	chr1	protein_coding
ENSG00000269425.1	4.91020659	-3.0511096	0.0100455	0.02582742	0	0	0.69	0.2	AC007292.7	--	chr19	antisense
ENSG00000165259.12	-3.3265784	-2.7860235	0.00943894	0.02469931	0.01	0.04	0	0	HDX	highly divergent homeobox	chrX	protein_coding
ENSG00000036054.11	2.19566531	0.97393744	4.43E-09	3.58E-08	0.08	0.2	0.61	1.09	TBC1D23	TBC1 domain family, member 23	chr3	protein_coding
ENSG00000173210.18	-2.9031451	7.60681555	1.93E-21	5.21E-20	42.1	45.67	7.53	8.1	ABLIM3	actin binding LIM protein family, member 3	chr5	protein_coding
ENSG00000204611.5	2.2731749	0.31383471	5.64E-08	3.91E-07	0.06	0.08	0.32	0.59	ZNF616	zinc finger protein 616	chr19	protein_coding
ENSG00000163293.10	6.69726829	-1.8825742	3.73E-07	2.30E-06	0	0	0.04	0.11	NIPAL1	NIPA-like domain containing 1	chr4	protein_coding
ENSG00000178386.11	5.22761104	-2.8805399	0.00290522	0.00856312	0	0	0.1	0.02	ZNF223	zinc finger protein 223	chr19	protein_coding
ENSG00000054277.11	-1.6186202	2.75890304	1.73E-07	1.12E-06	3.14	3.15	1.36	1.37	OPN3	opsin 3	chr1	protein_coding
ENSG00000236671.6	5.47921296	-2.7333202	0.00158008	0.00495521	0	0	0.09	0.04	PRKG1-AS1	PRKG1 antisense RNA 1	chr10	antisense
ENSG00000180044.4	-1.5754199	-0.6618522	0.00054065	0.0018794	0.18	0.38	0.06	0.18	C3orf80	chromosome 3 open reading frame 80	chr3	protein_coding
ENSG00000169926.8	-1.3011609	7.2651605	6.76E-06	3.40E-05	25.73	25.77	13.32	14.55	KLF13	Kruppel-like factor 13	chr15	protein_coding
ENSG00000179406.6	-2.5684934	0.22265531	1.07E-09	9.33E-09	0.3	0.37	0.07	0.08	LINC00174	long intergenic non-protein coding RNA 174	chr7	lincRNA
ENSG00000106829.17	-1.4514548	0.89680799	5.15E-05	0.00021937	0.34	0.5	0.28	0.13	TLE4	transducin-like enhancer of split 4	chr9	protein_coding
ENSG00000109819.7	2.54320949	0.53325481	6.53E-10	5.85E-09	0.03	0.04	0.17	0.38	PPARGC1A	peroxisome proliferator-activated receptor gamma, coactivator 1 alpha	chr4	protein_coding
ENSG00000069696.6	1.96606712	-1.2733758	0.00055173	0.00191543	0.04	0.12	0.63	0.24	DRD4	dopamine receptor D4	chr11	protein_coding
ENSG00000115266.10	1.75637602	2.41571913	3.78E-08	2.66E-07	0.12	0.21	0.67	0.81	APC2	adenomatosis polyposis coli 2	chr19	protein_coding
ENSG00000238107.1	-6.9966414	-1.8361611	3.05E-08	2.18E-07	0.03	0.38	0	0	RP11-495P1(	--	chr1	lincRNA
ENSG00000169026.11	1.85200532	-1.8875558	0.01381259	0.03443739	0.05	0.01	0.21	0.11	MFSD7	major facilitator superfamily domain containing 7	chr4	protein_coding
ENSG00000271384.1	4.72173804	-3.1445547	0.01889873	0.04519557	0	0	0.24	0.08	RP11-435O5	--	chr9	lincRNA
ENSG00000178685.12	1.29339519	5.78364971	8.52E-06	4.20E-05	7.26	6.93	23.47	22.89	PARP10	poly (ADP-ribose) polymerase family, member 10	chr8	protein_coding
ENSG00000122870.10	-3.2993011	-0.6231009	1.29E-09	1.11E-08	0.14	0.17	0.04	0	BICC1	BicC family RNA binding protein 1	chr10	protein_coding
ENSG00000124587.12	-2.2581722	5.1305196	6.61E-14	9.40E-13	14.42	12.37	3.67	3.8	PEX6	peroxisomal biogenesis factor 6	chr6	protein_coding
ENSG00000100285.9	2.19649776	8.93536854	1.31E-13	1.79E-12	29.93	45.26	227.34	231.02	NEFH	neurofilament, heavy polypeptide	chr22	protein_coding
ENSG00000142657.19	-1.6294861	8.94860818	2.17E-08	1.58E-07	312.26	329.42	136.03	140.3	PGD	phosphogluconate dehydrogenase	chr1	protein_coding
ENSG00000146221.9	5.04175262	-2.9826774	0.0100455	0.02582742	0	0	0	0.08	TCTE1	t-complex-associated-testis-expressed 1	chr6	protein_coding
ENSG00000148803.10	-1.8187043	3.92730692	1.64E-09	1.40E-08	23.41	21.21	7.57	9.32	FUOM	fucose mutarotase	chr10	protein_coding
ENSG00000174606.11	1.37726843	2.36607616	1.33E-05	6.30E-05	0.35	0.52	1.36	1.65	ANGEL2	angel homolog 2 (Drosophila)	chr1	protein_coding
ENSG00000172818.8	8.95617281	0.05273013	6.55E-21	1.68E-19	0	0	0.73	0.47	OVOL1	ovo-like zinc finger 1	chr11	protein_coding
ENSG00000101333.15	-2.9713668	-2.4460208	0.00112121	0.00364442	0.02	0.05	0	0.01	PLCB4	phospholipase C, beta 4	chr20	protein_coding
ENSG00000167065.12	1.72874724	0.00427386	3.58E-05	0.0001572	0.11	0.06	0.37	0.4	DUSP18	dual specificity phosphatase 18	chr22	protein_coding
ENSG00000180263.12	4.293105	0.25472439	2.90E-16	5.17E-15	0	0.02	0.2	0.27	FGD6	FYVE, RhoGEF and PH domain containing 6	chr12	protein_coding
ENSG00000204052.4	-2.1822625	1.21233581	1.10E-09	9.58E-09	1.25	1.63	0.5	0.35	LRRC73	leucine rich repeat containing 73	chr6	protein_coding
ENSG00000165271.15	-1.1291912	6.04933034	9.32E-05	0.00037686	19.67	18.6	11.55	11.77	NOL6	nucleolar protein 6 (RNA-associated)	chr9	protein_coding
ENSG00000137601.14	2.00012332	0.81826009	1.21E-07	8.04E-07	0.07	0.11	0.34	0.66	NEK1	NIMA-related kinase 1	chr4	protein_coding
ENSG00000188042.7	-1.2816205	1.20525552	0.00013874	0.00054266	0.73	0.56	0.26	0.45	ARL4C	ADP-ribosylation factor-like 4C	chr2	protein_coding
ENSG00000089280.17	-1.2179513	10.3365782	2.33E-05	0.00010563	274.14	297.86	160.22	167.44	FUS	FUS RNA binding protein	chr16	protein_coding
ENSG00000130592.12	-1.9176823	-0.9021731	0.00014293	0.00055686	0.16	0.18	0.06	0.06	LSP1	lymphocyte-specific protein 1	chr11	protein_coding
ENSG00000163283.6	4.46848755	9.28292612	2.37E-42	2.05E-40	13.36	16.01	443.36	421.4	ALPP	alkaline phosphatase, placental	chr2	protein_coding
ENSG00000109063.13	3.99929796	0.66237665	5.37E-18	1.10E-16	0	0.04	0.53	0.47	MYH3	myosin, heavy chain 3, skeletal muscle, embryonic	chr17	protein_coding
ENSG00000180929.5	6.47125344	-2.0523303	3.03E-06	1.62E-05	0	0	0.03	0.29	GPR62	G protein-coupled receptor 62	chr3	protein_coding
ENSG00000140463.12	1.09082156	3.47749366	0.00026285	0.00097225	2.17	2.4	6.05	6.93	BBS4	Bardet-Biedl syndrome 4	chr15	protein_coding
ENSG00000107187.14	2.35559746	-2.4791882	0.01191236	0.03011315	0	0.02	0.05	0.08	LHX3	LIM homeobox 3	chr9	protein_coding
ENSG00000101115.11	4.58028174	2.33066958	3.29E-31	1.60E-29	0.12	0.06	2.76	3.06	SALL4	spalt-like transcription factor 4	chr20	protein_coding
ENSG00000275874.1	1.09939877	5.33833019	0.00015058	0.00058185	32.88	23.78	79.93	82.14	LINC00162	long intergenic non-protein coding RNA 162	chr21	lincRNA
ENSG00000277013.1	6.94331287	-1.6925944	3.05E-08	2.18E-07	0	0	0.22	0.27	CTC-526N19	--	chr19	lincRNA
ENSG00000142856.15	1.56733153	1.27195835	6.91E-06	3.46E-05	1.1	0.67	2.56	4.5	ITGB3BP	integrin beta 3 binding protein (beta3-endonexin)	chr1	protein_coding
ENSG00000146457.13	1.61869389	4.56704443	4.95E-08	3.46E-07	1.9	1.92	6.23	9.49	WTAP	Wilms tumor 1 associated protein	chr6	protein_coding
ENSG00000153896.16	1.0280042	1.06731984	0.00275782	0.00824835	0.19	0.15	0.36	0.57	ZNF599	zinc finger protein 599	chr19	protein_coding
ENSG00000225518.2	-1.8163195	-0.8811757	0.0003468	0.00125054	1.21	0.9	0.48	0.32	RP11-396C2	--	chr1	lincRNA
ENSG00000213906.8	1.20654024	1.70895866	0.00023043	0.00086216	0.47	0.54	1.43	1.67	LTB4R2	leukotriene B4 receptor 2	chr14	protein_coding
ENSG00000150457.8	2.02088196	2.83853535	1.72E-10	1.66E-09	0.29	0.53	1.66	2.79	LATS2	large tumor suppressor kinase 2	chr13	protein_coding
ENSG00000215458.7	1.90078618	0.02258295	6.72E-06	3.38E-05	0.08	0.05	0.27	0.38	AATBC	apoptosis associated transcript in bladder cancer	chr21	antisense
ENSG00000158321.14	-3.1079443	2.72738342	2.12E-20	5.24E-19	1.26	1.55	0.24	0.19	AUTS2	autism susceptibility candidate 2	chr7	protein_coding
ENSG00000091129.18	-1.5656047	0.11606235	8.47E-05	0.00034404	0.18	0.19	0.11	0.05	NRCAM	neuronal cell adhesion molecule	chr7	protein_coding
ENSG00000144218.17	-5.0187832	-3.1315465	0.01889873	0.04519557	0.02	0	0	0	AFF3	AF4/FMR2 family, member 3	chr2	protein_coding
ENSG00000100934.13	2.50920893	3.20269898	3.57E-15	5.78E-14	0.29	0.46	1.72	4	SEC23A	Sec23 homolog A (S. cerevisiae)	chr14	protein_coding
ENSG00000272157.1	8.1146298	-0.7023214	8.73E-14	1.22E-12	0	0	2.09	2.5	CTD-2168K2	--	chr8	antisense
ENSG00000281566.1	-5.8907277	-2.6380107	0.00026535	0.0009747	0.4	0.08	0	0	RP11-485F1	--	chrX	lincRNA
ENSG00000279443.1	1.09542338	1.37450926	0.00116984	0.00378965	0.56	0.68	2	1.54	RP4-669K10.	--	chr1	TEC
ENSG00000232070.7	1.07589485	-0.2009571	0.01188628	0.03011073	0.25	0.3	0.8	0.73	TMEM253	transmembrane protein 253	chr14	protein_coding
ENSG00000175130.6	-1.0659107	6.43427486	0.00021692	0.00081478	56.7	54.79	36.65	34.25	MARCKSL1	MARCKS-like 1	chr1	protein_coding
ENSG00000181826.8	1.67641834	1.06667176	3.03E-06	1.62E-05	0.38	0.5	1.96	1.77	RELL1	RELT-like 1	chr4	protein_coding
ENSG00000119946.10	2.29112007	4.22374944	7.31E-14	1.04E-12	0.91	0.8	5.53	5.64	CNNM1	cyclin and CBS domain divalent metal cation transport mediator 1	chr10	protein_coding
ENSG00000174938.13	-1.1453019	7.36620012	7.00E-05	0.00029069	49.85	45.92	31.71	25.84	SEZ6L2	seizure related 6 homolog (mouse)-like 2	chr16	protein_coding
ENSG00000152503.8	2.64379577	-0.6204342	6.49E-07	3.85E-06	0.03	0.02	0.12	0.25	TRIM36	tripartite motif containing 36	chr5	protein_coding
ENSG00000060339.12	2.37228349	4.19562921	1.30E-14	2.01E-13	0.74	1.27	4.38	9.58	CCAR1	cell division cycle and apoptosis regulator 1	chr10	protein_coding
ENSG00000142279.11	1.14033978	4.02959997	0.00011477	0.00045564	0.58	0.62	1.8	1.72	WTIP	Wilms tumor 1 interacting protein	chr19	protein_coding
ENSG00000184860.8	-4.6902948	-0.3103941	9.96E-16	1.69E-14	0.11	0.1	0.01	0	SDR42E1	short chain dehydrogenase/reductase family 42E, member 1	chr16	protein_coding
ENSG00000183813.6	5.32454168	-2.8239292	0.00290522	0.00856312	0	0	0.01	0.09	CCR4	chemokine (C-C motif) receptor 4	chr3	protein_coding
ENSG00000138600.8	1.40081742	1.90704099	1.79E-05	8.28E-05	0.21	0.23	0.55	1.01	SPPL2A	signal peptide peptidase like 2A	chr15	protein_coding
ENSG00000246898.1	3.12034076	-1.9872293	0.00060878	0.00209711	0	0.03	0.13	0.19	LINC00920	long intergenic non-protein coding RNA 920	chr16	lincRNA
ENSG00000273333.2	-2.2633947	2.6278778	1.68E-12	2.07E-11	11.39	13.61	3.68	3.25	XXbac-BPG3	--	chr6	3prime_overlapping_ncrna
ENSG00000235978.5	-1.2749221	-1.027862	0.00917505	0.0241697	0.47	0.34	0.18	0.26	AC018816.3	--	chr3	antisense
ENSG00000148704.11	4.88946445	-3.0622981	0.01889873	0.04519557	0	0	0.01	0.03	VAX1	ventral anterior homeobox 1	chr10	protein_coding
ENSG00000255071.2	-3.8051468	-0.8189461	1.65E-10	1.59E-09	0.91	0.93	0.11	0.06	SAA2-SAA4	SAA2-SAA4 readthrough	chr11	protein_coding
ENSG00000122958.13	2.63638508	2.69255962	1.39E-15	2.34E-14	0.36	0.53	2.43	4.93	VPS26A	vacuolar protein sorting 26 homolog A (S. pombe)	chr10	protein_coding
ENSG00000227375.4	4.52007078	-1.6348974	1.71E-06	9.52E-06	0	0.02	0.45	0.16	DLG1-AS1	DLG1 antisense RNA 1	chr3	antisense
ENSG00000126773.11	2.43512312	2.62977476	9.90E-14	1.38E-12	0.05	0.09	0.39	0.65	PCNXL4	pecanex-like 4 (Drosophila)	chr14	protein_coding
ENSG00000213401.7	-6.0940654	-2.5041043	8.36E-05	0.00034052	0.17	0.04	0	0	MAGEA12	melanoma antigen family A12	chrX	protein_coding
ENSG00000279117.1	2.07907084	1.95447944	5.29E-10	4.80E-09	0.55	0.45	3.19	2.43	CTD-2562J1	--	chr11	TEC
ENSG00000258924.4	3.63778757	1.34507568	1.97E-19	4.54E-18	0.15	0.4	4.73	4.51	CTB-96E2.3	--	chr17	processed_transcript
ENSG00000277218.1	2.00427301	-2.0233994	0.01397446	0.03477946	0	0.26	0.6	0.82	RP11-500G1	--	chr10	lincRNA
ENSG00000114098.16	2.5671607	3.41238835	6.61E-16	1.14E-14	0.41	0.33	2.37	3.49	ARMC8	armadillo repeat containing 8	chr3	protein_coding
ENSG00000280033.1	3.26021471	-0.6358456	5.87E-09	4.66E-08	0.05	0.11	1.06	1	CTD-2095E4	--	chr17	TEC
ENSG00000124193.13	1.08588617	6.95452666	0.00016261	0.00062431	13.91	15.83	38.49	45.75	SRSF6	serine/arginine-rich splicing factor 6	chr20	protein_coding
ENSG00000139687.12	2.6547581	1.1121595	2.88E-12	3.44E-11	0.1	0.09	0.64	0.95	RB1	retinoblastoma 1	chr13	protein_coding
ENSG00000184619.3	1.35676993	0.85440545	0.00016421	0.00062863	0.35	0.2	0.95	0.96	KRBA2	KRAB-A domain containing 2	chr17	protein_coding
ENSG00000197409.7	2.23424133	-0.8075224	3.15E-05	0.00013943	0.39	0.28	2.57	1.72	HIST1H3D	histone cluster 1, H3d	chr6	protein_coding
ENSG00000124507.9	-6.5097928	-2.2110744	3.03E-06	1.62E-05	0.04	0.08	0	0	PACSIN1	protein kinase C and casein kinase substrate in neurons 1	chr6	protein_coding
ENSG00000013375.14	1.11455803	1.99371205	0.00048612	0.00169997	0.35	0.28	0.81	1.01	PGM3	phosphoglucomutase 3	chr6	protein_coding
ENSG00000232040.2	-7.3905717	-1.5081958	4.49E-10	4.11E-09	0.08	0.13	0	0	ZBED9	zinc finger, BED-type containing 9	chr6	protein_coding
ENSG00000141622.12	-2.0572797	3.56605013	2.23E-11	2.40E-10	1.48	1.28	0.48	0.4	RNF165	ring finger protein 165	chr18	protein_coding
ENSG00000164951.14	1.48207379	0.329117	0.00017023	0.00065042	0.04	0.18	0.26	0.55	PDP1	pyruvate dehyrogenase phosphatase catalytic subunit 1	chr8	protein_coding
ENSG00000197948.9	-5.902363	3.92734098	3.42E-52	5.43E-50	6.11	4.62	0.16	0.08	FCHSD1	FCH and double SH3 domains 1	chr5	protein_coding
ENSG00000254936.4	7.06470337	-1.6026383	7.18E-09	5.62E-08	0	0	0.48	0.16	AF131215.3	--	chr8	sense_intronic
ENSG00000180318.3	-6.2717826	0.27969954	5.09E-24	1.64E-22	1.22	1.45	0	0.04	ALX1	ALX homeobox 1	chr12	protein_coding
ENSG00000241106.5	-1.6425142	-1.7069078	0.01303785	0.03267232	0.29	0.17	0.09	0.1	HLA-DOB	major histocompatibility complex, class II, DO beta	chr6	protein_coding
ENSG00000265254.1	2.30443341	-1.1900493	9.52E-05	0.00038451	0.11	0.29	1.49	1.16	CTD-2350C1	--	chr17	antisense
ENSG00000185418.14	1.9463631	2.62429822	1.01E-09	8.82E-09	0.62	0.49	2.27	3.44	TARSL2	threonyl-tRNA synthetase-like 2	chr15	protein_coding
ENSG00000012232.7	-1.4211787	5.78167739	1.07E-06	6.11E-06	10.33	9.41	4.92	4.91	EXTL3	exostosin-like glycosyltransferase 3	chr8	protein_coding
ENSG00000090263.14	2.31128776	4.77404892	2.90E-14	4.30E-13	1.52	1.71	10.21	11.17	MRPS33	mitochondrial ribosomal protein S33	chr7	protein_coding
ENSG00000129315.8	1.9435688	2.61008713	1.13E-09	9.73E-09	0.21	0.36	1.23	1.68	CCNT1	cyclin T1	chr12	protein_coding
ENSG00000236519.1	2.73304743	-2.2509888	0.00174589	0.00541921	0.05	0	0.34	0.18	LINC01424	long intergenic non-protein coding RNA 1424	chr21	antisense
ENSG00000272269.1	-1.7719992	2.02494513	3.60E-08	2.55E-07	3.91	5.44	1.21	2.43	RP11-500C1	--	chr6	antisense
ENSG00000095794.18	-1.0451954	1.38033141	0.00161669	0.00505878	0.64	0.46	0.27	0.44	CREM	cAMP responsive element modulator	chr10	protein_coding
ENSG00000135953.9	1.08500915	2.45739568	0.00049088	0.00171585	0.76	0.62	1.76	2.16	MFSD9	major facilitator superfamily domain containing 9	chr2	protein_coding
ENSG00000143641.9	-1.0427157	8.85337686	0.00027933	0.00102277	109.77	116.69	73.03	73.38	GALNT2	polypeptide N-acetylgalactosaminyltransferase 2	chr1	protein_coding
ENSG00000170776.18	1.23314438	5.66028348	2.16E-05	9.86E-05	1.38	1.54	4.56	4.58	AKAP13	A kinase (PRKA) anchor protein 13	chr15	protein_coding
ENSG00000243232.4	-5.2109276	-3.0346387	0.00538165	0.01488851	0.01	0.03	0	0	PCDHAC2	protocadherin alpha subfamily C, 2	chr5	protein_coding
ENSG00000158987.18	3.524711	0.48542908	1.76E-14	2.67E-13	0.01	0.02	0.26	0.29	RAPGEF6	Rap guanine nucleotide exchange factor (GEF) 6	chr5	protein_coding
ENSG00000116017.9	1.57315308	5.65042033	7.85E-08	5.35E-07	3.34	3.53	13.81	13.43	ARID3A	AT rich interactive domain 3A (BRIGHT-like)	chr19	protein_coding
ENSG00000227407.1	5.06713725	-2.9688687	0.0100455	0.02582742	0	0	0.3	0.21	AC008746.3	--	chr19	antisense
ENSG00000185499.15	-2.1165511	6.57896765	9.81E-13	1.23E-11	26.27	32.66	9.5	8.56	MUC1	mucin 1, cell surface associated	chr1	protein_coding
ENSG00000166689.13	1.98945145	2.89888683	2.64E-10	2.49E-09	0.41	0.37	1.66	2.46	PLEKHA7	pleckstrin homology domain containing, family A member 7	chr11	protein_coding
ENSG00000021300.12	-5.6548552	-2.7847371	0.00086564	0.00288128	0.08	0.02	0	0	PLEKHB1	pleckstrin homology domain containing, family B (evectins) member 1	chr11	protein_coding
ENSG00000109929.8	4.17121225	3.07536481	4.18E-31	2.03E-29	0.22	0.31	3.37	9.4	SC5D	sterol-C5-desaturase	chr11	protein_coding
ENSG00000108960.6	1.22123563	0.40903312	0.00151619	0.00481335	0.23	0.21	0.7	0.68	MMD	monocyte to macrophage differentiation-associated	chr17	protein_coding
ENSG00000133055.7	-7.0376708	-1.80064	3.05E-08	2.18E-07	0.24	0.2	0	0	MYBPH	myosin binding protein H	chr1	protein_coding
ENSG00000272050.1	4.87917439	-3.0678699	0.01889873	0.04519557	0	0	0	0.3	RP13-379O2	--	chr20	lincRNA
ENSG00000179111.7	-3.9647223	4.61129931	3.63E-33	1.96E-31	23.69	20.74	1.86	1.94	HES7	hes family bHLH transcription factor 7	chr17	protein_coding
ENSG00000236810.4	-1.6982506	-0.4987511	0.00019138	0.00072488	0.42	0.82	0.24	0.26	TCEB3-AS1	TCEB3 antisense RNA 1	chr1	antisense
ENSG00000076258.8	1.64915937	-1.8146179	0.01883218	0.04519557	0.05	0.03	0.17	0.15	FMO4	flavin containing monooxygenase 4	chr1	protein_coding
ENSG00000242622.1	1.84264411	-1.6853203	0.00684841	0.01852289	0	0.17	0.26	0.55	RP11-18H7.1	--	chr3	lincRNA
ENSG00000180875.4	-10.546449	3.69600357	1.45E-71	6.22E-69	4.36	5.73	0	0.01	GREM2	gremlin 2, DAN family BMP antagonist	chr1	protein_coding
ENSG00000181143.14	-5.1958384	3.62153639	2.36E-44	2.35E-42	0.39	0.5	0.01	0.02	MUC16	mucin 16, cell surface associated	chr19	protein_coding
ENSG00000236824.1	1.4901301	8.09806505	2.82E-07	1.77E-06	7.78	9.55	39.14	25.24	BCYRN1	brain cytoplasmic RNA 1	chr2	lincRNA
ENSG00000135778.10	-1.1696417	5.7155214	5.41E-05	0.00022921	8.09	7.6	4.86	4.43	NTPCR	nucleoside-triphosphatase, cancer-related	chr1	protein_coding
ENSG00000267041.4	4.35291654	-1.0423105	4.33E-09	3.50E-08	0	0	0.12	0.09	ZNF850	zinc finger protein 850	chr19	protein_coding
ENSG00000134014.15	-1.5753376	5.75975502	7.24E-08	4.96E-07	18.13	18.83	7.98	8.56	ELP3	elongator acetyltransferase complex subunit 3	chr8	protein_coding
ENSG00000171310.9	-6.0365595	3.69203633	3.20E-52	5.12E-50	3.19	3.69	0.09	0.05	CHST11	carbohydrate (chondroitin 4) sulfotransferase 11	chr12	protein_coding
ENSG00000150750.6	-2.0557978	-2.330262	0.01324986	0.0331641	0.09	0.24	0.05	0.05	C11orf53	chromosome 11 open reading frame 53	chr11	protein_coding
ENSG00000050438.15	6.3003026	1.20745511	1.03E-29	4.71E-28	0	0.01	0.26	0.46	SLC4A8	solute carrier family 4, sodium bicarbonate cotransporter, member 8	chr12	protein_coding
ENSG00000180438.13	1.98656152	-0.9909771	0.00020218	0.00076264	0.1	0	0.29	0.27	TPRXL	tetra-peptide repeat homeobox-like	chr3	protein_coding
ENSG00000040199.17	2.49953343	2.25240208	1.02E-13	1.42E-12	0.11	0.14	0.85	1.02	PHLPP2	PH domain and leucine rich repeat protein phosphatase 2	chr16	protein_coding
ENSG00000158164.6	3.15135947	-1.2110318	2.24E-06	1.22E-05	0.08	0.08	0.79	1.19	TMSB15A	thymosin beta 15a	chrX	protein_coding
ENSG00000261115.4	5.00222497	-0.4835325	2.12E-13	2.85E-12	0	0.01	0.11	0.13	TMEM178B	transmembrane protein 178B	chr7	protein_coding

ENSG00000279849.1	1.44932555	-1.4777295	0.01609851	0.03953627	0.36	0.3	1.37	1.09	FKSG70	--	chrX	protein_coding
ENSG00000204172.10	2.09470676	0.65834174	6.83E-08	4.69E-07	0.18	0.14	0.75	1.07	AGAP10	--	chr10	protein_coding
ENSG00000133773.10	1.23345394	3.05998429	5.14E-05	0.0002188	1.42	2.62	4.3	8.38	CCDC59	coiled-coil domain containing 59	chr12	protein_coding
ENSG00000138078.14	1.48979934	2.17299617	3.26E-06	1.73E-05	0.22	0.29	0.76	1.16	PREPL	prolyl endopeptidase-like	chr2	protein_coding
ENSG00000229951.4	2.33107014	-1.5635633	0.0007657	0.00258598	0.04	0.01	0.1	0.22	FLJ31356	--	chr2	antisense
ENSG00000134321.10	6.55373997	-1.9945061	1.78E-06	9.85E-06	0	0	0.08	0.13	RSAD2	radical S-adenosyl methionine domain containing 2	chr2	protein_coding
ENSG00000261008.5	1.71435924	-1.596395	0.00684911	0.01852289	0	0.09	0.26	0.14	LINC01572	long intergenic non-protein coding RNA 1572	chr16	lincRNA
ENSG00000279560.1	5.58503228	-2.6679206	0.00086564	0.00288128	0	0	0.98	0.72	AC007326.1	--	chr22	protein_coding
ENSG00000261997.1	2.48491293	-2.4048442	0.00737015	0.01977584	0.01	0.01	0.1	0.02	RP11-212I21	--	chr16	lincRNA
ENSG00000106244.11	-1.2086793	9.01977925	2.70E-05	0.00012094	218.54	239.82	131.84	132.25	PDAP1	PDGFA associated protein 1	chr7	protein_coding
ENSG00000151287.15	1.25820247	0.93012519	0.00039505	0.0014082	0.37	0.46	1.19	1.46	TEX30	testis expressed 30	chr13	protein_coding
ENSG00000279289.1	4.73235456	-3.1388833	0.01889873	0.04519557	0	0	0.06	0	RP3-331H24	--	chr6	TEC
ENSG00000136237.17	5.58023914	-2.6705827	0.00086564	0.00288128	0	0	0.02	0.02	RAPGEF5	Rap guanine nucleotide exchange factor (GEF) 5	chr7	protein_coding
ENSG00000071189.20	1.37068102	0.79257599	0.00017428	0.00066504	0.07	0.05	0.14	0.28	SNX13	sorting nexin 13	chr7	protein_coding
ENSG00000110172.10	1.30612255	3.28858459	1.53E-05	7.23E-05	0.58	0.75	1.4	3.02	CHORDC1	cysteine and histidine-rich domain (CHORD) containing 1	chr11	protein_coding
ENSG00000102385.11	-1.4304606	-1.3302519	0.01100763	0.02808973	0.01	0.1	0.01	0.04	DRP2	dystrophin related protein 2	chrX	protein_coding
ENSG00000197312.10	1.53607249	2.4093494	1.26E-06	7.14E-06	0.17	0.23	0.86	0.72	DDI2	DNA-damage inducible 1 homolog 2 (S. cerevisiae)	chr1	protein_coding
ENSG00000231439.4	8.11400389	-0.7064499	6.16E-14	8.78E-13	0	0	1.19	0.96	WASIR2	WASH and IL9R antisense RNA 2	chr16	lincRNA
ENSG00000161798.6	6.97603365	-1.6725948	1.87E-08	1.38E-07	0	0	0.51	0.21	AQP5	aquaporin 5	chr12	protein_coding
ENSG00000159556.8	1.66461513	4.44027532	2.25E-08	1.63E-07	5.02	3.89	18.83	18.85	ISL2	ISL LIM homeobox 2	chr15	protein_coding
ENSG00000210176.1	3.67143716	-1.5849299	8.20E-06	4.05E-05	0	0.82	7.89	7.1	MT-TH	mitochondrially encoded tRNA histidine	chrM	Mt_tRNA
ENSG00000114480.11	1.95997811	4.36915119	7.83E-11	7.92E-10	2.06	1.79	7.05	13.06	GBE1	glucan (1,4-alpha-), branching enzyme 1	chr3	protein_coding
ENSG00000167772.10	-2.6681863	7.17215385	1.16E-18	2.53E-17	123.38	91.08	22.89	22.1	ANGPTL4	angiopoietin-like 4	chr19	protein_coding
ENSG00000149599.14	3.60627287	1.31221142	5.16E-19	1.16E-17	0.1	0.07	1.33	1.48	DUSP15	dual specificity phosphatase 15	chr20	protein_coding
ENSG00000142534.5	-1.2357431	11.0907018	1.77E-05	8.23E-05	2992.6	2908.61	1738.57	1596.36	RPS11	ribosomal protein S11	chr19	protein_coding
ENSG00000242689.2	5.76922753	-2.5489645	0.00047761	0.00167191	0	0	0.07	0.16	CNTF	ciliary neurotrophic factor	chr11	protein_coding
ENSG00000198246.7	2.13084657	4.23002721	2.51E-12	3.03E-11	2.5	2.47	15.09	13.92	SLC29A3	solute carrier family 29 (equilibrative nucleoside transporter), member 3	chr10	protein_coding
ENSG00000137691.11	3.07199637	1.5856306	1.16E-16	2.13E-15	0.27	0.14	1.7	2.93	C11orf70	chromosome 11 open reading frame 70	chr11	protein_coding
ENSG00000280237.1	5.3196533	-2.8266272	0.00290522	0.00856312	0	0	0	0.06	MIR4697HG	MIR4697 host gene	chr11	TEC
ENSG00000135625.7	5.16881758	-0.3515447	7.52E-15	1.19E-13	0.02	0	1	0.24	EGR4	early growth response 4	chr2	protein_coding
ENSG00000183718.5	2.25038325	2.08274598	1.70E-11	1.86E-10	0.25	0.42	2.01	2.24	TRIM52	tripartite motif containing 52	chr5	protein_coding
ENSG00000151748.13	2.97372248	3.3042094	8.58E-20	2.04E-18	0.45	0.72	4.84	7.41	SAV1	salvador family WW domain containing protein 1	chr14	protein_coding
ENSG00000185515.13	1.07304042	1.26937054	0.00163918	0.0051238	0.35	0.47	1.01	1.3	BRCC3	BRCA1/BRCA2-containing complex, subunit 3	chrX	protein_coding
ENSG00000230922.1	5.76922753	-2.5489645	0.00047761	0.00167191	0	0	0.08	0.18	RP4-753M9.1	--	chr22	sense_intronic
ENSG00000151014.5	-1.0316863	1.71294903	0.00139472	0.00446265	1.32	2.31	1.36	0.99	CCRN4L	CCR4 carbon catabolite repression 4-like (S. cerevisiae)	chr4	protein_coding
ENSG00000142794.17	2.29419372	2.9459837	6.40E-13	8.20E-12	0.47	0.6	3.62	3.38	NBPF3	neuroblastoma breakpoint family, member 3	chr1	protein_coding
ENSG00000140600.15	3.91965173	-0.5800061	8.22E-11	8.29E-10	0.02	0.02	0.25	0.75	SH3GL3	SH3-domain GRB2-like 3	chr15	protein_coding
ENSG00000144426.17	2.17760611	-0.0861556	1.22E-06	6.90E-06	0.03	0.02	0.05	0.22	NBEAL1	neurobeachin-like 1	chr2	protein_coding
ENSG00000163539.14	1.83635995	2.02493967	2.29E-08	1.66E-07	0.16	0.16	0.6	0.92	CLASP2	cytoplasmic linker associated protein 2	chr3	protein_coding
ENSG00000166851.13	-1.013223	8.05358519	0.00041447	0.00147281	131.53	126.76	84.33	86.23	PLK1	polo-like kinase 1	chr16	protein_coding
ENSG00000254860.4	-1.6095459	0.37008287	2.86E-05	0.00012786	1.59	1.05	0.44	0.7	TMEM9B-AS	TMEM9B antisense RNA 1	chr11	antisense
ENSG00000116991.9	-1.2940856	4.63231356	9.91E-06	4.81E-05	3.89	4.36	2.02	2.47	SIPA1L2	signal-induced proliferation-associated 1 like 2	chr1	protein_coding
ENSG00000258910.2	-7.0322814	-1.8036183	3.05E-08	2.18E-07	0.92	0.2	0	0	RP11-19E11.	--	chr2	lincRNA
ENSG00000198821.9	2.45724496	0.05009652	3.49E-08	2.48E-07	0.21	0.07	0.95	1.1	CD247	CD247 molecule	chr1	protein_coding
ENSG00000091009.7	2.17050399	0.71299007	1.37E-08	1.03E-07	0.11	0.03	0.35	0.49	RBM27	RNA binding motif protein 27	chr5	protein_coding
ENSG00000170667.13	-2.5147374	0.37266148	3.24E-10	3.03E-09	0.71	0.44	0.08	0.18	RASA4B	RAS p21 protein activator 4B	chr7	protein_coding
ENSG00000111404.5	4.87917439	-3.0678699	0.01889873	0.04519557	0	0	0	0.19	RERGL	RERG/RAS-like	chr12	protein_coding
ENSG00000242110.6	1.81228211	2.92078934	6.31E-09	4.99E-08	0.45	0.7	2.24	3.16	AMACR	alpha-methylacyl-CoA racemase	chr5	protein_coding
ENSG00000165215.6	-8.7300758	-0.2901168	9.48E-20	2.23E-18	0.86	1.18	0	0	CLDN3	claudin 3	chr7	protein_coding
ENSG00000101928.11	1.29580894	0.52127399	0.00056485	0.00195872	0.26	0.28	0.97	0.78	MOSPD1	motile sperm domain containing 1	chrX	protein_coding
ENSG00000117472.8	-3.4513582	1.73339842	1.07E-20	2.71E-19	3.31	2.79	0.3	0.44	TSPAN1	tetraspanin 1	chr1	protein_coding
ENSG00000172466.14	2.00622916	2.1565778	9.93E-10	8.71E-09	0.19	0.18	0.86	1.12	ZNF24	zinc finger protein 24	chr18	protein_coding
ENSG00000111664.9	-3.4536773	0.74853383	3.23E-16	5.72E-15	0.89	1.69	0.22	0.1	GNB3	guanine nucleotide binding protein (G protein), beta polypeptide 3	chr12	protein_coding
ENSG00000170469.9	-1.7372056	1.75777925	1.60E-07	1.04E-06	6.2	5.61	3.04	1.69	SPATA24	spermatogenesis associated 24	chr5	protein_coding
ENSG00000239636.1	-5.3715723	-2.9480397	0.00290522	0.00856312	0.21	0.03	0	0	RP4-728D4.2	--	chr1	antisense
ENSG00000100697.13	2.43202382	1.75569562	2.68E-12	3.21E-11	0.08	0.08	0.51	0.61	DICER1	dicer 1, ribonuclease type III	chr14	protein_coding
ENSG00000078725.11	6.05360204	-2.3605161	4.74E-05	0.00020301	0	0	0.1	0.05	BRINP1	bone morphogenetic protein/retinoic acid inducible neural-specific 1	chr9	protein_coding
ENSG00000167193.7	1.32696084	3.98281518	7.94E-06	3.94E-05	1.68	2.07	5.61	6.91	CRK	v-crk avian sarcoma virus CT10 oncogene homolog	chr17	protein_coding
ENSG00000122481.15	1.63783489	-0.2253038	0.00019848	0.00075024	0.34	0.14	0.26	1.74	RWDD3	RWD domain containing 3	chr1	protein_coding
ENSG00000259052.1	2.58567549	1.44856652	8.89E-13	1.12E-11	3.38	2.48	23.64	23.53	AL157871.2	--	chr14	sense_overlapping
ENSG00000102471.12	1.07125302	2.03751262	0.0008164	0.00274429	0.39	0.52	0.74	1.8	NDFIP2	Nedd4 family interacting protein 2	chr13	protein_coding
ENSG00000262772.1	-4.8866057	-1.7262061	1.16E-08	8.76E-08	0.25	0.21	0	0.02	RP11-353N1	--	chr17	lincRNA
ENSG00000107874.9	-1.3489435	7.54093135	3.12E-06	1.66E-05	187.44	176.03	96.72	93.36	CUEDC2	CUE domain containing 2	chr10	protein_coding
ENSG00000106789.11	-2.4081468	4.005482	5.77E-15	9.19E-14	3.71	3.95	0.99	0.93	CORO2A	coronin, actin binding protein, 2A	chr9	protein_coding
ENSG00000197561.5	-1.7954713	-1.0555957	0.00095474	0.003149	0.62	0.44	0.31	0.11	ELANE	elastase, neutrophil expressed	chr19	protein_coding
ENSG00000249923.1	-6.0021727	-2.5665039	0.00014842	0.00057423	0.05	0.13	0	0	XXbac-B444	--	chr22	antisense
ENSG00000144485.9	1.01408168	3.59754525	0.00065205	0.00223172	3.95	3.34	10.01	9.62	HES6	hes family bHLH transcription factor 6	chr2	protein_coding
ENSG00000121486.10	1.11807841	1.21486206	0.00108783	0.00354652	0.29	0.23	0.43	1.08	TRMT1L	tRNA methyltransferase 1 homolog (S. cerevisiae)-like	chr1	protein_coding
ENSG00000169762.15	1.3268213	0.93036157	0.00021568	0.00081051	0.2	0.16	0.5	0.73	TAPT1	transmembrane anterior posterior transformation 1	chr4	protein_coding
ENSG00000141098.11	1.22970702	5.2923047	2.39E-05	0.00010805	2.63	2.67	8.01	8.54	GFOD2	glucose-fructose oxidoreductase domain containing 2	chr16	protein_coding
ENSG00000163879.10	-5.2127486	-3.0337949	0.00538165	0.01488851	0	0.09	0	0	DNALI1	dynein, axonemal, light intermediate chain 1	chr1	protein_coding
ENSG00000103043.13	1.24149763	7.02552293	1.73E-05	8.03E-05	15.19	15.48	50.46	46.03	VAC14	Vac14 homolog (S. cerevisiae)	chr16	protein_coding
ENSG00000273141.1	2.89881843	0.06269666	5.90E-10	5.31E-09	0.05	0.66	3.57	3.51	RP11-820I16	--	chr18	antisense
ENSG00000278899.1	2.0263382	-0.7260839	7.18E-05	0.00029725	0.06	0.2	0.71	0.73	AL358852.1	--	chr6	protein_coding
ENSG00000166278.13	3.59115389	0.29590801	3.74E-14	5.44E-13	0.04	0.03	0.68	0.47	C2	complement component 2	chr6	protein_coding
ENSG00000103550.12	-1.1931458	7.24630855	3.51E-05	0.00015453	23.28	27.59	14.07	15.58	KNOP1	lysine-rich nucleolar protein 1	chr16	protein_coding
ENSG00000167333.11	-7.658625	1.64938526	1.18E-40	9.40E-39	1.42	1.61	0.01	0.01	TRIM68	tripartite motif containing 68	chr11	protein_coding
ENSG00000165689.15	-1.2869636	7.54685949	8.37E-06	4.13E-05	96.25	83.63	45.78	52.68	SDCCAG3	serologically defined colon cancer antigen 3	chr9	protein_coding
ENSG00000178074.5	5.03718549	-0.4469868	1.14E-13	1.57E-12	0.01	0	0.16	0.55	C2orf69	chromosome 2 open reading frame 69	chr2	protein_coding
ENSG00000103196.10	6.69580902	4.61410879	2.07E-62	5.85E-60	0.01	0.14	10.65	10.26	CRISPLD2	cysteine-rich secretory protein LCCL domain containing 2	chr16	protein_coding
ENSG00000174720.14	2.06747215	2.22543077	3.23E-10	3.02E-09	0.38	0.7	2.1	3.94	LARP7	La ribonucleoprotein domain family, member 7	chr4	protein_coding
ENSG00000181830.8	-1.5280614	6.49822336	1.56E-07	1.02E-06	22.41	25.87	10.8	11.5	SLC35C1	solute carrier family 35 (GDP-fucose transporter), member C1	chr11	protein_coding
ENSG00000170961.6	2.00739804	-0.2202403	1.01E-05	4.87E-05	0.07	0.05	0.27	0.37	HAS2	hyaluronan synthase 2	chr8	protein_coding
ENSG00000136158.9	-1.5909042	1.34039125	2.19E-06	1.20E-05	0.85	1.17	0.34	0.55	SPRY2	sprouty homolog 2 (Drosophila)	chr13	protein_coding
ENSG00000113594.8	2.40293409	-0.146349	2.77E-07	1.73E-06	0.01	0.03	0.09	0.19	LIFR	leukemia inhibitory factor receptor alpha	chr5	protein_coding
ENSG00000231156.1	-5.2118379	-3.0342168	0.00538165	0.01488851	0.07	0.54	0	0	AC093702.1	--	chr2	lincRNA
ENSG00000163946.12	2.15167915	2.73516591	1.77E-11	1.93E-10	0.18	0.26	1.02	1.63	FAM208A	family with sequence similarity 208, member A	chr3	protein_coding
ENSG00000063177.11	-1.3368987	9.7926911	3.65E-06	1.92E-05	818.84	615.08	407.01	348.73	RPL18	ribosomal protein L18	chr19	protein_coding
ENSG00000106105.12	-1.9529905	7.83308905	3.24E-11	3.42E-10	102.69	118.78	34.7	41.63	GARS	glycyl-tRNA synthetase	chr7	protein_coding
ENSG00000129951.17	-6.3207115	2.18108369	3.79E-42	3.21E-40	3.54	2.48	0.04	0.06	LPPR3	--	chr19	protein_coding
ENSG00000161533.10	1.24944464	3.28531025	3.51E-05	0.00015457	0.78	0.42	1.57	2.26	ACOX1	acyl-CoA oxidase 1, palmitoyl	chr17	protein_coding
ENSG00000255145.2	5.47921296	-2.7333202	0.00158008	0.00495521	0	0	0.47	0.2	STX17-AS1	STX17 antisense RNA 1	chr9	antisense
ENSG00000259943.1	-1.3850199	0.76388909	0.00012313	0.00048619	1.58	0.94	0.71	0.58	RP1-39G22.7	--	chr1	antisense
ENSG00000163811.10	1.46234423	3.54247973	1.29E-06	7.27E-06	0.98	1.84	4.02	6.39	WDR43	WD repeat domain 43	chr2	protein_coding
ENSG00000181885.17	-4.4081813	2.47179655	2.17E-31	1.07E-29	4.55	3.76	0.33	0.2	CLDN7	claudin 7	chr17	protein_coding
ENSG00000233223.2	2.3054118	4.72686568	3.34E-14	4.90E-13	6.83	6.75	44.54	44.91	AC113189.5	--	chr17	antisense
ENSG00000164978.16	-1.162168	5.16055927	6.37E-05	0.00026723	39.28	35.09	21.1	23.25	NUDT2	nudix (nucleoside diphosphate linked moiety X)-type motif 2	chr9	protein_coding
ENSG00000235314.1	-6.630556	1.53951203	8.57E-38	5.76E-36	2	1.6	0.02	0.02	LINC00957	long intergenic non-protein coding RNA 957	chr7	lincRNA
ENSG00000274769.1	-1.1593706	-0.0748112	0.00381432	0.01098079	0.85	0.61	0.23	0.63	RP11-493E1	--	chr2	processed_transcript
ENSG00000252835.1	3.7350065	-1.5398605	3.56E-06	1.88E-05	0	0.41	5.53	2.35	SCARNA21	small Cajal body-specific RNA 21	chr17	scaRNA
ENSG00000123091.4	1.35403605	3.44807336	7.10E-06	3.54E-05	1.65	1.54	4.03	6.91	RNF11	ring finger protein 11	chr1	protein_coding
ENSG00000231721.5	1.40536613	1.85368576	1.80E-05	8.36E-05	0.24	0.45	1.01	1.44	LINC-PINT	long intergenic non-protein coding RNA, p53 induced transcript	chr7	antisense
ENSG00000138750.13	1.52617706	1.68386756	4.35E-06	2.26E-05	0.36	0.74	1.48	2.76	NUP54	nucleoporin 54kDa	chr4	protein_coding
ENSG00000103502.12	-1.0661162	6.1074002	0.00022018	0.000826	28.2	25.95	17.85	16.59	CDIPT	CDP-diacylglycerol--inositol 3-phosphatidyltransferase	chr16	protein_coding
ENSG00000085978.20	-1.3953028	5.37190338	1.73E-06	9.62E-06	13.61	14.5	6.64	7.62	ATG16L1	autophagy related 16-like 1 (S. cerevisiae)	chr2	protein_coding
ENSG00000155330.8	2.40040317	0.94445313	2.91E-10	2.74E-09	0.08	0.06	0.29	0.64	C16orf87	chromosome 16 open reading frame 87	chr16	protein_coding
ENSG00000126856.12	5.21752153	-2.8860363	0.00538165	0.01488851	0	0	0.1	0.05	PRDM7	PR domain containing 7	chr16	protein_coding
ENSG00000070018.7	1.16147087	3.44933138	0.00010578	0.00042439	0.48	0.6	1.53	1.69	LRP6	low density lipoprotein receptor-related protein 6	chr12	protein_coding
ENSG00000169379.14	3.52406676	1.37091937	7.04E-19	1.56E-17	0.06	0.1	0.85	1.63	ARL13B	ADP-ribosylation factor-like 13B	chr3	protein_coding
ENSG00000073282.11	4.88946445	-3.0622981	0.01889873	0.04519557	0	0	0.01	0.03	TP63	tumor protein p63	chr3	protein_coding
ENSG00000100097.10	-2.5105257	7.9339275	6.36E-17	1.20E-15	653.5	548.52	148.44	132.41	LGALS1	lectin, galactoside-binding, soluble, 1	chr22	protein_coding
ENSG00000105499.12	1.86567089	2.2611943	9.00E-09	6.94E-08	0.73	0.56	3.09	3.19	PLA2G4C	phospholipase A2, group IVC (cytosolic, calcium-independent)	chr19	protein_coding
ENSG00000027075.12	-1.2313328	2.72056048	5.95E-05	0.00025046	2.08	1.97	0.91	1.4	PRKCH	protein kinase C, eta	chr14	protein_coding
ENSG00000156222.10	5.05186882	-2.9771646	0.0100455	0.02582742	0	0	0.02	0.06	SLC28A1	solute carrier family 28 (concentrative nucleoside transporter), member 1	chr15	protein_coding
ENSG00000049249.7	-7.5574718	2.08315006	4.17E-45	4.33E-43	0.97	1.32	0.02	0	TNFRSF9	tumor necrosis factor receptor superfamily, member 9	chr1	protein_coding
ENSG00000165732.11	1.1242442	4.71873239	0.00011784	0.00046677	2.37	2.96	5.75	9.81	DDX21	DEAD (Asp-Glu-Ala-Asp) box helicase 21	chr10	protein_coding
ENSG00000111199.9	2.63759819	-1.3474151	4.20E-05	0.00018265	0	0.04	0.31	0.06	TRPV4	transient receptor potential cation channel, subfamily V, member 4	chr12	protein_coding
ENSG00000280138.1	1.05346469	0.83535048	0.00286341	0.00852903	0.04	0.06	0.14	0.13	RP11-463O1	--	chr12	TEC
ENSG00000176971.3	-11.068358	2.01565374	2.84E-47	3.39E-45	2.9	3.89	0	0	FIBIN	fin bud initiation factor homolog (zebrafish)	chr11	protein_coding
ENSG00000138439.11	1.23266265	1.24548563	0.00030606	0.00111182	0.15	0.23	0.58	0.62	FAM117B	family with sequence similarity 117, member B	chr2	protein_coding
ENSG00000054179.10	2.71874663	0.67224623	2.98E-11	3.17E-10	0.17	0.14	1.1	1.57	ENTPD2	ectonucleoside triphosphate diphosphohydrolase 2	chr9	protein_coding
ENSG00000174547.12	-1.0503428	7.42972449	0.000258	0.00095651	111.8	109.77	75.11	67.22	MRPL11	mitochondrial ribosomal protein L11	chr11	protein_coding
ENSG00000132854.17	1.0945768	2.29772197	0.00048985	0.00171276	0.38	0.45	1.17	1.2	KANK4	KN motif and ankyrin repeat domains 4	chr1	protein_coding
ENSG00000115183.12	1.6812226	3.35109925	3.74E-08	2.64E-07	0.46	0.57	2.16	2.27	TANC1	tetratricopeptide repeat, ankyrin repeat and coiled-coil containing 1	chr2	protein_coding
ENSG00000280987.1	2.06308628	4.28597688	9.90E-12	1.11E-10	1.04	0.9	3.06	7.85	MATR3	matrin 3	chr5	protein_coding
ENSG00000201592.1	1.82721647	0.32101261	4.60E-06	2.38E-05	5.68	4.61	30.03	19.06	snoU2_19	--	chrX	snoRNA
ENSG00000175899.13	1.20169162	0.52480769	0.00144206	0.00459877	0.04	0.23	0.42	0.43	A2M	alpha-2-macroglobulin	chr12	protein_coding
ENSG00000279246.1	-1.258104	0.20880662	0.00094725	0.00312733	0.69	0.44	0.22	0.41	RP11-286N2	--	chr11	TEC
ENSG00000273691.1	5.06713725	-2.9688687	0.0100455	0.02582742	0	0	0.15	0.1	RP11-617F2	--	chr15	sense_intronic
ENSG00000115694.13	-1.7212956	7.48391296	3.90E-09	3.17E-08	43.88	41.65	17.33	17.23	STK25	serine/threonine kinase 25	chr2	protein_coding
ENSG00000067221.12	-1.3664995	3.96570096	4.30E-06	2.24E-05	3.04	2.52	1.48	1.4	STOML1	stomatin (EPB72)-like 1	chr15	protein_coding
ENSG00000082213.16	1.29392971	2.77828019	2.66E-05	0.00011945	0.98	0.78	2.39	3.38	C5orf22	chromosome 5 open reading frame 22	chr5	protein_coding
ENSG00000156475.17	-4.9464667	-0.9494105	1.56E-12	1.92E-11	0.09	0.04	0	0	PPP2R2B	protein phosphatase 2, regulatory subunit B, beta	chr5	protein_coding
ENSG00000267322.2	1.0584726	3.42101417	0.00040448	0.00143883	1.99	2.32	6.74	5.17	SNHG22	small nucleolar RNA host gene 22	chr18	antisense
ENSG00000154734.13	1.09566246	3.97895995	0.00020763	0.0007821	1.26	1.86	3.5	5.42	ADAMTS1	ADAM metallopeptidase with thrombospondin type 1 motif, 1	chr21	protein_coding
ENSG00000126602.9	-1.6687728	9.41755613	1.02E-08	7.83E-08	290.91	281.66	128.46	111.03	TRAP1	TNF receptor-associated protein 1	chr16	protein_coding
ENSG00000138131.3	-13.087569	4.03777405	1.05E-76	6.00E-74	8.49	6.07	0	0	LOXL4	lysyl oxidase-like 4	chr10	protein_coding
ENSG00000104154.6	1.21971776	-0.0204643	0.00281411	0.00840201	0.06	0.06	0.17	0.21	SLC30A4	solute carrier family 30 (zinc transporter), member 4	chr15	protein_coding
ENSG00000139154.13	1.37595004	3.35625667	5.34E-06	2.72E-05	0.65	0.91	2.4	3	AEBP2	AE binding protein 2	chr12	protein_coding
ENSG00000183625.13	5.20747907	-2.8915189	0.00538165	0.01488851	0	0	0.06	0.07	CCR3	chemokine (C-C motif) receptor 3	chr3	protein_coding
ENSG00000269890.1	7.04230204	-1.6089617	1.16E-08	8.76E-08	0	0	0.07	0.19	RP5-1139B1	--	chr1	antisense
ENSG00000101331.14	7.21138667	-1.4823343	1.11E-09	9.61E-09	0	0	0.34	0.14	CCM2L	cerebral cavernous malformation 2-like	chr20	protein_coding
ENSG00000198719.8	3.54465215	-1.6812889	2.93E-05	0.00013031	0	0.02	0.16	0.14	DLL1	delta-like 1 (Drosophila)	chr6	protein_coding
ENSG00000278978.1	1.60976804	0.16115885	7.20E-05	0.00029802	0.78	0.36	2.19	2.5	RP11-164P1	--	chr4	TEC
ENSG00000101966.11	1.50095983	1.98350095	3.67E-06	1.93E-05	0.13	0.23	0.57	0.81	XIAP	X-linked inhibitor of apoptosis, E3 ubiquitin protein ligase	chrX	protein_coding
ENSG00000125534.8	-1.9785798	8.35849412	1.82E-11	1.99E-10	378.93	518.65	169.81	132.13	PPDPF	pancreatic progenitor cell differentiation and proliferation factor	chr20	protein_coding
ENSG00000173542.7	3.05949451	0.55184738	1.15E-12	1.43E-11	0.05	0.01	0.18	0.57	MOB1B	MOB kinase activator 1B	chr4	protein_coding
ENSG00000166147.12	-8.3673022	6.65116957	4.23E-91	4.83E-88	10.18	10.94	0.05	0.04	FBN1	fibrillin 1	chr15	protein_coding
ENSG00000126838.8	7.18079027	-1.5014478	2.80E-09	2.31E-08	0	0	0.13	0.13	PZP	pregnancy-zone protein	chr12	protein_coding
ENSG00000116586.10	-1.048635	5.72528758	0.00028465	0.00104084	91.37	75.27	54.76	52.61	LAMTOR2	late endosomal/lysosomal adaptor, MAPK and MTOR activator 2	chr1	protein_coding
ENSG00000113578.16	-5.2109276	-3.0346387	0.00538165	0.01488851	0.01	0.03	0	0	FGF1	fibroblast growth factor 1 (acidic)	chr5	protein_coding
ENSG00000232021.5	3.50145688	-2.3642542	0.00066294	0.00226478	0	0.01	0.09	0.09	LEF1-AS1	LEF1 antisense RNA 1	chr4	processed_transcript
ENSG00000188647.11	1.3173441	0.78429777	0.00030319	0.00110305	0.06	0.07	0.13	0.3	PTAR1	protein prenyltransferase alpha subunit repeat containing 1	chr9	protein_coding
ENSG00000138381.8	2.04633156	1.56233188	3.38E-09	2.76E-08	0.27	0.46	1.26	2.74	ASNSD1	asparagine synthetase domain containing 1	chr2	protein_coding
ENSG00000171813.12	-3.1333506	6.09732698	4.23E-24	1.38E-22	38.69	36.38	5.52	5.89	PWWP2B	PWWP domain containing 2B	chr10	protein_coding
ENSG00000082996.18	1.6933382	1.47058508	7.61E-07	4.46E-06	0.27	0.42	1.04	1.95	RNF13	ring finger protein 13	chr3	protein_coding
ENSG00000254973.1	3.65605315	-0.3099896	1.28E-11	1.42E-10	0.09	0	0.83	0.75	RP11-429J17	--	chr8	lincRNA
ENSG00000124786.9	1.13354625	1.0875018	0.00105509	0.00345008	0.44	0.53	0.99	1.85	SLC35B3	solute carrier family 35 (adenosine 3'-phospho 5'-phosphosulfate transporter), member B3	chr6	protein_coding
ENSG00000269900.3	2.52601752	2.56306994	1.72E-14	2.61E-13	5.19	5.18	62.27	16.56	RMRP	RNA component of mitochondrial RNA processing endoribonuclease	chr9	lincRNA
ENSG00000259215.1	3.10258254	-2.6126038	0.00943894	0.02469931	0.07	0	0.16	0.77	RP11-253M7	--	chr15	lincRNA
ENSG00000139323.12	2.09080643	0.04683616	1.10E-06	6.28E-06	0.1	0.07	0.38	0.58	POC1B	POC1 centriolar protein B	chr12	protein_coding
ENSG00000163590.12	1.32605426	1.00914495	0.00018627	0.00070708	0.02	0.13	0.22	0.28	PPM1L	protein phosphatase, Mg2+/Mn2+ dependent, 1L	chr3	protein_coding
ENSG00000263874.1	6.81605451	-1.7941831	1.35E-07	8.87E-07	0	0	0.19	0.2	LINC00672	long intergenic non-protein coding RNA 672	chr17	lincRNA
ENSG00000138443.14	-1.4359247	4.06763553	1.36E-06	7.64E-06	0.82	0.94	0.45	0.41	ABI2	abl-interactor 2	chr2	protein_coding
ENSG00000275143.1	-1.0563597	-0.4877029	0.01608835	0.03952751	3.43	4.39	1.88	3.06	SCARNA16	small Cajal body-specific RNA 16	chr17	scaRNA
ENSG00000185008.16	-4.1945353	-0.7748406	1.04E-11	1.17E-10	0.05	0.13	0	0.01	ROBO2	roundabout, axon guidance receptor, homolog 2 (Drosophila)	chr3	protein_coding
ENSG00000154358.18	-4.7801959	5.19831814	3.65E-44	3.56E-42	1.89	2.13	0.11	0.09	OBSCN	obscurin, cytoskeletal calmodulin and titin-interacting RhoGEF	chr1	protein_coding
ENSG00000168675.17	-2.6115615	0.92777017	3.44E-12	4.07E-11	0.25	0.23	0.04	0.06	LDLRAD4	low density lipoprotein receptor class A domain containing 4	chr18	protein_coding
ENSG00000113319.10	-2.7629557	-1.1023001	8.65E-07	5.03E-06	0.04	0.11	0	0.03	RASGRF2	Ras protein-specific guanine nucleotide-releasing factor 2	chr5	protein_coding
ENSG00000210049.1	4.90663588	2.04807318	1.07E-31	5.33E-30	0.75	5.19	156.1	79.01	MT-TF	mitochondrially encoded tRNA phenylalanine	chrM	Mt_tRNA
ENSG00000101470.8	1.79953002	-0.0539935	2.10E-05	9.59E-05	0.37	0.36	2.05	1.34	TNNC2	troponin C type 2 (fast)	chr20	protein_coding
ENSG00000127928.11	-5.2109276	-3.0346387	0.00538165	0.01488851	0.02	0.07	0	0	GNGT1	guanine nucleotide binding protein (G protein), gamma transducing activity polypeptide 1	chr7	protein_coding
ENSG00000179361.16	1.00957705	3.51689972	0.00069857	0.00237667	1.5	1.35	3.89	3.77	ARID3B	AT rich interactive domain 3B (BRIGHT-like)	chr15	protein_coding
ENSG00000177426.19	1.0854516	5.29757316	0.00018296	0.0006954	2.87	3.1	8.14	8.73	TGIF1	TGFB-induced factor homeobox 1	chr18	protein_coding
ENSG00000204936.8	-4.2458331	2.9565681	2.28E-32	1.18E-30	4.75	5.42	0.34	0.37	CD177	CD177 molecule	chr19	protein_coding
ENSG00000260170.1	-2.6552755	4.8503182	5.65E-18	1.16E-16	39.51	36.52	7.37	8.73	RP11-96O20	--	chr15	protein_coding
ENSG00000126106.12	1.06975308	2.2248492	0.00065588	0.00224325	0.85	0.46	2.01	1.68	TMEM53	transmembrane protein 53	chr1	protein_coding
ENSG00000250232.1	-1.8975668	4.52703185	2.33E-10	2.21E-09	25.79	24.22	9.62	8.24	AF196779.12	--	chrX	protein_coding
ENSG00000237190.3	-1.2110227	5.57562237	3.00E-05	0.0001332	31.45	29.89	17.14	18.2	CDKN2AIPNL	CDKN2A interacting protein N-terminal like	chr5	protein_coding
ENSG00000164897.11	-1.7635294	6.41181091	1.86E-09	1.56E-08	56.68	51.26	22.87	19.42	TMUB1	transmembrane and ubiquitin-like domain containing 1	chr7	protein_coding
ENSG00000159658.9	1.62624894	4.39764853	4.65E-08	3.26E-07	1.2	1.69	5.42	6.46	EFCAB14	EF-hand calcium binding domain 14	chr1	protein_coding

ENSG00000140374.14	1.09059955	6.91408382	0.00015272	0.00058927	26.2	28.35	68.58	86.61	ETFA	electron-transfer-flavoprotein, alpha polypeptide	chr15	protein_coding
ENSG00000070614.13	-1.5274824	6.75026989	1.55E-07	1.01E-06	15.41	16.04	7.57	6.94	NDST1	N-deacetylase/N-sulfotransferase (heparan glucosaminyl) 1	chr5	protein_coding
ENSG00000133943.19	-2.9876658	4.14133024	3.38E-21	8.96E-20	5.72	5.67	0.83	1.08	C14orf159	chromosome 14 open reading frame 159	chr14	protein_coding
ENSG00000189057.9	4.05803834	1.17221808	5.22E-21	1.36E-19	0.05	0.06	0.69	1.76	FAM111B	family with sequence similarity 111, member B	chr11	protein_coding
ENSG00000274818.1	2.12482149	2.26836509	1.07E-10	1.07E-09	2.41	3.86	18.59	17.78	RP1-292L20.	--	chr14	lincRNA
ENSG00000047346.11	3.36767579	0.24176094	5.95E-13	7.64E-12	0.01	0.04	0.18	0.41	FAM214A	family with sequence similarity 214, member A	chr15	protein_coding
ENSG00000279765.2	2.54979441	1.48251511	1.82E-12	2.23E-11	0.76	0.81	6.34	5.93	RP11-437B1(	--	chr15	protein_coding
ENSG00000158006.12	-1.3617564	3.72216398	5.09E-06	2.61E-05	4.26	4.18	2.38	1.99	PAFAH2	platelet-activating factor acetylhydrolase 2, 40kDa	chr1	protein_coding
ENSG00000111860.12	4.73595578	-1.4554304	1.96E-07	1.26E-06	0	0	0.05	0.1	CEP85L	centrosomal protein 85kDa-like	chr6	protein_coding
ENSG00000136840.17	-1.705723	6.37092292	5.79E-09	4.60E-08	64.48	56.73	25.93	23.54	ST6GALNAC	ST6 (alpha-N-acetyl-neuraminyl-2,3-beta-galactosyl-1,3)-N-acetylgalactosaminide alpha-2,6-sialyltransferase 4	chr9	protein_coding
ENSG00000235493.1	5.55167921	-2.6864918	0.00158008	0.00495521	0	0	0	0.47	AC092415.1	--	chr3	lincRNA
ENSG00000145908.11	2.22289462	0.33024549	8.19E-08	5.55E-07	0.08	0.07	0.33	0.61	ZNF300	zinc finger protein 300	chr5	protein_coding
ENSG00000273088.1	-2.3190387	1.04841374	2.45E-10	2.32E-09	3.76	3.58	0.98	0.98	RP11-201K1(	--	chr1	protein_coding
ENSG00000273381.1	3.09783676	-2.6152287	0.00943894	0.02469931	0.01	0	0.01	0.08	RP11-305L7.	--	chr9	lincRNA
ENSG00000104321.9	-5.2227349	-1.4452014	4.50E-09	3.61E-08	0.08	0.12	0.01	0	TRPA1	transient receptor potential cation channel, subfamily A, member 1	chr8	protein_coding
ENSG00000182324.6	-1.0238512	2.42566794	0.00093893	0.00310457	1.32	1.35	0.91	0.84	KCNJ14	potassium channel, inwardly rectifying subfamily J, member 14	chr19	protein_coding
ENSG00000133114.16	1.91415901	1.4834119	3.09E-08	2.20E-07	0.06	0.16	0.37	0.69	GPALPP1	GPALPP motifs containing 1	chr13	protein_coding
ENSG00000113272.12	-1.4695058	4.22124447	7.24E-07	4.26E-06	6.71	7.56	3.5	3.37	THG1L	tRNA-histidine guanylyltransferase 1-like (S. cerevisiae)	chr5	protein_coding
ENSG00000198551.8	1.84950659	0.20117719	6.65E-06	3.34E-05	0.13	0.13	0.5	0.77	ZNF627	zinc finger protein 627	chr19	protein_coding
ENSG00000281028.1	1.56224158	-0.7723688	0.00171128	0.00532903	0.12	0.18	0.37	0.8	PI4K2B	phosphatidylinositol 4-kinase type 2 beta	chr4	protein_coding
ENSG00000269720.1	2.14388032	-1.1388948	0.00015583	0.00060009	0	0.26	0.49	1.05	CTD-2521M	--	chr19	lincRNA
ENSG00000280594.1	2.74446279	-2.2426929	0.00174589	0.00541921	0	0.1	0.81	0.2	AP000432.1	--	chr21	processed_transcript
ENSG00000151320.9	2.70106082	-1.5504408	0.00013983	0.00054609	0	0.01	0.03	0.03	AKAP6	A kinase (PRKA) anchor protein 6	chr14	protein_coding
ENSG00000186522.13	2.1151362	2.12239038	1.98E-10	1.90E-09	0.22	0.51	1.15	3.05	10-Sep	septin 10	chr2	protein_coding
ENSG00000162576.15	3.92126437	-2.0772674	5.06E-05	0.00021569	0.01	0	0.08	0.13	MXRA8	matrix-remodelling associated 8	chr1	protein_coding
ENSG00000138375.11	-1.1415423	4.02565337	0.00010828	0.00043261	4.9	6.01	3.47	3.1	SMARCAL1	SWI/SNF related, matrix associated, actin dependent regulator of chromatin, subfamily a-like 1	chr2	protein_coding
ENSG00000077150.16	-1.3428655	7.92855298	3.41E-06	1.80E-05	72.84	67.45	38.3	35.33	NFKB2	nuclear factor of kappa light polypeptide gene enhancer in B-cells 2 (p49/p100)	chr10	protein_coding
ENSG00000115239.20	1.66328239	3.08135507	6.54E-08	4.50E-07	1.13	1.18	4.82	4.92	GPR75-ASB3	GPR75-ASB3 readthrough	chr2	protein_coding
ENSG00000197712.10	-1.2910886	3.65105499	1.50E-05	7.09E-05	2.9	4.12	1.98	1.83	FAM114A1	family with sequence similarity 114, member A1	chr4	protein_coding
ENSG00000174749.5	-1.0155455	1.57932346	0.002013	0.00616687	0.33	0.39	0.26	0.21	C4orf32	chromosome 4 open reading frame 32	chr4	protein_coding
ENSG00000275532.1	-1.493209	-1.080033	0.00587472	0.01613343	0.94	1.17	0.79	0.22	CTB-58E17.3	--	chr17	antisense
ENSG00000155066.14	-4.4867843	0.04074009	3.92E-17	7.49E-16	0.36	0.3	0.04	0	PROM2	prominin 2	chr2	protein_coding
ENSG00000071626.15	-1.637523	8.50794952	1.89E-08	1.38E-07	190.33	198.83	83.89	82.68	DAZAP1	DAZ associated protein 1	chr19	protein_coding
ENSG00000204628.10	-1.8114697	11.0521131	5.94E-10	5.34E-09	1505.79	1349.99	554.73	529.09	GNB2L1	guanine nucleotide binding protein (G protein), beta polypeptide 2-like 1	chr5	protein_coding
ENSG00000137692.10	1.51979702	5.84439529	1.98E-07	1.27E-06	16.26	19.69	58.41	79.37	DCUN1D5	DCN1, defective in cullin neddylation 1, domain containing 5	chr11	protein_coding
ENSG00000122435.8	2.37158318	-0.3631318	8.80E-07	5.11E-06	0.09	0.02	0.16	0.56	TRMT13	tRNA methyltransferase 13 homolog (S. cerevisiae)	chr1	protein_coding
ENSG00000169902.12	-1.3459025	5.18464684	3.98E-06	2.08E-05	19.72	20.21	9.75	11.21	TPST1	tyrosylprotein sulfotransferase 1	chr7	protein_coding
ENSG00000276027.1	1.96029974	-2.0544475	0.01397446	0.03477946	0.36	0.38	2.78	1.27	RNU12	RNA, U12 small nuclear	chr22	snRNA
ENSG00000185808.12	1.47009438	2.01455707	5.36E-06	2.73E-05	0.22	0.23	0.61	1.06	PIGP	phosphatidylinositol glycan anchor biosynthesis, class P	chr21	protein_coding
ENSG00000101096.18	2.20065141	-0.404534	3.69E-06	1.94E-05	0.04	0	0.12	0.18	NFATC2	nuclear factor of activated T-cells, cytoplasmic, calcineurin-dependent 2	chr20	protein_coding
ENSG00000126603.7	-2.1269261	5.53704167	9.72E-13	1.22E-11	13.46	13.92	4.44	3.9	GLIS2	GLIS family zinc finger 2	chr16	protein_coding
ENSG00000277782.1	2.71911887	-1.0677054	5.07E-06	2.60E-05	0.41	0	1.73	2.1	RP11-775C2	--	chr15	lincRNA
ENSG00000125691.11	-1.0006407	9.62351043	0.00048071	0.00168228	278.22	271.68	176.37	190.18	RPL23	ribosomal protein L23	chr17	protein_coding
ENSG00000100722.17	1.07737929	4.09145279	0.00025839	0.00095782	0.4	0.52	1.08	1.53	ZC3H14	zinc finger CCCH-type containing 14	chr14	protein_coding
ENSG00000137573.12	-8.3722983	-0.6245513	1.25E-16	2.30E-15	0.27	0.05	0	0	SULF1	sulfatase 1	chr8	protein_coding
ENSG00000165929.11	2.41694417	-0.1327907	2.21E-07	1.41E-06	0.02	0.06	0.09	0.45	TC2N	tandem C2 domains, nuclear	chr14	protein_coding
ENSG00000147124.11	1.43837413	0.5945367	0.00012761	0.00050255	0.11	0.16	0.43	0.53	ZNF41	zinc finger protein 41	chrX	protein_coding
ENSG00000163191.5	-1.6671328	8.45722619	1.08E-08	8.20E-08	628.56	830.72	349.77	259.11	S100A11	S100 calcium binding protein A11	chr1	protein_coding
ENSG00000164330.15	-3.9557246	0.97635749	2.58E-20	6.34E-19	0.45	0.69	0.07	0.03	EBF1	early B-cell factor 1	chr5	protein_coding
ENSG00000123843.11	-7.6965424	3.1473065	6.63E-58	1.44E-55	14.02	8.79	0.05	0.09	C4BPB	complement component 4 binding protein, beta	chr1	protein_coding
ENSG00000231728.3	5.44044058	-2.7547732	0.00290522	0.00856312	0	0	0	0.25	LL0XNC01-1	--	chrX	antisense
ENSG00000008056.11	-4.8429195	-1.0407663	6.98E-12	7.96E-11	0.26	0.19	0	0.02	SYN1	synapsin I	chrX	protein_coding
ENSG00000255337.1	4.05200644	-1.286006	2.06E-07	1.32E-06	0.08	0	0.83	1.1	RP11-315O6	--	chr11	antisense
ENSG00000149531.13	-4.6628729	0.90118462	2.51E-23	7.69E-22	2.12	3.51	0.25	0.05	FRG1B	FSHD region gene 1 family, member B	chr20	protein_coding
ENSG00000135116.8	1.82465428	1.69187023	6.85E-08	4.70E-07	0.18	0.21	0.92	0.9	HRK	harakiri, BCL2 interacting protein	chr12	protein_coding
ENSG00000139350.10	2.28521885	0.89879837	1.54E-09	1.32E-08	0.04	0.19	0.61	0.86	NEDD1	neural precursor cell expressed, developmentally down-regulated 1	chr12	protein_coding
ENSG00000174989.11	1.26318938	2.55940975	4.92E-05	0.00021041	0.42	0.72	1.87	1.77	FBXW8	F-box and WD repeat domain containing 8	chr12	protein_coding
ENSG00000072042.11	1.74349602	5.43593336	3.34E-09	2.74E-08	5.79	6.65	22.22	33.51	RDH11	retinol dehydrogenase 11 (all-trans/9-cis/11-cis)	chr14	protein_coding
ENSG00000217555.11	1.2428943	5.13536697	2.00E-05	9.20E-05	22.81	17.75	62.6	65.59	CKLF	chemokine-like factor	chr16	protein_coding
ENSG00000107282.6	4.32373293	-1.7842079	1.62E-06	9.05E-06	0	0	0.08	0.05	APBA1	amyloid beta (A4) precursor protein-binding, family A, member 1	chr9	protein_coding
ENSG00000103888.14	2.74852468	3.80057526	3.86E-18	7.99E-17	0.46	0.32	3.48	3.55	CEMIP	cell migration inducing protein, hyaluronan binding	chr15	protein_coding
ENSG00000039139.9	-3.286662	-1.0182238	3.09E-08	2.20E-07	0.04	0.05	0.01	0	DNAH5	dynein, axonemal, heavy chain 5	chr5	protein_coding
ENSG00000183770.5	1.00861671	5.01409202	0.0005123	0.00178657	6.43	5.44	16.87	14.93	FOXL2	forkhead box L2	chr3	protein_coding
ENSG00000231536.1	1.93112369	-1.1568623	0.00060303	0.00208087	0.32	0	0.64	1.06	AC123886.2	--	chr2	antisense
ENSG00000185070.9	-3.1011013	-0.5266283	1.01E-09	8.82E-09	0.02	0.04	0	0	FLRT2	fibronectin leucine rich transmembrane protein 2	chr14	protein_coding
ENSG00000081377.15	1.62805729	0.46244042	2.40E-05	0.00010869	0.08	0.08	0.28	0.39	CDC14B	cell division cycle 14B	chr9	protein_coding
ENSG00000225891.1	2.99529641	-2.6745053	0.00943894	0.02469931	0	0.05	0.18	0.46	RP3-476K8.3	--	chr1	antisense
ENSG00000213465.6	-1.5309821	6.16448682	1.53E-07	1.00E-06	90.78	85.39	43.09	38.02	ARL2	ADP-ribosylation factor-like 2	chr11	protein_coding
ENSG00000254166.1	-5.0197065	-3.1311226	0.0100455	0.02582742	0.44	0.08	0	0	CASC19	cancer susceptibility candidate 19 (non-protein coding)	chr8	lincRNA
ENSG00000171425.8	-1.8476241	4.85509427	5.51E-10	4.98E-09	24.97	21.3	8.82	8.31	ZNF581	zinc finger protein 581	chr19	protein_coding
ENSG00000172361.5	2.38585956	-0.9673566	3.95E-05	0.00017233	0.03	0.09	0.48	0.37	CFAP53	cilia and flagella associated protein 53	chr18	protein_coding
ENSG00000137198.8	-1.2736105	3.07157761	2.71E-05	0.00012153	6.09	6.58	3.72	3.25	GMPR	guanosine monophosphate reductase	chr6	protein_coding
ENSG00000243477.4	-1.0097298	3.70715429	0.00064818	0.00222098	7.61	6.99	5.54	4.09	NAT6	N-acetyltransferase 6 (GCN5-related)	chr3	protein_coding
ENSG00000089060.10	1.36900977	5.27767274	2.76E-06	1.49E-05	3.62	3.91	13.81	12.04	SLC8B1	solute carrier family 8 (sodium/lithium/calcium exchanger), member B1	chr12	protein_coding
ENSG00000157168.17	1.04644485	0.88920406	0.00298877	0.00879425	0.12	0.08	0.18	0.38	NRG1	neuregulin 1	chr8	protein_coding
ENSG00000187808.4	5.35568693	0.97973834	3.32E-25	1.16E-23	0.03	0.05	2.66	2.39	SOWAHD	sosondowah ankyrin repeat domain family member D	chrX	protein_coding
ENSG00000130255.11	-1.1318821	9.31710615	8.21E-05	0.00033755	507.19	506.69	324.7	290.75	RPL36	ribosomal protein L36	chr19	protein_coding
ENSG00000128965.10	-1.6143207	4.28983629	5.84E-08	4.05E-07	11.88	11.29	5.95	4.1	CHAC1	ChaC glutathione-specific gamma-glutamylcyclotransferase 1	chr15	protein_coding
ENSG00000133858.14	2.01828172	1.51704819	6.63E-09	5.23E-08	0.09	0.13	0.42	0.8	ZFC3H1	zinc finger, C3H1-type containing	chr12	protein_coding
ENSG00000163898.8	4.68067354	-0.2784436	2.87E-14	4.25E-13	0	0.02	0.32	0.43	LIPH	lipase, member H	chr3	protein_coding
ENSG00000171532.4	5.32944093	-2.821228	0.00290522	0.00856312	0	0	0.03	0.12	NEUROD2	neuronal differentiation 2	chr17	protein_coding
ENSG00000179826.6	3.22108013	-0.1562288	1.39E-10	1.37E-09	0.08	0.07	1.07	0.85	MRGPRX3	MAS-related GPR, member X3	chr11	protein_coding
ENSG00000188157.12	-1.9932509	9.24948795	1.29E-11	1.43E-10	112.44	100.51	38.11	33.05	AGRN	agrin	chr1	protein_coding
ENSG00000250571.5	1.17356809	3.26571415	9.87E-05	0.00039794	1.94	2.2	6.94	5.45	GLI4	GLI family zinc finger 4	chr8	protein_coding
ENSG00000148019.11	2.39470335	2.49009693	3.49E-13	4.59E-12	0.1	0.15	0.51	1.22	CEP78	centrosomal protein 78kDa	chr9	protein_coding
ENSG00000135604.9	1.1936884	-0.1642627	0.00484505	0.01366418	0.1	0.32	0.68	0.59	STX11	syntaxin 11	chr6	protein_coding
ENSG00000281902.1	-1.7446289	-1.4111577	0.00194789	0.00597963	4.46	2.36	1.07	1.59	AC091053.2	--	chr11	miRNA
ENSG00000067064.9	2.49267882	3.46623827	3.06E-15	4.99E-14	1.2	1.12	5.99	11.55	IDI1	isopentenyl-diphosphate delta isomerase 1	chr10	protein_coding
ENSG00000134830.5	-8.5011889	6.54979675	3.09E-92	4.12E-89	21.46	23.06	0.06	0.1	C5AR2	complement component 5a receptor 2	chr19	protein_coding
ENSG00000259945.1	3.27276373	-0.4350387	1.80E-09	1.52E-08	0.32	0.25	4.29	3.32	CTD-2012K1	--	chr16	antisense
ENSG00000247081.6	1.78085814	-0.9056924	0.0007615	0.00257252	0.04	0.04	0.21	0.19	BAALC-AS1	BAALC antisense RNA 1	chr8	antisense
ENSG00000143952.18	2.934986	1.30615991	1.20E-14	1.85E-13	0.11	0.07	0.63	1.18	VPS54	vacuolar protein sorting 54 homolog (S. cerevisiae)	chr2	protein_coding
ENSG00000119328.10	1.15785915	0.75122768	0.00159996	0.00500773	0.27	0.41	0.47	1.54	FAM206A	family with sequence similarity 206, member A	chr9	protein_coding
ENSG00000196586.12	3.54408754	2.15247492	3.30E-22	9.40E-21	0.07	0.06	0.8	1.18	MYO6	myosin VI	chr6	protein_coding
ENSG00000250091.2	-3.4744379	-2.118789	3.06E-05	0.00013563	0.07	0.04	0	0.01	DNAH10OS	dynein, axonemal, heavy chain 10 opposite strand	chr12	protein_coding
ENSG00000132330.15	-1.0590775	4.28583155	0.00030254	0.00110102	6.37	6.19	3.92	4.12	SCLY	selenocysteine lyase	chr2	protein_coding
ENSG00000273590.3	-1.7066094	-1.6685087	0.00945988	0.02474059	0.08	0.09	0.03	0.03	SMIM11	small integral membrane protein 11	chr21	protein_coding
ENSG00000170312.14	2.28690475	2.49133243	2.46E-12	2.97E-11	0.7	0.79	3.32	6.43	CDK1	cyclin-dependent kinase 1	chr10	protein_coding
ENSG00000078018.18	3.07702495	4.14121595	5.62E-22	1.57E-20	0.27	0.31	2.69	3.94	MAP2	microtubule-associated protein 2	chr2	protein_coding
ENSG00000174705.10	-2.3287659	5.34549415	1.19E-14	1.84E-13	7.27	6.49	2.04	1.6	SH3PXD2B	SH3 and PX domains 2B	chr5	protein_coding
ENSG00000204580.10	-1.0928697	6.7814225	0.00014838	0.00057423	23.52	21.93	14.75	13.62	DDR1	discoidin domain receptor tyrosine kinase 1	chr6	protein_coding
ENSG00000142623.9	-1.9503292	-0.280651	7.09E-06	3.54E-05	0.3	0.23	0.05	0.13	PADI1	peptidyl arginine deiminase, type I	chr1	protein_coding
ENSG00000181649.5	-1.0937544	6.08164676	0.00015193	0.00058649	82.81	73.42	50.85	46.62	PHLDA2	pleckstrin homology-like domain, family A, member 2	chr11	protein_coding
ENSG00000254615.2	-5.2426453	3.61862091	2.58E-44	2.55E-42	14.57	14.86	0.78	0.27	RP11-395G2	--	chr8	lincRNA
ENSG00000164620.7	-1.1961487	4.19554233	4.87E-05	0.00020818	8.3	7.58	4.76	4.47	RELL2	RELT-like 2	chr5	protein_coding
ENSG00000131067.15	1.21559168	4.13558645	3.84E-05	0.00016801	3.45	2.99	9.7	10.28	GGT7	gamma-glutamyltransferase 7	chr20	protein_coding
ENSG00000004897.10	1.84431489	3.2613576	2.22E-09	1.86E-08	0.59	0.56	2.11	3.42	CDC27	cell division cycle 27	chr17	protein_coding
ENSG00000272768.1	1.81950244	1.5818769	9.51E-08	6.39E-07	0.59	0.85	3.51	3.25	RP4-673M15	--	chr7	antisense
ENSG00000223638.3	4.92065931	-3.045493	0.0100455	0.02582742	0	0	0.25	0	RFPL4A	ret finger protein-like 4A	chr19	protein_coding
ENSG00000197044.9	7.15027942	-1.5204842	4.48E-09	3.59E-08	0	0	0.07	0.19	ZNF441	zinc finger protein 441	chr19	protein_coding
ENSG00000088766.10	1.07956366	3.01943405	0.0003815	0.00136537	1.01	1.01	2.16	3.57	CRLS1	cardiolipin synthase 1	chr20	protein_coding
ENSG00000010404.16	-1.652677	2.90694761	7.97E-08	5.43E-07	1.1	1.08	0.45	0.47	IDS	iduronate 2-sulfatase	chrX	protein_coding
ENSG00000232063.1	2.37824709	-0.5690232	3.30E-06	1.75E-05	0.04	0.1	0.34	0.64	RP11-307E1	--	chr9	lincRNA
ENSG00000276814.1	3.00013785	-2.6718476	0.00943894	0.02469931	0	0.03	0.13	0.22	RP1-228P16.	--	chr12	lincRNA
ENSG00000112144.14	2.65337954	-0.0435892	6.69E-09	5.27E-08	0.03	0.03	0.2	0.32	ICK	intestinal cell (MAK-like) kinase	chr6	protein_coding
ENSG00000176148.14	1.35639674	2.64563258	1.25E-05	5.97E-05	1.04	0.82	2.84	3.53	TCP11L1	t-complex 11, testis-specific-like 1	chr11	protein_coding
ENSG00000135407.9	1.44903776	-1.3571272	0.01151564	0.0292584	0.08	0.01	0.23	0.12	AVIL	advillin	chr12	protein_coding
ENSG00000101190.11	-1.0555603	5.43191742	0.00026475	0.0009747	12.09	15.4	7.64	10.01	TCFL5	transcription factor-like 5 (basic helix-loop-helix)	chr20	protein_coding
ENSG00000165568.16	4.48443239	2.11873291	3.22E-29	1.44E-27	0.11	0.24	5.95	4.72	AKR1E2	aldo-keto reductase family 1, member E2	chr10	protein_coding
ENSG00000154065.15	-1.5139902	1.54656322	4.78E-06	2.47E-05	0.79	0.93	0.35	0.45	ANKRD29	ankyrin repeat domain 29	chr18	protein_coding
ENSG00000257594.3	1.70199696	-0.5946139	0.00033978	0.00122705	0.03	0.05	0.16	0.2	GALNT4	polypeptide N-acetylgalactosaminyltransferase 4	chr12	protein_coding
ENSG00000213977.6	1.17696248	5.83073851	4.85E-05	0.00020759	19.59	21.23	60.94	62	TAX1BP3	Tax1 (human T-cell leukemia virus type I) binding protein 3	chr17	protein_coding
ENSG00000069493.13	2.87624075	-0.7493914	4.37E-07	2.67E-06	0.01	0.03	0.13	0.23	CLEC2D	C-type lectin domain family 2, member D	chr12	protein_coding
ENSG00000071859.13	-2.2159862	8.96961618	8.23E-14	1.16E-12	439.88	549.39	146.59	136.5	FAM50A	family with sequence similarity 50, member A	chrX	protein_coding
ENSG00000177335.9	-6.3740419	-0.4168397	1.40E-18	3.02E-17	0.74	0.5	0.02	0	C8orf31	chromosome 8 open reading frame 31	chr8	processed_transcript
ENSG00000004766.14	1.86451225	-0.1868331	2.88E-05	0.00012856	0.05	0.04	0.17	0.28	CCDC132	coiled-coil domain containing 132	chr7	protein_coding
ENSG00000197557.6	1.46825532	-0.4681089	0.00169689	0.00528559	0.04	0.05	0.11	0.23	TTC30A	tetratricopeptide repeat domain 30A	chr2	protein_coding
ENSG00000130299.15	-1.2719273	5.56345608	1.20E-05	5.75E-05	20.24	17.34	10.5	10.25	GTPBP3	GTP binding protein 3 (mitochondrial)	chr19	protein_coding
ENSG00000182870.11	6.41244915	0.79387287	1.12E-26	4.33E-25	0.02	0	1.34	1.06	GALNT9	polypeptide N-acetylgalactosaminyltransferase 9	chr12	protein_coding
ENSG00000026025.12	-5.282429	8.69378521	3.14E-54	5.71E-52	310.7	301.33	11.01	9.92	VIM	vimentin	chr10	protein_coding
ENSG00000259366.1	4.89980841	-3.0567113	0.01889873	0.04519557	0	0	0.23	0.2	CTD-2647L4	--	chr8	sense_intronic
ENSG00000175600.14	1.71271886	1.27705478	8.55E-07	4.98E-06	0.56	0.46	1.84	2.64	SUGCT	succinyl-CoA:glutarate-CoA transferase	chr7	protein_coding
ENSG00000068831.17	-2.3073484	-1.7298333	0.00158671	0.00496952	0.1	0.06	0.05	0	RASGRP2	RAS guanyl releasing protein 2 (calcium and DAG-regulated)	chr11	protein_coding
ENSG00000005020.11	2.59323184	1.6685339	2.57E-13	3.42E-12	0.13	0.22	0.95	1.9	SKAP2	src kinase associated phosphoprotein 2	chr7	protein_coding
ENSG00000241720.2	-6.1871196	-2.4408021	4.74E-05	0.00020301	0.36	0.18	0	0	RP4-735C1.4	--	chr1	antisense
ENSG00000210195.2	2.57450956	-0.6829212	1.51E-06	8.44E-06	2.84	0.86	19.41	11.14	MT-TT	mitochondrially encoded tRNA threonine	chrM	Mt_tRNA
ENSG00000100519.10	1.35405332	3.4475203	7.10E-06	3.54E-05	1.64	2.88	5.58	9.84	PSMC6	proteasome (prosome, macropain) 26S subunit, ATPase, 6	chr14	protein_coding
ENSG00000272931.1	1.00060712	0.98429303	0.00435144	0.01238574	0.89	2.08	3.6	4.27	RP5-943J3.2	--	chr1	lincRNA
ENSG00000275329.1	1.76240365	-0.0730246	3.71E-05	0.00016263	0.13	0.22	0.45	1.11	RP11-83N9.6	--	chr9	lincRNA
ENSG00000188158.13	9.6205977	0.68100275	7.68E-28	3.17E-26	0	0	0.23	0.4	NHS	Nance-Horan syndrome (congenital cataracts and dental anomalies)	chrX	protein_coding
ENSG00000260892.1	2.89449306	-2.7319096	0.01615662	0.03958166	0	0.05	0.4	0.19	CTD-2026K1	--	chr15	antisense
ENSG00000119636.14	1.32043854	-0.3937376	0.00291737	0.00859154	0.14	0.05	0.31	0.33	CCDC176	coiled-coil domain containing 176	chr14	protein_coding
ENSG00000243766.6	1.86615627	-1.0729103	0.0009149	0.00303221	0.03	0.02	0.15	0.12	HOTTIP	HOXA distal transcript antisense RNA	chr7	antisense
ENSG00000206532.2	5.48410531	-2.7306244	0.00158008	0.00495521	0	0	0.34	0.1	RP11-553A1	--	chr3	lincRNA
ENSG00000275342.3	-2.1209016	4.06524889	3.23E-12	3.84E-11	4.51	4.47	1.38	1.37	SGK223	--	chr8	protein_coding
ENSG00000134762.15	-8.5525616	-0.4590943	3.56E-18	7.37E-17	0.12	0.2	0	0	DSC3	desmocollin 3	chr18	protein_coding
ENSG00000125703.13	1.68173339	0.5827567	1.25E-05	5.96E-05	0.08	0.29	0.44	1.14	ATG4C	autophagy related 4C, cysteine peptidase	chr1	protein_coding
ENSG00000119979.14	2.05978476	2.56929802	1.39E-10	1.37E-09	0.6	1.08	4.08	5.27	FAM45A	family with sequence similarity 45, member A	chr10	protein_coding
ENSG00000280248.1	5.06713725	-2.9688687	0.0100455	0.02582742	0	0	0.07	0.05	CTD-2047H1	--	chr17	TEC
ENSG00000128923.9	2.49648035	1.34539657	6.96E-12	7.95E-11	0.07	0.06	0.39	0.58	FAM63B	family with sequence similarity 63, member B	chr15	protein_coding
ENSG00000172738.10	1.51284748	-0.5085165	0.00138669	0.00444053	0.19	0.14	0.65	0.62	TMEM217	transmembrane protein 217	chr6	protein_coding
ENSG00000152270.7	1.79770283	0.0585767	2.25E-05	0.00010217	0.07	0.05	0.2	0.33	PDE3B	phosphodiesterase 3B, cGMP-inhibited	chr11	protein_coding
ENSG00000089091.15	3.33059048	0.20496604	1.13E-12	1.41E-11	0.02	0.05	0.42	0.53	DZANK1	double zinc ribbon and ankyrin repeat domains 1	chr20	protein_coding
ENSG00000126858.15	1.81674331	1.9019343	4.83E-08	3.38E-07	0.18	0.38	0.85	1.77	RHOT1	ras homolog family member T1	chr17	protein_coding
ENSG00000187860.9	1.23007283	2.42523941	8.52E-05	0.00034604	0.73	0.57	1.9	2.21	CCDC157	coiled-coil domain containing 157	chr22	protein_coding
ENSG00000158104.10	-6.6380907	0.64878952	3.64E-28	1.53E-26	1.33	1.54	0.04	0	HPD	4-hydroxyphenylpyruvate dioxygenase	chr12	protein_coding
ENSG00000010318.18	1.09241407	2.43361273	0.0004229	0.0015001	0.97	1.5	3.68	3.33	PHF7	PHD finger protein 7	chr3	protein_coding
ENSG00000135521.8	1.21407737	3.3647899	5.41E-05	0.00022923	1.95	3.45	5.72	11.06	LTV1	LTV1 ribosome biogenesis factor	chr6	protein_coding
ENSG00000135828.10	3.33593383	0.53162399	7.03E-14	9.98E-13	0.03	0.04	0.28	0.58	RNASEL	ribonuclease L (2',5'-oligoisoadenylate synthetase-dependent)	chr1	protein_coding
ENSG00000227533.4	1.26252248	-0.8946376	0.01049292	0.02688216	0.17	0.23	0.71	0.56	SLC2A1-AS1	SLC2A1 antisense RNA 1	chr1	lincRNA
ENSG00000171877.18	3.19683744	0.20386506	3.83E-12	4.50E-11	0.03	0.03	0.31	0.41	FRMD5	FERM domain containing 5	chr15	protein_coding
ENSG00000273473.1	-2.9917676	-0.1769213	8.50E-11	8.57E-10	2.1	2.23	0.22	0.48	LL09NC01-1	--	chr9	lincRNA
ENSG00000280278.1	4.06923606	-1.9746469	1.86E-05	8.57E-05	0.01	0	0.26	0.12	FLJ30679	--	chr16	TEC
ENSG00000162437.13	2.07762216	1.42171962	3.43E-09	2.80E-08	0.19	0.17	0.91	1.14	RAVER2	ribonucleoprotein, PTB-binding 2	chr1	protein_coding
ENSG00000039068.17	3.22598849	0.51654017	1.01E-13	1.41E-12	0.08	0	0.54	0.5	CDH1	cadherin 1, type 1, E-cadherin (epithelial)	chr16	protein_coding
ENSG00000189143.9	-6.0687598	3.00012445	1.19E-47	1.45E-45	3.33	2.76	0.06	0.06	CLDN4	claudin 4	chr7	protein_coding
ENSG00000137414.5	1.56853922	0.59167783	3.69E-05	0.00016173	0.14	0.11	0.32	0.66	FAM8A1	family with sequence similarity 8, member A1	chr6	protein_coding
ENSG00000087510.6	1.86888053	4.8423306	3.77E-10	3.50E-09	3.53	3.41	16.32	17.49	TFAP2C	transcription factor AP-2 gamma (activating enhancer binding protein 2 gamma)	chr20	protein_coding
ENSG00000163530.4	-5.653126	-2.7855647	0.00086564	0.00288128	0.21	0	0	0	DPPA2	developmental pluripotency associated 2	chr3	protein_coding
ENSG00000259070.4	-6.0932521	-2.5045083	8.36E-05	0.00034052	0.08	0.01	0	0	LINC00639	long intergenic non-protein coding RNA 639	chr14	lincRNA
ENSG00000131773.12	-3.7150379	2.39393855	2.40E-25	8.53E-24	2.84	3.93	0.27	0.41	KHDRBS3	KH domain containing, RNA binding, signal transduction associated 3	chr8	protein_coding
ENSG00000185837.3	1.32900469	-1.0165595	0.01161423	0.02949325	0.25	0.36	0.71	1.34	CECR5-AS1	CECR5 antisense RNA 1	chr22	antisense
ENSG00000230798.4	1.42006116	3.65705402	2.17E-06	1.19E-05	5.45	3.66	15.94	16.64	FOXD3-AS1	FOXD3 antisense RNA 1 (head to head)	chr1	antisense
ENSG00000070269.12	2.657366	1.27297333	1.23E-12	1.53E-11	0.11	0.13	0.84	1.18	TMEM260	transmembrane protein 260	chr14	protein_coding
ENSG00000234147.1	-4.8079387	-3.2306674	0.01889873	0.04519557	0.12	0.25	0	0	RP3-460G2.2	--	chr6	lincRNA
ENSG00000279425.1	-5.3742532	-2.9467828	0.00290522	0.00856312	0.06	0.05	0	0	CTD-2017D1	--	chr19	TEC
ENSG00000163867.15	4.01186828	-0.8486185	1.79E-09	1.51E-08	0.01	0	0.05	0.24	ZMYM6	zinc finger, MYM-type 6	chr1	protein_coding
ENSG00000180422.3	7.71074103	-1.0553577	1.76E-12	2.16E-11	0	0	0.36	0.4	LINC00304	long intergenic non-protein coding RNA 304	chr16	lincRNA
ENSG00000279878.1	4.91020659	-3.0511096	0.0100455	0.02582742	0	0	0.21	0.06	RP11-286N2	--	chr11	TEC
ENSG00000259264.4	-6.9843499	-1.8429092	4.98E-08	3.47E-07	0.23	0.1	0	0	RP11-60L3.1	--	chr15	antisense
ENSG00000177733.6	-1.0864974	8.07234038	0.00015645	0.00060237	35.88	31.99	20.85	21.78	HNRNPA0	heterogeneous nuclear ribonucleoprotein A0	chr5	protein_coding

ENSG00000124659.6	-1.0263624	5.31447156	0.00039154	0.00139818	27.36	26.08	15.46	19.6	TBCC	tubulin folding cofactor C	chr6	protein_coding
ENSG00000279204.1	-5.3787292	-2.9446861	0.00290522	0.00856312	0	0.11	0	0	RP11-175K6.	--	chr5	TEC
ENSG00000157540.18	1.92718826	3.12316122	5.71E-10	5.14E-09	0.25	0.4	1.44	1.85	DYRK1A	dual-specificity tyrosine-(Y)-phosphorylation regulated kinase 1A	chr21	protein_coding
ENSG00000215182.8	-4.4755733	-0.518271	8.36E-14	1.18E-12	0.06	0.06	0	0	MUC5AC	mucin 5AC, oligomeric mucus/gel-forming	chr11	protein_coding
ENSG00000073969.17	1.31733069	3.79155813	9.85E-06	4.79E-05	1.46	1.58	4.25	5.89	NSF	N-ethylmaleimide-sensitive factor	chr17	protein_coding
ENSG00000065361.13	4.31831262	3.36826934	8.74E-34	4.83E-32	0.09	0.12	3.08	2.55	ERBB3	erb-b2 receptor tyrosine kinase 3	chr12	protein_coding
ENSG00000247746.4	6.33087282	-2.1624822	8.95E-06	4.38E-05	0	0	0.08	0.07	USP51	ubiquitin specific peptidase 51	chrX	protein_coding
ENSG00000156709.12	-1.0760803	6.88513573	0.00018549	0.00070448	41.24	38.51	24.35	26.1	AIFM1	apoptosis-inducing factor, mitochondrion-associated, 1	chrX	protein_coding
ENSG00000115353.9	-6.4354995	-2.265336	5.19E-06	2.66E-05	0.04	0.06	0	0	TACR1	tachykinin receptor 1	chr2	protein_coding
ENSG00000197763.11	1.22285925	3.16510836	5.31E-05	0.00022536	1.3	0.96	2.86	4.21	TXNRD3	thioredoxin reductase 3	chr3	protein_coding
ENSG00000064601.15	1.31326233	8.20197642	5.45E-06	2.78E-05	58.07	58.95	204.76	181.99	CTSA	cathepsin A	chr20	protein_coding
ENSG00000280564.1	1.37858992	-1.0804627	0.01224023	0.03086062	0.78	0.25	1.4	2.22	MAN2C1	mannosidase, alpha, class 2C, member 1	chr15	processed_transcript
ENSG00000277224.1	1.81168437	0.31174108	5.40E-06	2.75E-05	0.98	1.19	6.14	4.07	HIST1H2BF	histone cluster 1, H2bf	chr6	protein_coding
ENSG00000145241.9	2.89865464	0.34049002	4.23E-11	4.41E-10	0.02	0.04	0.18	0.46	CENPC	centromere protein C	chr4	protein_coding
ENSG00000257509.1	3.496945	-2.3667948	0.00112121	0.00364442	0	0.05	0.42	0.51	RP11-762I7.	--	chr12	sense_intronic
ENSG00000023734.9	-1.05601	6.57282226	0.00024496	0.00091296	50.26	59.01	28.02	42.32	STRAP	serine/threonine kinase receptor associated protein	chr12	protein_coding
ENSG00000165476.11	1.55475132	3.29020346	3.50E-07	2.17E-06	0.58	0.96	2.29	3.73	REEP3	receptor accessory protein 3	chr10	protein_coding
ENSG00000049656.12	-1.1081664	9.06209204	0.00011471	0.00045549	237.63	235.33	143.19	149.28	CLPTM1L	CLPTM1-like	chr5	protein_coding
ENSG00000117616.16	2.19613242	3.21066518	2.61E-12	3.14E-11	0.82	1.24	4.89	7.73	RSRP1	arginine/serine-rich protein 1	chr1	protein_coding
ENSG00000108379.8	1.31704607	2.69363074	2.13E-05	9.71E-05	0.66	1.11	2.89	2.96	WNT3	wingless-type MMTV integration site family, member 3	chr17	protein_coding
ENSG00000209082.1	2.19451222	2.24082143	3.00E-11	3.19E-10	12.84	23.05	109.34	108.93	MT-TL1	mitochondrially encoded tRNA leucine 1 (UUA/G)	chrM	Mt_tRNA
ENSG00000266498.1	5.07737931	-2.9633201	0.00538165	0.01488851	0	0	0.33	0.08	RP11-45M22	--	chr17	lincRNA
ENSG00000272973.1	-3.8872927	-2.4433959	0.00066294	0.00226478	0.61	0.05	0.05	0	KB-1125A3.1	--	chr22	antisense
ENSG00000157111.11	2.35063561	2.8195852	2.94E-13	3.89E-12	1.27	1.13	7.78	8.57	TMEM171	transmembrane protein 171	chr5	protein_coding
ENSG00000100350.13	2.8250886	7.13506042	1.83E-20	4.57E-19	5.49	5.39	54.93	47.56	FOXRED2	FAD-dependent oxidoreductase domain containing 2	chr22	protein_coding
ENSG00000117697.13	1.02294872	1.50657897	0.00203474	0.006229	0.44	0.41	0.93	1.4	NSL1	NSL1, MIS12 kinetochore complex component	chr1	protein_coding
ENSG00000136381.11	3.40009247	3.10790703	1.08E-23	3.42E-22	0.1	0.16	1.14	2.41	IREB2	iron-responsive element binding protein 2	chr15	protein_coding
ENSG00000186469.7	-6.011625	1.21746389	1.95E-32	1.01E-30	0.7	0.73	0	0.03	GNG2	guanine nucleotide binding protein (G protein), gamma 2	chr14	protein_coding
ENSG00000174791.9	-2.9473292	6.72815532	6.64E-22	1.84E-20	56.7	49.5	9.43	8.91	RIN1	Ras and Rab interactor 1	chr11	protein_coding
ENSG00000161551.11	8.54399952	-0.3226818	2.65E-17	5.13E-16	0	0	0.41	0.3	ZNF577	zinc finger protein 577	chr19	protein_coding
ENSG00000148356.12	-1.8784515	5.84750334	1.95E-10	1.87E-09	21.41	21.07	7.66	7.74	LRSAM1	leucine rich repeat and sterile alpha motif containing 1	chr9	protein_coding
ENSG00000163155.10	-1.4549956	4.60412578	8.13E-07	4.74E-06	11.42	11.57	5.8	5.36	LYSMD1	LysM, putative peptidoglycan-binding, domain containing 1	chr1	protein_coding
ENSG00000274877.1	6.05040182	0.96818729	8.12E-27	3.15E-25	0.01	0.02	1.11	1.31	RP11-65I12.1	--	chr2	antisense
ENSG00000270091.1	6.63760135	-1.9357476	6.25E-07	3.72E-06	0	0	0.8	0.4	RP11-78O7.2	--	chr17	lincRNA
ENSG00000146066.2	-1.056061	5.33978344	0.00026786	0.00098318	68.87	63.55	46.95	37.71	HIGD2A	HIG1 hypoxia inducible domain family, member 2A	chr5	protein_coding
ENSG00000233515.1	-7.3096941	-1.5763721	1.11E-09	9.61E-09	0.77	1.09	0	0	LINC01518	long intergenic non-protein coding RNA 1518	chr10	lincRNA
ENSG00000165630.12	1.68590064	2.14849056	2.00E-07	1.28E-06	0.51	1.44	3.26	5.08	PRPF18	pre-mRNA processing factor 18	chr10	protein_coding
ENSG00000177225.15	1.21296852	6.16932907	2.78E-05	0.00012447	8.61	7.31	24.51	24.69	PDDC1	Parkinson disease 7 domain containing 1	chr11	protein_coding
ENSG00000127129.8	-2.3141844	3.00698801	2.69E-13	3.57E-12	11.38	6.01	2.32	2.35	EDN2	endothelin 2	chr1	protein_coding
ENSG00000115414.17	-4.2188558	10.4005471	7.54E-39	5.40E-37	224.81	232.05	16.7	15.96	FN1	fibronectin 1	chr2	protein_coding
ENSG00000088205.11	1.86072389	3.64384973	1.07E-09	9.31E-09	1.2	1.08	4.52	6.57	DDX18	DEAD (Asp-Glu-Ala-Asp) box polypeptide 18	chr2	protein_coding
ENSG00000092067.5	2.18248313	-1.4586108	0.00061439	0.00211279	0.03	0.07	0.33	0.33	CEBPE	CCAAT/enhancer binding protein (C/EBP), epsilon	chr14	protein_coding
ENSG00000163535.16	3.72752041	0.13495456	1.17E-13	1.62E-12	0.02	0.03	0.17	0.72	SGOL2	shugoshin-like 2 (S. pombe)	chr2	protein_coding
ENSG00000270154.1	5.21249443	-2.8887793	0.00538165	0.01488851	0	0	0.24	0.21	RP11-419I17	--	chr8	lincRNA
ENSG00000174405.12	3.17484261	-1.9503966	0.00016274	0.00062431	0.01	0	0.06	0.11	LIG4	ligase IV, DNA, ATP-dependent	chr13	protein_coding
ENSG00000260566.2	1.82746309	-0.0871525	1.91E-05	8.80E-05	0.07	0.15	0.54	0.5	RP11-20G6.3	--	chr16	3prime_overlapping_ncrna
ENSG00000168917.8	1.09890536	2.57571961	0.00037416	0.00134039	1.52	1.42	3.42	5	SLC35G2	solute carrier family 35, member G2	chr3	protein_coding
ENSG00000118680.11	1.27026566	6.89881273	1.12E-05	5.38E-05	30.36	33.94	90.13	117.11	MYL12B	myosin, light chain 12B, regulatory	chr18	protein_coding
ENSG00000198707.13	2.27417454	0.12622786	1.30E-07	8.60E-07	0.05	0.01	0.07	0.32	CEP290	centrosomal protein 290kDa	chr12	protein_coding
ENSG00000112304.9	-1.1183345	4.5280803	0.00012939	0.00050899	5.05	6.6	3.51	3.63	ACOT13	acyl-CoA thioesterase 13	chr6	protein_coding
ENSG00000187601.4	-3.2388926	-2.2710156	0.00108225	0.0035312	0.16	0.16	0.02	0.02	MAGEH1	melanoma antigen family H1	chrX	protein_coding
ENSG00000185339.7	-3.4712901	-1.0900467	2.15E-08	1.57E-07	0.27	0.22	0.05	0.01	TCN2	transcobalamin II	chr22	protein_coding
ENSG00000133083.13	-4.3861857	-1.4146018	2.42E-08	1.75E-07	0.04	0.07	0.01	0	DCLK1	doublecortin-like kinase 1	chr13	protein_coding
ENSG00000188859.6	-2.9691886	-0.9149711	1.36E-07	8.92E-07	0.67	0.21	0.08	0.07	FAM78B	family with sequence similarity 78, member B	chr1	protein_coding
ENSG00000234171.2	-1.9546392	3.70875664	1.39E-10	1.36E-09	13.13	14.5	4.44	5.05	RNASEH1-AS	RNASEH1 antisense RNA 1	chr2	antisense
ENSG00000092108.19	2.09631944	1.63638382	1.19E-09	1.03E-08	0.44	0.32	1.68	2.68	SCFD1	sec1 family domain containing 1	chr14	protein_coding
ENSG00000268713.1	2.24876171	0.35307282	5.79E-08	4.01E-07	0.19	0.29	1.04	2.01	CTC-444N24	--	chr19	lincRNA
ENSG00000109099.12	-1.5447719	4.57502438	1.75E-07	1.13E-06	11.32	15.03	5.43	6.61	PMP22	peripheral myelin protein 22	chr17	protein_coding
ENSG00000214940.7	-4.8079387	-3.2306674	0.01889873	0.04519557	0.03	0.07	0	0	NPIPA8	nuclear pore complex interacting protein family, member A8	chr16	protein_coding
ENSG00000104093.12	2.52567995	0.3260025	2.41E-09	2.00E-08	0.03	0.02	0.16	0.24	DMXL2	Dmx-like 2	chr15	protein_coding
ENSG00000079482.12	1.06059965	1.77352262	0.00104254	0.00341232	0.21	0.23	0.55	0.69	OPHN1	oligophrenin 1	chrX	protein_coding
ENSG00000277971.1	1.4082639	1.70273736	1.91E-05	8.80E-05	2.49	1.85	7.26	8.16	XXbac-B562	--	chr22	protein_coding
ENSG00000196787.3	2.64125079	0.48136885	1.56E-10	1.52E-09	0.13	0.13	1.3	0.86	HIST1H2AG	histone cluster 1, H2ag	chr6	protein_coding
ENSG00000198346.9	-4.7336662	-1.8404509	1.01E-06	5.80E-06	0.04	0.08	0.01	0	ZNF813	zinc finger protein 813	chr19	protein_coding
ENSG00000189079.14	1.89884473	1.62199709	2.81E-08	2.02E-07	0.11	0.12	0.57	0.58	ARID2	AT rich interactive domain 2 (ARID, RFX-like)	chr12	protein_coding
ENSG00000137133.9	-1.6210019	4.97982248	3.72E-08	2.63E-07	65.2	57.45	28.56	24.5	HINT2	histidine triad nucleotide binding protein 2	chr9	protein_coding
ENSG00000253563.2	5.33927207	-2.8158158	0.00290522	0.00856312	0	0	0.07	0.11	NKX2-1-AS1	NKX2-1 antisense RNA 1	chr14	antisense
ENSG00000065548.16	1.07108307	3.66214149	0.00031301	0.00113536	2.76	3.38	5.56	11.77	ZC3H15	zinc finger CCCH-type containing 15	chr2	protein_coding
ENSG00000138448.10	1.06004222	3.58271598	0.00037109	0.00133081	0.92	0.81	1.64	3.21	ITGAV	integrin, alpha V	chr2	protein_coding
ENSG00000279406.1	1.68021612	2.10959701	2.30E-07	1.46E-06	0.77	0.76	3.52	2.99	RP11-327E2.	--	chr10	TEC
ENSG00000198681.6	-10.40879	1.35724481	1.73E-38	1.21E-36	2.42	2.44	0	0	MAGEA1	melanoma antigen family A1	chrX	protein_coding
ENSG00000074370.16	-3.1236464	5.53750125	1.39E-23	4.36E-22	14.59	13.02	2.28	1.94	ATP2A3	ATPase, Ca++ transporting, ubiquitous	chr17	protein_coding
ENSG00000175093.4	8.2761737	-0.559313	4.08E-15	6.58E-14	0	0	0.41	0.46	SPSB4	splA/ryanodine receptor domain and SOCS box containing 4	chr3	protein_coding
ENSG00000107581.11	1.11607609	6.81207924	0.00010803	0.00043191	6.85	10.41	20.37	29.61	EIF3A	eukaryotic translation initiation factor 3, subunit A	chr10	protein_coding
ENSG00000170445.11	-1.0737721	7.6168059	0.00018753	0.00071119	44.63	39.36	25.4	27.85	HARS	histidyl-tRNA synthetase	chr5	protein_coding
ENSG00000099284.12	9.07442289	4.84201563	1.61E-79	1.04E-76	0.05	0.03	31.71	30.34	H2AFY2	H2A histone family, member Y2	chr10	protein_coding
ENSG00000135919.11	-2.225687	7.74731485	6.92E-14	9.83E-13	92.34	89.69	23.12	28.88	SERPINE2	serpin peptidase inhibitor, clade E (nexin, plasminogen activator inhibitor type 1), member 2	chr2	protein_coding
ENSG00000118777.9	4.36185332	0.5079062	2.98E-18	6.25E-17	0.04	0	0.58	0.55	ABCG2	ATP-binding cassette, sub-family G (WHITE), member 2 (Junior blood group)	chr4	protein_coding
ENSG00000120370.11	1.96315654	-0.8781721	0.00024605	0.00091688	0.03	0.06	0.11	0.39	GORAB	golgin, RAB6-interacting	chr1	protein_coding
ENSG00000177034.13	2.10863159	0.54056943	7.59E-08	5.17E-07	0.06	0.04	0.21	0.39	MTX3	metaxin 3	chr5	protein_coding
ENSG00000033178.11	2.34227845	1.09269649	3.39E-10	3.17E-09	0.04	0.07	0.17	0.56	UBA6	ubiquitin-like modifier activating enzyme 6	chr4	protein_coding
ENSG00000122420.8	-5.5188333	-2.8647712	0.00158008	0.00495521	0.06	0	0	0	PTGFR	prostaglandin F receptor (FP)	chr1	protein_coding
ENSG00000146477.5	-5.7928307	4.29121377	1.39E-53	2.38E-51	8.1	10.56	0.11	0.33	SLC22A3	solute carrier family 22 (organic cation transporter), member 3	chr6	protein_coding
ENSG00000142731.9	1.97021811	-0.1670728	1.14E-05	5.45E-05	0.02	0.11	0.19	0.51	PLK4	polo-like kinase 4	chr4	protein_coding
ENSG00000148481.12	1.09573018	1.661461	0.00082051	0.00275749	0.36	0.55	0.96	1.65	FAM188A	family with sequence similarity 188, member A	chr10	protein_coding
ENSG00000151474.18	-4.6434774	0.54536902	1.46E-21	4.00E-20	0.19	0.28	0.01	0.02	FRMD4A	FERM domain containing 4A	chr10	protein_coding
ENSG00000180953.10	1.11451553	0.14218328	0.00553249	0.01524948	0.28	0.37	0.81	1.06	ST20	suppressor of tumorigenicity 20	chr15	protein_coding
ENSG00000185885.14	-3.1466785	1.73546385	3.15E-18	6.60E-17	4.24	5.06	0.77	0.63	IFITM1	interferon induced transmembrane protein 1	chr11	protein_coding
ENSG00000165359.13	6.50347774	-2.0333137	1.78E-06	9.85E-06	0	0	0.08	0.11	DDX26B	DEAD/H (Asp-Glu-Ala-Asp/His) box polypeptide 26B	chrX	protein_coding
ENSG00000267360.5	1.56317832	-0.8568151	0.00217107	0.00661139	0.06	0.35	0.6	0.99	CTC-454I21.	--	chr19	protein_coding
ENSG00000105810.8	-1.8968221	0.37653208	9.31E-07	5.38E-06	0.17	0.1	0.05	0.04	CDK6	cyclin-dependent kinase 6	chr7	protein_coding
ENSG00000167771.5	-1.6071628	1.94517136	7.16E-07	4.21E-06	1.77	1.82	0.75	0.82	RCOR2	REST corepressor 2	chr11	protein_coding
ENSG00000144659.9	-1.1544002	5.73574637	6.69E-05	0.00027909	28.41	27.65	15.87	17.73	SLC25A38	solute carrier family 25, member 38	chr3	protein_coding
ENSG00000280186.1	4.11109881	-1.9329856	1.86E-05	8.57E-05	0	0.01	0	0.32	RP11-483I13	--	chr1	TEC
ENSG00000262693.1	6.02248524	-2.3782733	8.36E-05	0.00034052	0	0	0.26	0.78	RP11-46I8.3	--	chr17	antisense
ENSG00000140488.13	2.55454731	-2.3603613	0.00455824	0.01291303	0	0.02	0.07	0.06	CELF6	CUGBP, Elav-like family member 6	chr15	protein_coding
ENSG00000184492.6	3.39976777	-1.7901585	0.00010631	0.0004256	0.02	0	0.17	0.17	FOXD4L1	forkhead box D4-like 1	chr2	protein_coding
ENSG00000124882.3	6.39443202	2.42626151	1.15E-42	1.01E-40	0.01	0.04	1.24	3.64	EREG	epiregulin	chr4	protein_coding
ENSG00000236673.4	-5.9727733	-0.0063229	5.08E-21	1.32E-19	0.73	0.63	0	0.02	RP11-69I8.2	--	chr6	lincRNA
ENSG00000187553.7	6.27515402	-2.204389	8.95E-06	4.38E-05	0	0	0.27	0.14	CYP26C1	cytochrome P450, family 26, subfamily C, polypeptide 1	chr10	protein_coding
ENSG00000174010.9	2.49035959	0.89041353	1.42E-10	1.39E-09	0.08	0.06	0.38	0.64	KLHL15	kelch-like family member 15	chrX	protein_coding
ENSG00000175826.10	-1.3362353	6.97808165	3.92E-06	2.06E-05	74.86	66.58	38.44	36.18	CTDNEP1	CTD nuclear envelope phosphatase 1	chr17	protein_coding
ENSG00000068912.12	1.35052978	3.20677925	8.75E-06	4.30E-05	1.44	1.99	5.2	6.47	ERLEC1	endoplasmic reticulum lectin 1	chr2	protein_coding
ENSG00000234283.1	-5.6574516	-2.7834951	0.00086564	0.00288128	0.38	0.34	0	0	RP11-495P1	--	chr1	lincRNA
ENSG00000136205.15	-1.1180797	6.54692289	0.00010582	0.00042446	12.78	13.85	8.24	8.1	TNS3	tensin 3	chr7	protein_coding
ENSG00000203780.9	2.13490177	-0.3664103	4.43E-06	2.30E-05	0.24	0.04	0.83	0.87	FANK1	fibronectin type III and ankyrin repeat domains 1	chr10	protein_coding
ENSG00000083223.16	1.45734402	1.89367093	8.20E-06	4.05E-05	0.3	0.19	0.66	1.13	ZCCHC6	zinc finger, CCHC domain containing 6	chr9	protein_coding
ENSG00000119508.16	2.86036224	0.60200133	1.03E-11	1.15E-10	0.03	0.06	0.46	0.4	NR4A3	nuclear receptor subfamily 4, group A, member 3	chr9	protein_coding
ENSG00000084710.12	4.58898098	1.91802594	6.61E-29	2.89E-27	0.03	0.01	0.75	0.68	EFR3B	EFR3 homolog B (S. cerevisiae)	chr2	protein_coding
ENSG00000136490.7	1.68451262	1.95278246	2.50E-07	1.58E-06	0.58	0.2	1.78	1.57	LIMD2	LIM domain containing 2	chr17	protein_coding
ENSG00000101882.8	1.61029961	3.51017363	1.08E-07	7.22E-07	0.66	0.86	2.49	3.69	NKAP	NFKB activating protein	chrX	protein_coding
ENSG00000077097.12	3.02827766	4.01876428	2.69E-21	7.19E-20	0.45	0.59	3.99	7.36	TOP2B	topoisomerase (DNA) II beta 180kDa	chr3	protein_coding
ENSG00000070731.8	3.24590729	2.2810692	4.64E-20	1.12E-18	0.3	0.38	4.64	3.89	ST6GALNAC	ST6 (alpha-N-acetyl-neuraminyl-2,3-beta-galactosyl-1,3)-N-acetylgalactosaminide alpha-2,6-sialyltransferase 2	chr17	protein_coding
ENSG00000204947.7	3.16502494	1.59067859	2.38E-17	4.65E-16	0.11	0.18	1.89	1.61	ZNF425	zinc finger protein 425	chr7	protein_coding
ENSG00000176381.5	2.36704269	-2.4747985	0.01191236	0.03011315	0.02	0	0.07	0.06	PRR18	proline rich 18	chr6	protein_coding
ENSG00000273478.1	-7.6112023	-1.3192272	2.11E-11	2.27E-10	0.33	1.51	0	0	RP11-465N4	--	chr1	antisense
ENSG00000174307.6	-1.0236977	6.85085223	0.00037212	0.00133398	49.5	43.46	31.97	28.9	PHLDA3	pleckstrin homology-like domain, family A, member 3	chr1	protein_coding
ENSG00000278743.1	-2.1708136	-0.097527	5.28E-07	3.18E-06	1.87	2.33	0.56	0.67	RP11-707G1	--	chr12	lincRNA
ENSG00000165490.11	3.68468783	0.24762981	3.41E-14	4.98E-13	0.03	0.02	0.29	0.64	DDIAS	DNA damage-induced apoptosis suppressor	chr11	protein_coding
ENSG00000130382.8	-1.0429864	7.89075168	0.00028165	0.00103063	58.3	56.48	37	37.23	MLLT1	myeloid/lymphoid or mixed-lineage leukemia (trithorax homolog, Drosophila); translocated to, 1	chr19	protein_coding
ENSG00000168427.8	-6.5093465	1.71438707	7.09E-39	5.11E-37	1.87	0.94	0.01	0.03	KLHL30	kelch-like family member 30	chr2	protein_coding
ENSG00000130720.11	-3.295433	3.60271632	6.84E-24	2.17E-22	5.53	4.71	0.67	0.72	FIBCD1	fibrinogen C domain containing 1	chr9	protein_coding
ENSG00000146386.7	1.46325337	3.63698836	1.17E-06	6.65E-06	4.17	4.7	12.55	20.2	ABRACL	ABRA C-terminal like	chr6	protein_coding
ENSG00000122756.13	6.30985008	-2.1747134	8.95E-06	4.38E-05	0	0	0.09	0.21	CNTFR	ciliary neurotrophic factor receptor	chr9	protein_coding
ENSG00000213145.8	-8.0754898	6.76320013	1.42E-88	1.48E-85	132.14	106.56	0.7	0.48	CRIP1	cysteine-rich protein 1 (intestinal)	chr14	protein_coding
ENSG00000158406.3	1.48047768	-0.400681	0.00113746	0.00369419	0.74	0.71	3.07	2.4	HIST1H4H	histone cluster 1, H4h	chr6	protein_coding
ENSG00000157227.11	6.46898267	8.55479223	3.58E-71	1.51E-68	1.91	1.73	218.79	210.74	MMP14	matrix metallopeptidase 14 (membrane-inserted)	chr14	protein_coding
ENSG00000121310.15	-1.0671072	2.18421595	0.0006599	0.002256	3.52	2.69	2.14	1.82	ECHDC2	enoyl CoA hydratase domain containing 2	chr1	protein_coding
ENSG00000113838.11	1.14082439	2.19557141	0.00031479	0.00114162	0.77	0.68	1.75	2.5	TBCCD1	TBCC domain containing 1	chr3	protein_coding
ENSG00000182853.10	4.50186492	-1.6459463	2.66E-06	1.44E-05	0	0.04	0.76	0.51	VMO1	vitelline membrane outer layer 1 homolog (chicken)	chr17	protein_coding
ENSG00000280837.1	4.87917439	-3.0678699	0.01889873	0.04519557	0	0	0	0.09	CPS1-IT1	CPS1 intronic transcript 1	chr2	sense_intronic
ENSG00000147027.3	-7.3497585	-1.5424397	7.05E-10	6.26E-09	0.11	0.14	0	0	TMEM47	transmembrane protein 47	chrX	protein_coding
ENSG00000175938.6	1.09296319	1.693257	0.00085986	0.00288128	0.57	0.75	1.84	1.91	ORAI3	ORAI calcium release-activated calcium modulator 3	chr16	protein_coding
ENSG00000271746.1	1.32747106	-0.8558019	0.00671589	0.01821164	0.35	0.17	1.03	0.75	RP1-202O8.	--	chr1	antisense
ENSG00000131652.12	-1.3416854	5.84915714	3.91E-06	2.05E-05	50.4	43.77	24.9	24.62	THOC6	THO complex 6	chr16	protein_coding
ENSG00000235172.6	-3.5741879	-2.6382008	0.003237	0.00945156	0.22	0.12	0.03	0	LINC01366	long intergenic non-protein coding RNA 1366	chr5	lincRNA
ENSG00000273443.1	-4.5599072	-1.9720923	4.25E-06	2.21E-05	0.52	0.27	0.04	0	RP11-54O7.1	--	chr1	lincRNA
ENSG00000212588.1	-2.1970506	-2.0333975	0.00270895	0.00811738	2.41	1.86	0	1.12	SNORA26	small nucleolar RNA, H/ACA box 26	chr4	snoRNA
ENSG00000247011.2	-5.8932437	-2.6367837	0.00026535	0.0009747	0.1	0.07	0	0	RP11-700H6	--	chr17	lincRNA
ENSG00000162631.17	-7.0845666	-1.7620516	1.16E-08	8.76E-08	0.09	0.06	0	0	NTNG1	netrin G1	chr1	protein_coding
ENSG00000092439.12	3.35524441	2.08684759	1.56E-20	3.92E-19	0.06	0.06	0.5	1.07	TRPM7	transient receptor potential cation channel, subfamily M, member 7	chr15	protein_coding
ENSG00000170049.8	-1.3325199	-0.4252814	0.00291737	0.00859154	0.26	0.4	0.2	0.16	KCNAB3	potassium channel, voltage gated subfamily A regulatory beta subunit 3	chr17	protein_coding
ENSG00000172197.10	-3.2204655	0.76605592	2.02E-15	3.35E-14	1.61	1.14	0.14	0.24	MBOAT1	membrane bound O-acyltransferase domain containing 1	chr6	protein_coding
ENSG00000102904.13	3.43891476	2.28378662	9.43E-22	2.61E-20	0.23	0.23	3.25	3.49	TSNAXIP1	translin-associated factor X interacting protein 1	chr16	protein_coding
ENSG00000135336.13	1.28718833	0.71491334	0.0004619	0.00162595	0.24	0.33	0.5	1.36	ORC3	origin recognition complex, subunit 3	chr6	protein_coding
ENSG00000275799.1	5.86796777	-2.4845032	0.00026535	0.0009747	0	0	0.43	0.61	AP001059.7	--	chr21	lincRNA
ENSG00000115271.9	8.66799179	-0.2035596	2.54E-18	5.35E-17	0	0	0.57	0.83	GCA	grancalcin, EF-hand calcium binding protein	chr2	protein_coding
ENSG00000171988.16	5.27940241	1.97404407	6.12E-33	3.26E-31	0.02	0.01	0.5	1.28	JMJD1C	jumonji domain containing 1C	chr10	protein_coding
ENSG00000122694.14	-1.841967	2.55857826	4.69E-09	3.75E-08	3.9	2.89	1.08	1.45	GLIPR2	GLI pathogenesis-related 2	chr9	protein_coding
ENSG00000188707.5	1.86559106	5.47795175	2.87E-10	2.70E-09	5.42	5.15	23.91	27.51	ZBED6CL	ZBED6 C-terminal like	chr7	protein_coding
ENSG00000238280.1	3.1264535	-2.599436	0.00552196	0.01522223	0.04	0	0.33	0.24	RP11-436D1	--	chr10	antisense
ENSG00000154479.11	4.89980841	-3.0567113	0.01889873	0.04519557	0	0	0.06	0.05	CCDC173	coiled-coil domain containing 173	chr2	protein_coding
ENSG00000168438.13	1.69734455	1.52473698	6.56E-07	3.88E-06	0.26	0.23	0.68	1.45	CDC40	cell division cycle 40	chr6	protein_coding
ENSG00000006118.13	-2.723355	8.08114255	2.45E-19	5.59E-18	117.6	100.79	23.3	20.72	TMEM132A	transmembrane protein 132A	chr11	protein_coding
ENSG00000010310.7	-1.550474	0.22924365	7.05E-05	0.00029244	0.52	0.29	0.21	0.16	GIPR	gastric inhibitory polypeptide receptor	chr19	protein_coding
ENSG00000183246.6	7.19777854	-1.4908385	1.76E-09	1.50E-08	0	0	0.12	0.08	RIMBP3C	RIMS binding protein 3C	chr22	protein_coding
ENSG00000252549.1	4.91542612	-3.0483032	0.0100455	0.02582742	0	0	2.18	0.26	RNU6-759P	RNA, U6 small nuclear 759, pseudogene	chr20	snRNA
ENSG00000183010.15	-3.1390632	6.72473866	2.99E-24	9.81E-23	70.47	63.21	10.72	9.47	PYCR1	pyrroline-5-carboxylate reductase 1	chr17	protein_coding
ENSG00000239664.2	-2.4924232	-2.7157914	0.00943894	0.02469931	0.27	0.28	0	0.11	RP5-857K21.	--	chr1	lincRNA
ENSG00000109686.15	1.01771131	2.70084343	0.00089993	0.00298713	0.54	0.68	1.32	1.97	SH3D19	SH3 domain containing 19	chr4	protein_coding
ENSG00000163344.5	-1.3488633	6.51156752	3.28E-06	1.74E-05	87.82	77.8	41.87	44.89	PMVK	phosphomevalonate kinase	chr1	protein_coding
ENSG00000172732.10	-1.4353771	6.59220217	7.78E-07	4.56E-06	41.01	44.64	20.5	21.7	MUS81	MUS81 structure-specific endonuclease subunit	chr11	protein_coding
ENSG00000145491.10	7.82625294	-0.9549321	6.98E-12	7.96E-11	0	0	0.59	0.71	ROPN1L	rhophilin associated tail protein 1-like	chr5	protein_coding
ENSG00000117450.12	-1.8176886	9.77069028	5.28E-10	4.79E-09	910.63	848.94	339.05	325.8	PRDX1	peroxiredoxin 1	chr1	protein_coding
ENSG00000272953.1	-7.1384755	-1.7196791	7.18E-09	5.62E-08	0.38	0.99	0	0	RP11-1275H	--	chr7	lincRNA
ENSG00000141293.14	7.62484838	2.47567794	6.62E-47	7.75E-45	0.01	0.03	6.32	6.26	SKAP1	src kinase associated phosphoprotein 1	chr17	protein_coding
ENSG00000140263.12	-1.5425522	5.96909946	1.27E-07	8.41E-07	14.67	15.42	6.49	7.29	SORD	sorbitol dehydrogenase	chr15	protein_coding
ENSG00000117419.13	-1.3693537	6.28735121	2.40E-06	1.31E-05	48.55	42.4	26.05	20.75	ERI3	ERI1 exoribonuclease family member 3	chr1	protein_coding
ENSG00000259343.4	6.75188369	-1.8412616	2.24E-07	1.42E-06	0	0	0.21	0.42	TMC3-AS1	TMC3 antisense RNA 1	chr15	antisense
ENSG00000147536.10	-1.0925273	4.89262792	0.00017223	0.00065755	5.79	6.19	3.4	4.1	GINS4	GINS complex subunit 4 (Sld5 homolog)	chr8	protein_coding
ENSG00000236078.1	5.58023914	-2.6705827	0.00086564	0.00288128	0	0	0.07	0.07	LINC01447	long intergenic non-protein coding RNA 1447	chr7	lincRNA
ENSG00000125124.10	1.54700688	4.72412679	1.65E-07	1.07E-06	3.66	3.58	11.63	16.69	BBS2	Bardet-Biedl syndrome 2	chr16	protein_coding
ENSG00000114455.12	3.34695389	2.07812525	3.37E-20	8.21E-19	0.17	0.23	2.82	2.69	HHLA2	HERV-H LTR-associating 2	chr3	protein_coding
ENSG00000197119.11	2.82221233	4.35200552	2.14E-19	4.90E-18	0.77	0.7	6.92	6.9	SLC25A29	solute carrier family 25 (mitochondrial carnitine/acylcarnitine carrier), member 29	chr14	protein_coding
ENSG00000168124.2	-5.3760424	-2.9459444	0.00290522	0.00856312	0.09	0.18	0	0	OR1F1	olfactory receptor, family 1, subfamily F, member 1	chr16	protein_coding
ENSG00000188419.12	2.34703239	-0.1946267	5.41E-07	3.25E-06	0.01	0.04	0.17	0.19	CHM	choroideremia (Rab escort protein 1)	chrX	protein_coding
ENSG00000135108.13	1.76792189	4.23820434	3.80E-09	3.09E-08	1.12	1.16	4.68	5.67	FBXO21	F-box protein 21	chr12	protein_coding
ENSG00000144228.7	3.22841719	-0.0087969	3.54E-11	3.72E-10	0.04	0.01	0.19	0.39	SPOPL	speckle-type POZ protein-like	chr2	protein_coding
ENSG00000156804.6	1.72339877	3.35034233	1.78E-08	1.31E-07	0.56	0.57	2.38	2.58	FBXO32	F-box protein 32	chr8	protein_coding
ENSG00000141750.6	-3.5017355	4.29017536	3.86E-27	1.54E-25	8.84	8.48	1.14	0.89	STAC2	SH3 and cysteine rich domain 2	chr17	protein_coding
ENSG00000100664.9	1.33149163	5.00619112	5.30E-06	2.71E-05	1.96	2.42	5.66	9.11	EIF5	eukaryotic translation initiation factor 5	chr14	protein_coding

ENSG00000197275.11	1.69326667	0.18594749	3.00E-05	0.00013327	0.05	0.11	0.26	0.43	RAD54B	RAD54 homolog B (S. cerevisiae)	chr8	protein_coding
ENSG00000237436.1	1.69647976	-1.7832908	0.01883218	0.04519557	0.19	0.2	0.77	0.98	RP11-312B8.	--	chr1	antisense
ENSG00000118482.10	2.0437749	2.23665741	4.36E-10	4.01E-09	0.2	0.12	0.66	1.13	PHF3	PHD finger protein 3	chr6	protein_coding
ENSG00000280655.1	5.59464701	-2.6625875	0.00086564	0.00288128	0	0	0.83	0.31	RP11-361C1	--	chr15	IG_V_gene
ENSG00000224616.1	4.91542612	-3.0483032	0.0100455	0.02582742	0	0	0.53	0.06	RTCA-AS1	RTCA antisense RNA 1	chr1	antisense
ENSG00000060749.13	2.7314429	1.57393631	4.08E-14	5.93E-13	0.07	0.05	0.38	0.73	QSER1	glutamine and serine rich 1	chr11	protein_coding
ENSG00000171236.9	-3.7937177	-2.5038865	0.00112121	0.00364442	0.07	0.06	0.01	0	LRG1	leucine-rich alpha-2-glycoprotein 1	chr19	protein_coding
ENSG00000133027.16	-1.1551561	5.44464967	6.82E-05	0.00028404	39.53	36.92	22.84	22.91	PEMT	phosphatidylethanolamine N-methyltransferase	chr17	protein_coding
ENSG00000143473.10	-3.1977559	-0.1068132	5.06E-12	5.88E-11	0.11	0.22	0.01	0.03	KCNH1	potassium channel, voltage gated eag related subfamily H, member 1	chr1	protein_coding
ENSG00000035499.11	1.47492592	1.51962265	1.19E-05	5.69E-05	0.36	0.59	1.33	2.2	DEPDC1B	DEP domain containing 1B	chr5	protein_coding
ENSG00000159335.14	-1.0820278	11.2811789	0.00016294	0.00062493	1949.22	2517.76	1479.97	1323.28	PTMS	parathymosin	chr12	protein_coding
ENSG00000108242.11	-5.0206302	-3.1306986	0.0100455	0.02582742	0.06	0.02	0	0	CYP2C18	cytochrome P450, family 2, subfamily C, polypeptide 18	chr10	protein_coding
ENSG00000117906.12	2.85563523	4.969092	3.82E-20	9.30E-19	0.79	1.13	6.34	12.29	RCN2	reticulocalbin 2, EF-hand calcium binding domain	chr15	protein_coding
ENSG00000148848.13	-2.3242381	3.2595631	1.17E-13	1.61E-12	1.1	1.84	0.3	0.48	ADAM12	ADAM metallopeptidase domain 12	chr10	protein_coding
ENSG00000149273.13	-2.1136623	11.5805853	8.51E-13	1.07E-11	993.3	855.39	296.24	272.62	RPS3	ribosomal protein S3	chr11	protein_coding
ENSG00000165025.13	7.19777854	-1.4908385	1.76E-09	1.50E-08	0	0	0.14	0.09	SYK	spleen tyrosine kinase	chr9	protein_coding
ENSG00000124496.11	-2.1338367	3.38215532	5.53E-12	6.41E-11	1.85	1.7	0.56	0.51	TRERF1	transcriptional regulating factor 1	chr6	protein_coding
ENSG00000234409.6	5.94305985	-2.4328306	0.00014842	0.00057423	0	0	0.09	0.24	AC006547.14	--	chr22	protein_coding
ENSG00000279516.1	-6.1895265	-2.4395964	4.74E-05	0.00020301	0.11	0.12	0	0	FAM230C	family with sequence similarity 230, member C	chr13	processed_transcript
ENSG00000137273.3	-1.1118312	4.86778755	0.00013294	0.00052191	15.15	14.37	8.06	10.2	FOXF2	forkhead box F2	chr6	protein_coding
ENSG00000144369.11	6.60599572	-1.9545337	1.05E-06	6.02E-06	0	0	0.05	0.08	FAM171B	family with sequence similarity 171, member B	chr2	protein_coding
ENSG00000159640.13	1.68422166	-1.3274756	0.00272943	0.00817056	0.03	0.02	0.14	0.06	ACE	angiotensin I converting enzyme	chr17	protein_coding
ENSG00000078124.10	2.27602138	2.376787	4.05E-12	4.75E-11	0.16	0.22	0.94	1.5	ACER3	alkaline ceramidase 3	chr11	protein_coding
ENSG00000162367.10	5.98373061	-2.4097568	8.36E-05	0.00034052	0	0	0.08	0.02	TAL1	T-cell acute lymphocytic leukemia 1	chr1	protein_coding
ENSG00000270189.1	2.36109389	-0.9791669	5.34E-05	0.00022654	0.03	0.09	0.19	0.64	RP11-258C1	--	chrX	lincRNA
ENSG00000178531.5	-2.9917674	3.01408472	8.85E-20	2.09E-18	9.53	8.7	1.68	1.37	CTXN1	cortexin 1	chr19	protein_coding
ENSG00000101337.14	1.02731378	7.58003119	0.00034854	0.00125643	24.07	23.76	68.11	61.61	TM9SF4	transmembrane 9 superfamily protein member 4	chr20	protein_coding
ENSG00000125812.14	1.16669265	2.67259865	0.00015457	0.00059573	0.67	0.62	1.63	2.26	GZF1	GDNF-inducible zinc finger protein 1	chr20	protein_coding
ENSG00000172901.18	6.03134248	-2.373212	8.36E-05	0.00034052	0	0	0.04	0.07	AQPEP	--	chr5	protein_coding
ENSG00000181690.6	-2.1550219	-2.2739284	0.00854801	0.02266235	0.01	0.05	0.01	0.01	PLAG1	pleiomorphic adenoma gene 1	chr8	protein_coding
ENSG00000106772.16	-5.6609181	-2.7818379	0.00086564	0.00288128	0	0.02	0	0	PRUNE2	prune homolog 2 (Drosophila)	chr9	protein_coding
ENSG00000144959.8	2.12079085	1.8783336	3.69E-10	3.43E-09	0.15	0.31	0.83	1.84	NCEH1	neutral cholesterol ester hydrolase 1	chr3	protein_coding
ENSG00000130513.6	-2.6395186	5.09269584	6.96E-18	1.42E-16	34.01	45.46	9.02	7.92	GDF15	growth differentiation factor 15	chr19	protein_coding
ENSG00000188004.8	-1.2110083	0.00655561	0.00313294	0.00917679	0.4	0.48	0.31	0.2	C1orf204	chromosome 1 open reading frame 204	chr1	protein_coding
ENSG00000197332.8	2.42241419	-1.7514667	0.00140662	0.00449532	0.03	0	0.11	0.13	ZNF833P	zinc finger protein 833, pseudogene	chr19	lincRNA
ENSG00000092036.15	-1.2404915	6.53309104	1.79E-05	8.29E-05	61.66	54.02	32.65	32.62	HAUS4	HAUS augmin-like complex, subunit 4	chr14	protein_coding
ENSG00000166352.14	1.51221534	1.97979685	3.35E-06	1.78E-05	1	1.69	3.69	6.55	C11orf74	chromosome 11 open reading frame 74	chr11	protein_coding
ENSG00000178966.14	1.42611435	-1.1477781	0.01004567	0.02582742	0.07	0.02	0.14	0.21	RMI1	RecQ mediated genome instability 1	chr9	protein_coding
ENSG00000196422.9	-1.8138669	8.15558592	5.93E-10	5.33E-09	71.26	64.97	26.16	25.47	PPP1R26	protein phosphatase 1, regulatory subunit 26	chr9	protein_coding
ENSG00000137135.16	-1.4573324	3.59687465	1.14E-06	6.50E-06	3.62	3.99	1.71	1.99	ARHGEF39	Rho guanine nucleotide exchange factor (GEF) 39	chr9	protein_coding
ENSG00000235319.1	6.51968671	-2.0237618	1.78E-06	9.85E-06	0	0	0.67	0.44	AC012360.4	--	chr2	lincRNA
ENSG00000272639.1	1.39507661	-0.9734698	0.00723724	0.01946932	0.14	0.55	1.48	0.97	RP11-66B24.	--	chr15	processed_transcript
ENSG00000133103.15	3.7529136	-0.4514863	4.22E-11	4.40E-10	0.02	0	0.13	0.23	COG6	component of oligomeric golgi complex 6	chr13	protein_coding
ENSG00000248367.1	-1.7191949	1.87520199	1.31E-07	8.65E-07	10.89	12.19	3.91	5.4	CTB-129O4.	--	chr5	antisense
ENSG00000141837.17	-2.3500979	-0.3346559	4.27E-07	2.62E-06	0.13	0.09	0.03	0.02	CACNA1A	calcium channel, voltage-dependent, P/Q type, alpha 1A subunit	chr19	protein_coding
ENSG00000279133.1	8.20679733	-0.6278485	1.56E-14	2.37E-13	0	0	0.41	0.22	RP11-342K2.	--	chr17	TEC
ENSG00000230091.5	2.13594596	-0.451064	7.65E-06	3.81E-05	0.05	0.07	0.42	0.31	TMEM254-A	TMEM254 antisense RNA 1	chr10	antisense
ENSG00000161638.9	-3.8254841	9.39241803	2.22E-33	1.21E-31	243.84	213.35	22.55	20.39	ITGA5	integrin, alpha 5 (fibronectin receptor, alpha polypeptide)	chr12	protein_coding
ENSG00000131979.17	3.51782497	1.52709028	1.27E-19	2.97E-18	0.15	0.09	1.67	2.06	GCH1	GTP cyclohydrolase 1	chr14	protein_coding
ENSG00000128422.14	1.84627218	10.634174	2.91E-10	2.74E-09	354.38	380.72	1796.18	1721.94	KRT17	keratin 17, type I	chr17	protein_coding
ENSG00000128284.18	2.93082005	3.56102042	9.63E-20	2.26E-18	0.51	0.62	5.95	5.58	APOL3	apolipoprotein L, 3	chr22	protein_coding
ENSG00000167637.15	2.25907445	0.52011399	1.44E-08	1.08E-07	0.03	0.08	0.33	0.35	ZNF283	zinc finger protein 283	chr19	protein_coding
ENSG00000205476.7	-1.1130399	5.97436349	0.00011711	0.00046441	4.34	3.86	2.37	2.69	CCDC85C	coiled-coil domain containing 85C	chr14	protein_coding
ENSG00000023287.11	3.98777477	0.35966075	6.38E-16	1.10E-14	0.02	0.01	0.17	0.55	RB1CC1	RB1-inducible coiled-coil 1	chr8	protein_coding
ENSG00000145029.10	-1.3727598	2.93541935	7.19E-06	3.59E-05	3.34	3.47	1.82	1.68	NICN1	nicolin 1	chr3	protein_coding
ENSG00000266074.7	1.3242774	4.48670985	6.87E-06	3.45E-05	1.05	0.86	3.04	3.34	BAHCC1	BAH domain and coiled-coil containing 1	chr17	protein_coding
ENSG00000250509.1	-6.2307336	-0.5500973	2.41E-17	4.69E-16	1.66	0.95	0.04	0	CTC-573N18	--	chr5	lincRNA
ENSG00000184368.14	-2.1917514	4.68171485	4.10E-13	5.35E-12	6.35	10.02	2.36	2.41	MAP7D2	MAP7 domain containing 2	chrX	protein_coding
ENSG00000273253.2	3.62493312	-1.1731145	1.97E-07	1.27E-06	0	0.08	0.83	0.63	RP3-402G11	--	chr22	antisense
ENSG00000115137.10	1.86040295	-0.562491	0.00012395	0.0004892	0.05	0.03	0.08	0.32	DNAJC27	DnaJ (Hsp40) homolog, subfamily C, member 27	chr2	protein_coding
ENSG00000115520.7	1.28516657	1.93075809	7.39E-05	0.00030568	0.6	0.81	1.48	3.11	COQ10B	coenzyme Q10 homolog B (S. cerevisiae)	chr2	protein_coding
ENSG00000168813.15	2.06550938	1.46047766	3.34E-09	2.74E-08	0.1	0.12	0.6	0.59	ZNF507	zinc finger protein 507	chr19	protein_coding
ENSG00000166068.11	1.59651484	1.50197814	2.39E-06	1.30E-05	0.15	0.13	0.44	0.7	SPRED1	sprouty-related, EVH1 domain containing 1	chr15	protein_coding
ENSG00000111679.15	2.42500846	-0.3293772	3.31E-07	2.06E-06	0.1	0.03	0.6	0.42	PTPN6	protein tyrosine phosphatase, non-receptor type 6	chr12	protein_coding
ENSG00000068796.15	1.47042203	2.10355624	4.68E-06	2.42E-05	0.5	0.49	1.29	2.4	KIF2A	kinesin heavy chain member 2A	chr5	protein_coding
ENSG00000083807.8	-8.8851489	3.78430107	9.65E-70	3.79E-67	10.02	8.38	0.03	0.02	SLC27A5	solute carrier family 27 (fatty acid transporter), member 5	chr19	protein_coding
ENSG00000260273.1	2.06033987	-1.9870458	0.00956893	0.02497848	0	0.13	0.22	0.5	RP11-425D1	--	chr6	antisense
ENSG00000275538.1	6.06703713	-2.3528694	4.74E-05	0.00020301	0	0	2.69	0.65	RNVU1-19	RNA, variant U1 small nuclear 19	chr1	snRNA
ENSG00000114126.16	1.77704391	4.21691196	3.26E-09	2.67E-08	0.64	0.7	2.79	3.34	TFDP2	transcription factor Dp-2 (E2F dimerization partner 2)	chr3	protein_coding
ENSG00000207296.1	6.11950765	-2.3132648	4.74E-05	0.00020301	0	0	2.67	2.52	RNU6-140P	RNA, U6 small nuclear 140, pseudogene	chr19	snRNA
ENSG00000171681.11	1.66240828	2.65095058	1.24E-07	8.21E-07	0.18	0.26	0.78	1.09	ATF7IP	activating transcription factor 7 interacting protein	chr12	protein_coding
ENSG00000187678.8	-1.565827	2.55381656	4.96E-07	3.00E-06	1.63	1.21	0.52	0.76	SPRY4	sprouty homolog 4 (Drosophila)	chr5	protein_coding
ENSG00000131724.9	1.24102423	4.8671575	2.17E-05	9.86E-05	3.34	3.22	9.11	11.62	IL13RA1	interleukin 13 receptor, alpha 1	chrX	protein_coding
ENSG00000113721.12	-5.1946597	3.4745849	5.46E-44	5.28E-42	3.22	2.91	0.07	0.15	PDGFRB	platelet-derived growth factor receptor, beta polypeptide	chr5	protein_coding
ENSG00000184110.13	-1.4693544	7.27647099	4.17E-07	2.56E-06	50.75	53.05	24.6	25.35	EIF3C	eukaryotic translation initiation factor 3, subunit C	chr16	protein_coding
ENSG00000176842.13	-1.4465514	3.14447425	1.86E-06	1.03E-05	3.06	4.3	1.79	1.8	IRX5	iroquois homeobox 5	chr16	protein_coding
ENSG00000179873.13	4.64785914	-0.7940167	5.75E-11	5.92E-10	0.02	0	0.31	0.29	NLRP11	NLR family, pyrin domain containing 11	chr19	protein_coding
ENSG00000002587.8	-4.2536355	-1.0633472	1.47E-10	1.44E-09	0.09	0.08	0.01	0	HS3ST1	heparan sulfate (glucosamine) 3-O-sulfotransferase 1	chr4	protein_coding
ENSG00000137812.18	3.31427537	1.72024564	7.23E-19	1.60E-17	0.06	0.03	0.36	0.94	CASC5	cancer susceptibility candidate 5	chr15	protein_coding
ENSG00000114209.13	1.40466999	2.48410144	7.78E-06	3.87E-05	0.94	1.1	2.08	5.19	PDCD10	programmed cell death 10	chr3	protein_coding
ENSG00000275454.1	4.56709794	-0.8576274	2.34E-10	2.23E-09	0	0.05	0.68	0.9	CTD-2026K1	--	chr15	antisense
ENSG00000100578.13	1.31504425	1.13590051	0.00015453	0.00059567	0.09	0.13	0.35	0.39	KIAA0586	KIAA0586	chr14	protein_coding
ENSG00000107560.9	2.19143679	0.12178172	2.35E-07	1.49E-06	0.06	0.04	0.3	0.28	RAB11FIP2	RAB11 family interacting protein 2 (class I)	chr10	protein_coding
ENSG00000129250.10	-1.3433329	9.92853709	3.29E-06	1.74E-05	141.39	144.51	76.78	73.22	KIF1C	kinesin family member 1C	chr17	protein_coding
ENSG00000152256.12	1.49870788	2.67732839	1.52E-06	8.50E-06	0.21	0.16	0.52	0.89	PDK1	pyruvate dehydrogenase kinase, isozyme 1	chr2	protein_coding
ENSG00000175970.8	1.08832145	5.4870394	0.00017239	0.00065805	5.63	4.89	14.31	15.55	UNC119B	unc-119 homolog B (C. elegans)	chr12	protein_coding
ENSG00000134438.9	4.88946445	-3.0622981	0.01889873	0.04519557	0	0	0.02	0.05	RAX	retina and anterior neural fold homeobox	chr18	protein_coding
ENSG00000241553.11	-1.2111296	7.5220342	2.67E-05	0.00012003	83.37	70.32	44.9	43.59	ARPC4	actin related protein 2/3 complex, subunit 4, 20kDa	chr3	protein_coding
ENSG00000110717.9	-1.3906655	7.77169065	1.56E-06	8.72E-06	356.65	290.31	180.01	148.21	NDUFS8	NADH dehydrogenase (ubiquinone) Fe-S protein 8, 23kDa (NADH-coenzyme Q reductase)	chr11	protein_coding
ENSG00000167037.17	2.90794631	-0.4184917	1.85E-08	1.37E-07	0.03	0	0.21	0.16	SGSM1	small G protein signaling modulator 1	chr22	protein_coding
ENSG00000173482.15	-4.9357737	5.04475783	6.04E-46	6.61E-44	7.9	8.15	0.29	0.4	PTPRM	protein tyrosine phosphatase, receptor type, M	chr18	protein_coding
ENSG00000204610.11	-7.427058	0.58858177	4.71E-29	2.08E-27	1.29	0.86	0	0.01	TRIM15	tripartite motif containing 15	chr6	protein_coding
ENSG00000165650.10	2.24265475	1.89896054	4.15E-11	4.33E-10	0.09	0.12	0.44	0.9	PDZD8	PDZ domain containing 8	chr10	protein_coding
ENSG00000019169.10	-5.3256872	2.81752978	4.80E-41	3.88E-39	6.07	6.07	0.19	0.21	MARCO	macrophage receptor with collagenous structure	chr2	protein_coding
ENSG00000170477.11	5.79244689	-2.5359189	0.00026535	0.0009747	0	0	0.11	0.06	KRT4	keratin 4, type II	chr12	protein_coding
ENSG00000128585.16	1.42040279	2.70789826	4.76E-06	2.46E-05	0.25	0.25	0.79	0.99	MKLN1	muskelin 1, intracellular mediator containing kelch motifs	chr7	protein_coding
ENSG00000124575.6	3.30855315	-0.6073972	2.35E-09	1.96E-08	0.24	0	2.55	0.86	HIST1H1D	histone cluster 1, H1d	chr6	protein_coding
ENSG00000128915.10	1.59924724	1.3169216	3.87E-06	2.03E-05	0.12	0.08	0.27	0.53	ICE2	interactor of little elongation complex ELL subunit 2	chr15	protein_coding
ENSG00000078687.15	3.76256714	1.5795759	1.99E-21	5.35E-20	0.03	0.03	0.54	0.62	TNRC6C	trinucleotide repeat containing 6C	chr17	protein_coding
ENSG00000134852.13	2.40093846	1.02374417	1.44E-10	1.41E-09	0.04	0.05	0.3	0.32	CLOCK	clock circadian regulator	chr4	protein_coding
ENSG00000228661.1	3.00985124	-2.6665231	0.00943894	0.02469931	0	0.06	0.39	0.33	AC090587.5	--	chr11	antisense
ENSG00000128805.13	-1.8557197	3.19399756	1.64E-09	1.40E-08	2.9	2.65	1.05	0.99	ARHGAP22	Rho GTPase activating protein 22	chr10	protein_coding
ENSG00000145555.13	-2.4907944	6.79886959	1.20E-16	2.20E-15	10.41	12.96	2.85	2.68	MYO10	myosin X	chr5	protein_coding
ENSG00000114423.17	-1.995277	2.72043635	2.61E-10	2.47E-09	1.04	1.17	0.4	0.34	CBLB	Cbl proto-oncogene B, E3 ubiquitin protein ligase	chr3	protein_coding
ENSG00000249378.4	-5.5223491	-2.8631056	0.00158008	0.00495521	0.07	0.05	0	0	LINC01060	long intergenic non-protein coding RNA 1060	chr4	lincRNA
ENSG00000130511.14	-1.8123893	5.14831921	8.91E-10	7.85E-09	21.2	19.4	7.92	7.48	SSBP4	single stranded DNA binding protein 4	chr19	protein_coding
ENSG00000100345.19	-1.1113747	11.6643899	0.0001086	0.00043368	440.47	498.33	302.38	275.43	MYH9	myosin, heavy chain 9, non-muscle	chr22	protein_coding
ENSG00000198182.11	-2.5252177	-0.3127108	1.48E-08	1.10E-07	0.23	0.24	0.01	0.09	ZNF607	zinc finger protein 607	chr19	protein_coding
ENSG00000183484.10	7.87639461	-0.9080117	3.28E-12	3.89E-11	0	0	0.18	0.33	GPR132	G protein-coupled receptor 132	chr14	protein_coding
ENSG00000139974.14	1.03091098	1.40829077	0.00219879	0.00669241	0.48	0.89	1.88	1.83	SLC38A6	solute carrier family 38, member 6	chr14	protein_coding
ENSG00000131788.14	-1.4026348	4.80130754	1.73E-06	9.61E-06	11.59	10.97	5.95	5.41	PIAS3	protein inhibitor of activated STAT, 3	chr1	protein_coding
ENSG00000074181.7	-9.5965049	8.82471544	2.91E-114	9.97E-111	81.71	87.59	0.21	0.08	NOTCH3	notch 3	chr19	protein_coding
ENSG00000166734.17	2.1194346	2.71040334	3.39E-11	3.57E-10	0.37	0.62	2.16	3.57	CASC4	cancer susceptibility candidate 4	chr15	protein_coding
ENSG00000170848.14	4.89980841	-3.0567113	0.01889873	0.04519557	0	0	0.03	0.03	PSG6	pregnancy specific beta-1-glycoprotein 6	chr19	protein_coding
ENSG00000172845.12	3.31533377	1.75896606	4.83E-19	1.09E-17	0.1	0.04	0.69	1.23	SP3	Sp3 transcription factor	chr2	protein_coding
ENSG00000131236.15	-1.0045695	7.022651	0.00047302	0.00166388	42.28	47.65	28.79	30.94	CAP1	CAP, adenylate cyclase-associated protein 1 (yeast)	chr1	protein_coding
ENSG00000277728.1	4.72703895	-3.141721	0.01889873	0.04519557	0	0	0.41	0.06	RP11-143K1	--	chr17	antisense
ENSG00000244491.1	2.65829591	-1.5832403	0.00020516	0.00077306	0.12	0.12	1.04	1.06	RP3-508I15.	--	chr22	antisense
ENSG00000106261.15	1.08320762	3.71036996	0.00026958	0.00098917	0.58	0.83	1.87	2.12	ZKSCAN1	zinc finger with KRAB and SCAN domains 1	chr7	protein_coding
ENSG00000188626.6	5.04175262	-2.9826774	0.0100455	0.02582742	0	0	0	0.13	GOLGA8M	golgin A8 family, member M	chr15	protein_coding
ENSG00000270800.1	-1.9941569	9.79979511	1.25E-11	1.39E-10	1428.32	1203.43	450.21	430.08	RPS10-NUD	RPS10-NUDT3 readthrough	chr6	protein_coding
ENSG00000255874.2	-1.6372259	3.85216712	4.88E-08	3.41E-07	2.75	2.9	1.07	1.35	LINC00346	long intergenic non-protein coding RNA 346	chr13	lincRNA
ENSG00000261634.3	-3.9017869	-1.8090166	1.01E-06	5.80E-06	0.11	0.05	0	0.01	RP11-352D1	--	chr15	antisense
ENSG00000198598.5	-2.9695766	3.29543125	6.18E-20	1.48E-18	6.03	4.95	1.12	0.74	MMP17	matrix metallopeptidase 17 (membrane-inserted)	chr12	protein_coding
ENSG00000196449.3	-1.5206694	4.64656006	2.59E-07	1.63E-06	17.24	15.66	7.33	7.96	YRDC	yrdC N(6)-threonylcarbamoyltransferase domain containing	chr1	protein_coding
ENSG00000188523.7	6.72803071	-1.8641315	2.24E-07	1.42E-06	0	0	0.22	0.18	C9orf171	chromosome 9 open reading frame 171	chr9	protein_coding
ENSG00000073350.12	2.6585918	4.95337838	5.81E-18	1.19E-16	1.88	1.64	15.54	14.08	LLGL2	lethal giant larvae homolog 2 (Drosophila)	chr17	protein_coding
ENSG00000270069.1	2.43944279	-2.4309103	0.01191236	0.03011315	0.03	0	0.04	0.2	MIR222HG	MIR222 host gene	chrX	lincRNA
ENSG00000163528.11	-1.0154267	5.31486634	0.0004524	0.00159437	25.29	28.5	17.82	17.6	CHCHD4	coiled-coil-helix-coiled-coil-helix domain containing 4	chr3	protein_coding
ENSG00000113048.15	-1.1152709	6.54896964	0.00010977	0.00043762	30.49	27.97	16.02	20.06	MRPS27	mitochondrial ribosomal protein S27	chr5	protein_coding
ENSG00000138771.13	-1.9858683	4.5372008	3.76E-11	3.94E-10	2.55	2.9	0.94	0.89	SHROOM3	shroom family member 3	chr4	protein_coding
ENSG00000106868.15	-2.41995	2.47421191	8.42E-14	1.18E-12	2.22	2.53	0.41	0.77	SUSD1	sushi domain containing 1	chr9	protein_coding
ENSG00000253917.4	-1.9439546	-2.1781593	0.01384632	0.03449994	0.51	0.12	0.05	0.16	AC226119.5	--	chr4	processed_transcript
ENSG00000168079.15	-6.1820215	0.19426034	3.94E-23	1.19E-21	0.48	0.34	0	0.01	SCARA5	scavenger receptor class A, member 5	chr8	protein_coding
ENSG00000164764.10	3.67022649	-0.5176892	1.39E-10	1.37E-09	0.01	0.03	0.24	0.39	SBSPON	somatomedin B and thrombospondin, type 1 domain containing	chr8	protein_coding
ENSG00000185420.17	-1.0036873	5.20853018	0.00052845	0.00183942	20.27	16.21	11.06	13.25	SMYD3	SET and MYND domain containing 3	chr1	protein_coding
ENSG00000166548.14	1.21261105	5.38082162	3.03E-05	0.00013473	3.54	3.2	9.9	10.96	TK2	thymidine kinase 2, mitochondrial	chr16	protein_coding
ENSG00000162378.11	2.86608008	1.15677001	6.49E-14	9.24E-13	0.07	0.03	0.32	0.66	ZYG11B	zyg-11 family member B, cell cycle regulator	chr1	protein_coding
ENSG00000132879.12	1.39637908	4.57874655	2.16E-06	1.18E-05	3.22	2.79	11.03	10.05	FBXO44	F-box protein 44	chr1	protein_coding
ENSG00000274182.1	6.33087282	-2.1624822	8.95E-06	4.38E-05	0	0	5.78	4.91	AC012181.2	--	chr16	miRNA
ENSG00000152749.7	1.40656315	-0.3251976	0.00137911	0.00442096	0.02	0.05	0.09	0.16	GPR180	G protein-coupled receptor 180	chr13	protein_coding
ENSG00000104689.8	-1.5407525	4.45922436	1.96E-07	1.26E-06	8.97	10.57	4.05	4.91	TNFRSF10A	tumor necrosis factor receptor superfamily, member 10a	chr8	protein_coding
ENSG00000170759.10	2.81496851	4.01442037	4.94E-19	1.11E-17	0.46	0.64	3.53	6.86	KIF5B	kinesin family member 5B	chr10	protein_coding
ENSG00000272092.1	1.44468455	1.35913573	2.16E-05	9.84E-05	0.76	1.21	3.97	3.15	RP11-350N1	--	chr8	lincRNA
ENSG00000081014.9	2.13910061	2.0745328	1.40E-10	1.37E-09	0.15	0.22	0.84	1.3	AP4E1	adaptor-related protein complex 4, epsilon 1 subunit	chr15	protein_coding
ENSG00000245213.5	-3.9121579	-1.8034392	6.30E-07	3.74E-06	0.03	0.19	0	0.02	RP11-10K16.	--	chr4	antisense
ENSG00000076864.18	1.61967367	4.99179366	3.90E-08	2.74E-07	3.49	2.83	12.91	13.01	RAP1GAP	RAP1 GTPase activating protein	chr1	protein_coding
ENSG00000198825.10	3.61314413	3.1792134	5.14E-26	1.89E-24	0.15	0.22	2.41	3.54	INPP5F	inositol polyphosphate-5-phosphatase F	chr10	protein_coding
ENSG00000201302.1	-1.4045809	-1.4975909	0.02009733	0.04771922	2.26	3.27	1.97	0.84	SNORA65	small nucleolar RNA, H/ACA box 65	chr9	snoRNA
ENSG00000131966.12	1.25890227	4.26057563	2.01E-05	9.22E-05	3.34	4.19	9.44	14.68	ACTR10	actin-related protein 10 homolog (S. cerevisiae)	chr14	protein_coding
ENSG00000198816.5	-1.0713627	5.66307279	0.00021215	0.0007985	28.82	27.95	19.53	16.39	ZNF358	zinc finger protein 358	chr19	protein_coding
ENSG00000269921.1	2.04909581	-2.000139	0.00956893	0.02497848	0.1	0.11	1.09	0.15	RP11-646I6.	--	chr4	lincRNA
ENSG00000180773.13	1.38229652	1.95418789	1.87E-05	8.64E-05	0.23	0.31	0.62	1.27	SLC36A4	solute carrier family 36 (proton/amino acid symporter), member 4	chr11	protein_coding
ENSG00000144036.13	1.71019745	3.17419637	2.67E-08	1.93E-07	0.38	0.32	1.35	1.73	EXOC6B	exocyst complex component 6B	chr2	protein_coding
ENSG00000008405.10	1.60209305	2.8812035	2.30E-07	1.46E-06	0.7	1.08	2.44	4.83	CRY1	cryptochrome circadian clock 1	chr12	protein_coding
ENSG00000167680.14	-3.6482778	1.51516512	2.02E-21	5.42E-20	1.19	0.95	0.09	0.14	SEMA6B	sema domain, transmembrane domain (TM), and cytoplasmic domain, (semaphorin) 6B	chr19	protein_coding
ENSG00000130600.14	-2.5584316	5.74638835	2.83E-17	5.46E-16	32.27	30.66	7.75	6.46	H19	H19, imprinted maternally expressed transcript (non-protein coding)	chr11	processed_transcript
ENSG00000151233.9	2.57372984	1.38915865	2.41E-12	2.91E-11	0.02	0.14	0.43	0.81	GXYLT1	glucoside xylosyltransferase 1	chr12	protein_coding
ENSG00000269296.1	5.35410067	-2.8076728	0.00290522	0.00856312	0	0	0.06	0.03	AC005614.3	--	chr19	antisense
ENSG00000135945.8	2.36819779	1.8955941	4.69E-12	5.47E-11	0.25	0.14	1.02	1.74	REV1	REV1, polymerase (DNA directed)	chr2	protein_coding
ENSG00000165533.17	1.35522311	2.53288062	1.48E-05	7.00E-05	0.45	0.49	1.12	2.09	TTC8	tetratricopeptide repeat domain 8	chr14	protein_coding
ENSG00000077063.9	-3.0961609	-1.6599261	8.20E-06	4.05E-05	0.08	0.06	0.01	0.01	CTTNBP2	cortactin binding protein 2	chr7	protein_coding
ENSG00000163909.7	-4.7914951	-1.7967018	3.95E-07	2.43E-06	0.01	0.2	0.01	0	HEYL	hes-related family bHLH transcription factor with YRPW motif-like	chr1	protein_coding
ENSG00000111087.8	-3.6283191	-0.224138	5.02E-13	6.50E-12	0.36	0.34	0.02	0.05	GLI1	GLI family zinc finger 1	chr12	protein_coding
ENSG00000169908.9	-5.9305135	5.95440578	1.60E-60	3.99E-58	24.4	29.54	0.46	0.71	TM4SF1	transmembrane 4 L six family member 1	chr3	protein_coding
ENSG00000130477.13	-8.9813252	7.896164	1.16E-103	2.52E-100	34.17	42.28	0.14	0.06	UNC13A	unc-13 homolog A (C. elegans)	chr19	protein_coding
ENSG00000141424.11	1.48083148	4.28385911	6.09E-07	3.64E-06	1.76	2.75	5.59	11.27	SLC39A6	solute carrier family 39 (zinc transporter), member 6	chr18	protein_coding
ENSG00000261829.1	5.06203518	-2.9716376	0.0100455	0.02582742	0	0	0.21	0.23	RP11-223I10	--	chr2	sense_overlapping
ENSG00000100364.17	-1.1573956	7.76646724	5.83E-05	0.00024561	28.47	27.33	17.07	16.24	KIAA0930	KIAA0930	chr22	protein_coding
ENSG00000052126.13	1.04099622	1.32515518	0.0022107	0.0067261	0.12	0.15	0.3	0.44	PLEKHA5	pleckstrin homology domain containing, family A member 5	chr12	protein_coding
ENSG00000109472.12	1.65456947	5.54934365	1.79E-08	1.32E-07	6.81	8.28	26.21	37.28	CPE	carboxypeptidase E	chr4	protein_coding
ENSG00000091136.12	-1.1419127	7.20499597	7.36E-05	0.00030438	24.27	28.64	12.88	19.21	LAMB1	laminin, beta 1	chr7	protein_coding
ENSG00000138764.12	2.70287989	2.7095497	3.31E-16	5.85E-15	0.15	0.29	1.36	2.4	CCNG2	cyclin G2	chr4	protein_coding
ENSG00000136379.10	1.43729928	3.75480807	1.60E-06	8.91E-06	2.65	2.13	8.12	9.17	ABHD17C	abhydrolase domain containing 17C	chr15	protein_coding
ENSG00000099769.5	4.92065931	-3.045493	0.0100455	0.02582742	0	0	0.11	0	IGFALS	insulin-like growth factor binding protein, acid labile subunit	chr16	protein_coding
ENSG00000164061.4	2.60438271	1.58194005	4.21E-13	5.49E-12	0.05	0.03	0.38	0.3	BSN	bassoon presynaptic cytomatrix protein	chr3	protein_coding
ENSG00000120262.9	2.67276369	-1.8818633	0.00119227	0.00385709	0.01	0.01	0.02	0.12	CCDC170	coiled-coil domain containing 170	chr6	protein_coding
ENSG00000171017.9	1.15457045	4.23448637	8.73E-05	0.00035405	2.46	2.57	7.7	7.19	LRRC8E	leucine rich repeat containing 8 family, member E	chr19	protein_coding
ENSG00000217236.1	4.05690315	-0.4683709	9.53E-12	1.07E-10	0.04	0	0.35	0.65	SP9	Sp9 transcription factor	chr2	protein_coding
ENSG00000012124.13	2.79237689	0.61736104	2.07E-11	2.24E-10	0.02	0.16	0.7	0.94	CD22	CD22 molecule	chr19	protein_coding
ENSG00000273002.1	-1.2140273	0.39193255	0.00105131	0.00343874	0.77	0.56	0.28	0.48	RP11-336K2	--	chr1	antisense

ENSG00000130054.4	9.7269328	0.77797979	4.91E-29	2.16E-27	0	0	0.8	0.92	FAM155B	family with sequence similarity 155, member B	chrX	protein_coding
ENSG00000151657.10	1.8894139	1.77570635	2.08E-08	1.52E-07	0.19	0.17	0.64	1.15	KIN	Kin17 DNA and RNA binding protein	chr10	protein_coding
ENSG00000127328.20	1.59894799	0.68057676	1.89E-05	8.70E-05	0.04	0.07	0.18	0.24	RAB3IP	RAB3A interacting protein	chr12	protein_coding
ENSG00000278500.1	8.06318466	-0.7488582	1.77E-13	2.40E-12	0	0	0.3	0.32	AC009336.19	--	chr2	protein_coding
ENSG00000186862.16	-1.3967169	1.66085958	1.95E-05	8.95E-05	0.92	0.63	0.37	0.42	PDZD7	PDZ domain containing 7	chr10	protein_coding
ENSG00000101871.13	-1.1761865	1.28871832	0.00039609	0.00141171	0.22	0.38	0.1	0.26	MID1	midline 1	chrX	protein_coding
ENSG00000170423.11	-3.9717868	-0.9722822	3.35E-10	3.12E-09	0.18	0.31	0	0.04	KRT78	keratin 78, type II	chr12	protein_coding
ENSG00000160179.17	-1.2462045	1.58834002	0.00015374	0.00059298	0.69	0.8	0.39	0.45	ABCG1	ATP-binding cassette, sub-family G (WHITE), member 1	chr21	protein_coding
ENSG00000177311.9	3.26754109	2.56594676	4.26E-21	1.12E-19	0.11	0.11	1.19	1.75	ZBTB38	zinc finger and BTB domain containing 38	chr3	protein_coding
ENSG00000115392.10	1.33747476	2.48998123	1.82E-05	8.42E-05	1.4	1.58	3.84	6.24	FANCL	Fanconi anemia, complementation group L	chr2	protein_coding
ENSG00000278389.1	-4.2531405	0.4998444	1.78E-19	4.11E-18	4.87	4.72	0.36	0.3	RP11-626G1	--	chr16	lincRNA
ENSG00000214113.9	-1.1714155	4.59138673	6.14E-05	0.00025794	3.22	3.1	1.87	1.87	LYRM4	LYR motif containing 4	chr6	protein_coding
ENSG00000186918.12	-1.6514567	5.55414722	1.85E-08	1.37E-07	11.58	10.61	5.18	4.21	ZNF395	zinc finger protein 395	chr8	protein_coding
ENSG00000168672.3	6.28759928	0.68258876	2.42E-25	8.56E-24	0.01	0	0.46	0.72	FAM84B	family with sequence similarity 84, member B	chr8	protein_coding
ENSG00000269609.4	1.40702926	3.93627625	2.32E-06	1.27E-05	4.26	4.41	15.98	14.63	RPARP-AS1	RPARP antisense RNA 1	chr10	processed_transcript
ENSG00000156042.16	5.05186882	-2.9771646	0.0100455	0.02582742	0	0	0.02	0.05	CFAP70	cilia and flagella associated protein 70	chr10	protein_coding
ENSG00000263235.1	-1.6718798	1.6785246	3.94E-07	2.43E-06	3.13	4.22	1.38	1.68	RP11-461A8	--	chr16	sense_overlapping
ENSG00000155097.10	1.3162402	1.80110098	5.39E-05	0.00022856	0.22	0.34	0.71	1.15	ATP6V1C1	ATPase, H+ transporting, lysosomal 42kDa, V1 subunit C1	chr8	protein_coding
ENSG00000210135.1	1.48657115	-1.4508827	0.01203274	0.03038863	0.73	2.72	6.58	6.34	MT-TN	mitochondrially encoded tRNA asparagine	chrM	Mt_tRNA
ENSG00000250903.7	-5.0234041	-3.1294261	0.0100455	0.02582742	0.01	0.02	0	0	GMDS-AS1	GMDS antisense RNA 1 (head to head)	chr6	lincRNA
ENSG00000144031.10	-3.8452921	-1.8509682	1.62E-06	9.05E-06	0.27	0.05	0	0.02	ANKRD53	ankyrin repeat domain 53	chr2	protein_coding
ENSG00000221957.7	-7.8628492	1.84590166	6.48E-45	6.61E-43	2.74	4.51	0	0.03	KIR2DS4	killer cell immunoglobulin-like receptor, two domains, short cytoplasmic tail, 4	chr19	polymorphic_pseudogene
ENSG00000152580.8	3.52953385	0.49435223	1.46E-14	2.23E-13	0	0.03	0.21	0.26	IGSF10	immunoglobulin superfamily, member 10	chr3	protein_coding
ENSG00000106078.16	-1.8418279	4.23582599	8.25E-10	7.29E-09	2.57	2.68	0.81	1.14	COBL	cordon-bleu WH2 repeat protein	chr7	protein_coding
ENSG00000168404.11	-1.0246568	4.6124128	0.00043672	0.0015448	9.35	11.39	6.62	6.96	MLKL	mixed lineage kinase domain-like	chr16	protein_coding
ENSG00000162039.13	8.55273155	-0.3087059	2.65E-17	5.13E-16	0	0	0.68	0.94	MEIOB	meiosis specific with OB domains	chr16	protein_coding
ENSG00000262202.4	6.21753545	3.10391266	1.06E-47	1.30E-45	0.29	0.27	32.94	23.47	RP11-160E2.	--	chr17	lincRNA
ENSG00000163637.10	-5.9955597	-2.569758	0.00014842	0.00057423	0.04	0.01	0	0	PRICKLE2	prickle homolog 2 (Drosophila)	chr3	protein_coding
ENSG00000178974.8	1.36315539	2.60184219	1.11E-05	5.35E-05	0.8	0.71	2.6	2.58	FBXO34	F-box protein 34	chr14	protein_coding
ENSG00000187951.9	1.098985	2.30251911	0.00045179	0.00159247	0.6	1.86	3.15	3.84	ARHGAP11B	Rho GTPase activating protein 11B	chr15	protein_coding
ENSG00000264187.1	1.19523303	3.45264237	6.77E-05	0.0002825	3.46	2.85	10.71	8.51	RP11-45M22	--	chr17	protein_coding
ENSG00000280339.1	5.04680446	-2.9799228	0.0100455	0.02582742	0	0	0.01	0.07	RP11-736K2(	--	chr11	TEC
ENSG00000173706.11	2.21612563	6.77175717	9.86E-14	1.38E-12	3.19	3.65	20.01	22.38	HEG1	heart development protein with EGF-like domains 1	chr3	protein_coding
ENSG00000205464.10	-1.9043323	0.70502819	2.38E-07	1.50E-06	0.79	0.85	0.23	0.35	ATP6AP1L	ATPase, H+ transporting, lysosomal accessory protein 1-like	chr5	protein_coding
ENSG00000131650.12	-2.191117	3.09226329	2.68E-12	3.20E-11	4.55	4.1	1.34	1.19	KREMEN2	kringle containing transmembrane protein 2	chr16	protein_coding
ENSG00000171509.14	-5.0252555	-3.1285772	0.0100455	0.02582742	0	0.05	0	0	RXFP1	relaxin/insulin-like family peptide receptor 1	chr4	protein_coding
ENSG00000126264.8	6.25378322	-2.2167539	1.55E-05	7.26E-05	0	0	0.42	0.58	HCST	hematopoietic cell signal transducer	chr19	protein_coding
ENSG00000100167.18	2.22014759	3.96840776	5.12E-13	6.62E-12	0.75	1.12	6.01	5.55	3-Sep	septin 3	chr22	protein_coding
ENSG00000137962.11	3.25465018	1.49601217	1.69E-17	3.33E-16	0.06	0.07	0.67	0.88	ARHGAP29	Rho GTPase activating protein 29	chr1	protein_coding
ENSG00000108509.19	-1.3593632	6.0449568	2.88E-06	1.55E-05	16.21	15.02	8.19	8.02	CAMTA2	calmodulin binding transcription activator 2	chr17	protein_coding
ENSG00000156113.19	-5.0581611	0.95296584	2.98E-26	1.11E-24	0.37	0.34	0.01	0.01	KCNMA1	potassium channel, calcium activated large conductance subfamily M alpha, member 1	chr10	protein_coding
ENSG00000119919.10	5.36404116	-2.8022277	0.00290522	0.00856312	0	0	0.14	0.03	NKX2-3	NK2 homeobox 3	chr10	protein_coding
ENSG00000157617.15	1.07435424	2.26280141	0.00062594	0.00214972	0.31	0.45	1.01	1.12	C2CD2	C2 calcium-dependent domain containing 2	chr21	protein_coding
ENSG00000231365.4	-1.0145237	3.66098307	0.00061367	0.00211152	2.34	2.67	1.58	1.73	RP11-418J17	--	chr1	antisense
ENSG00000141574.6	2.35366423	6.93517109	3.61E-15	5.84E-14	14.42	14.57	106.57	90.39	SECTM1	secreted and transmembrane 1	chr17	protein_coding
ENSG00000170558.7	-8.6326883	5.26545483	9.91E-84	8.19E-81	12.13	15.86	0.04	0.06	CDH2	cadherin 2, type 1, N-cadherin (neuronal)	chr18	protein_coding
ENSG00000141497.12	4.40443615	-0.1761158	1.78E-14	2.70E-13	0.04	0	0.84	0.5	ZMYND15	zinc finger, MYND-type containing 15	chr17	protein_coding
ENSG00000230337.1	1.2988951	1.92468559	5.45E-05	0.0002309	3.09	3.38	13.15	8.03	RP4-635E18.	--	chr1	antisense
ENSG00000163788.12	2.87585643	1.07552678	2.10E-13	2.82E-12	0.06	0.09	0.61	0.84	SNRK	SNF related kinase	chr3	protein_coding
ENSG00000213064.8	1.38303214	3.11473028	5.53E-06	2.81E-05	0.33	0.37	1.04	1.38	SFT2D2	SFT2 domain containing 2	chr1	protein_coding
ENSG00000259642.2	1.89418575	3.0675835	1.12E-09	9.64E-09	0.59	0.76	3.15	3.53	ST20-AS1	ST20 antisense RNA 1	chr15	antisense
ENSG00000172594.11	2.06672619	0.72098202	6.96E-08	4.78E-07	0.19	0.3	1.1	1.64	SMPDL3A	sphingomyelin phosphodiesterase, acid-like 3A	chr6	protein_coding
ENSG00000281091.1	-7.1822848	-1.6830765	4.48E-09	3.59E-08	0.69	1.23	0	0	RP13-349O2	--	chrX	lincRNA
ENSG00000271151.1	5.35906543	-2.8049519	0.00290522	0.00856312	0	0	0.24	0.08	RP11-394I13	--	chr2	lincRNA
ENSG00000182771.16	-3.8253433	2.49652509	2.05E-26	7.77E-25	1.5	1.42	0.15	0.12	GRID1	glutamate receptor, ionotropic, delta 1	chr10	protein_coding
ENSG00000198160.13	1.57808896	1.57143294	2.58E-06	1.40E-05	0.19	0.11	0.34	0.87	MIER1	mesoderm induction early response 1, transcriptional regulator	chr1	protein_coding
ENSG00000157741.13	1.90086251	1.25841917	8.34E-08	5.65E-07	0.06	0.05	0.29	0.25	UBN2	ubinuclein 2	chr7	protein_coding
ENSG00000085831.14	1.23704718	-0.189468	0.00424613	0.01210611	0.05	0.03	0.15	0.12	TTC39A	tetratricopeptide repeat domain 39A	chr1	protein_coding
ENSG00000179023.8	6.18618221	-2.2653636	2.70E-05	0.00012094	0	0	0.05	0.06	KLHDC7A	kelch domain containing 7A	chr1	protein_coding
ENSG00000047188.14	1.89903601	1.85929982	1.33E-08	9.96E-08	0.09	0.29	0.72	1.12	YTHDC2	YTH domain containing 2	chr5	protein_coding
ENSG00000213398.6	1.13107775	3.11497251	0.00018017	0.00068558	2.77	3.01	8.25	8.62	LCAT	lecithin-cholesterol acyltransferase	chr16	protein_coding
ENSG00000071282.10	2.15382073	2.04457634	1.06E-10	1.06E-09	0.15	0.09	0.81	0.63	LMCD1	LIM and cysteine-rich domains 1	chr3	protein_coding
ENSG00000107159.11	-3.0540931	6.46734735	3.57E-23	1.08E-21	97.11	60.6	12.73	12.62	CA9	carbonic anhydrase IX	chr9	protein_coding
ENSG00000163512.12	1.31812957	2.15229849	3.43E-05	0.00015127	0.31	0.48	0.77	1.88	AZI2	5-azacytidine induced 2	chr3	protein_coding
ENSG00000177595.16	1.29559209	3.47864485	1.65E-05	7.68E-05	1.59	1.86	5.99	5.28	PIDD1	p53-induced death domain protein 1	chr11	protein_coding
ENSG00000083828.14	-2.5543761	0.95523924	7.27E-12	8.27E-11	1.01	0.62	0.17	0.2	ZNF586	zinc finger protein 586	chr19	protein_coding
ENSG00000108107.11	-1.023558	10.6477872	0.00035623	0.00128202	250.23	282.11	197.77	149.57	RPL28	ribosomal protein L28	chr19	protein_coding
ENSG00000165171.9	-1.3957789	1.103615	4.70E-05	0.00020261	3.07	2.2	1.19	1.48	WBSCR27	Williams Beuren syndrome chromosome region 27	chr7	protein_coding
ENSG00000102125.14	-1.9008036	4.43915098	2.29E-10	2.18E-09	12.82	13.88	4.77	4.75	TAZ	tafazzin	chrX	protein_coding
ENSG00000227036.5	-5.6583177	-2.783081	0.00086564	0.00288128	0.06	0.08	0	0	LINC00511	long intergenic non-protein coding RNA 511	chr17	lincRNA
ENSG00000225762.1	3.76381856	-1.5177932	3.56E-06	1.88E-05	0.11	0	1.39	0.9	LINC01389	long intergenic non-protein coding RNA 1389	chr1	antisense
ENSG00000140988.14	-1.4108345	11.5577518	1.07E-06	6.10E-06	3254.14	2842.07	1528.8	1527.71	RPS2	ribosomal protein S2	chr16	protein_coding
ENSG00000175581.12	-1.7286865	5.708784	4.09E-09	3.32E-08	20.67	22.07	8.91	8.25	MRPL48	mitochondrial ribosomal protein L48	chr11	protein_coding
ENSG00000267632.1	2.11514406	-0.5556969	1.76E-05	8.15E-05	0.03	0.18	0.52	0.69	RP11-400F19	--	chr17	sense_intronic
ENSG00000127954.11	6.3040663	6.13867337	4.13E-65	1.30E-62	0.11	0.14	9.15	17.71	STEAP4	STEAP family member 4	chr7	protein_coding
ENSG00000160223.15	2.69254319	4.48356156	4.25E-18	8.81E-17	0.63	0.56	5.16	5.09	ICOSLG	inducible T-cell co-stimulator ligand	chr21	protein_coding
ENSG00000094914.11	-1.2387391	6.34887839	1.86E-05	8.57E-05	46.15	44.83	25.05	26.35	AAAS	achalasia, adrenocortical insufficiency, alacrimia	chr12	protein_coding
ENSG00000130821.14	-1.5900126	7.4679726	4.76E-08	3.33E-07	57.38	53.03	26	22.8	SLC6A8	solute carrier family 6 (neurotransmitter transporter), member 8	chrX	protein_coding
ENSG00000111880.14	1.12730427	1.08112621	0.00114785	0.00372295	0.22	0.23	0.57	0.75	RNGTT	RNA guanylyltransferase and 5'-phosphatase	chr6	protein_coding
ENSG00000226913.1	-2.8596876	-2.1159158	0.00094481	0.00312013	0.15	0.04	0.04	0	BSN-AS2	BSN antisense RNA 2 (head to head)	chr3	lincRNA
ENSG00000169131.9	1.19102015	-0.3165604	0.00653665	0.01776746	0.19	0.09	0.27	0.6	ZNF354A	zinc finger protein 354A	chr5	protein_coding
ENSG00000245904.3	-1.6734695	0.02364919	2.69E-05	0.00012075	0.13	0.19	0.03	0.1	RP11-796E2.	--	chr12	antisense
ENSG00000201772.1	-3.2365208	-2.2721976	0.00108225	0.0035312	2.34	1.03	0.23	0.2	SNORA5C	small nucleolar RNA, H/ACA box 5C	chr7	snoRNA
ENSG00000269897.4	1.88589973	0.27909019	3.18E-06	1.69E-05	0.28	0.26	0.77	1.92	COMMD3-B	COMMD3-BMI1 readthrough	chr10	protein_coding
ENSG00000276966.1	1.93349006	0.82209207	1.77E-07	1.15E-06	2.31	1.27	13.35	5.06	HIST1H4E	histone cluster 1, H4e	chr6	protein_coding
ENSG00000160460.14	3.3803093	1.40896267	3.46E-18	7.20E-17	0.03	0.05	0.55	0.53	SPTBN4	spectrin, beta, non-erythrocytic 4	chr19	protein_coding
ENSG00000154217.13	-3.1202733	6.831781	4.94E-24	1.60E-22	26.03	25.94	3.96	4	PITPNC1	phosphatidylinositol transfer protein, cytoplasmic 1	chr17	protein_coding
ENSG00000173674.9	1.30824503	4.08865782	1.01E-05	4.91E-05	1.69	1.87	3.94	7.9	EIF1AX	eukaryotic translation initiation factor 1A, X-linked	chrX	protein_coding
ENSG00000184156.14	4.21667527	-1.145299	2.89E-08	2.08E-07	0	0	0.02	0.11	KCNQ3	potassium channel, voltage gated KQT-like subfamily Q, member 3	chr8	protein_coding
ENSG00000161647.17	-1.2369253	3.67234072	3.27E-05	0.00014439	4.57	4.69	2.36	2.88	MPP3	membrane protein, palmitoylated 3 (MAGUK p55 subfamily member 3)	chr17	protein_coding
ENSG00000269439.4	-2.4096313	-0.4035714	1.95E-07	1.26E-06	1.28	1.28	0.08	0.53	CTD-3131K8	--	chr19	lincRNA
ENSG00000261888.1	-2.5102129	-0.3048141	3.47E-08	2.47E-07	0.32	0.49	0.07	0.11	AC144831.1	--	chr17	lincRNA
ENSG00000073921.16	1.68649957	4.59651039	1.43E-08	1.07E-07	2.46	2.37	8.45	12.38	PICALM	phosphatidylinositol binding clathrin assembly protein	chr11	protein_coding
ENSG00000047644.16	-2.5256944	5.61655797	6.63E-17	1.24E-15	9.68	10.7	2.21	2.51	WWC3	WWC family member 3	chrX	protein_coding
ENSG00000117148.7	-6.1722403	0.19578664	3.94E-23	1.19E-21	0.52	1.44	0.03	0	ACTL8	actin-like 8	chr1	protein_coding
ENSG00000174721.9	-2.5309469	4.39199049	1.93E-16	3.49E-15	10.71	12.08	2.38	2.88	FGFBP3	fibroblast growth factor binding protein 3	chr10	protein_coding
ENSG00000152413.13	3.66420443	1.30635558	2.50E-19	5.72E-18	0.03	0.07	0.48	1.13	HOMER1	homer scaffolding protein 1	chr5	protein_coding
ENSG00000205542.9	-2.459099	9.02625171	2.19E-16	3.94E-15	460.15	377.01	89.2	114.5	TMSB4X	thymosin beta 4, X-linked	chrX	protein_coding
ENSG00000173812.9	-1.114584	7.95342431	0.00010637	0.00042568	122.75	112.19	70.3	74.42	EIF1	eukaryotic translation initiation factor 1	chr17	protein_coding
ENSG00000104361.8	1.53938166	-0.0197982	0.00024424	0.00091055	0.05	0.12	0.21	0.42	NIPAL2	NIPA-like domain containing 2	chr8	protein_coding
ENSG00000108828.14	-1.8434016	7.64402364	3.30E-10	3.09E-09	98.16	78.46	31.41	34.31	VAT1	vesicle amine transport 1	chr17	protein_coding
ENSG00000203441.2	-1.7373947	-0.0943364	2.54E-05	0.00011446	1.56	1.01	0.26	0.75	LINC00449	long intergenic non-protein coding RNA 449	chr13	antisense
ENSG00000266490.1	7.11736532	-1.555184	4.48E-09	3.59E-08	0	0	1.39	1.06	CTD-2349P2	--	chr17	lincRNA
ENSG00000105379.8	-1.2716336	7.70432892	1.06E-05	5.11E-05	69.7	59.26	37	34.14	ETFB	electron-transfer-flavoprotein, beta polypeptide	chr19	protein_coding
ENSG00000159917.13	1.61437375	-0.5009145	0.00073084	0.00247628	0.04	0.08	0.16	0.35	ZNF235	zinc finger protein 235	chr19	protein_coding
ENSG00000180776.14	5.01027743	0.90818322	1.89E-23	5.83E-22	0	0.03	0.4	0.95	ZDHHC20	zinc finger, DHHC-type containing 20	chr13	protein_coding
ENSG00000179262.8	-1.4502655	8.90177624	5.54E-07	3.33E-06	314.34	330.85	161.95	152.27	RAD23A	RAD23 homolog A (S. cerevisiae)	chr19	protein_coding
ENSG00000163527.8	1.55669381	5.10166245	1.19E-07	7.90E-07	2.98	3.64	9.29	16.85	STT3B	STT3B, subunit of the oligosaccharyltransferase complex (catalytic)	chr3	protein_coding
ENSG00000261744.1	5.08767213	-2.9577572	0.00538165	0.01488851	0	0	0.39	0	RP11-21B21.	--	chr16	antisense
ENSG00000158246.7	1.74100675	3.16183034	1.58E-08	1.18E-07	1.16	1.63	5.99	6.43	FAM46B	family with sequence similarity 46, member B	chr1	protein_coding
ENSG00000078674.16	2.22873394	2.91166171	2.56E-12	3.08E-11	0.21	0.25	1.07	1.81	PCM1	pericentriolar material 1	chr8	protein_coding
ENSG00000197989.12	-1.0401784	4.92745495	0.00034396	0.0012414	20.33	20.31	13.56	12.75	SNHG12	small nucleolar RNA host gene 12	chr1	antisense
ENSG00000171345.12	-1.4098384	2.10779252	9.71E-06	4.72E-05	3.66	3.59	2.26	1.37	KRT19	keratin 19, type I	chr17	protein_coding
ENSG00000180071.17	2.1179794	-0.5510294	1.76E-05	8.15E-05	0.04	0.04	0.04	0.44	ANKRD18A	ankyrin repeat domain 18A	chr9	protein_coding
ENSG00000148057.14	1.80055852	1.05884893	5.85E-07	3.50E-06	0.55	0.63	2.01	3.51	IDNK	idnK, gluconokinase homolog (E. coli)	chr9	protein_coding
ENSG00000047648.20	-5.9972113	-2.568945	0.00014842	0.00057423	0.04	0.02	0	0	ARHGAP6	Rho GTPase activating protein 6	chrX	protein_coding
ENSG00000234913.1	2.27459704	-2.1490922	0.00923116	0.02429359	0.01	0.02	0.15	0.08	XXbac-B476	--	chr22	sense_intronic
ENSG00000139793.17	2.48274541	0.64684902	5.64E-10	5.09E-09	0.07	0.08	0.43	0.68	MBNL2	muscleblind-like splicing regulator 2	chr13	protein_coding
ENSG00000184949.14	1.98580833	1.19779862	2.89E-08	2.07E-07	0.07	0.07	0.34	0.39	FAM227A	family with sequence similarity 227, member A	chr22	protein_coding
ENSG00000185621.10	1.41544282	0.80278073	9.87E-05	0.00039794	0.09	0.12	0.31	0.41	LMLN	leishmanolysin-like (metallopeptidase M8 family)	chr3	protein_coding
ENSG00000114854.6	2.42486037	0.92354874	2.31E-10	2.20E-09	0.56	0.71	4.17	5	TNNC1	troponin C type 1 (slow)	chr3	protein_coding
ENSG00000118004.16	6.04022639	-2.3681409	8.36E-05	0.00034052	0	0	0.1	0.11	COLEC11	collectin sub-family member 11	chr2	protein_coding
ENSG00000142303.12	-4.1638093	3.58992855	2.17E-33	1.19E-31	4.72	3.78	0.32	0.31	ADAMTS10	ADAM metallopeptidase with thrombospondin type 1 motif, 10	chr19	protein_coding
ENSG00000125122.13	1.72134685	-0.5831725	0.00033978	0.00122705	0.15	0.04	0.23	0.59	LRRC29	leucine rich repeat containing 29	chr16	protein_coding
ENSG00000177054.12	2.90331261	1.27152299	1.50E-14	2.29E-13	0.33	0.02	1.36	2.24	ZDHHC13	zinc finger, DHHC-type containing 13	chr11	protein_coding
ENSG00000133739.14	8.31964694	-0.5132102	2.12E-15	3.50E-14	0	0	0.15	0.4	LRRCC1	leucine rich repeat and coiled-coil centrosomal protein 1	chr8	protein_coding
ENSG00000151725.10	3.23203741	1.16752981	3.74E-16	6.59E-15	0.2	0.13	1.52	2.66	CENPU	centromere protein U	chr4	protein_coding
ENSG00000178172.5	-3.314345	3.21775216	1.88E-23	5.82E-22	21.45	15.85	2.36	2.64	SPINK6	serine peptidase inhibitor, Kazal type 6	chr5	protein_coding
ENSG00000170382.10	5.47533717	1.72024013	2.98E-32	1.53E-30	0.02	0.02	1.23	1.17	LRRN2	leucine rich repeat neuronal 2	chr1	protein_coding
ENSG00000169372.11	-1.1524977	2.07880275	0.00024997	0.00093047	1.68	1.91	0.92	1.23	CRADD	CASP2 and RIPK1 domain containing adaptor with death domain	chr12	protein_coding
ENSG00000114993.14	2.15930302	4.94783138	7.63E-13	9.67E-12	3.2	3.11	19.67	17.83	RTKN	rhotekin	chr2	protein_coding
ENSG00000121039.8	-1.0662425	2.15127987	0.00060841	0.00209711	1.11	1.24	0.5	1	RDH10	retinol dehydrogenase 10 (all-trans)	chr8	protein_coding
ENSG00000227603.1	1.61926985	-0.6558242	0.00068172	0.00232333	0.39	0.25	1.25	1.38	RP11-165J3.6	--	chr9	antisense
ENSG00000156162.15	2.38005684	0.27274561	1.39E-08	1.04E-07	0.06	0.04	0.27	0.38	DPY19L4	dpy-19-like 4 (C. elegans)	chr8	protein_coding
ENSG00000205213.12	1.19746294	0.08174522	0.00265243	0.00795995	0.13	0.05	0.16	0.39	LGR4	leucine-rich repeat containing G protein-coupled receptor 4	chr11	protein_coding
ENSG00000178404.8	1.39057678	-0.4599978	0.00249754	0.00752149	0.14	0.14	0.38	0.59	KIAA1731NL	--	chr17	protein_coding
ENSG00000136052.8	2.39478187	0.89891002	3.95E-10	3.65E-09	0.09	0.11	0.67	0.77	SLC41A2	solute carrier family 41 (magnesium transporter), member 2	chr12	protein_coding
ENSG00000173110.7	-3.416468	1.74565569	1.76E-20	4.39E-19	2.59	1.58	0.24	0.28	HSPA6	heat shock 70kDa protein 6 (HSP70B')	chr1	protein_coding
ENSG00000119471.13	1.79302006	3.10532213	7.13E-09	5.60E-08	0.95	0.89	3.38	5.16	HSDL2	hydroxysteroid dehydrogenase like 2	chr9	protein_coding
ENSG00000142182.7	5.24906365	-1.0287627	4.59E-10	4.19E-09	0	0.02	0.3	0.69	DNMT3L	DNA (cytosine-5-)-methyltransferase 3-like	chr21	protein_coding
ENSG00000175061.16	-2.3446532	7.21277114	4.21E-15	6.79E-14	72.38	61.35	17.38	17.73	LRRC75A-AS	LRRC75A antisense RNA 1	chr17	processed_transcript
ENSG00000185658.12	1.66320994	2.49614396	1.42E-07	9.28E-07	0.1	0.13	0.35	0.65	BRWD1	bromodomain and WD repeat domain containing 1	chr21	protein_coding
ENSG00000213047.10	2.24161536	-1.2302328	0.00018398	0.00069885	0	0.02	0.05	0.08	DENND1B	DENN/MADD domain containing 1B	chr1	protein_coding
ENSG00000240211.1	4.10611277	-1.9438862	1.13E-05	5.42E-05	0	0.09	1.27	1.26	RP11-758P17	--	chr7	antisense
ENSG00000103642.10	1.74298153	1.94240672	1.20E-07	7.97E-07	0.33	0.4	0.98	2.32	LACTB	lactamase, beta	chr15	protein_coding
ENSG00000028277.19	3.09246227	-1.5840852	4.58E-05	0.00019809	0	0.01	0.07	0.07	POU2F2	POU class 2 homeobox 2	chr19	protein_coding
ENSG00000089048.13	4.66793935	2.41534147	2.58E-32	1.33E-30	0.15	0.04	1.57	5.21	ESF1	ESF1, nucleolar pre-rRNA processing protein, homolog (S. cerevisiae)	chr20	protein_coding
ENSG00000142864.13	-1.5799258	8.40502739	5.53E-08	3.84E-07	56.64	65.51	23.28	31.33	SERBP1	SERPINE1 mRNA binding protein 1	chr1	protein_coding
ENSG00000274767.1	4.15924347	-1.9055178	6.92E-06	3.47E-05	0.02	0	0.27	0.29	AC131056.3	--	chr17	antisense
ENSG00000172667.9	5.11750692	4.42135384	8.86E-46	9.57E-44	0.11	0.09	4.45	4.93	ZMAT3	zinc finger, matrin-type 3	chr3	protein_coding
ENSG00000170802.14	2.73479465	-1.0521254	5.07E-06	2.60E-05	0.02	0.01	0.11	0.17	FOXN2	forkhead box N2	chr2	protein_coding
ENSG00000132122.10	7.7681461	-1.0076399	1.50E-11	1.66E-10	0	0	0.19	0.17	SPATA6	spermatogenesis associated 6	chr1	protein_coding
ENSG00000055147.16	1.00721651	2.07002806	0.00149227	0.00474313	0.3	0.58	0.97	1.41	FAM114A2	family with sequence similarity 114, member A2	chr5	protein_coding
ENSG00000115232.12	-5.2072905	-3.0363253	0.00538165	0.01488851	0.03	0.01	0	0	ITGA4	integrin, alpha 4 (antigen CD49D, alpha 4 subunit of VLA-4 receptor)	chr2	protein_coding
ENSG00000274751.1	1.62246682	-1.3614589	0.00496338	0.01395792	0	0.07	0.16	0.11	LA16c-358B7	--	chr16	antisense
ENSG00000256269.5	-1.2528549	4.75866048	1.83E-05	8.48E-05	18.37	16.48	10.81	8.64	HMBS	hydroxymethylbilane synthase	chr11	protein_coding
ENSG00000259080.1	-1.3316425	0.77357141	0.00021958	0.000824	1.08	1.16	0.55	0.63	RP11-158I13	--	chr2	processed_transcript
ENSG00000107984.8	3.93480138	4.31934215	4.87E-32	2.48E-30	1.09	1.11	18.52	26.55	DKK1	dickkopf WNT signaling pathway inhibitor 1	chr10	protein_coding
ENSG00000157150.4	-4.4377365	5.17028929	8.75E-40	6.55E-38	35.09	32.46	2.35	1.8	TIMP4	TIMP metallopeptidase inhibitor 4	chr3	protein_coding
ENSG00000108375.11	2.84791742	0.20900815	2.46E-10	2.33E-09	0.03	0.03	0.18	0.37	RNF43	ring finger protein 43	chr17	protein_coding
ENSG00000109066.12	1.16032488	5.090518	6.70E-05	0.00027955	3.3	3.63	11.24	9.36	TMEM104	transmembrane protein 104	chr17	protein_coding
ENSG00000206573.7	2.2831917	1.78329122	3.25E-11	3.43E-10	0.19	0.1	0.91	1	THUMPD3-A	THUMPD3 antisense RNA 1	chr3	antisense
ENSG00000264672.4	2.35089795	-2.4817749	0.01923175	0.0458365	0	0.04	0.08	0.17	SEPT4-AS1	SEPT4 antisense RNA 1	chr17	antisense
ENSG00000184432.8	1.34791238	6.57839663	3.35E-06	1.78E-05	13.27	12.68	36.83	51.58	COPB2	coatomer protein complex, subunit beta 2 (beta prime)	chr3	protein_coding
ENSG00000149929.14	-1.8062354	7.32530629	7.24E-10	6.42E-09	52.06	67.29	25.04	20.25	HIRIP3	HIRA interacting protein 3	chr16	protein_coding
ENSG00000137857.16	-3.1077508	-1.6589351	8.20E-06	4.05E-05	0.04	0.1	0	0.02	DUOX1	dual oxidase 1	chr15	protein_coding
ENSG00000172086.7	2.1840711	0.89330289	8.72E-09	6.75E-08	0.2	0.39	0.91	2.64	KRCC1	lysine-rich coiled-coil 1	chr2	protein_coding
ENSG00000107938.16	1.46497869	1.53606753	1.37E-05	6.48E-05	0.32	0.2	0.74	1.2	EDRF1	erythroid differentiation regulatory factor 1	chr10	protein_coding
ENSG00000020922.11	2.82511881	3.37736485	2.01E-18	4.30E-17	0.24	0.3	2.01	3.16	MRE11A	MRE11 meiotic recombination 11 homolog A (S. cerevisiae)	chr11	protein_coding
ENSG00000165195.12	2.26547207	-0.9097775	4.71E-05	0.00020301	0.04	0.03	0.15	0.29	PIGA	phosphatidylinositol glycan anchor biosynthesis, class A	chrX	protein_coding
ENSG00000132842.12	1.14540401	3.67483348	0.00011684	0.00046342	1.04	1.15	2.41	4.06	AP3B1	adaptor-related protein complex 3, beta 1 subunit	chr5	protein_coding
ENSG00000000938.11	-5.8949226	-2.6359652	0.00026535	0.0009747	0.05	0.06	0	0	FGR	FGR proto-oncogene, Src family tyrosine kinase	chr1	protein_coding
ENSG00000203727.3	2.87238515	0.61140311	4.22E-12	4.94E-11	0.06	0.03	0.3	0.59	SAMD5	sterile alpha motif domain containing 5	chr6	protein_coding
ENSG00000251537.4	-2.8183311	6.06822099	2.70E-20	6.63E-19	17.61	16.9	3.14	3.39	RP11-385D1	--	chr17	protein_coding
ENSG00000175854.10	-1.21875	5.31639498	2.76E-05	0.00012339	29.17	27.15	15.87	16.4	SWI5	SWI5 recombination repair homolog (yeast)	chr9	protein_coding
ENSG00000018510.11	1.08563789	3.22681076	0.00030779	0.00111693	0.58	0.66	1.33	2.19	AGPS	alkylglycerone phosphate synthase	chr2	protein_coding
ENSG00000261485.1	1.13244728	0.11923737	0.00497899	0.01399254	0.36	0.4	0.57	1.63	PAN3-AS1	PAN3 antisense RNA 1	chr13	antisense
ENSG00000253102.1	5.97010963	-2.4174709	8.36E-05	0.00034052	0	0	0.45	0.27	RP1-117B12.	--	chr17	antisense
ENSG00000185033.13	-1.397379	6.5585446	1.48E-06	8.29E-06	31.05	25.24	14.43	14.05	SEMA4B	sema domain, immunoglobulin domain (Ig), transmembrane domain (TM) and short cytoplasmic domain, (semaphorin) 4B	chr15	protein_coding
ENSG00000152404.14	4.76617235	2.84317196	1.81E-35	1.09E-33	0.04	0.21	2.97	6.03	CWF19L2	CWF19-like 2, cell cycle control (S. pombe)	chr11	protein_coding
ENSG00000179593.14	-2.865836	-2.1128166	0.00094481	0.00312013	0.07	0.12	0.04	0	ALOX15B	arachidonate 15-lipoxygenase, type B	chr17	protein_coding

ENSG00000173214.5	2.22709201	1.63937464	1.55E-10	1.51E-09	0.09	0.2	0.98	0.81	KIAA1919	KIAA1919	chr6	protein_coding
ENSG00000245317.2	1.42083348	-1.0542539	0.00959833	0.02503712	0.2	0.04	0.4	0.49	CTC-241N9.1	--	chr5	lincRNA
ENSG00000272462.2	-1.293789	2.07451836	4.48E-05	0.00019413	0.82	1.52	0.57	0.69	U91328.19	--	chr6	lincRNA
ENSG00000132405.17	-1.3038438	4.99639579	7.93E-06	3.93E-05	6.78	6.94	3.28	4.14	TBC1D14	TBC1 domain family, member 14	chr4	protein_coding
ENSG00000270074.1	4.90063394	-0.5739117	1.04E-12	1.30E-11	0.03	0	0.7	0.86	RP11-351I21	--	chr8	lincRNA
ENSG00000106538.8	-10.05405	1.00518575	4.62E-34	2.58E-32	2.38	1.46	0	0	RARRES2	retinoic acid receptor responder (tazarotene induced) 2	chr7	protein_coding
ENSG00000196455.6	1.46265968	3.15366804	1.60E-06	8.91E-06	0.65	0.86	2.41	3.17	PIK3R4	phosphoinositide-3-kinase, regulatory subunit 4	chr3	protein_coding
ENSG00000140941.11	1.03935031	5.8253551	0.00032098	0.00116321	9.34	10.57	24.23	30.42	MAP1LC3B	microtubule-associated protein 1 light chain 3 beta	chr16	protein_coding
ENSG00000280081.2	6.60993444	-1.9521913	1.05E-06	6.02E-06	0	0	0.23	0.33	MGC39584	--	chr21	lincRNA
ENSG00000078043.14	1.60963869	1.70572411	1.23E-06	6.96E-06	0.1	0.08	0.35	0.39	PIAS2	protein inhibitor of activated STAT, 2	chr18	protein_coding
ENSG00000196305.16	-1.0913227	6.13362003	0.00015552	0.0005992	15.5	16.8	7.92	12.38	IARS	isoleucyl-tRNA synthetase	chr9	protein_coding
ENSG00000272468.1	3.2854411	-1.8715938	0.00025363	0.00094222	0.09	0	0.51	0.87	RP1-86C11.7	--	chr6	lincRNA
ENSG00000267270.4	-3.9866211	1.11826444	6.85E-21	1.75E-19	0.71	0.72	0.13	0	PARD6G-AS1	PARD6G antisense RNA 1	chr18	antisense
ENSG00000129535.11	1.6058583	-0.0242666	0.0001402	0.00054692	0.04	0.2	0.54	0.45	NRL	neural retina leucine zipper	chr14	protein_coding
ENSG00000164211.11	-5.3981927	-0.031505	1.63E-19	3.78E-18	0.33	0.22	0.01	0	STARD4	StAR-related lipid transfer (START) domain containing 4	chr5	protein_coding
ENSG00000063438.15	-1.72754	5.60504255	4.21E-09	3.41E-08	9.47	9.78	3.37	4.39	AHRR	aryl-hydrocarbon receptor repressor	chr5	protein_coding
ENSG00000062282.13	-1.6532077	3.89494026	3.60E-08	2.55E-07	8.12	6.74	2.99	3.32	DGAT2	diacylglycerol O-acyltransferase 2	chr11	protein_coding
ENSG00000203288.3	2.65839846	0.49826111	1.31E-10	1.30E-09	0.26	0.25	2.11	2.2	RP11-98D18	--	chr1	antisense
ENSG00000162591.14	-1.944034	5.08403599	6.04E-11	6.21E-10	8.7	6.22	2.57	2.61	MEGF6	multiple EGF-like-domains 6	chr1	protein_coding
ENSG00000130363.10	1.61117791	2.85165594	1.99E-07	1.28E-06	0.36	0.51	1.8	1.73	RSPH3	radial spoke 3 homolog (Chlamydomonas)	chr6	protein_coding
ENSG00000117448.12	-1.1108223	7.23291079	0.00011405	0.00045301	79.58	59.76	43.78	42.25	AKR1A1	aldo-keto reductase family 1, member A1 (aldehyde reductase)	chr1	protein_coding
ENSG00000196470.10	1.58622668	1.94629122	1.22E-06	6.92E-06	0.3	0.44	1	1.97	SIAH1	siah E3 ubiquitin protein ligase 1	chr16	protein_coding
ENSG00000269386.4	1.82786684	2.3721831	1.14E-08	8.68E-08	1.43	1.69	6.98	7.82	RAB11B-AS1	RAB11B antisense RNA 1	chr19	antisense
ENSG00000226696.4	-1.1257828	1.92177836	0.00043739	0.00154672	3.43	3.25	2.44	1.64	LENG8-AS1	LENG8 antisense RNA 1	chr19	antisense
ENSG00000142910.14	-3.2562671	6.66724188	1.03E-25	3.72E-24	64.33	66.33	9.61	8.58	TINAGL1	tubulointerstitial nephritis antigen-like 1	chr1	protein_coding
ENSG00000073417.13	1.13729409	2.03230851	0.00038162	0.00136561	0.48	0.5	0.89	1.98	PDE8A	phosphodiesterase 8A	chr15	protein_coding
ENSG00000111727.10	1.36517317	-0.5396442	0.00377991	0.01089087	0.04	0.06	0.16	0.18	HCFC2	host cell factor C2	chr12	protein_coding
ENSG00000081479.11	4.88946445	-3.0622981	0.01889873	0.04519557	0	0	0	0.01	LRP2	low density lipoprotein receptor-related protein 2	chr2	protein_coding
ENSG00000197594.10	-1.9890135	0.26470354	6.41E-07	3.80E-06	0.2	0.21	0.09	0.05	ENPP1	ectonucleotide pyrophosphatase/phosphodiesterase 1	chr6	protein_coding
ENSG00000134871.16	-10.981985	10.0764588	1.13E-135	2.71E-131	277.38	279.1	0.18	0.18	COL4A2	collagen, type IV, alpha 2	chr13	protein_coding
ENSG00000131477.9	2.62329808	1.41419809	7.31E-13	9.31E-12	0.72	0.38	4.29	4.88	RAMP2	receptor (G protein-coupled) activity modifying protein 2	chr17	protein_coding
ENSG00000120539.13	2.80604198	2.31303101	2.16E-16	3.88E-15	0.18	0.36	2.14	2.92	MASTL	microtubule associated serine/threonine kinase-like	chr10	protein_coding
ENSG00000157212.17	1.48540359	3.91033159	6.97E-07	4.11E-06	1.56	1.76	5.71	6.71	PAXIP1	PAX interacting (with transcription-activation domain) protein 1	chr7	protein_coding
ENSG00000264745.1	7.52026235	-1.2167394	3.23E-11	3.41E-10	0	0	0.78	1.03	TTC39C-AS1	TTC39C antisense RNA 1	chr18	antisense
ENSG00000107957.15	-3.4531267	5.99184754	7.84E-28	3.24E-26	8	8.48	0.99	1.01	SH3PXD2A	SH3 and PX domains 2A	chr10	protein_coding
ENSG00000153147.5	2.0925229	4.36992693	4.93E-12	5.73E-11	0.76	0.84	2.93	6.22	SMARCA5	SWI/SNF related, matrix associated, actin dependent regulator of chromatin, subfamily a, member 5	chr4	protein_coding
ENSG00000100802.13	-1.1807326	3.09981654	9.29E-05	0.00037552	2.57	2.87	1.52	1.67	C14orf93	chromosome 14 open reading frame 93	chr14	protein_coding
ENSG00000260604.2	4.66297837	2.06897454	6.02E-30	2.76E-28	0.04	0.03	1.21	1.18	RP1-140K8.5	--	chr6	lincRNA
ENSG00000073605.17	1.13161305	0.98715651	0.00141502	0.00451978	0.58	0.49	1.2	1.91	GSDMB	gasdermin B	chr17	protein_coding
ENSG00000073849.13	-2.9728461	2.0196189	1.02E-17	2.04E-16	1	1.43	0.15	0.26	ST6GAL1	ST6 beta-galactosamide alpha-2,6-sialyltranferase 1	chr3	protein_coding
ENSG00000259826.1	2.2656848	-0.9070811	4.71E-05	0.00020301	0.02	0.09	0.26	0.46	RP11-467D6	--	chr7	antisense
ENSG00000105643.8	-1.6801142	6.0549909	9.84E-09	7.55E-08	28.35	27.35	11.4	11.77	ARRDC2	arrestin domain containing 2	chr19	protein_coding
ENSG00000167889.11	2.30829953	3.1034339	3.15E-13	4.15E-12	0.54	0.39	3.29	2.88	MGAT5B	mannosyl (alpha-1,6-)-glycoprotein beta-1,6-N-acetyl-glucosaminyltransferase, isozyme B	chr17	protein_coding
ENSG00000143184.4	5.94755028	-2.430277	0.00014842	0.00057423	0	0	0.12	0.24	XCL1	chemokine (C motif) ligand 1	chr1	protein_coding
ENSG00000111671.8	-2.0260598	3.6295812	3.85E-11	4.03E-10	9.95	6.83	3	2.49	SPSB2	splA/ryanodine receptor domain and SOCS box containing 2	chr12	protein_coding
ENSG00000173276.12	1.87099468	0.44660636	2.43E-06	1.32E-05	0.06	0.06	0.23	0.34	ZBTB21	zinc finger and BTB domain containing 21	chr21	protein_coding
ENSG00000178440.5	5.05694572	-2.9744029	0.0100455	0.02582742	0	0	0.17	0.29	LINC00843	long intergenic non-protein coding RNA 843	chr10	lincRNA
ENSG00000231760.4	-2.0975777	-0.3461729	1.73E-06	9.62E-06	0.49	0.44	0.09	0.19	RP11-350J20	--	chr6	antisense
ENSG00000273329.1	2.39765069	1.09125336	1.07E-10	1.07E-09	0.09	0.06	0.46	0.58	RP11-448A1	--	chr7	lincRNA
ENSG00000279110.1	5.45490402	-2.7467519	0.00158008	0.00495521	0	0	0.01	0.02	CTA-243E7.1	--	chr22	antisense
ENSG00000176853.14	2.02103029	2.18188481	7.14E-10	6.33E-09	0.26	0.24	1.08	1.65	FAM91A1	family with sequence similarity 91, member A1	chr8	protein_coding
ENSG00000277597.1	1.79446745	-0.891448	0.00058604	0.0020272	0.13	0.67	1.65	1.98	RP11-961A1	--	chr17	antisense
ENSG00000089356.15	-1.1861497	0.46272653	0.00147063	0.00467959	0.38	0.67	0.38	0.24	FXYD3	FXYD domain containing ion transport regulator 3	chr19	protein_coding
ENSG00000141219.14	2.10296372	1.56201732	1.57E-09	1.34E-08	0.16	0.27	1.04	1.42	C17orf80	chromosome 17 open reading frame 80	chr17	protein_coding
ENSG00000272264.1	-5.5267521	-2.8610217	0.00158008	0.00495521	0.05	0.44	0	0	RP11-92K15.	--	chr8	lincRNA
ENSG00000116489.11	1.1942687	3.72089558	6.11E-05	0.00025671	2.28	1.98	4.93	8.16	CAPZA1	capping protein (actin filament) muscle Z-line, alpha 1	chr1	protein_coding
ENSG00000144674.15	5.45174362	3.22665392	2.22E-43	2.07E-41	0.04	0.04	1.34	3.66	GOLGA4	golgin A4	chr3	protein_coding
ENSG00000263013.1	1.69533551	2.00083562	2.12E-07	1.36E-06	0.99	1.59	5.78	5.33	RP11-876N2	--	chr16	sense_intronic
ENSG00000099260.9	-5.4149148	0.36720498	1.67E-22	4.84E-21	0.7	0.79	0.05	0	PALMD	palmdelphin	chr1	protein_coding
ENSG00000100422.12	-1.3191818	6.44614715	5.31E-06	2.71E-05	23.87	21.39	11.52	12.69	CERK	ceramide kinase	chr22	protein_coding
ENSG00000276278.1	2.17150108	0.10494717	3.44E-07	2.14E-06	0.35	0.12	1.36	1.49	RP11-182J1.1	--	chr15	antisense
ENSG00000174032.15	1.84895166	0.93173454	5.40E-07	3.24E-06	0.06	0.28	0.84	0.8	SLC25A30	solute carrier family 25, member 30	chr13	protein_coding
ENSG00000168461.11	-1.7982881	5.33778069	1.09E-09	9.51E-09	11.52	13.58	3.79	5.86	RAB31	RAB31, member RAS oncogene family	chr18	protein_coding
ENSG00000159450.11	4.08439203	0.07124563	1.77E-14	2.69E-13	0.01	0.02	0.18	0.37	TCHH	trichohyalin	chr1	protein_coding
ENSG00000230453.8	1.44306558	0.63374579	0.00010226	0.00041131	0.09	0.25	0.37	0.85	ANKRD18B	ankyrin repeat domain 18B	chr9	protein_coding
ENSG00000154153.12	3.71336538	1.3423216	8.42E-20	2.00E-18	0.09	0.06	1.15	1.49	FAM134B	family with sequence similarity 134, member B	chr5	protein_coding
ENSG00000198755.9	-1.3305795	9.14318318	4.06E-06	2.12E-05	996.94	831.23	467.65	502.57	RPL10A	ribosomal protein L10a	chr6	protein_coding
ENSG00000099866.13	1.39826687	0.18218747	0.0003882	0.00138707	0.21	0.14	0.47	0.77	MADCAM1	mucosal vascular addressin cell adhesion molecule 1	chr19	protein_coding
ENSG00000230461.7	4.87917439	-3.0678699	0.01889873	0.04519557	0	0	0	0.02	PROX1-AS1	PROX1 antisense RNA 1	chr1	processed_transcript
ENSG00000137713.14	2.00349173	1.66824133	4.97E-09	3.97E-08	0.14	0.2	0.86	0.95	PPP2R1B	protein phosphatase 2, regulatory subunit A, beta	chr11	protein_coding
ENSG00000176406.19	5.19748351	-2.8969879	0.00538165	0.01488851	0	0	0.01	0.02	RIMS2	regulating synaptic membrane exocytosis 2	chr8	protein_coding
ENSG00000104133.13	1.06010573	2.03126935	0.00089645	0.00297599	0.19	0.32	0.55	0.87	SPG11	spastic paraplegia 11 (autosomal recessive)	chr15	protein_coding
ENSG00000114062.16	2.28746686	3.629048	2.00E-13	2.69E-12	0.36	0.36	1.75	2.95	UBE3A	ubiquitin protein ligase E3A	chr15	protein_coding
ENSG00000126803.9	1.2197973	3.07353355	5.89E-05	0.00024811	0.8	0.6	2.26	2.09	HSPA2	heat shock 70kDa protein 2	chr14	protein_coding
ENSG00000260464.1	-1.8832971	-1.0163741	0.00029743	0.00108441	0.41	0.24	0.11	0.12	RP4-561L24.	--	chr1	lincRNA
ENSG00000181938.12	1.30779571	5.06439111	7.56E-06	3.76E-05	6.92	6.07	21.48	21.41	GINS3	GINS complex subunit 3 (Psf3 homolog)	chr16	protein_coding
ENSG00000120915.12	2.36267768	2.34992478	7.41E-13	9.43E-12	0.6	0.23	2.96	2.78	EPHX2	epoxide hydrolase 2, cytoplasmic	chr8	protein_coding
ENSG00000169758.11	3.2874493	3.57039434	1.82E-23	5.65E-22	0.56	0.84	9.53	8.71	C15orf27	chromosome 15 open reading frame 27	chr15	protein_coding
ENSG00000178084.1	4.88946445	-3.0622981	0.01889873	0.04519557	0	0	0.04	0.1	HTR3C	5-hydroxytryptamine (serotonin) receptor 3C, ionotropic	chr3	protein_coding
ENSG00000177614.8	1.04850659	3.71782975	0.00040505	0.0014404	0.72	0.6	2.01	1.61	PGBD5	piggyBac transposable element derived 5	chr1	protein_coding
ENSG00000213780.9	-1.2109313	4.93548209	3.29E-05	0.00014524	20.58	19.26	12.24	10.66	GTF2H4	general transcription factor IIH, polypeptide 4, 52kDa	chr6	protein_coding
ENSG00000125149.10	1.122942	3.85183997	0.00015201	0.0005867	2.45	2.61	7.56	7.11	C16orf70	chromosome 16 open reading frame 70	chr16	protein_coding
ENSG00000165424.6	-1.0077338	3.5131843	0.00069857	0.00237667	2.32	2.56	1.53	1.7	ZCCHC24	zinc finger, CCHC domain containing 24	chr10	protein_coding
ENSG00000154553.12	-10.043513	0.99447407	6.30E-34	3.51E-32	1.21	0.87	0	0	PDLIM3	PDZ and LIM domain 3	chr4	protein_coding
ENSG00000106004.4	1.29473503	1.69654655	7.93E-05	0.00032667	0.76	1.03	1.8	4.06	HOXA5	homeobox A5	chr7	protein_coding
ENSG00000182782.7	2.49092422	0.22441987	6.76E-09	5.32E-08	0.2	0.06	0.87	1.05	HCAR2	hydroxycarboxylic acid receptor 2	chr12	protein_coding
ENSG00000150764.12	-2.2077048	1.42984798	2.86E-10	2.70E-09	0.37	0.56	0.15	0.11	DIXDC1	DIX domain containing 1	chr11	protein_coding
ENSG00000135241.15	2.17416038	0.18502312	3.39E-07	2.10E-06	0.01	0.12	0.24	0.52	PNPLA8	patatin-like phospholipase domain containing 8	chr7	protein_coding
ENSG00000169071.13	-4.9461961	4.9197456	8.95E-46	9.62E-44	9.9	9.7	0.4	0.45	ROR2	receptor tyrosine kinase-like orphan receptor 2	chr9	protein_coding
ENSG00000230439.2	-1.0409149	0.3365554	0.00500697	0.01406458	0.91	0.68	0.29	0.73	RP11-488P3.	--	chr1	sense_intronic
ENSG00000144048.9	1.02960363	2.31536714	0.00102362	0.00335589	0.78	0.85	1.68	2.79	DUSP11	dual specificity phosphatase 11 (RNA/RNP complex 1-interacting)	chr2	protein_coding
ENSG00000271040.1	2.06112484	-1.5415863	0.00173462	0.0053947	0	0.33	1.06	0.79	RP5-933K21.	--	chr6	lincRNA
ENSG00000268204.1	5.97010963	-2.4174709	8.36E-05	0.00034052	0	0	0.38	0.23	CTD-3214H1	--	chr19	antisense
ENSG00000180530.8	2.27706855	-0.0805636	4.31E-07	2.63E-06	0.01	0.05	0.12	0.27	NRIP1	nuclear receptor interacting protein 1	chr21	protein_coding
ENSG00000212864.3	-1.7552196	2.31904423	4.32E-08	3.03E-07	5.54	5.25	2.79	1.47	RNF208	ring finger protein 208	chr9	protein_coding
ENSG00000131469.11	-1.1322191	9.45614588	8.16E-05	0.00033552	800.52	597.14	377.01	476.38	RPL27	ribosomal protein L27	chr17	protein_coding
ENSG00000259319.1	4.91020659	-3.0511096	0.0100455	0.02582742	0	0	0.05	0.01	RP11-293M1	--	chr14	antisense
ENSG00000122641.9	-3.3256974	-2.7864371	0.00943894	0.02469931	0.01	0.03	0	0	INHBA	inhibin, beta A	chr7	protein_coding
ENSG00000266208.1	1.00349091	4.50592188	0.00058769	0.00203215	5.71	5.94	15.01	16.14	CTD-2267D1	--	chr17	antisense
ENSG00000172572.6	-3.9256603	2.03387029	2.60E-26	9.77E-25	0.78	0.86	0.03	0.12	PDE3A	phosphodiesterase 3A, cGMP-inhibited	chr12	protein_coding
ENSG00000260552.1	4.46886091	3.10621069	2.86E-34	1.61E-32	1.14	0.79	30.4	27.05	RP11-49I11.1	--	chr18	antisense
ENSG00000053372.4	-1.3053292	7.49625403	6.29E-06	3.17E-05	91.82	87.93	49.55	47.31	MRTO4	mRNA turnover 4 homolog (S. cerevisiae)	chr1	protein_coding
ENSG00000009844.14	2.07582974	1.64079756	1.59E-09	1.36E-08	0.15	0.11	0.56	0.93	VTA1	vesicle (multivesicular body) trafficking 1	chr6	protein_coding
ENSG00000274712.1	2.58254943	1.38547734	1.53E-12	1.88E-11	0.28	0.3	1.92	2.72	RP11-147L13	--	chr17	lincRNA
ENSG00000100744.13	1.04726934	0.85206157	0.00308823	0.00905801	0.2	0.27	0.62	0.66	GSKIP	GSK3B interacting protein	chr14	protein_coding
ENSG00000136560.12	2.5400456	1.02197964	2.83E-11	3.02E-10	0.14	0.18	0.67	1.9	TANK	TRAF family member-associated NFKB activator	chr2	protein_coding
ENSG00000164134.11	1.11436687	2.26804173	0.00039767	0.00141668	0.21	0.36	0.61	1.02	NAA15	N(alpha)-acetyltransferase 15, NatA auxiliary subunit	chr4	protein_coding
ENSG00000128272.13	-1.2474845	10.096218	1.49E-05	7.03E-05	462.74	530.12	294.97	260.84	ATF4	activating transcription factor 4	chr22	protein_coding
ENSG00000187650.3	1.63278506	0.96013144	5.61E-06	2.85E-05	0.25	0.4	1.34	1.35	VMAC	vimentin-type intermediate filament associated coiled-coil protein	chr19	protein_coding
ENSG00000162980.15	1.90629021	0.67856906	7.02E-07	4.14E-06	0.03	0.16	0.4	0.56	ARL5A	ADP-ribosylation factor-like 5A	chr2	protein_coding
ENSG00000138152.8	4.13120141	-1.9301309	1.13E-05	5.42E-05	0.01	0	0.31	0.12	BTBD16	BTB (POZ) domain containing 16	chr10	protein_coding
ENSG00000269825.1	4.00139403	-0.8661836	1.79E-09	1.51E-08	0.02	0.01	0.34	0.33	CTD-3099C6	--	chr19	sense_intronic
ENSG00000281298.1	2.62933165	3.58412142	9.79E-17	1.81E-15	10.48	14.24	140.38	61.24	SNORD3A	small nucleolar RNA, C/D box 3A	chr17	snoRNA
ENSG00000128346.9	1.06666669	-0.0794494	0.0090055	0.02376223	0.18	0.26	0.67	0.54	C22orf23	chromosome 22 open reading frame 23	chr22	protein_coding
ENSG00000180891.11	-1.4432349	5.66575313	7.50E-07	4.40E-06	14.43	14.08	7.23	6.73	CUEDC1	CUE domain containing 1	chr17	protein_coding
ENSG00000174173.6	1.65300212	1.98303475	4.38E-07	2.67E-06	0.69	0.97	2.11	4.87	TRMT10C	tRNA methyltransferase 10 homolog C (S. cerevisiae)	chr3	protein_coding
ENSG00000146411.5	1.6893395	-1.788206	0.01883218	0.04519557	0.02	0.01	0.06	0.1	SLC2A12	solute carrier family 2 (facilitated glucose transporter), member 12	chr6	protein_coding
ENSG00000119938.8	-8.1407153	2.12354401	1.02E-48	1.30E-46	2.92	2.66	0	0.02	PPP1R3C	protein phosphatase 1, regulatory subunit 3C	chr10	protein_coding
ENSG00000101335.8	-6.2882761	6.69107689	1.43E-66	4.91E-64	52.48	64.8	1.01	0.98	MYL9	myosin, light chain 9, regulatory	chr20	protein_coding
ENSG00000280391.1	-1.9137954	-1.5221406	0.00185375	0.00571918	0.03	0.06	0.02	0.01	RP11-46E17.	--	chr22	lincRNA
ENSG00000205078.5	-2.8167944	-0.3804242	1.28E-08	9.65E-08	1.25	1.19	0.41	0.06	SYCE1L	synaptonemal complex central element protein 1-like	chr16	protein_coding
ENSG00000186716.18	-1.0483709	7.4540336	0.00026464	0.0009747	29.06	24.94	17.98	16.8	BCR	breakpoint cluster region	chr22	protein_coding
ENSG00000143333.6	-2.0027969	1.22824754	1.27E-08	9.60E-08	0.98	1.51	0.4	0.43	RGS16	regulator of G-protein signaling 16	chr1	protein_coding
ENSG00000138646.7	2.4518346	1.67714172	3.20E-12	3.80E-11	0.22	0.21	1.05	2.1	HERC5	HECT and RLD domain containing E3 ubiquitin protein ligase 5	chr4	protein_coding
ENSG00000196227.9	2.96664555	1.95396893	6.52E-17	1.23E-15	0.13	0.13	1.2	1.55	FAM217B	family with sequence similarity 217, member B	chr20	protein_coding
ENSG00000107731.11	-1.2689527	4.0380501	1.82E-05	8.43E-05	2.53	2.96	1.72	1.3	UNC5B	unc-5 homolog B (C. elegans)	chr10	protein_coding
ENSG00000260371.1	2.00646864	-1.2443441	0.00055173	0.00191543	0.24	0.15	1.02	1.11	RP11-343C2	--	chr16	protein_coding
ENSG00000105640.11	-2.3665117	10.174647	2.14E-15	3.54E-14	827.2	714.54	220.3	177.11	RPL18A	ribosomal protein L18a	chr19	protein_coding
ENSG00000248360.6	4.28307915	-0.2693059	2.14E-13	2.87E-12	0.01	0.01	0.2	0.44	LINC00504	long intergenic non-protein coding RNA 504	chr4	lincRNA
ENSG00000069011.14	-2.563204	8.68326166	1.57E-17	3.13E-16	233.88	226.66	55.46	48.17	PITX1	paired-like homeodomain 1	chr5	protein_coding
ENSG00000112855.13	-1.3994123	6.21919204	1.47E-06	8.24E-06	37.97	32.41	17.76	17.81	HARS2	histidyl-tRNA synthetase 2, mitochondrial	chr5	protein_coding
ENSG00000162461.7	1.27162254	-0.4202255	0.00508517	0.01427016	0.1	0.11	0.24	0.45	SLC25A34	solute carrier family 25, member 34	chr1	protein_coding
ENSG00000233024.6	-2.0861204	1.90207682	3.64E-10	3.39E-09	2.22	3.8	0.93	0.95	RP11-1212A1	--	chr16	protein_coding
ENSG00000160013.7	-5.022479	-3.1298504	0.0100455	0.02582742	0.03	0.05	0	0	PTGIR	prostaglandin I2 (prostacyclin) receptor (IP)	chr19	protein_coding
ENSG00000113597.16	1.27290856	0.59767616	0.00071851	0.00243897	0.17	0.25	0.41	0.95	TRAPPC13	trafficking protein particle complex 13	chr5	protein_coding
ENSG00000107736.18	4.76039348	0.43543199	6.00E-19	1.34E-17	0.01	0	0.17	0.25	CDH23	cadherin-related 23	chr10	protein_coding
ENSG00000204116.10	1.01923989	-0.4284161	0.01799226	0.04368144	0.05	0.05	0.18	0.11	CHIC1	cysteine-rich hydrophobic domain 1	chrX	protein_coding
ENSG00000186468.11	-1.725763	9.57655668	3.36E-09	2.75E-08	251.17	233.89	84.31	111.88	RPS23	ribosomal protein S23	chr5	protein_coding
ENSG00000177822.6	-2.8768455	-1.8055032	6.90E-05	0.00028699	0.04	0.08	0.02	0	AC108142.1	--	chr4	antisense
ENSG00000089351.13	-1.3787366	6.45872717	2.03E-06	1.12E-05	34.92	27.39	17.23	14.67	GRAMD1A	GRAM domain containing 1A	chr19	protein_coding
ENSG00000136826.13	-1.4144062	3.25841315	2.83E-06	1.53E-05	3.05	4.64	1.69	2.14	KLF4	Kruppel-like factor 4 (gut)	chr9	protein_coding
ENSG00000266019.1	4.80587591	-0.1796695	2.32E-15	3.83E-14	0	1.06	31.23	10.21	MIR3609	microRNA 3609	chr7	miRNA
ENSG00000143819.11	1.42287247	8.61201395	8.91E-07	5.17E-06	78.26	88.98	321.62	274.12	EPHX1	epoxide hydrolase 1, microsomal (xenobiotic)	chr1	protein_coding
ENSG00000102908.19	4.02768275	3.02242396	1.21E-29	5.51E-28	0.04	0.07	1.19	1.19	NFAT5	nuclear factor of activated T-cells 5, tonicity-responsive	chr16	protein_coding
ENSG00000234476.1	-5.9972113	-2.568945	0.00014842	0.00057423	0.41	0.17	0	0	RP11-496N1	--	chr1	sense_intronic
ENSG00000237036.4	-1.3513643	0.3903604	0.00032488	0.00117608	0.4	0.37	0.1	0.3	ZEB1-AS1	ZEB1 antisense RNA 1	chr10	antisense
ENSG00000259797.1	2.42093072	-0.4240909	6.86E-07	4.06E-06	0	0.79	2.62	2.99	RP11-96D1.3	--	chr16	sense_intronic
ENSG00000255498.1	-1.9451912	-2.3907479	0.02049243	0.04852277	0.65	0.21	0.16	0.13	RP11-618K1	--	chr11	antisense
ENSG00000171621.12	-1.1446072	4.7154857	8.75E-05	0.00035487	9.76	7.76	5.41	5.16	SPSB1	splA/ryanodine receptor domain and SOCS box containing 1	chr1	protein_coding
ENSG00000162688.14	2.41962238	1.1162184	1.05E-10	1.05E-09	0.06	0.07	0.25	0.66	AGL	amylo-alpha-1, 6-glucosidase, 4-alpha-glucanotransferase	chr1	protein_coding
ENSG00000105550.7	-3.3331587	-1.4889998	1.03E-06	5.93E-06	0.29	0.43	0	0.08	FGF21	fibroblast growth factor 21	chr19	protein_coding
ENSG00000183186.6	1.69120083	0.86910028	4.13E-06	2.15E-05	0.14	0.29	0.84	1.01	C2CD4C	C2 calcium-dependent domain containing 4C	chr19	protein_coding
ENSG00000229587.2	5.08767213	-2.9577572	0.00538165	0.01488851	0	0	0.43	0	RP11-197P3.	--	chr9	sense_intronic
ENSG00000171368.11	-1.7096145	1.32496222	5.49E-07	3.30E-06	0.59	0.43	0.21	0.21	TPPP	tubulin polymerization promoting protein	chr5	protein_coding
ENSG00000005175.8	1.74902504	2.04435074	9.03E-08	6.08E-07	0.25	0.43	0.97	2.11	RPAP3	RNA polymerase II associated protein 3	chr12	protein_coding
ENSG00000168283.12	1.41802088	0.69171526	0.00011025	0.00043915	0.24	0.15	0.56	0.84	BMI1	BMI1 proto-oncogene, polycomb ring finger	chr10	protein_coding
ENSG00000134259.3	-5.3884183	0.63011801	2.24E-24	7.40E-23	2.09	2.57	0.09	0.05	NGF	nerve growth factor (beta polypeptide)	chr1	protein_coding
ENSG00000101144.11	8.50787763	4.02742382	5.86E-68	2.10E-65	0.03	0	8.73	7.74	BMP7	bone morphogenetic protein 7	chr20	protein_coding
ENSG00000172986.11	1.72956316	0.73628554	2.81E-06	1.51E-05	0.27	0.09	0.62	0.99	GXYLT2	glucoside xylosyltransferase 2	chr3	protein_coding
ENSG00000265752.2	5.44044058	-2.7547732	0.00290522	0.00856312	0	0	0	0.31	RP11-403A2	--	chr18	lincRNA
ENSG00000002746.13	-3.5211051	2.00202679	4.51E-22	1.27E-20	0.61	0.64	0.1	0.05	HECW1	HECT, C2 and WW domain containing E3 ubiquitin protein ligase 1	chr7	protein_coding
ENSG00000163071.9	5.13409619	-1.1289131	2.17E-09	1.82E-08	0.01	0	0.13	0.22	SPATA18	spermatogenesis associated 18	chr4	protein_coding
ENSG00000104356.9	-1.1681789	3.37694618	9.37E-05	0.00037857	2.33	2.57	1.34	1.56	POP1	processing of precursor 1, ribonuclease P/MRP subunit (S. cerevisiae)	chr8	protein_coding
ENSG00000091592.14	-4.9099254	-0.4788199	2.86E-15	4.68E-14	0.14	0.21	0.02	0	NLRP1	NLR family, pyrin domain containing 1	chr17	protein_coding
ENSG00000188352.11	-1.570855	3.9055858	1.50E-07	9.80E-07	3.12	2.79	1.36	1.29	FOCAD	focadhesin	chr9	protein_coding
ENSG00000106853.15	-1.9341531	7.37696062	4.99E-11	5.17E-10	124.54	150.27	45.66	50.17	PTGR1	prostaglandin reductase 1	chr9	protein_coding
ENSG00000083544.12	1.22071776	0.58524894	0.00101375	0.00332853	0.23	0.21	0.47	0.87	TDRD3	tudor domain containing 3	chr13	protein_coding
ENSG00000133101.8	-7.9336702	-1.0314215	1.65E-13	2.25E-12	0.23	0.39	0	0	CCNA1	cyclin A1	chr13	protein_coding
ENSG00000128596.15	3.1531154	3.31034589	1.13E-21	3.10E-20	0.29	0.46	4.76	4.06	CCDC136	coiled-coil domain containing 136	chr7	protein_coding
ENSG00000264812.1	1.68167454	-1.6223789	0.00945988	0.02474059	0.16	0.13	0.99	0.28	RP13-20L14.	--	chr17	sense_intronic
ENSG00000274922.1	1.81146425	-1.9112562	0.01967235	0.04677036	0.04	0	0.04	0.17	RP11-88E10.	--	chr13	lincRNA
ENSG00000148943.10	1.80684561	0.95766272	7.26E-07	4.27E-06	0.07	0.21	0.59	0.74	LIN7C	lin-7 homolog C (C. elegans)	chr11	protein_coding
ENSG00000172081.12	-1.1862228	5.89002111	4.20E-05	0.00018254	20.29	18.56	12.17	10.54	MOB3A	MOB kinase activator 3A	chr19	protein_coding
ENSG00000088451.9	1.31737242	-0.080351	0.00184437	0.0056998	0.17	0.25	0.32	1.07	TGDS	TDP-glucose 4,6-dehydratase	chr13	protein_coding
ENSG00000105289.13	4.99399776	2.72835953	1.02E-36	6.41E-35	0.05	0.13	4	3.6	TJP3	tight junction protein 3	chr19	protein_coding
ENSG00000174748.17	-1.1942575	10.3975713	3.31E-05	0.00014624	309.15	272.29	162.33	176.79	RPL15	ribosomal protein L15	chr3	protein_coding
ENSG00000115461.4	-2.7548519	-0.2554343	4.30E-09	3.48E-08	0.19	0.17	0.05	0.03	IGFBP5	insulin-like growth factor binding protein 5	chr2	protein_coding
ENSG00000104892.15	-8.709334	1.85844319	4.03E-45	4.20E-43	2.59	3.57	0	0.01	KLC3	kinesin light chain 3	chr19	protein_coding
ENSG00000022267.15	-2.2796163	4.15266354	8.56E-14	1.20E-12	5.25	5.72	1.26	1.75	FHL1	four and a half LIM domains 1	chrX	protein_coding
ENSG00000116663.9	3.68305123	1.96101725	1.40E-22	4.06E-21	0.39	0.19	5.37	4.57	FBXO6	F-box protein 6	chr1	protein_coding
ENSG00000272746.1	4.87917439	-3.0678699	0.01889873	0.04519557	0	0	0	0.36	RP11-53B2.6	--	chr18	sense_intronic

ENSG00000152240.11	1.28251252	1.63102906	0.00011155	0.00044381	1.31	1.21	2.48	5.74	HAUS1	HAUS augmin-like complex, subunit 1	chr18	protein_coding
ENSG00000131400.6	7.82070658	-0.954074	1.02E-11	1.15E-10	0	0	0.33	0.84	NAPSA	napsin A aspartic peptidase	chr19	protein_coding
ENSG00000123159.14	-1.0592379	7.6431283	0.00022799	0.00085384	121.75	102.94	72.51	71.22	GIPC1	GIPC PDZ domain containing family, member 1	chr19	protein_coding
ENSG00000166448.13	-3.6628925	-0.1637023	7.00E-13	8.92E-12	0.35	0.27	0.05	0.01	TMEM130	transmembrane protein 130	chr7	protein_coding
ENSG00000131127.12	8.3750456	-0.4652987	8.03E-16	1.37E-14	0	0	0.18	0.38	ZNF141	zinc finger protein 141	chr4	protein_coding
ENSG00000228791.6	1.88629448	-1.8601781	0.00967167	0.02519005	0	0.1	0.2	0.28	THRB-AS1	THRB antisense RNA 1	chr3	processed_transcript
ENSG00000227744.4	-5.5197117	-2.8643549	0.00158008	0.00495521	0.07	0.01	0	0	FLJ43879	--	chr2	lincRNA
ENSG00000188660.3	-2.5161943	-1.8098737	0.00034876	0.00125664	0.08	0.11	0.03	0.02	LINC00319	long intergenic non-protein coding RNA 319	chr21	lincRNA
ENSG00000204084.11	-1.4969259	4.81951179	3.51E-07	2.18E-06	4.04	4.58	2.05	2.02	INPP5B	inositol polyphosphate-5-phosphatase, 75kDa	chr1	protein_coding
ENSG00000043591.5	-4.9032476	1.10844302	1.80E-26	6.85E-25	1	1.37	0.04	0.06	ADRB1	adrenoceptor beta 1	chr10	protein_coding
ENSG00000178229.7	1.0894835	1.71221813	0.00090499	0.00300184	0.45	0.45	1.17	1.39	ZNF543	zinc finger protein 543	chr19	protein_coding
ENSG00000152495.9	-2.3473778	-0.8809982	6.55E-06	3.29E-05	0.09	0.02	0.01	0.02	CAMK4	calcium/calmodulin-dependent protein kinase IV	chr5	protein_coding
ENSG00000122483.16	4.30526925	0.93998109	4.22E-21	1.11E-19	0.02	0.03	0.49	0.87	CCDC18	coiled-coil domain containing 18	chr1	protein_coding
ENSG00000236544.1	5.2326735	-2.8777865	0.00290522	0.00856312	0	0	0.54	0.05	AC008060.8	--	chr7	lincRNA
ENSG00000214922.8	3.75034661	0.56751084	6.70E-16	1.15E-14	0.02	0.09	1.06	0.89	HLA-F-AS1	HLA-F antisense RNA 1	chr6	processed_transcript
ENSG00000175161.12	-5.7861603	-2.7054402	0.00047761	0.00167191	0	0.04	0	0	CADM2	cell adhesion molecule 2	chr3	protein_coding
ENSG00000171208.8	1.20228663	3.95149745	4.96E-05	0.00021199	1.1	0.92	2.79	3.41	NETO2	neuropilin (NRP) and tolloid (TLL)-like 2	chr16	protein_coding
ENSG00000177380.12	-1.3592304	5.34446657	3.15E-06	1.68E-05	10.4	9.56	5.45	4.91	PPFIA3	protein tyrosine phosphatase, receptor type, f polypeptide (PTPRF), interacting protein (liprin), alpha 3	chr19	protein_coding
ENSG00000270504.1	3.16330997	5.21794306	7.87E-24	2.50E-22	2.15	2.22	24.12	28.13	RP11-420L9.	--	chr6	antisense
ENSG00000163947.10	1.93484306	1.59678129	1.81E-08	1.33E-07	0.13	0.23	0.82	1.05	ARHGEF3	Rho guanine nucleotide exchange factor (GEF) 3	chr3	protein_coding
ENSG00000087299.10	1.79022842	2.64227322	1.48E-08	1.11E-07	0.21	0.45	1.17	1.9	L2HGDH	L-2-hydroxyglutarate dehydrogenase	chr14	protein_coding
ENSG00000033327.11	-3.5196335	4.24307779	2.69E-27	1.09E-25	4.7	4.38	0.52	0.54	GAB2	GRB2-associated binding protein 2	chr11	protein_coding
ENSG00000225611.1	5.80179215	-2.5306812	0.00026535	0.0009747	0	0	0.39	0.12	RP11-70C1.1	--	chr3	antisense
ENSG00000223764.2	5.30546885	4.96337402	1.03E-49	1.39E-47	0.94	0.84	47.11	46.99	RP11-54O7.	--	chr1	lincRNA
ENSG00000235244.3	2.45372387	-2.0329686	0.00408095	0.01167544	0.04	0	0.09	0.21	RP11-761E2	--	chrX	lincRNA
ENSG00000086289.10	-12.54975	3.49902168	1.27E-68	4.70E-66	5.41	7.42	0	0	EPDR1	ependymin related 1	chr7	protein_coding
ENSG00000279175.1	1.0439748	0.22773787	0.00718671	0.0193486	0.05	0.04	0.12	0.12	CTA-414D7.	--	chr22	sense_intronic
ENSG00000065057.6	-1.2047397	5.29189607	3.41E-05	0.00015036	44.82	37.98	24.86	23	NTHL1	nth endonuclease III-like 1 (E. coli)	chr16	protein_coding
ENSG00000261116.1	6.12830027	-2.3082274	2.70E-05	0.00012094	0	0	0.18	0.11	RP3-523K23.	--	chr6	sense_overlapping
ENSG00000272078.1	5.47921296	-2.7333202	0.00158008	0.00495521	0	0	0.03	0.01	RP4-734G22	--	chr1	antisense
ENSG00000133731.8	2.24185769	0.39750678	5.00E-08	3.49E-07	0.19	0.05	0.37	1.14	IMPA1	inositol(myo)-1(or 4)-monophosphatase 1	chr8	protein_coding
ENSG00000215021.7	-1.4573262	9.24289916	4.88E-07	2.96E-06	470.83	476.75	240.24	218.88	PHB2	prohibitin 2	chr12	protein_coding
ENSG00000196705.7	2.26636556	1.52646599	1.28E-10	1.26E-09	0.05	0.06	0.27	0.45	ZNF431	zinc finger protein 431	chr19	protein_coding
ENSG00000246982.5	2.60360575	-2.3285497	0.00455824	0.01291303	0.02	0.02	0	0.32	RP1-179N16	--	chr6	antisense
ENSG00000172365.2	-5.3787292	-2.9446861	0.00290522	0.00856312	0	0.24	0	0	OR5B2	olfactory receptor, family 5, subfamily B, member 2	chr11	protein_coding
ENSG00000176444.17	-1.1095072	5.63462616	0.00012663	0.00049911	24.46	26.29	16.13	15.16	CLK2	CDC-like kinase 2	chr1	protein_coding
ENSG00000279035.1	1.74355219	-0.4082057	0.00020204	0.00076235	0.07	0.16	0.3	0.73	RP11-649A1	--	chr17	TEC
ENSG00000244733.5	4.57919841	-1.5776414	1.10E-06	6.29E-06	0	0.02	0.14	0.43	RP11-506M1	--	chr10	lincRNA
ENSG00000154537.4	-3.8567099	3.32698537	1.68E-29	7.57E-28	20.02	29.49	2.24	2.3	FAM27C	family with sequence similarity 27, member C	chr9	protein_coding
ENSG00000205325.1	-6.938121	-1.8814296	8.18E-08	5.55E-07	0.1	0.15	0	0	AC005863.1	--	chr17	lincRNA
ENSG00000101187.14	-3.8236134	6.60753315	7.37E-33	3.91E-31	62.81	43.26	5.19	4.8	SLCO4A1	solute carrier organic anion transporter family, member 4A1	chr20	protein_coding
ENSG00000160888.6	-1.1444013	7.59050093	7.04E-05	0.00029231	75.6	64.16	42.91	41.35	IER2	immediate early response 2	chr19	protein_coding
ENSG00000123607.13	2.87387084	0.4032903	2.99E-11	3.18E-10	0.01	0.08	0.28	0.57	TTC21B	tetratricopeptide repeat domain 21B	chr2	protein_coding
ENSG00000280388.1	2.82813918	0.57662372	2.01E-11	2.18E-10	0.08	0.2	1.34	1.32	RP11-229D1	--	chr7	TEC
ENSG00000124214.18	1.25879088	6.8525806	1.34E-05	6.35E-05	12.52	16.34	41.81	50.28	STAU1	staufen double-stranded RNA binding protein 1	chr20	protein_coding
ENSG00000155530.2	5.1969984	-0.3067412	5.61E-15	8.96E-14	0	0.02	0.3	0.64	LRGUK	leucine-rich repeats and guanylate kinase domain containing	chr7	protein_coding
ENSG00000196998.14	-1.652099	6.16689049	1.66E-08	1.23E-07	42.72	41.95	18.41	17.46	WDR45	WD repeat domain 45	chrX	protein_coding
ENSG00000134339.7	-4.1024276	-0.8608447	4.07E-11	4.25E-10	0.24	0.48	0	0.05	SAA2	serum amyloid A2	chr11	protein_coding
ENSG00000082269.15	1.52128192	-0.7989621	0.00216733	0.00660335	0	0.06	0.13	0.09	FAM135A	family with sequence similarity 135, member A	chr6	protein_coding
ENSG00000255571.5	10.4978417	1.51115564	2.59E-38	1.79E-36	0	0	1.37	1.09	LINC00925	long intergenic non-protein coding RNA 925	chr15	lincRNA
ENSG00000167508.9	1.28222016	5.85410784	1.01E-05	4.87E-05	16.14	13.73	46.25	50.73	MVD	mevalonate (diphospho) decarboxylase	chr16	protein_coding
ENSG00000250714.3	-2.882006	-2.1206777	0.0002605	0.00096399	0.19	0.28	0	0.07	RP11-1149O	--	chr8	antisense
ENSG00000272808.2	-4.0327725	2.07880066	6.76E-27	2.65E-25	1.55	1	0.07	0.13	RP11-66B24.	--	chr15	processed_transcript
ENSG00000108669.15	-2.3161605	6.22137144	9.79E-15	1.53E-13	27.64	23.23	6.48	7.15	CYTH1	cytohesin 1	chr17	protein_coding
ENSG00000113790.9	2.41173568	-0.2376742	2.11E-07	1.35E-06	0.07	0.02	0.21	0.41	EHHADH	enoyl-CoA, hydratase/3-hydroxyacyl CoA dehydrogenase	chr3	protein_coding
ENSG00000179922.5	1.0567628	3.61105187	0.00037311	0.00133735	2.92	3.28	9.68	7.45	ZNF784	zinc finger protein 784	chr19	protein_coding
ENSG00000172878.12	-1.7449158	2.25694799	4.51E-08	3.16E-07	1.6	1.87	0.6	0.78	METAP1D	methionyl aminopeptidase type 1D (mitochondrial)	chr2	protein_coding
ENSG00000196436.8	2.75037	0.43709522	7.78E-11	7.88E-10	0.24	0.12	1.62	1.7	NPIPB15	nuclear pore complex interacting protein family, member B15	chr16	protein_coding
ENSG00000182218.8	1.26686967	2.40672258	5.40E-05	0.00022876	0.32	0.31	1.01	1.01	HHIPL1	HHIP-like 1	chr14	protein_coding
ENSG00000205683.10	5.97918319	-2.4123307	8.36E-05	0.00034052	0	0	0.08	0.03	DPF3	D4, zinc and double PHD fingers, family 3	chr14	protein_coding
ENSG00000280184.1	2.17172149	-1.9226925	0.00445943	0.01266454	0.05	0	0.26	0.08	AL023806.1	--	chr6	protein_coding
ENSG00000269054.1	2.46521223	-2.4147514	0.00737015	0.01977584	0	0.07	0.32	0.24	CTD-2619J1	--	chr19	antisense
ENSG00000198088.9	-1.1661783	1.74474446	0.00030075	0.00109586	1.95	2.29	0.84	1.68	NUP62CL	nucleoporin 62kDa C-terminal like	chrX	protein_coding
ENSG00000183775.9	-4.5037703	-2.0151289	6.92E-06	3.47E-05	0.02	0.03	0	0	KCTD16	potassium channel tetramerization domain containing 16	chr5	protein_coding
ENSG00000280130.1	2.31605591	-1.1847925	6.85E-05	0.00028517	0.28	0.22	2.19	1.29	AC073333.1	--	chr7	protein_coding
ENSG00000105639.17	-2.8596876	-2.1159158	0.00094481	0.00312013	0.07	0.02	0.02	0	JAK3	Janus kinase 3	chr19	protein_coding
ENSG00000171303.6	-2.8584356	3.27475538	7.02E-19	1.56E-17	2.36	2.06	0.43	0.39	KCNK3	potassium channel, two pore domain subfamily K, member 3	chr2	protein_coding
ENSG00000225556.1	5.68288014	-2.6056968	0.00047761	0.00167191	0	0	0.11	0.12	C2CD4D	C2 calcium-dependent domain containing 4D	chr1	protein_coding
ENSG00000124203.5	6.91320579	-1.7189016	4.98E-08	3.47E-07	0	0	0.06	0.05	ZNF831	zinc finger protein 831	chr20	protein_coding
ENSG00000262526.2	-1.2137621	1.33816693	0.00029551	0.00107806	3.55	3.73	2.11	2.07	CTD-2545G1	--	chr17	protein_coding
ENSG00000242372.5	-1.2587423	6.1840924	1.39E-05	6.58E-05	54.86	46.47	29.04	27.38	EIF6	eukaryotic translation initiation factor 6	chr20	protein_coding
ENSG00000183072.9	2.7995909	2.04129108	8.56E-16	1.46E-14	0.37	0.44	4.21	3.33	NKX2-5	NK2 homeobox 5	chr5	protein_coding
ENSG00000174197.15	2.10011164	3.94425302	6.27E-12	7.22E-11	0.31	0.31	1.62	1.91	MGA	MGA, MAX dimerization protein	chr15	protein_coding
ENSG00000250786.1	-8.4940882	-0.5128497	1.26E-17	2.52E-16	0.65	0.57	0	0	SNHG18	small nucleolar RNA host gene 18	chr5	lincRNA
ENSG00000183889.11	-1.4536947	-0.9162945	0.00276761	0.00827245	0.5	0.21	0.14	0.2	AC138969.4	--	chr16	protein_coding
ENSG00000168769.11	2.97363844	0.12856188	1.63E-10	1.58E-09	0.01	0.02	0.07	0.18	TET2	tet methylcytosine dioxygenase 2	chr4	protein_coding
ENSG00000128881.15	1.78218599	1.494506	1.90E-07	1.23E-06	0.08	0.07	0.27	0.45	TTBK2	tau tubulin kinase 2	chr15	protein_coding
ENSG00000162552.13	3.04211646	0.07269092	6.43E-11	6.58E-10	0.04	0.04	0.44	0.52	WNT4	wingless-type MMTV integration site family, member 4	chr1	protein_coding
ENSG00000170909.12	-2.5753123	0.90138968	6.83E-12	7.81E-11	1.42	1.03	0.25	0.3	OSCAR	osteoclast associated, immunoglobulin-like receptor	chr19	protein_coding
ENSG00000268568.1	1.36628608	0.59671932	0.00027549	0.0010101	0.66	0.3	1.25	2.09	AC007228.9	--	chr19	lincRNA
ENSG00000171388.11	-1.7193691	2.63158249	3.65E-08	2.58E-07	2.49	2.3	0.96	0.98	APLN	apelin	chrX	protein_coding
ENSG00000272902.2	1.45011188	-0.3579854	0.00114043	0.00370189	0.11	0.14	0.4	0.49	RP11-299H2	--	chr2	lincRNA
ENSG00000111700.11	-5.9955597	-2.569758	0.00014842	0.00057423	0.11	0.02	0	0	SLCO1B3	solute carrier organic anion transporter family, member 1B3	chr12	protein_coding
ENSG00000144589.19	-2.2539106	5.02999202	7.90E-14	1.12E-12	12.13	12.06	3.56	3.19	STK11IP	serine/threonine kinase 11 interacting protein	chr2	protein_coding
ENSG00000275793.1	3.26632317	1.64137967	5.40E-18	1.11E-16	0.04	0.12	1.03	0.9	RIMBP3	RIMS binding protein 3	chr22	protein_coding
ENSG00000176125.4	-1.1192968	1.17203315	0.00101657	0.00333525	2.79	2.13	1.38	1.64	UFSP1	UFM1-specific peptidase 1 (non-functional)	chr7	protein_coding
ENSG00000173145.10	2.42859003	1.80773666	2.63E-12	3.15E-11	0.15	0.35	1.04	2.55	NOC3L	nucleolar complex associated 3 homolog (S. cerevisiae)	chr10	protein_coding
ENSG00000231871.4	2.30116952	-1.0337522	3.23E-05	0.00014288	0.13	0.04	0.63	0.53	IPO9-AS1	IPO9 antisense RNA 1	chr1	antisense
ENSG00000138398.14	3.56240465	3.97953061	3.92E-27	1.57E-25	0.24	0.36	2.99	6.39	PPIG	peptidylprolyl isomerase G (cyclophilin G)	chr2	protein_coding
ENSG00000256633.1	-3.6229328	-0.3879979	3.12E-12	3.71E-11	2.26	1.07	0.05	0.29	RP11-169D4	--	chr11	antisense
ENSG00000158301.17	1.81700086	1.80599008	5.90E-08	4.09E-07	0.25	0.37	1.1	1.81	GPRASP2	G protein-coupled receptor associated sorting protein 2	chrX	protein_coding
ENSG00000133048.11	-11.020937	1.9683302	1.25E-46	1.44E-44	3.21	3.89	0	0	CHI3L1	chitinase 3-like 1 (cartilage glycoprotein-39)	chr1	protein_coding
ENSG00000137040.9	1.64790705	0.94024668	5.92E-06	3.00E-05	0.19	0.12	0.48	0.83	RANBP6	RAN binding protein 6	chr9	protein_coding
ENSG00000257557.2	4.33037422	-1.0606534	4.33E-09	3.50E-08	0	0.02	0.3	0.21	RP11-84G21	--	chr12	antisense
ENSG00000230124.5	-1.0444641	6.56162382	0.00028638	0.00104666	62.46	60.85	38.63	41.09	LHX4-AS1	LHX4 antisense RNA 1	chr1	protein_coding
ENSG00000105483.15	1.45357716	2.64175875	3.25E-06	1.73E-05	0.39	0.47	1.34	1.81	CARD8	caspase recruitment domain family, member 8	chr19	protein_coding
ENSG00000259954.1	6.83015404	-1.7774394	1.35E-07	8.87E-07	0	0	0.07	0.27	IL21R-AS1	IL21R antisense RNA 1	chr16	antisense
ENSG00000132639.11	-6.5052437	-2.2134303	3.03E-06	1.62E-05	0.16	0.09	0	0	SNAP25	synaptosomal-associated protein, 25kDa	chr20	protein_coding
ENSG00000182359.13	2.19722782	-0.7064474	2.15E-05	9.80E-05	0.01	0.05	0.14	0.24	KBTBD3	kelch repeat and BTB (POZ) domain containing 3	chr11	protein_coding
ENSG00000143498.16	1.94894767	0.9743225	1.06E-07	7.08E-07	0.26	0.29	0.77	2.04	TAF1A	TATA box binding protein (TBP)-associated factor, RNA polymerase I, A, 48kDa	chr1	protein_coding
ENSG00000104723.19	-6.5679868	3.10225414	6.59E-52	9.94E-50	3.39	3.51	0.03	0.06	TUSC3	tumor suppressor candidate 3	chr8	protein_coding
ENSG00000261693.1	6.60206048	-1.9568745	1.05E-06	6.02E-06	0	0	0.08	0.15	RP13-467H1	--	chr8	antisense
ENSG00000143190.20	1.80076032	2.68279823	1.14E-08	8.64E-08	0.11	0.21	0.75	0.73	POU2F1	POU class 2 homeobox 1	chr1	protein_coding
ENSG00000197837.3	1.42865283	-1.2538107	0.01363595	0.03404656	0.37	0.15	1.17	0.76	HIST4H4	histone cluster 4, H4	chr12	protein_coding
ENSG00000064655.17	-4.8534979	1.73358497	8.75E-31	4.18E-29	1.7	2.16	0.11	0.07	EYA2	EYA transcriptional coactivator and phosphatase 2	chr20	protein_coding
ENSG00000247157.5	2.53003393	-1.9815841	0.00270895	0.00811738	0.01	0.02	0.09	0.1	LINC01252	long intergenic non-protein coding RNA 1252	chr12	lincRNA
ENSG00000271605.4	3.32174663	0.60797237	2.97E-14	4.39E-13	0.14	0.13	1.53	2.12	MILR1	mast cell immunoglobulin-like receptor 1	chr17	protein_coding
ENSG00000125744.10	-2.0159854	4.92141718	1.62E-11	1.78E-10	19.07	14.1	6.01	4.91	RTN2	reticulon 2	chr19	protein_coding
ENSG00000115598.8	2.04790261	-0.3444028	1.50E-05	7.09E-05	0.08	0.09	0.5	0.44	IL1RL2	interleukin 1 receptor-like 2	chr2	protein_coding
ENSG00000168071.20	-5.151209	2.66889713	1.93E-38	1.35E-36	2.13	1.77	0.1	0.05	CCDC88B	coiled-coil domain containing 88B	chr11	protein_coding
ENSG00000101276.13	6.9600295	2.2097272	3.25E-42	2.77E-40	0.02	0.02	3.46	2.82	SLC52A3	solute carrier family 52 (riboflavin transporter), member 3	chr20	protein_coding
ENSG00000070159.12	1.43238258	2.34890916	5.54E-06	2.82E-05	0.23	0.23	0.81	0.87	PTPN3	protein tyrosine phosphatase, non-receptor type 3	chr9	protein_coding
ENSG00000169239.11	-1.154777	2.09545636	0.00024018	0.00089668	0.76	0.63	0.36	0.47	CA5B	carbonic anhydrase VB, mitochondrial	chrX	protein_coding
ENSG00000278970.1	-1.2864466	4.84700558	1.08E-05	5.19E-05	19.54	20.45	11.03	10.8	HEIH	hepatocellular carcinoma up-regulated EZH2-associated long non-coding RNA	chr5	TEC
ENSG00000125977.6	1.38652548	6.26484052	1.83E-06	1.01E-05	11.78	14.15	35.58	55.18	EIF2S2	eukaryotic translation initiation factor 2, subunit 2 beta, 38kDa	chr20	protein_coding
ENSG00000005238.18	-1.8784321	3.22428089	1.12E-09	9.70E-09	1.94	1.91	0.86	0.53	FAM214B	family with sequence similarity 214, member B	chr9	protein_coding
ENSG00000261713.5	6.1151206	-2.3157799	4.74E-05	0.00020301	0	0	0.09	0.1	SSTR5-AS1	SSTR5 antisense RNA 1	chr16	processed_transcript
ENSG00000276672.1	7.13576376	-1.5365574	4.48E-09	3.59E-08	0	0	0.1	0.14	RP11-142E9.	--	chr13	sense_intronic
ENSG00000146666.5	-4.2218429	0.73484263	1.22E-20	3.07E-19	2.27	2	0.22	0.09	LINC00525	long intergenic non-protein coding RNA 525	chr7	antisense
ENSG00000101974.13	1.37003347	0.07857257	0.00070308	0.002391	0.08	0.04	0.19	0.23	ATP11C	ATPase, class VI, type 11C	chrX	protein_coding
ENSG00000184371.12	-3.3741112	7.10350534	2.86E-27	1.16E-25	37.96	45.17	5.48	5.19	CSF1	colony stimulating factor 1 (macrophage)	chr1	protein_coding
ENSG00000198774.4	-6.1863178	-2.4412039	4.74E-05	0.00020301	0.06	0.02	0	0	RASSF9	Ras association (RalGDS/AF-6) domain family (N-terminal) member 9	chr12	protein_coding
ENSG00000255224.1	1.73769799	0.98281292	1.63E-06	9.08E-06	0.53	0.58	2.31	2.65	CTD-3065J1	--	chr8	antisense
ENSG00000138095.17	1.70154235	4.38579244	1.18E-08	8.90E-08	1.26	1.12	3.75	6.65	LRPPRC	leucine-rich pentatricopeptide repeat containing	chr2	protein_coding
ENSG00000261504.1	-2.9631034	-2.4500262	0.00112121	0.00364442	0.27	0	0	0.04	RP11-317P1	--	chr1	lincRNA
ENSG00000102030.14	-1.9150466	7.42609528	7.49E-11	7.63E-10	121.64	115.48	42.51	41.24	NAA10	N(alpha)-acetyltransferase 10, NatA catalytic subunit	chrX	protein_coding
ENSG00000272913.1	2.24648333	-0.3700136	1.79E-06	9.89E-06	0.2	0.04	0.72	0.82	RP11-440D1	--	chr2	lincRNA
ENSG00000206344.6	1.64650073	-0.0530437	8.92E-05	0.00036127	0.23	0.16	0.73	0.95	HCG27	HLA complex group 27 (non-protein coding)	chr6	protein_coding
ENSG00000275185.1	3.57447335	-1.6634645	1.91E-05	8.80E-05	0	0.1	1.42	0.4	RP13-753N3	--	chr17	antisense
ENSG00000261766.1	-1.6201705	1.17137133	3.06E-06	1.63E-05	4.33	5.68	2.53	1.8	RP11-22P6.2	--	chr16	antisense
ENSG00000110330.7	3.12958482	3.06043539	6.23E-21	1.61E-19	0.33	0.31	2.96	4.56	BIRC2	baculoviral IAP repeat containing 2	chr11	protein_coding
ENSG00000233611.3	-1.107007	-0.4567409	0.01142438	0.02905424	0.72	0.64	0.32	0.51	AC079135.1	--	chr2	antisense
ENSG00000198780.10	2.50443773	0.17089982	9.14E-09	7.04E-08	0.04	0.04	0.21	0.42	FAM169A	family with sequence similarity 169, member A	chr5	protein_coding
ENSG00000247844.1	-10.731457	1.67889014	1.07E-42	9.46E-41	2.15	2.74	0	0	CCAT1	colon cancer associated transcript 1 (non-protein coding)	chr8	lincRNA
ENSG00000196646.10	1.45907297	-1.3475397	0.01151564	0.0292584	0.06	0.02	0.11	0.16	ZNF136	zinc finger protein 136	chr19	protein_coding
ENSG00000163491.15	4.89980841	-3.0567113	0.01889873	0.04519557	0	0	0.02	0.02	NEK10	NIMA-related kinase 10	chr3	protein_coding
ENSG00000163126.13	1.95081406	2.23783526	2.33E-09	1.94E-08	0.39	0.8	3.32	2.77	ANKRD23	ankyrin repeat domain 23	chr2	protein_coding
ENSG00000122912.13	1.31374589	0.68187281	0.00036544	0.00131324	0.07	0.15	0.27	0.44	SLC25A16	solute carrier family 25 (mitochondrial carrier), member 16	chr10	protein_coding
ENSG00000128203.6	1.31437094	1.78015879	6.00E-05	0.00025253	0.53	0.4	1.39	1.68	ASPHD2	aspartate beta-hydroxylase domain containing 2	chr22	protein_coding
ENSG00000174106.2	1.9775763	2.59630238	5.88E-10	5.29E-09	0.33	0.49	1.74	2.57	LEMD3	LEM domain containing 3	chr12	protein_coding
ENSG00000175879.8	1.11207481	-0.056384	0.0067217	0.01821738	0.28	0.05	0.32	0.67	HOXD8	homeobox D8	chr2	protein_coding
ENSG00000136643.10	1.28332691	0.94628022	0.00029528	0.00107739	0.15	0.15	0.39	0.6	RPS6KC1	ribosomal protein S6 kinase, 52kDa, polypeptide 1	chr1	protein_coding
ENSG00000095015.5	2.21133691	0.52299802	2.84E-08	2.04E-07	0.08	0.03	0.29	0.39	MAP3K1	mitogen-activated protein kinase kinase kinase 1, E3 ubiquitin protein ligase	chr5	protein_coding
ENSG00000274997.1	1.32771904	-0.7717027	0.0077156	0.02061505	0.14	1.1	2.65	1.48	HIST1H2AH	histone cluster 1, H2ah	chr6	protein_coding
ENSG00000158882.11	-1.313217	4.74674075	7.25E-06	3.62E-05	10.75	10.17	5.52	5.7	TOMM40L	translocase of outer mitochondrial membrane 40 homolog (yeast)-like	chr1	protein_coding
ENSG00000275549.1	1.9108859	-1.466562	0.00257451	0.0077416	0.06	0.05	0.21	0.37	C9orf173-AS	C9orf173 antisense RNA 1	chr9	antisense
ENSG00000178919.8	1.12633159	3.71904559	0.00015296	0.00059006	2.05	1.79	5.23	5.97	FOXE1	forkhead box E1	chr9	protein_coding
ENSG00000177432.6	-7.0885619	-1.7598293	1.16E-08	8.76E-08	0.15	0.21	0	0	NAP1L5	nucleosome assembly protein 1-like 5	chr4	protein_coding
ENSG00000277203.1	-1.0586533	3.370502	0.00040208	0.00143131	5.42	7.78	4.99	3.4	F8A1	coagulation factor VIII-associated 1	chrX	protein_coding
ENSG00000089050.13	1.67282157	1.01475412	3.11E-06	1.66E-05	0.19	0.19	0.52	1.12	RBBP9	retinoblastoma binding protein 9	chr20	protein_coding
ENSG00000148334.13	-1.4206939	7.68452823	9.45E-07	5.46E-06	109.88	90	50.37	49.16	PTGES2	prostaglandin E synthase 2	chr9	protein_coding
ENSG00000205810.7	-2.6134178	-2.639617	0.01923175	0.0458365	0.19	0.07	0.06	0	KLRC3	killer cell lectin-like receptor subfamily C, member 3	chr12	protein_coding
ENSG00000264207.1	1.16444538	1.90783065	0.0003001	0.00109366	1.5	1.47	4.15	4.72	RP11-196G1	--	chr1	antisense
ENSG00000119231.9	1.17045543	2.66891722	0.00014889	0.00057587	0.5	0.49	1.52	1.45	SENP5	SUMO1/sentrin specific peptidase 5	chr3	protein_coding
ENSG00000214265.10	-5.2091082	-3.0354822	0.00538165	0.01488851	0.08	0.09	0	0	SNURF	SNRPN upstream reading frame	chr15	protein_coding
ENSG00000144736.12	1.07685106	3.5862576	0.00030584	0.00111135	2.02	2.08	5.19	6.36	SHQ1	SHQ1, H/ACA ribonucleoprotein assembly factor	chr3	protein_coding
ENSG00000256433.2	-2.0892415	1.27710091	1.85E-09	1.56E-08	1.6	1.9	0.41	0.68	RP1-102E24.	--	chr12	lincRNA
ENSG00000061337.14	3.13022729	-2.5963925	0.00552196	0.01522223	0	0	0.04	0.02	LZTS1	leucine zipper, putative tumor suppressor 1	chr8	protein_coding
ENSG00000229953.1	-5.9537289	0.88749895	1.95E-28	8.30E-27	4.06	3.7	0.17	0	RP11-284F2	--	chr1	antisense
ENSG00000269918.1	7.85888165	-0.9331486	4.78E-12	5.56E-11	0	0	0.6	0.34	AF131215.9	--	chr8	sense_intronic
ENSG00000244242.1	2.5868836	2.30816429	1.55E-14	2.36E-13	0.25	0.35	2.57	2.26	IFITM10	interferon induced transmembrane protein 10	chr11	protein_coding
ENSG00000108395.12	2.65097152	3.33954743	1.27E-16	2.33E-15	0.37	0.54	2.77	4.87	TRIM37	tripartite motif containing 37	chr17	protein_coding
ENSG00000179133.10	4.98196296	-0.4995511	2.91E-13	3.84E-12	0.02	0	0.32	0.59	C10orf67	chromosome 10 open reading frame 67	chr10	protein_coding
ENSG00000280053.1	5.04680446	-2.9799228	0.0100455	0.02582742	0	0	0.01	0.06	RP11-61K12.	--	chr3	TEC
ENSG00000025039.13	1.58944363	2.87067357	2.92E-07	1.83E-06	0.65	0.53	2.18	2.6	RRAGD	Ras-related GTP binding D	chr6	protein_coding
ENSG00000186812.11	1.51134393	1.96066462	3.14E-06	1.67E-05	0.21	0.19	0.58	0.92	ZNF397	zinc finger protein 397	chr18	protein_coding
ENSG00000265185.4	2.44822027	2.28892432	2.01E-13	2.70E-12	1.62	1.53	13.14	9.81	SNORD3B-1	small nucleolar RNA, C/D box 3B-1	chr17	snoRNA
ENSG00000130783.12	5.07737931	-2.9633201	0.00538165	0.01488851	0	0	0.05	0.01	CCDC62	coiled-coil domain containing 62	chr12	protein_coding
ENSG00000189013.13	-8.9168875	-0.1111228	1.42E-21	3.89E-20	0.74	1.04	0	0	KIR2DL4	killer cell immunoglobulin-like receptor, two domains, long cytoplasmic tail, 4	chr19	protein_coding
ENSG00000197020.9	2.42560612	-0.791203	4.87E-06	2.51E-05	0.04	0	0.08	0.22	ZNF100	zinc finger protein 100	chr19	protein_coding
ENSG00000105429.11	-1.0112123	8.31808778	0.00042398	0.00150372	30.33	31.54	20.99	19.87	MEGF8	multiple EGF-like-domains 8	chr19	protein_coding
ENSG00000248980.1	5.18753465	-2.9024431	0.00538165	0.01488851	0	0	0	0.45	RP11-87F15.	--	chr4	antisense
ENSG00000066279.15	3.70010265	2.95843611	2.83E-26	1.06E-24	0.08	0.08	0.74	2.09	ASPM	asp (abnormal spindle) homolog, microcephaly associated (Drosophila)	chr1	protein_coding
ENSG00000145246.12	1.09323337	2.04877491	0.00056133	0.00194735	0.27	0.3	0.8	0.82	ATP10D	ATPase, class V, type 10D	chr4	protein_coding
ENSG00000138061.10	5.52649354	6.07300607	3.91E-55	7.51E-53	0.48	0.36	17.15	35.02	CYP1B1	cytochrome P450, family 1, subfamily B, polypeptide 1	chr2	protein_coding
ENSG00000173267.12	-4.5278362	4.50417699	8.82E-40	6.59E-38	46.14	44.31	2.88	2.34	SNCG	synuclein, gamma (breast cancer-specific protein 1)	chr10	protein_coding
ENSG00000261740.4	-2.2556205	1.10674334	3.54E-10	3.30E-09	2.19	1.77	0.55	0.55	RP11-345J4.	--	chr16	protein_coding
ENSG00000145901.13	-2.5876345	9.64267619	8.08E-18	1.64E-16	347.07	373.58	82.72	76.78	TNIP1	TNFAIP3 interacting protein 1	chr5	protein_coding
ENSG00000253616.4	-3.5030607	-0.039689	2.78E-13	3.68E-12	1.76	1.45	0.25	0.12	RP11-875O1	--	chr8	antisense
ENSG00000175048.15	-1.0589005	4.32715301	0.0003066	0.0011136	2.92	3.03	1.99	1.81	ZDHHC14	zinc finger, DHHC-type containing 14	chr6	protein_coding
ENSG00000149499.10	-1.7222619	5.89469188	4.46E-09	3.59E-08	24.01	19.97	8.59	9.2	EML3	echinoderm microtubule associated protein like 3	chr11	protein_coding

ENSG00000186908.13	2.84711437	0.78369661	2.48E-12	2.99E-11	0.06	0.06	0.36	0.81	ZDHHC17	zinc finger, DHHC-type containing 17	chr12	protein_coding
ENSG00000198604.9	3.46505522	3.45804009	4.03E-25	1.41E-23	0.25	0.22	2.58	4.3	BAZ1A	bromodomain adjacent to zinc finger domain, 1A	chr14	protein_coding
ENSG00000227496.1	-1.8432625	-0.5805026	6.05E-05	0.00025435	0.49	0.56	0.19	0.2	RP11-145A3	--	chr1	antisense
ENSG00000117010.14	1.25965472	0.08531018	0.00149552	0.00475159	0.21	0.13	0.53	0.57	ZNF684	zinc finger protein 684	chr1	protein_coding
ENSG00000267940.1	1.87005625	-1.340948	0.00142213	0.00453952	0.12	0.04	0.45	0.36	RP11-290F2	--	chr11	lincRNA
ENSG00000066468.19	2.02914058	-1.7697499	0.00471212	0.01331279	0	0.02	0.08	0.04	FGFR2	fibroblast growth factor receptor 2	chr10	protein_coding
ENSG00000254859.1	5.57358239	0.02138063	3.53E-18	7.32E-17	0	0.11	4.57	3.23	RP11-661A1	--	chr8	antisense
ENSG00000145911.5	-3.0897486	4.68044518	9.93E-23	2.90E-21	5.92	6.31	1.08	0.83	N4BP3	NEDD4 binding protein 3	chr5	protein_coding
ENSG00000131711.13	-3.3690296	3.28128207	3.61E-24	1.18E-22	1.06	1.33	0.13	0.17	MAP1B	microtubule-associated protein 1B	chr5	protein_coding
ENSG00000176692.5	-2.3401521	5.22217663	9.69E-15	1.52E-13	19.82	20.93	5.53	5.18	FOXC2	forkhead box C2	chr16	protein_coding
ENSG00000178852.14	-3.5793056	-2.6357462	0.003237	0.00945156	0.01	0.06	0.01	0	EFCAB13	EF-hand calcium binding domain 13	chr17	protein_coding
ENSG00000159314.10	1.45866928	4.01496367	9.88E-07	5.70E-06	1.08	1.18	3.96	4.32	ARHGAP27	Rho GTPase activating protein 27	chr17	protein_coding
ENSG00000173641.16	-5.2072905	-3.0363253	0.00538165	0.01488851	0.05	0.02	0	0	HSPB7	heat shock 27kDa protein family, member 7 (cardiovascular)	chr1	protein_coding
ENSG00000163874.8	-1.1279751	6.70190147	9.15E-05	0.00037032	41.43	44.76	26.94	25.55	ZC3H12A	zinc finger CCCH-type containing 12A	chr1	protein_coding
ENSG00000249641.2	1.56643195	3.97798415	1.65E-07	1.07E-06	4.54	4.55	17.84	18.04	HOXC13-AS	HOXC13 antisense RNA	chr12	antisense
ENSG00000184675.8	-1.5561383	3.92013041	1.93E-07	1.25E-06	2.02	2.31	1.04	0.92	AMER1	APC membrane recruitment protein 1	chrX	protein_coding
ENSG00000080823.20	1.03954775	2.76537352	0.00067874	0.00231383	1.1	0.85	2.6	2.74	MOK	MOK protein kinase	chr14	protein_coding
ENSG00000159322.16	1.04234098	6.45552615	0.00029718	0.00108366	18.27	17.76	43.86	55.33	ADPGK	ADP-dependent glucokinase	chr15	protein_coding
ENSG00000255310.2	8.26682248	-0.5594557	5.69E-15	9.08E-14	0	0	0.31	0.97	AF131215.2	--	chr8	sense_intronic
ENSG00000170421.10	-1.7930087	10.9862623	8.65E-10	7.63E-09	1159.82	1127.17	441.13	438.14	KRT8	keratin 8, type II	chr12	protein_coding
ENSG00000187730.7	8.3870483	-0.462682	5.83E-16	1.01E-14	0	0	0.76	0.65	GABRD	gamma-aminobutyric acid (GABA) A receptor, delta	chr1	protein_coding
ENSG00000159459.10	2.75453381	1.3865715	7.72E-14	1.09E-12	0.08	0.05	0.59	0.61	UBR1	ubiquitin protein ligase E3 component n-recognin 1	chr15	protein_coding
ENSG00000175352.9	1.15315598	3.94969099	9.92E-05	0.00039981	1.77	2	5.24	5.91	NRIP3	nuclear receptor interacting protein 3	chr11	protein_coding
ENSG00000163075.11	6.11950765	-2.3132648	4.74E-05	0.00020301	0	0	0.08	0.07	CFAP221	cilia and flagella associated protein 221	chr2	protein_coding
ENSG00000165181.15	2.59544446	-1.6241145	0.00030126	0.00109685	0	0.02	0.05	0.1	C9orf84	chromosome 9 open reading frame 84	chr9	protein_coding
ENSG00000136944.16	6.76254233	2.31760772	7.80E-43	7.00E-41	0.01	0.01	1.93	1.63	LMX1B	LIM homeobox transcription factor 1, beta	chr9	protein_coding
ENSG00000183496.5	1.60802963	-0.1913878	0.00020673	0.00077884	0.1	0.04	0.18	0.41	MEX3B	mex-3 RNA binding family member B	chr15	protein_coding
ENSG00000272654.1	-1.2038685	-0.1528361	0.00299593	0.00881199	0.36	1.02	0.38	0.4	RP11-422P2	--	chr1	lincRNA
ENSG00000091409.13	2.74069269	4.73110518	9.10E-19	2.00E-17	1	0.9	6.29	10.82	ITGA6	integrin, alpha 6	chr2	protein_coding
ENSG00000269758.1	3.30602957	-2.4886951	0.003237	0.00945156	0.05	0	0.19	0.64	CTD-2245F1	--	chr19	lincRNA
ENSG00000154027.17	-5.459895	-1.2420884	1.61E-10	1.56E-09	0.17	0.1	0.01	0	AK5	adenylate kinase 5	chr1	protein_coding
ENSG00000140682.17	-1.3586919	5.62224918	3.06E-06	1.63E-05	26.29	31.79	16.06	14.04	TGFB1I1	transforming growth factor beta 1 induced transcript 1	chr16	protein_coding
ENSG00000118369.11	-1.4888206	4.81876199	4.09E-07	2.51E-06	6.26	7.39	3.65	2.8	USP35	ubiquitin specific peptidase 35	chr11	protein_coding
ENSG00000101546.11	-1.1763219	6.16299832	4.74E-05	0.00020331	55.63	58.28	36.07	30.93	RBFA	ribosome binding factor A (putative)	chr18	protein_coding
ENSG00000262172.1	-2.8188161	-1.3999353	9.86E-06	4.79E-05	1.03	1.15	0.07	0.31	CTD-2529O2	--	chr17	sense_intronic
ENSG00000198890.7	-1.0283839	3.0449413	0.00061199	0.00210696	3.03	3.81	1.68	2.8	PRMT6	protein arginine methyltransferase 6	chr1	protein_coding
ENSG00000261730.1	1.1499091	0.67014398	0.00167082	0.00520911	0.52	0.67	1.28	2.24	RP4-668J24.	--	chr6	antisense
ENSG00000130545.14	5.94305985	-2.4328306	0.00014842	0.00057423	0	0	0.08	0.22	CRB3	crumbs family member 3	chr19	protein_coding
ENSG00000071967.10	2.47359115	4.21165801	1.26E-15	2.12E-14	0.96	1.11	6.8	8.51	CYBRD1	cytochrome b reductase 1	chr2	protein_coding
ENSG00000128283.6	-1.8371585	7.08571309	3.92E-10	3.63E-09	71.32	86.41	32.33	26.27	CDC42EP1	CDC42 effector protein (Rho GTPase binding) 1	chr22	protein_coding
ENSG00000158023.8	1.15742515	-0.8866694	0.01896105	0.04532651	0.04	0.05	0.18	0.1	WDR66	WD repeat domain 66	chr12	protein_coding
ENSG00000111341.8	-3.5474727	-2.068536	1.86E-05	8.57E-05	0.16	0.23	0	0.04	MGP	matrix Gla protein	chr12	protein_coding
ENSG00000204815.7	-1.5324548	0.42173494	5.09E-05	0.00021703	0.62	0.78	0.33	0.31	TTC25	tetratricopeptide repeat domain 25	chr17	protein_coding
ENSG00000196890.4	2.18236186	0.44593811	6.18E-08	4.28E-07	0.27	0.04	1.03	0.88	HIST3H2BB	histone cluster 3, H2bb	chr1	protein_coding
ENSG00000261326.2	3.56156511	-1.6730966	1.91E-05	8.80E-05	0.01	0	0.12	0.06	LINC01355	long intergenic non-protein coding RNA 1355	chr1	lincRNA
ENSG00000154359.11	1.05051808	0.6748383	0.0036356	0.01051171	0.16	0.17	0.37	0.52	LONRF1	LON peptidase N-terminal domain and ring finger 1	chr8	protein_coding
ENSG00000180720.7	1.19195633	-0.7968881	0.01174651	0.02978814	0.27	0.06	0.59	0.41	CHRM4	cholinergic receptor, muscarinic 4	chr11	protein_coding
ENSG00000134533.5	-3.8146926	0.97794417	7.83E-20	1.86E-18	0.75	1.36	0.08	0.12	RERG	RAS-like, estrogen-regulated, growth inhibitor	chr12	protein_coding
ENSG00000146858.7	1.39188948	-0.3427076	0.00166446	0.005194	0.18	0.18	0.73	0.54	ZC3HAV1L	zinc finger CCCH-type, antiviral 1-like	chr7	protein_coding
ENSG00000104290.9	1.14352361	0.6883791	0.00165236	0.00516029	0.05	0.06	0.15	0.18	FZD3	frizzled class receptor 3	chr8	protein_coding
ENSG00000101782.13	1.03175073	2.6385168	0.00079091	0.00266183	0.77	0.85	1.57	2.86	RIOK3	RIO kinase 3	chr18	protein_coding
ENSG00000257839.1	-5.5241092	-2.8622723	0.00158008	0.00495521	0.14	0.22	0	0	RP11-290L1.	--	chr12	antisense
ENSG00000260704.1	-2.4915433	-2.7162016	0.00943894	0.02469931	0.09	0.06	0	0.03	LINC00543	long intergenic non-protein coding RNA 543	chr13	lincRNA
ENSG00000103496.13	-1.0959686	7.01477168	0.00014089	0.00054944	79.06	78.01	48.1	49.84	STX4	syntaxin 4	chr16	protein_coding
ENSG00000163558.11	1.67402389	1.86530133	3.96E-07	2.44E-06	0.19	0.37	0.93	1.44	PRKCI	protein kinase C, iota	chr3	protein_coding
ENSG00000100867.13	1.36857257	3.1946418	6.65E-06	3.34E-05	2.57	2.17	8.05	8.28	DHRS2	dehydrogenase/reductase (SDR family) member 2	chr14	protein_coding
ENSG00000156017.11	1.48710162	0.11911195	0.00023026	0.00086178	0.11	0.07	0.34	0.36	C9orf41	chromosome 9 open reading frame 41	chr9	protein_coding
ENSG00000138376.9	1.29513673	0.2299958	0.00108949	0.00355145	0.08	0.11	0.19	0.42	BARD1	BRCA1 associated RING domain 1	chr2	protein_coding
ENSG00000156471.11	-1.2969681	7.48458407	7.17E-06	3.58E-05	40.1	39.51	21.19	22	PTDSS1	phosphatidylserine synthase 1	chr8	protein_coding
ENSG00000244332.1	4.5741198	-1.5886403	1.57E-07	1.02E-06	0.04	0	0.84	0.8	RP11-119K6.	--	chr10	sense_overlapping
ENSG00000136235.14	3.51404595	4.02909044	1.16E-26	4.47E-25	0.48	0.61	8.48	8.08	GPNMB	glycoprotein (transmembrane) nmb	chr7	protein_coding
ENSG00000071794.14	3.16232393	2.56531189	4.30E-20	1.04E-18	0.12	0.24	1.16	3.09	HLTF	helicase-like transcription factor	chr3	protein_coding
ENSG00000230325.1	1.63718983	-1.817764	0.01883218	0.04519557	0	0.34	0.65	0.77	RP11-385F5.	--	chr1	antisense
ENSG00000229809.7	-1.0300574	4.1182375	0.00045989	0.00161959	7.16	6.73	5.16	3.87	ZNF688	zinc finger protein 688	chr16	protein_coding
ENSG00000237945.6	5.74149849	1.58434762	5.16E-32	2.62E-30	0.01	0	0.42	0.43	LINC00649	long intergenic non-protein coding RNA 649	chr21	antisense
ENSG00000142224.14	-6.0981358	-2.5020829	8.36E-05	0.00034052	0.09	0.11	0	0	IL19	interleukin 19	chr1	protein_coding
ENSG00000188056.10	4.73235456	-3.1388833	0.01889873	0.04519557	0	0	0.11	0	TREML4	triggering receptor expressed on myeloid cells-like 4	chr6	protein_coding
ENSG00000163362.9	-2.4888442	1.10557222	1.07E-11	1.20E-10	0.8	0.48	0.16	0.14	C1orf106	chromosome 1 open reading frame 106	chr1	protein_coding
ENSG00000071073.11	3.32414916	-1.8417887	0.00016407	0.00062818	0.01	0	0.02	0.07	MGAT4A	mannosyl (alpha-1,3-)-glycoprotein beta-1,4-N-acetylglucosaminyltransferase, isozyme A	chr2	protein_coding
ENSG00000270008.1	2.83261629	-0.3414482	1.66E-08	1.23E-07	0.18	0.19	2.18	1.32	RP11-298P3.	--	chr13	processed_transcript
ENSG00000162676.10	1.82197871	-0.2853752	4.90E-05	0.00020954	0.03	0.14	0.53	0.28	GFI1	growth factor independent 1 transcription repressor	chr1	protein_coding
ENSG00000064195.7	4.51684842	4.46660896	3.66E-39	2.69E-37	0.53	0.56	17.77	15.65	DLX3	distal-less homeobox 3	chr17	protein_coding
ENSG00000075618.16	-4.8142712	7.94851503	2.81E-47	3.36E-45	150.38	127.6	7.27	5.87	FSCN1	fascin actin-bundling protein 1	chr7	protein_coding
ENSG00000273321.1	4.49272476	-0.9148781	6.87E-10	6.13E-09	0	0.1	0.8	2.42	RP11-621L6.	--	chr18	lincRNA
ENSG00000125895.5	7.41154549	-1.3100436	1.18E-10	1.17E-09	0	0	0.39	0.41	TMEM74B	transmembrane protein 74B	chr20	protein_coding
ENSG00000277301.1	2.65761904	-2.2983538	0.00281991	0.00840887	0.02	0.02	0.22	0.08	RP5-1184F4.	--	chr20	antisense
ENSG00000229807.8	9.24505239	5.22443597	1.40E-84	1.20E-81	0.01	0	4.16	4.16	XIST	X inactive specific transcript (non-protein coding)	chrX	lincRNA
ENSG00000251247.8	7.48403407	-1.2516195	4.96E-11	5.14E-10	0	0	0.2	0.15	ZNF345	zinc finger protein 345	chr19	protein_coding
ENSG00000134202.9	-1.2986834	6.65674459	7.25E-06	3.61E-05	27.87	27.22	14.91	14.92	GSTM3	glutathione S-transferase mu 3 (brain)	chr1	protein_coding
ENSG00000072041.15	-5.305001	0.7897488	3.79E-26	1.41E-24	0.46	0.22	0.01	0.01	SLC6A15	solute carrier family 6 (neutral amino acid transporter), member 15	chr12	protein_coding
ENSG00000272308.1	4.88946445	-3.0622981	0.01889873	0.04519557	0	0	0.13	0.34	RP11-231G3	--	chr5	lincRNA
ENSG00000276529.1	-1.4141221	3.12524167	3.19E-06	1.70E-05	23.79	22.45	10.85	12.29	AP001505.10	--	chr21	lincRNA
ENSG00000272372.1	3.74390614	-2.2030658	0.00013996	0.00054609	0	0.02	0.23	0.13	RP11-441F2.	--	chr16	antisense
ENSG00000204514.8	4.53302611	-0.4091534	4.01E-13	5.24E-12	0.01	0	0.16	0.24	ZNF814	zinc finger protein 814	chr19	protein_coding
ENSG00000274654.1	-6.1013967	-2.5004644	8.36E-05	0.00034052	0.11	0.45	0	0	CTD-3247H4	--	chr15	sense_intronic
ENSG00000131668.12	-4.9168148	-0.1130326	1.66E-17	3.29E-16	1.22	0.37	0.04	0.03	BARX1	BARX homeobox 1	chr9	protein_coding
ENSG00000170160.15	4.4411708	0.75975523	2.41E-20	5.93E-19	0.02	0.01	0.32	0.39	CCDC144A	coiled-coil domain containing 144A	chr17	protein_coding
ENSG00000118557.14	4.87917439	-3.0678699	0.01889873	0.04519557	0	0	0	0.04	PMFBP1	polyamine modulated factor 1 binding protein 1	chr16	protein_coding
ENSG00000135111.13	-1.7163478	4.97271969	6.10E-09	4.83E-08	8.33	7.96	3.05	3.57	TBX3	T-box 3	chr12	protein_coding
ENSG00000130208.8	-2.3898	-0.6655333	6.49E-07	3.85E-06	0.89	1.04	0.11	0.36	APOC1	apolipoprotein C-I	chr19	protein_coding
ENSG00000244274.6	-2.2809606	4.47664212	6.18E-14	8.82E-13	11.22	10.4	2.84	3.09	DBNDD2	dysbindin (dystrobrevin binding protein 1) domain containing 2	chr20	protein_coding
ENSG00000102178.11	-2.3955683	6.78491791	1.28E-15	2.16E-14	61.93	64.07	15.55	16.37	UBL4A	ubiquitin-like 4A	chrX	protein_coding
ENSG00000166106.3	-2.1287277	2.73609089	1.62E-11	1.78E-10	1.79	1.29	0.39	0.55	ADAMTS15	ADAM metallopeptidase with thrombospondin type 1 motif, 15	chr11	protein_coding
ENSG00000172731.12	-1.1415582	3.17749945	0.00015133	0.00058436	3.19	2.35	1.8	1.54	LRRC20	leucine rich repeat containing 20	chr10	protein_coding
ENSG00000175390.11	-1.3579967	7.46496577	2.70E-06	1.46E-05	29.98	24.54	14.17	14.2	EIF3F	eukaryotic translation initiation factor 3, subunit F	chr11	protein_coding
ENSG00000133119.11	1.15451253	2.01696474	0.00031166	0.00113062	0.59	0.8	1.66	2.45	RFC3	replication factor C (activator 1) 3, 38kDa	chr13	protein_coding
ENSG00000105278.9	10.1007213	4.38388576	2.50E-76	1.40E-73	0.01	0	8.09	7.27	ZFR2	zinc finger RNA binding protein 2	chr19	protein_coding
ENSG00000210741.1	-3.0442028	-0.8740031	2.14E-08	1.56E-07	11.84	9.31	0.46	2.72	MIR196A1	microRNA 196a-1	chr17	miRNA
ENSG00000108691.8	-3.6294418	-1.585361	4.63E-07	2.81E-06	0.26	0.53	0	0.07	CCL2	chemokine (C-C motif) ligand 2	chr17	protein_coding
ENSG00000165724.5	-1.1492045	7.71357856	6.56E-05	0.00027409	177.01	163.57	103.69	100.89	ZMYND19	zinc finger, MYND-type containing 19	chr9	protein_coding
ENSG00000253731.2	-1.831391	-1.6067636	0.00244913	0.00738591	0.06	0.03	0	0.03	PCDHGA6	protocadherin gamma subfamily A, 6	chr5	protein_coding
ENSG00000175336.9	-3.3283414	-2.785196	0.00943894	0.02469931	0	0.17	0	0.02	APOF	apolipoprotein F	chr12	protein_coding
ENSG00000077147.13	1.37746129	4.8054726	2.66E-06	1.44E-05	2.02	2.07	5.27	8.96	TM9SF3	transmembrane 9 superfamily member 3	chr10	protein_coding
ENSG00000048544.5	-1.1845897	4.14081966	5.68E-05	0.00023972	8.49	10.3	4.81	6.22	MRPS10	mitochondrial ribosomal protein S10	chr6	protein_coding
ENSG00000179455.7	-8.8346182	-0.1903738	9.67E-21	2.47E-19	0.32	0.5	0	0	MKRN3	makorin ring finger protein 3	chr15	protein_coding
ENSG00000167123.17	-1.827035	7.18483278	4.79E-10	4.36E-09	36.69	33.94	14.84	11.61	CERCAM	cerebral endothelial cell adhesion molecule	chr9	protein_coding
ENSG00000137106.16	-1.0849304	7.53218984	0.00016152	0.00062081	106.88	85.6	59.97	61.07	GRHPR	glyoxylate reductase/hydroxypyruvate reductase	chr9	protein_coding
ENSG00000137628.15	1.97012527	-0.7626623	0.0001213	0.00047961	0.01	0.05	0.08	0.2	DDX60	DEAD (Asp-Glu-Ala-Asp) box polypeptide 60	chr4	protein_coding
ENSG00000148488.14	-6.8294869	-1.9677299	2.24E-07	1.42E-06	0.09	0.21	0	0	ST8SIA6	ST8 alpha-N-acetyl-neuraminide alpha-2,8-sialyltransferase 6	chr10	protein_coding
ENSG00000268655.1	6.68364233	-1.8994192	3.73E-07	2.30E-06	0	0	1.04	0.66	CTB-60B18.1	--	chr19	lincRNA
ENSG00000134516.14	-3.5498532	-0.1548834	5.44E-13	7.01E-12	0.14	0.24	0.01	0.03	DOCK2	dedicator of cytokinesis 2	chr5	protein_coding
ENSG00000281207.1	2.28506258	0.06906099	1.56E-07	1.02E-06	0.02	0.09	0.35	0.36	SLFNL1-AS1	SLFNL1 antisense RNA 1	chr1	antisense
ENSG00000273481.1	5.04175262	-2.9826774	0.0100455	0.02582742	0	0	0	0.51	RP11-126K1.	--	chr1	lincRNA
ENSG00000237989.1	11.2822837	2.27257232	8.90E-49	1.14E-46	0	0	3.86	3.39	AP001046.5	--	chr21	lincRNA
ENSG00000135077.7	-5.2081991	-3.0359038	0.00538165	0.01488851	0.05	0.03	0	0	HAVCR2	hepatitis A virus cellular receptor 2	chr5	protein_coding
ENSG00000260807.5	3.93269181	-2.06967	5.06E-05	0.00021569	0	0.01	0.09	0.09	RP11-161M6	--	chr16	lincRNA
ENSG00000155957.15	1.55109386	-0.3473735	0.00051464	0.00179394	0.04	0.11	0.34	0.28	TMBIM4	transmembrane BAX inhibitor motif containing 4	chr12	protein_coding
ENSG00000011465.15	-5.1652047	1.80533865	8.51E-34	4.71E-32	0.45	0.55	0	0.03	DCN	decorin	chr12	protein_coding
ENSG00000102109.8	9.73809467	6.35164086	2.10E-98	3.87E-95	0.15	0.13	180.51	153.38	PCSK1N	proprotein convertase subtilisin/kexin type 1 inhibitor	chrX	protein_coding
ENSG00000134146.10	-1.490223	1.7320653	4.70E-06	2.43E-05	0.7	0.75	0.27	0.42	DPH6	diphthamine biosynthesis 6	chr15	protein_coding
ENSG00000133315.9	-1.6677152	6.19991816	1.23E-08	9.28E-08	79.04	71.12	33.6	29.29	MACROD1	MACRO domain containing 1	chr11	protein_coding
ENSG00000118058.19	1.49013243	3.94058457	6.04E-07	3.61E-06	0.33	0.45	1.56	1.35	KMT2A	lysine (K)-specific methyltransferase 2A	chr11	protein_coding
ENSG00000104643.8	1.14609994	1.39877365	0.00066866	0.00228173	0.12	0.21	0.34	0.65	MTMR9	myotubularin related protein 9	chr8	protein_coding
ENSG00000099889.12	2.66137985	2.3329813	2.65E-15	4.35E-14	0.27	0.27	2.41	2.11	ARVCF	armadillo repeat gene deleted in velocardiofacial syndrome	chr22	protein_coding
ENSG00000257069.4	1.92180183	1.14794892	8.37E-08	5.66E-07	0.27	0.25	1.28	1.38	RP11-783K1	--	chr11	processed_transcript
ENSG00000130935.8	1.26913423	3.13243169	2.86E-05	0.00012777	1.34	1.45	2.99	6.05	NOL11	nucleolar protein 11	chr17	protein_coding
ENSG00000171873.7	-3.8923626	-1.8031984	6.51E-06	3.28E-05	0.16	0.11	0.02	0	ADRA1D	adrenoceptor alpha 1D	chr20	protein_coding
ENSG00000120137.6	1.17213167	2.36774076	0.00017928	0.00068259	0.21	0.27	0.52	0.93	PANK3	pantothenate kinase 3	chr5	protein_coding
ENSG00000102409.9	7.23663634	1.57965031	1.39E-36	8.71E-35	0.04	0	3.84	5.43	BEX4	brain expressed, X-linked 4	chrX	protein_coding
ENSG00000269929.1	2.21528684	-1.2527725	0.00018398	0.00069885	0.06	0.08	0.33	0.51	RP11-2B6.2	--	chr9	lincRNA
ENSG00000125966.9	2.66565318	0.67768003	2.55E-11	2.72E-10	0.13	0.02	0.65	0.62	MMP24	matrix metallopeptidase 24 (membrane-inserted)	chr20	protein_coding
ENSG00000161082.11	3.6760021	1.4986424	1.89E-20	4.69E-19	0.08	0.05	1.23	1.08	CELF5	CUGBP, Elav-like family member 5	chr19	protein_coding
ENSG00000106633.14	9.49727424	0.55944047	1.71E-26	6.53E-25	0	0	1.01	0.91	GCK	glucokinase (hexokinase 4)	chr7	protein_coding
ENSG00000171951.4	-5.5258708	-2.8614387	0.00158008	0.00495521	0.02	0.09	0	0	SCG2	secretogranin II	chr2	protein_coding
ENSG00000266970.1	-2.5257706	-1.0948393	9.67E-06	4.71E-05	0.76	0.77	0.29	0.07	RP11-806H1	--	chr17	lincRNA
ENSG00000066557.5	1.91142271	-0.7045852	0.00017223	0.00065755	0.05	0.08	0.12	0.5	LRRC40	leucine rich repeat containing 40	chr1	protein_coding
ENSG00000115896.14	6.37156228	-2.1293139	5.19E-06	2.66E-05	0	0	0.03	0.07	PLCL1	phospholipase C-like 1	chr2	protein_coding
ENSG00000261241.4	-5.7827447	-2.7070885	0.00047761	0.00167191	0.09	0.19	0	0	RP11-883G1	--	chr16	lincRNA
ENSG00000235098.7	3.98024429	-2.034788	3.06E-05	0.00013563	0.01	0	0.1	0.16	ANKRD65	ankyrin repeat domain 65	chr1	protein_coding
ENSG00000143387.11	2.25307508	0.28987927	4.58E-08	3.20E-07	0.24	0.13	1.36	1.03	CTSK	cathepsin K	chr1	protein_coding
ENSG00000142185.15	-5.6270696	2.63524321	1.94E-42	1.68E-40	1.47	1.58	0.03	0.05	TRPM2	transient receptor potential cation channel, subfamily M, member 2	chr21	protein_coding
ENSG00000108848.14	2.87048317	5.41558108	1.63E-20	4.09E-19	0.93	1.41	7.19	15.74	LUC7L3	LUC7-like 3 (S. cerevisiae)	chr17	protein_coding
ENSG00000162545.5	1.60066872	4.84957274	5.78E-08	4.01E-07	4.2	5.69	20.18	19.71	CAMK2N1	calcium/calmodulin-dependent protein kinase II inhibitor 1	chr1	protein_coding
ENSG00000272807.1	6.63363881	-1.9381017	6.25E-07	3.72E-06	0	0	0.69	0.4	RP11-260M2	--	chr2	antisense
ENSG00000108239.8	2.2869918	1.46572627	9.50E-11	9.54E-10	0.13	0.15	0.74	1.07	TBC1D12	TBC1 domain family, member 12	chr10	protein_coding
ENSG00000173511.8	-1.3181491	6.99439628	5.23E-06	2.67E-05	108.44	105.06	57.75	56.3	VEGFB	vascular endothelial growth factor B	chr11	protein_coding
ENSG00000237380.5	6.73295621	-1.8526589	3.73E-07	2.30E-06	0	0	0.09	0.5	HOXD-AS2	HOXD cluster antisense RNA 2	chr2	antisense
ENSG00000130338.11	1.52687752	2.70485031	9.19E-07	5.32E-06	0.27	0.21	0.9	0.92	TULP4	tubby like protein 4	chr6	protein_coding
ENSG00000198795.9	1.68589942	-0.2524098	0.00012743	0.000502	0.04	0.07	0.25	0.25	ZNF521	zinc finger protein 521	chr18	protein_coding
ENSG00000182621.15	-5.6583177	-2.783081	0.00086564	0.00288128	0.01	0.02	0	0	PLCB1	phospholipase C, beta 1 (phosphoinositide-specific)	chr20	protein_coding
ENSG00000134070.4	-3.2656951	4.75378961	7.93E-25	2.71E-23	11.86	10.86	1.59	1.56	IRAK2	interleukin-1 receptor-associated kinase 2	chr3	protein_coding
ENSG00000130528.10	2.77409472	-2.7984854	0.01615662	0.03958166	0.01	0	0.12	0.01	HRC	histidine rich calcium binding protein	chr19	protein_coding
ENSG00000204130.11	2.74383573	0.29166571	2.91E-10	2.73E-09	0.02	0.06	0.26	0.38	RUFY2	RUN and FYVE domain containing 2	chr10	protein_coding
ENSG00000172465.12	1.40565115	2.99883847	4.62E-06	2.40E-05	2.42	3.01	5.98	13.33	TCEAL1	transcription elongation factor A (SII)-like 1	chrX	protein_coding
ENSG00000125520.12	-1.2927981	8.29130489	7.51E-06	3.74E-05	202.14	214.18	120.43	105.48	SLC2A4RG	SLC2A4 regulator	chr20	protein_coding
ENSG00000179115.9	-1.2159691	7.9962865	2.46E-05	0.00011097	137.78	138.66	80.93	77.51	FARSA	phenylalanyl-tRNA synthetase, alpha subunit	chr19	protein_coding
ENSG00000240204.2	1.97963189	1.41118726	1.52E-08	1.13E-07	1.01	0.6	4.09	4.45	SMKR1	small lysine-rich protein 1	chr7	protein_coding
ENSG00000278274.1	-1.2146861	2.56250159	8.04E-05	0.00033073	48.76	53.63	28.58	30.16	SNORA61	small nucleolar RNA, H/ACA box 61	chr1	snoRNA
ENSG00000148671.12	6.91721106	1.27116162	1.30E-32	6.81E-31	0.03	0.03	6.29	4.42	ADIRF	adipogenesis regulatory factor	chr10	protein_coding
ENSG00000143942.4	1.96563961	0.63730582	3.77E-07	2.32E-06	0.55	0.17	0.9	2.91	CHAC2	ChaC, cation transport regulator homolog 2 (E. coli)	chr2	protein_coding
ENSG00000234636.2	2.45330034	-2.4232357	0.01191236	0.03011315	0.06	0	0.18	0.3	MED14OS	MED14 opposite strand	chrX	antisense
ENSG00000235423.7	1.01450318	5.63038128	0.00044884	0.00158275	14.01	16.2	36.96	44.51	RP11-282O1	--	chr12	antisense
ENSG00000119927.12	1.46538903	1.63090663	1.17E-05	5.60E-05	0.15	0.12	0.41	0.59	GPAM	glycerol-3-phosphate acyltransferase, mitochondrial	chr10	protein_coding
ENSG00000125618.15	2.24410773	1.51070448	1.85E-10	1.78E-09	0.11	0.25	1.23	1.09	PAX8	paired box 8	chr2	protein_coding
ENSG00000111247.13	2.33036248	1.18479527	2.15E-10	2.05E-09	0.2	0.34	0.83	2.79	RAD51AP1	RAD51 associated protein 1	chr12	protein_coding
ENSG00000162009.8	3.28071838	-1.1132741	5.22E-07	3.15E-06	0.08	0	0.52	0.58	SSTR5	somatostatin receptor 5	chr16	protein_coding
ENSG00000127838.12	-1.6182731	6.3424819	3.06E-08	2.18E-07	27.4	24.63	11.56	11.02	PNKD	paroxysmal nonkinesigenic dyskinesia	chr2	protein_coding
ENSG00000160803.7	-1.2184365	7.36518646	2.41E-05	0.00010885	50.93	53.51	30.34	29.42	UBQLN4	ubiquilin 4	chr1	protein_coding
ENSG00000118181.9	-1.045483	7.25135416	0.00027607	0.00101193	220.5	177.06	121.03	136.26	RPS25	ribosomal protein S25	chr11	protein_coding
ENSG00000169660.14	1.70881438	5.0901584	7.16E-09	5.62E-08	5.62	5.66	24.18	24.94	HEXDC	hexosaminidase (glycosyl hydrolase family 20, catalytic domain) containing	chr17	protein_coding
ENSG00000188996.4	-2.955735	-2.4427773	0.00455824	0.01291303	0.23	0.14	0.06	0	HUS1B	HUS1 checkpoint homolog b (S. pombe)	chr6	protein_coding
ENSG00000273706.3	5.7784906	-2.5437546	0.00026535	0.0009747	0	0	0.06	0.07	LHX1	LIM homeobox 1	chr17	protein_coding
ENSG00000103148.14	-1.012152	5.95778691	0.00045096	0.00158975	20.34	19.2	14.89	11.15	NPRL3	nitrogen permease regulator-like 3 (S. cerevisiae)	chr16	protein_coding
ENSG00000180425.9	1.55711348	0.97407498	1.25E-05	5.97E-05	0.39	0.31	1.66	1.09	C11orf71	chromosome 11 open reading frame 71	chr11	protein_coding
ENSG00000124766.5	-2.0927416	5.61464572	2.04E-12	2.48E-11	11.2	10.76	3.54	3.32	SOX4	SRY (sex determining region Y)-box 4	chr6	protein_coding
ENSG00000114520.9	2.28356869	1.84738158	2.34E-11	2.51E-10	0.49	0.27	1.49	3.51	SNX4	sorting nexin 4	chr3	protein_coding
ENSG00000101405.3	5.48900786	-2.7279255	0.00158008	0.00495521	0	0	0.62	0.11	OXT	oxytocin/neurophysin I prepropeptide	chr20	protein_coding
ENSG00000210196.2	3.66736172	-0.5271431	1.39E-10	1.37E-09	1.18	0.83	19.31	16.42	MT-TP	mitochondrially encoded tRNA proline	chrM	Mt_tRNA
ENSG00000132170.18	1.59229482	4.45240347	8.43E-08	5.70E-07	3.1	2.85	10.62	13.38	PPARG	peroxisome proliferator-activated receptor gamma	chr3	protein_coding
ENSG00000230234.1	-8.4358799	-0.566717	3.33E-17	6.40E-16	2.86	1.64	0	0	RP1-276N6.2	--	chr6	lincRNA
ENSG00000268049.1	-3.5314904	-2.0659034	0.00010192	0.00041007	0.35	0.15	0.06	0	CTD-2619J1	--	chr19	antisense

ENSG00000137764.18	-1.0230442	5.15460678	0.00041612	0.00147826	13.23	13.2	8.25	9.09	MAP2K5	mitogen-activated protein kinase kinase 5	chr15	protein_coding
ENSG00000105877.16	5.01941544	0.01682717	4.49E-17	8.55E-16	0.01	0	0.13	0.14	DNAH11	dynein, axonemal, heavy chain 11	chr7	protein_coding
ENSG00000162600.10	1.33312884	1.66837642	5.40E-05	0.00022876	0.64	0.83	2.25	2.69	OMA1	OMA1 zinc metallopeptidase	chr1	protein_coding
ENSG00000168807.15	2.10676163	4.02269781	5.16E-12	5.99E-11	0.44	0.57	2.67	3.12	SNTB2	syntrophin, beta 2 (dystrophin-associated protein A1, 59kDa, basic component 2)	chr16	protein_coding
ENSG00000228423.2	5.72086457	-2.5845337	0.00026535	0.0009747	0	0	1.27	0	RP4-633I8.4	--	chr1	lincRNA
ENSG00000075303.11	1.74617799	-0.2096543	7.00E-05	0.00029061	0.08	0.06	0.24	0.4	SLC25A40	solute carrier family 25, member 40	chr7	protein_coding
ENSG00000198586.12	2.72965267	2.47893902	4.36E-16	7.64E-15	0.2	0.18	1.2	2.21	TLK1	tousled-like kinase 1	chr2	protein_coding
ENSG00000134769.20	-6.9545537	0.13500469	5.25E-24	1.68E-22	0.17	0.16	0	0	DTNA	dystrobrevin, alpha	chr18	protein_coding
ENSG00000026297.14	1.31387526	4.11576852	9.29E-06	4.53E-05	3.85	3.86	13.81	11.7	RNASET2	ribonuclease T2	chr6	protein_coding
ENSG00000228192.6	-3.1126548	0.40848577	1.20E-13	1.66E-12	0.84	1.52	0.1	0.25	RP11-342M1	--	chr1	antisense
ENSG00000016402.11	3.77462089	1.08751861	4.79E-19	1.08E-17	0.08	0.02	0.96	0.91	IL20RA	interleukin 20 receptor, alpha	chr6	protein_coding
ENSG00000105676.12	-1.1124651	6.33311043	0.0001158	0.00045945	33.45	29.33	20.51	18.14	ARMC6	armadillo repeat containing 6	chr19	protein_coding
ENSG00000234965.2	-6.1895265	-2.4395964	4.74E-05	0.00020301	0.15	0.16	0	0	SHISA8	shisa family member 8	chr22	protein_coding
ENSG00000204362.5	-5.0679112	-1.58021	1.76E-09	1.50E-08	0.09	0.11	0	0.01	RP11-380J14	--	chr1	lincRNA
ENSG00000050030.12	5.22455067	-1.0542998	4.59E-10	4.19E-09	0	0	0.07	0.08	KIAA2022	KIAA2022	chrX	protein_coding
ENSG00000138193.13	2.43771903	1.55681485	6.63E-12	7.60E-11	0.07	0.03	0.31	0.47	PLCE1	phospholipase C, epsilon 1	chr10	protein_coding
ENSG00000157500.9	1.16879043	1.69947056	0.00035399	0.00127435	0.19	0.33	0.62	0.94	APPL1	adaptor protein, phosphotyrosine interaction, PH domain and leucine zipper containing 1	chr3	protein_coding
ENSG00000156787.15	1.48076329	1.56890114	9.71E-06	4.73E-05	0.34	0.36	0.77	1.85	TBC1D31	TBC1 domain family, member 31	chr8	protein_coding
ENSG00000174473.14	-4.0655482	-2.3253575	0.00023419	0.00087473	0.07	0.01	0.01	0	GALNTL6	polypeptide N-acetylgalactosaminyltransferase-like 6	chr4	protein_coding
ENSG00000084628.8	-3.5910596	1.29423687	1.21E-19	2.82E-18	1.15	1.69	0.2	0.12	NKAIN1	Na+/K+ transporting ATPase interacting 1	chr1	protein_coding
ENSG00000134744.12	2.50431876	2.24247725	9.82E-14	1.37E-12	0.12	0.2	0.91	1.53	ZCCHC11	zinc finger, CCHC domain containing 11	chr1	protein_coding
ENSG00000124593.13	-1.4264098	2.69074362	4.04E-06	2.11E-05	3.34	3.66	1.99	1.47	PRICKLE4	prickle homolog 4 (Drosophila)	chr6	protein_coding
ENSG00000163608.13	1.34590465	1.80144337	3.61E-05	0.00015857	0.38	0.24	0.88	1.23	C3orf17	chromosome 3 open reading frame 17	chr3	protein_coding
ENSG00000127914.15	4.71972534	2.63853729	3.67E-34	2.06E-32	0.03	0.02	0.47	1.26	AKAP9	A kinase (PRKA) anchor protein 9	chr7	protein_coding
ENSG00000172977.11	-1.1017319	6.57198075	0.0001326	0.00052084	43.74	40.89	27.4	25.09	KAT5	K(lysine) acetyltransferase 5	chr11	protein_coding
ENSG00000106069.19	4.13368871	-0.12889	5.23E-14	7.51E-13	0.03	0.01	0.45	0.37	CHN2	chimerin 2	chr7	protein_coding
ENSG00000132274.14	2.55347479	-1.6562561	0.00044263	0.00156293	0.02	0.02	0.11	0.19	TRIM22	tripartite motif containing 22	chr11	protein_coding
ENSG00000159307.17	8.03081882	-0.7709313	3.61E-13	4.73E-12	0	0	0.06	0.13	SCUBE1	signal peptide, CUB domain, EGF-like 1	chr22	protein_coding
ENSG00000133597.8	1.28519163	5.62321452	9.86E-06	4.79E-05	9.29	8.88	30.7	28.24	ADCK2	aarF domain containing kinase 2	chr7	protein_coding
ENSG00000168779.18	-2.1676017	-0.3421044	1.37E-06	7.72E-06	0.38	0.18	0.08	0.08	SHOX2	short stature homeobox 2	chr3	protein_coding
ENSG00000037965.5	1.64807071	1.83891003	5.25E-07	3.16E-06	0.71	0.74	3.12	2.91	HOXC8	homeobox C8	chr12	protein_coding
ENSG00000138346.13	2.88589053	0.9814814	2.35E-13	3.14E-12	0.09	0.08	0.57	1.09	DNA2	DNA replication helicase/nuclease 2	chr10	protein_coding
ENSG00000135951.13	1.41042069	-0.2216918	0.00095368	0.00314594	0.12	0.05	0.25	0.34	TSGA10	testis specific, 10	chr2	protein_coding
ENSG00000196268.10	-2.9606677	-2.4458032	0.00455824	0.01291303	0.03	0.01	0	0	ZNF493	zinc finger protein 493	chr19	protein_coding
ENSG00000107537.12	2.55887746	4.89730919	7.09E-17	1.32E-15	2.76	3.16	20.5	26.08	PHYH	phytanoyl-CoA 2-hydroxylase	chr10	protein_coding
ENSG00000163808.15	2.70880651	2.09953862	4.09E-15	6.59E-14	0.21	0.15	1.05	2.12	KIF15	kinesin family member 15	chr3	protein_coding
ENSG00000273018.4	5.60752246	1.46300103	2.31E-30	1.08E-28	0.02	0.01	0.64	1.72	CTD-2303H2	--	chr17	processed_transcript
ENSG00000130559.17	-1.7389066	5.59366463	3.40E-09	2.78E-08	7.25	8.32	2.91	3.31	CAMSAP1	calmodulin regulated spectrin-associated protein 1	chr9	protein_coding
ENSG00000119778.13	2.74273313	0.36035036	1.62E-10	1.57E-09	0.03	0.03	0.27	0.28	ATAD2B	ATPase family, AAA domain containing 2B	chr2	protein_coding
ENSG00000257551.1	1.54693717	-1.7083501	0.01792389	0.04353308	0.33	0	0.68	0.68	HLX-AS1	HLX antisense RNA 1	chr1	antisense
ENSG00000109625.17	1.20312692	1.80113939	0.00021516	0.0008087	0.62	0.69	2.49	1.53	CPZ	carboxypeptidase Z	chr4	protein_coding
ENSG00000168993.13	-2.2631229	1.7594071	2.57E-11	2.75E-10	1.86	2.29	0.48	0.66	CPLX1	complexin 1	chr4	protein_coding
ENSG00000204860.4	-1.3343966	2.09870708	2.84E-05	0.00012671	1.2	1.36	0.77	0.59	FAM201A	family with sequence similarity 201, member A	chr9	antisense
ENSG00000176697.17	-2.8204203	1.21274458	1.32E-14	2.03E-13	0.38	0.47	0.03	0.12	BDNF	brain-derived neurotrophic factor	chr11	protein_coding
ENSG00000188064.8	-5.2416162	6.41954703	5.59E-52	8.53E-50	27.14	26.33	1.03	0.85	WNT7B	wingless-type MMTV integration site family, member 7B	chr22	protein_coding
ENSG00000164850.13	10.7280063	1.73376861	2.71E-41	2.21E-39	0	0	2.36	2.02	GPER1	G protein-coupled estrogen receptor 1	chr7	protein_coding
ENSG00000247853.2	3.11418077	-1.9927002	0.0002605	0.00096399	0.02	0	0.1	0.21	RP5-940J5.6	--	chr12	antisense
ENSG00000254553.1	1.13180679	0.47003907	0.00260669	0.00783149	0.17	0.21	0.54	0.58	RP1-27O5.3	--	chr1	protein_coding
ENSG00000204576.10	-1.0897176	3.34058266	0.00026399	0.0009747	4.41	5.47	3.1	3.08	PRR3	proline rich 3	chr6	protein_coding
ENSG00000239467.4	7.8501087	-0.9299935	4.78E-12	5.56E-11	0	0	0.92	1.9	AC007405.6	--	chr2	lincRNA
ENSG00000077238.12	-3.3240604	6.06677466	2.92E-26	1.09E-24	26.79	24.58	3.45	3.38	IL4R	interleukin 4 receptor	chr16	protein_coding
ENSG00000216588.7	5.58023914	-2.6705827	0.00086564	0.00288128	0	0	0.19	0.19	IGSF23	immunoglobulin superfamily, member 23	chr19	protein_coding
ENSG00000238650.1	-1.7774977	-2.0751485	0.01397446	0.03477946	2.4	4.65	1.43	1.22	SNORD54	small nucleolar RNA, C/D box 54	chr8	snoRNA
ENSG00000213619.8	-1.3485293	7.3062063	3.17E-06	1.69E-05	83.54	69.9	39.04	41.37	NDUFS3	NADH dehydrogenase (ubiquinone) Fe-S protein 3, 30kDa (NADH-coenzyme Q reductase)	chr11	protein_coding
ENSG00000240694.7	-8.4978206	4.15237178	7.29E-72	3.24E-69	5.83	5.75	0.01	0.03	PNMA2	paraneoplastic Ma antigen 2	chr8	protein_coding
ENSG00000173193.12	2.97611368	2.14723191	1.72E-17	3.40E-16	0.09	0.11	0.9	1.19	PARP14	poly (ADP-ribose) polymerase family, member 14	chr3	protein_coding
ENSG00000263823.1	2.02810717	-0.6320538	3.75E-05	0.00016418	0.19	0.04	0.75	0.5	RP11-326K1	--	chr18	antisense
ENSG00000235529.1	1.29617058	-0.5865206	0.00561695	0.01547162	0.36	0.47	1.25	1.48	AGAP1-IT1	AGAP1 intronic transcript 1	chr2	sense_intronic
ENSG00000122033.13	1.10334273	2.66786869	0.00034508	0.00124524	2.7	2.86	6.58	9.4	MTIF3	mitochondrial translational initiation factor 3	chr13	protein_coding
ENSG00000164318.16	-9.7253842	2.87452642	1.96E-59	4.52E-57	2.51	2.23	0	0.01	EGFLAM	EGF-like, fibronectin type III and laminin G domains	chr5	protein_coding
ENSG00000102316.15	-1.6054523	7.41411717	3.59E-08	2.54E-07	86.68	70.38	36.36	32.37	MAGED2	melanoma antigen family D2	chrX	protein_coding
ENSG00000128408.8	1.13061395	5.12052725	0.00010099	0.00040654	11.83	11.99	36.69	32.7	RIBC2	RIB43A domain with coiled-coils 2	chr22	protein_coding
ENSG00000153214.8	1.39407791	2.2111	1.19E-05	5.68E-05	0.38	0.39	1.1	1.63	TMEM87B	transmembrane protein 87B	chr2	protein_coding
ENSG00000256073.3	1.24786108	1.68244049	0.00014387	0.00056016	2	1.6	6.55	4.85	URB1-AS1	URB1 antisense RNA 1 (head to head)	chr21	lincRNA
ENSG00000114331.11	1.14881261	1.03516621	0.00107319	0.00350545	0.17	0.1	0.28	0.53	ACAP2	ArfGAP with coiled-coil, ankyrin repeat and PH domains 2	chr3	protein_coding
ENSG00000275395.3	2.94943925	-1.68674	0.0001543	0.00059496	0	0.01	0.03	0.04	FCGBP	Fc fragment of IgG binding protein	chr19	protein_coding
ENSG00000196388.7	1.59054725	0.19214595	7.54E-05	0.00031153	0.34	0.2	1.07	1.14	INCA1	inhibitor of CDK, cyclin A1 interacting protein 1	chr17	protein_coding
ENSG00000038295.6	-4.6514505	0.85741275	3.07E-24	1.01E-22	0.41	0.36	0.01	0.03	TLL1	tolloid-like 1	chr4	protein_coding
ENSG00000260077.1	-2.5333753	0.89300075	1.60E-11	1.76E-10	1.75	1.48	0.29	0.45	RP11-254F7.	--	chr2	lincRNA
ENSG00000250320.4	-8.6755977	-0.34011	3.09E-19	7.03E-18	1.17	0.64	0	0	CTD-2269F5	--	chr5	antisense
ENSG00000180573.9	2.1691828	4.35131918	9.52E-13	1.20E-11	3.95	3.27	17.57	25.96	HIST1H2AC	histone cluster 1, H2ac	chr6	protein_coding
ENSG00000231196.3	-2.3425533	-2.7918514	0.01615662	0.03958166	0.24	0.13	0	0.08	RP11-495P1(	--	chr1	lincRNA
ENSG00000147403.15	-2.7778733	8.87106073	5.38E-20	1.29E-18	241.81	234.69	50.4	41.95	RPL10	ribosomal protein L10	chrX	protein_coding
ENSG00000258441.1	1.95705695	-1.1361891	0.00044528	0.00157159	0.11	0.03	0.25	0.48	LINC00641	long intergenic non-protein coding RNA 641	chr14	processed_transcript
ENSG00000066084.11	1.67023669	3.1962913	5.11E-08	3.56E-07	0.41	0.4	1.69	1.78	DIP2B	DIP2 disco-interacting protein 2 homolog B (Drosophila)	chr12	protein_coding
ENSG00000086506.2	-4.8812818	-1.729147	1.87E-08	1.38E-07	1.25	0.26	0	0.05	HBQ1	hemoglobin, theta 1	chr16	protein_coding
ENSG00000107551.19	-3.6268196	-0.5451081	9.53E-12	1.07E-10	0.37	0.17	0.03	0.03	RASSF4	Ras association (RalGDS/AF-6) domain family member 4	chr10	protein_coding
ENSG00000186446.10	3.86705067	-2.1158911	8.40E-05	0.0003415	0	0.01	0.09	0.1	ZNF501	zinc finger protein 501	chr3	protein_coding
ENSG00000102218.5	2.69551692	-0.105748	7.38E-09	5.76E-08	0.03	0.07	0.4	0.43	RP2	retinitis pigmentosa 2 (X-linked recessive)	chrX	protein_coding
ENSG00000012660.12	1.37271126	6.47976653	2.25E-06	1.23E-05	10.58	12.52	31.27	48.82	ELOVL5	ELOVL fatty acid elongase 5	chr6	protein_coding
ENSG00000157514.15	-3.4622116	7.15903194	2.03E-28	8.63E-27	61.38	54.18	6.9	7.09	TSC22D3	TSC22 domain family, member 3	chrX	protein_coding
ENSG00000182747.4	2.62699567	-2.3163956	0.00455824	0.01291303	0.02	0	0.07	0.14	SLC35D3	solute carrier family 35, member D3	chr6	protein_coding
ENSG00000189152.9	5.46459707	-2.7413886	0.00158008	0.00495521	0	0	0.09	0.11	GRAPL	GRB2-related adaptor protein-like	chr17	protein_coding
ENSG00000170234.11	1.30654942	1.59259886	8.00E-05	0.00032928	0.23	0.24	0.61	0.96	PWWP2A	PWWP domain containing 2A	chr5	protein_coding
ENSG00000279794.1	-1.9562509	-2.3959331	0.02049243	0.04852277	0.4	0.13	0	0.16	CTD-2621I17	--	chr19	TEC
ENSG00000177674.14	-1.2142777	5.05376462	3.08E-05	0.00013636	29.51	27.5	17.83	14.85	AGTRAP	angiotensin II receptor-associated protein	chr1	protein_coding
ENSG00000125398.5	-3.3025261	1.04407937	3.61E-17	6.90E-16	0.58	0.97	0.06	0.15	SOX9	SRY (sex determining region Y)-box 9	chr17	protein_coding
ENSG00000083642.17	2.56961255	2.18302163	3.73E-14	5.43E-13	0.15	0.12	0.73	1.47	PDS5B	PDS5 cohesin associated factor B	chr13	protein_coding
ENSG00000248710.1	3.64041425	0.05722201	5.91E-13	7.60E-12	0.02	0.05	0.37	0.75	RP11-432B6.	--	chr3	protein_coding
ENSG00000171204.11	1.39520296	0.50158706	0.00026098	0.0009656	0.34	0.46	1.24	1.59	TMEM126B	transmembrane protein 126B	chr11	protein_coding
ENSG00000169718.16	-1.0479062	7.27817024	0.00026743	0.00098176	71.29	61.77	44.31	41.41	DUS1L	dihydrouridine synthase 1-like (S. cerevisiae)	chr17	protein_coding
ENSG00000176532.3	4.10399499	2.82970521	1.40E-29	6.33E-28	0.38	0.35	9.51	7.43	PRR15	proline rich 15	chr7	protein_coding
ENSG00000075539.12	2.57186811	1.58446353	6.43E-13	8.23E-12	0.07	0.04	0.46	0.42	FRYL	FRY-like	chr4	protein_coding
ENSG00000172071.10	1.68016289	2.35494168	1.55E-07	1.01E-06	0.35	0.45	1.45	1.97	EIF2AK3	eukaryotic translation initiation factor 2-alpha kinase 3	chr2	protein_coding
ENSG00000114670.12	2.51303156	-0.3577781	2.51E-07	1.58E-06	0.04	0.05	0.34	0.37	NEK11	NIMA-related kinase 11	chr3	protein_coding
ENSG00000096060.13	-2.9194179	5.9914298	1.75E-21	4.74E-20	7.42	9.73	1.36	1.66	FKBP5	FK506 binding protein 5	chr6	protein_coding
ENSG00000173889.14	2.52133698	2.39680111	3.28E-14	4.81E-13	0.1	0.07	0.55	0.78	PHC3	polyhomeotic homolog 3 (Drosophila)	chr3	protein_coding
ENSG00000106066.12	2.83248657	4.64369783	1.06E-19	2.48E-18	1.71	1.81	14.75	18.77	CPVL	carboxypeptidase, vitellogenic-like	chr7	protein_coding
ENSG00000225127.2	4.88946445	-3.0622981	0.01889873	0.04519557	0	0	0.05	0.13	LINC00237	long intergenic non-protein coding RNA 237	chr20	lincRNA
ENSG00000188517.13	-6.4889253	1.02632788	2.53E-31	1.24E-29	0.52	0.52	0.02	0	COL25A1	collagen, type XXV, alpha 1	chr4	protein_coding
ENSG00000140057.7	-1.9213912	0.1143397	2.36E-06	1.28E-05	0.35	0.27	0.11	0.11	AK7	adenylate kinase 7	chr14	protein_coding
ENSG00000268205.1	1.24293946	1.70697408	0.00015717	0.00060496	0.35	0.37	1.31	0.95	CTC-444N24	--	chr19	lincRNA
ENSG00000164292.11	2.9514704	4.66543246	5.10E-21	1.33E-19	0.61	0.86	5.63	9.62	RHOBTB3	Rho-related BTB domain containing 3	chr5	protein_coding
ENSG00000258603.3	2.16264419	-0.1036484	1.50E-06	8.40E-06	0.35	0.19	1.46	1.78	RP3-414A15	--	chr14	antisense
ENSG00000180739.13	1.04976822	3.42046044	0.00043707	0.00154582	1.95	2.63	6.98	5.6	S1PR5	sphingosine-1-phosphate receptor 5	chr19	protein_coding
ENSG00000164329.12	1.36150071	2.02265963	2.35E-05	0.00010681	0.56	0.44	1.34	2.09	PAPD4	PAP associated domain containing 4	chr5	protein_coding
ENSG00000135999.10	2.50408155	1.38969112	6.48E-12	7.43E-11	0.07	0.18	0.82	1.05	EPC2	enhancer of polycomb homolog 2 (Drosophila)	chr2	protein_coding
ENSG00000242288.9	1.48725079	-0.5947531	0.00211472	0.00646114	0.06	0.07	0.28	0.21	RP11-464F9.	--	chr10	processed_transcript
ENSG00000062194.14	1.39898403	2.91034369	5.15E-06	2.65E-05	0.71	0.7	1.86	3.1	GPBP1	GC-rich promoter binding protein 1	chr5	protein_coding
ENSG00000108654.10	1.11556357	8.53189462	0.00010417	0.00041829	64.5	72.44	157.67	239.63	DDX5	DEAD (Asp-Glu-Ala-Asp) box helicase 5	chr17	protein_coding
ENSG00000184923.11	2.52223374	-0.7213858	2.66E-06	1.44E-05	0.03	0.02	0.23	0.16	NUTM2A	NUT family member 2A	chr10	protein_coding
ENSG00000085117.10	-2.4089734	5.22007077	1.87E-15	3.11E-14	23.58	20.76	6.45	4.64	CD82	CD82 molecule	chr11	protein_coding
ENSG00000173681.15	1.65863617	0.44644274	1.97E-05	9.02E-05	0.08	0.07	0.3	0.34	CXorf23	chromosome X open reading frame 23	chrX	protein_coding
ENSG00000046604.11	1.17220173	3.54241031	9.00E-05	0.00036419	0.81	1.05	2.04	3.58	DSG2	desmoglein 2	chr18	protein_coding
ENSG00000150477.13	1.59901864	1.28514371	3.83E-06	2.01E-05	0.06	0.16	0.39	0.53	KIAA1328	KIAA1328	chr18	protein_coding
ENSG00000161642.16	-6.2571699	4.22030613	2.18E-57	4.71E-55	10.77	11.03	0.2	0.18	ZNF385A	zinc finger protein 385A	chr12	protein_coding
ENSG00000074660.14	4.27702844	0.23364563	3.62E-16	6.37E-15	0.03	0.02	0.64	0.64	SCARF1	scavenger receptor class F, member 1	chr17	protein_coding
ENSG00000266094.5	1.00508914	2.50541553	0.00111404	0.00362851	0.53	0.44	0.93	1.69	RASSF5	Ras association (RalGDS/AF-6) domain family member 5	chr1	protein_coding
ENSG00000170345.8	3.76118418	4.66800477	1.58E-30	7.45E-29	0.88	1.03	17.42	17.17	FOS	FBJ murine osteosarcoma viral oncogene homolog	chr14	protein_coding
ENSG00000166250.10	-10.987496	1.93505486	3.65E-46	4.09E-44	1.2	1.25	0	0	CLMP	CXADR-like membrane protein	chr11	protein_coding
ENSG00000062370.15	2.8190771	0.17936659	2.02E-10	1.93E-09	0.11	0	0.33	0.73	ZNF112	zinc finger protein 112	chr19	protein_coding
ENSG00000197943.8	4.4104167	2.87585254	1.48E-32	7.74E-31	0.08	0.04	1.63	1.79	PLCG2	phospholipase C, gamma 2 (phosphatidylinositol-specific)	chr16	protein_coding
ENSG00000076554.14	1.3106332	1.44448251	9.78E-05	0.00039443	0.28	0.22	0.57	1.09	TPD52	tumor protein D52	chr8	protein_coding
ENSG00000275481.1	5.46945885	-2.7387023	0.00158008	0.00495521	0	0	0.06	0.05	RP11-474P2.	--	chr12	lincRNA
ENSG00000148842.16	1.30010345	4.80093801	8.98E-06	4.39E-05	0.8	0.81	2.82	2.46	CNNM2	cyclin and CBS domain divalent metal cation transport mediator 2	chr10	protein_coding
ENSG00000247498.8	-7.0856692	0.25593632	2.50E-25	8.82E-24	0.71	0.4	0	0.01	RP11-392P7.	--	chr12	antisense
ENSG00000163378.12	2.3300276	1.4746209	4.90E-11	5.09E-10	0.15	0.15	0.68	1.36	EOGT	EGF domain-specific O-linked N-acetylglucosamine (GlcNAc) transferase	chr3	protein_coding
ENSG00000275632.1	3.03619917	0.17098274	2.31E-11	2.48E-10	0.41	0.27	3.73	3.85	RP5-967N21	--	chr20	lincRNA
ENSG00000176396.9	1.67578352	3.14709551	5.26E-08	3.66E-07	1.51	1.9	5.24	9.38	EID2	EP300 interacting inhibitor of differentiation 2	chr19	protein_coding
ENSG00000188206.6	2.84367216	1.27171371	5.41E-14	7.74E-13	0.1	0.08	0.61	1.14	HNRNPU-AS	HNRNPU antisense RNA 1	chr1	antisense
ENSG00000231025.1	2.40021663	0.22770004	1.57E-08	1.17E-07	0.06	0.1	0.45	0.67	RP11-175O1	--	chr10	antisense
ENSG00000224712.9	-1.5950781	3.06004921	1.88E-07	1.21E-06	4.09	5.17	2.12	1.95	NPIPA3	nuclear pore complex interacting protein family, member A3	chr16	protein_coding
ENSG00000089335.19	2.76822895	1.99451763	2.05E-15	3.39E-14	0.21	0.28	1.52	2.93	ZNF302	zinc finger protein 302	chr19	protein_coding
ENSG00000233251.6	1.7658281	-1.9364543	0.01967235	0.04677036	0	0.04	0.08	0.1	AC007743.1	--	chr2	antisense
ENSG00000131242.14	4.77662343	2.98731323	4.20E-36	2.62E-34	0.03	0.07	1.8	1.85	RAB11FIP4	RAB11 family interacting protein 4 (class II)	chr17	protein_coding
ENSG00000143970.15	1.51191681	2.86709865	9.85E-07	5.68E-06	0.18	0.29	0.8	0.98	ASXL2	additional sex combs like transcriptional regulator 2	chr2	protein_coding
ENSG00000265399.1	5.23774779	-2.8750297	0.00290522	0.00856312	0	0	0.18	0	RP13-270P1	--	chr18	antisense
ENSG00000256603.1	4.91020659	-3.0511096	0.0100455	0.02582742	0	0	0.33	0.09	RP11-667M1	--	chr11	antisense
ENSG00000106333.11	-2.3166509	3.27345689	1.57E-13	2.13E-12	7.83	7.48	2.39	1.7	PCOLCE	procollagen C-endopeptidase enhancer	chr7	protein_coding
ENSG00000135083.13	-1.4785981	3.59675199	8.45E-07	4.93E-06	4.09	4.46	2.1	1.98	CCNJL	cyclin J-like	chr5	protein_coding
ENSG00000279744.1	4.39110003	0.7168839	6.82E-20	1.63E-18	0.02	0.12	2.03	1.79	RP13-20L14.	--	chr17	TEC
ENSG00000050555.16	-1.9103095	7.04159259	8.55E-11	8.61E-10	28.43	26.5	10.63	8.79	LAMC3	laminin, gamma 3	chr9	protein_coding
ENSG00000037757.12	-1.2599863	5.04555852	1.55E-05	7.26E-05	11.87	11.27	6.38	6.5	MRI1	methylthioribose-1-phosphate isomerase 1	chr19	protein_coding
ENSG00000115548.15	1.8023769	3.68353587	3.16E-09	2.60E-08	0.75	1.06	3.22	5.24	KDM3A	lysine (K)-specific demethylase 3A	chr2	protein_coding
ENSG00000090539.14	4.36170098	0.99107134	3.76E-21	9.94E-20	0.02	0.06	0.96	1.23	CHRD	chordin	chr3	protein_coding
ENSG00000164308.15	1.55554649	-0.5465785	0.00111944	0.0036441	0.04	0.04	0.15	0.18	ERAP2	endoplasmic reticulum aminopeptidase 2	chr5	protein_coding
ENSG00000187775.15	-9.8218423	2.97120986	7.94E-61	2.05E-58	1.1	0.75	0	0	DNAH17	dynein, axonemal, heavy chain 17	chr17	protein_coding
ENSG00000165757.8	1.50532389	-0.8185957	0.00274289	0.00820777	0.04	0.01	0.07	0.1	KIAA1462	KIAA1462	chr10	protein_coding
ENSG00000259230.1	-1.2186429	0.18077102	0.00215032	0.00655654	0.9	0.64	0.62	0.27	CTD-2555C1	--	chr14	lincRNA
ENSG00000280214.1	5.1052845	0.45805163	5.05E-20	1.22E-18	0.03	0.03	1.33	1.18	CTD-2012K1	--	chr16	TEC
ENSG00000155729.11	1.29963216	0.84446171	0.00034751	0.0012529	0.25	0.3	0.8	0.98	KCTD18	potassium channel tetramerization domain containing 18	chr2	protein_coding
ENSG00000107404.16	-2.5116135	8.03523354	6.11E-17	1.15E-15	127.86	94.97	24.64	27.58	DVL1	dishevelled segment polarity protein 1	chr1	protein_coding
ENSG00000226328.5	-1.5040336	3.61020975	5.75E-07	3.44E-06	7.55	6.5	3.35	3.25	NUP50-AS1	NUP50 antisense RNA 1 (head to head)	chr22	lincRNA
ENSG00000167491.16	-1.2831769	7.56023055	8.90E-06	4.37E-05	33.5	35.94	20.77	17.13	GATAD2A	GATA zinc finger domain containing 2A	chr19	protein_coding
ENSG00000112812.14	2.72634055	0.49248921	6.56E-11	6.71E-10	0.08	0.12	0.75	1.04	PRSS16	protease, serine, 16 (thymus)	chr6	protein_coding
ENSG00000143314.11	-2.3139942	8.15033513	8.22E-15	1.29E-13	443.99	382.68	113.12	108.4	MRPL24	mitochondrial ribosomal protein L24	chr1	protein_coding
ENSG00000143924.17	1.19673314	2.3910777	0.00012543	0.00049463	0.42	0.49	1.24	1.54	EML4	echinoderm microtubule associated protein like 4	chr2	protein_coding
ENSG00000263585.1	-5.2063823	-3.0367468	0.00538165	0.01488851	0.42	0.06	0	0	RP11-498C9	--	chr17	antisense
ENSG00000112282.16	1.44811956	1.76653152	9.99E-06	4.85E-05	0.19	0.32	0.83	1.04	MED23	mediator complex subunit 23	chr6	protein_coding
ENSG00000185686.16	-11.131038	5.11337617	3.98E-93	5.97E-90	18.51	18.93	0	0.02	PRAME	preferentially expressed antigen in melanoma	chr22	protein_coding
ENSG00000273980.1	5.58503228	-2.6679206	0.00086564	0.00288128	0	0	0.55	0.4	RP13-49I15.6	--	chr10	lincRNA
ENSG00000126775.8	1.90927527	3.25051058	7.01E-10	6.25E-09	0.73	0.62	2.84	3.95	ATG14	autophagy related 14	chr14	protein_coding
ENSG00000185513.13	1.08466269	1.23280533	0.00144812	0.00461689	0.2	0.42	0.74	1.01	L3MBTL1	l(3)mbt-like 1 (Drosophila)	chr20	protein_coding
ENSG00000188321.12	1.59453847	0.35985169	5.14E-05	0.00021906	0.12	0.09	0.44	0.42	ZNF559	zinc finger protein 559	chr19	protein_coding
ENSG00000131378.12	-5.4690317	3.29397643	1.40E-44	1.41E-42	5.27	4.72	0.22	0.09	RFTN1	raftlin, lipid raft linker 1	chr3	protein_coding
ENSG00000179399.12	-10.071663	1.02183466	2.91E-34	1.64E-32	1.13	1.15	0	0	GPC5	glypican 5	chr13	protein_coding
ENSG00000076043.8	-1.0375396	4.85607726	0.00035745	0.00128604	19.92	26.99	14.38	16.06	REXO2	RNA exonuclease 2	chr11	protein_coding
ENSG00000102900.11	1.32780136	8.36322061	4.31E-06	2.24E-05	16.67	15.81	54.3	54.34	NUP93	nucleoporin 93kDa	chr16	protein_coding
ENSG00000146729.8	1.46024763	2.16799547	5.20E-06	2.66E-05	1.03	0.86	2.52	4.48	GBAS	glioblastoma amplified sequence	chr7	protein_coding
ENSG00000075711.19	2.70939489	3.21180236	5.07E-17	9.61E-16	0.25	0.41	2.16	3.61	DLG1	discs, large homolog 1 (Drosophila)	chr3	protein_coding
ENSG00000235381.1	2.64300916	-2.305546	0.00281991	0.00840887	0	0.02	0.09	0.07	RP11-477D1	--	chr6	antisense
ENSG00000074410.12	-5.6861418	5.46859011	3.29E-56	6.80E-54	11.52	10.42	0.27	0.3	CA12	carbonic anhydrase XII	chr15	protein_coding
ENSG00000105193.7	-2.1320879	9.85322483	5.67E-13	7.30E-12	1941.85	1626.1	553.98	530.72	RPS16	ribosomal protein S16	chr19	protein_coding
ENSG00000116183.9	-8.8901016	-0.1369844	3.21E-21	8.52E-20	0.11	0.17	0	0	PAPPA2	pappalysin 2	chr1	protein_coding
ENSG00000168077.12	-2.342553	6.04754083	5.46E-15	8.73E-14	21.01	23.4	6.18	5.46	SCARA3	scavenger receptor class A, member 3	chr8	protein_coding
ENSG00000250305.7	-3.0881797	-1.1961396	5.22E-07	3.15E-06	0.02	0.08	0	0.01	KIAA1456	KIAA1456	chr8	protein_coding
ENSG00000100739.9	-1.8227997	-0.8304666	0.00015773	0.00060702	0.29	0.31	0.04	0.17	BDKRB1	bradykinin receptor B1	chr14	protein_coding
ENSG00000166923.9	-8.6520763	2.64619149	1.95E-54	3.59E-52	0.72	0.67	0	0	GREM1	gremlin 1, DAN family BMP antagonist	chr15	protein_coding
ENSG00000132394.9	-1.2127588	4.55285135	3.44E-05	0.00015163	12.44	11.31	7.3	6.33	EEFSEC	eukaryotic elongation factor, selenocysteine-tRNA-specific	chr3	protein_coding
ENSG00000206612.1	4.73235456	-3.1388833	0.01889873	0.04519557	0	0	1.66	0	SNORA2A	small nucleolar RNA, H/ACA box 2A	chr12	snoRNA
ENSG00000108602.16	-2.6956597	5.60375536	1.16E-18	2.53E-17	32.54	27.86	5.9	6.54	ALDH3A1	aldehyde dehydrogenase 3 family, member A1	chr17	protein_coding

ENSG00000152061.20	1.26437533	1.38225349	0.00018781	0.00071214	0.1	0.11	0.26	0.41	RABGAP1L	RAB GTPase activating protein 1-like	chr1	protein_coding
ENSG00000083097.13	1.41146788	-0.2775775	0.0011429	0.00370788	0.06	0.01	0.14	0.15	DOPEY1	dopey family member 1	chr6	protein_coding
ENSG00000270136.4	-1.7385341	7.33921631	2.80E-09	2.31E-08	135.89	112.47	51.91	47.22	MINOS1-NB	MINOS1-NBL1 readthrough	chr1	protein_coding
ENSG00000139496.14	1.44203329	1.77226553	1.17E-05	5.60E-05	0.15	0.17	0.46	0.69	NUPL1	nucleoporin like 1	chr13	protein_coding
ENSG00000144730.15	1.62134695	-0.9184932	0.0016871	0.00525645	0.02	0.02	0.1	0.07	IL17RD	interleukin 17 receptor D	chr3	protein_coding
ENSG00000116120.9	-1.9214131	4.2920095	1.59E-10	1.55E-09	7.45	7.44	2.24	3.01	FARSB	phenylalanyl-tRNA synthetase, beta subunit	chr2	protein_coding
ENSG00000137714.2	1.13712715	3.23719178	0.0001658	0.00063449	1.38	1.56	3.14	5.56	FDX1	ferredoxin 1	chr11	protein_coding
ENSG00000135074.14	-11.213042	2.16060455	2.86E-49	3.74E-47	0.97	1.09	0	0	ADAM19	ADAM metallopeptidase domain 19	chr5	protein_coding
ENSG00000170385.9	1.68444142	3.22157532	3.96E-08	2.78E-07	0.58	0.71	2.01	3.57	SLC30A1	solute carrier family 30 (zinc transporter), member 1	chr1	protein_coding
ENSG00000233621.1	-2.8049581	3.75113795	7.28E-19	1.61E-17	17.79	9.47	2.37	2.85	LINC01137	long intergenic non-protein coding RNA 1137	chr1	antisense
ENSG00000270000.1	-6.0981358	-2.5020829	8.36E-05	0.00034052	0.43	0.53	0	0	RP3-449M8.9	--	chr14	lincRNA
ENSG00000129911.7	-1.1976919	5.14412882	3.84E-05	0.00016793	15.02	11.91	7.71	7.96	KLF16	Kruppel-like factor 16	chr19	protein_coding
ENSG00000144843.10	-1.5803896	-0.9781302	0.00128412	0.00413416	0.14	0.1	0.04	0.06	ADPRH	ADP-ribosylarginine hydrolase	chr3	protein_coding
ENSG00000280191.2	-1.5066326	0.47556014	4.83E-05	0.00020679	2.06	2.5	0.94	1.18	CH507-42P1	--	chr21	lincRNA
ENSG00000059769.18	1.76424561	1.84207039	1.10E-07	7.32E-07	0.48	0.65	2.16	2.98	DNAJC25	DnaJ (Hsp40) homolog, subfamily C , member 25	chr9	protein_coding
ENSG00000166670.8	6.62967961	-1.9404541	6.25E-07	3.72E-06	0	0	0.27	0.19	MMP10	matrix metallopeptidase 10	chr11	protein_coding
ENSG00000174996.10	-1.0140532	6.60597253	0.00042514	0.00150739	23.41	24.56	16.35	15.26	KLC2	kinesin light chain 2	chr11	protein_coding
ENSG00000083520.13	1.98359767	1.81560322	3.34E-09	2.74E-08	0.1	0.1	0.4	0.69	DIS3	DIS3 exosome endoribonuclease and 3'-5' exoribonuclease	chr13	protein_coding
ENSG00000135926.11	-1.1883822	6.77780033	3.84E-05	0.00016782	41.15	39.61	23.81	23.4	TMBIM1	transmembrane BAX inhibitor motif containing 1	chr2	protein_coding
ENSG00000213625.7	1.66765965	3.01394801	7.28E-08	4.98E-07	0.55	0.75	2.14	3.4	LEPROT	leptin receptor overlapping transcript	chr1	protein_coding
ENSG00000185482.6	1.63018323	0.67520684	1.39E-05	6.58E-05	0.18	0.55	1.3	1.68	STAC3	SH3 and cysteine rich domain 3	chr12	protein_coding
ENSG00000196199.12	2.09421218	3.90357095	7.85E-12	8.90E-11	0.78	1.38	5.1	7.21	MPHOSPH8	M-phase phosphoprotein 8	chr13	protein_coding
ENSG00000279689.1	1.91328772	0.87827076	2.50E-07	1.58E-06	0.19	0.13	0.75	0.91	RP11-574K1	--	chr10	TEC
ENSG00000136824.17	2.89843163	1.9520197	2.20E-16	3.94E-15	0.12	0.11	0.68	1.61	SMC2	structural maintenance of chromosomes 2	chr9	protein_coding
ENSG00000183340.6	1.94233236	1.7485703	8.51E-09	6.59E-08	0.38	0.38	1.37	2.56	JRKL	JRK-like	chr11	protein_coding
ENSG00000118523.5	-4.2250596	4.81770572	1.21E-36	7.58E-35	17.54	19.57	1.23	1.41	CTGF	connective tissue growth factor	chr6	protein_coding
ENSG00000128708.11	2.19774526	2.77703579	7.19E-12	8.19E-11	0.72	1.69	5.28	9.5	HAT1	histone acetyltransferase 1	chr2	protein_coding
ENSG00000001460.16	-1.6488173	2.62749001	1.25E-07	8.27E-07	2.41	2.8	0.95	1.27	STPG1	sperm-tail PG-rich repeat containing 1	chr1	protein_coding
ENSG00000204381.10	5.08251936	-2.9605404	0.00538165	0.01488851	0	0	0.09	0.01	LAYN	layilin	chr11	protein_coding
ENSG00000204392.9	-1.1966136	5.67144322	3.67E-05	0.00016108	70.07	60.95	38.54	37.65	LSM2	LSM2 homolog, U6 small nuclear RNA associated (S. cerevisiae)	chr6	protein_coding
ENSG00000008083.12	-1.2473161	5.12150054	1.86E-05	8.57E-05	6.07	7.41	3.9	3.66	JARID2	jumonji, AT rich interactive domain 2	chr6	protein_coding
ENSG00000142347.15	-2.8841224	-1.3381041	4.68E-06	2.42E-05	0.15	0.09	0.02	0.02	MYO1F	myosin IF	chr19	protein_coding
ENSG00000236404.7	-3.206034	-1.8865793	2.54E-05	0.00011474	0.05	0.1	0.01	0.01	VLDLR-AS1	VLDLR antisense RNA 1	chr9	antisense
ENSG00000099624.6	-1.3446877	8.47920193	3.27E-06	1.74E-05	426.48	401.59	226.79	207.19	ATP5D	ATP synthase, H+ transporting, mitochondrial F1 complex, delta subunit	chr19	protein_coding
ENSG00000171806.10	1.53376496	-0.5643521	0.00138456	0.00443488	0.17	0.14	0.69	0.53	METTL18	methyltransferase like 18	chr1	protein_coding
ENSG00000226472.6	-1.7720313	0.24552871	5.78E-06	2.93E-05	0.76	0.7	0.31	0.26	RP11-551L1	--	chr12	antisense
ENSG00000224945.1	2.61496029	-1.1469007	1.40E-05	6.61E-05	0.18	0.1	1.31	1.07	RP11-82L18.	--	chr9	sense_intronic
ENSG00000100605.15	-1.0227201	7.16260942	0.00037319	0.00133745	25.29	25	16.89	16.06	ITPK1	inositol-tetrakisphosphate 1-kinase	chr14	protein_coding
ENSG00000042317.15	1.15134037	0.17001428	0.00369859	0.01067581	0.15	0.3	0.47	0.83	SPATA7	spermatogenesis associated 7	chr14	protein_coding
ENSG00000227014.4	3.21948151	-1.9245043	6.40E-05	0.00026805	0.06	0	0.64	0.17	AC007285.6	--	chr7	antisense
ENSG00000130775.14	-6.8252312	-1.9700183	2.24E-07	1.42E-06	0.14	0.11	0	0	THEMIS2	thymocyte selection associated family member 2	chr1	protein_coding
ENSG00000271870.1	6.14153525	-2.300654	2.70E-05	0.00012094	0	0	0.91	0.28	RP11-97C16	--	chr3	antisense
ENSG00000128710.5	12.7253453	3.69320572	1.23E-69	4.77E-67	0	0	11.89	15.96	HOXD10	homeobox D10	chr2	protein_coding
ENSG00000249614.1	5.54436966	0.5023802	3.43E-22	9.74E-21	0	0.1	2.41	4.39	RP11-703G6	--	chr4	lincRNA
ENSG00000144366.14	-5.3742532	-2.9467828	0.00290522	0.00856312	0.03	0.02	0	0	GULP1	GULP, engulfment adaptor PTB domain containing 1	chr2	protein_coding
ENSG00000104365.12	-1.9220094	5.64836861	8.18E-11	8.26E-10	13.3	14.29	4.74	4.96	IKBKB	inhibitor of kappa light polypeptide gene enhancer in B-cells, kinase beta	chr8	protein_coding
ENSG00000186493.10	11.3328508	4.75226438	4.17E-83	3.33E-80	0	0.01	31.9	28.08	C5orf38	chromosome 5 open reading frame 38	chr5	protein_coding
ENSG00000182919.13	1.48897241	1.25690225	1.67E-05	7.79E-05	0.25	0.19	0.56	1.12	C11orf54	chromosome 11 open reading frame 54	chr11	protein_coding
ENSG00000261560.1	6.30985008	-2.1747134	8.95E-06	4.38E-05	0	0	0.13	0.29	RP11-166B2.	--	chr16	sense_intronic
ENSG00000108187.14	1.17121282	-0.0027545	0.00485861	0.01370081	0.04	0.25	0.34	0.54	PBLD	phenazine biosynthesis-like protein domain containing	chr10	protein_coding
ENSG00000228144.2	1.45224401	2.68016363	2.87E-06	1.55E-05	3.07	2.48	7.62	12.76	RP11-745O1	--	chr12	protein_coding
ENSG00000100478.13	1.40945529	1.07245178	5.93E-05	0.0002496	0.17	0.09	0.43	0.48	AP4S1	adaptor-related protein complex 4, sigma 1 subunit	chr14	protein_coding
ENSG00000198130.13	1.0412584	1.77857591	0.00128361	0.00413307	0.7	0.52	1.64	1.71	HIBCH	3-hydroxyisobutyryl-CoA hydrolase	chr2	protein_coding
ENSG00000229372.1	4.15135565	-1.9101722	1.13E-05	5.42E-05	0.04	0	0.46	0.66	SZT2-AS1	SZT2 antisense RNA 1	chr1	antisense
ENSG00000170899.9	-5.9750113	0.00918509	3.94E-21	1.04E-19	1.4	1.36	0.06	0	GSTA4	glutathione S-transferase alpha 4	chr6	protein_coding
ENSG00000129675.14	4.91020659	-3.0511096	0.0100455	0.02582742	0	0	0.03	0.01	ARHGEF6	Rac/Cdc42 guanine nucleotide exchange factor (GEF) 6	chrX	protein_coding
ENSG00000270562.1	5.20747907	-2.8915189	0.00538165	0.01488851	0	0	0.05	0.07	RP11-154H2	--	chr3	lincRNA
ENSG00000125731.11	-1.4489975	3.58244961	1.44E-06	8.11E-06	6.35	4.9	3.08	2.4	SH2D3A	SH2 domain containing 3A	chr19	protein_coding
ENSG00000148110.14	1.64287895	3.99831166	4.35E-08	3.05E-07	1.58	2.07	6.05	9.17	HIATL1	hippocampus abundant transcript-like 1	chr9	protein_coding
ENSG00000277363.3	1.58984548	0.95077603	9.17E-06	4.48E-05	0.1	0.1	0.42	0.41	SRCIN1	SRC kinase signaling inhibitor 1	chr17	protein_coding
ENSG00000188816.3	-9.0312512	-0.0003168	1.34E-22	3.88E-21	0.92	1.04	0	0	HMX2	H6 family homeobox 2	chr10	protein_coding
ENSG00000244649.3	3.13675198	-1.9777454	0.00060878	0.00209711	0	0.03	0.2	0.14	CTD-2377D2	--	chr17	lincRNA
ENSG00000112559.12	3.69348609	-0.782237	2.06E-09	1.73E-08	0.06	0	0.66	0.45	MDFI	MyoD family inhibitor	chr6	protein_coding
ENSG00000166582.8	-1.1870775	5.65568663	4.23E-05	0.00018375	31.72	30.81	18.78	17.78	CENPV	centromere protein V	chr17	protein_coding
ENSG00000277459.1	2.93652693	0.85319893	3.53E-13	4.64E-12	0.42	0.58	6.09	4.23	RP11-732A2	--	chr11	lincRNA
ENSG00000187098.13	1.54618496	0.11331012	0.00016219	0.00062317	0.11	0.02	0.2	0.32	MITF	microphthalmia-associated transcription factor	chr3	protein_coding
ENSG00000198133.7	3.5160821	-1.7052417	2.93E-05	0.00013031	0	0.01	0.15	0.07	TMEM229B	transmembrane protein 229B	chr14	protein_coding
ENSG00000124713.5	2.20192936	-1.6531997	0.00158671	0.00496952	0.07	0.05	0.3	0.51	GNMT	glycine N-methyltransferase	chr6	protein_coding
ENSG00000233117.2	-5.3787292	-2.9446861	0.00290522	0.00856312	0	0.04	0	0	LINC00702	long intergenic non-protein coding RNA 702	chr10	lincRNA
ENSG00000130147.14	-1.1499321	5.86147294	7.06E-05	0.0002928	10.83	12.8	7.04	7.14	SH3BP4	SH3-domain binding protein 4	chr2	protein_coding
ENSG00000165807.6	1.93876567	-1.2929263	0.00103764	0.00339765	0.11	0.04	0.41	0.41	PPP1R36	protein phosphatase 1, regulatory subunit 36	chr14	protein_coding
ENSG00000186432.7	1.24466882	3.41719354	3.47E-05	0.00015293	0.51	0.62	1.25	2.35	KPNA4	karyopherin alpha 4 (importin alpha 3)	chr3	protein_coding
ENSG00000163406.9	3.03045527	-2.0542498	0.00094481	0.00312013	0	0	0.09	0.03	SLC15A2	solute carrier family 15 (oligopeptide transporter), member 2	chr3	protein_coding
ENSG00000141367.10	1.24729992	5.80192071	1.72E-05	8.02E-05	3.2	2.94	7.87	11.66	CLTC	clathrin, heavy chain (Hc)	chr17	protein_coding
ENSG00000162104.8	-1.5479662	6.70639114	1.08E-07	7.20E-07	14.71	17.94	7.79	7.05	ADCY9	adenylate cyclase 9	chr16	protein_coding
ENSG00000072818.10	-1.1148516	-0.2154158	0.00665587	0.0180614	0.39	0.24	0.17	0.22	ACAP1	ArfGAP with coiled-coil, ankyrin repeat and PH domains 1	chr17	protein_coding
ENSG00000253372.4	5.21249443	-2.8887793	0.00538165	0.01488851	0	0	0.21	0.18	RP11-44N11	--	chr8	sense_intronic
ENSG00000146966.11	1.90750781	-1.6482872	0.00486779	0.01371541	0.03	0.01	0.07	0.1	DENND2A	DENN/MADD domain containing 2A	chr7	protein_coding
ENSG00000179083.6	-2.753646	-2.573854	0.003237	0.00945156	0.02	0.08	0	0.02	FAM133A	family with sequence similarity 133, member A	chrX	protein_coding
ENSG00000188659.8	3.98564737	-0.8706941	2.54E-09	2.11E-08	0	0.02	0.14	0.33	FAM154B	--	chr15	protein_coding
ENSG00000204183.1	4.8843127	-3.0650858	0.01889873	0.04519557	0	0	0.02	0.11	GDF5OS	growth differentiation factor 5 opposite strand	chr20	protein_coding
ENSG00000168014.15	1.19170419	4.79864661	4.50E-05	0.00019489	1.38	1.52	4.2	4.62	C2CD3	C2 calcium-dependent domain containing 3	chr11	protein_coding
ENSG00000260001.5	-1.4554682	3.35620566	1.34E-06	7.54E-06	10.63	8.59	3.74	5.64	TGFBR3L	transforming growth factor, beta receptor III-like	chr19	protein_coding
ENSG00000116001.14	2.89599298	2.30625351	3.17E-17	6.10E-16	0.16	0.22	1.62	2.15	TIA1	TIA1 cytotoxic granule-associated RNA binding protein	chr2	protein_coding
ENSG00000136371.8	1.47768551	3.69840074	8.55E-07	4.98E-06	6.58	5.56	20.06	25.16	MTHFS	5,10-methenyltetrahydrofolate synthetase (5-formyltetrahydrofolate cyclo-ligase)	chr15	protein_coding
ENSG00000118690.11	1.41419188	0.50309765	0.00017448	0.00066571	0.27	0.04	0.53	0.59	ARMC2	armadillo repeat containing 2	chr6	protein_coding
ENSG00000255150.1	2.03306287	-0.2010901	8.13E-06	4.03E-05	0.22	0.12	0.72	1.13	EID3	EP300 interacting inhibitor of differentiation 3	chr12	protein_coding
ENSG00000109452.11	-3.0948131	-1.6606516	8.20E-06	4.05E-05	0.04	0.02	0	0.01	INPP4B	inositol polyphosphate-4-phosphatase, type II, 105kDa	chr4	protein_coding
ENSG00000119844.13	1.90920402	1.74657202	1.61E-08	1.20E-07	0.17	0.37	1.04	1.66	AFTPH	aftiphilin	chr2	protein_coding
ENSG00000072195.13	-2.3804529	4.07996146	9.38E-15	1.47E-13	1.9	1.69	0.43	0.49	SPEG	SPEG complex locus	chr2	protein_coding
ENSG00000266412.4	1.20756912	4.28237284	4.12E-05	0.00017959	2.24	2.41	5.4	8.98	NCOA4	nuclear receptor coactivator 4	chr10	protein_coding
ENSG00000112249.12	2.31602323	1.87529401	1.18E-11	1.31E-10	0.09	0.07	0.44	0.69	ASCC3	activating signal cointegrator 1 complex subunit 3	chr6	protein_coding
ENSG00000244752.2	-3.8958825	1.55874699	2.85E-23	8.69E-22	5.22	4.86	0.43	0.46	CRYBB2	crystallin, beta B2	chr22	protein_coding
ENSG00000156232.7	2.71423782	3.97804591	6.47E-18	1.32E-16	0.46	0.86	5.53	5.96	WHAMM	WAS protein homolog associated with actin, golgi membranes and microtubules	chr15	protein_coding
ENSG00000164938.12	2.34364593	0.11420756	7.03E-08	4.82E-07	0.04	0.05	0.23	0.4	TP53INP1	tumor protein p53 inducible nuclear protein 1	chr8	protein_coding
ENSG00000139971.14	5.32944093	-2.821228	0.00290522	0.00856312	0	0	0.01	0.04	C14orf37	chromosome 14 open reading frame 37	chr14	protein_coding
ENSG00000215808.2	-5.3742532	-2.9467828	0.00290522	0.00856312	0.09	0.07	0	0	LINC01139	long intergenic non-protein coding RNA 1139	chr1	lincRNA
ENSG00000124216.3	2.59570647	3.52789493	2.52E-16	4.50E-15	1.24	1.88	13.15	11.9	SNAI1	snail family zinc finger 1	chr20	protein_coding
ENSG00000147813.14	-2.3355802	5.75158156	7.05E-15	1.12E-13	37.89	29.84	9.82	8.04	NAPRT	nicotinate phosphoribosyltransferase	chr8	protein_coding
ENSG00000117971.10	3.42172985	-1.7682616	6.90E-05	0.00028699	0	0.02	0.09	0.24	CHRNB4	cholinergic receptor, nicotinic, beta 4 (neuronal)	chr15	protein_coding
ENSG00000224652.1	5.20747907	-2.8915189	0.00538165	0.01488851	0	0	0.07	0.09	LINC00885	long intergenic non-protein coding RNA 885	chr3	lincRNA
ENSG00000153930.9	-6.8830937	-1.9248975	1.35E-07	8.87E-07	0.08	0.1	0	0	ANKFN1	ankyrin-repeat and fibronectin type III domain containing 1	chr17	protein_coding
ENSG00000177200.14	3.05010645	0.54689253	1.15E-12	1.43E-11	0.02	0.02	0.13	0.27	CHD9	chromodomain helicase DNA binding protein 9	chr16	protein_coding
ENSG00000267317.2	-1.1700688	4.16325913	7.10E-05	0.00029435	21.75	22.27	14.52	11.48	CTB-25B13.1	--	chr19	antisense
ENSG00000171045.13	1.79773453	3.32648123	4.76E-09	3.81E-08	1.15	1.1	5.61	4.83	TSNARE1	t-SNARE domain containing 1	chr8	protein_coding
ENSG00000083937.7	2.69641771	2.51518309	6.28E-16	1.08E-14	0.52	0.4	2.74	5.3	CHMP2B	charged multivesicular body protein 2B	chr3	protein_coding
ENSG00000143891.15	-8.6708306	3.19382184	5.61E-61	1.48E-58	5.6	6.33	0.04	0	GALM	galactose mutarotase (aldose 1-epimerase)	chr2	protein_coding
ENSG00000231925.10	-1.2417388	7.19251272	1.71E-05	7.94E-05	39.9	39.45	24.71	19.86	TAPBP	TAP binding protein (tapasin)	chr6	protein_coding
ENSG00000100209.8	-1.0221138	3.16142246	0.00062933	0.00216073	7.09	10.16	3.68	7.68	HSCB	HscB mitochondrial iron-sulfur cluster co-chaperone	chr22	protein_coding
ENSG00000237289.8	2.6170078	0.8026248	3.13E-11	3.32E-10	0.23	0.14	1.5	1.58	CKMT1B	creatine kinase, mitochondrial 1B	chr15	protein_coding
ENSG00000260766.1	1.48517258	-1.2138974	0.0105162	0.02693027	0.09	0.08	0.3	0.33	RP11-226L1	--	chr1	sense_overlapping
ENSG00000113163.14	1.39464413	1.68819741	2.34E-05	0.00010604	0.16	0.33	0.84	0.85	COL4A3BP	collagen, type IV, alpha 3 (Goodpasture antigen) binding protein	chr5	protein_coding
ENSG00000111057.9	-1.0926188	10.7618167	0.00014108	0.00055006	956.5	948.89	627.66	560.87	KRT18	keratin 18, type I	chr12	protein_coding
ENSG00000105792.18	7.61364781	-1.1291154	9.11E-12	1.03E-10	0	0	0.07	0.33	CFAP69	cilia and flagella associated protein 69	chr7	protein_coding
ENSG00000146701.10	-1.3359225	8.3091146	3.77E-06	1.98E-05	147.09	125.47	69.56	74.53	MDH2	malate dehydrogenase 2, NAD (mitochondrial)	chr7	protein_coding
ENSG00000142408.2	-7.286838	3.43708714	2.90E-59	6.57E-57	1.71	2.29	0	0.03	CACNG8	calcium channel, voltage-dependent, gamma subunit 8	chr19	protein_coding
ENSG00000143486.14	-1.4267224	6.30976082	9.21E-07	5.33E-06	49.34	43.97	22.56	23.74	EIF2D	eukaryotic translation initiation factor 2D	chr1	protein_coding
ENSG00000267058.1	4.72173804	-3.1445547	0.01889873	0.04519557	0	0	0.07	0.02	RP11-15A1.3	--	chr19	lincRNA
ENSG00000069482.6	-2.5037719	4.896741	2.22E-16	3.99E-15	54.18	46.01	9.55	14.08	GAL	galanin/GMAP prepropeptide	chr11	protein_coding
ENSG00000151150.19	1.1359766	0.35546062	0.00357643	0.01035688	0.03	0.03	0.06	0.13	ANK3	ankyrin 3, node of Ranvier (ankyrin G)	chr10	protein_coding
ENSG00000092020.9	1.02196041	1.612195	0.00177387	0.00549607	0.54	0.83	1.31	2.4	PPP2R3C	protein phosphatase 2, regulatory subunit B'', gamma	chr14	protein_coding
ENSG00000068784.11	2.3009161	0.55242992	8.94E-09	6.90E-08	0.08	0.12	0.53	0.81	SRBD1	S1 RNA binding domain 1	chr2	protein_coding
ENSG00000204802.3	2.924753	0.27004454	6.06E-11	6.22E-10	0.05	0.23	1.71	1.16	RP11-111F5.	--	chr9	lincRNA
ENSG00000214194.7	1.1990816	1.20228389	0.00049587	0.00173253	1.02	1.01	2.39	3.84	LINC00998	long intergenic non-protein coding RNA 998	chr7	lincRNA
ENSG00000173153.12	-2.2699224	6.85799702	2.66E-14	3.96E-13	68.44	58.57	17.86	17.23	ESRRA	estrogen-related receptor alpha	chr11	protein_coding
ENSG00000125430.7	-3.7359271	0.03393612	1.11E-14	1.72E-13	0.28	0.28	0.04	0.02	HS3ST3B1	heparan sulfate (glucosamine) 3-O-sulfotransferase 3B1	chr17	protein_coding
ENSG00000163412.11	3.85828255	2.1314232	1.02E-24	3.48E-23	0.03	0.06	0.61	1.01	EIF4E3	eukaryotic translation initiation factor 4E family member 3	chr3	protein_coding
ENSG00000259422.1	-2.0730175	-1.5537436	0.00086862	0.00288921	0.16	0.17	0.1	0.01	RP11-593F2	--	chr15	antisense
ENSG00000240288.6	1.90142376	0.0868631	6.96E-06	3.48E-05	0.06	0.33	0.97	0.97	GHRLOS	ghrelin opposite strand/antisense RNA	chr3	antisense
ENSG00000266997.2	3.91871156	2.08801591	7.16E-25	2.46E-23	0.57	0.3	7.84	10.07	RP11-886H2	--	chr18	protein_coding
ENSG00000221886.3	2.54667332	-0.1299845	3.28E-08	2.33E-07	0.08	0.05	0.39	0.64	ZBED8	zinc finger, BED-type containing 8	chr5	protein_coding
ENSG00000180806.4	1.17419366	4.51710645	6.11E-05	0.00025708	6.29	8.3	22.9	20.85	HOXC9	homeobox C9	chr12	protein_coding
ENSG00000154930.13	3.61202233	2.42987096	6.52E-24	2.07E-22	0.2	0.02	1.73	1.93	ACSS1	acyl-CoA synthetase short-chain family member 1	chr20	protein_coding
ENSG00000135124.13	1.44554496	4.83712771	8.63E-07	5.02E-06	5.89	6.02	22.21	20.99	P2RX4	purinergic receptor P2X, ligand gated ion channel, 4	chr12	protein_coding
ENSG00000114378.15	-6.2353684	3.44422662	2.81E-52	4.56E-50	5.68	5.55	0.05	0.14	HYAL1	hyaluronoglucosaminidase 1	chr3	protein_coding
ENSG00000180855.14	7.1254824	-1.5429511	4.48E-09	3.59E-08	0	0	0.14	0.28	ZNF443	zinc finger protein 443	chr19	protein_coding
ENSG00000181192.10	1.44001522	5.01755096	9.06E-07	5.25E-06	2.68	2.78	9.23	10.54	DHTKD1	dehydrogenase E1 and transketolase domain containing 1	chr10	protein_coding
ENSG00000174796.11	2.0270813	-0.2775884	1.10E-05	5.28E-05	0.04	0.04	0.15	0.27	THAP6	THAP domain containing 6	chr4	protein_coding
ENSG00000119280.15	-1.1865336	5.40600694	4.38E-05	0.00018979	9.88	11.33	6.35	6.05	C1orf198	chromosome 1 open reading frame 198	chr1	protein_coding
ENSG00000275126.1	5.90476671	-2.4637601	0.00014842	0.00057423	0	0	1.44	0.17	HIST1H4L	histone cluster 1, H4l	chr6	protein_coding
ENSG00000270020.1	-5.7827447	-2.7070885	0.00047761	0.00167191	0.03	0.06	0	0	RP11-463O9	--	chr16	lincRNA
ENSG00000186871.6	2.67036689	1.16203685	1.63E-12	2.01E-11	0.16	0.05	0.66	1.17	ERCC6L	excision repair cross-complementation group 6-like	chrX	protein_coding
ENSG00000172458.4	-1.6510286	-0.610267	0.00021339	0.0008028	0.4	0.41	0.09	0.25	IL17D	interleukin 17D	chr13	protein_coding
ENSG00000134186.10	1.33327965	4.38941856	6.23E-06	3.14E-05	1.74	2.53	5.15	9.24	PRPF38B	pre-mRNA processing factor 38B	chr1	protein_coding
ENSG00000261542.1	5.80179215	-2.5306812	0.00026535	0.0009747	0	0	0.17	0.05	RP11-16E18.	--	chr8	lincRNA
ENSG00000261236.4	-1.1591805	8.21289799	5.63E-05	0.0002378	150.71	137.33	89.73	81.97	BOP1	block of proliferation 1	chr8	protein_coding
ENSG00000177917.9	1.9639002	1.14830748	3.90E-08	2.74E-07	0.39	0.1	0.91	1.69	ARL6IP6	ADP-ribosylation factor-like 6 interacting protein 6	chr2	protein_coding
ENSG00000244405.6	-1.4997919	5.25204491	3.03E-07	1.89E-06	10.28	10.77	4.93	4.98	ETV5	ets variant 5	chr3	protein_coding
ENSG00000132549.17	1.61136659	1.66449948	1.32E-06	7.46E-06	0.07	0.09	0.33	0.35	VPS13B	vacuolar protein sorting 13 homolog B (yeast)	chr8	protein_coding
ENSG00000251364.5	5.58023914	-2.6705827	0.00086564	0.00288128	0	0	0.03	0.03	CTD-2516F1	--	chr11	antisense
ENSG00000115355.14	5.63104815	1.87867291	2.74E-34	1.55E-32	0	0.02	0.37	1.17	CCDC88A	coiled-coil domain containing 88A	chr2	protein_coding
ENSG00000107789.14	3.50953131	2.85592011	4.29E-24	1.39E-22	0.18	0.46	3.17	6.65	MINPP1	multiple inositol-polyphosphate phosphatase 1	chr10	protein_coding
ENSG00000179344.15	7.32962882	1.66629947	8.79E-38	5.89E-36	0.03	0	3.83	3.51	HLA-DQB1	major histocompatibility complex, class II, DQ beta 1	chr6	protein_coding
ENSG00000260404.2	1.06782357	1.64017838	0.00104109	0.00340802	0.3	0.43	1.2	0.81	RP11-384K6.	--	chr4	lincRNA
ENSG00000238266.1	-7.0496683	2.741634	4.83E-50	6.61E-48	2.58	4.46	0.07	0	LINC00707	long intergenic non-protein coding RNA 707	chr10	lincRNA
ENSG00000268584.1	2.01858971	-1.5716669	0.00244913	0.00738591	0.2	0	0.16	0.96	RP11-464F9.	--	chr10	antisense
ENSG00000107819.12	-1.286792	6.00258003	9.19E-06	4.49E-05	25.76	21.72	14.53	11.34	SFXN3	sideroflexin 3	chr10	protein_coding
ENSG00000172548.13	-7.5997001	-1.3261144	2.11E-11	2.27E-10	0.23	0.13	0	0	NIPAL4	NIPA-like domain containing 4	chr5	protein_coding
ENSG00000196196.2	-5.5081196	2.5376396	1.99E-40	1.56E-38	10.7	8.68	0.34	0.23	HRCT1	histidine rich carboxyl terminus 1	chr9	protein_coding
ENSG00000254870.4	-2.1523199	-1.854862	0.00206772	0.00632398	0.08	0.22	0.03	0.05	ATP6V1G2-D	ATP6V1G2-DDX39B readthrough (NMD candidate)	chr6	protein_coding
ENSG00000160932.9	1.45079757	9.06623146	5.49E-07	3.29E-06	108.24	108.39	414.24	373.59	LY6E	lymphocyte antigen 6 complex, locus E	chr8	protein_coding
ENSG00000153237.16	3.38663858	-2.4369723	0.00190233	0.00584723	0.01	0	0.01	0.12	CCDC148	coiled-coil domain containing 148	chr2	protein_coding
ENSG00000173320.8	2.2873299	1.04384329	8.93E-10	7.87E-09	0.03	0.07	0.34	0.34	STOX2	storkhead box 2	chr4	protein_coding
ENSG00000241058.2	1.46843239	0.78826995	6.70E-05	0.00027959	0.28	0.36	0.83	1.53	NSUN6	NOP2/Sun domain family, member 6	chr10	protein_coding
ENSG00000243902.5	-2.6661838	-0.4603076	1.85E-08	1.37E-07	0.43	0.56	0.02	0.18	RP1-63G5.5	--	chr22	sense_overlapping
ENSG00000057252.11	2.98805841	4.71075254	1.77E-21	4.80E-20	0.56	0.81	5.81	8.69	SOAT1	sterol O-acyltransferase 1	chr1	protein_coding
ENSG00000150938.8	1.17889652	4.9354394	5.30E-05	0.00022496	2.22	2.91	5.7	9.85	CRIM1	cysteine rich transmembrane BMP regulator 1 (chordin-like)	chr2	protein_coding
ENSG00000179222.16	-1.789296	-0.6179818	0.00012395	0.0004892	0.29	0.2	0.09	0.1	MAGED1	melanoma antigen family D1	chrX	protein_coding
ENSG00000144648.13	3.67854412	0.07980291	2.33E-13	3.12E-12	0.04	0.04	0.78	0.52	ACKR2	atypical chemokine receptor 2	chr3	protein_coding
ENSG00000179051.12	-1.3154264	8.5496693	5.22E-06	2.67E-05	102.1	107.76	57.9	54.31	RCC2	regulator of chromosome condensation 2	chr1	protein_coding
ENSG00000100916.12	1.65620272	0.75748201	7.38E-06	3.68E-05	0.14	0.33	0.59	1.38	BRMS1L	breast cancer metastasis-suppressor 1-like	chr14	protein_coding
ENSG00000100941.7	2.14356929	6.13956046	5.95E-13	7.64E-12	4.16	7.13	23.3	43.53	PNN	pinin, desmosome associated protein	chr14	protein_coding
ENSG00000152219.4	1.62060983	1.43491466	2.20E-06	1.20E-05	0.34	0.51	1.2	2.31	ARL14EP	ADP-ribosylation factor-like 14 effector protein	chr11	protein_coding
ENSG00000269646.1	-4.806998	-3.2310942	0.01889873	0.04519557	0.17	0.17	0	0	CTD-2331H1	--	chr19	lincRNA
ENSG00000146540.13	-1.4328784	7.39274386	7.77E-07	4.55E-06	93.74	89.37	45.76	44.59	C7orf50	chromosome 7 open reading frame 50	chr7	protein_coding
ENSG00000169891.16	-1.0713913	1.26711394	0.00151066	0.0047965	0.34	0.31	0.23	0.18	REPS2	RALBP1 associated Eps domain containing 2	chrX	protein_coding
ENSG00000204616.9	7.07923632	-1.5788187	7.18E-09	5.62E-08	0	0	0.14	0.39	TRIM31	tripartite motif containing 31	chr6	protein_coding
ENSG00000275091.1	1.74669609	-0.734304	0.00039049	0.00139484	0.15	0.97	3.55	1.47	CTB-55O6.1	--	chr19	antisense
ENSG00000279338.1	7.47784289	-1.2555988	4.96E-11	5.14E-10	0	0	0.03	0.02	RP1-309I22.2	--	chr22	sense_overlapping
ENSG00000125885.12	2.76149811	2.59205504	1.42E-16	2.60E-15	0.13	0.2	1.25	1.65	MCM8	minichromosome maintenance complex component 8	chr20	protein_coding
ENSG00000120159.10	1.47527656	2.17545115	3.92E-06	2.05E-05	0.51	0.78	1.29	3.5	CAAP1	caspase activity and apoptosis inhibitor 1	chr9	protein_coding

ENSG00000272853.1	-2.1864719	-1.106912	4.42E-05	0.00019159	0.75	0.37	0.16	0.16	RP11-398C1:	--	chr10	lincRNA
ENSG00000267645.4	1.12482082	-0.2590193	0.00951434	0.02486403	0.79	1.11	2.52	3.01	POLR2J2	polymerase (RNA) II (DNA directed) polypeptide J2	chr7	protein_coding
ENSG00000172954.12	2.14029732	2.3822464	5.27E-11	5.45E-10	0.24	0.21	0.96	1.65	LCLAT1	lysocardiolipin acyltransferase 1	chr2	protein_coding
ENSG00000276727.1	5.46945885	-2.7387023	0.00158008	0.00495521	0	0	0.27	0.23	RP11-123K3.	--	chr12	antisense
ENSG00000163565.17	1.29797585	3.04674667	2.11E-05	9.64E-05	1.06	1.56	3.81	4.75	IFI16	interferon, gamma-inducible protein 16	chr1	protein_coding
ENSG00000182022.16	-1.1822637	3.539409	7.22E-05	0.00029894	1.82	2.16	1.1	1.24	CHST15	carbohydrate (N-acetylgalactosamine 4-sulfate 6-O) sulfotransferase 15	chr10	protein_coding
ENSG00000148300.10	-1.451263	7.84480142	5.58E-07	3.35E-06	108.71	117.09	56.5	53.38	REXO4	REX4, RNA exonuclease 4 homolog (S. cerevisiae)	chr9	protein_coding
ENSG00000111711.8	1.19386944	1.48376547	0.00035738	0.00128596	0.44	0.37	0.97	1.5	GOLT1B	golgi transport 1B	chr12	protein_coding
ENSG00000175274.17	-1.1442534	6.06915462	7.54E-05	0.00031153	14.68	15.54	9.35	8.85	TP53I11	tumor protein p53 inducible protein 11	chr11	protein_coding
ENSG00000256690.1	-1.6376954	-1.9860808	0.01967235	0.04677036	0.45	0.19	0.17	0.11	RP11-727F1:	--	chr11	antisense
ENSG00000136010.12	-3.6059342	-1.2754036	4.26E-08	2.99E-07	0.04	0.12	0	0.01	ALDH1L2	aldehyde dehydrogenase 1 family, member L2	chr12	protein_coding
ENSG00000224078.11	-8.3637944	-0.6353741	1.25E-16	2.30E-15	0.03	0.03	0	0	SNHG14	small nucleolar RNA host gene 14	chr15	processed_transcript
ENSG00000153827.12	-6.3928616	3.2409113	8.67E-52	1.29E-49	1.39	1.58	0.03	0.02	TRIP12	thyroid hormone receptor interactor 12	chr2	protein_coding
ENSG00000090661.10	7.08517016	2.33359864	6.06E-44	5.83E-42	0	0.06	5.96	5.48	CERS4	ceramide synthase 4	chr19	protein_coding
ENSG00000175745.10	-2.9849489	2.98096537	9.06E-20	2.14E-18	2.74	3.35	0.39	0.63	NR2F1	nuclear receptor subfamily 2, group F, member 1	chr5	protein_coding
ENSG00000280389.1	5.05694572	-2.9744029	0.0100455	0.02582742	0	0	0.14	0.24	RP11-512M8	--	chr12	TEC
ENSG00000234545.6	1.59895183	3.56547312	1.27E-07	8.44E-07	1.88	1.68	6.08	8.37	FAM133B	family with sequence similarity 133, member B	chr7	protein_coding
ENSG00000106070.16	-4.9147823	6.04250746	3.84E-47	4.55E-45	16.14	15.91	0.73	0.68	GRB10	growth factor receptor-bound protein 10	chr7	protein_coding
ENSG00000105204.12	1.07953229	4.07717732	0.00024708	0.00092042	2.43	3.27	9.11	6.84	DYRK1B	dual-specificity tyrosine-(Y)-phosphorylation regulated kinase 1B	chr19	protein_coding
ENSG00000056487.14	4.93613345	1.7694393	1.10E-29	4.99E-28	0.01	0.07	1.87	1.63	PHF21B	PHD finger protein 21B	chr22	protein_coding
ENSG00000089195.13	1.93445748	3.25050009	4.23E-10	3.91E-09	1.17	1.52	5.74	8.01	TRMT6	tRNA methyltransferase 6	chr20	protein_coding
ENSG00000166407.12	-1.1567872	1.69026611	0.00042556	0.00150866	2.74	1.79	1.8	0.92	LMO1	LIM domain only 1 (rhombotin 1)	chr11	protein_coding
ENSG00000160813.5	-1.6046392	4.9753388	4.99E-08	3.48E-07	42.65	31.56	16.44	16.11	PPP1R35	protein phosphatase 1, regulatory subunit 35	chr7	protein_coding
ENSG00000181754.6	-2.1225977	3.01539094	1.20E-11	1.34E-10	2.09	2.04	0.6	0.67	AMIGO1	adhesion molecule with Ig-like domain 1	chr1	protein_coding
ENSG00000170946.13	2.54986742	0.13309773	8.35E-09	6.48E-08	0.09	0.14	0.59	1.25	DNAJC24	DnaJ (Hsp40) homolog, subfamily C, member 24	chr11	protein_coding
ENSG00000261423.1	2.05011566	0.85656135	5.57E-08	3.87E-07	0.17	0.29	1.03	1.53	RP11-1007O	--	chr15	lincRNA
ENSG00000183891.5	1.85710745	0.67152368	9.22E-07	5.33E-06	0.52	0.61	1.86	3.59	TTC32	tetratricopeptide repeat domain 32	chr2	protein_coding
ENSG00000126432.12	-2.1938787	9.22632387	1.37E-13	1.88E-12	986.05	794.62	273.26	245.02	PRDX5	peroxiredoxin 5	chr11	protein_coding
ENSG00000099326.7	-1.2547082	4.77846937	1.77E-05	8.21E-05	10.29	9.23	5.83	5.06	MZF1	myeloid zinc finger 1	chr19	protein_coding
ENSG00000257315.1	5.10319244	-0.3980236	3.35E-14	4.90E-13	0.01	0	0.32	0.33	ZBED6	zinc finger, BED-type containing 6	chr1	protein_coding
ENSG00000124787.12	-1.7701301	0.15335054	7.89E-06	3.91E-05	0.63	1.1	0.29	0.38	RPP40	ribonuclease P/MRP 40kDa subunit	chr6	protein_coding
ENSG00000158055.14	-4.5631253	-0.4331721	1.36E-14	2.08E-13	0.29	0.36	0.03	0.01	GRHL3	grainyhead-like 3 (Drosophila)	chr1	protein_coding
ENSG00000128917.6	2.75643004	-0.2723003	1.89E-08	1.39E-07	0.02	0.08	0.38	0.44	DLL4	delta-like 4 (Drosophila)	chr15	protein_coding
ENSG00000272540.1	-1.3946568	-0.5985263	0.00171161	0.00532936	1.56	0.29	0.37	0.56	XXbac-BPG2	--	chr6	antisense
ENSG00000134802.16	-1.1837163	5.48088376	4.51E-05	0.00019518	15.85	18.88	10.05	10.3	SLC43A3	solute carrier family 43, member 3	chr11	protein_coding
ENSG00000103253.16	-1.0068526	4.16436451	0.00059991	0.00207102	11.26	7.85	5.98	6.74	HAGHL	hydroxyacylglutathione hydrolase-like	chr16	protein_coding
ENSG00000100344.9	-2.3984422	2.91488663	5.23E-14	7.51E-13	3.22	4.1	0.8	1.05	PNPLA3	patatin-like phospholipase domain containing 3	chr22	protein_coding
ENSG00000143776.17	1.96956346	2.9006641	3.58E-10	3.34E-09	0.22	0.22	0.93	1.38	CDC42BPA	CDC42 binding protein kinase alpha (DMPK-like)	chr1	protein_coding
ENSG00000182670.12	2.16785795	3.08033907	6.63E-12	7.60E-11	0.15	0.23	0.81	1.45	TTC3	tetratricopeptide repeat domain 3	chr21	protein_coding
ENSG00000107833.9	-1.5414226	6.40455321	1.24E-07	8.22E-07	129.09	103.44	54.06	52.45	NPM3	nucleophosmin/nucleoplasmin 3	chr10	protein_coding
ENSG00000140367.10	2.41888736	2.05848717	1.01E-12	1.27E-11	0.29	0.29	1.56	2.58	UBE2Q2	ubiquitin-conjugating enzyme E2Q family member 2	chr15	protein_coding
ENSG00000137266.13	-3.7072159	4.71550368	4.45E-30	2.05E-28	4.82	5.62	0.55	0.51	SLC22A23	solute carrier family 22, member 23	chr6	protein_coding
ENSG00000101624.9	1.49370567	1.8444787	5.21E-06	2.66E-05	0.6	0.4	1.56	2.24	CEP76	centrosomal protein 76kDa	chr18	protein_coding
ENSG00000171858.16	-1.3321452	9.72330083	3.94E-06	2.06E-05	1067.2	845.81	480.86	533.99	RPS21	ribosomal protein S21	chr20	protein_coding
ENSG00000162636.14	2.35539137	0.60197709	3.46E-09	2.83E-08	0.03	0.1	0.26	0.62	FAM102B	family with sequence similarity 102, member B	chr1	protein_coding
ENSG00000277879.1	7.29530124	-1.4026834	7.05E-10	6.26E-09	0	0	0.27	0.54	RP11-129M1	--	chr10	antisense
ENSG00000122779.15	1.86782998	2.30329011	6.75E-09	5.31E-08	0.18	0.22	0.86	1.09	TRIM24	tripartite motif containing 24	chr7	protein_coding
ENSG00000139637.12	-1.2200653	6.06613911	2.51E-05	0.00011343	64.08	55.52	35.52	32.86	C12orf10	chromosome 12 open reading frame 10	chr12	protein_coding
ENSG00000120149.8	-8.7361334	2.72980738	1.23E-55	2.41E-53	3.79	5.69	0.03	0	MSX2	msh homeobox 2	chr5	protein_coding
ENSG00000253764.1	1.56473664	-1.4056201	0.00667945	0.0181213	0.24	0.2	1.38	0.44	RP11-439C1	--	chr8	lincRNA
ENSG00000128052.8	-4.7939316	-1.8009225	3.05E-08	2.18E-07	0.03	0.1	0	0	KDR	kinase insert domain receptor	chr4	protein_coding
ENSG00000165449.10	-1.0662448	-0.337492	0.01168849	0.02965671	0.06	0.33	0.14	0.1	SLC16A9	solute carrier family 16, member 9	chr10	protein_coding
ENSG00000154252.11	-6.2738548	-2.380434	2.70E-05	0.00012094	0.21	0.12	0	0	GAL3ST2	galactose-3-O-sulfotransferase 2	chr2	protein_coding
ENSG00000109689.13	2.17212033	2.79169034	1.10E-11	1.23E-10	0.3	0.46	1.87	2.73	STIM2	stromal interaction molecule 2	chr4	protein_coding
ENSG00000171680.19	-3.5471463	5.26840507	1.28E-28	5.47E-27	9.78	9.15	1.11	1.05	PLEKHG5	pleckstrin homology domain containing, family G (with RhoGef domain) member 5	chr1	protein_coding
ENSG00000198900.5	1.46216162	5.75314565	5.46E-07	3.28E-06	5.04	7.4	17.51	28.37	TOP1	topoisomerase (DNA) I	chr20	protein_coding
ENSG00000255468.5	1.49988592	-0.5174423	0.00138669	0.00444053	0.17	0.17	0.73	0.57	RP11-867G2	--	chr11	antisense
ENSG00000188997.6	-1.8751366	3.11462963	1.20E-09	1.04E-08	2.9	3.24	0.96	1.28	KCTD21	potassium channel tetramerization domain containing 21	chr11	protein_coding
ENSG00000259972.2	1.81322036	-1.532182	0.00494993	0.0139272	0.09	0.04	0.16	0.48	AC009120.6	--	chr16	processed_transcript
ENSG00000249637.1	1.21269034	0.52322933	0.00115193	0.00373568	1.27	1.14	3.03	4.41	CTC-329D1.2	--	chr5	antisense
ENSG00000142530.9	2.05334585	0.24001521	4.73E-07	2.87E-06	0.48	0.04	1.88	1.11	FAM71E1	family with sequence similarity 71, member E1	chr19	protein_coding
ENSG00000204406.10	1.17915017	-0.0448325	0.00381432	0.01098079	0.03	0.05	0.11	0.11	MBD5	methyl-CpG binding domain protein 5	chr2	protein_coding
ENSG00000103995.12	2.64728035	1.40424619	5.07E-13	6.56E-12	0.1	0.07	0.57	0.84	CEP152	centrosomal protein 152kDa	chr15	protein_coding
ENSG00000105497.6	1.58252251	0.86680822	1.54E-05	7.24E-05	0.11	0.08	0.3	0.46	ZNF175	zinc finger protein 175	chr19	protein_coding
ENSG00000221874.4	-6.4370381	-2.2645457	5.19E-06	2.66E-05	0.13	0.29	0	0	ZNF321P	zinc finger protein 321, pseudogene	chr19	protein_coding
ENSG00000244115.1	1.61281907	3.14624538	1.53E-07	1.00E-06	2.09	2.74	6.19	13.62	DNAJC25-GI	DNAJC25-GNG10 readthrough	chr9	protein_coding
ENSG00000074590.12	-2.7396953	3.28275553	1.25E-17	2.49E-16	1.94	2.08	0.46	0.34	NUAK1	NUAK family, SNF1-like kinase, 1	chr12	protein_coding
ENSG00000174332.4	-3.4156583	1.4325295	7.22E-19	1.60E-17	1.44	1.11	0.23	0.1	GLIS1	GLIS family zinc finger 1	chr1	protein_coding
ENSG00000124795.13	1.73788992	4.27464195	6.49E-09	5.12E-08	2.04	2.15	5.52	13.31	DEK	DEK proto-oncogene	chr6	protein_coding
ENSG00000176170.12	-1.1968992	5.03313441	3.94E-05	0.00017212	13.58	10.99	6.89	7.42	SPHK1	sphingosine kinase 1	chr17	protein_coding
ENSG00000171862.8	1.75217641	2.63266703	2.70E-08	1.95E-07	0.23	0.26	0.92	1.32	PTEN	phosphatase and tensin homolog	chr10	protein_coding
ENSG00000134982.15	2.65921802	1.23970295	1.48E-12	1.83E-11	0.03	0.06	0.32	0.46	APC	adenomatous polyposis coli	chr5	protein_coding
ENSG00000176087.13	-1.1135059	6.39200807	0.00011377	0.00045205	22.59	23.64	15.07	13.34	SLC35A4	solute carrier family 35, member A4	chr5	protein_coding
ENSG00000144810.14	-5.2415769	2.20770291	6.74E-36	4.16E-34	1.09	1.48	0.07	0.02	COL8A1	collagen, type VIII, alpha 1	chr3	protein_coding
ENSG00000184489.10	1.02637238	1.94145835	0.00135433	0.00434678	0.86	0.53	1.87	1.91	PTP4A3	protein tyrosine phosphatase type IVA, member 3	chr8	protein_coding
ENSG00000213904.7	2.2182482	3.13587755	2.02E-12	2.46E-11	0.67	0.51	4.1	3.22	LIPE-AS1	LIPE antisense RNA 1	chr19	antisense
ENSG00000229980.4	1.04498144	-0.0904379	0.01326645	0.03319311	0.11	0.36	0.81	0.47	TOB1-AS1	TOB1 antisense RNA 1	chr17	processed_transcript
ENSG00000174327.6	1.30102994	1.97810526	5.59E-05	0.00023628	1.18	0.88	3.54	3.22	SLC16A13	solute carrier family 16, member 13	chr17	protein_coding
ENSG00000174891.11	1.13698945	1.56245249	0.00057668	0.00199684	0.39	0.65	1.07	1.99	RSRC1	arginine/serine-rich coiled-coil 1	chr3	protein_coding
ENSG00000111859.15	-2.2465861	0.79776047	1.76E-09	1.50E-08	0.3	0.3	0.07	0.1	NEDD9	neural precursor cell expressed, developmentally down-regulated 9	chr6	protein_coding
ENSG00000198837.8	-1.18937	5.70420093	4.06E-05	0.00017696	10.28	10.28	5.79	6.23	DENND4B	DENN/MADD domain containing 4B	chr1	protein_coding
ENSG00000121879.3	2.87579109	0.61323786	4.22E-12	4.94E-11	0.05	0.01	0.19	0.41	PIK3CA	phosphatidylinositol-4,5-bisphosphate 3-kinase, catalytic subunit alpha	chr3	protein_coding
ENSG00000176595.3	-6.1013967	-2.5004644	8.36E-05	0.00034052	0.01	0.05	0	0	KBTBD11	kelch repeat and BTB (POZ) domain containing 11	chr8	protein_coding
ENSG00000078098.12	1.08177987	0.21741826	0.00576197	0.01584923	0.28	0.12	0.44	0.7	FAP	fibroblast activation protein, alpha	chr2	protein_coding
ENSG00000264624.1	-2.221066	-2.5115698	0.01191236	0.03011315	2.15	1.63	0.37	0.63	MIR3615	microRNA 3615	chr17	miRNA
ENSG00000154640.13	1.02113446	3.13672356	0.00072435	0.00245636	2.36	3.52	5.97	9.96	BTG3	BTG family, member 3	chr21	protein_coding
ENSG00000271857.1	-4.4322292	0.6501617	7.31E-22	2.03E-20	2.71	2.51	0.07	0.24	RP1-244F24.	--	chr6	antisense
ENSG00000134324.10	2.21928197	3.18970843	1.70E-12	2.08E-11	0.38	0.47	2.13	3.14	LPIN1	lipin 1	chr2	protein_coding
ENSG00000162522.9	-1.1930956	6.31609827	3.68E-05	0.00016136	15.59	15.39	9.77	8.24	KIAA1522	KIAA1522	chr1	protein_coding
ENSG00000234203.1	-5.3787292	-2.9446861	0.00290522	0.00856312	0	0.58	0	0	RP5-1050D4	--	chr17	antisense
ENSG00000144362.10	1.2081624	-0.4117784	0.00705264	0.01902182	0.33	0.14	0.63	0.85	PHOSPHO2	phosphatase, orphan 2	chr2	protein_coding
ENSG00000176155.17	-1.2242217	4.56663381	2.91E-05	0.00012966	4.34	4.23	2.64	2.24	CCDC57	coiled-coil domain containing 57	chr17	protein_coding
ENSG00000262601.1	-2.5406454	-2.3292898	0.00281991	0.00840887	0.17	0.36	0.04	0.07	CTC-786C10	--	chr16	lincRNA
ENSG00000120327.6	1.64198256	1.19096048	3.37E-06	1.78E-05	0.08	0.28	0.58	0.91	PCDHB14	protocadherin beta 14	chr5	protein_coding
ENSG00000260852.1	-1.6946211	1.34831167	6.89E-07	4.07E-06	0.92	0.65	0.38	0.27	FBXL19-AS1	FBXL19 antisense RNA 1 (head to head)	chr16	antisense
ENSG00000179913.9	-6.2778101	-2.3784356	2.70E-05	0.00012094	0.07	0.14	0	0	B3GNT3	UDP-GlcNAc:betaGal beta-1,3-N-acetylglucosaminyltransferase 3	chr19	protein_coding
ENSG00000111877.16	2.52790518	1.55992078	1.50E-12	1.86E-11	0.07	0.15	0.54	1.17	MCM9	minichromosome maintenance complex component 9	chr6	protein_coding
ENSG00000227252.1	1.95816331	-1.0046175	0.00039402	0.00140474	0.02	0.1	0.36	0.27	AC105760.2	--	chr2	antisense
ENSG00000058453.15	-1.3845655	5.29476238	2.09E-06	1.15E-05	7.09	6.71	3.36	3.69	CROCC	ciliary rootlet coiled-coil, rootletin	chr1	protein_coding
ENSG00000125686.10	1.34625069	3.95630038	6.00E-06	3.04E-05	0.99	1.07	3.14	3.83	MED1	mediator complex subunit 1	chr17	protein_coding
ENSG00000278828.1	2.83874664	1.7251702	3.36E-15	5.45E-14	0.56	0.46	4.82	4.98	HIST1H3H	histone cluster 1, H3h	chr6	protein_coding
ENSG00000182195.7	-7.130947	5.03895583	8.73E-70	3.49E-67	38.83	37.74	0.6	0.14	LDOC1	leucine zipper, down-regulated in cancer 1	chrX	protein_coding
ENSG00000112379.8	3.02385301	1.88506741	4.60E-17	8.75E-16	0.05	0.04	0.42	0.51	KIAA1244	--	chr6	protein_coding
ENSG00000126561.15	1.46285015	5.25785681	5.85E-07	3.50E-06	3.38	3.53	12.51	12.87	STAT5A	signal transducer and activator of transcription 5A	chr17	protein_coding
ENSG00000238795.1	-1.165471	2.29907945	0.00019577	0.00074047	23.18	17.32	10.8	13.31	SCARNA12	small Cajal body-specific RNA 12	chr12	snoRNA
ENSG00000197619.12	2.41851865	-1.753724	0.00140662	0.00449532	0.02	0	0.08	0.1	ZNF615	zinc finger protein 615	chr19	protein_coding
ENSG00000168350.7	5.20247544	-2.8942551	0.00538165	0.01488851	0	0	0.02	0.04	DEGS2	delta(4)-desaturase, sphingolipid 2	chr14	protein_coding
ENSG00000101367.8	1.00626245	6.96536695	0.00046528	0.00163737	22.51	28.79	56.07	81.76	MAPRE1	microtubule-associated protein, RP/EB family, member 1	chr20	protein_coding
ENSG00000198146.4	3.04857941	1.91449737	2.38E-17	4.65E-16	0.09	0.14	0.67	1.94	ZNF770	zinc finger protein 770	chr15	protein_coding
ENSG00000278344.1	-3.1397382	-1.6268026	8.20E-06	4.05E-05	1.88	0.4	0.09	0.23	RP11-18C24.	--	chr12	lincRNA
ENSG00000262482.1	4.88946445	-3.0622981	0.01889873	0.04519557	0	0	0.13	0.32	LA16c-321D4	--	chr16	antisense
ENSG00000273210.1	5.66409976	-2.616209	0.00086564	0.00288128	0	0	0.14	0.72	AP001437.1	--	chr21	antisense
ENSG00000260830.1	2.35971642	-0.9869873	2.36E-05	0.00010713	0.26	0.09	0.92	1.52	RP11-524O1	--	chr14	antisense
ENSG00000207200.1	5.07225195	-2.9660962	0.0100455	0.02582742	0	0	1.8	0.76	RNU6-45P	RNA, U6 small nuclear 45, pseudogene	chr11	snRNA
ENSG00000213585.9	-1.1129789	8.16717198	0.00010837	0.0004329	141.87	154.46	86.95	95.74	VDAC1	voltage-dependent anion channel 1	chr5	protein_coding
ENSG00000224645.1	-5.2118379	-3.0342168	0.00538165	0.01488851	0.07	0.55	0	0	RP11-126K1.	--	chr1	antisense
ENSG00000196177.11	3.34341361	0.9446265	5.39E-16	9.37E-15	0.05	0.03	0.51	0.7	ACADSB	acyl-CoA dehydrogenase, short/branched chain	chr10	protein_coding
ENSG00000260588.1	-1.3457604	-0.1484511	0.0011376	0.00369419	0.66	0.46	0.22	0.37	RP11-930P14	--	chr8	lincRNA
ENSG00000102978.11	1.02494579	6.87344905	0.00036662	0.00131704	30.02	30.76	76.76	88.33	POLR2C	polymerase (RNA) II (DNA directed) polypeptide C, 33kDa	chr16	protein_coding
ENSG00000162817.6	-12.128802	3.07766066	2.18E-62	6.08E-60	4.13	3.89	0	0	C1orf115	chromosome 1 open reading frame 115	chr1	protein_coding
ENSG00000166897.13	-1.074968	5.75801852	0.00020024	0.00075652	6.65	7.57	4.62	4.36	ELFN2	extracellular leucine-rich repeat and fibronectin type III domain containing 2	chr22	protein_coding
ENSG00000177519.3	-2.5018734	0.66307391	1.40E-10	1.38E-09	1.25	1.68	0.39	0.3	RPRM	reprimo, TP53 dependent G2 arrest mediator candidate	chr2	protein_coding
ENSG00000224389.7	-6.0013451	-2.5669109	0.00014842	0.00057423	0.02	0.05	0	0	C4B	complement component 4B (Chido blood group)	chr6	protein_coding
ENSG00000172650.11	1.08270773	0.11927204	0.00632161	0.01723826	0.17	0.2	0.59	0.48	AGAP5	ArfGAP with GTPase domain, ankyrin repeat and PH domain 5	chr10	protein_coding
ENSG00000140297.11	-7.0772991	2.95278858	3.57E-52	5.63E-50	2.78	2.13	0.04	0.01	GCNT3	glucosaminyl (N-acetyl) transferase 3, mucin type	chr15	protein_coding
ENSG00000158427.13	5.34914687	-2.8103904	0.00290522	0.00856312	0	0	0.05	0.04	TMSB15B	thymosin beta 15B	chrX	protein_coding
ENSG00000258334.1	3.72874421	-1.5391598	5.39E-06	2.75E-05	0.01	0	0.08	0.17	RP11-161H2	--	chr12	antisense
ENSG00000206559.6	-5.2054745	-3.0371681	0.0100455	0.02582742	0.12	0	0	0	ZCWPW2	zinc finger, CW type with PWWP domain 2	chr3	protein_coding
ENSG00000227124.7	-1.1591967	0.00279183	0.00362804	0.0104924	0.2	0.26	0.1	0.17	ZNF717	zinc finger protein 717	chr3	protein_coding
ENSG00000125740.12	1.33615712	2.37768189	2.13E-05	9.70E-05	0.35	0.5	1.75	1.08	FOSB	FBJ murine osteosarcoma viral oncogene homolog B	chr19	protein_coding
ENSG00000076826.8	3.39352413	3.3559609	3.67E-24	1.20E-22	0.28	0.41	5.07	4.45	CAMSAP3	calmodulin regulated spectrin-associated protein family, member 3	chr19	protein_coding
ENSG00000149925.15	-1.4662057	11.4280942	4.13E-07	2.53E-06	865	689.24	386.1	363.56	ALDOA	aldolase A, fructose-bisphosphate	chr16	protein_coding
ENSG00000162889.9	-1.4062653	7.65079278	1.21E-06	6.84E-06	67.27	64.28	32.39	33.78	MAPKAPK2	mitogen-activated protein kinase-activated protein kinase 2	chr1	protein_coding
ENSG00000145703.14	-3.4946199	-0.3403604	5.59E-12	6.47E-11	0.21	0.17	0.01	0.03	IQGAP2	IQ motif containing GTPase activating protein 2	chr5	protein_coding
ENSG00000110218.7	1.05773343	3.42841889	0.00040479	0.00143971	1.84	2.22	5.03	6.24	PANX1	pannexin 1	chr11	protein_coding
ENSG00000154059.8	1.79246479	2.82287535	1.03E-08	7.91E-08	0.51	0.83	2.71	3.46	IMPACT	impact RWD domain protein	chr18	protein_coding
ENSG00000198393.6	3.15771236	1.05204122	3.29E-15	5.35E-14	0.01	0.03	0.14	0.24	ZNF26	zinc finger protein 26	chr12	protein_coding
ENSG00000167619.10	2.90695375	1.23416731	3.05E-14	4.49E-13	0.22	0.11	1.65	1.66	TMEM145	transmembrane protein 145	chr19	protein_coding
ENSG00000171346.12	3.8522805	2.2472959	5.85E-25	2.02E-23	0.08	0.1	1.54	2.04	KRT15	keratin 15, type I	chr17	protein_coding
ENSG00000271984.1	5.9251699	-2.4430195	0.00014842	0.00057423	0	0	0	0.81	RP3-337O18	--	chr20	antisense
ENSG00000186642.14	-10.794951	9.26230841	4.79E-130	5.74E-126	210.15	213.25	0.18	0.13	PDE2A	phosphodiesterase 2A, cGMP-stimulated	chr11	protein_coding
ENSG00000082898.15	2.25999764	4.45882104	1.22E-13	1.68E-12	0.95	1.36	5.06	9.75	XPO1	exportin 1	chr2	protein_coding
ENSG00000123685.7	-3.0580618	3.55068348	2.68E-21	7.18E-20	17.82	16.74	2.94	2.58	BATF3	basic leucine zipper transcription factor, ATF-like 3	chr1	protein_coding
ENSG00000265972.4	-1.8460087	6.73879929	3.35E-10	3.13E-09	42.68	41.53	14.78	16.48	TXNIP	thioredoxin interacting protein	chr1	protein_coding
ENSG00000272502.1	1.1991272	-0.933274	0.0202624	0.0480683	0.16	0.22	0.39	0.79	RP11-713M1	--	chr8	antisense
ENSG00000163029.14	4.54510271	2.17930535	5.49E-30	2.52E-28	0.04	0.07	0.94	2.3	SMC6	structural maintenance of chromosomes 6	chr2	protein_coding
ENSG00000188603.15	-2.4885136	-0.0374487	1.29E-08	9.73E-08	0.66	0.39	0.09	0.15	CLN3	ceroid-lipofuscinosis, neuronal 3	chr16	protein_coding
ENSG00000047621.10	2.04686581	0.7401937	7.02E-08	4.81E-07	0.13	0.12	0.63	0.79	C12orf4	chromosome 12 open reading frame 4	chr12	protein_coding
ENSG00000138604.8	1.99272265	1.33079018	1.89E-08	1.39E-07	0.17	0.13	0.61	1.05	GLCE	glucuronic acid epimerase	chr15	protein_coding
ENSG00000126804.12	3.68808116	2.54060314	7.54E-25	2.59E-23	0.13	0.14	1.77	2.95	ZBTB1	zinc finger and BTB domain containing 1	chr14	protein_coding
ENSG00000100503.22	2.01819739	2.42734637	4.10E-10	3.78E-09	0.18	0.12	0.64	1.04	NIN	ninein (GSK3B interacting protein)	chr14	protein_coding
ENSG00000169031.17	-2.3368336	1.74855974	6.42E-12	7.37E-11	0.56	0.56	0.12	0.17	COL4A3	collagen, type IV, alpha 3 (Goodpasture antigen)	chr2	protein_coding
ENSG00000104808.6	3.09273003	-0.4152381	5.25E-09	4.18E-08	0.05	0.16	1.66	0.73	DHDH	dihydrodiol dehydrogenase (dimeric)	chr19	protein_coding
ENSG00000062650.16	1.48276013	3.44505854	9.59E-07	5.53E-06	0.53	0.93	2.11	3.33	WAPAL	wings apart-like homolog (Drosophila)	chr10	protein_coding
ENSG00000260456.5	1.324498	1.86941738	4.39E-05	0.00019029	1.76	1.08	4.84	4.66	C16orf95	chromosome 16 open reading frame 95	chr16	protein_coding
ENSG00000230185.4	3.12154028	-1.5546714	4.58E-05	0.00019809	0.02	0.03	0.04	0.54	C9orf147	chromosome 9 open reading frame 147	chr9	antisense
ENSG00000210077.1	4.02894623	3.62995308	1.39E-31	6.90E-30	15.89	17.25	398.32	322.04	MT-TV	mitochondrially encoded tRNA valine	chrM	Mt_tRNA
ENSG00000264772.4	-1.4630514	5.62829353	5.39E-07	3.24E-06	12.15	13.67	6.65	5.8	RP11-186B7.	--	chr17	processed_transcript
ENSG00000182791.4	1.8644806	-0.8440585	0.0003468	0.00125054	0.06	0.06	0.21	0.35	CCDC87	coiled-coil domain containing 87	chr11	protein_coding
ENSG00000111716.11	-3.075592	7.85951781	1.48E-23	4.62E-22	207.22	216.85	28.22	39.06	LDHB	lactate dehydrogenase B	chr12	protein_coding
ENSG00000058404.18	10.6370196	1.64454285	4.31E-40	3.29E-38	0	0	1.66	1.14	CAMK2B	calcium/calmodulin-dependent protein kinase II beta	chr7	protein_coding
ENSG00000103540.15	3.13349294	1.2970743	7.04E-16	1.21E-14	0.06	0.08	0.6	1.01	CCP110	centriolar coiled coil protein 110kDa	chr16	protein_coding
ENSG00000174485.13	2.73860211	0.56808281	5.60E-11	5.78E-10	0.03	0.04	0.25	0.35	DENND4A	DENN/MADD domain containing 4A	chr15	protein_coding
ENSG00000010295.18	-2.1821716	-0.4423715	1.13E-06	6.41E-06	0.22	0.25	0	0.13	IFFO1	intermediate filament family orphan 1	chr12	protein_coding
ENSG00000273599.1	2.69855117	-0.7211151	1.12E-06	6.36E-06	0.01	0.03	0.11	0.25	RP11-59C5.3	--	chr10	antisense
ENSG00000251357.4	-1.0670492	8.9218059	0.00020171	0.00076149	762.86	584.52	440.12	417.15	AP000350.10	--	chr22	protein_coding
ENSG00000266983.1	2.15119273	-1.6875947	0.0022827	0.00692407	0.13	0.09	0.45	0.85	CTC-232P5.1	--	chr19	antisense
ENSG00000125266.6	-4.8374047	4.37644405	3.24E-43	3.00E-41	5.85	8.77	0.33	0.35	EFNB2	ephrin-B2	chr13	protein_coding
ENSG00000164414.15	1.94096399	1.08924179	8.37E-08	5.66E-07	0.24	0.48	1.04	2.62	SLC35A1	solute carrier family 35 (CMP-sialic acid transporter), member A1	chr6	protein_coding
ENSG00000198743.5	1.43284793	1.90229801	1.04E-05	5.01E-05	0.15	0.12	0.46	0.53	SLC5A3	solute carrier family 5 (sodium/myo-inositol cotransporter), member 3	chr21	protein_coding
ENSG00000135205.13	1.9957622	-2.0281806	0.01397446	0.03477946	0	0.03	0.06	0.12	CCDC146	coiled-coil domain containing 146	chr7	protein_coding
ENSG00000167617.2	1.82078905	-0.7786347	0.00028644	0.00104674	0.19	0.23	1.3	0.69	CDC42EP5	CDC42 effector protein (Rho GTPase binding) 5	chr19	protein_coding
ENSG00000240184.5	2.15804396	2.67081168	1.87E-11	2.04E-10	0.4	0.33	2.64	1.74	PCDHGC3	protocadherin gamma subfamily C, 3	chr5	protein_coding
ENSG00000187624.8	-1.4541418	2.8552368	2.18E-06	1.19E-05	3.48	4.08	1.74	1.94	C17orf97	chromosome 17 open reading frame 97	chr17	protein_coding
ENSG00000166435.14	1.86261692	3.13823171	1.84E-09	1.55E-08	0.58	0.48	2.42	2.75	XRRA1	X-ray radiation resistance associated 1	chr11	protein_coding
ENSG00000167912.5	-5.6591842	-2.7826667	0.00086564	0.00288128	0.06	0.11	0	0	RP11-25K19.	--	chr8	antisense
ENSG00000104081.12	-2.4572866	4.41897684	1.19E-15	2.01E-14	6.39	5.53	1.5	1.39	BMF	Bcl2 modifying factor	chr15	protein_coding
ENSG00000164949.6	-2.4948051	-2.0784202	0.00094481	0.00312013	0.18	0.05	0	0.05	GEM	GTP binding protein overexpressed in skeletal muscle	chr8	protein_coding
ENSG00000107201.8	3.48718386	0.65932401	2.92E-15	4.77E-14	0.05	0.04	0.53	0.81	DDX58	DEAD (Asp-Glu-Ala-Asp) box polypeptide 58	chr9	protein_coding
ENSG00000120051.13	-4.8079387	-3.2306674	0.01889873	0.04519557	0.02	0.03	0	0	CFAP58	cilia and flagella associated protein 58	chr10	protein_coding
ENSG00000272674.3	-3.2485879	-2.2716019	0.00013996	0.00054609	0.02	0.07	0	0.01	PCDHB16	protocadherin beta 16	chr5	protein_coding

ENSG00000159239.10	1.71715323	0.33193065	1.77E-05	8.19E-05	0.09	0.24	0.93	0.48	C2orf81	chromosome 2 open reading frame 81	chr2	protein_coding
ENSG00000147050.13	4.06348946	1.97234049	1.45E-25	5.23E-24	0.05	0.07	1.13	1.49	KDM6A	lysine (K)-specific demethylase 6A	chrX	protein_coding
ENSG00000134215.14	2.76600381	-0.3924231	4.67E-08	3.27E-07	0.05	0	0.17	0.32	VAV3	vav 3 guanine nucleotide exchange factor	chr1	protein_coding
ENSG00000085491.14	1.46873544	2.13163038	4.56E-06	2.37E-05	0.32	0.24	0.76	1.33	SLC25A24	solute carrier family 25 (mitochondrial carrier; phosphate carrier), member 24	chr1	protein_coding
ENSG00000174788.8	4.51782461	0.8311066	3.76E-21	9.93E-20	0.13	0.14	4.01	4.64	PCP2	Purkinje cell protein 2	chr19	protein_coding
ENSG00000124588.18	-1.3872527	6.59525852	1.75E-06	9.70E-06	10.61	9.89	5.65	4.77	NQO2	NAD(P)H dehydrogenase, quinone 2	chr6	protein_coding
ENSG00000225177.4	-3.2744453	0.7463293	1.13E-15	1.92E-14	2.08	1.93	0.16	0.38	RP11-390P2.	--	chr6	antisense
ENSG00000163623.8	3.47163846	0.98947198	1.26E-16	2.31E-15	0	0.17	1.36	1.05	NKX6-1	NK6 homeobox 1	chr4	protein_coding
ENSG00000171435.12	3.48839891	-0.6785953	2.25E-09	1.88E-08	0.01	0	0.07	0.05	KSR2	kinase suppressor of ras 2	chr12	protein_coding
ENSG00000198576.3	1.57856923	1.15894538	6.02E-06	3.04E-05	0.24	0.36	1.21	1.15	ARC	activity-regulated cytoskeleton-associated protein	chr8	protein_coding
ENSG00000163626.15	1.0036105	1.4561942	0.00269964	0.00809554	0.2	0.22	0.4	0.72	COX18	COX18 cytochrome c oxidase assembly factor	chr4	protein_coding
ENSG00000121858.9	6.05135458	0.46149026	8.89E-23	2.61E-21	0.03	0	1.23	1.53	TNFSF10	tumor necrosis factor (ligand) superfamily, member 10	chr3	protein_coding
ENSG00000006432.14	1.08202725	4.20137439	0.0002319	0.00086713	0.85	0.74	2.5	1.97	MAP3K9	mitogen-activated protein kinase kinase kinase 9	chr14	protein_coding
ENSG00000110002.14	-5.8153074	2.02855485	1.55E-37	1.03E-35	1.17	0.95	0.05	0	VWA5A	von Willebrand factor A domain containing 5A	chr11	protein_coding
ENSG00000168234.11	1.58976331	4.48134056	8.54E-08	5.77E-07	1.48	1.34	5.44	5.9	TTC39C	tetratricopeptide repeat domain 39C	chr18	protein_coding
ENSG00000196230.11	-1.0870415	10.2103462	0.00015257	0.00058875	403.65	372.68	254.79	231.63	TUBB	tubulin, beta class I	chr6	protein_coding
ENSG00000216895.7	2.0398274	1.36728045	5.72E-09	4.55E-08	0.54	0.39	1.85	3.28	AC009403.2	--	chr7	antisense
ENSG00000141744.3	-3.0002638	0.0657834	6.73E-11	6.89E-10	1.23	0.78	0.33	0.02	PNMT	phenylethanolamine N-methyltransferase	chr17	protein_coding
ENSG00000122861.14	-3.6223439	-0.2053836	1.50E-12	1.86E-11	0.52	0.42	0.07	0.03	PLAU	plasminogen activator, urokinase	chr10	protein_coding
ENSG00000117262.17	1.56863559	2.19241218	1.01E-06	5.82E-06	0.97	0.66	2.96	3.53	GPR89A	G protein-coupled receptor 89A	chr1	protein_coding
ENSG00000267757.3	-1.1820318	2.62763262	0.00012528	0.00049412	8.99	5.96	4.34	4.46	C19orf83	--	chr19	protein_coding
ENSG00000224167.1	4.12594222	0.48024458	2.55E-17	4.95E-16	0	0.39	4.53	4.45	RP3-522D1.1	--	chr1	lincRNA
ENSG00000183291.14	1.15220688	3.34167956	0.00012839	0.00050537	2.39	2.98	5.01	11.02	15-Sep	--	chr1	protein_coding
ENSG00000143164.14	2.00982058	3.72737661	5.36E-11	5.54E-10	1.11	1.23	4.65	7.98	DCAF6	DDB1 and CUL4 associated factor 6	chr1	protein_coding
ENSG00000241472.5	5.0212713	-1.2215183	7.16E-09	5.62E-08	0	0.01	0.13	0.23	PTPRG-AS1	PTPRG antisense RNA 1	chr3	processed_transcript
ENSG00000174444.13	-1.3369862	11.1957552	3.62E-06	1.91E-05	826.21	839.2	424.78	454.22	RPL4	ribosomal protein L4	chr15	protein_coding
ENSG00000169047.5	-1.8323554	3.52831117	1.78E-09	1.51E-08	1.36	1.65	0.52	0.61	IRS1	insulin receptor substrate 1	chr2	protein_coding
ENSG00000267424.1	-5.8234587	0.73619258	8.99E-28	3.70E-26	4.34	2.8	0	0.15	CTD-2265O2	--	chr19	antisense
ENSG00000117335.17	2.36537682	4.95912252	7.04E-15	1.12E-13	2.55	2.29	11.4	22.09	CD46	CD46 molecule, complement regulatory protein	chr1	protein_coding
ENSG00000196748.8	5.22256039	-2.8832898	0.00538165	0.01488851	0	0	0.46	0.17	CLPSL2	colipase-like 2	chr6	protein_coding
ENSG00000277462.1	3.3224238	-1.0761253	3.64E-07	2.26E-06	0.03	0.03	0.31	0.46	ZNF670	zinc finger protein 670	chr1	protein_coding
ENSG00000139289.12	-3.457689	4.9796134	2.34E-27	9.53E-26	5.74	5.87	0.59	0.82	PHLDA1	pleckstrin homology-like domain, family A, member 1	chr12	protein_coding
ENSG00000124215.15	3.10630896	-2.6095771	0.00552196	0.01522223	0	0.01	0.02	0.06	CDH26	cadherin 26	chr20	protein_coding
ENSG00000125676.18	3.03035226	4.68055413	5.89E-22	1.64E-20	0.57	0.62	4.79	8.16	THOC2	THO complex 2	chrX	protein_coding
ENSG00000180008.8	3.2663568	1.01256482	6.62E-16	1.14E-14	0.07	0.01	0.33	0.73	SOCS4	suppressor of cytokine signaling 4	chr14	protein_coding
ENSG00000186300.10	1.94398469	-0.1251437	1.04E-05	5.03E-05	0.03	0.04	0.11	0.22	ZNF555	zinc finger protein 555	chr19	protein_coding
ENSG00000156273.14	1.52304147	0.56105587	6.19E-05	0.00026021	0.09	0.11	0.26	0.5	BACH1	BTB and CNC homology 1, basic leucine zipper transcription factor 1	chr21	protein_coding
ENSG00000269335.4	-1.578771	6.52930931	6.22E-08	4.30E-07	39.35	42.13	17.51	18.85	IKBKG	inhibitor of kappa light polypeptide gene enhancer in B-cells, kinase gamma	chrX	protein_coding
ENSG00000080839.10	1.48195664	1.67431567	8.17E-06	4.04E-05	0.22	0.23	0.76	0.94	RBL1	retinoblastoma-like 1	chr20	protein_coding
ENSG00000116983.11	8.44632016	-0.4089872	2.26E-16	4.05E-15	0	0	0.3	0.27	HPCAL4	hippocalcin like 4	chr1	protein_coding
ENSG00000074219.12	-1.6091195	5.8305779	3.88E-08	2.73E-07	27.96	23.65	12.01	10.51	TEAD2	TEA domain family member 2	chr19	protein_coding
ENSG00000198915.10	6.71895204	-1.878359	2.24E-07	1.42E-06	0	0	0.14	0.01	RASGEF1A	RasGEF domain family, member 1A	chr10	protein_coding
ENSG00000121316.9	3.57100349	3.38990021	4.34E-26	1.61E-24	0.37	0.7	7.12	9.93	PLBD1	phospholipase B domain containing 1	chr12	protein_coding
ENSG00000164543.5	1.98391613	2.5233441	5.68E-10	5.12E-09	0.6	0.5	2.22	3.58	STK17A	serine/threonine kinase 17a	chr7	protein_coding
ENSG00000197566.8	3.0345025	-2.0457762	0.00041794	0.0014836	0.01	0	0.02	0.13	ZNF624	zinc finger protein 624	chr17	protein_coding
ENSG00000026103.18	2.27433428	0.76256712	4.38E-09	3.54E-08	0.09	0.24	1.1	1.02	FAS	Fas cell surface death receptor	chr10	protein_coding
ENSG00000115828.14	10.0792536	1.1143683	3.89E-33	2.10E-31	0	0	2.03	3.05	QPCT	glutaminyl-peptide cyclotransferase	chr2	protein_coding
ENSG00000068885.13	3.94665451	-0.2810428	1.35E-12	1.67E-11	0	0.03	0.24	0.39	IFT80	intraflagellar transport 80	chr3	protein_coding
ENSG00000099994.10	-1.8255613	4.3654009	1.06E-09	9.24E-09	7.55	7.73	3.13	2.6	SUSD2	sushi domain containing 2	chr22	protein_coding
ENSG00000152558.13	3.44036691	3.26792578	2.03E-24	6.72E-23	0.33	0.36	2.55	7.55	TMEM123	transmembrane protein 123	chr11	protein_coding
ENSG00000278872.1	8.4895436	-0.3752599	8.93E-17	1.66E-15	0	0	1.19	0.6	RP13-895J2.4	--	chr12	TEC
ENSG00000173960.11	1.87410755	2.46212356	4.77E-09	3.82E-08	0.26	0.32	1.11	1.72	UBXN2A	UBX domain protein 2A	chr2	protein_coding
ENSG00000062038.12	3.94429656	-1.3682962	6.88E-07	4.06E-06	0	0.01	0.09	0.16	CDH3	cadherin 3, type 1, P-cadherin (placental)	chr16	protein_coding
ENSG00000197056.8	1.89924848	0.03255045	1.09E-05	5.24E-05	0.02	0.12	0.22	0.47	ZMYM1	zinc finger, MYM-type 1	chr1	protein_coding
ENSG00000108854.14	2.23043786	1.56733346	1.71E-10	1.66E-09	0.28	0.27	1.64	1.86	SMURF2	SMAD specific E3 ubiquitin protein ligase 2	chr17	protein_coding
ENSG00000260916.4	2.31708856	3.56417517	1.13E-13	1.56E-12	0.44	0.46	1.78	4.25	CCPG1	cell cycle progression 1	chr15	protein_coding
ENSG00000264322.2	4.89462967	-3.0595066	0.01889873	0.04519557	0	0	0.33	0.46	RN7SL448P	RNA, 7SL, cytoplasmic 448, pseudogene	chr17	misc_RNA
ENSG00000272686.1	1.04622611	0.2917438	0.00597183	0.016372	0.34	0.23	0.64	0.95	RP11-390E2	--	chr7	antisense
ENSG00000158220.12	2.69748125	2.00477353	8.83E-15	1.39E-13	0.14	0.22	1.36	1.77	ESYT3	extended synaptotagmin-like protein 3	chr3	protein_coding
ENSG00000269729.1	-4.0801013	-2.3235737	0.00013996	0.00054609	0.17	0.6	0	0.04	AC006262.4	--	chr19	lincRNA
ENSG00000198157.9	-4.3865119	0.11402786	7.45E-18	1.51E-16	0.65	0.91	0	0.09	HMGN5	high mobility group nucleosome binding domain 5	chrX	protein_coding
ENSG00000263843.1	-1.2806919	2.21626572	4.86E-05	0.00020777	1.78	2.42	1.1	1.2	RP11-649A1	--	chr17	antisense
ENSG00000100749.6	1.46643485	2.3200018	3.82E-06	2.01E-05	1.18	1.23	3.89	5.02	VRK1	vaccinia related kinase 1	chr14	protein_coding
ENSG00000187140.5	3.32468643	1.88510474	2.00E-19	4.60E-18	0.27	0.23	2.55	4.2	FOXD3	forkhead box D3	chr1	protein_coding
ENSG00000276115.1	6.28373948	-2.199428	8.95E-06	4.38E-05	0	0	0.27	0.09	RP11-817I4.2	--	chr12	sense_intronic
ENSG00000090554.11	-1.1440504	1.81902614	0.00043253	0.00153134	1.88	2.81	1.89	0.93	FLT3LG	fms-related tyrosine kinase 3 ligand	chr19	protein_coding
ENSG00000196950.12	2.5013455	0.81460218	1.98E-10	1.90E-09	0.05	0.1	0.34	0.75	SLC39A10	solute carrier family 39 (zinc transporter), member 10	chr2	protein_coding
ENSG00000167962.11	-1.2054065	8.22657846	2.86E-05	0.0001277	94.45	106.37	54.87	61.26	ZNF598	zinc finger protein 598	chr16	protein_coding
ENSG00000165672.6	1.37037585	4.26979696	3.70E-06	1.95E-05	5.06	5.84	14.62	23.12	PRDX3	peroxiredoxin 3	chr10	protein_coding
ENSG00000135624.14	-1.207669	9.69048872	2.72E-05	0.00012188	492.28	421.5	262.33	265.24	CCT7	chaperonin containing TCP1, subunit 7 (eta)	chr2	protein_coding
ENSG00000215765.3	-13.660498	4.61116268	2.01E-85	1.78E-82	31.4	29.81	0	0	CTB-61M7.1	--	chr19	processed_transcript
ENSG00000179304.15	-2.3139885	-1.1218842	4.42E-05	0.00019159	0.13	0.17	0.06	0.02	FAM156B	family with sequence similarity 156, member B	chrX	protein_coding
ENSG00000083535.14	2.17451905	-1.1130324	0.00015583	0.00060009	0	0.06	0.03	0.32	PIBF1	progesterone immunomodulatory binding factor 1	chr13	protein_coding
ENSG00000213903.7	1.62747679	4.13560423	5.36E-08	3.72E-07	1.25	1.89	6.62	6.28	LTB4R	leukotriene B4 receptor	chr14	protein_coding
ENSG00000155906.15	1.01492524	2.01117122	0.00142109	0.00453795	1.11	0.82	2.27	2.95	RMND1	required for meiotic nuclear division 1 homolog (S. cerevisiae)	chr6	protein_coding
ENSG00000213937.3	2.76378248	-0.6684267	4.67E-07	2.84E-06	0	0.11	0.33	0.66	CLDN9	claudin 9	chr16	protein_coding
ENSG00000239775.1	7.04690795	-1.6136004	1.16E-08	8.76E-08	0	0	0.95	0.58	AC017116.11	--	chr7	sense_overlapping
ENSG00000179271.2	-1.2646643	9.34681258	1.15E-05	5.50E-05	418.23	417.32	236.95	226.04	GADD45GIP1	growth arrest and DNA-damage-inducible, gamma interacting protein 1	chr19	protein_coding
ENSG00000178921.12	-1.647776	6.5022309	1.73E-08	1.28E-07	22.12	19.27	9.05	8.55	PFAS	phosphoribosylformylglycinamidine synthase	chr17	protein_coding
ENSG00000256894.1	5.66409976	-2.616209	0.00086564	0.00288128	0	0	0.08	0.4	RP11-283G6	--	chr12	antisense
ENSG00000237037.8	1.54249417	1.1240046	1.19E-05	5.67E-05	0.26	0.11	0.66	0.79	NDUFA6-AS	NDUFA6 antisense RNA 1 (head to head)	chr22	processed_transcript
ENSG00000099800.6	-1.0625718	6.68256556	0.00022441	0.0008412	59.9	54.02	38.04	34.57	TIMM13	translocase of inner mitochondrial membrane 13 homolog (yeast)	chr19	protein_coding
ENSG00000167994.10	-1.3400809	5.4571478	4.21E-06	2.19E-05	20.88	19.67	10.95	10.38	RAB3IL1	RAB3A interacting protein (rabin3)-like 1	chr11	protein_coding
ENSG00000156931.14	2.06393866	1.67182877	1.58E-09	1.35E-08	0.16	0.12	0.65	0.92	VPS8	vacuolar protein sorting 8 homolog (S. cerevisiae)	chr3	protein_coding
ENSG00000169122.10	-10.310298	1.25909634	3.04E-37	1.97E-35	1.11	1.15	0	0	FAM110B	family with sequence similarity 110, member B	chr8	protein_coding
ENSG00000268896.1	-1.8662066	-2.0296004	0.00956893	0.02497848	0.24	0.89	0.14	0.24	RP11-256I23	--	chr2	antisense
ENSG00000135097.5	4.02452461	-0.5056631	1.29E-11	1.43E-10	0.02	0.02	0.6	0.25	MSI1	musashi RNA-binding protein 1	chr12	protein_coding
ENSG00000162687.15	-5.2127486	-3.0337949	0.00538165	0.01488851	0	0.03	0	0	KCNT2	potassium channel, sodium activated subfamily T, member 2	chr1	protein_coding
ENSG00000169764.13	1.87023522	3.21090549	1.47E-09	1.26E-08	0.75	1.46	4.3	6.43	UGP2	UDP-glucose pyrophosphorylase 2	chr2	protein_coding
ENSG00000271344.1	2.6544571	-1.8987001	0.00119227	0.00385709	0.04	0.02	0.3	0.28	RP11-274B2	--	chr7	lincRNA
ENSG00000155367.14	-1.7623824	-0.1636953	3.43E-05	0.00015114	0.5	0.26	0.21	0.1	PPM1J	protein phosphatase, Mg2+/Mn2+ dependent, 1J	chr1	protein_coding
ENSG00000147010.16	-1.5847348	5.05266529	6.95E-08	4.77E-07	6.84	7.87	2.95	3.59	SH3KBP1	SH3-domain kinase binding protein 1	chrX	protein_coding
ENSG00000171766.14	8.27118393	-0.5532683	5.69E-15	9.08E-14	0	0	0.16	0.71	GATM	glycine amidinotransferase (L-arginine:glycine amidinotransferase)	chr15	protein_coding
ENSG00000176593.6	9.01359651	0.10662656	1.85E-21	5.00E-20	0	0	0.31	0.22	CTD-2368P2	--	chr19	processed_transcript
ENSG00000197857.12	1.59600774	0.24116123	5.76E-05	0.00024305	0.17	0.14	0.58	0.71	ZNF44	zinc finger protein 44	chr19	protein_coding
ENSG00000261534.1	-1.0396545	1.32283307	0.00192273	0.00590691	0.71	0.7	0.52	0.4	RP11-244O1	--	chr9	sense_overlapping
ENSG00000133302.11	3.26937084	0.65442988	3.08E-14	4.53E-13	0.03	0.05	0.31	0.66	ANKRD32	ankyrin repeat domain 32	chr5	protein_coding
ENSG00000003137.7	1.45519544	2.32514167	4.11E-06	2.14E-05	0.34	0.55	1.56	1.67	CYP26B1	cytochrome P450, family 26, subfamily B, polypeptide 1	chr2	protein_coding
ENSG00000255152.7	-2.0008983	-1.3403885	0.00075675	0.00255792	0.55	0.55	0.27	0.1	MSH5-SAPC	MSH5-SAPCD1 readthrough (NMD candidate)	chr6	protein_coding
ENSG00000143344.14	3.15807128	2.53576103	4.31E-20	1.04E-18	0.15	0.2	1.77	2.4	RGL1	ral guanine nucleotide dissociation stimulator-like 1	chr1	protein_coding
ENSG00000198721.11	-7.9579648	4.11618719	2.01E-69	7.66E-67	18.2	16.66	0.06	0.12	ECI2	enoyl-CoA delta isomerase 2	chr6	protein_coding
ENSG00000144935.13	4.17500786	0.1504352	3.45E-15	5.60E-14	0.02	0.02	0.28	0.63	TRPC1	transient receptor potential cation channel, subfamily C, member 1	chr3	protein_coding
ENSG00000277150.1	-1.8107005	1.89356925	3.95E-08	2.78E-07	2.6	2.69	1.22	0.79	F8A3	coagulation factor VIII-associated 3	chrX	protein_coding
ENSG00000113448.15	1.46664875	2.17005766	4.94E-06	2.54E-05	0.11	0.21	0.44	0.74	PDE4D	phosphodiesterase 4D, cAMP-specific	chr5	protein_coding
ENSG00000116525.12	1.69537084	2.73816619	6.43E-08	4.44E-07	0.61	0.8	3.12	2.93	TRIM62	tripartite motif containing 62	chr1	protein_coding
ENSG00000243566.5	-1.6922912	0.03790851	3.45E-05	0.0001518	0.44	0.52	0.23	0.17	UPK3B	uroplakin 3B	chr7	protein_coding
ENSG00000121892.13	1.45110447	3.30846261	1.67E-06	9.31E-06	0.57	0.56	1.55	2.58	PDS5A	PDS5 cohesin associated factor A	chr4	protein_coding
ENSG00000180376.15	4.28765897	1.3799615	1.65E-23	5.16E-22	0.07	0.05	0.88	2.43	CCDC66	coiled-coil domain containing 66	chr3	protein_coding
ENSG00000234327.6	-1.0928029	1.412593	0.00100301	0.00329507	4.12	2.62	2.28	1.94	AC012146.7	--	chr17	processed_transcript
ENSG00000168143.8	6.75188369	-1.8412616	2.24E-07	1.42E-06	0	0	0.09	0.18	FAM83B	family with sequence similarity 83, member B	chr6	protein_coding
ENSG00000197429.9	1.79625325	1.79869768	7.31E-08	5.00E-07	0.46	0.32	1.57	2.07	IPP	intracisternal A particle-promoted polypeptide	chr1	protein_coding
ENSG00000152475.6	2.22571052	1.3302214	5.11E-10	4.64E-09	0.36	0.3	2.47	1.67	ZNF837	zinc finger protein 837	chr19	protein_coding
ENSG00000011105.10	1.30410729	4.54143515	9.23E-06	4.51E-05	2.56	2.09	7.25	8.11	TSPAN9	tetraspanin 9	chr12	protein_coding
ENSG00000134970.13	2.10266287	1.6497433	1.01E-09	8.83E-09	0.19	0.31	0.78	2.07	TMED7	transmembrane emp24 protein transport domain containing 7	chr5	protein_coding
ENSG00000179862.6	-2.2489706	3.0158881	1.02E-12	1.28E-11	9.08	7.32	2.26	2.34	CITED4	Cbp/p300-interacting transactivator, with Glu/Asp-rich carboxy-terminal domain, 4	chr1	protein_coding
ENSG00000174482.9	-9.4002981	0.35926205	1.75E-26	6.66E-25	0.66	0.67	0	0	LINGO2	leucine rich repeat and Ig domain containing 2	chr9	protein_coding
ENSG00000142676.11	-1.6333005	9.7898504	2.00E-08	1.46E-07	1823.38	1593.72	730.03	738.65	RPL11	ribosomal protein L11	chr1	protein_coding
ENSG00000183671.11	-3.3567949	-0.0692982	1.93E-12	2.35E-11	0.51	0.52	0.05	0.08	GPR1	G protein-coupled receptor 1	chr2	protein_coding
ENSG00000131187.8	-1.2446061	2.29431108	6.60E-05	0.00027587	3.29	2.12	1.28	1.77	F12	coagulation factor XII (Hageman factor)	chr5	protein_coding
ENSG00000109738.9	-3.8972146	-2.4440147	0.00066294	0.00226478	0.07	0.05	0	0.01	GLRB	glycine receptor, beta	chr4	protein_coding
ENSG00000174928.14	3.08502842	0.21389336	9.96E-12	1.12E-10	0.16	0.03	0.71	1.46	C3orf33	chromosome 3 open reading frame 33	chr3	protein_coding
ENSG00000106392.9	3.60577128	0.85924666	7.73E-17	1.44E-15	0.04	0.02	0.26	0.82	C1GALT1	core 1 synthase, glycoprotein-N-acetylgalactosamine 3-beta-galactosyltransferase 1	chr7	protein_coding
ENSG00000169740.12	-1.0724746	3.80395824	0.00028869	0.0010543	11.28	13.08	7.62	7.8	ZNF32	zinc finger protein 32	chr10	protein_coding
ENSG00000176896.8	1.84987825	-1.0792577	0.00121058	0.00391053	0.02	0.07	0.2	0.24	TCEANC	transcription elongation factor A (SII) N-terminal and central domain containing	chrX	protein_coding
ENSG00000281392.1	4.71439462	-1.4742853	3.01E-07	1.88E-06	0	0.01	0.12	0.15	LINC00506	long intergenic non-protein coding RNA 506	chr3	lincRNA
ENSG00000108175.15	-2.2888464	5.41865704	2.39E-14	3.57E-13	6.94	7.7	2.08	1.91	ZMIZ1	zinc finger, MIZ-type containing 1	chr10	protein_coding
ENSG00000272009.1	1.35505331	-1.0974463	0.01224023	0.03086062	0.31	0.21	1.31	0.52	RP1-313I6.12	--	chr6	antisense
ENSG00000142188.15	1.78441965	2.52465913	2.03E-08	1.49E-07	0.68	1.06	3.2	4.79	TMEM50B	transmembrane protein 50B	chr21	protein_coding
ENSG00000131188.10	-1.316912	4.24318102	8.09E-06	4.01E-05	14.49	13.62	7.87	7.15	PRR7	proline rich 7 (synaptic)	chr5	protein_coding
ENSG00000279579.2	-5.7759286	-2.7103808	0.00047761	0.00167191	0.38	0	0	0	bP-2189O9.3	--	chr21	lincRNA
ENSG00000229869.1	4.88946445	-3.0622981	0.01889873	0.04519557	0	0	0.16	0.4	RP11-363N2	--	chr10	antisense
ENSG00000114026.20	-1.0829422	4.15209957	0.00023196	0.00086721	6.28	6.67	4.31	3.82	OGG1	8-oxoguanine DNA glycosylase	chr3	protein_coding
ENSG00000278558.3	3.51810182	-1.7059563	2.93E-05	0.00013031	0.04	0	0.51	0.24	TMEM191B	transmembrane protein 191B	chr22	protein_coding
ENSG00000166704.10	6.38840955	1.66363145	9.52E-35	5.43E-33	0	0.02	0.81	0.93	ZNF606	zinc finger protein 606	chr19	protein_coding
ENSG00000231177.4	1.39072962	-0.4017381	0.0016763	0.00522485	0.2	0.26	1.06	0.55	LINC00852	long intergenic non-protein coding RNA 852	chr3	antisense
ENSG00000269926.1	-1.3205533	0.31333433	0.00047684	0.00167191	2.56	0.7	0.68	1.06	RP11-442H2	--	chr10	antisense
ENSG00000179456.10	2.1450288	-0.6372733	1.94E-05	8.92E-05	0.03	0.06	0.21	0.3	ZBTB18	zinc finger and BTB domain containing 18	chr1	protein_coding
ENSG00000269693.1	1.15589255	-0.1136258	0.00532358	0.0148859	0.12	0.02	0.26	0.18	CTD-2192J1	--	chr19	protein_coding
ENSG00000158769.16	3.54219145	5.87874251	7.73E-29	3.36E-27	1.71	1.39	22.8	25.52	F11R	F11 receptor	chr1	protein_coding
ENSG00000185278.13	1.58770445	-0.2634623	0.00029313	0.00106971	0.01	0.02	0.08	0.05	ZBTB37	zinc finger and BTB domain containing 37	chr1	protein_coding
ENSG00000165632.7	1.13364523	4.36265826	0.00011384	0.00045228	1.73	2.54	6.02	6.46	TAF3	TAF3 RNA polymerase II, TATA box binding protein (TBP)-associated factor, 140kDa	chr10	protein_coding
ENSG00000110375.2	2.86793214	-2.1598594	0.00354424	0.01027109	0	0.06	0.31	0.33	UPK2	uroplakin 2	chr11	protein_coding
ENSG00000060140.7	3.70339362	-0.7622253	2.06E-09	1.73E-08	0.01	0.03	0.2	0.46	STYK1	serine/threonine/tyrosine kinase 1	chr12	protein_coding
ENSG00000265750.1	2.467358	-0.9070948	1.60E-05	7.49E-05	0.11	0.19	1.31	0.96	RP11-799B1	--	chr18	lincRNA
ENSG00000231806.2	1.03987121	2.29063454	0.00088773	0.0029491	2.21	2.17	4.73	7.3	PCAT7	prostate cancer associated transcript 7 (non-protein coding)	chr9	antisense
ENSG00000187231.12	1.39250233	2.4214492	9.40E-06	4.59E-05	0.16	0.28	0.58	0.98	SESTD1	SEC14 and spectrin domains 1	chr2	protein_coding
ENSG00000167114.11	-1.2854628	7.2801759	8.66E-06	4.26E-05	60.67	54.02	33.21	29.41	SLC27A4	solute carrier family 27 (fatty acid transporter), member 4	chr9	protein_coding
ENSG00000213949.7	1.29668802	2.24982668	4.14E-05	0.0001802	0.23	0.18	0.61	0.72	ITGA1	integrin, alpha 1	chr5	protein_coding
ENSG00000073910.18	2.6434911	0.92622293	1.39E-11	1.54E-10	0.02	0.04	0.3	0.2	FRY	furry homolog (Drosophila)	chr13	protein_coding
ENSG00000259623.1	2.03467629	1.23855479	1.25E-08	9.40E-08	0.23	0.26	1.09	1.59	RP11-156E6.	--	chr17	sense_overlapping
ENSG00000152455.14	2.20304981	0.70628918	1.58E-08	1.18E-07	0.11	0.16	0.56	1.11	SUV39H2	suppressor of variegation 3-9 homolog 2 (Drosophila)	chr10	protein_coding
ENSG00000081277.10	-4.6786453	2.69851045	1.11E-35	6.81E-34	1.91	1.82	0.01	0.17	PKP1	plakophilin 1	chr1	protein_coding
ENSG00000144567.9	-1.2529384	7.2151341	1.43E-05	6.79E-05	37.58	35.79	20.91	20.1	FAM134A	family with sequence similarity 134, member A	chr2	protein_coding
ENSG00000278535.3	1.30552581	4.17977429	1.03E-05	4.96E-05	5.72	4.79	17.3	17.36	DHRS11	dehydrogenase/reductase (SDR family) member 11	chr17	protein_coding
ENSG00000115594.10	1.2926616	1.05966984	0.00025688	0.00095265	0.15	0.14	0.27	0.7	IL1R1	interleukin 1 receptor, type I	chr2	protein_coding
ENSG00000122705.15	-1.2686785	8.50289008	1.09E-05	5.25E-05	309.28	362.93	180.51	191.17	CLTA	clathrin, light chain A	chr9	protein_coding
ENSG00000234665.7	-4.8790778	2.47562976	8.05E-35	4.65E-33	5.21	5.68	0.4	0.1	RP11-262H1	--	chr9	lincRNA
ENSG00000165730.13	3.22319278	-0.0056794	3.54E-11	3.72E-10	0	0.08	0.33	0.65	STOX1	storkhead box 1	chr10	protein_coding
ENSG00000101350.7	1.83261383	5.1512233	6.20E-10	5.56E-09	1.95	2.17	9.42	10.16	KIF3B	kinesin family member 3B	chr20	protein_coding
ENSG00000217576.7	1.34889564	0.52724075	0.00034375	0.00124082	0.18	0.15	0.57	0.55	RP11-248G5	--	chr13	processed_transcript
ENSG00000151553.13	2.52041255	1.30332116	6.71E-12	7.68E-11	0.08	0.1	0.49	0.91	FAM160B1	family with sequence similarity 160, member B1	chr10	protein_coding
ENSG00000090905.16	1.28687496	3.79392391	1.57E-05	7.36E-05	0.75	0.78	2.34	2.66	TNRC6A	trinucleotide repeat containing 6A	chr16	protein_coding
ENSG00000259583.2	-1.3467741	1.29006855	6.32E-05	0.00026504	0.4	0.43	0.15	0.28	RP11-66B24.	--	chr15	antisense
ENSG00000100321.13	1.90188243	3.9463291	3.58E-10	3.34E-09	1.06	0.83	4.79	4.61	SYNGR1	synaptogyrin 1	chr22	protein_coding
ENSG00000114446.4	1.70752742	2.73803767	5.10E-08	3.55E-07	0.77	0.79	2.37	4.45	IFT57	intraflagellar transport 57	chr3	protein_coding
ENSG00000259426.4	3.28631471	0.89276795	1.77E-15	2.95E-14	0.14	0.09	1.53	1.5	RP11-253M7	--	chr15	antisense
ENSG00000171992.11	-3.2540227	6.11550235	2.12E-25	7.54E-24	9.86	10.23	1.27	1.54	SYNPO	synaptopodin	chr5	protein_coding
ENSG00000187068.2	6.76327383	-1.8344055	2.24E-07	1.42E-06	0	0	0.07	0.08	C3orf70	chromosome 3 open reading frame 70	chr3	protein_coding
ENSG00000134250.16	-1.2148538	6.705092	2.60E-05	0.00011714	6.83	7.87	4.02	4.42	NOTCH2	notch 2	chr1	protein_coding
ENSG00000147883.10	-1.6487719	4.99063557	2.17E-08	1.58E-07	9.59	9.76	3.6	4.64	CDKN2B	cyclin-dependent kinase inhibitor 2B (p15, inhibits CDK4)	chr9	protein_coding
ENSG00000258947.5	1.21002985	0.72888329	0.00088092	0.0029285	0.29	0.17	0.69	0.75	TUBB3	tubulin, beta 3 class III	chr16	protein_coding
ENSG00000213214.4	5.31297515	4.01895829	2.37E-46	2.70E-44	0.13	0.4	14.69	13.11	ARHGEF35	Rho guanine nucleotide exchange factor (GEF) 35	chr7	protein_coding
ENSG00000136935.12	1.22309375	4.10024968	3.59E-05	0.00015754	1.26	2.19	4.23	6.54	GOLGA1	golgin A1	chr9	protein_coding
ENSG00000272223.1	5.49884361	-2.7225181	0.00086564	0.00288128	0	0	0.76	0	RP1-20C7.6	--	chr6	antisense
ENSG00000107263.17	-1.3533883	7.22919657	2.94E-06	1.58E-05	26.74	27.05	14.47	13.55	RAPGEF1	Rap guanine nucleotide exchange factor (GEF) 1	chr9	protein_coding
ENSG00000251246.1	-1.5910181	1.78922778	1.17E-06	6.66E-06	3.9	4.11	1.81	1.72	RP11-540D1	--	chr1	protein_coding
ENSG00000149932.15	-1.5379548	4.25181376	2.25E-07	1.43E-06	11.97	11.07	5.6	4.96	TMEM219	transmembrane protein 219	chr16	protein_coding
ENSG00000280417.1	1.45167142	-1.033758	0.00751458	0.02012506	0.15	0.03	0.29	0.38	RP11-5O17.1	--	chr3	TEC
ENSG00000144893.11	3.54122413	-0.6279887	1.20E-09	1.03E-08	0	0.01	0.09	0.1	MED12L	mediator complex subunit 12-like	chr3	protein_coding
ENSG00000115816.12	2.90984592	2.27298013	3.44E-17	6.60E-16	0.3	0.21	1.22	4.01	CEBPZ	CCAAT/enhancer binding protein (C/EBP), zeta	chr2	protein_coding
ENSG00000103021.8	1.15511956	3.63053191	0.00010551	0.00042337	1.14	1.2	3.04	3.92	CCDC113	coiled-coil domain containing 113	chr16	protein_coding

ENSG00000236081.1	-3.170931	3.92371532	7.84E-23	2.31E-21	32.16	37.33	6.74	3.51	ELFN1-AS1	ELFN1 antisense RNA 1	chr7	lincRNA
ENSG00000271270.4	-2.6439302	1.16292499	7.01E-13	8.93E-12	0.71	0.63	0.13	0.15	TMCC1-AS1	TMCC1 antisense RNA 1 (head to head)	chr3	antisense
ENSG00000165434.7	1.6709748	-0.3269354	0.00022232	0.00083364	0.02	0.04	0.14	0.13	PGM2L1	phosphoglucomutase 2-like 1	chr11	protein_coding
ENSG00000188199.9	3.44522375	-1.3220469	1.32E-06	7.43E-06	0	0.02	0.22	0.04	NUTM2B	NUT family member 2B	chr10	protein_coding
ENSG00000117425.12	2.00055063	-0.9784648	0.00015102	0.00058343	0.05	0.01	0.13	0.17	PTCH2	patched 2	chr1	protein_coding
ENSG00000156052.9	1.02412814	2.14903906	0.00120293	0.00388947	0.27	0.44	0.72	1.21	GNAQ	guanine nucleotide binding protein (G protein), q polypeptide	chr9	protein_coding
ENSG00000139970.15	2.89968835	3.96058944	7.31E-20	1.74E-18	0.83	0.67	7.4	7.62	RTN1	reticulon 1	chr14	protein_coding
ENSG00000143228.11	2.29139225	1.64102108	4.87E-11	5.06E-10	0.48	0.34	1.41	3.96	NUF2	NUF2, NDC80 kinetochore complex component	chr1	protein_coding
ENSG00000109944.9	1.87019748	0.26577972	3.75E-06	1.97E-05	0.08	0.11	0.45	0.5	C11orf63	chromosome 11 open reading frame 63	chr11	protein_coding
ENSG00000100532.10	2.06368176	-0.4930794	1.59E-05	7.44E-05	0.01	0.18	0.2	0.85	CGRRF1	cell growth regulator with ring finger domain 1	chr14	protein_coding
ENSG00000087842.9	-1.6305015	4.56199638	3.77E-08	2.66E-07	19.31	18.23	6.39	9.84	PIR	pirin (iron-binding nuclear protein)	chrX	protein_coding
ENSG00000136478.6	1.14606099	4.12811235	0.00010239	0.00041167	1.33	1.89	4.14	5.37	TEX2	testis expressed 2	chr17	protein_coding
ENSG00000205853.9	2.35291767	-2.4825637	0.01923175	0.0458365	0.04	0	0.09	0.19	RFPL3S	RFPL3 antisense	chr22	protein_coding
ENSG00000125875.12	1.5135655	4.95352109	2.63E-07	1.65E-06	2.78	3.03	11.56	10.51	TBC1D20	TBC1 domain family, member 20	chr20	protein_coding
ENSG00000196440.10	-2.2100012	-0.5835166	4.10E-06	2.14E-05	0.11	0.11	0.05	0.01	ARMCX4	armadillo repeat containing, X-linked 4	chrX	protein_coding
ENSG00000165487.12	2.51829335	1.46454296	3.31E-12	3.92E-11	0.2	0.47	1.8	3.29	MICU2	mitochondrial calcium uptake 2	chr13	protein_coding
ENSG00000273565.1	2.34586639	-0.9964725	5.34E-05	0.00022654	0.16	0.29	1.5	1.61	CTD-3075F1	--	chr14	lincRNA
ENSG00000054267.19	3.48766496	2.24629474	4.96E-22	1.39E-20	0.03	0.16	0.87	2.01	ARID4B	AT rich interactive domain 4B (RBP1-like)	chr1	protein_coding
ENSG00000226738.1	-6.8252312	-1.9700183	2.24E-07	1.42E-06	2.38	1.99	0	0	CTA-384D8.3	--	chr22	antisense
ENSG00000229873.1	2.90879791	-1.4003822	2.09E-05	9.56E-05	0.08	0.08	1.01	0.73	OGFR-AS1	OGFR antisense RNA 1	chr20	antisense
ENSG00000106462.9	1.33345861	4.42342672	6.22E-06	3.14E-05	2.69	3.74	10.41	11.16	EZH2	enhancer of zeste 2 polycomb repressive complex 2 subunit	chr7	protein_coding
ENSG00000227403.1	-5.941432	-0.0237185	8.47E-21	2.16E-19	0.52	0.81	0.03	0	AC009299.3	--	chr2	lincRNA
ENSG00000278727.1	-3.1030297	-1.6614745	8.20E-06	4.05E-05	0.11	0.11	0	0.03	AC000403.4	--	chr13	lincRNA
ENSG00000197756.8	-1.7679639	9.29001695	1.46E-09	1.25E-08	198.11	147.41	66.55	68.86	RPL37A	ribosomal protein L37a	chr2	protein_coding
ENSG00000138081.18	1.35385167	3.72767331	6.04E-06	3.06E-05	1.28	1.3	3.26	5.61	FBXO11	F-box protein 11	chr2	protein_coding
ENSG00000257913.2	1.26467042	1.56577176	0.00014951	0.00057808	0.47	0.76	2.15	1.76	RP11-386G1	--	chr12	antisense
ENSG00000152782.15	1.56243732	1.64225264	2.68E-06	1.45E-05	0.17	0.18	0.66	0.71	PANK1	pantothenate kinase 1	chr10	protein_coding
ENSG00000078237.5	1.63091831	2.42506461	2.90E-07	1.82E-06	0.66	0.84	2.42	3.79	C12orf5	--	chr12	protein_coding
ENSG00000267368.1	2.47381779	3.78152521	2.28E-15	3.77E-14	2.69	2.32	20.89	16.17	UPK3BL	uroplakin 3B-like	chr7	protein_coding
ENSG00000221955.9	-1.5250148	3.98575375	3.20E-07	1.99E-06	5.01	5.32	2.5	2.27	SLC12A8	solute carrier family 12, member 8	chr3	protein_coding
ENSG00000139921.11	2.22048982	2.28327386	1.48E-11	1.63E-10	0.42	0.26	1.24	3.02	TMX1	thioredoxin-related transmembrane protein 1	chr14	protein_coding
ENSG00000104324.14	-2.1457779	1.68220142	2.46E-10	2.33E-09	1.88	2.46	0.56	0.74	CPQ	carboxypeptidase Q	chr8	protein_coding
ENSG00000164761.7	-6.0005178	-2.5673179	0.00014842	0.00057423	0.07	0.09	0	0	TNFRSF11B	tumor necrosis factor receptor superfamily, member 11b	chr8	protein_coding
ENSG00000276122.1	-7.3040449	-1.5796114	1.11E-09	9.61E-09	0.31	0.15	0	0	RP11-143E2	--	chr12	lincRNA
ENSG00000198846.5	-6.8830937	-1.9248975	1.35E-07	8.87E-07	0.08	0.1	0	0	TOX	thymocyte selection-associated high mobility group box	chr8	protein_coding
ENSG00000240032.1	4.92065931	-3.045493	0.0100455	0.02582742	0	0	0.36	0	RP11-274H2	--	chr3	antisense
ENSG00000139515.5	-11.652393	2.60061027	1.80E-55	3.51E-53	4.11	3.76	0	0	PDX1	pancreatic and duodenal homeobox 1	chr13	protein_coding
ENSG00000100554.10	1.34331992	3.5372496	7.82E-06	3.88E-05	2.9	2.81	7.67	11.73	ATP6V1D	ATPase, H+ transporting, lysosomal 34kDa, V1 subunit D	chr14	protein_coding
ENSG00000163961.4	1.38052809	2.72027309	7.80E-06	3.87E-05	0.54	0.55	1.79	2	RNF168	ring finger protein 168, E3 ubiquitin protein ligase	chr3	protein_coding
ENSG00000153201.14	3.03265226	3.12543672	3.91E-20	9.50E-19	0.13	0.13	0.97	1.88	RANBP2	RAN binding protein 2	chr2	protein_coding
ENSG00000168826.14	1.40452003	1.62931669	2.38E-05	0.000108	0.54	0.38	1.51	1.75	ZBTB49	zinc finger and BTB domain containing 49	chr4	protein_coding
ENSG00000232628.4	5.57545548	-2.6732417	0.00086564	0.00288128	0	0	0.2	0.28	RP11-365O1	--	chr1	sense_intronic
ENSG00000155428.11	1.88411786	-1.8632211	0.01381259	0.03443739	0.04	0.06	0.17	0.3	TRIM74	tripartite motif containing 74	chr7	protein_coding
ENSG00000137492.6	1.26550875	1.6065105	0.00014386	0.00056016	0.41	0.45	0.99	1.75	PRKRIR	protein-kinase, interferon-inducible double stranded RNA dependent inhibitor, repressor of (P58 repressor)	chr11	protein_coding
ENSG00000163449.9	3.52918945	-1.2512508	6.12E-07	3.65E-06	0.02	0	0.19	0.18	TMEM169	transmembrane protein 169	chr2	protein_coding
ENSG00000233198.3	-3.1559581	0.40236226	1.45E-13	1.98E-12	1.07	1.04	0.18	0.14	RNF224	ring finger protein 224	chr9	protein_coding
ENSG00000136811.15	-1.6168092	7.55498582	2.88E-08	2.07E-07	45.73	50.11	20.93	20.68	ODF2	outer dense fiber of sperm tails 2	chr9	protein_coding
ENSG00000249693.2	3.71036418	-2.2232147	0.00023419	0.00087473	0.02	0	0.08	0.26	THEGL	theg spermatid protein-like	chr4	protein_coding
ENSG00000066654.11	1.49215342	1.41675121	1.30E-05	6.16E-05	0.14	0.34	0.64	1.14	THUMPD1	THUMP domain containing 1	chr16	protein_coding
ENSG00000114626.16	-1.6305223	5.73112531	2.65E-08	1.91E-07	31.05	26.7	13.27	11.55	ABTB1	ankyrin repeat and BTB (POZ) domain containing 1	chr3	protein_coding
ENSG00000139218.16	2.82013651	3.25652108	3.42E-18	7.14E-17	0.13	0.22	1.27	1.99	SCAF11	SR-related CTD-associated factor 11	chr12	protein_coding
ENSG00000199266.1	3.71897221	-2.2182862	0.00023419	0.00087473	0.2	0	1.43	2.85	SNORA60	small nucleolar RNA, H/ACA box 60	chr20	snoRNA
ENSG00000257900.2	4.14238929	2.55155553	9.77E-29	4.22E-27	0.88	0.93	24.2	18.49	RP11-454K7.	--	chr14	lincRNA
ENSG00000204519.9	5.33514628	0.9679229	5.30E-25	1.84E-23	0	0.03	0.58	1.07	ZNF551	zinc finger protein 551	chr19	protein_coding
ENSG00000109062.8	-2.011771	7.29872931	9.37E-12	1.06E-10	106.08	100.42	35.13	33.06	SLC9A3R1	solute carrier family 9, subfamily A (NHE3, cation proton antiporter 3), member 3 regulator 1	chr17	protein_coding
ENSG00000239002.3	2.16997593	-1.4723902	0.00061439	0.00211279	0.32	0.17	2.23	0.91	SCARNA10	small Cajal body-specific RNA 10	chr12	snoRNA
ENSG00000129295.7	2.40685132	-1.7604879	0.00140662	0.00449532	0.05	0	0.13	0.27	LRRC6	leucine rich repeat containing 6	chr8	protein_coding
ENSG00000013441.14	2.09364518	2.49670856	9.33E-11	9.37E-10	0.52	0.86	2.46	5.42	CLK1	CDC-like kinase 1	chr2	protein_coding
ENSG00000089116.3	5.63493951	1.23950035	2.04E-28	8.66E-27	0.02	0.03	1.9	1.33	LHX5	LIM homeobox 5	chr12	protein_coding
ENSG00000271127.1	5.19748351	-2.8969879	0.00538165	0.01488851	0	0	0.05	0.16	LL22NC03-N	--	chr22	sense_intronic
ENSG00000154319.13	-5.8225815	3.95094348	3.83E-52	5.96E-50	5.04	6.18	0.07	0.19	FAM167A	family with sequence similarity 167, member A	chr8	protein_coding
ENSG00000273046.1	1.73212547	-1.296187	0.00202074	0.0061882	0.12	0.06	0.62	0.2	RP11-834C1	--	chr12	processed_transcript
ENSG00000264456.1	1.87179549	-1.6704508	0.00486779	0.01371541	0.03	0.07	0.29	0.21	RP11-848P1.	--	chr17	sense_intronic
ENSG00000167925.14	-5.2439671	3.81654489	1.40E-45	1.49E-43	7.25	6.49	0.24	0.24	GHDC	GH3 domain containing	chr17	protein_coding
ENSG00000137269.13	1.21443055	0.94848105	0.00070255	0.00238955	0.18	0.35	0.47	1.19	LRRC1	leucine rich repeat containing 1	chr6	protein_coding
ENSG00000135916.14	1.43225981	7.52612289	7.84E-07	4.59E-06	38.83	33.94	134.97	126.64	ITM2C	integral membrane protein 2C	chr2	protein_coding
ENSG00000240964.3	4.89462967	-3.0595066	0.01889873	0.04519557	0	0	0.34	0.48	RN7SL751P	RNA, 7SL, cytoplasmic 751, pseudogene	chr3	misc_RNA
ENSG00000173391.7	4.28005599	3.28524282	4.56E-33	2.45E-31	0.22	0.4	5.94	10.03	OLR1	oxidized low density lipoprotein (lectin-like) receptor 1	chr12	protein_coding
ENSG00000165474.5	10.3096911	1.33207315	6.26E-36	3.87E-34	0	0	2.09	2.18	GJB2	gap junction protein, beta 2, 26kDa	chr13	protein_coding
ENSG00000133138.18	5.35726909	0.69421728	2.04E-23	6.27E-22	0.01	0	0.22	0.48	TBC1D8B	TBC1 domain family, member 8B (with GRAM domain)	chrX	protein_coding
ENSG00000184838.13	-5.672782	-1.0575379	6.98E-12	7.96E-11	0.28	0.37	0.01	0	PRR16	proline rich 16	chr5	protein_coding
ENSG00000086065.12	1.53460664	4.40129604	2.36E-07	1.50E-06	3.54	4.91	11.39	21.44	CHMP5	charged multivesicular body protein 5	chr9	protein_coding
ENSG00000152944.7	1.23060018	0.88856173	0.00058148	0.0020123	0.48	0.42	0.73	2.1	MED21	mediator complex subunit 21	chr12	protein_coding
ENSG00000263004.1	1.6132056	1.67807863	1.29E-06	7.29E-06	1.8	1.67	7.41	6.76	RP11-166P1	--	chr17	lincRNA
ENSG00000148655.13	-1.4652778	1.3755793	1.21E-05	5.79E-05	0.66	0.56	0.25	0.34	C10orf11	chromosome 10 open reading frame 11	chr10	protein_coding
ENSG00000276368.1	1.9667032	-0.4083181	2.95E-05	0.00013143	0.41	0.73	4.72	1.34	HIST1H2AJ	histone cluster 1, H2aj	chr6	protein_coding
ENSG00000198467.12	-2.6309143	11.7656839	2.54E-18	5.36E-17	1380.83	1584.92	322.56	314.65	TPM2	tropomyosin 2 (beta)	chr9	protein_coding
ENSG00000268129.1	1.26685067	1.77330029	0.00010635	0.00042567	3	2.63	7.37	10.78	RP11-91G21	--	chr3	lincRNA
ENSG00000197608.10	1.87886733	0.98659696	2.95E-07	1.85E-06	0.06	0.27	0.61	0.99	ZNF841	zinc finger protein 841	chr19	protein_coding
ENSG00000148400.9	-1.2238552	5.882571	2.42E-05	0.00010936	7.12	7.15	4.49	3.63	NOTCH1	notch 1	chr9	protein_coding
ENSG00000160679.11	-1.2045047	7.11872565	2.99E-05	0.00013272	36.73	38.09	22.16	21.06	CHTOP	chromatin target of PRMT1	chr1	protein_coding
ENSG00000153714.5	-4.4279027	3.1536971	4.02E-35	2.37E-33	7.52	8.23	0.31	0.65	LURAP1L	leucine rich adaptor protein 1-like	chr9	protein_coding
ENSG00000019485.11	-1.5405193	2.26804665	1.05E-06	6.02E-06	0.53	0.51	0.2	0.28	PRDM11	PR domain containing 11	chr11	protein_coding
ENSG00000176771.14	-8.0595661	-0.9167936	2.49E-14	3.72E-13	0.09	0.12	0	0	NCKAP5	NCK-associated protein 5	chr2	protein_coding
ENSG00000236663.1	-6.2754363	-2.3796349	2.70E-05	0.00012094	0.57	0.54	0	0	AP001631.9	--	chr21	antisense
ENSG00000182873.5	-3.4722668	-0.6950969	1.54E-10	1.51E-09	0.72	0.28	0	0.11	RP11-181G1	--	chr1	antisense
ENSG00000261054.1	3.65654182	-2.2619288	0.00039332	0.00140265	0.01	0	0.1	0.12	RP11-6O2.4	--	chr15	antisense
ENSG00000102452.14	-5.9588029	0.50417379	3.62E-25	1.27E-23	0.39	0.23	0.01	0	NALCN	sodium leak channel, non selective	chr13	protein_coding
ENSG00000153563.14	1.08985644	-0.6008175	0.01915356	0.04574946	0.1	0.06	0.21	0.26	CD8A	CD8a molecule	chr2	protein_coding
ENSG00000138592.12	2.35048159	2.63895417	4.99E-13	6.47E-12	0.1	0.07	0.4	0.77	USP8	ubiquitin specific peptidase 8	chr15	protein_coding
ENSG00000274372.3	-2.0668575	-2.3354085	0.01324986	0.0331641	0.22	0.57	0	0.22	RP11-94I2.4	--	chr1	lincRNA
ENSG00000214736.6	-1.2957889	5.20257883	8.76E-06	4.31E-05	72.01	60.82	36.71	35.4	TOMM6	translocase of outer mitochondrial membrane 6 homolog (yeast)	chr6	protein_coding
ENSG00000076356.6	-1.5104499	5.54778203	2.39E-07	1.52E-06	4.77	5.03	2.38	2.2	PLXNA2	plexin A2	chr1	protein_coding
ENSG00000154174.7	1.00283357	3.43177442	0.00078213	0.00263515	1.27	1.34	2.66	4.37	TOMM70A	translocase of outer mitochondrial membrane 70 homolog A (S. cerevisiae)	chr3	protein_coding
ENSG00000279605.1	-2.033501	2.05630807	5.29E-10	4.79E-09	1.79	0.37	0.32	0.39	RP11-661A1	--	chr8	TEC
ENSG00000127528.5	-1.1245631	2.76506802	0.00024435	0.00091082	4.24	3.19	2.71	1.82	KLF2	Kruppel-like factor 2	chr19	protein_coding
ENSG00000167291.14	-1.4076902	6.55714494	1.24E-06	7.04E-06	9.84	9.77	4.85	5	TBC1D16	TBC1 domain family, member 16	chr17	protein_coding
ENSG00000171044.9	5.57865117	-0.7515672	2.75E-12	3.28E-11	0.01	0	0.23	0.28	XKR6	XK, Kell blood group complex subunit-related family, member 6	chr8	protein_coding
ENSG00000157106.15	1.15080478	3.80547933	0.00010719	0.00042868	0.38	0.43	1.18	1.19	SMG1	SMG1 phosphatidylinositol 3-kinase-related kinase	chr16	protein_coding
ENSG00000078900.13	-1.5628222	1.57023614	2.82E-06	1.52E-05	0.7	0.58	0.32	0.25	TP73	tumor protein p73	chr1	protein_coding
ENSG00000179241.11	-1.688798	4.70555645	1.27E-08	9.55E-08	7.48	8.6	3.62	3	LDLRAD3	low density lipoprotein receptor class A domain containing 3	chr11	protein_coding
ENSG00000153404.12	6.38535349	3.03673575	5.77E-48	7.16E-46	0.01	0.02	1.5	1.49	PLEKHG4B	pleckstrin homology domain containing, family G (with RhoGef domain) member 4B	chr5	protein_coding
ENSG00000272279.1	-5.6557203	-2.7843232	0.00086564	0.00288128	0.39	0.16	0	0	RP11-157J24	--	chr6	lincRNA
ENSG00000258122.1	2.39468228	-2.0694926	0.0061421	0.01678695	0	0.2	0.59	0.82	RP11-61A14	--	chr16	antisense
ENSG00000128567.15	-1.9325065	5.43481298	7.00E-11	7.15E-10	23.39	29.33	10.02	8.32	PODXL	podocalyxin-like	chr7	protein_coding
ENSG00000253626.3	3.42933577	3.35652645	1.32E-24	4.42E-23	0.37	0.35	5.48	4.91	EIF5AL1	eukaryotic translation initiation factor 5A-like 1	chr10	protein_coding
ENSG00000101109.10	1.44006857	3.8602267	1.43E-06	8.03E-06	0.97	0.98	3.28	3.8	STK4	serine/threonine kinase 4	chr20	protein_coding
ENSG00000121749.14	2.07618759	2.05560484	4.32E-10	3.97E-09	0.18	0.22	0.57	1.7	TBC1D15	TBC1 domain family, member 15	chr12	protein_coding
ENSG00000227057.6	-1.369702	7.46401788	2.23E-06	1.22E-05	90.44	84.31	43.54	46.67	WDR46	WD repeat domain 46	chr6	protein_coding
ENSG00000135905.17	-4.2404723	-1.0891509	2.14E-10	2.05E-09	0.07	0.11	0	0.01	DOCK10	dedicator of cytokinesis 10	chr2	protein_coding
ENSG00000236088.8	1.87444761	0.21938863	5.61E-06	2.85E-05	0.07	0.05	0.21	0.41	COX10-AS1	COX10 antisense RNA 1	chr17	processed_transcript
ENSG00000127870.15	2.85998385	1.44735538	1.55E-14	2.37E-13	0.11	0.15	0.82	1.7	RNF6	ring finger protein (C3H2C3 type) 6	chr13	protein_coding
ENSG00000197558.10	6.17324407	-2.2728252	2.70E-05	0.00012094	0	0	0.01	0.03	SSPO	SCO-spondin	chr7	protein_coding
ENSG00000145536.14	-9.5174054	0.47464125	9.24E-28	3.80E-26	0.43	0.34	0	0	ADAMTS16	ADAM metallopeptidase with thrombospondin type 1 motif, 16	chr5	protein_coding
ENSG00000196876.12	6.46990886	2.03617813	7.20E-39	5.17E-37	0.01	0	0.63	0.83	SCN8A	sodium channel, voltage gated, type VIII alpha subunit	chr12	protein_coding
ENSG00000147874.9	1.58226581	1.49742902	3.45E-06	1.82E-05	0.11	0.18	0.4	0.73	HAUS6	HAUS augmin-like complex, subunit 6	chr9	protein_coding
ENSG00000127311.8	2.2130352	-0.4951651	3.89E-06	2.04E-05	0.01	0.01	0.14	0.03	HELB	helicase (DNA) B	chr12	protein_coding
ENSG00000120896.12	-1.0940814	7.26417442	0.00014357	0.00055927	46.97	47.01	30.43	28.16	SORBS3	sorbin and SH3 domain containing 3	chr8	protein_coding
ENSG00000237978.4	5.34420401	-2.8131047	0.00290522	0.00856312	0	0	0.09	0.1	KCNMB2-AS	KCNMB2 antisense RNA 1	chr3	antisense
ENSG00000073792.14	-2.6672882	2.12551618	2.76E-15	4.52E-14	1.37	1.82	0.32	0.35	IGF2BP2	insulin-like growth factor 2 mRNA binding protein 2	chr3	protein_coding
ENSG00000110844.12	-1.1966849	4.25129322	4.69E-05	0.00020246	5.53	5.57	3.28	3.17	PRPF40B	PRP40 pre-mRNA processing factor 40 homolog B (S. cerevisiae)	chr12	protein_coding
ENSG00000119408.15	-3.5453249	5.53187495	8.87E-29	3.84E-27	17.98	15.8	2.07	1.78	NEK6	NIMA-related kinase 6	chr9	protein_coding
ENSG00000271020.1	7.24773016	-1.4458174	1.11E-09	9.61E-09	0	0	1.08	1.23	RP11-10C24	--	chr3	lincRNA
ENSG00000188322.4	2.84787722	1.38786616	1.92E-14	2.89E-13	0.13	0.06	1.1	0.8	SBK1	SH3 domain binding kinase 1	chr16	protein_coding
ENSG00000173530.5	-1.2550101	4.6403172	1.80E-05	8.33E-05	7.17	8.92	4.37	4.6	TNFRSF10D	tumor necrosis factor receptor superfamily, member 10d, decoy with truncated death domain	chr8	protein_coding
ENSG00000248672.4	3.56186738	-0.1855913	1.90E-11	2.07E-10	0	0.03	0.25	0.3	LY75-CD302	LY75-CD302 readthrough	chr2	protein_coding
ENSG00000130508.9	-10.815542	8.77613893	9.17E-128	5.50E-124	100.79	107.73	0.05	0.1	PXDN	peroxidasin	chr2	protein_coding
ENSG00000267080.4	1.11629021	2.6192455	0.00029803	0.00108626	0.91	1.61	4.22	3.03	ASB16-AS1	ASB16 antisense RNA 1	chr17	antisense
ENSG00000176753.5	5.87712165	-2.4793333	0.00014842	0.00057423	0	0	0.11	0.09	C15orf56	chromosome 15 open reading frame 56	chr15	antisense
ENSG00000262454.1	1.1105548	2.27207903	0.00041491	0.00147418	4.31	3.13	9.69	11.84	RP11-65J21.	--	chr16	lincRNA
ENSG00000175782.9	1.08635705	4.03922719	0.00022993	0.00086069	0.43	0.52	1.17	1.53	SLC35E3	solute carrier family 35, member E3	chr12	protein_coding
ENSG00000050405.12	5.45409148	4.08734117	6.24E-48	7.70E-46	0.11	0.12	4.58	9.32	LIMA1	LIM domain and actin binding 1	chr12	protein_coding
ENSG00000274512.3	-6.8886984	-1.9218624	1.35E-07	8.87E-07	0.04	0.21	0	0	TBC1D3L	TBC1 domain family, member 3L	chr17	protein_coding
ENSG00000232859.8	4.50532071	1.12048174	9.92E-23	2.90E-21	0.02	0.09	1.65	1.57	LYRM9	LYR motif containing 9	chr17	protein_coding
ENSG00000273331.1	-1.8121762	-1.7472904	0.00684841	0.01852289	0.53	0.12	0.07	0.17	TM4SF19-TC	TM4SF19-TCTEX1D2 readthrough (NMD candidate)	chr3	protein_coding
ENSG00000243710.6	2.25263098	-2.1613812	0.01384632	0.03449994	0.01	0.01	0.04	0.06	CFAP57	cilia and flagella associated protein 57	chr1	protein_coding
ENSG00000059377.14	-4.2006458	4.04176566	4.71E-35	2.76E-33	7.16	6.78	0.42	0.58	TBXAS1	thromboxane A synthase 1 (platelet)	chr7	protein_coding
ENSG00000138018.16	1.01445449	2.32292804	0.00114119	0.00370334	0.27	0.38	0.66	1.1	EPT1	ethanolaminephosphotransferase 1	chr2	protein_coding
ENSG00000106351.11	1.46417071	5.28330814	5.68E-07	3.40E-06	3.95	3.01	13.14	12.5	AGFG2	ArfGAP with FG repeats 2	chr7	protein_coding
ENSG00000229299.2	2.11100868	-2.2488815	0.02071392	0.04894569	0	0.18	0.68	0.4	RP4-583P15.	--	chr20	antisense
ENSG00000114735.8	-1.3910765	4.18868959	2.62E-06	1.41E-05	1.2	1.26	0.63	0.62	HEMK1	HemK methyltransferase family member 1	chr3	protein_coding
ENSG00000229647.1	-7.9024186	-1.0583848	2.44E-13	3.25E-12	0.8	0.7	0	0	AC007879.7	--	chr2	lincRNA
ENSG00000102786.13	2.98034091	2.40048113	3.47E-18	7.22E-17	0.04	0.05	0.32	0.55	INTS6	integrator complex subunit 6	chr13	protein_coding
ENSG00000100626.15	2.20917244	0.74661141	1.07E-08	8.19E-08	0.08	0.06	0.42	0.48	GALNT16	polypeptide N-acetylgalactosaminyltransferase 16	chr14	protein_coding
ENSG00000169992.8	-1.2035926	5.71706115	3.31E-05	0.00014602	10.82	10.56	6.48	5.88	NLGN2	neuroligin 2	chr17	protein_coding
ENSG00000140527.13	3.04777815	-1.028848	6.13E-07	3.66E-06	0.02	0.02	0.18	0.24	WDR93	WD repeat domain 93	chr15	protein_coding
ENSG00000010438.15	-9.4447753	0.40338996	6.00E-27	2.37E-25	1.92	1.47	0	0	PRSS3	protease, serine, 3	chr9	protein_coding
ENSG00000240591.1	-5.5223491	-2.8631056	0.00158008	0.00495521	0.2	0.14	0	0	RP11-247I13	--	chr22	antisense
ENSG00000095739.10	2.20240552	2.92794528	4.37E-12	5.11E-11	0.98	1.33	5.73	8.49	BAMBI	BMP and activin membrane-bound inhibitor	chr10	protein_coding
ENSG00000197958.11	-2.051285	10.153197	3.52E-12	4.16E-11	2280.13	2414.72	767.92	739.94	RPL12	ribosomal protein L12	chr9	protein_coding
ENSG00000268475.1	5.48900786	-2.7279255	0.00158008	0.00495521	0	0	0.93	0.16	CTC-435M1(	--	chr19	antisense
ENSG00000264608.1	2.75969356	-2.2296611	0.00281991	0.00840887	0.12	0	0	1.13	RP11-192H2	--	chr17	sense_intronic
ENSG00000140563.13	2.69135032	2.96618888	1.57E-16	2.85E-15	0.16	0.22	1.43	1.89	MCTP2	multiple C2 domains, transmembrane 2	chr15	protein_coding
ENSG00000228812.6	-1.7067571	0.27628656	1.65E-05	7.68E-05	1.65	1.82	0.84	0.58	LAMA5-AS1	LAMA5 antisense RNA 1	chr20	antisense
ENSG00000245552.5	3.74524511	-0.7329453	1.07E-09	9.30E-09	0.02	0.01	0.16	0.23	RP11-712B9.	--	chr11	antisense
ENSG00000164305.16	3.97909633	1.9519117	1.44E-24	4.82E-23	0.07	0.18	2.08	3.18	CASP3	caspase 3, apoptosis-related cysteine peptidase	chr4	protein_coding
ENSG00000118193.10	3.91148462	1.04109074	1.97E-19	4.54E-18	0.01	0.04	0.34	0.74	KIF14	kinesin family member 14	chr1	protein_coding
ENSG00000221916.3	1.13650554	0.43777146	0.00265289	0.00796034	1.11	0.61	3.01	2.09	C19orf73	chromosome 19 open reading frame 73	chr19	protein_coding
ENSG00000138271.5	4.72702494	1.02465916	1.76E-23	5.47E-22	0.03	0.11	2.33	2.64	GPR87	G protein-coupled receptor 87	chr3	protein_coding
ENSG00000234444.8	5.85167916	0.27625388	8.59E-21	2.19E-19	0.01	0	0.31	0.33	ZNF736	zinc finger protein 736	chr7	protein_coding
ENSG00000120800.4	1.4775551	3.92181365	7.73E-07	4.53E-06	0.79	0.63	2.64	2.66	UTP20	UTP20, small subunit (SSU) processome component, homolog (yeast)	chr12	protein_coding
ENSG00000183431.10	-1.1019775	7.71843453	0.00012726	0.00050151	59.83	66.91	37.54	41.18	SF3A3	splicing factor 3a, subunit 3, 60kDa	chr1	protein_coding
ENSG00000250397.2	2.83035235	-0.3432908	1.66E-08	1.23E-07	0.1	0.03	0.67	0.62	RP11-1391J7	--	chr11	antisense
ENSG00000123444.12	-1.1488042	3.89434955	0.00010255	0.00041213	5.33	4.71	2.94	3.11	KBTBD4	kelch repeat and BTB (POZ) domain containing 4	chr11	protein_coding
ENSG00000171551.10	-6.075059	1.94387692	6.36E-39	4.59E-37	2.08	2.22	0.04	0.04	ECEL1	endothelin converting enzyme-like 1	chr2	protein_coding
ENSG00000147955.15	-1.5631553	9.14999595	7.43E-08	5.08E-07	370.62	384.79	177.69	162.4	SIGMAR1	sigma non-opioid intracellular receptor 1	chr9	protein_coding
ENSG00000132950.17	2.90492926	0.42564333	1.72E-11	1.88E-10	0.05	0.09	0.46	0.9	ZMYM5	zinc finger, MYM-type 5	chr13	protein_coding
ENSG00000197969.10	3.73148815	1.06005914	1.11E-18	2.42E-17	0.01	0.02	0.16	0.3	VPS13A	vacuolar protein sorting 13 homolog A (S. cerevisiae)	chr9	protein_coding
ENSG00000270055.1	1.92028564	0.79337014	3.38E-07	2.10E-06	0.17	0.3	0.67	1.71	CTD-3092A1	--	chr15	sense_intronic
ENSG00000152527.12	3.4842322	-1.7261185	4.49E-05	0.00019431	0.01	0	0.05	0.08	PLEKHH2	pleckstrin homology domain containing, family H (with MyTH4 domain) member 2	chr2	protein_coding
ENSG00000212195.2	5.2326735	-2.8777865	0.00290522	0.00856312	0	0	1.47	0.14	U3	--	chr17	snoRNA
ENSG00000170836.10	2.09586951	2.61395285	7.07E-11	7.22E-10	0.38	0.38	1.78	2.6	PPM1D	protein phosphatase, Mg2+/Mn2+ dependent, 1D	chr17	protein_coding
ENSG00000228437.4	5.18753465	-2.9024431	0.00538165	0.01488851	0	0	0	0.24	RP11-400N1	--	chr1	lincRNA
ENSG00000047634.13	2.25675218	-0.3541272	1.79E-06	9.89E-06	0.07	0.06	0.23	0.61	SCML1	sex comb on midleg-like 1 (Drosophila)	chrX	protein_coding
ENSG00000006015.16	-1.0480606	5.92789888	0.00028244	0.00103339	87.25	70.9	49.9	52.16	C19orf60	chromosome 19 open reading frame 60	chr19	protein_coding
ENSG00000141027.19	1.08107366	5.36749345	0.00019263	0.00072913	1.74	2.13	5.28	5.63	NCOR1	nuclear receptor corepressor 1	chr17	protein_coding
ENSG00000171428.12	2.73549948	-2.2477106	0.00174589	0.00541921	0	0.02	0.13	0.06	NAT1	N-acetyltransferase 1 (arylamine N-acetyltransferase)	chr8	protein_coding
ENSG00000170561.11	2.92818955	4.08437766	2.67E-20	6.56E-19	0.94	0.93	9.26	9.74	IRX2	iroquois homeobox 2	chr5	protein_coding
ENSG00000160190.12	1.31447939	3.38454851	1.28E-05	6.09E-05	1.19	1.28	4.24	3.93	SLC37A1	solute carrier family 37 (glucose-6-phosphate transporter), member 1	chr21	protein_coding
ENSG00000132604.9	1.09257281	5.833676	0.00015785	0.00060738	9.25	8.83	25.8	25.58	TERF2	telomeric repeat binding factor 2	chr16	protein_coding
ENSG00000156875.12	1.02487089	2.56539375	0.00092076	0.00305035	1.1	1.14	2.48	3.62	HIAT1	hippocampus abundant transcript 1	chr1	protein_coding

ENSG00000261488.1	2.22313099	-2.1788768	0.02071392	0.04894569	0.03	0.01	0	0.27	RP11-757F18	--	chr3	antisense
ENSG00000230551.4	2.79432164	1.14492754	3.09E-13	4.07E-12	0.04	0.06	0.42	0.5	CTB-89H12.4	--	chr5	processed_transcript
ENSG00000265190.5	-3.1422986	4.98710122	1.42E-23	4.45E-22	11.71	11.49	1.64	1.87	ANXA8	annexin A8	chr10	protein_coding
ENSG00000173013.5	1.3807499	2.1221595	1.56E-05	7.32E-05	0.9	0.94	3	3.36	CCDC96	coiled-coil domain containing 96	chr4	protein_coding
ENSG00000261140.1	4.72173804	-3.1445547	0.01889873	0.04519557	0	0	0.35	0.12	RP11-20I23.6	--	chr16	antisense
ENSG00000118849.8	1.3025209	0.43116358	0.00055696	0.00193276	0.35	0.22	0.96	0.9	RARRES1	retinoic acid receptor responder (tazarotene induced) 1	chr3	protein_coding
ENSG00000090376.7	2.2005004	1.07406908	2.48E-09	2.06E-08	0.06	0.06	0.27	0.45	IRAK3	interleukin-1 receptor-associated kinase 3	chr12	protein_coding
ENSG00000230537.1	-5.3769376	-2.9455251	0.00290522	0.00856312	0.07	0.24	0	0	RP11-305L7.	--	chr9	lincRNA
ENSG00000145945.5	-1.3543008	4.72093308	3.83E-06	2.01E-05	16.29	15.45	8.32	8.22	FAM50B	family with sequence similarity 50, member B	chr6	protein_coding
ENSG00000136866.12	6.41030161	-2.0973515	5.19E-06	2.66E-05	0	0	0.01	0.1	ZFP37	ZFP37 zinc finger protein	chr9	protein_coding
ENSG00000152484.12	2.49197602	2.32757814	7.72E-14	1.09E-12	0.26	0.24	1.36	2.44	USP12	ubiquitin specific peptidase 12	chr13	protein_coding
ENSG00000113758.12	-2.7306261	8.70464512	1.93E-19	4.46E-18	116.68	146.44	27.52	25.17	DBN1	drebrin 1	chr5	protein_coding
ENSG00000270246.3	-5.0252555	-3.1285772	0.0100455	0.02582742	0	0.39	0	0	RP11-701H2	--	chr15	sense_intronic
ENSG00000104626.13	1.09381141	1.40424017	0.00115646	0.00374885	0.24	0.29	0.62	0.88	ERI1	exoribonuclease 1	chr8	protein_coding
ENSG00000171224.8	1.34988304	3.05342062	9.48E-06	4.62E-05	4.22	2.79	12.27	11.61	C10orf35	chromosome 10 open reading frame 35	chr10	protein_coding
ENSG00000151715.6	-4.8713807	-0.5134533	7.25E-15	1.14E-13	0.38	0.46	0.04	0	TMEM45B	transmembrane protein 45B	chr11	protein_coding
ENSG00000131374.13	1.51313308	2.07690459	2.53E-06	1.37E-05	0.22	0.17	0.65	0.82	TBC1D5	TBC1 domain family, member 5	chr3	protein_coding
ENSG00000004700.14	3.72561056	2.20616462	4.64E-24	1.50E-22	0.19	0.08	1.35	3.55	RECQL	RecQ helicase-like	chr12	protein_coding
ENSG00000197601.11	3.26436909	1.55545587	7.44E-18	1.51E-16	0.04	0.08	0.49	1.08	FAR1	fatty acyl CoA reductase 1	chr11	protein_coding
ENSG00000120708.15	-7.4299106	11.011478	4.95E-87	4.95E-84	1148.04	1164.16	9.24	8.61	TGFBI	transforming growth factor, beta-induced, 68kDa	chr5	protein_coding
ENSG00000076351.11	1.65585849	6.40786281	1.52E-08	1.14E-07	5.17	5.2	21.94	21.61	SLC46A1	solute carrier family 46 (folate transporter), member 1	chr17	protein_coding
ENSG00000106692.12	1.24176449	0.2311319	0.00163639	0.00511642	0.05	0.09	0.13	0.31	FKTN	fukutin	chr9	protein_coding
ENSG00000164338.8	1.07146034	2.37410537	0.00059371	0.00205136	0.3	0.75	1.06	1.88	UTP15	UTP15, U3 small nucleolar ribonucleoprotein, homolog (S. cerevisiae)	chr5	protein_coding
ENSG00000160789.18	-1.047836	10.5338397	0.00025874	0.00095857	397.25	371.67	251.94	243.49	LMNA	lamin A/C	chr1	protein_coding
ENSG00000279066.1	2.17504443	-0.97521	0.00011282	0.0004486	0.01	0.13	0.39	0.45	HEXDC-IT1	HEXDC intronic transcript 1	chr17	sense_intronic
ENSG00000250697.1	-5.7776308	-2.7095582	0.00047761	0.00167191	0.43	0.09	0	0	CTD-2066L2	--	chr5	lincRNA
ENSG00000170471.13	1.2267484	4.20383457	3.28E-05	0.00014476	0.95	1.1	2.99	3.41	RALGAPB	Ral GTPase activating protein, beta subunit (non-catalytic)	chr20	protein_coding
ENSG00000008294.19	2.043446	3.8091974	2.38E-11	2.55E-10	0.38	0.63	2.15	3.42	SPAG9	sperm associated antigen 9	chr17	protein_coding
ENSG00000178078.10	3.34789831	-2.4653852	0.00190233	0.00584723	0.02	0	0.25	0.05	STAP2	signal transducing adaptor family member 2	chr19	protein_coding
ENSG00000130768.13	3.28231233	0.16307913	2.67E-12	3.20E-11	0.06	0.07	0.7	1.05	SMPDL3B	sphingomyelin phosphodiesterase, acid-like 3B	chr1	protein_coding
ENSG00000105202.6	-1.2332363	9.27670036	1.86E-05	8.58E-05	596.75	608.85	343.34	339.69	FBL	fibrillarin	chr19	protein_coding
ENSG00000278963.1	5.68288014	-2.6056968	0.00047761	0.00167191	0	0	0.09	0.1	RP11-294J22	--	chr17	TEC
ENSG00000275888.1	1.20613265	-0.8514204	0.01533929	0.03782283	0.26	0.5	0.87	1.43	RP13-516M1	--	chr17	antisense
ENSG00000259884.1	4.72703895	-3.141721	0.01889873	0.04519557	0	0	0.28	0.04	RP11-1100L3	--	chr12	lincRNA
ENSG00000114120.10	2.69967593	1.58018935	6.51E-14	9.26E-13	0.08	0.08	0.46	0.96	SLC25A36	solute carrier family 25 (pyrimidine nucleotide carrier), member 36	chr3	protein_coding
ENSG00000187147.16	-1.1520098	7.19384371	6.39E-05	0.00026797	54.33	50.69	32.27	30.67	RNF220	ring finger protein 220	chr1	protein_coding
ENSG00000114439.17	3.16520931	2.93833084	5.68E-21	1.47E-19	0.04	0.22	1.07	2	BBX	bobby sox homolog (Drosophila)	chr3	protein_coding
ENSG00000141448.7	-4.5516945	2.07686977	7.48E-31	3.58E-29	1.49	1.84	0.11	0.08	GATA6	GATA binding protein 6	chr18	protein_coding
ENSG00000179403.11	-4.0530951	-1.6931074	1.57E-07	1.02E-06	0.11	0.06	0	0.01	VWA1	von Willebrand factor A domain containing 1	chr1	protein_coding
ENSG00000185650.9	-1.3948842	5.7725406	1.65E-06	9.21E-06	11.94	13.99	6.25	6.89	ZFP36L1	ZFP36 ring finger protein-like 1	chr14	protein_coding
ENSG00000184730.9	3.31375064	1.40026367	9.98E-18	2.01E-16	0.1	0.1	1.27	1.35	APOBR	apolipoprotein B receptor	chr16	protein_coding
ENSG00000218336.6	1.09265577	1.6020894	0.00088556	0.0029427	0.07	0.21	0.29	0.51	TENM3	teneurin transmembrane protein 3	chr4	protein_coding
ENSG00000100522.7	1.30430567	3.17195414	1.73E-05	8.02E-05	1.21	0.95	3.21	3.9	GNPNAT1	glucosamine-phosphate N-acetyltransferase 1	chr14	protein_coding
ENSG00000242028.4	-1.3363779	7.84695926	3.79E-06	1.99E-05	40.92	48.9	23.68	23.66	HYPK	huntingtin interacting protein K	chr15	protein_coding
ENSG00000167613.14	2.99348999	-2.0763513	0.00041794	0.0014836	0.01	0	0.04	0.08	LAIR1	leukocyte-associated immunoglobulin-like receptor 1	chr19	protein_coding
ENSG00000141384.10	2.0272804	0.60961045	2.18E-07	1.39E-06	0.1	0.08	0.21	0.73	TAF4B	TAF4b RNA polymerase II, TATA box binding protein (TBP)-associated factor, 105kDa	chr18	protein_coding
ENSG00000149084.10	3.48682252	3.4440216	2.44E-25	8.64E-24	0.34	0.59	3.74	10.22	HSD17B12	hydroxysteroid (17-beta) dehydrogenase 12	chr11	protein_coding
ENSG00000232527.6	-1.5073071	0.87466208	1.88E-05	8.68E-05	4.04	3.82	1.69	1.98	RP11-14N7.2	--	chr1	lincRNA
ENSG00000139163.14	1.47773105	1.50181046	1.25E-05	5.94E-05	0.15	0.16	0.4	0.76	ETNK1	ethanolamine kinase 1	chr12	protein_coding
ENSG00000107815.6	-1.4200518	6.62253388	1.01E-06	5.79E-06	36.26	36.89	17.78	18.66	C10orf2	chromosome 10 open reading frame 2	chr10	protein_coding
ENSG00000080819.5	1.5202901	3.73832128	4.21E-07	2.58E-06	2.31	1.61	5.68	9.41	CPOX	coproporphyrinogen oxidase	chr3	protein_coding
ENSG00000145390.10	2.53640093	-0.0529649	1.82E-08	1.34E-07	0.06	0	0.19	0.29	USP53	ubiquitin specific peptidase 53	chr4	protein_coding
ENSG00000197372.8	2.92391909	0.36262806	2.88E-11	3.07E-10	0.03	0.08	0.3	0.79	ZNF675	zinc finger protein 675	chr19	protein_coding
ENSG00000131409.11	7.32783747	-1.3821107	4.49E-10	4.11E-09	0	0	0.24	0.18	LRRC4B	leucine rich repeat containing 4B	chr19	protein_coding
ENSG00000115602.15	-6.0956928	-2.503296	8.36E-05	0.00034052	0.05	0.03	0	0	IL1RL1	interleukin 1 receptor-like 1	chr2	protein_coding
ENSG00000197457.8	-3.5510023	5.94066524	4.49E-29	1.99E-27	38.77	35.97	4	4.51	STMN3	stathmin-like 3	chr20	protein_coding
ENSG00000175575.11	-1.5630853	4.86362975	1.08E-07	7.22E-07	6.29	5.85	2.58	2.91	PAAF1	proteasomal ATPase-associated factor 1	chr11	protein_coding
ENSG00000164168.6	1.37662429	1.17819238	6.94E-05	0.00028852	0.2	0.25	0.76	0.8	TMEM184C	transmembrane protein 184C	chr4	protein_coding
ENSG00000175445.13	-12.08065	3.02934436	1.09E-61	2.96E-59	3.29	3.6	0	0	LPL	lipoprotein lipase	chr8	protein_coding
ENSG00000079332.13	1.17747938	5.96417553	4.76E-05	0.00020407	4.54	5.23	13.35	16.13	SAR1A	secretion associated, Ras related GTPase 1A	chr10	protein_coding
ENSG00000171928.12	1.45437963	2.19857061	5.46E-06	2.78E-05	0.44	0.62	1.69	2.19	TVP23B	trans-golgi network vesicle protein 23 homolog B (S. cerevisiae)	chr17	protein_coding
ENSG00000156384.13	2.03517504	-0.7212399	7.18E-05	0.00029725	0.12	0.09	0.46	0.73	SFR1	SWI5-dependent recombination repair 1	chr10	protein_coding
ENSG00000253187.2	1.96400594	2.69328251	5.70E-10	5.14E-09	1.42	1.11	7.57	5.61	HOXA10-AS	HOXA10 antisense RNA	chr7	antisense
ENSG00000120519.13	1.0765487	-0.495909	0.0170188	0.0415326	0.06	0.04	0.12	0.17	SLC10A7	solute carrier family 10, member 7	chr4	protein_coding
ENSG00000197142.9	4.58984911	5.45687342	6.03E-42	5.02E-40	0.67	0.78	22.18	24.59	ACSL5	acyl-CoA synthetase long-chain family member 5	chr10	protein_coding
ENSG00000072364.11	2.02363605	2.94836896	1.21E-10	1.19E-09	0.18	0.31	1.26	1.41	AFF4	AF4/FMR2 family, member 4	chr5	protein_coding
ENSG00000134986.12	-1.6190332	3.29065451	1.01E-07	6.80E-07	2.42	2.42	1.03	1.07	NREP	neuronal regeneration related protein	chr5	protein_coding
ENSG00000175344.15	2.85244042	-1.1819637	7.05E-06	3.52E-05	0.02	0	0.11	0.13	CHRNA7	cholinergic receptor, nicotinic, alpha 7 (neuronal)	chr15	protein_coding
ENSG00000185442.11	4.66876239	1.72858219	4.92E-28	2.05E-26	0.03	0.09	2.44	1.66	FAM174B	family with sequence similarity 174, member B	chr15	protein_coding
ENSG00000255120.4	8.89264924	-0.0023793	2.39E-20	5.90E-19	0	0	2.95	2.7	OVOL1-AS1	OVOL1 antisense RNA 1	chr11	antisense
ENSG00000173698.16	-3.9731506	2.70447946	3.48E-29	1.55E-27	1.72	2.39	0.07	0.27	GPR64	--	chrX	protein_coding
ENSG00000137760.13	2.18122053	0.40390869	8.45E-08	5.72E-07	0.11	0.05	0.26	0.7	ALKBH8	alkB, alkylation repair homolog 8 (E. coli)	chr11	protein_coding
ENSG00000109511.9	-5.022479	-3.1298504	0.0100455	0.02582742	0.06	0.08	0	0	ANXA10	annexin A10	chr4	protein_coding
ENSG00000198108.3	-5.7117674	-0.2387742	1.08E-18	2.35E-17	0.41	0.27	0.02	0	CHSY3	chondroitin sulfate synthase 3	chr5	protein_coding
ENSG00000113719.14	-2.5579636	8.75223407	1.79E-17	3.53E-16	88.96	86.02	19.71	19.89	ERGIC1	endoplasmic reticulum-golgi intermediate compartment (ERGIC) 1	chr5	protein_coding
ENSG00000269556.6	-1.0779888	4.53912738	0.00022197	0.00083259	7.91	8.46	5.16	5.17	TMEM185A	transmembrane protein 185A	chrX	protein_coding
ENSG00000058668.13	1.4295282	5.83199621	9.37E-07	5.42E-06	2.59	2.87	9.16	10.44	ATP2B4	ATPase, Ca++ transporting, plasma membrane 4	chr1	protein_coding
ENSG00000163286.6	3.87337944	-0.6256678	1.54E-10	1.51E-09	0.01	0.03	0.45	0.46	ALPPL2	alkaline phosphatase, placental-like 2	chr2	protein_coding
ENSG00000265984.1	-4.806998	-3.2310942	0.01889873	0.04519557	0.19	0.2	0	0	RP11-699A5	--	chr18	lincRNA
ENSG00000231133.5	8.50937496	-0.3528827	6.57E-17	1.23E-15	0	0	1.82	1.49	HAR1B	highly accelerated region 1B (non-protein coding)	chr20	lincRNA
ENSG00000264112.1	2.14278237	0.08913705	5.07E-07	3.06E-06	0.03	0.09	0.4	0.33	RP11-159D1	--	chr17	lincRNA
ENSG00000254726.2	-1.757133	3.85242601	5.67E-09	4.51E-08	2.66	3.24	1.13	1.19	MEX3A	mex-3 RNA binding family member A	chr1	protein_coding
ENSG00000166130.13	1.70305229	2.60192066	6.84E-08	4.70E-07	0.58	0.5	1.37	3.4	IKBIP	IKBKB interacting protein	chr12	protein_coding
ENSG00000150995.16	1.40175622	3.52904146	3.18E-06	1.69E-05	0.52	0.48	1.55	1.96	ITPR1	inositol 1,4,5-trisphosphate receptor, type 1	chr3	protein_coding
ENSG00000232874.1	4.72703895	-3.141721	0.01889873	0.04519557	0	0	0.45	0.06	RP11-135A1	--	chr3	antisense
ENSG00000077935.15	5.0421559	1.6210453	6.64E-29	2.90E-27	0.03	0.04	1.02	1.9	SMC1B	structural maintenance of chromosomes 1B	chr22	protein_coding
ENSG00000275038.1	-8.1490555	-0.8335495	5.76E-15	9.17E-14	0.37	0.26	0	0	RP11-546B8.	--	chr5	protein_coding
ENSG00000186017.13	2.93316863	-0.3869936	1.42E-08	1.07E-07	0.01	0.03	0.18	0.28	ZNF566	zinc finger protein 566	chr19	protein_coding
ENSG00000233013.7	3.13606823	-2.5941487	0.00552196	0.01522223	0.01	0	0.16	0.06	FAM157B	family with sequence similarity 157, member B	chr9	lincRNA
ENSG00000225555.1	5.89090986	-2.4715586	0.00014842	0.00057423	0	0	0.75	0.29	AP000320.6	--	chr21	antisense
ENSG00000099377.12	-1.3079534	3.43309996	1.28E-05	6.08E-05	6.16	4.31	2.84	2.8	HSD3B7	hydroxy-delta-5-steroid dehydrogenase, 3 beta- and steroid delta-isomerase 7	chr16	protein_coding
ENSG00000188986.4	-1.2264672	7.94981947	2.10E-05	9.60E-05	110.37	97.4	59.77	58.57	NELFB	negative elongation factor complex member B	chr9	protein_coding
ENSG00000100156.9	9.41830938	0.48492801	1.31E-25	4.72E-24	0	0	1.6	1.36	SLC16A8	solute carrier family 16 (monocarboxylate transporter), member 8	chr22	protein_coding
ENSG00000106927.10	-6.709645	-2.0605469	6.25E-07	3.72E-06	0.15	0.3	0	0	AMBP	alpha-1-microglobulin/bikunin precursor	chr9	protein_coding
ENSG00000268307.1	4.91020659	-3.0511096	0.0100455	0.02582742	0	0	0.42	0.12	CTD-2619J1	--	chr19	lincRNA
ENSG00000183508.4	2.52786439	-1.9846967	0.00270895	0.00811738	0.01	0	0.05	0.07	FAM46C	family with sequence similarity 46, member C	chr1	protein_coding
ENSG00000127324.7	3.14094422	-1.2223373	2.24E-06	1.22E-05	0.03	0.03	0.41	0.35	TSPAN8	tetraspanin 8	chr12	protein_coding
ENSG00000271503.4	-1.654018	-0.1723314	7.96E-05	0.00032796	0.75	0.71	0.29	0.32	CCL5	chemokine (C-C motif) ligand 5	chr17	protein_coding
ENSG00000169884.12	-2.1900302	2.41442453	1.25E-11	1.39E-10	3.38	2.83	0.99	0.83	WNT10B	wingless-type MMTV integration site family, member 10B	chr12	protein_coding
ENSG00000160471.11	-3.2184607	-1.3102446	6.12E-07	3.65E-06	0.23	0.14	0.01	0.04	COX6B2	cytochrome c oxidase subunit VIb polypeptide 2 (testis)	chr19	protein_coding
ENSG00000135720.11	2.67595605	5.34931222	2.64E-18	5.57E-17	1.68	1.99	12.44	19.03	DYNC1LI2	dynein, cytoplasmic 1, light intermediate chain 2	chr16	protein_coding
ENSG00000231453.1	6.80103599	-1.8032579	1.35E-07	8.87E-07	0	0	0.1	0.18	LINC01305	long intergenic non-protein coding RNA 1305	chr2	lincRNA
ENSG00000106305.8	-1.5104778	6.86320579	2.08E-07	1.33E-06	103.76	98.98	47.93	46.86	AIMP2	aminoacyl tRNA synthetase complex-interacting multifunctional protein 2	chr7	protein_coding
ENSG00000067177.13	-1.4927461	4.51621818	4.27E-07	2.61E-06	4.39	4.21	1.83	2.26	PHKA1	phosphorylase kinase, alpha 1 (muscle)	chrX	protein_coding
ENSG00000109846.6	1.6785404	1.5293858	7.55E-07	4.43E-06	0.39	0.48	1.79	1.91	CRYAB	crystallin, alpha B	chr11	protein_coding
ENSG00000127337.5	1.29741585	1.32421659	0.00013969	0.00054609	0.76	0.81	1.41	3.78	YEATS4	YEATS domain containing 4	chr12	protein_coding
ENSG00000268186.1	3.88073794	-2.1089698	8.40E-05	0.0003415	0.06	0	0.95	0.56	CTC-241F20.	--	chr19	lincRNA
ENSG00000122859.4	2.88069329	-2.7403887	0.01615662	0.03958166	0.02	0	0.09	0.14	NEUROG3	neurogenin 3	chr10	protein_coding
ENSG00000060982.13	-7.2402706	1.74928388	5.84E-42	4.88E-40	0.42	0.65	0	0.01	BCAT1	branched chain amino-acid transaminase 1, cytosolic	chr12	protein_coding
ENSG00000162076.11	-1.4977294	6.27864564	2.73E-07	1.71E-06	79.11	80.8	39.32	36.03	FLYWCH2	FLYWCH family member 2	chr16	protein_coding
ENSG00000273344.1	1.03848829	2.8330865	0.00065101	0.00222909	1.6	1.71	4.09	4.99	PAXIP1-AS1	PAXIP1 antisense RNA 1 (head to head)	chr7	lincRNA
ENSG00000183779.6	-1.5413822	5.4394313	1.42E-07	9.32E-07	16.26	14.94	8.06	6.18	ZNF703	zinc finger protein 703	chr8	protein_coding
ENSG00000145833.14	1.07222869	5.46313428	0.00021495	0.00080802	3.43	4.63	8.98	13.66	DDX46	DEAD (Asp-Glu-Ala-Asp) box polypeptide 46	chr5	protein_coding
ENSG00000123992.17	-1.0832979	4.97551951	0.00019328	0.0007315	13.3	12.72	7.82	8.56	DNPEP	aspartyl aminopeptidase	chr2	protein_coding
ENSG00000169242.10	1.66680273	5.82474752	1.34E-08	1.01E-07	11.16	11.62	49.33	47	EFNA1	ephrin-A1	chr1	protein_coding
ENSG00000188153.11	-1.0670236	5.90461556	0.00021926	0.00082295	9.91	10.33	5.51	7.41	COL4A5	collagen, type IV, alpha 5	chrX	protein_coding
ENSG00000167110.15	-1.5980763	8.63627454	3.94E-08	2.77E-07	105.12	111.64	45.18	50.31	GOLGA2	golgin A2	chr9	protein_coding
ENSG00000117155.15	1.93054441	0.76316595	2.68E-07	1.69E-06	0.08	0.08	0.23	0.58	SSX2IP	synovial sarcoma, X breakpoint 2 interacting protein	chr1	protein_coding
ENSG00000096384.18	-1.3370273	10.7298397	3.62E-06	1.91E-05	618.31	704.76	331.2	367.03	HSP90AB1	heat shock protein 90kDa alpha (cytosolic), class B member 1	chr6	protein_coding
ENSG00000100206.8	1.08381243	0.31182988	0.00467704	0.01322614	0.26	0.09	0.51	0.49	DMC1	DNA meiotic recombinase 1	chr22	protein_coding
ENSG00000179820.14	-7.3671839	7.55984246	1.16E-82	8.69E-80	84.98	90.38	0.78	0.64	MYADM	myeloid-associated differentiation marker	chr19	protein_coding
ENSG00000109927.8	2.43024563	-2.436014	0.01191236	0.03011315	0.01	0	0	0.06	TECTA	tectorin alpha	chr11	protein_coding
ENSG00000227189.2	3.02552652	-2.6589186	0.00943894	0.02469931	0.01	0	0.13	0.04	AC092535.3	--	chr4	antisense
ENSG00000263276.1	7.14351214	-1.5247038	4.48E-09	3.59E-08	0	0	0.07	0.3	RP11-96D1.1	--	chr16	sense_overlapping
ENSG00000236438.5	6.96511009	-1.6792724	1.87E-08	1.38E-07	0	0	0.56	0.33	FAM157A	family with sequence similarity 157, member A	chr3	lincRNA
ENSG00000198399.13	1.88474593	1.80922089	1.83E-08	1.35E-07	0.14	0.2	0.65	1.02	ITSN2	intersectin 2	chr2	protein_coding
ENSG00000260612.1	2.36818918	-2.0920738	0.0061421	0.01678695	0.05	0.03	0.53	0.08	RP11-432I5.4	--	chr16	lincRNA
ENSG00000279407.1	-1.3612764	4.29888829	4.00E-06	2.09E-05	7.06	8.25	3.84	4.1	AC007191.4	--	chr19	TEC
ENSG00000261770.1	2.22925428	0.32461774	4.73E-08	3.31E-07	0.17	0.01	0.54	0.59	CTC-459F4.1	--	chr19	lincRNA
ENSG00000272325.1	-1.7593783	6.15018269	2.08E-09	1.74E-08	8.75	9.18	3.55	3.5	NUDT3	nudix (nucleoside diphosphate linked moiety X)-type motif 3	chr6	protein_coding
ENSG00000178982.8	-1.2704659	8.7297799	1.06E-05	5.10E-05	340.35	305.11	184.11	172.22	EIF3K	eukaryotic translation initiation factor 3, subunit K	chr19	protein_coding
ENSG00000224032.5	-2.5895116	5.49524004	1.85E-17	3.63E-16	23.33	18.81	4.56	4.78	EPB41L4A-A	EPB41L4A antisense RNA 1	chr5	lincRNA
ENSG00000109805.8	2.98736643	2.96359414	2.17E-19	4.97E-18	0.3	0.29	2.36	3.99	NCAPG	non-SMC condensin I complex, subunit G	chr4	protein_coding
ENSG00000157388.12	5.8658494	-0.4970902	2.56E-14	3.81E-13	0	0	0.1	0.17	CACNA1D	calcium channel, voltage-dependent, L type, alpha 1D subunit	chr3	protein_coding
ENSG00000134363.10	-2.776076	2.97769173	1.21E-17	2.42E-16	4.08	3.91	0.77	0.78	FST	follistatin	chr5	protein_coding
ENSG00000231305.3	1.15782605	0.06423597	0.0045882	0.01299176	0.15	0.53	1.01	0.97	RP11-723O4	--	chr3	antisense
ENSG00000172340.13	1.03695803	2.56894531	0.00078212	0.00263515	0.91	1.1	2.15	3.37	SUCLG2	succinate-CoA ligase, GDP-forming, beta subunit	chr3	protein_coding
ENSG00000188130.12	-1.7640178	6.20101562	1.88E-09	1.58E-08	44.65	39.44	15.95	17.08	MAPK12	mitogen-activated protein kinase 12	chr22	protein_coding
ENSG00000256185.1	2.16979333	-2.2135654	0.02071392	0.04894569	0.31	0.16	0.93	2.06	RP11-612B6.	--	chr12	3prime_overlapping_ncrna
ENSG00000168743.11	-3.0714615	1.09713608	9.26E-16	1.58E-14	0.67	0.63	0.1	0.1	NPNT	nephronectin	chr4	protein_coding
ENSG00000132517.13	9.54700507	0.60716578	5.22E-27	2.08E-25	0	0	0.82	0.82	SLC52A1	solute carrier family 52 (riboflavin transporter), member 1	chr17	protein_coding
ENSG00000185522.7	2.97192837	1.45604326	1.58E-15	2.65E-14	0.31	0.15	2.66	2.18	LMNTD2	lamin tail domain containing 2	chr11	protein_coding
ENSG00000137965.9	-2.1869934	-0.7458846	5.94E-06	3.01E-05	0.21	0.69	0	0.24	IFI44	interferon-induced protein 44	chr1	protein_coding
ENSG00000184709.7	-1.6936747	1.76181711	3.14E-07	1.96E-06	3.21	3.68	1.96	0.88	LRRC26	leucine rich repeat containing 26	chr9	protein_coding
ENSG00000267699.2	1.53190077	0.39585772	8.59E-05	0.0003489	0.37	0.46	0.99	2.22	RP11-729L2.	--	chr18	protein_coding
ENSG00000139438.5	1.44964014	2.23628385	5.19E-06	2.66E-05	0.44	0.74	2.03	2.23	FAM222A	family with sequence similarity 222, member A	chr12	protein_coding
ENSG00000244468.1	6.16625843	-0.2333659	8.15E-17	1.52E-15	0.05	0	2.96	2.87	RP11-206M1	--	chr3	antisense
ENSG00000114654.7	6.3350923	-2.1600298	8.95E-06	4.38E-05	0	0	0.14	0.1	EFCC1	EF-hand and coiled-coil domain containing 1	chr3	protein_coding
ENSG00000269399.2	-1.0785582	1.94281714	0.00072332	0.00245358	1.53	2.53	1.39	1.16	CTD-3222D1	--	chr19	lincRNA
ENSG00000185352.8	-2.1031013	-0.4868083	3.03E-06	1.62E-05	0.12	0.11	0.01	0.06	HS6ST3	heparan sulfate 6-O-sulfotransferase 3	chr13	protein_coding
ENSG00000115363.12	2.997854	1.12329317	1.41E-14	2.16E-13	0.2	0.11	1.56	1.81	EVA1A	eva-1 homolog A (C. elegans)	chr2	protein_coding
ENSG00000179630.9	3.45993741	-0.6923601	3.09E-09	2.54E-08	0.01	0.02	0.15	0.31	LACC1	laccase (multicopper oxidoreductase) domain containing 1	chr13	protein_coding
ENSG00000065717.13	1.17387533	4.60474712	6.02E-05	0.00025314	4.38	3.84	12	12.77	TLE2	transducin-like enhancer of split 2	chr19	protein_coding
ENSG00000254165.1	5.66409976	-2.616209	0.00086564	0.00288128	0	0	0.09	0.46	RP11-503E24	--	chr8	antisense
ENSG00000099998.16	-8.4120263	1.56936094	3.11E-41	2.53E-39	2.26	1.58	0.01	0	GGT5	gamma-glutamyltransferase 5	chr22	protein_coding
ENSG00000072864.11	-1.5032068	5.54209248	2.72E-07	1.71E-06	13.95	13.81	6.9	6.13	NDE1	nudE neurodevelopment protein 1	chr16	protein_coding
ENSG00000137310.10	-1.0857375	6.31409786	0.00016685	0.00063822	25.18	28.07	16.23	17.2	TCF19	transcription factor 19	chr6	protein_coding
ENSG00000261513.1	2.33887212	-2.4903051	0.01923175	0.0458365	0.07	0	0.04	0.46	RP11-432I5.8	--	chr16	antisense
ENSG00000199377.1	-3.0407039	-1.6941526	1.25E-05	5.96E-05	3.66	2.91	0.55	0.47	RNU5F-1	RNA, U5F small nuclear 1	chr1	snRNA
ENSG00000272792.1	2.22563902	-1.8813423	0.00445943	0.01266454	0.23	0	0.14	1.33	RP11-141C7	--	chr3	lincRNA
ENSG00000108405.3	-10.151538	1.10066922	3.09E-35	1.83E-33	1.08	1.5	0	0	P2RX1	purinergic receptor P2X, ligand gated ion channel, 1	chr17	protein_coding
ENSG00000261327.4	4.45753975	-0.9604318	9.89E-10	8.68E-09	0.02	0.02	1	0.37	RP11-863P13	--	chr16	lincRNA
ENSG00000111879.17	3.39791962	-1.3457728	2.86E-06	1.54E-05	0	0.02	0.06	0.15	FAM184A	family with sequence similarity 184, member A	chr6	protein_coding
ENSG00000161298.15	4.72703895	-3.141721	0.01889873	0.04519557	0	0	0.02	0	ZNF382	zinc finger protein 382	chr19	protein_coding
ENSG00000055130.14	1.29862963	4.52877679	1.01E-05	4.90E-05	2.24	3.84	7.74	12.25	CUL1	cullin 1	chr7	protein_coding
ENSG00000134716.8	6.3839906	-2.1220471	5.19E-06	2.66E-05	0	0	0.15	0.2	CYP2J2	cytochrome P450, family 2, subfamily J, polypeptide 2	chr1	protein_coding
ENSG00000112759.15	-3.3562504	6.06930683	1.19E-26	4.60E-25	32.58	32.75	4.18	4.32	SLC29A1	solute carrier family 29 (equilibrative nucleoside transporter), member 1	chr6	protein_coding
ENSG00000231887.5	-1.1715725	0.31125373	0.00226413	0.00687906	1.57	1.2	0.83	0.81	PRH1	proline-rich protein HaeIII subfamily 1	chr12	protein_coding
ENSG00000233895.1	-2.9593356	-2.0700233	0.00016274	0.00062431	0.12	0.71	0	0.12	RP1-122P22.	--	chr20	lincRNA
ENSG00000111667.12	-1.2482357	7.760098	1.52E-05	7.14E-05	82.06	73.15	43.82	43.26	USP5	ubiquitin specific peptidase 5 (isopeptidase T)	chr12	protein_coding
ENSG00000234684.5	1.24022836	-0.9881905	0.01735348	0.04227188	0.04	0.06	0.13	0.19	SDCBP2-AS1	SDCBP2 antisense RNA 1	chr20	antisense
ENSG00000224975.1	1.10015792	1.43933398	0.00093287	0.00308706	1.05	1.83	4.61	3.57	INE1	inactivation escape 1 (non-protein coding)	chrX	sense_intronic
ENSG00000173660.10	-1.0475282	7.69882743	0.00026621	0.00097757	397.99	352.23	249.05	234.4	UQCRH	ubiquinol-cytochrome c reductase hinge protein	chr1	protein_coding
ENSG00000181555.18	1.61953425	3.6157648	8.31E-08	5.63E-07	0.58	0.6	1.83	3.06	SETD2	SET domain containing 2	chr3	protein_coding
ENSG00000180479.12	3.78840225	-1.0380859	3.09E-08	2.20E-07	0.01	0	0.1	0.16	ZNF571	zinc finger protein 571	chr19	protein_coding
ENSG00000226510.1	7.52072171	-1.2221611	3.23E-11	3.41E-10	0	0	1.17	0.72	UPK1A-AS1	UPK1A antisense RNA 1	chr19	antisense
ENSG00000034693.13	1.17209601	2.02248682	0.00024234	0.0009039	1.17	0.83	2.46	3.6	PEX3	peroxisomal biogenesis factor 3	chr6	protein_coding
ENSG00000007264.12	-1.837965	3.24364621	2.03E-09	1.71E-08	5.08	4.3	1.56	1.94	MATK	megakaryocyte-associated tyrosine kinase	chr19	protein_coding
ENSG00000214510.8	10.2427615	1.27367211	4.08E-35	2.40E-33	0	0	2.4	6.05	SPINK13	serine peptidase inhibitor, Kazal type 13 (putative)	chr5	protein_coding
ENSG00000122299.10	1.29040414	3.78048284	1.49E-05	7.03E-05	1.08	1.34	2.66	5.29	ZC3H7A	zinc finger CCCH-type containing 7A	chr16	protein_coding
ENSG00000239911.2	-1.3411191	3.0249648	1.04E-05	5.03E-05	7.73	8.48	4.32	4.19	PRKAG2-AS1	PRKAG2 antisense RNA 1	chr7	antisense

ENSG00000173875.12	1.49244557	1.95636007	4.36E-06	2.27E-05	0.3	0.2	0.69	1.18	ZNF791	zinc finger protein 791	chr19	protein_coding
ENSG00000204469.11	-1.0358516	8.85209578	0.00030591	0.00111145	73.78	72.02	51.04	43.51	PRRC2A	proline-rich coiled-coil 2A	chr6	protein_coding
ENSG00000143321.17	-1.1790575	11.1423728	4.13E-05	0.00017993	856.47	963.97	542.67	527.37	HDGF	hepatoma-derived growth factor	chr1	protein_coding
ENSG00000278206.1	-2.4915433	-2.7162016	0.00943894	0.02469931	0.08	0.05	0	0.03	RP1-20N2.8	--	chr6	processed_transcript
ENSG00000204428.11	1.63632161	-1.3568723	0.00496338	0.01395792	0.32	0.04	0.48	1.05	LY6G5C	lymphocyte antigen 6 complex, locus G5C	chr6	protein_coding
ENSG00000148429.13	1.18486761	1.60510111	0.00032351	0.00117129	0.1	0.12	0.31	0.36	USP6NL	USP6 N-terminal like	chr10	protein_coding
ENSG00000124635.8	3.10636999	2.2345109	8.26E-19	1.81E-17	0.73	0.89	10.66	8	HIST1H2BJ	histone cluster 1, H2bj	chr6	protein_coding
ENSG00000153044.8	-1.276729	3.15779379	2.25E-05	0.00010217	6.49	8.22	2.63	5.49	CENPH	centromere protein H	chr5	protein_coding
ENSG00000163093.10	1.42954728	0.80956599	7.91E-05	0.0003261	0.19	0.23	0.88	0.63	BBS5	Bardet-Biedl syndrome 5	chr2	protein_coding
ENSG00000187720.13	-2.773774	5.91398994	9.58E-20	2.25E-18	8.42	9.62	1.83	1.67	THSD4	thrombospondin, type I, domain containing 4	chr15	protein_coding
ENSG00000158417.9	1.20365044	5.58657384	3.38E-05	0.00014932	3.24	4.91	9.15	15.98	EIF5B	eukaryotic translation initiation factor 5B	chr2	protein_coding
ENSG00000148516.20	1.62279475	0.63862803	1.93E-05	8.89E-05	0.06	0.12	0.15	0.59	ZEB1	zinc finger E-box binding homeobox 1	chr10	protein_coding
ENSG00000168564.5	3.10574023	1.77056124	1.84E-17	3.62E-16	0.1	0.26	1.47	2.69	CDKN2AIP	CDKN2A interacting protein	chr4	protein_coding
ENSG00000168874.11	2.06187145	5.04682698	6.05E-12	6.97E-11	4.21	4.24	24.74	22.24	ATOH8	atonal homolog 8 (Drosophila)	chr2	protein_coding
ENSG00000256128.4	-6.3510329	-2.3247802	1.55E-05	7.26E-05	0.51	0.03	0	0	LINC00944	long intergenic non-protein coding RNA 944	chr12	lincRNA
ENSG00000145014.16	-1.8484978	4.79440356	5.39E-10	4.88E-09	9.72	11.45	3.6	4.24	TMEM44	transmembrane protein 44	chr3	protein_coding
ENSG00000251580.1	1.14419953	-0.5042789	0.01262798	0.03174808	0.57	0.94	2.13	2.32	RP11-539L1(	--	chr4	lincRNA
ENSG00000074266.16	1.25341754	4.75104777	1.85E-05	8.54E-05	3.11	3.97	10.38	12.14	EED	embryonic ectoderm development	chr11	protein_coding
ENSG00000273394.1	5.05186882	-2.9771646	0.0100455	0.02582742	0	0	0.11	0.34	RP11-255E6.	--	chr3	antisense
ENSG00000224729.5	-1.1791764	-0.4293097	0.00808017	0.02151958	0.32	0.26	0.2	0.14	PCOLCE-AS1	PCOLCE antisense RNA 1	chr7	antisense
ENSG00000281205.1	1.5655094	-1.2737091	0.00839289	0.02229301	0.02	0.03	0.06	0.1	LINC00950	long intergenic non-protein coding RNA 950	chr9	lincRNA
ENSG00000138696.9	-3.3697632	-1.7617933	1.91E-05	8.80E-05	0.04	0.09	0.02	0	BMPR1B	bone morphogenetic protein receptor, type IB	chr4	protein_coding
ENSG00000178381.10	-1.5264116	4.54919684	2.42E-07	1.53E-06	25.09	24.49	10.9	12.05	ZFAND2A	zinc finger, AN1-type domain 2A	chr7	protein_coding
ENSG00000116157.5	4.62358735	2.7455656	9.26E-34	5.09E-32	0.39	0.28	10.28	11.97	GPX7	glutathione peroxidase 7	chr1	protein_coding
ENSG00000164379.5	-1.2407001	5.20651334	2.02E-05	9.28E-05	24.86	24.09	14.12	13.47	FOXQ1	forkhead box Q1	chr6	protein_coding
ENSG00000164736.5	-7.6679699	0.82534368	6.99E-32	3.51E-30	1.44	0.99	0	0.01	SOX17	SRY (sex determining region Y)-box 17	chr8	protein_coding
ENSG00000239791.1	2.02812474	-1.3923622	0.00095912	0.00316084	0.1	0	0.35	0.21	AC002310.7	--	chr16	antisense
ENSG00000259546.1	2.23708631	-1.4200231	0.00043455	0.00153757	0	0.25	0.98	0.6	RP11-351M8	--	chr15	antisense
ENSG00000141446.9	2.98784906	0.95777642	8.12E-14	1.15E-12	0.08	0.06	0.54	0.97	ESCO1	establishment of sister chromatid cohesion N-acetyltransferase 1	chr18	protein_coding
ENSG00000248932.4	1.12326044	-0.1737619	0.00749897	0.02009224	0.21	0.17	0.76	0.39	RP11-319G6	--	chr3	antisense
ENSG00000176222.8	4.87917439	-3.0678699	0.01889873	0.04519557	0	0	0	0.12	ZNF404	zinc finger protein 404	chr19	protein_coding
ENSG00000120594.15	-1.5134293	2.80553961	9.27E-07	5.36E-06	0.59	0.75	0.34	0.29	PLXDC2	plexin domain containing 2	chr10	protein_coding
ENSG00000111254.6	1.936672	-2.0657851	0.02035181	0.04824221	0	0.03	0.08	0.1	AKAP3	A kinase (PRKA) anchor protein 3	chr12	protein_coding
ENSG00000174564.11	-1.1583548	5.0788028	6.82E-05	0.00028422	18.68	18.21	11.3	10.71	IL20RB	interleukin 20 receptor beta	chr3	protein_coding
ENSG00000251562.6	5.45057226	9.92082383	9.14E-57	1.92E-54	2.89	5.19	141.62	333.04	MALAT1	metastasis associated lung adenocarcinoma transcript 1 (non-protein coding)	chr11	lincRNA
ENSG00000113013.11	-1.2874146	9.12340519	8.07E-06	4.00E-05	176.47	209.67	91.92	119.39	HSPA9	heat shock 70kDa protein 9 (mortalin)	chr5	protein_coding
ENSG00000180992.6	-1.1465748	5.86219042	7.42E-05	0.00030684	73.57	61.55	43.29	37.97	MRPL14	mitochondrial ribosomal protein L14	chr6	protein_coding
ENSG00000228672.3	1.53700926	4.71355838	1.96E-07	1.26E-06	2.09	2.15	7.99	8.4	PROB1	proline-rich basic protein 1	chr5	protein_coding
ENSG00000273466.1	-1.4364891	2.37667379	4.63E-06	2.40E-05	7.89	8.21	3.59	4.34	RP11-548H3	--	chr2	antisense
ENSG00000128311.12	1.12647234	4.05589096	0.00013347	0.00052366	5.1	3.85	13.77	12.25	TST	thiosulfate sulfurtransferase (rhodanese)	chr22	protein_coding
ENSG00000204564.10	-1.0107484	3.23305422	0.00075187	0.00254395	4.41	5.86	3.5	3.27	C6orf136	chromosome 6 open reading frame 136	chr6	protein_coding
ENSG00000084636.16	-3.9132185	4.90298201	7.82E-33	4.14E-31	7.28	7.53	0.68	0.63	COL16A1	collagen, type XVI, alpha 1	chr1	protein_coding
ENSG00000173212.4	7.25373882	-1.4353982	1.11E-09	9.61E-09	0	0	0.1	0.28	MAB21L3	mab-21-like 3 (C. elegans)	chr1	protein_coding
ENSG00000253284.2	2.72656253	-2.2527193	0.00174589	0.00541921	0	0.01	0.06	0.04	RP11-282K2	--	chr12	sense_intronic
ENSG00000107859.8	-3.6218558	-1.2530837	2.89E-08	2.08E-07	0.63	0.18	0.02	0.06	PITX3	paired-like homeodomain 3	chr10	protein_coding
ENSG00000253508.1	1.64950469	-1.8108604	0.01883218	0.04519557	0	0.31	0.75	0.54	RP1-170O19	--	chr7	lincRNA
ENSG00000257178.3	5.04680446	-2.9799228	0.0100455	0.02582742	0	0	0.01	0.08	RP11-357H1	--	chr17	sense_intronic
ENSG00000091986.14	-1.5793438	4.33913626	1.03E-07	6.88E-07	1.85	2.09	0.83	0.92	CCDC80	coiled-coil domain containing 80	chr3	protein_coding
ENSG00000164904.14	-9.6644795	6.8802648	3.57E-104	8.56E-101	38.76	38.02	0.06	0.06	ALDH7A1	aldehyde dehydrogenase 7 family, member A1	chr5	protein_coding
ENSG00000279369.1	4.79658741	1.23823098	1.95E-25	6.94E-24	0.13	0	2.93	2.19	RP11-51F16.	--	chr17	TEC
ENSG00000204767.3	-7.5043988	-1.4111571	7.65E-11	7.77E-10	0.11	0.26	0	0	FAM196B	family with sequence similarity 196, member B	chr5	protein_coding
ENSG00000181744.7	1.81533026	0.80493429	1.26E-06	7.13E-06	0.16	0.07	0.27	0.86	C3orf58	chromosome 3 open reading frame 58	chr3	protein_coding
ENSG00000126522.15	-1.3438993	6.62271172	3.54E-06	1.87E-05	54.25	44.48	26.62	25.21	ASL	argininosuccinate lyase	chr7	protein_coding
ENSG00000215252.10	3.68341803	1.09564088	1.25E-18	2.71E-17	0.06	0.04	0.57	1.2	GOLGA8B	golgin A8 family, member B	chr15	protein_coding
ENSG00000186510.10	-2.0841266	-1.0889809	9.78E-05	0.00039441	0.23	0.21	0.02	0.11	CLCNKA	chloride channel, voltage-sensitive Ka	chr1	protein_coding
ENSG00000102043.14	-5.0243296	-3.1290017	0.0100455	0.02582742	0.01	0.06	0	0	MTMR8	myotubularin related protein 8	chrX	protein_coding
ENSG00000197977.3	-5.6539904	-2.7851509	0.00086564	0.00288128	0.07	0.01	0	0	ELOVL2	ELOVL fatty acid elongase 2	chr6	protein_coding
ENSG00000171867.15	1.32318503	4.89045188	6.19E-06	3.13E-05	4.22	6.09	14.46	19.99	PRNP	prion protein	chr20	protein_coding
ENSG00000134184.11	-12.436617	3.38591524	6.15E-67	2.14E-64	12.29	11.53	0	0	GSTM1	glutathione S-transferase mu 1	chr1	protein_coding
ENSG00000163516.12	-1.2599131	6.92098769	1.31E-05	6.20E-05	55.29	49.62	29.88	28.47	ANKZF1	ankyrin repeat and zinc finger domain containing 1	chr2	protein_coding
ENSG00000204315.3	-1.351627	3.93629875	5.35E-06	2.73E-05	13.66	12.77	7.29	6.5	FKBPL	FK506 binding protein like	chr6	protein_coding
ENSG00000124664.9	5.98373061	-2.4097568	8.36E-05	0.00034052	0	0	0.22	0.06	SPDEF	SAM pointed domain containing ETS transcription factor	chr6	protein_coding
ENSG00000010219.12	-1.8120017	2.53348436	1.02E-08	7.83E-08	2.21	1.9	0.94	0.62	DYRK4	dual-specificity tyrosine-(Y)-phosphorylation regulated kinase 4	chr12	protein_coding
ENSG00000134444.12	1.44873215	2.26391141	5.45E-06	2.77E-05	0.26	0.39	1.01	1.33	KIAA1468	KIAA1468	chr18	protein_coding
ENSG00000196792.10	2.54037103	1.28119421	5.88E-12	6.80E-11	0.15	0.11	0.83	1.21	STRN3	striatin, calmodulin binding protein 3	chr14	protein_coding
ENSG00000224914.2	1.23861914	1.62284875	0.00017979	0.00068444	0.5	0.37	1.55	1.21	LINC00863	long intergenic non-protein coding RNA 863	chr10	lincRNA
ENSG00000273485.1	-1.4094635	-0.8672359	0.00536284	0.01488851	0.81	0.99	0.49	0.41	RP11-225H2	--	chr10	antisense
ENSG00000164056.9	-2.8786839	0.02727556	1.61E-10	1.56E-09	0.5	0.44	0.13	0.05	SPRY1	sprouty homolog 1, antagonist of FGF signaling (Drosophila)	chr4	protein_coding
ENSG00000006576.15	2.5134672	1.36406024	4.54E-12	5.30E-11	0.06	0.11	0.55	0.78	PHTF2	putative homeodomain transcription factor 2	chr7	protein_coding
ENSG00000180596.7	3.09908944	1.04704283	7.76E-15	1.22E-13	0.48	0.31	5.34	3.75	HIST1H2BC	histone cluster 1, H2bc	chr6	protein_coding
ENSG00000269906.1	3.2438874	-1.4708759	1.38E-05	6.54E-05	0.12	0	0.72	0.94	RP11-248J18	--	chr14	sense_intronic
ENSG00000109089.7	-1.3580596	6.97684828	2.75E-06	1.48E-05	42.98	39.97	21.33	21.8	CDR2L	cerebellar degeneration-related protein 2-like	chr17	protein_coding
ENSG00000268001.1	2.16984184	-1.4642573	0.00086862	0.00288921	0	0.09	0.21	0.3	CARD8-AS1	CARD8 antisense RNA 1	chr19	antisense
ENSG00000072415.7	1.78739234	1.79325776	8.43E-08	5.70E-07	0.23	0.17	0.73	1.15	MPP5	membrane protein, palmitoylated 5 (MAGUK p55 subfamily member 5)	chr14	protein_coding
ENSG00000153914.14	2.85054818	3.23932603	1.66E-18	3.56E-17	0.17	0.24	1.27	2.72	SREK1	splicing regulatory glutamine/lysine-rich protein 1	chr5	protein_coding
ENSG00000135740.15	2.096014	2.61904321	6.15E-11	6.31E-10	0.62	0.37	2.69	2.97	SLC9A5	solute carrier family 9, subfamily A (NHE5, cation proton antiporter 5), member 5	chr16	protein_coding
ENSG00000112699.9	-2.0617989	5.97685331	3.72E-12	4.39E-11	45.52	45.28	15.04	13.9	GMDS	GDP-mannose 4,6-dehydratase	chr6	protein_coding
ENSG00000251151.2	-6.0843633	2.94100195	8.39E-47	9.76E-45	12.22	12.38	0.32	0.16	HOXC-AS3	HOXC cluster antisense RNA 3	chr12	processed_transcript
ENSG00000027869.10	-2.2681003	2.9566398	8.20E-13	1.04E-11	5.61	6.72	2.13	1.27	SH2D2A	SH2 domain containing 2A	chr1	protein_coding
ENSG00000167676.3	7.7882133	5.02174151	2.92E-74	1.49E-71	0.07	0	11.68	10.19	PLIN4	perilipin 4	chr19	protein_coding
ENSG00000136830.10	-1.0715743	10.143318	0.00018838	0.00071407	325.89	289.72	193.46	197.09	FAM129B	family with sequence similarity 129, member B	chr9	protein_coding
ENSG00000147206.15	6.03134248	-2.373212	8.36E-05	0.00034052	0	0	0.1	0.17	NXF3	nuclear RNA export factor 3	chrX	protein_coding
ENSG00000061676.13	2.37385154	3.11935041	8.07E-14	1.14E-12	0.11	0.11	0.49	1.01	NCKAP1	NCK-associated protein 1	chr2	protein_coding
ENSG00000257065.1	1.64056374	-0.6376855	0.00054065	0.0018794	0.21	0.37	1.18	1.25	RP3-468K18.	--	chr6	protein_coding
ENSG00000198695.2	1.78384694	8.94644777	1.07E-09	9.31E-09	248.76	437.89	1746.9	1384.56	MT-ND6	mitochondrially encoded NADH dehydrogenase 6	chrM	protein_coding
ENSG00000137752.21	1.79570176	3.41178774	4.50E-09	3.61E-08	1.36	1.5	6.28	6.97	CASP1	caspase 1, apoptosis-related cysteine peptidase	chr11	protein_coding
ENSG00000269481.1	4.8843127	-3.0650858	0.01889873	0.04519557	0	0	0.05	0.28	CTD-2521M	--	chr19	antisense
ENSG00000177663.12	1.17443805	6.51180167	4.79E-05	0.00020504	4.95	5.59	16.31	15.32	IL17RA	interleukin 17 receptor A	chr22	protein_coding
ENSG00000261609.3	2.21957894	1.84098657	7.75E-11	7.86E-10	0.05	0.08	0.43	0.38	GAN	gigaxonin	chr16	protein_coding
ENSG00000143033.16	1.91752501	2.26141216	3.52E-09	2.88E-08	0.29	0.48	1.58	2.32	MTF2	metal response element binding transcription factor 2	chr1	protein_coding
ENSG00000167232.12	-2.211525	-1.3493446	0.00010901	0.00043503	0.1	0.06	0.01	0.03	ZNF91	zinc finger protein 91	chr19	protein_coding
ENSG00000113966.8	1.39707921	-1.2718956	0.01764505	0.04292107	0.13	0.05	0.23	0.4	ARL6	ADP-ribosylation factor-like 6	chr3	protein_coding
ENSG00000172432.17	-1.8591616	6.253086	2.73E-10	2.57E-09	33.25	30.91	12.42	11.12	GTPBP2	GTP binding protein 2	chr6	protein_coding
ENSG00000221988.11	-1.9753427	4.88568274	3.85E-11	4.03E-10	17.71	17.5	5.65	6.3	PPT2	palmitoyl-protein thioesterase 2	chr6	protein_coding
ENSG00000187838.15	-1.4274658	3.99779987	1.60E-06	8.91E-06	9.73	6.76	4.01	4.18	TMEM256-P	TMEM256-PLSCR3 readthrough (NMD candidate)	chr17	protein_coding
ENSG00000198018.6	1.03123305	2.28327105	0.00099927	0.00328453	0.76	0.67	1.82	2.09	ENTPD7	ectonucleoside triphosphate diphosphohydrolase 7	chr10	protein_coding
ENSG00000146242.7	-1.7963187	6.63652997	9.31E-10	8.18E-09	16.52	19.74	6.24	7.7	TPBG	trophoblast glycoprotein	chr6	protein_coding
ENSG00000149823.6	-2.5602159	7.2104914	1.92E-17	3.77E-16	83.87	67.07	17.5	16.61	VPS51	vacuolar protein sorting 51 homolog (S. cerevisiae)	chr11	protein_coding
ENSG00000128833.11	3.65410532	0.35488704	1.08E-14	1.68E-13	0.03	0.01	0.28	0.39	MYO5C	myosin VC	chr15	protein_coding
ENSG00000156140.7	-5.0252555	-3.1285772	0.0100455	0.02582742	0	0.03	0	0	ADAMTS3	ADAM metallopeptidase with thrombospondin type 1 motif, 3	chr4	protein_coding
ENSG00000088970.14	1.9725504	1.64156896	8.76E-09	6.78E-08	0.43	0.48	2.3	2.42	KIZ	kizuna centrosomal protein	chr20	protein_coding
ENSG00000174446.11	-1.0398656	3.30592013	0.00050115	0.00174923	5.05	7.34	3.39	4.64	SNAPC5	small nuclear RNA activating complex, polypeptide 5, 19kDa	chr15	protein_coding
ENSG00000278267.1	2.08245038	-2.2675181	0.01324986	0.0331641	1.18	0	1.41	5.61	MIR6859-3	microRNA 6859-3	chr1	miRNA
ENSG00000222041.9	-1.9672532	1.83753311	4.25E-09	3.45E-08	4.39	3.96	1.96	0.89	LINC00152	long intergenic non-protein coding RNA 152	chr2	lincRNA
ENSG00000162078.10	-4.2067941	0.16515861	1.03E-16	1.90E-15	2.83	1	0.22	0.06	ZG16B	zymogen granule protein 16B	chr16	protein_coding
ENSG00000271851.1	4.1463881	-1.9121319	1.13E-05	5.42E-05	0	0.01	0.12	0.21	RP11-565F1	--	chr17	sense_intronic
ENSG00000123473.14	1.76045465	2.25401906	5.01E-08	3.49E-07	0.32	0.34	1.08	1.91	STIL	SCL/TAL1 interrupting locus	chr1	protein_coding
ENSG00000165288.10	1.07358134	1.68369618	0.00102328	0.00335523	0.1	0.19	0.37	0.43	BRWD3	bromodomain and WD repeat domain containing 3	chrX	protein_coding
ENSG00000056097.14	1.75354355	3.98547045	5.91E-09	4.69E-08	1.18	1.28	3.69	7.45	ZFR	zinc finger RNA binding protein	chr5	protein_coding
ENSG00000129451.10	6.86350877	-1.7572063	8.18E-08	5.55E-07	0	0	0.14	0.14	KLK10	kallikrein-related peptidase 10	chr19	protein_coding
ENSG00000166501.11	3.79417561	-2.1660723	0.00013996	0.00054609	0	0	0.03	0.04	PRKCB	protein kinase C, beta	chr16	protein_coding
ENSG00000261655.1	-7.3825945	-1.5128223	4.49E-10	4.11E-09	0.37	0.14	0	0	CTD-3064M	--	chr8	sense_overlapping
ENSG00000176208.7	3.81967082	-0.1622842	6.53E-13	8.35E-12	0.02	0	0.15	0.32	ATAD5	ATPase family, AAA domain containing 5	chr17	protein_coding
ENSG00000139269.2	-6.5845241	3.1285619	2.82E-52	4.56E-50	5.59	5.97	0.08	0.08	INHBE	inhibin, beta E	chr12	protein_coding
ENSG00000271858.4	2.58071431	-1.9464163	0.00270895	0.00811738	0.02	0.05	0.21	0.36	CYB561D2	cytochrome b561 family, member D2	chr3	antisense
ENSG00000270024.4	2.41683758	-1.500183	0.00036944	0.00132576	0.01	0.04	0.12	0.22	C8orf44-SGK	C8orf44-SGK3 readthrough	chr8	protein_coding
ENSG00000198482.9	6.34320035	-0.069864	2.22E-18	4.72E-17	0.01	0	0.37	0.53	ZNF808	zinc finger protein 808	chr19	protein_coding
ENSG00000224592.4	5.19748351	-2.8969879	0.00538165	0.01488851	0	0	0.11	0.37	RP5-884C9.2	--	chr1	lincRNA
ENSG00000239213.4	1.76264982	1.46320651	2.71E-07	1.70E-06	0.62	0.75	2.13	4.1	NCK1-AS1	NCK1 antisense RNA 1 (head to head)	chr3	antisense
ENSG00000073536.16	-1.2635174	4.68261261	1.58E-05	7.38E-05	5.75	5.27	3.18	2.93	NLE1	notchless homolog 1 (Drosophila)	chr17	protein_coding
ENSG00000059758.6	1.98718301	0.94216806	7.35E-08	5.03E-07	0.11	0.15	0.42	0.97	CDK17	cyclin-dependent kinase 17	chr12	protein_coding
ENSG00000146859.6	4.72236738	-0.2460574	1.23E-14	1.89E-13	0.03	0.01	0.75	0.8	TMEM140	transmembrane protein 140	chr7	protein_coding
ENSG00000068438.13	-1.9485689	6.37995709	4.07E-11	4.25E-10	50.59	46.86	16.23	17.45	FTSJ1	FtsJ RNA methyltransferase homolog 1 (E. coli)	chrX	protein_coding
ENSG00000165355.7	2.77167069	0.94262878	2.49E-12	3.00E-11	0.03	0.16	0.69	1.09	FBXO33	F-box protein 33	chr14	protein_coding
ENSG00000132294.12	2.94179283	2.77507246	1.43E-18	3.08E-17	0.24	0.22	1.94	2.76	EFR3A	EFR3 homolog A (S. cerevisiae)	chr8	protein_coding
ENSG00000124374.8	3.13194027	1.98204302	2.96E-18	6.23E-17	0.09	0.11	1.21	1.16	PAIP2B	poly(A) binding protein interacting protein 2B	chr2	protein_coding
ENSG00000280571.1	1.50827881	2.98732946	9.19E-07	5.32E-06	6.18	4.75	13.78	28.05	RP4-613B23.	--	chr3	protein_coding
ENSG00000139116.16	2.60087759	1.85662091	8.64E-14	1.21E-12	0.08	0.16	0.58	1.39	KIF21A	kinesin family member 21A	chr12	protein_coding
ENSG00000134198.8	-5.3724655	-2.9476208	0.00290522	0.00856312	0.06	0.02	0	0	TSPAN2	tetraspanin 2	chr1	protein_coding
ENSG00000114744.7	1.39551539	3.09937827	4.76E-06	2.46E-05	0.91	1.13	2.58	4.61	COMMD2	COMM domain containing 2	chr3	protein_coding
ENSG00000163349.20	1.2419742	3.08591656	4.27E-05	0.00018537	0.49	0.41	1.23	1.64	HIPK1	homeodomain interacting protein kinase 1	chr1	protein_coding
ENSG00000164587.10	-1.5707725	8.93445069	6.49E-08	4.47E-07	338.22	311.04	154.44	136.37	RPS14	ribosomal protein S14	chr5	protein_coding
ENSG00000250709.1	-5.2127486	-3.0337949	0.00538165	0.01488851	0	0.11	0	0	CCDC169-SC	CCDC169-SOHLH2 readthrough	chr13	protein_coding
ENSG00000253307.1	5.21249443	-2.8887793	0.00538165	0.01488851	0	0	0.4	0.34	RP11-10J21.	--	chr8	antisense
ENSG00000166246.12	2.74939245	-1.2563346	2.05E-05	9.38E-05	0.01	0.04	0.18	0.27	C16orf71	chromosome 16 open reading frame 71	chr16	protein_coding
ENSG00000205726.12	1.04569995	4.10008558	0.00038245	0.00136838	0.43	0.52	1.06	1.56	ITSN1	intersectin 1 (SH3 domain protein)	chr21	protein_coding
ENSG00000185567.6	-1.0108049	8.33506939	0.00042627	0.00151074	17.42	20.76	13.84	11.32	AHNAK2	AHNAK nucleoprotein 2	chr14	protein_coding
ENSG00000186523.13	-1.1321094	3.37511856	0.0001547	0.00059614	4.28	4.88	2.84	2.72	FAM86B1	family with sequence similarity 86, member B1	chr8	protein_coding
ENSG00000143554.12	-2.3557078	3.74044434	2.86E-14	4.24E-13	5.85	4.94	1.54	1.27	SLC27A3	solute carrier family 27 (fatty acid transporter), member 3	chr1	protein_coding
ENSG00000224152.1	1.29709456	0.11630936	0.00148143	0.00470993	0.25	0.38	0.86	1.2	AC009506.1	--	chr2	antisense
ENSG00000071575.10	-6.910905	3.36190066	1.09E-55	2.16E-53	4.2	2.99	0.07	0.01	TRIB2	tribbles pseudokinase 2	chr2	protein_coding
ENSG00000117298.13	-2.4896738	9.50405329	1.00E-16	1.85E-15	178.98	181.17	43.46	41.92	ECE1	endothelin converting enzyme 1	chr1	protein_coding
ENSG00000153976.2	-5.6698633	-0.2936072	3.38E-18	7.06E-17	0.37	0.6	0	0.02	HS3ST3A1	heparan sulfate (glucosamine) 3-O-sulfotransferase 3A1	chr17	protein_coding
ENSG00000188596.8	1.92332854	-2.0769241	0.02035181	0.04824221	0.01	0	0.01	0.04	CFAP54	cilia and flagella associated 54	chr12	protein_coding
ENSG00000259881.1	-2.690065	-1.2756695	2.76E-05	0.0001235	0.22	0.12	0.07	0	RP11-830F9.	--	chr16	antisense
ENSG00000120694.18	2.38594613	4.08476745	1.07E-14	1.66E-13	0.59	0.95	3.74	7.09	HSPH1	heat shock 105kDa/110kDa protein 1	chr13	protein_coding
ENSG00000179583.16	2.80475871	1.32129404	1.01E-13	1.41E-12	0.03	0.05	0.38	0.33	CIITA	class II, major histocompatibility complex, transactivator	chr16	protein_coding
ENSG00000173801.15	-1.6206507	7.85800319	2.64E-08	1.91E-07	76.4	76.58	33.59	32.66	JUP	junction plakoglobin	chr17	protein_coding
ENSG00000164086.9	-1.7161999	5.1608421	5.89E-09	4.67E-08	13.92	12.65	5.63	5.14	DUSP7	dual specificity phosphatase 7	chr3	protein_coding
ENSG00000100479.11	1.32853263	1.06574769	0.00017602	0.00067128	0.39	0.45	0.81	1.98	POLE2	polymerase (DNA directed), epsilon 2, accessory subunit	chr14	protein_coding
ENSG00000159579.12	2.04173148	3.01243217	7.88E-11	7.97E-10	0.42	0.8	2.54	4.16	RSPRY1	ring finger and SPRY domain containing 1	chr16	protein_coding
ENSG00000156313.11	1.40070135	0.14601736	0.00044617	0.00157405	0.07	0.07	0.19	0.31	RPGR	retinitis pigmentosa GTPase regulator	chrX	protein_coding
ENSG00000274749.1	5.36404116	-2.8022277	0.00290522	0.00856312	0	0	0.4	0.08	KRTAP7-1	keratin associated protein 7-1 (gene/pseudogene)	chr21	protein_coding
ENSG00000136021.16	2.35343597	1.38627508	6.28E-11	6.44E-10	0.08	0.15	0.55	1.02	SCYL2	SCY1-like 2 (S. cerevisiae)	chr12	protein_coding
ENSG00000138780.13	1.95984018	0.37525726	1.13E-06	6.44E-06	0.13	0.02	0.24	0.54	GSTCD	glutathione S-transferase, C-terminal domain containing	chr4	protein_coding
ENSG00000184786.5	4.91020659	-3.0511096	0.0100455	0.02582742	0	0	0.25	0.07	TCTE3	t-complex-associated-testis-expressed 3	chr6	protein_coding
ENSG00000144182.15	2.81896091	-2.1942896	0.00354424	0.01027109	0	0.04	0.31	0.1	LIPT1	lipoyltransferase 1	chr2	protein_coding
ENSG00000146904.7	4.89980841	-3.0567113	0.01889873	0.04519557	0	0	0.04	0.03	EPHA1	EPH receptor A1	chr7	protein_coding
ENSG00000164164.14	1.10859587	1.66953507	0.00068602	0.00233732	0.15	0.25	0.61	0.56	OTUD4	OTU deubiquitinase 4	chr4	protein_coding
ENSG00000126777.16	3.09568969	4.95012165	6.98E-23	2.07E-21	1.13	0.85	6.29	16.67	KTN1	kinectin 1 (kinesin receptor)	chr14	protein_coding
ENSG00000107331.15	-2.4243819	7.45056192	5.79E-16	1.00E-14	30.3	26.71	7.14	7.01	ABCA2	ATP-binding cassette, sub-family A (ABC1), member 2	chr9	protein_coding
ENSG00000258813.2	2.11993601	-0.7726967	4.83E-05	0.00020679	0.19	0.11	1.03	0.76	RP11-109N2	--	chr14	antisense
ENSG00000196757.6	2.98863482	0.71625313	7.61E-13	9.66E-12	0.02	0.15	0.82	0.96	ZNF700	zinc finger protein 700	chr19	protein_coding
ENSG00000140526.15	1.26989811	5.84278742	1.22E-05	5.81E-05	2.59	3.31	9.25	9.68	ABHD2	abhydrolase domain containing 2	chr15	protein_coding
ENSG00000182054.8	1.20050488	7.00991352	3.19E-05	0.00014124	24.72	20.17	71.27	66.19	IDH2	isocitrate dehydrogenase 2 (NADP+), mitochondrial	chr15	protein_coding
ENSG00000080200.8	4.79853605	1.38836029	1.32E-26	5.07E-25	0.01	0.01	0.47	0.5	CRYBG3	beta-gamma crystallin domain containing 3	chr3	protein_coding
ENSG00000167311.12	-6.2762273	-2.3792352	2.70E-05	0.00012094	0.13	0.15	0	0	ART5	ADP-ribosyltransferase 5	chr11	protein_coding
ENSG00000235545.1	-5.2054745	-3.0371681	0.0100455	0.02582742	0.17	0	0	0	RP11-230B2	--	chr1	antisense
ENSG00000179104.7	2.59942274	1.7494342	1.87E-13	2.53E-12	0.09	0.11	0.71	0.84	TMTC2	transmembrane and tetratricopeptide repeat containing 2	chr12	protein_coding
ENSG00000258777.1	1.77851453	-0.6262459	0.00025423	0.00094401	0.41	0.22	1.38	1.55	HIF1A-AS1	HIF1A antisense RNA 1	chr14	lincRNA
ENSG00000188643.9	-2.9757335	7.80817379	2.52E-22	7.24E-21	219.95	178.89	36.12	31.37	S100A16	S100 calcium binding protein A16	chr1	protein_coding
ENSG00000169116.10	3.15069883	-1.9644347	0.00016274	0.00062431	0.01	0	0.01	0.12	PARM1	prostate androgen-regulated mucin-like protein 1	chr4	protein_coding
ENSG00000180155.17	3.39500141	0.6690081	6.32E-15	1.00E-13	0.03	0.04	0.57	0.43	LYNX1	Ly6/neurotoxin 1	chr8	protein_coding
ENSG00000142166.11	1.43500111	2.06830646	8.25E-06	4.07E-05	0.28	0.3	0.63	1.49	IFNAR1	interferon (alpha, beta and omega) receptor 1	chr21	protein_coding
ENSG00000172020.11	-1.6784701	-1.8176476	0.01347572	0.03367454	0.12	0.16	0.06	0.05	GAP43	growth associated protein 43	chr3	protein_coding
ENSG00000113231.12	-1.0671964	0.94624047	0.00201089	0.00616196	0.24	0.38	0.18	0.22	PDE8B	phosphodiesterase 8B	chr5	protein_coding
ENSG00000103381.10	-9.3270386	2.48199022	8.95E-54	1.57E-51	1.29	1.6	0.01	0	CPPED1	calcineurin-like phosphoesterase domain containing 1	chr16	protein_coding
ENSG00000145293.13	1.34028388	2.53591144	1.78E-05	8.27E-05	1.29	1.29	3.62	5.12	ENOPH1	enolase-phosphatase 1	chr4	protein_coding
ENSG00000135473.13	-1.3685145	3.91470381	4.12E-06	2.15E-05	3.37	2.94	1.45	1.81	PAN2	PAN2 poly(A) specific ribonuclease subunit	chr12	protein_coding
ENSG00000173917.10	1.92320901	2.18580785	3.90E-09	3.17E-08	0.86	1.07	5.58	4.13	HOXB2	homeobox B2	chr17	protein_coding
ENSG00000066027.10	1.56295715	2.2322956	9.97E-07	5.75E-06	0.63	0.47	1.39	2.98	PPP2R5A	protein phosphatase 2, regulatory subunit B', alpha	chr1	protein_coding

ENSG00000169371.12	-1.6421437	4.08573931	3.80E-08	2.68E-07	9.1	9.71	3.63	4.41	SNUPN	snurportin 1	chr15	protein_coding
ENSG00000129244.7	4.9672026	-1.2786058	1.07E-08	8.19E-08	0	0.01	0.36	0.1	ATP1B2	ATPase, Na+/K+ transporting, beta 2 polypeptide	chr17	protein_coding
ENSG00000225932.3	5.67347229	-2.6109587	0.00047761	0.00167191	0	0	0.05	0.11	CTAGE4	CTAGE family, member 4	chr7	protein_coding
ENSG00000153933.8	2.08657184	1.19598472	7.52E-09	5.86E-08	0.06	0.07	0.38	0.4	DGKE	diacylglycerol kinase, epsilon 64kDa	chr17	protein_coding
ENSG00000232807.2	-2.4040612	0.09447097	1.36E-08	1.02E-07	0.36	0.37	0.1	0.08	RP11-536K7.	--	chr10	antisense
ENSG00000157985.16	-1.1369085	5.17692418	9.05E-05	0.00036647	2.9	3.38	1.77	2.03	AGAP1	ArfGAP with GTPase domain, ankyrin repeat and PH domain 1	chr2	protein_coding
ENSG00000134369.14	-4.4366041	5.91183531	1.46E-40	1.16E-38	6.36	6.83	0.5	0.31	NAV1	neuron navigator 1	chr1	protein_coding
ENSG00000165185.13	2.31765822	1.29745778	1.67E-10	1.62E-09	0.03	0.07	0.32	0.32	KIAA1958	KIAA1958	chr9	protein_coding
ENSG00000157426.12	1.56975046	0.30855066	7.67E-05	0.00031656	0.07	0.17	0.51	0.44	AASDH	aminoadipate-semialdehyde dehydrogenase	chr4	protein_coding
ENSG00000188015.8	-6.5291538	5.4993637	4.67E-66	1.55E-63	78.67	73.09	1.31	0.88	S100A3	S100 calcium binding protein A3	chr1	protein_coding
ENSG00000277476.1	1.30384385	-0.0081158	0.00153861	0.00487879	0.19	0.12	0.49	0.54	RP11-147L1:	--	chr17	lincRNA
ENSG00000273341.1	4.87917439	-3.0678699	0.01889873	0.04519557	0	0	0	0.3	RP5-899E9.1	--	chr7	lincRNA
ENSG00000088035.14	1.7441404	0.7272937	3.19E-06	1.70E-05	0.09	0.26	0.65	0.89	ALG6	ALG6, alpha-1,3-glucosyltransferase	chr1	protein_coding
ENSG00000152193.7	1.60944913	1.5338651	2.24E-06	1.23E-05	0.26	0.38	0.9	1.69	RNF219	ring finger protein 219	chr13	protein_coding
ENSG00000100767.14	-1.4523276	3.94866371	1.08E-06	6.18E-06	3.42	2.59	1.62	1.3	PAPLN	papilin, proteoglycan-like sulfated glycoprotein	chr14	protein_coding
ENSG00000270419.1	1.75172234	0.57817356	4.18E-06	2.18E-05	0.93	0.22	3.5	1.73	CAHM	colon adenocarcinoma hypermethylated (non-protein coding)	chr6	lincRNA
ENSG00000057149.13	-6.7729581	-2.0122722	3.73E-07	2.30E-06	0.06	0.25	0	0	SERPINB3	serpin peptidase inhibitor, clade B (ovalbumin), member 3	chr18	protein_coding
ENSG00000210082.2	1.96191538	12.8009132	2.48E-11	2.66E-10	1250.18	1783.01	8719.44	6947.33	MT-RNR2	mitochondrially encoded 16S RNA	chrM	Mt_rRNA
ENSG00000188677.13	-4.5895068	6.69749298	2.84E-43	2.64E-41	28.64	25.62	1.59	1.41	PARVB	parvin, beta	chr22	protein_coding
ENSG00000139354.9	1.99310907	-1.5909308	0.00345484	0.01003143	0.05	0	0.09	0.19	GAS2L3	growth arrest-specific 2 like 3	chr12	protein_coding
ENSG00000137166.13	-1.2135627	6.21042104	2.74E-05	0.00012274	13.89	14.27	8.92	7.21	FOXP4	forkhead box P4	chr6	protein_coding
ENSG00000256802.2	-4.8298291	-1.7636928	3.95E-07	2.43E-06	0.2	0.09	0.01	0	RP11-680F8.	--	chr15	antisense
ENSG00000131126.17	1.97315743	0.55816501	4.53E-07	2.76E-06	0.36	0.22	1.98	1.06	TEX101	testis expressed 101	chr19	protein_coding
ENSG00000128602.8	-5.9644579	-0.7944111	2.81E-15	4.60E-14	0.31	0.16	0.01	0	SMO	smoothened, frizzled class receptor	chr7	protein_coding
ENSG00000166394.13	-2.0533787	2.582366	1.40E-10	1.37E-09	4.97	3.4	1.71	0.98	CYB5R2	cytochrome b5 reductase 2	chr11	protein_coding
ENSG00000115758.11	-1.1549502	8.1997422	5.98E-05	0.00025171	116.25	105.61	61.03	72.06	ODC1	ornithine decarboxylase 1	chr2	protein_coding
ENSG00000115841.18	3.71724114	-0.4784703	7.64E-11	7.77E-10	0.03	0	0.15	0.43	RMDN2	regulator of microtubule dynamics 2	chr2	protein_coding
ENSG00000133026.11	2.65486984	5.84748139	2.45E-18	5.19E-17	1.51	1.78	12.81	14.8	MYH10	myosin, heavy chain 10, non-muscle	chr17	protein_coding
ENSG00000267549.4	-1.775735	-2.081061	0.02035181	0.04824221	0.16	0.02	0.03	0.04	AC006116.17	--	chr19	antisense
ENSG00000130283.8	-1.9885181	1.06086112	2.11E-08	1.54E-07	1.06	1.06	0.33	0.38	GDF1	growth differentiation factor 1	chr19	protein_coding
ENSG00000185386.13	-1.1134922	4.81678876	0.0001322	0.00051953	14.37	11.25	8.2	7.58	MAPK11	mitogen-activated protein kinase 11	chr22	protein_coding
ENSG00000267882.2	-2.7353283	-2.5717591	0.01191236	0.03011315	0.12	0.03	0.03	0	RP4-569M2:	--	chr20	antisense
ENSG00000159596.6	-1.0357934	3.39368818	0.00050031	0.00174678	4.41	4.95	2.71	3.38	TMEM69	transmembrane protein 69	chr1	protein_coding
ENSG00000164684.12	1.63171395	0.34388791	2.97E-05	0.00013206	0.05	0.02	0.13	0.14	ZNF704	zinc finger protein 704	chr8	protein_coding
ENSG00000173805.14	2.17342382	2.26381932	3.95E-11	4.13E-10	0.2	0.26	1.42	1.38	HAP1	huntingtin-associated protein 1	chr17	protein_coding
ENSG00000143256.4	-1.5539993	7.7992202	9.07E-08	6.11E-07	396.4	398.39	178.9	181.77	PFDN2	prefoldin subunit 2	chr1	protein_coding
ENSG00000014123.9	2.39165688	0.7212881	8.86E-10	7.81E-09	0.1	0.09	0.41	0.92	UFL1	UFM1-specific ligase 1	chr6	protein_coding
ENSG00000104343.18	1.33266558	0.45932017	0.00055915	0.00194006	0.06	0.08	0.14	0.33	UBE2W	ubiquitin-conjugating enzyme E2W (putative)	chr8	protein_coding
ENSG00000145832.11	5.57068129	-2.6758978	0.00086564	0.00288128	0	0	0.03	0.05	SLC25A48	solute carrier family 25, member 48	chr5	protein_coding
ENSG00000188487.10	-3.7887629	-2.5063098	0.00112121	0.00364442	0.12	0	0.01	0	INSC	inscuteable homolog (Drosophila)	chr11	protein_coding
ENSG00000125531.6	-5.4337322	-1.2718081	1.38E-11	1.53E-10	0.24	0.15	0	0.01	C20orf195	chromosome 20 open reading frame 195	chr20	protein_coding
ENSG00000107485.14	9.007271	2.45654538	5.73E-50	7.80E-48	0	0.01	3.36	4.15	GATA3	GATA binding protein 3	chr10	protein_coding
ENSG00000213402.2	1.55702003	-0.867657	0.00217107	0.00661139	0.17	0.13	0.57	0.61	PTPRCAP	protein tyrosine phosphatase, receptor type, C-associated protein	chr11	protein_coding
ENSG00000163421.7	7.45331672	-1.2655328	7.65E-11	7.77E-10	0	0	0.14	0.73	PROK2	prokineticin 2	chr3	protein_coding
ENSG00000235590.6	-2.942957	-1.2748499	7.05E-06	3.52E-05	0.19	0.1	0.05	0	GNAS-AS1	GNAS antisense RNA 1	chr20	antisense
ENSG00000197496.5	5.53794007	0.86003432	1.41E-24	4.72E-23	0.01	0.01	0.65	0.84	SLC2A10	solute carrier family 2 (facilitated glucose transporter), member 10	chr20	protein_coding
ENSG00000247400.3	2.14814526	-1.4851705	0.00086862	0.00288921	0.04	0.02	0.19	0.16	DNAJC3-AS1	DNAJC3 antisense RNA 1 (head to head)	chr13	lincRNA
ENSG00000101306.9	2.526226	0.56459868	6.22E-10	5.58E-09	0.14	0.08	1.06	0.66	MYLK2	myosin light chain kinase 2	chr20	protein_coding
ENSG00000276410.2	2.05010012	-1.2119617	0.00040219	0.00143131	0.1	0.3	1.25	0.97	HIST1H2BB	histone cluster 1, H2bb	chr6	protein_coding
ENSG00000280789.1	-1.6444443	0.65881732	1.08E-05	5.19E-05	1.21	1.02	0.6	0.35	PAGR1	PAXIP1 associated glutamate-rich protein 1	chr16	protein_coding
ENSG00000227591.4	-4.731592	-1.841575	1.01E-06	5.80E-06	0.73	0.83	0.07	0	RP1-28O10.1	--	chr1	antisense
ENSG00000205302.5	1.54339393	2.64228169	8.01E-07	4.68E-06	1.1	0.79	2.7	4.71	SNX2	sorting nexin 2	chr5	protein_coding
ENSG00000137710.13	1.76520319	4.36194012	3.74E-09	3.05E-08	1.07	1.64	4.51	7.84	RDX	radixin	chr11	protein_coding
ENSG00000169885.8	-2.2571974	-1.3180987	7.72E-05	0.00031861	0.39	0.49	0.06	0.17	CALML6	calmodulin-like 6	chr1	protein_coding
ENSG00000280589.1	1.94627049	-1.4451036	0.00185375	0.00571918	0.78	1.1	6.53	3.44	AL449212.1	--	chr3	miRNA
ENSG00000137393.9	1.55198089	-0.1757005	0.00025876	0.00095857	0.07	0.06	0.27	0.25	RNF144B	ring finger protein 144B	chr6	protein_coding
ENSG00000125753.12	-1.0235932	8.32486409	0.00036118	0.00129866	154.08	147.81	106.92	90.51	VASP	vasodilator-stimulated phosphoprotein	chr19	protein_coding
ENSG00000244479.5	3.86702792	-1.4358209	1.03E-06	5.93E-06	0.01	0	0.14	0.12	OR2A1-AS1	OR2A1 antisense RNA 1	chr7	antisense
ENSG00000267446.1	2.26245794	-2.5331601	0.01923175	0.0458365	0	0.15	0.58	0.42	CTB-39G8.2	--	chr17	lincRNA
ENSG00000034677.10	1.85655932	1.3178015	1.22E-07	8.08E-07	0.21	0.14	0.73	0.96	RNF19A	ring finger protein 19A, RBR E3 ubiquitin protein ligase	chr8	protein_coding
ENSG00000116285.11	-1.1056877	5.44443768	0.00013427	0.00052644	11.08	12.01	5.27	9.11	ERRFI1	ERBB receptor feedback inhibitor 1	chr1	protein_coding
ENSG00000166165.11	-1.3930342	8.2212627	1.48E-06	8.31E-06	225.58	183.55	106.13	101.5	CKB	creatine kinase, brain	chr14	protein_coding
ENSG00000087338.4	1.47852024	0.96677898	3.15E-05	0.00013953	0.22	0.16	0.65	0.77	GMCL1	germ cell-less, spermatogenesis associated 1	chr2	protein_coding
ENSG00000175449.12	-1.5530261	-1.0053764	0.00166616	0.00519662	0.18	0.1	0.03	0.09	RFESD	Rieske (Fe-S) domain containing	chr5	protein_coding
ENSG00000118046.13	-5.6597623	1.15029169	1.36E-29	6.16E-28	1.11	1.14	0.05	0.01	STK11	serine/threonine kinase 11	chr19	protein_coding
ENSG00000182109.6	-2.069208	0.98291041	1.53E-08	1.14E-07	0.69	0.98	0.35	0.19	RP11-69E11.	--	chr1	antisense
ENSG00000185222.7	1.21806773	4.79292652	3.09E-05	0.00013709	9.35	15.26	29.52	46.98	WBP5	WW domain binding protein 5	chrX	protein_coding
ENSG00000276334.1	5.96106449	-2.422601	8.36E-05	0.00034052	0	0	0.16	0.15	AL133243.1	--	chr2	sense_intronic
ENSG00000271380.1	1.20520423	-0.7795909	0.01671588	0.04086408	0.23	0.39	0.68	1.23	RP11-307C1:	--	chr1	antisense
ENSG00000186260.15	2.22754718	2.32308339	1.17E-11	1.31E-10	0.11	0.16	0.79	0.91	MKL2	MKL/myocardin-like 2	chr16	protein_coding
ENSG00000082258.11	3.640056	0.3493606	1.33E-14	2.05E-13	0.02	0.02	0.2	0.48	CCNT2	cyclin T2	chr2	protein_coding
ENSG00000130066.15	1.62509084	6.23262754	2.77E-08	2.00E-07	15.75	19.67	68.1	77.54	SAT1	spermidine/spermine N1-acetyltransferase 1	chrX	protein_coding
ENSG00000054938.14	-3.3180827	-2.7845174	0.00943894	0.02469931	0.07	0.07	0.02	0	CHRDL2	chordin-like 2	chr11	protein_coding
ENSG00000165282.12	-1.3163975	5.24254755	6.36E-06	3.20E-05	10.8	9.97	6.23	4.85	PIGO	phosphatidylinositol glycan anchor biosynthesis, class O	chr9	protein_coding
ENSG00000129673.8	6.19483651	-2.260378	2.70E-05	0.00012094	0	0	0.15	0.13	AANAT	aralkylamine N-acetyltransferase	chr17	protein_coding
ENSG00000155111.13	1.57728444	1.48529333	3.30E-06	1.75E-05	0.2	0.14	0.62	0.75	CDK19	cyclin-dependent kinase 19	chr6	protein_coding
ENSG00000119397.15	3.13579658	1.56465521	4.82E-17	9.16E-16	0.06	0.05	0.52	0.8	CNTRL	centriolin	chr9	protein_coding
ENSG00000108443.12	2.26808158	1.99374975	2.11E-11	2.27E-10	0.15	0.23	0.89	1.58	RPS6KB1	ribosomal protein S6 kinase, 70kDa, polypeptide 1	chr17	protein_coding
ENSG00000102871.14	1.66148016	4.65512298	2.15E-08	1.57E-07	4.2	3.69	16.96	16.3	TRADD	TNFRSF1A-associated via death domain	chr16	protein_coding
ENSG00000165392.8	1.16183209	1.70566862	0.00037689	0.0013495	0.26	0.35	0.53	1.29	WRN	Werner syndrome, RecQ helicase-like	chr8	protein_coding
ENSG00000270123.3	-1.234609	-1.2063757	0.01656825	0.04052383	5.48	1.34	0.76	3	VTRNA2-1	vault RNA 2-1	chr5	vaultRNA
ENSG00000006071.10	-6.8983945	0.08051631	1.78E-23	5.52E-22	0.33	0.36	0.01	0	ABCC8	ATP-binding cassette, sub-family C (CFTR/MRP), member 8	chr11	protein_coding
ENSG00000224846.2	-6.4324245	-2.2669157	5.19E-06	2.66E-05	0.6	0.37	0	0	RP1-90J20.8	--	chr6	antisense
ENSG00000006634.6	1.56578038	0.72069888	2.35E-05	0.00010681	0.1	0.22	0.41	0.88	DBF4	DBF4 zinc finger	chr7	protein_coding
ENSG00000183773.14	3.59152281	-0.8639965	1.11E-08	8.42E-08	0.04	0	0.38	0.35	AIFM3	apoptosis-inducing factor, mitochondrion-associated, 3	chr22	protein_coding
ENSG00000090989.16	1.13240819	1.3407013	0.00077872	0.00262551	0.31	0.38	0.72	1.32	EXOC1	exocyst complex component 1	chr4	protein_coding
ENSG00000196159.10	-3.522463	-1.6534026	1.10E-06	6.29E-06	0.02	0.03	0	0	FAT4	FAT atypical cadherin 4	chr4	protein_coding
ENSG00000213639.8	2.00802983	3.42101408	8.20E-11	8.28E-10	0.65	0.71	2.48	4.9	PPP1CB	protein phosphatase 1, catalytic subunit, beta isozyme	chr2	protein_coding
ENSG00000232759.1	-5.0206302	-3.1306986	0.0100455	0.02582742	0.05	0.02	0	0	AC002480.3	--	chr7	antisense
ENSG00000005102.11	-5.0182885	0.53908768	2.34E-22	6.71E-21	0.95	0.72	0.07	0	MEOX1	mesenchyme homeobox 1	chr17	protein_coding
ENSG00000113739.9	-5.4048952	10.6211139	4.03E-56	8.27E-54	453.59	477.55	15.1	14.14	STC2	stanniocalcin 2	chr5	protein_coding
ENSG00000185947.13	2.39151149	0.97694553	2.47E-10	2.34E-09	0.14	0.15	0.87	1.15	ZNF267	zinc finger protein 267	chr16	protein_coding
ENSG00000164690.6	7.22117676	-1.4624919	1.76E-09	1.50E-08	0	0	0.07	0.2	SHH	sonic hedgehog	chr7	protein_coding
ENSG00000198858.8	-1.120181	6.45166049	0.00010341	0.00041536	49.08	53.33	33.82	28.78	R3HDM4	R3H domain containing 4	chr19	protein_coding
ENSG00000151718.14	3.88608851	2.86171097	1.11E-27	4.55E-26	0.08	0.08	1.2	2.02	WWC2	WW and C2 domain containing 2	chr4	protein_coding
ENSG00000136942.13	-1.7429162	11.576457	2.36E-09	1.97E-08	7512.16	6607.21	2903.61	2714.73	RPL35	ribosomal protein L35	chr9	protein_coding
ENSG00000140534.12	1.05651724	5.03166689	0.00027696	0.00101488	2.49	2.57	6.94	7.1	TICRR	TOPBP1-interacting checkpoint and replication regulator	chr15	protein_coding
ENSG00000144063.3	-6.0449874	5.21296943	1.65E-59	3.84E-57	21.33	17.3	0.46	0.32	MALL	mal, T-cell differentiation protein-like	chr2	protein_coding
ENSG00000102753.8	1.54380635	1.50380011	5.56E-06	2.83E-05	0.2	0.3	0.53	1.45	KPNA3	karyopherin alpha 3 (importin alpha 4)	chr13	protein_coding
ENSG00000139631.17	2.15200194	1.48613944	8.38E-10	7.39E-09	0.21	0.33	1.16	2.02	CSAD	cysteine sulfinic acid decarboxylase	chr12	protein_coding
ENSG00000278434.1	-2.1669132	-1.2314283	0.00021372	0.00080378	0.44	0.25	0.13	0.07	RP11-709D2	--	chr16	sense_intronic
ENSG00000092621.10	-2.4705094	10.2367443	1.60E-16	2.92E-15	488.06	469.63	120.87	109.05	PHGDH	phosphoglycerate dehydrogenase	chr1	protein_coding
ENSG00000117122.12	-8.0285443	3.70014427	4.67E-65	1.44E-62	16.07	17.27	0.1	0.06	MFAP2	microfibrillar-associated protein 2	chr1	protein_coding
ENSG00000177606.6	-1.5306121	7.30281129	1.41E-07	9.26E-07	56.3	50.94	23.67	25.86	JUN	jun proto-oncogene	chr1	protein_coding
ENSG00000140688.15	-1.0779764	6.73070618	0.00018199	0.00069225	40.82	42.2	26.62	25.74	C16orf58	chromosome 16 open reading frame 58	chr16	protein_coding
ENSG00000160111.11	-4.8413846	-1.0362256	6.98E-12	7.96E-11	0.1	0.14	0.01	0	CPAMD8	C3 and PZP-like, alpha-2-macroglobulin domain containing 8	chr19	protein_coding
ENSG00000176723.8	-1.1061789	-0.6395142	0.01404995	0.03495281	0.17	0.12	0.06	0.12	ZNF843	zinc finger protein 843	chr16	protein_coding
ENSG00000101856.9	1.90481753	5.27770214	1.35E-10	1.33E-09	7.55	6.19	27.46	41.55	PGRMC1	progesterone receptor membrane component 1	chrX	protein_coding
ENSG00000247828.6	-1.8814846	2.75570387	1.77E-09	1.50E-08	1.76	2.11	0.54	0.86	TMEM161B-.	TMEM161B antisense RNA 1	chr5	antisense
ENSG00000110871.13	-1.6849462	4.45233528	1.47E-08	1.10E-07	19.18	15.99	6.7	7.91	COQ5	coenzyme Q5 homolog, methyltransferase (S. cerevisiae)	chr12	protein_coding
ENSG00000100473.14	1.12032456	3.15229048	0.00020388	0.00076859	1.13	1.76	3.25	5.15	COCH	cochlin	chr14	protein_coding
ENSG00000196693.13	2.16924415	0.39727691	9.99E-08	6.71E-07	0.08	0.04	0.13	0.59	ZNF33B	zinc finger protein 33B	chr10	protein_coding
ENSG00000095587.8	4.64313903	0.04317617	1.70E-16	3.08E-15	0	0.02	0.3	0.27	TLL2	tolloid-like 2	chr10	protein_coding
ENSG00000101417.10	2.03774446	4.08588134	2.09E-11	2.26E-10	0.97	0.9	5.04	5.19	PXMP4	peroxisomal membrane protein 4, 24kDa	chr20	protein_coding
ENSG00000235092.4	3.35715987	-1.8204565	0.00010631	0.0004256	0	0.03	0.23	0.17	ID2-AS1	ID2 antisense RNA 1 (head to head)	chr2	antisense
ENSG00000137936.15	-1.0059655	5.95497191	0.00048576	0.00169895	16.41	16.44	10.39	11.43	BCAR3	breast cancer anti-estrogen resistance 3	chr1	protein_coding
ENSG00000196611.4	7.44071534	-1.2794212	1.18E-10	1.17E-09	0	0	0.2	0.51	MMP1	matrix metallopeptidase 1	chr11	protein_coding
ENSG00000131626.14	1.36713418	4.43993239	3.66E-06	1.93E-05	1.43	2.05	5.34	6.65	PPFIA1	protein tyrosine phosphatase, receptor type, f polypeptide (PTPRF), interacting protein (liprin), alpha 1	chr11	protein_coding
ENSG00000256950.2	2.90443354	-2.7265114	0.01615662	0.03958166	0	0.05	0.5	0.09	RP11-87C12	--	chr12	protein_coding
ENSG00000172794.18	2.59000584	-0.299263	7.24E-08	4.96E-07	0.04	0.03	0.32	0.28	RAB37	RAB37, member RAS oncogene family	chr17	protein_coding
ENSG00000114857.16	3.73563012	2.64611694	1.67E-25	5.98E-24	0.06	0.12	1.02	2.2	NKTR	natural killer cell triggering receptor	chr3	protein_coding
ENSG00000000971.14	-5.4735234	1.81401092	1.15E-34	6.52E-33	1.23	1.31	0.06	0.02	CFH	complement factor H	chr1	protein_coding
ENSG00000274419.3	-5.2523797	-0.6775234	7.94E-15	1.25E-13	0.28	0.3	0	0.02	TBC1D3D	TBC1 domain family, member 3D	chr17	protein_coding
ENSG00000269994.1	6.75188369	-1.8412616	2.24E-07	1.42E-06	0	0	0.14	0.28	RP11-276H1	--	chr9	lincRNA
ENSG00000178163.6	6.24102512	-2.2241472	1.55E-05	7.26E-05	0	0	0.02	0.06	ZNF518B	zinc finger protein 518B	chr4	protein_coding
ENSG00000254641.1	1.58086914	-0.5338567	0.00073084	0.00247628	0.62	0.53	3.29	1.4	RP11-732A1!	--	chr11	sense_overlapping
ENSG00000104881.13	-1.7109039	6.86199007	4.99E-09	3.99E-08	37.14	35.17	15.31	14.09	PPP1R13L	protein phosphatase 1, regulatory subunit 13 like	chr19	protein_coding
ENSG00000117620.11	1.97333657	0.39154962	8.19E-07	4.78E-06	0.06	0.07	0.27	0.41	SLC35A3	solute carrier family 35 (UDP-N-acetylglucosamine (UDP-GlcNAc) transporter), member A3	chr1	protein_coding
ENSG00000259087.4	-2.4880275	-2.7178412	0.00943894	0.02469931	0.71	0	0	0.14	RP11-356O9	--	chr14	lincRNA
ENSG00000272405.1	-5.7508224	4.95897729	1.98E-55	3.82E-53	14.81	15.91	0.45	0.31	RP11-284F2:	--	chr1	antisense
ENSG00000189091.11	1.06969569	8.91615893	0.00019476	0.00073686	26.27	25.65	73.17	72.01	SF3B3	splicing factor 3b, subunit 3, 130kDa	chr16	protein_coding
ENSG00000071537.12	1.12614141	3.39058212	0.00017354	0.00066233	0.59	0.64	1.27	2.34	SEL1L	sel-1 suppressor of lin-12-like (C. elegans)	chr14	protein_coding
ENSG00000162892.14	-4.5001961	1.37108691	8.08E-27	3.14E-25	2.55	1.49	0.06	0.17	IL24	interleukin 24	chr1	protein_coding
ENSG00000143127.11	-1.8206037	-0.4920685	4.19E-05	0.00018225	0.19	0.13	0.03	0.09	ITGA10	integrin, alpha 10	chr1	protein_coding
ENSG00000100353.16	-1.358261	8.79136047	2.60E-06	1.41E-05	228.27	228.26	116.95	120.37	EIF3D	eukaryotic translation initiation factor 3, subunit D	chr22	protein_coding
ENSG00000255455.2	1.58432452	-1.5299003	0.01254665	0.03155686	0.05	0	0.06	0.16	RP11-890B1!	--	chr11	lincRNA
ENSG00000138182.13	3.14607734	1.61372703	2.91E-17	5.61E-16	0.09	0.06	0.5	1.29	KIF20B	kinesin family member 20B	chr10	protein_coding
ENSG00000020181.16	-3.6908769	4.9186217	4.31E-30	1.99E-28	6.73	6.41	0.71	0.64	GPR124	--	chr8	protein_coding
ENSG00000009694.12	5.05694572	-2.9744029	0.0100455	0.02582742	0	0	0.01	0.01	TENM1	teneurin transmembrane protein 1	chrX	protein_coding
ENSG00000238622.1	3.06538058	-1.2909968	3.23E-06	1.72E-05	0.75	0	8.35	0.96	SNORD97	small nucleolar RNA, C/D box 97	chr11	snoRNA
ENSG00000134917.9	-9.530665	0.48682543	6.15E-28	2.55E-26	0.51	0.61	0	0	ADAMTS8	ADAM metallopeptidase with thrombospondin type 1 motif, 8	chr11	protein_coding
ENSG00000198003.10	1.49892041	1.97295451	3.77E-06	1.98E-05	0.83	0.63	2.77	2.74	CCDC151	coiled-coil domain containing 151	chr19	protein_coding
ENSG00000184206.10	2.49200375	0.65355257	4.83E-10	4.40E-09	0.08	0.08	0.46	0.72	GOLGA6L4	golgin A6 family-like 4	chr15	protein_coding
ENSG00000115194.9	2.26464263	1.1917523	4.95E-10	4.50E-09	0.19	0.35	1.67	1.78	SLC30A3	solute carrier family 30 (zinc transporter), member 3	chr2	protein_coding
ENSG00000234225.2	5.48410531	-2.7306244	0.00158008	0.00495521	0	0	0.39	0.11	RP4-704D21	--	chr1	lincRNA
ENSG00000198074.8	-1.1127009	-0.0906249	0.0067217	0.01821738	0.61	0.64	0.46	0.31	AKR1B10	aldo-keto reductase family 1, member B10 (aldose reductase)	chr7	protein_coding
ENSG00000072201.12	-3.3248169	-2.7868506	0.00943894	0.02469931	0.02	0.04	0	0.01	LNX1	ligand of numb-protein X 1, E3 ubiquitin protein ligase	chr4	protein_coding
ENSG00000175322.10	2.32754714	-1.0104642	5.34E-05	0.00022654	0.01	0.04	0.18	0.15	ZNF519	zinc finger protein 519	chr18	protein_coding
ENSG00000101298.12	3.38226786	2.85974218	7.72E-23	2.28E-21	0.11	0.25	2.72	2.31	SNPH	syntaphilin	chr20	protein_coding
ENSG00000126970.14	-7.9388059	1.92196334	5.75E-46	6.32E-44	1.79	2.1	0	0.02	ZC4H2	zinc finger, C4H2 domain containing	chrX	protein_coding
ENSG00000183134.4	-5.7844519	-2.7062645	0.00047761	0.00167191	0.02	0.1	0	0	PTGDR2	prostaglandin D2 receptor 2	chr11	protein_coding
ENSG00000006837.10	-1.385428	-1.3173084	0.01764505	0.04292107	0.15	0.16	0.12	0.04	CDKL3	cyclin-dependent kinase-like 3	chr5	protein_coding
ENSG00000234405.1	-6.9843499	-1.8429092	4.98E-08	3.47E-07	0.85	0.36	0	0	LL0XNC01-2	--	chrX	antisense
ENSG00000274618.1	5.81117022	-2.5254325	0.00026535	0.0009747	0	0	1.33	0.17	HIST1H4F	histone cluster 1, H4f	chr6	protein_coding
ENSG00000157796.16	1.9975046	0.85187564	1.06E-07	7.12E-07	0.07	0.13	0.36	0.71	WDR19	WD repeat domain 19	chr4	protein_coding
ENSG00000144744.15	1.4392372	2.36327123	4.95E-06	2.55E-05	0.97	0.96	2.79	4.23	UBA3	ubiquitin-like modifier activating enzyme 3	chr3	protein_coding
ENSG00000196387.8	2.58480051	1.05757224	1.15E-11	1.29E-10	0.11	0.14	0.66	1.35	ZNF140	zinc finger protein 140	chr12	protein_coding
ENSG00000166845.12	2.46749782	-0.4960609	8.82E-07	5.12E-06	0.01	0.04	0.09	0.3	C18orf54	chromosome 18 open reading frame 54	chr18	protein_coding
ENSG00000103202.11	-1.6484252	6.61616763	1.69E-08	1.26E-07	61.18	59.7	27.88	23.38	NME4	NME/NM23 nucleoside diphosphate kinase 4	chr16	protein_coding
ENSG00000146223.13	-1.2003045	5.37637865	3.57E-05	0.00015673	9.87	10.19	4.97	6.7	RPL7L1	ribosomal protein L7-like 1	chr6	protein_coding
ENSG00000124120.9	1.309367	3.56229039	1.27E-05	6.06E-05	0.95	0.79	2.82	2.95	TTPAL	tocopherol (alpha) transfer protein-like	chr20	protein_coding
ENSG00000186998.14	8.45813106	-0.3977621	1.66E-16	3.01E-15	0	0	0.65	0.61	EMID1	EMI domain containing 1	chr22	protein_coding
ENSG00000188176.10	6.54975397	-1.9968671	1.78E-06	9.85E-06	0	0	0.11	0.21	SMTNL2	smoothelin-like 2	chr17	protein_coding
ENSG00000165813.15	3.92194577	-0.0688601	4.23E-13	5.50E-12	0.01	0.01	0.1	0.31	CCDC186	coiled-coil domain containing 186	chr10	protein_coding
ENSG00000136156.11	2.12079755	6.15215532	9.91E-13	1.24E-11	1.91	2.33	10.23	14.46	ITM2B	integral membrane protein 2B	chr13	protein_coding
ENSG00000245849.5	1.12294081	0.07730701	0.00588421	0.01615395	0.38	0.81	1.56	1.88	RAD51-AS1	RAD51 antisense RNA 1 (head to head)	chr15	processed_transcript
ENSG00000167306.17	5.06915146	3.221823	3.80E-40	2.93E-38	0.03	0.06	1.76	2.07	MYO5B	myosin VB	chr18	protein_coding
ENSG00000164916.10	-1.7613172	6.65056259	1.88E-09	1.59E-08	11.97	10.48	4.52	4.31	FOXK1	forkhead box K1	chr7	protein_coding
ENSG00000184828.8	-5.2948532	2.7873498	1.27E-40	1.01E-38	1.77	2	0.06	0.07	ZBTB7C	zinc finger and BTB domain containing 7C	chr18	protein_coding
ENSG00000147065.15	-1.2579505	10.3168774	1.27E-05	6.03E-05	348.95	390.01	202.14	209.48	MSN	moesin	chrX	protein_coding
ENSG00000070669.15	-2.3977509	7.29236611	1.14E-15	1.92E-14	83.06	88.28	21.18	22.15	ASNS	asparagine synthetase (glutamine-hydrolyzing)	chr7	protein_coding
ENSG00000182134.14	1.53419452	3.17679048	5.13E-07	3.10E-06	0.74	1.19	3.3	4.15	TDRKH	tudor and KH domain containing	chr1	protein_coding
ENSG00000167658.14	-1.1613757	12.439178	5.33E-05	0.00022639	2034.81	1891.9	1181.96	1157.05	EEF2	eukaryotic translation elongation factor 2	chr19	protein_coding
ENSG00000137054.14	-1.998434	6.22740042	1.44E-11	1.58E-10	40.33	39.92	15	11.68	POLR1E	polymerase (RNA) I polypeptide E, 53kDa	chr9	protein_coding
ENSG00000034510.5	-1.2727212	11.4109947	1.00E-05	4.86E-05	6041.57	6538.38	3692.65	3228.38	TMSB10	thymosin beta 10	chr2	protein_coding
ENSG00000174373.14	2.34253418	0.68660967	3.15E-09	2.59E-08	0.02	0.08	0.3	0.37	RALGAPA1	Ral GTPase activating protein, alpha subunit 1 (catalytic)	chr14	protein_coding
ENSG00000116044.14	3.05596108	3.76107847	2.86E-21	7.63E-20	0.42	0.61	4.11	7.38	NFE2L2	nuclear factor, erythroid 2-like 2	chr2	protein_coding
ENSG00000259415.1	2.02099544	-2.3053695	0.01324986	0.0331641	0.19	0	0.6	0.51	RP11-7M10.:	--	chr15	sense_intronic
ENSG00000213199.6	2.12878226	-0.7590952	4.83E-05	0.00020679	0.05	0.09	0.28	0.49	ASIC3	acid sensing (proton gated) ion channel 3	chr7	protein_coding
ENSG00000023892.10	1.64136357	-0.4231334	0.00039779	0.00141692	0.17	0.06	0.62	0.35	DEF6	differentially expressed in FDCP 6 homolog (mouse)	chr6	protein_coding
ENSG00000096872.14	1.73883541	-0.1473225	6.15E-05	0.00025862	0.08	0.14	0.21	0.77	IFT74	intraflagellar transport 74	chr9	protein_coding

ENSG00000260992.1	2.16090096	-0.1070556	1.50E-06	8.40E-06	0.5	0.11	1.59	2.09	DOCK9-AS2	DOCK9 antisense RNA 2 (head to head)	chr13	antisense
ENSG00000207118.1	5.21249443	-2.8887793	0.00538165	0.01488851	0	0	1.84	1.57	SNORD14D	small nucleolar RNA, C/D box 14D	chr11	snoRNA
ENSG00000184178.14	-1.3578494	3.3653342	6.78E-06	3.40E-05	3.97	3.54	2.13	1.77	SCFD2	sec1 family domain containing 2	chr4	protein_coding
ENSG00000196923.12	-2.6366377	7.94590252	2.44E-18	5.19E-17	155.96	152.92	33.59	32.56	PDLIM7	PDZ and LIM domain 7 (enigma)	chr5	protein_coding
ENSG00000136536.13	3.1690078	1.55323086	3.56E-17	6.82E-16	0.08	0.07	0.58	1.19	7-Mar	membrane-associated ring finger (C3HC4) 7, E3 ubiquitin protein ligase	chr2	protein_coding
ENSG00000077232.15	1.03918286	2.8493321	0.00064935	0.00222439	0.18	0.18	0.29	0.7	DNAJC10	DnaJ (Hsp40) homolog, subfamily C, member 10	chr2	protein_coding
ENSG00000101888.10	2.45987292	-1.2578639	7.72E-05	0.00031861	0.03	0.03	0.15	0.26	NXT2	nuclear transport factor 2-like export factor 2	chrX	protein_coding
ENSG00000171150.7	2.002214	0.88863344	8.26E-08	5.60E-07	0.09	0.14	0.43	0.77	SOCS5	suppressor of cytokine signaling 5	chr2	protein_coding
ENSG00000273045.4	4.93279224	-0.5394703	7.54E-13	9.57E-12	0.03	0	0.36	1.03	C2orf15	chromosome 2 open reading frame 15	chr2	protein_coding
ENSG00000198168.7	-6.6914892	-0.1167396	1.86E-21	5.03E-20	0.38	0.26	0.01	0	SVIP	small VCP/p97-interacting protein	chr11	protein_coding
ENSG00000272144.1	5.97464286	-2.4149021	8.36E-05	0.00034052	0	0	0.34	0.16	CTD-2035E1	--	chr5	lincRNA
ENSG00000267037.1	3.10750404	-0.7582994	5.21E-08	3.63E-07	0.06	0.13	1.05	1.24	AC005757.7	--	chr19	lincRNA
ENSG00000155849.14	-1.7414	0.20516773	1.11E-05	5.36E-05	0.36	0.16	0.08	0.12	ELMO1	engulfment and cell motility 1	chr7	protein_coding
ENSG00000175066.14	2.70563334	1.83762138	1.95E-14	2.94E-13	0.08	0.07	0.54	0.78	GK5	glycerol kinase 5 (putative)	chr3	protein_coding
ENSG00000278053.3	2.75656975	2.68277077	1.12E-16	2.08E-15	0.14	0.28	1.43	2.31	DDX52	DEAD (Asp-Glu-Ala-Asp) box polypeptide 52	chr17	protein_coding
ENSG00000166908.16	1.23440877	4.95342031	2.35E-05	0.00010665	4.4	4.94	14.74	14.54	PIP4K2C	phosphatidylinositol-5-phosphate 4-kinase, type II, gamma	chr12	protein_coding
ENSG00000147255.16	-11.667767	2.61579223	1.05E-55	2.11E-53	1.41	1.68	0	0	IGSF1	immunoglobulin superfamily, member 1	chrX	protein_coding
ENSG00000099282.8	-2.8378863	1.07013999	4.53E-14	6.55E-13	1.87	1.62	0.21	0.43	TSPAN15	tetraspanin 15	chr10	protein_coding
ENSG00000170485.15	-2.4137284	6.42584231	8.73E-16	1.49E-14	27.3	31.25	7.18	7.46	NPAS2	neuronal PAS domain protein 2	chr2	protein_coding
ENSG00000260460.1	1.91686402	-1.3062033	0.00103764	0.00339765	0.03	0.09	0.4	0.22	RP11-284F2:	--	chr1	lincRNA
ENSG00000134013.14	-1.4098355	8.06452817	1.12E-06	6.39E-06	94.77	82.59	43.38	45.67	LOXL2	lysyl oxidase-like 2	chr8	protein_coding
ENSG00000278973.1	2.6409349	-1.5944372	0.00030126	0.00109685	0.1	0.04	0.41	0.8	RP11-83B20.	--	chr12	TEC
ENSG00000063660.7	-1.3251812	8.54367236	4.47E-06	2.32E-05	106.57	109.56	58.76	56.06	GPC1	glypican 1	chr2	protein_coding
ENSG00000204175.5	5.41925961	0.74283061	4.00E-24	1.30E-22	0.05	0	1.99	1.37	GPRIN2	G protein regulated inducer of neurite outgrowth 2	chr10	protein_coding
ENSG00000270058.1	-6.6406389	-2.111908	1.05E-06	6.02E-06	0.71	0.35	0	0	RP11-514D2	--	chr16	lincRNA
ENSG00000006062.12	-1.0951364	5.07985615	0.00016361	0.00062695	7.1	7.28	4.77	4.17	MAP3K14	mitogen-activated protein kinase kinase kinase 14	chr17	protein_coding
ENSG00000204410.13	-1.9996032	2.4826393	4.42E-10	4.07E-09	1.83	1.59	0.71	0.43	MSH5	mutS homolog 5	chr6	protein_coding
ENSG00000223396.2	-1.8535146	2.13109256	8.22E-09	6.38E-08	2.31	2.45	0.76	0.99	RP11-134G8	--	chr1	lincRNA
ENSG00000255284.1	1.02883388	3.0887078	0.00065283	0.00223374	4.96	6.78	14.91	17.02	AP006621.5	--	chr11	lincRNA
ENSG00000186314.10	-1.6546114	1.66593836	5.30E-07	3.19E-06	1.17	0.89	0.34	0.53	PRELID2	PRELI domain containing 2	chr5	protein_coding
ENSG00000177788.5	-4.806998	-3.2310942	0.01889873	0.04519557	0.09	0.1	0	0	RP5-1061H2	--	chr1	lincRNA
ENSG00000262160.1	2.29785183	0.98005934	1.28E-09	1.10E-08	0.12	0.21	0.92	1.2	RP11-96D1.1	--	chr16	antisense
ENSG00000132965.8	-2.8866617	-1.8056239	6.90E-05	0.00028699	0.06	0.49	0.05	0.04	ALOX5AP	arachidonate 5-lipoxygenase-activating protein	chr13	protein_coding
ENSG00000012048.18	1.76701016	2.89751932	1.45E-08	1.09E-07	0.32	0.37	1.34	1.8	BRCA1	breast cancer 1, early onset	chr17	protein_coding
ENSG00000266340.1	1.31285046	3.38769976	1.29E-05	6.14E-05	6.85	7.59	26.28	21.4	RP11-848P1.	--	chr17	antisense
ENSG00000279267.1	2.53172013	-2.3730592	0.00737015	0.01977584	0	0.03	0.05	0.17	RP11-395L1⁴	--	chr2	TEC
ENSG00000198712.1	1.14873197	12.5701204	6.40E-05	0.00026805	3565.52	5402.28	14176.64	12204.9	MT-CO2	mitochondrially encoded cytochrome c oxidase II	chrM	protein_coding
ENSG00000168268.9	-1.7810105	7.43972295	1.19E-09	1.03E-08	103.6	83.99	37.36	35.38	NT5DC2	5'-nucleotidase domain containing 2	chr3	protein_coding
ENSG00000266236.1	2.17437314	-0.8451825	5.51E-05	0.00023313	0.1	0.26	0.87	1.31	NARF-IT1	NARF intronic transcript 1	chr17	sense_intronic
ENSG00000139835.12	-6.2507323	3.00631666	4.63E-49	6.00E-47	5.7	5.75	0.11	0.08	GRTP1	growth hormone regulated TBC protein 1	chr13	protein_coding
ENSG00000138036.17	1.62736077	-0.2313262	0.00019848	0.00075024	0.05	0.11	0.31	0.37	DYNC2LI1	dynein, cytoplasmic 2, light intermediate chain 1	chr2	protein_coding
ENSG00000165685.7	3.00105428	-0.653708	9.89E-08	6.64E-07	0.03	0.04	0.33	0.35	TMEM52B	transmembrane protein 52B	chr12	protein_coding
ENSG00000267750.4	3.28894056	1.03798892	7.26E-16	1.24E-14	0.02	0.08	0.71	0.71	RUNDC3A-A	RUNDC3A antisense RNA 1	chr17	antisense
ENSG00000120688.8	1.07344504	2.05350035	0.00070822	0.00240643	0.82	0.8	1.81	2.74	WBP4	WW domain binding protein 4	chr13	protein_coding
ENSG00000136327.6	2.87437899	0.7406019	2.71E-12	3.24E-11	0.3	0.18	2.03	2.7	NKX2-8	NK2 homeobox 8	chr14	protein_coding
ENSG00000135525.17	1.49344874	3.66719946	6.75E-07	3.99E-06	0.74	1.23	3.47	3.91	MAP7	microtubule-associated protein 7	chr6	protein_coding
ENSG00000107854.5	2.40904918	2.1426053	9.53E-13	1.20E-11	0.17	0.18	0.79	1.77	TNKS2	tankyrase, TRF1-interacting ankyrin-related ADP-ribose polymerase 2	chr10	protein_coding
ENSG00000255306.1	-1.6950541	-0.8218393	0.00063882	0.0021908	0.58	1.19	0.35	0.37	RP5-901A4.1	--	chr11	antisense
ENSG00000111913.14	5.32944093	-2.821228	0.00290522	0.00856312	0	0	0.01	0.03	FAM65B	family with sequence similarity 65, member B	chr6	protein_coding
ENSG00000258472.7	1.33947269	-0.769634	0.00520073	0.01456448	0.58	0.33	1.57	1.55	RP11-192H2	--	chr17	protein_coding
ENSG00000275023.3	-1.3333465	6.76957379	4.15E-06	2.17E-05	13.71	13.24	7.26	6.99	MLLT6	myeloid/lymphoid or mixed-lineage leukemia (trithorax homolog, Drosophila); translocated to, 6	chr17	protein_coding
ENSG00000103723.11	1.02544735	4.06819523	0.00050061	0.00174757	1.15	1.33	3.3	3.41	AP3B2	adaptor-related protein complex 3, beta 2 subunit	chr15	protein_coding
ENSG00000186088.14	1.1632958	-0.0581661	0.00515988	0.01445684	0.13	0.11	0.28	0.44	GSAP	gamma-secretase activating protein	chr7	protein_coding
ENSG00000160062.13	2.21031293	0.67089174	1.53E-08	1.14E-07	0.02	0.1	0.37	0.38	ZBTB8A	zinc finger and BTB domain containing 8A	chr1	protein_coding
ENSG00000204632.10	1.87190973	-0.8422967	0.0003468	0.00125054	0.1	0.11	0.6	0.43	HLA-G	major histocompatibility complex, class I, G	chr6	protein_coding
ENSG00000261824.5	-2.5088104	1.65386249	3.24E-13	4.26E-12	1.67	1.82	0.27	0.54	LINC00662	long intergenic non-protein coding RNA 662	chr19	lincRNA
ENSG00000114796.14	2.16125998	1.49026861	7.64E-10	6.76E-09	0.09	0.11	0.49	0.71	KLHL24	kelch-like family member 24	chr3	protein_coding
ENSG00000236668.2	4.8843127	-3.0650858	0.01889873	0.04519557	0	0	0.01	0.09	RP11-85O21	--	chr9	antisense
ENSG00000144827.7	1.16101617	2.37907775	0.00020471	0.00077161	1.01	0.92	2.73	3.05	ABHD10	abhydrolase domain containing 10	chr3	protein_coding
ENSG00000025708.11	-2.536705	5.5793885	5.21E-17	9.86E-16	39.27	31.74	8.69	7.61	TYMP	thymidine phosphorylase	chr22	protein_coding
ENSG00000164181.12	4.09317207	0.87783154	9.78E-20	2.30E-18	0.04	0.03	0.41	1.22	ELOVL7	ELOVL fatty acid elongase 7	chr5	protein_coding
ENSG00000165458.12	-2.5056489	8.8817	6.81E-17	1.28E-15	133.81	123.7	30.37	30.06	INPPL1	inositol polyphosphate phosphatase-like 1	chr11	protein_coding
ENSG00000206530.7	2.79479553	0.32972277	1.35E-10	1.33E-09	0.02	0.03	0.2	0.21	CFAP44	cilia and flagella associated protein 44	chr3	protein_coding
ENSG00000196951.9	4.20762491	-0.3387744	6.69E-13	8.54E-12	0.02	0.02	0.51	0.68	SCOC-AS1	SCOC antisense RNA 1	chr4	antisense
ENSG00000224086.5	1.11636148	1.76505573	0.00060382	0.00208331	0.04	0.05	0.14	0.12	LL22NC03-8(	--	chr22	antisense
ENSG00000196866.2	2.76949383	0.13930395	1.03E-09	9.01E-09	0.68	0.36	4.32	5.34	HIST1H2AD	histone cluster 1, H2ad	chr6	protein_coding
ENSG00000253878.4	5.37402558	-2.7967694	0.00158008	0.00495521	0	0	0.5	0	RP11-347C1:	--	chr8	sense_intronic
ENSG00000278493.1	2.02505507	-1.5706682	0.00173462	0.0053947	0.21	0.11	1.13	0.69	CTD-2382E5	--	chr15	sense_intronic
ENSG00000123684.11	1.93052751	2.68094807	1.18E-09	1.02E-08	0.24	0.25	0.88	1.65	LPGAT1	lysophosphatidylglycerol acyltransferase 1	chr1	protein_coding
ENSG00000180667.9	2.12875884	-0.2726215	6.13E-06	3.10E-05	0.02	0.05	0.08	0.32	YOD1	YOD1 deubiquitinase	chr1	protein_coding
ENSG00000178917.13	1.04111646	0.31971962	0.00714234	0.01923994	0.37	0.26	0.62	1.1	ZNF852	zinc finger protein 852	chr3	protein_coding
ENSG00000162174.11	3.02387912	3.51646979	1.22E-20	3.07E-19	0.98	0.74	9.43	9.27	ASRGL1	asparaginase like 1	chr11	protein_coding
ENSG00000272940.1	2.40484614	-1.2972417	0.00010901	0.00043503	0	0.38	1.29	1.4	CTA-384D8.:	--	chr22	lincRNA
ENSG00000101052.11	1.77949132	3.26172972	7.32E-09	5.72E-08	1.63	2.32	7.91	10.18	IFT52	intraflagellar transport 52	chr20	protein_coding
ENSG00000136044.10	1.30538726	2.63724078	2.45E-05	0.0001109	0.81	0.65	1.99	2.84	APPL2	adaptor protein, phosphotyrosine interaction, PH domain and leucine zipper containing 2	chr12	protein_coding
ENSG00000184500.13	3.25596053	3.47247604	5.83E-23	1.73E-21	0.4	0.48	4.44	6.89	PROS1	protein S (alpha)	chr3	protein_coding
ENSG00000124198.8	1.5712837	3.51828461	2.12E-07	1.36E-06	0.49	0.56	1.82	2.34	ARFGEF2	ADP-ribosylation factor guanine nucleotide-exchange factor 2 (brefeldin A-inhibited)	chr20	protein_coding
ENSG00000171812.9	2.60543408	-0.806026	1.47E-06	8.23E-06	0.03	0.01	0.25	0.14	COL8A2	collagen, type VIII, alpha 2	chr1	protein_coding
ENSG00000091844.6	-2.8740726	-2.1193489	0.0002605	0.00096399	0.04	0.03	0	0.01	RGS17	regulator of G-protein signaling 17	chr6	protein_coding
ENSG00000156735.9	1.15287535	1.16244413	0.00080872	0.0027198	0.11	0.37	0.52	0.92	BAG4	BCL2-associated athanogene 4	chr8	protein_coding
ENSG00000177692.10	5.65942674	-2.6188299	0.00086564	0.00288128	0	0	0.01	0.14	DNAJC28	DnaJ (Hsp40) homolog, subfamily C, member 28	chr21	protein_coding
ENSG00000272155.1	3.02940024	-2.6558379	0.00943894	0.02469931	0	0.06	0.72	0.12	RP11-707M3	--	chr8	antisense
ENSG00000107554.14	-1.112444	5.18344999	0.0001275	0.00050221	5.75	6.58	3.94	3.64	DNMBP	dynamin binding protein	chr10	protein_coding
ENSG00000157064.9	-2.4030316	0.48131731	9.11E-10	8.02E-09	0.32	0.32	0.07	0.09	NMNAT2	nicotinamide nucleotide adenylyltransferase 2	chr1	protein_coding
ENSG00000278730.1	1.63099657	2.11220315	4.39E-07	2.68E-06	0.49	0.65	1.92	2.82	RP11-147L1:	--	chr17	lincRNA
ENSG00000237499.5	2.61807982	0.46498745	2.64E-10	2.49E-09	0.08	0.06	0.54	0.63	RP11-356I2.4	--	chr6	antisense
ENSG00000253507.4	-3.6914353	-2.5734304	0.00190233	0.00584723	0.33	0.06	0	0.03	CTD-2501M5	--	chr8	lincRNA
ENSG00000117602.10	1.09266761	2.5063197	0.00041126	0.00146184	0.39	0.42	1.24	1.09	RCAN3	RCAN family member 3	chr1	protein_coding
ENSG00000260498.4	-2.8619918	-2.1147541	0.00094481	0.00312013	0.56	0.35	0.17	0	RP4-536B24.	--	chr16	lincRNA
ENSG00000100354.19	1.51683904	3.84152122	4.06E-07	2.49E-06	0.33	0.31	1.14	1.28	TNRC6B	trinucleotide repeat containing 6B	chr22	protein_coding
ENSG00000269476.1	6.26658971	-2.2093414	1.55E-05	7.26E-05	0	0	0.35	0.27	CTD-2583A1	--	chr19	protein_coding
ENSG00000120949.13	1.3929634	-1.1671298	0.01291344	0.03239781	0.04	0.05	0.14	0.17	TNFRSF8	tumor necrosis factor receptor superfamily, member 8	chr1	protein_coding
ENSG00000183049.11	-1.6788511	4.93419871	1.29E-08	9.70E-08	4.12	4.63	2.01	1.62	CAMK1D	calcium/calmodulin-dependent protein kinase ID	chr10	protein_coding
ENSG00000233221.5	-1.0899881	-0.1646821	0.00840575	0.02231973	0.29	0.26	0.14	0.2	AC133785.1	--	chr2	antisense
ENSG00000059804.14	-7.9298321	3.14849282	6.10E-58	1.34E-55	3.86	3.45	0.04	0	SLC2A3	solute carrier family 2 (facilitated glucose transporter), member 3	chr12	protein_coding
ENSG00000112425.12	2.49417629	0.60587659	5.92E-10	5.33E-09	0.04	0.11	0.48	0.69	EPM2A	epilepsy, progressive myoclonus type 2A, Lafora disease (laforin)	chr6	protein_coding
ENSG00000226007.2	2.63227131	-0.7740945	2.69E-06	1.45E-05	0	0.1	0.36	0.47	RP11-211N8	--	chr9	lincRNA
ENSG00000185946.14	1.11447244	0.24804725	0.00392442	0.01127202	0.1	0.19	0.28	0.56	RNPC3	RNA-binding region (RNP1, RRM) containing 3	chr1	protein_coding
ENSG00000115053.14	-1.0491349	9.19218612	0.00025576	0.00094951	135.35	181.71	89.25	115.28	NCL	nucleolin	chr2	protein_coding
ENSG00000152443.11	2.08539205	1.13919347	1.10E-08	8.38E-08	0.12	0.13	0.49	0.89	ZNF776	zinc finger protein 776	chr19	protein_coding
ENSG00000100253.11	-5.894083	-2.6363745	0.00026535	0.0009747	0.1	0.09	0	0	MIOX	myo-inositol oxygenase	chr22	protein_coding
ENSG00000047936.9	2.64054131	-1.1269345	9.95E-06	4.83E-05	0.01	0.01	0.1	0.09	ROS1	ROS proto-oncogene 1 , receptor tyrosine kinase	chr6	protein_coding
ENSG00000056277.14	1.81599537	-1.5261896	0.00494993	0.0139272	0	0.04	0.06	0.14	ZNF280C	zinc finger protein 280C	chrX	protein_coding
ENSG00000124177.13	2.20881138	4.48688346	3.71E-13	4.85E-12	0.47	0.58	3.44	3.03	CHD6	chromodomain helicase DNA binding protein 6	chr20	protein_coding
ENSG00000151239.12	3.33873997	2.03474388	3.87E-20	9.40E-19	0.27	0.09	1.03	4.02	TWF1	twinfilin actin binding protein 1	chr12	protein_coding
ENSG00000135535.13	1.00942407	3.44245132	0.0007248	0.00245755	1.75	1.75	3.05	6.42	CD164	CD164 molecule, sialomucin	chr6	protein_coding
ENSG00000250584.2	5.37402558	-2.7967694	0.00158008	0.00495521	0	0	0.22	0	LINC01511	long intergenic non-protein coding RNA 1511	chr5	lincRNA
ENSG00000099625.11	-7.8653393	3.07273589	5.97E-58	1.32E-55	3.14	3.11	0.02	0.01	C19orf26	chromosome 19 open reading frame 26	chr19	protein_coding
ENSG00000114547.8	5.19748351	-2.8969879	0.00538165	0.01488851	0	0	0.02	0.07	ROPN1B	rhophilin associated tail protein 1B	chr3	protein_coding
ENSG00000204308.7	-1.0485508	5.94454015	0.00028094	0.00102837	59.93	55.08	39.38	34.6	RNF5	ring finger protein 5, E3 ubiquitin protein ligase	chr6	protein_coding
ENSG00000279691.1	-1.4727845	-0.5394927	0.00112717	0.00366331	4.35	3.21	1.74	1.88	RP11-485M7	--	chr5	TEC
ENSG00000136305.10	6.50526211	3.27266639	8.71E-51	1.21E-48	0.07	0.05	7.43	6.73	CIDEB	cell death-inducing DFFA-like effector b	chr14	protein_coding
ENSG00000129226.12	-1.0617496	0.78920029	0.00225787	0.00686177	1.25	0.71	0.41	0.84	CD68	CD68 molecule	chr17	protein_coding
ENSG00000237248.4	5.25966968	-0.2548939	1.76E-15	2.94E-14	0	0.02	0.54	0.72	LINC00987	long intergenic non-protein coding RNA 987	chr12	lincRNA
ENSG00000143867.6	-2.7874954	-0.7677434	2.40E-07	1.52E-06	0.48	0.32	0.1	0.06	OSR1	odd-skipped related transciption factor 1	chr2	protein_coding
ENSG00000198961.8	1.61955302	2.0333529	6.76E-07	4.00E-06	0.26	0.39	1.09	1.58	PJA2	praja ring finger 2, E3 ubiquitin protein ligase	chr5	protein_coding
ENSG00000126249.6	-2.7585634	3.80089633	2.28E-18	4.85E-17	16.78	14.03	3.39	2.67	PDCD2L	programmed cell death 2-like	chr19	protein_coding
ENSG00000276984.1	5.04680446	-2.9799228	0.0100455	0.02582742	0	0	0.11	0.72	LA16c-359F1	--	chr16	lincRNA
ENSG00000119630.12	1.85487296	-0.4914292	6.67E-05	0.00027839	0.15	0.05	0.58	0.42	PGF	placental growth factor	chr14	protein_coding
ENSG00000184445.10	2.97519932	2.61948296	1.19E-18	2.59E-17	0.17	0.11	1.14	1.88	KNTC1	kinetochore associated 1	chr12	protein_coding
ENSG00000273335.1	2.32840702	-1.3566278	0.00015394	0.00059366	0	0.29	1.42	0.56	RP11-61L19.	--	chr18	lincRNA
ENSG00000244588.4	5.87712165	-2.4793333	0.00014842	0.00057423	0	0	0.12	0.1	RAD21L1	RAD21-like 1 (S. pombe)	chr20	protein_coding
ENSG00000278192.1	-2.719571	-2.2246936	0.00108225	0.0035312	0.17	0.07	0	0.04	RP5-1056H1	--	chr20	lincRNA
ENSG00000173451.5	-1.1975194	0.95666647	0.00070036	0.00238244	0.36	0.32	0.25	0.14	THAP2	THAP domain containing, apoptosis associated protein 2	chr12	protein_coding
ENSG00000166974.11	-1.1975744	3.32925767	6.53E-05	0.00027327	1.89	2.29	1.13	1.3	MAPRE2	microtubule-associated protein, RP/EB family, member 2	chr18	protein_coding
ENSG00000077942.16	1.02807764	11.3451986	0.00033533	0.00121207	283.08	279.4	794.87	731.69	FBLN1	fibulin 1	chr22	protein_coding
ENSG00000174827.12	2.37176868	-2.4722048	0.01191236	0.03011315	0.02	0	0.11	0.07	PDZK1	PDZ domain containing 1	chr1	protein_coding
ENSG00000182197.9	-1.0027937	5.25267318	0.00053416	0.00185821	5.2	4.75	3.33	3.28	EXT1	exostosin glycosyltransferase 1	chr8	protein_coding
ENSG00000177150.11	1.28606597	1.81664649	8.16E-05	0.00033542	0.28	0.46	1.06	1.35	FAM210A	family with sequence similarity 210, member A	chr18	protein_coding
ENSG00000140750.15	-1.1711931	5.83551829	5.22E-05	0.000222	18.19	18.8	10.64	11.25	ARHGAP17	Rho GTPase activating protein 17	chr16	protein_coding
ENSG00000165915.12	-2.2655268	6.13192119	3.37E-14	4.93E-13	21.46	21.6	6.26	5.66	SLC39A13	solute carrier family 39 (zinc transporter), member 13	chr11	protein_coding
ENSG00000198753.10	-2.3427952	2.81745542	2.38E-13	3.18E-12	1.6	1.38	0.39	0.4	PLXNB3	plexin B3	chrX	protein_coding
ENSG00000232586.1	1.29365916	-0.1467458	0.00259752	0.00780494	0.24	0.3	0.73	1.02	KIAA1614-AS	KIAA1614 antisense RNA 1	chr1	antisense
ENSG00000097046.11	2.4101417	2.54255261	2.03E-13	2.73E-12	0.36	0.51	2.22	3.98	CDC7	cell division cycle 7	chr1	protein_coding
ENSG00000153130.16	2.36737042	1.24623154	8.53E-11	8.59E-10	0.07	0.12	0.35	0.96	SCOC	short coiled-coil protein	chr4	protein_coding
ENSG00000145194.16	-1.1703937	3.73690702	8.22E-05	0.0003377	3.33	2.4	1.89	1.5	ECE2	endothelin converting enzyme 2	chr3	protein_coding
ENSG00000276259.1	6.74809254	-1.8435441	2.24E-07	1.42E-06	0	0	0.29	0.69	RP11-481J2.4	--	chr16	antisense
ENSG00000137720.6	-1.1925086	0.96770596	0.00053127	0.00184868	0.5	0.64	0.25	0.41	C11orf1	chromosome 11 open reading frame 1	chr11	protein_coding
ENSG00000198125.11	-1.7523946	1.60750288	1.75E-07	1.13E-06	2.25	2.35	1.19	0.63	MB	myoglobin	chr22	protein_coding
ENSG00000134864.9	-1.2084206	1.53196461	0.0002586	0.00095843	0.9	1.37	0.69	0.62	GGACT	gamma-glutamylamine cyclotransferase	chr13	protein_coding
ENSG00000275709.1	2.87577227	-2.7430732	0.01615662	0.03958166	0.05	0	0.18	0.4	RP11-96O20	--	chr15	lincRNA
ENSG00000032742.16	1.86757718	-0.9541348	0.00055548	0.00192788	0.07	0.03	0.21	0.26	IFT88	intraflagellar transport 88	chr13	protein_coding
ENSG00000175785.11	8.61834386	-0.2537866	8.09E-18	1.64E-16	0	0	0.44	0.38	PRIMA1	proline rich membrane anchor 1	chr14	protein_coding
ENSG00000168070.10	10.0920945	1.12198765	2.89E-33	1.56E-31	0	0	3.88	3.1	C11orf85	chromosome 11 open reading frame 85	chr11	protein_coding
ENSG00000001497.15	-1.55338	8.36276601	9.02E-08	6.08E-07	149.33	176.95	75.53	72.36	LAS1L	LAS1-like (S. cerevisiae)	chrX	protein_coding
ENSG00000177683.12	2.42048841	1.00210301	1.47E-10	1.44E-09	0.15	0.11	0.42	1.46	THAP5	THAP domain containing 5	chr7	protein_coding
ENSG00000099795.5	-1.4586896	7.75386894	4.92E-07	2.98E-06	498.34	466.51	248.84	218.16	NDUFB7	NADH dehydrogenase (ubiquinone) 1 beta subcomplex, 7, 18kDa	chr19	protein_coding
ENSG00000277602.1	-1.6310863	-1.0204617	0.00166616	0.00519662	2.32	2.23	0.79	1.13	AC005363.11	--	chr16	lincRNA
ENSG00000131584.17	-1.6847693	5.88934554	9.20E-09	7.08E-08	19.85	17.7	7.32	8.28	ACAP3	ArfGAP with coiled-coil, ankyrin repeat and PH domains 3	chr1	protein_coding
ENSG00000116661.9	6.41660953	7.00019161	1.25E-68	4.69E-66	1.59	1.47	172.65	177.06	FBXO2	F-box protein 2	chr1	protein_coding
ENSG00000120992.16	1.02547998	0.96027247	0.0035729	0.01034915	0.31	0.41	0.48	1.45	LYPLA1	lysophospholipase I	chr8	protein_coding
ENSG00000198000.10	2.26110664	2.44353594	5.03E-12	5.84E-11	0.3	0.32	1.38	2.58	NOL8	nucleolar protein 8	chr9	protein_coding
ENSG00000136045.10	-1.1026084	5.91910123	0.00013559	0.00053109	24.53	24.66	13.89	16.7	PWP1	PWP1 homolog (S. cerevisiae)	chr12	protein_coding
ENSG00000100078.3	-7.1842364	-1.6819771	4.48E-09	3.59E-08	0.09	0.24	0	0	PLA2G3	phospholipase A2, group III	chr22	protein_coding
ENSG00000066322.11	-1.1631926	5.84797971	5.86E-05	0.00024684	37.42	34.72	21.97	20.93	ELOVL1	ELOVL fatty acid elongase 1	chr1	protein_coding
ENSG00000135722.7	1.09508773	2.59115193	0.00038818	0.00138707	0.94	1.46	3.41	3.39	FBXL8	F-box and leucine-rich repeat protein 8	chr16	protein_coding
ENSG00000163602.9	3.08358074	2.2869282	9.08E-19	1.99E-17	0.12	0.09	0.98	1.48	RYBP	RING1 and YY1 binding protein	chr3	protein_coding
ENSG00000155304.5	2.72974585	1.04716611	1.71E-12	2.10E-11	0.07	0.14	0.8	1.05	HSPA13	heat shock protein 70kDa family, member 13	chr21	protein_coding
ENSG00000235499.1	5.81117022	-2.5254325	0.00026535	0.0009747	0	0	1.07	0.14	AC073046.25	--	chr2	lincRNA
ENSG00000072832.13	-10.137885	4.66032322	4.94E-83	3.82E-80	10.39	10.25	0.02	0	CRMP1	collapsin response mediator protein 1	chr4	protein_coding
ENSG00000116095.9	1.34466759	1.07955609	0.00013338	0.0005234	0.05	0.09	0.18	0.27	PLEKHA3	pleckstrin homology domain containing, family A (phosphoinositide binding specific) member 3	chr2	protein_coding
ENSG00000182545.6	1.87480854	2.2481127	7.45E-09	5.81E-08	1.25	1.44	6.67	6.47	RNASE10	ribonuclease, RNase A family, 10 (non-active)	chr14	protein_coding
ENSG00000249709.6	1.26497979	0.13842639	0.00169431	0.00527824	0.07	0.25	0.35	0.65	ZNF564	zinc finger protein 564	chr19	protein_coding
ENSG00000134780.8	-1.4779454	3.04137413	1.24E-06	7.03E-06	1.31	2.1	0.83	0.79	DAGLA	diacylglycerol lipase, alpha	chr11	protein_coding
ENSG00000204568.10	-1.8385663	6.97400779	3.83E-10	3.55E-09	106.82	100.78	38.12	39.27	MRPS18B	mitochondrial ribosomal protein S18B	chr6	protein_coding
ENSG00000229124.5	-5.362193	-1.3262572	5.48E-10	4.95E-09	0.34	0.24	0.02	0	VIM-AS1	VIM antisense RNA 1	chr10	antisense
ENSG00000249790.2	2.70876814	-2.26271	0.00281991	0.00840887	0	0.03	0.1	0.17	RP11-20D14	--	chr12	lincRNA
ENSG00000184792.14	-2.1383514	7.31871715	5.47E-13	7.05E-12	40.07	38.47	12.48	11.26	OSBP2	oxysterol binding protein 2	chr22	protein_coding
ENSG00000272510.1	5.45974547	-2.7440718	0.00158008	0.00495521	0	0	0.37	0.63	RP4-680D5.8	--	chr1	antisense
ENSG00000242265.4	-6.1213954	2.42874445	7.71E-44	7.36E-42	1.14	1.47	0.02	0.03	PEG10	paternally expressed 10	chr7	protein_coding
ENSG00000006756.14	7.36343777	1.70092605	3.41E-37	2.20E-35	0	0.01	1.36	1.24	ARSD	arylsulfatase D	chrX	protein_coding
ENSG00000131115.14	1.78802461	2.27571691	3.10E-08	2.21E-07	0.53	0.57	1.69	3.43	ZNF227	zinc finger protein 227	chr19	protein_coding
ENSG00000205363.5	7.36512689	3.36329562	2.63E-56	5.48E-54	0.03	0	3.23	3.41	C15orf59	chromosome 15 open reading frame 59	chr15	protein_coding
ENSG00000171223.5	-1.2244915	8.29762104	2.15E-05	9.80E-05	206.87	184.99	120.74	102.3	JUNB	jun B proto-oncogene	chr19	protein_coding
ENSG00000198040.9	2.76815445	0.93010705	1.78E-12	2.18E-11	0.06	0.03	0.34	0.54	ZNF84	zinc finger protein 84	chr12	protein_coding
ENSG00000110344.8	2.11719836	3.1021443	1.68E-11	1.84E-10	0.3	0.48	1.72	2.82	UBE4A	ubiquitination factor E4A	chr11	protein_coding
ENSG00000105701.14	-1.4930495	8.75250204	2.64E-07	1.66E-06	280.04	248.41	132.63	117.21	FKBP8	FK506 binding protein 8, 38kDa	chr19	protein_coding
ENSG00000231560.1	3.18706495	-1.1897481	1.55E-06	8.67E-06	0.02	0.01	0.23	0.13	AC091814.3	--	chr12	lincRNA
ENSG00000101745.14	4.04498406	2.30370319	5.70E-27	2.26E-25	0.03	0.05	0.32	1.37	ANKRD12	ankyrin repeat domain 12	chr18	protein_coding
ENSG00000058600.14	-1.465577	6.3931899	4.71E-07	2.86E-06	25.77	27.8	12.3	13.56	POLR3E	polymerase (RNA) III (DNA directed) polypeptide E (80kD)	chr16	protein_coding
ENSG00000010626.13	-1.4032005	3.27134536	3.35E-06	1.78E-05	4.68	4.31	2.28	2.25	LRRC23	leucine rich repeat containing 23	chr12	protein_coding

ENSG00000155158.19	1.32775234	-0.694467	0.0074044	0.01985438	0.01	0.04	0.03	0.12	TTC39B	tetratricopeptide repeat domain 39B	chr9	protein_coding
ENSG00000153975.8	1.39823478	0.06766034	0.00059739	0.00206259	0.07	0.32	0.48	0.89	ZUFSP	zinc finger with UFM1-specific peptidase domain	chr6	protein_coding
ENSG00000249731.1	2.26965698	-1.4015052	0.00030738	0.0011156	0.29	0	1.03	0.92	RP11-259O2	--	chr5	lincRNA
ENSG00000111678.9	-1.9050743	6.95565085	9.62E-11	9.65E-10	140.11	102.05	45.94	40.18	C12orf57	chromosome 12 open reading frame 57	chr12	protein_coding
ENSG00000281183.1	5.32944093	-2.821228	0.00290522	0.00856312	0	0	0.03	0.11	NPTN-IT1	NPTN intronic transcript 1	chr15	sense_intronic
ENSG00000164066.11	2.19555074	-0.2312383	2.52E-06	1.36E-05	0.02	0.01	0.09	0.11	INTU	inturned planar cell polarity protein	chr4	protein_coding
ENSG00000183474.14	1.20239342	0.373544	0.00173632	0.00539789	0.08	0.28	0.5	0.58	GTF2H2C	GTF2H2 family member C	chr5	protein_coding
ENSG00000157890.16	-7.698587	-1.2399188	6.01E-12	6.93E-11	0.11	0.09	0	0	MEGF11	multiple EGF-like-domains 11	chr15	protein_coding
ENSG00000005893.14	1.39101035	4.54670081	2.38E-06	1.29E-05	1.9	1.86	4.68	8.57	LAMP2	lysosomal-associated membrane protein 2	chrX	protein_coding
ENSG00000205423.10	1.59486535	1.27710012	4.19E-06	2.19E-05	0.27	0.34	1	1.44	CNEP1R1	CTD nuclear envelope phosphatase 1 regulatory subunit 1	chr16	protein_coding
ENSG00000115419.11	2.47427109	2.75896765	2.54E-14	3.79E-13	0.19	0.23	0.95	2.21	GLS	glutaminase	chr2	protein_coding
ENSG00000235106.7	-1.6802572	5.25993599	1.13E-08	8.61E-08	7.58	8.2	3.16	3.4	LINC00094	long intergenic non-protein coding RNA 94	chr9	antisense
ENSG00000122406.11	-1.0749847	9.67897731	0.00018018	0.00068558	790.47	936.92	463.1	632.93	RPL5	ribosomal protein L5	chr1	protein_coding
ENSG00000137275.12	1.3713987	3.69250922	4.60E-06	2.39E-05	1.22	1.57	4.53	5.08	RIPK1	receptor (TNFRSF)-interacting serine-threonine kinase 1	chr6	protein_coding
ENSG00000258984.4	-1.4276022	1.8469975	1.05E-05	5.10E-05	4.13	4.74	1.95	2.43	UBE2F-SCLY	UBE2F-SCLY readthrough (NMD candidate)	chr2	protein_coding
ENSG00000257038.1	1.15983023	-0.2825081	0.00844515	0.02240948	0.14	0.3	0.62	0.69	RP11-800A3	--	chr11	antisense
ENSG00000280310.1	-3.5834524	-2.6392635	0.003237	0.00945156	0.1	0.22	0	0.03	RP11-326I19	--	chr4	TEC
ENSG00000166669.12	2.77910848	0.06329533	9.12E-10	8.02E-09	0.06	0.04	0.18	0.78	ATF7IP2	activating transcription factor 7 interacting protein 2	chr16	protein_coding
ENSG00000263718.2	7.18448191	-1.492238	2.80E-09	2.31E-08	0	0	0.38	1.16	RP11-285E9.	--	chr17	lincRNA
ENSG00000184524.5	-7.0383449	-1.8002674	1.87E-08	1.38E-07	0.24	0.24	0	0	CEND1	cell cycle exit and neuronal differentiation 1	chr11	protein_coding
ENSG00000102805.12	1.33860616	0.82047478	0.00019994	0.00075551	0.22	0.16	0.5	0.78	CLN5	ceroid-lipofuscinosis, neuronal 5	chr13	protein_coding
ENSG00000092978.9	2.01518897	1.19593624	1.86E-08	1.37E-07	0.09	0.09	0.37	0.59	GPATCH2	G patch domain containing 2	chr1	protein_coding
ENSG00000090776.5	-1.0306637	4.64345597	0.00040308	0.00143405	8.15	8.23	5.27	5.42	EFNB1	ephrin-B1	chrX	protein_coding
ENSG00000006740.15	-9.6650556	0.6205211	1.81E-29	8.14E-28	0.71	0.4	0	0	ARHGAP44	Rho GTPase activating protein 44	chr17	protein_coding
ENSG00000276564.1	5.19748351	-2.8969879	0.00538165	0.01488851	0	0	0.03	0.09	CTA-276F8.1	--	chr16	lincRNA
ENSG00000218739.8	1.20958717	2.6698114	8.76E-05	0.0003551	0.85	0.73	2.19	2.71	CEBPZOS	CEBPZ opposite strand	chr2	protein_coding
ENSG00000130313.5	-1.2816407	5.61279792	1.03E-05	4.99E-05	63.61	45.38	30.6	29.19	PGLS	6-phosphogluconolactonase	chr19	protein_coding
ENSG00000082014.15	1.11370759	5.06415119	0.00012824	0.00050488	7.04	6.41	21.26	17.45	SMARCD3	SWI/SNF related, matrix associated, actin dependent regulator of chromatin, subfamily d, member 3	chr7	protein_coding
ENSG00000223652.2	1.34484043	-0.5542121	0.00377991	0.01089087	0.24	0.31	0.88	0.96	AC106786.1	--	chr5	antisense
ENSG00000023516.8	3.77910976	0.32967675	5.08E-15	8.13E-14	0.01	0.01	0.15	0.32	AKAP11	A kinase (PRKA) anchor protein 11	chr13	protein_coding
ENSG00000181315.9	2.11206372	0.8610803	1.91E-08	1.40E-07	0.16	0.04	0.39	0.81	ZNF322	zinc finger protein 322	chr6	protein_coding
ENSG00000167656.4	4.23775973	1.32985265	5.64E-23	1.68E-21	0.2	0.28	6.37	5.86	LY6D	lymphocyte antigen 6 complex, locus D	chr8	protein_coding
ENSG00000163520.12	-4.2885973	-0.152039	2.78E-15	4.56E-14	0.41	0.16	0.03	0.01	FBLN2	fibulin 2	chr3	protein_coding
ENSG00000205744.8	-8.0387361	-0.9367973	3.62E-14	5.28E-13	0.17	0.4	0	0	DENND1C	DENN/MADD domain containing 1C	chr19	protein_coding
ENSG00000262420.3	2.81215119	-2.2000281	0.00108225	0.0035312	0.02	0	0.15	0.07	RP11-490O6	--	chr16	antisense
ENSG00000162402.11	1.16575538	3.35099575	0.00010593	0.00042485	0.57	0.39	1.35	1.52	USP24	ubiquitin specific peptidase 24	chr1	protein_coding
ENSG00000174442.10	1.57976701	2.03469871	1.22E-06	6.90E-06	0.2	0.6	0.97	2.24	ZWILCH	zwilch kinetochore protein	chr15	protein_coding
ENSG00000181790.9	7.45285177	-1.2775735	7.65E-11	7.77E-10	0	0	0.13	0.1	BAI1	--	chr8	protein_coding
ENSG00000280069.1	5.22761104	-2.8805399	0.00290522	0.00856312	0	0	0.12	0.03	CTD-2349P2	--	chr17	TEC
ENSG00000258588.3	3.10266834	-1.5709932	4.58E-05	0.00019809	0	0.03	0.08	0.22	TRIM6-TRIM	TRIM6-TRIM34 readthrough	chr11	protein_coding
ENSG00000158092.5	1.80062529	3.11600711	6.14E-09	4.86E-08	0.92	1.3	3.75	6.59	NCK1	NCK adaptor protein 1	chr3	protein_coding
ENSG00000113555.5	7.42159991	-1.2975784	1.18E-10	1.17E-09	0	0	0.07	0.14	PCDH12	protocadherin 12	chr5	protein_coding
ENSG00000164989.14	4.13979408	-1.2125842	6.30E-08	4.35E-07	0	0.01	0.06	0.09	CCDC171	coiled-coil domain containing 171	chr9	protein_coding
ENSG00000152556.14	-1.2763959	6.99738472	1.01E-05	4.88E-05	39.29	34.01	19.23	21.17	PFKM	phosphofructokinase, muscle	chr12	protein_coding
ENSG00000150672.15	1.93542426	-1.6242536	0.00486779	0.01371541	0	0.01	0.02	0.06	DLG2	discs, large homolog 2 (Drosophila)	chr11	protein_coding
ENSG00000135245.9	-1.1061817	4.41592243	0.00016083	0.00061835	19.67	14.16	10.18	10.81	HILPDA	hypoxia inducible lipid droplet-associated	chr7	protein_coding
ENSG00000144355.13	1.72693383	4.61229535	6.61E-09	5.21E-08	3.52	3.41	14.45	16.16	DLX1	distal-less homeobox 1	chr2	protein_coding
ENSG00000121690.8	2.53802485	1.31108644	5.39E-12	6.25E-11	0.37	0.14	1.28	2.75	DEPDC7	DEP domain containing 7	chr11	protein_coding
ENSG00000100558.7	-1.6874746	5.03076919	1.05E-08	8.06E-08	27.92	24.17	11	10.55	PLEK2	pleckstrin 2	chr14	protein_coding
ENSG00000175105.5	3.84875994	-0.1302679	1.50E-12	1.86E-11	0.01	0.03	0.15	0.5	ZNF654	zinc finger protein 654	chr3	protein_coding
ENSG00000179431.6	-3.5767996	4.1685046	5.68E-28	2.36E-26	10.71	11.24	1.07	1.38	FJX1	four jointed box 1 (Drosophila)	chr11	protein_coding
ENSG00000133818.11	1.50126638	3.66611537	6.06E-07	3.62E-06	1.68	2.13	6.06	8.36	RRAS2	related RAS viral (r-ras) oncogene homolog 2	chr11	protein_coding
ENSG00000232653.7	4.44727833	-1.6872995	6.30E-07	3.74E-06	0.01	0	0.09	0.09	GOLGA8N	golgin A8 family, member N	chr15	protein_coding
ENSG00000143248.11	2.28099126	-2.1473929	0.00354424	0.01027109	0.01	0	0.07	0.03	RGS5	regulator of G-protein signaling 5	chr1	protein_coding
ENSG00000090054.12	2.17505896	4.66082897	6.71E-13	8.56E-12	2.74	3.4	13.35	23.87	SPTLC1	serine palmitoyltransferase, long chain base subunit 1	chr9	protein_coding
ENSG00000250948.1	4.88946445	-3.0622981	0.01889873	0.04519557	0	0	0.26	0.67	RP11-1079K	--	chr17	antisense
ENSG00000271584.1	5.48900786	-2.7279255	0.00158008	0.00495521	0	0	0.63	0.11	RP11-89C3.4	--	chr11	lincRNA
ENSG00000228434.1	1.80402197	2.56178183	1.38E-08	1.04E-07	1.16	1.08	5.18	5.25	AC004951.6	--	chr7	lincRNA
ENSG00000127947.14	1.46994833	3.28925869	1.36E-06	7.64E-06	1.22	1.17	3.3	5.57	PTPN12	protein tyrosine phosphatase, non-receptor type 12	chr7	protein_coding
ENSG00000184347.13	-6.5082757	-2.21186	3.03E-06	1.62E-05	0.02	0.03	0	0	SLIT3	slit homolog 3 (Drosophila)	chr5	protein_coding
ENSG00000169857.6	-1.5939799	4.21603873	8.74E-08	5.90E-07	12.18	16	6.75	5.65	AVEN	apoptosis, caspase activation inhibitor	chr15	protein_coding
ENSG00000156463.16	-4.1459204	2.46485524	1.98E-29	8.88E-28	1.25	1.42	0.11	0.09	SH3RF2	SH3 domain containing ring finger 2	chr5	protein_coding
ENSG00000174945.12	-5.6119902	1.09801629	5.63E-29	2.47E-27	0.64	0.57	0.02	0.01	AMZ1	archaelysin family metallopeptidase 1	chr7	protein_coding
ENSG00000154274.13	3.1869694	-1.943355	0.00010192	0.00041007	0.02	0	0.13	0.14	C4orf19	chromosome 4 open reading frame 19	chr4	protein_coding
ENSG00000139842.13	-1.0460448	6.02443989	0.0002873	0.0010497	9.48	10.66	5.51	7.53	CUL4A	cullin 4A	chr13	protein_coding
ENSG00000166444.16	-2.2751081	5.05013314	4.72E-14	6.81E-13	7.01	7.46	2	1.98	ST5	suppression of tumorigenicity 5	chr11	protein_coding
ENSG00000152457.16	1.30287808	0.27004558	0.00082295	0.00276531	0.07	0.11	0.27	0.3	DCLRE1C	DNA cross-link repair 1C	chr10	protein_coding
ENSG00000174502.17	-7.9797222	1.13247616	1.29E-35	7.86E-34	0.65	0.83	0	0.01	SLC26A9	solute carrier family 26 (anion exchanger), member 9	chr1	protein_coding
ENSG00000214491.7	10.468776	1.48518858	5.97E-38	4.02E-36	0	0	1.62	1.78	SEC14L6	SEC14-like 6 (S. cerevisiae)	chr22	protein_coding
ENSG00000118156.11	5.27219449	2.19092537	9.50E-35	5.43E-33	0.06	0.01	1.98	2.06	ZNF541	zinc finger protein 541	chr19	protein_coding
ENSG00000157510.12	-1.4198135	5.88448858	1.08E-06	6.16E-06	15.3	18.06	8.1	8.51	AFAP1L1	actin filament associated protein 1-like 1	chr5	protein_coding
ENSG00000169213.6	-1.7093521	0.15316223	1.69E-05	7.88E-05	0.08	0.13	0.04	0.04	RAB3B	RAB3B, member RAS oncogene family	chr1	protein_coding
ENSG00000164488.10	4.89980841	-3.0567113	0.01889873	0.04519557	0	0	0.03	0.03	DACT2	dishevelled-binding antagonist of beta-catenin 2	chr6	protein_coding
ENSG00000144857.13	-4.3912564	2.13821892	3.07E-30	1.43E-28	1.12	1.13	0.02	0.12	BOC	BOC cell adhesion associated, oncogene regulated	chr3	protein_coding
ENSG00000168172.7	1.66001582	1.63438117	8.01E-07	4.68E-06	0.08	0.08	0.3	0.38	HOOK3	hook microtubule-tethering protein 3	chr8	protein_coding
ENSG00000095303.13	-2.782153	0.27013556	2.76E-11	2.95E-10	0.32	0.25	0.04	0.07	PTGS1	prostaglandin-endoperoxide synthase 1 (prostaglandin G/H synthase and cyclooxygenase)	chr9	protein_coding
ENSG00000107679.13	2.06133468	1.03054921	1.99E-08	1.46E-07	0.04	0.04	0.12	0.31	PLEKHA1	pleckstrin homology domain containing, family A (phosphoinositide binding specific) member 1	chr10	protein_coding
ENSG00000272825.1	2.10384933	0.73951669	3.48E-08	2.47E-07	0.9	0.57	5.6	2.88	LL21NC02-1	--	chr21	antisense
ENSG00000166341.7	-5.9955597	-2.569758	0.00014842	0.00057423	0.03	0.01	0	0	DCHS1	dachsous cadherin-related 1	chr11	protein_coding
ENSG00000177469.12	1.10216484	10.1945383	0.00012391	0.00048919	155.74	159.32	450.37	450.61	PTRF	polymerase I and transcript release factor	chr17	protein_coding
ENSG00000188010.11	1.19269715	3.00393372	8.68E-05	0.00035215	5.54	3.53	13.19	14.53	MORN2	MORN repeat containing 2	chr2	protein_coding
ENSG00000235750.8	-2.0036978	4.14208567	3.37E-11	3.55E-10	4.73	5.11	1.54	1.73	KIAA0040	KIAA0040	chr1	protein_coding
ENSG00000147601.12	1.2045642	1.84148239	0.00019532	0.00073889	0.43	0.6	0.9	2.28	TERF1	telomeric repeat binding factor (NIMA-interacting) 1	chr8	protein_coding
ENSG00000257950.3	-1.7596425	5.47430852	2.33E-09	1.94E-08	9.83	8.98	3.68	3.73	P2RX5-TAX1	P2RX5-TAX1BP3 readthrough (NMD candidate)	chr17	protein_coding
ENSG00000043093.12	6.51393512	1.41044552	2.79E-32	1.44E-30	0.01	0	0.34	1.01	DCUN1D1	DCN1, defective in cullin neddylation 1, domain containing 1	chr3	protein_coding
ENSG00000138613.12	3.80207232	1.93558978	3.02E-23	9.17E-22	0.11	0.08	1.64	1.92	APH1B	APH1B gamma secretase subunit	chr15	protein_coding
ENSG00000176919.10	1.58635986	-1.2649861	0.00491991	0.01384598	0.25	0.07	0.64	0.67	C8G	complement component 8, gamma polypeptide	chr9	protein_coding
ENSG00000101188.4	-3.3195409	-2.7893297	0.00943894	0.02469931	0.06	0	0	0.01	NTSR1	neurotensin receptor 1 (high affinity)	chr20	protein_coding
ENSG00000104213.11	-1.2214441	2.97441132	5.72E-05	0.00024148	5.08	4.22	2.45	2.87	PDGFRL	platelet-derived growth factor receptor-like	chr8	protein_coding
ENSG00000155903.10	3.71069281	-0.2517475	1.59E-11	1.75E-10	0	0.03	0.17	0.36	RASA2	RAS p21 protein activator 2	chr3	protein_coding
ENSG00000249430.1	8.11156092	-0.7081297	8.73E-14	1.22E-12	0	0	1.73	1.47	CTD-2231H1	--	chr5	lincRNA
ENSG00000124134.7	9.01971857	0.12114853	1.44E-21	3.94E-20	0	0	0.32	0.61	KCNS1	potassium voltage-gated channel, modifier subfamily S, member 1	chr20	protein_coding
ENSG00000225806.6	1.64667179	-1.6386878	0.01303785	0.03267232	0	0.13	0.27	0.27	RP1-309F20.	--	chr20	antisense
ENSG00000157851.15	1.40513459	3.1966683	3.76E-06	1.98E-05	0.81	0.69	2.81	2.5	DPYSL5	dihydropyrimidinase-like 5	chr2	protein_coding
ENSG00000167792.10	-1.0882651	9.01853217	0.00015106	0.0005835	388.56	313.62	228.72	211.25	NDUFV1	NADH dehydrogenase (ubiquinone) flavoprotein 1, 51kDa	chr11	protein_coding
ENSG00000115252.17	-9.46982	0.42667789	3.19E-27	1.28E-25	0.29	0.42	0	0	PDE1A	phosphodiesterase 1A, calmodulin-dependent	chr2	protein_coding
ENSG00000130396.19	1.39073918	3.34984124	4.32E-06	2.25E-05	0.46	0.46	1.49	1.73	MLLT4	myeloid/lymphoid or mixed-lineage leukemia (trithorax homolog, Drosophila); translocated to, 4	chr6	protein_coding
ENSG00000154001.12	1.95218917	2.84189554	5.59E-10	5.06E-09	0.28	0.42	1.45	2.14	PPP2R5E	protein phosphatase 2, regulatory subunit B', epsilon isoform	chr14	protein_coding
ENSG00000115944.13	-1.1511796	6.45333752	6.66E-05	0.00027812	30.52	27.31	15.77	19.02	COX7A2L	cytochrome c oxidase subunit VIIa polypeptide 2 like	chr2	protein_coding
ENSG00000159167.10	5.05654181	2.51799953	1.37E-35	8.32E-34	0.06	0.06	2.4	3.27	STC1	stanniocalcin 1	chr8	protein_coding
ENSG00000196834.10	-4.4377047	-2.0634194	1.13E-05	5.42E-05	0.05	0.04	0	0	POTEI	POTE ankyrin domain family, member I	chr2	protein_coding
ENSG00000103066.11	1.12808132	5.29729617	0.00010219	0.00041111	7.68	6.72	21.83	20.1	PLA2G15	phospholipase A2, group XV	chr16	protein_coding
ENSG00000082397.14	-6.0940654	-2.5041043	8.36E-05	0.00034052	0.06	0.02	0	0	EPB41L3	erythrocyte membrane protein band 4.1-like 3	chr18	protein_coding
ENSG00000137154.11	-2.3535287	10.7564926	2.92E-15	4.77E-14	913.55	941.7	225.2	259.26	RPS6	ribosomal protein S6	chr9	protein_coding
ENSG00000234719.7	2.76024977	0.13407659	1.03E-09	9.01E-09	0.07	0.15	1.02	0.93	RP11-166B2.	--	chr16	protein_coding
ENSG00000251169.2	-1.1323938	1.37128436	0.00082689	0.002777	3.38	1.72	1.91	1.21	AC005355.2	--	chr5	lincRNA
ENSG00000197405.6	-4.4739929	1.38523897	8.84E-26	3.21E-24	1.42	2.08	0.17	0.05	C5AR1	complement component 5a receptor 1	chr19	protein_coding
ENSG00000169439.10	-6.9755233	3.27499012	5.15E-56	1.05E-53	3.63	4.2	0.03	0.05	SDC2	syndecan 2	chr8	protein_coding
ENSG00000005469.10	3.10109088	0.7366673	8.63E-14	1.21E-12	0.08	0.05	0.52	0.92	CROT	carnitine O-octanoyltransferase	chr7	protein_coding
ENSG00000130822.14	-2.5240456	-1.09582	9.67E-06	4.71E-05	0.3	0.23	0.1	0.02	PNCK	pregnancy up-regulated nonubiquitous CaM kinase	chrX	protein_coding
ENSG00000129173.11	2.84473932	1.50492345	1.15E-14	1.78E-13	0.12	0.15	1.09	1.55	E2F8	E2F transcription factor 8	chr11	protein_coding
ENSG00000144908.12	-1.0284544	1.61796171	0.00156528	0.00495521	0.99	0.78	0.49	0.67	ALDH1L1	aldehyde dehydrogenase 1 family, member L1	chr3	protein_coding
ENSG00000158019.19	-1.0999692	4.34677604	0.00017774	0.00067756	10.79	11.76	7.52	6.47	BRE	brain and reproductive organ-expressed (TNFRSF1A modulator)	chr2	protein_coding
ENSG00000110075.13	1.52399349	3.36349825	5.21E-07	3.15E-06	0.76	0.87	2.72	3.56	PPP6R3	protein phosphatase 6, regulatory subunit 3	chr11	protein_coding
ENSG00000275413.1	4.88946445	-3.0622981	0.01889873	0.04519557	0	0	0.19	0.49	CTC-529I10.	--	chr17	antisense
ENSG00000172840.5	1.56031696	2.64870661	5.92E-07	3.54E-06	0.37	0.34	1.35	1.43	PDP2	pyruvate dehyrogenase phosphatase catalytic subunit 2	chr16	protein_coding
ENSG00000173588.13	3.09199979	0.22718105	9.96E-12	1.12E-10	0.04	0.03	0.15	0.63	CEP83	centrosomal protein 83kDa	chr12	protein_coding
ENSG00000120784.14	1.44729625	0.09469216	0.00037112	0.00133081	0.08	0.06	0.14	0.34	ZFP30	ZFP30 zinc finger protein	chr19	protein_coding
ENSG00000162894.10	-1.1798288	2.75406515	0.00011544	0.00045818	2.98	4.78	2.55	2	FAIM3	--	chr1	protein_coding
ENSG00000167447.11	1.55239422	4.2452411	1.84E-07	1.19E-06	2.01	2.13	7	9.23	SMG8	SMG8 nonsense mediated mRNA decay factor	chr17	protein_coding
ENSG00000162927.12	1.59239237	-1.0286103	0.00362278	0.01047843	0.02	0.08	0.1	0.3	PUS10	pseudouridylate synthase 10	chr2	protein_coding
ENSG00000251144.1	-11.360177	2.30794313	2.52E-51	3.68E-49	27.4	28.64	0	0	CTD-2532K1	--	chr5	lincRNA
ENSG00000069667.14	5.6781718	-2.6083292	0.00047761	0.00167191	0	0	0.01	0.02	RORA	RAR-related orphan receptor A	chr15	protein_coding
ENSG00000240137.4	2.05900471	-0.9306853	0.00016946	0.00064769	0.06	0.27	0.93	0.95	ERICH6-AS1	ERICH6 antisense RNA 1	chr3	antisense
ENSG00000112306.7	-1.9820136	9.62442346	1.64E-11	1.79E-10	1687.25	1543.99	498.66	594.09	RPS12	ribosomal protein S12	chr6	protein_coding
ENSG00000161016.14	-1.8423091	12.3486231	3.13E-10	2.93E-09	5791	4621.8	1920.46	1953.42	RPL8	ribosomal protein L8	chr8	protein_coding
ENSG00000175893.10	2.99347016	-0.4884684	2.73E-08	1.97E-07	0.01	0.01	0.09	0.17	ZDHHC21	zinc finger, DHHC-type containing 21	chr9	protein_coding
ENSG00000206538.6	5.45153848	0.41412135	3.36E-21	8.92E-20	0	0	0.17	0.28	VGLL3	vestigial-like family member 3	chr3	protein_coding
ENSG00000135837.14	4.38438601	2.08889356	2.27E-28	9.60E-27	0.02	0.02	0.54	0.72	CEP350	centrosomal protein 350kDa	chr1	protein_coding
ENSG00000096654.14	1.51564284	-1.0802845	0.00604922	0.01656143	0	0.11	0.15	0.25	ZNF184	zinc finger protein 184	chr6	protein_coding
ENSG00000271853.4	5.7784906	-2.5437546	0.00026535	0.0009747	0	0	0.26	0.33	RP1-178F15.	--	chr1	processed_transcript
ENSG00000104529.16	-1.6712551	11.1115891	9.61E-09	7.38E-08	716.51	723.65	306.48	295.61	EEF1D	eukaryotic translation elongation factor 1 delta (guanine nucleotide exchange protein)	chr8	protein_coding
ENSG00000247271.5	1.89736951	1.48930986	3.71E-08	2.62E-07	0.24	0.7	2.34	2.28	ZBED5-AS1	ZBED5 antisense RNA 1	chr11	antisense
ENSG00000189337.14	3.73193941	1.21656146	1.46E-19	3.40E-18	0.02	0.03	0.43	0.51	KAZN	kazrin, periplakin interacting protein	chr1	protein_coding
ENSG00000215012.7	1.31260386	5.58598541	6.47E-06	3.26E-05	3.47	3.08	10.75	10.93	C22orf29	chromosome 22 open reading frame 29	chr22	protein_coding
ENSG00000152767.14	-1.8781772	5.65969933	2.06E-10	1.97E-09	5.54	5.61	2.2	1.83	FARP1	FERM, RhoGEF (ARHGEF) and pleckstrin domain protein 1 (chondrocyte-derived)	chr13	protein_coding
ENSG00000134243.10	1.16754984	3.94912564	8.01E-05	0.00032967	1.09	0.99	3.01	3.22	SORT1	sortilin 1	chr1	protein_coding
ENSG00000267079.1	5.97464286	-2.4149021	8.36E-05	0.00034052	0	0	0.93	0.43	RP11-820I16	--	chr18	lincRNA
ENSG00000138658.14	3.42139272	-0.9899914	8.81E-08	5.94E-07	0	0.02	0.05	0.19	ZGRF1	zinc finger, GRF-type containing 1	chr4	protein_coding
ENSG00000255650.4	-6.324496	-0.4636828	3.56E-18	7.37E-17	1.13	0.98	0.03	0	FAM222A-A	FAM222A antisense RNA 1	chr12	antisense
ENSG00000272767.1	4.210503	0.55058728	4.50E-18	9.31E-17	0.08	0.08	2.28	1.9	JMJD1C-AS1	JMJD1C antisense RNA 1	chr10	antisense
ENSG00000070729.12	-1.640593	-0.6353456	0.00033978	0.00122705	0.12	0.11	0.01	0.09	CNGB1	cyclic nucleotide gated channel beta 1	chr16	protein_coding
ENSG00000273010.1	-8.9283866	-0.0996117	1.09E-21	3.00E-20	1.03	1.18	0	0	RP11-96K19.	--	chr1	antisense
ENSG00000213983.10	1.02447024	4.12618314	0.00049993	0.0017457	2.86	2.76	7.1	8.17	AP1G2	adaptor-related protein complex 1, gamma 2 subunit	chr14	protein_coding
ENSG00000170915.8	-5.0525462	0.29133553	1.15E-21	3.17E-20	0.43	0.36	0	0.03	PAQR8	progestin and adipoQ receptor family member VIII	chr6	protein_coding
ENSG00000165113.11	3.92841701	0.83997947	9.81E-19	2.15E-17	0.15	0.03	1.46	2.37	GKAP1	G kinase anchoring protein 1	chr9	protein_coding
ENSG00000121552.3	3.84560578	0.65204481	3.01E-17	5.80E-16	0.14	0.08	1.61	2.7	CSTA	cystatin A (stefin A)	chr3	protein_coding
ENSG00000145476.14	-1.3308921	-0.2795482	0.00230895	0.00699749	0.6	0.28	0.31	0.16	CYP4V2	cytochrome P450, family 4, subfamily V, polypeptide 2	chr4	protein_coding
ENSG00000124383.7	1.37618747	2.68004796	9.11E-06	4.45E-05	0.75	0.99	1.94	4.1	MPHOSPH10	M-phase phosphoprotein 10 (U3 small nucleolar ribonucleoprotein)	chr2	protein_coding
ENSG00000170442.10	-3.0871967	1.6403597	2.40E-17	4.68E-16	2.3	1.66	0.41	0.22	KRT86	keratin 86, type II	chr12	protein_coding
ENSG00000123689.5	-2.5532382	5.80563264	3.15E-17	6.06E-16	84.37	94.44	21.6	18.9	G0S2	G0/G1 switch 2	chr1	protein_coding
ENSG00000204257.13	2.00969145	0.98714578	4.05E-08	2.85E-07	0.43	0.66	3.37	2.45	HLA-DMA	major histocompatibility complex, class II, DM alpha	chr6	protein_coding
ENSG00000178821.11	-5.1793985	1.83999168	1.55E-33	8.50E-32	5.01	3.7	0.1	0.21	TMEM52	transmembrane protein 52	chr1	protein_coding
ENSG00000224536.1	-4.1475777	-1.6195461	6.31E-08	4.36E-07	1.42	1.17	0	0.16	RP11-134G8	--	chr1	antisense
ENSG00000186106.10	1.30159941	-0.9499749	0.01103277	0.0281449	0.05	0.05	0.13	0.19	ANKRD46	ankyrin repeat domain 46	chr8	protein_coding
ENSG00000150760.11	1.72600923	3.74680268	1.20E-08	9.04E-08	0.76	0.7	2.89	3.56	DOCK1	dedicator of cytokinesis 1	chr10	protein_coding
ENSG00000278341.1	-2.5239024	-1.5826887	0.00025672	0.00095219	0.22	0.33	0.13	0	RP5-1142A6	--	chr16	antisense
ENSG00000132507.16	-1.8470789	9.5665607	2.89E-10	2.72E-09	481.85	434.89	178.53	160.63	EIF5A	eukaryotic translation initiation factor 5A	chr17	protein_coding
ENSG00000274020.1	-3.6326385	0.82679391	4.68E-18	9.66E-17	1.16	1.23	0.14	0.11	LINC01138	long intergenic non-protein coding RNA 1138	chr1	lincRNA
ENSG00000152705.7	-2.7440737	-0.8371718	2.15E-07	1.38E-06	0.49	0.6	0	0.2	CATSPER3	cation channel, sperm associated 3	chr5	protein_coding
ENSG00000173559.11	1.32070936	3.8905207	8.96E-06	4.38E-05	0.53	0.62	1.87	1.97	NABP1	nucleic acid binding protein 1	chr2	protein_coding
ENSG00000142937.10	-1.684133	10.3997034	7.52E-09	5.86E-08	2007.28	1777.73	742.89	829.59	RPS8	ribosomal protein S8	chr1	protein_coding
ENSG00000213186.6	2.29889701	-1.3732538	0.00030738	0.0011156	0.02	0.01	0.03	0.13	TRIM59	tripartite motif containing 59	chr3	protein_coding
ENSG00000213995.10	-1.4522117	5.25087121	6.86E-07	4.06E-06	15.11	16.61	7.66	7.78	CARKD	carbohydrate kinase domain containing	chr13	protein_coding
ENSG00000127314.16	1.06934335	4.2248989	0.00027313	0.00100177	0.7	0.66	1.42	2.39	RAP1B	RAP1B, member of RAS oncogene family	chr12	protein_coding
ENSG00000275202.1	1.28000865	2.55444396	3.82E-05	0.00016698	2.11	2.86	8.52	7.52	RP11-156K2	--	chr13	antisense
ENSG00000124731.11	-7.1796837	-1.6845418	4.48E-09	3.59E-08	0.13	0.14	0	0	TREM1	triggering receptor expressed on myeloid cells 1	chr6	protein_coding
ENSG00000121621.6	3.09000674	-0.1243228	3.86E-10	3.58E-09	0.06	0.02	0.28	0.61	KIF18A	kinesin family member 18A	chr11	protein_coding
ENSG00000132646.9	1.16592202	6.20054998	5.52E-05	0.00023355	22.6	27.3	64.44	85.14	PCNA	proliferating cell nuclear antigen	chr20	protein_coding
ENSG00000089127.11	1.91148767	4.0486755	2.69E-10	2.54E-09	1.7	1.01	7.32	6.27	OAS1	2'-5'-oligoadenylate synthetase 1, 40/46kDa	chr12	protein_coding
ENSG00000147853.15	1.00610589	1.9080256	0.00174741	0.0054232	0.36	0.52	0.91	1.46	AK3	adenylate kinase 3	chr9	protein_coding
ENSG00000149474.12	1.01170031	3.66540227	0.00066434	0.00226891	1.9	1.71	4.65	5.04	CSRP2BP	CSRP2 binding protein	chr20	protein_coding
ENSG00000227268.3	1.84300653	-0.8587416	0.00045087	0.00158969	0.05	0.03	0.08	0.29	KLLN	killin, p53-regulated DNA replication inhibitor	chr10	protein_coding
ENSG00000207112.1	1.52293855	-1.5639724	0.01700209	0.04149604	0.6	1.06	0.96	5.28	SNORA25	small nucleolar RNA, H/ACA box 25	chr11	snoRNA
ENSG00000066427.20	1.32704113	2.32011328	2.50E-05	0.00011271	0.07	0.1	0.28	0.27	ATXN3	ataxin 3	chr14	protein_coding
ENSG00000273783.1	1.74611867	-0.2055085	7.00E-05	0.00029061	0.05	0.48	1.33	0.99	CTD-2506P8	--	chr14	antisense
ENSG00000172247.3	-2.7438578	-2.5676946	0.01191236	0.03011315	0	0.2	0.04	0	C1QTNF4	C1q and tumor necrosis factor related protein 4	chr11	protein_coding
ENSG00000271949.1	2.03807146	-0.6153209	3.75E-05	0.00016418	0.29	0.26	0.93	2.08	RP11-302M6	--	chr1	protein_coding
ENSG00000173762.6	5.47030895	3.17961597	3.56E-43	3.27E-41	0.16	0.09	7.61	7.59	CD7	CD7 molecule	chr17	protein_coding
ENSG00000023608.4	2.88148687	2.02214694	2.26E-16	4.05E-15	0.17	0.41	1.64	4.04	SNAPC1	small nuclear RNA activating complex, polypeptide 1, 43kDa	chr14	protein_coding
ENSG00000135390.16	-1.7807544	7.04000064	1.24E-09	1.06E-08	80.18	66.78	30.03	26.92	ATP5G2	ATP synthase, H+ transporting, mitochondrial Fo complex, subunit C2 (subunit 9)	chr12	protein_coding

ENSG00000213654.8	-3.3014014	-0.9929666	2.19E-08	1.59E-07	0.42	0.32	0.1	0	GPSM3	G-protein signaling modulator 3	chr6	protein_coding
ENSG00000167964.11	-1.8012976	2.94172752	6.36E-09	5.02E-08	6.11	4.26	2.19	1.78	RAB26	RAB26, member RAS oncogene family	chr16	protein_coding
ENSG00000152439.11	2.49557709	-1.2389254	3.89E-05	0.00016973	0.02	0	0.1	0.07	ZNF773	zinc finger protein 773	chr19	protein_coding
ENSG00000244754.7	1.83078958	2.3137486	1.33E-08	1.00E-07	0.1	0.19	0.44	0.95	N4BP2L2	NEDD4 binding protein 2-like 2	chr13	protein_coding
ENSG00000064225.11	4.42126848	1.04015688	2.75E-22	7.87E-21	0.05	0.01	0.79	1.07	ST3GAL6	ST3 beta-galactoside alpha-2,3-sialyltransferase 6	chr3	protein_coding
ENSG00000138660.10	1.7102993	0.5629845	7.26E-06	3.62E-05	0.17	0.2	0.4	1.18	AP1AR	adaptor-related protein complex 1 associated regulatory protein	chr4	protein_coding
ENSG00000235884.3	-8.6088306	1.76457017	7.44E-44	7.13E-42	2.85	3.86	0.02	0	LINC00941	long intergenic non-protein coding RNA 941	chr12	lincRNA
ENSG00000134668.11	-3.6264827	2.1792206	2.02E-23	6.22E-22	1.62	1.8	0.29	0.08	SPOCD1	SPOC domain containing 1	chr1	protein_coding
ENSG00000174640.11	-7.1976936	6.80366613	8.83E-79	5.43E-76	39.05	46.14	0.27	0.5	SLCO2A1	solute carrier organic anion transporter family, member 2A1	chr3	protein_coding
ENSG00000242732.4	3.88225096	2.53180315	2.31E-26	8.73E-25	0.11	0.17	2.54	3.04	RGAG4	retrotransposon gag domain containing 4	chrX	protein_coding
ENSG00000117707.14	6.49943546	-2.0356972	1.78E-06	9.85E-06	0	0	0.03	0.05	PROX1	prospero homeobox 1	chr1	protein_coding
ENSG00000197841.13	1.87690491	0.17971991	6.02E-06	3.04E-05	0.03	0.1	0.15	0.47	ZNF181	zinc finger protein 181	chr19	protein_coding
ENSG00000248092.6	1.30536941	4.34584482	9.49E-06	4.63E-05	1.43	1.44	4.69	4.76	NNT-AS1	NNT antisense RNA 1	chr5	antisense
ENSG00000196557.9	-4.6674355	-1.1810689	9.92E-10	8.71E-09	0.02	0.14	0.01	0	CACNA1H	calcium channel, voltage-dependent, T type, alpha 1H subunit	chr16	protein_coding
ENSG00000112739.15	2.73800743	2.82024213	9.23E-17	1.71E-15	0.26	0.4	2.12	3.82	PRPF4B	pre-mRNA processing factor 4B	chr6	protein_coding
ENSG00000176946.10	-1.1603462	7.06246854	5.70E-05	0.00024046	61.35	56.41	36.29	33.87	THAP4	THAP domain containing 4	chr2	protein_coding
ENSG00000268350.6	-1.2259606	1.33023222	0.00027241	0.00099926	0.59	0.78	0.43	0.35	FAM156A	family with sequence similarity 156, member A	chrX	protein_coding
ENSG00000273703.1	5.12408284	-0.3867272	1.83E-14	2.77E-13	0	0.15	5.63	1.86	HIST1H2BM	histone cluster 1, H2bm	chr6	protein_coding
ENSG00000276863.1	-3.1700013	-2.8637184	0.01615662	0.03958166	0.2	0.27	0.06	0	CTD-2047H1	--	chr17	antisense
ENSG00000183828.13	1.08506387	4.11610414	0.00023029	0.00086178	11.14	10.03	33.81	25.88	NUDT14	nudix (nucleoside diphosphate linked moiety X)-type motif 14	chr14	protein_coding
ENSG00000162976.11	1.01717205	1.48714367	0.00232879	0.00704781	0.92	0.67	1.8	2.5	PQLC3	PQ loop repeat containing 3	chr2	protein_coding
ENSG00000184357.4	3.86692284	0.89078916	2.14E-18	4.56E-17	0.12	0.42	7.29	3.08	HIST1H1B	histone cluster 1, H1b	chr6	protein_coding
ENSG00000168811.5	5.66409976	-2.616209	0.00086564	0.00288128	0	0	0.04	0.21	IL12A	interleukin 12A	chr3	protein_coding
ENSG00000276724.1	6.45521709	-2.0709636	3.03E-06	1.62E-05	0	0	0.55	0.47	RP11-1000B	--	chr15	sense_intronic
ENSG00000172795.14	4.56132731	0.25297988	1.06E-17	2.12E-16	0.01	0	0.21	0.29	DCP2	decapping mRNA 2	chr5	protein_coding
ENSG00000120645.10	-5.9996908	-2.5677248	0.00014842	0.00057423	0.02	0.03	0	0	IQSEC3	IQ motif and Sec7 domain 3	chr12	protein_coding
ENSG00000198720.11	-2.211648	4.96115968	2.20E-13	2.95E-12	13.33	12.47	3.95	3.47	ANKRD13B	ankyrin repeat domain 13B	chr17	protein_coding
ENSG00000100296.12	-1.1913406	6.98942764	3.64E-05	0.00015978	27.28	29.09	16.61	16.26	THOC5	THO complex 5	chr22	protein_coding
ENSG00000102119.9	-2.2836356	6.77874822	1.94E-14	2.92E-13	109.79	104.07	29.15	29.38	EMD	emerin	chrX	protein_coding
ENSG00000134313.13	1.81263957	3.22080923	4.21E-09	3.41E-08	0.39	0.44	1.53	2.37	KIDINS220	kinase D-interacting substrate, 220kDa	chr2	protein_coding
ENSG00000066735.13	-1.8852184	6.80056221	1.48E-10	1.44E-09	22.44	19.58	7.7	7.45	KIF26A	kinesin family member 26A	chr14	protein_coding
ENSG00000198420.8	2.41437054	4.24797707	4.80E-15	7.69E-14	0.73	0.95	4.29	7.71	TCAF1	TRPM8 channel-associated factor 1	chr7	protein_coding
ENSG00000159208.14	1.38415866	2.92620427	6.59E-06	3.31E-05	1.57	2.13	6.17	6.7	CIART	circadian associated repressor of transcription	chr1	protein_coding
ENSG00000136270.12	-1.1696965	6.54792962	5.09E-05	0.00021703	42.81	38.56	24.46	23.73	TBRG4	transforming growth factor beta regulator 4	chr7	protein_coding
ENSG00000163644.13	1.10549555	-0.3165704	0.01373279	0.03427406	0.03	0.06	0.07	0.18	PPM1K	protein phosphatase, Mg2+/Mn2+ dependent, 1K	chr4	protein_coding
ENSG00000184557.4	-2.1505724	4.91501686	8.84E-13	1.11E-11	14.29	14.87	4.3	4.45	SOCS3	suppressor of cytokine signaling 3	chr17	protein_coding
ENSG00000043355.9	-2.5876151	3.47910667	2.75E-16	4.91E-15	4.6	5.79	1.42	0.88	ZIC2	Zic family member 2	chr13	protein_coding
ENSG00000162607.11	2.18262274	2.68884751	1.05E-11	1.17E-10	0.35	0.52	1.52	3.79	USP1	ubiquitin specific peptidase 1	chr1	protein_coding
ENSG00000237438.5	1.99087718	0.66116324	2.14E-07	1.37E-06	0.12	0.24	0.99	0.9	CECR7	cat eye syndrome chromosome region, candidate 7 (non-protein coding)	chr22	lincRNA
ENSG00000260589.1	5.45974547	-2.7440718	0.00158008	0.00495521	0	0	0.05	0.08	STAM-AS1	STAM antisense RNA 1 (head to head)	chr10	antisense
ENSG00000031698.11	-2.2820736	9.11028068	1.70E-14	2.59E-13	379.25	383.7	100.59	108.59	SARS	seryl-tRNA synthetase	chr1	protein_coding
ENSG00000101076.15	-2.6140898	-1.7300416	0.00044263	0.00156293	0.07	0.02	0.02	0	HNF4A	hepatocyte nuclear factor 4, alpha	chr20	protein_coding
ENSG00000204970.8	-2.2480816	-1.9782395	0.0017973	0.00556364	0.06	0.04	0.02	0	PCDHA1	protocadherin alpha 1	chr5	protein_coding
ENSG00000263126.1	1.84997605	0.42802342	2.82E-06	1.52E-05	0.1	0.13	0.67	0.42	CTC-479C5.1	--	chr16	lincRNA
ENSG00000241333.3	4.88946445	-3.0622981	0.01889873	0.04519557	0	0	0.23	0.59	RN7SL385P	RNA, 7SL, cytoplasmic 385, pseudogene	chr22	misc_RNA
ENSG00000272145.1	1.57356491	-0.9504497	0.00278681	0.00832258	0.1	0.12	0.57	0.29	NFYC-AS1	NFYC antisense RNA 1	chr1	antisense
ENSG00000114573.8	1.32880139	3.5043587	9.80E-06	4.77E-05	1.32	0.93	2.87	4.74	ATP6V1A	ATPase, H+ transporting, lysosomal 70kDa, V1 subunit A	chr3	protein_coding
ENSG00000140932.8	6.24102512	-2.2241472	1.55E-05	7.26E-05	0	0	0.15	0.41	CMTM2	CKLF-like MARVEL transmembrane domain containing 2	chr16	protein_coding
ENSG00000172927.6	-6.5881435	1.49756298	4.36E-36	2.71E-34	1.97	1.66	0.03	0.02	MYEOV	myeloma overexpressed	chr11	protein_coding
ENSG00000080345.16	4.0833537	2.09676941	4.62E-26	1.70E-24	0.03	0.02	0.36	0.77	RIF1	replication timing regulatory factor 1	chr2	protein_coding
ENSG00000245857.2	3.041371	-0.8096546	1.36E-07	8.92E-07	0.1	0.05	0.52	1.15	GS1-24F4.2	--	chr8	lincRNA
ENSG00000170881.4	1.89289686	2.22589904	5.33E-09	4.24E-08	0.57	0.62	2.48	3.44	RNF139	ring finger protein 139	chr8	protein_coding
ENSG00000257621.6	1.4915109	2.73813069	1.59E-06	8.88E-06	0.41	0.34	0.83	2.02	PSMA3-AS1	PSMA3 antisense RNA 1	chr14	antisense
ENSG00000115008.5	-5.8924048	-2.6371928	0.00026535	0.0009747	0.11	0.05	0	0	IL1A	interleukin 1, alpha	chr2	protein_coding
ENSG00000129083.11	1.89322774	3.77436194	5.16E-10	4.69E-09	1.41	1.23	4.31	8.89	COPB1	coatomer protein complex, subunit beta 1	chr11	protein_coding
ENSG00000198093.9	8.55185225	-0.3118748	2.65E-17	5.13E-16	0	0	0.46	0.49	ZNF649	zinc finger protein 649	chr19	protein_coding
ENSG00000145040.3	-1.679726	2.94245411	5.11E-08	3.56E-07	7.13	5.63	2.79	2.52	UCN2	urocortin 2	chr3	protein_coding
ENSG00000162601.8	3.00803997	0.8558504	1.75E-13	2.37E-12	0.05	0.03	0.29	0.54	MYSM1	Myb-like, SWIRM and MPN domains 1	chr1	protein_coding
ENSG00000112715.19	-1.2256546	7.40735857	2.15E-05	9.82E-05	46.84	55.33	27.43	30.81	VEGFA	vascular endothelial growth factor A	chr6	protein_coding
ENSG00000178107.2	-5.5258708	-2.8614387	0.00158008	0.00495521	0.15	0.63	0	0	RP11-998D1	--	chr14	lincRNA
ENSG00000102078.14	-1.2167518	3.05404364	5.60E-05	0.00023652	5.53	4.89	2.14	3.86	SLC25A14	solute carrier family 25 (mitochondrial carrier, brain), member 14	chrX	protein_coding
ENSG00000087303.15	-1.9823095	1.54204382	4.92E-09	3.93E-08	0.8	0.69	0.23	0.27	NID2	nidogen 2 (osteonidogen)	chr14	protein_coding
ENSG00000221845.4	-6.0956928	-2.503296	8.36E-05	0.00034052	0.09	0.05	0	0	C7orf65	chromosome 7 open reading frame 65	chr7	lincRNA
ENSG00000211459.2	3.57476701	11.6414776	4.93E-30	2.27E-28	359.24	481.65	8464.01	4764.58	MT-RNR1	mitochondrially encoded 12S RNA	chrM	Mt_rRNA
ENSG00000150347.13	2.40783811	3.19318871	3.34E-14	4.90E-13	0.24	0.33	1.95	2.07	ARID5B	AT rich interactive domain 5B (MRF1-like)	chr10	protein_coding
ENSG00000164093.14	-2.374424	1.32825788	2.53E-11	2.71E-10	0.62	0.68	0.17	0.17	PITX2	paired-like homeodomain 2	chr4	protein_coding
ENSG00000114859.13	-1.1913805	4.57676271	4.63E-05	0.00020002	8.87	7.43	4.71	4.81	CLCN2	chloride channel, voltage-sensitive 2	chr3	protein_coding
ENSG00000250682.4	8.49701916	-0.3617536	6.57E-17	1.23E-15	0	0	1.53	1.59	LINC00491	long intergenic non-protein coding RNA 491	chr5	lincRNA
ENSG00000155816.18	-2.689034	-1.9282179	0.00079104	0.00266183	0.02	0.05	0.01	0	FMN2	formin 2	chr1	protein_coding
ENSG00000092421.15	-5.5188333	-2.8647712	0.00158008	0.00495521	0.03	0	0	0	SEMA6A	sema domain, transmembrane domain (TM), and cytoplasmic domain, (semaphorin) 6A	chr5	protein_coding
ENSG00000215218.3	2.85238474	0.37303083	4.35E-11	4.53E-10	0.06	0.05	0.6	0.45	UBE2QL1	ubiquitin-conjugating enzyme E2Q family-like 1	chr5	protein_coding
ENSG00000267552.5	-4.2346693	-2.2161448	5.06E-05	0.00021569	0.38	0.36	0	0.04	CTD-2528L1	--	chr19	protein_coding
ENSG00000084623.10	-1.1709063	8.51874759	4.73E-05	0.00020301	298.29	265.47	172.75	160.7	EIF3I	eukaryotic translation initiation factor 3, subunit I	chr1	protein_coding
ENSG00000268536.1	2.47599136	-2.0175039	0.00408095	0.01167544	0	0.2	0.92	0.65	AC005523.3	--	chr19	lincRNA
ENSG00000213096.8	-8.037246	-0.9377505	3.62E-14	5.28E-13	0.12	0.23	0	0	ZNF254	zinc finger protein 254	chr19	protein_coding
ENSG00000251131.1	-1.4069138	2.22567906	8.63E-06	4.25E-05	10.98	8.76	5.44	4.48	CTD-2035E1	--	chr5	antisense
ENSG00000013619.12	-2.0955052	3.3761852	1.29E-11	1.43E-10	2.8	2.81	0.94	0.8	MAMLD1	mastermind-like domain containing 1	chrX	protein_coding
ENSG00000118197.12	1.1186986	2.23045286	0.00037851	0.00135489	0.51	0.86	1.15	2.83	DDX59	DEAD (Asp-Glu-Ala-Asp) box polypeptide 59	chr1	protein_coding
ENSG00000267546.2	-2.6120463	-1.0581961	1.86E-06	1.03E-05	0.55	0.27	0.04	0.13	RP11-666A8	--	chr17	antisense
ENSG00000164070.10	2.22757174	0.7261857	1.03E-08	7.88E-08	0.03	0.05	0.17	0.32	HSPA4L	heat shock 70kDa protein 4-like	chr4	protein_coding
ENSG00000166037.9	2.49187513	2.77108611	1.86E-14	2.81E-13	0.37	0.36	1.84	3.62	CEP57	centrosomal protein 57kDa	chr11	protein_coding
ENSG00000139372.13	1.58876468	2.89543283	2.75E-07	1.73E-06	1.05	0.73	2.97	4.21	TDG	thymine-DNA glycosylase	chr12	protein_coding
ENSG00000075240.15	-1.6687708	5.83844997	1.26E-08	9.47E-08	16.44	14.6	5.91	7.13	GRAMD4	GRAM domain containing 4	chr22	protein_coding
ENSG00000162909.16	-1.1473771	6.1967882	7.13E-05	0.00029551	21.03	21.9	11.73	14.14	CAPN2	calpain 2, (m/II) large subunit	chr1	protein_coding
ENSG00000257475.2	5.80647709	-2.5280583	0.00026535	0.0009747	0	0	0.72	0.15	RP11-983P1	--	chr12	sense_intronic
ENSG00000172037.12	2.7570864	9.62155264	9.22E-20	2.18E-18	28.19	28.88	266.97	246.41	LAMB2	laminin, beta 2 (laminin S)	chr3	protein_coding
ENSG00000186767.5	2.06857767	0.36742977	3.21E-07	2.00E-06	0.09	0.09	0.42	0.6	SPIN4	spindlin family, member 4	chrX	protein_coding
ENSG00000074696.11	1.84646676	5.15788209	4.67E-10	4.26E-09	3.42	3.07	11.55	19.79	PTPLAD1	--	chr15	protein_coding
ENSG00000120063.8	1.48568159	0.46484798	9.50E-05	0.00038402	0.12	0.1	0.33	0.47	GNA13	guanine nucleotide binding protein (G protein), alpha 13	chr17	protein_coding
ENSG00000139173.8	2.51713732	-0.4630817	5.23E-07	3.15E-06	0.04	0.07	0.33	0.51	TMEM117	transmembrane protein 117	chr12	protein_coding
ENSG00000186470.12	-1.5375445	4.85749115	1.71E-07	1.11E-06	8.32	8.63	3.75	4.03	BTN3A2	butyrophilin, subfamily 3, member A2	chr6	protein_coding
ENSG00000016082.13	-6.1022125	-2.5000596	8.36E-05	0.00034052	0.02	0.15	0	0	ISL1	ISL LIM homeobox 1	chr5	protein_coding
ENSG00000255723.1	1.84383748	-1.0862209	0.00121058	0.00391053	0.46	0.32	1.47	2.34	RP11-529H2	--	chr4	processed_transcript
ENSG00000177406.4	-1.3574774	3.09175486	7.91E-06	3.92E-05	2.92	2.68	1.44	1.47	RP11-218M2	--	chr12	antisense
ENSG00000172007.5	7.65951485	-1.0889863	6.01E-12	6.93E-11	0	0	0.05	0.3	RAB33B	RAB33B, member RAS oncogene family	chr4	protein_coding
ENSG00000140471.15	1.9167341	0.67682471	4.78E-07	2.90E-06	0.12	0.08	0.35	0.66	LINS	lines homolog (Drosophila)	chr15	protein_coding
ENSG00000121895.7	-6.9494547	0.12875255	5.25E-24	1.68E-22	0.65	1.16	0.02	0	TMEM156	transmembrane protein 156	chr4	protein_coding
ENSG00000065989.14	-6.2643269	4.76007323	2.62E-60	6.46E-58	7.56	7.16	0.12	0.13	PDE4A	phosphodiesterase 4A, cAMP-specific	chr19	protein_coding
ENSG00000226950.5	-2.552294	5.26220887	5.34E-17	1.01E-15	61.39	56.02	12.12	14.61	DANCR	differentiation antagonizing non-protein coding RNA	chr4	processed_transcript
ENSG00000273129.1	-6.4339616	-2.266126	5.19E-06	2.66E-05	0.32	0.31	0	0	PACERR	PTGS2 antisense NFKB1 complex-mediated expression regulator RNA	chr1	lincRNA
ENSG00000137225.11	-6.2801858	-2.3772357	1.55E-05	7.26E-05	0.03	0.15	0	0	CAPN11	calpain 11	chr6	protein_coding
ENSG00000033030.12	1.532368	2.73436753	8.45E-07	4.92E-06	0.45	0.48	1.5	2.08	ZCCHC8	zinc finger, CCHC domain containing 8	chr12	protein_coding
ENSG00000153162.8	-3.9153797	4.36422961	3.33E-32	1.70E-30	11.42	11.09	0.97	1.02	BMP6	bone morphogenetic protein 6	chr6	protein_coding
ENSG00000273189.1	-1.7049092	1.2969756	6.75E-07	4.00E-06	2.2	2.03	0.95	0.79	CTD-3148I1	--	chr19	processed_transcript
ENSG00000234072.1	2.4983735	5.5413142	1.50E-16	2.73E-15	3.35	3.28	26.41	23.42	AC074117.1	--	chr2	antisense
ENSG00000145781.7	1.77311534	0.6783691	3.10E-06	1.66E-05	0.3	0.39	1.16	1.99	COMMD10	COMM domain containing 10	chr5	protein_coding
ENSG00000131981.14	1.65352632	7.27694024	1.47E-08	1.10E-07	41.08	44.21	172.01	185.69	LGALS3	lectin, galactoside-binding, soluble, 3	chr14	protein_coding
ENSG00000164742.13	5.09154583	2.81789887	5.68E-38	3.85E-36	0.02	0.02	1.14	1.1	ADCY1	adenylate cyclase 1 (brain)	chr7	protein_coding
ENSG00000133703.10	1.26084512	2.01917586	8.78E-05	0.0003559	0.23	0.28	0.62	1.04	KRAS	Kirsten rat sarcoma viral oncogene homolog	chr12	protein_coding
ENSG00000197461.12	-1.0410211	4.66137113	0.00035257	0.00126981	11.12	12.09	7.6	7.42	PDGFA	platelet-derived growth factor alpha polypeptide	chr7	protein_coding
ENSG00000244968.5	7.94163075	-0.8472467	1.56E-12	1.92E-11	0	0	0.09	0.29	LIFR-AS1	LIFR antisense RNA 1	chr5	antisense
ENSG00000157554.17	-1.7599819	1.18260644	3.07E-07	1.92E-06	0.47	0.57	0.12	0.29	ERG	v-ets avian erythroblastosis virus E26 oncogene homolog	chr21	protein_coding
ENSG00000157181.13	3.15587222	1.3150378	2.54E-16	4.54E-15	0.12	0.08	0.65	1.72	C1orf27	chromosome 1 open reading frame 27	chr1	protein_coding
ENSG00000146232.13	-1.6431947	4.22789471	3.52E-08	2.50E-07	9.5	8.28	3.43	4.18	NFKBIE	nuclear factor of kappa light polypeptide gene enhancer in B-cells inhibitor, epsilon	chr6	protein_coding
ENSG00000196247.10	1.71831821	0.10846342	3.69E-05	0.00016155	0.05	0.06	0.1	0.36	ZNF107	zinc finger protein 107	chr7	protein_coding
ENSG00000119686.8	-1.1155125	2.11952401	0.00037092	0.0013307	1.11	1.24	0.62	0.82	FLVCR2	feline leukemia virus subgroup C cellular receptor family, member 2	chr14	protein_coding
ENSG00000215845.9	-7.8326884	0.98681682	7.36E-34	4.08E-32	3.71	5.01	0	0.04	TSTD1	thiosulfate sulfurtransferase (rhodanese)-like domain containing 1	chr1	protein_coding
ENSG00000137561.4	5.04175262	-2.9826774	0.0100455	0.02582742	0	0	0	0.1	TTPA	tocopherol (alpha) transfer protein	chr8	protein_coding
ENSG00000158315.9	1.0638148	1.75455816	0.00105133	0.00343874	0.56	0.67	1.4	2.05	RHBDL2	rhomboid, veinlet-like 2 (Drosophila)	chr1	protein_coding
ENSG00000184227.6	1.53033072	3.26571532	5.11E-07	3.09E-06	2.33	2.3	8.23	9.62	ACOT1	acyl-CoA thioesterase 1	chr14	protein_coding
ENSG00000196465.9	-1.4960755	6.40458827	2.78E-07	1.74E-06	78.42	69.41	36.99	32.76	MYL6B	myosin, light chain 6B, alkali, smooth muscle and non-muscle	chr12	protein_coding
ENSG00000150556.15	-9.2591117	0.22128466	4.97E-25	1.73E-23	0.94	0.94	0	0	LYPD6B	LY6/PLAUR domain containing 6B	chr2	protein_coding
ENSG00000131435.11	14.2079334	5.16535701	3.79E-92	4.78E-89	0	0	38.42	28.93	PDLIM4	PDZ and LIM domain 4	chr5	protein_coding
ENSG00000163510.12	3.11141249	1.46655681	1.74E-16	3.16E-15	0.08	0.19	0.89	2.25	CWC22	CWC22 spliceosome-associated protein	chr2	protein_coding
ENSG00000008710.16	-1.2449595	7.26498527	1.62E-05	7.55E-05	12.31	12.46	7.26	6.65	PKD1	polycystic kidney disease 1 (autosomal dominant)	chr16	protein_coding
ENSG00000205707.9	3.36074502	-0.1824859	7.80E-11	7.90E-10	0.08	0.03	0.37	1.25	LYRM5	LYR motif containing 5	chr12	protein_coding
ENSG00000102931.6	1.33489789	4.30488044	6.22E-06	3.14E-05	4.26	4.23	12.2	16.44	ARL2BP	ADP-ribosylation factor-like 2 binding protein	chr16	protein_coding
ENSG00000108932.10	2.57367816	3.85835067	2.18E-16	3.92E-15	0.79	0.9	5.4	8.08	SLC16A6	solute carrier family 16, member 6	chr17	protein_coding
ENSG00000157450.14	1.17271293	2.87466206	0.00012513	0.00049361	0.6	0.55	1.45	2.03	RNF111	ring finger protein 111	chr15	protein_coding
ENSG00000258056.2	-1.3647794	3.64591984	4.92E-06	2.54E-05	10.01	10.19	4.53	5.94	RP11-644F5.	--	chr12	antisense
ENSG00000183486.11	2.7411836	0.02687126	3.16E-09	2.60E-08	0.06	0.07	0.53	0.62	MX2	MX dynamin-like GTPase 2	chr21	protein_coding
ENSG00000235151.1	-5.3715723	-2.9480397	0.00290522	0.00856312	0.37	0.05	0	0	AC114730.2	--	chr2	lincRNA
ENSG00000223571.4	4.72703895	-3.141721	0.01889873	0.04519557	0	0	0.3	0.04	DHRSX-IT1	DHRSX intronic transcript 1	chrX	sense_intronic
ENSG00000279692.1	-1.3513474	-0.0744171	0.00096074	0.00316531	0.66	0.72	0.25	0.46	RP11-1055B	--	chr17	TEC
ENSG00000272071.1	-9.2588208	0.22106169	6.26E-25	2.16E-23	3.51	3.4	0	0	RP11-332J1	--	chr5	lincRNA
ENSG00000173705.7	-9.1306815	0.09530825	1.09E-23	3.43E-22	0.26	0.42	0	0	SUSD5	sushi domain containing 5	chr3	protein_coding
ENSG00000137944.15	1.26574077	2.39566329	5.51E-05	0.00023321	0.94	1.39	2.35	5.16	CCBL2	cysteine conjugate-beta lyase 2	chr1	protein_coding
ENSG00000260822.1	3.69713595	0.84542288	2.89E-17	5.56E-16	0.05	0.03	0.43	0.94	GS1-358P8.4	--	chrX	sense_overlapping
ENSG00000179240.7	1.47209567	0.2767589	0.00022219	0.00083326	0.08	0.11	0.29	0.38	RP11-111M2	--	chr11	protein_coding
ENSG00000058063.14	3.99722649	2.84784829	9.59E-29	4.15E-27	0.13	0.05	1.49	2.36	ATP11B	ATPase, class VI, type 11B	chr3	protein_coding
ENSG00000254979.4	-1.3365285	1.58138353	5.58E-05	0.000236	2.95	7.2	2.96	2.36	RP11-872D1	--	chr11	protein_coding
ENSG00000035928.13	1.63541928	2.94916372	1.21E-07	8.04E-07	0.74	0.48	2.07	3.05	RFC1	replication factor C (activator 1) 1, 145kDa	chr4	protein_coding
ENSG00000119729.9	1.49419263	2.02277139	3.70E-06	1.95E-05	0.51	0.62	1.38	2.89	RHOQ	ras homolog family member Q	chr2	protein_coding
ENSG00000162929.12	1.97944875	1.3234099	2.09E-08	1.52E-07	0.11	0.18	0.77	0.74	KIAA1841	KIAA1841	chr2	protein_coding
ENSG00000186395.6	-1.2529045	5.61546978	1.60E-05	7.47E-05	25.28	27.37	14.89	14.53	KRT10	keratin 10, type I	chr17	protein_coding
ENSG00000260190.1	-4.2385469	-0.42918	1.22E-13	1.68E-12	0.82	1.9	0.2	0	RP11-229P1	--	chr9	sense_overlapping
ENSG00000156469.7	1.25626351	0.40485938	0.00119218	0.00385709	0.33	0.37	0.83	1.4	MTERF3	mitochondrial transcription termination factor 3	chr8	protein_coding
ENSG00000116473.13	1.28736303	3.07289728	2.37E-05	0.00010765	0.67	0.8	1.63	3.2	RAP1A	RAP1A, member of RAS oncogene family	chr1	protein_coding
ENSG00000149679.10	1.27294993	4.43716529	1.53E-05	7.21E-05	2.72	2.68	9.39	7.96	CABLES2	Cdk5 and Abl enzyme substrate 2	chr20	protein_coding
ENSG00000279891.1	5.85884455	-2.4896625	0.00026535	0.0009747	0	0	0.06	0.14	FLJ42393	--	chr3	TEC
ENSG00000237686.5	-1.6038997	3.04957652	1.63E-07	1.06E-06	3.56	5.22	1.83	2.02	RP5-1120P1	--	chr6	antisense
ENSG00000184271.14	-1.2111198	2.06789328	0.00013609	0.00053288	0.74	1	0.59	0.41	POU6F1	POU class 6 homeobox 1	chr12	protein_coding
ENSG00000163840.8	1.36391616	2.16657087	1.84E-05	8.50E-05	0.39	0.29	1.08	1.26	DTX3L	deltex 3 like, E3 ubiquitin ligase	chr3	protein_coding
ENSG00000136048.12	1.09624817	3.10246185	0.00028828	0.00105299	1.35	1.14	2.38	4.77	DRAM1	DNA-damage regulated autophagy modulator 1	chr12	protein_coding
ENSG00000053770.10	1.59479984	1.05261448	6.63E-06	3.34E-05	0.08	0.05	0.18	0.37	AP5M1	adaptor-related protein complex 5, mu 1 subunit	chr14	protein_coding
ENSG00000273802.1	2.92573148	1.15880132	2.71E-14	4.03E-13	0.77	0.5	7	5.95	HIST1H2BG	histone cluster 1, H2bg	chr6	protein_coding
ENSG00000168476.10	-1.1333873	6.82911682	8.44E-05	0.00034287	79.53	68.23	45.5	44.27	REEP4	receptor accessory protein 4	chr8	protein_coding
ENSG00000138606.18	-1.4621541	-0.1569992	0.00056862	0.00196986	0.24	0.23	0.14	0.09	SHF	Src homology 2 domain containing F	chr15	protein_coding
ENSG00000184304.13	1.17354066	1.07782935	0.00075619	0.0025571	0.23	0.3	0.53	1.09	PRKD1	protein kinase D1	chr14	protein_coding
ENSG00000181381.12	2.2113975	-0.2140806	2.01E-06	1.11E-05	0.03	0.04	0.12	0.26	DDX60L	DEAD (Asp-Glu-Ala-Asp) box polypeptide 60-like	chr4	protein_coding
ENSG00000172379.17	3.98831391	1.65582325	3.35E-23	1.02E-21	0.05	0.03	0.89	0.9	ARNT2	aryl-hydrocarbon receptor nuclear translocator 2	chr15	protein_coding
ENSG00000224177.5	-8.4911882	-0.5148394	1.26E-17	2.52E-16	1.03	0.62	0	0	LINC00570	long intergenic non-protein coding RNA 570	chr2	lincRNA
ENSG00000196730.11	-11.019871	1.96736252	1.37E-46	1.56E-44	0.96	1.07	0	0	DAPK1	death-associated protein kinase 1	chr9	protein_coding
ENSG00000236287.6	2.2532623	0.82513511	4.26E-09	3.45E-08	0.2	0.13	0.7	1.48	ZBED5	zinc finger, BED-type containing 5	chr11	protein_coding
ENSG00000118620.11	1.69228141	-0.8616742	0.00101857	0.00334071	0.01	0.04	0.08	0.15	ZNF430	zinc finger protein 430	chr19	protein_coding
ENSG00000175287.17	-2.8210263	-1.8448139	0.00010631	0.0004256	0.06	0.25	0.64	0.01	PHYHD1	phytanoyl-CoA dioxygenase domain containing 1	chr9	protein_coding
ENSG00000197535.13	2.36992672	2.41123575	6.60E-13	8.43E-12	0.1	0.13	0.64	0.89	MYO5A	myosin VA (heavy chain 12, myoxin)	chr15	protein_coding
ENSG00000111077.16	-3.5026529	5.75580006	2.33E-28	9.84E-27	15.51	14.45	1.75	1.77	TNS2	tensin 2	chr12	protein_coding
ENSG00000278610.1	-1.4245929	-1.4122557	0.00151564	0.0292584	0.19	1.21	0.23	0.43	RP11-455P2	--	chr1	lincRNA
ENSG00000235568.5	8.82315529	-0.0638542	1.18E-19	2.77E-18	0	0	0.3	0.35	NFAM1	NFAT activating protein with ITAM motif 1	chr22	protein_coding
ENSG00000273314.1	1.2287467	1.58390837	0.00021423	0.00080555	0.51	0.77	1.43	2.59	RP5-1136G1	--	chr7	antisense
ENSG00000166924.7	1.14954643	2.81651197	0.00016732	0.00063992	1.16	0.79	2.73	3.05	NYAP1	neuronal tyrosine-phosphorylated phosphoinositide-3-kinase adaptor 1	chr7	protein_coding
ENSG00000138080.12	1.10854233	0.30544349	0.00467704	0.01322614	0.12	0.18	0.44	0.43	EMILIN1	elastin microfibril interfacer 1	chr2	protein_coding
ENSG00000212232.1	3.89571407	-0.6160701	1.13E-10	1.12E-09	0	0.48	7.97	1.84	SNORD17	small nucleolar RNA, C/D box 17	chr20	snoRNA
ENSG00000236432.6	1.16857965	-0.7327904	0.01574469	0.03875864	0.01	0.13	0.28	0.16	AC097662.2	--	chr2	antisense
ENSG00000271192.1	-5.2072905	-3.0363253	0.00538165	0.01488851	0.29	0.1	0	0	RP4-555D20	--	chr3	lincRNA
ENSG00000158373.8	2.12256275	4.05124922	3.69E-12	4.35E-11	4.08	7.39	30.82	35.64	HIST1H2BD	histone cluster 1, H2bd	chr6	protein_coding
ENSG00000168785.6	-1.6758798	1.77240937	3.56E-07	2.21E-06	1.19	1.19	0.52	0.47	TSPAN5	tetraspanin 5	chr4	protein_coding
ENSG00000175040.5	-4.3495517	4.02608129	1.72E-36	1.08E-34	5.88	5.1	0.43	0.29	CHST2	carbohydrate (N-acetylglucosamine-6-O) sulfotransferase 2	chr3	protein_coding
ENSG00000135913.9	1.86127628	1.4087713	7.53E-08	4.54E-07	0.13	0.07	0.38	0.63	USP37	ubiquitin specific peptidase 37	chr2	protein_coding
ENSG00000273983.1	4.27946365	1.54838828	1.28E-24	4.30E-23	0.11	0.07	2.51	2.27	HIST1H3G	histone cluster 1, H3g	chr6	protein_coding

ENSG00000188706.11	-1.9426347	6.3683143	4.66E-11	4.84E-10	20.71	24.46	8.43	7.18	ZDHHC9	zinc finger, DHHC-type containing 9	chrX	protein_coding
ENSG00000128604.17	11.2863354	2.27589922	7.67E-49	9.89E-47	0	0	3.7	2.81	IRF5	interferon regulatory factor 5	chr7	protein_coding
ENSG00000272703.1	6.69343649	-1.8848726	3.73E-07	2.30E-06	0	0	0.48	1.57	RP11-78A19	--	chr18	antisense
ENSG00000124151.17	2.09697487	4.30706107	4.76E-12	5.55E-11	0.59	0.93	3.91	4.73	NCOA3	nuclear receptor coactivator 3	chr20	protein_coding
ENSG00000108506.10	3.92091673	1.14170923	3.86E-20	9.38E-19	0.03	0.04	0.44	0.94	INTS2	integrator complex subunit 2	chr17	protein_coding
ENSG00000133816.12	-1.9103692	5.49689345	1.09E-10	1.08E-09	7.83	7.53	2.78	2.66	MICAL2	microtubule associated monooxygenase, calponin and LIM domain containing 2	chr11	protein_coding
ENSG00000021826.13	2.63454592	8.86840631	2.47E-18	5.22E-17	15.97	17.97	136.61	144.19	CPS1	carbamoyl-phosphate synthase 1, mitochondrial	chr2	protein_coding
ENSG00000130517.12	-1.1305547	4.15264668	0.0001217	0.00048099	2.96	2.42	1.66	1.62	PGPEP1	pyroglutamyl-peptidase I	chr19	protein_coding
ENSG00000182871.13	1.39191295	3.47897127	3.82E-06	2.01E-05	0.74	0.71	2.52	2.54	COL18A1	collagen, type XVIII, alpha 1	chr21	protein_coding
ENSG00000278200.1	6.49235139	-2.0492034	1.78E-06	9.85E-06	0	0	0.64	0.08	RP13-766D2	--	chr17	lincRNA
ENSG00000137776.15	1.33700561	4.85838552	5.03E-06	2.59E-05	2.35	4.07	8.06	13.63	SLTM	SAFB-like, transcription modulator	chr15	protein_coding
ENSG00000130270.15	-1.2238199	3.50496095	4.22E-05	0.0001834	2.33	1.65	1.1	1.17	ATP8B3	ATPase, aminophospholipid transporter, class I, type 8B, member 3	chr19	protein_coding
ENSG00000210107.1	1.87302616	0.78489695	6.28E-07	3.73E-06	7.43	8.27	36.04	40.85	MT-TQ	mitochondrially encoded tRNA glutamine	chrM	Mt_tRNA
ENSG00000119888.9	4.30908353	1.74436339	1.14E-25	4.10E-24	0.11	0.14	2.26	4.36	EPCAM	epithelial cell adhesion molecule	chr2	protein_coding
ENSG00000120658.11	-8.1233874	-0.8563968	1.19E-14	1.84E-13	0.38	0.18	0	0	ENOX1	ecto-NOX disulfide-thiol exchanger 1	chr13	protein_coding
ENSG00000163006.10	2.35674427	-0.3692261	8.80E-07	5.11E-06	0.04	0.11	0.27	0.76	CCDC138	coiled-coil domain containing 138	chr2	protein_coding
ENSG00000164323.11	1.93662468	-0.2638139	2.36E-05	0.00010714	0.02	0.06	0.1	0.31	CFAP97	cilia and flagella associated protein 97	chr4	protein_coding
ENSG00000151092.15	1.10483349	2.84760439	0.00030196	0.0010991	1.14	1.37	2.83	4.4	NGLY1	N-glycanase 1	chr3	protein_coding
ENSG00000255621.1	-5.5188333	-2.8647712	0.00158008	0.00495521	0.11	0	0	0	RP11-377D9	--	chr12	lincRNA
ENSG00000230002.2	3.29919697	-1.8616257	0.00016407	0.00062818	0	0.03	0.2	0.18	ALMS1-IT1	ALMS1 intronic transcript 1	chr2	sense_intronic
ENSG00000203326.8	1.32265224	-0.3280461	0.00238311	0.0072004	0.04	0.06	0.11	0.21	ZNF525	zinc finger protein 525	chr19	protein_coding
ENSG00000232324.1	-6.9060661	0.90447687	3.53E-31	1.72E-29	4.53	6.33	0.06	0.05	AC008440.1C	--	chr19	lincRNA
ENSG00000189376.10	1.73292522	2.59624176	3.76E-08	2.66E-07	1.85	1.46	6.86	7.88	C8orf76	chromosome 8 open reading frame 76	chr8	protein_coding
ENSG00000104447.10	-2.6664415	-0.438533	3.60E-08	2.55E-07	0.09	0.1	0.02	0.02	TRPS1	trichorhinophalangeal syndrome I	chr8	protein_coding
ENSG00000099849.13	1.33639834	5.14868176	4.75E-06	2.45E-05	8.47	5.77	23.58	24.47	RASSF7	Ras association (RalGDS/AF-6) domain family (N-terminal) member 7	chr11	protein_coding
ENSG00000185896.10	-1.0951027	8.9077312	0.00013764	0.0005386	196.57	195.94	124.37	120.31	LAMP1	lysosomal-associated membrane protein 1	chr13	protein_coding
ENSG00000070882.11	1.07740138	3.29632897	0.00032833	0.00118768	0.84	0.65	1.68	2.56	OSBPL3	oxysterol binding protein-like 3	chr7	protein_coding
ENSG00000170525.17	1.11763981	6.24156112	0.00010877	0.0004343	8.09	7.37	20.05	24.8	PFKFB3	6-phosphofructo-2-kinase/fructose-2,6-biphosphatase 3	chr10	protein_coding
ENSG00000121644.17	1.3156781	2.63054943	2.23E-05	0.00010133	0.77	0.57	1.54	2.95	DESI2	desumoylating isopeptidase 2	chr1	protein_coding
ENSG00000156711.15	2.88166193	3.1332666	1.20E-18	2.59E-17	0.32	0.18	2.38	2.49	MAPK13	mitogen-activated protein kinase 13	chr6	protein_coding
ENSG00000136108.13	3.19804466	2.07588879	4.17E-19	9.44E-18	0.11	0.19	1.08	2.7	CKAP2	cytoskeleton associated protein 2	chr13	protein_coding
ENSG00000152778.8	1.14760514	0.16869719	0.0042317	0.01206928	0.09	0.17	0.16	0.61	IFIT5	interferon-induced protein with tetratricopeptide repeats 5	chr10	protein_coding
ENSG00000124098.9	1.46085521	4.44423191	7.93E-07	4.64E-06	2.96	3.18	10.01	12.57	FAM210B	family with sequence similarity 210, member B	chr20	protein_coding
ENSG00000131931.7	2.06518222	0.21354433	8.06E-07	4.71E-06	0.07	0.24	0.55	1.17	THAP1	THAP domain containing, apoptosis associated protein 1	chr8	protein_coding
ENSG00000160991.14	-1.1538701	5.52344237	6.90E-05	0.00028699	5.14	4.35	2.96	2.73	ORAI2	ORAI calcium release-activated calcium modulator 2	chr7	protein_coding
ENSG00000141404.14	-1.5210703	2.44767011	1.20E-06	6.83E-06	0.88	0.84	0.46	0.34	GNAL	guanine nucleotide binding protein (G protein), alpha activating activity polypeptide, olfactory type	chr18	protein_coding
ENSG00000270580.4	-1.4676087	5.34214134	5.21E-07	3.15E-06	14.56	18.71	8.33	7.66	PKD1P6	polycystic kidney disease 1 (autosomal dominant) pseudogene 6	chr16	processed_transcript
ENSG00000114416.16	1.50779604	4.31962564	3.87E-07	2.38E-06	0.81	0.99	2.44	4.41	FXR1	fragile X mental retardation, autosomal homolog 1	chr3	protein_coding
ENSG00000130300.7	-4.5884464	-1.2442568	2.17E-09	1.82E-08	0.37	0.15	0.03	0	PLVAP	plasmalemma vesicle associated protein	chr19	protein_coding
ENSG00000157045.7	-1.2812134	4.39260878	1.30E-05	6.16E-05	18.96	20.55	9.25	12.47	NTAN1	N-terminal asparagine amidase	chr16	protein_coding
ENSG00000275966.1	-1.2536841	0.67735767	0.00057519	0.00199196	4.52	2.71	1.83	2.22	RP11-1055B	--	chr17	lincRNA
ENSG00000272977.1	2.03592449	-0.4395213	1.45E-05	6.84E-05	0.07	0.04	0.33	0.28	CTA-390C10	--	chr22	sense_intronic
ENSG00000205978.5	-6.2420973	-0.5480314	2.41E-17	4.69E-16	0.13	0.14	0	0	NYNRIN	NYN domain and retroviral integrase containing	chr14	protein_coding
ENSG00000142700.10	11.879377	2.85773214	3.33E-57	7.13E-55	0	0	4.94	4.58	DMRTA2	DMRT-like family A2	chr1	protein_coding
ENSG00000265096.1	4.07362912	1.66235716	3.59E-24	1.17E-22	0.5	0.06	6.63	6.23	C1QTNF1-AS	C1QTNF1 antisense RNA 1	chr17	antisense
ENSG00000147689.15	-3.9323591	3.15768048	1.24E-29	5.64E-28	2.86	1.88	0.22	0.2	FAM83A	family with sequence similarity 83, member A	chr8	protein_coding
ENSG00000104856.12	-2.4327353	5.17174737	1.08E-15	1.83E-14	22.11	20.7	5.13	5.45	RELB	v-rel avian reticuloendotheliosis viral oncogene homolog B	chr19	protein_coding
ENSG00000177728.13	-1.1067252	5.18776003	0.00013797	0.00053979	7.29	7.47	4.85	4.26	KIAA0195	KIAA0195	chr17	protein_coding
ENSG00000135046.12	1.33124371	4.85158634	5.50E-06	2.80E-05	5.31	5.84	12.44	25.26	ANXA1	annexin A1	chr9	protein_coding
ENSG00000279900.1	5.45007274	-2.7494288	0.00158008	0.00495521	0	0	0.14	0.6	RP11-113K2	--	chr11	TEC
ENSG00000168393.11	-1.1602242	5.66249806	6.23E-05	0.00026157	51.49	40.32	29.87	24.8	DTYMK	deoxythymidylate kinase (thymidylate kinase)	chr2	protein_coding
ENSG00000081791.7	-1.039336	5.79888393	0.00031894	0.00115597	12.65	11.73	7.37	8.47	KIAA0141	KIAA0141	chr5	protein_coding
ENSG00000158711.12	1.66700214	2.35756001	1.78E-07	1.15E-06	0.13	0.22	0.64	0.87	ELK4	ELK4, ETS-domain protein (SRF accessory protein 1)	chr1	protein_coding
ENSG00000145362.15	1.35644864	-1.1891145	0.01656825	0.04052383	0.01	0.02	0.03	0.04	ANK2	ankyrin 2, neuronal	chr4	protein_coding
ENSG00000149308.15	3.86371308	0.98898336	7.34E-19	1.62E-17	0.05	0.01	0.49	0.75	NPAT	nuclear protein, ataxia-telangiectasia locus	chr11	protein_coding
ENSG00000205765.7	1.95646232	1.53480882	1.42E-08	1.07E-07	0.13	0.22	0.49	1.35	C5orf51	chromosome 5 open reading frame 51	chr5	protein_coding
ENSG00000261302.4	2.89802919	-0.9281159	6.90E-07	4.07E-06	0.13	0	0.59	0.82	RP11-343H1	--	chr16	antisense
ENSG00000001631.13	2.08507732	1.85875888	6.17E-10	5.54E-09	0.24	0.18	0.85	1.58	KRIT1	KRIT1, ankyrin repeat containing	chr7	protein_coding
ENSG00000172123.11	-6.1911323	-2.4387921	4.74E-05	0.00020301	0.06	0.1	0	0	SLFN12	schlafen family member 12	chr17	protein_coding
ENSG00000065526.9	1.38541281	5.63223859	2.00E-06	1.10E-05	1.54	2.09	6.69	5.92	SPEN	spen family transcriptional repressor	chr1	protein_coding
ENSG00000154122.11	-1.0544324	6.48639394	0.00025178	0.00093636	12.14	11.75	7.34	8	ANKH	ANKH inorganic pyrophosphate transport regulator	chr5	protein_coding
ENSG00000275630.1	5.20747907	-2.8915189	0.00538165	0.01488851	0	0	0.45	0.58	RP6-65G23.5	--	chr14	lincRNA
ENSG00000234477.1	-3.9902342	-2.3832208	0.00039332	0.00140265	0.26	0.28	0	0.03	AC004231.2	--	chr17	antisense
ENSG00000124920.12	-4.5287086	3.63374069	3.03E-37	1.97E-35	3.16	3.15	0.24	0.13	MYRF	myelin regulatory factor	chr11	protein_coding
ENSG00000056998.17	-3.1125771	-1.1426866	8.65E-07	5.03E-06	0.27	0.07	0.06	0	GYG2	glycogenin 2	chrX	protein_coding
ENSG00000235863.3	-7.3065551	-1.5781722	1.11E-09	9.61E-09	0.32	0.25	0	0	B3GALT4	UDP-Gal:betaGlcNAc beta 1,3-galactosyltransferase, polypeptide 4	chr6	protein_coding
ENSG00000143416.19	-5.9018396	4.16772524	3.31E-54	5.96E-52	16.46	12.73	0.2	0.44	SELENBP1	selenium binding protein 1	chr1	protein_coding
ENSG00000198585.10	-1.1543271	5.34788255	6.95E-05	0.0002889	6.08	6.87	3.62	4.13	NUDT16	nudix (nucleoside diphosphate linked moiety X)-type motif 16	chr3	protein_coding
ENSG00000256982.1	3.03820646	-0.6272418	5.50E-08	3.82E-07	0.05	0.04	0.58	0.43	CTD-2555A7	--	chr16	lincRNA
ENSG00000109705.7	-1.4294407	0.54143241	0.00012455	0.00049141	0.65	0.55	0.32	0.27	NKX3-2	NK3 homeobox 2	chr4	protein_coding
ENSG00000245870.2	-6.5014576	-2.2153917	5.19E-06	2.66E-05	0.3	0.03	0	0	LINC00682	long intergenic non-protein coding RNA 682	chr4	lincRNA
ENSG00000054392.11	2.91342395	1.15109204	5.72E-14	8.19E-13	0.06	0.06	0.66	0.55	HHAT	hedgehog acyltransferase	chr1	protein_coding
ENSG00000267581.1	5.33927207	-2.8158158	0.00290522	0.00856312	0	0	0.21	0.31	CTC-559E9.4	--	chr19	lincRNA
ENSG00000224902.5	-5.5188333	-2.8647712	0.00158008	0.00495521	0.51	0	0	0	GAGE12H	G antigen 12H	chrX	protein_coding
ENSG00000177879.13	1.78928926	4.76025937	1.86E-09	1.57E-08	5.38	5.96	21.02	31.47	AP3S1	adaptor-related protein complex 3, sigma 1 subunit	chr5	protein_coding
ENSG00000115808.10	1.92509191	1.82448313	9.41E-09	7.23E-08	0.12	0.17	0.48	0.97	STRN	striatin, calmodulin binding protein	chr2	protein_coding
ENSG00000163823.3	3.93382269	-1.3825535	6.88E-07	4.06E-06	0.02	0	0.23	0.22	CCR1	chemokine (C-C motif) receptor 1	chr3	protein_coding
ENSG00000170439.6	4.1220835	1.63693364	2.55E-24	8.40E-23	0.25	0.09	4.85	3.25	METTL7B	methyltransferase like 7B	chr12	protein_coding
ENSG00000226453.1	4.89462967	-3.0595066	0.01889873	0.04519557	0	0	0.12	0.17	RP11-379B8.	--	chr6	lincRNA
ENSG00000154146.11	1.0247985	3.29231019	0.00061648	0.00211904	4.27	3.64	11.11	10.34	NRGN	neurogranin (protein kinase C substrate, RC3)	chr11	protein_coding
ENSG00000198208.10	-8.7669942	-0.2528581	3.98E-20	9.65E-19	0.32	0.17	0	0	RPS6KL1	ribosomal protein S6 kinase-like 1	chr14	protein_coding
ENSG00000233608.3	-1.5193409	4.69380888	2.63E-07	1.66E-06	17.22	15.83	7.62	7.74	TWIST2	twist family bHLH transcription factor 2	chr2	protein_coding
ENSG00000166432.13	2.20288577	-1.6535513	0.00158671	0.00496952	0.03	0.01	0.1	0.17	ZMAT1	zinc finger, matrin-type 1	chrX	protein_coding
ENSG00000138685.11	-1.8465836	2.44210323	5.79E-09	4.60E-08	1.03	1	0.34	0.41	FGF2	fibroblast growth factor 2 (basic)	chr4	protein_coding
ENSG00000148288.10	-5.8966026	-2.6351464	0.00026535	0.0009747	0.04	0.1	0	0	GBGT1	globoside alpha-1,3-N-acetylgalactosaminyltransferase 1	chr9	protein_coding
ENSG00000280587.1	2.73614222	-0.7004159	6.24E-07	3.72E-06	0.05	0.02	0.3	0.31	LINC01348	long intergenic non-protein coding RNA 1348	chr1	lincRNA
ENSG00000263968.2	-5.3760424	-2.9459444	0.00290522	0.00856312	0.27	0.57	0	0	RN7SL381P	RNA, 7SL, cytoplasmic 381, pseudogene	chr16	misc_RNA
ENSG00000159387.7	9.41204656	0.4777358	1.61E-25	5.79E-24	0	0	1.02	0.75	IRX6	iroquois homeobox 6	chr16	protein_coding
ENSG00000280216.1	5.07225195	-2.9660962	0.0100455	0.02582742	0	0	0.47	0.2	RP3-333H23	--	chr22	TEC
ENSG00000113240.11	1.57982508	0.52763854	3.22E-05	0.00014247	0.21	0.06	0.41	0.69	CLK4	CDC-like kinase 4	chr5	protein_coding
ENSG00000234277.2	-1.5855764	-1.4183841	0.00496338	0.01395792	0.26	0.14	0.03	0.14	CTD-2090I1	--	chr1	lincRNA
ENSG00000135744.7	-3.9854724	2.48538666	4.60E-28	1.92E-26	3.44	4.02	0.27	0.36	AGT	angiotensinogen (serpin peptidase inhibitor, clade A, member 8)	chr1	protein_coding
ENSG00000171450.5	2.35452394	1.05381882	2.24E-10	2.13E-09	0.25	0.17	1.69	1.16	CDK5R2	cyclin-dependent kinase 5, regulatory subunit 2 (p39)	chr2	protein_coding
ENSG00000184270.4	3.10572403	-0.9940384	3.09E-07	1.93E-06	0.34	0	3.26	0.97	HIST2H2AB	histone cluster 2, H2ab	chr1	protein_coding
ENSG00000197620.9	-1.8665706	3.01630114	1.80E-09	1.52E-08	2.83	2.29	1	0.87	CXorf40A	chromosome X open reading frame 40A	chrX	protein_coding
ENSG00000169100.11	-1.4695592	10.2032299	3.91E-07	2.41E-06	713.01	646.67	336.19	317.72	SLC25A6	solute carrier family 25 (mitochondrial carrier; adenine nucleotide translocator), member 6	chrX	protein_coding
ENSG00000204390.9	-1.8742182	1.69269192	1.98E-08	1.45E-07	1.61	1.6	0.57	0.6	HSPA1L	heat shock 70kDa protein 1-like	chr6	protein_coding
ENSG00000263120.1	4.72173804	-3.1445547	0.01889873	0.04519557	0	0	0.07	0.02	RP5-1107A1	--	chr17	sense_intronic
ENSG00000166896.6	2.11798146	0.13973124	8.55E-07	4.98E-06	0.11	0.39	0.69	2.14	XRCC6BP1	XRCC6 binding protein 1	chr12	protein_coding
ENSG00000110944.7	2.13973598	-0.642317	1.94E-05	8.92E-05	0.08	0.24	0.95	0.94	IL23A	interleukin 23, alpha subunit p19	chr12	protein_coding
ENSG00000058866.13	9.82792068	0.87343563	3.61E-30	1.67E-28	0	0	0.6	0.68	DGKG	diacylglycerol kinase, gamma 90kDa	chr3	protein_coding
ENSG00000164031.15	1.09720258	0.36440505	0.00394406	0.01131621	0.05	0.16	0.29	0.29	DNAJB14	DnaJ (Hsp40) homolog, subfamily B, member 14	chr4	protein_coding
ENSG00000281741.1	3.41751404	-2.4186794	0.00112121	0.00364442	0	0.03	0.24	0.2	AC241377.2	--	chr1	protein_coding
ENSG00000099338.21	3.22214223	-1.1555546	1.08E-06	6.15E-06	0	0.03	0.19	0.2	CATSPERG	catsper channel auxiliary subunit gamma	chr19	protein_coding
ENSG00000166595.10	1.521042	6.0018694	1.89E-07	1.22E-06	44.43	34.67	152.39	150.42	FAM96B	family with sequence similarity 96, member B	chr16	protein_coding
ENSG00000130731.14	-1.2881929	5.26854449	9.85E-06	4.79E-05	52.16	50.62	33.3	22.52	C16orf13	chromosome 16 open reading frame 13	chr16	protein_coding
ENSG00000229043.2	-1.926868	2.58701101	1.08E-09	9.41E-09	3.69	3.99	1.33	1.36	AC091729.9	--	chr7	antisense
ENSG00000174672.14	1.73383531	4.37092466	6.55E-09	5.16E-08	1.5	1.22	6.61	5.41	BRSK2	BR serine/threonine kinase 2	chr11	protein_coding
ENSG00000272567.1	5.3196533	-2.8266272	0.00290522	0.00856312	0	0	0	0.86	RP11-73K9.3	--	chr4	lincRNA
ENSG00000274964.1	3.90782128	-2.0840989	8.40E-05	0.0003415	0	0.02	0.12	0.36	RP11-817I4.1	--	chr12	sense_intronic
ENSG00000100483.12	1.01763358	-0.0701138	0.01326645	0.03319311	0.24	0.28	0.48	0.92	VCPKMT	valosin containing protein lysine (K) methyltransferase	chr14	protein_coding
ENSG00000163499.10	2.20035297	0.82415189	6.74E-09	5.30E-08	0.44	0.63	3.05	3.53	CRYBA2	crystallin, beta A2	chr2	protein_coding
ENSG00000170540.13	1.09504631	5.21696953	0.00016242	0.00062388	7.53	7	16.16	25.46	ARL6IP1	ADP-ribosylation factor-like 6 interacting protein 1	chr16	protein_coding
ENSG00000175305.15	5.2308123	-0.2751085	3.13E-15	5.10E-14	0.02	0	0.19	0.7	CCNE2	cyclin E2	chr8	protein_coding
ENSG00000205593.10	1.09439711	3.75286688	0.00022589	0.00084622	1.3	1.5	4.27	3.69	DENND6B	DENN/MADD domain containing 6B	chr22	protein_coding
ENSG00000019549.7	-2.4329115	3.17734326	1.21E-14	1.87E-13	4.29	6.57	0.9	1.78	SNAI2	snail family zinc finger 2	chr8	protein_coding
ENSG00000182199.9	-1.6234718	8.43592066	2.46E-08	1.78E-07	152.81	126.46	63.82	56.85	SHMT2	serine hydroxymethyltransferase 2 (mitochondrial)	chr12	protein_coding
ENSG00000204397.6	1.84795707	0.92293051	4.36E-07	2.66E-06	0.74	0.78	3.69	3.62	CARD16	caspase recruitment domain family, member 16	chr11	protein_coding
ENSG00000197632.7	7.37893744	-1.3369895	1.84E-10	1.77E-09	0	0	0.3	0.33	SERPINB2	serpin peptidase inhibitor, clade B (ovalbumin), member 2	chr18	protein_coding
ENSG00000100121.11	-8.7323209	-0.2885192	9.48E-20	2.23E-18	0.79	1.41	0	0	GGTLC2	gamma-glutamyltransferase light chain 2	chr22	protein_coding
ENSG00000132031.11	1.6089617	-0.6574438	0.00085931	0.00288128	0.04	0.13	0.32	0.4	MATN3	matrilin 3	chr2	protein_coding
ENSG00000185745.9	1.61864856	0.25932484	4.93E-05	0.00021051	0.12	0.07	0.17	0.61	IFIT1	interferon-induced protein with tetratricopeptide repeats 1	chr10	protein_coding
ENSG00000170949.16	-8.5101245	-0.4972787	9.17E-18	1.85E-16	0.23	0.16	0	0	ZNF160	zinc finger protein 160	chr19	protein_coding
ENSG00000256514.1	1.42801498	-0.5669236	0.00257384	0.00774153	0.41	0.32	1.4	1.26	AP003419.11	--	chr11	protein_coding
ENSG00000005483.18	1.64183248	3.33223126	7.34E-08	5.02E-07	0.37	0.73	1.78	2.79	KMT2E	lysine (K)-specific methyltransferase 2E	chr7	protein_coding
ENSG00000169228.12	-1.9292961	5.6087811	7.12E-11	7.27E-10	43.34	44.8	16.08	14.71	RAB24	RAB24, member RAS oncogene family	chr5	protein_coding
ENSG00000175426.9	-4.3737486	2.18718868	2.08E-30	9.74E-29	1.46	1.27	0.06	0.12	PCSK1	proprotein convertase subtilisin/kexin type 1	chr5	protein_coding
ENSG00000122877.12	-7.878979	-1.0805299	3.60E-13	4.72E-12	0.18	0.28	0	0	EGR2	early growth response 2	chr10	protein_coding
ENSG00000236499.2	-6.3596049	-2.3204118	8.95E-06	4.38E-05	0.08	0.16	0	0	LINC00896	long intergenic non-protein coding RNA 896	chr22	lincRNA
ENSG00000270087.4	-1.5780714	0.54246003	2.26E-05	0.0001029	4.16	2.71	1.15	1.9	RP11-399K2	--	chr10	lincRNA
ENSG00000196544.7	-1.2029449	4.30730541	4.28E-05	0.00018583	9.82	7.49	4.99	5.04	C17orf59	chromosome 17 open reading frame 59	chr17	protein_coding
ENSG00000080224.16	-3.4257009	-1.3899929	1.32E-06	7.43E-06	0.14	0.06	0.02	0	EPHA6	EPH receptor A6	chr3	protein_coding
ENSG00000173083.13	2.01173809	3.06553087	1.36E-10	1.34E-09	0.53	0.59	3.12	2.93	HPSE	heparanase	chr4	protein_coding
ENSG00000100399.14	4.41039519	-0.5173916	3.42E-12	4.04E-11	0	0.03	0.52	0.47	CHADL	chondroadherin-like	chr22	protein_coding
ENSG00000204291.9	-5.5294056	5.48039189	3.39E-54	6.06E-52	11.71	14.02	0.39	0.35	COL15A1	collagen, type XV, alpha 1	chr9	protein_coding
ENSG00000267898.1	-2.1134665	-1.2916669	0.00018398	0.00069885	0.42	0.76	0.08	0.27	CTD-2639E6	--	chr19	lincRNA
ENSG00000253427.1	7.55588405	-1.1880946	2.11E-11	2.27E-10	0	0	1.78	1.9	RP11-103H7	--	chr8	lincRNA
ENSG00000088538.12	1.87813508	1.56448129	3.82E-08	2.69E-07	0.13	0.09	0.52	0.59	DOCK3	dedicator of cytokinesis 3	chr3	protein_coding
ENSG00000163755.7	1.50146581	2.06345426	3.53E-06	1.87E-05	0.32	0.43	0.89	1.91	HPS3	Hermansky-Pudlak syndrome 3	chr3	protein_coding
ENSG00000169246.15	-1.3095979	0.59086026	0.00027889	0.00102146	0.49	0.84	0.25	0.46	NPIPB3	nuclear pore complex interacting protein family, member B3	chr16	protein_coding
ENSG00000086544.2	-1.0320159	5.08370525	0.00037442	0.00134103	12.26	9.48	7	7.18	ITPKC	inositol-trisphosphate 3-kinase C	chr19	protein_coding
ENSG00000157445.13	-4.6342835	4.00842494	1.34E-39	9.95E-38	6.23	6.97	0.42	0.29	CACNA2D3	calcium channel, voltage-dependent, alpha 2/delta subunit 3	chr3	protein_coding
ENSG00000113580.13	1.17868523	3.00865627	0.0001098	0.00043765	0.4	0.56	1.07	1.85	NR3C1	nuclear receptor subfamily 3, group C, member 1 (glucocorticoid receptor)	chr5	protein_coding
ENSG00000151617.14	-7.0918936	-1.757976	1.16E-08	8.76E-08	0.05	0.15	0	0	EDNRA	endothelin receptor type A	chr4	protein_coding
ENSG00000108679.11	-5.675698	8.80188286	4.05E-60	9.70E-58	278.16	271.93	7.33	7	LGALS3BP	lectin, galactoside-binding, soluble, 3 binding protein	chr17	protein_coding
ENSG00000232725.1	-7.1822848	-1.6830765	4.48E-09	3.59E-08	0.3	0.52	0	0	U52111.14	--	chrX	antisense
ENSG00000267248.1	-1.5745828	0.53278625	2.59E-05	0.00011644	1.36	3.03	0.98	0.97	CTD-2319I1	--	chr17	processed_transcript
ENSG00000188112.7	-3.6261118	3.6135534	1.59E-27	6.48E-26	2.73	2.63	0.27	0.31	C6orf132	chromosome 6 open reading frame 132	chr6	protein_coding
ENSG00000260597.1	3.19913209	-1.9362984	0.00010192	0.00041007	0.02	0	0.17	0.11	AC012531.25	--	chr12	lincRNA
ENSG00000207165.1	-1.7661395	0.76327799	1.43E-06	8.03E-06	17.83	13.02	6.88	5.24	SNORA70	small nucleolar RNA, H/ACA box 70	chrX	snoRNA
ENSG00000239653.1	5.44044058	-2.7547732	0.00290522	0.00856312	0	0	0	0.13	PSMD6-AS2	PSMD6 antisense RNA 2	chr3	antisense
ENSG00000278882.1	2.58170598	-0.5374312	5.09E-07	3.08E-06	0.02	0.23	1.24	0.75	AC253572.1	--	chr1	protein_coding
ENSG00000107897.17	1.85396567	2.17119944	1.22E-08	9.24E-08	0.35	0.37	1.2	2.3	ACBD5	acyl-CoA binding domain containing 5	chr10	protein_coding
ENSG00000141452.8	1.15936715	3.96649184	8.96E-05	0.00036291	3.68	3.31	9.02	11.85	C18orf8	chromosome 18 open reading frame 8	chr18	protein_coding
ENSG00000112210.10	1.31876369	0.4164307	0.00063328	0.00217307	0.11	0.13	0.28	0.5	RAB23	RAB23, member RAS oncogene family	chr6	protein_coding
ENSG00000184384.12	-1.1596065	0.22181677	0.00271461	0.00813064	0.09	0.26	0.1	0.11	MAML2	mastermind-like 2 (Drosophila)	chr11	protein_coding
ENSG00000130702.12	-1.6372607	9.05520624	1.88E-08	1.38E-07	61.26	52.75	25.93	22.85	LAMA5	laminin, alpha 5	chr20	protein_coding
ENSG00000180066.8	-9.857546	0.80957498	1.17E-31	5.81E-30	1.52	1.55	0	0	C10orf91	chromosome 10 open reading frame 91	chr10	lincRNA
ENSG00000275179.1	-4.8088799	-3.2302406	0.01889873	0.04519557	0.04	0.21	0	0	RP4-737E23.	--	chr20	lincRNA
ENSG00000163781.11	2.39596899	2.57169214	2.76E-13	3.66E-12	0.2	0.36	1.58	2.37	TOPBP1	topoisomerase (DNA) II binding protein 1	chr3	protein_coding
ENSG00000114270.14	-1.8860761	10.0126071	1.27E-10	1.26E-09	141.4	142.46	49.68	52.71	COL7A1	collagen, type VII, alpha 1	chr3	protein_coding
ENSG00000103044.9	3.63433552	0.68770824	4.61E-16	8.07E-15	0.03	0.05	0.64	0.6	HAS3	hyaluronan synthase 3	chr16	protein_coding
ENSG00000164266.9	-3.5975993	-2.0191312	6.40E-05	0.00026805	0.29	0.12	0.04	0	SPINK1	serine peptidase inhibitor, Kazal type 1	chr5	protein_coding
ENSG00000259877.2	1.02385611	0.95117888	0.0035729	0.01034915	0.49	0.31	1.05	1.15	RP11-46C24	--	chr16	antisense
ENSG00000248871.1	1.86519934	3.2588226	1.56E-09	1.34E-08	1.4	2.25	9.77	7.84	TNFSF12-TN	TNFSF12-TNFSF13 readthrough	chr17	protein_coding
ENSG00000100796.16	1.59220696	3.95776904	1.08E-07	7.21E-07	1	1.79	3.92	7.32	SMEK1	SMEK homolog 1, suppressor of mek1 (Dictyostelium)	chr14	protein_coding
ENSG00000107317.10	-5.3733592	-2.9472019	0.00290522	0.00856312	0.2	0.11	0	0	PTGDS	prostaglandin D2 synthase 21kDa (brain)	chr9	protein_coding
ENSG00000105281.11	-1.3780906	9.78877649	1.86E-06	1.03E-05	313.96	306.82	160.48	157.66	SLC1A5	solute carrier family 1 (neutral amino acid transporter), member 5	chr19	protein_coding
ENSG00000258738.1	1.05692018	-0.5066505	0.02023038	0.04800659	0.18	0.13	0.26	0.6	RP11-73E17.	--	chr14	antisense
ENSG00000152601.16	2.78548888	1.38293952	8.60E-14	1.21E-12	0.07	0.08	0.49	0.92	MBNL1	muscleblind-like splicing regulator 1	chr3	protein_coding
ENSG00000174371.15	1.13255568	3.24948702	0.00017135	0.00065449	1.1	1.66	3.75	4.3	EXO1	exonuclease 1	chr1	protein_coding
ENSG00000142583.16	-4.0060906	3.2125907	2.12E-30	9.91E-29	2.67	2.24	0.26	0.15	SLC2A5	solute carrier family 2 (facilitated glucose/fructose transporter), member 5	chr1	protein_coding
ENSG00000196154.10	-6.3981791	6.97981925	1.56E-68	5.66E-66	267.39	220.72	3.99	3.72	S100A4	S100 calcium binding protein A4	chr1	protein_coding
ENSG00000270276.2	1.79121233	3.06371351	7.47E-09	5.83E-08	2.86	2.43	11.52	12.94	HIST2H4B	histone cluster 2, H4b	chr1	protein_coding
ENSG00000267121.4	-1.4506951	2.44351985	3.36E-06	1.78E-05	1.37	2.1	0.73	0.95	CTD-2020K1	--	chr17	antisense
ENSG00000278311.3	1.23191476	4.8311857	2.50E-05	0.00011287	2.57	3.18	7.62	10.42	GGNBP2	gametogenetin binding protein 2	chr17	protein_coding
ENSG00000276255.2	5.35410067	-2.8076728	0.00290522	0.00856312	0	0	0.08	0.04	RP5-881P19.	--	chr1	lincRNA
ENSG00000200084.1	1.52965572	-0.3113087	0.00052551	0.00182996	4.78	2.36	20.97	7.13	SNORD68	small nucleolar RNA, C/D box 68	chr16	snoRNA
ENSG00000215067.8	1.51565426	1.15898562	1.62E-05	7.55E-05	0.18	0.38	1.07	1.07	ALOX12-AS1	ALOX12 antisense RNA 1	chr17	antisense
ENSG00000261251.1	2.59472318	-0.9745408	5.07E-06	2.61E-05	0.05	0.04	0.42	0.38	RP3-388M5.	--	chr22	antisense
ENSG00000007402.10	5.58895981	2.38926269	4.38E-38	2.99E-36	0.01	0.04	1.88	1.93	CACNA2D2	calcium channel, voltage-dependent, alpha 2/delta subunit 2	chr3	protein_coding

ENSG00000170835.13	2.62245609	0.2656336	1.07E-09	9.33E-09	0.02	0.17	0.92	0.62	CEL	carboxyl ester lipase	chr9	protein_coding
ENSG00000152939.13	1.93299337	1.4304795	3.01E-08	2.16E-07	0.13	0.26	0.74	1.27	MARVELD2	MARVEL domain containing 2	chr5	protein_coding
ENSG00000168439.15	-1.0357901	9.33434763	0.00030502	0.00110887	235.46	238.96	157.97	150.08	STIP1	stress-induced phosphoprotein 1	chr11	protein_coding
ENSG00000266953.5	-3.7199094	1.74394202	5.22E-23	1.57E-21	8.03	7.42	0.49	1.04	RP11-618P1	--	chr19	protein_coding
ENSG00000245680.8	-3.233606	-1.5463486	2.35E-06	1.28E-05	0.03	0.11	0.01	0	ZNF585B	zinc finger protein 585B	chr19	protein_coding
ENSG00000174501.13	8.02970686	-0.7755719	3.61E-13	4.73E-12	0	0	0.15	0.21	ANKRD36C	ankyrin repeat domain 36C	chr2	protein_coding
ENSG00000204386.9	1.22876587	5.79131048	2.28E-05	0.00010343	14.24	11.92	41.87	39.84	NEU1	sialidase 1 (lysosomal sialidase)	chr6	protein_coding
ENSG00000103326.9	-1.310845	5.95961406	6.30E-06	3.18E-05	15.79	14.47	7.99	8.27	CAPN15	calpain 15	chr16	protein_coding
ENSG00000169607.11	1.17411841	1.59516326	0.0003923	0.00140048	0.35	0.29	0.62	1.32	CKAP2L	cytoskeleton associated protein 2-like	chr2	protein_coding
ENSG00000125246.14	-1.456494	1.94103217	6.59E-06	3.31E-05	0.7	0.64	0.35	0.29	CLYBL	citrate lyase beta like	chr13	protein_coding
ENSG00000175544.12	-1.4240064	0.37297385	0.00018285	0.00069522	0.54	0.49	0.26	0.26	CABP4	calcium binding protein 4	chr11	protein_coding
ENSG00000123496.6	7.1000136	-1.5659428	7.18E-09	5.62E-08	0	0	0.32	0.42	IL13RA2	interleukin 13 receptor, alpha 2	chrX	protein_coding
ENSG00000112655.14	-1.4666799	6.7180064	4.52E-07	2.75E-06	23.38	19.5	10.91	9.75	PTK7	protein tyrosine kinase 7 (inactive)	chr6	protein_coding
ENSG00000147133.14	1.51091483	4.72626296	3.07E-07	1.92E-06	1.35	1.53	4.79	6.15	TAF1	TAF1 RNA polymerase II, TATA box binding protein (TBP)-associated factor, 250kDa	chrX	protein_coding
ENSG00000166813.13	1.23684844	7.01234436	1.85E-05	8.57E-05	13.26	14.09	42.95	42.92	KIF7	kinesin family member 7	chr15	protein_coding
ENSG00000255916.1	6.4429151	-2.0781831	3.03E-06	1.62E-05	0	0	0.61	0.86	RP13-895J2.	--	chr12	antisense
ENSG00000160867.13	-2.7718226	4.27064555	6.94E-19	1.54E-17	8.9	8.15	1.67	1.65	FGFR4	fibroblast growth factor receptor 4	chr5	protein_coding
ENSG00000189419.6	3.01014089	-1.3229691	9.86E-06	4.79E-05	0.03	0.03	0.32	0.28	SPATA41	spermatogenesis associated 41 (non-protein coding)	chr15	lincRNA
ENSG00000144306.12	1.81982077	0.74907842	1.36E-06	7.65E-06	0.16	0.21	0.5	1.26	SCRN3	secernin 3	chr2	protein_coding
ENSG00000120318.14	-1.7236872	5.19046842	5.04E-09	4.03E-08	9.27	7.91	3.53	3.4	ARAP3	ArfGAP with RhoGAP domain, ankyrin repeat and PH domain 3	chr5	protein_coding
ENSG00000161835.9	2.53627021	-2.3705245	0.00737015	0.01977584	0	0.03	0.06	0.16	GRASP	GRP1 (general receptor for phosphoinositides 1)-associated scaffold protein	chr12	protein_coding
ENSG00000237357.2	3.85863134	-0.3591689	7.34E-12	8.35E-11	0.03	0.02	0.39	0.69	RP11-475I24	--	chr9	lincRNA
ENSG00000261071.1	1.12583411	-0.5143489	0.01510105	0.03730772	0.26	0.35	0.4	1.36	RP1-223E5.4	--	chr6	antisense
ENSG00000053918.14	-1.1829426	1.11472743	0.0004924	0.00172092	0.67	0.69	0.36	0.43	KCNQ1	potassium channel, voltage gated KQT-like subfamily Q, member 1	chr11	protein_coding
ENSG00000149201.8	-2.6311557	-1.9785986	0.00039269	0.00140167	0.09	0.11	0.02	0.02	CCDC81	coiled-coil domain containing 81	chr11	protein_coding
ENSG00000165775.16	-1.16184	5.4203566	6.19E-05	0.00026003	7.44	7.4	3.96	4.89	FUNDC2	FUN14 domain containing 2	chrX	protein_coding
ENSG00000059915.15	1.81353488	-0.5187118	0.00013398	0.0005254	0.09	0.01	0.16	0.32	PSD	pleckstrin and Sec7 domain containing	chr10	protein_coding
ENSG00000172869.13	2.21043855	1.62047012	1.74E-10	1.68E-09	0.08	0.07	0.36	0.58	DMXL1	Dmx-like 1	chr5	protein_coding
ENSG00000159714.9	-2.2803732	-0.1175828	1.46E-07	9.57E-07	0.6	0.57	0.19	0.13	ZDHHC1	zinc finger, DHHC-type containing 1	chr16	protein_coding
ENSG00000147324.9	-1.2264487	4.23557846	3.07E-05	0.00013604	3.33	3.32	1.77	2.02	MFHAS1	malignant fibrous histiocytoma amplified sequence 1	chr8	protein_coding
ENSG00000198883.10	-1.6073509	-1.3742277	0.0065433	0.01777607	0.12	0.12	0.07	0.03	PNMA5	paraneoplastic Ma antigen family member 5	chrX	protein_coding
ENSG00000275234.1	5.05186882	-2.9771646	0.0100455	0.02582742	0	0	0.09	0.28	CTD-2396E7	--	chr19	antisense
ENSG00000154639.17	3.12286407	1.43031516	2.23E-16	4.00E-15	0.04	0.09	0.58	0.93	CXADR	coxsackie virus and adenovirus receptor	chr21	protein_coding
ENSG00000258941.3	2.55519371	2.20630286	4.13E-14	5.99E-13	0.29	0.19	1.36	2.44	RP11-407N1	--	chr14	protein_coding
ENSG00000267952.1	-1.0166034	5.58069152	0.00043628	0.00154347	127.01	144.52	93.83	84.62	CTD-2207O2	--	chr19	protein_coding
ENSG00000154040.19	4.85744779	2.71090737	1.31E-35	7.93E-34	0.16	0.13	5.92	5.19	CABYR	calcium binding tyrosine-(Y)-phosphorylation regulated	chr18	protein_coding
ENSG00000151413.15	1.68616497	1.69105824	4.57E-07	2.78E-06	0.32	0.47	1.25	2.14	NUBPL	nucleotide binding protein-like	chr14	protein_coding
ENSG00000134294.12	2.07464785	5.26637077	3.61E-12	4.26E-11	2.21	2.56	8.68	18.29	SLC38A2	solute carrier family 38, member 2	chr12	protein_coding
ENSG00000159905.13	1.02542841	0.3745101	0.00672049	0.01821738	0.15	0.34	0.65	0.65	ZNF221	zinc finger protein 221	chr19	protein_coding
ENSG00000165983.13	2.17693306	0.9503497	4.80E-09	3.84E-08	0.18	0.1	0.59	1.1	PTER	phosphotriesterase related	chr10	protein_coding
ENSG00000118276.10	3.68178275	-1.5721729	8.20E-06	4.05E-05	0	0.01	0.07	0.19	B4GALT6	UDP-Gal:betaGlcNAc beta 1,4- galactosyltransferase, polypeptide 6	chr18	protein_coding
ENSG00000170006.10	7.30963459	-1.4001605	4.49E-10	4.11E-09	0	0	0.08	0.04	TMEM154	transmembrane protein 154	chr4	protein_coding
ENSG00000174130.11	-3.1050525	-1.6603865	8.20E-06	4.05E-05	0.05	0.08	0	0.02	TLR6	toll-like receptor 6	chr4	protein_coding
ENSG00000274825.1	1.5347244	-0.4249678	0.00075536	0.00255504	0.42	0.44	1.61	1.75	RP4-616B8.5	--	chr20	lincRNA
ENSG00000196954.11	1.69526939	5.19724738	8.99E-09	6.94E-08	6.31	6.06	23.36	30.18	CASP4	caspase 4, apoptosis-related cysteine peptidase	chr11	protein_coding
ENSG00000120057.4	5.45974547	-2.7440718	0.00158008	0.00495521	0	0	0.07	0.12	SFRP5	secreted frizzled-related protein 5	chr10	protein_coding
ENSG00000261220.2	-4.5649938	3.5501361	2.91E-37	1.91E-35	21.82	31.93	2.03	1	RP11-629O1	--	chr8	lincRNA
ENSG00000151012.12	-1.2785794	2.63606961	3.42E-05	0.00015064	0.68	0.79	0.42	0.39	SLC7A11	solute carrier family 7 (anionic amino acid transporter light chain, xc- system), member 11	chr4	protein_coding
ENSG00000101384.10	-2.4737082	2.67345178	2.14E-14	3.21E-13	1.23	1.66	0.33	0.36	JAG1	jagged 1	chr20	protein_coding
ENSG00000158715.5	-1.3715838	2.95831231	6.94E-06	3.47E-05	3.04	2.37	1.28	1.52	SLC45A3	solute carrier family 45, member 3	chr1	protein_coding
ENSG00000184719.10	-6.8795944	-1.9267926	1.35E-07	8.87E-07	0.12	0.06	0	0	RNLS	renalase, FAD-dependent amine oxidase	chr10	protein_coding
ENSG00000123219.11	2.26694407	1.0319227	1.29E-09	1.11E-08	0.13	0.3	0.84	1.92	CENPK	centromere protein K	chr5	protein_coding
ENSG00000172965.13	-1.2698171	-0.4980599	0.00434978	0.01238249	0.25	0.5	0.28	0.13	MIR4435-1H	--	chr2	lincRNA
ENSG00000196275.12	2.22509189	-1.8861128	0.00445943	0.01266454	0.02	0.01	0.12	0.05	GTF2IRD2	GTF2I repeat domain containing 2	chr7	protein_coding
ENSG00000183421.10	1.22397032	3.39487424	4.63E-05	0.00019983	1.3	1.21	4.03	3.78	RIPK4	receptor-interacting serine-threonine kinase 4	chr21	protein_coding
ENSG00000118873.14	1.19353241	2.10924339	0.00017055	0.00065152	0.27	0.31	0.71	1.04	RAB3GAP2	RAB3 GTPase activating protein subunit 2 (non-catalytic)	chr1	protein_coding
ENSG00000196420.6	-4.7353727	-1.8450712	8.18E-08	5.55E-07	0.55	0.4	0	0.03	S100A5	S100 calcium binding protein A5	chr1	protein_coding
ENSG00000153071.13	-3.224238	3.22426297	2.13E-22	6.16E-21	2.27	3.69	0.56	0.29	DAB2	Dab, mitogen-responsive phosphoprotein, homolog 2 (Drosophila)	chr5	protein_coding
ENSG00000250999.1	3.53780763	-2.3438418	0.00039332	0.00140265	0	0.03	0.62	0.03	RP11-1379J2	--	chr5	antisense
ENSG00000221890.2	3.03952849	5.35281605	1.91E-22	5.53E-21	1.27	1.2	14.53	12.51	NPTXR	neuronal pentraxin receptor	chr22	protein_coding
ENSG00000175832.11	-4.7844372	7.09889475	3.67E-46	4.09E-44	78.5	74.25	4.23	3.14	ETV4	ets variant 4	chr17	protein_coding
ENSG00000049323.14	-1.8664942	4.74786807	3.77E-10	3.50E-09	5.4	4.81	1.78	1.95	LTBP1	latent transforming growth factor beta binding protein 1	chr2	protein_coding
ENSG00000103160.10	2.06763378	2.90106743	5.86E-11	6.02E-10	0.73	0.57	3.14	4.17	HSDL1	hydroxysteroid dehydrogenase like 1	chr16	protein_coding
ENSG00000280433.1	1.93812338	-1.0216093	0.0005219	0.00181847	0.03	0.05	0.25	0.15	CH507-9B2.9	--	chr21	protein_coding
ENSG00000107779.10	2.03591564	2.00622684	9.81E-10	8.62E-09	0.1	0.12	0.52	0.69	BMPR1A	bone morphogenetic protein receptor, type IA	chr10	protein_coding
ENSG00000102313.8	4.88946445	-3.0622981	0.01889873	0.04519557	0	0	0.01	0.03	ITIH6	inter-alpha-trypsin inhibitor heavy chain family, member 6	chrX	protein_coding
ENSG00000110700.5	-1.3685418	8.06484823	2.23E-06	1.22E-05	528.45	438.21	247.58	251.75	RPS13	ribosomal protein S13	chr11	protein_coding
ENSG00000132000.10	1.34236388	1.66149752	4.65E-05	0.00020059	0.32	0.51	1.67	1.14	PODNL1	podocan-like 1	chr19	protein_coding
ENSG00000108582.10	1.80727102	3.4270132	3.82E-09	3.11E-08	0.36	0.42	1.46	2.21	CPD	carboxypeptidase D	chr17	protein_coding
ENSG00000171401.13	2.10857651	-1.1676326	0.00021372	0.00080378	0	0.12	0.36	0.34	KRT13	keratin 13, type I	chr17	protein_coding
ENSG00000141449.13	-6.7907586	-0.0291396	2.24E-22	6.45E-21	0.2	0.15	0	0	GREB1L	growth regulation by estrogen in breast cancer-like	chr18	protein_coding
ENSG00000243560.3	3.29543728	-1.8711151	0.00016407	0.00062818	0	0.21	2.94	0.1	RN7SL364P	RNA, 7SL, cytoplasmic 364, pseudogene	chr19	misc_RNA
ENSG00000116254.16	3.24660152	2.52659156	8.18E-21	2.09E-19	0.07	0.11	1.32	0.96	CHD5	chromodomain helicase DNA binding protein 5	chr1	protein_coding
ENSG00000215039.5	2.06192669	0.30979214	4.56E-07	2.78E-06	0.2	0.21	1.16	1.11	CD27-AS1	CD27 antisense RNA 1	chr12	processed_transcript
ENSG00000101844.16	1.01427571	1.52755364	0.00212515	0.00648803	0.66	0.64	1.23	2.27	ATG4A	autophagy related 4A, cysteine peptidase	chrX	protein_coding
ENSG00000179941.6	2.77732768	-0.3806612	3.60E-08	2.55E-07	0.04	0.04	0.3	0.41	BBS10	Bardet-Biedl syndrome 10	chr12	protein_coding
ENSG00000138768.13	2.43047888	2.61198238	9.76E-14	1.37E-12	0.3	0.43	1.8	3.51	USO1	USO1 vesicle transport factor	chr4	protein_coding
ENSG00000171124.11	-5.2054745	-3.0371681	0.0100455	0.02582742	0.08	0	0	0	FUT3	fucosyltransferase 3 (galactoside 3(4)-L-fucosyltransferase, Lewis blood group)	chr19	protein_coding
ENSG00000146054.16	-2.2972375	5.68738873	1.80E-14	2.72E-13	14.73	13.96	4.25	3.51	TRIM7	tripartite motif containing 7	chr5	protein_coding
ENSG00000006459.9	1.5787894	0.09002256	0.00011265	0.00044808	0.06	0.02	0.22	0.12	KDM7A	lysine (K)-specific demethylase 7A	chr7	protein_coding
ENSG00000106123.10	9.57471189	0.62945023	2.92E-27	1.18E-25	0	0	0.94	0.6	EPHB6	EPH receptor B6	chr7	protein_coding
ENSG00000104687.11	-1.6671786	6.14427626	1.23E-08	9.30E-08	27.11	28.75	9.28	14.27	GSR	glutathione reductase	chr8	protein_coding
ENSG00000215246.5	-3.2172184	-0.8841202	2.14E-08	1.56E-07	0.16	0.27	0.04	0.02	RP11-43F13.	--	chr5	antisense
ENSG00000110442.10	-1.0761891	4.40480931	0.00024045	0.0008974	8.23	7.39	5.28	4.58	COMMD9	COMM domain containing 9	chr11	protein_coding
ENSG00000229474.5	6.23678324	-2.2266073	1.55E-05	7.26E-05	0	0	0.06	0.22	PATL2	protein associated with topoisomerase II homolog 2 (yeast)	chr15	protein_coding
ENSG00000122257.17	1.85032266	4.30850229	7.33E-10	6.50E-09	1.01	1.08	3.58	6.5	RBBP6	retinoblastoma binding protein 6	chr16	protein_coding
ENSG00000275591.3	4.12800318	-0.1329594	5.23E-14	7.51E-13	0.02	0.01	0.36	0.32	XKR5	XK, Kell blood group complex subunit-related family, member 5	chr8	protein_coding
ENSG00000204843.11	-1.1286753	9.11198849	8.60E-05	0.00034923	110.71	126.41	73.51	70.79	DCTN1	dynactin 1	chr2	protein_coding
ENSG00000105426.13	-1.7850536	7.15973168	1.12E-09	9.70E-09	24.54	21.57	9.53	8.27	PTPRS	protein tyrosine phosphatase, receptor type, S	chr19	protein_coding
ENSG00000165655.15	-2.0378178	5.24169478	7.50E-12	8.51E-11	14.92	14.51	4.06	5.52	ZNF503	zinc finger protein 503	chr10	protein_coding
ENSG00000105695.13	6.9396867	-1.6948104	3.05E-08	2.18E-07	0	0	0.17	0.23	MAG	myelin associated glycoprotein	chr19	protein_coding
ENSG00000157625.14	1.07748727	1.15271142	0.00176569	0.00547217	0.18	0.16	0.47	0.46	TAB3	TGF-beta activated kinase 1/MAP3K7 binding protein 3	chrX	protein_coding
ENSG00000187583.9	1.1308658	3.84555479	0.00013542	0.00053051	2.24	3.44	8.41	8.12	PLEKHN1	pleckstrin homology domain containing, family N member 1	chr1	protein_coding
ENSG00000188283.10	1.3032014	0.27703906	0.00094465	0.00312013	0.04	0.11	0.1	0.38	ZNF383	zinc finger protein 383	chr19	protein_coding
ENSG00000155744.8	2.42215133	0.49053374	3.23E-09	2.65E-08	0.03	0.04	0.19	0.32	FAM126B	family with sequence similarity 126, member B	chr2	protein_coding
ENSG00000213760.9	-1.5388785	-1.6279969	0.01700209	0.04149604	0.24	0.18	0.16	0.03	ATP6V1G2	ATPase, H+ transporting, lysosomal 13kDa, V1 subunit G2	chr6	protein_coding
ENSG00000075568.15	1.28024901	3.64031748	1.85E-05	8.57E-05	0.81	0.96	2.49	3.22	TMEM131	transmembrane protein 131	chr2	protein_coding
ENSG00000151702.15	-5.3349278	-1.3513739	8.29E-10	7.32E-09	0.07	0.18	0.01	0	FLI1	Fli-1 proto-oncogene, ETS transcription factor	chr11	protein_coding
ENSG00000205189.10	1.25159292	-0.0369047	0.00245879	0.00741317	0.04	0.05	0.06	0.21	ZBTB10	zinc finger and BTB domain containing 10	chr8	protein_coding
ENSG00000138035.13	1.51565978	2.19018967	2.27E-06	1.24E-05	0.41	0.44	1.15	2.1	PNPT1	polyribonucleotide nucleotidyltransferase 1	chr2	protein_coding
ENSG00000143322.18	1.45234274	4.30324177	9.49E-07	5.48E-06	0.7	0.65	2.49	2.42	ABL2	ABL proto-oncogene 2, non-receptor tyrosine kinase	chr1	protein_coding
ENSG00000179588.7	1.41365927	4.75629203	1.49E-06	8.36E-06	2.03	1.99	7.92	6.32	ZFPM1	zinc finger protein, FOG family member 1	chr16	protein_coding
ENSG00000173818.15	1.07075338	3.45818653	0.00033569	0.00121321	1.51	1.05	3.75	3.43	ENDOV	endonuclease V	chr17	protein_coding
ENSG00000134827.6	7.28555401	-1.4088394	7.05E-10	6.26E-09	0	0	0.2	0.59	TCN1	transcobalamin I (vitamin B12 binding protein, R binder family)	chr11	protein_coding
ENSG00000103184.10	-2.210245	-1.112512	4.42E-05	0.00019159	0.09	0.09	0	0.04	SEC14L5	SEC14-like 5 (S. cerevisiae)	chr16	protein_coding
ENSG00000180535.3	-3.6176375	0.48331527	4.63E-16	8.09E-15	3.15	2.02	0.32	0.24	BHLHA15	basic helix-loop-helix family, member a15	chr7	protein_coding
ENSG00000205089.6	3.5481151	-1.2333271	6.12E-07	3.65E-06	0.02	0.01	0.23	0.29	CCNI2	cyclin I family, member 2	chr5	protein_coding
ENSG00000117868.14	1.23205148	5.66525101	2.20E-05	0.0001001	4.11	3.81	10.89	13.99	ESYT2	extended synaptotagmin-like protein 2	chr7	protein_coding
ENSG00000276952.1	2.0104855	-2.0217538	0.01397446	0.03477946	0.08	0.08	0.43	0.48	RP5-965G21	--	chr20	antisense
ENSG00000159873.8	1.12386471	3.31023436	0.0001829	0.0006953	1.11	1.4	2.96	4.35	CCDC117	coiled-coil domain containing 117	chr22	protein_coding
ENSG00000101493.9	2.37411487	-1.5313682	0.00053182	0.00185033	0.01	0.01	0.04	0.08	ZNF516	zinc finger protein 516	chr18	protein_coding
ENSG00000115145.8	1.57002728	0.13796903	0.00011721	0.00046471	0.08	0.07	0.2	0.37	STAM2	signal transducing adaptor molecule (SH3 domain and ITAM motif) 2	chr2	protein_coding
ENSG00000125351.9	2.03177731	4.55776913	1.55E-11	1.70E-10	2.53	3.75	13.42	20.94	UPF3B	UPF3 regulator of nonsense transcripts homolog B (yeast)	chrX	protein_coding
ENSG00000168795.4	-1.2172833	3.29067076	5.20E-05	0.00022127	2.39	2.38	1.27	1.47	ZBTB5	zinc finger and BTB domain containing 5	chr9	protein_coding
ENSG00000136928.4	3.22901852	-2.5375541	0.003237	0.00945156	0	0	0.04	0.03	GABBR2	gamma-aminobutyric acid (GABA) B receptor, 2	chr9	protein_coding
ENSG00000257605.2	-2.4713175	-0.6668473	1.51E-06	8.43E-06	0.54	2.01	0.45	0.17	RP11-680A1	--	chr12	antisense
ENSG00000135392.14	-1.526037	5.44126445	1.84E-07	1.19E-06	14.17	13.3	5.94	6.79	DNAJC14	DnaJ (Hsp40) homolog, subfamily C, member 14	chr12	protein_coding
ENSG00000107249.20	-2.4466677	-0.8089875	2.01E-06	1.10E-05	0.04	0.15	0.02	0.02	GLIS3	GLIS family zinc finger 3	chr9	protein_coding
ENSG00000115935.15	-5.751021	3.48228397	1.15E-48	1.45E-46	2.39	2.99	0.05	0.08	WIPF1	WAS/WASL interacting protein family, member 1	chr2	protein_coding
ENSG00000061987.13	1.57039348	1.96149795	1.65E-06	9.18E-06	0.08	0.15	0.35	0.56	MON2	MON2 homolog (S. cerevisiae)	chr12	protein_coding
ENSG00000237943.5	-7.3423127	-1.5467334	7.05E-10	6.26E-09	0.66	0.2	0	0	PRKCQ-AS1	PRKCQ antisense RNA 1	chr10	processed_transcript
ENSG00000234840.1	-2.6275119	-2.643781	0.00552196	0.01522223	0.06	0.07	0	0.02	LINC01239	long intergenic non-protein coding RNA 1239	chr9	lincRNA
ENSG00000114450.8	1.00646925	2.10603569	0.00151897	0.00482098	0.35	0.33	0.72	1.13	GNB4	guanine nucleotide binding protein (G protein), beta polypeptide 4	chr3	protein_coding
ENSG00000273174.1	5.19748351	-2.8969879	0.00538165	0.01488851	0	0	0.11	0.39	RP11-434H6	--	chr3	antisense
ENSG00000165238.15	-1.9595534	3.90413941	1.07E-10	1.06E-09	2.6	2.64	0.99	0.81	WNK2	WNK lysine deficient protein kinase 2	chr9	protein_coding
ENSG00000248161.4	-1.3151634	-0.4971444	0.00249754	0.00752149	1.39	0.88	0.52	0.69	RP11-499E18	--	chr4	lincRNA
ENSG00000136143.12	1.69929797	1.49328403	6.85E-07	4.05E-06	0.41	0.55	1.24	2.92	SUCLA2	succinate-CoA ligase, ADP-forming, beta subunit	chr13	protein_coding
ENSG00000205609.11	-2.151686	1.31748873	1.05E-09	9.19E-09	0.91	1.21	0.35	0.28	EIF3CL	eukaryotic translation initiation factor 3, subunit C-like	chr16	protein_coding
ENSG00000241316.5	3.15648413	-1.5386704	2.06E-05	9.41E-05	0	0.04	0.33	0.15	SUCLG2-AS1	SUCLG2 antisense RNA 1 (head to head)	chr3	lincRNA
ENSG00000189223.12	2.52973381	3.29562674	1.87E-15	3.12E-14	0.12	0.17	1.07	1.2	PAX8-AS1	PAX8 antisense RNA 1	chr2	processed_transcript
ENSG00000151882.10	4.35653639	2.18702994	8.49E-29	3.69E-27	0.08	0.12	2.14	3.31	CCL28	chemokine (C-C motif) ligand 28	chr5	protein_coding
ENSG00000227345.7	1.08785717	2.07426429	0.00061453	0.00211296	0.44	0.56	1.5	1.32	PARG	poly (ADP-ribose) glycohydrolase	chr10	protein_coding
ENSG00000158458.18	6.50017466	0.88199023	1.01E-27	4.15E-26	0	0.01	0.81	1	NRG2	neuregulin 2	chr5	protein_coding
ENSG00000132744.6	7.01558508	-1.6405308	1.16E-08	8.76E-08	0	0	0.57	0.25	ACY3	aminoacylase 3	chr11	protein_coding
ENSG00000109618.10	1.28944846	-0.7334494	0.00624182	0.01703231	0.08	0.01	0.15	0.14	SEPSECS	Sep (O-phosphoserine) tRNA:Sec (selenocysteine) tRNA synthase	chr4	protein_coding
ENSG00000134590.12	-1.3990889	5.81421543	1.55E-06	8.65E-06	55.85	55.98	31.49	24.83	FAM127A	family with sequence similarity 127, member A	chrX	protein_coding
ENSG00000261572.1	5.56116133	-2.6812008	0.00086564	0.00288128	0	0	0.03	0.16	RP11-384L8.	--	chr3	sense_intronic
ENSG00000178035.10	-1.2761234	9.21152839	9.62E-06	4.69E-05	438.67	387.87	226.1	228.93	IMPDH2	IMP (inosine 5'-monophosphate) dehydrogenase 2	chr3	protein_coding
ENSG00000146243.12	1.72005924	-1.7633638	0.01347572	0.03367454	0	0.03	0.01	0.1	IRAK1BP1	interleukin-1 receptor-associated kinase 1 binding protein 1	chr6	protein_coding
ENSG00000271810.4	-2.6024674	-1.2091201	4.95E-06	2.55E-05	1.09	0.24	0.08	0.2	RP11-426L16	--	chr1	protein_coding
ENSG00000119715.13	5.18753465	-2.9024431	0.00538165	0.01488851	0	0	0	0.09	ESRRB	estrogen-related receptor beta	chr14	protein_coding
ENSG00000151689.11	-1.0063739	5.1151	0.00051616	0.00179899	17.45	17.16	10.83	12.16	INPP1	inositol polyphosphate-1-phosphatase	chr2	protein_coding
ENSG00000250208.5	-8.5988659	3.48674945	2.63E-65	8.42E-63	1.67	1.89	0	0.01	FZD10-AS1	FZD10 antisense RNA 1 (head to head)	chr12	processed_transcript
ENSG00000139192.10	4.81710279	4.32900207	3.04E-42	2.61E-40	0.44	0.82	25.76	21.59	TAPBPL	TAP binding protein-like	chr12	protein_coding
ENSG00000087266.14	-1.3566511	5.18858358	3.36E-06	1.78E-05	4.3	4.37	2.28	2.22	SH3BP2	SH3-domain binding protein 2	chr4	protein_coding
ENSG00000241343.8	-1.3934707	9.56509801	1.44E-06	8.09E-06	697.24	723.33	342.06	379.2	RPL36A	ribosomal protein L36a	chrX	protein_coding
ENSG00000262410.1	-2.4274129	-2.1221793	0.0014675	0.00467247	0.08	0.71	0	0.17	RP11-388C1	--	chr17	sense_intronic
ENSG00000063046.16	-1.1700157	8.42917641	4.79E-05	0.0002053	89.06	96.65	50.01	60.17	EIF4B	eukaryotic translation initiation factor 4B	chr12	protein_coding
ENSG00000214106.6	2.42894498	-0.9371145	2.16E-05	9.86E-05	0.02	0.06	0.37	0.18	PAXIP1-AS2	PAXIP1 antisense RNA 2	chr7	antisense
ENSG00000255872.3	-2.430836	6.00917693	6.26E-16	1.08E-14	33.98	33.93	7.74	9.07	RP11-613M1	--	chr9	protein_coding
ENSG00000164176.11	-9.0886857	0.05603648	2.93E-23	8.90E-22	0.39	0.31	0	0	EDIL3	EGF-like repeats and discoidin I-like domains 3	chr5	protein_coding
ENSG00000251259.1	-1.6907714	-1.4400038	0.00266587	0.00799727	0.97	0.46	0.29	0.3	AC004069.2	--	chr4	lincRNA
ENSG00000161955.15	1.90332496	0.70369468	4.67E-07	2.84E-06	0.2	0.26	1.24	1.06	TNFSF13	tumor necrosis factor (ligand) superfamily, member 13	chr17	protein_coding
ENSG00000081189.12	-2.8347667	-1.8534163	0.00010631	0.0004256	0.03	0.05	0	0.01	MEF2C	myocyte enhancer factor 2C	chr5	protein_coding
ENSG00000181444.11	-2.6188006	-0.0355981	3.93E-09	3.19E-08	0.53	0.44	0.15	0.06	ZNF467	zinc finger protein 467	chr7	protein_coding
ENSG00000204049.1	5.59946861	-2.6599164	0.00086564	0.00288128	0	0	0.17	0.04	RP11-126H7	--	chr10	antisense
ENSG00000187672.11	2.70262225	-1.8626855	0.00079104	0.00266183	0	0.01	0.06	0.06	ERC2	ELKS/RAB6-interacting/CAST family member 2	chr3	protein_coding
ENSG00000134758.12	3.01565424	0.16529543	8.63E-11	8.68E-10	0.01	0.09	0.3	0.79	RNF138	ring finger protein 138, E3 ubiquitin protein ligase	chr18	protein_coding
ENSG00000221817.8	1.72293407	0.15276687	2.80E-05	0.00012533	0.1	0.04	0.22	0.39	PPP3CB-AS1	PPP3CB antisense RNA 1 (head to head)	chr10	processed_transcript
ENSG00000228701.1	2.24254132	-1.2305571	0.00018398	0.00069885	0.04	0.22	0.59	1.04	TNKS2-AS1	TNKS2 antisense RNA 1 (head to head)	chr10	antisense
ENSG00000154856.11	2.15484468	-0.4309864	6.00E-06	3.04E-05	0.03	0.07	0.24	0.37	APCDD1	adenomatosis polyposis coli down-regulated 1	chr18	protein_coding
ENSG00000221963.5	2.5578694	3.85494999	3.17E-16	5.61E-15	0.33	0.34	2.49	2.75	APOL6	apolipoprotein L, 6	chr22	protein_coding
ENSG00000243836.4	-7.0852323	-1.7616814	1.16E-08	8.76E-08	0.51	0.36	0	0	WDR86-AS1	WDR86 antisense RNA 1	chr7	processed_transcript
ENSG00000215256.3	-1.2810789	3.45467638	1.89E-05	8.72E-05	3.28	3.71	1.64	2.2	DHRS4-AS1	DHRS4 antisense RNA 1	chr14	antisense
ENSG00000106034.16	-3.3303603	-2.2176817	8.40E-05	0.0003415	0.02	0.07	0	0.01	CPED1	cadherin-like and PC-esterase domain containing 1	chr7	protein_coding
ENSG00000258388.6	-1.3576396	3.13837517	7.38E-06	3.68E-05	2.22	2.68	1.24	1.3	PPT2-EGFL8	PPT2-EGFL8 readthrough (NMD candidate)	chr6	protein_coding
ENSG00000171130.16	1.88237216	4.73228979	3.04E-10	2.85E-09	2.98	3.33	17.05	13.83	ATP6V0E2	ATPase, H+ transporting V0 subunit e2	chr7	protein_coding
ENSG00000146463.10	1.64959715	2.64613941	1.40E-07	9.17E-07	0.33	0.36	1.3	1.59	ZMYM4	zinc finger, MYM-type 4	chr1	protein_coding
ENSG00000105793.14	1.68524659	0.50600371	1.18E-05	5.67E-05	0.07	0.06	0.17	0.42	GTPBP10	GTP-binding protein 10 (putative)	chr7	protein_coding
ENSG00000182272.10	-2.6535664	5.07642572	5.27E-18	1.09E-16	13.7	13.68	3.19	2.59	B4GALNT4	beta-1,4-N-acetyl-galactosaminyl transferase 4	chr11	protein_coding
ENSG00000138622.3	6.79854347	0.34541982	1.04E-22	3.03E-21	0	0	0.35	0.34	HCN4	hyperpolarization activated cyclic nucleotide gated potassium channel 4	chr15	protein_coding
ENSG00000198848.11	1.91624369	4.32741793	1.99E-10	1.91E-09	2.71	3.5	15.91	15.25	CES1	carboxylesterase 1	chr16	protein_coding
ENSG00000270761.1	1.68991264	-0.4548249	0.00019138	0.00072488	1.13	0.34	2.43	4.04	RP11-385F7.	--	chr6	lincRNA
ENSG00000274929.1	-2.1571239	-2.2833494	0.00854801	0.02266235	0.64	0.05	0	0.19	RP11-165D7	--	chr13	sense_intronic
ENSG00000198919.11	4.34354133	0.30286002	9.75E-17	1.81E-15	0	0.03	0.26	0.53	DZIP3	DAZ interacting zinc finger protein 3	chr3	protein_coding
ENSG00000131398.12	7.06023305	2.85304139	1.23E-49	1.63E-47	0.03	0	2.45	2.39	KCNC3	potassium channel, voltage gated Shaw related subfamily C, member 3	chr19	protein_coding
ENSG00000271781.1	1.30927581	3.35637273	1.46E-05	6.88E-05	4.67	7.31	19.72	19.73	CTD-2589H1	--	chr5	antisense
ENSG00000134317.16	3.39745364	-0.0010314	7.63E-12	8.65E-11	0.01	0.05	0.44	0.54	GRHL1	grainyhead-like 1 (Drosophila)	chr2	protein_coding
ENSG00000272692.1	2.25490086	-2.5391884	0.01923175	0.0458365	0.03	0	0.08	0.12	RP11-399K2	--	chr10	lincRNA
ENSG00000272711.1	-2.0573495	2.61528451	9.43E-11	9.47E-10	5.9	6.08	1.85	1.98	RP11-259N1	--	chr2	lincRNA
ENSG00000143774.15	-1.2763796	7.49586386	9.90E-06	4.80E-05	113.34	90.93	57.43	54.97	GUK1	guanylate kinase 1	chr1	protein_coding

ENSG00000280302.1	-5.2091082	-3.0354822	0.00538165	0.01488851	0.03	0.03	0	0	RP11-64C12	--	chr18	TEC
ENSG00000073146.14	-1.5318469	-1.5329408	0.01203274	0.03038863	0.07	0.05	0.03	0.02	MOV10L1	Mov10 RISC complex RNA helicase like 1	chr22	protein_coding
ENSG00000259207.6	-1.5224124	2.42856465	1.15E-06	6.55E-06	1.49	1.44	0.67	0.69	ITGB3	integrin, beta 3 (platelet glycoprotein IIIa, antigen CD61)	chr17	protein_coding
ENSG00000124171.7	3.31131784	0.50735042	1.01E-13	1.41E-12	0.02	0.06	0.55	0.55	PARD6B	par-6 family cell polarity regulator beta	chr20	protein_coding
ENSG00000171903.15	-6.5113106	-2.2102885	3.03E-06	1.62E-05	0.03	0.11	0	0	CYP4F11	cytochrome P450, family 4, subfamily F, polypeptide 11	chr19	protein_coding
ENSG00000165617.13	-3.2700954	2.54187827	1.28E-21	3.52E-20	1.75	2.15	0.27	0.27	DACT1	dishevelled-binding antagonist of beta-catenin 1	chr14	protein_coding
ENSG00000111799.19	1.32211457	8.03656661	4.75E-06	2.46E-05	9.76	10.29	30.51	36.42	COL12A1	collagen, type XII, alpha 1	chr6	protein_coding
ENSG00000175455.13	4.02664562	2.19498035	3.00E-26	1.12E-24	0.03	0.05	0.61	1.16	CCDC14	coiled-coil domain containing 14	chr3	protein_coding
ENSG00000183386.8	-3.2131869	4.55965516	4.79E-24	1.55E-22	22.47	19.36	3.23	2.78	FHL3	four and a half LIM domains 3	chr1	protein_coding
ENSG00000232220.2	-7.5289145	-1.3869791	7.65E-11	7.77E-10	1.96	0.67	0	0	AC008440.5	--	chr19	antisense
ENSG00000242349.4	3.1608275	-1.2051455	2.24E-06	1.22E-05	0.04	0.04	0.45	0.52	NPPA-AS1	NPPA antisense RNA 1	chr1	antisense
ENSG00000108406.8	2.57578504	2.67671631	4.07E-15	6.58E-14	0.42	0.37	2.28	4.1	DHX40	DEAH (Asp-Glu-Ala-His) box polypeptide 40	chr17	protein_coding
ENSG00000187079.13	1.48526835	2.6350089	1.91E-06	1.05E-05	0.26	0.3	0.78	1.3	TEAD1	TEA domain family member 1 (SV40 transcriptional enhancer factor)	chr11	protein_coding
ENSG00000187608.7	-1.319719	6.2914329	5.33E-06	2.72E-05	99.86	92.27	53.08	49.4	ISG15	ISG15 ubiquitin-like modifier	chr1	protein_coding
ENSG00000143452.14	1.62369259	-0.9062787	0.00216917	0.00660813	0.01	0.18	0.2	0.58	HORMAD1	HORMA domain containing 1	chr1	protein_coding
ENSG00000154803.11	1.36109765	4.23235176	4.27E-06	2.22E-05	2.01	2.23	7.36	7.14	FLCN	folliculin	chr17	protein_coding
ENSG00000204267.12	-1.3103828	4.49376745	8.32E-06	4.10E-05	3.92	4.49	2.34	2.17	TAP2	transporter 2, ATP-binding cassette, sub-family B (MDR/TAP)	chr6	protein_coding
ENSG00000143971.7	2.76624354	0.4625149	5.44E-11	5.62E-10	0.02	0.14	0.52	0.89	ETAA1	Ewing tumor-associated antigen 1	chr2	protein_coding
ENSG00000106688.10	-2.0021328	2.63490824	2.14E-10	2.05E-09	2.09	2.23	0.37	1.07	SLC1A1	solute carrier family 1 (neuronal/epithelial high affinity glutamate transporter, system Xag), member 1	chr9	protein_coding
ENSG00000112406.4	2.21879879	0.80205255	6.43E-09	5.08E-08	0.06	0.11	0.42	0.62	HECA	headcase homolog (Drosophila)	chr6	protein_coding
ENSG00000181061.12	1.77601599	2.1962011	4.22E-08	2.96E-07	0.96	1	3.46	5.51	HIGD1A	HIG1 hypoxia inducible domain family, member 1A	chr3	protein_coding
ENSG00000163960.10	1.46730645	3.34844361	1.29E-06	7.28E-06	0.4	0.42	1.19	1.84	UBXN7	UBX domain protein 7	chr3	protein_coding
ENSG00000075043.16	4.45795897	0.16296448	4.03E-16	7.08E-15	0.01	0.01	0.2	0.24	KCNQ2	potassium channel, voltage gated KQT-like subfamily Q, member 2	chr20	protein_coding
ENSG00000250222.1	-1.0509688	0.48712838	0.0047011	0.01328791	3.3	2.8	2.37	1.57	CTC-338M12	--	chr5	antisense
ENSG00000048162.18	-2.1484851	7.81513167	4.16E-13	5.42E-12	151.1	171.84	46.5	50.58	NOP16	NOP16 nucleolar protein	chr5	protein_coding
ENSG00000279347.1	2.39042418	-2.0719026	0.0061421	0.01678695	0	0.05	0.14	0.23	RP11-85I17.2	--	chr8	TEC
ENSG00000248370.1	-7.9071019	-1.0554499	2.44E-13	3.25E-12	0.79	1.38	0	0	RP11-366H4	--	chr4	lincRNA
ENSG00000170161.6	1.10731694	1.55075268	0.00084567	0.00283809	0.56	0.59	1.46	1.84	RP11-262H1	--	chr9	lincRNA
ENSG00000174669.10	-2.8569267	3.28829287	7.72E-19	1.70E-17	5.4	4.81	1.04	0.84	SLC29A2	solute carrier family 29 (equilibrative nucleoside transporter), member 2	chr11	protein_coding
ENSG00000148187.16	-1.5409133	4.89366602	1.59E-07	1.03E-06	3.26	4	1.57	1.75	MRRF	mitochondrial ribosome recycling factor	chr9	protein_coding
ENSG00000259804.1	2.24612679	-1.2351597	0.0001323	0.00051985	0.12	0.09	0.86	0.55	CTD-2012K1	--	chr16	antisense
ENSG00000138180.14	1.72697617	3.08738252	2.41E-08	1.75E-07	0.86	1.54	3.62	7	CEP55	centrosomal protein 55kDa	chr10	protein_coding
ENSG00000119771.13	-2.0690044	2.97220161	3.67E-11	3.85E-10	1.99	1.89	0.58	0.65	KLHL29	kelch-like family member 29	chr2	protein_coding
ENSG00000108510.8	2.67134597	2.21563759	4.66E-15	7.49E-14	0.07	0.12	0.73	0.89	MED13	mediator complex subunit 13	chr17	protein_coding
ENSG00000214694.9	1.1927574	0.67483336	0.00122544	0.00395638	0.14	0.2	0.36	0.65	ARHGEF33	Rho guanine nucleotide exchange factor (GEF) 33	chr2	protein_coding
ENSG00000027847.12	-1.1756962	4.3093046	6.22E-05	0.00026102	13.17	12.26	7.21	7.81	B4GALT7	xylosylprotein beta 1,4-galactosyltransferase, polypeptide 7	chr5	protein_coding
ENSG00000005801.15	3.45329856	0.18439355	6.00E-13	7.70E-12	0.05	0.01	0.26	0.7	ZNF195	zinc finger protein 195	chr11	protein_coding
ENSG00000168918.12	5.58983491	-2.6652556	0.00086564	0.00288128	0	0	0.05	0.03	INPP5D	inositol polyphosphate-5-phosphatase, 145kDa	chr2	protein_coding
ENSG00000181722.14	-2.9680582	-2.4476239	0.00112121	0.00364442	0.05	0.04	0	0.01	ZBTB20	zinc finger and BTB domain containing 20	chr3	protein_coding
ENSG00000280620.1	2.38263229	-0.9719626	1.73E-05	8.03E-05	0.52	0.33	3.27	2.88	SCAANT1	SCA7/ATXN7 antisense RNA 1	chr3	antisense
ENSG00000250131.1	2.09927191	-1.9619154	0.00956893	0.02497848	0.02	0.03	0	0.29	RP11-130F1(	--	chr4	antisense
ENSG00000100092.19	1.22750117	2.27426943	9.29E-05	0.00037553	0.75	0.86	2.73	2.3	SH3BP1	SH3-domain binding protein 1	chr22	protein_coding
ENSG00000134602.14	1.33176674	0.52644543	0.00044005	0.00155496	0.09	0.29	0.36	0.88	STK26	serine/threonine protein kinase 26	chrX	protein_coding
ENSG00000154096.12	-9.5331947	0.48883341	6.15E-28	2.55E-26	0.4	0.61	0	0	THY1	Thy-1 cell surface antigen	chr11	protein_coding
ENSG00000080854.13	6.81539267	-1.7863963	1.35E-07	8.87E-07	0	0	0.01	0.16	IGSF9B	immunoglobulin superfamily, member 9B	chr11	protein_coding
ENSG00000168824.13	3.70403917	-1.5596214	5.39E-06	2.75E-05	0.01	0.01	0.11	0.14	D4S234E	--	chr4	protein_coding
ENSG00000185591.8	1.18730448	5.49348413	4.33E-05	0.00018797	2.56	3.21	8.66	8.84	SP1	Sp1 transcription factor	chr12	protein_coding
ENSG00000258593.2	2.30423205	-2.1289741	0.01384632	0.03449994	0.08	0	0.06	0.47	CTD-3051D2	--	chr14	lincRNA
ENSG00000156374.13	1.46031419	1.23956557	2.39E-05	0.00010805	0.41	0.47	1.03	2.2	PCGF6	polycomb group ring finger 6	chr10	protein_coding
ENSG00000178947.8	7.62523829	-1.1215563	9.11E-12	1.03E-10	0	0	0.07	0.23	LINC00086	--	chrX	lincRNA
ENSG00000179314.12	4.30849725	-1.7933077	2.62E-06	1.42E-05	0	0	0.07	0.08	WSCD1	WSC domain containing 1	chr17	protein_coding
ENSG00000069188.15	-5.2054745	-3.0371681	0.0100455	0.02582742	0.02	0	0	0	SDK2	sidekick cell adhesion molecule 2	chr17	protein_coding
ENSG00000109501.12	1.42528617	5.77447162	1.01E-06	5.83E-06	6.27	6.92	25.24	21.85	WFS1	Wolfram syndrome 1 (wolframin)	chr4	protein_coding
ENSG00000157110.14	-2.3691215	4.68645324	7.03E-15	1.11E-13	6.74	7.47	1.8	1.87	RBPMS	RNA binding protein with multiple splicing	chr8	protein_coding
ENSG00000116962.13	-6.6642467	6.4440415	8.51E-71	3.52E-68	22.82	24.82	0.39	0.23	NID1	nidogen 1	chr1	protein_coding
ENSG00000266903.1	-1.5282059	1.49321202	4.76E-06	2.46E-05	2	2.74	1.11	1.08	CTB-171A8.1	--	chr19	antisense
ENSG00000038210.12	2.30034672	-0.3131664	2.19E-06	1.20E-05	0.02	0.1	0.14	0.58	PI4K2B	phosphatidylinositol 4-kinase type 2 beta	chr4	protein_coding
ENSG00000236104.2	-1.2234285	4.39438914	3.14E-05	0.00013904	9.11	8.34	4.67	5.3	ZBTB22	zinc finger and BTB domain containing 22	chr6	protein_coding
ENSG00000175482.7	-1.4269185	5.15770686	1.08E-06	6.15E-06	24.1	18.79	11.65	9.58	POLD4	polymerase (DNA-directed), delta 4, accessory subunit	chr11	protein_coding
ENSG00000120437.8	-1.551077	5.8103448	1.11E-07	7.42E-07	20.58	22.03	9	10.4	ACAT2	acetyl-CoA acetyltransferase 2	chr6	protein_coding
ENSG00000269533.4	2.87474344	-2.7426684	0.01615662	0.03958166	0	0.04	0.15	0.35	AC003002.6	--	chr19	protein_coding
ENSG00000146426.16	-3.5356771	-0.6257216	1.89E-10	1.81E-09	0.07	0.17	0.01	0.01	TIAM2	T-cell lymphoma invasion and metastasis 2	chr6	protein_coding
ENSG00000064651.12	1.00247716	1.28395355	0.00299627	0.00881199	0.16	0.21	0.45	0.53	SLC12A2	solute carrier family 12 (sodium/potassium/chloride transporter), member 2	chr5	protein_coding
ENSG00000143494.14	1.69982226	-1.4476937	0.00497703	0.01399196	0.01	0.03	0.02	0.12	VASH2	vasohibin 2	chr1	protein_coding
ENSG00000130762.13	-1.4395201	6.39278011	7.36E-07	4.32E-06	29.21	29.23	13.86	14.87	ARHGEF16	Rho guanine nucleotide exchange factor (GEF) 16	chr1	protein_coding
ENSG00000163435.14	-8.6513237	4.66240361	1.01E-77	6.06E-75	6.81	6.45	0.02	0.03	ELF3	E74-like factor 3 (ets domain transcription factor, epithelial-specific)	chr1	protein_coding
ENSG00000151090.16	1.79379596	1.66797458	9.32E-08	6.27E-07	0.13	0.14	0.58	0.67	THRB	thyroid hormone receptor, beta	chr3	protein_coding
ENSG00000080824.17	2.88265515	8.18851387	3.29E-21	8.73E-20	12.32	15.71	99.34	177.92	HSP90AA1	heat shock protein 90kDa alpha (cytosolic), class A member 1	chr14	protein_coding
ENSG00000269469.1	-1.0313403	0.4682372	0.00650327	0.01769137	2.38	1.93	1.95	0.89	CTD-3148I1(	--	chr19	protein_coding
ENSG00000103227.17	2.23435631	3.98235856	3.54E-13	4.65E-12	1.83	1.55	10.71	10.56	LMF1	lipase maturation factor 1	chr16	protein_coding
ENSG00000101752.10	2.87470538	1.49688078	7.56E-15	1.19E-13	0.03	0.08	0.51	0.57	MIB1	mindbomb E3 ubiquitin protein ligase 1	chr18	protein_coding
ENSG00000105357.14	12.9327407	3.89807653	9.78E-73	4.60E-70	0	0	4.51	4.55	MYH14	myosin, heavy chain 14, non-muscle	chr19	protein_coding
ENSG00000034533.10	1.89551608	1.07759582	1.45E-07	9.50E-07	0.19	0.32	1.26	1.24	ASTE1	asteroid homolog 1 (Drosophila)	chr3	protein_coding
ENSG00000119929.11	-1.8685627	3.54083301	8.51E-10	7.51E-09	10.99	10.97	3.69	4.33	CUTC	cutC copper transporter	chr10	protein_coding
ENSG00000164828.16	-1.2911768	6.7606888	8.07E-06	4.00E-05	25.92	26.97	11.85	17.08	SUN1	Sad1 and UNC84 domain containing 1	chr7	protein_coding
ENSG00000209702.1	8.88937262	-0.0085973	2.39E-20	5.90E-19	0	0	33.41	21.79	SNORD41	small nucleolar RNA, C/D box 41	chr19	snoRNA
ENSG00000204003.7	-1.7309114	-1.3271606	0.00202074	0.0061882	0.15	0.21	0.03	0.11	RP11-216L13	--	chr9	protein_coding
ENSG00000259793.1	-5.523229	-2.862689	0.00158008	0.00495521	0.06	0.06	0	0	RP11-400N9	--	chr2	antisense
ENSG00000008513.13	-4.3721347	7.32176148	7.50E-41	6.04E-39	35.08	35.32	2.56	1.95	ST3GAL1	ST3 beta-galactoside alpha-2,3-sialyltransferase 1	chr8	protein_coding
ENSG00000182223.7	5.71131553	-2.5898418	0.00047761	0.00167191	0	0	0.26	0.04	ZAR1	zygote arrest 1	chr4	protein_coding
ENSG00000265303.1	1.52883328	0.52894552	6.81E-05	0.00028389	1.01	1.02	3.91	3.91	CTD-2510F5	--	chr17	protein_coding
ENSG00000162086.13	2.03515177	2.13622289	5.73E-10	5.16E-09	0.42	0.36	1.8	2.51	ZNF75A	zinc finger protein 75a	chr16	protein_coding
ENSG00000182795.12	-1.9891378	-0.1727772	4.96E-06	2.55E-05	0.14	0.27	0.07	0.06	C1orf116	chromosome 1 open reading frame 116	chr1	protein_coding
ENSG00000280153.1	2.79456599	-2.2099401	0.00174589	0.00541921	0.02	0	0.11	0.13	RP11-876N2	--	chr16	TEC
ENSG00000110395.4	1.14419977	3.18086339	0.0001439	0.00056016	0.46	0.33	1.11	1.22	CBL	Cbl proto-oncogene, E3 ubiquitin protein ligase	chr11	protein_coding
ENSG00000128791.10	2.46505129	2.36929064	1.33E-13	1.82E-12	0.31	0.28	1.27	3.08	TWSG1	twisted gastrulation BMP signaling modulator 1	chr18	protein_coding
ENSG00000164458.8	-10.848516	1.79555077	2.76E-44	2.71E-42	1.69	2.8	0	0	T	T, brachyury homolog (mouse)	chr6	protein_coding
ENSG00000188385.10	1.83415936	-1.3605389	0.00194789	0.00597963	0	0.03	0.1	0.06	JAKMIP3	Janus kinase and microtubule interacting protein 3	chr10	protein_coding
ENSG00000187642.8	3.73442394	-0.0495596	2.57E-13	3.41E-12	0.05	0	0.65	0.38	PERM1	PPARGC1 and ESRR induced regulator, muscle 1	chr1	protein_coding
ENSG00000272068.1	-4.4689459	1.44885285	1.52E-26	5.82E-25	1.2	1.43	0.09	0.07	RP11-284F21	--	chr1	lincRNA
ENSG00000183840.6	-1.6345815	2.92568248	1.14E-07	7.62E-07	3.11	3.46	1.33	1.49	GPR39	G protein-coupled receptor 39	chr2	protein_coding
ENSG00000189190.8	1.652428	0.15196344	4.92E-05	0.00021047	0.09	0.08	0.24	0.48	ZNF600	zinc finger protein 600	chr19	protein_coding
ENSG00000273080.1	7.13474846	-1.5443995	2.80E-09	2.31E-08	0	0	1.74	0.77	RP11-301O1	--	chr2	antisense
ENSG00000117592.8	-1.0980196	8.2600529	0.00013318	0.00052279	204.81	181.38	117.63	123.02	PRDX6	peroxiredoxin 6	chr1	protein_coding
ENSG00000223414.2	-9.082917	0.04908065	3.76E-23	1.13E-21	0.59	0.91	0	0	LINC00473	long intergenic non-protein coding RNA 473	chr6	lincRNA
ENSG00000092096.13	8.96352904	3.7904655	2.82E-66	9.53E-64	0.01	0.02	12.01	10.99	SLC22A17	solute carrier family 22, member 17	chr14	protein_coding
ENSG00000171320.13	2.87996165	0.74825154	2.32E-12	2.81E-11	0.09	0.06	0.47	1.05	ESCO2	establishment of sister chromatid cohesion N-acetyltransferase 2	chr8	protein_coding
ENSG00000102893.14	1.26479287	3.21649221	2.82E-05	0.00012605	0.76	0.78	1.8	3.14	PHKB	phosphorylase kinase, beta	chr16	protein_coding
ENSG00000273442.1	5.87712165	-2.4793333	0.00014842	0.00057423	0	0	0.34	0.29	AC006946.17	--	chr22	lincRNA
ENSG00000162695.10	1.20162319	2.09924973	0.00016957	0.00064802	0.2	0.32	0.65	0.93	SLC30A7	solute carrier family 30 (zinc transporter), member 7	chr1	protein_coding
ENSG00000110422.10	2.44013323	1.26608695	2.84E-11	3.03E-10	0.08	0.06	0.43	0.64	HIPK3	homeodomain interacting protein kinase 3	chr11	protein_coding
ENSG00000151651.14	2.59886597	-0.0767183	2.84E-08	2.04E-07	0.01	0.11	0.21	0.76	ADAM8	ADAM metallopeptidase domain 8	chr10	protein_coding
ENSG00000143382.12	1.58475157	5.4601801	6.59E-08	4.54E-07	3.88	3.96	16.07	15.21	ADAMTSL4	ADAMTS-like 4	chr1	protein_coding
ENSG00000165626.15	9.54123424	0.59668563	6.35E-27	2.50E-25	0	0	1.46	0.8	BEND7	BEN domain containing 7	chr10	protein_coding
ENSG00000103534.15	-1.1493417	-0.8880102	0.01533929	0.03782283	0.05	0.13	0.01	0.09	TMC5	transmembrane channel-like 5	chr16	protein_coding
ENSG00000048028.10	1.33044992	1.98769694	3.52E-05	0.00015468	0.45	0.28	0.89	1.55	USP28	ubiquitin specific peptidase 28	chr11	protein_coding
ENSG00000123552.16	1.23218992	-0.6310643	0.01226038	0.03090491	0.06	0.02	0.04	0.21	USP45	ubiquitin specific peptidase 45	chr6	protein_coding
ENSG00000115963.12	2.03136397	2.59629214	2.15E-10	2.05E-09	0.58	0.72	2.74	4.39	RND3	Rho family GTPase 3	chr2	protein_coding
ENSG00000174939.9	-4.5115538	6.54752665	4.33E-42	3.66E-40	64.05	86.96	5.14	3.64	ASPHD1	aspartate beta-hydroxylase domain containing 1	chr16	protein_coding
ENSG00000183780.11	-9.1440403	3.12570497	4.40E-63	1.30E-60	4.01	4.85	0	0.02	SLC35F3	solute carrier family 35, member F3	chr1	protein_coding
ENSG00000164830.16	2.36663214	-0.986282	1.73E-05	8.03E-05	0.03	0.01	0.15	0.1	OXR1	oxidation resistance 1	chr8	protein_coding
ENSG00000163468.13	-1.1009079	9.34069042	0.00012659	0.00049902	329.36	303.19	189.96	203.46	CCT3	chaperonin containing TCP1, subunit 3 (gamma)	chr1	protein_coding
ENSG00000103490.13	1.91453246	1.95731486	7.28E-09	5.69E-08	1.05	1.55	6.76	6.27	PYCARD	PYD and CARD domain containing	chr16	protein_coding
ENSG00000234311.1	-9.2442343	0.20767123	7.90E-25	2.70E-23	2.22	1.43	0	0	RP11-432J24	--	chr10	antisense
ENSG00000005700.13	2.17195871	1.61535444	4.24E-10	3.91E-09	0.14	0.15	0.53	1.21	IBTK	inhibitor of Bruton agammaglobulinemia tyrosine kinase	chr6	protein_coding
ENSG00000133661.14	8.02605792	-0.7819921	3.61E-13	4.73E-12	0	0	0.83	0.83	SFTPD	surfactant protein D	chr10	protein_coding
ENSG00000188505.4	2.95881418	0.61062313	1.68E-12	2.06E-11	0.18	0.09	1.44	1.32	NCCRP1	non-specific cytotoxic cell receptor protein 1 homolog (zebrafish)	chr19	protein_coding
ENSG00000273821.1	2.19551564	-1.6638136	0.00158671	0.00496952	0.29	0	1.39	0.53	RP5-963E22.	--	chr20	lincRNA
ENSG00000255031.4	-1.8095812	3.05526862	4.45E-09	3.59E-08	8.27	9.02	3.26	3.31	RP11-802E1(	--	chr11	antisense
ENSG00000182504.9	1.864219	0.44711647	2.43E-06	1.32E-05	0.03	0.08	0.2	0.31	CEP97	centrosomal protein 97kDa	chr3	protein_coding
ENSG00000203618.5	3.7273547	3.82539383	1.04E-28	4.49E-27	0.46	1.61	18.19	18.1	GP1BB	glycoprotein Ib (platelet), beta polypeptide	chr22	protein_coding
ENSG00000104413.14	4.99966663	-1.2408092	1.07E-08	8.19E-08	0	0.01	0.15	0.22	ESRP1	epithelial splicing regulatory protein 1	chr8	protein_coding
ENSG00000258466.4	1.62586856	-0.010522	0.00011764	0.00046621	0.28	0.8	2.2	2.24	RP11-1012A	--	chr14	protein_coding
ENSG00000155629.13	2.61600225	-2.3206464	0.00455824	0.01291303	0	0.01	0.02	0.07	PIK3AP1	phosphoinositide-3-kinase adaptor protein 1	chr10	protein_coding
ENSG00000258602.1	-5.9081069	-0.0537316	1.84E-20	4.59E-19	3.04	2.15	0.11	0	RP11-7F17.7	--	chr14	lincRNA
ENSG00000095637.19	1.60398596	0.88209384	1.10E-05	5.28E-05	0.09	0.07	0.27	0.36	SORBS1	sorbin and SH3 domain containing 1	chr10	protein_coding
ENSG00000235609.7	2.67589428	-0.3361742	6.68E-08	4.59E-07	0.02	0.03	0.16	0.28	AF127936.9	--	chr21	lincRNA
ENSG00000107175.9	-1.7485316	6.20084955	2.54E-09	2.11E-08	51.54	44.04	17.14	20.87	CREB3	cAMP responsive element binding protein 3	chr9	protein_coding
ENSG00000146587.16	5.41641907	0.37970984	6.38E-21	1.64E-19	0	0.01	0.36	0.4	RBAK	RB-associated KRAB zinc finger	chr7	protein_coding
ENSG00000170871.10	1.47694081	2.38591158	3.01E-06	1.61E-05	0.22	0.34	0.95	1.12	KIAA0232	KIAA0232	chr4	protein_coding
ENSG00000174851.13	-1.0366188	6.08717861	0.0003258	0.00117925	51.06	42.07	32.9	27.51	YIF1A	Yip1 interacting factor homolog A (S. cerevisiae)	chr11	protein_coding
ENSG00000273248.1	-2.2986258	0.10509762	5.99E-08	4.14E-07	3.55	4.4	1.9	0.31	RP11-399K21	--	chr10	lincRNA
ENSG00000138050.13	2.32774536	0.47208837	1.11E-08	8.42E-08	0.14	0.22	0.7	1.66	THUMPD2	THUMP domain containing 2	chr2	protein_coding
ENSG00000137821.10	3.47033074	-0.4513942	1.01E-09	8.82E-09	0	0.02	0.07	0.24	LRRC49	leucine rich repeat containing 49	chr15	protein_coding
ENSG00000198888.2	1.67197327	11.4978425	9.47E-09	7.28E-08	785.1	1562.71	5610.32	4287.62	MT-ND1	mitochondrially encoded NADH dehydrogenase 1	chrM	protein_coding
ENSG00000175294.5	-5.177607	0.41087559	5.87E-23	1.75E-21	1.01	0.57	0	0.05	CATSPER1	cation channel, sperm associated 1	chr11	protein_coding
ENSG00000145354.8	1.03158796	2.95414758	0.000688	0.00234305	1.26	2	3.82	5.07	CISD2	CDGSH iron sulfur domain 2	chr4	protein_coding
ENSG00000131370.14	-1.457589	4.36811513	8.01E-07	4.68E-06	6.36	7.65	2.59	4.23	SH3BP5	SH3-domain binding protein 5 (BTK-associated)	chr3	protein_coding
ENSG00000122565.17	1.1508731	3.89844407	0.00010255	0.00041213	3.18	3.41	5.52	14.17	CBX3	chromobox homolog 3	chr7	protein_coding
ENSG00000168398.6	-2.9477871	2.22700473	4.80E-18	9.89E-17	1	0.96	0.12	0.21	BDKRB2	bradykinin receptor B2	chr14	protein_coding
ENSG00000180767.8	-6.9312117	-1.8851973	8.18E-08	5.55E-07	0.31	0.1	0	0	CHST13	carbohydrate (chondroitin 4) sulfotransferase 13	chr3	protein_coding
ENSG00000162368.12	1.04418378	3.71892347	0.00043853	0.0015505	1.88	2.07	3.48	7.44	CMPK1	cytidine monophosphate (UMP-CMP) kinase 1, cytosolic	chr1	protein_coding
ENSG00000122642.9	-1.6601528	8.86805695	1.22E-08	9.19E-08	141.55	166.7	66.15	63.61	FKBP9	FK506 binding protein 9, 63 kDa	chr7	protein_coding
ENSG00000203668.1	3.56604719	1.14349403	2.99E-18	6.28E-17	0.05	0.02	0.32	0.87	CHML	choroideremia-like (Rab escort protein 2)	chr1	protein_coding
ENSG00000136848.15	-1.7254837	6.46742508	3.89E-09	3.17E-08	15.77	15.44	6.34	6.23	DAB2IP	DAB2 interacting protein	chr9	protein_coding
ENSG00000130700.6	2.67994949	2.24159003	3.19E-15	5.19E-14	0.3	0.48	3.48	3.16	GATA5	GATA binding protein 5	chr20	protein_coding
ENSG00000164520.10	-3.9323078	4.54586304	1.27E-32	6.70E-31	12.4	15.43	1.24	1.18	RAET1E	retinoic acid early transcript 1E	chr6	protein_coding
ENSG00000204394.11	-1.9651871	7.81321423	2.49E-11	2.67E-10	71.28	63.11	22.19	23.73	VARS	valyl-tRNA synthetase	chr6	protein_coding
ENSG00000160161.8	-3.5053518	-0.8388676	1.48E-09	1.27E-08	0.15	0.23	0.03	0.01	CILP2	cartilage intermediate layer protein 2	chr19	protein_coding
ENSG00000011295.14	1.04036788	3.98355876	0.00041718	0.00148156	2.91	2.63	6.25	9.03	TTC19	tetratricopeptide repeat domain 19	chr17	protein_coding
ENSG00000162366.6	-2.0250222	-0.9782144	0.00016946	0.00064769	0.58	0.43	0.32	0.02	PDZK1IP1	PDZK1 interacting protein 1	chr1	protein_coding
ENSG00000079387.12	1.49899712	2.35339371	2.24E-06	1.23E-05	0.41	0.45	1.41	1.82	SENP1	SUMO1/sentrin specific peptidase 1	chr12	protein_coding
ENSG00000169994.17	-2.0946698	-2.0723496	0.0061421	0.01678695	0.06	0.01	0.02	0	MYO7B	myosin VIIB	chr2	protein_coding
ENSG00000196476.10	5.97223749	2.19224342	1.06E-38	7.56E-37	0.09	0.03	6.39	4.5	C20orf96	chromosome 20 open reading frame 96	chr20	protein_coding
ENSG00000176533.11	-2.4889059	-2.7174314	0.00943894	0.02469931	0.06	0.01	0	0.01	GNG7	guanine nucleotide binding protein (G protein), gamma 7	chr19	protein_coding
ENSG00000182307.11	-1.7232998	8.25540927	3.62E-09	2.96E-08	135.69	148.18	53.3	61.4	C8orf33	chromosome 8 open reading frame 33	chr8	protein_coding
ENSG00000048649.12	1.78559535	2.53131339	1.77E-08	1.31E-07	0.16	0.16	0.46	1.06	RSF1	remodeling and spacing factor 1	chr11	protein_coding
ENSG00000176928.5	2.33887212	-2.4903051	0.01923175	0.0458365	0.01	0	0.01	0.06	GCNT4	glucosaminyl (N-acetyl) transferase 4, core 2	chr5	protein_coding
ENSG00000171295.11	2.70409209	-0.5722598	3.72E-07	2.30E-06	0.05	0.01	0.12	0.4	ZNF440	zinc finger protein 440	chr19	protein_coding
ENSG00000065325.11	-2.3453498	4.95658474	1.00E-14	1.57E-13	9.5	8.55	2.28	2.46	GLP2R	glucagon-like peptide 2 receptor	chr17	protein_coding
ENSG00000213516.8	1.02960563	1.76433388	0.00159238	0.00498531	0.33	0.36	0.76	1.13	RBMXL1	RNA binding motif protein, X-linked-like 1	chr1	protein_coding
ENSG00000228393.3	3.3549389	-1.0520583	1.78E-07	1.15E-06	0.07	0.03	0.59	0.79	LINC01004	long intergenic non-protein coding RNA 1004	chr7	antisense
ENSG00000134627.10	-2.9847266	-1.2818751	4.18E-07	2.56E-06	0.08	0.24	0	0.05	PIWIL4	piwi-like RNA-mediated gene silencing 4	chr11	protein_coding
ENSG00000281538.1	5.45974547	-2.7440718	0.00158008	0.00495521	0	0	0.2	0.35	RP4-669P10.	--	chr22	lincRNA
ENSG00000121741.15	2.63177522	1.945899	2.96E-14	4.37E-13	0.09	0.08	0.52	0.84	ZMYM2	zinc finger, MYM-type 2	chr13	protein_coding
ENSG00000165028.10	6.13711747	-2.3031808	2.70E-05	0.00012094	0	0	0.24	0.09	NIPSNAP3B	nipsnap homolog 3B (C. elegans)	chr9	protein_coding
ENSG00000183682.7	-5.3760424	-2.9459444	0.00290522	0.00856312	0.05	0.1	0	0	BMP8A	bone morphogenetic protein 8a	chr1	protein_coding
ENSG00000250510.6	-6.2730644	-2.3808334	2.70E-05	0.00012094	0.12	0.05	0	0	GPR162	G protein-coupled receptor 162	chr12	protein_coding
ENSG00000244625.4	2.13116791	-2.2396172	0.02071392	0.04894569	0.05	0.03	0.45	0.08	MIATNB	MIAT neighbor (non-protein coding)	chr22	lincRNA
ENSG00000101079.19	-1.1229404	5.65941388	0.00010465	0.00042015	18.64	18.64	10.76	12.08	NDRG3	NDRG family member 3	chr20	protein_coding
ENSG00000273033.2	2.75368557	0.37748225	1.35E-10	1.33E-09	0.03	0.06	0.25	0.57	RP11-67L2.2	--	chr3	lincRNA
ENSG00000110768.10	1.19279094	3.08116435	8.54E-05	0.00034671	0.69	0.9	1.78	3.08	GTF2H1	general transcription factor IIH, polypeptide 1, 62kDa	chr11	protein_coding
ENSG00000276408.1	2.42625442	0.17649326	1.78E-08	1.31E-07	0.32	0.47	3.02	2.65	RP11-490B1	--	chr16	lincRNA
ENSG00000273203.1	6.36742889	-2.1317322	8.95E-06	4.38E-05	0	0	0.06	0.17	AC006946.16	--	chr22	lincRNA
ENSG00000165209.17	1.07681867	2.71444942	0.00046223	0.00162686	0.4	0.64	1	1.91	STRBP	spermatid perinuclear RNA binding protein	chr9	protein_coding
ENSG00000163728.9	4.90777193	2.6462716	1.62E-35	9.74E-34	0.06	0.06	1.27	3.81	TTC14	tetratricopeptide repeat domain 14	chr3	protein_coding
ENSG00000115267.5	3.06325565	-0.428372	9.06E-09	6.99E-08	0	0.05	0.23	0.37	IFIH1	interferon induced with helicase C domain 1	chr2	protein_coding
ENSG00000116151.12	-1.2533133	3.82337334	2.47E-05	0.00011156	7.58	7.86	4.31	4.32	MORN1	MORN repeat containing 1	chr1	protein_coding
ENSG00000188573.7	4.89386602	-0.5797917	1.04E-12	1.30E-11	0	0.04	0.82	0.77	FBLL1	fibrillarin-like 1	chr5	protein_coding
ENSG00000127507.16	-2.9835551	2.28342765	2.42E-18	5.13E-17	0.91	0.99	0.16	0.16	EMR2	--	chr19	protein_coding
ENSG00000198001.12	1.04360832	0.71005065	0.00385844	0.01109844	0.27	0.1	0.43	0.61	IRAK4	interleukin-1 receptor-associated kinase 4	chr12	protein_coding

ENSG00000149970.13	6.16035826	-2.2802665	2.70E-05	0.00012094	0	0	0.01	0.07	CNKSR2	connector enhancer of kinase suppressor of Ras 2	chrX	protein_coding
ENSG00000259146.3	2.6201356	0.18357694	3.06E-09	2.52E-08	0.36	0.12	1.48	2.49	RP1-261D10	--	chr14	antisense
ENSG00000178177.13	2.17912678	-0.510717	8.25E-06	4.07E-05	0.02	0.01	0.07	0.15	LCORL	ligand dependent nuclear receptor corepressor-like	chr4	protein_coding
ENSG00000070367.14	4.27909496	1.16618576	5.60E-22	1.57E-20	0.01	0.02	0.17	0.66	EXOC5	exocyst complex component 5	chr14	protein_coding
ENSG00000110104.10	-1.1209957	6.88775133	0.00010017	0.00040332	62.77	65.76	40.87	37.76	CCDC86	coiled-coil domain containing 86	chr11	protein_coding
ENSG00000229656.5	-4.6853502	-0.3281712	1.76E-15	2.94E-14	1.2	1.11	0.06	0.05	RP11-462L8.	--	chr10	lincRNA
ENSG00000245025.2	-1.743031	-0.452226	5.82E-05	0.00024531	0.34	0.42	0.01	0.28	RP11-875O1	--	chr8	antisense
ENSG00000275512.1	2.34540499	-2.1039184	0.00923116	0.02429359	0.01	0.02	0.15	0.09	RP11-322E1	--	chr18	lincRNA
ENSG00000105948.12	1.19309632	1.88757536	0.00022558	0.00084533	0.33	0.43	0.98	1.35	TTC26	tetratricopeptide repeat domain 26	chr7	protein_coding
ENSG00000136943.9	2.68794808	2.67689444	4.66E-16	8.14E-15	0.29	0.31	2.03	3.13	CTSV	cathepsin V	chr9	protein_coding
ENSG00000095539.14	-1.2863689	-0.5552454	0.00764142	0.02043737	0.2	0.13	0.18	0.01	SEMA4G	sema domain, immunoglobulin domain (Ig), transmembrane domain (TM) and short cytoplasmic domain, (semaphorin) 4G	chr10	protein_coding
ENSG00000140986.7	5.97464286	-2.4149021	8.36E-05	0.00034052	0	0	0.16	0.07	RPL3L	ribosomal protein L3-like	chr16	protein_coding
ENSG00000070081.14	2.40000053	3.85447411	1.11E-14	1.72E-13	1.15	1.59	6.61	12.76	NUCB2	nucleobindin 2	chr11	protein_coding
ENSG00000105699.15	3.12462756	6.47629481	5.26E-24	1.68E-22	5.8	5.85	73.47	61.61	LSR	lipolysis stimulated lipoprotein receptor	chr19	protein_coding
ENSG00000263884.1	3.06343599	-2.0291067	0.0002605	0.00096399	0.03	0	0.14	0.18	RP11-705O1	--	chr18	lincRNA
ENSG00000102007.9	-2.2695668	8.87317627	2.32E-14	3.47E-13	437.8	546.67	143.63	127.64	PLP2	proteolipid protein 2 (colonic epithelium-enriched)	chrX	protein_coding
ENSG00000166016.5	-3.0216025	5.46393648	2.31E-22	6.64E-21	12.01	13.98	1.93	2.33	ABTB2	ankyrin repeat and BTB (POZ) domain containing 2	chr11	protein_coding
ENSG00000197448.12	1.07239445	7.53384056	0.00019183	0.00072649	77.73	79.98	222.5	219.19	GSTK1	glutathione S-transferase kappa 1	chr7	protein_coding
ENSG00000163060.7	-6.356485	-2.3220013	8.95E-06	4.38E-05	0.18	0.15	0	0	TEKT4	tektin 4	chr2	protein_coding
ENSG00000169967.15	2.90047708	1.65416808	1.56E-15	2.61E-14	0.06	0.04	0.32	0.74	MAP3K2	mitogen-activated protein kinase kinase kinase 2	chr2	protein_coding
ENSG00000196872.9	8.38686753	5.13166306	7.03E-79	4.43E-76	0.03	0.06	18.52	19.96	KIAA1211L	KIAA1211-like	chr2	protein_coding
ENSG00000147677.9	-1.0517421	6.11796719	0.00026518	0.0009747	13.69	18.13	8.81	11.69	EIF3H	eukaryotic translation initiation factor 3, subunit H	chr8	protein_coding
ENSG00000181031.14	-2.1017841	2.08409922	1.82E-10	1.76E-09	1.81	1.87	0.63	0.52	RPH3AL	rabphilin 3A-like (without C2 domains)	chr17	protein_coding
ENSG00000144034.13	1.15183742	0.74079159	0.00146003	0.00465299	1.1	0.71	1.38	4	TPRKB	TP53RK binding protein	chr2	protein_coding
ENSG00000233930.3	4.72703895	-3.141721	0.01889873	0.04519557	0	0	0.07	0.01	KRTAP5-AS1	KRTAP5-1/KRTAP5-2 antisense RNA 1	chr11	antisense
ENSG00000134318.12	4.08033889	2.8153781	2.41E-29	1.08E-27	0.05	0.1	1.14	2.09	ROCK2	Rho-associated, coiled-coil containing protein kinase 2	chr2	protein_coding
ENSG00000239335.4	-3.3212981	-2.7885037	0.00943894	0.02469931	0.34	0.09	0	0.04	LLPH-AS1	LLPH antisense RNA 1 (head to head)	chr12	antisense
ENSG00000169184.5	-5.069216	4.44420409	5.39E-46	5.96E-44	4.43	4.64	0.16	0.2	MN1	meningioma (disrupted in balanced translocation) 1	chr22	protein_coding
ENSG00000132485.11	1.02172173	3.29551529	0.00066865	0.00228173	1.47	1.83	2.93	6.06	ZRANB2	zinc finger, RAN-binding domain containing 2	chr1	protein_coding
ENSG00000100228.11	3.01490844	0.86005492	1.51E-13	2.05E-12	0.08	0.08	0.89	0.83	RAB36	RAB36, member RAS oncogene family	chr22	protein_coding
ENSG00000167705.10	2.12092269	4.43890755	2.54E-12	3.06E-11	3.44	3.94	21.67	21.07	RILP	Rab interacting lysosomal protein	chr17	protein_coding
ENSG00000157214.12	3.66067347	-1.59144	8.20E-06	4.05E-05	0	0.01	0.06	0.08	STEAP2	STEAP family member 2, metalloreductase	chr7	protein_coding
ENSG00000127252.5	7.31987163	-1.3807582	4.49E-10	4.11E-09	0	0	0.33	0.87	HRASLS	HRAS-like suppressor	chr3	protein_coding
ENSG00000000419.11	1.23003252	4.37493872	2.92E-05	0.00013027	7.07	10.18	20.64	33.49	DPM1	dolichyl-phosphate mannosyltransferase polypeptide 1, catalytic subunit	chr20	protein_coding
ENSG00000263307.1	5.22761104	-2.8805399	0.00290522	0.00856312	0	0	0.46	0.1	RP11-166B2.	--	chr16	antisense
ENSG00000242515.4	5.55293893	2.10405916	2.14E-35	1.28E-33	0.01	0.08	2.8	2.79	UGT1A10	UDP glucuronosyltransferase 1 family, polypeptide A10	chr2	protein_coding
ENSG00000132964.10	1.8509613	0.43886313	2.82E-06	1.52E-05	0.06	0.23	0.47	0.93	CDK8	cyclin-dependent kinase 8	chr13	protein_coding
ENSG00000241935.7	2.58313158	-0.1943961	3.81E-08	2.68E-07	0.02	0.13	0.57	0.6	HOGA1	4-hydroxy-2-oxoglutarate aldolase 1	chr10	protein_coding
ENSG00000119865.7	1.97779973	2.84853844	3.72E-10	3.46E-09	0.68	0.65	3.07	3.92	CNRIP1	cannabinoid receptor interacting protein 1	chr2	protein_coding
ENSG00000230438.6	-3.0209797	-0.1473745	5.21E-11	5.39E-10	0.78	1.07	0.12	0.18	SERPINB9P1	serpin peptidase inhibitor, clade B (ovalbumin), member 9, pseudogene 1	chr6	lincRNA
ENSG00000182168.13	-6.5775305	-2.1602418	1.78E-06	9.85E-06	0.03	0.03	0	0	UNC5C	unc-5 homolog C (C. elegans)	chr4	protein_coding
ENSG00000102390.9	-3.7020023	1.57624072	3.72E-22	1.05E-20	3.18	4.29	0.37	0.39	PBDC1	polysaccharide biosynthesis domain containing 1	chrX	protein_coding
ENSG00000059122.15	-1.2609946	6.33090907	1.33E-05	6.31E-05	19.39	17.1	10.77	9.49	FLYWCH1	FLYWCH-type zinc finger 1	chr16	protein_coding
ENSG00000083844.9	1.12906501	3.11432665	0.00019441	0.00073565	0.32	0.38	1.07	0.95	ZNF264	zinc finger protein 264	chr19	protein_coding
ENSG00000273076.1	2.60022538	-1.9342871	0.0017973	0.00556364	0	0.2	1	0.72	RP3-508I15.2	--	chr22	antisense
ENSG00000174013.7	1.73153031	2.40905125	5.56E-08	3.86E-07	0.36	0.29	1.31	1.59	FBXO45	F-box protein 45	chr3	protein_coding
ENSG00000151806.12	2.19968504	1.04020296	2.99E-09	2.46E-08	0.12	0.13	0.66	0.9	GUF1	GUF1 GTPase homolog (S. cerevisiae)	chr4	protein_coding
ENSG00000198855.5	2.1725706	1.84338902	1.60E-10	1.55E-09	0.18	0.36	1.46	1.77	FICD	FIC domain containing	chr12	protein_coding
ENSG00000161405.15	4.79851921	0.18152852	7.45E-18	1.51E-16	0	0.01	0.22	0.21	IKZF3	IKAROS family zinc finger 3 (Aiolos)	chr17	protein_coding
ENSG00000254602.1	3.2321422	-0.1419389	1.39E-10	1.37E-09	0.04	0.05	0.48	0.65	AP000662.4	--	chr11	sense_overlapping
ENSG00000011485.13	-1.1666437	8.719862	5.02E-05	0.0002143	231.54	216.21	135.63	130.03	PPP5C	protein phosphatase 5, catalytic subunit	chr19	protein_coding
ENSG00000269640.4	-1.9261107	2.87866218	6.80E-10	6.07E-09	5.3	5.12	1.83	1.82	CTD-2521M	--	chr19	lincRNA
ENSG00000136997.13	-3.2801382	7.20848149	4.44E-26	1.64E-24	71.89	72.84	9.48	10.39	MYC	v-myc avian myelocytomatosis viral oncogene homolog	chr8	protein_coding
ENSG00000256683.5	-7.4624228	-1.4457573	1.84E-10	1.77E-09	0.24	0.22	0	0	ZNF350	zinc finger protein 350	chr19	protein_coding
ENSG00000197785.12	-1.3221339	8.46492798	4.70E-06	2.43E-05	150.9	141.02	80.08	75.39	ATAD3A	ATPase family, AAA domain containing 3A	chr1	protein_coding
ENSG00000140497.15	1.23273732	5.22379309	2.30E-05	0.00010461	6.92	7.78	24.92	20.98	SCAMP2	secretory carrier membrane protein 2	chr15	protein_coding
ENSG00000277726.3	3.8712652	-2.1134597	8.40E-05	0.0003415	0	0.02	0.2	0.17	TARDBP	TAR DNA binding protein	chr1	protein_coding
ENSG00000281832.1	-5.7776308	-2.7095582	0.00047761	0.00167191	0.13	0.03	0	0	LINC00602	long intergenic non-protein coding RNA 602	chr6	lincRNA
ENSG00000255423.1	6.66802862	-1.9087382	6.25E-07	3.72E-06	0	0	0.23	0.26	EBLN2	endogenous Bornavirus-like nucleoprotein 2	chr3	protein_coding
ENSG00000165138.15	-2.9166536	5.23910038	4.59E-21	1.20E-19	7.58	6.87	1.21	1.35	ANKS6	ankyrin repeat and sterile alpha motif domain containing 6	chr9	protein_coding
ENSG00000058673.14	1.19530134	4.71106222	4.36E-05	0.00018907	1.29	1.74	4.36	4.89	ZC3H11A	zinc finger CCCH-type containing 11A	chr1	protein_coding
ENSG00000259663.2	-7.348517	-1.5431557	7.05E-10	6.26E-09	0.07	0.07	0	0	CTD-2314G2	--	chr5	antisense
ENSG00000137098.12	-2.5438169	-1.2287424	1.96E-05	9.01E-05	0.45	0.05	0.09	0.03	SPAG8	sperm associated antigen 8	chr9	protein_coding
ENSG00000167766.17	2.23417531	1.85847138	5.72E-11	5.89E-10	0.09	0.22	0.54	1.38	ZNF83	zinc finger protein 83	chr19	protein_coding
ENSG00000273486.1	4.1434817	-1.9148203	1.13E-05	5.42E-05	0.02	0	0.15	0.31	RP11-731C1	--	chr3	antisense
ENSG00000254639.1	-3.4046937	-2.1671824	5.06E-05	0.00021569	0.33	0.48	0	0.08	CTD-2589M	--	chr11	lincRNA
ENSG00000253537.2	-5.474398	-0.4612647	1.22E-16	2.23E-15	0.16	0.25	0.01	0	PCDHGA7	protocadherin gamma subfamily A, 7	chr5	protein_coding
ENSG00000129003.14	3.39104284	2.04768115	1.29E-20	3.25E-19	0.04	0.03	0.41	0.52	VPS13C	vacuolar protein sorting 13 homolog C (S. cerevisiae)	chr15	protein_coding
ENSG00000143994.12	1.45730658	-1.2336712	0.0105162	0.02693027	0.13	0.08	0.41	0.35	ABHD1	abhydrolase domain containing 1	chr2	protein_coding
ENSG00000164327.11	3.17998024	1.606723	1.76E-17	3.47E-16	0.04	0.06	0.38	0.81	RICTOR	RPTOR independent companion of MTOR, complex 2	chr5	protein_coding
ENSG00000130589.15	1.15304093	5.56180036	6.99E-05	0.00029048	2.85	2.06	7.21	7.37	HELZ2	helicase with zinc finger 2, transcriptional coactivator	chr20	protein_coding
ENSG00000143816.7	2.3292665	4.05033661	3.90E-14	5.67E-13	1.07	1.16	7.17	7.76	WNT9A	wingless-type MMTV integration site family, member 9A	chr1	protein_coding
ENSG00000121104.6	6.61967026	1.88235775	1.07E-37	7.15E-36	0.02	0.02	3.1	3.09	FAM117A	family with sequence similarity 117, member A	chr17	protein_coding
ENSG00000134352.18	2.69525979	1.02128867	3.29E-12	3.90E-11	0.03	0.06	0.24	0.56	IL6ST	interleukin 6 signal transducer	chr5	protein_coding
ENSG00000237976.1	1.37034287	-1.0836347	0.01224023	0.03086062	0.35	0.23	0.67	1.32	RP11-126K1.	--	chr1	antisense
ENSG00000102981.8	2.18384353	1.88185245	1.14E-10	1.13E-09	0.89	0.76	5.47	4.54	PARD6A	par-6 family cell polarity regulator alpha	chr16	protein_coding
ENSG00000125695.12	1.01703883	3.24223694	0.00070542	0.00239827	2.09	2.64	4.62	8.18	RP11-51F16.	--	chr17	protein_coding
ENSG00000236311.5	5.06203518	-2.9716376	0.0100455	0.02582742	0	0	0.07	0.07	TLX1NB	TLX1 neighbor	chr10	protein_coding
ENSG00000196126.9	7.09975508	1.44768529	6.72E-35	3.91E-33	0	0.04	4.62	3.95	HLA-DRB1	major histocompatibility complex, class II, DR beta 1	chr6	protein_coding
ENSG00000168488.17	-1.3606755	7.90488587	2.55E-06	1.38E-05	56.72	69.5	34.92	30.37	ATXN2L	ataxin 2-like	chr16	protein_coding
ENSG00000169783.11	-1.2656626	0.05226385	0.00149552	0.00475159	0.37	0.28	0.16	0.19	LINGO1	leucine rich repeat and Ig domain containing 1	chr15	protein_coding
ENSG00000186897.4	-1.611286	1.64549983	1.09E-06	6.20E-06	1.73	1.93	0.71	0.88	C1QL4	complement component 1, q subcomponent-like 4	chr12	protein_coding
ENSG00000224794.1	-5.8924048	-2.6371928	0.00026535	0.0009747	0.14	0.06	0	0	RP3-333H23	--	chr22	antisense
ENSG00000196337.9	6.6758292	-1.9040819	3.73E-07	2.30E-06	0	0	0.13	0.11	CGB7	chorionic gonadotropin, beta polypeptide 7	chr19	protein_coding
ENSG00000234753.4	-2.9168726	0.48657071	3.64E-12	4.29E-11	2.86	1.34	0.58	0.17	FOXP4-AS1	FOXP4 antisense RNA 1	chr6	antisense
ENSG00000267106.4	-8.0060196	-0.96446	7.69E-14	1.09E-12	0.34	0.26	0	0	ZNF561-AS1	ZNF561 antisense RNA 1 (head to head)	chr19	processed_transcript
ENSG00000023839.9	1.41504933	0.93021353	8.24E-05	0.00033846	0.09	0.18	0.35	0.59	ABCC2	ATP-binding cassette, sub-family C (CFTR/MRP), member 2	chr10	protein_coding
ENSG00000196810.4	-1.1405001	3.71604877	0.00012231	0.00048311	1.82	1.91	1.16	1.1	CTBP1-AS2	CTBP1 antisense RNA 2 (head to head)	chr4	antisense
ENSG00000271447.4	-1.9452789	2.4199367	1.10E-09	9.58E-09	2.64	2.85	1.03	0.87	MMP28	matrix metallopeptidase 28	chr17	protein_coding
ENSG00000259705.1	-7.3875027	-1.5099761	4.49E-10	4.11E-09	0.26	0.26	0	0	RP11-227D1	--	chr15	lincRNA
ENSG00000158864.11	-1.0240701	8.33050346	0.00035872	0.00129039	164.27	142.54	97.59	103.71	NDUFS2	NADH dehydrogenase (ubiquinone) Fe-S protein 2, 49kDa (NADH-coenzyme Q reductase)	chr1	protein_coding
ENSG00000168214.19	1.62754237	1.67669731	1.06E-06	6.07E-06	0.17	0.21	0.68	0.88	RBPJ	recombination signal binding protein for immunoglobulin kappa J region	chr4	protein_coding
ENSG00000214029.4	4.68064115	-0.2803768	2.87E-14	4.25E-13	0	0	0.08	0.11	ZNF891	zinc finger protein 891	chr12	protein_coding
ENSG00000116604.16	-1.0998072	6.87467682	0.00013437	0.00052676	19.51	22.39	13.17	12.84	MEF2D	myocyte enhancer factor 2D	chr1	protein_coding
ENSG00000270006.2	1.86380099	1.09959746	2.20E-07	1.41E-06	2.4	1.13	7.75	9.58	RP11-178L8.	--	chr16	antisense
ENSG00000175984.13	3.85588326	0.53097848	1.45E-16	2.65E-15	0.03	0.01	0.37	0.49	DENND2C	DENN/MADD domain containing 2C	chr1	protein_coding
ENSG00000196814.13	4.12419084	3.57498681	1.87E-32	9.78E-31	0.15	0.16	3.22	3.92	MVB12B	multivesicular body subunit 12B	chr9	protein_coding
ENSG00000099821.12	-1.2464269	7.54830801	1.57E-05	7.34E-05	59.82	51.25	32.37	29.98	POLRMT	polymerase (RNA) mitochondrial (DNA directed)	chr19	protein_coding
ENSG00000176438.11	-2.3299663	3.62311423	6.43E-14	9.16E-13	1.16	1.09	0.37	0.22	SYNE3	spectrin repeat containing, nuclear envelope family member 3	chr14	protein_coding
ENSG00000243649.7	-2.6336262	-2.2782687	0.00174589	0.00541921	0.1	0.05	0	0.03	CFB	complement factor B	chr6	protein_coding
ENSG00000107807.11	12.2320697	3.20485034	2.45E-62	6.75E-60	0	0	7.16	6.2	TLX1	T-cell leukemia homeobox 1	chr10	protein_coding
ENSG00000177990.10	3.56229785	-1.6649094	2.93E-05	0.00013031	0.01	0.01	0.07	0.16	DPY19L2	dpy-19-like 2 (C. elegans)	chr12	protein_coding
ENSG00000151572.15	-6.1774727	0.18857158	4.97E-23	1.49E-21	0.38	0.52	0	0.01	ANO4	anoctamin 4	chr12	protein_coding
ENSG00000148926.8	1.72739407	6.89154316	3.64E-09	2.97E-08	18.56	14.18	70.02	74.74	ADM	adrenomedullin	chr11	protein_coding
ENSG00000116852.13	-4.4295791	5.00611851	2.12E-39	1.57E-37	4.77	5.25	0.37	0.24	KIF21B	kinesin family member 21B	chr1	protein_coding
ENSG00000279821.1	-3.1950933	3.72297003	5.51E-23	1.64E-21	5.27	7.5	0.95	0.9	RP11-1334A	--	chr5	TEC
ENSG00000253392.2	3.11210251	-2.6073453	0.00552196	0.01522223	0.03	0	0.17	0.32	AC006277.2	--	chr19	antisense
ENSG00000267432.4	-10.39743	1.34553341	2.53E-38	1.76E-36	2.26	2.95	0	0	RP11-559N1	--	chr17	processed_transcript
ENSG00000244486.6	-1.8676497	1.68485265	2.31E-08	1.67E-07	0.91	1.4	0.46	0.39	SCARF2	scavenger receptor class F, member 2	chr22	protein_coding
ENSG00000135047.13	-2.9765589	8.78006834	2.27E-22	6.53E-21	261.29	272.96	45.32	45.08	CTSL	cathepsin L	chr9	protein_coding
ENSG00000180096.10	-1.0633001	3.32204742	0.00037533	0.00134411	6.69	7.03	4.73	4	1-Sep	septin 1	chr16	protein_coding
ENSG00000267147.2	2.7415935	0.20283173	6.57E-10	5.88E-09	0.28	0.3	2.59	2.59	CTC-548K16	--	chr19	lincRNA
ENSG00000251349.3	5.78313448	-2.5411454	0.00026535	0.0009747	0	0	0.11	0.11	MSANTD3-T	MSANTD3-TMEFF1 readthrough	chr9	protein_coding
ENSG00000214708.4	2.02570306	-0.0668537	3.79E-06	1.99E-05	0.2	0.14	0.72	1.16	AC090616.2	--	chr17	antisense
ENSG00000280219.1	-5.3769376	-2.9455251	0.00290522	0.00856312	0.01	0.02	0	0	RP11-752L2(	--	chr4	TEC
ENSG00000064763.9	-9.5814191	0.53693276	1.85E-28	7.90E-27	0.58	0.63	0	0	FAR2	fatty acyl CoA reductase 2	chr12	protein_coding
ENSG00000092068.17	4.43035689	3.44412879	4.21E-35	2.47E-33	0.19	0.13	4.96	4.42	SLC7A8	solute carrier family 7 (amino acid transporter light chain, L system), member 8	chr14	protein_coding
ENSG00000135968.18	5.92638778	1.22717073	4.88E-29	2.16E-27	0.01	0	0.34	0.91	GCC2	GRIP and coiled-coil domain containing 2	chr2	protein_coding
ENSG00000185247.13	-5.673336	-0.2764423	2.54E-18	5.35E-17	0.5	0.76	0.03	0	MAGEA11	melanoma antigen family A11	chrX	protein_coding
ENSG00000248323.4	-1.6847886	0.52719798	7.26E-06	3.62E-05	0.41	0.9	0.26	0.28	LUCAT1	lung cancer associated transcript 1 (non-protein coding)	chr5	lincRNA
ENSG00000272720.1	4.43093514	0.13562342	1.69E-16	3.06E-15	0.09	0	1.3	1.58	CTA-228A9.3	--	chr22	lincRNA
ENSG00000188001.8	3.496945	-2.3667948	0.00112121	0.00364442	0	0	0.03	0.04	TPRG1	tumor protein p63 regulated 1	chr3	protein_coding
ENSG00000226520.1	6.05360204	-2.3605161	4.74E-05	0.00020301	0	0	0.6	0.32	KIRREL-IT1	KIRREL intronic transcript 1	chr1	sense_intronic
ENSG00000083799.16	1.66983513	2.23380429	1.87E-07	1.21E-06	0.26	0.14	0.67	1.01	CYLD	cylindromatosis (turban tumor syndrome)	chr16	protein_coding
ENSG00000148600.13	5.59946861	-2.6599164	0.00086564	0.00288128	0	0	0.04	0.01	CDHR1	cadherin-related family member 1	chr10	protein_coding
ENSG00000230590.6	3.00324176	-0.3432631	4.94E-09	3.95E-08	0.07	0.02	0.75	0.29	FTX	FTX transcript, XIST regulator (non-protein coding)	chrX	lincRNA
ENSG00000198336.8	5.36404116	-2.8022277	0.00290522	0.00856312	0	0	0.3	0.06	MYL4	myosin, light chain 4, alkali; atrial, embryonic	chr17	protein_coding
ENSG00000280128.1	2.045251	0.23947488	5.65E-07	3.39E-06	0.14	0.05	0.52	0.51	XXbac-BPG2	--	chr6	TEC
ENSG00000085377.12	-1.3078034	5.37084001	7.03E-06	3.51E-05	17.76	15.05	7.56	10.17	PREP	prolyl endopeptidase	chr6	protein_coding
ENSG00000159479.15	-1.2613864	5.67085277	1.39E-05	6.59E-05	29.1	28.02	14.59	17.22	MED8	mediator complex subunit 8	chr1	protein_coding
ENSG00000196323.10	1.7150123	2.53524673	6.34E-08	4.37E-07	0.17	0.24	0.82	0.98	ZBTB44	zinc finger and BTB domain containing 44	chr11	protein_coding
ENSG00000160469.15	-1.9425473	2.22563257	2.20E-09	1.84E-08	1.73	1.65	0.73	0.44	BRSK1	BR serine/threonine kinase 1	chr19	protein_coding
ENSG00000157927.15	9.22429522	0.305949	1.25E-23	3.94E-22	0	0	0.63	0.69	RADIL	Ras association and DIL domains	chr7	protein_coding
ENSG00000075340.21	8.90100338	4.42184976	5.90E-74	2.95E-71	0	0.01	4.15	4.03	ADD2	adducin 2 (beta)	chr2	protein_coding
ENSG00000237330.2	1.94696031	-0.4157084	3.70E-05	0.00016218	0.03	0.21	0.72	0.49	RNF223	ring finger protein 223	chr1	protein_coding
ENSG00000091317.7	2.65850259	2.97985746	3.11E-16	5.52E-15	0.24	0.66	2.8	4.73	CMTM6	CKLF-like MARVEL transmembrane domain containing 6	chr3	protein_coding
ENSG00000189308.9	3.15922417	0.64093258	1.21E-13	1.67E-12	0.01	0.07	0.43	0.47	LIN54	lin-54 DREAM MuvB core complex component	chr4	protein_coding
ENSG00000215196.4	-1.2789131	1.437449	0.00010677	0.00042715	1.29	0.79	0.4	0.74	AC091878.1	--	chr5	antisense
ENSG00000272993.1	7.8342183	-0.9450734	6.98E-12	7.96E-11	0	0	1	1.75	RP11-196G1	--	chr1	lincRNA
ENSG00000160055.18	-1.0328992	2.99304283	0.00064827	0.00222098	6.86	5.34	4.13	3.82	TMEM234	transmembrane protein 234	chr1	protein_coding
ENSG00000134755.13	-6.9367384	-1.8821835	8.18E-08	5.55E-07	0.03	0.03	0	0	DSC2	desmocollin 2	chr18	protein_coding
ENSG00000108306.10	1.38821104	4.05879786	3.05E-06	1.63E-05	0.69	0.69	2.47	2.35	FBXL20	F-box and leucine-rich repeat protein 20	chr17	protein_coding
ENSG00000136933.15	-2.4277215	5.9380384	6.92E-16	1.19E-14	24.8	24.42	5.82	6.38	RABEPK	Rab9 effector protein with kelch motifs	chr9	protein_coding
ENSG00000185664.13	1.43670437	-0.8590692	0.00438177	0.01246521	0.07	0.08	0.18	0.35	PMEL	premelanosome protein	chr12	protein_coding
ENSG00000086717.17	-3.9468237	-1.7623731	2.66E-06	1.44E-05	0.12	0.15	0.02	0	PPEF1	protein phosphatase, EF-hand calcium binding domain 1	chrX	protein_coding
ENSG00000272514.4	6.63760135	-1.9357476	6.25E-07	3.72E-06	0	0	0.25	0.12	C6orf165	chromosome 6 open reading frame 165	chr6	protein_coding
ENSG00000167601.10	-2.944209	8.13013065	5.87E-22	1.64E-20	76.44	87.72	13.97	14.44	AXL	AXL receptor tyrosine kinase	chr19	protein_coding
ENSG00000136689.17	-6.1903293	-2.4391943	4.74E-05	0.00020301	0.08	0.11	0	0	IL1RN	interleukin 1 receptor antagonist	chr2	protein_coding
ENSG00000185122.9	-1.174905	7.77488829	4.52E-05	0.00019566	108.95	111.19	68.64	61.04	HSF1	heat shock transcription factor 1	chr8	protein_coding
ENSG00000235077.1	5.18753465	-2.9024431	0.00538165	0.01488851	0	0	0	0.8	AC073842.19	--	chr7	antisense
ENSG00000153208.15	2.29659127	-0.7629918	8.33E-06	4.11E-05	0.06	0	0.22	0.2	MERTK	MER proto-oncogene, tyrosine kinase	chr2	protein_coding
ENSG00000100784.8	1.13014986	0.41244156	0.00304162	0.00893004	0.02	0.03	0.05	0.08	RPS6KA5	ribosomal protein S6 kinase, 90kDa, polypeptide 5	chr14	protein_coding
ENSG00000276170.3	-10.783636	1.73145377	2.04E-43	1.91E-41	5.06	4.6	0	0	AC124789.1	--	chr17	processed_transcript
ENSG00000088756.11	-1.6563196	-1.7096608	0.00684841	0.01852289	0.03	0.04	0.01	0.02	ARHGAP28	Rho GTPase activating protein 28	chr18	protein_coding
ENSG00000242798.1	-1.6467184	-1.2689992	0.00277048	0.00827794	0.89	0.69	0.28	0.38	RP11-506M1	--	chr7	antisense
ENSG00000138311.14	1.10218035	-0.2735614	0.01115546	0.02843971	0.04	0.06	0.13	0.17	ZNF365	zinc finger protein 365	chr10	protein_coding
ENSG00000272482.1	2.04498234	-0.721884	5.52E-05	0.00023355	0.93	0	2.97	2.37	RP11-474O2	--	chr1	lincRNA
ENSG00000156049.6	7.1604394	-1.5141474	2.80E-09	2.31E-08	0	0	0.15	0.31	GNA14	guanine nucleotide binding protein (G protein), alpha 14	chr9	protein_coding
ENSG00000131061.12	1.21918999	3.32213312	5.05E-05	0.00021563	1.45	1.17	4.46	3.65	ZNF341	zinc finger protein 341	chr20	protein_coding
ENSG00000154309.8	-1.6351339	2.84202715	1.19E-07	7.95E-07	1.6	2.09	0.79	0.78	DISP1	dispatched homolog 1 (Drosophila)	chr1	protein_coding
ENSG00000185267.8	4.32521474	-1.0707269	4.33E-09	3.50E-08	0.04	0	0.77	0.33	CDNF	cerebral dopamine neurotrophic factor	chr10	protein_coding
ENSG00000158571.9	5.94755028	-2.430277	0.00014842	0.00057423	0	0	0.08	0.16	PFKFB1	6-phosphofructo-2-kinase/fructose-2,6-biphosphatase 1	chrX	protein_coding
ENSG00000186847.5	-3.9795686	-2.3830119	0.00039332	0.00140265	0.23	0.03	0.02	0	KRT14	keratin 14, type I	chr17	protein_coding
ENSG00000066739.10	2.25487837	1.8630143	3.40E-11	3.57E-10	0.09	0.06	0.37	0.55	ATG2B	autophagy related 2B	chr14	protein_coding
ENSG00000189221.8	-2.9864139	5.06566407	9.37E-22	2.59E-20	8.87	8.84	1.57	1.41	MAOA	monoamine oxidase A	chrX	protein_coding
ENSG00000127990.14	-7.3786188	1.37767671	4.24E-37	2.73E-35	1.62	2.04	0.03	0	SGCE	sarcoglycan, epsilon	chr7	protein_coding
ENSG00000126882.11	1.86347016	3.72328925	9.59E-10	8.43E-09	1	1.1	4.99	5.21	FAM78A	family with sequence similarity 78, member A	chr9	protein_coding
ENSG00000156976.13	1.67446206	7.00549054	1.01E-08	7.72E-08	25.44	26.17	94.31	125.99	EIF4A2	eukaryotic translation initiation factor 4A2	chr3	protein_coding
ENSG00000184898.6	3.12700233	-0.5450387	1.73E-08	1.28E-07	0	0.04	0.18	0.31	RBM43	RNA binding motif protein 43	chr2	protein_coding
ENSG00000105373.17	-1.6272923	9.85182824	2.24E-08	1.63E-07	760.92	718.21	342.63	294.1	GLTSCR2	glioma tumor suppressor candidate region gene 2	chr19	protein_coding
ENSG00000269403.1	-2.9341358	-1.3076562	2.24E-06	1.22E-05	0.68	1.23	0.12	0.2	CTD-2616J1	--	chr19	protein_coding
ENSG00000171617.12	-1.5674636	2.69347191	4.25E-07	2.60E-06	1.34	1.42	0.6	0.64	ENC1	ectodermal-neural cortex 1 (with BTB domain)	chr5	protein_coding
ENSG00000198763.3	1.56092248	11.1124062	7.63E-08	5.20E-07	589.12	1158.33	3910.92	2908.12	MT-ND2	mitochondrially encoded NADH dehydrogenase 2	chrM	protein_coding
ENSG00000077254.13	1.61587808	1.84420607	1.03E-06	5.91E-06	0.3	0.31	1.02	1.46	USP33	ubiquitin specific peptidase 33	chr1	protein_coding
ENSG00000181704.10	1.38043844	3.17409524	5.64E-06	2.87E-05	0.53	0.66	1.75	2.4	YIPF6	Yip1 domain family, member 6	chrX	protein_coding
ENSG00000236017.6	1.10302834	1.06955513	0.00159139	0.00498286	0.54	1.02	1.94	2.51	ASMTL-AS1	ASMTL antisense RNA 1	chrX	antisense
ENSG00000187736.11	-1.5793894	4.17845335	1.16E-07	7.74E-07	9.47	11.47	4.98	4.34	NHEJ1	nonhomologous end-joining factor 1	chr2	protein_coding
ENSG00000146350.12	2.19201969	-2.201185	0.01384632	0.03449994	0.01	0.01	0.07	0.05	TBC1D32	TBC1 domain family, member 32	chr6	protein_coding
ENSG00000103194.14	1.02904588	5.25027318	0.00038582	0.0013796	6.33	6.59	15.43	19.81	USP10	ubiquitin specific peptidase 10	chr16	protein_coding
ENSG00000132470.12	-2.2185404	7.23899929	8.59E-14	1.21E-12	35.15	29.89	9.87	8.73	ITGB4	integrin, beta 4	chr17	protein_coding
ENSG00000222009.7	-1.571161	-1.6404219	0.00945988	0.02474059	0.19	0.23	0.04	0.14	BTBD19	BTB (POZ) domain containing 19	chr1	protein_coding

ENSG00000232931.4	8.14272985	-0.6721839	4.35E-14	6.30E-13	0	0	0.3	0.67	LINC00342	long intergenic non-protein coding RNA 342	chr2	lincRNA
ENSG00000254290.1	-6.5075175	-2.2122527	3.03E-06	1.62E-05	0.11	0.12	0	0	RP11-150O1	--	chr8	lincRNA
ENSG00000147168.11	-1.8341332	-0.0827609	1.07E-05	5.16E-05	1.06	0.42	0.29	0.27	IL2RG	interleukin 2 receptor, gamma	chrX	protein_coding
ENSG00000263786.1	2.47110292	-0.1942084	8.27E-08	5.60E-07	0.22	0.32	2.64	1.41	RP11-649A1	--	chr17	sense_intronic
ENSG00000168803.13	1.32175122	1.81626174	5.23E-05	0.00022233	0.27	0.42	1.16	1.12	ADAL	adenosine deaminase-like	chr15	protein_coding
ENSG00000255276.1	6.62177131	-1.945154	6.25E-07	3.72E-06	0	0	1.2	1.1	RRM1-AS1	RRM1 antisense RNA 1	chr11	antisense
ENSG00000168899.4	-1.5684934	0.06844507	0.00011265	0.00044808	1.71	1.85	0.93	0.67	VAMP5	vesicle-associated membrane protein 5	chr2	protein_coding
ENSG00000227811.2	1.66634048	-1.3352298	0.00496338	0.01395792	0.09	0.03	0.12	0.37	FAM212B-AS	FAM212B antisense RNA 1	chr1	antisense
ENSG00000180035.9	-1.26582	5.08534422	1.41E-05	6.68E-05	10.12	11.21	5.96	5.85	ZNF48	zinc finger protein 48	chr16	protein_coding
ENSG00000273424.1	-6.6435887	-2.1103563	1.05E-06	6.02E-06	0.67	0.71	0	0	CTA-223H9.9	--	chr22	lincRNA
ENSG00000107669.16	1.35764432	1.04114557	0.0001051	0.00042187	0.17	0.15	0.43	0.68	ATE1	arginyltransferase 1	chr10	protein_coding
ENSG00000079337.14	-6.0127549	3.85599256	8.04E-53	1.33E-50	2.42	2.76	0.06	0.05	RAPGEF3	Rap guanine nucleotide exchange factor (GEF) 3	chr12	protein_coding
ENSG00000257298.1	4.09099088	-1.2560165	9.32E-08	6.27E-07	0.01	0	0.14	0.15	RP3-405J10.	--	chr12	sense_intronic
ENSG00000164463.11	3.13186525	-0.2254758	6.27E-10	5.63E-09	0.02	0.01	0.11	0.23	CREBRF	CREB3 regulatory factor	chr5	protein_coding
ENSG00000134569.8	-1.8978365	4.87308764	1.92E-10	1.85E-09	4.46	4.81	1.68	1.63	LRP4	low density lipoprotein receptor-related protein 4	chr11	protein_coding
ENSG00000102890.13	1.20864648	2.97823505	7.05E-05	0.00029232	1.23	1.74	4.68	4.43	ELMO3	engulfment and cell motility 3	chr16	protein_coding
ENSG00000157259.6	1.07005164	3.76683846	0.0003079	0.00111716	2.02	2.18	5.21	6.58	GATAD1	GATA zinc finger domain containing 1	chr7	protein_coding
ENSG00000279207.1	1.79818704	1.40559697	2.00E-07	1.29E-06	0.15	0.17	0.85	0.62	RP11-159D1	--	chr17	TEC
ENSG00000172061.8	-7.7047195	-1.2361932	6.01E-12	6.93E-11	0.07	0.15	0	0	LRRC15	leucine rich repeat containing 15	chr3	protein_coding
ENSG00000163848.17	3.49786466	1.40114625	7.68E-19	1.69E-17	0.04	0.03	0.46	0.57	ZNF148	zinc finger protein 148	chr3	protein_coding
ENSG00000174579.3	1.86319747	2.74499665	3.55E-09	2.90E-08	0.42	0.53	1.8	2.83	MSL2	male-specific lethal 2 homolog (Drosophila)	chr3	protein_coding
ENSG00000277200.1	-2.6125527	-2.6400256	0.01923175	0.0458365	0.08	0.02	0.02	0	RP11-74E22.	--	chr17	lincRNA
ENSG00000164542.11	2.16585948	-0.022613	7.83E-07	4.58E-06	0.03	0.05	0.16	0.32	KIAA0895	KIAA0895	chr7	protein_coding
ENSG00000114127.9	3.85048859	0.97759949	9.88E-19	2.16E-17	0.03	0.01	0.28	0.41	XRN1	5'-3' exoribonuclease 1	chr3	protein_coding
ENSG00000161544.8	3.30843212	-0.3997727	1.04E-09	9.04E-09	0	0.07	0.51	0.48	CYGB	cytoglobin	chr17	protein_coding
ENSG00000109072.12	9.94074378	4.75947298	4.99E-81	3.42E-78	0.01	0.03	29.41	24.52	VTN	vitronectin	chr17	protein_coding
ENSG00000102804.13	-1.6876422	6.32120713	8.19E-09	6.35E-08	13.81	15.69	5.21	7.03	TSC22D1	TSC22 domain family, member 1	chr13	protein_coding
ENSG00000188342.10	1.07190177	2.27004177	0.0006513	0.00222977	0.49	1.73	2.19	4.03	GTF2F2	general transcription factor IIF, polypeptide 2, 30kDa	chr13	protein_coding
ENSG00000162924.12	4.09283183	0.74476242	6.63E-19	1.48E-17	0.01	0.02	0.29	0.28	REL	v-rel avian reticuloendotheliosis viral oncogene homolog	chr2	protein_coding
ENSG00000213445.7	-1.393429	5.66843211	1.72E-06	9.58E-06	16.37	14.13	8.06	7.4	SIPA1	signal-induced proliferation-associated 1	chr11	protein_coding
ENSG00000109756.7	2.14054805	1.34847687	1.50E-09	1.28E-08	0.1	0.11	0.49	0.75	RAPGEF2	Rap guanine nucleotide exchange factor (GEF) 2	chr4	protein_coding
ENSG00000152217.15	-9.2152067	0.17869174	1.59E-24	5.30E-23	0.17	0.16	0	0	SETBP1	SET binding protein 1	chr18	protein_coding
ENSG00000267194.1	4.89980841	-3.0567113	0.01889873	0.04519557	0	0	0.05	0.04	RP1-193H18	--	chr17	lincRNA
ENSG00000113361.11	-1.6449927	-0.6595825	0.00042865	0.00151894	0.11	0.05	0.03	0.04	CDH6	cadherin 6, type 2, K-cadherin (fetal kidney)	chr5	protein_coding
ENSG00000153790.10	1.79389294	-0.3719066	0.00010606	0.0004253	0.01	0.12	0.3	0.32	C7orf31	chromosome 7 open reading frame 31	chr7	protein_coding
ENSG00000198879.10	-9.1126391	0.07918776	1.78E-23	5.52E-22	0.19	0.15	0	0	SFMBT2	Scm-like with four mbt domains 2	chr10	protein_coding
ENSG00000213020.7	3.03022741	0.35783872	8.63E-12	9.76E-11	0.04	0.05	0.46	0.48	ZNF611	zinc finger protein 611	chr19	protein_coding
ENSG00000240065.6	1.86680381	2.20916079	9.49E-09	7.29E-08	2.32	1.08	8.45	8.19	PSMB9	proteasome (prosome, macropain) subunit, beta type, 9	chr6	protein_coding
ENSG00000139133.5	2.23630613	-0.4690451	3.89E-06	2.04E-05	0.08	0.03	0.23	0.48	ALG10	ALG10, alpha-1,2-glucosyltransferase	chr12	protein_coding
ENSG00000130584.9	1.22289508	1.47181053	0.00023139	0.00086549	0.29	0.2	0.73	0.79	ZBTB46	zinc finger and BTB domain containing 46	chr20	protein_coding
ENSG00000144115.15	6.52223186	3.29103066	4.71E-51	6.69E-49	0.03	0.05	5.27	4.84	THNSL2	threonine synthase-like 2 (S. cerevisiae)	chr2	protein_coding
ENSG00000115947.12	1.81757579	2.05831499	3.01E-08	2.15E-07	0.13	0.29	0.78	1.21	ORC4	origin recognition complex, subunit 4	chr2	protein_coding
ENSG00000246089.3	-1.9624798	1.75885929	3.98E-09	3.24E-08	1.46	1.5	0.55	0.46	RP11-115C2	--	chr8	antisense
ENSG00000021776.9	1.57324618	4.15937472	1.34E-07	8.84E-07	0.68	0.81	2.73	3.21	AQR	aquarius intron-binding spliceosomal factor	chr15	protein_coding
ENSG00000246228.5	-7.0905607	-1.7587175	1.16E-08	8.76E-08	0.11	0.24	0	0	CASC8	cancer susceptibility candidate 8 (non-protein coding)	chr8	antisense
ENSG00000258429.1	1.26503701	1.4120381	0.00017745	0.00067659	1.13	0.98	3.21	3.57	PDF	peptide deformylase (mitochondrial)	chr16	protein_coding
ENSG00000175054.13	3.63678097	1.35127683	1.97E-19	4.54E-18	0.01	0.07	0.4	0.78	ATR	ATR serine/threonine kinase	chr3	protein_coding
ENSG00000070601.8	5.97010963	-2.4174709	8.36E-05	0.00034052	0	0	0.06	0.03	FRMPD1	FERM and PDZ domain containing 1	chr9	protein_coding
ENSG00000185436.10	1.04339703	3.3355558	0.00049086	0.00171585	1.13	1.02	2.92	2.99	IFNLR1	interferon, lambda receptor 1	chr1	protein_coding
ENSG00000118260.13	1.41084529	1.55452776	2.56E-05	0.00011523	0.11	0.22	0.44	0.71	CREB1	cAMP responsive element binding protein 1	chr2	protein_coding
ENSG00000173926.5	2.14135787	1.42248008	1.34E-09	1.15E-08	0.17	0.18	1.15	0.88	3-Mar	membrane-associated ring finger (C3HC4) 3, E3 ubiquitin protein ligase	chr5	protein_coding
ENSG00000141127.13	1.62682287	3.63784477	7.43E-08	5.07E-07	2.17	2.32	7.02	11.57	PRPSAP2	phosphoribosyl pyrophosphate synthetase-associated protein 2	chr17	protein_coding
ENSG00000213123.9	-2.2186003	-0.6129171	2.53E-06	1.37E-05	1.19	1.26	0.24	0.45	TCTEX1D2	Tctex1 domain containing 2	chr3	protein_coding
ENSG00000199753.1	-1.5060115	-0.065979	0.00028998	0.00105871	17.96	13.36	6.86	7.78	SNORD104	small nucleolar RNA, C/D box 104	chr17	snoRNA
ENSG00000185015.6	-1.7737128	-2.0719142	0.01397446	0.03477946	0.03	0.1	0.03	0.01	CA13	carbonic anhydrase XIII	chr8	protein_coding
ENSG00000116171.15	1.16609585	2.26062667	0.00021688	0.00081477	0.3	0.43	0.71	1.47	SCP2	sterol carrier protein 2	chr1	protein_coding
ENSG00000125629.13	1.12810239	1.60022213	0.00061273	0.00210918	0.68	0.47	1.41	1.97	INSIG2	insulin induced gene 2	chr2	protein_coding
ENSG00000175395.14	6.00485112	-2.3883666	8.36E-05	0.00034052	0	0	0	0.12	ZNF25	zinc finger protein 25	chr10	protein_coding
ENSG00000137819.12	-3.6698676	2.72324904	5.24E-26	1.92E-24	1.68	1.77	0.16	0.2	PAQR5	progestin and adipoQ receptor family member V	chr15	protein_coding
ENSG00000175550.6	-1.6997494	8.75619085	5.68E-09	4.51E-08	484.45	520.29	210.86	200.79	DRAP1	DR1-associated protein 1 (negative cofactor 2 alpha)	chr11	protein_coding
ENSG00000260920.2	2.83778392	-2.1769859	0.00550654	0.01520944	0	0.02	0.03	0.2	RP1-228H13	--	chr1	sense_overlapping
ENSG00000169093.13	-1.6413907	5.94221362	2.09E-08	1.52E-07	33.81	33.79	14.85	13.99	ASMTL	acetylserotonin O-methyltransferase-like	chrX	protein_coding
ENSG00000156453.12	-3.4074785	4.43188144	3.57E-26	1.33E-24	5.97	5.83	0.9	0.58	PCDH1	protocadherin 1	chr5	protein_coding
ENSG00000116701.13	-7.9601682	1.94306132	3.05E-46	3.43E-44	2.13	2.96	0	0.02	NCF2	neutrophil cytosolic factor 2	chr1	protein_coding
ENSG00000184216.10	-2.0638091	9.83044671	2.67E-12	3.20E-11	333.95	301.25	100.77	101.77	IRAK1	interleukin-1 receptor-associated kinase 1	chrX	protein_coding
ENSG00000049769.11	-1.3549777	2.95689024	8.79E-06	4.32E-05	2.09	2.35	1.18	1.13	PPP1R3F	protein phosphatase 1, regulatory subunit 3F	chrX	protein_coding
ENSG00000221083.1	-3.313687	-2.7865859	0.00943894	0.02469931	2.14	0	0.26	0	SNORA77	small nucleolar RNA, H/ACA box 77	chr1	snoRNA
ENSG00000169193.10	4.33101328	-1.0518339	6.30E-09	4.98E-08	0.01	0.01	0.16	0.45	CCDC126	coiled-coil domain containing 126	chr7	protein_coding
ENSG00000155755.17	1.3926674	3.28832663	4.42E-06	2.29E-05	0.63	0.87	2.09	3.19	TMEM237	transmembrane protein 237	chr2	protein_coding
ENSG00000174599.5	-5.6565858	-2.7839092	0.00086564	0.00288128	0.09	0.06	0	0	TRAM1L1	translocation associated membrane protein 1-like 1	chr4	protein_coding
ENSG00000108551.4	1.94734697	1.85111735	6.22E-09	4.92E-08	0.77	0.57	3.23	3.66	RASD1	RAS, dexamethasone-induced 1	chr17	protein_coding
ENSG00000054983.15	3.10341979	1.80096493	1.55E-17	3.08E-16	0.09	0.15	0.94	1.84	GALC	galactosylceramidase	chr14	protein_coding
ENSG00000261490.1	3.39014446	-2.4340326	0.00190233	0.00584723	0	0.01	0.02	0.11	RP11-448G1	--	chr4	sense_overlapping
ENSG00000112218.8	6.78231171	-1.8229491	1.35E-07	8.87E-07	0	0	0.09	0.06	GPR63	G protein-coupled receptor 63	chr6	protein_coding
ENSG00000128594.6	3.58952926	-2.3068871	0.00039332	0.00140265	0	0.01	0.08	0.06	LRRC4	leucine rich repeat containing 4	chr7	protein_coding
ENSG00000134463.13	-10.044905	4.03872903	5.32E-75	2.77E-72	18.12	14.13	0.04	0	ECHDC3	enoyl CoA hydratase domain containing 3	chr10	protein_coding
ENSG00000213927.3	-2.8641168	-2.5097724	0.00190233	0.00584723	0.36	0.44	0	0.12	CCL27	chemokine (C-C motif) ligand 27	chr9	protein_coding
ENSG00000274270.1	2.07961147	0.379546	2.30E-07	1.46E-06	0.03	0.26	1	0.6	RP11-34F20.	--	chr13	sense_intronic
ENSG00000154582.15	-1.4245925	4.6558644	1.29E-06	7.28E-06	5.65	6.4	3.16	2.81	TCEB1	transcription elongation factor B (SIII), polypeptide 1 (15kDa, elongin C)	chr8	protein_coding
ENSG00000177666.14	1.38824165	5.82886068	1.86E-06	1.03E-05	7.78	6.85	26.7	24.31	PNPLA2	patatin-like phospholipase domain containing 2	chr11	protein_coding
ENSG00000095370.18	1.34609062	-1.0113928	0.00917505	0.0241697	0.06	0.03	0.21	0.1	SH2D3C	SH2 domain containing 3C	chr9	protein_coding
ENSG00000166825.12	-11.164362	8.64066478	1.99E-129	1.59E-125	172.46	178.9	0.13	0.07	ANPEP	alanyl (membrane) aminopeptidase	chr15	protein_coding
ENSG00000177453.6	3.06679279	0.09178153	4.13E-11	4.31E-10	0.03	0.07	0.7	0.51	NIM1K	NIM1 serine/threonine protein kinase	chr5	protein_coding
ENSG00000074317.9	6.04022639	-2.3681409	8.36E-05	0.00034052	0	0	0.16	0.17	SNCB	synuclein, beta	chr5	protein_coding
ENSG00000145375.7	1.32629933	1.32798839	9.23E-05	0.00037338	0.12	0.16	0.36	0.56	SPATA5	spermatogenesis associated 5	chr4	protein_coding
ENSG00000188372.13	-1.7292191	3.16730359	1.86E-08	1.37E-07	4.67	4.23	2.12	1.45	ZP3	zona pellucida glycoprotein 3 (sperm receptor)	chr7	protein_coding
ENSG00000226572.1	1.86989634	-1.0681558	0.0009149	0.00303221	1.11	2.76	11.12	7.94	SNORD57	small nucleolar RNA, C/D box 57	chr20	snoRNA
ENSG00000256087.5	3.28547272	0.4867332	1.76E-13	2.38E-12	0.04	0.04	0.37	0.7	ZNF432	zinc finger protein 432	chr19	protein_coding
ENSG00000172159.14	-2.2566967	2.35825353	4.29E-12	5.01E-11	1.03	1.2	0.36	0.26	FRMD3	FERM domain containing 3	chr9	protein_coding
ENSG00000110427.13	-2.4758137	1.48187187	1.88E-12	2.30E-11	0.29	0.33	0.07	0.08	KIAA1549L	KIAA1549-like	chr11	protein_coding
ENSG00000205084.9	-1.0349417	3.83179619	0.00045494	0.0016026	3.44	3.63	2.26	2.33	TMEM231	transmembrane protein 231	chr16	protein_coding
ENSG00000177425.9	1.46560163	3.58635358	1.14E-06	6.47E-06	0.43	0.7	1.49	2.67	PAWR	PRKC, apoptosis, WT1, regulator	chr12	protein_coding
ENSG00000204131.7	5.45007274	-2.7494288	0.00158008	0.00495521	0	0	0.02	0.07	NHSL2	NHS-like 2	chrX	protein_coding
ENSG00000168477.16	-1.7446619	4.13165294	6.01E-09	4.76E-08	1.75	1.22	0.73	0.45	TNXB	tenascin XB	chr6	protein_coding
ENSG00000169118.14	1.11903314	2.67561577	0.00027482	0.00100781	0.33	0.46	0.95	1.33	CSNK1G1	casein kinase 1, gamma 1	chr15	protein_coding
ENSG00000112697.14	1.61753619	3.10561784	1.40E-07	9.21E-07	0.53	0.82	1.83	3.71	TMEM30A	transmembrane protein 30A	chr6	protein_coding
ENSG00000239672.6	-1.458404	8.64507854	4.84E-07	2.93E-06	268.2	213.77	118.8	115.02	NME1	NME/NM23 nucleoside diphosphate kinase 1	chr17	protein_coding
ENSG00000145416.12	-5.3742532	-2.9467828	0.00290522	0.00856312	0.02	0.02	0	0	1-Mar	membrane-associated ring finger (C3HC4) 1, E3 ubiquitin protein ligase	chr4	protein_coding
ENSG00000234233.1	-2.2961639	-0.7419822	2.00E-06	1.10E-05	0.9	1.54	0.1	0.52	KCNH1-IT1	KCNH1 intronic transcript 1	chr1	sense_intronic
ENSG00000142619.4	-6.5075175	-2.2122527	3.03E-06	1.62E-05	0.08	0.09	0	0	PADI3	peptidyl arginine deiminase, type III	chr1	protein_coding
ENSG00000258137.4	-5.5249898	-2.8618556	0.00158008	0.00495521	0.09	0.21	0	0	RP11-753H1	--	chr12	antisense
ENSG00000235194.7	1.34075355	2.41508472	2.01E-05	9.23E-05	0.41	0.6	1.64	1.76	PPP1R3E	protein phosphatase 1, regulatory subunit 3E	chr14	protein_coding
ENSG00000164078.11	2.37700696	3.61344622	2.76E-14	4.10E-13	0.8	0.52	4.8	4.39	MST1R	macrophage stimulating 1 receptor	chr3	protein_coding
ENSG00000138434.15	2.12011386	3.52335326	7.07E-12	8.05E-11	0.59	0.71	2.68	4.95	SSFA2	sperm specific antigen 2	chr2	protein_coding
ENSG00000136870.9	1.63547956	0.46983388	2.40E-05	0.00010869	0.12	0.17	0.51	0.72	ZNF189	zinc finger protein 189	chr9	protein_coding
ENSG00000139998.13	1.67637217	2.53792044	1.16E-07	7.74E-07	0.35	0.85	2.6	2.47	RAB15	RAB15, member RAS oncogene family	chr14	protein_coding
ENSG00000138459.7	1.59199067	1.3318543	4.06E-06	2.12E-05	0.24	0.18	0.6	1.1	SLC35A5	solute carrier family 35, member A5	chr3	protein_coding
ENSG00000174738.11	2.63125516	1.42961526	5.35E-13	6.90E-12	0.08	0.14	0.72	1.11	NR1D2	nuclear receptor subfamily 1, group D, member 2	chr3	protein_coding
ENSG00000122884.11	1.34538921	4.07806813	5.84E-06	2.96E-05	2.89	2.35	7.88	9.94	P4HA1	prolyl 4-hydroxylase, alpha polypeptide I	chr10	protein_coding
ENSG00000215386.9	-4.2792529	0.51792035	1.00E-19	2.35E-18	0.55	0.71	0.04	0.05	MIR99AHG	mir-99a-let-7c cluster host gene	chr21	lincRNA
ENSG00000154330.11	8.50015885	-0.3568403	6.57E-17	1.23E-15	0	0	0.34	0.45	PGM5	phosphoglucomutase 5	chr9	protein_coding
ENSG00000272097.1	-2.6240494	-2.6454115	0.00552196	0.01522223	0.22	0.05	0	0.05	RP11-421M1	--	chr6	lincRNA
ENSG00000162891.9	3.01037881	-1.0533246	1.22E-06	6.93E-06	0.07	0.02	0.16	0.8	IL20	interleukin 20	chr1	protein_coding
ENSG00000130038.8	5.89552119	-2.4689618	0.00014842	0.00057423	0	0	0.12	0.03	CRACR2A	calcium release activated channel regulator 2A	chr12	protein_coding
ENSG00000279873.2	5.60914027	-2.6545652	0.00047761	0.00167191	0	0	0.23	0.02	LINC01126	long intergenic non-protein coding RNA 1126	chr2	lincRNA
ENSG00000157570.10	-5.9291464	1.63489074	8.56E-35	4.92E-33	1.15	1.1	0.05	0	TSPAN18	tetraspanin 18	chr11	protein_coding
ENSG00000233903.2	1.09885546	-0.6581289	0.02038174	0.04829885	0.69	0.36	2.17	0.9	Z83851.4	--	chr22	lincRNA
ENSG00000131831.16	3.871173	-0.3520044	5.51E-12	6.39E-11	0.02	0.03	0.54	0.53	RAI2	retinoic acid induced 2	chrX	protein_coding
ENSG00000078401.6	-4.2199254	-0.2290842	2.32E-15	3.83E-14	0.53	0.74	0.03	0.05	EDN1	endothelin 1	chr6	protein_coding
ENSG00000015171.17	2.36423869	2.97834365	1.46E-13	1.99E-12	0.33	0.49	1.88	3.77	ZMYND11	zinc finger, MYND-type containing 11	chr10	protein_coding
ENSG00000240207.5	1.94213991	0.87146685	1.98E-07	1.27E-06	0.14	0.14	0.54	0.88	RP11-379F4.	--	chr3	antisense
ENSG00000226029.1	1.43419489	-0.7801409	0.00340877	0.00991568	0.07	0.07	0.2	0.29	RP4-798A10	--	chr1	lincRNA
ENSG00000236754.4	7.06681771	-1.59382	7.18E-09	5.62E-08	0	0	0.26	0.29	AC004019.1	--	chr22	antisense
ENSG00000163873.8	2.90942026	-2.7238075	0.00943894	0.02469931	0	0	0.03	0	GRIK3	glutamate receptor, ionotropic, kainate 3	chr1	protein_coding
ENSG00000260367.2	-2.6256661	-1.5270823	6.51E-05	0.00027249	0.51	0.06	0.07	0.06	RP11-264B1	--	chr16	antisense
ENSG00000103067.10	2.48033157	4.66338969	5.95E-16	1.03E-14	1.41	1.82	12.64	11.37	ESRP2	epithelial splicing regulatory protein 2	chr16	protein_coding
ENSG00000130997.15	2.48390275	-2.404449	0.00737015	0.01977584	0	0.02	0.12	0.03	POLN	polymerase (DNA directed) nu	chr4	protein_coding
ENSG00000240006.1	4.87917439	-3.0678699	0.01889873	0.04519557	0	0	0	0.74	RP11-200A1	--	chr3	lincRNA
ENSG00000119714.9	6.69726829	-1.8825742	3.73E-07	2.30E-06	0	0	0.07	0.2	GPR68	G protein-coupled receptor 68	chr14	protein_coding
ENSG00000273432.1	5.3196533	-2.8266272	0.00290522	0.00856312	0	0	0	0.14	RP5-1165K1	--	chr7	processed_transcript
ENSG00000116039.10	2.27089123	1.12610947	6.82E-10	6.09E-09	0.31	0.3	2.37	1.57	ATP6V1B1	ATPase, H+ transporting, lysosomal 56/58kDa, V1 subunit B1	chr2	protein_coding
ENSG00000137992.13	2.28773634	1.57026659	7.23E-11	7.38E-10	0.06	0.08	0.31	0.6	DBT	dihydrolipoamide branched chain transacylase E2	chr1	protein_coding
ENSG00000108515.16	-1.9302937	5.30979104	7.55E-11	7.69E-10	35.4	28.46	11.6	10.73	ENO3	enolase 3 (beta, muscle)	chr17	protein_coding
ENSG00000122707.10	-1.4374474	0.79932305	7.09E-05	0.00029385	0.3	0.53	0.27	0.14	RECK	reversion-inducing-cysteine-rich protein with kazal motifs	chr9	protein_coding
ENSG00000272449.1	-3.5942655	-0.5747572	1.75E-11	1.91E-10	0.83	1.11	0.1	0.11	RP3-395M2	--	chr1	lincRNA
ENSG00000183208.11	1.5837784	2.76190026	3.79E-07	2.34E-06	0.39	0.63	2.19	1.86	GDPGP1	GDP-D-glucose phosphorylase 1	chr15	protein_coding
ENSG00000136867.9	1.30241292	2.14565938	4.45E-05	0.00019273	1.11	1.16	3.5	3.97	SLC31A2	solute carrier family 31 (copper transporter), member 2	chr9	protein_coding
ENSG00000132821.10	2.71879525	0.34874642	2.36E-10	2.24E-09	0	0.26	1.13	1.13	VSTM2L	V-set and transmembrane domain containing 2 like	chr20	protein_coding
ENSG00000134490.12	-1.0482178	2.99056046	0.00054412	0.00189066	3.2	2.45	2.32	1.31	TMEM241	transmembrane protein 241	chr18	protein_coding
ENSG00000260793.2	2.39261217	-0.1610068	2.77E-07	1.73E-06	0.03	0.18	0.91	0.55	RP5-882C2.2	--	chr17	antisense
ENSG00000280175.1	3.03042908	-2.6562433	0.00943894	0.02469931	0.04	0	0.47	0.08	AL354828.2	--	chr13	protein_coding
ENSG00000119514.6	-2.0400849	-2.128309	0.0061421	0.01678695	0.08	0.09	0	0.05	GALNT12	polypeptide N-acetylgalactosaminyltransferase 12	chr9	protein_coding
ENSG00000174842.15	2.65236534	0.21571516	1.68E-09	1.44E-08	0.13	0.1	0.48	1.5	GLMN	glomulin, FKBP associated protein	chr1	protein_coding
ENSG00000167644.10	-1.6348201	1.84483973	5.79E-07	3.47E-06	7.53	9.11	4.02	3.11	C19orf33	chromosome 19 open reading frame 33	chr19	protein_coding
ENSG00000251965.1	-5.0234041	-3.1294261	0.0100455	0.02582742	0.69	1.82	0	0	RNA5SP208	RNA, 5S ribosomal pseudogene 208	chr6	rRNA
ENSG00000145782.11	1.24247331	4.00300607	2.77E-05	0.00012385	1.75	1.79	4.48	6.73	ATG12	autophagy related 12	chr5	protein_coding
ENSG00000198853.10	-2.8434047	7.05198075	1.09E-20	2.77E-19	33.02	33.92	6.7	5.7	RUSC2	RUN and SH3 domain containing 2	chr9	protein_coding
ENSG00000233203.5	5.19250325	-2.8997172	0.00538165	0.01488851	0	0	0.04	0.32	RP11-67L3.4	--	chr1	antisense
ENSG00000224186.7	-1.1419341	1.21616092	0.00079661	0.00267967	0.36	0.41	0.25	0.22	C5orf66	chromosome 5 open reading frame 66	chr5	antisense
ENSG00000262302.1	-2.7114606	-2.2180661	0.00228047	0.00692082	0.44	0.52	0.13	0.06	RP1-4G17.5	--	chr17	protein_coding
ENSG00000275457.1	2.32874041	1.71497192	1.73E-11	1.89E-10	0.99	0.76	5.2	6.57	RP1-198K11.	--	chr20	antisense
ENSG00000065243.17	3.44154176	2.40453083	2.84E-22	8.11E-21	0.1	0.12	0.95	2.17	PKN2	protein kinase N2	chr1	protein_coding
ENSG00000074800.12	-1.3906388	12.901203	1.49E-06	8.37E-06	5356.42	4695.46	2500.87	2612.99	ENO1	enolase 1, (alpha)	chr1	protein_coding
ENSG00000168685.13	-2.3413933	-0.8751459	4.87E-06	2.51E-05	0.18	0.1	0.05	0.03	IL7R	interleukin 7 receptor	chr5	protein_coding
ENSG00000238741.1	3.29167326	4.09798205	2.47E-24	8.16E-23	6.89	8.59	141.04	58.75	SCARNA7	small Cajal body-specific RNA 7	chr3	scaRNA
ENSG00000122729.17	-1.0369072	6.4557203	0.00031766	0.0011517	12.99	12.29	7.92	8.51	ACO1	aconitase 1, soluble	chr9	protein_coding
ENSG00000138386.15	1.09966747	2.58044713	0.00038748	0.00138494	0.38	0.94	1.24	2.53	NAB1	NGFI-A binding protein 1 (EGR1 binding protein 1)	chr2	protein_coding
ENSG00000145723.15	1.87590186	-1.6655475	0.00684841	0.01852289	0.03	0.02	0.03	0.22	GIN1	gypsy retrotransposon integrase 1	chr5	protein_coding
ENSG00000113583.7	1.67211408	4.07771683	2.50E-08	1.80E-07	2.59	2.92	8.63	14.9	C5orf15	chromosome 5 open reading frame 15	chr5	protein_coding
ENSG00000198886.2	1.61661555	11.6743885	2.71E-08	1.96E-07	628.65	1266.6	4366.65	3321.73	MT-ND4	mitochondrially encoded NADH dehydrogenase 4	chrM	protein_coding
ENSG00000182810.6	1.15769461	5.27318936	6.78E-05	0.0002826	9.57	8.24	25.61	27.43	DDX28	DEAD (Asp-Glu-Ala-Asp) box polypeptide 28	chr16	protein_coding
ENSG00000173531.14	-1.0426284	0.86257309	0.00277464	0.0082883	0.71	0.77	0.33	0.62	MST1	macrophage stimulating 1	chr3	protein_coding
ENSG00000279041.1	3.20917399	-2.5475935	0.00552196	0.01522223	0	0.02	0.07	0.21	CTD-2373N4	--	chr8	TEC
ENSG00000167711.12	-3.1836579	-0.7433093	7.98E-09	6.19E-08	0.31	0.4	0.05	0.05	SERPINF2	serpin peptidase inhibitor, clade F (alpha-2 antiplasmin, pigment epithelium derived factor), member 2	chr17	protein_coding
ENSG00000213160.8	2.72457116	1.1724319	6.59E-13	8.42E-12	0.12	0.09	0.52	1.31	KLHL23	kelch-like family member 23	chr2	protein_coding
ENSG00000158352.14	-5.5276337	-2.8606046	0.00158008	0.00495521	0	0.03	0	0	SHROOM4	shroom family member 4	chrX	protein_coding
ENSG00000229852.2	-1.6830069	0.51249861	9.58E-06	4.67E-05	0.24	0.28	0.13	0.09	RP11-398K2	--	chr6	antisense
ENSG00000155366.15	-1.0372065	8.01798748	0.0003034	0.00110367	101.09	91.7	64.45	60.66	RHOC	ras homolog family member C	chr1	protein_coding
ENSG00000279970.1	-1.3810208	-0.2835177	0.00095011	0.00313547	0.13	0.8	0.18	0.29	RP11-299G2	--	chr15	TEC
ENSG00000189283.8	-2.9098096	0.83497912	1.12E-13	1.55E-12	1	1.67	0.29	0.19	FHIT	fragile histidine triad	chr3	protein_coding
ENSG00000151151.5	3.01105147	-0.3224154	3.80E-09	3.10E-08	0.01	0.03	0.17	0.27	IPMK	inositol polyphosphate multikinase	chr10	protein_coding
ENSG00000185561.9	1.32978519	3.78035872	8.30E-06	4.09E-05	0.8	1.31	3.41	3.65	TLCD2	TLC domain containing 2	chr17	protein_coding
ENSG00000183735.8	3.46154001	1.58663866	3.19E-19	7.26E-18	0.11	0.13	0.97	2.55	TBK1	TANK-binding kinase 1	chr12	protein_coding
ENSG00000272142.1	-2.2581902	-0.9374789	1.19E-05	5.68E-05	0.52	0.68	0.21	0.13	RP11-428J1.	--	chr6	lincRNA
ENSG00000128274.14	-4.6608797	7.59094064	5.22E-45	5.37E-43	121.75	122.03	7.58	5.21	A4GALT	alpha 1,4-galactosyltransferase	chr22	protein_coding
ENSG00000057294.12	-1.7009027	-0.6937669	0.00032297	0.00116954	0.13	0.19	0.04	0.09	PKP2	plakophilin 2	chr12	protein_coding
ENSG00000163810.10	-5.0206302	-3.1306986	0.0100455	0.02582742	0.04	0.02	0	0	TGM4	transglutaminase 4	chr3	protein_coding
ENSG00000279352.1	1.14435761	1.35290607	0.0007223	0.00245047	0.8	2.1	4.93	3.56	RP11-411B1	--	chr18	TEC
ENSG00000210174.1	3.55809421	-0.3926619	5.20E-11	5.38E-10	1.65	0.87	24.15	17.18	MT-TR	mitochondrially encoded tRNA arginine	chrM	Mt_tRNA
ENSG00000158669.10	-1.8382016	7.86831167	3.64E-10	3.39E-09	49.15	44.7	17.05	17.95	AGPAT6	1-acylglycerol-3-phosphate O-acyltransferase 6	chr8	protein_coding
ENSG00000129353.13	-1.2508952	7.19128405	1.48E-05	7.00E-05	38.25	42.97	23.64	21.75	SLC44A2	solute carrier family 44 (choline transporter), member 2	chr19	protein_coding

ENSG00000087077.10	-1.0654649	7.51295626	0.00021033	0.00079194	101.57	88.23	61.1	59.78	TRIP6	thyroid hormone receptor interactor 6	chr7	protein_coding
ENSG00000263082.1	-8.3437318	-0.6540328	1.75E-16	3.17E-15	0.44	0.44	0	0	CTD-2034I21	--	chr16	lincRNA
ENSG00000275783.1	5.46945885	-2.7387023	0.00158008	0.00495521	0	0	1.07	0.91	FP236383.5	--	chr21	miRNA
ENSG00000158887.14	-5.6784839	1.16222789	8.97E-30	4.10E-28	1.59	1.97	0.06	0.03	MPZ	myelin protein zero	chr1	protein_coding
ENSG00000260877.1	-3.4717321	-2.1147493	3.06E-05	0.00013563	0.65	0.84	0.09	0.07	RP11-211G2	--	chr11	lincRNA
ENSG00000264964.1	5.46459707	-2.7413886	0.00158008	0.00495521	0	0	0.34	0.41	RP11-888D1	--	chr18	lincRNA
ENSG00000138032.19	2.15520147	2.70309542	1.70E-11	1.86E-10	0.36	0.35	1.72	2.51	PPM1B	protein phosphatase, Mg2+/Mn2+ dependent, 1B	chr2	protein_coding
ENSG00000251365.1	-2.9555407	-2.0719458	0.00016274	0.00062431	0.36	0.57	0	0.14	RP11-332J15	--	chr5	lincRNA
ENSG00000164251.4	1.86451	0.78136657	6.28E-07	3.73E-06	0.13	0.26	0.97	0.93	F2RL1	coagulation factor II (thrombin) receptor-like 1	chr5	protein_coding
ENSG00000153936.15	1.47925637	2.59454339	2.32E-06	1.27E-05	0.42	0.28	0.93	1.69	HS2ST1	heparan sulfate 2-O-sulfotransferase 1	chr1	protein_coding
ENSG00000122786.18	1.72292956	4.43285677	7.74E-09	6.02E-08	1.32	1.57	4.79	7.98	CALD1	caldesmon 1	chr7	protein_coding
ENSG00000008277.13	2.57629552	0.96367071	2.46E-11	2.63E-10	0.04	0.04	0.22	0.4	ADAM22	ADAM metallopeptidase domain 22	chr7	protein_coding
ENSG00000132141.12	4.79227752	-0.6642817	7.46E-12	8.46E-11	0.03	0	0.41	0.77	CCT6B	chaperonin containing TCP1, subunit 6B (zeta 2)	chr17	protein_coding
ENSG00000221909.2	1.99183744	-0.6466058	6.23E-05	0.00026157	0.08	0.07	0.12	0.65	FAM200A	family with sequence similarity 200, member A	chr7	protein_coding
ENSG00000272217.1	6.03134248	-2.373212	8.36E-05	0.00034052	0	0	0.32	0.55	XXbac-BPG1	--	chr6	lincRNA
ENSG00000215018.8	3.00116668	-2.6722499	0.00943894	0.02469931	0.01	0	0.03	0.05	COL28A1	collagen, type XXVIII, alpha 1	chr7	protein_coding
ENSG00000265967.1	-5.3733592	-2.9472019	0.00290522	0.00856312	0.29	0.15	0	0	AC100830.4	--	chr15	antisense
ENSG00000148346.10	-3.0959371	2.42351572	1.11E-19	2.60E-18	5.83	9.14	1.2	1.12	LCN2	lipocalin 2	chr9	protein_coding
ENSG00000166833.18	1.39478975	5.72058074	1.69E-06	9.43E-06	1.9	2.1	7.8	6.14	NAV2	neuron navigator 2	chr11	protein_coding
ENSG00000104976.10	-1.0848849	4.68026531	0.00019866	0.0007508	15.74	18.51	11.38	10.09	SNAPC2	small nuclear RNA activating complex, polypeptide 2, 45kDa	chr19	protein_coding
ENSG00000115750.15	3.30345331	0.4960372	5.69E-14	8.14E-13	0.13	0.02	0.85	1.25	TAF1B	TATA box binding protein (TBP)-associated factor, RNA polymerase I, B, 63kDa	chr2	protein_coding
ENSG00000184441.4	4.90300768	3.22589176	8.40E-39	6.00E-37	0.15	0.07	4.65	4.19	AP001062.7	--	chr21	antisense
ENSG00000126709.13	1.69859605	5.51111022	7.80E-09	6.06E-08	20.01	20.12	90.88	82.42	IFI6	interferon, alpha-inducible protein 6	chr1	protein_coding
ENSG00000143375.13	3.25438898	3.75154499	1.76E-23	5.47E-22	0.44	0.37	5.72	4.49	CGN	cingulin	chr1	protein_coding
ENSG00000121864.8	2.40171484	1.17916427	8.01E-11	8.10E-10	0.03	0.15	0.42	0.82	ZNF639	zinc finger protein 639	chr3	protein_coding
ENSG00000027697.11	1.27321211	2.88729426	3.21E-05	0.00014208	1.45	1.79	3.5	6.99	IFNGR1	interferon gamma receptor 1	chr6	protein_coding
ENSG00000171634.15	2.8082715	4.03981237	5.67E-19	1.27E-17	0.31	0.28	2.32	3.16	BPTF	bromodomain PHD finger transcription factor	chr17	protein_coding
ENSG00000153310.17	1.02921895	3.48231201	0.00056764	0.00196697	1.17	1.52	2.66	4.69	FAM49B	family with sequence similarity 49, member B	chr8	protein_coding
ENSG00000081721.10	-1.1077759	3.83790167	0.00017905	0.00068195	12.99	11.85	7.21	8.17	DUSP12	dual specificity phosphatase 12	chr1	protein_coding
ENSG00000226380.6	4.03266946	0.01888969	4.62E-14	6.67E-13	0	0.03	0.35	0.23	AC058791.1	--	chr7	lincRNA
ENSG00000152133.13	2.76680712	1.60792993	1.72E-14	2.61E-13	0.14	0.17	0.91	1.93	GPATCH11	G patch domain containing 11	chr2	protein_coding
ENSG00000230479.1	-2.0855639	-1.896868	0.00303791	0.00892022	0.18	0.48	0.07	0.12	AP000695.6	--	chr21	antisense
ENSG00000112742.8	2.46396228	0.28520716	6.10E-09	4.83E-08	0.04	0.11	0.25	0.82	TTK	TTK protein kinase	chr6	protein_coding
ENSG00000187908.14	-10.655021	1.60321249	1.14E-41	9.44E-40	0.69	0.53	0	0	DMBT1	deleted in malignant brain tumors 1	chr10	protein_coding
ENSG00000137449.14	-1.7758456	0.15848387	7.89E-06	3.91E-05	0.24	0.15	0.07	0.09	CPEB2	cytoplasmic polyadenylation element binding protein 2	chr4	protein_coding
ENSG00000070961.13	1.69417465	3.76315879	2.03E-08	1.49E-07	0.58	0.83	2.35	3.75	ATP2B1	ATPase, Ca++ transporting, plasma membrane 1	chr12	protein_coding
ENSG00000132952.10	2.21938399	0.21179521	1.95E-07	1.26E-06	0.04	0.08	0.34	0.42	USPL1	ubiquitin specific peptidase like 1	chr13	protein_coding
ENSG00000112319.16	-9.7893403	0.74152219	6.69E-31	3.21E-29	0.37	0.52	0	0	EYA4	EYA transcriptional coactivator and phosphatase 4	chr6	protein_coding
ENSG00000133328.3	-5.36045	-1.3272891	5.48E-10	4.95E-09	1.01	0.53	0.04	0	HRASLS2	HRAS-like suppressor 2	chr11	protein_coding
ENSG00000119900.7	1.2943869	1.02509637	0.00025245	0.0009387	0.07	0.15	0.23	0.48	OGFRL1	opioid growth factor receptor-like 1	chr6	protein_coding
ENSG00000198730.6	1.06932756	3.33630626	0.00036834	0.00132243	0.94	1.34	2.61	3.8	CTR9	CTR9, Paf1/RNA polymerase II complex component	chr11	protein_coding
ENSG00000117411.15	-1.9680375	5.88594479	2.93E-11	3.11E-10	27.28	24.54	8.92	8.73	B4GALT2	UDP-Gal:betaGlcNAc beta 1,4- galactosyltransferase, polypeptide 2	chr1	protein_coding
ENSG00000009413.14	3.0209163	1.04439251	2.04E-14	3.07E-13	0.02	0.04	0.26	0.4	REV3L	REV3-like, polymerase (DNA directed), zeta, catalytic subunit	chr6	protein_coding
ENSG00000137563.10	1.00381653	4.31958831	0.00061184	0.00210673	5.59	7.76	12.56	23.32	GGH	gamma-glutamyl hydrolase (conjugase, folylpolygammaglutamyl hydrolase)	chr8	protein_coding
ENSG00000260257.2	1.81726753	0.31664394	5.40E-06	2.75E-05	0.19	0.2	0.96	0.87	RP5-1085F17	--	chr20	lincRNA
ENSG00000221994.9	1.65494446	0.10666555	6.51E-05	0.00027249	0.16	0.11	0.31	0.81	ZNF630	zinc finger protein 630	chrX	protein_coding
ENSG00000185361.7	-1.5535079	4.91787181	1.27E-07	8.44E-07	9.46	8.98	4.26	4.11	TNFAIP8L1	tumor necrosis factor, alpha-induced protein 8-like 1	chr19	protein_coding
ENSG00000005513.9	7.57392263	-1.1764002	1.38E-11	1.53E-10	0	0	0.3	0.21	SOX8	SRY (sex determining region Y)-box 8	chr16	protein_coding
ENSG00000247363.2	-5.2081991	-3.0359038	0.00538165	0.01488851	0.08	0.05	0	0	RP11-637A1	--	chr12	lincRNA
ENSG00000134326.10	8.49471319	1.96021388	5.66E-43	5.10E-41	0	0.01	1.44	2.71	CMPK2	cytidine monophosphate (UMP-CMP) kinase 2, mitochondrial	chr2	protein_coding
ENSG00000118496.4	3.10639733	1.27814881	1.27E-15	2.15E-14	0.02	0.06	0.3	0.7	FBXO30	F-box protein 30	chr6	protein_coding
ENSG00000032219.17	4.37068348	1.87865527	6.45E-27	2.54E-25	0.02	0.06	0.82	1.51	ARID4A	AT rich interactive domain 4A (RBP1-like)	chr14	protein_coding
ENSG00000204387.11	-2.1223694	5.16736124	1.40E-12	1.73E-11	29.3	23.18	7.45	8.65	C6orf48	chromosome 6 open reading frame 48	chr6	protein_coding
ENSG00000197978.9	4.12016072	-1.2269365	9.32E-08	6.27E-07	0.03	0	0.22	0.59	GOLGA6L9	golgin A6 family-like 9	chr15	protein_coding
ENSG00000124067.15	1.77194601	7.55853541	1.43E-09	1.23E-08	13.92	12.74	61.07	60.27	SLC12A4	solute carrier family 12 (potassium/chloride transporter), member 4	chr16	protein_coding
ENSG00000167981.5	1.78921672	-0.1145621	3.43E-05	0.00015114	0.06	0.05	0.12	0.39	ZNF597	zinc finger protein 597	chr16	protein_coding
ENSG00000108448.19	-3.3494462	6.45635991	7.07E-27	2.77E-25	35.63	33.19	4.5	4.5	TRIM16L	tripartite motif containing 16-like	chr17	protein_coding
ENSG00000130222.9	3.12644029	-0.5591065	5.74E-09	4.56E-08	0.12	0.02	1.09	0.66	GADD45G	growth arrest and DNA-damage-inducible, gamma	chr9	protein_coding
ENSG00000165752.15	-2.3541673	5.88227906	4.28E-15	6.87E-14	29.04	24.42	6.97	6.97	STK32C	serine/threonine kinase 32C	chr10	protein_coding
ENSG00000226816.2	6.19483651	-2.260378	2.70E-05	0.00012094	0	0	0.16	0.13	AC005082.12	--	chr7	lincRNA
ENSG00000223496.2	1.25301579	5.78767956	1.58E-05	7.41E-05	4.83	5.42	14.91	17.69	EXOSC6	exosome component 6	chr16	protein_coding
ENSG00000142669.12	-1.3009037	6.93949614	6.91E-06	3.46E-05	79.38	78.89	47.03	38.33	SH3BGRL3	SH3 domain binding glutamate-rich protein like 3	chr1	protein_coding
ENSG00000176024.15	3.63080596	0.04961374	5.91E-13	7.60E-12	0.01	0.06	0.36	0.69	ZNF613	zinc finger protein 613	chr19	protein_coding
ENSG00000237781.3	-3.4479797	-2.711395	0.00552196	0.01522223	0.44	0	0.05	0	RP11-54A4.2	--	chr1	antisense
ENSG00000172915.17	2.41762465	0.23487715	1.30E-08	9.76E-08	0.03	0.02	0.17	0.17	NBEA	neurobeachin	chr13	protein_coding
ENSG00000277369.1	1.99405029	0.02643217	2.29E-06	1.25E-05	0.5	0.25	2.97	1.04	RP11-486I11	--	chr16	lincRNA
ENSG00000160188.8	1.99965494	-0.3798687	2.36E-05	0.00010694	0.07	0.24	1.12	0.55	RSPH1	radial spoke head 1 homolog (Chlamydomonas)	chr21	protein_coding
ENSG00000259736.1	2.88953926	-2.734604	0.01615662	0.03958166	0	0.03	0.2	0.14	CRTC3-AS1	CRTC3 antisense RNA 1	chr15	antisense
ENSG00000074527.10	2.25062746	4.53862552	1.38E-13	1.89E-12	1.48	2.07	8.51	14.12	NTN4	netrin 4	chr12	protein_coding
ENSG00000138399.16	3.11656881	1.46594137	1.74E-16	3.16E-15	0.13	0.17	1.38	2.08	FASTKD1	FAST kinase domains 1	chr2	protein_coding
ENSG00000204219.8	3.72614052	5.14461679	1.27E-30	6.03E-29	1.55	1.68	27.46	29.47	TCEA3	transcription elongation factor A (SII), 3	chr1	protein_coding
ENSG00000144061.11	1.21425034	0.06186093	0.00306544	0.00899555	0.07	0.15	0.29	0.39	NPHP1	nephronophthisis 1 (juvenile)	chr2	protein_coding
ENSG00000236008.1	3.26089881	-1.1284655	5.22E-07	3.15E-06	0.01	0.01	0.09	0.08	AC011747.4	--	chr2	lincRNA
ENSG00000106714.16	-1.2737881	2.85839134	2.97E-05	0.00013228	1.22	1.12	0.53	0.76	CNTNAP3	contactin associated protein-like 3	chr9	protein_coding
ENSG00000158813.16	2.78541349	0.78972652	4.15E-12	4.86E-11	0.02	0.09	0.55	0.45	EDA	ectodysplasin A	chrX	protein_coding
ENSG00000069812.10	-3.3055798	2.59106911	4.95E-22	1.39E-20	1.92	1.35	0.22	0.22	HES2	hes family bHLH transcription factor 2	chr1	protein_coding
ENSG00000113504.18	-3.3768577	7.59850772	2.41E-27	9.77E-26	53.76	54.69	7.22	6.67	SLC12A7	solute carrier family 12 (potassium/chloride transporter), member 7	chr5	protein_coding
ENSG00000260328.1	-1.2950316	1.78595445	6.56E-05	0.00027409	1.37	1.18	0.75	0.64	RP11-416I2.1	--	chr17	lincRNA
ENSG00000160050.13	-1.3210356	5.07482793	6.05E-06	3.06E-05	24.56	22.29	13.07	11.9	CCDC28B	coiled-coil domain containing 28B	chr1	protein_coding
ENSG00000165115.13	2.19690331	-0.8273635	4.16E-05	0.00018125	0.01	0.05	0.14	0.21	KIF27	kinesin family member 27	chr9	protein_coding
ENSG00000134371.9	1.39189867	2.36819957	9.82E-06	4.77E-05	0.38	0.54	1.18	2.02	CDC73	cell division cycle 73	chr1	protein_coding
ENSG00000131669.8	1.08519404	5.80080933	0.00017488	0.00066701	22.47	22.61	66.27	61.01	NINJ1	ninjurin 1	chr9	protein_coding
ENSG00000165283.14	-2.0776441	7.74985983	2.10E-12	2.55E-11	208.6	184.99	60.19	64.21	STOML2	stomatin (EPB72)-like 2	chr9	protein_coding
ENSG00000173597.7	5.6875973	-2.6030615	0.00047761	0.00167191	0	0	0.03	0.03	SULT1B1	sulfotransferase family, cytosolic, 1B, member 1	chr4	protein_coding
ENSG00000198898.11	2.14980504	1.92949048	1.62E-10	1.58E-09	0.21	0.18	0.76	1.58	CAPZA2	capping protein (actin filament) muscle Z-line, alpha 2	chr7	protein_coding
ENSG00000163888.3	1.70618181	3.68245225	1.77E-08	1.31E-07	3.22	3.89	17.31	13.5	CAMK2N2	calcium/calmodulin-dependent protein kinase II inhibitor 2	chr3	protein_coding
ENSG00000140931.18	-2.2986769	2.55573044	1.45E-12	1.79E-11	2.99	2.16	0.94	0.45	CMTM3	CKLF-like MARVEL transmembrane domain containing 3	chr16	protein_coding
ENSG00000116035.2	3.26640118	1.67607685	2.05E-18	4.38E-17	0.4	0.42	6.2	4.3	VAX2	ventral anterior homeobox 2	chr2	protein_coding
ENSG00000123094.14	2.83567177	1.72261633	2.51E-15	4.14E-14	0.11	0.07	0.66	1.05	RASSF8	Ras association (RalGDS/AF-6) domain family (N-terminal) member 8	chr12	protein_coding
ENSG00000096401.7	1.04922445	3.2071637	0.00048043	0.00168155	0.69	0.82	1.32	2.85	CDC5L	cell division cycle 5-like	chr6	protein_coding
ENSG00000186204.13	3.09443163	1.15352761	2.58E-15	4.24E-14	0.19	0.15	2.31	1.6	CYP4F12	cytochrome P450, family 4, subfamily F, polypeptide 12	chr19	protein_coding
ENSG00000268941.2	-5.0206302	-3.1306986	0.0100455	0.02582742	0.14	0.06	0	0	RP5-907D15	--	chr20	lincRNA
ENSG00000245522.2	-1.3058936	-0.6989693	0.00489333	0.01377764	0.37	0.2	0.15	0.15	RP11-540A2	--	chr11	lincRNA
ENSG00000221995.5	1.30982274	-0.3973696	0.00291737	0.00859154	0.06	0.06	0.22	0.17	TIAF1	TGFB1-induced anti-apoptotic factor 1	chr17	protein_coding
ENSG00000069702.9	1.37991248	1.3494001	5.15E-05	0.00021919	0.13	0.18	0.37	0.72	TGFBR3	transforming growth factor, beta receptor III	chr1	protein_coding
ENSG00000272636.3	-3.6702574	-1.9688304	4.03E-05	0.00017569	0.02	0.09	0.01	0	DOC2B	double C2-like domains, beta	chr17	protein_coding
ENSG00000197622.11	-1.0660565	6.44970496	0.00021632	0.00081277	25.94	26.77	17.27	16.24	CDC42SE1	CDC42 small effector 1	chr1	protein_coding
ENSG00000147509.12	-1.5225967	0.22601471	8.22E-05	0.00033796	0.57	0.61	0.25	0.29	RGS20	regulator of G-protein signaling 20	chr8	protein_coding
ENSG00000178105.8	2.41737942	4.66987597	2.60E-15	4.27E-14	0.76	0.96	4.85	7.51	DDX10	DEAD (Asp-Glu-Ala-Asp) box polypeptide 10	chr11	protein_coding
ENSG00000185900.8	1.40724509	-0.0284323	0.00069215	0.00235586	0.27	0.15	0.89	0.62	POMK	protein-O-mannose kinase	chr8	protein_coding
ENSG00000279077.1	4.87917439	-3.0678699	0.01889873	0.04519557	0	0	0	0.1	RP11-800A1	--	chr18	TEC
ENSG00000116544.10	2.42292626	0.16773439	2.16E-08	1.57E-07	0.1	0.03	0.53	0.44	DLGAP3	discs, large (Drosophila) homolog-associated protein 3	chr1	protein_coding
ENSG00000126016.12	4.88814447	-0.1019231	6.03E-16	1.04E-14	0.01	0	0.22	0.2	AMOT	angiomotin	chrX	protein_coding
ENSG00000004660.13	-4.3316069	5.52859669	6.31E-39	4.57E-37	16.83	14.96	0.95	1.16	CAMKK1	calcium/calmodulin-dependent protein kinase kinase 1, alpha	chr17	protein_coding
ENSG00000187867.7	-4.2906714	1.96308404	2.14E-28	9.06E-27	2.48	2.84	0.11	0.24	PALM3	paralemmin 3	chr19	protein_coding
ENSG00000186868.14	3.47327204	3.35581536	4.93E-25	1.72E-23	0.1	0.12	1.58	1.67	MAPT	microtubule-associated protein tau	chr17	protein_coding
ENSG00000115159.14	1.60497564	1.89538527	1.06E-06	6.04E-06	0.17	0.29	0.76	1.11	GPD2	glycerol-3-phosphate dehydrogenase 2 (mitochondrial)	chr2	protein_coding
ENSG00000075292.17	2.74074839	3.73037658	5.80E-18	1.19E-16	0.34	0.38	2.21	4.18	ZNF638	zinc finger protein 638	chr2	protein_coding
ENSG00000167543.14	1.49065518	5.40119675	3.52E-07	2.18E-06	14.09	9.28	46.21	41.36	TP53I13	tumor protein p53 inducible protein 13	chr17	protein_coding
ENSG00000070950.8	1.22280163	1.61581686	0.00021846	0.00082032	0.25	0.24	0.57	0.97	RAD18	RAD18 E3 ubiquitin protein ligase	chr3	protein_coding
ENSG00000065613.12	3.65265616	2.21365398	2.79E-23	8.52E-22	0.09	0.05	0.75	1.59	SLK	STE20-like kinase	chr10	protein_coding
ENSG00000139726.9	1.38178734	4.54609727	2.79E-06	1.50E-05	2.94	4.63	9.19	17.21	DENR	density-regulated protein	chr12	protein_coding
ENSG00000261949.4	-5.6275551	-0.3195355	6.04E-18	1.23E-16	0.47	0.86	0.03	0	GFY	golgi-associated, olfactory signaling regulator	chr19	protein_coding
ENSG00000155974.10	9.45185896	0.52018193	5.75E-26	2.10E-24	0	0	0.47	0.62	GRIP1	glutamate receptor interacting protein 1	chr12	protein_coding
ENSG00000105855.8	3.12249614	0.83052419	5.38E-14	7.71E-13	0.01	0.05	0.26	0.45	ITGB8	integrin, beta 8	chr7	protein_coding
ENSG00000166793.9	-2.0940056	-2.5789043	0.01923175	0.0458365	0.07	0	0.01	0.01	YPEL4	yippee-like 4 (Drosophila)	chr11	protein_coding
ENSG00000146112.10	-1.6151449	7.92396657	2.93E-08	2.10E-07	60.85	68.15	29.69	26.29	PPP1R18	protein phosphatase 1, regulatory subunit 18	chr6	protein_coding
ENSG00000166387.10	1.32006627	1.95749977	4.25E-05	0.00018459	0.53	0.32	1.24	1.59	PPFIBP2	PTPRF interacting protein, binding protein 2 (liprin beta 2)	chr11	protein_coding
ENSG00000138231.11	1.91838117	3.49585214	4.26E-10	3.93E-09	1.14	1.7	5.23	9.16	DBR1	debranching RNA lariats 1	chr3	protein_coding
ENSG00000230555.2	1.60923892	0.02418792	0.00012281	0.000485	0.35	0.63	2.4	1.61	RP11-517P1	--	chr10	lincRNA
ENSG00000251417.2	2.17078431	-2.2139473	0.00854801	0.02266235	0.04	0	0.08	0.18	RP11-1348G	--	chr16	lincRNA
ENSG00000136783.9	1.02698297	1.71182517	0.00156895	0.00495521	1.31	0.76	2.35	3.32	NIPSNAP3A	nipsnap homolog 3A (C. elegans)	chr9	protein_coding
ENSG00000170919.14	-1.7287227	0.38701109	7.64E-06	3.81E-05	0.25	0.39	0.14	0.12	TPT1-AS1	TPT1 antisense RNA 1	chr13	antisense
ENSG00000139977.12	1.5183791	1.73307902	4.86E-06	2.51E-05	0.15	0.2	0.47	0.88	NAA30	N(alpha)-acetyltransferase 30, NatC catalytic subunit	chr14	protein_coding
ENSG00000225978.3	8.9524408	0.05344711	6.55E-21	1.68E-19	0	0	1.07	1.07	HAR1A	highly accelerated region 1A (non-protein coding)	chr20	lincRNA
ENSG00000116260.15	-1.0259409	7.64875795	0.00035388	0.00127413	24.25	22.54	15.59	15.02	QSOX1	quiescin Q6 sulfhydryl oxidase 1	chr1	protein_coding
ENSG00000183876.8	1.78322471	0.20507934	1.21E-05	5.78E-05	0.04	0.2	0.58	0.54	ARSI	arylsulfatase family, member I	chr5	protein_coding
ENSG00000175414.6	-1.1043156	3.11256342	0.00025005	0.0009305	0.87	0.89	0.58	0.5	ARL10	ADP-ribosylation factor-like 10	chr5	protein_coding
ENSG00000166183.14	-5.0234041	-3.1294261	0.0100455	0.02582742	0.01	0.03	0	0	ASPG	asparaginase	chr14	protein_coding
ENSG00000117318.8	1.85553143	8.07378284	2.54E-10	2.40E-09	76.62	74.3	387.1	339.38	ID3	inhibitor of DNA binding 3, dominant negative helix-loop-helix protein	chr1	protein_coding
ENSG00000205420.9	-1.4636442	1.80987828	6.11E-06	3.09E-05	1.99	1.58	0.83	0.9	KRT6A	keratin 6A, type II	chr12	protein_coding
ENSG00000186994.10	4.37738193	1.0019593	7.83E-22	2.17E-20	0.03	0.05	1.07	1.1	KANK3	KN motif and ankyrin repeat domains 3	chr19	protein_coding
ENSG00000164011.16	-1.5427529	4.92842224	1.55E-07	1.01E-06	17.98	18.73	9.2	7.55	ZNF691	zinc finger protein 691	chr1	protein_coding
ENSG00000279312.1	5.76922753	-2.5489645	0.00047761	0.00167191	0	0	0.06	0.14	RP3-331H24	--	chr6	TEC
ENSG00000114405.9	5.93078347	0.85665706	2.69E-26	1.01E-24	0.02	0	0.75	1.26	C3orf14	chromosome 3 open reading frame 14	chr3	protein_coding
ENSG00000141756.17	-8.1205269	7.87178134	3.04E-93	4.86E-90	146.93	136.94	0.73	0.62	FKBP10	FK506 binding protein 10, 65 kDa	chr17	protein_coding
ENSG00000268603.1	1.90947462	-1.6453756	0.00486779	0.01371541	0.13	0.13	0.6	0.73	RP11-316O1	--	chr2	antisense
ENSG00000160352.14	2.49502872	0.70105137	2.93E-10	2.75E-09	0.07	0.03	0.38	0.4	ZNF714	zinc finger protein 714	chr19	protein_coding
ENSG00000170325.13	1.65264501	0.64752637	9.53E-06	4.65E-05	0.12	0.05	0.33	0.41	PRDM10	PR domain containing 10	chr11	protein_coding
ENSG00000173546.7	-1.2850295	6.41904148	9.09E-06	4.45E-05	12.28	11.41	6.25	6.71	CSPG4	chondroitin sulfate proteoglycan 4	chr15	protein_coding
ENSG00000170100.12	1.17453257	2.72512327	0.00013651	0.00053444	0.24	0.34	0.95	0.8	ZNF778	zinc finger protein 778	chr16	protein_coding
ENSG00000203880.10	1.63145184	2.31571556	3.28E-07	2.04E-06	0.26	0.69	1.25	2.69	PCMTD2	protein-L-isoaspartate (D-aspartate) O-methyltransferase domain containing 2	chr20	protein_coding
ENSG00000207445.1	-4.1544586	-2.26591	8.40E-05	0.0003415	1.1	2.33	0.22	0	SNORD15B	small nucleolar RNA, C/D box 15B	chr11	snoRNA
ENSG00000224081.6	-2.0713112	2.03833898	2.99E-10	2.81E-09	3.96	4.09	1.16	1.39	LINC01057	long intergenic non-protein coding RNA 1057	chr1	lincRNA
ENSG00000153531.11	-2.6388952	2.32622967	2.08E-15	3.45E-14	3.22	3.2	0.63	0.74	ADPRHL1	ADP-ribosylhydrolase like 1	chr13	protein_coding
ENSG00000196152.9	1.05178702	3.74411842	0.00039382	0.00140424	2.81	3.29	8.5	8.33	ZNF79	zinc finger protein 79	chr9	protein_coding
ENSG00000277147.3	-1.2328978	4.72749613	2.52E-05	0.00011384	5.3	5.18	3.9	1.99	LINC00869	long intergenic non-protein coding RNA 869	chr1	lincRNA
ENSG00000132321.15	-8.2750522	-0.7159354	6.89E-16	1.18E-14	0.41	0.14	0	0	IQCA1	IQ motif containing with AAA domain 1	chr2	protein_coding
ENSG00000100968.12	-13.39446	4.34491169	2.31E-81	1.63E-78	4.8	4.16	0	0	NFATC4	nuclear factor of activated T-cells, cytoplasmic, calcineurin-dependent 4	chr14	protein_coding
ENSG00000135317.11	2.30880492	2.24139338	3.84E-12	4.52E-11	0.32	0.35	1.44	3.01	SNX14	sorting nexin 14	chr6	protein_coding
ENSG00000128872.8	1.887362	0.0729624	8.34E-06	4.11E-05	0.03	0.05	0.17	0.19	TMOD2	tropomodulin 2 (neuronal)	chr15	protein_coding
ENSG00000154743.16	-1.1623211	3.96992473	8.25E-05	0.00033913	4	4.9	2.39	2.91	TSEN2	TSEN2 tRNA splicing endonuclease subunit	chr3	protein_coding
ENSG00000198331.9	3.05436557	1.41096819	7.16E-16	1.23E-14	0.11	0.19	1.69	1.59	HYLS1	hydrolethalus syndrome 1	chr11	protein_coding
ENSG00000107290.12	2.20495499	3.21613964	2.22E-12	2.70E-11	0.28	0.19	1.4	1.54	SETX	senataxin	chr9	protein_coding
ENSG00000196110.6	1.37117778	-0.7413751	0.00624182	0.01703231	0.01	0.14	0.29	0.2	ZNF699	zinc finger protein 699	chr19	protein_coding
ENSG00000017260.18	1.22544404	5.0371127	2.65E-05	0.00011911	2.34	2.84	6.92	9.24	ATP2C1	ATPase, Ca++ transporting, type 2C, member 1	chr3	protein_coding
ENSG00000119681.10	-2.6090689	5.73025019	7.67E-18	1.56E-16	8.58	8.66	1.89	1.87	LTBP2	latent transforming growth factor beta binding protein 2	chr14	protein_coding
ENSG00000213463.4	1.31070174	2.13428804	3.92E-05	0.00017132	0.26	0.32	0.78	1.11	SYNJ2BP	synaptojanin 2 binding protein	chr14	protein_coding
ENSG00000168090.8	-1.4096153	8.05059677	1.13E-06	6.42E-06	223.69	211.79	113.61	104.59	COPS6	COP9 signalosome subunit 6	chr7	protein_coding
ENSG00000047597.5	1.47160564	-0.4712165	0.00138696	0.0044408	0.05	0.06	0.28	0.12	XK	X-linked Kx blood group	chrX	protein_coding
ENSG00000225302.2	1.61833118	0.52560734	2.03E-05	9.31E-05	0.1	0.11	0.47	0.41	RP11-539I5.1	--	chr10	antisense
ENSG00000230454.1	-1.496825	0.3874315	8.90E-05	0.00036046	0.71	0.44	0.24	0.3	U73166.2	--	chr3	lincRNA
ENSG00000154222.13	-1.1434568	6.31913421	7.52E-05	0.00031067	17.06	14.45	10.33	8.65	CC2D1B	coiled-coil and C2 domain containing 1B	chr1	protein_coding
ENSG00000107338.9	-2.8533125	3.99940792	1.35E-19	3.14E-18	9.15	7.18	1.55	1.46	SHB	Src homology 2 domain containing adaptor protein B	chr9	protein_coding
ENSG00000120868.12	3.2259208	1.4236709	5.54E-17	1.05E-15	0.05	0.06	0.58	0.77	APAF1	apoptotic peptidase activating factor 1	chr12	protein_coding
ENSG00000181982.16	3.67525828	1.87387477	4.13E-22	1.17E-20	0.05	0.15	1.69	1.73	CCDC149	coiled-coil domain containing 149	chr4	protein_coding
ENSG00000163660.10	1.34279945	3.38753002	8.82E-06	4.33E-05	1.04	2.04	3.79	6.64	CCNL1	cyclin L1	chr3	protein_coding
ENSG00000181544.12	2.25827311	-1.0599935	6.05E-05	0.0002546	0.05	0.02	0.12	0.35	FANCB	Fanconi anemia, complementation group B	chrX	protein_coding
ENSG00000279069.1	1.42241191	-0.6415156	0.0026473	0.00794553	0.28	0.04	0.32	0.85	RP11-159D1	--	chr17	TEC
ENSG00000281332.1	1.34379704	-1.4226305	0.02009733	0.04771922	0.09	0.01	0.18	0.17	LINC00997	long intergenic non-protein coding RNA 997	chr7	lincRNA
ENSG00000171608.14	-1.7263494	2.65303259	3.52E-08	2.50E-07	1.72	1.02	0.59	0.52	PIK3CD	phosphatidylinositol-4,5-bisphosphate 3-kinase, catalytic subunit delta	chr1	protein_coding
ENSG00000169519.18	1.85908535	0.59690314	1.31E-06	7.38E-06	0.12	0.1	0.39	0.67	METTL15	methyltransferase like 15	chr11	protein_coding
ENSG00000237950.1	-1.4436889	-0.7929301	0.00215901	0.00657968	1.34	0.92	0.46	0.64	RP11-7O11.3	--	chr1	antisense
ENSG00000197889.8	2.38860766	-1.5228173	0.00036944	0.00132576	0.09	0.14	0.72	0.87	MEIG1	meiosis/spermiogenesis associated 1	chr10	protein_coding
ENSG00000162994.14	2.25464565	-0.4564547	3.03E-06	1.62E-05	0.1	0.05	0.4	0.53	CLHC1	clathrin heavy chain linker domain containing 1	chr2	protein_coding
ENSG00000114904.11	2.00292987	2.49623983	4.64E-10	4.24E-09	0.31	0.27	1.02	2.11	NEK4	NIMA-related kinase 4	chr3	protein_coding
ENSG00000280734.1	2.88355909	0.74240986	1.15E-12	1.43E-11	0.17	0.04	1.16	0.98	LINC01232	long intergenic non-protein coding RNA 1232	chr13	processed_transcript
ENSG00000162755.12	-1.845955	1.34730999	8.28E-08	5.61E-07	2.35	2.44	0.94	0.84	KLHDC9	kelch domain containing 9	chr1	protein_coding
ENSG00000029534.18	-1.4088643	1.269829	2.93E-05	0.00013049	0.24	0.36	0.14	0.16	ANK1	ankyrin 1, erythrocytic	chr8	protein_coding
ENSG00000135469.12	1.78589283	3.55918583	4.46E-09	3.59E-08	2.68	1.74	8.87	11.58	COQ10A	coenzyme Q10 homolog A (S. cerevisiae)	chr12	protein_coding
ENSG00000274810.2	4.0039394	0.52769422	4.26E-17	8.13E-16	0.02	0.01	0.24	0.46	NPHP3-ACA	NPHP3-ACAD11 readthrough (NMD candidate)	chr3	protein_coding

ENSG00000277157.1	-5.3778332	-2.9451057	0.00290522	0.00856312	0.09	0.73	0	0	HIST1H4D	histone cluster 1, H4d	chr6	protein_coding
ENSG00000108797.10	-1.608312	5.80448682	3.96E-08	2.78E-07	12.77	13.06	6.09	5.17	CNTNAP1	contactin associated protein 1	chr17	protein_coding
ENSG00000122484.8	2.17824281	1.57760707	4.55E-10	4.16E-09	0.04	0.06	0.24	0.37	RPAP2	RNA polymerase II associated protein 2	chr1	protein_coding
ENSG00000125458.5	-1.3136406	7.31169298	5.55E-06	2.82E-05	143.45	136.08	78.49	71.17	NT5C	5', 3'-nucleotidase, cytosolic	chr17	protein_coding
ENSG00000187240.12	4.13717824	1.50197319	1.52E-23	4.75E-22	0.03	0.01	0.32	0.49	DYNC2H1	dynein, cytoplasmic 2, heavy chain 1	chr11	protein_coding
ENSG00000137807.12	2.12671358	4.7078701	1.86E-12	2.27E-11	1.56	2.48	8.18	15.42	KIF23	kinesin family member 23	chr15	protein_coding
ENSG00000186451.1	-2.8657968	-2.5089661	0.00190233	0.00584723	0.04	0.11	0	0.02	SPATA12	spermatogenesis associated 12	chr3	protein_coding
ENSG00000113734.16	-1.3081189	3.33345488	1.35E-05	6.41E-05	8.41	8.32	3.95	5.07	BNIP1	BCL2/adenovirus E1B 19kDa interacting protein 1	chr5	protein_coding
ENSG00000115648.12	-4.9891792	2.4518425	5.95E-37	3.80E-35	2.21	1.94	0.02	0.15	MLPH	melanophilin	chr2	protein_coding
ENSG00000198723.9	-1.609099	-1.8631355	0.01883218	0.04519557	0.24	0.08	0.06	0.08	C19orf45	chromosome 19 open reading frame 45	chr19	protein_coding
ENSG00000106268.14	-1.4636524	5.67271034	5.29E-07	3.18E-06	73.4	57.17	32.24	30.87	NUDT1	nudix (nucleoside diphosphate linked moiety X)-type motif 1	chr7	protein_coding
ENSG00000204435.12	-1.1324914	7.4592471	8.37E-05	0.00034109	133.92	145.32	90.89	78.39	CSNK2B	casein kinase 2, beta polypeptide	chr6	protein_coding
ENSG00000144824.18	1.98784562	3.17837948	1.63E-10	1.58E-09	0.28	0.24	1.14	1.64	PHLDB2	pleckstrin homology-like domain, family B, member 2	chr3	protein_coding
ENSG00000273449.1	4.72703895	-3.141721	0.01889873	0.04519557	0	0	0.21	0.03	RP11-218F1(	--	chr4	lincRNA
ENSG00000101940.16	-1.831104	6.35442135	4.76E-10	4.34E-09	19.99	15.55	6.46	6.87	WDR13	WD repeat domain 13	chrX	protein_coding
ENSG00000269514.1	3.27555441	-1.8754785	0.00025363	0.00094222	0	0.02	0.06	0.15	RP11-370I10	--	chr12	lincRNA
ENSG00000267165.1	5.33435105	-2.8185235	0.00290522	0.00856312	0	0	0.17	0.39	RP11-78A19.	--	chr18	antisense
ENSG00000134108.11	1.65160345	2.77350211	1.16E-07	7.73E-07	0.8	0.95	2.87	4.5	ARL8B	ADP-ribosylation factor-like 8B	chr3	protein_coding
ENSG00000023902.12	-1.3673429	3.2574077	5.87E-06	2.98E-05	4.54	3.83	2.24	2.08	PLEKHO1	pleckstrin homology domain containing, family O member 1	chr1	protein_coding
ENSG00000137841.10	-2.6389467	2.58037926	9.45E-16	1.61E-14	1.34	1.67	0.29	0.35	PLCB2	phospholipase C, beta 2	chr15	protein_coding
ENSG00000133488.13	2.49951172	3.81785758	1.25E-15	2.11E-14	1.29	1.43	10.61	9.89	SEC14L4	SEC14-like 4 (S. cerevisiae)	chr22	protein_coding
ENSG00000182165.16	1.22186216	2.01977412	0.00013604	0.00053277	2.22	2.29	7.42	6.59	TP53TG1	TP53 target 1 (non-protein coding)	chr7	lincRNA
ENSG00000163681.13	1.95033844	3.03828734	4.44E-10	4.08E-09	0.17	0.32	0.92	1.63	SLMAP	sarcolemma associated protein	chr3	protein_coding
ENSG00000189120.4	1.46389542	-0.5398478	0.00170514	0.00531058	0.04	0.09	0.3	0.18	SP6	Sp6 transcription factor	chr17	protein_coding
ENSG00000187017.13	-2.3425678	2.06295637	2.33E-12	2.82E-11	1.44	1.68	0.43	0.38	ESPN	espin	chr1	protein_coding
ENSG00000196369.9	1.46941901	0.8943993	3.91E-05	0.00017073	0.07	0.14	0.37	0.42	SRGAP2B	SLIT-ROBO Rho GTPase activating protein 2B	chr1	protein_coding
ENSG00000142065.12	2.59804034	0.63830688	1.46E-10	1.43E-09	0.02	0.07	0.24	0.45	ZFP14	ZFP14 zinc finger protein	chr19	protein_coding
ENSG00000279742.1	2.08648906	0.52735659	1.64E-07	1.07E-06	0.11	0.24	0.85	1.13	RP11-700A2·	--	chr11	TEC
ENSG00000172031.6	5.12073744	-0.3803433	2.47E-14	3.69E-13	0.04	0	0.85	1.12	EPHX4	epoxide hydrolase 4	chr1	protein_coding
ENSG00000272057.1	-1.2923234	-1.0313746	0.00917505	0.0241697	1.05	1.63	0.34	1.07	CTB-40H15.4	--	chr5	lincRNA
ENSG00000237862.1	7.15635911	-1.523743	2.80E-09	2.31E-08	0	0	0.34	0.25	RP1-63G5.7	--	chr22	sense_intronic
ENSG00000008988.8	-1.1591347	9.25008936	5.56E-05	0.0002352	257.77	244.51	143.12	156.96	RPS20	ribosomal protein S20	chr8	protein_coding
ENSG00000169230.8	-1.4786909	8.9525663	3.38E-07	2.10E-06	460.85	475.68	228.44	218.91	PRELID1	PRELI domain containing 1	chr5	protein_coding
ENSG00000270372.1	-5.3787292	-2.9446861	0.00290522	0.00856312	0	2.32	0	0	RP11-109M1	--	chr9	lincRNA
ENSG00000265800.1	1.80772034	-1.9166807	0.01381259	0.03443739	0.27	0.07	1.44	0.27	RP11-649A1:	--	chr17	sense_intronic
ENSG00000178814.14	-7.1182992	4.3414133	4.34E-65	1.35E-62	8.83	7.34	0.08	0.07	OPLAH	5-oxoprolinase (ATP-hydrolysing)	chr8	protein_coding
ENSG00000105409.14	5.42820242	3.14196567	1.27E-42	1.11E-40	0.07	0.1	5.58	4.12	ATP1A3	ATPase, Na+/K+ transporting, alpha 3 polypeptide	chr19	protein_coding
ENSG00000156671.11	2.61687439	2.02711534	3.02E-14	4.45E-13	0.09	0.16	0.88	1.18	SAMD8	sterile alpha motif domain containing 8	chr10	protein_coding
ENSG00000227372.9	-6.7332566	4.50952147	1.63E-63	4.88E-61	4.86	5.3	0.06	0.07	TP73-AS1	TP73 antisense RNA 1	chr1	antisense
ENSG00000231566.1	-1.9250435	-1.5308723	0.00185375	0.00571918	1.14	0.91	0.34	0.36	RP5-1158E1:	--	chrX	lincRNA
ENSG00000153006.13	1.47082069	1.83105939	7.15E-06	3.57E-05	0.23	0.2	0.54	1.07	SREK1IP1	SREK1-interacting protein 1	chr5	protein_coding
ENSG00000139044.9	3.87670736	2.04430996	1.21E-24	4.08E-23	0.13	0.03	1.73	1.47	B4GALNT3	beta-1,4-N-acetyl-galactosaminyl transferase 3	chr12	protein_coding
ENSG00000085563.13	-3.3963132	-1.1757392	6.44E-08	4.44E-07	0.16	0.09	0	0.03	ABCB1	ATP-binding cassette, sub-family B (MDR/TAP), member 1	chr7	protein_coding
ENSG00000177932.6	5.06648085	0.42391243	1.92E-20	4.76E-19	0.02	0	0.41	0.55	ZNF354C	zinc finger protein 354C	chr5	protein_coding
ENSG00000108511.9	1.27139859	4.27931195	1.63E-05	7.60E-05	3.89	5.48	14.33	15.78	HOXB6	homeobox B6	chr17	protein_coding
ENSG00000185187.11	-5.2381342	3.55725752	4.12E-44	4.00E-42	10.23	7.34	0.36	0.26	SIGIRR	single immunoglobulin and toll-interleukin 1 receptor (TIR) domain	chr11	protein_coding
ENSG00000176428.5	-1.372338	-0.0936975	0.00113143	0.00367616	0.59	0.7	0.43	0.23	VPS37D	vacuolar protein sorting 37 homolog D (S. cerevisiae)	chr7	protein_coding
ENSG00000261130.4	-7.4799666	1.47860733	2.30E-38	1.60E-36	7.22	6.56	0.1	0	RP11-1021N	--	chr16	protein_coding
ENSG00000125780.11	9.93016392	0.97069117	2.55E-31	1.25E-29	0	0	1.38	1.63	TGM3	transglutaminase 3	chr20	protein_coding
ENSG00000261373.1	-7.35197	3.02040668	4.97E-55	9.38E-53	6.96	7.99	0.05	0.06	VPS9D1-AS1	VPS9D1 antisense RNA 1	chr16	antisense
ENSG00000272102.1	6.11073974	-2.3182927	4.74E-05	0.00020301	0	0	0.41	0.6	RP3-355L5.5	--	chr6	lincRNA
ENSG00000182923.15	1.8427875	1.83701451	3.12E-08	2.22E-07	0.19	0.17	0.72	1.03	CEP63	centrosomal protein 63kDa	chr3	protein_coding
ENSG00000189157.12	2.07233539	-1.5386521	0.00122775	0.00396173	0.05	0.03	0.3	0.17	FAM47E	family with sequence similarity 47, member E	chr4	protein_coding
ENSG00000141753.6	1.50924192	9.08872465	1.97E-07	1.27E-06	108.25	100	399.55	390.27	IGFBP4	insulin-like growth factor binding protein 4	chr17	protein_coding
ENSG00000173237.4	2.43673312	5.05099411	1.20E-15	2.02E-14	8.99	5.01	53.7	47.51	C11orf86	chromosome 11 open reading frame 86	chr11	protein_coding
ENSG00000110756.16	2.11784046	2.75799298	3.28E-11	3.46E-10	0.26	0.42	1.65	2.34	HPS5	Hermansky-Pudlak syndrome 5	chr11	protein_coding
ENSG00000259539.1	-2.8641168	-2.5097724	0.00190233	0.00584723	0.27	0.33	0	0.09	CTD-2651B2	--	chr15	3prime_overlapping_ncrna
ENSG00000100625.8	1.53363322	0.11399871	0.00016219	0.00062317	0.05	0.09	0.22	0.29	SIX4	SIX homeobox 4	chr14	protein_coding
ENSG00000255847.4	3.8680795	-2.1162687	8.40E-05	0.0003415	0.02	0	0.23	0.23	RP11-167N4	--	chr11	antisense
ENSG00000241357.1	6.03578111	-2.3706777	8.36E-05	0.00034052	0	0	0.67	0.9	RP11-758P1'	--	chr7	antisense
ENSG00000182040.7	1.23456952	-0.9965163	0.01384859	0.03450201	0.05	0.06	0.31	0.07	USH1G	Usher syndrome 1G (autosomal recessive)	chr17	protein_coding
ENSG00000102531.15	1.44412102	1.6175607	1.45E-05	6.85E-05	0.16	0.18	0.63	0.61	FNDC3A	fibronectin type III domain containing 3A	chr13	protein_coding
ENSG00000006283.16	-2.8941862	3.09551864	6.83E-19	1.52E-17	1.37	1.43	0.28	0.22	CACNA1G	calcium channel, voltage-dependent, T type, alpha 1G subunit	chr17	protein_coding
ENSG00000177485.6	2.3119631	-0.136006	5.38E-07	3.24E-06	0.04	0.05	0.17	0.38	ZBTB33	zinc finger and BTB domain containing 33	chrX	protein_coding
ENSG00000146414.14	3.51028872	0.23322304	1.64E-13	2.23E-12	0.02	0.01	0.16	0.34	SHPRH	SNF2 histone linker PHD RING helicase, E3 ubiquitin protein ligase	chr6	protein_coding
ENSG00000116337.14	-1.154022	6.454241	6.40E-05	0.00026808	23.19	18.6	12.02	13.06	AMPD2	adenosine monophosphate deaminase 2	chr1	protein_coding
ENSG00000106723.15	1.37776942	3.21749054	5.47E-06	2.79E-05	0.94	0.91	2.78	3.64	SPIN1	spindlin 1	chr9	protein_coding
ENSG00000112146.15	1.63523455	4.65198278	3.63E-08	2.57E-07	1.88	1.99	6.21	9.9	FBXO9	F-box protein 9	chr6	protein_coding
ENSG00000277151.1	5.57068129	-2.6758978	0.00086564	0.00288128	0	0	0.02	0.05	RP11-380B4.	--	chr13	lincRNA
ENSG00000279089.1	6.11073974	-2.3182927	4.74E-05	0.00020301	0	0	0.07	0.1	RP11-481C4.	--	chr17	TEC
ENSG00000252690.3	4.7326838	-0.7185229	2.05E-11	2.22E-10	0.01	0	0.21	0.28	SCARNA15	small Cajal body-specific RNA 15	chr15	processed_transcript
ENSG00000280088.1	2.88295914	-2.1532515	0.00067192	0.00229154	0.03	0	0.22	0.12	RP11-135F9.	--	chr12	TEC
ENSG00000116299.15	-1.3977476	-0.6368678	0.00261113	0.00784386	0.13	0.28	0.11	0.09	KIAA1324	KIAA1324	chr1	protein_coding
ENSG00000224940.7	5.9852199	0.904025	5.99E-27	2.37E-25	0.02	0.01	1.1	1.01	PRRT4	proline-rich transmembrane protein 4	chr7	protein_coding
ENSG00000163517.13	1.04835658	4.07963185	0.00037179	0.00133301	3.01	2.16	7.93	6.28	HDAC11	histone deacetylase 11	chr3	protein_coding
ENSG00000227741.1	2.4906156	-2.4026582	0.00737015	0.01977584	0.03	0	0.26	0.03	RP11-536C5.	--	chr1	antisense
ENSG00000156650.11	1.20931896	2.79431069	8.30E-05	0.00034052	0.28	0.52	1.1	1.36	KAT6B	K(lysine) acetyltransferase 6B	chr10	protein_coding
ENSG00000185338.4	2.49184735	0.00288529	3.28E-08	2.33E-07	0.09	0.28	1.49	1.24	SOCS1	suppressor of cytokine signaling 1	chr16	protein_coding
ENSG00000152684.10	-1.1935567	4.68529958	4.37E-05	0.00018966	7.25	6.05	3.56	4.21	PELO	pelota homolog (Drosophila)	chr5	protein_coding
ENSG00000117308.13	-1.298042	5.94250903	7.75E-06	3.85E-05	49.86	39.5	26.95	21.38	GALE	UDP-galactose-4-epimerase	chr1	protein_coding
ENSG00000080031.8	-1.8185957	-0.3590286	4.49E-05	0.00019447	0.18	0.31	0.08	0.1	PTPRH	protein tyrosine phosphatase, receptor type, H	chr19	protein_coding
ENSG00000167740.8	1.13603507	4.28999317	0.00011044	0.00043976	4.97	4.59	13.73	14.32	CYB5D2	cytochrome b5 domain containing 2	chr17	protein_coding
ENSG00000074276.9	4.9050007	-3.0539124	0.01889873	0.04519557	0	0	0.04	0.02	CDHR2	cadherin-related family member 2	chr5	protein_coding
ENSG00000225905.1	5.87712165	-2.4793333	0.00014842	0.00057423	0	0	0.47	0.4	RP4-758J18.'	--	chr1	antisense
ENSG00000261040.5	-1.0003453	5.35978519	0.00054453	0.0018918	72.17	68.42	44.95	48.8	WFDC21P	WAP four-disulfide core domain 21, pseudogene	chr17	processed_transcript
ENSG00000137100.14	-1.2004115	6.51700291	3.27E-05	0.00014439	70.81	67.35	44.25	35.65	DCTN3	dynactin 3 (p22)	chr9	protein_coding
ENSG00000165055.14	-1.2172998	4.50938588	3.33E-05	0.00014699	4.28	4.51	2.56	2.48	METTL2B	methyltransferase like 2B	chr7	protein_coding
ENSG00000141084.9	1.49673101	5.38305605	3.20E-07	1.99E-06	2.72	3.76	11.98	12.35	RANBP10	RAN binding protein 10	chr16	protein_coding
ENSG00000140832.8	5.1764309	-0.3347656	7.52E-15	1.19E-13	0.01	0.01	0.35	0.27	MARVELD3	MARVEL domain containing 3	chr16	protein_coding
ENSG00000268460.1	-3.0762186	3.17001188	5.40E-21	1.40E-19	7.81	8.15	1.12	1.4	DKFZp434J0:	--	chr19	processed_transcript
ENSG00000206077.9	3.13125612	-2.5967938	0.00552196	0.01522223	0.01	0	0.1	0.05	ZDHHC11B	zinc finger, DHHC-type containing 11B	chr5	protein_coding
ENSG00000166912.15	1.6502739	0.55702687	1.88E-05	8.65E-05	0.07	0.12	0.19	0.6	MTMR10	myotubularin related protein 10	chr15	protein_coding
ENSG00000119912.14	1.97602159	2.67856285	5.45E-10	4.93E-09	0.34	0.35	1.63	2.01	IDE	insulin-degrading enzyme	chr10	protein_coding
ENSG00000180447.6	-3.5463685	5.63320027	7.25E-29	3.16E-27	24.77	27.65	2.91	3.06	GAS1	growth arrest-specific 1	chr9	protein_coding
ENSG00000135636.12	3.96056124	1.98820636	1.15E-24	3.89E-23	0.02	0.09	1.07	1.11	DYSF	dysferlin	chr2	protein_coding
ENSG00000279837.1	5.45974547	-2.7440718	0.00158008	0.00495521	0	0	0.17	0.28	RP11-504G3	--	chr11	TEC
ENSG00000170191.4	2.5252833	0.18956543	6.16E-09	4.88E-08	0.03	0.09	0.38	0.59	NANP	N-acetylneuraminic acid phosphatase	chr20	protein_coding
ENSG00000038219.11	3.92443683	3.31841067	1.29E-29	5.86E-28	0.06	0.13	1.16	2.64	BOD1L1	biorientation of chromosomes in cell division 1-like 1	chr4	protein_coding
ENSG00000169174.10	-1.4314166	7.5665598	7.91E-07	4.63E-06	66.57	56.45	31.95	28.78	PCSK9	proprotein convertase subtilisin/kexin type 9	chr1	protein_coding
ENSG00000071564.13	-1.2478569	8.01345652	1.52E-05	7.14E-05	50.9	53.1	30.14	28.13	TCF3	transcription factor 3	chr19	protein_coding
ENSG00000267890.1	7.90823843	-0.8866459	2.26E-12	2.73E-11	0	0	1.84	1.56	CTD-2126E3	--	chr19	lincRNA
ENSG00000114853.12	-1.1572996	5.85136311	6.37E-05	0.00026726	14.99	19.18	10.56	9.81	ZBTB47	zinc finger and BTB domain containing 47	chr3	protein_coding
ENSG00000129472.11	1.04694369	3.44347309	0.00045475	0.00160219	1.65	2.25	4.88	5.86	RAB2B	RAB2B, member RAS oncogene family	chr14	protein_coding
ENSG00000180998.10	7.52329753	-1.2147799	3.23E-11	3.41E-10	0	0	0.17	0.21	GPR137C	G protein-coupled receptor 137C	chr14	protein_coding
ENSG00000229771.2	-5.2091082	-3.0354822	0.00538165	0.01488851	0.06	0.07	0	0	RP4-644L1.2	--	chr20	lincRNA
ENSG00000246308.1	6.05360204	-2.3605161	4.74E-05	0.00020301	0	0	0.15	0.08	RP11-685M7	--	chr11	antisense
ENSG00000116752.5	1.1458955	2.7419393	0.000186	0.00070617	2.12	3.01	6.83	8.3	BCAS2	breast carcinoma amplified sequence 2	chr1	protein_coding
ENSG00000144476.5	1.02641877	5.58495066	0.00038659	0.00138217	11.45	11.74	28.31	34.8	ACKR3	atypical chemokine receptor 3	chr2	protein_coding
ENSG00000179021.8	3.80792028	0.84520895	8.81E-18	1.78E-16	0.07	0.06	0.82	1.77	C3orf38	chromosome 3 open reading frame 38	chr3	protein_coding
ENSG00000231233.1	-5.6574516	-2.7834951	0.00086564	0.00288128	0.2	0.18	0	0	CCDC147-A‹	--	chr10	antisense
ENSG00000010072.14	1.1536803	1.38429549	0.00058071	0.00201023	0.21	0.25	0.71	0.67	SPRTN	SprT-like N-terminal domain	chr1	protein_coding
ENSG00000158486.12	2.06304783	-1.5458297	0.00173462	0.0053947	0.01	0	0.04	0.04	DNAH3	dynein, axonemal, heavy chain 3	chr16	protein_coding
ENSG00000127418.13	-1.8346216	6.75523732	4.24E-10	3.91E-09	32.59	37	13.35	12.61	FGFRL1	fibroblast growth factor receptor-like 1	chr4	protein_coding
ENSG00000169679.13	1.29214321	3.54603823	1.63E-05	7.58E-05	1.67	1.31	3.76	6.03	BUB1	BUB1 mitotic checkpoint serine/threonine kinase	chr2	protein_coding
ENSG00000077044.8	-3.1799385	6.8989839	8.83E-25	3.00E-23	28.59	25.78	4.17	3.81	DGKD	diacylglycerol kinase, delta 130kDa	chr2	protein_coding
ENSG00000280353.1	1.47923364	-1.33744	0.0065433	0.01777607	0.1	0.07	0.6	0.07	CTC-453G23	--	chr19	TEC
ENSG00000109107.12	-2.3807541	4.86260547	4.68E-15	7.52E-14	22.34	16.38	4.94	4.98	ALDOC	aldolase C, fructose-bisphosphate	chr17	protein_coding
ENSG00000159915.11	5.78313448	-2.5411454	0.00026535	0.0009747	0	0	0.06	0.06	ZNF233	zinc finger protein 233	chr19	protein_coding
ENSG00000140009.17	1.23915645	-0.8314677	0.01238926	0.0311959	0.03	0.02	0.03	0.13	ESR2	estrogen receptor 2 (ER beta)	chr14	protein_coding
ENSG00000140299.10	1.59217216	2.27502281	6.35E-07	3.77E-06	0.32	0.27	0.84	1.53	BNIP2	BCL2/adenovirus E1B 19kDa interacting protein 2	chr15	protein_coding
ENSG00000187486.5	-3.543148	0.23557445	1.17E-14	1.82E-13	0.78	0.38	0.11	0.03	KCNJ11	potassium channel, inwardly rectifying subfamily J, member 11	chr11	protein_coding
ENSG00000198538.9	3.07719614	-0.7795092	7.16E-08	4.91E-07	0	0.03	0.14	0.2	ZNF28	zinc finger protein 28	chr19	protein_coding
ENSG00000132561.12	1.51677352	2.38864122	1.77E-06	9.80E-06	0.38	0.59	1.4	2.31	MATN2	matrilin 2	chr8	protein_coding
ENSG00000260841.1	-2.6362074	-0.3406445	2.43E-08	1.76E-07	0.39	0.29	0.11	0.04	CTC-205M6.	--	chr5	sense_overlapping
ENSG00000115297.10	6.79985599	2.05392226	4.75E-40	3.59E-38	0.03	0.01	3.68	3.04	TLX2	T-cell leukemia homeobox 2	chr2	protein_coding
ENSG00000280311.1	5.45490402	-2.7467519	0.00158008	0.00495521	0	0	0.1	0.25	RP13-554M1	--	chr12	TEC
ENSG00000099860.7	-1.6263	7.53641916	2.41E-08	1.74E-07	150.48	140.88	60.2	65.72	GADD45B	growth arrest and DNA-damage-inducible, beta	chr19	protein_coding
ENSG00000277978.1	3.74390614	-2.2030658	0.00013996	0.00054609	0	0.07	1.01	0.57	RP11-403P1'	--	chr16	antisense
ENSG00000198924.6	1.52948315	-0.2396733	0.00044287	0.00156354	0.02	0.12	0.26	0.29	DCLRE1A	DNA cross-link repair 1A	chr10	protein_coding
ENSG00000198865.8	-5.0197065	-3.1311226	0.0100455	0.02582742	0.05	0.01	0	0	CCDC152	coiled-coil domain containing 152	chr5	protein_coding
ENSG00000135932.9	1.38041721	2.9116216	6.93E-06	3.47E-05	0.68	0.85	1.83	3.52	CAB39	calcium binding protein 39	chr2	protein_coding
ENSG00000173442.10	-2.780855	8.61566989	5.02E-20	1.21E-18	102.16	95.95	19.11	19.31	EHBP1L1	EH domain binding protein 1-like 1	chr11	protein_coding
ENSG00000140575.11	1.81800824	6.20145288	6.51E-10	5.83E-09	3.38	3.89	14.14	20.17	IQGAP1	IQ motif containing GTPase activating protein 1	chr15	protein_coding
ENSG00000175920.14	4.75452647	4.48306653	6.97E-42	5.78E-40	0.4	0.61	19	17.05	DOK7	docking protein 7	chr4	protein_coding
ENSG00000019995.6	1.17247287	3.7029262	8.26E-05	0.00033933	1.05	1.21	3.13	3.67	ZRANB1	zinc finger, RAN-binding domain containing 1	chr10	protein_coding
ENSG00000204574.11	-1.1070039	8.00939166	0.00011807	0.00046745	73.38	90.49	49.01	52.33	ABCF1	ATP-binding cassette, sub-family F (GCN20), member 1	chr6	protein_coding
ENSG00000196139.10	-1.0006122	6.19999483	0.00051301	0.00178878	35.77	33.9	21.79	24.68	AKR1C3	aldo-keto reductase family 1, member C3	chr10	protein_coding
ENSG00000140993.9	2.78807901	0.40009083	7.73E-11	7.83E-10	0.1	0.03	0.35	0.93	TIGD7	tigger transposable element derived 7	chr16	protein_coding
ENSG00000109667.10	2.37650351	-2.4696084	0.01191236	0.03011315	0.03	0	0.14	0.06	SLC2A9	solute carrier family 2 (facilitated glucose transporter), member 9	chr4	protein_coding
ENSG00000267680.4	3.12985343	1.67642488	3.51E-17	6.71E-16	0.14	0.11	1.09	1.92	ZNF224	zinc finger protein 224	chr19	protein_coding
ENSG00000170482.15	2.45330034	-2.4232357	0.01191236	0.03011315	0.02	0	0.07	0.12	SLC23A1	solute carrier family 23 (ascorbic acid transporter), member 1	chr5	protein_coding
ENSG00000109919.8	-1.0378443	7.93189416	0.00030102	0.00109667	111.72	93.65	60.12	73.62	MTCH2	mitochondrial carrier 2	chr11	protein_coding
ENSG00000182827.8	1.63071953	3.11301551	1.14E-07	7.60E-07	0.59	1.31	3.29	4.54	ACBD3	acyl-CoA binding domain containing 3	chr1	protein_coding
ENSG00000279432.1	6.74735162	-1.8525552	2.24E-07	1.42E-06	0	0	0.21	0.08	RP11-135N5	--	chr17	TEC
ENSG00000070761.6	1.48755596	6.18080604	3.32E-07	2.07E-06	23.29	22.25	87.27	82.78	CFAP20	cilia and flagella associated protein 20	chr16	protein_coding
ENSG00000137312.13	-2.152097	9.03286396	3.64E-13	4.76E-12	363.67	360.56	112.48	104.38	FLOT1	flotillin 1	chr6	protein_coding
ENSG00000128266.8	-2.8330113	4.67215042	8.14E-20	1.94E-18	10.58	11.15	2.07	1.99	GNAZ	guanine nucleotide binding protein (G protein), alpha z polypeptide	chr22	protein_coding
ENSG00000099365.8	-1.5643573	1.49434039	2.71E-06	1.46E-05	0.6	0.78	0.28	0.34	STX1B	syntaxin 1B	chr16	protein_coding
ENSG00000181638.16	1.24601191	5.05930235	1.94E-05	8.92E-05	2.4	2.74	8.71	7.5	ZFP41	ZFP41 zinc finger protein	chr8	protein_coding
ENSG00000279409.1	5.91404262	-2.4585478	8.36E-05	0.00034052	0	0	0.27	0	RP11-133K1.	--	chr15	TEC
ENSG00000278979.1	4.88946445	-3.0622981	0.01889873	0.04519557	0	0	0.04	0.1	RP11-165M1	--	chr16	TEC
ENSG00000127824.12	-1.9164868	7.88517495	7.06E-11	7.22E-10	122.41	103.58	39.97	39.85	TUBA4A	tubulin, alpha 4a	chr2	protein_coding
ENSG00000138207.11	2.52187201	3.12229524	3.19E-15	5.20E-14	1.53	1.31	11.12	10.67	RBP4	retinol binding protein 4, plasma	chr10	protein_coding
ENSG00000170458.12	6.09257043	4.00831699	1.11E-53	1.93E-51	0.3	0.09	19.79	16.11	CD14	CD14 molecule	chr5	protein_coding
ENSG00000260233.3	1.48337342	-1.2203371	0.0105162	0.02693027	0.39	0.09	1.36	0.53	SSSCA1-AS1	SSSCA1 antisense RNA 1 (head to head)	chr11	processed_transcript
ENSG00000276903.1	1.65533102	0.00510983	8.28E-05	0.0003401	0.54	1.3	5.95	1.8	HIST1H2AL	histone cluster 1, H2al	chr6	protein_coding
ENSG00000176884.13	4.39785858	0.34320361	2.71E-17	5.25E-16	0.01	0.02	0.47	0.45	GRIN1	glutamate receptor, ionotropic, N-methyl D-aspartate 1	chr9	protein_coding
ENSG00000154727.9	3.67039959	1.5568516	1.04E-20	2.65E-19	0.07	0.06	0.8	1.33	GABPA	GA binding protein transcription factor, alpha subunit 60kDa	chr21	protein_coding
ENSG00000130475.13	-3.7352715	2.24032075	7.91E-25	2.71E-23	2.21	1.93	0.22	0.19	FCHO1	FCH domain only 1	chr19	protein_coding
ENSG00000232237.3	-5.5205905	-2.8639386	0.00158008	0.00495521	0.11	0.03	0	0	ASCL5	achaete-scute family bHLH transcription factor 5	chr1	protein_coding
ENSG00000080822.15	2.27709159	2.86026981	1.17E-12	1.46E-11	0.3	0.64	2.59	3.5	CLDND1	claudin domain containing 1	chr3	protein_coding
ENSG00000135048.12	1.78782588	3.42063199	5.09E-09	4.06E-08	0.57	0.59	2.23	3.12	TMEM2	transmembrane protein 2	chr9	protein_coding
ENSG00000118432.12	-6.2437918	-0.5468831	2.41E-17	4.69E-16	0.14	0.2	0	0	CNR1	cannabinoid receptor 1 (brain)	chr6	protein_coding
ENSG00000258725.1	2.53373985	-2.3738362	0.00737015	0.01977584	0.03	0	0.05	0.2	PRC1-AS1	PRC1 antisense RNA 1	chr15	antisense
ENSG00000136367.13	4.27727947	0.22871311	3.62E-16	6.37E-15	0.01	0.01	0.33	0.13	ZFHX2	zinc finger homeobox 2	chr14	protein_coding
ENSG00000184307.12	2.78591625	1.38183685	7.72E-14	1.09E-12	0.1	0.16	1.2	1.27	ZDHHC23	zinc finger, DHHC-type containing 23	chr3	protein_coding
ENSG00000256294.6	3.01907996	1.62223064	3.11E-16	5.52E-15	0.08	0.14	0.84	1.57	ZNF225	zinc finger protein 225	chr19	protein_coding
ENSG00000113851.12	1.16067656	1.40227131	0.00054039	0.00187906	0.64	0.51	1.15	2.32	CRBN	cereblon	chr3	protein_coding
ENSG00000279266.1	6.74809254	-1.8435441	2.24E-07	1.42E-06	0	0	0.07	0.16	RP11-371I20	--	chr11	TEC
ENSG00000204758.6	-2.5825881	-0.1511104	1.32E-08	9.91E-08	0.45	0.42	0.12	0.08	CTC-308K20	--	chr5	antisense
ENSG00000067167.6	1.42905836	3.34677386	2.43E-06	1.32E-05	1.21	1.18	3.16	5.49	TRAM1	translocation associated membrane protein 1	chr8	protein_coding
ENSG00000254389.3	2.5113877	-0.0731274	2.84E-08	2.04E-07	0.18	0.03	0.78	0.87	RHPN1-AS1	RHPN1 antisense RNA 1 (head to head)	chr8	antisense
ENSG00000121988.16	1.12610712	0.02873624	0.00544495	0.01505303	0.11	0.01	0.15	0.2	ZRANB3	zinc finger, RAN-binding domain containing 3	chr2	protein_coding
ENSG00000163389.9	1.26273526	1.10592778	0.00028765	0.00105082	0.16	0.4	0.54	1.24	POGLUT1	protein O-glucosyltransferase 1	chr3	protein_coding
ENSG00000267123.4	-7.8596167	1.84299888	7.13E-45	7.24E-43	5.89	7.37	0	0.06	CTD-2357A8	--	chr17	lincRNA
ENSG00000279912.1	3.27305803	-0.4262273	1.80E-09	1.52E-08	0	0.05	0.27	0.38	RP11-624A2	--	chr15	TEC
ENSG00000260851.5	1.23983486	3.66245018	3.24E-05	0.00014325	7.59	9.7	26.09	28.32	RP11-403P1'	--	chr16	protein_coding
ENSG00000238083.6	1.78489331	0.04583892	1.73E-05	8.02E-05	0.08	0.03	0.24	0.32	LRRC37A2	leucine rich repeat containing 37, member A2	chr17	protein_coding
ENSG00000182628.11	1.24966612	4.55373146	2.04E-05	9.35E-05	3.81	3.68	8.77	15.13	SKA2	spindle and kinetochore associated complex subunit 2	chr17	protein_coding
ENSG00000269924.1	5.32944093	-2.821228	0.00290522	0.00856312	0	0	0.09	0.33	RP11-697N1	--	chr8	sense_intronic
ENSG00000140459.16	3.37826396	6.09773251	7.10E-27	2.77E-25	3.82	4.2	57.7	53.14	CYP11A1	cytochrome P450, family 11, subfamily A, polypeptide 1	chr15	protein_coding

ENSG00000070214.14	1.55208642	3.20928024	3.69E-07	2.28E-06	0.28	0.38	1.01	1.59	SLC44A1	solute carrier family 44 (choline transporter), member 1	chr9	protein_coding
ENSG00000198286.8	-7.3819812	-1.5131779	4.49E-10	4.11E-09	0.17	0.06	0	0	CARD11	caspase recruitment domain family, member 11	chr7	protein_coding
ENSG00000111816.7	5.48410531	-2.7306244	0.00158008	0.00495521	0	0	0.02	0.01	FRK	fyn-related Src family tyrosine kinase	chr6	protein_coding
ENSG00000122966.12	1.12041812	5.66712145	0.00010942	0.00043643	2.68	3.27	8.95	8.25	CIT	citron rho-interacting serine/threonine kinase	chr12	protein_coding
ENSG00000103353.14	-1.8775264	5.48207076	2.15E-10	2.05E-09	11.05	10.94	3.83	4.15	UBFD1	ubiquitin family domain containing 1	chr16	protein_coding
ENSG00000148143.11	2.35266725	2.02195406	3.60E-12	4.26E-11	0.1	0.1	0.59	0.76	ZNF462	zinc finger protein 462	chr9	protein_coding
ENSG00000106263.16	-1.6707374	9.50237807	9.83E-09	7.54E-08	297.98	286.59	120.82	123.93	EIF3B	eukaryotic translation initiation factor 3, subunit B	chr7	protein_coding
ENSG00000165572.7	2.51906832	1.156973	1.38E-11	1.53E-10	0.11	0.08	0.51	1	KBTBD6	kelch repeat and BTB (POZ) domain containing 6	chr13	protein_coding
ENSG00000029153.13	1.44323114	0.43105152	0.00016327	0.00062582	0.1	0.06	0.28	0.3	ARNTL2	aryl hydrocarbon receptor nuclear translocator-like 2	chr12	protein_coding
ENSG00000159871.13	2.33458685	-0.121827	3.48E-07	2.16E-06	0.06	0.1	0.53	0.55	LYPD5	LY6/PLAUR domain containing 5	chr19	protein_coding
ENSG00000185989.10	-2.8658109	7.1109409	5.79E-21	1.50E-19	49.97	43.9	8.26	8.92	RASA3	RAS p21 protein activator 3	chr13	protein_coding
ENSG00000132424.13	1.8770365	4.04453612	5.37E-10	4.87E-09	1.02	1.19	3.21	7.72	PNISR	PNN-interacting serine/arginine-rich protein	chr6	protein_coding
ENSG00000104442.8	1.67634461	1.4529008	8.48E-07	4.94E-06	0.37	0.3	0.91	1.96	ARMC1	armadillo repeat containing 1	chr8	protein_coding
ENSG00000261270.1	1.9084471	-1.6486393	0.00486779	0.01371541	0.27	0	0.59	0.82	RP11-325K4.	--	chr16	sense_intronic
ENSG00000238258.1	-5.2109276	-3.0346387	0.00538165	0.01488851	0.07	0.22	0	0	RP11-342D1	--	chr10	antisense
ENSG00000111186.11	-3.0419079	3.17829607	1.39E-20	3.50E-19	5.38	4.76	0.86	0.78	WNT5B	wingless-type MMTV integration site family, member 5B	chr12	protein_coding
ENSG00000170989.8	-4.8907605	0.76810751	5.86E-25	2.02E-23	0.64	1.2	0	0.07	S1PR1	sphingosine-1-phosphate receptor 1	chr1	protein_coding
ENSG00000170786.11	-2.9156446	1.80555176	1.28E-16	2.35E-15	1.61	1.43	0.24	0.29	SDR16C5	short chain dehydrogenase/reductase family 16C, member 5	chr8	protein_coding
ENSG00000214562.12	-1.7936154	-1.7369728	0.00684841	0.01852289	0.06	0.03	0.03	0.01	NUTM2D	NUT family member 2D	chr10	protein_coding
ENSG00000064199.5	1.97738852	2.34815125	9.14E-10	8.04E-09	0.39	0.27	1.46	1.98	SPA17	sperm autoantigenic protein 17	chr11	protein_coding
ENSG00000135870.10	1.76995003	1.25704528	4.26E-07	2.61E-06	0.06	0.09	0.33	0.35	RC3H1	ring finger and CCCH-type domains 1	chr1	protein_coding
ENSG00000163017.12	-1.8007773	-1.9074071	0.00967167	0.02519005	0.18	0.02	0.01	0.06	ACTG2	actin, gamma 2, smooth muscle, enteric	chr2	protein_coding
ENSG00000119669.4	-1.2616562	7.06680707	1.26E-05	6.01E-05	37.59	36.08	20.83	20.1	IRF2BPL	interferon regulatory factor 2 binding protein-like	chr14	protein_coding
ENSG00000214253.7	-2.137949	7.17447973	5.58E-13	7.19E-12	169.41	138.69	45.43	47.99	FIS1	fission 1 (mitochondrial outer membrane) homolog (S. cerevisiae)	chr7	protein_coding
ENSG00000159352.14	-1.0305315	8.55343798	0.00032887	0.0011891	299.22	301.83	193.68	198.42	PSMD4	proteasome (prosome, macropain) 26S subunit, non-ATPase, 4	chr1	protein_coding
ENSG00000075624.12	-1.6268177	12.8267327	2.23E-08	1.62E-07	4668.73	4601.87	2038.42	1958.86	ACTB	actin, beta	chr7	protein_coding
ENSG00000154114.11	2.12697483	1.48427271	1.27E-09	1.09E-08	0.16	0.15	0.88	0.94	TBCEL	tubulin folding cofactor E-like	chr11	protein_coding
ENSG00000112787.11	-1.2136563	6.46635847	2.69E-05	0.00012094	16.98	18.97	11.41	9.17	FBRSL1	fibrosin-like 1	chr12	protein_coding
ENSG00000250742.1	1.51283316	0.05069248	0.00025756	0.00095503	0.27	0.31	1.08	1.15	RP11-834C1	--	chr12	lincRNA
ENSG00000164674.14	1.72674301	1.0272168	1.51E-06	8.47E-06	0.22	0.28	1.26	0.97	SYTL3	synaptotagmin-like 3	chr6	protein_coding
ENSG00000198954.5	1.36921149	3.90041388	4.39E-06	2.28E-05	2.29	2.99	7.66	10.55	KIAA1279	--	chr10	protein_coding
ENSG00000196417.11	3.01196646	0.98174658	4.03E-14	5.85E-13	0.01	0.04	0.29	0.32	ZNF765	zinc finger protein 765	chr19	protein_coding
ENSG00000083845.7	-1.8745181	9.43580526	1.64E-10	1.59E-09	452.05	401.67	163.69	146.2	RPS5	ribosomal protein S5	chr19	protein_coding
ENSG00000234546.2	1.57303477	-0.0608601	0.0001608	0.00061833	0.62	0.09	1.56	1.33	RP3-510D11	--	chr1	lincRNA
ENSG00000174567.7	2.70518459	-1.2916409	2.93E-05	0.00013052	0.15	0	0.4	0.99	GOLT1A	golgi transport 1A	chr1	protein_coding
ENSG00000010361.12	-3.5248243	3.09260615	2.26E-25	8.02E-24	6.87	8.06	0.84	0.88	FUZ	fuzzy planar cell polarity protein	chr19	protein_coding
ENSG00000172757.11	-1.3788761	10.3823684	1.83E-06	1.01E-05	673.45	611.29	336.26	321.8	CFL1	cofilin 1 (non-muscle)	chr11	protein_coding
ENSG00000169429.9	-6.3476014	-0.4491322	2.60E-18	5.49E-17	0.61	0.52	0	0.01	CXCL8	chemokine (C-X-C motif) ligand 8	chr4	protein_coding
ENSG00000107951.11	1.32209683	1.39071169	9.96E-05	0.00040127	0.19	0.23	0.69	0.71	MTPAP	mitochondrial poly(A) polymerase	chr10	protein_coding
ENSG00000205250.7	1.35377187	7.78175548	2.87E-06	1.55E-05	47.49	48.09	167.65	157.53	E2F4	E2F transcription factor 4, p107/p130-binding	chr16	protein_coding
ENSG00000120837.6	1.60934826	3.176616	1.58E-07	1.03E-06	0.75	0.98	2.72	4.31	NFYB	nuclear transcription factor Y, beta	chr12	protein_coding
ENSG00000120686.10	2.31691538	2.04875063	5.64E-12	6.53E-11	0.33	0.32	1.26	3.11	UFM1	ubiquitin-fold modifier 1	chr13	protein_coding
ENSG00000271989.1	5.19748351	-2.8969879	0.00538165	0.01488851	0	0	0.08	0.27	RP4-736L20.	--	chr1	antisense
ENSG00000178952.7	-1.4398457	10.3579622	6.54E-07	3.87E-06	764.02	735.19	370.19	366.06	TUFM	Tu translation elongation factor, mitochondrial	chr16	protein_coding
ENSG00000145088.7	2.68467611	-1.8749565	0.00119227	0.00385709	0.05	0.02	0.16	0.47	EAF2	ELL associated factor 2	chr3	protein_coding
ENSG00000137203.9	2.82168235	5.04528441	8.31E-20	1.97E-18	1.5	1.36	13.18	13.8	TFAP2A	transcription factor AP-2 alpha (activating enhancer binding protein 2 alpha)	chr6	protein_coding
ENSG00000273274.1	3.53875995	-1.2452763	6.12E-07	3.65E-06	0.01	0	0.06	0.04	ZBTB8B	zinc finger and BTB domain containing 8B	chr1	protein_coding
ENSG00000109534.15	-1.6396242	6.06675949	2.11E-08	1.54E-07	61.33	68.84	25.39	30.36	GAR1	GAR1 ribonucleoprotein	chr4	protein_coding
ENSG00000197122.10	2.05254586	5.05434946	7.42E-12	8.43E-11	2.39	2.04	13.08	11.39	SRC	SRC proto-oncogene, non-receptor tyrosine kinase	chr20	protein_coding
ENSG00000274191.1	2.28237454	-1.8451984	0.00303791	0.00892022	0	0.21	1	0.4	RP11-324E6.	--	chr12	lincRNA
ENSG00000254761.1	-6.5730459	-2.1625829	1.78E-06	9.85E-06	0.51	0.17	0	0	RP11-672A2	--	chr11	lincRNA
ENSG00000188786.9	1.33364125	2.46358279	2.05E-05	9.38E-05	0.29	0.34	1.1	1	MTF1	metal-regulatory transcription factor 1	chr1	protein_coding
ENSG00000116871.14	-1.2847227	9.56779708	8.39E-06	4.13E-05	220.53	253.82	136.34	122.49	MAP7D1	MAP7 domain containing 1	chr1	protein_coding
ENSG00000140284.9	1.42693335	4.1205032	1.59E-06	8.89E-06	2.31	3.23	7.8	12.11	SLC27A2	solute carrier family 27 (fatty acid transporter), member 2	chr15	protein_coding
ENSG00000017483.13	-10.231677	7.43416308	2.19E-113	6.57E-110	84.71	93.01	0.07	0.12	SLC38A5	solute carrier family 38, member 5	chrX	protein_coding
ENSG00000179761.10	5.06203518	-2.9716376	0.0100455	0.02582742	0	0	0.05	0.06	PIPOX	pipecolic acid oxidase	chr17	protein_coding
ENSG00000225822.4	5.04175262	-2.9826774	0.0100455	0.02582742	0	0	0	0.63	UBXN7-AS1	UBXN7 antisense RNA 1	chr3	antisense
ENSG00000102572.13	-1.4495505	7.25510817	5.87E-07	3.51E-06	17.22	19.48	8.75	9.15	STK24	serine/threonine kinase 24	chr13	protein_coding
ENSG00000178665.13	1.83992923	-0.6668683	0.00018637	0.00070726	0.05	0.06	0.21	0.31	ZNF713	zinc finger protein 713	chr7	protein_coding
ENSG00000170113.14	2.83046499	3.9404116	4.23E-19	9.56E-18	0.4	0.34	3.12	3.96	NIPA1	non imprinted in Prader-Willi/Angelman syndrome 1	chr15	protein_coding
ENSG00000133401.14	1.6270721	1.81220509	8.97E-07	5.20E-06	0.1	0.14	0.46	0.5	PDZD2	PDZ domain containing 2	chr5	protein_coding
ENSG00000174953.12	2.06156444	2.64052521	1.31E-10	1.29E-09	0.26	0.31	1.24	1.95	DHX36	DEAH (Asp-Glu-Ala-His) box polypeptide 36	chr3	protein_coding
ENSG00000107890.15	4.15422619	0.67055215	1.28E-18	2.76E-17	0	0.03	0.16	0.71	ANKRD26	ankyrin repeat domain 26	chr10	protein_coding
ENSG00000214357.7	-1.6857206	5.2342484	1.04E-08	7.97E-08	7.05	7.42	3.33	2.65	NEURL1B	neuralized E3 ubiquitin protein ligase 1B	chr5	protein_coding
ENSG00000261187.1	1.99868488	-2.0292992	0.01397446	0.03477946	0.07	0.02	0.17	0.36	RP11-1007O	--	chr15	lincRNA
ENSG00000166682.9	-1.7082747	-1.3205192	0.00202074	0.0061882	0.22	0.16	0.08	0.07	TMPRSS5	transmembrane protease, serine 5	chr11	protein_coding
ENSG00000259644.1	4.87917439	-3.0678699	0.01889873	0.04519557	0	0	0	0.16	RP11-680F8.	--	chr15	sense_intronic
ENSG00000226332.2	-1.6333767	3.36727296	7.44E-08	5.08E-07	18.89	18.14	7.71	8.2	RP11-157P1.	--	chr20	antisense
ENSG00000176454.12	-1.4781755	4.52200164	5.61E-07	3.37E-06	12.2	11.98	6.19	5.36	LPCAT4	lysophosphatidylcholine acyltransferase 4	chr15	protein_coding
ENSG00000165240.16	1.34821297	1.32657209	7.72E-05	0.00031861	0.12	0.14	0.26	0.62	ATP7A	ATPase, Cu++ transporting, alpha polypeptide	chrX	protein_coding
ENSG00000169255.12	2.81977693	0.35988866	7.64E-11	7.77E-10	0.03	0.09	0.23	0.91	B3GALNT1	beta-1,3-N-acetylgalactosaminyltransferase 1 (globoside blood group)	chr3	protein_coding
ENSG00000261351.2	-3.0019582	0.50722823	3.50E-13	4.59E-12	0.83	1.05	0.09	0.21	CTD-3185P2	--	chr15	antisense
ENSG00000204348.8	-1.6551901	3.90683535	3.36E-08	2.39E-07	8.92	10.75	3.93	4.39	DXO	decapping exoribonuclease	chr6	protein_coding
ENSG00000168140.4	-2.2563578	8.55187216	3.21E-14	4.72E-13	182.39	175.72	50.07	49.8	VASN	vasorin	chr16	protein_coding
ENSG00000171960.9	-1.1100701	6.01040035	0.00012179	0.00048122	73.06	70.24	42.59	45.95	PPIH	peptidylprolyl isomerase H (cyclophilin H)	chr1	protein_coding
ENSG00000138663.7	1.660151	1.8660442	5.08E-07	3.07E-06	0.48	0.57	1.22	3.23	COPS4	COP9 signalosome subunit 4	chr4	protein_coding
ENSG00000148942.13	3.78915183	-1.0297823	3.09E-08	2.20E-07	0.01	0	0.01	0.22	SLC5A12	solute carrier family 5 (sodium/monocarboxylate cotransporter), member 12	chr11	protein_coding
ENSG00000112237.11	1.08221748	1.96339905	0.00072199	0.00244975	0.51	0.42	0.84	1.81	CCNC	cyclin C	chr6	protein_coding
ENSG00000267745.1	-5.0187832	-3.1315465	0.01889873	0.04519557	0.15	0	0	0	RP11-686D2	--	chr17	processed_transcript
ENSG00000156510.12	-4.7780343	0.28595639	1.52E-20	3.81E-19	0.43	0.6	0.02	0.03	HKDC1	hexokinase domain containing 1	chr10	protein_coding
ENSG00000253948.1	-1.7111467	-0.8006757	0.00049952	0.00174477	1.83	1.64	0.84	0.57	RP11-410L1	--	chr8	lincRNA
ENSG00000257342.1	2.53373985	-2.3738362	0.00737015	0.01977584	0.08	0	0.14	0.51	RP11-571M6	--	chr12	antisense
ENSG00000100393.9	1.19040372	5.83740808	3.99E-05	0.00017425	2.42	3.47	8.71	9.17	EP300	E1A binding protein p300	chr22	protein_coding
ENSG00000116977.17	2.03600302	2.81186903	1.33E-10	1.31E-09	0.28	0.35	1.1	2.37	LGALS8	lectin, galactoside-binding, soluble, 8	chr1	protein_coding
ENSG00000177853.13	5.40838688	-0.8885113	4.83E-11	5.01E-10	0	0	0.04	0.18	ZNF518A	zinc finger protein 518A	chr10	protein_coding
ENSG00000169629.10	1.84987825	-1.0792577	0.00121058	0.00391053	0.01	0.04	0.1	0.12	RGPD8	RANBP2-like and GRIP domain containing 8	chr2	protein_coding
ENSG00000125821.10	-1.3248577	5.45762266	5.33E-06	2.72E-05	34.46	38.88	18.27	20.77	DTD1	D-tyrosyl-tRNA deacylase 1	chr20	protein_coding
ENSG00000198680.4	-2.0235677	4.51607241	1.77E-11	1.93E-10	14.85	14.11	5.14	4.34	TUSC1	tumor suppressor candidate 1	chr9	protein_coding
ENSG00000152078.8	2.96218896	0.47149966	5.74E-12	6.64E-11	0.03	0.04	0.26	0.46	TMEM56	transmembrane protein 56	chr1	protein_coding
ENSG00000111863.11	2.65040683	-1.901033	0.00119227	0.00385709	0.03	0.01	0.17	0.18	ADTRP	androgen-dependent TFPI-regulating protein	chr6	protein_coding
ENSG00000176244.6	8.46564965	-0.3896297	1.22E-16	2.23E-15	0	0	1.04	1.13	ACBD7	acyl-CoA binding domain containing 7	chr10	protein_coding
ENSG00000075399.11	1.50924586	4.39852041	3.65E-07	2.26E-06	2.65	3.21	10.85	11.4	VPS9D1	VPS9 domain containing 1	chr16	protein_coding
ENSG00000179388.8	5.21752153	-2.8860363	0.00538165	0.01488851	0	0	0.04	0.02	EGR3	early growth response 3	chr8	protein_coding
ENSG00000092758.14	-1.5700491	2.67536831	4.20E-07	2.58E-06	2.98	3.23	1.39	1.39	COL9A3	collagen, type IX, alpha 3	chr20	protein_coding
ENSG00000106031.7	1.3539761	1.10338434	0.00011274	0.00044836	0.17	0.2	0.48	0.8	HOXA13	homeobox A13	chr7	protein_coding
ENSG00000251603.1	1.56947742	-1.5349331	0.01254665	0.03155686	0.35	0.37	0.42	2.42	RP11-164P1	--	chr4	antisense
ENSG00000151067.19	11.010214	2.00733245	4.64E-45	4.79E-43	0	0	0.52	0.44	CACNA1C	calcium channel, voltage-dependent, L type, alpha 1C subunit	chr12	protein_coding
ENSG00000140090.16	2.48960606	-2.4022627	0.00737015	0.01977584	0	0	0.04	0.01	SLC24A4	solute carrier family 24 (sodium/potassium/calcium exchanger), member 4	chr14	protein_coding
ENSG00000157778.7	-1.6799436	7.07753294	8.91E-09	6.88E-08	85.41	84.66	33.05	37.79	PSMG3	proteasome (prosome, macropain) assembly chaperone 3	chr7	protein_coding
ENSG00000137216.17	-1.0364371	5.6443951	0.00033596	0.001214	15.42	17.01	10.86	10.18	TMEM63B	transmembrane protein 63B	chr6	protein_coding
ENSG00000167996.14	-1.0208328	13.7107022	0.00036793	0.00132135	9606.64	8368.24	5711.34	6109.36	FTH1	ferritin, heavy polypeptide 1	chr11	protein_coding
ENSG00000280198.1	3.02097172	-2.0587142	0.0014675	0.00467247	0	0.08	0.64	0.31	RP11-384M2	--	chr17	TEC
ENSG00000135090.12	2.49279201	3.81985316	1.38E-15	2.32E-14	0.55	0.68	3.53	5.77	TAOK3	TAO kinase 3	chr12	protein_coding
ENSG00000147852.14	-1.905664	3.02443044	7.16E-10	6.36E-09	0.97	1.3	0.29	0.51	VLDLR	very low density lipoprotein receptor	chr9	protein_coding
ENSG00000136636.11	1.37471853	3.41747801	5.19E-06	2.66E-05	1.1	1.33	2.49	5.98	KCTD3	potassium channel tetramerization domain containing 3	chr1	protein_coding
ENSG00000167646.12	2.62341577	2.33550903	7.06E-15	1.12E-13	0.51	0.43	3.65	4.06	DNAAF3	dynein, axonemal, assembly factor 3	chr19	protein_coding
ENSG00000246876.5	-4.806998	-3.2310942	0.01889873	0.04519557	0.04	0.04	0	0	RP11-519M1	--	chr4	lincRNA
ENSG00000135299.15	4.63720635	0.76104824	4.85E-21	1.27E-19	0.03	0	0.46	0.6	ANKRD6	ankyrin repeat domain 6	chr6	protein_coding
ENSG00000204060.5	-2.4088157	2.64202054	1.14E-13	1.57E-12	3.27	3.82	1.16	0.61	FOXO6	forkhead box O6	chr1	protein_coding
ENSG00000185532.13	-7.490152	-1.4195541	1.18E-10	1.17E-09	0.13	0.02	0	0	PRKG1	protein kinase, cGMP-dependent, type I	chr10	protein_coding
ENSG00000178053.16	1.82398948	1.17846428	2.58E-07	1.63E-06	0.28	0.28	0.89	1.75	MLF1	myeloid leukemia factor 1	chr3	protein_coding
ENSG00000102349.13	7.63351575	1.1272865	5.69E-32	2.89E-30	0.01	0	0.97	0.78	KLF8	Kruppel-like factor 8	chrX	protein_coding
ENSG00000169180.10	-1.4639556	6.67832467	4.74E-07	2.88E-06	26.55	26.66	12.96	12.73	XPO6	exportin 6	chr16	protein_coding
ENSG00000140416.18	-1.9130288	7.44533007	7.77E-11	7.88E-10	26.44	34.47	9.55	12.04	TPM1	tropomyosin 1 (alpha)	chr15	protein_coding
ENSG00000134531.8	1.06036926	6.64049441	0.00023217	0.00086787	7.7	8.08	19.88	24.06	EMP1	epithelial membrane protein 1	chr12	protein_coding
ENSG00000163050.15	-1.0092216	5.45971424	0.00048228	0.00168754	9.27	7.59	5.23	5.96	ADCK3	aarF domain containing kinase 3	chr1	protein_coding
ENSG00000183914.13	5.09636367	2.22585404	4.11E-34	2.30E-32	0.01	0.02	0.69	0.5	DNAH2	dynein, axonemal, heavy chain 2	chr17	protein_coding
ENSG00000011405.12	4.13607741	1.73277963	1.37E-24	4.59E-23	0.02	0.05	0.55	1.04	PIK3C2A	phosphatidylinositol-4-phosphate 3-kinase, catalytic subunit type 2 alpha	chr11	protein_coding
ENSG00000124596.15	-1.568583	3.56648127	1.82E-07	1.18E-06	3.4	3.85	1.33	1.94	OARD1	O-acyl-ADP-ribose deacylase 1	chr6	protein_coding
ENSG00000279608.1	-2.1195631	-2.0806693	0.00408095	0.01167544	0.05	0.03	0	0.02	RP11-182N2	--	chr9	TEC
ENSG00000055208.16	1.11973774	2.88618255	0.00025005	0.0009305	0.68	0.95	1.83	2.9	TAB2	TGF-beta activated kinase 1/MAP3K7 binding protein 2	chr6	protein_coding
ENSG00000187837.3	2.33335038	6.77390317	6.06E-15	9.65E-14	45.78	45.51	315.39	297.17	HIST1H1C	histone cluster 1, H1c	chr6	protein_coding
ENSG00000233593.5	3.33546502	1.37024783	9.68E-18	1.95E-16	0.29	0.58	5.92	5.82	RP4-665J23.	--	chr1	lincRNA
ENSG00000099219.12	-1.9530154	4.20048502	9.23E-11	9.28E-10	4.32	4.48	1.37	1.66	ERMP1	endoplasmic reticulum metallopeptidase 1	chr9	protein_coding
ENSG00000234857.2	-1.078961	8.8279315	0.00017177	0.000656	117.64	132.15	77.41	80.1	HNRNPUL2-	HNRNPUL2-BSCL2 readthrough (NMD candidate)	chr11	protein_coding
ENSG00000145248.6	2.0875311	-1.973552	0.00956893	0.02497848	0.03	0.03	0.21	0.15	SLC10A4	solute carrier family 10, member 4	chr4	protein_coding
ENSG00000227467.3	-9.8049841	0.75804973	4.70E-31	2.28E-29	1.24	0.98	0	0	LINC01537	long intergenic non-protein coding RNA 1537	chr11	lincRNA
ENSG00000188211.7	1.20726233	2.43237305	0.00010611	0.00042542	0.3	0.51	1.27	1.21	NCR3LG1	natural killer cell cytotoxicity receptor 3 ligand 1	chr11	protein_coding
ENSG00000167635.10	2.04317825	2.8887883	9.92E-11	9.94E-10	0.48	0.72	2.51	4.09	ZNF146	zinc finger protein 146	chr19	protein_coding
ENSG00000006715.14	1.46394013	2.73054243	2.39E-06	1.30E-05	0.39	0.57	1.36	2.17	VPS41	vacuolar protein sorting 41 homolog (S. cerevisiae)	chr7	protein_coding
ENSG00000198836.7	2.03062024	2.68559598	1.77E-10	1.70E-09	0.28	0.34	1.33	2.05	OPA1	optic atrophy 1 (autosomal dominant)	chr3	protein_coding
ENSG00000165548.9	-2.2923739	1.82200592	1.72E-11	1.88E-10	1.07	0.7	0.29	0.19	TMEM63C	transmembrane protein 63C	chr14	protein_coding
ENSG00000100300.16	-1.3481979	5.80111124	3.54E-06	1.87E-05	60.18	50.39	30.42	27.42	TSPO	translocator protein (18kDa)	chr22	protein_coding
ENSG00000088899.13	2.07175871	3.41728731	2.34E-11	2.51E-10	0.61	0.61	3.32	3.51	LZTS3	--	chr20	protein_coding
ENSG00000170515.12	-1.0075533	7.6622434	0.00044869	0.00158246	81.97	85.06	54.28	56.44	PA2G4	proliferation-associated 2G4, 38kDa	chr12	protein_coding
ENSG00000120963.10	-1.3223451	3.47603856	9.82E-06	4.77E-05	1.84	2.18	0.92	1.22	ZNF706	zinc finger protein 706	chr8	protein_coding
ENSG00000184840.10	-1.0533217	8.67284744	0.00024307	0.00090647	315.1	290.13	201.92	186.35	TMED9	transmembrane emp24 protein transport domain containing 9	chr5	protein_coding
ENSG00000129159.6	-5.7759286	-2.7103808	0.00047761	0.00167191	0.04	0	0	0	KCNC1	potassium channel, voltage gated Shaw related subfamily C, member 1	chr11	protein_coding
ENSG00000273345.4	3.02042161	-1.635083	0.00010281	0.00041304	0.07	0	0.21	0.65	CTD-2410N1	--	chr5	processed_transcript
ENSG00000159214.11	1.62902208	2.03600278	5.11E-07	3.09E-06	1.02	1.14	4.88	4.04	CCDC24	coiled-coil domain containing 24	chr1	protein_coding
ENSG00000126261.11	1.21855536	4.97731417	2.97E-05	0.00013192	2.83	4.09	8.2	13.35	UBA2	ubiquitin-like modifier activating enzyme 2	chr19	protein_coding
ENSG00000213121.2	5.56116133	-2.6812008	0.00086564	0.00288128	0	0	0.05	0.25	RP11-306O1	--	chr6	lincRNA
ENSG00000262691.1	1.62774902	0.76506745	1.00E-05	4.85E-05	0.15	0.33	0.74	1.22	CTC-277H1.	--	chr16	processed_transcript
ENSG00000188060.6	-1.6548332	2.65669997	1.18E-07	7.86E-07	4.33	3.58	2	1.34	RAB42	RAB42, member RAS oncogene family	chr1	protein_coding
ENSG00000156587.14	3.7693325	4.7827407	9.59E-31	4.57E-29	1.64	1.74	31.98	29.28	UBE2L6	ubiquitin-conjugating enzyme E2L 6	chr11	protein_coding
ENSG00000071242.10	-1.0302478	3.80622502	0.00049656	0.00173472	2.11	2.47	1.52	1.46	RPS6KA2	ribosomal protein S6 kinase, 90kDa, polypeptide 2	chr6	protein_coding
ENSG00000150687.10	-2.6662962	7.27395003	1.19E-18	2.59E-17	35.89	37.19	6.49	8.91	PRSS23	protease, serine, 23	chr11	protein_coding
ENSG00000149136.6	-1.2899883	10.153919	7.69E-06	3.83E-05	427.67	502.01	243.98	262.64	SSRP1	structure specific recognition protein 1	chr11	protein_coding
ENSG00000128573.21	-2.3470274	-2.7897881	0.01615662	0.03958166	0	0.03	0	0.01	FOXP2	forkhead box P2	chr7	protein_coding
ENSG00000132688.10	-2.7503965	5.83420613	1.79E-19	4.15E-18	15.81	13.11	2.61	3.13	NES	nestin	chr1	protein_coding
ENSG00000186020.11	2.56064704	-0.8383601	4.87E-06	2.51E-05	0.02	0.03	0.23	0.11	ZNF529	zinc finger protein 529	chr19	protein_coding
ENSG00000197291.7	2.2249715	-1.4325423	0.00043455	0.00153757	0.05	0	0.16	0.17	RAMP2-AS1	RAMP2 antisense RNA 1	chr17	lincRNA
ENSG00000249306.4	-3.6855814	-2.5707588	0.00190233	0.00584723	0.35	0.07	0.04	0	LINC01411	long intergenic non-protein coding RNA 1411	chr5	lincRNA
ENSG00000003989.15	-4.5986332	3.62364611	2.59E-38	1.79E-36	2.69	2.17	0.09	0.18	SLC7A2	solute carrier family 7 (cationic amino acid transporter, y+ system), member 2	chr8	protein_coding
ENSG00000100271.15	1.70816263	2.58100219	6.30E-08	4.36E-07	1.28	1.43	5.61	6.22	TTLL1	tubulin tyrosine ligase-like family member 1	chr22	protein_coding
ENSG00000198754.5	1.73414898	3.83159711	9.06E-09	6.99E-08	3.19	2.59	13.09	12.59	OXCT2	3-oxoacid CoA transferase 2	chr1	protein_coding
ENSG00000169291.8	6.00451321	2.04670112	2.17E-37	1.43E-35	0.01	0.02	1.25	1.48	SHE	Src homology 2 domain containing E	chr1	protein_coding
ENSG00000128739.19	-10.327654	5.22619878	1.02E-91	1.23E-88	23.8	21.8	0.02	0.02	SNRPN	small nuclear ribonucleoprotein polypeptide N	chr15	protein_coding
ENSG00000185088.11	1.72908862	4.69644502	6.14E-09	4.86E-08	1.31	1.13	4.83	5.98	RPS27L	ribosomal protein S27-like	chr15	protein_coding
ENSG00000119943.9	-2.137463	3.77969503	3.09E-12	3.67E-11	10.74	7.08	2.51	2.9	PYROXD2	pyridine nucleotide-disulphide oxidoreductase domain 2	chr10	protein_coding
ENSG00000204642.12	2.21853795	4.03131014	4.78E-13	6.20E-12	2.41	2.27	13.94	15.15	HLA-F	major histocompatibility complex, class I, F	chr6	protein_coding
ENSG00000262074.5	3.83416221	0.04140675	1.36E-13	1.86E-12	0.17	0.07	2.63	2.13	SNORD3B-2	small nucleolar RNA, C/D box 3B-2	chr17	snoRNA
ENSG00000171067.9	-1.2079701	6.48766462	2.92E-05	0.00013027	42.33	46.88	26.55	24.82	C11orf24	chromosome 11 open reading frame 24	chr11	protein_coding
ENSG00000254966.1	4.8843127	-3.0650858	0.01889873	0.04519557	0	0	0.04	0.22	RP11-1081L1	--	chr11	antisense
ENSG00000168016.12	2.35928282	3.10246387	1.05E-13	1.46E-12	0.26	0.17	1.31	1.61	TRANK1	tetratricopeptide repeat and ankyrin repeat containing 1	chr3	protein_coding
ENSG00000267905.4	2.04639783	-1.7559342	0.00471212	0.01331279	0.06	0.26	0.59	1.18	CTD-2616J1	--	chr19	antisense
ENSG00000225077.2	-1.5403399	0.88423654	1.51E-05	7.12E-05	1.85	1.5	0.93	0.61	LINC00337	long intergenic non-protein coding RNA 337	chr1	lincRNA
ENSG00000277268.1	7.57091627	-1.1783506	1.38E-11	1.53E-10	0	0	0.35	0.26	RP11-445F1	--	chr17	antisense
ENSG00000269210.2	3.77434626	2.12669447	5.09E-24	1.64E-22	0.68	0.19	8.19	7.99	RP11-173C1	--	chr2	antisense
ENSG00000049246.13	1.06278777	2.23866324	0.00073952	0.00250464	0.31	0.44	1.03	1.05	PER3	period circadian clock 3	chr1	protein_coding
ENSG00000244509.3	8.02352487	4.98531951	2.09E-75	1.11E-72	0.09	0.05	24.23	23.86	APOBEC3C	apolipoprotein B mRNA editing enzyme, catalytic polypeptide-like 3C	chr22	protein_coding
ENSG00000236772.1	3.22798969	-2.5371562	0.003237	0.00945156	0	0.03	0.23	0.19	RP5-1184F4.	--	chr20	antisense
ENSG00000134882.14	-1.6497795	6.18111742	1.72E-08	1.28E-07	32.33	29.29	12.92	13.26	UBAC2	UBA domain containing 2	chr13	protein_coding
ENSG00000008311.13	7.40126841	-1.3227675	1.18E-10	1.17E-09	0	0	0.26	0.15	AASS	aminoadipate-semialdehyde synthase	chr7	protein_coding
ENSG00000134323.10	7.34239909	-1.3664804	2.87E-10	2.70E-09	0	0	0.22	0.28	MYCN	v-myc avian myelocytomatosis viral oncogene neuroblastoma derived homolog	chr2	protein_coding
ENSG00000214753.2	-1.1684952	7.09840817	5.06E-05	0.00021569	29.64	30.51	17.72	17.94	HNRNPUL2	heterogeneous nuclear ribonucleoprotein U-like 2	chr11	protein_coding
ENSG00000204604.8	3.31451201	-0.2227691	1.68E-10	1.63E-09	0.02	0.02	0.22	0.43	ZNF468	zinc finger protein 468	chr19	protein_coding
ENSG00000257732.1	-2.8988284	0.45174155	3.34E-12	3.96E-11	2.94	2.55	0.59	0.39	RP11-818F2	--	chr12	antisense
ENSG00000198089.13	1.46332334	5.43663434	5.62E-07	3.37E-06	3.96	4.38	15.01	15.62	SFI1	Sfi1 homolog, spindle assembly associated (yeast)	chr22	protein_coding
ENSG00000156869.11	1.72103706	-0.4217863	0.00025043	0.00093174	0.03	0.16	0.23	0.59	FRRS1	ferric-chelate reductase 1	chr1	protein_coding
ENSG00000186318.15	-1.6056255	5.18848131	4.64E-08	3.25E-07	6.36	7.39	2.95	3.07	BACE1	beta-site APP-cleaving enzyme 1	chr11	protein_coding

ENSG00000197299.9	2.00472498	1.48647078	9.27E-09	7.14E-08	0.12	0.22	0.58	1.2	BLM	Bloom syndrome, RecQ helicase-like	chr15	protein_coding
ENSG00000175595.13	2.3430536	1.37434557	7.71E-11	7.82E-10	0.07	0.1	0.56	0.6	ERCC4	excision repair cross-complementation group 4	chr16	protein_coding
ENSG00000185761.9	-1.6119674	3.06054378	1.44E-07	9.43E-07	3.31	3.8	1.64	1.46	ADAMTSL5	ADAMTS-like 5	chr19	protein_coding
ENSG00000100292.15	2.1756415	6.71637688	2.54E-13	3.38E-12	17.66	16.16	102.48	101.15	HMOX1	heme oxygenase 1	chr22	protein_coding
ENSG00000136869.13	1.2537642	0.27697831	0.00123508	0.00398431	0.05	0.16	0.22	0.47	TLR4	toll-like receptor 4	chr9	protein_coding
ENSG00000149798.4	-1.6969394	5.17634357	8.50E-09	6.58E-08	22.5	17.67	8.74	7.76	CDC42EP2	CDC42 effector protein (Rho GTPase binding) 2	chr11	protein_coding
ENSG00000210100.1	2.72709853	2.44470992	5.20E-16	9.05E-15	13.57	19.3	155.98	133.4	MT-TI	mitochondrially encoded tRNA isoleucine	chrM	Mt_tRNA
ENSG00000112276.12	1.39623614	0.18426148	0.00061313	0.00210994	0.08	0.08	0.18	0.38	BVES	blood vessel epicardial substance	chr6	protein_coding
ENSG00000181827.13	1.5367727	1.29177526	8.23E-06	4.06E-05	0.1	0.09	0.29	0.44	RFX7	regulatory factor X, 7	chr15	protein_coding
ENSG00000130751.8	-1.149566	1.48072477	0.00054072	0.0018794	1.58	1.12	0.93	0.7	NPAS1	neuronal PAS domain protein 1	chr19	protein_coding
ENSG00000147604.12	-1.4785605	7.64951366	3.49E-07	2.17E-06	103.49	99.39	46.96	50.13	RPL7	ribosomal protein L7	chr8	protein_coding
ENSG00000112183.13	-3.4070019	-2.1660142	5.06E-05	0.00021569	0.05	0.13	0	0.02	RBM24	RNA binding motif protein 24	chr6	protein_coding
ENSG00000121297.6	5.06713725	-2.9688687	0.0100455	0.02582742	0	0	0.03	0.02	TSHZ3	teashirt zinc finger homeobox 3	chr19	protein_coding
ENSG00000004139.12	3.26875879	4.8103081	8.69E-25	2.96E-23	0.43	0.4	5.84	4.72	SARM1	sterile alpha and TIR motif containing 1	chr17	protein_coding
ENSG00000269473.1	-1.3962748	1.56348337	2.50E-05	0.00011271	0.58	0.69	0.39	0.25	CTD-2619J1	--	chr19	lincRNA
ENSG00000167207.10	-3.8603597	-1.0384257	5.13E-09	4.09E-08	0.17	0.13	0.03	0	NOD2	nucleotide-binding oligomerization domain containing 2	chr16	protein_coding
ENSG00000167103.10	-2.3371255	1.5954495	2.06E-11	2.22E-10	1.81	1.62	0.71	0.21	PIP5KL1	phosphatidylinositol-4-phosphate 5-kinase-like 1	chr9	protein_coding
ENSG00000123407.3	5.4903065	2.50269245	1.70E-38	1.20E-36	0.08	0.03	3.8	3.2	HOXC12	homeobox C12	chr12	protein_coding
ENSG00000204681.9	-1.3062927	1.95970195	4.62E-05	0.00019969	0.72	0.86	0.42	0.43	GABBR1	gamma-aminobutyric acid (GABA) B receptor, 1	chr6	protein_coding
ENSG00000178395.6	6.21221381	-2.25038	1.55E-05	7.26E-05	0	0	0.22	0.08	CCDC185	coiled-coil domain containing 185	chr1	protein_coding
ENSG00000204287.12	7.60097577	-1.1588232	9.11E-12	1.03E-10	0	0	0.95	0.3	HLA-DRA	major histocompatibility complex, class II, DR alpha	chr6	protein_coding
ENSG00000116127.16	2.17983537	1.57212777	3.61E-10	3.36E-09	0.06	0.07	0.44	0.34	ALMS1	Alstrom syndrome protein 1	chr2	protein_coding
ENSG00000111670.13	2.36417217	2.08770475	2.70E-12	3.23E-11	0.16	0.18	0.88	1.48	GNPTAB	N-acetylglucosamine-1-phosphate transferase, alpha and beta subunits	chr12	protein_coding
ENSG00000079819.15	-2.1621833	4.69988308	7.77E-13	9.84E-12	4.1	5.2	1.29	1.48	EPB41L2	erythrocyte membrane protein band 4.1-like 2	chr6	protein_coding
ENSG00000273445.1	5.58503228	-2.6679206	0.00086564	0.00288128	0	0	0.42	0.3	RP11-1399P	--	chr2	antisense
ENSG00000119326.13	1.32472388	2.34131633	2.60E-05	0.00011718	0.54	1.13	1.99	3.6	CTNNAL1	catenin (cadherin-associated protein), alpha-like 1	chr9	protein_coding
ENSG00000154429.9	2.39762445	1.16251301	1.01E-10	1.01E-09	0.13	0.07	0.55	0.9	CCSAP	centriole, cilia and spindle-associated protein	chr1	protein_coding
ENSG00000144224.15	2.65245578	4.08996781	2.28E-17	4.48E-16	0.76	1.12	6.09	9.71	UBXN4	UBX domain protein 4	chr2	protein_coding
ENSG00000117758.12	1.53870303	2.51916051	9.55E-07	5.52E-06	0.85	0.66	2.55	3.34	STX12	syntaxin 12	chr1	protein_coding
ENSG00000116754.12	1.96991624	4.93253655	4.64E-11	4.83E-10	1.54	2.88	8.35	14.82	SRSF11	serine/arginine-rich splicing factor 11	chr1	protein_coding
ENSG00000256188.2	4.89980841	-3.0567113	0.01889873	0.04519557	0	0	0.07	0.06	TAS2R30	taste receptor, type 2, member 30	chr12	protein_coding
ENSG00000145284.10	1.44553265	3.63096373	1.53E-06	8.53E-06	1.19	1.3	3.74	5.34	SCD5	stearoyl-CoA desaturase 5	chr4	protein_coding
ENSG00000187957.7	-6.5257283	4.32306974	3.06E-60	7.40E-58	10.03	9.66	0.18	0.11	DNER	delta/notch-like EGF repeat containing	chr2	protein_coding
ENSG00000259658.3	8.00331167	-0.7974545	5.19E-13	6.70E-12	0	0	1.23	2.09	RP11-89K11.	--	chr15	lincRNA
ENSG00000228106.4	-1.1033749	1.79669255	0.00056944	0.00197235	5.75	4.13	2.63	3.51	RP11-452F1	--	chr1	lincRNA
ENSG00000228436.2	5.89627893	-0.4728275	1.36E-14	2.08E-13	0.03	0	1.25	1.48	RP5-864K19.	--	chr1	antisense
ENSG00000120693.12	4.91074829	0.29055222	3.48E-18	7.25E-17	0	0.02	0.26	0.57	SMAD9	SMAD family member 9	chr13	protein_coding
ENSG00000119707.12	1.25673603	5.7704297	1.50E-05	7.09E-05	2.25	3.96	7.87	11.95	RBM25	RNA binding motif protein 25	chr14	protein_coding
ENSG00000164162.11	1.99944585	0.8476084	1.06E-07	7.12E-07	0.29	0.28	1.35	1.68	ANAPC10	anaphase promoting complex subunit 10	chr4	protein_coding
ENSG00000078081.6	-1.7803096	0.80090651	7.65E-07	4.48E-06	0.45	0.8	0.14	0.34	LAMP3	lysosomal-associated membrane protein 3	chr3	protein_coding
ENSG00000077549.16	-1.2840562	8.11446803	8.64E-06	4.25E-05	114.42	118.32	65.41	61.79	CAPZB	capping protein (actin filament) muscle Z-line, beta	chr1	protein_coding
ENSG00000270765.4	6.94694098	-1.6903772	3.05E-08	2.18E-07	0	0	0.16	0.17	GAS2L2	growth arrest-specific 2 like 2	chr17	protein_coding
ENSG00000229686.1	1.21293782	-0.5749705	0.00963638	0.02512271	2.26	5.99	9.93	15.34	SNORD56	small nucleolar RNA, C/D box 56	chr20	snoRNA
ENSG00000178401.13	1.960521	1.61410852	9.16E-09	7.06E-08	0.2	0.24	0.99	1.31	DNAJC22	DnaJ (Hsp40) homolog, subfamily C, member 22	chr12	protein_coding
ENSG00000164930.10	2.39290154	2.49087855	3.49E-13	4.59E-12	0.25	0.48	1.62	3.5	FZD6	frizzled class receptor 6	chr8	protein_coding
ENSG00000129810.13	1.52487823	1.00922707	1.95E-05	8.95E-05	0.14	0.28	0.6	1.03	SGOL1	shugoshin-like 1 (S. pombe)	chr3	protein_coding
ENSG00000270412.1	-6.3557055	-2.3223985	1.55E-05	7.26E-05	0.67	0.45	0	0	RP11-92C4.6	--	chr9	antisense
ENSG00000129636.11	1.35011855	4.19544407	5.13E-06	2.63E-05	2.42	2.47	6.57	10.13	ITFG1	integrin alpha FG-GAP repeat containing 1	chr16	protein_coding
ENSG00000150527.15	1.94582597	-1.6209306	0.00345484	0.01003143	0.02	0.01	0.04	0.08	CTAGE5	CTAGE family, member 5	chr14	protein_coding
ENSG00000114315.3	1.396366	5.13057716	1.82E-06	1.00E-05	10.49	9.23	32.72	36.6	HES1	hes family bHLH transcription factor 1	chr3	protein_coding
ENSG00000115641.17	-1.2040932	5.59746053	3.31E-05	0.00014625	8.63	9.14	5.04	5.24	FHL2	four and a half LIM domains 2	chr2	protein_coding
ENSG00000154654.13	-6.1911323	-2.4387921	4.74E-05	0.00020301	0.02	0.03	0	0	NCAM2	neural cell adhesion molecule 2	chr21	protein_coding
ENSG00000261832.3	-1.0547245	4.61822228	0.00029788	0.00108589	17.17	14.12	10.38	9.69	CLN3	ceroid-lipofuscinosis, neuronal 3	chr16	protein_coding
ENSG00000126562.15	1.7657735	-1.1405955	0.0021156	0.00646136	0.03	0.04	0.17	0.15	WNK4	WNK lysine deficient protein kinase 4	chr17	protein_coding
ENSG00000280422.1	5.33927207	-2.8158158	0.00290522	0.00856312	0	0	0.06	0.09	RP11-155D1	--	chr3	TEC
ENSG00000108064.9	2.51631763	2.44187163	3.51E-14	5.12E-13	0.26	0.21	1.12	2.51	TFAM	transcription factor A, mitochondrial	chr10	protein_coding
ENSG00000152520.12	2.64484618	1.47383233	3.05E-13	4.03E-12	0.08	0.14	0.7	1.07	PAN3	PAN3 poly(A) specific ribonuclease subunit	chr13	protein_coding
ENSG00000261821.2	2.05987578	1.50810326	3.67E-09	3.00E-08	0.1	0.25	0.85	1.08	CTD-2311M	--	chr15	antisense
ENSG00000278023.3	3.68904994	1.01437005	3.49E-18	7.25E-17	0.18	0.04	1.51	2.37	RDM1	RAD52 motif containing 1	chr17	protein_coding
ENSG00000225855.5	1.50511077	0.66973795	4.34E-05	0.00018836	0.14	0.23	0.92	0.45	RUSC1-AS1	RUSC1 antisense RNA 1	chr1	antisense
ENSG00000112297.13	3.34578591	2.70530975	4.01E-22	1.13E-20	0.08	0.17	1.56	1.78	AIM1	absent in melanoma 1	chr6	protein_coding
ENSG00000161860.7	1.21066345	3.03920677	6.85E-05	0.0002851	3.48	2.97	11.16	8.69	SYCE2	synaptonemal complex central element protein 2	chr19	protein_coding
ENSG00000270164.1	6.79728773	-1.8055231	1.35E-07	8.87E-07	0	0	0.17	0.35	LINC01480	long intergenic non-protein coding RNA 1480	chr19	lincRNA
ENSG00000037280.14	6.95783683	-1.683718	3.05E-08	2.18E-07	0	0	0.09	0.07	FLT4	fms-related tyrosine kinase 4	chr5	protein_coding
ENSG00000197063.9	-1.0865882	6.51650955	0.00016337	0.00062613	18.12	20.51	12.09	12.13	MAFG	v-maf avian musculoaponeurotic fibrosarcoma oncogene homolog G	chr17	protein_coding
ENSG00000144468.15	-1.2860064	2.26146269	3.87E-05	0.00016922	1.18	0.83	0.43	0.67	RHBDD1	rhomboid domain containing 1	chr2	protein_coding
ENSG00000172179.10	6.43882307	-2.0805858	3.03E-06	1.62E-05	0	0	0.19	0.32	PRL	prolactin	chr6	protein_coding
ENSG00000100580.7	1.50889913	2.18089506	2.32E-06	1.27E-05	0.27	0.2	0.81	1.01	TMED8	transmembrane emp24 protein transport domain containing 8	chr14	protein_coding
ENSG00000100298.14	1.06580501	-0.2988624	0.01306847	0.03273955	0.26	0.42	1.26	0.64	APOBEC3H	apolipoprotein B mRNA editing enzyme, catalytic polypeptide-like 3H	chr22	protein_coding
ENSG00000258904.1	2.89056809	-2.7350104	0.01615662	0.03958166	0.05	0	0.36	0.26	RP11-638I2.	--	chr14	sense_intronic
ENSG00000118473.20	-5.9972113	-2.568945	0.00014842	0.00057423	0.03	0.01	0	0	SGIP1	SH3-domain GRB2-like (endophilin) interacting protein 1	chr1	protein_coding
ENSG00000135164.17	2.64104272	1.73624788	1.09E-13	1.51E-12	0.11	0.23	1.09	1.75	DMTF1	cyclin D binding myb-like transcription factor 1	chr7	protein_coding
ENSG00000164924.16	-1.0444175	6.53181674	0.00028567	0.00104424	15.32	17.03	7.56	13.48	YWHAZ	tyrosine 3-monooxygenase/tryptophan 5-monooxygenase activation protein, zeta	chr8	protein_coding
ENSG00000259380.4	7.08615643	-1.574531	7.18E-09	5.62E-08	0	0	0.14	0.3	RP11-346D1	--	chr15	lincRNA
ENSG00000056586.14	1.16944891	3.27972106	0.00010253	0.00041213	0.4	0.44	1.1	1.42	RC3H2	ring finger and CCCH-type domains 2	chr9	protein_coding
ENSG00000273001.1	5.6781718	-2.6083292	0.00047761	0.00167191	0	0	0.28	0.42	RP11-118K6.	--	chr10	lincRNA
ENSG00000117395.9	-1.6760261	8.20635268	9.10E-09	7.02E-08	187.63	262.27	82.63	105.23	EBNA1BP2	EBNA1 binding protein 2	chr1	protein_coding
ENSG00000269113.3	-5.6779546	4.90553584	1.28E-54	2.37E-52	6.43	7.13	0.15	0.21	TRABD2B	TraB domain containing 2B	chr1	protein_coding
ENSG00000221983.6	-1.0080843	8.75366904	0.00043949	0.00155323	130.44	118.33	86.87	77.75	UBA52	ubiquitin A-52 residue ribosomal protein fusion product 1	chr19	protein_coding
ENSG00000155846.15	-1.2695521	4.01978215	1.74E-05	8.08E-05	1.37	1.64	0.77	0.89	PPARGC1B	peroxisome proliferator-activated receptor gamma, coactivator 1 beta	chr5	protein_coding
ENSG00000130813.16	1.30396296	5.30614583	7.70E-06	3.83E-05	7.52	7.45	25.8	23.37	C19orf66	chromosome 19 open reading frame 66	chr19	protein_coding
ENSG00000033867.15	1.96755044	1.30676418	2.06E-08	1.50E-07	0.11	0.08	0.57	0.41	SLC4A7	solute carrier family 4, sodium bicarbonate cotransporter, member 7	chr3	protein_coding
ENSG00000272574.1	3.77784864	-1.0548621	3.09E-08	2.20E-07	0.16	0	2.03	1.19	RP11-359K1	--	chr1	sense_intronic
ENSG00000230989.5	1.19019245	6.72238903	3.77E-05	0.00016473	5.51	6.35	16.84	19.26	HSBP1	heat shock factor binding protein 1	chr16	protein_coding
ENSG00000013364.17	-1.3788211	7.43133899	1.92E-06	1.06E-05	73.11	64.82	34.22	36.54	MVP	major vault protein	chr16	protein_coding
ENSG00000118515.10	2.57746526	6.30664699	1.54E-17	3.06E-16	2.96	2.83	20.07	26.15	SGK1	serum/glucocorticoid regulated kinase 1	chr6	protein_coding
ENSG00000116191.16	1.39235215	0.97489259	7.79E-05	0.00032117	0.14	0.08	0.26	0.51	RALGPS2	Ral GEF with PH domain and SH3 binding motif 2	chr1	protein_coding
ENSG00000010818.7	3.54438212	3.32940136	9.79E-26	3.56E-24	0.13	0.13	1.83	2.23	HIVEP2	human immunodeficiency virus type I enhancer binding protein 2	chr6	protein_coding
ENSG00000214309.4	1.27887397	2.07189495	6.08E-05	0.00025568	1.75	0.83	4.65	3.72	MBLAC1	metallo-beta-lactamase domain containing 1	chr7	protein_coding
ENSG00000186132.13	2.59259249	-0.809172	3.62E-06	1.91E-05	0.08	0.09	0.63	0.79	C2orf76	chromosome 2 open reading frame 76	chr2	protein_coding
ENSG00000280511.1	6.86722649	-1.7549514	8.18E-08	5.55E-07	0	0	0.82	0.69	RP11-35J1.2	--	chr6	lincRNA
ENSG00000100629.15	1.11787898	1.60113834	0.00073768	0.00249876	0.25	0.38	0.71	1.12	CEP128	centrosomal protein 128kDa	chr14	protein_coding
ENSG00000137501.15	4.46053937	-0.9540318	9.89E-10	8.68E-09	0	0.01	0.2	0.13	SYTL2	synaptotagmin-like 2	chr11	protein_coding
ENSG00000166200.13	2.83326423	1.42300981	1.92E-14	2.89E-13	0.09	0.06	0.25	1.21	COPS2	COP9 signalosome subunit 2	chr15	protein_coding
ENSG00000213626.10	-4.798664	3.12800329	2.70E-38	1.86E-36	4.22	3.39	0.12	0.24	LBH	limb bud and heart development	chr2	protein_coding
ENSG00000261068.2	-2.4593322	-1.4610357	8.64E-05	0.00035076	0.29	0.14	0.06	0.04	RP11-7K24.3	--	chr6	lincRNA
ENSG00000184232.7	-1.0188195	5.2015264	0.00043926	0.00155262	15.24	16.34	11.11	9.61	OAF	OAF homolog (Drosophila)	chr11	protein_coding
ENSG00000197822.9	3.28054872	-0.0981014	5.21E-11	5.39E-10	0.01	0.03	0.18	0.32	OCLN	occludin	chr5	protein_coding
ENSG00000164209.15	1.3478423	0.62613391	0.0003173	0.00115056	0.09	0.32	0.44	0.93	SLC25A46	solute carrier family 25, member 46	chr5	protein_coding
ENSG00000233483.2	1.93761475	-1.8292784	0.00967167	0.02519005	0.07	0.05	0.21	0.46	CTD-2020K1	--	chr17	antisense
ENSG00000173905.7	2.57212204	4.63103476	7.00E-17	1.31E-15	1.04	1.59	8.03	12.92	GOLIM4	golgi integral membrane protein 4	chr3	protein_coding
ENSG00000107771.14	1.74705964	0.35880375	1.30E-05	6.17E-05	0.03	0.08	0.18	0.32	CCSER2	coiled-coil serine-rich protein 2	chr10	protein_coding
ENSG00000185453.11	-1.2403441	7.54575455	1.72E-05	8.00E-05	51.87	66.77	34.31	32.45	C19orf68	chromosome 19 open reading frame 68	chr19	protein_coding
ENSG00000106018.12	-10.333298	1.2823103	1.56E-37	1.03E-35	0.82	0.69	0	0	VIPR2	vasoactive intestinal peptide receptor 2	chr7	protein_coding
ENSG00000137474.18	3.1400402	0.61480727	2.02E-13	2.72E-12	0.04	0.01	0.34	0.37	MYO7A	myosin VIIA	chr11	protein_coding
ENSG00000137845.13	2.06169476	3.49581803	2.44E-11	2.61E-10	0.28	0.25	1.1	1.88	ADAM10	ADAM metallopeptidase domain 10	chr15	protein_coding
ENSG00000078699.20	1.82833652	5.32535592	6.39E-10	5.72E-09	1.53	1.51	7.43	6.97	CBFA2T2	core-binding factor, runt domain, alpha subunit 2; translocated to, 2	chr20	protein_coding
ENSG00000182372.6	-1.0754304	2.83513602	0.00040285	0.00143345	1.21	0.98	0.61	0.78	CLN8	ceroid-lipofuscinosis, neuronal 8 (epilepsy, progressive with mental retardation)	chr8	protein_coding
ENSG00000053254.14	1.24299091	2.90504218	4.77E-05	0.00020428	0.39	0.49	1.18	1.6	FOXN3	forkhead box N3	chr14	protein_coding
ENSG00000163635.16	2.4170715	2.28379286	4.67E-13	6.05E-12	0.13	0.18	1.05	1.17	ATXN7	ataxin 7	chr3	protein_coding
ENSG00000067829.17	-1.8937892	6.94570694	1.22E-10	1.20E-09	87.74	84.49	30.63	31.14	IDH3G	isocitrate dehydrogenase 3 (NAD+) gamma	chrX	protein_coding
ENSG00000154736.5	-5.5223491	-2.8631056	0.00158008	0.00495521	0.02	0.01	0	0	ADAMTS5	ADAM metallopeptidase with thrombospondin type 1 motif, 5	chr21	protein_coding
ENSG00000109321.9	5.02095246	4.9515589	2.46E-46	2.78E-44	1.12	1.28	44.64	59.73	AREG	amphiregulin	chr4	protein_coding
ENSG00000271643.1	6.09399745	-0.2915798	3.49E-16	6.15E-15	0	0.02	0.86	1.55	RP11-10C24	--	chr3	lincRNA
ENSG00000171469.9	-4.4853518	-0.5224139	9.91E-15	1.55E-13	0.23	0.21	0	0.02	ZNF561	zinc finger protein 561	chr19	protein_coding
ENSG00000130741.9	-1.4015326	7.4272597	1.31E-06	7.41E-06	66.99	74.55	32.27	39.26	EIF2S3	eukaryotic translation initiation factor 2, subunit 3 gamma, 52kDa	chrX	protein_coding
ENSG00000091157.12	1.18834143	1.35438009	0.00040059	0.00142646	0.12	0.05	0.2	0.32	WDR7	WD repeat domain 7	chr18	protein_coding
ENSG00000116584.16	-1.9444839	7.00135901	4.14E-11	4.32E-10	35.32	34.76	12.28	11.97	ARHGEF2	Rho/Rac guanine nucleotide exchange factor (GEF) 2	chr1	protein_coding
ENSG00000171316.10	-1.4400037	3.18536787	2.02E-06	1.11E-05	0.74	0.92	0.33	0.49	CHD7	chromodomain helicase DNA binding protein 7	chr8	protein_coding
ENSG00000274267.1	1.97196619	-0.3208163	2.12E-05	9.67E-05	0.65	0.48	2.42	3.58	HIST1H3B	histone cluster 1, H3b	chr6	protein_coding
ENSG00000112029.8	1.38010806	1.84475934	2.30E-05	0.00010452	0.61	0.71	1.42	3.18	FBXO5	F-box protein 5	chr6	protein_coding
ENSG00000008952.15	2.63532164	4.27411128	2.45E-17	4.76E-16	0.6	0.72	4.08	6.87	SEC62	SEC62 homolog (S. cerevisiae)	chr3	protein_coding
ENSG00000133111.3	1.95027042	0.77912956	2.64E-07	1.66E-06	0.19	0.28	0.9	1.51	RFXAP	regulatory factor X-associated protein	chr13	protein_coding
ENSG00000144161.11	1.0340384	2.3839263	0.00092344	0.00305884	0.44	0.47	0.97	1.54	ZC3H8	zinc finger CCCH-type containing 8	chr2	protein_coding
ENSG00000227279.1	-4.8088799	-3.2302406	0.01889873	0.04519557	0.06	0.32	0	0	AC015933.2	--	chr18	antisense
ENSG00000147364.15	1.14725538	3.16410654	0.00014906	0.00057642	0.37	0.48	1.04	1.47	FBXO25	F-box protein 25	chr8	protein_coding
ENSG00000187566.4	2.83930181	-0.4697892	5.44E-08	3.78E-07	0.16	0.05	1	1.03	NHLRC1	NHL repeat containing E3 ubiquitin protein ligase 1	chr6	protein_coding
ENSG00000125827.7	1.08061277	4.76058577	0.00021051	0.00079246	1.72	2.84	5.44	7.44	TMX4	thioredoxin-related transmembrane protein 4	chr20	protein_coding
ENSG00000204524.6	1.35696621	-0.1569756	0.00113993	0.00370076	0.2	0.09	0.46	0.55	ZNF805	zinc finger protein 805	chr19	protein_coding
ENSG00000107643.14	1.61723448	1.719008	1.20E-06	6.81E-06	0.15	0.28	0.77	0.95	MAPK8	mitogen-activated protein kinase 8	chr10	protein_coding
ENSG00000278514.1	6.24527241	-2.2216849	1.55E-05	7.26E-05	0	0	0.44	0.94	AC068831.16	--	chr15	lincRNA
ENSG00000135378.3	4.07416573	2.18478536	1.64E-26	6.29E-25	0.08	0.07	1.67	1.59	PRRG4	proline rich Gla (G-carboxyglutamic acid) 4 (transmembrane)	chr11	protein_coding
ENSG00000117528.10	1.96503287	2.16192162	2.07E-09	1.74E-08	0.47	0.3	1.38	2.66	ABCD3	ATP-binding cassette, sub-family D (ALD), member 3	chr1	protein_coding
ENSG00000198932.11	1.63197328	-0.8140216	0.0013331	0.0042838	0.03	0.04	0.12	0.14	GPRASP1	G protein-coupled receptor associated sorting protein 1	chrX	protein_coding
ENSG00000262874.1	4.87917439	-3.0678699	0.01889873	0.04519557	0	0	0	0.15	C19orf84	chromosome 19 open reading frame 84	chr19	protein_coding
ENSG00000166233.11	1.43795771	4.66430367	1.08E-06	6.17E-06	0.43	0.59	1.84	1.83	ARIH1	ariadne RBR E3 ubiquitin protein ligase 1	chr15	protein_coding
ENSG00000165671.17	1.06765046	4.88374567	0.00024415	0.00091037	1.15	1.12	3.07	3.27	NSD1	nuclear receptor binding SET domain protein 1	chr5	protein_coding
ENSG00000003147.16	-4.8336068	-0.553126	1.36E-14	2.08E-13	0.38	0.22	0.02	0.01	ICA1	islet cell autoantigen 1, 69kDa	chr7	protein_coding
ENSG00000164062.11	-1.2073849	8.11305879	2.78E-05	0.00012447	107.97	85.2	56.63	54.9	APEH	acylaminoacyl-peptide hydrolase	chr3	protein_coding
ENSG00000178860.8	-2.8045511	-2.171035	0.00041794	0.0014836	0.11	0.14	0	0.04	MSC	musculin	chr8	protein_coding
ENSG00000176681.13	3.29119263	-2.4960254	0.003237	0.00945156	0	0	0	0.06	LRRC37A	leucine rich repeat containing 37A	chr17	protein_coding
ENSG00000011566.13	2.92438255	0.51224353	6.17E-12	7.10E-11	0.06	0.05	0.53	0.6	MAP4K3	mitogen-activated protein kinase kinase kinase kinase 3	chr2	protein_coding
ENSG00000185630.17	-1.0646645	4.46089052	0.00026793	0.0009833	3.01	3.39	2.03	2.05	PBX1	pre-B-cell leukemia homeobox 1	chr1	protein_coding
ENSG00000152147.9	-1.2373502	4.1904549	2.60E-05	0.00011712	4.8	4.7	2.13	3.27	GEMIN6	gem (nuclear organelle) associated protein 6	chr2	protein_coding
ENSG00000236269.1	1.58954705	-1.6755621	0.01792389	0.04353308	0.04	0.23	0.47	0.59	ENO1-IT1	ENO1 intronic transcript 1	chr1	sense_intronic
ENSG00000277287.1	3.30186759	-1.1008251	2.55E-07	1.61E-06	0	0.02	0.22	0.05	RP4-794I6.4	--	chr20	lincRNA
ENSG00000188846.12	-1.4314508	9.64220085	7.58E-07	4.45E-06	122.15	140.86	62.64	67.31	RPL14	ribosomal protein L14	chr3	protein_coding
ENSG00000158710.13	-1.6431474	9.32035551	1.67E-08	1.24E-07	436.28	369.78	172.11	171.98	TAGLN2	transgelin 2	chr1	protein_coding
ENSG00000178700.6	2.37769746	1.08029336	2.17E-10	2.07E-09	0.11	0.14	0.7	1.05	DHFRL1	dihydrofolate reductase-like 1	chr3	protein_coding
ENSG00000267040.5	-5.5205905	-2.8639386	0.00158008	0.00495521	0.09	0.02	0	0	RP11-35G9.	--	chr18	antisense
ENSG00000254772.8	-1.9677576	11.8566176	2.19E-11	2.36E-10	3328.61	2947.91	1097.81	1039.6	EEF1G	eukaryotic translation elongation factor 1 gamma	chr11	protein_coding
ENSG00000244642.3	4.67964987	-1.5100563	3.01E-07	1.88E-06	0.09	0	2.82	1.11	RN7SL396P	RNA, 7SL, cytoplasmic 396, pseudogene	chr8	misc_RNA
ENSG00000198938.2	1.22288169	10.7546858	2.16E-05	9.83E-05	900.14	1243.79	3524.84	3120.29	MT-CO3	mitochondrially encoded cytochrome c oxidase III	chrM	protein_coding
ENSG00000139946.8	-2.1666871	0.42682978	2.25E-08	1.63E-07	0.24	0.35	0.04	0.13	PELI2	pellino E3 ubiquitin protein ligase family member 2	chr14	protein_coding
ENSG00000278396.1	3.01556774	-1.6413198	0.00010281	0.00041304	0	0.16	1.09	0.72	RP11-120E1	--	chr14	sense_intronic
ENSG00000075223.12	1.45189872	1.92748725	7.89E-06	3.92E-05	0.37	0.24	0.69	1.55	SEMA3C	sema domain, immunoglobulin domain (Ig), short basic domain, secreted, (semaphorin) 3C	chr7	protein_coding
ENSG00000143179.11	-1.1447626	5.57677368	7.78E-05	0.00032101	28.01	25.29	15.74	16.41	UCK2	uridine-cytidine kinase 2	chr1	protein_coding
ENSG00000049089.12	-2.8386657	2.25350411	5.34E-17	1.01E-15	2.07	2.66	0.4	0.48	COL9A2	collagen, type IX, alpha 2	chr1	protein_coding
ENSG00000105711.9	-2.4148324	1.5235578	4.92E-12	5.73E-11	1.74	2.2	0.5	0.48	SCN1B	sodium channel, voltage gated, type I beta subunit	chr19	protein_coding
ENSG00000067082.13	-1.2474124	4.59896445	2.04E-05	9.35E-05	4.68	4.55	2.65	2.53	KLF6	Kruppel-like factor 6	chr10	protein_coding
ENSG00000084110.9	5.6923233	-2.6004234	0.00047761	0.00167191	0	0	0.07	0.04	HAL	histidine ammonia-lyase	chr12	protein_coding
ENSG00000207421.1	-1.4710123	-1.1177158	0.00604922	0.01656143	10.38	4.23	3.35	3.66	SNORD38B	small nucleolar RNA, C/D box 38B	chr1	snoRNA
ENSG00000145494.10	-1.1641108	9.33367453	5.18E-05	0.00022027	592.27	501.37	329.98	320.47	NDUFS6	NADH dehydrogenase (ubiquinone) Fe-S protein 6, 13kDa (NADH-coenzyme Q reductase)	chr5	protein_coding
ENSG00000087302.7	-1.5376922	6.84370855	1.28E-07	8.45E-07	18.69	20.42	8.25	9.73	C14orf166	chromosome 14 open reading frame 166	chr14	protein_coding
ENSG00000179295.14	1.38356112	4.06803141	3.22E-06	1.72E-05	0.97	1.37	3.51	4.65	PTPN11	protein tyrosine phosphatase, non-receptor type 11	chr12	protein_coding
ENSG00000245146.5	4.91295344	-1.312951	3.66E-08	2.59E-07	0	0.01	0.17	0.22	LINC01024	long intergenic non-protein coding RNA 1024	chr5	antisense
ENSG00000182492.14	-6.573793	-2.1621929	1.78E-06	9.85E-06	0.17	0.07	0	0	BGN	biglycan	chrX	protein_coding
ENSG00000254485.4	1.28124228	0.24892178	0.00108413	0.00353636	0.39	0.24	1.07	0.99	RP11-380O2	--	chr3	antisense
ENSG00000196428.11	1.43032456	1.83068924	1.21E-05	5.76E-05	0.11	0.16	0.5	0.48	TSC22D2	TSC22 domain family, member 2	chr3	protein_coding
ENSG00000140015.18	2.36916364	-0.3660799	6.88E-07	4.07E-06	0.01	0.02	0.11	0.11	KCNH5	potassium channel, voltage gated eag related subfamily H, member 5	chr14	protein_coding
ENSG00000267939.1	1.64158793	-0.5623942	0.00044579	0.00157293	0.7	0.4	2.13	2.52	CTD-2325M	--	chr19	lincRNA
ENSG00000259604.4	-5.7810388	-2.7079121	0.00047761	0.00167191	0.16	0.17	0	0	RP11-66B24.	--	chr15	lincRNA
ENSG00000103269.12	-1.5608019	2.32155501	7.63E-07	4.47E-06	3.8	3.17	1.43	1.72	RHBDL1	rhomboid, veinlet-like 1 (Drosophila)	chr16	protein_coding
ENSG00000047932.12	1.19969467	3.06425833	7.90E-05	0.00032575	1.03	0.72	1.92	3.49	GOPC	golgi-associated PDZ and coiled-coil motif containing	chr6	protein_coding
ENSG00000235373.1	1.80575578	-1.715946	0.00961728	0.02507838	0.03	0	0.07	0.1	RP11-206L1	--	chr1	lincRNA
ENSG00000133878.7	-5.5223491	-2.8631056	0.00158008	0.00495521	0.08	0.06	0	0	DUSP26	dual specificity phosphatase 26 (putative)	chr8	protein_coding
ENSG00000143337.17	1.61498545	3.36061856	1.11E-07	7.43E-07	0.5	0.4	1.54	2.14	TOR1AIP1	torsin A interacting protein 1	chr1	protein_coding
ENSG00000120254.14	-1.8348261	6.58874092	4.27E-10	3.93E-09	31.41	32.26	10.24	13.62	MTHFD1L	methylenetetrahydrofolate dehydrogenase (NADP+ dependent) 1-like	chr6	protein_coding
ENSG00000160183.12	2.95483199	4.01404846	1.66E-20	4.16E-19	0.9	0.63	8.1	7.79	TMPRSS3	transmembrane protease, serine 3	chr21	protein_coding
ENSG00000203999.7	-1.5966506	2.70849934	2.34E-07	1.48E-06	3.28	3.68	1.05	2.02	LINC01270	long intergenic non-protein coding RNA 1270	chr20	lincRNA
ENSG00000176490.4	-4.8454091	3.57868496	1.31E-40	1.04E-38	5.73	5.1	0.23	0.27	DIRAS1	DIRAS family, GTP-binding RAS-like 1	chr19	protein_coding

ENSG00000120756.11	4.31304882	-0.2449548	1.22E-13	1.68E-12	0.01	0.02	0.29	0.44	PLS1	plastin 1	chr3	protein_coding
ENSG00000169083.14	-7.1371587	-1.7204162	7.18E-09	5.62E-08	0.02	0.05	0	0	AR	androgen receptor	chrX	protein_coding
ENSG00000261789.1	3.09310043	-2.6178507	0.00943894	0.02469931	0.02	0	0	0.21	RP11-709D2	--	chr16	antisense
ENSG00000087470.16	1.20497869	2.50146837	0.00010952	0.00043673	0.55	0.63	1.23	2.4	DNM1L	dynamin 1-like	chr12	protein_coding
ENSG00000142541.15	-1.8540509	12.6598284	2.45E-10	2.33E-09	7094.03	6803.6	2528.78	2595.61	RPL13A	ribosomal protein L13a	chr19	protein_coding
ENSG00000175768.11	-1.2219005	6.06960387	2.43E-05	0.00010979	78.62	88.72	39.28	56.68	TOMM5	translocase of outer mitochondrial membrane 5 homolog (yeast)	chr9	protein_coding
ENSG00000163395.15	-9.4019307	0.3586235	1.75E-26	6.66E-25	0.08	0.27	0	0	IGFN1	immunoglobulin-like and fibronectin type III domain containing 1	chr1	protein_coding
ENSG00000185507.18	2.85672691	2.81802301	7.81E-18	1.58E-16	0.7	0.57	6.74	5.54	IRF7	interferon regulatory factor 7	chr11	protein_coding
ENSG00000107443.14	1.78713173	-0.1165767	3.43E-05	0.00015114	0.08	0.07	0.2	0.47	CCNJ	cyclin J	chr10	protein_coding
ENSG00000144278.13	-4.1451879	-1.6099575	4.63E-07	2.81E-06	0.02	0.14	0.01	0	GALNT13	polypeptide N-acetylgalactosaminyltransferase 13	chr2	protein_coding
ENSG00000158201.8	1.96444632	2.19237219	2.09E-09	1.75E-08	0.55	0.59	2.42	3.54	ABHD3	abhydrolase domain containing 3	chr18	protein_coding
ENSG00000205659.9	1.2917214	2.82375849	2.64E-05	0.00011887	1.05	1.32	4.04	3.67	LIN52	lin-52 DREAM MuvB core complex component	chr14	protein_coding
ENSG00000110092.3	-6.2311143	6.75559242	5.08E-66	1.67E-63	35.97	42.51	0.65	0.74	CCND1	cyclin D1	chr11	protein_coding
ENSG00000196632.9	8.72647188	-0.1475639	8.15E-19	1.79E-17	0	0	0.1	0.2	WNK3	WNK lysine deficient protein kinase 3	chrX	protein_coding
ENSG00000104129.8	-1.2563116	5.93621616	1.47E-05	6.96E-05	16.83	20.5	11.27	9.48	DNAJC17	DnaJ (Hsp40) homolog, subfamily C, member 17	chr15	protein_coding
ENSG00000244578.1	1.65673474	-0.8903829	0.0016871	0.00525645	0.07	0.03	0.11	0.32	LINC01391	long intergenic non-protein coding RNA 1391	chr3	lincRNA
ENSG00000171453.16	-1.0817226	5.34045639	0.00018936	0.00071756	27.25	25.36	15.92	17.24	POLR1C	polymerase (RNA) I polypeptide C, 30kDa	chr6	protein_coding
ENSG00000197106.6	3.07238205	3.6346885	2.62E-21	7.02E-20	0.24	0.42	3.94	3.43	SLC6A17	solute carrier family 6 (neutral amino acid transporter), member 17	chr1	protein_coding
ENSG00000267383.5	2.31964219	-0.1357486	4.32E-07	2.64E-06	0.07	0.05	0.38	0.42	CTC-260E6.6	--	chr19	antisense
ENSG00000133195.10	1.02593063	2.93094444	0.00070966	0.00241098	1.54	1.07	3.33	3.77	SLC39A11	solute carrier family 39, member 11	chr17	protein_coding
ENSG00000128165.8	-2.2460312	4.28722288	1.79E-13	2.43E-12	5.8	6.42	1.92	1.5	ADM2	adrenomedullin 2	chr22	protein_coding
ENSG00000198211.8	1.52539376	6.9769601	1.59E-07	1.04E-06	20.77	17.05	69.74	75.61	TUBB3	tubulin, beta 3 class III	chr16	protein_coding
ENSG00000274677.1	-5.3778332	-2.9451057	0.00290522	0.00856312	0.04	0.37	0	0	RP11-505K9.	--	chr16	sense_intronic
ENSG00000159720.10	1.88874633	7.02399324	1.36E-10	1.34E-09	27.23	21.4	119.98	120.25	ATP6V0D1	ATPase, H+ transporting, lysosomal 38kDa, V0 subunit d1	chr16	protein_coding
ENSG00000005889.14	3.31798891	1.88164313	2.18E-19	4.99E-18	0.05	0.08	0.74	1.03	ZFX	zinc finger protein, X-linked	chrX	protein_coding
ENSG00000065060.15	1.12905289	3.76754576	0.00014148	0.00055146	0.74	0.65	1.8	2.26	UHRF1BP1	UHRF1 binding protein 1	chr6	protein_coding
ENSG00000270038.1	-5.3724655	-2.9476208	0.00290522	0.00856312	0.26	0.08	0	0	RP11-1070N	--	chr14	lincRNA
ENSG00000197563.8	1.24658985	2.80388813	5.07E-05	0.00021634	0.55	0.65	1.5	2.31	PIGN	phosphatidylinositol glycan anchor biosynthesis, class N	chr18	protein_coding
ENSG00000178015.4	1.6651007	0.16134288	4.17E-05	0.00018165	0.14	0.25	0.87	0.78	GPR150	G protein-coupled receptor 150	chr5	protein_coding
ENSG00000267318.1	4.57766982	-1.5864702	1.57E-07	1.02E-06	0.06	0	1.2	1.02	RP11-178C3	--	chr17	protein_coding
ENSG00000138738.9	-7.1796837	-1.6845418	4.48E-09	3.59E-08	0.06	0.06	0	0	PRDM5	PR domain containing 5	chr4	protein_coding
ENSG00000163239.11	6.03844038	-0.3474003	1.15E-15	1.94E-14	0.01	0	0.57	0.59	TDRD10	tudor domain containing 10	chr1	protein_coding
ENSG00000119522.14	-1.0424285	5.93726005	0.00030429	0.00110637	8.17	8.14	5.42	5.12	DENND1A	DENN/MADD domain containing 1A	chr9	protein_coding
ENSG00000132970.11	6.28089655	0.67461083	2.91E-25	1.03E-23	0.01	0	0.56	0.7	WASF3	WAS protein family, member 3	chr13	protein_coding
ENSG00000121274.11	1.74798738	1.07659538	1.14E-06	6.50E-06	0.08	0.25	0.68	0.76	PAPD5	PAP associated domain containing 5	chr16	protein_coding
ENSG00000104047.13	1.31976712	1.23593786	0.00012925	0.0005085	0.06	0.09	0.21	0.29	DTWD1	DTW domain containing 1	chr15	protein_coding
ENSG00000129038.14	-5.0987257	5.18078919	4.10E-48	5.14E-46	25.38	23.17	0.99	0.89	LOXL1	lysyl oxidase-like 1	chr15	protein_coding
ENSG00000013016.13	2.42142355	1.85449044	1.70E-12	2.09E-11	0.24	0.15	1.41	1.35	EHD3	EH-domain containing 3	chr2	protein_coding
ENSG00000106366.8	-4.6264859	7.64313556	1.51E-44	1.51E-42	101.63	93.35	4.78	5.76	SERPINE1	serpin peptidase inhibitor, clade E (nexin, plasminogen activator inhibitor type 1), member 1	chr7	protein_coding
ENSG00000105427.8	2.7436444	3.80423578	4.68E-18	9.66E-17	3.9	5.78	48.13	37.89	CNFN	cornifelin	chr19	protein_coding
ENSG00000161395.11	1.01697585	3.00591877	0.00076897	0.00259667	1.79	1.31	4.24	4.15	PGAP3	post-GPI attachment to proteins 3	chr17	protein_coding
ENSG00000157107.12	1.84911396	-0.1891986	3.53E-05	0.00015536	0.04	0.07	0.04	0.46	FCHO2	FCH domain only 2	chr5	protein_coding
ENSG00000279078.1	7.20458075	-1.4865882	1.76E-09	1.50E-08	0	0	0.32	0.16	SND1-IT1	SND1 intronic transcript 1	chr7	lincRNA
ENSG00000162409.9	1.23546031	0.86915719	0.00058108	0.00201122	0.09	0.09	0.2	0.37	PRKAA2	protein kinase, AMP-activated, alpha 2 catalytic subunit	chr1	protein_coding
ENSG00000164574.14	-1.6380231	5.99486172	2.20E-08	1.60E-07	7.97	9.19	3.54	3.81	GALNT10	polypeptide N-acetylgalactosaminyltransferase 10	chr5	protein_coding
ENSG00000086159.11	3.55948786	-2.3248717	0.00066294	0.00226478	0.01	0	0.03	0.13	AQP6	aquaporin 6, kidney specific	chr12	protein_coding
ENSG00000097007.16	-1.5716076	8.1853448	6.50E-08	4.48E-07	63.49	58.79	29.2	25.53	ABL1	ABL proto-oncogene 1, non-receptor tyrosine kinase	chr9	protein_coding
ENSG00000232233.1	1.44558113	0.33321996	0.00020106	0.00075926	0.22	0.34	0.94	1.09	RP11-573D1	--	chr3	lincRNA
ENSG00000179750.14	-6.9526082	1.85787753	5.18E-42	4.34E-40	2.95	3.21	0.03	0.03	APOBEC3B	apolipoprotein B mRNA editing enzyme, catalytic polypeptide-like 3B	chr22	protein_coding
ENSG00000130449.5	1.1792486	1.95367475	0.00024674	0.00091931	0.29	0.4	0.75	1.32	ZSWIM6	zinc finger, SWIM-type containing 6	chr5	protein_coding
ENSG00000183723.11	1.4545995	4.33787865	9.11E-07	5.28E-06	2.3	2.28	7.64	9.16	CMTM4	CKLF-like MARVEL transmembrane domain containing 4	chr16	protein_coding
ENSG00000166145.13	4.6193585	4.9140356	2.12E-41	1.74E-39	0.66	0.58	21.94	18.7	SPINT1	serine peptidase inhibitor, Kunitz type 1	chr15	protein_coding
ENSG00000263528.6	-1.6709682	3.82950013	2.77E-08	2.00E-07	4.64	5.78	2.28	2.07	IKBKE	inhibitor of kappa light polypeptide gene enhancer in B-cells, kinase epsilon	chr1	protein_coding
ENSG00000274370.1	-5.653126	-2.7855647	0.00086564	0.00288128	0.48	0	0	0	AC144831.3	--	chr17	lincRNA
ENSG00000260428.2	1.52133617	1.37184367	7.77E-06	3.86E-05	1.23	0.85	4.14	3.87	SCX	scleraxis basic helix-loop-helix transcription factor	chr8	protein_coding
ENSG00000120820.11	1.02057941	2.51040013	0.0010061	0.00330431	1.14	1.08	2.31	3.74	GLT8D2	glycosyltransferase 8 domain containing 2	chr12	protein_coding
ENSG00000119782.12	-1.610712	-0.5532145	0.00046171	0.00162553	0.97	0.47	0.33	0.3	FKBP1B	FK506 binding protein 1B, 12.6 kDa	chr2	protein_coding
ENSG00000198466.11	1.18285811	2.88338884	0.00010813	0.00043222	0.37	0.66	1.39	1.73	ZNF587	zinc finger protein 587	chr19	protein_coding
ENSG00000248405.8	4.30853741	0.64126932	4.86E-19	1.09E-17	0.01	0.08	1.27	0.99	PRR5-ARHG/	PRR5-ARHGAP8 readthrough	chr22	protein_coding
ENSG00000164040.15	-1.2773888	5.61815276	1.09E-05	5.27E-05	11.88	12.9	6.01	7.64	PGRMC2	progesterone receptor membrane component 2	chr4	protein_coding
ENSG00000138642.13	2.31138621	1.19027383	2.93E-10	2.75E-09	0.2	0.1	0.89	1.12	HERC6	HECT and RLD domain containing E3 ubiquitin protein ligase family member 6	chr4	protein_coding
ENSG00000028137.15	-1.738375	2.85595761	2.05E-08	1.50E-07	2.5	2.37	1.06	0.88	TNFRSF1B	tumor necrosis factor receptor superfamily, member 1B	chr1	protein_coding
ENSG00000074211.12	-1.7232774	-1.6745884	0.00486779	0.01371541	0.05	0.09	0.01	0.04	PPP2R2C	protein phosphatase 2, regulatory subunit B, gamma	chr4	protein_coding
ENSG00000122547.9	-1.4314799	2.40835984	4.63E-06	2.40E-05	1.21	1.41	0.63	0.67	EEPD1	endonuclease/exonuclease/phosphatase family domain containing 1	chr7	protein_coding
ENSG00000178567.7	3.98544042	1.59231495	8.82E-23	2.59E-21	0.04	0.03	0.42	1.04	EPM2AIP1	EPM2A (laforin) interacting protein 1	chr3	protein_coding
ENSG00000280120.1	1.54965909	0.07307468	0.00015685	0.00060382	0.43	0.32	1.73	1.24	RP11-546D6	--	chr12	TEC
ENSG00000110321.14	1.18190177	6.68912069	4.26E-05	0.00018503	9.68	13.11	28.01	41.13	EIF4G2	eukaryotic translation initiation factor 4 gamma, 2	chr11	protein_coding
ENSG00000109762.14	1.30410231	1.66474423	7.61E-05	0.00031436	0.31	0.52	0.97	1.74	SNX25	sorting nexin 25	chr4	protein_coding
ENSG00000184515.9	6.31824416	-2.1698271	8.95E-06	4.38E-05	0	0	0.24	0.36	BEX5	brain expressed, X-linked 5	chrX	protein_coding
ENSG00000198727.2	1.4830553	10.0478514	3.10E-07	1.93E-06	290.15	503.53	1621.61	1317.96	MT-CYB	mitochondrially encoded cytochrome b	chrM	protein_coding
ENSG00000204147.8	1.91081157	-0.7066998	0.00013355	0.00052381	0.01	0.09	0.24	0.25	ASAH2B	N-acylsphingosine amidohydrolase (non-lysosomal ceramidase) 2B	chr10	protein_coding
ENSG00000175279.20	1.35278711	-1.1978941	0.01656825	0.04052383	0.27	0.09	0.86	0.43	APITD1	apoptosis-inducing, TAF9-like domain 1	chr1	protein_coding
ENSG00000171155.7	1.20736381	3.16980898	6.77E-05	0.0002825	2.17	2.71	5.22	9.9	C1GALT1C1	C1GALT1-specific chaperone 1	chrX	protein_coding
ENSG00000235277.1	6.79758941	-1.8137577	1.35E-07	8.87E-07	0	0	0.86	0.31	AF127577.8	--	chr21	antisense
ENSG00000134152.9	1.44967607	-0.355843	0.00138295	0.00443091	0.13	0.09	0.09	0.71	KATNBL1	katanin p80 subunit B-like 1	chr15	protein_coding
ENSG00000132429.8	-5.7564047	0.30085988	5.37E-23	1.61E-21	1.13	1.77	0.02	0.04	POPDC3	popeye domain containing 3	chr6	protein_coding
ENSG00000172380.5	1.05217109	2.24043812	0.00083476	0.00280224	0.45	0.66	1.26	1.82	GNG12	guanine nucleotide binding protein (G protein), gamma 12	chr1	protein_coding
ENSG00000209042.1	3.41854287	-2.4190721	0.00112121	0.00364442	0.3	0	2.88	2.45	SNORD12C	small nucleolar RNA, C/D box 12C	chr20	snoRNA
ENSG00000111110.10	3.55245626	3.91551696	6.45E-27	2.54E-25	0.29	0.31	4.32	5.07	PPM1H	protein phosphatase, Mg2+/Mn2+ dependent, 1H	chr12	protein_coding
ENSG00000136051.12	3.80700443	0.84516803	1.03E-17	2.07E-16	0.03	0.03	0.34	0.83	KIAA1033	KIAA1033	chr12	protein_coding
ENSG00000241322.7	-1.8446325	-0.6501121	6.95E-05	0.0002889	0.14	0.23	0.06	0.07	CDRT1	CMT1A duplicated region transcript 1	chr17	protein_coding
ENSG00000151338.17	3.51533279	-0.9193561	3.09E-08	2.20E-07	0.01	0.01	0.08	0.14	MIPOL1	mirror-image polydactyly 1	chr14	protein_coding
ENSG00000144357.15	2.6192915	1.27340671	1.38E-12	1.71E-11	0.09	0.04	0.36	0.71	UBR3	ubiquitin protein ligase E3 component n-recognin 3 (putative)	chr2	protein_coding
ENSG00000178927.15	1.90483157	5.96172834	1.13E-10	1.13E-09	6.5	6.08	32.06	30.74	C17orf62	chromosome 17 open reading frame 62	chr17	protein_coding
ENSG00000171016.10	1.66285355	-0.0946767	8.44E-05	0.0003431	0.05	0.03	0.12	0.21	PYGO1	pygopus family PHD finger 1	chr15	protein_coding
ENSG00000087116.12	1.94268932	-1.4507014	0.00185375	0.00571918	0.03	0	0.09	0.06	ADAMTS2	ADAM metallopeptidase with thrombospondin type 1 motif, 2	chr5	protein_coding
ENSG00000124226.9	1.04380552	6.18785847	0.00029561	0.00107825	15.48	15.24	39.23	45.32	RNF114	ring finger protein 114	chr20	protein_coding
ENSG00000232936.4	-3.3118446	-2.2161377	0.00067192	0.00229154	0.57	0.23	0.11	0	RP11-80H5.2	--	chr10	antisense
ENSG00000103489.10	1.17614371	-0.0066088	0.00371897	0.01072816	0.04	0.05	0.1	0.17	XYLT1	xylosyltransferase I	chr16	protein_coding
ENSG00000246379.5	7.1000136	-1.5659428	7.18E-09	5.62E-08	0	0	0.18	0.23	RP11-461O7	--	chr16	lincRNA
ENSG00000177854.7	-1.3877396	3.74141063	3.35E-06	1.78E-05	8.44	8.2	4.16	4.32	TMEM187	transmembrane protein 187	chrX	protein_coding
ENSG00000253270.1	-6.6384281	-2.1130712	1.05E-06	6.02E-06	1.03	0.24	0	0	RP11-1105O	--	chr8	lincRNA
ENSG00000226711.5	4.08287986	-0.8046057	4.52E-10	4.14E-09	0.01	0.01	0.31	0.12	FAM66C	family with sequence similarity 66, member C	chr12	antisense
ENSG00000176105.12	4.03851787	2.1556495	4.32E-26	1.60E-24	0.09	0.08	1.06	2.6	YES1	YES proto-oncogene 1, Src family tyrosine kinase	chr18	protein_coding
ENSG00000262869.1	5.35410067	-2.8076728	0.00290522	0.00856312	0	0	0.4	0.19	CTD-2545H1	--	chr17	sense_intronic
ENSG00000228594.3	-1.0554854	4.4814069	0.00030387	0.00110505	12.34	11.04	8.5	6.45	C1orf233	chromosome 1 open reading frame 233	chr1	protein_coding
ENSG00000137955.14	-1.7847024	4.18278166	2.60E-09	2.16E-08	14.04	15.32	4.95	6.42	RABGGTB	Rab geranylgeranyltransferase, beta subunit	chr1	protein_coding
ENSG00000213057.5	2.62746485	-0.3847626	1.11E-07	7.42E-07	0.08	0.03	0.58	0.4	C1orf220	chromosome 1 open reading frame 220	chr1	lincRNA
ENSG00000257167.2	1.30706147	3.31022221	1.51E-05	7.11E-05	1.17	1.56	5.09	3.89	TMPO-AS1	TMPO antisense RNA 1	chr12	antisense
ENSG00000064205.9	-2.5038355	2.82793068	9.47E-15	1.48E-13	5.3	5.47	1.63	0.9	WISP2	WNT1 inducible signaling pathway protein 2	chr20	protein_coding
ENSG00000210151.2	2.47554567	-0.4980156	8.82E-07	5.12E-06	0.78	3.7	19.03	14.21	MT-TS1	mitochondrially encoded tRNA serine 1 (UCN)	chrM	Mt_tRNA
ENSG00000077157.19	1.05505792	2.69764249	0.00060989	0.00210062	0.17	0.24	0.56	0.6	PPP1R12B	protein phosphatase 1, regulatory subunit 12B	chr1	protein_coding
ENSG00000171466.8	1.49740786	3.0971278	9.91E-07	5.71E-06	0.2	0.37	0.99	1.15	ZNF562	zinc finger protein 562	chr19	protein_coding
ENSG00000055332.15	2.01782638	2.82044836	1.84E-10	1.77E-09	0.22	0.21	0.89	1.46	EIF2AK2	eukaryotic translation initiation factor 2-alpha kinase 2	chr2	protein_coding
ENSG00000179859.7	1.10839142	3.88134195	0.00017859	0.00068041	2.94	3	8.8	8.26	AC025335.1	--	chr17	antisense
ENSG00000227258.4	-2.1432203	0.58363731	1.64E-08	1.22E-07	0.45	0.85	0.17	0.22	SMIM2-AS1	SMIM2 antisense RNA 1	chr13	antisense
ENSG00000277586.1	8.06189439	2.90284035	4.49E-53	7.52E-51	0.01	0.01	3.83	3.91	NEFL	neurofilament, light polypeptide	chr8	protein_coding
ENSG00000117479.11	1.40787092	0.60216652	0.00016684	0.00063822	0.17	0.18	0.45	0.78	SLC19A2	solute carrier family 19 (thiamine transporter), member 2	chr1	protein_coding
ENSG00000147251.14	3.00979682	0.59258364	1.08E-12	1.35E-11	0.01	0.06	0.31	0.5	DOCK11	dedicator of cytokinesis 11	chrX	protein_coding
ENSG00000265091.4	-5.5258708	-2.8614387	0.00158008	0.00495521	0.05	0.23	0	0	RP11-835E18	--	chr18	lincRNA
ENSG00000269973.1	1.8085193	-0.8878712	0.00058604	0.0020272	0.07	0.04	0.25	0.24	RP11-95D17	--	chr2	lincRNA
ENSG00000130675.13	2.56521552	3.12532922	1.48E-15	2.48E-14	0.62	0.85	5.75	5.85	MNX1	motor neuron and pancreas homeobox 1	chr7	protein_coding
ENSG00000064393.14	-2.110889	5.18533452	1.80E-12	2.20E-11	2.67	3.55	1	0.92	HIPK2	homeodomain interacting protein kinase 2	chr7	protein_coding
ENSG00000273367.1	5.88630615	-2.4741528	0.00014842	0.00057423	0	0	0.51	0.26	RP5-827C21	--	chr1	antisense
ENSG00000178301.3	3.99131696	-0.8709012	1.79E-09	1.51E-08	0	0.04	0.4	0.47	AQP11	aquaporin 11	chr11	protein_coding
ENSG00000100593.16	1.85416057	0.92912038	3.87E-07	2.38E-06	0.21	0.22	0.86	1.2	ISM2	isthmin 2	chr14	protein_coding
ENSG00000179041.3	-2.1505541	6.77232229	4.37E-13	5.68E-12	74.94	94.62	24.3	26.58	RRS1	RRS1 ribosome biogenesis regulator homolog (S. cerevisiae)	chr8	protein_coding
ENSG00000172888.10	1.22750696	2.17039411	0.00010995	0.00043806	0.24	0.3	0.72	0.98	ZNF621	zinc finger protein 621	chr3	protein_coding
ENSG00000179855.5	-4.450164	4.31498645	1.55E-38	1.10E-36	7	7.23	0.41	0.45	GIPC3	GIPC PDZ domain containing family, member 3	chr19	protein_coding
ENSG00000130309.9	-2.1481508	9.33815357	3.96E-13	5.17E-12	242.02	219.49	71.7	66.95	COLGALT1	collagen beta(1-O)galactosyltransferase 1	chr19	protein_coding
ENSG00000165060.10	-1.3939634	3.25864256	3.94E-06	2.06E-05	3.83	3.79	1.66	2.21	FXN	frataxin	chr9	protein_coding
ENSG00000254469.6	1.48697754	-0.3333349	0.00077463	0.00261395	0.08	0.19	0.66	0.35	RP11-849H4	--	chr11	protein_coding
ENSG00000153234.12	1.32843483	1.91288653	4.16E-05	0.00018119	0.45	0.44	1.03	1.99	NR4A2	nuclear receptor subfamily 4, group A, member 2	chr2	protein_coding
ENSG00000157653.10	2.00853228	0.32059932	7.38E-07	4.34E-06	0.11	0.23	1.14	0.7	C9orf43	chromosome 9 open reading frame 43	chr9	protein_coding
ENSG00000225830.9	1.47740045	2.53197164	2.40E-06	1.30E-05	0.22	0.39	0.98	1.26	ERCC6	excision repair cross-complementation group 6	chr10	protein_coding
ENSG00000211891.5	-7.3435532	-1.5460182	7.05E-10	6.26E-09	0.49	0.2	0	0	IGHE	immunoglobulin heavy constant epsilon	chr14	IG_C_gene
ENSG00000272030.1	1.74850099	0.31036591	1.12E-05	5.36E-05	0.41	0.4	1.73	1.91	RP1-178F15.	--	chr1	antisense
ENSG00000226953.6	-6.0965069	-2.5028917	8.36E-05	0.00034052	0.3	0.21	0	0	AC010890.1	--	chr2	antisense
ENSG00000115760.12	2.25617215	3.08257086	1.01E-12	1.26E-11	0.14	0.16	0.86	1.04	BIRC6	baculoviral IAP repeat containing 6	chr2	protein_coding
ENSG00000198840.2	1.135092	9.7958298	7.83E-05	0.00032253	1063.87	1543.6	4032.49	3570.5	MT-ND3	mitochondrially encoded NADH dehydrogenase 3	chrM	protein_coding
ENSG00000261254.2	5.05694572	-2.9744029	0.0100455	0.02582742	0	0	0.02	0.03	RP4-714D9.5	--	chr1	sense_overlapping
ENSG00000180694.12	1.11998491	0.62464947	0.00232638	0.00704318	0.11	0.16	0.35	0.43	TMEM64	transmembrane protein 64	chr8	protein_coding
ENSG00000125962.13	1.061249	1.36712255	0.00164359	0.00513625	0.25	0.28	0.63	0.86	ARMCX5	armadillo repeat containing, X-linked 5	chrX	protein_coding
ENSG00000164941.12	1.25107401	1.38673551	0.00022015	0.000826	0.29	0.23	0.66	1.01	INTS8	integrator complex subunit 8	chr8	protein_coding
ENSG00000176809.9	4.08834631	-0.4448557	5.22E-12	6.06E-11	0.01	0.01	0.2	0.18	LRRC37A3	leucine rich repeat containing 37, member A3	chr17	protein_coding
ENSG00000230825.1	-4.9420536	-0.9411782	1.56E-12	1.92E-11	1.07	1.4	0.1	0	AC005532.5	--	chr7	processed_transcript
ENSG00000230387.2	-8.9151689	-0.1123791	1.42E-21	3.89E-20	0.58	0.68	0	0	RP4-737E23.	--	chr20	lincRNA
ENSG00000132016.10	2.07811287	4.43392497	6.31E-12	7.26E-11	2.16	2.4	13.22	12.4	C19orf57	chromosome 19 open reading frame 57	chr19	protein_coding
ENSG00000084093.14	2.08100511	1.2216702	6.97E-09	5.47E-08	0.08	0.11	0.43	0.61	REST	RE1-silencing transcription factor	chr4	protein_coding
ENSG00000111554.13	2.09968581	1.2880924	3.98E-09	3.23E-08	0.17	0.12	0.51	1.14	MDM1	Mdm1 nuclear protein homolog (mouse)	chr12	protein_coding
ENSG00000115504.13	1.25678831	2.62638943	4.88E-05	0.00020861	0.37	0.69	1.14	2.23	EHBP1	EH domain binding protein 1	chr2	protein_coding
ENSG00000263740.2	4.12937396	-0.4155018	2.15E-12	2.61E-11	0.09	0.29	6.73	2.49	RN7SL4P	RNA, 7SL, cytoplasmic 4, pseudogene	chr3	misc_RNA
ENSG00000101849.14	-1.0729565	6.31910345	0.00019831	0.00074996	13.55	14.84	8.9	9.08	TBL1X	transducin (beta)-like 1X-linked	chrX	protein_coding
ENSG00000100889.10	-1.585187	7.12924141	5.29E-08	3.68E-07	54.01	52.29	23.87	23.32	PCK2	phosphoenolpyruvate carboxykinase 2 (mitochondrial)	chr14	protein_coding
ENSG00000243370.3	2.17224153	-2.2103288	0.02071392	0.04894569	0	0.25	0.57	0.97	RN7SL775P	RNA, 7SL, cytoplasmic 775, pseudogene	chr17	misc_RNA
ENSG00000100316.14	-2.1079821	11.5547492	9.69E-13	1.22E-11	2686.42	2708.2	855.26	810.62	RPL3	ribosomal protein L3	chr22	protein_coding
ENSG00000268670.1	5.08767213	-2.9577572	0.00538165	0.01488851	0	0	0.42	0	CTD-2622I13	--	chr19	sense_intronic
ENSG00000173457.9	-2.6237803	9.24734579	3.19E-18	6.67E-17	538.01	484.72	115.92	104.96	PPP1R14B	protein phosphatase 1, regulatory (inhibitor) subunit 14B	chr11	protein_coding
ENSG00000204019.4	-9.9958225	0.94682301	2.60E-33	1.41E-31	6.05	5.33	0	0	CT83	cancer/testis antigen 83	chrX	protein_coding
ENSG00000114124.2	-5.3733592	-2.9472019	0.00290522	0.00856312	0.05	0.03	0	0	GRK7	G protein-coupled receptor kinase 7	chr3	protein_coding
ENSG00000165389.6	3.01641078	3.97296983	3.76E-21	9.93E-20	0.87	1.13	8.02	13.66	SPTSSA	serine palmitoyltransferase, small subunit A	chr14	protein_coding
ENSG00000143669.12	1.29900918	0.82185512	0.00034513	0.00124524	0.05	0.07	0.17	0.21	LYST	lysosomal trafficking regulator	chr1	protein_coding
ENSG00000164190.15	3.43119932	3.95867945	1.12E-25	4.04E-24	0.23	0.17	1.87	3.9	NIPBL	Nipped-B homolog (Drosophila)	chr5	protein_coding
ENSG00000141696.11	-1.510195	6.77208236	2.10E-07	1.35E-06	48.97	42.94	22.38	20.59	P3H4	prolyl 3-hydroxylase family member 4 (non-enzymatic)	chr17	protein_coding
ENSG00000179094.12	-1.2692732	6.90620563	1.13E-05	5.42E-05	25.11	25.19	13.84	13.96	PER1	period circadian clock 1	chr17	protein_coding
ENSG00000131013.3	3.06196128	3.12801914	1.98E-20	4.90E-19	0.49	0.64	3.78	8.88	PPIL4	peptidylprolyl isomerase (cyclophilin)-like 4	chr6	protein_coding
ENSG00000171956.6	5.44525159	-2.7521026	0.00158008	0.00495521	0	0	0.01	0.1	FOXB1	forkhead box B1	chr15	protein_coding
ENSG00000118263.13	-4.178354	2.31982524	6.21E-29	2.72E-27	0.95	0.83	0.07	0.06	KLF7	Kruppel-like factor 7 (ubiquitous)	chr2	protein_coding
ENSG00000171843.14	1.08079642	0.55209735	0.00370162	0.01068227	0.06	0.15	0.18	0.4	MLLT3	myeloid/lymphoid or mixed-lineage leukemia (trithorax homolog, Drosophila); translocated to, 3	chr9	protein_coding
ENSG00000221829.8	-1.7619544	6.14354866	1.98E-09	1.67E-08	35.6	31.55	14.27	12.07	FANCG	Fanconi anemia, complementation group G	chr9	protein_coding
ENSG00000164151.10	2.23544215	3.40597516	8.28E-13	1.04E-11	0.38	0.37	1.69	3.05	ICE1	interactor of little elongation complex ELL subunit 1	chr5	protein_coding
ENSG00000167642.11	5.19377838	6.94423448	1.01E-51	1.49E-49	3.49	2.37	142.53	144.21	SPINT2	serine peptidase inhibitor, Kunitz type, 2	chr19	protein_coding
ENSG00000165521.14	3.73237841	0.12488022	9.36E-14	1.31E-12	0.02	0.01	0.24	0.17	EML5	echinoderm microtubule associated protein like 5	chr14	protein_coding
ENSG00000181523.11	1.48542025	4.57934095	5.05E-07	3.06E-06	3.2	2.85	11.83	10.7	SGSH	N-sulfoglucosamine sulfohydrolase	chr17	protein_coding
ENSG00000132825.6	2.39778961	1.92031493	2.38E-12	2.87E-11	0.32	0.2	1.39	2.3	PPP1R3D	protein phosphatase 1, regulatory subunit 3D	chr20	protein_coding
ENSG00000139197.9	-1.1044898	6.46564108	0.00012808	0.00050434	22.64	21.6	13.06	14.39	PEX5	peroxisomal biogenesis factor 5	chr12	protein_coding
ENSG00000266241.1	1.87017068	0.40204013	1.97E-06	1.08E-05	4.81	3.68	15.06	26.69	MIR5047	microRNA 5047	chr17	miRNA
ENSG00000243772.5	-8.612318	-0.4005406	1.03E-18	2.25E-17	0.56	0.37	0	0	KIR2DL3	killer cell immunoglobulin-like receptor, two domains, long cytoplasmic tail, 3	chr19	protein_coding
ENSG00000135679.20	2.47403851	2.29756109	1.27E-13	1.75E-12	0.09	0.1	0.52	0.87	MDM2	MDM2 proto-oncogene, E3 ubiquitin protein ligase	chr12	protein_coding
ENSG00000270127.2	3.92717555	-1.3866744	6.88E-07	4.06E-06	0.02	0	0.24	0.27	RP11-526I2.5	--	chr15	lincRNA
ENSG00000153922.9	3.57943709	2.41360548	2.08E-23	6.39E-22	0.07	0.09	0.88	1.69	CHD1	chromodomain helicase DNA binding protein 1	chr5	protein_coding
ENSG00000279861.1	2.14728274	-0.4454124	6.00E-06	3.04E-05	0.11	0.09	0.77	0.46	RP11-43N16	--	chr19	TEC
ENSG00000158292.6	-2.5101513	2.73842843	1.05E-14	1.64E-13	2.22	2.28	0.6	0.45	GPR153	G protein-coupled receptor 153	chr1	protein_coding
ENSG00000187961.12	-1.1118231	3.96876567	0.00016231	0.00062354	6.84	5.35	3.89	3.63	KLHL17	kelch-like family member 17	chr1	protein_coding
ENSG00000279555.1	2.7310277	-2.2502161	0.00174589	0.00541921	0	0.03	0.17	0.09	RP11-426D1	--	chr17	TEC
ENSG00000031003.9	3.45944075	2.07846621	2.89E-21	7.71E-20	0.09	0.11	1.15	1.77	FAM13B	family with sequence similarity 13, member B	chr5	protein_coding
ENSG00000280241.2	-5.5188333	-2.8647712	0.00158008	0.00495521	0.19	0	0	0	RP11-290O1	--	chr4	antisense
ENSG00000155640.6	3.30253888	0.18120159	2.15E-12	2.61E-11	0.02	0.04	0.33	0.49	C10orf12	chromosome 10 open reading frame 12	chr10	protein_coding
ENSG00000151773.11	3.00803624	-0.1919359	1.32E-09	1.13E-08	0.11	0.03	0.45	1.04	CCDC122	coiled-coil domain containing 122	chr13	protein_coding
ENSG00000233785.1	6.30985008	-2.1747134	8.95E-06	4.38E-05	0	0	0.29	0.65	RP13-314C1	--	chrX	antisense
ENSG00000245910.7	-1.5494002	4.84015819	1.38E-07	9.03E-07	55.6	50.99	20.91	27.78	SNHG6	small nucleolar RNA host gene 6	chr8	processed_transcript

ENSG00000149179.12	-1.804587	4.96084401	1.10E-09	9.52E-09	17.87	17.27	6.54	6.88	C11orf49	chromosome 11 open reading frame 49	chr11	protein_coding
ENSG00000177098.7	2.26928029	-2.5313377	0.01923175	0.0458365	0.01	0	0.06	0.03	SCN4B	sodium channel, voltage gated, type IV beta subunit	chr11	protein_coding
ENSG00000143434.14	1.12605922	-0.1736026	0.00749897	0.02009224	0.26	0.12	0.63	0.47	SEMA6C	sema domain, transmembrane domain (TM), and cytoplasmic domain, (semaphorin) 6C	chr1	protein_coding
ENSG00000103319.10	-1.3309483	5.6423954	4.75E-06	2.45E-05	7.62	8.05	4.17	4.12	EEF2K	eukaryotic elongation factor 2 kinase	chr16	protein_coding
ENSG00000177283.5	-4.3005068	4.37532641	8.82E-37	5.60E-35	8.63	7.24	0.48	0.59	FZD8	frizzled class receptor 8	chr10	protein_coding
ENSG00000279122.1	6.56572038	-1.9874127	1.05E-06	6.02E-06	0	0	0.31	0.3	RP11-394B2.	--	chr16	TEC
ENSG00000260011.2	5.46945885	-2.7387023	0.00158008	0.00495521	0	0	0.53	0.45	RP13-20L14.	--	chr17	lincRNA
ENSG00000057019.14	1.46825141	3.24299253	1.39E-06	7.83E-06	0.57	0.74	2.09	2.74	DCBLD2	discoidin, CUB and LCCL domain containing 2	chr3	protein_coding
ENSG00000278112.1	1.93235425	-0.2131509	1.72E-05	8.02E-05	0.86	0.24	3.77	1.98	RP11-972P1.	--	chr12	sense_intronic
ENSG00000196151.9	1.16820694	0.31501803	0.00291275	0.0085832	0.23	0.32	0.56	1.07	WDSUB1	WD repeat, sterile alpha motif and U-box domain containing 1	chr2	protein_coding
ENSG00000279841.1	3.33853663	-2.4705837	0.00190233	0.00584723	0.01	0	0.17	0.07	RP11-274A1	--	chr16	TEC
ENSG00000254258.1	3.25115021	2.18001492	9.97E-20	2.34E-18	0.39	0.93	8.43	8.19	RP11-398H6	--	chr8	lincRNA
ENSG00000104967.6	3.43591358	-2.408393	0.00112121	0.00364442	0	0	0.05	0.01	NOVA2	neuro-oncological ventral antigen 2	chr19	protein_coding
ENSG00000101017.12	7.65250091	1.97725187	5.03E-42	4.23E-40	0.03	0	4.68	3.98	CD40	CD40 molecule, TNF receptor superfamily member 5	chr20	protein_coding
ENSG00000175182.12	-2.0461034	3.8108257	1.97E-11	2.14E-10	4.16	3.96	1.4	1.22	FAM131A	family with sequence similarity 131, member A	chr3	protein_coding
ENSG00000266036.1	-3.3195409	-2.7893297	0.00943894	0.02469931	0.47	0	0	0.05	RP11-452I5.2	--	chr17	antisense
ENSG00000255867.1	3.62073853	-1.6221396	1.25E-05	5.96E-05	0	0.04	0.34	0.42	DENND5B-A	DENND5B antisense RNA 1	chr12	antisense
ENSG00000180917.15	2.96649567	-0.3569168	8.36E-09	6.48E-08	0.01	0.04	0.16	0.35	CMTR2	cap methyltransferase 2	chr16	protein_coding
ENSG00000105290.10	1.26886365	6.00010382	1.22E-05	5.81E-05	10.21	9.59	32.64	30.92	APLP1	amyloid beta (A4) precursor-like protein 1	chr19	protein_coding
ENSG00000260837.1	4.72173804	-3.1445547	0.01889873	0.04519557	0	0	0.1	0.04	RP11-434B12	--	chr2	lincRNA
ENSG00000223725.5	-5.2109276	-3.0346387	0.00538165	0.01488851	0.04	0.13	0	0	AC007879.5	--	chr2	antisense
ENSG00000179958.7	-1.0531982	5.20774571	0.00028043	0.00102663	29.52	21.44	15.95	16.85	DCTPP1	dCTP pyrophosphatase 1	chr16	protein_coding
ENSG00000196878.11	-3.1586569	9.17700773	1.25E-24	4.20E-23	182.37	193.28	29.72	26.21	LAMB3	laminin, beta 3	chr1	protein_coding
ENSG00000165338.15	1.5479768	-1.2886474	0.01100763	0.02808973	0.02	0.02	0.08	0.08	HECTD2	HECT domain containing E3 ubiquitin protein ligase 2	chr10	protein_coding
ENSG00000054598.6	-1.2117922	4.02725083	4.18E-05	0.00018169	3.95	5.44	2.86	2.53	FOXC1	forkhead box C1	chr6	protein_coding
ENSG00000261324.2	4.71108305	0.10467085	5.00E-17	9.48E-16	0.01	0.01	0.32	0.42	RP11-174G6	--	chr12	sense_overlapping
ENSG00000176658.15	6.37679838	2.74765807	8.54E-46	9.27E-44	0.02	0.02	2.26	1.98	MYO1D	myosin ID	chr17	protein_coding
ENSG00000111452.11	-9.1300322	2.28521803	5.19E-51	7.32E-49	1.4	1.45	0.01	0	GPR133	--	chr12	protein_coding
ENSG00000179242.14	-5.589264	-1.137072	1.76E-12	2.16E-11	0.11	0.08	0	0	CDH4	cadherin 4, type 1, R-cadherin (retinal)	chr20	protein_coding
ENSG00000172766.17	3.03224159	0.68305922	3.73E-13	4.88E-12	0.04	0.07	0.4	0.85	NAA16	N(alpha)-acetyltransferase 16, NatA auxiliary subunit	chr13	protein_coding
ENSG00000077420.14	1.42857968	1.39872052	2.55E-05	0.00011498	0.29	0.33	1	1.23	APBB1IP	amyloid beta (A4) precursor protein-binding, family B, member 1 interacting protein	chr10	protein_coding
ENSG00000279764.1	3.74493497	-2.2034493	0.00013996	0.00054609	0.02	0	0.26	0.15	RP11-441F2.	--	chr16	TEC
ENSG00000164438.5	-8.6920113	3.19744657	1.92E-62	5.54E-60	9.76	10.14	0	0.05	TLX3	T-cell leukemia homeobox 3	chr5	protein_coding
ENSG00000135862.5	-1.4620318	8.99622886	4.51E-07	2.75E-06	69.04	84.92	35	39.5	LAMC1	laminin, gamma 1 (formerly LAMB2)	chr1	protein_coding
ENSG00000130287.12	-2.4282933	-1.6601404	0.00030126	0.00109685	0.07	0.04	0.02	0.01	NCAN	neurocan	chr19	protein_coding
ENSG00000249459.7	2.96625556	-1.3592662	9.86E-06	4.79E-05	0.01	0.02	0.17	0.06	ZNF286B	zinc finger protein 286B	chr17	protein_coding
ENSG00000185669.5	2.17626927	-2.5817366	0.01923175	0.0458365	0	0.03	0.22	0	SNAI3	snail family zinc finger 3	chr16	protein_coding
ENSG00000155858.5	-1.1241261	1.96343814	0.0003773	0.00135076	0.63	0.68	0.25	0.55	LSM11	LSM11, U7 small nuclear RNA associated	chr5	protein_coding
ENSG00000123600.17	1.43931803	1.85140648	1.09E-05	5.27E-05	0.16	0.21	0.36	0.98	METTL8	methyltransferase like 8	chr2	protein_coding
ENSG00000137699.15	3.11852816	7.11785766	5.18E-24	1.67E-22	4.45	4.18	53.24	46.51	TRIM29	tripartite motif containing 29	chr11	protein_coding
ENSG00000167769.4	4.87917439	-3.0678699	0.01889873	0.04519557	0	0	0	0.15	ACER1	alkaline ceramidase 1	chr19	protein_coding
ENSG00000105329.8	-1.4092632	6.92460787	1.19E-06	6.74E-06	52.12	51.07	28.89	22.72	TGFB1	transforming growth factor, beta 1	chr19	protein_coding
ENSG00000173727.10	-3.6100469	1.31246393	7.47E-20	1.78E-18	5.07	3.69	0.61	0.35	CMB9-22P13	--	chr11	lincRNA
ENSG00000110723.10	8.26902875	1.74068172	5.89E-40	4.43E-38	0	0	0.57	0.79	EXPH5	exophilin 5	chr11	protein_coding
ENSG00000105928.12	-1.0586021	3.67071984	0.00035302	0.00127122	5.91	4.22	3.24	3.25	DFNA5	deafness, autosomal dominant 5	chr7	protein_coding
ENSG00000196218.10	4.39366316	6.12930126	4.54E-40	3.46E-38	0.32	0.34	9.95	8.71	RYR1	ryanodine receptor 1 (skeletal)	chr19	protein_coding
ENSG00000162694.12	1.31477352	1.57942867	8.45E-05	0.00034323	0.17	0.51	0.71	1.5	EXTL2	exostosin-like glycosyltransferase 2	chr1	protein_coding
ENSG00000250479.7	-1.5074374	7.98987821	2.08E-07	1.34E-06	234.43	276.9	136.92	101.55	CHCHD10	coiled-coil-helix-coiled-coil-helix domain containing 10	chr22	protein_coding
ENSG00000148841.14	-2.284954	6.44796797	1.99E-14	2.99E-13	16.87	16.52	4.78	4.33	ITPRIP	inositol 1,4,5-trisphosphate receptor interacting protein	chr10	protein_coding
ENSG00000145734.17	4.0550821	2.21414721	1.67E-26	6.41E-25	0.05	0.03	0.65	1.02	BDP1	B double prime 1, subunit of RNA polymerase III transcription initiation factor IIIB	chr5	protein_coding
ENSG00000121281.11	-5.7022721	4.22982997	5.18E-52	7.96E-50	4.52	4.98	0.11	0.13	ADCY7	adenylate cyclase 7	chr16	protein_coding
ENSG00000006468.12	-6.0798457	-0.6909776	3.46E-16	6.11E-15	0.1	0.14	0	0	ETV1	ets variant 1	chr7	protein_coding
ENSG00000234284.5	2.0735054	-2.2724913	0.01324986	0.0331641	0.02	0	0.01	0.13	ZNF879	zinc finger protein 879	chr5	protein_coding
ENSG00000089597.15	-1.0770927	10.5705584	0.00017454	0.00066585	417.89	413.85	263.14	262.06	GANAB	glucosidase, alpha; neutral AB	chr11	protein_coding
ENSG00000004478.7	-1.6487467	9.0555606	1.51E-08	1.13E-07	176.2	169.09	72.63	74.14	FKBP4	FK506 binding protein 4, 59kDa	chr12	protein_coding
ENSG00000106153.12	-1.2720705	8.15871892	1.04E-05	5.03E-05	384.14	341.77	197.6	203.23	CHCHD2	coiled-coil-helix-coiled-coil-helix domain containing 2	chr7	protein_coding
ENSG00000184261.4	5.35906543	-2.8049519	0.00290522	0.00856312	0	0	0.04	0.01	KCNK12	potassium channel, two pore domain subfamily K, member 12	chr2	protein_coding
ENSG00000165959.10	2.44599817	2.38861702	1.84E-13	2.49E-12	0.06	0.14	0.78	0.66	CLMN	calmin (calponin-like, transmembrane)	chr14	protein_coding
ENSG00000120833.12	3.03443734	0.45070164	3.05E-12	3.63E-11	0.02	0.05	0.38	0.39	SOCS2	suppressor of cytokine signaling 2	chr12	protein_coding
ENSG00000160678.10	-2.8733041	-2.1197356	0.0002605	0.00096399	0.32	0.2	0.03	0.05	S100A1	S100 calcium binding protein A1	chr1	protein_coding
ENSG00000277440.1	2.72210398	-2.2552204	0.00281991	0.00840887	0	0.1	0.5	0.42	RP11-295D4	--	chr16	sense_intronic
ENSG00000163104.16	1.82781073	0.5762941	2.00E-06	1.10E-05	0.09	0.09	0.23	0.62	SMARCAD1	SWI/SNF-related, matrix-associated actin-dependent regulator of chromatin, subfamily a, containing DEAD/H box 1	chr4	protein_coding
ENSG00000137809.15	-4.8774757	4.17886216	3.93E-43	3.58E-41	2.68	2.9	0.11	0.14	ITGA11	integrin, alpha 11	chr15	protein_coding
ENSG00000178295.13	2.30925537	0.6147698	8.52E-09	6.59E-08	0.02	0.06	0.15	0.37	GEN1	GEN1 Holliday junction 5' flap endonuclease	chr2	protein_coding
ENSG00000015285.9	2.18202121	0.99215388	4.64E-09	3.72E-08	0.23	0.37	1.96	1.66	WAS	Wiskott-Aldrich syndrome	chrX	protein_coding
ENSG00000230699.2	2.00021504	1.83052484	2.96E-09	2.44E-08	0.31	0.43	1.78	2.15	RP11-54O7.1	--	chr1	lincRNA
ENSG00000153339.12	3.29961508	0.59557437	5.03E-14	7.24E-13	0.02	0.04	0.18	0.59	TRAPPC8	trafficking protein particle complex 8	chr18	protein_coding
ENSG00000154864.10	-8.3707774	2.35264898	6.11E-52	9.28E-50	0.66	1.01	0	0.01	PIEZO2	piezo-type mechanosensitive ion channel component 2	chr18	protein_coding
ENSG00000279557.1	-1.696976	-2.1259992	0.02035181	0.04824221	0.11	0.26	0.05	0.09	CTD-2210P1	--	chr5	TEC
ENSG00000066827.14	-1.1784165	4.29175977	6.13E-05	0.00025765	4.3	4.7	2.63	2.66	ZFAT	zinc finger and AT hook domain containing	chr8	protein_coding
ENSG00000111731.11	1.48830779	1.11381299	2.20E-05	0.00010032	0.19	0.17	0.41	0.95	C2CD5	C2 calcium-dependent domain containing 5	chr12	protein_coding
ENSG00000182400.13	2.62509745	1.71706256	1.12E-13	1.55E-12	0.23	0.2	1.31	2.26	TRAPPC6B	trafficking protein particle complex 6B	chr14	protein_coding
ENSG00000120875.7	-1.1272795	6.02793351	9.58E-05	0.00038697	10.26	9.7	5.92	6.27	DUSP4	dual specificity phosphatase 4	chr8	protein_coding
ENSG00000272563.1	5.32454168	-2.8239292	0.00290522	0.00856312	0	0	0.01	0.08	RP11-480C1	--	chr2	lincRNA
ENSG00000140022.8	2.20623622	1.54536401	2.94E-10	2.76E-09	0.2	0.16	1.18	1.05	STON2	stonin 2	chr14	protein_coding
ENSG00000159256.11	3.10644053	0.12057511	2.14E-11	2.30E-10	0.08	0	0.43	0.47	MORC3	MORC family CW-type zinc finger 3	chr21	protein_coding
ENSG00000268756.1	6.77468854	-1.827536	1.35E-07	8.87E-07	0	0	0.85	0.72	AC104534.2	--	chr19	antisense
ENSG00000248890.1	-7.6014249	-1.3250822	2.11E-11	2.27E-10	0.43	0.33	0	0	HHIP-AS1	HHIP antisense RNA 1	chr4	antisense
ENSG00000197442.9	2.05328773	2.15667334	4.64E-10	4.23E-09	0.21	0.32	1	1.92	MAP3K5	mitogen-activated protein kinase kinase kinase 5	chr6	protein_coding
ENSG00000137727.11	7.06681771	-1.59382	7.18E-09	5.62E-08	0	0	0.07	0.08	ARHGAP20	Rho GTPase activating protein 20	chr11	protein_coding
ENSG00000055044.9	1.37178615	4.72075025	2.99E-06	1.61E-05	4.51	7.11	14.31	25.94	NOP58	NOP58 ribonucleoprotein	chr2	protein_coding
ENSG00000152465.16	1.21392488	2.94742247	6.95E-05	0.00028893	0.94	1.59	3.55	4.26	NMT2	N-myristoyltransferase 2	chr10	protein_coding
ENSG00000172985.10	-3.8099655	5.2802871	6.52E-32	3.28E-30	9.85	10.4	1.02	0.91	SH3RF3	SH3 domain containing ring finger 3	chr2	protein_coding
ENSG00000128383.11	-5.1447423	0.08489614	1.35E-20	3.41E-19	0.82	0.93	0	0.06	APOBEC3A	apolipoprotein B mRNA editing enzyme, catalytic polypeptide-like 3A	chr22	protein_coding
ENSG00000225578.1	3.43130224	-2.4109684	0.00112121	0.00364442	0	0.05	0.63	0.24	NCBP2-AS1	NCBP2 antisense RNA 1	chr3	antisense
ENSG00000118564.13	1.90481434	2.26254481	4.47E-09	3.59E-08	0.4	0.55	1.76	2.98	FBXL5	F-box and leucine-rich repeat protein 5	chr4	protein_coding
ENSG00000256751.4	2.7063559	-2.2659701	0.00281991	0.00840887	0.09	0	0.27	0.59	PLBD1-AS1	PLBD1 antisense RNA 1	chr12	antisense
ENSG00000262533.1	2.44405374	-2.4283546	0.01191236	0.03011315	0.13	0	0.23	0.77	RP11-667K1	--	chr17	antisense
ENSG00000165912.14	-2.8652682	7.91556626	5.32E-21	1.38E-19	170.68	159.3	30.91	29.4	PACSIN3	protein kinase C and casein kinase substrate in neurons 3	chr11	protein_coding
ENSG00000272277.1	1.28398617	2.07238641	5.78E-05	0.00024369	2.33	2.23	7.95	6.86	RP1-40E16.1	--	chr6	antisense
ENSG00000007384.14	-1.6868224	4.30523514	1.58E-08	1.17E-07	8.5	7.8	3.43	3.32	RHBDF1	rhomboid 5 homolog 1 (Drosophila)	chr16	protein_coding
ENSG00000166819.10	3.35429375	-0.1929112	1.01E-10	1.01E-09	0.05	0.02	0.41	0.62	PLIN1	perilipin 1	chr15	protein_coding
ENSG00000235663.1	-3.780094	-1.886657	1.61E-05	7.52E-05	1.04	0.43	0.14	0	SAPCD1-AS1	SAPCD1 antisense RNA 1	chr6	antisense
ENSG00000170832.11	1.02957721	3.21718689	0.00061062	0.00210284	0.77	0.88	2.09	2.41	USP32	ubiquitin specific peptidase 32	chr17	protein_coding
ENSG00000125538.10	-6.4241717	0.4278551	1.07E-25	3.85E-24	1.43	1.2	0	0.03	IL1B	interleukin 1, beta	chr2	protein_coding
ENSG00000188051.6	2.77977433	0.45677564	4.55E-11	4.74E-10	0.21	0.06	1.51	0.99	TMEM221	transmembrane protein 221	chr19	protein_coding
ENSG00000253848.1	3.77717563	-2.1758498	0.00013996	0.00054609	0	0.05	0.24	0.87	RP11-10N23	--	chr8	antisense
ENSG00000068305.16	2.44315936	2.35238507	1.99E-13	2.68E-12	0.19	0.23	1.27	1.79	MEF2A	myocyte enhancer factor 2A	chr15	protein_coding
ENSG00000173702.6	4.59487284	0.00604901	4.62E-16	8.09E-15	0	0.04	0.46	0.84	MUC13	mucin 13, cell surface associated	chr3	protein_coding
ENSG00000173868.10	-1.2310274	-0.611899	0.00963638	0.02512271	0.2	0.44	0.28	0.09	PHOSPHO1	phosphatase, orphan 1	chr17	protein_coding
ENSG00000166333.12	-1.2392144	7.02751354	1.78E-05	8.26E-05	70.22	58.63	37.64	35.07	ILK	integrin-linked kinase	chr11	protein_coding
ENSG00000162267.11	-4.8079387	-3.2306674	0.01889873	0.04519557	0.02	0.04	0	0	ITIH3	inter-alpha-trypsin inhibitor heavy chain 3	chr3	protein_coding
ENSG00000187790.9	3.89480711	0.10465072	4.21E-14	6.11E-13	0.01	0.02	0.16	0.38	FANCM	Fanconi anemia, complementation group M	chr14	protein_coding
ENSG00000092607.12	-3.8774613	-0.1477219	1.85E-14	2.79E-13	0.27	0.46	0.03	0.03	TBX15	T-box 15	chr1	protein_coding
ENSG00000267858.4	1.46198256	2.74882617	2.50E-06	1.36E-05	0.78	1.15	3.72	3.34	MZF1-AS1	MZF1 antisense RNA 1	chr19	antisense
ENSG00000162191.12	-1.339698	9.18677647	3.51E-06	1.86E-05	462.2	538.39	276.64	248.89	UBXN1	UBX domain protein 1	chr11	protein_coding
ENSG00000226752.6	-1.4805531	2.27376977	2.98E-06	1.60E-05	0.7	1.11	0.51	0.35	PSMD5-AS1	PSMD5 antisense RNA 1 (head to head)	chr9	antisense
ENSG00000121940.14	1.41223185	4.3499862	1.84E-06	1.02E-05	1.63	1.99	5.74	7.12	CLCC1	chloride channel CLIC-like 1	chr1	protein_coding
ENSG00000130748.6	-1.0111022	4.1565545	0.00056718	0.00196567	33.44	24.58	20.1	18.28	TMEM160	transmembrane protein 160	chr19	protein_coding
ENSG00000145431.9	-5.8827486	-0.0865332	3.11E-20	7.60E-19	0.25	0.46	0.01	0.01	PDGFC	platelet derived growth factor C	chr4	protein_coding
ENSG00000185112.5	-3.0039154	5.0804477	5.43E-22	1.52E-20	18.67	20.42	3.22	3.26	FAM43A	family with sequence similarity 43, member A	chr3	protein_coding
ENSG00000169189.15	-1.8443102	6.13579331	3.75E-10	3.49E-09	90.01	76.76	30.89	31.03	NSMCE1	non-SMC element 1 homolog (S. cerevisiae)	chr16	protein_coding
ENSG00000265107.2	9.34319399	0.42119022	8.81E-25	3.00E-23	0	0	0.57	1.03	GJA5	gap junction protein, alpha 5, 40kDa	chr1	protein_coding
ENSG00000207501.1	5.80647709	-2.5280583	0.00026535	0.0009747	0	0	2.34	0.5	RNVU1-14	RNA, variant U1 small nuclear 14	chr1	snRNA
ENSG00000104375.14	1.22743305	1.24205116	0.0003318	0.0011995	0.28	0.38	0.8	1.27	STK3	serine/threonine kinase 3	chr8	protein_coding
ENSG00000115041.11	-4.6769548	2.37696598	6.40E-34	3.56E-32	2.2	2.65	0.07	0.18	KCNIP3	Kv channel interacting protein 3, calsenilin	chr2	protein_coding
ENSG00000205269.5	2.21808907	-1.6447292	0.00158671	0.00496952	0.01	0	0.05	0.05	TMEM170B	transmembrane protein 170B	chr6	protein_coding
ENSG00000147118.9	4.61147192	1.0797929	2.22E-23	6.81E-22	0.02	0.05	0.75	1.56	ZNF182	zinc finger protein 182	chrX	protein_coding
ENSG00000161249.19	7.35656433	4.89920568	7.61E-70	3.09E-67	0.13	0.08	23.21	22.79	DMKN	dermokine	chr19	protein_coding
ENSG00000102547.17	1.83407588	0.24056568	6.19E-06	3.13E-05	0.05	0.13	0.45	0.41	CAB39L	calcium binding protein 39-like	chr13	protein_coding
ENSG00000086696.9	2.63087269	1.45870037	4.14E-13	5.40E-12	0.31	0.51	3.7	3.05	HSD17B2	hydroxysteroid (17-beta) dehydrogenase 2	chr16	protein_coding
ENSG00000095380.10	-1.0850229	7.69673337	0.00016077	0.00061833	193.89	196.37	125.28	119.63	NANS	N-acetylneuraminic acid synthase	chr9	protein_coding
ENSG00000114948.11	1.12800334	0.11731322	0.00497899	0.01399254	0.06	0.1	0.18	0.28	ADAM23	ADAM metallopeptidase domain 23	chr2	protein_coding
ENSG00000046651.13	2.49852743	2.92135065	1.19E-14	1.84E-13	0.29	0.69	2.63	4.73	OFD1	oral-facial-digital syndrome 1	chrX	protein_coding
ENSG00000170417.13	3.70150351	-1.5621186	8.20E-06	4.05E-05	0.01	0	0.12	0.16	TMEM182	transmembrane protein 182	chr2	protein_coding
ENSG00000085365.16	1.644214	1.26417353	2.26E-06	1.23E-05	0.28	0.11	0.6	1.08	SCAMP1	secretory carrier membrane protein 1	chr5	protein_coding
ENSG00000260361.1	1.24867193	0.07551073	0.00173512	0.00539487	0.35	0.18	0.96	0.75	CTD-2313J1	--	chr15	sense_intronic
ENSG00000279113.1	6.30985008	-2.1747134	8.95E-06	4.38E-05	0	0	0.45	1.02	RP13-895J2.8	--	chr12	TEC
ENSG00000177410.11	-1.6273809	6.97933371	2.44E-08	1.76E-07	74.06	63.45	28.39	31.02	ZFAS1	ZNFX1 antisense RNA 1	chr20	antisense
ENSG00000268069.2	-2.8613113	-0.0716644	3.90E-10	3.61E-09	1.58	0.84	0.33	0.13	RP5-1057I20	--	chr12	antisense
ENSG00000184867.12	-12.055571	3.00419627	2.59E-61	6.91E-59	4.06	4.67	0	0	ARMCX2	armadillo repeat containing, X-linked 2	chrX	protein_coding
ENSG00000074964.15	-1.6890219	5.32413992	9.53E-09	7.32E-08	11.33	9.74	4.6	4.1	ARHGEF10L	Rho guanine nucleotide exchange factor (GEF) 10-like	chr1	protein_coding
ENSG00000146676.6	1.31404181	2.69050761	2.10E-05	9.61E-05	0.27	0.38	0.83	1.34	PURB	purine-rich element binding protein B	chr7	protein_coding
ENSG00000115020.15	1.76208919	0.97223817	1.35E-06	7.61E-06	0.07	0.06	0.26	0.33	PIKFYVE	phosphoinositide kinase, FYVE finger containing	chr2	protein_coding
ENSG00000175548.7	2.26262576	0.11834373	1.30E-07	8.60E-07	0.03	0.02	0.11	0.24	ALG10B	ALG10B, alpha-1,2-glucosyltransferase	chr12	protein_coding
ENSG00000143036.15	4.24783121	0.21094313	7.05E-16	1.21E-14	0.02	0.05	0.81	0.93	SLC44A3	solute carrier family 44, member 3	chr1	protein_coding
ENSG00000271980.1	3.33853663	-2.4705837	0.00190233	0.00584723	0.01	0	0.13	0.05	CTD-2256P1	--	chr5	antisense
ENSG00000255693.1	5.45974547	-2.7440718	0.00158008	0.00495521	0	0	0.04	0.06	RP11-766N7	--	chr12	lincRNA
ENSG00000008086.9	1.05106717	-0.305718	0.01788526	0.04345686	0.02	0.04	0.08	0.08	CDKL5	cyclin-dependent kinase-like 5	chrX	protein_coding
ENSG00000169914.5	1.05057582	2.62312888	0.00063642	0.00218322	0.41	0.6	1.47	1.29	OTUD3	OTU deubiquitinase 3	chr1	protein_coding
ENSG00000232677.5	6.06205277	2.72635488	1.84E-43	1.74E-41	0.01	0.1	4.5	5.61	LINC00665	long intergenic non-protein coding RNA 665	chr19	lincRNA
ENSG00000102359.5	-2.6358462	0.20078078	7.67E-10	6.78E-09	0.9	0.56	0.28	0.04	SRPX2	sushi-repeat containing protein, X-linked 2	chrX	protein_coding
ENSG00000275713.1	2.34836927	-0.3968548	8.80E-07	5.11E-06	0.91	0	5.21	1.29	HIST1H2BH	histone cluster 1, H2bh	chr6	protein_coding
ENSG00000112541.12	-3.7768476	-0.8421459	3.22E-10	3.01E-09	0.1	0.06	0.01	0.01	PDE10A	phosphodiesterase 10A	chr6	protein_coding
ENSG00000165417.10	3.03718171	2.50655393	5.73E-19	1.28E-17	0.18	0.13	1.27	2.1	GTF2A1	general transcription factor IIA, 1, 19/37kDa	chr14	protein_coding
ENSG00000261780.2	-8.2706839	3.1647912	1.50E-60	3.82E-58	12.95	11.94	0.03	0.07	CTD-2354A1	--	chr18	processed_transcript
ENSG00000151690.13	1.86614297	2.31299845	6.84E-09	5.37E-08	0.33	0.36	1.17	2.23	MFSD6	major facilitator superfamily domain containing 6	chr2	protein_coding
ENSG00000223865.9	3.27948614	-1.8731736	0.00025363	0.00094222	0	0.04	0.16	0.33	HLA-DPB1	major histocompatibility complex, class II, DP beta 1	chr6	protein_coding
ENSG00000139190.15	1.47907159	4.55099135	5.61E-07	3.36E-06	3.3	2.47	10.49	11.02	VAMP1	vesicle-associated membrane protein 1 (synaptobrevin 1)	chr12	protein_coding
ENSG00000165923.14	3.56706626	-0.8837769	1.56E-08	1.16E-07	0.03	0	0.24	0.23	AGBL2	ATP/GTP binding protein-like 2	chr11	protein_coding
ENSG00000197774.11	1.53064222	0.68551081	3.84E-05	0.00016787	0.56	0.46	2.13	1.83	EME2	essential meiotic structure-specific endonuclease subunit 2	chr16	protein_coding
ENSG00000131263.11	1.96738217	3.33814421	1.92E-10	1.85E-09	0.43	0.36	1.96	2.15	RLIM	ring finger protein, LIM domain interacting	chrX	protein_coding
ENSG00000151414.13	5.2256525	0.57926797	3.15E-21	8.39E-20	0.01	0.01	0.26	1.08	NEK7	NIMA-related kinase 7	chr1	protein_coding
ENSG00000169592.13	-1.6192473	6.76177268	2.89E-08	2.08E-07	63.91	58.42	27.97	25.03	INO80E	INO80 complex subunit E	chr16	protein_coding
ENSG00000117643.13	1.50283863	0.57626357	7.29E-05	0.00030168	0.11	0.11	0.37	0.48	MAN1C1	mannosidase, alpha, class 1C, member 1	chr1	protein_coding
ENSG00000198797.6	-6.5105516	-2.2106815	3.03E-06	1.62E-05	0.05	0.11	0	0	BRINP2	bone morphogenetic protein/retinoic acid inducible neural-specific 2	chr1	protein_coding
ENSG00000156414.17	-10.954497	1.90180726	9.95E-46	1.07E-43	1.11	1.43	0	0	TDRD9	tudor domain containing 9	chr14	protein_coding
ENSG00000280381.1	2.80199254	-0.6436416	2.63E-07	1.65E-06	0	0.04	0.21	0.17	RP11-197N1	--	chr12	TEC
ENSG00000205436.6	2.80582965	-0.0224471	1.19E-09	1.02E-08	0.1	0.05	0.81	0.67	EXOC3L4	exocyst complex component 3-like 4	chr14	protein_coding
ENSG00000177508.11	-1.4994891	6.53631611	2.58E-07	1.63E-06	38.69	46.74	20.96	19.21	IRX3	iroquois homeobox 3	chr16	protein_coding
ENSG00000164099.3	-4.3605454	3.4662129	2.55E-35	1.52E-33	3.8	3.3	0.14	0.32	PRSS12	protease, serine, 12 (neurotrypsin, motopsin)	chr4	protein_coding
ENSG00000159792.8	1.17248225	5.29689967	5.48E-05	0.00023179	5.37	4.73	16.01	14.29	PSKH1	protein serine kinase H1	chr16	protein_coding
ENSG00000146733.12	-1.3481173	5.96507568	3.47E-06	1.84E-05	30.86	35.83	16.02	18.92	PSPH	phosphoserine phosphatase	chr7	protein_coding
ENSG00000169856.8	5.34420401	-2.8131047	0.00290522	0.00856312	0	0	0.06	0.06	ONECUT1	one cut homeobox 1	chr15	protein_coding
ENSG00000082153.16	1.75508405	5.48321787	2.63E-09	2.18E-08	2.32	2.31	7.02	13.97	BZW1	basic leucine zipper and W2 domains 1	chr2	protein_coding
ENSG00000260261.1	1.62212372	0.99775424	5.64E-06	2.87E-05	0.22	0.21	1.1	0.67	RP11-480A1	--	chr3	lincRNA
ENSG00000070182.16	4.47520707	1.72839578	6.26E-27	2.47E-25	0.03	0.01	0.62	0.64	SPTB	spectrin, beta, erythrocytic	chr14	protein_coding
ENSG00000069956.10	3.07316509	2.47699768	3.70E-19	8.39E-18	0.21	0.14	1.47	2.48	MAPK6	mitogen-activated protein kinase 6	chr15	protein_coding
ENSG00000280161.1	3.33429521	0.31809698	3.17E-13	4.18E-12	0.22	0.12	2.15	2.44	CTC-205M6.	--	chr5	TEC
ENSG00000259462.2	4.44788234	-0.9589179	9.89E-10	8.68E-09	0.01	0	0.13	0.22	CPEB1-AS1	CPEB1 antisense RNA 1	chr15	antisense
ENSG00000260894.1	1.95424316	-0.4966423	3.75E-05	0.00016418	1.16	0.82	4.33	6.05	CTD-2012K1	--	chr16	antisense
ENSG00000157423.16	11.4583304	2.44512366	3.17E-51	4.55E-49	0	0	0.66	0.66	HYDIN	HYDIN, axonemal central pair apparatus protein	chr16	protein_coding
ENSG00000041515.14	-2.1724363	-1.22841	0.00015583	0.00060009	0.05	0.08	0.02	0.01	MYO16	myosin XVI	chr13	protein_coding
ENSG00000136828.17	1.21597688	2.03520733	0.00014518	0.00056506	0.24	0.21	0.57	0.82	RALGPS1	Ral GEF with PH domain and SH3 binding motif 1	chr9	protein_coding
ENSG00000275198.1	6.20351375	-2.2553835	1.55E-05	7.26E-05	0	0	0.08	0.05	RP11-471B2	--	chr14	lincRNA
ENSG00000144445.14	3.21421327	-1.4870086	2.06E-05	9.41E-05	0.01	0.01	0.05	0.17	KANSL1L	KAT8 regulatory NSL complex subunit 1-like	chr2	protein_coding
ENSG00000161243.7	7.01108703	3.01857158	3.08E-51	4.45E-49	0.04	0.03	7.62	6.24	FBXO27	F-box protein 27	chr19	protein_coding
ENSG00000137693.12	1.67833508	5.53911749	1.14E-08	8.68E-08	3.23	3.12	11.24	15.97	YAP1	Yes-associated protein 1	chr11	protein_coding
ENSG00000147100.8	5.07746812	1.16411543	6.76E-26	2.47E-24	0.04	0	1.07	1.07	SLC16A2	solute carrier family 16, member 2 (thyroid hormone transporter)	chrX	protein_coding
ENSG00000175536.6	1.25938384	1.56014426	0.00013829	0.00054099	1.16	1.53	4.78	3.77	LIPT2	lipoyl(octanoyl) transferase 2 (putative)	chr11	protein_coding
ENSG00000085185.14	-1.5836013	4.44817752	9.17E-08	6.17E-07	3.68	3.59	1.61	1.62	BCORL1	BCL6 corepressor-like 1	chrX	protein_coding

ENSG00000226979.7	-4.8079387	-3.2306674	0.01889873	0.04519557	0.03	0.07	0	0	LTA	lymphotoxin alpha	chr6	protein_coding
ENSG00000169991.9	-1.2823956	3.48872801	1.87E-05	8.64E-05	2.11	2.04	1.33	0.94	IFFO2	intermediate filament family orphan 2	chr1	protein_coding
ENSG00000204956.5	1.04933495	3.48365306	0.00043902	0.00155201	1.01	0.87	2.74	2.42	PCDHGA1	protocadherin gamma subfamily A, 1	chr5	protein_coding
ENSG00000183111.10	2.01437528	3.0389805	1.25E-10	1.23E-09	0.66	0.39	2.55	3.11	ARHGEF37	Rho guanine nucleotide exchange factor (GEF) 37	chr5	protein_coding
ENSG00000138802.10	1.39823927	1.57215357	3.09E-05	0.00013712	0.22	0.27	0.57	1.16	SEC24B	SEC24 family member B	chr4	protein_coding
ENSG00000275052.3	2.06322319	2.76811637	8.93E-11	8.98E-10	0.36	0.4	1.55	2.72	SMEK2	SMEK homolog 2, suppressor of mek1 (Dictyostelium)	chr2	protein_coding
ENSG00000226644.4	10.3822765	1.40165827	7.95E-37	5.06E-35	0	0	9.98	10.31	RP11-128M1	--	chr20	lincRNA
ENSG00000278600.1	1.15547669	0.67561225	0.00150326	0.00477425	0.25	0.43	0.79	1.2	RP11-81A1.6	--	chr15	sense_intronic
ENSG00000145861.7	2.65877901	0.29013827	7.32E-10	6.49E-09	0.09	0.12	1.05	0.7	C1QTNF2	C1q and tumor necrosis factor related protein 2	chr5	protein_coding
ENSG00000153558.12	1.47534278	0.12068025	0.00027	0.00099058	0.06	0.17	0.27	0.55	FBXL2	F-box and leucine-rich repeat protein 2	chr3	protein_coding
ENSG00000081307.11	1.05798755	2.30231027	0.00072854	0.00246952	0.59	0.56	1.26	1.94	UBA5	ubiquitin-like modifier activating enzyme 5	chr3	protein_coding
ENSG00000004799.7	-11.971667	2.92002969	4.24E-60	1.01E-57	2.86	4.03	0	0	PDK4	pyruvate dehydrogenase kinase, isozyme 4	chr7	protein_coding
ENSG00000114978.16	2.09031812	3.28554285	2.04E-11	2.21E-10	0.51	0.7	2.37	4.58	MOB1A	MOB kinase activator 1A	chr2	protein_coding
ENSG00000102048.14	-1.2308425	0.803324	0.00038282	0.00136948	0.82	1.07	0.3	0.77	ASB9	ankyrin repeat and SOCS box containing 9	chrX	protein_coding
ENSG00000260898.4	5.84975207	-2.4948111	0.00026535	0.0009747	0	0	0.02	0.12	ADPGK-AS1	ADPGK antisense RNA 1	chr15	antisense
ENSG00000224376.1	-2.7901595	-2.1675702	0.0014675	0.00467247	0.45	0.24	0.09	0.04	AC017104.6	--	chr2	processed_transcript
ENSG00000123838.9	-6.576035	-2.1610225	1.78E-06	9.85E-06	0.14	0.11	0	0	C4BPA	complement component 4 binding protein, alpha	chr1	protein_coding
ENSG00000082482.12	-2.0978179	1.16361448	2.25E-09	1.88E-08	0.78	0.84	0.15	0.35	KCNK2	potassium channel, two pore domain subfamily K, member 2	chr1	protein_coding
ENSG00000214513.3	7.55588405	-1.1880946	2.11E-11	2.27E-10	0	0	0.27	0.29	NOTO	notochord homeobox	chr2	protein_coding
ENSG00000105996.6	2.29343573	-2.5181978	0.01191236	0.03011315	0.03	0	0.23	0	HOXA2	homeobox A2	chr7	protein_coding
ENSG00000075089.8	3.03602171	1.16798566	9.82E-15	1.53E-13	0.08	0.21	0.82	2.34	ACTR6	ARP6 actin-related protein 6 homolog (yeast)	chr12	protein_coding
ENSG00000104522.14	-1.1323374	6.74877804	8.59E-05	0.0003489	93.17	76.48	53.65	49.45	TSTA3	tissue specific transplantation antigen P35B	chr8	protein_coding
ENSG00000092531.8	1.13059655	2.77101631	0.00022932	0.00085855	0.94	1.15	2.25	3.88	SNAP23	synaptosomal-associated protein, 23kDa	chr15	protein_coding
ENSG00000174574.14	1.26199405	4.64640627	1.66E-05	7.74E-05	4.1	4.58	11.63	16.2	AKIRIN1	akirin 1	chr1	protein_coding
ENSG00000134419.14	-1.6008174	9.96734565	3.68E-08	2.60E-07	280.36	280.14	129.67	116.15	RPS15A	ribosomal protein S15a	chr16	protein_coding
ENSG00000090857.12	1.44292777	5.89923015	7.41E-07	4.35E-06	2.58	2.98	10.45	9.66	PDPR	pyruvate dehydrogenase phosphatase regulatory subunit	chr16	protein_coding
ENSG00000153898.11	-2.5942685	-1.5572507	6.51E-05	0.00027249	0.11	0.17	0.02	0.04	MCOLN2	mucolipin 2	chr1	protein_coding
ENSG00000198471.1	5.2326735	-2.8777865	0.00290522	0.00856312	0	0	0.21	0.02	RTP2	receptor (chemosensory) transporter protein 2	chr3	protein_coding
ENSG00000167476.9	2.98742783	0.31740576	1.87E-11	2.03E-10	0.21	0.15	2.28	1.6	JSRP1	junctional sarcoplasmic reticulum protein 1	chr19	protein_coding
ENSG00000270482.1	3.21959301	-2.5427774	0.003237	0.00945156	0.04	0	0.26	0.4	RP11-131L1	--	chr12	lincRNA
ENSG00000103449.10	-10.524579	1.47255673	5.56E-40	4.19E-38	0.8	0.88	0	0	SALL1	spalt-like transcription factor 1	chr16	protein_coding
ENSG00000136754.15	1.2097012	2.86371874	7.70E-05	0.00031789	0.9	0.96	1.98	3.79	ABI1	abl-interactor 1	chr10	protein_coding
ENSG00000115339.12	-2.2224352	-2.5162216	0.01191236	0.03011315	0.08	0	0	0.02	GALNT3	polypeptide N-acetylgalactosaminyltransferase 3	chr2	protein_coding
ENSG00000136950.12	-1.5650928	6.80868418	7.80E-08	5.32E-07	61.4	46.67	23.32	25.46	ARPC5L	actin related protein 2/3 complex, subunit 5-like	chr9	protein_coding
ENSG00000184979.9	4.58152399	3.11590691	2.71E-35	1.61E-33	0.2	0.32	8.15	8.47	USP18	ubiquitin specific peptidase 18	chr22	protein_coding
ENSG00000196850.5	1.90708864	1.09285766	1.30E-07	8.59E-07	0.07	0.21	0.45	0.94	PPTC7	PTC7 protein phosphatase homolog (S. cerevisiae)	chr12	protein_coding
ENSG00000262222.1	1.39134628	-0.80759	0.00536284	0.01488851	0.1	0.11	0.21	0.51	RP11-876N2	--	chr16	antisense
ENSG00000264324.1	-1.2629805	6.3480442	1.28E-05	6.11E-05	19.28	17.5	10.02	10.42	RP11-287D1	--	chr2	protein_coding
ENSG00000280331.1	4.8843127	-3.0650858	0.01889873	0.04519557	0	0	0.18	1.08	RP11-545D2	--	chr11	TEC
ENSG00000137500.8	1.68005813	2.29385359	1.62E-07	1.05E-06	0.33	0.44	1.08	2.23	CCDC90B	coiled-coil domain containing 90B	chr11	protein_coding
ENSG00000106415.11	3.12170581	0.60501781	2.85E-13	3.78E-12	0.05	0.05	0.49	0.67	GLCCI1	glucocorticoid induced 1	chr7	protein_coding
ENSG00000110852.4	3.51811437	0.10258745	9.44E-13	1.19E-11	0.12	0	0.39	1.48	CLEC2B	C-type lectin domain family 2, member B	chr12	protein_coding
ENSG00000237857.2	-1.3081198	0.15472845	0.00061313	0.00210994	0.72	1.05	0.32	0.62	RP11-435O5	--	chr9	lincRNA
ENSG00000148120.13	-1.0603055	5.37064956	0.00025103	0.00093383	14.68	12.67	8.37	9.13	C9orf3	chromosome 9 open reading frame 3	chr9	protein_coding
ENSG00000148730.6	1.25615055	4.92921925	1.71E-05	7.95E-05	1.92	2.08	6.26	6.48	EIF4EBP2	eukaryotic translation initiation factor 4E binding protein 2	chr10	protein_coding
ENSG00000166801.14	5.37700236	2.90103968	1.60E-40	1.26E-38	0.02	0.07	2.05	2.51	FAM111A	family with sequence similarity 111, member A	chr11	protein_coding
ENSG00000169251.11	1.95824608	2.2609191	1.80E-09	1.52E-08	0.2	0.54	1.32	2.49	NMD3	NMD3 ribosome export adaptor	chr3	protein_coding
ENSG00000127241.15	-3.9774295	0.08757858	6.40E-16	1.10E-14	0.19	0.17	0.01	0.02	MASP1	mannan-binding lectin serine peptidase 1 (C4/C2 activating component of Ra-reactive factor)	chr3	protein_coding
ENSG00000163806.14	3.25115145	-1.8968943	0.00025363	0.00094222	0	0.03	0.24	0.18	SPDYA	speedy/RINGO cell cycle regulator family member A	chr2	protein_coding
ENSG00000170889.12	-1.3593666	9.4940235	2.54E-06	1.38E-05	499.45	401.01	252.34	214.71	RPS9	ribosomal protein S9	chr19	protein_coding
ENSG00000103257.7	1.02154224	10.9868734	0.00036545	0.00131324	238.99	233.82	681.08	595.46	SLC7A5	solute carrier family 7 (amino acid transporter light chain, L system), member 5	chr16	protein_coding
ENSG00000128656.12	1.2627969	2.47424822	5.27E-05	0.00022404	0.78	0.81	2.12	2.98	CHN1	chimerin 1	chr2	protein_coding
ENSG00000269058.4	-2.1056907	-2.5787353	0.01923175	0.0458365	0.08	0.15	0	0.06	CALR3	calreticulin 3	chr19	protein_coding
ENSG00000182809.9	-3.0865112	5.60545251	3.51E-23	1.06E-21	33.14	25.36	5.21	3.95	CRIP2	cysteine-rich protein 2	chr14	protein_coding
ENSG00000101230.5	-3.4071723	-0.5812811	1.03E-10	1.03E-09	0.28	0.46	0	0.08	ISM1	isthmin 1, angiogenesis inhibitor	chr20	protein_coding
ENSG00000183826.15	-1.0150018	2.97081812	0.00077568	0.0026171	0.89	0.98	0.6	0.64	BTBD9	BTB (POZ) domain containing 9	chr6	protein_coding
ENSG00000141540.9	-3.661487	0.21192174	4.26E-15	6.86E-14	0.5	0.24	0.04	0.04	TTYH2	tweety family member 2	chr17	protein_coding
ENSG00000120328.6	-5.0187832	-3.1315465	0.01889873	0.04519557	0.05	0	0	0	PCDHB12	protocadherin beta 12	chr5	protein_coding
ENSG00000189050.13	2.03473576	0.29681724	7.60E-07	4.46E-06	0.09	0.22	0.52	1.17	RNFT1	ring finger protein, transmembrane 1	chr17	protein_coding
ENSG00000276517.1	1.94370624	-1.8314326	0.00675696	0.01829845	0.04	0.01	0.23	0.02	AL133243.2	--	chr2	sense_intronic
ENSG00000126458.3	-1.8817868	6.34819204	1.70E-10	1.64E-09	105.11	93.52	43.69	27.75	RRAS	related RAS viral (r-ras) oncogene homolog	chr19	protein_coding
ENSG00000244459.2	-2.3018293	0.17424422	1.90E-08	1.39E-07	3.05	2.06	0.72	0.66	RP11-1398P	--	chr4	lincRNA
ENSG00000206630.1	-2.0330838	-2.1265976	0.0061421	0.01678695	3.22	2.39	0.39	1.31	SNORD60	small nucleolar RNA, C/D box 60	chr16	snoRNA
ENSG00000077713.17	1.01623898	1.52699544	0.00212515	0.00648803	0.58	0.5	1.32	1.59	SLC25A43	solute carrier family 25, member 43	chrX	protein_coding
ENSG00000117543.18	-1.6304374	5.44059043	2.77E-08	2.00E-07	36.06	43.44	15.9	18.34	DPH5	diphthamide biosynthesis 5	chr1	protein_coding
ENSG00000122691.11	-1.9735735	3.05934612	2.11E-10	2.02E-09	6.21	6.69	2.33	2.04	TWIST1	twist family bHLH transcription factor 1	chr7	protein_coding
ENSG00000169018.5	1.37012717	1.94559518	2.22E-05	0.00010109	0.21	0.27	0.46	1.19	FEM1B	fem-1 homolog b (C. elegans)	chr15	protein_coding
ENSG00000243926.1	-2.0460028	0.04943362	8.80E-07	5.11E-06	1.11	0.97	0.49	0.2	TIPARP-AS1	TIPARP antisense RNA 1	chr3	antisense
ENSG00000173366.11	-1.8261451	5.37271142	6.38E-10	5.72E-09	15.95	12.33	5.7	4.92	RP11-330H6	--	chr3	protein_coding
ENSG00000169126.14	-8.9370667	-0.0901928	1.09E-21	3.00E-20	0.49	0.34	0	0	ARMC4	armadillo repeat containing 4	chr10	protein_coding
ENSG00000181652.17	6.22302796	0.61756577	1.10E-24	3.71E-23	0	0.02	1.06	0.69	ATG9B	autophagy related 9B	chr7	protein_coding
ENSG00000275527.1	3.23271569	-2.5345399	0.003237	0.00945156	0	0.06	0.53	0.34	CTD-3154N5	--	chr15	lincRNA
ENSG00000274292.1	3.22527223	-1.4851934	1.38E-05	6.54E-05	0	0.03	0.26	0.17	RP11-347I19	--	chr12	lincRNA
ENSG00000271869.1	-1.0899692	0.50157199	0.00309574	0.00907892	3.25	4.29	1.99	2.7	RP11-51J9.5	--	chr8	lincRNA
ENSG00000031691.6	3.28573613	-0.6108395	4.32E-09	3.49E-08	0.05	0.05	0.43	0.85	CENPQ	centromere protein Q	chr6	protein_coding
ENSG00000136492.7	2.940902	0.59851358	2.35E-12	2.85E-11	0.06	0.01	0.23	0.52	BRIP1	BRCA1 interacting protein C-terminal helicase 1	chr17	protein_coding
ENSG00000183814.14	3.57910175	0.53441384	2.02E-15	3.35E-14	0.05	0.03	0.44	0.92	LIN9	lin-9 DREAM MuvB core complex component	chr1	protein_coding
ENSG00000006453.12	-1.0336989	5.45506614	0.00035241	0.00126941	12.79	13.55	8.02	9.14	BAIAP2L1	BAI1-associated protein 2-like 1	chr7	protein_coding
ENSG00000171408.12	1.96813602	-0.5764376	4.25E-05	0.0001846	0.02	0.05	0.23	0.13	PDE7B	phosphodiesterase 7B	chr6	protein_coding
ENSG00000130713.14	-1.3739571	7.30325898	2.09E-06	1.15E-05	91.99	85.98	43.37	48.26	EXOSC2	exosome component 2	chr9	protein_coding
ENSG00000081181.6	2.02804305	0.00078785	2.41E-06	1.31E-05	0.16	0.12	0.53	1.01	ARG2	arginase 2	chr14	protein_coding
ENSG00000278249.1	1.32007121	-1.2159008	0.01656825	0.04052383	0.32	0.47	1.98	0.71	SCARNA2	small Cajal body-specific RNA 2	chr1	scaRNA
ENSG00000277117.3	2.90471122	1.10077859	1.10E-13	1.53E-12	0.07	0.16	1.05	1.27	ICOSLG	inducible T-cell co-stimulator ligand	chr21	protein_coding
ENSG00000197748.11	2.50626927	-1.691379	0.00065065	0.00222818	0.02	0	0.04	0.08	CFAP43	cilia and flagella associated protein 43	chr10	protein_coding
ENSG00000127989.12	1.18489455	0.61812484	0.00138891	0.00444645	0.13	0.22	0.45	0.62	MTERF1	mitochondrial transcription termination factor 1	chr7	protein_coding
ENSG00000130948.8	-5.7776308	-2.7095582	0.00047761	0.00167191	0.24	0.05	0	0	HSD17B3	hydroxysteroid (17-beta) dehydrogenase 3	chr9	protein_coding
ENSG00000203805.9	-5.0243296	-3.1290017	0.0100455	0.02582742	0.02	0.11	0	0	PPAPDC1A	phosphatidic acid phosphatase type 2 domain containing 1A	chr10	protein_coding
ENSG00000198959.10	-2.0902196	8.79446865	1.51E-12	1.86E-11	124.09	105.4	36.83	34.98	TGM2	transglutaminase 2	chr20	protein_coding
ENSG00000129646.12	1.63814692	2.1927356	3.77E-07	2.32E-06	0.34	0.32	1.36	1.37	QRICH2	glutamine rich 2	chr17	protein_coding
ENSG00000102580.13	1.41759224	1.88650734	1.40E-05	6.61E-05	0.21	0.36	0.89	1.13	DNAJC3	DnaJ (Hsp40) homolog, subfamily C, member 3	chr13	protein_coding
ENSG00000147394.17	-5.5962546	2.470802	3.96E-40	3.03E-38	1.84	1.85	0.08	0.02	ZNF185	zinc finger protein 185 (LIM domain)	chrX	protein_coding
ENSG00000186976.13	5.56116133	-2.6812008	0.00086564	0.00288128	0	0	0.01	0.06	EFCAB6	EF-hand calcium binding domain 6	chr22	protein_coding
ENSG00000212694.7	1.44597661	1.89569554	9.51E-06	4.64E-05	1.3	0.76	4.02	3.51	LINC01089	long intergenic non-protein coding RNA 1089	chr12	lincRNA
ENSG00000166181.11	1.1897415	3.67258631	6.71E-05	0.00028004	1.57	1.51	3.24	6.18	API5	apoptosis inhibitor 5	chr11	protein_coding
ENSG00000155918.6	-3.2462128	-2.2727838	0.00013996	0.00054609	0.14	0.21	0	0.04	RAET1L	retinoic acid early transcript 1L	chr6	protein_coding
ENSG00000144649.7	-5.2091082	-3.0354822	0.00538165	0.01488851	0.03	0.04	0	0	FAM198A	family with sequence similarity 198, member A	chr3	protein_coding
ENSG00000135324.5	-8.9790686	-0.0503622	3.77E-22	1.07E-20	0.73	0.7	0	0	MRAP2	melanocortin 2 receptor accessory protein 2	chr6	protein_coding
ENSG00000129204.15	3.07340232	-1.0063327	6.13E-07	3.66E-06	0.01	0	0.04	0.12	USP6	ubiquitin specific peptidase 6	chr17	protein_coding
ENSG00000131746.11	-1.5255789	8.01668401	1.51E-07	9.85E-07	69.9	83.28	41.02	29.49	TNS4	tensin 4	chr17	protein_coding
ENSG00000265282.1	-3.1713871	-2.8685684	0.01615662	0.03958166	0.44	0	0	0.05	RP11-269G2	--	chr17	lincRNA
ENSG00000243051.3	5.23774779	-2.8750297	0.00290522	0.00856312	0	0	1.05	0	RN7SL269P	RNA, 7SL, cytoplasmic 269, pseudogene	chr1	misc_RNA
ENSG00000121753.11	-2.5819589	4.93681066	3.33E-17	6.40E-16	7.9	6.72	1.71	1.54	BAI2	--	chr1	protein_coding
ENSG00000100276.9	5.19748351	-2.8969879	0.00538165	0.01488851	0	0	0.04	0.12	RASL10A	RAS-like, family 10, member A	chr22	protein_coding
ENSG00000179299.15	5.06203518	-2.9716376	0.0100455	0.02582742	0	0	0.03	0.04	NSUN7	NOP2/Sun domain family, member 7	chr4	protein_coding
ENSG00000129009.11	-7.8274829	1.82417348	5.51E-43	4.98E-41	2.65	1.88	0.03	0	ISLR	immunoglobulin superfamily containing leucine-rich repeat	chr15	protein_coding
ENSG00000187186.13	-2.3393303	-2.1756582	0.00228047	0.00692082	0.16	0.12	0	0.06	RP11-195F1	--	chr9	protein_coding
ENSG00000142661.17	-3.4523356	-0.3887692	1.28E-11	1.42E-10	0.19	0.16	0	0.04	MYOM3	myomesin 3	chr1	protein_coding
ENSG00000188404.7	6.74809254	-1.8435441	2.24E-07	1.42E-06	0	0	0.11	0.25	SELL	selectin L	chr1	protein_coding
ENSG00000165072.9	-5.0123405	-0.8779355	5.19E-13	6.70E-12	0.27	0.18	0.02	0	MAMDC2	MAM domain containing 2	chr9	protein_coding
ENSG00000125107.15	1.85805571	6.77154141	2.65E-10	2.50E-09	4.12	4.62	21.23	20.93	CNOT1	CCR4-NOT transcription complex, subunit 1	chr16	protein_coding
ENSG00000251455.1	3.39117329	-2.4344227	0.00190233	0.00584723	0.01	0	0.02	0.15	RP11-164P1	--	chr4	antisense
ENSG00000259863.1	-6.8816936	-1.9256557	1.35E-07	8.87E-07	0.23	0.21	0	0	SH3RF3-AS1	SH3RF3 antisense RNA 1	chr2	lincRNA
ENSG00000249992.1	-3.0942324	5.30904129	3.66E-23	1.11E-21	36.19	27.12	5.11	4.78	TMEM158	transmembrane protein 158 (gene/pseudogene)	chr3	protein_coding
ENSG00000164953.14	1.22386838	0.60789125	0.00090066	0.00298916	0.2	0.09	0.47	0.44	TMEM67	transmembrane protein 67	chr8	protein_coding
ENSG00000138433.14	1.8297213	2.90746437	4.73E-09	3.79E-08	1.41	1.33	4.14	8.93	CIR1	corepressor interacting with RBPJ, 1	chr2	protein_coding
ENSG00000085274.14	2.5346315	0.33869467	2.00E-09	1.68E-08	0.04	0.06	0.22	0.55	MYNN	myoneurin	chr3	protein_coding
ENSG00000112031.14	1.32735915	2.34948303	2.53E-05	0.00011408	0.47	0.79	1.52	2.7	MTRF1L	mitochondrial translational release factor 1-like	chr6	protein_coding
ENSG00000071889.15	-2.2550882	6.96954786	3.74E-14	5.44E-13	89.86	83.58	25.34	23.02	FAM3A	family with sequence similarity 3, member A	chrX	protein_coding
ENSG00000139445.16	3.25170792	-2.5240472	0.003237	0.00945156	0	0.01	0.11	0.02	FOXN4	forkhead box N4	chr12	protein_coding
ENSG00000198502.5	10.4118939	1.42875907	3.31E-37	2.14E-35	0	0	4.78	4.02	HLA-DRB5	major histocompatibility complex, class II, DR beta 5	chr6	protein_coding
ENSG00000268621.4	2.03613214	6.23773043	6.42E-12	7.37E-11	22.23	23.91	125.23	126.89	AC006262.5	--	chr19	lincRNA
ENSG00000165660.7	1.41101575	1.63310585	2.02E-05	9.28E-05	0.58	0.3	0.93	2.24	FAM175B	family with sequence similarity 175, member B	chr10	protein_coding
ENSG00000187037.7	-5.7759286	-2.7103808	0.00047761	0.00167191	0.25	0	0	0	GPR141	G protein-coupled receptor 141	chr7	protein_coding
ENSG00000171889.3	-3.6516206	2.22461332	5.25E-24	1.68E-22	8.76	10.08	1.12	0.88	MIR31HG	MIR31 host gene	chr9	sense_overlapping
ENSG00000245532.5	1.35985244	8.96706931	2.54E-06	1.37E-05	8.84	11.16	31.23	37.24	NEAT1	nuclear paraspeckle assembly transcript 1 (non-protein coding)	chr11	lincRNA
ENSG00000197579.6	2.29964995	1.80555972	2.19E-11	2.35E-10	0.2	0.25	0.88	2.11	TOPORS	topoisomerase I binding, arginine/serine-rich, E3 ubiquitin protein ligase	chr9	protein_coding
ENSG00000276007.1	4.118141	-1.2262944	9.32E-08	6.27E-07	0	0.05	0.35	0.92	RP11-358L2	--	chr16	sense_intronic
ENSG00000004846.15	3.7796366	-0.7023782	7.71E-10	6.81E-09	0.01	0.01	0.11	0.16	ABCB5	ATP-binding cassette, sub-family B (MDR/TAP), member 5	chr7	protein_coding
ENSG00000258297.1	2.23592811	0.64413174	1.47E-08	1.10E-07	0.1	0.25	1.1	1.14	RP11-658F2.	--	chr11	antisense
ENSG00000196372.11	-1.3344013	5.23223563	4.73E-06	2.44E-05	17.61	14.64	7.63	9.46	ASB13	ankyrin repeat and SOCS box containing 13	chr10	protein_coding
ENSG00000144320.12	2.7079928	1.384514	2.64E-13	3.51E-12	0.08	0.06	0.58	0.64	KIAA1715	KIAA1715	chr2	protein_coding
ENSG00000116717.10	-2.5638812	5.66457566	2.38E-17	4.65E-16	41.39	52.66	7.64	13.65	GADD45A	growth arrest and DNA-damage-inducible, alpha	chr1	protein_coding
ENSG00000175701.9	2.09288033	2.95354376	3.13E-11	3.32E-10	1.34	0.94	5.98	7.06	LINC00116	long intergenic non-protein coding RNA 116	chr2	protein_coding
ENSG00000161513.10	1.52366737	4.82174706	2.28E-07	1.45E-06	5.41	4.57	19.18	19.08	FDXR	ferredoxin reductase	chr17	protein_coding
ENSG00000166170.9	1.37204951	3.21514779	6.14E-06	3.10E-05	0.89	0.69	2.65	2.8	BAG5	BCL2-associated athanogene 5	chr14	protein_coding
ENSG00000204624.7	11.0014935	2.00037687	6.04E-45	6.19E-43	0	0	1.46	1.65	PTCHD2	patched domain containing 2	chr1	protein_coding
ENSG00000103034.13	1.60868082	0.16009979	7.20E-05	0.00029802	0.12	0.06	0.39	0.36	NDRG4	NDRG family member 4	chr16	protein_coding
ENSG00000104691.13	-1.0187356	1.06973379	0.00271959	0.00814315	1.08	1.54	0.71	1.01	UBXN8	UBX domain protein 8	chr8	protein_coding
ENSG00000146433.8	2.46453666	2.66622918	4.98E-14	7.17E-13	0.23	0.35	1.57	2.68	TMEM181	transmembrane protein 181	chr6	protein_coding
ENSG00000270084.1	4.88946445	-3.0622981	0.01889873	0.04519557	0	0	0.09	0.24	GAS5-AS1	GAS5 antisense RNA 1	chr1	antisense
ENSG00000135502.15	3.49494539	-1.2768776	8.97E-07	5.20E-06	0.02	0.01	0.28	0.27	SLC26A10	solute carrier family 26, member 10	chr12	protein_coding
ENSG00000156642.15	1.40447932	5.97645144	1.40E-06	7.87E-06	8.06	9.59	25.14	37.38	NPTN	neuroplastin	chr15	protein_coding
ENSG00000169169.13	-6.5920325	-0.2110531	1.70E-20	4.24E-19	0.59	0.34	0.01	0	CPT1C	carnitine palmitoyltransferase 1C	chr19	protein_coding
ENSG00000279696.1	3.67704827	-1.5834542	8.20E-06	4.05E-05	0.02	0	0.18	0.15	RP11-178H8	--	chr11	TEC
ENSG00000246889.2	1.90576451	-0.9305747	0.0002245	0.00084143	0.1	0	0.25	0.25	AP000487.5	--	chr11	antisense
ENSG00000274515.1	4.79509099	-0.6604369	7.46E-12	8.46E-11	0	0.13	1.97	3.35	CTD-2026K1	--	chr15	antisense
ENSG00000122176.10	-9.5946053	0.55058852	1.25E-28	5.35E-27	0.81	0.62	0	0	FMOD	fibromodulin	chr1	protein_coding
ENSG00000135540.10	-1.0569074	3.45285226	0.0003911	0.0013968	1.36	1.67	1.09	0.85	NHSL1	NHS-like 1	chr6	protein_coding
ENSG00000279766.1	1.77918435	-1.1277406	0.0021156	0.00646136	0.08	0.34	0.76	1.14	RP11-642A1	--	chr8	TEC
ENSG00000163666.7	6.22409033	-2.2339746	2.70E-05	0.00012094	0	0	0.03	0.56	HESX1	HESX homeobox 1	chr3	protein_coding
ENSG00000189269.11	-2.6778041	-1.3046811	1.00E-05	4.87E-05	0.16	0.63	0.1	0.06	DRICH1	aspartate-rich 1	chr22	protein_coding
ENSG00000135912.9	-1.3103116	4.36282242	8.59E-06	4.23E-05	4.07	5.42	2.42	2.68	TTLL4	tubulin tyrosine ligase-like family member 4	chr2	protein_coding
ENSG00000245248.6	-3.4057173	-0.7515214	4.03E-10	3.72E-09	0.22	0.12	0	0.04	USP2-AS1	USP2 antisense RNA 1 (head to head)	chr11	antisense
ENSG00000110628.12	-1.1810755	2.77752267	0.00011061	0.00044015	3.67	3.96	2.48	1.99	SLC22A18	solute carrier family 22, member 18	chr11	protein_coding
ENSG00000275297.1	-5.3760424	-2.9459444	0.00290522	0.00856312	0.09	0.19	0	0	RP11-292F9.	--	chr9	processed_transcript
ENSG00000100842.11	5.64744643	2.65704309	1.11E-40	8.89E-39	0.06	0.06	3.95	4.13	EFS	embryonal Fyn-associated substrate	chr14	protein_coding
ENSG00000188611.13	3.21856418	-2.5423804	0.003237	0.00945156	0	0	0.02	0.04	ASAH2	N-acylsphingosine amidohydrolase (non-lysosomal ceramidase) 2	chr10	protein_coding
ENSG00000092140.13	4.03675919	0.02654154	1.43E-14	2.19E-13	0.02	0	0.14	0.36	G2E3	G2/M-phase specific E3 ubiquitin protein ligase	chr14	protein_coding
ENSG00000255236.2	1.52163857	-1.7224669	0.01792389	0.04353308	0.1	0.15	0.83	0.18	CTD-2655K5	--	chr11	antisense
ENSG00000079134.10	2.04346765	1.91363655	1.10E-09	9.56E-09	0.54	0.54	2.36	3.6	THOC1	THO complex 1	chr18	protein_coding
ENSG00000096093.13	-1.7005008	0.37619634	8.88E-06	4.36E-05	0.28	0.29	0.09	0.14	EFHC1	EF-hand domain (C-terminal) containing 1	chr6	protein_coding
ENSG00000260000.2	1.49876546	-0.7359338	0.00215901	0.00657968	0.11	0.19	0.55	0.56	RP3-467N11	--	chr6	antisense
ENSG00000103275.17	-1.0524801	7.29543883	0.00025152	0.00093553	28.06	28.68	18.56	17.87	UBE2I	ubiquitin-conjugating enzyme E2I	chr16	protein_coding
ENSG00000151729.9	1.25909329	4.89682616	1.64E-05	7.66E-05	3.23	3.15	9	11.42	SLC25A4	solute carrier family 25 (mitochondrial carrier; adenine nucleotide translocator), member 4	chr4	protein_coding
ENSG00000107104.17	-1.3125623	3.8225682	1.03E-05	5.00E-05	2.26	2.68	1.43	1.21	KANK1	KN motif and ankyrin repeat domains 1	chr9	protein_coding
ENSG00000144746.6	1.09816492	3.59840394	0.00023077	0.0008633	2.46	3.45	6.37	10.55	ARL6IP5	ADP-ribosylation factor-like 6 interacting protein 5	chr3	protein_coding
ENSG00000265055.1	2.58421209	-2.3458297	0.00455824	0.01291303	0.08	0	0.69	0.08	AC145343.2	--	chr17	lincRNA
ENSG00000105518.12	-1.5456827	4.73001204	1.64E-07	1.06E-06	26.39	20.3	11.54	9.75	TMEM205	transmembrane protein 205	chr19	protein_coding
ENSG00000155380.10	1.2753763	5.72552562	1.14E-05	5.44E-05	4.58	6.15	13.92	20.8	SLC16A1	solute carrier family 16 (monocarboxylate transporter), member 1	chr1	protein_coding
ENSG00000105088.7	1.34424591	-0.2638753	0.00230895	0.00699749	0.12	0.22	0.75	0.38	OLFM2	olfactomedin 2	chr19	protein_coding
ENSG00000087253.10	1.30388689	2.1861543	3.86E-05	0.00016877	0.32	0.36	0.85	1.38	LPCAT2	lysophosphatidylcholine acyltransferase 2	chr16	protein_coding
ENSG00000279977.1	2.23042559	-1.8803172	0.00303791	0.00892022	0	0.06	0.32	0.09	CTD-3222D1	--	chr19	TEC
ENSG00000266265.2	5.75916799	-0.5953071	1.30E-13	1.78E-12	0	0.01	0.45	0.4	KLF14	Kruppel-like factor 14	chr7	protein_coding
ENSG00000152332.14	2.25054775	2.60603097	3.39E-12	4.02E-11	0.21	0.18	0.79	1.71	UHMK1	U2AF homology motif (UHM) kinase 1	chr1	protein_coding
ENSG00000186026.6	-3.7973138	-1.0899029	1.50E-08	1.12E-07	0.22	0.08	0.03	0	ZNF284	zinc finger protein 284	chr19	protein_coding
ENSG00000139318.7	-1.1917262	1.82136152	0.00021872	0.00082104	0.76	0.98	0.51	0.5	DUSP6	dual specificity phosphatase 6	chr12	protein_coding
ENSG00000206075.12	-3.0014573	3.39527776	1.48E-20	3.71E-19	4.16	4.79	0.6	0.89	SERPINB5	serpin peptidase inhibitor, clade B (ovalbumin), member 5	chr18	protein_coding
ENSG00000212907.2	1.56960905	8.45658244	6.71E-08	4.62E-07	318.96	649.42	2159.88	1642.43	MT-ND4L	mitochondrially encoded NADH dehydrogenase 4L	chrM	protein_coding
ENSG00000260269.4	-1.4989338	3.59931414	5.97E-07	3.57E-06	18.68	18	8.53	8.77	CTD-2323K1	--	chr15	processed_transcript
ENSG00000265681.5	-1.1668646	2.62886378	0.0001395	0.00054552	4.91	6.62	2.75	4.09	RPL17	ribosomal protein L17	chr18	protein_coding
ENSG00000244198.4	7.2865931	-1.4146993	7.05E-10	6.26E-09	0	0	0.28	0.28	RP4-545C24	--	chr7	antisense

ENSG00000105865.9	1.4674989	0.15364897	0.00032833	0.00118768	0.14	0.26	0.65	0.82	DUS4L	dihydrouridine synthase 4-like (S. cerevisiae)	chr7	protein_coding
ENSG00000184402.13	1.44425397	2.71241715	3.13E-06	1.67E-05	0.79	0.45	2.09	2.44	SS18L1	synovial sarcoma translocation gene on chromosome 18-like 1	chr20	protein_coding
ENSG00000116406.17	2.403959	0.23584344	1.57E-08	1.17E-07	0.01	0.07	0.22	0.35	EDEM3	ER degradation enhancer, mannosidase alpha-like 3	chr1	protein_coding
ENSG00000115661.12	-1.9090453	5.35995746	1.16E-10	1.15E-09	16.33	15.67	6.02	5.31	STK16	serine/threonine kinase 16	chr2	protein_coding
ENSG00000204876.4	4.19785579	-1.881781	4.25E-06	2.21E-05	0	0.01	0.28	0.05	AC021218.2	--	chr7	lincRNA
ENSG00000166454.8	2.00053076	3.54607282	8.27E-11	8.34E-10	0.61	0.64	2.68	4	ATMIN	ATM interactor	chr16	protein_coding
ENSG00000181908.5	2.44195762	-1.7364555	0.00095666	0.0031536	0	0.05	0.25	0.12	AP003774.4	--	chr11	lincRNA
ENSG00000116711.9	-3.3226757	0.11736278	6.00E-13	7.70E-12	0.53	0.56	0.07	0.08	PLA2G4A	phospholipase A2, group IVA (cytosolic, calcium-dependent)	chr1	protein_coding
ENSG00000261120.1	6.19050648	-2.2628719	2.70E-05	0.00012094	0	0	0.57	0.59	RP11-62H20	--	chr14	antisense
ENSG00000267280.4	-1.8313795	2.4904603	7.24E-09	5.66E-08	6.21	5.52	2.34	2.05	TBX2-AS1	TBX2 antisense RNA 1	chr17	antisense
ENSG00000126787.11	2.02094718	2.22534211	7.15E-10	6.35E-09	0.45	0.5	1.38	3.78	DLGAP5	discs, large (Drosophila) homolog-associated protein 5	chr14	protein_coding
ENSG00000179846.8	5.45080994	0.77686591	1.97E-24	6.54E-23	0.02	0	0.63	0.79	NKPD1	NTPase, KAP family P-loop domain containing 1	chr19	protein_coding
ENSG00000147592.7	1.79304045	1.6907756	1.08E-07	7.21E-07	0.55	0.87	1.71	4.91	LACTB2	lactamase, beta 2	chr8	protein_coding
ENSG00000083312.16	1.63093344	4.31362762	4.46E-08	3.12E-07	0.69	0.7	2.16	3.61	TNPO1	transportin 1	chr5	protein_coding
ENSG00000278677.1	1.42717934	1.483649	2.19E-05	9.98E-05	2.18	3.89	12.72	8.94	HIST1H2AM	histone cluster 1, H2am	chr6	protein_coding
ENSG00000164331.8	1.27400167	1.38917183	0.00016144	0.00062059	0.56	0.6	1.54	2.22	ANKRA2	ankyrin repeat, family A (RFXANK-like), 2	chr5	protein_coding
ENSG00000119655.7	1.32008222	7.27834949	5.03E-06	2.59E-05	29.45	26.67	87.59	99.51	NPC2	Niemann-Pick disease, type C2	chr14	protein_coding
ENSG00000214063.9	-1.1369894	6.57863663	8.12E-05	0.00033385	60.17	50.87	35.87	31.34	TSPAN4	tetraspanin 4	chr11	protein_coding
ENSG00000074047.19	-4.4159877	4.14482792	1.08E-37	7.21E-36	3.86	4.12	0.25	0.25	GLI2	GLI family zinc finger 2	chr2	protein_coding
ENSG00000279495.1	1.63937282	0.27561626	5.18E-05	0.00022046	0.16	0.19	0.58	0.89	RP11-521B2	--	chr14	TEC
ENSG00000143951.14	1.9595662	0.38507241	8.19E-07	4.78E-06	0.02	0.07	0.22	0.27	WDPCP	WD repeat containing planar cell polarity effector	chr2	protein_coding
ENSG00000082497.10	3.20139439	-1.49573	2.06E-05	9.41E-05	0.01	0.01	0.02	0.18	SERTAD4	SERTA domain containing 4	chr1	protein_coding
ENSG00000101336.11	3.49926079	-1.7171703	4.49E-05	0.00019431	0.02	0	0.22	0.2	HCK	HCK proto-oncogene, Src family tyrosine kinase	chr20	protein_coding
ENSG00000139291.12	1.47669184	1.48090352	1.17E-05	5.59E-05	0.21	0.15	0.48	0.87	TMEM19	transmembrane protein 19	chr12	protein_coding
ENSG00000205517.11	-6.7624536	2.22936794	8.86E-45	8.92E-43	2.91	2.98	0.05	0.02	RGL3	ral guanine nucleotide dissociation stimulator-like 3	chr19	protein_coding
ENSG00000251095.5	2.401895	-1.3025998	0.00010901	0.00043503	0.02	0.01	0.08	0.11	RP11-115D1	--	chr4	antisense
ENSG00000104427.10	2.03723318	-0.6166327	3.75E-05	0.00016418	0.04	0.07	0.26	0.33	ZC2HC1A	zinc finger, C2HC-type containing 1A	chr8	protein_coding
ENSG00000107362.12	2.42293196	0.02764436	6.50E-08	4.48E-07	0.09	0.07	0.42	0.72	ABHD17B	abhydrolase domain containing 17B	chr9	protein_coding
ENSG00000124562.8	-1.1151766	6.76954506	0.00010917	0.00043559	104.33	96.99	64.95	58.75	SNRPC	small nuclear ribonucleoprotein polypeptide C	chr6	protein_coding
ENSG00000164089.7	3.07805235	-2.018793	0.00094481	0.00312013	0	0.03	0.2	0.13	ETNPPL	ethanolamine-phosphate phospho-lyase	chr4	protein_coding
ENSG00000178904.17	1.71461973	0.59997958	5.94E-06	3.01E-05	0.06	0.08	0.23	0.34	DPY19L3	dpy-19-like 3 (C. elegans)	chr19	protein_coding
ENSG00000197415.10	1.68912621	-1.3236966	0.0036829	0.0106382	0.05	0	0.08	0.12	VEPH1	ventricular zone expressed PH domain-containing 1	chr3	protein_coding
ENSG00000166664.12	-3.1682129	-2.8645511	0.01615662	0.03958166	0.02	0.01	0	0	CHRFAM7A	CHRNA7 (cholinergic receptor, nicotinic, alpha 7, exons 5-10) and FAM7A (family with sequence similarity 7A, exons A-E) fusion	chr15	protein_coding
ENSG00000111432.4	-4.7717303	3.67400489	4.67E-40	3.55E-38	6.14	6.02	0.29	0.3	FZD10	frizzled class receptor 10	chr12	protein_coding
ENSG00000167202.10	1.29085697	5.78511752	8.86E-06	4.35E-05	4.16	4.27	14.38	13.05	TBC1D2B	TBC1 domain family, member 2B	chr15	protein_coding
ENSG00000103241.6	-1.0455727	3.25979819	0.00047053	0.00165537	3.45	2.47	1.77	2.06	FOXF1	forkhead box F1	chr16	protein_coding
ENSG00000172493.19	2.24793601	4.05281879	2.39E-13	3.18E-12	0.46	0.49	2.7	3.36	AFF1	AF4/FMR2 family, member 1	chr4	protein_coding
ENSG00000100979.13	-3.1829653	3.12686529	4.31E-22	1.22E-20	5.4	4.63	0.58	0.89	PLTP	phospholipid transfer protein	chr20	protein_coding
ENSG00000174799.9	2.19117588	0.06959028	4.22E-07	2.59E-06	0.02	0.06	0.11	0.39	CEP135	centrosomal protein 135kDa	chr4	protein_coding
ENSG00000166866.11	-2.3267993	-2.4471895	0.02049243	0.04852277	0.07	0.02	0.02	0.01	MYO1A	myosin IA	chr12	protein_coding
ENSG00000130203.8	2.24324088	3.44056916	6.57E-13	8.40E-12	2.21	2.82	16.86	14.79	APOE	apolipoprotein E	chr19	protein_coding
ENSG00000108771.11	1.829589	0.54694673	2.10E-06	1.15E-05	0.02	0.36	0.73	1.03	DHX58	DEXH (Asp-Glu-X-His) box polypeptide 58	chr17	protein_coding
ENSG00000165525.16	2.39554279	2.66895082	1.81E-13	2.45E-12	0.23	0.27	1.1	2.46	NEMF	nuclear export mediator factor	chr14	protein_coding
ENSG00000228395.1	-3.9902342	-2.3832208	0.00039332	0.00140265	0.17	0.18	0	0.02	RP11-216B9.	--	chr9	antisense
ENSG00000175324.8	-1.148531	4.7049989	8.26E-05	0.00033936	25.37	25.33	14.63	15.86	LSM1	LSM1, U6 small nuclear RNA associated	chr8	protein_coding
ENSG00000184058.11	-1.4924581	1.71596612	5.20E-06	2.66E-05	1.47	1.3	0.73	0.58	TBX1	T-box 1	chr22	protein_coding
ENSG00000221867.7	-10.534518	1.48285984	3.90E-40	3.00E-38	2.75	2.31	0	0	MAGEA3	melanoma antigen family A3	chrX	protein_coding
ENSG00000133475.15	-8.0292978	-0.9428312	5.27E-14	7.56E-13	0.42	0.25	0	0	GGT2	gamma-glutamyltransferase 2	chr22	protein_coding
ENSG00000099139.12	7.15844477	1.50461986	1.25E-35	7.63E-34	0	0	0.43	0.49	PCSK5	proprotein convertase subtilisin/kexin type 5	chr9	protein_coding
ENSG00000160752.13	-1.073003	8.78387787	0.00018633	0.00070721	307.01	280.14	185.37	186.65	FDPS	farnesyl diphosphate synthase	chr1	protein_coding
ENSG00000242125.3	-1.1760525	4.72243982	5.67E-05	0.00023954	11.56	11.08	7.72	5.58	SNHG3	small nucleolar RNA host gene 3	chr1	sense_intronic
ENSG00000106211.8	-1.8663076	8.31871361	2.00E-10	1.91E-09	398.41	366.78	147.89	131.38	HSPB1	heat shock 27kDa protein 1	chr7	protein_coding
ENSG00000198162.11	2.39905902	3.57766831	1.96E-14	2.94E-13	0.3	0.42	2.16	2.85	MAN1A2	mannosidase, alpha, class 1A, member 2	chr1	protein_coding
ENSG00000272631.1	2.68566927	0.5261302	1.54E-10	1.50E-09	0.03	0.06	0.31	0.46	RP11-359E3.	--	chr10	antisense
ENSG00000271789.1	5.19748351	-2.8969879	0.00538165	0.01488851	0	0	0.05	0.16	RP5-1112D6	--	chr6	lincRNA
ENSG00000166263.12	4.46014382	-0.1256314	6.12E-15	9.74E-14	0.01	0	0.12	0.08	STXBP4	syntaxin binding protein 4	chr17	protein_coding
ENSG00000132563.14	2.81255874	3.38890257	2.72E-18	5.74E-17	1.03	0.82	9.6	7.76	REEP2	receptor accessory protein 2	chr5	protein_coding
ENSG00000010030.12	4.82068142	-0.6498189	3.84E-12	4.51E-11	0	0.02	0.69	0.29	ETV7	ets variant 7	chr6	protein_coding
ENSG00000182263.12	-2.62578	-2.6445964	0.00552196	0.01522223	0.02	0.01	0	0.01	FIGN	fidgetin	chr2	protein_coding
ENSG00000125434.9	1.21699307	2.88497439	7.01E-05	0.0002908	1.09	0.73	2.68	2.98	SLC25A35	solute carrier family 25, member 35	chr17	protein_coding
ENSG00000162913.9	-1.4440536	-1.5146779	0.01203274	0.03038863	0.3	0.51	0	0.37	C1orf145	chromosome 1 open reading frame 145	chr1	protein_coding
ENSG00000272702.1	1.23392154	-0.6328739	0.01010345	0.025949	0.83	0.47	2.67	1.49	RP11-44N22	--	chr2	processed_transcript
ENSG00000065054.12	-2.5649071	7.61002413	1.62E-17	3.22E-16	106.16	118.45	25.02	25.54	SLC9A3R2	solute carrier family 9, subfamily A (NHE3, cation proton antiporter 3), member 3 regulator 2	chr16	protein_coding
ENSG00000228253.1	1.40055234	10.0587526	1.28E-06	7.22E-06	1534.18	3064.91	9104.66	6958.95	MT-ATP8	mitochondrially encoded ATP synthase 8	chrM	protein_coding
ENSG00000182580.2	2.92319827	1.16390082	4.46E-14	6.45E-13	0.06	0.13	0.91	1.02	EPHB3	EPH receptor B3	chr3	protein_coding
ENSG00000128709.11	3.100442	2.8682343	3.01E-20	7.35E-19	0.6	0.7	7.43	7.41	HOXD9	homeobox D9	chr2	protein_coding
ENSG00000269821.1	5.50465016	2.30812583	6.11E-37	3.89E-35	0	0	0.09	0.13	KCNQ1OT1	KCNQ1 opposite strand/antisense transcript 1 (non-protein coding)	chr11	antisense
ENSG00000099194.5	1.04268956	9.49244001	0.00027832	0.00101954	67.01	75.22	183.79	206.98	SCD	stearoyl-CoA desaturase (delta-9-desaturase)	chr10	protein_coding
ENSG00000281357.1	1.58683703	-0.7591829	0.00135033	0.00433451	0.09	0.08	0.31	0.36	ARRDC3-AS	ARRDC3 antisense RNA 1	chr5	lincRNA
ENSG00000163312.9	2.08439766	0.10935679	1.24E-06	7.02E-06	0.04	0.13	0.4	0.56	HELQ	helicase, POLQ-like	chr4	protein_coding
ENSG00000163521.14	-1.0495083	2.72011268	0.00060735	0.00209399	2.29	2.92	1.9	1.45	GLB1L	galactosidase, beta 1-like	chr2	protein_coding
ENSG00000047365.10	5.6875973	-2.6030615	0.00047761	0.00167191	0	0	0.03	0.03	ARAP2	ArfGAP with RhoGAP domain, ankyrin repeat and PH domain 2	chr4	protein_coding
ENSG00000139567.11	-4.8079387	-3.2306674	0.01889873	0.04519557	0.01	0.03	0	0	ACVRL1	activin A receptor type II-like 1	chr12	protein_coding
ENSG00000103150.5	1.46815382	3.94727576	8.86E-07	5.14E-06	0.58	0.43	1.82	1.91	MLYCD	malonyl-CoA decarboxylase	chr16	protein_coding
ENSG00000128340.13	-4.6158336	-1.9292317	2.62E-06	1.42E-05	0.26	0.07	0.02	0	RAC2	ras-related C3 botulinum toxin substrate 2 (rho family, small GTP binding protein Rac2)	chr22	protein_coding
ENSG00000172602.8	3.34691098	-0.0470353	1.98E-11	2.15E-10	0.1	0.05	0.86	1.18	RND1	Rho family GTPase 1	chr12	protein_coding
ENSG00000197019.4	1.79228475	4.23387668	2.37E-09	1.97E-08	3.49	2.92	16.02	13.56	SERTAD1	SERTA domain containing 1	chr19	protein_coding
ENSG00000145990.9	-1.0426403	2.64820699	0.00065345	0.00223554	0.71	0.57	0.35	0.48	GFOD1	glucose-fructose oxidoreductase domain containing 1	chr6	protein_coding
ENSG00000232194.1	-5.8924048	-2.6371928	0.00026535	0.0009747	0.34	0.16	0	0	RP1-313L4.3	--	chr1	antisense
ENSG00000113441.14	1.35454022	2.21966689	1.98E-05	9.07E-05	0.17	0.15	0.41	0.68	LNPEP	leucyl/cystinyl aminopeptidase	chr5	protein_coding
ENSG00000169851.14	-1.5488965	2.39139211	8.11E-07	4.74E-06	0.87	0.9	0.38	0.43	PCDH7	protocadherin 7	chr4	protein_coding
ENSG00000130544.10	1.46931637	1.60734944	1.10E-05	5.30E-05	0.18	0.24	0.77	0.79	ZNF557	zinc finger protein 557	chr19	protein_coding
ENSG00000079616.11	-1.0182871	7.89178969	0.00038928	0.00139072	98.23	85.86	63.09	57.93	KIF22	kinesin family member 22	chr16	protein_coding
ENSG00000077458.11	3.40600371	1.48710077	1.98E-18	4.23E-17	0.07	0.12	0.99	1.7	FAM76B	family with sequence similarity 76, member B	chr11	protein_coding
ENSG00000005884.16	-1.0663833	10.1456169	0.00020209	0.00076244	272.87	234.69	173	149.5	ITGA3	integrin, alpha 3 (antigen CD49C, alpha 3 subunit of VLA-3 receptor)	chr17	protein_coding
ENSG00000205085.7	1.75746236	-0.9153769	0.00098911	0.00325251	0.02	0.04	0.05	0.21	FAM71F2	family with sequence similarity 71, member F2	chr7	protein_coding
ENSG00000132600.15	1.46054036	7.27206435	4.88E-07	2.96E-06	16.14	14.98	57.93	56.15	PRMT7	protein arginine methyltransferase 7	chr16	protein_coding
ENSG00000268812.3	5.57068129	-2.6758978	0.00086564	0.00288128	0	0	0.16	0.31	RP1-102K2.8	--	chr22	antisense
ENSG00000179148.8	-1.2492568	0.01049895	0.00175617	0.00544689	0.26	0.33	0.15	0.17	ALOXE3	arachidonate lipoxygenase 3	chr17	protein_coding
ENSG00000069869.14	2.34841094	0.58735469	4.05E-09	3.29E-08	0.06	0.04	0.22	0.44	NEDD4	neural precursor cell expressed, developmentally down-regulated 4, E3 ubiquitin protein ligase	chr15	protein_coding
ENSG00000074582.11	-1.649323	5.61651207	1.90E-08	1.39E-07	29.82	26.64	12.61	11.36	BCS1L	BC1 (ubiquinol-cytochrome c reductase) synthesis-like	chr2	protein_coding
ENSG00000227477.1	-1.6419347	-1.266944	0.00277048	0.00827794	0.25	0.19	0.09	0.09	STK4-AS1	STK4 antisense RNA 1 (head to head)	chr20	lincRNA
ENSG00000278784.1	1.12833793	1.18578149	0.00101657	0.00333525	1	0.85	3.1	2.31	RP11-468E2.	--	chr14	sense_intronic
ENSG00000206028.1	6.21657235	-2.2478749	1.55E-05	7.26E-05	0	0	0.18	0.05	CTA-373H7.	--	chr22	lincRNA
ENSG00000081052.11	-3.2270023	3.61693011	2.85E-23	8.69E-22	1.78	1.85	0.21	0.3	COL4A4	collagen, type IV, alpha 4	chr2	protein_coding
ENSG00000118412.11	3.82743129	1.05234925	4.13E-19	9.35E-18	0.05	0.01	0.42	0.87	CASP8AP2	caspase 8 associated protein 2	chr6	protein_coding
ENSG00000270179.1	1.9506875	-1.2841715	0.00075675	0.00255792	0.17	0.18	1.1	0.72	RP11-159N1	--	chr11	sense_intronic
ENSG00000135605.11	1.65218488	-0.4821973	0.00047649	0.00167191	0.09	0.05	0.29	0.28	TEC	tec protein tyrosine kinase	chr4	protein_coding
ENSG00000166575.15	2.56417382	0.4274565	1.22E-09	1.05E-08	0.05	0.1	0.42	0.73	TMEM135	transmembrane protein 135	chr11	protein_coding
ENSG00000188760.9	-3.2999557	2.7675833	1.04E-22	3.04E-21	4.31	3.49	0.27	0.77	TMEM198	transmembrane protein 198	chr2	protein_coding
ENSG00000136883.11	-5.0679112	-1.58021	1.76E-09	1.50E-08	0.21	0.25	0	0.01	KIF12	kinesin family member 12	chr9	protein_coding
ENSG00000110799.12	-5.897443	-2.6347369	0.00026535	0.0009747	0.01	0.03	0	0	VWF	von Willebrand factor	chr12	protein_coding
ENSG00000066422.4	4.160451	0.93659829	2.02E-20	5.01E-19	0.03	0.01	0.22	0.81	ZBTB11	zinc finger and BTB domain containing 11	chr3	protein_coding
ENSG00000280132.1	-1.3083881	-0.154346	0.00186729	0.00575801	0.6	0.43	0.29	0.27	RP11-452L6.	--	chr16	TEC
ENSG00000204054.10	-1.4704234	6.65430006	4.25E-07	2.60E-06	13.1	11.96	6.36	5.67	LINC00963	long intergenic non-protein coding RNA 963	chr9	processed_transcript
ENSG00000123570.3	4.89980841	-3.0567113	0.01889873	0.04519557	0	0	0.03	0.03	RAB9B	RAB9B, member RAS oncogene family	chrX	protein_coding
ENSG00000145780.7	1.68183401	1.00323334	2.61E-06	1.41E-05	0.06	0.19	0.32	0.76	FEM1C	fem-1 homolog c (C. elegans)	chr5	protein_coding
ENSG00000004961.13	-1.0149129	6.11188259	0.00042915	0.00152047	31.05	33.11	19.48	22.89	HCCS	holocytochrome c synthase	chrX	protein_coding
ENSG00000139278.8	-2.0628657	1.02116395	7.50E-09	5.85E-08	0.46	0.44	0.09	0.19	GLIPR1	GLI pathogenesis-related 1	chr12	protein_coding
ENSG00000239305.5	2.00011268	3.33458749	1.08E-10	1.07E-09	0.75	1.11	4.25	5.69	RNF103	ring finger protein 103	chr2	protein_coding
ENSG00000272031.2	-1.4848914	2.46665514	2.09E-06	1.15E-05	1.82	1.81	0.97	0.76	ANKRD34A	ankyrin repeat domain 34A	chr1	protein_coding
ENSG00000145912.7	-1.5615119	7.21805854	8.12E-08	5.52E-07	220.02	196.05	90.47	97.62	NHP2	NHP2 ribonucleoprotein	chr5	protein_coding
ENSG00000139132.13	1.23119269	-0.1893546	0.00424613	0.01210611	0.04	0.04	0.09	0.16	FGD4	FYVE, RhoGEF and PH domain containing 4	chr12	protein_coding
ENSG00000120158.10	-1.0621587	4.37615369	0.0002834	0.00103656	8.43	7.87	5.11	5.3	RCL1	RNA terminal phosphate cyclase-like 1	chr9	protein_coding
ENSG00000111817.15	1.48808168	1.57388049	8.37E-06	4.13E-05	0.1	0.12	0.33	0.51	DSE	dermatan sulfate epimerase	chr6	protein_coding
ENSG00000275741.1	-5.0252555	-3.1285772	0.0100455	0.02582742	0	0.38	0	0	RP11-40E6.1	--	chr13	antisense
ENSG00000120913.22	-1.4355876	4.63460662	1.09E-06	6.22E-06	4.82	3.6	2.07	2.08	PDLIM2	PDZ and LIM domain 2 (mystique)	chr8	protein_coding
ENSG00000250616.2	2.67092115	-0.0260343	9.40E-09	7.23E-08	0.08	0.15	0.74	1.27	RP11-455F5.	--	chr16	antisense
ENSG00000280414.1	4.81805931	-1.3969263	8.43E-08	5.70E-07	0	0.01	0.28	0.13	RP11-572N2	--	chr2	TEC
ENSG00000171564.10	8.54894951	-0.3165268	2.65E-17	5.13E-16	0	0	0.83	0.72	FGB	fibrinogen beta chain	chr4	protein_coding
ENSG00000200795.1	4.72703895	-3.141721	0.01889873	0.04519557	0	0	1.36	0.19	RNU4-1	RNA, U4 small nuclear 1	chr12	snRNA
ENSG00000101343.13	1.62583358	3.19438087	1.06E-07	7.12E-07	0.9	0.73	2.69	4.04	CRNKL1	crooked neck pre-mRNA splicing factor 1	chr20	protein_coding
ENSG00000268193.4	-1.5661993	5.08737861	9.74E-08	6.54E-07	66.22	53.74	27.45	26.56	AC002985.3	--	chr19	protein_coding
ENSG00000273391.1	1.66667128	0.67117639	8.92E-06	4.38E-05	0.45	1.75	3.83	5.4	RP11-634H2	--	chr7	antisense
ENSG00000177181.13	5.46511354	2.01397838	8.60E-35	4.93E-33	0.02	0.01	0.92	0.94	RIMKLA	ribosomal modification protein rimK-like family member A	chr1	protein_coding
ENSG00000238290.1	-1.3918772	-0.9562191	0.0044853	0.01273198	0.2	0.35	0.11	0.16	RP11-431K2	--	chr1	lincRNA
ENSG00000257017.7	7.14867602	-1.5357536	2.80E-09	2.31E-08	0	0	0.51	0.13	HP	haptoglobin	chr16	protein_coding
ENSG00000260316.2	5.25291352	-1.0272243	4.59E-10	4.19E-09	0.01	0	0.12	0.24	LA16c-306A	--	chr16	sense_intronic
ENSG00000163794.6	-2.2629953	0.51654788	7.62E-09	5.94E-08	2.59	2.14	0.81	0.51	UCN	urocortin	chr2	protein_coding
ENSG00000101132.8	2.07089824	2.46281766	1.43E-10	1.40E-09	1.15	1.14	3.02	9.98	PFDN4	prefoldin subunit 4	chr20	protein_coding
ENSG00000107185.9	-1.4039563	7.16550428	1.28E-06	7.22E-06	21.94	26.17	11.45	12.79	RGP1	RGP1 retrograde golgi transport homolog (S. cerevisiae)	chr9	protein_coding
ENSG00000141040.13	10.2786853	1.3068071	1.39E-35	8.37E-34	0	0	0.98	2	ZNF287	zinc finger protein 287	chr17	protein_coding
ENSG00000228265.4	2.51415447	-0.4697499	5.23E-07	3.15E-06	0.16	0.3	2.04	1.53	RALY-AS1	RALY antisense RNA 1	chr20	lincRNA
ENSG00000210154.1	1.85302104	1.3894834	1.09E-07	7.26E-07	8.26	17.92	62.65	62.87	MT-TD	mitochondrially encoded tRNA aspartic acid	chrM	Mt_tRNA
ENSG00000069712.10	6.08024817	-2.335817	4.74E-05	0.00020301	0	0	0	0.11	KIAA1107	KIAA1107	chr1	protein_coding
ENSG00000155465.17	5.69134915	1.00394378	2.55E-26	9.61E-25	0	0.04	1.42	1.54	SLC7A7	solute carrier family 7 (amino acid transporter light chain, y+L system), member 7	chr14	protein_coding
ENSG00000279827.1	5.19748351	-2.8969879	0.00538165	0.01488851	0	0	0.06	0.21	RP11-886P1	--	chr19	TEC
ENSG00000137767.12	-2.7561221	4.00098912	1.54E-18	3.30E-17	11.72	9.02	2.15	1.94	SQRDL	sulfide quinone reductase-like (yeast)	chr15	protein_coding
ENSG00000107371.11	-1.2230659	4.82165524	2.77E-05	0.00012385	17.75	17.59	8.85	11.37	EXOSC3	exosome component 3	chr9	protein_coding
ENSG00000174607.9	-6.4539973	0.97975559	1.01E-30	4.78E-29	0.78	0.59	0.01	0.01	UGT8	UDP glycosyltransferase 8	chr4	protein_coding
ENSG00000188818.11	2.25833652	0.3037374	6.73E-08	4.63E-07	0.04	0.14	0.54	0.57	ZDHHC11	zinc finger, DHHC-type containing 11	chr5	protein_coding
ENSG00000107863.15	1.32706748	3.30275102	1.13E-05	5.41E-05	0.44	0.82	1.83	2.38	ARHGAP21	Rho GTPase activating protein 21	chr10	protein_coding
ENSG00000228107.1	-1.8161226	-0.743675	0.00013355	0.00052381	0.85	1.52	0.38	0.5	AP000692.9	--	chr21	sense_overlapping
ENSG00000012223.11	-5.2072905	-3.0363253	0.00538165	0.01488851	0.05	0.02	0	0	LTF	lactotransferrin	chr3	protein_coding
ENSG00000088808.15	1.70311493	4.22858994	1.29E-08	9.71E-08	1.36	1.5	6.29	6.12	PPP1R13B	protein phosphatase 1, regulatory subunit 13B	chr14	protein_coding
ENSG00000132881.10	-1.2650075	1.56490212	0.00014109	0.00055006	2.2	2.13	1.55	0.85	RSG1	REM2 and RAB-like small GTPase 1	chr1	protein_coding
ENSG00000180900.15	-1.1479	8.9008118	6.56E-05	0.00027409	99.74	99.42	59.62	60.14	SCRIB	scribbled planar cell polarity protein	chr8	protein_coding
ENSG00000274949.1	1.54802379	0.44017559	5.09E-05	0.00021703	0.62	1.26	5.13	2.18	RP5-1009E2	--	chr20	processed_transcript
ENSG00000112852.6	-3.6071931	-2.0141484	6.40E-05	0.00026805	0.02	0.13	0.02	0	PCDHB2	protocadherin beta 2	chr5	protein_coding
ENSG00000123104.10	2.47062904	2.31024711	1.24E-13	1.71E-12	0.1	0.11	0.7	0.85	ITPR2	inositol 1,4,5-trisphosphate receptor, type 2	chr12	protein_coding
ENSG00000055211.11	1.91271724	3.65494446	3.94E-10	3.64E-09	2.02	2.17	7.12	14.07	GINM1	glycoprotein integral membrane 1	chr6	protein_coding
ENSG00000173991.5	1.21620246	1.54586855	0.00027658	0.00101362	0.54	0.76	1.83	2.21	TCAP	titin-cap	chr17	protein_coding
ENSG00000077348.7	-1.0134277	5.6205107	0.00045293	0.00159602	48.8	46.41	32.89	29.89	EXOSC5	exosome component 5	chr19	protein_coding
ENSG00000130520.9	-1.9949639	8.75736717	1.26E-11	1.40E-10	324.24	262.33	103.08	92.9	LSM4	LSM4 homolog, U6 small nuclear RNA associated (S. cerevisiae)	chr19	protein_coding
ENSG00000141934.8	-1.3241943	5.73571761	5.21E-06	2.66E-05	39.32	36.89	19.02	21.61	PPAP2C	phosphatidic acid phosphatase type 2C	chr19	protein_coding
ENSG00000201003.1	5.08767213	-2.9577572	0.00538165	0.01488851	0	0	2.12	0	SNORA58	small nucleolar RNA, H/ACA box 58	chr1	snoRNA
ENSG00000198887.8	2.96675445	1.20904575	1.66E-14	2.53E-13	0.02	0.12	0.43	0.98	SMC5	structural maintenance of chromosomes 5	chr9	protein_coding
ENSG00000178199.12	-1.7969655	0.05844282	1.10E-05	5.30E-05	0.18	0.24	0.07	0.09	ZC3H12D	zinc finger CCCH-type containing 12D	chr6	protein_coding
ENSG00000197576.12	4.23027804	1.10887657	1.69E-21	4.61E-20	0.08	0.11	3.3	1.5	HOXA4	homeobox A4	chr7	protein_coding
ENSG00000157152.15	-1.6170433	-1.6020978	0.01254665	0.03155686	0.08	0.06	0.03	0.03	SYN2	synapsin II	chr3	protein_coding
ENSG00000127533.3	-3.4591551	-2.1157425	0.00016274	0.00062431	0.14	0.02	0.02	0	F2RL3	coagulation factor II (thrombin) receptor-like 3	chr19	protein_coding
ENSG00000228340.4	-3.3221773	-2.7880906	0.00943894	0.02469931	0.09	0.04	0	0.01	MIR646HG	MIR646 host gene	chr20	lincRNA
ENSG00000082516.8	-1.0740851	4.44019685	0.00023756	0.00088706	4.47	4.35	2.74	2.84	GEMIN5	gem (nuclear organelle) associated protein 5	chr5	protein_coding
ENSG00000164597.12	1.14972855	1.87779576	0.00036976	0.00132672	0.22	0.25	0.67	0.71	COG5	component of oligomeric golgi complex 5	chr7	protein_coding
ENSG00000260651.1	-5.3760424	-2.9459444	0.00290522	0.00856312	0.17	0.35	0	0	AF213884.2	--	chr4	antisense
ENSG00000247982.5	-1.6330249	-1.7019698	0.01303785	0.03267232	0.15	0.1	0.08	0.03	LINC00926	long intergenic non-protein coding RNA 926	chr15	lincRNA
ENSG00000105991.7	4.33125699	0.28046111	2.71E-17	5.25E-16	0.06	0	0.87	0.93	HOXA1	homeobox A1	chr7	protein_coding
ENSG00000229544.8	6.02248524	-2.3782733	8.36E-05	0.00034052	0	0	0.04	0.11	NKX1-2	NK1 homeobox 2	chr10	protein_coding
ENSG00000275719.1	-1.322725	2.78258954	1.64E-05	7.64E-05	5.7	7.22	3.54	3.32	CTB-147N14	--	chr19	antisense
ENSG00000112773.14	2.00345817	4.16973226	3.94E-11	4.13E-10	1	1.02	4.98	5.82	FAM46A	family with sequence similarity 46, member A	chr6	protein_coding
ENSG00000265692.1	1.91622098	0.88705805	2.21E-07	1.41E-06	0.21	0.45	1.63	1.69	RP13-516M1	--	chr17	lincRNA
ENSG00000167965.16	-1.6297717	7.52178858	2.26E-08	1.64E-07	106.54	99.04	44.97	43.53	MLST8	MTOR associated protein, LST8 homolog (S. cerevisiae)	chr16	protein_coding
ENSG00000073670.12	1.58333433	2.13280686	9.21E-07	5.33E-06	0.32	0.42	1.56	1.37	ADAM11	ADAM metallopeptidase domain 11	chr17	protein_coding
ENSG00000133454.14	-1.8570472	-2.0239453	0.00956893	0.02497848	0.01	0.05	0.01	0.01	MYO18B	myosin XVIIIB	chr22	protein_coding
ENSG00000127920.5	-5.4688636	4.71883481	1.37E-51	2.02E-49	13.62	13.7	0.35	0.47	GNG11	guanine nucleotide binding protein (G protein), gamma 11	chr7	protein_coding
ENSG00000264294.1	2.40233961	0.52178387	2.97E-09	2.45E-08	4.68	3.9	36.84	24.17	SNORD55	small nucleolar RNA, C/D box 55	chr1	snoRNA
ENSG00000101098.11	3.93555106	5.26151138	2.44E-33	1.33E-31	0.6	0.86	15.55	14.15	RIMS4	regulating synaptic membrane exocytosis 4	chr20	protein_coding
ENSG00000164430.14	1.20694709	1.95972484	0.00016919	0.00064695	0.51	0.5	0.83	2.29	MB21D1	Mab-21 domain containing 1	chr6	protein_coding
ENSG00000130827.6	-1.6960553	5.95371314	7.37E-09	5.75E-08	7.03	7.06	2.93	2.86	PLXNA3	plexin A3	chrX	protein_coding
ENSG00000172780.15	-2.2204217	3.71227299	6.22E-13	7.97E-12	3.76	3.71	1.23	0.9	RAB43	RAB43, member RAS oncogene family	chr3	protein_coding
ENSG00000095713.12	10.415079	1.43407736	2.92E-37	1.91E-35	0	0	1.66	1.97	CRTAC1	cartilage acidic protein 1	chr10	protein_coding

ENSG00000132953.15	1.331684	1.06768314	0.00016048	0.00061731	0.09	0.1	0.17	0.46	XPO4	exportin 4	chr13	protein_coding
ENSG00000132535.17	-3.0174104	4.26671864	1.34E-21	3.66E-20	6.37	6.27	1.01	1.07	DLG4	discs, large homolog 4 (Drosophila)	chr17	protein_coding
ENSG00000181577.14	-8.8095344	3.99858158	1.01E-71	4.40E-69	7.04	6.16	0	0.03	C6orf223	chromosome 6 open reading frame 223	chr6	protein_coding
ENSG00000166441.11	-1.9836444	9.70074396	1.58E-11	1.73E-10	190.17	149.36	58.33	56.12	RPL27A	ribosomal protein L27a	chr11	protein_coding
ENSG00000118257.15	-2.5322095	3.78032647	4.81E-16	8.38E-15	1.92	1.77	0.48	0.37	NRP2	neuropilin 2	chr2	protein_coding
ENSG00000253741.1	1.38593297	-1.0738094	0.00959833	0.02503712	0.03	0.03	0.1	0.12	CTD-2292P1	--	chr8	antisense
ENSG00000254788.5	2.18513157	-1.4561586	0.00061439	0.00211279	0.02	0.11	0.44	0.4	CKLF-CMTM	CKLF-CMTM1 readthrough	chr16	protein_coding
ENSG00000067334.12	1.64767257	2.75304412	1.36E-07	8.92E-07	0.46	0.42	1.21	2.5	DNTTIP2	deoxynucleotidyltransferase, terminal, interacting protein 2	chr1	protein_coding
ENSG00000128713.12	6.32623166	3.95867691	4.04E-55	7.70E-53	0.03	0.37	23.15	19.61	HOXD11	homeobox D11	chr2	protein_coding
ENSG00000131375.8	1.8629623	1.43854435	8.23E-08	5.58E-07	0.18	0.23	0.66	1.38	CAPN7	calpain 7	chr3	protein_coding
ENSG00000095752.5	1.34036896	1.38726916	8.40E-05	0.00034174	0.44	0.54	1.5	1.8	IL11	interleukin 11	chr19	protein_coding
ENSG00000070610.13	-1.8539481	5.89384278	3.22E-10	3.01E-09	18.21	18.18	6.93	6.47	GBA2	glucosidase, beta (bile acid) 2	chr9	protein_coding
ENSG00000161714.10	-2.2512215	8.37922907	3.65E-14	5.33E-13	73.77	72.13	20.22	20.62	PLCD3	phospholipase C, delta 3	chr17	protein_coding
ENSG00000181619.11	1.24283063	-0.4929055	0.00633842	0.01727626	0.02	0.12	0.19	0.23	GPR135	G protein-coupled receptor 135	chr14	protein_coding
ENSG00000198087.7	2.28173045	0.48633568	1.62E-08	1.20E-07	0.03	0.1	0.44	0.43	CD2AP	CD2-associated protein	chr6	protein_coding
ENSG00000109906.12	-1.3673525	3.74086104	4.61E-06	2.39E-05	5.48	6.5	3.17	3.02	ZBTB16	zinc finger and BTB domain containing 16	chr11	protein_coding
ENSG00000279594.1	5.36404116	-2.8022277	0.00290522	0.00856312	0	0	0.18	0.03	RP11-950C1	--	chr14	TEC
ENSG00000009830.10	-1.2980563	4.64001754	9.45E-06	4.61E-05	6.07	5.68	3.4	2.97	POMT2	protein-O-mannosyltransferase 2	chr14	protein_coding
ENSG00000205426.9	-2.197326	1.98205557	2.77E-11	2.96E-10	3.33	2.09	0.6	0.97	KRT81	keratin 81, type II	chr12	protein_coding
ENSG00000197647.10	-6.9339742	-1.8836908	8.18E-08	5.55E-07	0.19	0.12	0	0	ZNF433	zinc finger protein 433	chr19	protein_coding
ENSG00000141232.4	2.24980024	4.71796022	1.17E-13	1.62E-12	2.76	3.11	17.61	19.63	TOB1	transducer of ERBB2, 1	chr17	protein_coding
ENSG00000167653.4	4.49184235	9.29224176	1.10E-42	9.66E-41	31.93	31.77	1006.33	900.39	PSCA	prostate stem cell antigen	chr8	protein_coding
ENSG00000235560.3	-1.1202169	1.66373092	0.00050856	0.00177434	2.08	1.72	1.03	1.3	AC002310.12	--	chr16	antisense
ENSG00000124191.16	-1.8743929	5.57602767	2.26E-10	2.15E-09	21.07	19.4	7.76	6.93	TOX2	TOX high mobility group box family member 2	chr20	protein_coding
ENSG00000277423.1	2.99566982	-2.0731952	0.0014675	0.00467247	0	0.14	0.62	0.92	RP11-173P1	--	chr12	lincRNA
ENSG00000068985.4	-2.4164631	-1.3457308	2.93E-05	0.00013052	0.84	0.6	0.1	0.25	PAGE1	P antigen family, member 1 (prostate associated)	chrX	protein_coding
ENSG00000147889.15	-1.5918336	9.25706303	4.38E-08	3.07E-07	219.95	251.66	112.59	95.25	CDKN2A	cyclin-dependent kinase inhibitor 2A	chr9	protein_coding
ENSG00000131747.13	2.28006451	5.3488427	3.25E-14	4.77E-13	1.78	2.12	9.39	16.03	TOP2A	topoisomerase (DNA) II alpha 170kDa	chr17	protein_coding
ENSG00000169299.12	1.00760168	1.28331543	0.00299627	0.00881199	0.42	0.32	0.77	1.22	PGM2	phosphoglucomutase 2	chr4	protein_coding
ENSG00000054219.10	6.98669944	-1.6506602	3.05E-08	2.18E-07	0	0	0.02	0.13	LY75	lymphocyte antigen 75	chr2	protein_coding
ENSG00000278989.1	-5.5276337	-2.8606046	0.00158008	0.00495521	0	0.12	0	0	RP11-762L8.	--	chr11	TEC
ENSG00000224358.1	3.99971871	-2.0224751	3.06E-05	0.00013563	0	0.01	0.19	0.14	RP11-466F5.	--	chr1	antisense
ENSG00000177189.11	1.11567161	2.88005882	0.0002567	0.00095219	0.46	0.48	1.11	1.61	RPS6KA3	ribosomal protein S6 kinase, 90kDa, polypeptide 3	chrX	protein_coding
ENSG00000153823.17	-1.9625984	3.51612542	1.37E-10	1.34E-09	5.16	4.91	1.46	1.99	PID1	phosphotyrosine interaction domain containing 1	chr2	protein_coding
ENSG00000219626.7	-2.072335	-1.5541004	0.00086862	0.00288921	0.25	0.23	0.14	0.02	FAM228B	family with sequence similarity 228, member B	chr2	protein_coding
ENSG00000259940.2	-2.5767546	-2.0269711	0.00060878	0.00209711	0.06	0.27	0	0.06	CTD-3203P2	--	chr16	antisense
ENSG00000152223.11	1.0050855	2.661707	0.00106194	0.00347011	0.25	0.28	0.72	0.7	EPG5	ectopic P-granules autophagy protein 5 homolog (C. elegans)	chr18	protein_coding
ENSG00000170542.5	2.65924918	2.49155534	1.61E-15	2.69E-14	0.18	0.42	2.4	2.59	SERPINB9	serpin peptidase inhibitor, clade B (ovalbumin), member 9	chr6	protein_coding
ENSG00000139874.5	-5.4909165	3.33560292	1.30E-45	1.39E-43	3.55	3.91	0.11	0.11	SSTR1	somatostatin receptor 1	chr14	protein_coding
ENSG00000154493.16	-1.9062571	-1.3979174	0.00142213	0.00453952	0.11	0.11	0.06	0.01	C10orf90	chromosome 10 open reading frame 90	chr10	protein_coding
ENSG00000168237.16	-1.7278526	1.39939512	3.32E-07	2.06E-06	1.41	1.62	0.58	0.63	GLYCTK	glycerate kinase	chr3	protein_coding
ENSG00000270604.4	-3.1758553	-2.8664902	0.01615662	0.03958166	0.15	0.19	0	0.04	HCG17	HLA complex group 17 (non-protein coding)	chr6	lincRNA
ENSG00000182103.4	5.77385495	-2.5463609	0.00026535	0.0009747	0	0	0.04	0.07	FAM181B	family with sequence similarity 181, member B	chr11	protein_coding
ENSG00000112414.13	1.44622945	0.40245925	0.00018285	0.00069522	0.09	0.06	0.19	0.36	GPR126	--	chr6	protein_coding
ENSG00000186577.10	-1.2063248	3.60263315	5.31E-05	0.00022541	10.41	7.23	6.04	4.13	C6orf1	chromosome 6 open reading frame 1	chr6	protein_coding
ENSG00000185950.8	-2.0269318	4.98979342	1.22E-11	1.35E-10	4.99	5.17	1.35	1.98	IRS2	insulin receptor substrate 2	chr13	protein_coding
ENSG00000196696.11	1.06480821	2.78249976	0.00050634	0.00176705	0.75	0.95	2.23	2.52	PDXDC2P	pyridoxal-dependent decarboxylase domain containing 2, pseudogene	chr16	processed_transcript
ENSG00000215915.8	-1.8578194	-0.0493215	9.82E-06	4.77E-05	0.34	0.27	0.15	0.08	ATAD3C	ATPase family, AAA domain containing 3C	chr1	protein_coding
ENSG00000173432.9	-1.7800888	4.55221795	2.31E-09	1.93E-08	36.29	40.64	13.23	16.66	SAA1	serum amyloid A1	chr11	protein_coding
ENSG00000172775.15	1.29254946	7.36601287	7.75E-06	3.85E-05	25.25	26.92	81.18	89.23	FAM192A	family with sequence similarity 192, member A	chr16	protein_coding
ENSG00000255153.1	2.24829903	0.28801844	8.03E-08	5.47E-07	0.37	0.3	2.39	1.91	TOLLIP-AS1	TOLLIP antisense RNA 1 (head to head)	chr11	antisense
ENSG00000177025.3	8.34034453	-0.5047731	1.53E-15	2.56E-14	0	0	1.58	1.31	C19orf18	chromosome 19 open reading frame 18	chr19	protein_coding
ENSG00000087502.16	1.38693951	1.81067149	2.44E-05	0.00011054	0.31	0.3	0.57	1.56	ERGIC2	ERGIC and golgi 2	chr12	protein_coding
ENSG00000108622.9	-8.0176817	2.01330648	1.50E-45	1.58E-43	4.55	3.96	0.04	0	ICAM2	intercellular adhesion molecule 2	chr17	protein_coding
ENSG00000138363.13	-1.976471	7.98310114	1.94E-11	2.10E-10	127.35	128.99	42.32	44.51	ATIC	5-aminoimidazole-4-carboxamide ribonucleotide formyltransferase/IMP cyclohydrolase	chr2	protein_coding
ENSG00000162714.11	-1.4428711	6.26065285	7.04E-07	4.15E-06	17.44	16.57	8.36	8.3	ZNF496	zinc finger protein 496	chr1	protein_coding
ENSG00000263272.1	-1.3740902	1.58398903	2.81E-05	0.00012546	1.74	1.84	0.83	1.01	CTC-524C5.2	--	chr17	antisense
ENSG00000179954.13	-2.6151491	-2.6387997	0.01923175	0.0458365	0.03	0.03	0.01	0	SSC5D	scavenger receptor cysteine rich family, 5 domains	chr19	protein_coding
ENSG00000189410.10	-1.278178	1.31956714	0.00013065	0.00051368	0.87	0.6	0.27	0.54	SH2D5	SH2 domain containing 5	chr1	protein_coding
ENSG00000214367.6	2.82786505	0.51231035	1.88E-11	2.04E-10	0.02	0.05	0.21	0.5	HAUS3	HAUS augmin-like complex, subunit 3	chr4	protein_coding
ENSG00000075891.20	8.68880876	-0.1883369	1.90E-18	4.07E-17	0	0	0.4	0.39	PAX2	paired box 2	chr10	protein_coding
ENSG00000113657.11	-5.9221543	4.18269106	4.61E-54	8.13E-52	4.84	5	0.11	0.11	DPYSL3	dihydropyrimidinase-like 3	chr5	protein_coding
ENSG00000141738.12	5.16774232	3.5192037	1.02E-42	9.09E-41	0.25	0.12	10.08	7.66	GRB7	growth factor receptor-bound protein 7	chr17	protein_coding
ENSG00000126091.18	-1.3571465	3.4101395	6.56E-06	3.30E-05	5.09	4.8	2.77	2.37	ST3GAL3	ST3 beta-galactoside alpha-2,3-sialyltransferase 3	chr1	protein_coding
ENSG00000140945.14	-2.1901671	6.14973771	1.92E-13	2.59E-12	9.62	9.83	2.38	3.32	CDH13	cadherin 13	chr16	protein_coding
ENSG00000185269.10	4.1096202	3.71717936	1.18E-32	6.23E-31	0.63	0.35	11.92	10.86	NOTUM	notum pectinacetylesterase homolog (Drosophila)	chr17	protein_coding
ENSG00000266951.2	4.87917439	-3.0678699	0.01889873	0.04519557	0	0	0	0.36	AD000091.2	--	chr19	lincRNA
ENSG00000272077.1	4.10476032	-1.2454106	9.32E-08	6.27E-07	0	0.03	0.43	0.29	RP11-348P1(	--	chr3	lincRNA
ENSG00000173517.9	1.30963624	2.70094767	2.15E-05	9.81E-05	0.16	0.13	0.43	0.55	PEAK1	pseudopodium-enriched atypical kinase 1	chr15	protein_coding
ENSG00000036530.7	7.31156115	-1.3924071	4.49E-10	4.11E-09	0	0	0.26	0.31	CYP46A1	cytochrome P450, family 46, subfamily A, polypeptide 1	chr14	protein_coding
ENSG00000121931.14	-6.6539197	-0.162285	5.55E-21	1.44E-19	0.21	0.57	0	0.01	LRIF1	ligand dependent nuclear receptor interacting factor 1	chr1	protein_coding
ENSG00000151461.18	1.77644273	3.3168796	7.01E-09	5.51E-08	0.46	0.76	2.36	3.24	UPF2	UPF2 regulator of nonsense transcripts homolog (yeast)	chr10	protein_coding
ENSG00000174007.7	2.83693372	1.34836324	2.99E-14	4.41E-13	0.21	0.23	1.63	2.57	CEP19	centrosomal protein 19kDa	chr3	protein_coding
ENSG00000180964.15	1.39107572	3.70852795	3.45E-06	1.83E-05	4.49	5.01	14.34	18.94	TCEAL8	transcription elongation factor A (SII)-like 8	chrX	protein_coding
ENSG00000206878.1	2.24911714	-2.5414026	0.01923175	0.0458365	0.21	0.23	1.03	1.96	SNORA51	small nucleolar RNA, H/ACA box 51	chr1	snoRNA
ENSG00000254815.4	2.46189393	-1.4675292	0.00025672	0.00095219	0	0.06	0.19	0.28	RP11-496I9.1	--	chr11	antisense
ENSG00000176225.11	-1.1306512	2.34159092	0.00024748	0.00092177	0.75	0.78	0.3	0.64	RTTN	rotatin	chr18	protein_coding
ENSG00000275183.1	-1.0738288	5.17031444	0.00021426	0.00080555	20.03	21.21	12.57	13.54	LENG9	leukocyte receptor cluster (LRC) member 9	chr19	protein_coding
ENSG00000149573.7	5.29735717	1.17142887	1.02E-26	3.97E-25	0.02	0.04	1.05	1.76	MPZL2	myelin protein zero-like 2	chr11	protein_coding
ENSG00000069966.17	-1.0819976	5.2992104	0.00018904	0.00071646	4.51	4.48	2.57	3.09	GNB5	guanine nucleotide binding protein (G protein), beta 5	chr15	protein_coding
ENSG00000278309.1	-4.3760883	3.81488629	5.19E-36	3.21E-34	4.08	3.2	0.33	0.14	RP11-102K1	--	chr13	lincRNA
ENSG00000172113.7	-1.1615774	3.60545345	9.56E-05	0.00038601	4.85	5.81	2.87	3.48	NME6	NME/NM23 nucleoside diphosphate kinase 6	chr3	protein_coding
ENSG00000268403.2	-1.5058441	1.57854342	5.37E-06	2.74E-05	3.45	2.72	1.42	1.47	CTD-2371O	--	chr11	antisense
ENSG00000261616.1	4.88946445	-3.0622981	0.01889873	0.04519557	0	0	0.02	0.06	RP11-6O2.3	--	chr15	antisense
ENSG00000187257.13	1.0299639	2.67044116	0.00078343	0.00263879	0.53	0.48	1.2	1.55	RSBN1L	round spermatid basic protein 1-like	chr7	protein_coding
ENSG00000138735.14	3.56298559	-0.1814469	1.90E-11	2.07E-10	0.01	0.01	0.1	0.3	PDE5A	phosphodiesterase 5A, cGMP-specific	chr4	protein_coding
ENSG00000051825.13	3.43201596	0.95593843	1.46E-16	2.67E-15	0.02	0.06	0.4	0.74	MPHOSPH9	M-phase phosphoprotein 9	chr12	protein_coding
ENSG00000202474.1	-1.4068489	-1.416685	0.01523097	0.03758211	5.17	1.19	1.35	1.83	RNA5SP283	RNA, 5S ribosomal pseudogene 283	chr9	rRNA
ENSG00000274422.1	8.43191339	-0.4054682	3.10E-16	5.50E-15	0	0	0.07	0.78	LL22NC03-2	--	chr22	lincRNA
ENSG00000119121.20	5.48410531	-2.7306244	0.00158008	0.00495521	0	0	0.03	0.01	TRPM6	transient receptor potential cation channel, subfamily M, member 6	chr9	protein_coding
ENSG00000111885.6	-2.221066	-2.5115698	0.01191236	0.03011315	0.04	0.03	0.01	0.01	MAN1A1	mannosidase, alpha, class 1A, member 1	chr6	protein_coding
ENSG00000159423.15	1.81140935	3.09719391	5.04E-09	4.03E-08	0.98	0.72	3.86	4.12	ALDH4A1	aldehyde dehydrogenase 4 family, member A1	chr1	protein_coding
ENSG00000028310.16	-1.1817879	8.21154123	4.06E-05	0.00017679	135.47	148.89	87.47	79.22	BRD9	bromodomain containing 9	chr5	protein_coding
ENSG00000119969.13	2.92115045	2.9100406	1.43E-18	3.08E-17	0.32	0.52	3.5	4.99	HELLS	helicase, lymphoid-specific	chr10	protein_coding
ENSG00000081665.12	5.23714944	0.21933221	3.53E-19	8.01E-18	0.01	0.01	0.34	0.54	ZNF506	zinc finger protein 506	chr19	protein_coding
ENSG00000269947.1	-2.4768655	-0.6773448	6.49E-07	3.85E-06	1.23	1.18	0.29	0.28	RP11-849F2.	--	chr17	lincRNA
ENSG00000256618.2	1.80593791	-1.7122785	0.00961728	0.02507838	0.03	0.07	0.23	0.28	MTRNR2L1	MT-RNR2-like 1	chr17	protein_coding
ENSG00000177875.4	4.56162197	1.16509325	7.72E-24	2.45E-22	0.11	0	1.86	1.91	CCDC184	coiled-coil domain containing 184	chr12	protein_coding
ENSG00000261663.1	-1.706847	-1.9407102	0.01381259	0.03443739	0.58	0.07	0.2	0.07	RP11-304L1	--	chr16	antisense
ENSG00000135318.10	-4.2014203	3.58829895	9.17E-34	5.06E-32	4.81	3.89	0.35	0.28	NT5E	5'-nucleotidase, ecto (CD73)	chr6	protein_coding
ENSG00000100815.11	2.06488565	1.73156361	1.27E-09	1.09E-08	0.12	0.08	0.36	0.77	TRIP11	thyroid hormone receptor interactor 11	chr14	protein_coding
ENSG00000238141.2	6.12390088	-2.3107473	4.74E-05	0.00020301	0	0	0.46	0.35	BRWD1-AS1	BRWD1 antisense RNA 1	chr21	antisense
ENSG00000007866.17	-1.2230462	5.39126868	2.57E-05	0.00011564	15.79	16.09	9.17	9.02	TEAD3	TEA domain family member 3	chr6	protein_coding
ENSG00000203883.6	-1.1210297	1.49355976	0.00065975	0.00225583	1.56	1.75	0.95	1.08	SOX18	SRY (sex determining region Y)-box 18	chr20	protein_coding
ENSG00000272489.1	6.68960776	-1.8871694	3.73E-07	2.30E-06	0	0	0.1	0.41	RP11-182L2	--	chr10	sense_intronic
ENSG00000113328.17	-4.6283706	-1.2123969	1.47E-09	1.25E-08	0.21	0.3	0.03	0	CCNG1	cyclin G1	chr5	protein_coding
ENSG00000171943.10	1.24644383	0.83072878	0.00058016	0.00200859	0.11	0.12	0.31	0.41	SRGAP2C	SLIT-ROBO Rho GTPase activating protein 2C	chr1	protein_coding
ENSG00000050165.16	-4.4496648	3.38638614	1.14E-35	6.97E-34	5.71	5.01	0.4	0.25	DKK3	dickkopf WNT signaling pathway inhibitor 3	chr11	protein_coding
ENSG00000181666.16	-1.2002371	5.28963148	3.63E-05	0.00015928	8.47	9.39	5.07	5.29	HKR1	HKR1, GLI-Kruppel zinc finger family member	chr19	protein_coding
ENSG00000164076.15	1.27397481	-1.1463673	0.01979609	0.04704122	0.06	0.04	0.14	0.21	CAMKV	CaM kinase-like vesicle-associated	chr3	protein_coding
ENSG00000261242.1	3.31438067	-1.8456324	0.00016407	0.00062818	0	0.05	0.12	0.6	CTD-2302E2	--	chr14	antisense
ENSG00000178752.14	-2.6311256	1.58201997	8.28E-14	1.17E-12	3.59	4.23	0.75	0.92	FAM132B	family with sequence similarity 132, member B	chr2	protein_coding
ENSG00000134709.9	7.88382811	-0.8986966	3.28E-12	3.89E-11	0	0	0.09	0.24	HOOK1	hook microtubule-tethering protein 1	chr1	protein_coding
ENSG00000109736.13	-2.0962744	4.5843313	3.61E-12	4.26E-11	14.5	11.94	4.61	3.62	MFSD10	major facilitator superfamily domain containing 10	chr4	protein_coding
ENSG00000261105.4	1.65361817	-1.8085564	0.01883218	0.04519557	0	0.29	0.76	0.46	LMO7-AS1	LMO7 antisense RNA 1	chr13	antisense
ENSG00000180488.13	1.5643555	0.29742114	8.90E-05	0.00036048	0.09	0.06	0.31	0.27	FAM73A	family with sequence similarity 73, member A	chr1	protein_coding
ENSG00000039987.5	-6.6314455	0.6420763	4.38E-28	1.83E-26	0.97	1.27	0.03	0	BEST2	bestrophin 2	chr19	protein_coding
ENSG00000129214.13	2.29130251	-0.5329176	4.10E-06	2.14E-05	0.14	0.07	0.74	0.67	SHBG	sex hormone-binding globulin	chr17	protein_coding
ENSG00000024526.15	3.03108473	1.34682105	1.86E-15	3.10E-14	0.08	0.07	0.59	1.14	DEPDC1	DEP domain containing 1	chr1	protein_coding
ENSG00000274349.3	3.11624873	-1.9895959	0.00060878	0.00209711	0	0.01	0.06	0.1	ZNF658	zinc finger protein 658	chr9	protein_coding
ENSG00000185304.13	7.73405147	-1.0350439	1.76E-12	2.16E-11	0	0	0.12	0.13	RGPD2	RANBP2-like and GRIP domain containing 2	chr2	protein_coding
ENSG00000104450.11	1.64292369	0.73821238	1.07E-05	5.16E-05	0.17	0.1	0.39	0.74	SPAG1	sperm associated antigen 1	chr8	protein_coding
ENSG00000266924.1	6.25378322	-2.2167539	1.55E-05	7.26E-05	0	0	0.4	0.56	RP11-154H1	--	chr18	lincRNA
ENSG00000049192.13	-3.6196521	-0.0629772	3.53E-13	4.64E-12	0.22	0.16	0.03	0.01	ADAMTS6	ADAM metallopeptidase with thrombospondin type 1 motif, 6	chr5	protein_coding
ENSG00000006611.14	-6.9351732	0.92703699	1.85E-31	9.13E-30	0.84	0.9	0	0.02	USH1C	Usher syndrome 1C (autosomal recessive, severe)	chr11	protein_coding
ENSG00000235445.1	5.06203518	-2.9716376	0.0100455	0.02582742	0	0	0.35	0.38	XXbac-B476	--	chr22	sense_intronic
ENSG00000070814.16	-1.9005472	9.6133244	9.42E-11	9.47E-10	157.34	192.67	64.18	60.49	TCOF1	Treacher Collins-Franceschetti syndrome 1	chr5	protein_coding
ENSG00000057657.13	-1.7940579	1.58741166	7.45E-08	5.08E-07	0.58	0.84	0.19	0.35	PRDM1	PR domain containing 1, with ZNF domain	chr6	protein_coding
ENSG00000267140.1	-1.4934706	2.50388118	1.72E-06	9.57E-06	13.93	15.76	6.62	7.42	RP11-322E1	--	chr18	protein_coding
ENSG00000165695.8	1.84788145	0.51162691	2.20E-06	1.20E-05	0.15	0.3	1.24	0.95	AK8	adenylate kinase 8	chr9	protein_coding
ENSG00000231412.2	-7.272922	-1.6085602	1.76E-09	1.50E-08	0.62	2.54	0	0	CTC-490G23	--	chr19	lincRNA
ENSG00000183778.16	-6.6391649	-2.1126835	1.05E-06	6.02E-06	0.03	0.01	0	0	B3GALT5	UDP-Gal:betaGlcNAc beta 1,3-galactosyltransferase, polypeptide 5	chr21	protein_coding
ENSG00000158552.11	-2.2236127	4.53088479	2.23E-13	2.98E-12	15.6	11.75	3.6	4.22	ZFAND2B	zinc finger, AN1-type domain 2B	chr2	protein_coding
ENSG00000102312.19	-1.3074904	5.23637924	7.34E-06	3.66E-05	22.98	22.51	14.31	10.08	PORCN	porcupine homolog (Drosophila)	chrX	protein_coding
ENSG00000163964.12	1.25318768	2.23870446	6.98E-05	0.00029015	3.36	3.18	10.03	10.78	PIGX	phosphatidylinositol glycan anchor biosynthesis, class X	chr3	protein_coding
ENSG00000280893.1	-1.4987859	7.3571688	2.48E-07	1.57E-06	87.01	87.13	39.44	42.73	PAGR1	PAXIP1 associated glutamate-rich protein 1	chr16	protein_coding
ENSG00000182095.13	-1.2961316	7.3298983	7.31E-06	3.64E-05	15.25	16.31	8.75	8.36	TNRC18	trinucleotide repeat containing 18	chr7	protein_coding
ENSG00000111481.8	-1.547189	6.80302288	1.08E-07	7.23E-07	48.16	42.8	20.86	20.62	COPZ1	coatomer protein complex, subunit zeta 1	chr12	protein_coding
ENSG00000124813.19	-3.9185407	1.07976961	6.03E-21	1.56E-19	0.47	0.43	0.04	0.04	RUNX2	runt-related transcription factor 2	chr6	protein_coding
ENSG00000154016.12	5.14718502	-0.3590513	1.36E-14	2.08E-13	0.02	0	0.5	0.52	GRAP	GRB2-related adaptor protein	chr17	protein_coding
ENSG00000108588.12	1.11049371	5.13421715	0.00013334	0.0005233	4.29	5.37	10.44	17.49	CCDC47	coiled-coil domain containing 47	chr17	protein_coding
ENSG00000272114.1	-4.8079387	-3.2306674	0.01889873	0.04519557	0.28	0.6	0	0	RP1-261G23	--	chr6	antisense
ENSG00000125850.9	9.69880067	0.74776913	1.21E-28	5.19E-27	0	0	2.49	1.86	OVOL2	ovo-like zinc finger 2	chr20	protein_coding
ENSG00000103226.16	1.60700463	2.83598223	2.16E-07	1.38E-06	0.5	0.79	2.67	2.58	NOMO3	NODAL modulator 3	chr16	protein_coding
ENSG00000120832.8	1.37497348	1.34644056	5.15E-05	0.00021919	0.25	0.38	0.79	1.38	MTERF2	mitochondrial transcription termination factor 2	chr12	protein_coding
ENSG00000160948.12	-1.1759397	5.73802697	4.95E-05	0.00021135	57.33	51.38	36.45	27.45	VPS28	vacuolar protein sorting 28 homolog (S. cerevisiae)	chr8	protein_coding
ENSG00000138757.13	1.32208906	4.01246294	8.57E-06	4.22E-05	1.31	1.67	3.22	6.8	G3BP2	GTPase activating protein (SH3 domain) binding protein 2	chr4	protein_coding
ENSG00000154328.14	-1.8220852	4.58345102	1.00E-09	8.79E-09	12.2	9.57	4.06	4.15	NEIL2	nei endonuclease VIII-like 2 (E. coli)	chr8	protein_coding
ENSG00000113522.12	2.81431636	2.94966005	9.88E-18	1.99E-16	0.17	0.19	0.75	2.75	RAD50	RAD50 homolog (S. cerevisiae)	chr5	protein_coding
ENSG00000226287.6	3.56688915	-0.8810947	1.56E-08	1.16E-07	0.01	0.04	0.49	0.44	TMEM191A	transmembrane protein 191A (pseudogene)	chr22	processed_transcript
ENSG00000176076.7	-3.7023545	-0.6844266	1.13E-10	1.12E-09	0.58	0.63	0.04	0.07	KCNE5	potassium channel, voltage gated subfamily E regulatory beta subunit 5	chrX	protein_coding
ENSG00000163430.8	1.44429164	6.36805851	6.89E-07	4.07E-06	5.08	7.02	18.95	25.01	FSTL1	follistatin-like 1	chr3	protein_coding
ENSG00000123505.13	1.15884321	3.66096127	9.95E-05	0.00040105	1.5	1.92	4	6.21	AMD1	adenosylmethionine decarboxylase 1	chr6	protein_coding
ENSG00000204334.7	7.60617989	-1.1391976	9.11E-12	1.03E-10	0	0	0.75	1.86	ERICH2	glutamate-rich 2	chr2	protein_coding
ENSG00000129514.5	2.43475547	-0.2200203	1.66E-07	1.08E-06	0.07	0.06	0.46	0.52	FOXA1	forkhead box A1	chr14	protein_coding
ENSG00000149968.10	6.40812948	0.79695221	1.12E-26	4.33E-25	0	0.03	1.4	2.04	MMP3	matrix metallopeptidase 3	chr11	protein_coding
ENSG00000131737.5	-1.5475345	1.39572383	4.50E-06	2.34E-05	1.73	1.86	0.95	0.68	KRT34	keratin 34, type I	chr17	protein_coding
ENSG00000148153.12	1.50969515	2.40008444	1.87E-06	1.03E-05	0.45	0.58	1.37	2.56	INIP	INTS3 and NABP interacting protein	chr9	protein_coding
ENSG00000182552.13	1.45090867	1.87217895	8.77E-06	4.31E-05	0.5	0.68	1.31	2.98	RWDD4	RWD domain containing 4	chr4	protein_coding
ENSG00000172482.4	5.84975207	-2.4948111	0.00026535	0.0009747	0	0	0.03	0.2	AGXT	alanine-glyoxylate aminotransferase	chr2	protein_coding
ENSG00000083444.15	-1.8347859	9.88838946	3.71E-10	3.45E-09	428.55	386.45	155.94	148.39	PLOD1	procollagen-lysine, 2-oxoglutarate 5-dioxygenase 1	chr1	protein_coding
ENSG00000129317.13	2.53804529	0.26564539	3.15E-09	2.59E-08	0.02	0.01	0.11	0.18	PUS7L	pseudouridylate synthase 7 homolog (S. cerevisiae)-like	chr12	protein_coding
ENSG00000174652.16	1.33565844	1.80858176	4.49E-05	0.00019439	0.34	0.27	0.84	1.24	ZNF266	zinc finger protein 266	chr19	protein_coding
ENSG00000171604.10	-5.3655688	4.67404567	4.78E-50	6.59E-48	15.02	14.51	0.52	0.44	CXXC5	CXXC finger protein 5	chr5	protein_coding
ENSG00000184068.2	2.42544996	-0.6626845	3.42E-06	1.81E-05	0.07	0.02	0.34	0.33	RP5-821D11	--	chr22	antisense
ENSG00000143842.13	-1.5863212	4.6661125	8.16E-08	5.55E-07	8.44	8.75	3.77	3.86	SOX13	SRY (sex determining region Y)-box 13	chr1	protein_coding
ENSG00000155329.10	2.01731145	0.89979748	6.43E-08	4.44E-07	0.16	0.3	1.06	1.45	ZCCHC10	zinc finger, CCHC domain containing 10	chr5	protein_coding
ENSG00000158457.5	3.60428636	3.07541663	1.01E-25	3.67E-24	0.5	0.59	9.13	8.64	TSPAN33	tetraspanin 33	chr7	protein_coding
ENSG00000237476.1	4.8556806	-0.132853	1.03E-15	1.75E-14	0	0.12	3.42	1.59	XXbac-B135	--	chr22	lincRNA
ENSG00000003400.13	2.07329306	1.79641119	1.00E-09	8.77E-09	0.16	0.15	0.7	1.06	CASP10	caspase 10, apoptosis-related cysteine peptidase	chr2	protein_coding
ENSG00000164236.10	-4.9969602	2.7690306	5.76E-38	3.89E-36	0.9	1.44	0.06	0.04	ANKRD33B	ankyrin repeat domain 33B	chr5	protein_coding
ENSG00000087245.11	-1.1812981	2.6112072	0.00011447	0.00045461	1.5	1.39	0.67	1.03	MMP2	matrix metallopeptidase 2	chr16	protein_coding
ENSG00000081087.13	4.89245504	1.17865142	3.08E-25	1.08E-23	0.04	0.01	0.57	1.44	OSTM1	osteopetrosis associated transmembrane protein 1	chr6	protein_coding
ENSG00000229719.3	5.70180177	-2.5951384	0.00047761	0.00167191	0	0	0.09	0.03	AP001187.9	--	chr11	lincRNA
ENSG00000103351.11	-1.2243854	2.83537903	6.37E-05	0.00026726	0.79	1.36	0.68	0.54	CLUAP1	clusterin associated protein 1	chr16	protein_coding
ENSG00000203879.10	-1.3356766	7.11532781	3.94E-06	2.06E-05	60.64	66.16	35.54	31.25	GDI1	GDP dissociation inhibitor 1	chrX	protein_coding
ENSG00000112182.13	-5.2118379	-3.0342168	0.00538165	0.01488851	0	0.02	0	0	BACH2	BTB and CNC homology 1, basic leucine zipper transcription factor 2	chr6	protein_coding
ENSG00000164161.8	-9.4611817	0.41987993	3.93E-27	1.57E-25	0.25	0.16	0	0	HHIP	hedgehog interacting protein	chr4	protein_coding
ENSG00000135519.6	1.44724949	0.174009	0.0002816	0.00103062	0.15	0.08	0.4	0.46	KCNH3	potassium channel, voltage gated eag related subfamily H, member 3	chr12	protein_coding
ENSG00000064726.8	2.69617908	3.26866279	4.93E-17	9.36E-16	0.49	0.77	3.63	7.24	BTBD1	BTB (POZ) domain containing 1	chr15	protein_coding
ENSG00000261460.1	1.67756181	-1.6226175	0.00945988	0.02474059	0.05	0.01	0.07	0.19	RP11-106M3	--	chr15	antisense

ENSG00000171757.14	5.45007274	-2.7494288	0.00158008	0.00495521	0	0	0.03	0.13	LRRC34	leucine rich repeat containing 34	chr3	protein_coding
ENSG00000088356.5	1.90701128	6.62992211	9.71E-11	9.73E-10	18.07	15.66	83.32	85.4	PDRG1	p53 and DNA-damage regulated 1	chr20	protein_coding
ENSG00000149380.10	-8.4767682	5.75881146	8.75E-87	8.39E-84	40.67	36.22	0.14	0.14	P4HA3	prolyl 4-hydroxylase, alpha polypeptide III	chr11	protein_coding
ENSG00000134684.9	-2.1186201	9.52449357	7.76E-13	9.84E-12	299.16	299.58	94.01	89.54	YARS	tyrosyl-tRNA synthetase	chr1	protein_coding
ENSG00000175727.12	1.01408238	6.60805455	0.0004259	0.00150964	5.61	5.49	15.85	13.96	MLXIP	MLX interacting protein	chr12	protein_coding
ENSG00000179428.2	-5.9963854	-2.5693515	0.00014842	0.00057423	0.22	0.06	0	0	AC073072.5	--	chr7	antisense
ENSG00000105821.13	2.60619134	3.1875936	5.12E-16	8.92E-15	0.24	0.64	1.92	5.23	DNAJC2	DnaJ (Hsp40) homolog, subfamily C, member 2	chr7	protein_coding
ENSG00000271614.1	1.76954714	-1.7406754	0.00961728	0.02507838	0.04	0	0.12	0.09	LINC00936	long intergenic non-protein coding RNA 936	chr12	lincRNA
ENSG00000115325.12	-1.7931667	4.14603712	2.28E-09	1.91E-08	7.83	8.63	3.4	2.91	DOK1	docking protein 1, 62kDa (downstream of tyrosine kinase 1)	chr2	protein_coding
ENSG00000115827.12	3.5973204	0.54768857	4.00E-15	6.46E-14	0.02	0.04	0.51	0.43	DCAF17	DDB1 and CUL4 associated factor 17	chr2	protein_coding
ENSG00000185736.14	2.35927285	-0.9785574	5.34E-05	0.00022654	0	0.03	0.04	0.14	ADARB2	adenosine deaminase, RNA-specific, B2 (non-functional)	chr10	protein_coding
ENSG00000262712.1	1.0448957	1.08334761	0.00271959	0.00814315	0.45	0.65	1.28	1.76	RP11-295D4	--	chr16	sense_intronic
ENSG00000188725.6	1.29664984	2.00159839	5.44E-05	0.00023036	0.63	0.64	1.11	3.07	SMIM15	small integral membrane protein 15	chr5	protein_coding
ENSG00000241043.1	-2.7530435	-1.2535356	4.95E-06	2.55E-05	0.26	0.32	0.05	0.06	GVQW1	GVQW motif containing 1	chr9	protein_coding
ENSG00000125945.13	1.0870107	2.59061907	0.00042969	0.00152217	0.61	0.79	1.64	2.34	ZNF436	zinc finger protein 436	chr1	protein_coding
ENSG00000123609.9	1.57404585	2.06855464	1.15E-06	6.52E-06	0.94	1.12	3.61	4.56	NMI	N-myc (and STAT) interactor	chr2	protein_coding
ENSG00000104043.13	7.52281736	-1.2094832	3.23E-11	3.41E-10	0	0	0.07	0.19	ATP8B4	ATPase, class I, type 8B, member 4	chr15	protein_coding
ENSG00000230257.1	-9.4690357	0.42606014	3.19E-27	1.28E-25	1.72	2.33	0	0	NFE4	nuclear factor, erythroid 4	chr7	antisense
ENSG00000163734.4	-7.4746734	3.14268722	8.21E-57	1.74E-54	13.51	13	0.09	0.1	CXCL3	chemokine (C-X-C motif) ligand 3	chr4	protein_coding
ENSG00000204588.5	-1.8031101	3.77080996	2.49E-09	2.07E-08	7.3	6.78	2.63	2.75	LINC01123	long intergenic non-protein coding RNA 1123	chr2	lincRNA
ENSG00000116005.10	2.01490792	5.33099078	1.29E-11	1.43E-10	2.44	2.38	10.44	15.67	PCYOX1	prenylcysteine oxidase 1	chr2	protein_coding
ENSG00000187260.14	-5.3590848	-0.5660695	1.11E-15	1.88E-14	0.49	0.43	0.03	0	WDR86	WD repeat domain 86	chr7	protein_coding
ENSG00000224397.4	-5.1633981	0.6467021	1.55E-24	5.17E-23	1.33	1.11	0.02	0.07	LINC01272	long intergenic non-protein coding RNA 1272	chr20	lincRNA
ENSG00000128683.12	3.26608551	1.13546481	3.41E-16	6.02E-15	0.1	0.06	1.03	1.06	GAD1	glutamate decarboxylase 1 (brain, 67kDa)	chr2	protein_coding
ENSG00000183864.4	1.28069342	6.52666268	9.73E-06	4.73E-05	9.16	9.92	30.05	31.72	TOB2	transducer of ERBB2, 2	chr22	protein_coding
ENSG00000231485.1	5.32944093	-2.821228	0.00290522	0.00856312	0	0	0.21	0.79	RP4-535B20.	--	chr1	lincRNA
ENSG00000232164.1	4.96168166	-0.0272676	1.25E-16	2.30E-15	0.02	0.03	0.74	1.44	AC092669.3	--	chr2	lincRNA
ENSG00000255856.2	2.24810695	-2.5410053	0.01923175	0.0458365	0	0.07	0.16	0.3	RP11-87C12	--	chr12	lincRNA
ENSG00000250920.1	-7.9782845	-0.9889937	1.13E-13	1.56E-12	1.19	0.65	0	0	RP11-297P1	--	chr4	lincRNA
ENSG00000156983.14	-1.056738	6.86960373	0.00024077	0.00089826	26.37	27.43	17.67	16.76	BRPF1	bromodomain and PHD finger containing, 1	chr3	protein_coding
ENSG00000235848.3	3.99253335	-2.0276299	3.06E-05	0.00013563	0.01	0	0.11	0.11	RMDN2-AS1	RMDN2 antisense RNA 1	chr2	antisense
ENSG00000137038.7	-1.9426462	6.59586856	4.48E-11	4.67E-10	46.31	43.89	14.96	16.34	TMEM261	transmembrane protein 261	chr9	protein_coding
ENSG00000139629.14	3.23427694	-0.0036535	2.80E-11	2.99E-10	0	0.05	0.36	0.29	GALNT6	polypeptide N-acetylgalactosaminyltransferase 6	chr12	protein_coding
ENSG00000174004.5	2.80484967	-1.481136	6.51E-05	0.00027249	0.04	0	0.27	0.15	NRROS	negative regulator of reactive oxygen species	chr3	protein_coding
ENSG00000278090.1	-2.0602446	-2.3385662	0.01324986	0.0331641	0.13	0.02	0	0.04	RP11-6O2.2	--	chr15	lincRNA
ENSG00000279185.1	3.02449769	-2.6585137	0.00943894	0.02469931	0	0	0.01	0	CTA-929C8.9	--	chr22	sense_intronic
ENSG00000211584.12	-1.2260198	5.46473145	2.44E-05	0.00011039	15.66	13.28	8.77	7.71	SLC48A1	solute carrier family 48 (heme transporter), member 1	chr12	protein_coding
ENSG00000078487.16	1.48047242	1.87924392	5.90E-06	2.99E-05	0.52	0.72	2.32	2.3	ZCWPW1	zinc finger, CW type with PWWP domain 1	chr7	protein_coding
ENSG00000168876.7	5.37729561	0.71531433	7.58E-23	2.24E-21	0.02	0.02	0.58	1.33	ANKRD49	ankyrin repeat domain 49	chr11	protein_coding
ENSG00000261215.1	-2.5461273	2.05102705	5.06E-14	7.28E-13	1.98	1.51	0.49	0.31	RP11-195F19	--	chr9	processed_transcript
ENSG00000188811.11	2.01424045	2.69744983	2.39E-10	2.26E-09	0.62	0.53	2.82	3.4	NHLRC3	NHL repeat containing 3	chr13	protein_coding
ENSG00000053108.15	-14.133248	5.08424228	1.11E-92	1.56E-89	9.89	10.4	0	0	FSTL4	follistatin-like 4	chr5	protein_coding
ENSG00000160193.10	-1.6920666	5.39450439	8.82E-09	6.82E-08	22.9	22.11	9.48	9.07	WDR4	WD repeat domain 4	chr21	protein_coding
ENSG00000149948.12	-3.9561737	0.36142224	2.65E-17	5.13E-16	0.18	0.1	0.01	0.01	HMGA2	high mobility group AT-hook 2	chr12	protein_coding
ENSG00000121966.6	-10.093682	5.30410087	1.61E-90	1.76E-87	34.01	29.42	0.06	0.01	CXCR4	chemokine (C-X-C motif) receptor 4	chr2	protein_coding
ENSG00000243224.1	-1.937465	-1.9868187	0.00653791	0.01776746	0.29	0.18	0.06	0.1	RP5-1157M2	--	chr3	antisense
ENSG00000233175.2	2.81573838	0.62716475	7.39E-12	8.40E-11	0.15	0.1	1.17	1.21	CTD-2020K1	--	chr17	antisense
ENSG00000235280.2	6.37984316	-2.1244714	5.19E-06	2.66E-05	0	0	0.36	0.57	MCF2L-AS1	MCF2L antisense RNA 1	chr13	antisense
ENSG00000166736.10	-7.7281064	-1.2134677	3.98E-12	4.67E-11	0.3	0.2	0	0	HTR3A	5-hydroxytryptamine (serotonin) receptor 3A, ionotropic	chr11	protein_coding
ENSG00000100814.16	-1.224785	4.77188734	2.74E-05	0.00012274	17.23	12.55	8.21	8.81	CCNB1IP1	cyclin B1 interacting protein 1, E3 ubiquitin protein ligase	chr14	protein_coding
ENSG00000143919.13	-1.0074148	1.98661783	0.00143604	0.00458202	1.69	1.5	0.88	1.24	CAMKMT	calmodulin-lysine N-methyltransferase	chr2	protein_coding
ENSG00000133134.10	2.38638562	4.30628601	8.35E-15	1.31E-13	4.03	4.68	28.82	31.84	BEX2	brain expressed X-linked 2	chrX	protein_coding
ENSG00000171033.11	-3.7886039	-1.8821375	1.61E-05	7.52E-05	0.04	0.13	0.02	0	PKIA	protein kinase (cAMP-dependent, catalytic) inhibitor alpha	chr8	protein_coding
ENSG00000074657.12	-1.416889	3.3294794	2.44E-06	1.33E-05	1.36	1.76	0.57	0.99	ZNF532	zinc finger protein 532	chr18	protein_coding
ENSG00000166986.11	-1.5173871	8.3145835	1.73E-07	1.12E-06	131.54	131.49	61.91	60.45	MARS	methionyl-tRNA synthetase	chr12	protein_coding
ENSG00000276649.1	-3.3958809	-2.1662272	0.0002605	0.00096399	0.2	0.12	0.02	0.02	RP5-858L17.	--	chr20	antisense
ENSG00000184678.9	2.46241452	4.10892941	1.90E-15	3.16E-14	1.66	2.12	14.37	13.3	HIST2H2BE	histone cluster 2, H2be	chr1	protein_coding
ENSG00000105647.13	-1.0897058	7.60353131	0.00015111	0.00058359	56.08	49.3	33.48	32.5	PIK3R2	phosphoinositide-3-kinase, regulatory subunit 2 (beta)	chr19	protein_coding
ENSG00000135686.11	2.06396554	4.73814189	7.00E-12	7.97E-11	3.99	3.65	21.36	21.23	KLHL36	kelch-like family member 36	chr16	protein_coding
ENSG00000151332.17	1.76125179	1.25222692	5.17E-07	3.12E-06	0.39	0.52	1.23	2.86	MBIP	MAP3K12 binding inhibitory protein 1	chr14	protein_coding
ENSG00000137076.17	-1.8573798	9.61093683	2.33E-10	2.22E-09	112.71	112.22	43.92	38.65	TLN1	talin 1	chr9	protein_coding
ENSG00000185127.6	1.17974706	0.78272143	0.00096449	0.00317723	0.34	0.27	0.77	1.07	C6orf120	chromosome 6 open reading frame 120	chr6	protein_coding
ENSG00000120798.15	2.59631253	1.21519584	2.97E-12	3.54E-11	0.15	0.07	0.95	0.84	NR2C1	nuclear receptor subfamily 2, group C, member 1	chr12	protein_coding
ENSG00000149311.16	4.96212077	2.09926105	2.46E-32	1.27E-30	0.01	0.01	0.38	0.57	ATM	ATM serine/threonine kinase	chr11	protein_coding
ENSG00000146476.9	3.09087049	0.50237261	1.22E-12	1.52E-11	0.08	0.09	0.43	1.52	C6orf211	--	chr6	protein_coding
ENSG00000150316.10	1.48342638	5.21529619	4.17E-07	2.56E-06	8.89	11.18	29.76	45.36	CWC15	CWC15 spliceosome-associated protein	chr11	protein_coding
ENSG00000261582.1	-1.239445	5.37442563	2.02E-05	9.24E-05	101.42	76.61	50.74	49.83	RP4-614O4.1	--	chr20	antisense
ENSG00000114346.12	2.85906792	2.44780067	3.48E-17	6.67E-16	0.26	0.19	1.51	2.96	ECT2	epithelial cell transforming 2	chr3	protein_coding
ENSG00000183668.16	6.73575057	-1.8595055	2.24E-07	1.42E-06	0	0	0.16	0.1	PSG9	pregnancy specific beta-1-glycoprotein 9	chr19	protein_coding
ENSG00000106701.10	3.56494809	-1.6623614	2.93E-05	0.00013031	0	0.01	0.03	0.06	FSD1L	fibronectin type III and SPRY domain containing 1-like	chr9	protein_coding
ENSG00000143147.13	-1.2036391	3.65100642	5.31E-05	0.00022556	1.6	1.51	0.9	0.9	GPR161	G protein-coupled receptor 161	chr1	protein_coding
ENSG00000170271.9	4.91020659	-3.0511096	0.0100455	0.02582742	0	0	0.05	0.02	FAXDC2	fatty acid hydroxylase domain containing 2	chr5	protein_coding
ENSG00000183044.10	5.37731176	-0.9218147	6.98E-11	7.14E-10	0	0	0.12	0.19	ABAT	4-aminobutyrate aminotransferase	chr16	protein_coding
ENSG00000109794.12	-10.730944	1.67925997	1.07E-42	9.46E-41	1.87	1.2	0	0	FAM149A	family with sequence similarity 149, member A	chr4	protein_coding
ENSG00000159140.16	1.21972071	7.19969748	2.38E-05	0.00010789	6.15	6.71	19.66	20.25	SON	SON DNA binding protein	chr21	protein_coding
ENSG00000168216.9	1.25547481	1.68477369	0.00014387	0.00056016	0.46	0.79	1.05	2.92	LMBRD1	LMBR1 domain containing 1	chr6	protein_coding
ENSG00000100311.15	-3.2199443	1.80207718	2.68E-19	6.13E-18	1.3	1.58	0.15	0.25	PDGFB	platelet-derived growth factor beta polypeptide	chr22	protein_coding
ENSG00000167077.11	-7.0314087	2.68517788	3.40E-51	4.85E-49	2.68	2.43	0	0.05	MEI1	meiosis inhibitor 1	chr22	protein_coding
ENSG00000168243.9	1.12281444	-0.3063231	0.01168849	0.02965671	0.05	0.09	0.15	0.25	GNG4	guanine nucleotide binding protein (G protein), gamma 4	chr1	protein_coding
ENSG00000136938.8	-1.312523	9.11945908	5.43E-06	2.76E-05	337.29	542.54	222.54	248.99	ANP32B	acidic (leucine-rich) nuclear phosphoprotein 32 family, member B	chr9	protein_coding
ENSG00000255020.1	6.26658971	-2.2093414	1.55E-05	7.26E-05	0	0	0.32	0.25	AF131216.5	--	chr8	antisense
ENSG00000176046.8	-1.6783952	7.94154781	8.80E-09	6.80E-08	50.01	59.47	24.15	21.3	NUPR1	nuclear protein, transcriptional regulator, 1	chr16	protein_coding
ENSG00000148200.15	-1.4508022	2.30519259	4.20E-06	2.19E-05	0.78	0.87	0.47	0.33	NR6A1	nuclear receptor subfamily 6, group A, member 1	chr9	protein_coding
ENSG00000135315.10	2.55046707	-1.0021119	9.67E-06	4.71E-05	0.02	0.02	0.07	0.22	CEP162	centrosomal protein 162kDa	chr6	protein_coding
ENSG00000048342.14	-1.5788501	2.78007083	3.32E-07	2.06E-06	1.05	1.51	0.49	0.65	CC2D2A	coiled-coil and C2 domain containing 2A	chr4	protein_coding
ENSG00000131149.16	1.20170466	6.46626941	3.22E-05	0.00014265	5.54	5.84	16.91	17.99	GSE1	Gse1 coiled-coil protein	chr16	protein_coding
ENSG00000259288.4	1.78952886	1.07131887	6.37E-07	3.78E-06	0.24	0.13	0.67	1.07	PAK6	p21 protein (Cdc42/Rac)-activated kinase 6	chr15	protein_coding
ENSG00000246548.3	-5.5258708	-2.8614387	0.00158008	0.00495521	0.02	0.08	0	0	RP11-7F17.5	--	chr14	lincRNA
ENSG00000120802.12	1.54292295	4.58207194	1.88E-07	1.21E-06	1.17	1.58	3.41	7.33	TMPO	thymopoietin	chr12	protein_coding
ENSG00000165152.7	-1.6459119	3.97548081	3.92E-08	2.76E-07	3.56	3.63	1.54	1.51	TMEM246	transmembrane protein 246	chr9	protein_coding
ENSG00000072571.18	2.67827953	2.07699508	7.72E-15	1.22E-13	0.26	0.28	1.39	3.3	HMMR	hyaluronan-mediated motility receptor (RHAMM)	chr5	protein_coding
ENSG00000138190.15	2.65096372	-0.7563642	2.01E-06	1.10E-05	0.02	0.02	0.04	0.28	EXOC6	exocyst complex component 6	chr10	protein_coding
ENSG00000089006.15	1.58715573	4.60819158	8.52E-08	5.76E-07	2.92	3.19	9.99	14.57	SNX5	sorting nexin 5	chr20	protein_coding
ENSG00000154917.9	-1.3861269	3.44937122	3.94E-06	2.06E-05	2.55	1.95	1.02	1.29	RAB6B	RAB6B, member RAS oncogene family	chr3	protein_coding
ENSG00000136738.13	1.08026354	2.9178611	0.00038513	0.00137735	1	1.62	2.97	4.42	STAM	signal transducing adaptor molecule (SH3 domain and ITAM motif) 1	chr10	protein_coding
ENSG00000237101.1	2.64033452	-1.9049618	0.0017973	0.00556364	0	0.06	0.22	0.33	RP11-365O1	--	chr1	antisense
ENSG00000070915.8	10.2041508	8.72810528	2.15E-120	8.58E-117	0.1	0.11	162.53	165.83	SLC12A3	solute carrier family 12 (sodium/chloride transporter), member 3	chr16	protein_coding
ENSG00000270638.1	2.32708127	-2.1132856	0.00923116	0.02429359	0	0.05	0.16	0.2	RP3-466P17.	--	chr6	lincRNA
ENSG00000125257.12	1.37427042	2.38058743	1.31E-05	6.24E-05	0.31	0.43	1.11	1.44	ABCC4	ATP-binding cassette, sub-family C (CFTR/MRP), member 4	chr13	protein_coding
ENSG00000250328.4	-6.8830937	-1.9248975	1.35E-07	8.87E-07	0.14	0.18	0	0	MGC32805	--	chr5	antisense
ENSG00000047617.13	2.53417289	-0.344318	1.52E-07	9.94E-07	0.03	0.06	0.44	0.21	ANO2	anoctamin 2, calcium activated chloride channel	chr12	protein_coding
ENSG00000123342.14	-2.4060906	-0.6472648	4.91E-07	2.97E-06	0.4	0.3	0.05	0.12	MMP19	matrix metallopeptidase 19	chr12	protein_coding
ENSG00000013588.5	1.21087971	8.22445386	2.64E-05	0.00011886	19.92	19.76	61.47	60.88	GPRC5A	G protein-coupled receptor, class C, group 5, member A	chr12	protein_coding
ENSG00000175707.8	7.79596894	-0.9940403	1.02E-11	1.15E-10	0	0	0.84	0.18	KDF1	keratinocyte differentiation factor 1	chr1	protein_coding
ENSG00000133424.19	6.91750873	3.11363483	7.50E-52	1.12E-49	0.02	0.03	3.97	4.3	LARGE	like-glycosyltransferase	chr22	protein_coding
ENSG00000278922.1	2.44665319	-2.4250132	0.01191236	0.03011315	0	0.04	0.09	0.22	AC002310.14	--	chr16	TEC
ENSG00000137074.17	-1.1864222	5.34165856	4.41E-05	0.00019142	17.23	18.45	10.6	10.27	APTX	aprataxin	chr9	protein_coding
ENSG00000063601.15	-1.0708438	3.20237819	0.00035896	0.00129107	2.13	2.25	1.31	1.47	MTMR1	myotubularin related protein 1	chrX	protein_coding
ENSG00000129354.10	-6.7037963	4.13138625	2.78E-60	6.80E-58	14.78	17.27	0.26	0.15	AP1M2	adaptor-related protein complex 1, mu 2 subunit	chr19	protein_coding
ENSG00000230176.2	3.86161154	-1.4372358	1.56E-06	8.69E-06	0	0.08	0.87	0.82	LINC01433	long intergenic non-protein coding RNA 1433	chr20	antisense
ENSG00000256771.2	1.92845617	-0.9059401	0.00032282	0.00116916	0.04	0.05	0.15	0.29	ZNF253	zinc finger protein 253	chr19	protein_coding
ENSG00000235643.1	5.47433081	-2.7360128	0.00158008	0.00495521	0	0	0.17	0.11	RP1-69M21.2	--	chr1	lincRNA
ENSG00000197361.6	-2.0051183	-1.1383275	0.00017921	0.00068247	0.19	0.11	0.03	0.07	FBXL22	F-box and leucine-rich repeat protein 22	chr15	protein_coding
ENSG00000136141.13	1.50273623	1.76969932	4.71E-06	2.44E-05	0.24	0.17	0.82	0.74	LRCH1	leucine-rich repeats and calponin homology (CH) domain containing 1	chr13	protein_coding
ENSG00000165272.13	-3.3355596	7.83271655	7.88E-27	3.07E-25	179.93	183.43	23.58	24.39	AQP3	aquaporin 3 (Gill blood group)	chr9	protein_coding
ENSG00000155545.18	3.06968492	0.4865505	1.76E-12	2.16E-11	0.03	0.05	0.19	0.74	MIER3	mesoderm induction early response 1, family member 3	chr5	protein_coding
ENSG00000197323.9	2.53154792	2.73083073	1.06E-14	1.64E-13	0.18	0.19	1.35	1.53	TRIM33	tripartite motif containing 33	chr1	protein_coding
ENSG00000170571.10	-5.894083	-2.6363745	0.00026535	0.0009747	0.04	0.04	0	0	EMB	embigin	chr5	protein_coding
ENSG00000105974.10	-1.1662944	3.82688827	8.05E-05	0.00033119	4.09	4.56	1.97	3.18	CAV1	caveolin 1, caveolae protein, 22kDa	chr7	protein_coding
ENSG00000260816.2	5.34914687	-2.8103904	0.00290522	0.00856312	0	0	0.04	0.03	RP11-319G9	--	chr16	sense_intronic
ENSG00000148690.11	1.00161739	3.05409632	0.00093616	0.00309669	1.16	1.72	2.98	4.72	FRA10AC1	fragile site, folic acid type, rare, fra(10)(q23.3) or fra(10)(q24.2) candidate 1	chr10	protein_coding
ENSG00000203965.11	2.06499003	-0.9306304	8.43E-05	0.00034273	0.12	0	0.21	0.46	EFCAB7	EF-hand calcium binding domain 7	chr1	protein_coding
ENSG00000064309.13	-1.0031555	2.66124331	0.00105923	0.00346269	0.64	0.79	0.52	0.43	CDON	cell adhesion associated, oncogene regulated	chr11	protein_coding
ENSG00000101680.12	-2.0082287	1.97692059	1.02E-09	8.88E-09	0.51	0.55	0.2	0.15	LAMA1	laminin, alpha 1	chr18	protein_coding
ENSG00000204179.9	7.33274073	-1.3726041	4.49E-10	4.11E-09	0	0	0.15	0.26	PTPN20A	--	chr10	protein_coding
ENSG00000171246.5	2.12072183	5.77379468	1.09E-12	1.36E-11	3.36	3.09	17.65	19.8	NPTX1	neuronal pentraxin I	chr17	protein_coding
ENSG00000052850.5	5.05773746	4.69503069	4.81E-46	5.34E-44	0.18	0.25	9.69	9.07	ALX4	ALX homeobox 4	chr11	protein_coding
ENSG00000161270.18	2.58469761	-1.1688385	1.96E-05	9.01E-05	0.02	0.02	0.18	0.14	NPHS1	nephrosis 1, congenital, Finnish type (nephrin)	chr19	protein_coding
ENSG00000128268.11	1.77767326	-1.3971723	0.00266587	0.00799727	0	0.04	0.1	0.1	MGAT3	mannosyl (beta-1,4-)-glycoprotein beta-1,4-N-acetylglucosaminyltransferase	chr22	protein_coding
ENSG00000105270.13	1.45936606	0.53927781	0.00010398	0.0004176	0.23	0.09	0.57	0.63	CLIP3	CAP-GLY domain containing linker protein 3	chr19	protein_coding
ENSG00000104164.9	2.57019969	1.92470245	1.22E-13	1.68E-12	0.09	0.19	0.69	1.52	BLOC1S6	biogenesis of lysosomal organelles complex-1, subunit 6, pallidin	chr15	protein_coding
ENSG00000099822.2	-2.1878131	6.64368203	1.90E-13	2.56E-12	40.6	37.27	12.47	10.25	HCN2	hyperpolarization activated cyclic nucleotide gated potassium channel 2	chr19	protein_coding
ENSG00000213722.7	-2.414305	5.34705926	1.48E-15	2.49E-14	24.87	24.34	5.85	6.46	DDAH2	dimethylarginine dimethylaminohydrolase 2	chr6	protein_coding
ENSG00000115568.14	-1.1165331	4.50679429	0.00013529	0.00053012	3.91	4.5	3.08	2.07	ZNF142	zinc finger protein 142	chr2	protein_coding
ENSG00000077800.10	7.93471048	-0.8688155	1.08E-12	1.34E-11	0	0	0.81	0.37	FKBP6	FK506 binding protein 6, 36kDa	chr7	protein_coding
ENSG00000276223.1	1.33279015	0.21872935	0.00082366	0.00276731	0.57	1.21	2.92	3.06	RP4-781B1.5	--	chr20	sense_intronic
ENSG00000165810.15	4.57766982	-1.5864702	1.57E-07	1.02E-06	0.01	0	0.13	0.11	BTNL9	butyrophilin-like 9	chr5	protein_coding
ENSG00000168062.8	2.55650086	-0.3268546	1.52E-07	9.94E-07	0.13	0.01	0.48	0.64	BATF2	basic leucine zipper transcription factor, ATF-like 2	chr11	protein_coding
ENSG00000163931.14	-1.401811	11.4395323	1.24E-06	7.03E-06	1103.33	1046.83	561.88	521.4	TKT	transketolase	chr3	protein_coding
ENSG00000008382.14	1.26156148	4.83268929	1.60E-05	7.47E-05	9.22	7.1	25.92	26.28	MPND	MPN domain containing	chr19	protein_coding
ENSG00000254369.5	4.98694622	-0.4970812	2.91E-13	3.84E-12	0	0.01	0.2	0.23	HOXA-AS3	HOXA cluster antisense RNA 3	chr7	antisense
ENSG00000276073.1	2.66123076	0.0526436	4.46E-09	3.59E-08	0.02	0.1	0.65	0.4	RP5-1125A1	--	chr20	lincRNA
ENSG00000138767.11	2.26830167	1.36061247	1.89E-10	1.82E-09	0.11	0.04	0.5	0.5	CNOT6L	CCR4-NOT transcription complex, subunit 6-like	chr4	protein_coding
ENSG00000159164.8	-5.6420892	1.81504564	2.79E-36	1.74E-34	1.13	1.19	0.01	0.05	SV2A	synaptic vesicle glycoprotein 2A	chr1	protein_coding
ENSG00000131781.11	6.49539722	-2.0380788	3.03E-06	1.62E-05	0	0	0.08	0.15	FMO5	flavin containing monooxygenase 5	chr1	protein_coding
ENSG00000261505.1	2.01184156	-2.3104126	0.02049243	0.04852277	0.02	0	0.04	0.06	LA16c-358B7	--	chr16	antisense
ENSG00000274641.1	1.13122782	0.90513319	0.00134817	0.00432933	2.32	2.45	8.16	5.79	HIST1H2BO	histone cluster 1, H2bo	chr6	protein_coding
ENSG00000232434.2	-1.7960202	4.31537387	1.97E-09	1.66E-08	6.12	5.27	2.48	1.89	C9orf172	chromosome 9 open reading frame 172	chr9	protein_coding
ENSG00000164619.7	-1.1071776	-0.7824409	0.01671588	0.04086408	0.12	0.11	0.05	0.09	BMPER	BMP binding endothelial regulator	chr7	protein_coding
ENSG00000182308.6	5.23774779	-2.8750297	0.00290522	0.00856312	0	0	0.07	0	DCAF4L1	DDB1 and CUL4 associated factor 4-like 1	chr4	protein_coding
ENSG00000047410.12	2.09160227	4.74444141	3.78E-12	4.45E-11	0.87	0.82	3.6	6.06	TPR	translocated promoter region, nuclear basket protein	chr1	protein_coding
ENSG00000158106.11	1.98984915	2.77489364	3.25E-10	3.03E-09	0.71	0.44	3.47	2.64	RHPN1	rhophilin, Rho GTPase binding protein 1	chr8	protein_coding
ENSG00000165046.11	-5.9127857	2.4447075	1.57E-42	1.36E-40	2.44	2.53	0.02	0.09	LETM2	leucine zipper-EF-hand containing transmembrane protein 2	chr8	protein_coding
ENSG00000069329.14	1.16483417	4.64789982	6.81E-05	0.00028375	1.67	2	3.99	7.07	VPS35	vacuolar protein sorting 35 homolog (S. cerevisiae)	chr16	protein_coding
ENSG00000265688.1	-2.0525466	3.43461486	2.78E-11	2.97E-10	7.93	6.92	2.91	1.85	MAFG-AS1	MAFG antisense RNA 1 (head to head)	chr17	antisense
ENSG00000183032.9	1.19037991	-0.792245	0.01446422	0.0358903	0.06	0.05	0.15	0.21	SLC25A21	solute carrier family 25 (mitochondrial oxoadipate carrier), member 21	chr14	protein_coding
ENSG00000197536.9	-1.046754	1.90525651	0.00103092	0.00337751	0.57	0.63	0.37	0.4	C5orf56	chromosome 5 open reading frame 56	chr5	protein_coding
ENSG00000258064.1	1.50791748	0.00454038	0.00029703	0.00108329	0.24	0.77	1.95	1.87	RP11-293I14	--	chr12	protein_coding
ENSG00000153391.14	-1.049351	3.46073544	0.00043026	0.00152396	2.83	3.22	1.91	1.98	INO80C	INO80 complex subunit C	chr18	protein_coding
ENSG00000157782.8	3.36308771	-1.8188851	0.00010631	0.0004256	0.02	0	0.16	0.1	CABP1	calcium binding protein 1	chr12	protein_coding
ENSG00000159884.10	-1.806442	6.60353225	7.63E-10	6.76E-09	85.2	82.61	30.98	33	CCDC107	coiled-coil domain containing 107	chr9	protein_coding
ENSG00000267246.1	5.08251936	-2.9605404	0.00538165	0.01488851	0	0	0.31	0.03	RP11-798G7	--	chr17	processed_transcript
ENSG00000268947.1	5.89090986	-2.4715586	0.00014842	0.00057423	0	0	0.35	0.13	AD000684.2	--	chr19	sense_intronic
ENSG00000102103.14	-1.0142155	8.35858204	0.00040775	0.00144958	245.23	304.34	194.92	166.42	PQBP1	polyglutamine binding protein 1	chrX	protein_coding
ENSG00000138688.14	1.8593473	0.74058392	8.37E-07	4.88E-06	0.03	0.04	0.18	0.16	KIAA1109	KIAA1109	chr4	protein_coding
ENSG00000011523.12	1.60431828	3.03546228	1.87E-07	1.21E-06	0.43	0.39	1.68	1.65	CEP68	centrosomal protein 68kDa	chr2	protein_coding
ENSG00000170242.16	1.55375033	3.28761088	3.43E-07	2.13E-06	0.41	0.58	1.53	2.36	USP47	ubiquitin specific peptidase 47	chr11	protein_coding
ENSG00000226824.5	-3.3263588	-1.2257079	1.35E-07	8.89E-07	1.26	1	0.06	0.21	RP4-756H11	--	chr7	sense_intronic
ENSG00000102287.15	-1.015101	4.20642507	0.00053962	0.00187667	5.91	6.77	4.4	3.94	GABRE	gamma-aminobutyric acid (GABA) A receptor, epsilon	chrX	protein_coding
ENSG00000255471.1	-7.2659334	-1.6125451	1.76E-09	1.50E-08	0.4	0.37	0	0	RP11-736K2	--	chr11	antisense
ENSG00000088305.17	1.90338978	3.65295534	4.56E-10	4.17E-09	0.84	1	3.98	5.22	DNMT3B	DNA (cytosine-5-)-methyltransferase 3 beta	chr20	protein_coding
ENSG00000142677.3	1.23857414	1.96859757	0.00010994	0.00043806	0.67	0.65	2.36	1.78	IL22RA1	interleukin 22 receptor, alpha 1	chr1	protein_coding
ENSG00000134853.10	-2.5752365	-2.0277347	0.00060878	0.00209711	0.02	0.05	0	0.01	PDGFRA	platelet-derived growth factor receptor, alpha polypeptide	chr4	protein_coding
ENSG00000172935.8	-4.8106622	-1.0566907	1.01E-10	1.01E-09	0.17	0.35	0.02	0	MRGPRF	MAS-related GPR, member F	chr11	protein_coding
ENSG00000088992.16	-4.848943	3.4462331	3.35E-40	2.60E-38	17.39	15.41	0.62	0.89	TESC	tescalcin	chr12	protein_coding
ENSG00000234745.8	1.43544852	8.11231899	7.28E-07	4.28E-06	82.21	74.03	282.87	280.33	HLA-B	major histocompatibility complex, class I, B	chr6	protein_coding
ENSG00000259767.1	5.20747907	-2.8915189	0.00538165	0.01488851	0	0	0.28	0.36	RP11-90B9.2	--	chr15	sense_intronic
ENSG00000250312.5	3.21866125	-1.4852488	2.06E-05	9.41E-05	0.01	0.01	0.06	0.2	ZNF718	zinc finger protein 718	chr4	protein_coding
ENSG00000113742.11	-2.0009795	0.06816267	7.28E-07	4.28E-06	0.14	0.13	0.03	0.06	CPEB4	cytoplasmic polyadenylation element binding protein 4	chr5	protein_coding
ENSG00000164219.8	1.72400066	0.89149524	2.91E-06	1.56E-05	0.06	0.08	0.22	0.39	PGGT1B	protein geranylgeranyltransferase type I, beta subunit	chr5	protein_coding
ENSG00000162999.11	7.53722989	-1.1946656	3.23E-11	3.41E-10	0	0	0.05	0.23	DUSP19	dual specificity phosphatase 19	chr2	protein_coding

ENSG00000168542.11	-6.7478987	3.18986739	3.56E-53	6.01E-51	2.69	2.6	0.05	0.02	COL3A1	collagen, type III, alpha 1	chr2	protein_coding
ENSG00000154305.15	1.30619783	3.92227401	1.14E-05	5.45E-05	0.71	0.96	2.18	3.34	MIA3	melanoma inhibitory activity family, member 3	chr1	protein_coding
ENSG00000086666.17	1.72732592	4.32951046	7.54E-09	5.88E-08	3.32	3.15	10.79	17.93	ZFAND6	zinc finger, AN1-type domain 6	chr15	protein_coding
ENSG00000261716.1	3.34534836	1.47613418	3.06E-18	6.41E-17	0.12	0.05	0.93	1.4	RP11-196G1	--	chr1	sense_overlapping
ENSG00000137831.13	3.04996161	2.25741265	2.13E-18	4.54E-17	0.14	0.08	0.63	1.83	UACA	uveal autoantigen with coiled-coil domains and ankyrin repeats	chr15	protein_coding
ENSG00000175866.14	1.47366809	5.80680968	4.43E-07	2.70E-06	5.43	5.39	20.75	19.25	BAIAP2	BAI1-associated protein 2	chr17	protein_coding
ENSG00000095564.12	2.18894442	2.36779316	2.04E-11	2.21E-10	0.24	0.17	0.92	1.55	BTAF1	BTAF1 RNA polymerase II, B-TFIID transcription factor-associated, 170kDa	chr10	protein_coding
ENSG00000280424.1	-1.1980067	-0.1723861	0.00412997	0.01179879	0.18	0.28	0.17	0.1	CITF22-92A6	--	chr22	TEC
ENSG00000163814.6	-2.2977849	6.00104492	1.61E-14	2.45E-13	14.13	13.38	3.82	3.63	CDCP1	CUB domain containing protein 1	chr3	protein_coding
ENSG00000171219.8	2.2371179	4.88849834	1.45E-13	1.97E-12	1.26	1.66	8.61	9.71	CDC42BPG	CDC42 binding protein kinase gamma (DMPK-like)	chr11	protein_coding
ENSG00000164818.14	-1.4801604	5.54924531	4.03E-07	2.47E-06	14.9	12.02	5.88	7.02	DNAAF5	dynein, axonemal, assembly factor 5	chr7	protein_coding
ENSG00000154760.12	-3.1776452	-2.8656583	0.01615662	0.03958166	0.01	0.02	0	0	SLFN13	schlafen family member 13	chr17	protein_coding
ENSG00000253251.2	6.78232074	-1.8145695	2.24E-07	1.42E-06	0	0	0.09	0.4	CTC-534A2.2	--	chr5	protein_coding
ENSG00000169957.9	-1.1001154	7.09634802	0.00013291	0.00052188	60.43	65.78	42.5	35.71	ZNF768	zinc finger protein 768	chr16	protein_coding
ENSG00000189334.7	-6.0609014	3.20863762	1.99E-49	2.63E-47	12.69	10.43	0.15	0.3	S100A14	S100 calcium binding protein A14	chr1	protein_coding
ENSG00000186166.7	1.06118071	3.28178483	0.00040569	0.00144247	3.29	5.14	11.01	12.42	CCDC84	coiled-coil domain containing 84	chr11	protein_coding
ENSG00000126368.5	-1.3818611	4.51212784	2.75E-06	1.48E-05	9.49	9.75	5.55	4.26	NR1D1	nuclear receptor subfamily 1, group D, member 1	chr17	protein_coding
ENSG00000163507.12	2.79569914	1.84140707	2.61E-15	4.29E-14	0.13	0.21	0.74	2.42	KIAA1524	KIAA1524	chr3	protein_coding
ENSG00000048991.15	1.05644413	3.59343108	0.00038507	0.00137733	1.16	1.4	3.01	4.11	R3HDM1	R3H domain containing 1	chr2	protein_coding
ENSG00000130812.9	2.39480554	-0.5634035	2.53E-06	1.37E-05	0.08	0.07	0.59	0.52	ANGPTL6	angiopoietin-like 6	chr19	protein_coding
ENSG00000178726.6	-1.9609707	1.49540319	6.63E-09	5.23E-08	1.03	0.72	0.23	0.36	THBD	thrombomodulin	chr20	protein_coding
ENSG00000100523.13	1.97365909	1.46001159	1.59E-08	1.18E-07	0.05	0.08	0.27	0.44	DDHD1	DDHD domain containing 1	chr14	protein_coding
ENSG00000099954.17	6.04913687	-2.3630601	4.74E-05	0.00020301	0	0	0.03	0.02	CECR2	cat eye syndrome chromosome region, candidate 2	chr22	protein_coding
ENSG00000274933.3	8.40305	-0.4454779	4.25E-16	7.46E-15	0	0	0.36	0.42	TBC1D3I	TBC1 domain family, member 3I	chr17	protein_coding
ENSG00000253797.2	2.05419295	0.95708127	3.10E-08	2.21E-07	0.11	0.1	0.53	0.64	UTP14C	UTP14, U3 small nucleolar ribonucleoprotein, homolog C (yeast)	chr13	protein_coding
ENSG00000113595.13	4.3568684	-1.0295638	4.33E-09	3.50E-08	0	0.01	0.11	0.29	TRIM23	tripartite motif containing 23	chr5	protein_coding
ENSG00000007520.3	-1.0543495	8.42195891	0.00024037	0.00089725	310.51	292.42	192.97	193.95	TSR3	TSR3, 20S rRNA accumulation, homolog (S. cerevisiae)	chr16	protein_coding
ENSG00000132912.11	1.17125539	2.77335365	0.00013757	0.00053844	0.71	0.73	1.91	2.41	DCTN4	dynactin 4 (p62)	chr5	protein_coding
ENSG00000173557.13	1.25741954	-0.494791	0.00633842	0.01727626	0.7	0.07	0.84	1.67	C2orf70	chromosome 2 open reading frame 70	chr2	protein_coding
ENSG00000063127.14	1.2316299	-0.5102789	0.00764142	0.02043737	0.14	0.05	0.27	0.33	SLC6A16	solute carrier family 6, member 16	chr19	protein_coding
ENSG00000237232.6	3.35527459	-1.8234775	0.00010631	0.0004256	0.06	0	0.47	0.4	ZNF295-AS1	ZNF295 antisense RNA 1	chr21	lincRNA
ENSG00000149150.7	-3.1004161	2.99143865	1.21E-20	3.06E-19	4.7	3.82	0.91	0.41	SLC43A1	solute carrier family 43 (amino acid system L transporter), member 1	chr11	protein_coding
ENSG00000087301.7	2.51602894	0.78196473	2.36E-10	2.24E-09	0.04	0.14	0.38	0.92	TXNDC16	thioredoxin domain containing 16	chr14	protein_coding
ENSG00000266338.5	-1.186184	3.72294615	6.39E-05	0.0002678	2.77	3.19	1.44	2.06	NBPF15	neuroblastoma breakpoint family, member 15	chr1	protein_coding
ENSG00000278175.2	1.44220104	2.83236829	2.97E-06	1.60E-05	1.05	1.31	4.41	4.16	GLIDR	glioblastoma down-regulated RNA	chr9	lincRNA
ENSG00000096717.10	3.19856639	2.41460209	5.53E-20	1.33E-18	0.16	0.2	2.01	2.45	SIRT1	sirtuin 1	chr10	protein_coding
ENSG00000100485.10	2.37099309	1.31237767	7.28E-11	7.43E-10	0.07	0.16	0.59	0.98	SOS2	son of sevenless homolog 2 (Drosophila)	chr14	protein_coding
ENSG00000101204.14	-4.7309009	-1.8419496	1.01E-06	5.80E-06	0.07	0.06	0.01	0	CHRNA4	cholinergic receptor, nicotinic, alpha 4 (neuronal)	chr20	protein_coding
ENSG00000145388.13	2.01112896	1.27630241	1.80E-08	1.33E-07	0.08	0.14	0.4	0.8	METTL14	methyltransferase like 14	chr4	protein_coding
ENSG00000149596.6	-3.1242528	4.44657582	5.39E-23	1.61E-21	5.33	5.34	0.68	0.95	JPH2	junctophilin 2	chr20	protein_coding
ENSG00000206527.8	3.47781466	4.1265482	1.98E-26	7.53E-25	0.36	0.73	6.39	9.82	PTPLB	--	chr3	protein_coding
ENSG00000278709.1	-1.160054	1.35494167	0.00046641	0.0016411	1.37	0.8	0.52	0.78	RP5-1059L7.	--	chr20	antisense
ENSG00000102854.13	-1.2468393	7.61826671	1.55E-05	7.28E-05	95.97	80.87	51.53	47.73	MSLN	mesothelin	chr16	protein_coding
ENSG00000039560.12	1.10694114	3.45780637	0.00021802	0.0008188	0.74	1.14	2.06	3.35	RAI14	retinoic acid induced 14	chr5	protein_coding
ENSG00000164683.15	-3.032897	3.26953211	1.18E-20	2.98E-19	4.9	5.91	0.8	0.96	HEY1	hes-related family bHLH transcription factor with YRPW motif 1	chr8	protein_coding
ENSG00000272572.1	1.38618334	-0.4633632	0.00249754	0.00752149	0.2	0.16	0.45	0.78	RP11-179B2.	--	chr10	antisense
ENSG00000188910.7	-2.2589387	5.3011219	6.06E-14	8.66E-13	23.47	20.74	5.89	6.43	GJB3	gap junction protein, beta 3, 31kDa	chr1	protein_coding
ENSG00000103154.8	-2.5924101	0.54842342	1.75E-10	1.69E-09	1.29	0.43	0.32	0.07	NECAB2	N-terminal EF-hand calcium binding protein 2	chr16	protein_coding
ENSG00000266680.1	7.91281115	-0.8792445	2.26E-12	2.73E-11	0	0	0.62	0.78	RP5-1148A2	--	chr6	antisense
ENSG00000231856.2	1.56098716	0.3358955	7.98E-05	0.00032858	0.23	0.16	0.7	0.84	RP11-327P2.	--	chr13	antisense
ENSG00000141905.16	1.17493593	8.33640387	4.48E-05	0.00019402	17.89	21.38	63.54	54.24	NFIC	nuclear factor I/C (CCAAT-binding transcription factor)	chr19	protein_coding
ENSG00000253671.1	6.54003613	-2.0117813	1.05E-06	6.02E-06	0	0	0.91	0.24	RP11-806O1	--	chr8	processed_transcript
ENSG00000080546.12	2.38429967	0.6104516	2.53E-09	2.10E-08	0.17	0.01	0.43	0.88	SESN1	sestrin 1	chr6	protein_coding
ENSG00000134905.15	-1.1920791	7.21264122	3.57E-05	0.00015694	88.36	89.44	51.13	52.58	CARS2	cysteinyl-tRNA synthetase 2, mitochondrial (putative)	chr13	protein_coding
ENSG00000130413.14	-6.5745401	-2.1618028	1.78E-06	9.85E-06	0.1	0.05	0	0	STK33	serine/threonine kinase 33	chr11	protein_coding
ENSG00000111252.9	-1.7672004	3.09753724	9.28E-09	7.14E-08	1.77	2.08	0.7	0.8	SH2B3	SH2B adaptor protein 3	chr12	protein_coding
ENSG00000185065.6	-1.4948566	0.52368078	5.24E-05	0.00022267	0.82	0.78	0.35	0.4	AC000068.5	--	chr22	antisense
ENSG00000126351.11	-1.3648965	7.13698246	2.44E-06	1.33E-05	26.68	26.3	13.52	13.9	THRA	thyroid hormone receptor, alpha	chr17	protein_coding
ENSG00000165661.14	-1.3332737	7.28703417	4.05E-06	2.12E-05	41.29	38.6	19.81	22.51	QSOX2	quiescin Q6 sulfhydryl oxidase 2	chr9	protein_coding
ENSG00000188293.5	-7.9592424	-1.0080987	1.13E-13	1.56E-12	0.8	1.18	0	0	IGFL1	IGF-like family member 1	chr19	protein_coding
ENSG00000099250.16	-2.5301791	5.50614115	7.78E-17	1.45E-15	5.1	6.51	1.22	1.46	NRP1	neuropilin 1	chr10	protein_coding
ENSG00000166821.7	2.00918419	4.10582409	3.71E-11	3.90E-10	2	1.71	8.99	10.99	PEX11A	peroxisomal biogenesis factor 11 alpha	chr15	protein_coding
ENSG00000278041.1	-6.192739	-2.4379876	4.74E-05	0.00020301	0.03	0.09	0	0	RP5-984P4.6	--	chr20	lincRNA
ENSG00000140043.10	1.40702428	1.42134395	3.49E-05	0.00015372	0.22	0.35	0.71	1.31	PTGR2	prostaglandin reductase 2	chr14	protein_coding
ENSG00000080573.6	-2.067388	2.98275974	3.90E-11	4.09E-10	1.81	1.53	0.52	0.54	COL5A3	collagen, type V, alpha 3	chr19	protein_coding
ENSG00000105696.7	2.61742468	2.16037877	1.34E-14	2.05E-13	0.75	0.4	4.89	4.54	TMEM59L	transmembrane protein 59-like	chr19	protein_coding
ENSG00000105176.16	1.10713851	3.97756983	0.00018012	0.00068558	1.99	2.54	5.14	7.91	URI1	URI1, prefoldin-like chaperone	chr19	protein_coding
ENSG00000137770.12	1.91781469	1.43181417	3.01E-08	2.16E-07	0.15	0.1	0.49	0.79	CTDSPL2	CTD (carboxy-terminal domain, RNA polymerase II, polypeptide A) small phosphatase like 2	chr15	protein_coding
ENSG00000129534.12	4.38947376	0.54182841	1.37E-18	2.96E-17	0.02	0.02	0.37	0.78	MIS18BP1	MIS18 binding protein 1	chr14	protein_coding
ENSG00000229152.2	5.05694572	-2.9744029	0.0100455	0.02582742	0	0	0.12	0.2	ANKRD10-IT	ANKRD10 intronic transcript 1	chr13	sense_intronic
ENSG00000076053.9	2.46366218	1.53052229	4.84E-12	5.63E-11	0.09	0.1	0.47	0.88	RBM7	RNA binding motif protein 7	chr11	protein_coding
ENSG00000138138.12	1.99222585	2.99622576	2.26E-10	2.15E-09	0.36	0.68	2.04	3.52	ATAD1	ATPase family, AAA domain containing 1	chr10	protein_coding
ENSG00000117000.8	3.38409276	0.66166139	8.89E-15	1.39E-13	0.05	0.01	0.28	0.65	RLF	rearranged L-myc fusion	chr1	protein_coding
ENSG00000053900.9	2.36092324	1.72775074	1.23E-11	1.37E-10	0.19	0.44	1.66	2.66	ANAPC4	anaphase promoting complex subunit 4	chr4	protein_coding
ENSG00000233396.6	2.01280532	-2.018611	0.01397446	0.03477946	0	0.06	0.16	0.15	RP11-458D2	--	chr1	lincRNA
ENSG00000116106.10	6.22093654	-2.2453676	1.55E-05	7.26E-05	0	0	0.08	0.02	EPHA4	EPH receptor A4	chr2	protein_coding
ENSG00000198839.8	3.6139676	0.32388008	2.01E-14	3.01E-13	0.02	0.05	0.48	0.67	ZNF277	zinc finger protein 277	chr7	protein_coding
ENSG00000129128.11	1.42229046	3.339959	2.57E-06	1.39E-05	1.05	0.88	2.73	4.19	SPCS3	signal peptidase complex subunit 3 homolog (S. cerevisiae)	chr4	protein_coding
ENSG00000158555.13	-1.3700281	3.02603396	6.82E-06	3.42E-05	1.64	1.38	0.8	0.75	GDPD5	glycerophosphodiester phosphodiesterase domain containing 5	chr11	protein_coding
ENSG00000115825.8	2.37730634	2.37615926	6.91E-13	8.81E-12	0.16	0.28	1.33	1.68	PRKD3	protein kinase D3	chr2	protein_coding
ENSG00000235931.2	3.11584787	-2.6043119	0.00552196	0.01522223	0	0.01	0.04	0.05	LINC01553	long intergenic non-protein coding RNA 1553	chr10	lincRNA
ENSG00000102317.16	-1.8471063	7.5082981	3.07E-10	2.88E-09	76.04	70.46	22.61	31.9	RBM3	RNA binding motif (RNP1, RRM) protein 3	chrX	protein_coding
ENSG00000137142.4	1.09706413	0.70143153	0.00242831	0.00732956	0.91	0.54	1.85	2.32	IGFBPL1	insulin-like growth factor binding protein-like 1	chr9	protein_coding
ENSG00000259725.1	5.46459707	-2.7413886	0.00158008	0.00495521	0	0	0.29	0.34	CTD-3032H1	--	chr16	lincRNA
ENSG00000259345.4	-2.3465442	-1.361029	0.00025593	0.00094973	0.31	0.37	0.19	0	RP11-624L4.	--	chr15	lincRNA
ENSG00000102189.15	4.23692355	1.12503599	1.69E-21	4.61E-20	0.02	0.02	0.23	0.63	EEA1	early endosome antigen 1	chr12	protein_coding
ENSG00000071246.9	1.96989118	3.50990313	1.46E-10	1.43E-09	0.61	0.6	3.28	3	VASH1	vasohibin 1	chr14	protein_coding
ENSG00000204977.8	1.23114464	2.50440263	7.99E-05	0.0003288	0.34	0.39	1.09	1.2	TRIM13	tripartite motif containing 13	chr13	protein_coding
ENSG00000142694.6	-2.2885556	2.74444921	8.54E-13	1.08E-11	10.53	6.48	2.74	1.91	EVA1B	eva-1 homolog B (C. elegans)	chr1	protein_coding
ENSG00000232973.10	2.64776459	1.05876404	3.01E-12	3.59E-11	0.09	0.02	0.37	0.62	CYP1B1-AS1	CYP1B1 antisense RNA 1	chr2	antisense
ENSG00000165140.8	5.77385495	-2.5463609	0.00026535	0.0009747	0	0	0.1	0.16	FBP1	fructose-1,6-bisphosphatase 1	chr9	protein_coding
ENSG00000127084.16	-2.3439092	-1.2674728	3.89E-05	0.00016973	0.15	0.11	0	0.06	FGD3	FYVE, RhoGEF and PH domain containing 3	chr9	protein_coding
ENSG00000178691.9	2.78556853	2.61958796	6.56E-17	1.23E-15	0.36	0.19	2.02	3.06	SUZ12	SUZ12 polycomb repressive complex 2 subunit	chr17	protein_coding
ENSG00000130940.13	1.3723911	2.64378727	9.76E-06	4.74E-05	0.24	0.42	1.3	0.94	CASZ1	castor zinc finger 1	chr1	protein_coding
ENSG00000275055.1	-1.2868875	-0.7777687	0.00624182	0.01703231	0.93	0.72	0.34	0.55	CTC-471J1.1	--	chr19	antisense
ENSG00000028116.15	1.57429761	1.17888924	6.32E-06	3.18E-05	0.27	0.37	0.94	1.6	VRK2	vaccinia related kinase 2	chr2	protein_coding
ENSG00000275740.1	2.10843826	2.15493049	1.98E-10	1.90E-09	0.35	0.45	2.01	2.6	RP11-449H3	--	chr5	protein_coding
ENSG00000136153.18	-1.5881667	4.35458512	8.63E-08	5.83E-07	2.52	3.07	1.04	1.44	LMO7	LIM domain 7	chr13	protein_coding
ENSG00000103145.9	-1.8433742	6.25855799	3.77E-10	3.50E-09	95.65	90.18	36.98	31.91	HCFC1R1	host cell factor C1 regulator 1 (XPO1 dependent)	chr16	protein_coding
ENSG00000187498.13	-12.512783	6.50692787	5.58E-113	1.49E-109	17.3	22.35	0.01	0	COL4A1	collagen, type IV, alpha 1	chr13	protein_coding
ENSG00000102230.12	-2.820307	-1.8451881	0.00010631	0.0004256	0.03	0.09	0.02	0	PCYT1B	phosphate cytidylyltransferase 1, choline, beta	chrX	protein_coding
ENSG00000270933.1	4.78582907	-1.4180336	1.28E-07	8.50E-07	0.04	0	0.73	0.91	CTD-2227E1	--	chr7	lincRNA
ENSG00000106290.13	-1.3056902	7.63345128	6.23E-06	3.14E-05	69.61	72.36	41.47	34.86	TAF6	TAF6 RNA polymerase II, TATA box binding protein (TBP)-associated factor, 80kDa	chr7	protein_coding
ENSG00000115380.17	-1.9513706	6.13912549	3.96E-11	4.14E-10	26.17	33.06	9.12	11.31	EFEMP1	EGF containing fibulin-like extracellular matrix protein 1	chr2	protein_coding
ENSG00000067182.6	-1.0335059	8.02095786	0.00031857	0.00115482	95.85	93.94	65.59	57.73	TNFRSF1A	tumor necrosis factor receptor superfamily, member 1A	chr12	protein_coding
ENSG00000258811.1	-5.0206302	-3.1306986	0.0100455	0.02582742	0.04	0.02	0	0	CTD-3051D2	--	chr14	lincRNA
ENSG00000228526.2	4.89980841	-3.0567113	0.01889873	0.04519557	0	0	0.28	0.24	RP3-510D11	--	chr1	lincRNA
ENSG00000227191.5	4.87917439	-3.0678699	0.01889873	0.04519557	0	0	0	0.09	TRGC2	T cell receptor gamma constant 2	chr7	TR_C_gene
ENSG00000230061.2	-6.4339616	-2.266126	5.19E-06	2.66E-05	0.12	0.11	0	0	TRPM2-AS	TRPM2 antisense RNA	chr21	antisense
ENSG00000136874.9	1.74940773	1.06618716	1.06E-06	6.06E-06	0.27	0.17	0.95	1.01	STX17	syntaxin 17	chr9	protein_coding
ENSG00000103994.15	1.18597117	4.27734902	5.69E-05	0.00023998	0.86	0.91	2.42	2.99	ZNF106	zinc finger protein 106	chr15	protein_coding
ENSG00000135486.16	-1.1174587	10.426779	0.0001	0.0004027	347.17	365.79	196.11	242.83	HNRNPA1	heterogeneous nuclear ribonucleoprotein A1	chr12	protein_coding
ENSG00000180902.15	1.09048117	3.37735825	0.00027896	0.00102157	1.59	1.33	4.46	3.83	D2HGDH	D-2-hydroxyglutarate dehydrogenase	chr2	protein_coding
ENSG00000167815.10	-1.2954391	9.43290303	7.10E-06	3.54E-05	873.09	736.89	451.86	421.91	PRDX2	peroxiredoxin 2	chr19	protein_coding
ENSG00000133316.14	-1.3528053	7.34074561	2.96E-06	1.59E-05	97.44	87.37	52.46	43.76	WDR74	WD repeat domain 74	chr11	protein_coding
ENSG00000139926.14	1.61196139	1.27389001	3.64E-06	1.92E-05	0.12	0.23	0.46	0.97	FRMD6	FERM domain containing 6	chr14	protein_coding
ENSG00000101605.11	-4.5222885	0.7345662	7.39E-23	2.18E-21	0.4	0.48	0.01	0.04	MYOM1	myomesin 1	chr18	protein_coding
ENSG00000251257.2	-6.4354995	-2.265336	5.19E-06	2.66E-05	0.29	0.43	0	0	CTD-2263F2	--	chr5	antisense
ENSG00000168028.12	-1.8185868	9.98941063	5.17E-10	4.70E-09	1220.08	939.74	400.62	416.6	RPSA	ribosomal protein SA	chr3	protein_coding
ENSG00000011052.20	-1.0147754	9.0622184	0.00040204	0.00143131	520.56	411.48	304.81	310.59	NME2	NME/NM23 nucleoside diphosphate kinase 2	chr17	protein_coding
ENSG00000182240.14	-1.8941896	5.29625628	1.60E-10	1.56E-09	5.55	5.7	1.98	2.06	BACE2	beta-site APP-cleaving enzyme 2	chr21	protein_coding
ENSG00000115084.11	1.65049736	2.03746757	4.09E-07	2.51E-06	0.32	0.22	0.74	1.56	SLC35F5	solute carrier family 35, member F5	chr2	protein_coding
ENSG00000261613.2	-1.7539402	0.89815317	9.43E-07	5.45E-06	1.26	1.03	0.38	0.52	RP11-20I23.1	--	chr16	antisense
ENSG00000118160.12	7.03989843	3.23395988	1.25E-53	2.16E-51	0.01	0.03	3.91	3.67	SLC8A2	solute carrier family 8 (sodium/calcium exchanger), member 2	chr19	protein_coding
ENSG00000277837.1	-7.4230262	-1.4789356	2.87E-10	2.70E-09	0.13	0.08	0	0	RP11-714M2	--	chr18	lincRNA
ENSG00000111912.17	1.55813454	2.00012204	1.70E-06	9.45E-06	0.19	0.28	0.65	1.23	NCOA7	nuclear receptor coactivator 7	chr6	protein_coding
ENSG00000172469.13	7.28555401	-1.4088394	7.05E-10	6.26E-09	0	0	0.06	0.19	MANEA	mannosidase, endo-alpha	chr6	protein_coding
ENSG00000249685.1	-2.5406454	-2.3292898	0.00281991	0.00840887	0.29	0.62	0.07	0.12	RP11-360F5.	--	chr4	lincRNA
ENSG00000085224.19	4.40405016	3.42670625	7.94E-35	4.60E-33	0.05	0.08	1.26	2.67	ATRX	alpha thalassemia/mental retardation syndrome X-linked	chrX	protein_coding
ENSG00000237441.8	-1.6172266	3.98180815	6.28E-08	4.34E-07	7.11	4.62	2.44	2.67	RGL2	ral guanine nucleotide dissociation stimulator-like 2	chr6	protein_coding
ENSG00000224023.9	2.31896233	-0.6265073	5.62E-06	2.85E-05	0.08	0.03	0.53	0.26	FLJ37035	--	chr10	lincRNA
ENSG00000280163.1	3.2200002	-0.6703752	1.09E-08	8.28E-08	0.06	0.03	0.63	0.63	CTC-277H1.6	--	chr16	TEC
ENSG00000250838.1	-3.0899481	-1.6580144	8.20E-06	4.05E-05	1.12	0.73	0.15	0.13	RP11-501C1	--	chr17	antisense
ENSG00000267481.1	6.39229942	-2.1171927	5.19E-06	2.66E-05	0	0	0.38	0.35	CTC-559E9.5	--	chr19	sense_intronic
ENSG00000096433.9	-1.8337157	9.16530496	3.84E-10	3.56E-09	74.18	72.93	28.7	26.22	ITPR3	inositol 1,4,5-trisphosphate receptor, type 3	chr6	protein_coding
ENSG00000145147.18	-3.1832186	-0.5750827	1.79E-09	1.51E-08	0.15	0.13	0.03	0.01	SLIT2	slit homolog 2 (Drosophila)	chr4	protein_coding
ENSG00000189420.8	-3.3248997	-2.2204227	8.40E-05	0.0003415	0.05	0.03	0	0.01	ZFP92	ZFP92 zinc finger protein	chrX	protein_coding
ENSG00000082438.14	3.49755974	1.88335726	6.69E-21	1.71E-19	0.06	0.04	0.51	0.95	COBLL1	cordon-bleu WH2 repeat protein-like 1	chr2	protein_coding
ENSG00000196072.10	1.47646122	2.14944664	3.80E-06	2.00E-05	0.67	0.62	1.9	2.93	BLOC1S2	biogenesis of lysosomal organelles complex-1, subunit 2	chr10	protein_coding
ENSG00000142875.18	2.15456725	-0.6295402	1.94E-05	8.92E-05	0.03	0.02	0.06	0.25	PRKACB	protein kinase, cAMP-dependent, catalytic, beta	chr1	protein_coding
ENSG00000169359.12	1.25250251	3.07724599	3.63E-05	0.00015939	0.41	0.46	1.09	1.66	SLC33A1	solute carrier family 33 (acetyl-CoA transporter), member 1	chr3	protein_coding
ENSG00000152234.14	-1.156143	8.64746046	5.84E-05	0.00024622	72.24	71.15	39.39	46.52	ATP5A1	ATP synthase, H+ transporting, mitochondrial F1 complex, alpha subunit 1, cardiac muscle	chr18	protein_coding
ENSG00000261033.1	5.07251188	-0.4230901	6.15E-14	8.78E-13	0	0.05	1.16	1.15	RP11-209D1	--	chr17	antisense
ENSG00000234779.1	-5.1829701	-1.4780234	6.93E-09	5.44E-08	1.12	1.26	0.07	0	RP11-62F24.	--	chr9	antisense
ENSG00000143756.10	1.67308584	1.64312842	6.89E-07	4.07E-06	0.24	0.19	0.65	1.18	FBXO28	F-box protein 28	chr1	protein_coding
ENSG00000112893.8	1.06997649	0.14779572	0.00698117	0.0188439	0.1	0.13	0.3	0.34	MAN2A1	mannosidase, alpha, class 2A, member 1	chr5	protein_coding
ENSG00000261474.1	-1.3012424	-1.0648562	0.01161423	0.02949325	0.89	1.33	0.71	0.49	RP11-452L6.	--	chr16	lincRNA
ENSG00000174808.10	9.33431308	0.41775365	1.09E-24	3.71E-23	0	0	0.41	1.46	BTC	betacellulin	chr4	protein_coding
ENSG00000242366.3	5.32555564	5.91689467	3.68E-52	5.77E-50	0.84	1.13	47.32	58.39	UGT1A8	UDP glucuronosyltransferase 1 family, polypeptide A8	chr2	protein_coding
ENSG00000254703.2	-5.0206302	-3.1306986	0.0100455	0.02582742	0.1	0.04	0	0	SENCR	smooth muscle and endothelial cell enriched migration/differentiation-associated long non-coding RNA	chr11	antisense
ENSG00000258920.1	-1.2217924	0.81802713	0.00052442	0.0018267	1.61	2.03	1.01	1.06	FOXN3-AS1	FOXN3 antisense RNA 1	chr14	antisense
ENSG00000184916.7	-1.4173422	6.58026368	1.06E-06	6.04E-06	20.88	17.72	9.49	9.79	JAG2	jagged 2	chr14	protein_coding
ENSG00000125845.6	4.31826306	6.60528381	2.07E-39	1.54E-37	2.1	2.07	44.82	66.7	BMP2	bone morphogenetic protein 2	chr20	protein_coding
ENSG00000258964.1	3.59223995	0.01353244	1.52E-12	1.88E-11	0.26	0.04	1.2	3.82	RP11-618G2	--	chr14	processed_transcript
ENSG00000180628.13	1.26012719	0.85804665	0.00038714	0.0013839	0.15	0.08	0.31	0.41	PCGF5	polycomb group ring finger 5	chr10	protein_coding
ENSG00000249082.2	-7.5334855	2.04207272	6.40E-46	6.97E-44	10.34	5.38	0	0.1	C5orf66-AS1	C5orf66 antisense RNA 1	chr5	lincRNA
ENSG00000178150.7	2.92220084	1.41296537	5.74E-15	9.15E-14	0.29	0.06	1.45	2.17	ZNF114	zinc finger protein 114	chr19	protein_coding
ENSG00000151422.11	2.76783769	1.02982481	1.24E-12	1.54E-11	0.03	0.03	0.27	0.32	FER	fer (fps/fes related) tyrosine kinase	chr5	protein_coding
ENSG00000175087.8	2.80350588	-0.9976458	1.86E-06	1.03E-05	0	0.04	0.18	0.17	PDIK1L	PDLIM1 interacting kinase 1 like	chr1	protein_coding
ENSG00000136261.13	-1.5004895	5.47732929	2.86E-07	1.79E-06	25.92	28.14	11.84	13.64	BZW2	basic leucine zipper and W2 domains 2	chr7	protein_coding
ENSG00000272459.1	1.68818488	-0.250976	0.00012743	0.000502	0.4	0.68	2.37	2.27	RP11-1277A	--	chr5	lincRNA
ENSG00000090097.19	-1.9934921	5.30502112	1.98E-11	2.14E-10	25.12	19.05	7.8	6.98	PCBP4	poly(rC) binding protein 4	chr3	protein_coding
ENSG00000268649.3	-6.0005178	-2.5673179	0.00014842	0.00057423	0.27	0.38	0	0	RP4-806M20	--	chr20	lincRNA
ENSG00000180336.16	9.31481373	0.39423558	1.69E-24	5.60E-23	0	0	0.29	0.5	C17orf104	chromosome 17 open reading frame 104	chr17	protein_coding
ENSG00000136026.12	-1.2322999	7.82770642	1.93E-05	8.87E-05	80.34	86.56	47.13	47.48	CKAP4	cytoskeleton-associated protein 4	chr12	protein_coding
ENSG00000196083.8	2.59113675	0.31497504	1.22E-09	1.05E-08	0.03	0.03	0.1	0.32	IL1RAP	interleukin 1 receptor accessory protein	chr3	protein_coding
ENSG00000102387.14	4.92101162	3.77934818	1.91E-41	1.57E-39	0.31	0.21	9.52	11.45	TAF7L	TAF7-like RNA polymerase II, TATA box binding protein (TBP)-associated factor, 50kDa	chrX	protein_coding
ENSG00000235387.1	-3.0631392	-0.8523028	1.54E-08	1.15E-07	0.6	0.33	0.04	0.1	LINC00961	long intergenic non-protein coding RNA 961	chr9	lincRNA
ENSG00000146374.12	7.75138439	-1.0187299	1.18E-12	1.47E-11	0	0	0.24	0.33	RSPO3	R-spondin 3	chr6	protein_coding
ENSG00000078399.14	4.50627501	3.23901098	5.10E-35	2.97E-33	0.34	0.28	8.07	10.67	HOXA9	homeobox A9	chr7	protein_coding
ENSG00000255690.2	2.428268	-1.7462306	0.00095666	0.0031536	0.01	0.01	0.09	0.08	TRIL	TLR4 interactor with leucine-rich repeats	chr7	protein_coding
ENSG00000118007.11	1.61043572	2.04718532	7.35E-07	4.32E-06	0.24	0.25	0.88	1.12	STAG1	stromal antigen 1	chr3	protein_coding
ENSG00000170122.5	6.94637422	-1.6987039	3.05E-08	2.18E-07	0	0	0.42	0.1	FOXD4	forkhead box D4	chr9	protein_coding
ENSG00000176907.4	-7.0863688	1.99659249	1.04E-42	9.24E-41	3.32	3.62	0.05	0.01	C8orf4	chromosome 8 open reading frame 4	chr8	protein_coding
ENSG00000273973.1	-1.8038699	-1.9009144	0.00967167	0.02519005	0.33	0.66	0.11	0.24	RP11-973D8	--	chr12	sense_intronic
ENSG00000021574.10	3.0126144	-0.1826569	1.03E-09	9.00E-09	0.02	0.04	0.16	0.42	SPAST	spastin	chr2	protein_coding
ENSG00000138071.12	1.25840235	4.15515766	2.10E-05	9.58E-05	2.12	1.96	4.42	8.69	ACTR2	ARP2 actin-related protein 2 homolog (yeast)	chr2	protein_coding
ENSG00000243426.3	-6.5067594	-2.2126453	3.03E-06	1.62E-05	1.21	1.05	0	0	RN7SL454P	RNA, 7SL, cytoplasmic 454, pseudogene	chr17	misc_RNA
ENSG00000006555.9	3.9577283	-2.0551732	3.06E-05	0.00013563	0	0.01	0.12	0.04	TTC22	tetratricopeptide repeat domain 22	chr1	protein_coding
ENSG00000116014.8	2.86451136	-0.9568902	9.58E-07	5.53E-06	0.1	0	0.53	0.49	KISS1R	KISS1 receptor	chr19	protein_coding
ENSG00000099910.15	1.19408497	3.99112152	5.38E-05	0.00022811	3.63	2.31	9.17	8.97	KLHL22	kelch-like family member 22	chr22	protein_coding

ENSG00000072506.11	-1.1029272	7.61427905	0.00012591	0.00049644	243.16	207.44	136.34	143.56	HSD17B10	hydroxysteroid (17-beta) dehydrogenase 10	chrX	protein_coding
ENSG00000210156.1	1.66962897	0.21340707	3.20E-05	0.00014151	2.29	9.72	28.37	22.17	MT-TK	mitochondrially encoded tRNA lysine	chrM	Mt_tRNA
ENSG00000109586.10	-4.4246784	-0.0168852	1.33E-16	2.43E-15	0.31	0.33	0.04	0	GALNT7	polypeptide N-acetylgalactosaminyltransferase 7	chr4	protein_coding
ENSG00000152056.15	1.51767047	-1.087237	0.00604922	0.01656143	0.12	0.02	0.18	0.36	AP1S3	adaptor-related protein complex 1, sigma 3 subunit	chr2	protein_coding
ENSG00000148331.10	-1.0447418	6.79175663	0.00028294	0.00103504	26.85	25.41	16.88	16.87	ASB6	ankyrin repeat and SOCS box containing 6	chr9	protein_coding
ENSG00000151849.13	2.2621733	2.2363433	8.07E-12	9.14E-11	0.19	0.3	1.3	1.84	CENPJ	centromere protein J	chr13	protein_coding
ENSG00000184939.14	3.75220114	2.19216907	6.18E-24	1.97E-22	0.11	0.09	1.76	1.85	ZFP90	ZFP90 zinc finger protein	chr16	protein_coding
ENSG00000110367.10	2.25413388	4.41353087	1.41E-13	1.93E-12	1.05	0.83	5.57	6.42	DDX6	DEAD (Asp-Glu-Ala-Asp) box helicase 6	chr11	protein_coding
ENSG00000144909.7	2.59235444	1.13830305	5.71E-12	6.61E-11	0.16	0.07	0.64	1.22	OSBPL11	oxysterol binding protein-like 11	chr3	protein_coding
ENSG00000187800.12	-4.8413698	2.07923767	5.44E-33	2.91E-31	1.35	1.31	0.03	0.09	PEAR1	platelet endothelial aggregation receptor 1	chr1	protein_coding
ENSG00000108256.7	2.56572954	3.69953906	3.30E-16	5.83E-15	0.22	0.34	2.02	2.37	NUFIP2	nuclear fragile X mental retardation protein interacting protein 2	chr17	protein_coding
ENSG00000125726.9	3.85687202	7.34545532	1.16E-33	6.38E-32	15.62	12.45	284.93	256.8	CD70	CD70 molecule	chr19	protein_coding
ENSG00000235652.6	1.9703601	-2.0424807	0.02035181	0.04824221	0	0.02	0	0.09	RP11-545I5.3	--	chr6	antisense
ENSG00000163328.12	2.2453539	-0.5646737	6.87E-06	3.45E-05	0.03	0.01	0.11	0.17	GPR155	G protein-coupled receptor 155	chr2	protein_coding
ENSG00000273171.1	6.28873448	2.10495369	5.19E-39	3.77E-37	0.16	0.03	11.67	9.22	CTB-96E2.2	--	chr17	protein_coding
ENSG00000266010.1	-2.320176	-0.8921903	6.55E-06	3.29E-05	0.27	0.49	0.13	0.08	GATA6-AS1	GATA6 antisense RNA 1 (head to head)	chr18	lincRNA
ENSG00000089505.16	2.0420823	-1.0791503	0.00036242	0.00130273	0.09	0.13	0.9	0.34	CMTM1	CKLF-like MARVEL transmembrane domain containing 1	chr16	protein_coding
ENSG00000204231.9	-1.3774855	5.58213929	2.26E-06	1.23E-05	20.69	17.62	9.85	9.81	RXRB	retinoid X receptor, beta	chr6	protein_coding
ENSG00000261094.2	3.61348816	-1.6265084	1.25E-05	5.96E-05	0	0.03	0.19	0.3	RP11-355O1	--	chr9	sense_overlapping
ENSG00000106089.10	-2.2914163	4.9388948	3.42E-14	5.00E-13	18.21	17.6	4.46	5.3	STX1A	syntaxin 1A (brain)	chr7	protein_coding
ENSG00000244041.6	-1.5080752	-0.3877129	0.00051464	0.00179394	0.21	0.41	0.08	0.2	LINC01011	long intergenic non-protein coding RNA 1011	chr6	lincRNA
ENSG00000095209.10	3.05398206	1.9413306	1.72E-17	3.40E-16	0.19	0.12	1.26	2.18	TMEM38B	transmembrane protein 38B	chr9	protein_coding
ENSG00000125633.9	1.15633097	3.60821414	0.00010713	0.00042851	0.76	1.01	2.53	2.7	CCDC93	coiled-coil domain containing 93	chr2	protein_coding
ENSG00000198682.11	4.17582305	4.65661763	1.78E-35	1.07E-33	0.64	0.43	10.71	15.29	PAPSS2	3'-phosphoadenosine 5'-phosphosulfate synthase 2	chr10	protein_coding
ENSG00000186364.10	-1.5398824	1.63592585	3.00E-06	1.61E-05	2.61	2.42	1.09	1.22	NUDT17	nudix (nucleoside diphosphate linked moiety X)-type motif 17	chr1	protein_coding
ENSG00000142235.7	-1.75854	3.3434981	8.45E-09	6.55E-08	2.39	2.51	1.02	0.91	LMTK3	lemur tyrosine kinase 3	chr19	protein_coding
ENSG00000153207.13	1.90461429	3.37554574	6.27E-10	5.62E-09	0.34	0.45	1.38	2.61	AHCTF1	AT hook containing transcription factor 1	chr1	protein_coding
ENSG00000118985.13	1.37117126	3.52471913	5.10E-06	2.62E-05	0.81	0.89	2.24	3.67	ELL2	elongation factor, RNA polymerase II, 2	chr5	protein_coding
ENSG00000092929.10	-1.1009835	6.75120338	0.00013287	0.0005218	28.72	25.4	17.06	16.56	UNC13D	unc-13 homolog D (C. elegans)	chr17	protein_coding
ENSG00000148468.15	-1.3309156	4.29987801	6.26E-06	3.16E-05	5.64	5.9	2.67	3.46	FAM171A1	family with sequence similarity 171, member A1	chr10	protein_coding
ENSG00000163545.7	-1.904412	2.83419393	1.04E-09	9.04E-09	2.23	3.12	0.94	0.96	NUAK2	NUAK family, SNF1-like kinase, 2	chr1	protein_coding
ENSG00000275835.3	1.0868145	1.6118877	0.00093818	0.0031025	0.41	0.33	0.84	1.28	TUBGCP5	tubulin, gamma complex associated protein 5	chr15	protein_coding
ENSG00000116667.11	-1.1065667	4.60804148	0.0001485	0.00057446	2.23	3.03	1.42	1.83	C1orf21	chromosome 1 open reading frame 21	chr1	protein_coding
ENSG00000021488.11	5.95655261	-2.4251622	0.00014842	0.00057423	0	0	0.13	0.15	SLC7A9	solute carrier family 7 (amino acid transporter light chain, bo,+ system), member 9	chr19	protein_coding
ENSG00000263531.1	5.56116133	-2.6812008	0.00086564	0.00288128	0	0	0.08	0.36	RP13-753N3	--	chr17	antisense
ENSG00000145506.12	-2.2974768	4.37184133	5.09E-14	7.33E-13	11.33	12.54	3.53	2.93	NKD2	naked cuticle homolog 2 (Drosophila)	chr5	protein_coding
ENSG00000225335.3	2.60107755	-0.0800562	2.84E-08	2.04E-07	0.04	0.14	0.49	0.93	XXbac-B476	--	chr22	antisense
ENSG00000248671.6	5.22256039	-2.8832898	0.00538165	0.01488851	0	0	0.1	0.04	ALG1L9P	asparagine-linked glycosylation 1-like 9, pseudogene	chr11	processed_transcript
ENSG00000271930.1	2.48592247	-2.4052393	0.00737015	0.01977584	0.11	0	0.76	0.16	RP11-44N12	--	chr8	lincRNA
ENSG00000260160.1	5.45007274	-2.7494288	0.00158008	0.00495521	0	0	0.09	0.4	CTC-471J1.2	--	chr19	sense_overlapping
ENSG00000262001.1	1.88395621	1.14794963	1.28E-07	8.46E-07	0.19	0.27	1.13	1.14	DLGAP1-AS2	DLGAP1 antisense RNA 2	chr18	antisense
ENSG00000281348.1	-1.6100337	2.52259525	2.70E-07	1.70E-06	12.07	13.05	5.76	5.19	CTD-2574D2	--	chr16	protein_coding
ENSG00000155313.14	1.38660487	2.10632539	1.58E-05	7.40E-05	0.35	0.36	1.03	1.44	USP25	ubiquitin specific peptidase 25	chr21	protein_coding
ENSG00000162004.15	-1.5776935	2.9678155	2.61E-07	1.64E-06	5.41	6.35	2.23	3.03	CCDC78	coiled-coil domain containing 78	chr16	protein_coding
ENSG00000073737.15	-5.2091082	-3.0354822	0.00538165	0.01488851	0.03	0.03	0	0	DHRS9	dehydrogenase/reductase (SDR family) member 9	chr2	protein_coding
ENSG00000249242.6	4.0694406	0.05206715	2.86E-14	4.24E-13	0.03	0.02	0.48	0.6	TMEM150C	transmembrane protein 150C	chr4	protein_coding
ENSG00000199879.1	2.90443354	-2.7265114	0.01615662	0.03958166	0	0.18	1.84	0.35	RNU1-120P	RNA, U1 small nuclear 120, pseudogene	chr1	snRNA
ENSG00000181804.13	-2.3951501	-0.4570267	5.33E-07	3.21E-06	0.35	0.16	0.08	0.05	SLC9A9	solute carrier family 9, subfamily A (NHE9, cation proton antiporter 9), member 9	chr3	protein_coding
ENSG00000271933.1	-1.8351846	-0.8882306	0.00018753	0.00071119	0.32	0.26	0.07	0.14	RP11-338I21	--	chr10	antisense
ENSG00000136758.17	1.59778231	4.15824473	8.70E-08	5.87E-07	0.82	1.18	3.14	4.94	YME1L1	YME1-like 1 ATPase	chr10	protein_coding
ENSG00000185130.5	1.43565862	0.61778184	0.00011558	0.00045864	1.26	1.34	6.04	3.35	HIST1H2BL	histone cluster 1, H2bl	chr6	protein_coding
ENSG00000011347.8	1.24949629	3.56690533	2.95E-05	0.00013117	1.05	0.93	3.04	3.22	SYT7	synaptotagmin VII	chr11	protein_coding
ENSG00000162664.15	1.23362812	3.84127356	3.37E-05	0.0001488	0.5	0.76	1.35	2.63	ZNF326	zinc finger protein 326	chr1	protein_coding
ENSG00000085982.12	-2.9117837	4.55710125	1.17E-20	2.96E-19	4.3	4.71	0.69	0.91	USP40	ubiquitin specific peptidase 40	chr2	protein_coding
ENSG00000056736.8	2.95662918	2.20171096	1.60E-17	3.18E-16	0.37	0.45	4.18	4.36	IL17RB	interleukin 17 receptor B	chr3	protein_coding
ENSG00000135423.11	2.23178783	1.86669191	5.65E-11	5.83E-10	0.34	0.4	2.46	2.17	GLS2	glutaminase 2 (liver, mitochondrial)	chr12	protein_coding
ENSG00000267342.1	-2.4364393	-2.3901683	0.00455824	0.01291303	0.23	0.04	0.02	0.04	RP11-552F3.	--	chr17	antisense
ENSG00000101222.11	1.29525966	-0.0073965	0.00153861	0.00487879	0.14	0.41	0.97	0.81	SPEF1	sperm flagellar 1	chr20	protein_coding
ENSG00000110675.11	4.23732124	-0.6655051	6.09E-11	6.25E-10	0.02	0.01	0.32	0.39	ELMOD1	ELMO/CED-12 domain containing 1	chr11	protein_coding
ENSG00000171148.12	-1.4018675	7.77185123	1.29E-06	7.31E-06	73.69	75.36	40.06	34.95	TADA3	transcriptional adaptor 3	chr3	protein_coding
ENSG00000074603.17	1.59000585	2.48316687	4.90E-07	2.97E-06	0.25	0.25	0.79	1.21	DPP8	dipeptidyl-peptidase 8	chr15	protein_coding
ENSG00000182179.9	3.43108673	2.18142884	1.78E-21	4.82E-20	0.21	0.15	2.79	2.5	UBA7	ubiquitin-like modifier activating enzyme 7	chr3	protein_coding
ENSG00000198910.11	-2.5023789	9.33177344	7.29E-17	1.36E-15	170.8	175.63	41.75	39.64	L1CAM	L1 cell adhesion molecule	chrX	protein_coding
ENSG00000122971.7	1.40368123	3.83904778	2.57E-06	1.39E-05	3.47	2.56	12	9.24	ACADS	acyl-CoA dehydrogenase, C-2 to C-3 short chain	chr12	protein_coding
ENSG00000167178.14	-3.4813712	1.66179378	3.23E-20	7.88E-19	0.92	0.88	0.16	0.06	ISLR2	immunoglobulin superfamily containing leucine-rich repeat 2	chr15	protein_coding
ENSG00000277669.1	-5.0252555	-3.1285772	0.0100455	0.02582742	0	0.33	0	0	AC009133.22	--	chr16	protein_coding
ENSG00000258890.5	1.1979697	2.88008352	8.67E-05	0.00035173	0.84	1.14	2.48	3.59	CEP95	centrosomal protein 95kDa	chr17	protein_coding
ENSG00000117013.13	-4.9732372	-1.6497204	6.31E-08	4.36E-07	0.12	0.1	0.01	0	KCNQ4	potassium channel, voltage gated KQT-like subfamily Q, member 4	chr1	protein_coding
ENSG00000186130.4	1.4124313	0.60079465	0.00016684	0.00063822	0.21	0.1	0.35	0.74	ZBTB6	zinc finger and BTB domain containing 6	chr9	protein_coding
ENSG00000280434.1	-1.9788393	0.44877772	3.55E-07	2.20E-06	0.09	0.15	0.05	0.03	RP4-671O14	--	chr22	sense_overlapping
ENSG00000042832.10	-4.7422872	-1.8413269	8.18E-08	5.55E-07	0.02	0.07	0	0	TG	thyroglobulin	chr8	protein_coding
ENSG00000163714.16	2.57576699	4.37234716	8.68E-17	1.61E-15	0.68	0.63	3.42	7.12	U2SURP	U2 snRNP-associated SURP domain containing	chr3	protein_coding
ENSG00000171055.13	1.32884891	3.97449743	7.73E-06	3.84E-05	2.83	3.78	7.51	14.74	FEZ2	fasciculation and elongation protein zeta 2 (zygin II)	chr2	protein_coding
ENSG00000165175.14	-2.0491489	6.53482264	4.48E-12	5.23E-11	31.48	32.33	11.06	9.43	MID1IP1	MID1 interacting protein 1	chrX	protein_coding
ENSG00000066248.13	-4.9007055	6.13296487	4.92E-47	5.81E-45	32.1	28.44	1.53	1.17	NGEF	neuronal guanine nucleotide exchange factor	chr2	protein_coding
ENSG00000172367.14	6.24952512	-2.2192205	1.55E-05	7.26E-05	0	0	0.07	0.13	PDZD3	PDZ domain containing 3	chr11	protein_coding
ENSG00000137309.18	-3.0829117	9.19077511	1.10E-23	3.46E-22	360.92	366.6	60.18	54.03	HMGA1	high mobility group AT-hook 1	chr6	protein_coding
ENSG00000160447.6	-2.3438467	6.52755795	4.78E-15	7.66E-14	37.71	36.23	10.51	8.86	PKN3	protein kinase N3	chr9	protein_coding
ENSG00000151376.15	-4.352929	4.43646009	2.14E-37	1.41E-35	13.55	11.9	1.04	0.62	ME3	malic enzyme 3, NADP(+)-dependent, mitochondrial	chr11	protein_coding
ENSG00000006016.9	-2.7186446	2.70838056	9.25E-17	1.71E-15	6.18	4.05	0.84	1.23	CRLF1	cytokine receptor-like factor 1	chr19	protein_coding
ENSG00000166503.7	2.77839413	3.06366991	1.63E-17	3.23E-16	0.12	0.15	0.96	1.49	HDGFRP3	--	chr15	protein_coding
ENSG00000145882.9	2.30823159	4.99661612	2.54E-14	3.77E-13	2.49	3.4	18.96	19.87	PCYOX1L	prenylcysteine oxidase 1 like	chr5	protein_coding
ENSG00000177106.13	1.19122957	7.24907882	3.62E-05	0.0001591	20.66	19.78	60.18	62.96	EPS8L2	EPS8-like 2	chr11	protein_coding
ENSG00000170214.3	-3.1874933	-1.5861463	3.56E-06	1.88E-05	0.25	0.24	0.04	0.03	ADRA1B	adrenoceptor alpha 1B	chr5	protein_coding
ENSG00000152661.7	-5.3928502	-0.5498047	8.03E-16	1.37E-14	0.3	0.39	0	0.02	GJA1	gap junction protein, alpha 1, 43kDa	chr6	protein_coding
ENSG00000140876.10	3.88873543	-1.4144752	1.03E-06	5.93E-06	0	0.04	0.34	0.41	NUDT7	nudix (nucleoside diphosphate linked moiety X)-type motif 7	chr16	protein_coding
ENSG00000124762.12	2.0077858	7.11885709	1.05E-11	1.18E-10	17.96	17.73	101.84	89.09	CDKN1A	cyclin-dependent kinase inhibitor 1A (p21, Cip1)	chr6	protein_coding
ENSG00000228863.7	5.04018138	1.46904765	2.58E-28	1.09E-26	0.03	0.03	1.56	1.13	RP11-404F1(	--	chr1	antisense
ENSG00000138175.8	-1.062765	4.32760793	0.0002844	0.00104006	5.61	5.82	3.43	3.86	ARL3	ADP-ribosylation factor-like 3	chr10	protein_coding
ENSG00000184574.8	-2.2224319	-1.8208999	0.00140662	0.00449532	0.1	0.1	0	0.05	LPAR5	lysophosphatidic acid receptor 5	chr12	protein_coding
ENSG00000277501.1	5.9655835	-2.4200372	8.36E-05	0.00034052	0	0	0.26	0.2	RP11-697E2	--	chr17	antisense
ENSG00000081386.11	3.15306433	0.16878844	8.99E-12	1.02E-10	0.02	0.04	0.3	0.41	ZNF510	zinc finger protein 510	chr9	protein_coding
ENSG00000126705.12	-1.7166953	4.69325861	7.51E-09	5.85E-08	4.92	4.65	2.06	1.81	AHDC1	AT hook, DNA binding motif, containing 1	chr1	protein_coding
ENSG00000201512.1	-5.3787292	-2.9446861	0.00290522	0.00856312	0	1.9	0	0	SNORA71C	small nucleolar RNA, H/ACA box 71C	chr20	snoRNA
ENSG00000117480.14	4.80762964	3.50188304	4.60E-39	3.37E-37	0.14	0.46	11.72	10.53	FAAH	fatty acid amide hydrolase	chr1	protein_coding
ENSG00000280077.1	2.24396317	-0.4653586	3.03E-06	1.62E-05	0.08	0.1	0.71	0.5	RP11-87H9.4	--	chr9	TEC
ENSG00000007038.9	-2.0682744	5.62127378	3.54E-12	4.19E-11	59.64	52.38	19.92	15.58	PRSS21	protease, serine, 21 (testisin)	chr16	protein_coding
ENSG00000187091.12	-1.7376097	1.98167484	1.17E-07	7.79E-07	1.74	1.29	0.82	0.4	PLCD1	phospholipase C, delta 1	chr3	protein_coding
ENSG00000243135.4	4.53245661	-0.0501739	1.66E-15	2.77E-14	0	0.03	0.3	0.5	UGT1A3	UDP glucuronosyltransferase 1 family, polypeptide A3	chr2	protein_coding
ENSG00000119917.12	1.50392835	1.6261252	6.44E-06	3.24E-05	0.2	0.76	1.36	2.27	IFIT3	interferon-induced protein with tetratricopeptide repeats 3	chr10	protein_coding
ENSG00000111843.12	-1.1433172	5.99506485	7.66E-05	0.00031603	58.25	61.53	32.92	39.46	TMEM14C	transmembrane protein 14C	chr6	protein_coding
ENSG00000275162.1	5.66878161	-2.6135853	0.00086564	0.00288128	0	0	0.28	0.88	LLNLR-260G	--	chr19	sense_intronic
ENSG00000083290.18	2.28132782	3.04144456	6.62E-13	8.46E-12	0.37	0.35	2.27	2.37	ULK2	unc-51 like autophagy activating kinase 2	chr17	protein_coding
ENSG00000122008.14	3.03289826	-0.4546439	1.57E-08	1.17E-07	0.02	0.02	0.12	0.29	POLK	polymerase (DNA directed) kappa	chr5	protein_coding
ENSG00000167377.16	1.91817335	0.38865055	1.50E-06	8.40E-06	0.14	0.04	0.4	0.52	ZNF23	zinc finger protein 23	chr16	protein_coding
ENSG00000276509.1	7.50205423	-1.2284838	4.96E-11	5.14E-10	0	0	0.22	0.5	CH17-353B1	--	chr1	lincRNA
ENSG00000260092.1	-1.0199017	-0.100534	0.01326645	0.03319311	0.45	0.45	0.29	0.31	RP11-77K12.	--	chr16	protein_coding
ENSG00000183530.12	1.43985422	2.76649189	3.29E-06	1.75E-05	0.3	0.24	1	0.95	PRR14L	proline rich 14-like	chr22	protein_coding
ENSG00000196312.10	1.10238463	0.63685329	0.00282618	0.00842548	0.87	1.03	2.41	3.04	HIATL2	hippocampus abundant transcript-like 2	chr9	protein_coding
ENSG00000182584.4	1.85790061	1.10177503	2.20E-07	1.41E-06	0.3	0.41	1.63	1.8	ACTL10	actin-like 10	chr20	protein_coding
ENSG00000101670.10	-5.1996784	1.78798646	7.07E-33	3.76E-31	0.47	0.46	0.03	0.01	LIPG	lipase, endothelial	chr18	protein_coding
ENSG00000029363.14	2.08952992	4.2580853	5.97E-12	6.89E-11	0.73	0.88	3.63	5.51	BCLAF1	BCL2-associated transcription factor 1	chr6	protein_coding
ENSG00000215014.4	3.45097627	-0.4750206	3.93E-10	3.64E-09	0.04	0.04	0.44	0.74	RP5-832C2.5	--	chr1	lincRNA
ENSG00000104889.4	-1.7160749	7.89925183	4.24E-09	3.43E-08	276.96	231.34	103.99	102.26	RNASEH2A	ribonuclease H2, subunit A	chr19	protein_coding
ENSG00000249328.2	-7.762469	-1.1841467	2.65E-12	3.17E-11	0.28	0.35	0	0	RP11-26J3.1	--	chr8	lincRNA
ENSG00000140386.11	1.40301063	1.37723287	3.81E-05	0.00016672	0.24	0.17	0.58	0.89	SCAPER	S-phase cyclin A-associated protein in the ER	chr15	protein_coding
ENSG00000273003.1	-1.0314691	4.71159879	0.00039758	0.00141657	11.29	12.51	8.5	6.97	ARL2-SNX15	ARL2-SNX15 readthrough (NMD candidate)	chr11	protein_coding
ENSG00000198358.4	3.12542467	-2.5990352	0.00552196	0.01522223	0	0.01	0.1	0.08	RP11-544M2	--	chr1	antisense
ENSG00000166473.15	5.6923233	-2.6004234	0.00047761	0.00167191	0	0	0.02	0.01	PKD1L2	polycystic kidney disease 1-like 2 (gene/pseudogene)	chr16	polymorphic_pseudogene
ENSG00000196116.7	2.94713012	1.72668878	4.07E-16	7.14E-15	0.18	0.13	0.98	2.22	TDRD7	tudor domain containing 7	chr9	protein_coding
ENSG00000082458.10	-2.566645	3.65282432	2.43E-16	4.35E-15	2.38	2.73	0.46	0.69	DLG3	discs, large homolog 3 (Drosophila)	chrX	protein_coding
ENSG00000260855.1	1.71144986	-1.7775325	0.01347572	0.03367454	0.05	0	0.12	0.09	RP11-439E1	--	chr1	antisense
ENSG00000133321.9	1.30334769	-0.0977137	0.00184437	0.0056998	0.36	0.27	1.13	0.94	RARRES3	retinoic acid receptor responder (tazarotene induced) 3	chr11	protein_coding
ENSG00000078070.10	2.15831169	4.89045131	7.95E-13	1.01E-11	2.72	2.85	16.01	17.12	MCCC1	methylcrotonoyl-CoA carboxylase 1 (alpha)	chr3	protein_coding
ENSG00000148634.14	1.84580917	3.08087387	2.83E-09	2.33E-08	0.38	0.36	1.49	2.05	HERC4	HECT and RLD domain containing E3 ubiquitin protein ligase 4	chr10	protein_coding
ENSG00000106628.9	-1.3363671	7.97262267	3.78E-06	1.99E-05	136.8	116.44	71.06	62.42	POLD2	polymerase (DNA directed), delta 2, accessory subunit	chr7	protein_coding
ENSG00000272602.4	9.22164567	0.30656901	1.57E-23	4.90E-22	0	0	0.54	0.82	ZNF595	zinc finger protein 595	chr4	protein_coding
ENSG00000162542.12	-1.3610265	5.07794967	3.19E-06	1.70E-05	12.95	12.06	6.37	6.62	TMCO4	transmembrane and coiled-coil domains 4	chr1	protein_coding
ENSG00000257390.4	-1.5444123	4.91958064	1.48E-07	9.69E-07	20.69	19.67	8.51	9.96	RP11-762I7.5	--	chr12	protein_coding
ENSG00000270959.1	1.03581504	1.44950993	0.00177166	0.00548994	0.47	0.52	1.12	1.58	LPP-AS2	LPP antisense RNA 2	chr3	antisense
ENSG00000186272.11	1.43525391	-0.3669401	0.00138295	0.00443091	0.12	0.1	0.2	0.6	ZNF17	zinc finger protein 17	chr19	protein_coding
ENSG00000149231.10	3.4803355	1.45082643	5.32E-19	1.19E-17	0.08	0.14	0.62	2.67	CCDC82	coiled-coil domain containing 82	chr11	protein_coding
ENSG00000152518.6	1.18145266	3.38677641	8.43E-05	0.00034273	1.11	1.67	3.78	4.64	ZFP36L2	ZFP36 ring finger protein-like 2	chr2	protein_coding
ENSG00000007944.13	2.4241558	0.10781934	3.02E-08	2.16E-07	0.05	0.11	0.45	0.73	MYLIP	myosin regulatory light chain interacting protein	chr6	protein_coding
ENSG00000169715.13	-10.277441	5.94117631	2.60E-99	5.20E-96	96.02	93.02	0.09	0.1	MT1E	metallothionein 1E	chr16	protein_coding
ENSG00000264895.1	5.3196533	-2.8266272	0.00290522	0.00856312	0	0	0	0.14	RP11-421E1	--	chr17	sense_intronic
ENSG00000171522.5	-3.3916308	-1.4176934	1.94E-06	1.07E-05	0.12	0.14	0.03	0	PTGER4	prostaglandin E receptor 4 (subtype EP4)	chr5	protein_coding
ENSG00000131653.11	-1.4079922	7.76161547	1.17E-06	6.63E-06	64.55	58.8	32.02	29.88	TRAF7	TNF receptor-associated factor 7, E3 ubiquitin protein ligase	chr16	protein_coding
ENSG00000198189.9	1.68073654	2.14994298	1.89E-07	1.22E-06	0.75	1.02	2.99	4.61	HSD17B11	hydroxysteroid (17-beta) dehydrogenase 11	chr4	protein_coding
ENSG00000135127.10	1.79937892	3.87176063	2.81E-09	2.31E-08	1.3	2.15	7.71	8.25	CCDC64	coiled-coil domain containing 64	chr12	protein_coding
ENSG00000172346.13	-2.9158689	1.89636923	1.56E-16	2.85E-15	1.7	2.12	0.47	0.21	CSDC2	cold shock domain containing C2, RNA binding	chr22	protein_coding
ENSG00000122482.19	3.03230229	1.16824988	9.82E-15	1.53E-13	0.02	0.11	0.33	1.05	ZNF644	zinc finger protein 644	chr1	protein_coding
ENSG00000280164.1	5.96106449	-2.422601	8.36E-05	0.00034052	0	0	0.25	0.24	CH507-254N	--	chr21	lincRNA
ENSG00000160767.19	-1.1521046	5.68347806	6.96E-05	0.00028904	19.38	16.52	10.9	10.63	FAM189B	family with sequence similarity 189, member B	chr1	protein_coding
ENSG00000166025.16	-1.6563548	3.96658469	3.24E-08	2.31E-07	2.14	2.13	1	0.8	AMOTL1	angiomotin like 1	chr11	protein_coding
ENSG00000176124.10	-1.074731	2.93022388	0.00039697	0.00141462	7.99	9.01	5.39	5.36	DLEU1	deleted in lymphocytic leukemia 1 (non-protein coding)	chr13	protein_coding
ENSG00000106537.7	1.20965594	1.852277	0.00018576	0.00070536	0.82	0.83	2.07	3.02	TSPAN13	tetraspanin 13	chr7	protein_coding
ENSG00000154642.9	1.91673584	-0.1435054	1.56E-05	7.32E-05	0.04	0.06	0.14	0.37	C21orf91	chromosome 21 open reading frame 91	chr21	protein_coding
ENSG00000234160.1	-2.1483031	-0.1099902	6.50E-07	3.85E-06	4.07	4.12	1.22	1.22	RP11-613M1	--	chr9	antisense
ENSG00000127837.8	-1.8176977	6.96930999	5.87E-10	5.29E-09	81.6	69.37	27.91	29.22	AAMP	angio-associated, migratory cell protein	chr2	protein_coding
ENSG00000170075.8	-2.2010514	-1.8115204	0.00140662	0.00449532	0.05	0.05	0.02	0.01	GPR37L1	G protein-coupled receptor 37 like 1	chr1	protein_coding
ENSG00000176165.9	-2.3202132	-0.6923755	2.60E-06	1.41E-05	0.11	0.21	0.05	0.03	FOXG1	forkhead box G1	chr14	protein_coding
ENSG00000175772.10	-2.0321069	5.0199695	1.09E-11	1.21E-10	16.33	18.24	5.97	5.27	LINC01106	long intergenic non-protein coding RNA 1106	chr2	lincRNA
ENSG00000225506.2	-3.1758553	-2.8664902	0.01615662	0.03958166	0.12	0.15	0	0.03	CYP4A22-AS	CYP4A22 antisense RNA 1	chr1	lincRNA
ENSG00000278705.1	6.75567764	-1.8389777	2.24E-07	1.42E-06	0	0	0.73	1.24	HIST1H4B	histone cluster 1, H4b	chr6	protein_coding
ENSG00000204764.11	-6.1863178	-2.4412039	4.74E-05	0.00020301	0.07	0.02	0	0	RANBP17	RAN binding protein 17	chr5	protein_coding
ENSG00000242574.7	3.10887381	1.10934391	3.63E-15	5.87E-14	0.33	0.16	3.58	2.11	HLA-DMB	major histocompatibility complex, class II, DM beta	chr6	protein_coding
ENSG00000184160.7	1.59620716	3.30044836	1.68E-07	1.09E-06	1.76	1.85	6.7	7.84	ADRA2C	adrenoceptor alpha 2C	chr4	protein_coding
ENSG00000230366.8	2.29343573	-2.5181978	0.01191236	0.03011315	0.03	0	0.25	0	DSCR9	Down syndrome critical region 9 (non-protein coding)	chr21	lincRNA
ENSG00000185684.11	1.48547142	2.6829664	1.78E-06	9.86E-06	1.12	0.98	3.9	3.95	EP400NL	EP400 N-terminal like	chr12	protein_coding
ENSG00000119915.4	1.47557103	3.6904599	8.82E-07	5.12E-06	4.17	3.82	14.79	14.88	ELOVL3	ELOVL fatty acid elongase 3	chr10	protein_coding
ENSG00000135698.8	1.84034115	4.55983715	7.59E-10	6.72E-09	5.07	7.33	23.36	36	MPHOSPH6	M-phase phosphoprotein 6	chr16	protein_coding
ENSG00000170545.15	-1.2864373	4.27089013	1.27E-05	6.05E-05	6.21	6.7	3.55	3.5	SMAGP	small cell adhesion glycoprotein	chr12	protein_coding
ENSG00000266088.4	-1.3271552	-0.2125627	0.00161092	0.00504139	0.79	1.09	0.57	0.43	RP5-1028K7.	--	chr17	lincRNA
ENSG00000132718.8	2.62422629	-0.7844409	1.08E-06	6.18E-06	0.03	0.01	0.12	0.23	SYT11	synaptotagmin XI	chr1	protein_coding
ENSG00000260018.1	1.95233194	1.2216325	4.04E-08	2.84E-07	1.64	1.2	7.62	7.11	RP11-505K9.	--	chr16	antisense
ENSG00000131725.12	1.61632511	2.34708291	3.98E-07	2.45E-06	0.41	0.55	1.7	2.23	WDR44	WD repeat domain 44	chrX	protein_coding
ENSG00000109670.12	1.2233567	1.45088749	0.00028474	0.00104101	0.17	0.25	0.46	0.85	FBXW7	F-box and WD repeat domain containing 7, E3 ubiquitin protein ligase	chr4	protein_coding
ENSG00000140368.11	-6.1847149	-2.4420072	4.74E-05	0.00020301	0.19	0.03	0	0	PSTPIP1	proline-serine-threonine phosphatase interacting protein 1	chr15	protein_coding
ENSG00000135334.8	-1.4294182	4.3978479	1.29E-06	7.27E-06	13.97	15.98	7.29	7.52	AKIRIN2	akirin 2	chr6	protein_coding
ENSG00000175505.10	2.25916602	4.43646359	1.21E-13	1.67E-12	3.1	3	19.87	19.04	CLCF1	cardiotrophin-like cytokine factor 1	chr11	protein_coding
ENSG00000180787.5	9.85623348	0.90299456	1.84E-30	8.65E-29	0	0	0.55	0.9	ZFP3	ZFP3 zinc finger protein	chr17	protein_coding
ENSG00000103089.7	-2.9304994	-0.413772	1.78E-09	1.51E-08	0.36	0.49	0.09	0.06	FA2H	fatty acid 2-hydroxylase	chr16	protein_coding
ENSG00000112651.10	-1.4673286	6.64718782	4.48E-07	2.73E-06	55.48	53.43	25.2	27.34	MRPL2	mitochondrial ribosomal protein L2	chr6	protein_coding
ENSG00000164221.11	2.66016926	0.68869941	4.83E-11	5.02E-10	0.09	0.13	0.4	1.44	CCDC112	coiled-coil domain containing 112	chr5	protein_coding
ENSG00000136002.15	-1.8641401	6.00134015	2.55E-10	2.41E-09	10.29	9.25	3.46	3.7	ARHGEF4	Rho guanine nucleotide exchange factor (GEF) 4	chr2	protein_coding
ENSG00000165156.13	2.36989039	0.9176208	5.64E-10	5.09E-09	0.12	0.05	0.3	0.9	ZHX1	zinc fingers and homeoboxes 1	chr8	protein_coding
ENSG00000054654.14	2.08280208	3.98749197	8.97E-12	1.01E-10	0.2	0.2	1.16	1.1	SYNE2	spectrin repeat containing, nuclear envelope 2	chr14	protein_coding
ENSG00000175764.13	-1.4408062	1.68760447	1.13E-05	5.43E-05	1.2	0.94	0.57	0.48	TTLL11	tubulin tyrosine ligase-like family member 11	chr9	protein_coding
ENSG00000087903.11	-1.4874617	3.79934582	6.54E-07	3.87E-06	4.08	4.03	1.84	2.02	RFX2	regulatory factor X, 2 (influences HLA class II expression)	chr19	protein_coding
ENSG00000137700.15	-1.2841972	3.98038977	1.50E-05	7.06E-05	7.21	5.83	3.94	3.18	SLC37A4	solute carrier family 37 (glucose-6-phosphate transporter), member 4	chr11	protein_coding

ENSG00000215644.8	-3.3210053	-2.2223785	0.00013996	0.00054609	0.19	0.03	0	0.02	GCGR	glucagon receptor	chr17	protein_coding
ENSG00000197863.7	2.0569076	-0.9328081	0.00016946	0.00064769	0.02	0.05	0.2	0.22	ZNF790	zinc finger protein 790	chr19	protein_coding
ENSG00000158747.12	-2.1291633	2.98158982	1.34E-11	1.48E-10	3.35	2.96	1.11	0.81	NBL1	neuroblastoma 1, DAN family BMP antagonist	chr1	protein_coding
ENSG00000137642.11	2.78597203	3.74754586	1.71E-18	3.67E-17	0.22	0.24	1.92	2.31	SORL1	sortilin-related receptor, L(DLR class) A repeats containing	chr11	protein_coding
ENSG00000160191.16	-2.9291516	3.64478088	5.03E-20	1.21E-18	8.25	8.49	1.44	1.49	PDE9A	phosphodiesterase 9A	chr21	protein_coding
ENSG00000144579.6	-1.153399	6.22539522	6.56E-05	0.00027409	31.07	29.01	18.91	17.04	CTDSP1	CTD (carboxy-terminal domain, RNA polymerase II, polypeptide A) small phosphatase 1	chr2	protein_coding
ENSG00000102081.12	1.1524997	2.72491539	0.00017776	0.00067756	0.38	0.64	1.18	1.85	FMR1	fragile X mental retardation 1	chrX	protein_coding
ENSG00000105122.11	2.47724646	-1.248238	5.48E-05	0.00023182	0.03	0.02	0.18	0.2	RASAL3	RAS protein activator like 3	chr19	protein_coding
ENSG00000211456.9	2.54781238	2.00595066	1.16E-13	1.60E-12	0.24	0.25	1.96	1.84	SACM1L	SAC1 suppressor of actin mutations 1-like (yeast)	chr3	protein_coding
ENSG00000274750.1	4.73235456	-3.1388833	0.01889873	0.04519557	0	0	0.55	0	HIST1H3E	histone cluster 1, H3e	chr6	protein_coding
ENSG00000225614.2	-1.7583198	4.77878637	3.26E-09	2.67E-08	2.3	2.87	1.04	0.99	ZNF469	zinc finger protein 469	chr16	protein_coding
ENSG00000265943.1	3.00601829	-2.6695897	0.00943894	0.02469931	0.05	0	0.28	0.33	RP11-739L1(	--	chr18	antisense
ENSG00000237188.3	1.05034286	-0.2573423	0.01695247	0.04139601	0.07	0.16	0.2	0.41	RP11-337C1!	--	chr1	antisense
ENSG00000156531.15	1.76194838	0.76768971	2.36E-06	1.29E-05	0.07	0.07	0.24	0.43	PHF6	PHD finger protein 6	chrX	protein_coding
ENSG00000104848.1	2.10347269	-1.5159029	0.00122775	0.00396173	0.02	0.01	0.12	0.11	KCNA7	potassium channel, voltage gated shaker related subfamily A, member 7	chr19	protein_coding
ENSG00000204099.10	-9.9030749	0.85397244	3.01E-32	1.54E-30	0.79	1.18	0	0	NEU4	sialidase 4	chr2	protein_coding
ENSG00000165934.11	1.47123312	3.10711014	1.48E-06	8.28E-06	0.24	0.32	0.78	1.3	CPSF2	cleavage and polyadenylation specific factor 2, 100kDa	chr14	protein_coding
ENSG00000198948.10	1.19188022	1.14449782	0.00063415	0.00217573	0.13	0.14	0.33	0.51	MFAP3L	microfibrillar-associated protein 3-like	chr4	protein_coding
ENSG00000261553.4	-1.7748986	1.1006468	4.42E-07	2.70E-06	0.66	0.62	0.26	0.24	RP11-29G8.3	--	chr13	processed_transcript
ENSG00000280195.1	-1.7146946	0.93592834	1.25E-06	7.06E-06	1.54	0.86	0.3	0.67	XX-FW83563	--	chrX	antisense
ENSG00000137941.15	3.1582004	-0.3522106	2.33E-09	1.95E-08	0.02	0.01	0.09	0.24	TTLL7	tubulin tyrosine ligase-like family member 7	chr1	protein_coding
ENSG00000118200.13	1.83976891	2.33282594	1.22E-08	9.22E-08	0.15	0.32	0.84	1.43	CAMSAP2	calmodulin regulated spectrin-associated protein family, member 2	chr1	protein_coding
ENSG00000236383.6	3.10155371	-2.6122053	0.00943894	0.02469931	0	0.01	0.02	0.1	LINC00854	long intergenic non-protein coding RNA 854	chr17	processed_transcript
ENSG00000126870.14	1.1167424	4.33507092	0.00014213	0.0005539	2.5	2.95	6.26	9.56	WDR60	WD repeat domain 60	chr7	protein_coding
ENSG00000260924.2	-1.054033	3.31576825	0.00042488	0.00150667	7.33	7.69	4.7	4.94	LINC01311	long intergenic non-protein coding RNA 1311	chr22	antisense
ENSG00000279877.1	4.0105399	-1.3232	3.07E-07	1.92E-06	0.06	0	0.93	0.66	RP11-420N3	--	chr16	processed_transcript
ENSG00000137942.15	2.13552624	2.56511481	3.92E-11	4.10E-10	0.22	0.41	1.29	2.43	FNBP1L	formin binding protein 1-like	chr1	protein_coding
ENSG00000274828.1	-1.2069499	2.31195247	0.00010232	0.00041147	2.56	2.83	1.28	1.83	RP11-567M1	--	chr18	lincRNA
ENSG00000259332.2	1.04503933	2.6631898	0.0006522	0.00223192	3.92	3.76	9.73	11.44	ST20-MTHFS	ST20-MTHFS readthrough	chr15	protein_coding
ENSG00000164849.8	3.064192	0.55409767	8.09E-13	1.02E-11	0.05	0.19	1.68	1.01	GPR146	G protein-coupled receptor 146	chr7	protein_coding
ENSG00000126749.13	-1.3526637	7.24692882	2.97E-06	1.60E-05	37.03	35.11	19.21	18.41	EMG1	EMG1 N1-specific pseudouridine methyltransferase	chr12	protein_coding
ENSG00000146757.12	3.71987147	-0.047458	4.23E-13	5.50E-12	0.06	0	0.18	0.9	ZNF92	zinc finger protein 92	chr7	protein_coding
ENSG00000156136.8	1.44150409	1.05865659	4.21E-05	0.00018297	0.31	0.3	0.74	1.5	DCK	deoxycytidine kinase	chr4	protein_coding
ENSG00000233654.1	-1.0955652	0.27434235	0.00374251	0.01079134	4.1	2.24	1.67	2.29	AC093388.3	--	chr2	antisense
ENSG00000185519.8	-3.0063215	3.35294423	1.89E-20	4.69E-19	9.04	8.31	1.61	1.27	FAM131C	family with sequence similarity 131, member C	chr1	protein_coding
ENSG00000168152.11	4.88946445	-3.0622981	0.01889873	0.04519557	0	0	0.02	0.04	THAP9	THAP domain containing 9	chr4	protein_coding
ENSG00000189129.12	2.98083297	-2.6824604	0.01615662	0.03958166	0	0.03	0	0.31	PLAC9	placenta-specific 9	chr10	protein_coding
ENSG00000176018.11	2.9580371	-0.2248711	2.79E-09	2.31E-08	0.01	0.05	0.14	0.52	LYSMD3	LysM, putative peptidoglycan-binding, domain containing 3	chr5	protein_coding
ENSG00000114279.12	-6.4516108	-0.3524315	3.09E-19	7.03E-18	0.16	0.21	0	0	FGF12	fibroblast growth factor 12	chr3	protein_coding
ENSG00000042493.14	-2.3060414	7.56659785	1.04E-14	1.62E-13	182.48	148.78	45.99	43.27	CAPG	capping protein (actin filament), gelsolin-like	chr2	protein_coding
ENSG00000111269.2	1.41072238	3.52311981	2.88E-06	1.55E-05	1.22	1.47	4.56	4.98	CREBL2	cAMP responsive element binding protein-like 2	chr12	protein_coding
ENSG00000063854.11	1.02434789	6.5895843	0.00037339	0.00133797	16.55	16.11	44.8	43.7	HAGH	hydroxyacylglutathione hydrolase	chr16	protein_coding
ENSG00000126217.19	3.06547399	-0.7940929	7.16E-08	4.91E-07	0.01	0.01	0.09	0.1	MCF2L	MCF.2 cell line derived transforming sequence-like	chr13	protein_coding
ENSG00000165300.7	-12.209672	3.15861999	1.41E-63	4.27E-61	0.69	0.68	0	0	SLITRK5	SLIT and NTRK-like family, member 5	chr13	protein_coding
ENSG00000006638.10	-4.9467932	3.27468624	2.24E-40	1.75E-38	6.37	5.28	0.18	0.31	TBXA2R	thromboxane A2 receptor	chr19	protein_coding
ENSG00000184903.8	-2.1670864	2.65082681	1.04E-11	1.17E-10	3	3.19	0.93	0.9	IMMP2L	IMP2 inner mitochondrial membrane peptidase-like (S. cerevisiae)	chr7	protein_coding
ENSG00000166225.7	5.74378249	0.1803263	8.79E-20	2.08E-18	0	0.01	0.3	0.34	FRS2	fibroblast growth factor receptor substrate 2	chr12	protein_coding
ENSG00000170745.10	-4.7876964	-0.6030425	3.53E-14	5.16E-13	0.32	0.47	0.01	0.02	KCNS3	potassium voltage-gated channel, modifier subfamily S, member 3	chr2	protein_coding
ENSG00000115839.16	1.11760962	4.65352285	0.00013035	0.00051266	1.71	2.43	5.61	6.36	RAB3GAP1	RAB3 GTPase activating protein subunit 1 (catalytic)	chr2	protein_coding
ENSG00000240583.9	-3.4629951	1.0342737	2.51E-18	5.30E-17	1.14	0.77	0.07	0.15	AQP1	aquaporin 1 (Colton blood group)	chr7	protein_coding
ENSG00000109445.9	-1.0126252	4.0792111	0.00055169	0.00191543	8.96	10.3	4.6	8.17	ZNF330	zinc finger protein 330	chr4	protein_coding
ENSG00000198796.6	-8.7805678	-0.24314	2.99E-20	7.31E-19	0.1	0.27	0	0	ALPK2	alpha-kinase 2	chr18	protein_coding
ENSG00000116652.6	5.9655835	-2.4200372	8.36E-05	0.00034052	0	0	0.08	0.06	DLEU2L	deleted in lymphocytic leukemia 2-like	chr1	sense_intronic
ENSG00000271895.2	6.04022639	-2.3681409	8.36E-05	0.00034052	0	0	0.29	0.31	RP4-635E18.	--	chr1	antisense
ENSG00000120458.8	1.41762043	0.72131725	0.00011287	0.00044869	0.2	0.14	0.38	0.87	MSANTD2	Myb/SANT-like DNA-binding domain containing 2	chr11	protein_coding
ENSG00000159086.13	2.17053774	1.71996302	2.60E-10	2.46E-09	0.22	0.22	0.84	1.84	PAXBP1	PAX3 and PAX7 binding protein 1	chr21	protein_coding
ENSG00000183578.5	-3.9664073	3.47395492	5.51E-31	2.66E-29	7.09	7.88	0.62	0.65	TNFAIP8L3	tumor necrosis factor, alpha-induced protein 8-like 3	chr15	protein_coding
ENSG00000113971.17	2.43322382	-0.2173834	1.66E-07	1.08E-06	0.05	0.04	0.18	0.41	NPHP3	nephronophthisis 3 (adolescent)	chr3	protein_coding
ENSG00000143420.16	-1.0229642	8.00982622	0.00036553	0.00131331	51.72	51.56	33.55	34.18	ENSA	endosulfine alpha	chr1	protein_coding
ENSG00000146281.5	2.21142863	1.7588137	1.07E-10	1.06E-09	0.14	0.26	0.65	1.84	PM20D2	peptidase M20 domain containing 2	chr6	protein_coding
ENSG00000204366.3	-1.4545824	3.31063021	1.50E-06	8.40E-06	5.21	7.35	2.87	3.22	ZBTB12	zinc finger and BTB domain containing 12	chr6	protein_coding
ENSG00000101213.6	-3.1770205	2.30918368	2.27E-20	5.61E-19	2.81	2.09	0.22	0.5	PTK6	protein tyrosine kinase 6	chr20	protein_coding
ENSG00000162426.13	-1.0473354	1.98687046	0.00095132	0.00313901	1.69	1.38	1.03	0.95	SLC45A1	solute carrier family 45, member 1	chr1	protein_coding
ENSG00000259921.1	3.10733779	-2.609976	0.00552196	0.01522223	0.01	0	0.02	0.07	AC022819.3	--	chr15	sense_intronic
ENSG00000115514.10	2.86528141	1.11509147	1.57E-13	2.14E-12	0.15	0.22	1.4	2.23	TXNDC9	thioredoxin domain containing 9	chr2	protein_coding
ENSG00000125864.10	1.24021749	2.4810306	6.84E-05	0.00028495	0.88	0.96	2.53	3.28	BFSP1	beaded filament structural protein 1, filensin	chr20	protein_coding
ENSG00000272872.1	-1.1451314	2.21186108	0.00026555	0.0009753	6.44	8.37	4.43	4.47	LL22NC03-N	--	chr22	sense_intronic
ENSG00000257556.1	3.09160473	1.15543515	2.58E-15	4.24E-14	0.3	0.44	4.51	3.98	RP11-44N21	--	chr14	lincRNA
ENSG00000184378.2	1.16192374	0.1006649	0.00446326	0.01267243	0.24	0.25	0.51	0.96	ACTRT3	actin-related protein T3	chr3	protein_coding
ENSG00000049130.12	1.88411786	-1.8632211	0.01381259	0.03443739	0.03	0.04	0.13	0.21	KITLG	KIT ligand	chr12	protein_coding
ENSG00000176912.3	1.71101656	2.87558019	3.71E-08	2.62E-07	2.98	3.53	15.9	12.39	TYMSOS	TYMS opposite strand	chr18	processed_transcript
ENSG00000158195.9	-1.1465128	7.72368642	6.81E-05	0.00028391	42.67	40.38	25.18	24.8	WASF2	WAS protein family, member 2	chr1	protein_coding
ENSG00000102996.4	1.93015221	6.7915572	5.83E-11	6.00E-10	9.09	8.43	46.93	41.94	MMP15	matrix metallopeptidase 15 (membrane-inserted)	chr16	protein_coding
ENSG00000163611.10	1.57066011	1.55677524	3.25E-06	1.73E-05	0.16	0.27	0.63	1.08	SPICE1	spindle and centriole associated protein 1	chr3	protein_coding
ENSG00000108094.13	2.15773565	1.44245413	1.01E-09	8.82E-09	0.14	0.18	0.46	1.48	CUL2	cullin 2	chr10	protein_coding
ENSG00000120329.6	5.99284665	-2.4046014	8.36E-05	0.00034052	0	0	0.34	0.04	SLC25A2	solute carrier family 25 (mitochondrial carrier; ornithine transporter) member 2	chr5	protein_coding
ENSG00000100033.15	2.41976737	0.29918258	7.97E-09	6.19E-08	0.17	0.06	0.91	0.77	PRODH	proline dehydrogenase (oxidase) 1	chr22	protein_coding
ENSG00000146083.10	-1.4602671	5.28412879	5.92E-07	3.54E-06	11.21	11.05	5.04	5.75	RNF44	ring finger protein 44	chr5	protein_coding
ENSG00000103740.8	4.97142534	-1.276911	1.07E-08	8.19E-08	0	0	0.17	0.04	ACSBG1	acyl-CoA synthetase bubblegum family member 1	chr15	protein_coding
ENSG00000107099.14	5.72174973	0.15713453	1.42E-19	3.29E-18	0.01	0	0.26	0.27	DOCK8	dedicator of cytokinesis 8	chr9	protein_coding
ENSG00000132164.8	3.94506152	3.63805102	1.15E-30	5.46E-29	0.15	0.23	3.92	3.88	SLC6A11	solute carrier family 6 (neurotransmitter transporter), member 11	chr3	protein_coding
ENSG00000203877.6	2.76442414	-1.5089989	6.51E-05	0.00027249	0.07	0.07	0.92	0.46	RIPPLY2	ripply transcriptional repressor 2	chr6	protein_coding
ENSG00000156299.11	5.37110141	0.34023439	1.89E-20	4.69E-19	0.01	0	0.26	0.42	TIAM1	T-cell lymphoma invasion and metastasis 1	chr21	protein_coding
ENSG00000092969.10	2.55871446	2.15744881	4.92E-14	7.10E-13	0.17	0.23	1.48	1.7	TGFB2	transforming growth factor, beta 2	chr1	protein_coding
ENSG00000105619.12	-1.2641802	6.10530875	1.29E-05	6.13E-05	59.25	67.74	38.67	31.54	TFPT	TCF3 (E2A) fusion partner (in childhood Leukemia)	chr19	protein_coding
ENSG00000147454.12	-2.0872381	5.52018137	2.35E-12	2.85E-11	11.99	12.96	3.83	4	SLC25A37	solute carrier family 25 (mitochondrial iron transporter), member 37	chr8	protein_coding
ENSG00000271784.1	1.95877198	-1.2729582	0.00075675	0.00255792	0.04	0.31	0.76	1.08	RP1-28H20.3	--	chr20	lincRNA
ENSG00000249592.4	1.48753141	-0.7432446	0.00215901	0.00657968	0.07	0.22	0.63	0.43	RP11-440L14	--	chr4	antisense
ENSG00000203867.7	6.07185618	0.47951319	4.78E-23	1.43E-21	0	0.01	0.41	0.35	RBM20	RNA binding motif protein 20	chr10	protein_coding
ENSG00000227198.1	-3.5766363	-2.6425303	0.003237	0.00945156	0.48	0	0	0.04	C6orf47-AS1	C6orf47 antisense RNA 1	chr6	antisense
ENSG00000122711.7	-6.1863178	-2.4412039	4.74E-05	0.00020301	0.49	0.17	0	0	SPINK4	serine peptidase inhibitor, Kazal type 4	chr9	protein_coding
ENSG00000238197.4	2.45623396	-1.071651	2.56E-05	0.00011557	0.03	0.02	0.05	0.3	PAXBP1-AS1	PAXBP1 antisense RNA 1	chr21	antisense
ENSG00000234741.6	-3.1663085	6.27984099	1.51E-24	5.02E-23	117.69	107.08	14.12	19.36	GAS5	growth arrest-specific 5 (non-protein coding)	chr1	processed_transcript
ENSG00000182985.15	-6.717315	2.58956134	7.19E-48	8.84E-46	0.9	1.29	0.02	0.01	CADM1	cell adhesion molecule 1	chr11	protein_coding
ENSG00000005059.14	1.566463	3.2866385	2.82E-07	1.76E-06	1.48	1.99	4.95	8.81	CCDC109B	coiled-coil domain containing 109B	chr4	protein_coding
ENSG00000147202.16	-4.5750429	-0.4358102	1.36E-14	2.08E-13	0.1	0.14	0	0.01	DIAPH2	diaphanous-related formin 2	chrX	protein_coding
ENSG00000262621.3	5.96106449	-2.422601	8.36E-05	0.00034052	0	0	0.09	0.08	LA16c-306E5	--	chr16	protein_coding
ENSG00000198554.10	1.53709261	2.2807938	1.44E-06	8.06E-06	0.42	0.24	0.81	1.74	WDHD1	WD repeat and HMG-box DNA binding protein 1	chr14	protein_coding
ENSG00000122376.10	2.16201082	1.00439111	4.64E-09	3.72E-08	0.24	0.07	0.64	1.23	FAM35A	family with sequence similarity 35, member A	chr10	protein_coding
ENSG00000123106.9	1.89855014	1.11135259	1.29E-07	8.55E-07	0.16	0.36	0.95	1.6	CCDC91	coiled-coil domain containing 91	chr12	protein_coding
ENSG00000111490.11	1.91897488	1.59811016	1.81E-08	1.33E-07	0.14	0.11	0.53	0.74	TBC1D30	TBC1 domain family, member 30	chr12	protein_coding
ENSG00000174839.11	2.37031269	2.21195556	1.23E-12	1.53E-11	0.24	0.27	1.43	2.09	DENND6A	DENN/MADD domain containing 6A	chr3	protein_coding
ENSG00000146648.14	-1.6443928	5.52952952	2.10E-08	1.53E-07	4.38	5.26	1.91	2.2	EGFR	epidermal growth factor receptor	chr7	protein_coding
ENSG00000229891.1	1.55501672	0.17133213	0.0001217	0.00048099	0.4	0.35	0.95	1.98	LINC01315	long intergenic non-protein coding RNA 1315	chr22	lincRNA
ENSG00000165029.14	4.20166196	-1.8724796	6.92E-06	3.47E-05	0	0	0.03	0.04	ABCA1	ATP-binding cassette, sub-family A (ABC1), member 1	chr9	protein_coding
ENSG00000243479.3	2.18215117	1.42985665	5.62E-10	5.08E-09	0.8	0.65	4.06	4.76	MNX1-AS1	MNX1 antisense RNA 1 (head to head)	chr7	lincRNA
ENSG00000246263.2	4.8843127	-3.0650858	0.01889873	0.04519557	0	0	0.01	0.08	KB-431C1.4	--	chr8	antisense
ENSG00000226091.6	6.55373997	-1.9945061	1.78E-06	9.85E-06	0	0	0.1	0.15	LINC00937	long intergenic non-protein coding RNA 937	chr12	lincRNA
ENSG00000213799.9	6.57969598	0.15113943	1.73E-20	4.31E-19	0	0.01	0.3	0.67	ZNF845	zinc finger protein 845	chr19	protein_coding
ENSG00000270977.1	5.90476671	-2.4637601	0.00014842	0.00057423	0	0	0.08	0.01	AC015849.16	--	chr17	lincRNA
ENSG00000140481.12	-8.4005264	-0.6004835	6.44E-17	1.21E-15	0.3	0.24	0	0	CCDC33	coiled-coil domain containing 33	chr15	protein_coding
ENSG00000126756.10	-1.3267114	6.99983823	4.56E-06	2.37E-05	192.62	201.47	104.96	104.29	UXT	ubiquitously-expressed, prefoldin-like chaperone	chrX	protein_coding
ENSG00000197714.7	1.96626839	-0.4127838	2.09E-05	9.56E-05	0.11	0.02	0.44	0.24	ZNF460	zinc finger protein 460	chr19	protein_coding
ENSG00000278662.3	1.74924834	-0.4031514	0.00016299	0.00062493	0.02	0.09	0.17	0.34	GOLGA6L10	golgin A6 family-like 10	chr15	protein_coding
ENSG00000214706.9	-1.4862285	6.73561924	3.20E-07	2.00E-06	57.67	48.77	24.81	25.87	IFRD2	interferon-related developmental regulator 2	chr3	protein_coding
ENSG00000183971.5	3.6294486	1.96115955	3.25E-22	9.24E-21	0.37	0.53	8.1	6.73	NPW	neuropeptide W	chr16	protein_coding
ENSG00000198786.2	2.21680291	10.3123705	7.88E-14	1.11E-12	130.49	273.23	1427.89	1053.39	MT-ND5	mitochondrially encoded NADH dehydrogenase 5	chrM	protein_coding
ENSG00000165501.15	-1.233733	3.49347957	3.55E-05	0.00015612	6.96	7.68	3.58	4.73	LRR1	leucine rich repeat protein 1	chr14	protein_coding
ENSG00000182931.8	4.92065931	-3.045493	0.0100455	0.02582742	0	0	0.41	0	WFDC10B	WAP four-disulfide core domain 10B	chr20	protein_coding
ENSG00000174807.3	-5.0197065	-3.1311226	0.0100455	0.02582742	0.06	0.01	0	0	CD248	CD248 molecule, endosialin	chr11	protein_coding
ENSG00000132359.12	-1.6095617	5.85248674	3.86E-08	2.72E-07	10.39	10.08	5.15	3.75	RAP1GAP2	RAP1 GTPase activating protein 2	chr17	protein_coding
ENSG00000110492.14	-1.467684	4.80833974	5.88E-07	3.52E-06	20.45	18.45	10.56	8.13	MDK	midkine (neurite growth-promoting factor 2)	chr11	protein_coding
ENSG00000116016.12	1.07571519	6.86068574	0.00018727	0.00071055	11.52	11.9	30.75	35.15	EPAS1	endothelial PAS domain protein 1	chr2	protein_coding
ENSG00000112701.16	2.70842165	2.1665908	2.44E-15	4.02E-14	0.12	0.14	0.7	1.63	SENP6	SUMO1/sentrin specific peptidase 6	chr6	protein_coding
ENSG00000263753.5	-1.163744	1.88771852	0.00025371	0.00094236	0.93	1.13	0.56	0.66	LINC00667	long intergenic non-protein coding RNA 667	chr18	lincRNA
ENSG00000112299.7	-5.8966026	-2.6351464	0.00026535	0.0009747	0.03	0.08	0	0	VNN1	vanin 1	chr6	protein_coding
ENSG00000184349.11	-1.0484633	4.34286032	0.00034248	0.00123662	4.09	4.19	2.38	2.97	EFNA5	ephrin-A5	chr5	protein_coding
ENSG00000247595.2	1.52693499	-0.9813309	0.00357708	0.01035751	0.11	0.1	0.44	0.36	SPTY2D1-AS	SPTY2D1 antisense RNA 1	chr11	processed_transcript
ENSG00000103528.15	-2.1045543	3.05324228	1.75E-11	1.91E-10	1.51	1.53	0.56	0.38	SYT17	synaptotagmin XVII	chr16	protein_coding
ENSG00000187479.5	-5.1526063	1.23404637	5.12E-28	2.13E-26	2.03	1.07	0.12	0	C11orf96	chromosome 11 open reading frame 96	chr11	protein_coding
ENSG00000279908.1	3.6071738	-0.8462107	7.88E-09	6.12E-08	0.03	0	0.2	0.38	RP11-325I22	--	chr5	TEC
ENSG00000164754.11	1.35346702	4.29152959	4.79E-06	2.47E-05	1.35	1.86	3.82	7.17	RAD21	RAD21 homolog (S. pombe)	chr8	protein_coding
ENSG00000171121.15	1.44509602	-1.0343862	0.00751458	0.02012506	0.02	0.02	0.07	0.09	KCNMB3	potassium channel subfamily M regulatory beta subunit 3	chr3	protein_coding
ENSG00000143315.6	1.23692936	2.99827504	4.77E-05	0.00020439	0.91	0.85	2.88	2.66	PIGM	phosphatidylinositol glycan anchor biosynthesis, class M	chr1	protein_coding
ENSG00000105376.4	-3.0306358	4.87092831	3.62E-22	1.03E-20	15.5	12.61	2.62	1.96	ICAM5	intercellular adhesion molecule 5, telencephalin	chr19	protein_coding
ENSG00000001629.8	1.64741889	1.38937056	1.63E-06	9.09E-06	0.2	0.13	0.63	0.73	ANKIB1	ankyrin repeat and IBR domain containing 1	chr7	protein_coding
ENSG00000211448.10	2.9318221	2.94237692	7.68E-19	1.69E-17	0.22	0.2	1.64	2.69	DIO2	deiodinase, iodothyronine, type II	chr14	protein_coding
ENSG00000251615.3	2.33867061	-1.1603573	6.85E-05	0.00028517	0.01	0.05	0.15	0.26	RP11-774O3	--	chr4	lincRNA
ENSG00000155792.8	-2.2187275	0.16164964	6.19E-08	4.28E-07	0.74	0.39	0.17	0.15	DEPTOR	DEP domain containing MTOR-interacting protein	chr8	protein_coding
ENSG00000183793.12	-1.1888573	2.12415133	0.00016545	0.00063327	2.69	3.73	2.02	1.72	NPIPA5	nuclear pore complex interacting protein family, member A5	chr16	protein_coding
ENSG00000144583.4	-5.5276337	-2.8606046	0.00158008	0.00495521	0	0.06	0	0	4-Mar	membrane-associated ring finger (C3HC4) 4, E3 ubiquitin protein ligase	chr2	protein_coding
ENSG00000161813.19	1.71483279	2.8664871	3.70E-08	2.61E-07	0.36	0.45	1.1	2.45	LARP4	La ribonucleoprotein domain family, member 4	chr12	protein_coding
ENSG00000135821.15	-2.3819801	6.22557405	1.99E-15	3.31E-14	20.09	16.54	4.67	4.7	GLUL	glutamate-ammonia ligase	chr1	protein_coding
ENSG00000162650.14	-1.190373	3.05080726	8.29E-05	0.00034026	4.13	3.89	2.32	2.36	ATXN7L2	ataxin 7-like 2	chr1	protein_coding
ENSG00000048392.10	3.22791422	0.43914846	5.37E-13	6.93E-12	0.03	0.04	0.3	0.65	RRM2B	ribonucleotide reductase M2 B (TP53 inducible)	chr8	protein_coding
ENSG00000182253.13	1.58553412	5.49594978	6.48E-08	4.47E-07	1.74	2.93	8.67	10	SYNM	synemin, intermediate filament protein	chr15	protein_coding
ENSG00000258724.1	-1.243996	2.80549246	4.65E-05	0.00020081	4.46	4.59	2.36	2.73	CTD-2135J3.	--	chr8	protein_coding
ENSG00000151490.12	-3.3736682	2.02395655	3.77E-21	9.95E-20	0.93	0.87	0.09	0.14	PTPRO	protein tyrosine phosphatase, receptor type, O	chr12	protein_coding
ENSG00000108061.10	2.52075606	2.07140639	1.56E-13	2.12E-12	0.18	0.32	1.41	2.42	SHOC2	soc-2 suppressor of clear homolog (C. elegans)	chr10	protein_coding
ENSG00000143702.14	1.91060989	1.21840745	6.80E-08	4.67E-07	0.1	0.1	0.45	0.56	CEP170	centrosomal protein 170kDa	chr1	protein_coding
ENSG00000198920.8	1.04263678	2.69608194	0.00068893	0.00234588	0.54	0.83	1.85	1.91	KIAA0753	KIAA0753	chr17	protein_coding
ENSG00000267892.1	3.26124275	-1.1329125	5.22E-07	3.15E-06	0.03	0.04	0.69	0.26	CTD-2540F1	--	chr19	antisense
ENSG00000146247.13	3.41651122	2.17320257	4.08E-21	1.07E-19	0.06	0.06	0.55	1.12	PHIP	pleckstrin homology domain interacting protein	chr6	protein_coding
ENSG00000104814.11	1.89256461	-0.4612487	5.28E-05	0.00022452	0.13	0.02	0.27	0.49	MAP4K1	mitogen-activated protein kinase kinase kinase kinase 1	chr19	protein_coding
ENSG00000148158.15	1.40153794	1.63182561	2.38E-05	0.000108	0.16	0.19	0.6	0.64	SNX30	sorting nexin family member 30	chr9	protein_coding
ENSG00000197043.12	-1.7392804	7.44821477	2.74E-09	2.27E-08	89.23	71.71	33.03	31.21	ANXA6	annexin A6	chr5	protein_coding
ENSG00000116266.9	3.1117548	1.22392053	1.13E-15	1.92E-14	0.21	0.09	1.11	2.37	STXBP3	syntaxin binding protein 3	chr1	protein_coding
ENSG00000232119.6	-1.1174964	4.34672404	0.00014069	0.00054874	14.12	14.67	9.23	8.42	MCTS1	malignant T cell amplified sequence 1	chrX	protein_coding
ENSG00000010319.5	-6.1640648	-0.6199327	8.97E-17	1.66E-15	0.21	0.2	0	0.01	SEMA3G	sema domain, immunoglobulin domain (Ig), short basic domain, secreted, (semaphorin) 3G	chr3	protein_coding
ENSG00000231290.4	-2.0223862	-1.592139	0.00173462	0.0053947	0.24	0.11	0.09	0.03	APCDD1L-A!	APCDD1L antisense RNA 1 (head to head)	chr20	processed_transcript
ENSG00000168904.13	1.07289959	1.95976237	0.00083498	0.00280261	0.29	0.35	0.91	0.89	LRRC28	leucine rich repeat containing 28	chr15	protein_coding
ENSG00000168348.3	4.45095163	-1.6850792	6.30E-07	3.74E-06	0.01	0	0.17	0.15	INSM2	insulinoma-associated 2	chr14	protein_coding
ENSG00000204344.13	-1.393047	4.31703273	2.36E-06	1.29E-05	13.51	14.38	6.37	7.8	STK19	serine/threonine kinase 19	chr6	protein_coding
ENSG00000162819.10	1.10610163	1.58691469	0.00078702	0.00264978	0.39	0.32	0.8	1.24	BROX	BRO1 domain and CAAX motif containing	chr1	protein_coding
ENSG00000175334.6	-1.1668125	7.50495164	5.12E-05	0.00021823	113.73	133.84	78.53	67.97	BANF1	barrier to autointegration factor 1	chr11	protein_coding
ENSG00000102760.12	-3.0195189	0.15156937	1.23E-11	1.37E-10	1.78	1.48	0.43	0.11	RGCC	regulator of cell cycle	chr13	protein_coding
ENSG00000206417.7	-1.3644697	1.02678226	9.55E-05	0.00038585	0.75	0.61	0.42	0.29	H1FX-AS1	H1FX antisense RNA 1	chr3	antisense
ENSG00000252202.1	2.56014592	-2.3582054	0.00455824	0.01291303	0.28	0.3	3	1.99	Y_RNA	--	chr5	misc_RNA
ENSG00000272644.1	-1.9925689	-1.9312008	0.00967167	0.02519005	0.47	0.75	0.42	0	RP11-33O4.1	--	chr2	lincRNA
ENSG00000102144.12	-1.105681	10.646818	0.00011779	0.00046664	420.15	304.09	206.85	243.2	PGK1	phosphoglycerate kinase 1	chrX	protein_coding
ENSG00000159147.16	1.79972318	3.07157068	6.36E-09	5.02E-08	0.8	1.29	3.44	6.32	DONSON	downstream neighbor of SON	chr21	protein_coding
ENSG00000184545.9	1.51371298	1.94110841	3.12E-06	1.66E-05	0.3	0.4	1.27	1.41	DUSP8	dual specificity phosphatase 8	chr11	protein_coding
ENSG00000148572.13	1.028256	2.30401183	0.00102984	0.0033749	1.01	1.71	2.78	4.64	NRBF2	nuclear receptor binding factor 2	chr10	protein_coding
ENSG00000107518.15	7.32308793	-1.3787208	4.49E-10	4.11E-09	0	0	0.04	0.1	ATRNL1	attractin-like 1	chr10	protein_coding
ENSG00000197696.8	-2.3459098	0.80194564	4.94E-10	4.49E-09	2.98	1.55	0.69	0.51	NMB	neuromedin B	chr15	protein_coding
ENSG00000006704.9	-1.3381269	7.07730711	3.79E-06	1.99E-05	25.33	23.55	13.13	12.62	GTF2IRD1	GTF2I repeat domain containing 1	chr7	protein_coding
ENSG00000156876.9	2.51461358	-0.0590832	5.89E-08	4.08E-07	0.06	0.05	0.16	0.66	SASS6	spindle assembly 6 homolog (C. elegans)	chr1	protein_coding
ENSG00000213599.8	-1.1766539	-0.3659469	0.00653665	0.01776746	0.53	0.2	0.19	0.25	SLX1A-SULT	SLX1A-SULT1A3 readthrough (NMD candidate)	chr16	protein_coding
ENSG00000233452.5	-4.8079387	-3.2306674	0.01889873	0.04519557	0.01	0.02	0	0	STXBP5-AS1	STXBP5 antisense RNA 1	chr6	antisense
ENSG00000101773.15	3.45741856	2.62714695	6.30E-23	1.87E-21	0.16	0.28	1.87	4.66	RBBP8	retinoblastoma binding protein 8	chr18	protein_coding
ENSG00000111300.8	1.69596309	2.25338794	1.43E-07	9.34E-07	0.23	0.37	1.06	1.51	NAA25	N(alpha)-acetyltransferase 25, NatB auxiliary subunit	chr12	protein_coding

ENSG00000263280.1	-2.5285753	-0.2137124	1.88E-08	1.38E-07	2.29	1.15	0.29	0.49	LA16c-325D	--	chr16	lincRNA
ENSG00000128645.12	7.51722729	-1.2186982	3.23E-11	3.41E-10	0	0	0.31	0.44	HOXD1	homeobox D1	chr2	protein_coding
ENSG00000125772.11	2.97227003	-0.3598551	6.42E-09	5.07E-08	0.01	0.04	0.29	0.2	GPCPD1	glycerophosphocholine phosphodiesterase GDE1 homolog (S. cerevisiae)	chr20	protein_coding
ENSG00000269815.1	1.96482321	-1.6099842	0.00345484	0.01003143	0.21	0.11	0.9	0.87	CTD-2278I1	--	chr19	sense_intronic
ENSG00000243660.8	-3.0812805	-1.9686177	0.00025363	0.00094222	0	0.3	0.05	0	ZNF487	zinc finger protein 487	chr10	protein_coding
ENSG00000206140.8	3.47176115	0.05921913	1.88E-12	2.29E-11	0.03	0.07	0.75	0.69	TMEM191C	transmembrane protein 191C	chr22	lincRNA
ENSG00000164530.12	5.04175262	-2.9826774	0.0100455	0.02582742	0	0	0	0.11	PI16	peptidase inhibitor 16	chr6	protein_coding
ENSG00000129422.12	2.33412604	3.07429002	2.21E-13	2.96E-12	0.21	0.38	1.89	2.09	MTUS1	microtubule associated tumor suppressor 1	chr8	protein_coding
ENSG00000169684.12	1.59884125	3.84783828	1.03E-07	6.90E-07	1.49	1.68	5.24	7.61	CHRNA5	cholinergic receptor, nicotinic, alpha 5 (neuronal)	chr15	protein_coding
ENSG00000206557.5	5.84975207	-2.4948111	0.00026535	0.0009747	0	0	0.01	0.04	TRIM71	tripartite motif containing 71, E3 ubiquitin protein ligase	chr3	protein_coding
ENSG00000186566.10	1.0523096	4.3721087	0.00032726	0.00118416	1.76	2.13	5.39	5.33	GPATCH8	G patch domain containing 8	chr17	protein_coding
ENSG00000242247.9	1.05275532	3.59874229	0.00041293	0.00146757	2.45	1.97	4.1	8.24	ARFGAP3	ADP-ribosylation factor GTPase activating protein 3	chr22	protein_coding
ENSG00000104388.13	-1.1542502	4.52236728	7.79E-05	0.00032102	6.48	6.85	3.15	4.86	RAB2A	RAB2A, member RAS oncogene family	chr8	protein_coding
ENSG00000165280.14	-1.245833	9.22473373	1.54E-05	7.23E-05	166.39	145.28	84.08	91.32	VCP	valosin containing protein	chr9	protein_coding
ENSG00000151687.13	5.7784906	-2.5437546	0.00026535	0.0009747	0	0	0.04	0.06	ANKAR	ankyrin and armadillo repeat containing	chr2	protein_coding
ENSG00000127585.10	-1.012285	6.04285639	0.00044684	0.00157619	19.42	19.68	13.51	12.28	FBXL16	F-box and leucine-rich repeat protein 16	chr16	protein_coding
ENSG00000213366.11	-3.1717913	-2.8628854	0.01615662	0.03958166	0.01	0.05	0.01	0	GSTM2	glutathione S-transferase mu 2 (muscle)	chr1	protein_coding
ENSG00000183161.4	-1.0186125	1.57774679	0.002013	0.00616687	0.73	0.77	0.61	0.38	FANCF	Fanconi anemia, complementation group F	chr11	protein_coding
ENSG00000086712.11	1.52773671	2.89381243	7.80E-07	4.57E-06	0.67	0.71	2.16	3.2	TXLNG	taxilin gamma	chrX	protein_coding
ENSG00000280273.1	5.06203518	-2.9716376	0.0100455	0.02582742	0	0	0.08	0.09	AF131216.1	--	chr8	protein_coding
ENSG00000173230.14	2.78292269	4.0517474	9.82E-19	2.15E-17	0.26	0.34	2.07	3.47	GOLGB1	golgin B1	chr3	protein_coding
ENSG00000204634.11	-2.0322511	5.21413584	8.60E-12	9.74E-11	11.63	13.79	3.71	4.58	TBC1D8	TBC1 domain family, member 8 (with GRAM domain)	chr2	protein_coding
ENSG00000155850.7	1.06795654	0.69251661	0.0039373	0.01130629	0.07	0.13	0.15	0.4	SLC26A2	solute carrier family 26 (anion exchanger), member 2	chr5	protein_coding
ENSG00000122545.16	1.92540894	3.75415579	2.76E-10	2.61E-09	0.83	1.22	3.71	6.72	7-Sep	septin 7	chr7	protein_coding
ENSG00000274516.1	5.21249443	-2.8887793	0.00538165	0.01488851	0	0	0.09	0.08	FAM74A6	family with sequence similarity 74, member A6	chr9	lincRNA
ENSG00000109466.12	1.42296164	1.87637435	1.18E-05	5.64E-05	0.35	0.54	1.47	1.71	KLHL2	kelch-like family member 2	chr4	protein_coding
ENSG00000135457.8	-1.0450447	4.93000241	0.00032137	0.00116429	8.68	9.49	5.52	6.21	TFCP2	transcription factor CP2	chr12	protein_coding
ENSG00000147036.10	-8.0055183	-0.9647791	7.69E-14	1.09E-12	0.09	0.06	0	0	LANCL3	LanC lantibiotic synthetase component C-like 3 (bacterial)	chrX	protein_coding
ENSG00000229739.2	-5.2054745	-3.0371681	0.0100455	0.02582742	0.28	0	0	0	RP11-295K2.	--	chr1	antisense
ENSG00000281344.1	1.62554584	2.08224641	5.34E-07	3.22E-06	0.01	0.01	0.04	0.03	HELLPAR	HELLP associated long non-coding RNA	chr12	macro_lncRNA
ENSG00000153094.20	1.04613817	3.58202745	0.00043426	0.00153724	0.99	1.07	2.59	3.1	BCL2L11	BCL2-like 11 (apoptosis facilitator)	chr2	protein_coding
ENSG00000184185.8	-5.1179676	4.76573698	1.60E-47	1.94E-45	8.11	7.14	0.24	0.34	KCNJ12	potassium channel, inwardly rectifying subfamily J, member 12	chr17	protein_coding
ENSG00000255441.1	5.35906543	-2.8049519	0.00290522	0.00856312	0	0	0.25	0.08	CTD-2616J1	--	chr19	antisense
ENSG00000074621.12	-1.1050406	1.90010545	0.00053761	0.00186993	0.46	0.78	0.3	0.46	SLC24A1	solute carrier family 24 (sodium/potassium/calcium exchanger), member 1	chr15	protein_coding
ENSG00000157193.13	-1.2928724	5.77984677	8.47E-06	4.17E-05	7.53	8.61	3.94	4.86	LRP8	low density lipoprotein receptor-related protein 8, apolipoprotein e receptor	chr1	protein_coding
ENSG00000205220.10	1.70505146	3.62708334	1.86E-08	1.37E-07	4.24	3.4	17.28	15.92	PSMB10	proteasome (prosome, macropain) subunit, beta type, 10	chr16	protein_coding
ENSG00000197971.13	-1.350896	1.0532188	8.06E-05	0.00033163	0.35	0.3	0.12	0.22	MBP	myelin basic protein	chr18	protein_coding
ENSG00000106299.7	1.3590871	2.01206643	2.58E-05	0.00011607	0.42	0.39	1	1.77	WASL	Wiskott-Aldrich syndrome-like	chr7	protein_coding
ENSG00000100346.16	8.52169697	-0.344016	4.85E-17	9.21E-16	0	0	0.17	0.11	CACNA1I	calcium channel, voltage-dependent, T type, alpha 1I subunit	chr22	protein_coding
ENSG00000169750.7	-2.0766798	5.27042178	3.31E-12	3.92E-11	50.64	41.94	17.02	12.14	RAC3	ras-related C3 botulinum toxin substrate 3 (rho family, small GTP binding protein Rac3)	chr17	protein_coding
ENSG00000214049.6	8.60945082	3.82514403	8.00E-66	2.59E-63	0.03	0.01	14.25	11.98	UCA1	urothelial cancer associated 1 (non-protein coding)	chr19	processed_transcript
ENSG00000167920.7	-1.685697	1.69098427	3.16E-07	1.97E-06	1.89	1.82	0.62	0.92	TMEM99	transmembrane protein 99	chr17	protein_coding
ENSG00000144645.12	-1.6556974	4.6158398	2.38E-08	1.73E-07	4.36	4.77	1.87	1.99	OSBPL10	oxysterol binding protein-like 10	chr3	protein_coding
ENSG00000279265.1	5.21752153	-2.8860363	0.00538165	0.01488851	0	0	0.42	0.24	AC000123.3	--	chr7	TEC
ENSG00000244731.6	-2.4968279	-2.7137396	0.00943894	0.02469931	0	0.05	0	0.01	C4A	complement component 4A (Rodgers blood group)	chr6	protein_coding
ENSG00000157326.17	-1.1466697	6.22840233	7.20E-05	0.00029802	39.4	33.07	20.94	22.75	DHRS4	dehydrogenase/reductase (SDR family) member 4	chr14	protein_coding
ENSG00000204950.3	1.27044485	-1.0635217	0.01851475	0.04482283	0.17	0	0.33	0.23	LRRC10B	leucine rich repeat containing 10B	chr11	protein_coding
ENSG00000269846.1	2.96839882	-2.0907488	0.00067192	0.00229154	0.04	0	0.02	0.41	RP4-621N11	--	chr20	antisense
ENSG00000146678.8	-10.75964	1.70687061	4.64E-43	4.21E-41	2.6	3.82	0	0	IGFBP1	insulin-like growth factor binding protein 1	chr7	protein_coding
ENSG00000178537.8	-1.5262975	4.6595694	2.34E-07	1.48E-06	16.07	16.16	7.14	7.78	SLC25A20	solute carrier family 25 (carnitine/acylcarnitine translocase), member 20	chr3	protein_coding
ENSG00000280102.2	3.31469784	-0.4056931	1.04E-09	9.04E-09	1	1.42	25.63	7.49	AL627171.2	--	chr14	miRNA
ENSG00000112039.3	-1.8334526	5.10411284	5.88E-10	5.29E-09	16.86	17.19	6.26	6.47	FANCE	Fanconi anemia, complementation group E	chr6	protein_coding
ENSG00000136444.8	-2.1147646	4.58801513	2.36E-12	2.86E-11	12.95	12.06	4.07	3.62	RSAD1	radical S-adenosyl methionine domain containing 1	chr17	protein_coding
ENSG00000188820.11	1.86552249	0.04458994	9.04E-06	4.42E-05	0.56	0	1.43	1.39	FAM26F	family with sequence similarity 26, member F	chr6	protein_coding
ENSG00000135249.6	1.09343173	0.79931112	0.00248499	0.00748746	0.23	0.34	0.43	1.21	RINT1	RAD50 interactor 1	chr7	protein_coding
ENSG00000110429.12	1.15367389	2.43191179	0.00020295	0.0007653	0.27	0.2	0.49	0.92	FBXO3	F-box protein 3	chr11	protein_coding
ENSG00000152315.4	6.51968671	-2.0237618	1.78E-06	9.85E-06	0	0	0.18	0.12	KCNK13	potassium channel, two pore domain subfamily K, member 13	chr14	protein_coding
ENSG00000161048.10	1.8861165	0.41592001	2.55E-06	1.38E-05	0.06	0.09	0.34	0.42	NAPEPLD	N-acyl phosphatidylethanolamine phospholipase D	chr7	protein_coding
ENSG00000178573.6	2.18147066	-0.4165742	4.71E-06	2.44E-05	0.04	0.02	0.14	0.2	MAF	v-maf avian musculoaponeurotic fibrosarcoma oncogene homolog	chr16	protein_coding
ENSG00000078177.12	1.7672999	-0.397568	0.00016299	0.00062493	0.04	0.01	0.07	0.16	N4BP2	NEDD4 binding protein 2	chr4	protein_coding
ENSG00000182986.11	-4.4637847	0.37704629	9.34E-20	2.20E-18	0.26	0.32	0.01	0.02	ZNF320	zinc finger protein 320	chr19	protein_coding
ENSG00000011201.9	-7.5667583	-1.3550377	3.23E-11	3.41E-10	0.11	0.07	0	0	KAL1	--	chrX	protein_coding
ENSG00000119965.11	1.24724361	-0.0399288	0.00245879	0.00741317	0.14	0.21	0.31	0.78	C10orf88	chromosome 10 open reading frame 88	chr10	protein_coding
ENSG00000198373.11	1.40759909	6.75803292	1.24E-06	7.02E-06	7.12	7.49	26.4	25.2	WWP2	WW domain containing E3 ubiquitin protein ligase 2	chr16	protein_coding
ENSG00000067900.6	4.19491203	2.85866418	1.69E-30	8.00E-29	0.04	0.09	0.92	2.18	ROCK1	Rho-associated, coiled-coil containing protein kinase 1	chr18	protein_coding
ENSG00000104870.11	3.20011245	2.1847709	1.61E-19	3.74E-18	0.36	0.33	4.32	4.17	FCGRT	Fc fragment of IgG, receptor, transporter, alpha	chr19	protein_coding
ENSG00000173226.15	1.78491844	2.9379967	1.01E-08	7.73E-08	1.08	1.04	3.89	5.9	IQCB1	IQ motif containing B1	chr3	protein_coding
ENSG00000279660.1	5.47921296	-2.7333202	0.00158008	0.00495521	0	0	0.19	0.08	RP11-849N1	--	chr17	TEC
ENSG00000084676.14	2.574661	2.36441782	1.43E-14	2.19E-13	0.15	0.16	1.09	1.39	NCOA1	nuclear receptor coactivator 1	chr2	protein_coding
ENSG00000100138.12	-1.304577	8.08171143	6.26E-06	3.16E-05	178.83	160.38	91.64	91.4	NHP2L1	NHP2 non-histone chromosome protein 2-like 1 (S. cerevisiae)	chr22	protein_coding
ENSG00000006047.11	2.96547183	2.34099923	7.07E-18	1.44E-16	0.56	0.59	7.17	4.71	YBX2	Y box binding protein 2	chr17	protein_coding
ENSG00000138246.14	2.97540187	2.56955826	1.63E-18	3.51E-17	0.13	0.14	1.14	1.76	DNAJC13	DnaJ (Hsp40) homolog, subfamily C, member 13	chr3	protein_coding
ENSG00000085511.18	1.56356021	3.40682243	2.53E-07	1.60E-06	0.71	0.84	2.88	3.24	MAP3K4	mitogen-activated protein kinase kinase kinase 4	chr6	protein_coding
ENSG00000197565.14	-6.7718548	6.32874581	3.37E-72	1.52E-69	19.29	18.29	0.13	0.33	COL4A6	collagen, type IV, alpha 6	chrX	protein_coding
ENSG00000166707.9	2.69575167	-1.555445	0.00013983	0.00054609	0.02	0.02	0.14	0.17	ZCCHC18	zinc finger, CCHC domain containing 18	chrX	protein_coding
ENSG00000223776.5	2.02961081	-1.5616899	0.00244913	0.00738591	0.04	0.08	0.18	0.45	LGALS8-AS1	LGALS8 antisense RNA 1	chr1	antisense
ENSG00000167107.11	1.45225506	5.5888845	6.61E-07	3.91E-06	8.76	9.25	33.99	31.6	ACSF2	acyl-CoA synthetase family member 2	chr17	protein_coding
ENSG00000095321.15	-6.6315419	5.61830393	7.10E-68	2.50E-65	24.78	23.8	0.31	0.34	CRAT	carnitine O-acetyltransferase	chr9	protein_coding
ENSG00000126500.3	-3.3828863	-0.0247563	7.48E-13	9.50E-12	0.3	0.42	0.06	0.03	FLRT1	fibronectin leucine rich transmembrane protein 1	chr11	protein_coding
ENSG00000267110.1	1.71403409	0.52675871	7.76E-06	3.86E-05	0.44	0.61	2.14	2.46	CTD-2587H2	--	chr19	protein_coding
ENSG00000168955.3	5.66409976	-2.616209	0.00086564	0.00288128	0	0	0.03	0.14	TM4SF20	transmembrane 4 L six family member 20	chr2	protein_coding
ENSG00000265801.1	5.47433081	-2.7360128	0.00158008	0.00495521	0	0	0.52	0.32	RP11-720N1	--	chr17	sense_intronic
ENSG00000234289.5	2.62505514	2.83027442	8.59E-16	1.47E-14	4.07	2.77	27.65	28.89	H2BFS	H2B histone family, member S (pseudogene)	chr21	protein_coding
ENSG00000132832.8	2.86232479	0.21431335	2.01E-10	1.93E-09	0.21	0.08	1.36	1.5	LINC01260	long intergenic non-protein coding RNA 1260	chr20	antisense
ENSG00000268592.3	4.86091664	1.44412052	2.43E-27	9.87E-26	0.13	0.09	5.22	3.76	RAET1E-AS1	RAET1E antisense RNA 1	chr6	antisense
ENSG00000162849.14	5.21752153	-2.8860363	0.00538165	0.01488851	0	0	0.01	0.01	KIF26B	kinesin family member 26B	chr1	protein_coding
ENSG00000185215.7	-1.0370428	8.60999538	0.00030175	0.00109847	94.53	87.06	58.8	59.15	TNFAIP2	tumor necrosis factor, alpha-induced protein 2	chr14	protein_coding
ENSG00000130176.6	-2.528518	-2.3245285	0.00854801	0.02266235	0.08	0.13	0.05	0	CNN1	calponin 1, basic, smooth muscle	chr19	protein_coding
ENSG00000074966.9	4.88946445	-3.0622981	0.01889873	0.04519557	0	0	0.02	0.06	TXK	TXK tyrosine kinase	chr4	protein_coding
ENSG00000204335.3	3.73595425	3.51714223	3.66E-28	1.54E-26	0.72	0.53	11.05	11.32	SP5	Sp5 transcription factor	chr2	protein_coding
ENSG00000011258.14	2.21962245	0.53862451	2.42E-08	1.75E-07	0.05	0.09	0.3	0.56	MBTD1	mbt domain containing 1	chr17	protein_coding
ENSG00000265257.4	5.06713725	-2.9688687	0.0100455	0.02582742	0	0	0.18	0.12	RP11-21J18.	--	chr18	processed_transcript
ENSG00000092295.10	-2.156274	1.53796783	4.71E-10	4.30E-09	1.33	1.42	0.52	0.3	TGM1	transglutaminase 1	chr14	protein_coding
ENSG00000227953.5	6.16882782	-0.2291856	8.15E-17	1.52E-15	0.01	0	0.55	0.65	LINC01341	long intergenic non-protein coding RNA 1341	chr1	processed_transcript
ENSG00000180198.14	-1.2882984	8.1767375	8.07E-06	4.00E-05	102.79	103.17	54.28	58.17	RCC1	regulator of chromosome condensation 1	chr1	protein_coding
ENSG00000181396.11	1.16835328	6.02800623	5.40E-05	0.00022876	7.35	7.09	21.23	22.03	OGFOD3	2-oxoglutarate and iron-dependent oxygenase domain containing 3	chr17	protein_coding
ENSG00000196411.8	-1.0127991	8.0368466	0.00041675	0.00148027	66.91	63.75	41.1	45.29	EPHB4	EPH receptor B4	chr7	protein_coding
ENSG00000169087.9	1.14355753	2.8009179	0.00018749	0.00071119	1.71	1.84	4.44	6.05	HSPBAP1	HSPB (heat shock 27kDa) associated protein 1	chr3	protein_coding
ENSG00000272668.1	-2.6326992	0.84303946	2.57E-12	3.09E-11	2.59	4.22	0.31	1.12	RP11-190A1	--	chr1	antisense
ENSG00000148798.8	2.1330591	5.1961957	1.19E-12	1.47E-11	3.09	3.72	17.4	22.48	INA	internexin neuronal intermediate filament protein, alpha	chr10	protein_coding
ENSG00000157613.9	-4.5722353	2.26499806	6.22E-32	3.14E-30	2.65	2.92	0.19	0.12	CREB3L1	cAMP responsive element binding protein 3-like 1	chr11	protein_coding
ENSG00000198947.13	-5.9051356	1.94951402	4.46E-38	3.03E-36	0.33	0.43	0.01	0.01	DMD	dystrophin	chrX	protein_coding
ENSG00000178343.4	-5.7793342	-2.7087353	0.00047761	0.00167191	0.11	0.06	0	0	SHISA3	shisa family member 3	chr4	protein_coding
ENSG00000234614.1	3.19082937	0.3055782	3.97E-12	4.66E-11	0.37	0.34	3.36	5.38	AL450992.2	--	chr1	antisense
ENSG00000135709.11	1.36286532	4.05589586	4.41E-06	2.29E-05	0.89	0.84	3.02	2.9	KIAA0513	KIAA0513	chr16	protein_coding
ENSG00000144331.17	-7.3134625	-1.5742106	1.11E-09	9.61E-09	0.06	0.18	0	0	ZNF385B	zinc finger protein 385B	chr2	protein_coding
ENSG00000206262.7	1.71385997	2.40999724	7.41E-08	5.06E-07	0.73	0.38	2.71	2.16	FOXL2NB	FOXL2 neighbor	chr3	protein_coding
ENSG00000051341.12	2.81394679	2.03306466	6.67E-16	1.15E-14	0.07	0.11	0.74	0.92	POLQ	polymerase (DNA directed), theta	chr3	protein_coding
ENSG00000159885.12	1.93091332	1.0235501	1.32E-07	8.72E-07	0.23	0.46	1.12	2.39	ZNF222	zinc finger protein 222	chr19	protein_coding
ENSG00000272841.1	2.88439247	-1.7341846	0.00034876	0.00125664	0.04	0	0.09	0.32	RP3-428L16.	--	chr6	antisense
ENSG00000117983.16	-6.0956928	-2.503296	8.36E-05	0.00034052	0.01	0.01	0	0	MUC5B	mucin 5B, oligomeric mucus/gel-forming	chr11	protein_coding
ENSG00000132356.10	1.99035934	3.2244716	1.55E-10	1.51E-09	0.45	0.58	2.09	3.37	PRKAA1	protein kinase, AMP-activated, alpha 1 catalytic subunit	chr5	protein_coding
ENSG00000255367.1	6.3839906	-2.1220471	5.19E-06	2.66E-05	0	0	0.29	0.39	RP13-726E6.	--	chr11	lincRNA
ENSG00000109680.9	1.3063983	-1.0265019	0.01467747	0.03636299	0.02	0.15	0.11	0.45	TBC1D19	TBC1 domain family, member 19	chr4	protein_coding
ENSG00000196220.14	3.4174246	-0.9966655	8.81E-08	5.94E-07	0	0.01	0.06	0.1	SRGAP3	SLIT-ROBO Rho GTPase activating protein 3	chr3	protein_coding
ENSG00000014824.12	1.62865234	2.36544159	3.09E-07	1.93E-06	0.29	0.35	1.04	1.62	SLC30A9	solute carrier family 30 (zinc transporter), member 9	chr4	protein_coding
ENSG00000202252.1	3.96579555	-0.9048795	2.54E-09	2.11E-08	0.3	0.64	17.84	3.4	SNORD14C	small nucleolar RNA, C/D box 14C	chr11	snoRNA
ENSG00000106052.12	1.81842851	3.48004224	2.74E-09	2.27E-08	1.05	1.06	2.73	7.31	TAX1BP1	Tax1 (human T-cell leukemia virus type I) binding protein 1	chr7	protein_coding
ENSG00000263212.2	-1.5826921	0.73144043	1.52E-05	7.16E-05	2.88	4.61	2.42	0.93	LA16c-306E	--	chr16	processed_transcript
ENSG00000273186.1	-2.2196295	3.0991157	1.52E-12	1.87E-11	31.39	24.32	8.22	7.72	RP11-339B2	--	chr9	sense_intronic
ENSG00000110090.11	-1.8714106	8.32559325	1.79E-10	1.73E-09	70.17	70.83	24.91	26.46	CPT1A	carnitine palmitoyltransferase 1A (liver)	chr11	protein_coding
ENSG00000145725.18	2.55446547	0.27861009	2.15E-09	1.80E-08	0.02	0.01	0.13	0.14	PPIP5K2	diphosphoinositol pentakisphosphate kinase 2	chr5	protein_coding
ENSG00000125650.4	1.16783232	2.56537688	0.00015794	0.00060763	0.67	0.82	2.63	1.81	PSPN	persephin	chr19	protein_coding
ENSG00000197191.4	-2.2421277	3.22750906	7.80E-13	9.88E-12	10.37	8.89	3.11	2.31	CYSRT1	cysteine-rich tail protein 1	chr9	protein_coding
ENSG00000119314.14	2.81825889	2.52919688	6.89E-17	1.29E-15	0.08	0.19	0.87	1.74	PTBP3	polypyrimidine tract binding protein 3	chr9	protein_coding
ENSG00000244239.1	-6.0932521	-2.5045083	8.36E-05	0.00034052	0.81	0.13	0	0	AC007009.1	--	chr7	antisense
ENSG00000214772.2	4.88946445	-3.0622981	0.01889873	0.04519557	0	0	0.1	0.25	RP11-174G6	--	chr12	antisense
ENSG00000117877.9	-1.398723	7.52851347	1.37E-06	7.73E-06	60.03	71.76	33.92	32.58	CD3EAP	CD3e molecule, epsilon associated protein	chr19	protein_coding
ENSG00000272447.1	1.69165921	2.18891137	1.48E-07	9.71E-07	0.99	1.02	4.39	4.26	RP11-182L2	--	chr10	lincRNA
ENSG00000132481.5	-1.3034274	5.76158036	7.23E-06	3.61E-05	30.02	24.53	14.89	14.57	TRIM47	tripartite motif containing 47	chr17	protein_coding
ENSG00000110076.17	1.53337663	-1.5571688	0.01700209	0.04149604	0.01	0.02	0.03	0.08	NRXN2	neurexin 2	chr11	protein_coding
ENSG00000180815.13	-3.5247071	-1.33821	1.38E-07	9.08E-07	0.14	0.08	0	0.02	MAP3K15	mitogen-activated protein kinase kinase kinase 15	chrX	protein_coding
ENSG00000274849.1	6.26218212	-0.1521842	1.14E-17	2.27E-16	0.06	0	4.99	2.67	RP11-49I11.	--	chr18	sense_intronic
ENSG00000173041.10	2.88364136	-0.2928352	7.67E-09	5.97E-08	0.03	0.02	0.19	0.38	ZNF680	zinc finger protein 680	chr7	protein_coding
ENSG00000137804.11	1.5047635	5.29369571	2.84E-07	1.78E-06	5.66	7.82	20.02	31.13	NUSAP1	nucleolar and spindle associated protein 1	chr15	protein_coding
ENSG00000135063.16	-2.4169261	-1.1742178	2.56E-05	0.00011557	0.27	0.11	0.08	0.02	FAM189A2	family with sequence similarity 189, member A2	chr9	protein_coding
ENSG00000115993.10	1.14995696	3.04725755	0.00015015	0.00058036	0.64	0.58	1.43	2.2	TRAK2	trafficking protein, kinesin binding 2	chr2	protein_coding
ENSG00000007372.19	-5.2594843	2.0381742	7.29E-35	4.23E-33	0.83	0.79	0.04	0.02	PAX6	paired box 6	chr11	protein_coding
ENSG00000135414.8	-1.4833258	5.6041424	3.80E-07	2.34E-06	6.4	7	3.26	3.12	GDF11	growth differentiation factor 11	chr12	protein_coding
ENSG00000248109.1	4.16570314	-0.3736166	1.60E-12	1.97E-11	0	0.5	5.53	6.75	CTC-295J13.	--	chr5	lincRNA
ENSG00000152683.13	1.38290763	1.83825223	2.12E-05	9.68E-05	0.39	0.2	0.81	1.27	SLC30A6	solute carrier family 30 (zinc transporter), member 6	chr2	protein_coding
ENSG00000269107.1	-5.0243296	-3.1290017	0.0100455	0.02582742	0.05	0.3	0	0	RP11-15H20	--	chr19	lincRNA
ENSG00000130779.18	3.79442309	4.37021608	1.96E-30	9.19E-29	0.24	0.32	3.99	6.46	CLIP1	CAP-GLY domain containing linker protein 1	chr12	protein_coding
ENSG00000124104.17	1.46237979	5.19783642	5.97E-07	3.57E-06	5.66	5.62	21.61	19.75	SNX21	sorting nexin family member 21	chr20	protein_coding
ENSG00000093010.10	-1.6692812	7.3552691	1.08E-08	8.21E-08	45.65	45.89	19.47	18.85	COMT	catechol-O-methyltransferase	chr22	protein_coding
ENSG00000141971.11	-1.9195283	5.98475456	8.12E-11	8.20E-10	36.07	27.49	12.33	10.02	MVB12A	multivesicular body subunit 12A	chr19	protein_coding
ENSG00000163428.3	1.40138368	2.43796929	8.23E-06	4.06E-05	0.3	0.3	0.99	1.12	LRRC58	leucine rich repeat containing 58	chr3	protein_coding
ENSG00000274421.1	2.7375192	-2.2484841	0.00174589	0.00541921	0.04	0	0.31	0.13	RP11-386J2	--	chr9	sense_intronic
ENSG00000163082.9	-2.0694583	-1.8897078	0.00303791	0.00892022	0.05	0.13	0.05	0.01	SGPP2	sphingosine-1-phosphate phosphatase 2	chr2	protein_coding
ENSG00000150776.16	1.16289215	2.41889125	0.00020141	0.00076048	0.46	0.88	1.54	2.46	C11orf57	chromosome 11 open reading frame 57	chr11	protein_coding
ENSG00000143162.7	1.74408784	4.0000744	6.86E-09	5.39E-08	2.64	3.36	10.9	15.97	CREG1	cellular repressor of E1A-stimulated genes 1	chr1	protein_coding
ENSG00000130487.5	-6.8901016	0.89483985	4.89E-31	2.36E-29	0.7	1.31	0.02	0	KLHDC7B	kelch domain containing 7B	chr22	protein_coding
ENSG00000154265.14	4.60359269	1.56672214	2.07E-26	7.84E-25	0.03	0.01	0.4	0.88	ABCA5	ATP-binding cassette, sub-family A (ABC1), member 5	chr17	protein_coding
ENSG00000183114.7	2.32374491	2.83805786	4.45E-13	5.77E-12	0.8	0.67	5.29	4.54	FAM43B	family with sequence similarity 43, member B	chr1	protein_coding
ENSG00000184481.15	1.19828238	5.75791934	3.58E-05	0.00015743	7.93	8.16	24.62	24.56	FOXO4	forkhead box O4	chrX	protein_coding
ENSG00000161940.9	-5.7784823	-2.7091468	0.00047761	0.00167191	0.07	0.02	0	0	BCL6B	B-cell CLL/lymphoma 6, member B	chr17	protein_coding
ENSG00000135218.16	7.00082201	-1.6419702	1.87E-08	1.38E-07	0	0	0.07	0.22	CD36	CD36 molecule (thrombospondin receptor)	chr7	protein_coding
ENSG00000259498.1	-2.006498	-1.9445928	0.00445943	0.01266454	0.14	0.02	0.02	0.03	RP11-244F1	--	chr15	antisense
ENSG00000272374.1	2.55917239	-1.6547568	0.00044263	0.00156293	0.03	0	0.08	0.13	RP3-329A5.8	--	chr6	lincRNA
ENSG00000177169.8	-1.3255668	6.30578408	4.85E-06	2.50E-05	18.65	15.8	9.01	9.33	ULK1	unc-51 like autophagy activating kinase 1	chr12	protein_coding
ENSG00000271983.1	4.92065931	-3.045493	0.0100455	0.02582742	0	0	0.52	0	RP11-28H5.2	--	chr15	lincRNA
ENSG00000151135.8	2.33078513	1.62881773	2.92E-11	3.11E-10	0.19	0.2	0.7	1.92	TMEM263	transmembrane protein 263	chr12	protein_coding
ENSG00000265106.1	5.66878161	-2.6135853	0.00086564	0.00288128	0	0	1.12	3.48	Z93241.1	--	chr22	miRNA
ENSG00000116791.12	-1.5045393	0.83366411	1.93E-05	8.88E-05	0.68	1.14	0.31	0.54	CRYZ	crystallin, zeta (quinone reductase)	chr1	protein_coding
ENSG00000105141.5	11.1777106	2.16940094	2.42E-47	2.92E-45	0	0	3.46	2.34	CASP14	caspase 14, apoptosis-related cysteine peptidase	chr19	protein_coding
ENSG00000204220.8	-1.2738941	6.67050498	1.06E-05	5.14E-05	45.98	43.49	23.66	25.7	PFDN6	prefoldin subunit 6	chr6	protein_coding
ENSG00000156256.13	3.13178071	1.02834067	6.53E-15	1.04E-13	0.1	0.11	0.56	1.92	USP16	ubiquitin specific peptidase 16	chr21	protein_coding
ENSG00000103495.12	-1.7512912	9.73100412	2.02E-09	1.70E-08	315.92	344.98	136.35	124.76	MAZ	MYC-associated zinc finger protein (purine-binding transcription factor)	chr16	protein_coding
ENSG00000226009.1	-6.7650535	-2.0164922	3.73E-07	2.30E-06	0.5	0.27	0	0	KCNIP2-AS1	KCNIP2 antisense RNA 1	chr10	antisense
ENSG00000221926.10	-2.7505189	4.45375184	9.42E-19	2.06E-17	7.92	6.12	1.68	1.1	TRIM16	tripartite motif containing 16	chr17	protein_coding
ENSG00000281350.1	-5.2063823	-3.0367468	0.00538165	0.01488851	0.71	0.11	0	0	Metazoa_SRI	--	chr1	misc_RNA
ENSG00000144747.13	5.71918816	1.57244557	9.02E-32	4.52E-30	0	0.02	0.48	1.29	TMF1	TATA element modulatory factor 1	chr3	protein_coding
ENSG00000112658.7	-1.123412	6.46777654	9.86E-05	0.00039739	23.56	23.18	13.9	14.7	SRF	serum response factor (c-fos serum response element-binding transcription factor)	chr6	protein_coding
ENSG00000232480.1	-3.1521803	-2.3269279	0.00174589	0.00541921	0.43	0.36	0.06	0.05	TGFB2-AS1	TGFB2 antisense RNA 1 (head to head)	chr1	antisense
ENSG00000108055.9	2.86131973	3.87251654	2.33E-19	5.35E-18	0.59	0.8	4.46	9.04	SMC3	structural maintenance of chromosomes 3	chr10	protein_coding
ENSG00000221972.3	-7.8653715	3.32624299	1.51E-60	3.82E-58	7.68	11.79	0.08	0.03	C3orf36	chromosome 3 open reading frame 36	chr3	protein_coding
ENSG00000228775.6	2.86228417	-0.4470307	4.15E-08	2.91E-07	0.03	0.04	0.34	0.43	WEE2-AS1	WEE2 antisense RNA 1	chr7	antisense
ENSG00000197045.11	2.76630717	0.45625058	6.50E-11	6.66E-10	0.05	0.02	0.18	0.51	GMFB	glia maturation factor, beta	chr14	protein_coding
ENSG00000268362.4	-4.0530951	-1.6931074	1.57E-07	1.02E-06	0.27	0.14	0	0.03	CTD-2017D1	--	chr19	lincRNA
ENSG00000213967.9	-2.8231575	-1.5828948	6.51E-05	0.00027249	0.07	0.06	0.03	0	ZNF726	zinc finger protein 726	chr19	protein_coding
ENSG00000197852.9	1.74392253	3.11919113	1.58E-08	1.17E-07	0.49	0.56	2.35	2.32	FAM212B	family with sequence similarity 212, member B	chr1	protein_coding

ENSG00000257139.1	5.07225195	-2.9660962	0.0100455	0.02582742	0	0	0.33	0.14	RP11-320P7.	--	chr12	lincRNA
ENSG00000100583.4	2.10924066	0.13079017	8.55E-07	4.98E-06	0.06	0.17	0.42	0.93	SAMD15	sterile alpha motif domain containing 15	chr14	protein_coding
ENSG00000172175.11	2.07435567	1.30037877	5.05E-09	4.03E-08	0.06	0.1	0.26	0.62	MALT1	MALT1 paracaspase	chr18	protein_coding
ENSG00000166004.13	2.52765449	2.22956944	7.00E-14	9.95E-13	0.09	0.18	0.55	1.54	CEP295	centrosomal protein 295kDa	chr11	protein_coding
ENSG00000162551.12	-5.2109276	-3.0346387	0.00538165	0.01488851	0.02	0.06	0	0	ALPL	alkaline phosphatase, liver/bone/kidney	chr1	protein_coding
ENSG00000213190.3	-1.4617668	1.08401584	1.93E-05	8.88E-05	0.66	0.56	0.11	0.47	MLLT11	myeloid/lymphoid or mixed-lineage leukemia (trithorax homolog, Drosophila); translocated to, 11	chr1	protein_coding
ENSG00000231595.1	3.8712652	-2.1134597	8.40E-05	0.0003415	0	0.02	0.23	0.19	AC005224.2	--	chr17	lincRNA
ENSG00000138829.9	-10.402474	5.60174196	2.96E-96	5.07E-93	5.64	6.88	0.01	0.01	FBN2	fibrillin 2	chr5	protein_coding
ENSG00000253846.2	-1.5390452	-0.153043	0.00022247	0.00083409	0.08	0.23	0.08	0.06	PCDHGA10	protocadherin gamma subfamily A, 10	chr5	protein_coding
ENSG00000152952.10	2.74007665	3.7417424	5.82E-18	1.19E-16	0.67	0.59	3.08	8.24	PLOD2	procollagen-lysine, 2-oxoglutarate 5-dioxygenase 2	chr3	protein_coding
ENSG00000204613.9	-6.3611661	-2.3196166	8.95E-06	4.38E-05	0.03	0.11	0	0	TRIM10	tripartite motif containing 10	chr6	protein_coding
ENSG00000145919.9	-1.3252528	6.72536764	4.73E-06	2.45E-05	67.98	89.01	38.74	44.77	BOD1	biorientation of chromosomes in cell division 1	chr5	protein_coding
ENSG00000197903.7	2.39324606	5.29117661	2.83E-15	4.64E-14	27.45	25.59	181.6	190.05	HIST1H2BK	histone cluster 1, H2bk	chr6	protein_coding
ENSG00000223343.1	1.84598459	-0.2688166	3.98E-05	0.00017345	0.31	0.36	1.6	1.64	RP13-131K1!	--	chr3	antisense
ENSG00000179580.8	-2.7451228	-2.5779072	0.003237	0.00945156	0.33	0	0	0.06	RNF151	ring finger protein 151	chr16	protein_coding
ENSG00000171533.10	11.4119013	2.39947592	1.41E-50	1.95E-48	0	0	1.82	1.71	MAP6	microtubule-associated protein 6	chr11	protein_coding
ENSG00000123124.12	1.2201065	0.8664561	0.00064136	0.0021989	0.1	0.27	0.35	0.77	WWP1	WW domain containing E3 ubiquitin protein ligase 1	chr8	protein_coding
ENSG00000166451.12	1.5223568	3.46666668	5.00E-07	3.03E-06	1.25	1.65	4.37	6.79	CENPN	centromere protein N	chr16	protein_coding
ENSG00000181222.13	1.05388262	9.07052887	0.00024053	0.00089757	36.56	44.13	117.07	105.61	POLR2A	polymerase (RNA) II (DNA directed) polypeptide A, 220kDa	chr17	protein_coding
ENSG00000120664.9	5.58023914	-2.6705827	0.00086564	0.00288128	0	0	0.27	0.27	SPG20-AS1	SPG20 antisense RNA 1	chr13	antisense
ENSG00000266835.4	3.85546582	-2.1235498	8.40E-05	0.0003415	0.04	0	0.35	0.64	LINC01540	--	chr18	lincRNA
ENSG00000126821.7	2.6916811	1.95372508	1.02E-14	1.59E-13	0.22	0.27	1.27	3	SGPP1	sphingosine-1-phosphate phosphatase 1	chr14	protein_coding
ENSG00000091039.15	2.57299939	0.87383644	5.56E-11	5.74E-10	0.05	0.06	0.17	0.7	OSBPL8	oxysterol binding protein-like 8	chr12	protein_coding
ENSG00000278834.1	-1.4029038	2.03734647	1.00E-05	4.85E-05	3.81	2.94	1.44	1.96	RP11-458J1.:	--	chr17	antisense
ENSG00000169964.5	-1.1174399	3.67002533	0.00016369	0.00062715	15.28	12.13	8.3	8.55	TMEM42	transmembrane protein 42	chr3	protein_coding
ENSG00000144395.16	2.6622958	0.99263622	6.37E-12	7.31E-11	0.07	0.13	0.58	1.08	CCDC150	coiled-coil domain containing 150	chr2	protein_coding
ENSG00000262668.1	-7.7253475	-1.2151504	3.98E-12	4.67E-11	1.45	0.59	0	0	AJ003147.9	--	chr16	antisense
ENSG00000211895.4	-5.6609181	-2.7818379	0.00086564	0.00288128	0.05	0.23	0	0	IGHA1	immunoglobulin heavy constant alpha 1	chr14	IG_C_gene
ENSG00000179387.8	1.92613954	1.28009463	5.60E-08	3.89E-07	0.17	0.15	0.61	1.04	ELMOD2	ELMO/CED-12 domain containing 2	chr4	protein_coding
ENSG00000267064.1	3.00116668	-2.6722499	0.00943894	0.02469931	0.02	0	0.08	0.14	UXT-AS1	UXT antisense RNA 1	chrX	antisense
ENSG00000082641.14	-1.051245	9.58641373	0.00024817	0.00092405	143.42	170.79	101.56	100.23	NFE2L1	nuclear factor, erythroid 2-like 1	chr17	protein_coding
ENSG00000118762.6	1.87724713	2.48445237	4.24E-09	3.44E-08	0.35	0.4	1.52	2.16	PKD2	polycystic kidney disease 2 (autosomal dominant)	chr4	protein_coding
ENSG00000272583.1	-6.0013451	-2.5669109	0.00014842	0.00057423	0.29	0.55	0	0	RP11-344P1:	--	chr1	lincRNA
ENSG00000259959.1	2.57496658	-2.3509436	0.00455824	0.01291303	0.01	0	0.09	0.03	RP11-121C2	--	chr4	lincRNA
ENSG00000114541.13	2.11200086	-2.2492681	0.02071392	0.04894569	0	0.01	0.06	0.03	FRMD4B	FERM domain containing 4B	chr3	protein_coding
ENSG00000254445.1	4.88946445	-3.0622981	0.01889873	0.04519557	0	0	0.04	0.11	HSPB2-C11c	HSPB2-C11orf52 readthrough (NMD candidate)	chr11	protein_coding
ENSG00000139117.12	2.04176356	0.13466604	1.72E-06	9.57E-06	0.04	0.11	0.38	0.46	CPNE8	copine VIII	chr12	protein_coding
ENSG00000188092.13	1.59069602	1.02727005	6.99E-06	3.50E-05	0.41	0.4	1.33	1.94	GPR89B	G protein-coupled receptor 89B	chr1	protein_coding
ENSG00000183317.15	7.57053505	1.89830756	6.48E-41	5.23E-39	0.01	0	1.2	1.08	EPHA10	EPH receptor A10	chr1	protein_coding
ENSG00000163605.13	4.04014657	2.20505304	2.24E-26	8.46E-25	0.07	0.08	1.23	2.19	PPP4R2	protein phosphatase 4, regulatory subunit 2	chr3	protein_coding
ENSG00000125384.6	-9.1696362	0.13462281	5.25E-24	1.68E-22	0.79	0.63	0	0	PTGER2	prostaglandin E receptor 2 (subtype EP2), 53kDa	chr14	protein_coding
ENSG00000116903.7	2.16404739	0.53753298	4.00E-08	2.81E-07	0.08	0.07	0.4	0.54	EXOC8	exocyst complex component 8	chr1	protein_coding
ENSG00000105668.6	7.43997135	-1.2918772	7.65E-11	7.77E-10	0	0	0.7	0.34	UPK1A	uroplakin 1A	chr19	protein_coding
ENSG00000213719.7	-1.8232123	9.43360119	4.74E-10	4.32E-09	483.79	414.62	168.29	170.25	CLIC1	chloride intracellular channel 1	chr6	protein_coding
ENSG00000277778.1	3.10155371	-2.6122053	0.00943894	0.02469931	0	0.01	0.03	0.15	PGM5P2	phosphoglucomutase 5 pseudogene 2	chr9	lincRNA
ENSG00000143226.12	-1.5160612	-1.4313253	0.01151564	0.0292584	0.04	0.28	0.11	0.05	FCGR2A	Fc fragment of IgG, low affinity IIa, receptor (CD32)	chr1	protein_coding
ENSG00000105825.10	-4.181213	0.42115091	1.26E-18	2.72E-17	0.72	0.95	0.04	0.08	TFPI2	tissue factor pathway inhibitor 2	chr7	protein_coding
ENSG00000255302.3	1.56042153	4.16950953	1.67E-07	1.08E-06	2.55	3.85	7.33	18.01	EID1	EP300 interacting inhibitor of differentiation 1	chr15	protein_coding
ENSG00000185924.6	5.68147601	2.69119431	3.80E-41	3.09E-39	0.04	0.06	3.33	4.07	RTN4RL1	reticulon 4 receptor-like 1	chr17	protein_coding
ENSG00000117525.12	2.08486343	3.84303775	9.73E-12	1.09E-10	1.75	2.09	8.89	12.94	F3	coagulation factor III (thromboplastin, tissue factor)	chr1	protein_coding
ENSG00000159761.13	1.11930311	-0.2224125	0.00810813	0.02158206	0.49	0.13	0.66	1.16	C16orf86	chromosome 16 open reading frame 86	chr16	protein_coding
ENSG00000168724.13	1.27420781	4.77283324	1.35E-05	6.40E-05	1.64	2.52	4.95	8.53	DNAJC21	DnaJ (Hsp40) homolog, subfamily C, member 21	chr5	protein_coding
ENSG00000036672.14	-2.4687657	1.07445166	1.19E-11	1.33E-10	0.61	0.79	0.15	0.19	USP2	ubiquitin specific peptidase 2	chr11	protein_coding
ENSG00000147231.12	6.30147617	-2.1795914	1.55E-05	7.26E-05	0	0	0.03	0.13	CXorf57	chromosome X open reading frame 57	chrX	protein_coding
ENSG00000246777.1	7.30830779	-1.394464	4.49E-10	4.11E-09	0	0	0.19	0.25	RP11-61A14	--	chr16	lincRNA
ENSG00000019144.15	1.60856944	6.86550497	3.53E-08	2.51E-07	7.98	7.89	31.78	32.75	PHLDB1	pleckstrin homology-like domain, family B, member 1	chr11	protein_coding
ENSG00000145819.14	-2.5401411	2.79519169	3.76E-15	6.08E-14	1.08	0.96	0.25	0.21	ARHGAP26	Rho GTPase activating protein 26	chr5	protein_coding
ENSG00000163682.14	-1.4843013	8.31267808	3.10E-07	1.93E-06	345.59	286.99	144.84	156.98	RPL9	ribosomal protein L9	chr4	protein_coding
ENSG00000067248.8	2.26853008	2.58613023	2.73E-12	3.26E-11	0.37	0.36	1.55	3.15	DHX29	DEAH (Asp-Glu-Ala-His) box polypeptide 29	chr5	protein_coding
ENSG00000115540.13	2.23788443	0.45039174	3.69E-08	2.61E-07	0.09	0.07	0.31	0.75	MOB4	MOB family member 4, phocein	chr2	protein_coding
ENSG00000196267.11	1.57795981	-0.4613589	0.00074382	0.00251885	0.1	0.05	0.17	0.42	ZNF836	zinc finger protein 836	chr19	protein_coding
ENSG00000131171.11	-6.4149303	0.9471303	2.20E-30	1.03E-28	1.49	1.72	0.03	0.01	SH3BGRL	SH3 domain binding glutamate-rich protein like	chrX	protein_coding
ENSG00000213341.9	2.58861601	1.18081099	4.10E-12	4.80E-11	0.11	0.16	0.78	1.43	CHUK	conserved helix-loop-helix ubiquitous kinase	chr10	protein_coding
ENSG00000163939.17	1.96404833	2.78834282	4.92E-10	4.47E-09	0.26	0.31	1.4	1.55	PBRM1	polybromo 1	chr3	protein_coding
ENSG00000179889.17	-1.0282257	6.24078698	0.00035945	0.00129262	12.77	14.03	8.58	8.93	PDXDC1	pyridoxal-dependent decarboxylase domain containing 1	chr16	protein_coding
ENSG00000124570.16	-1.422697	7.77982505	9.10E-07	5.27E-06	54.86	54.74	27.26	27.21	SERPINB6	serpin peptidase inhibitor, clade B (ovalbumin), member 6	chr6	protein_coding
ENSG00000102882.10	-1.3017973	5.65783491	7.52E-06	3.74E-05	21.39	18.73	11.33	10.34	MAPK3	mitogen-activated protein kinase 3	chr16	protein_coding
ENSG00000185551.11	-1.059197	3.41950875	0.00037418	0.00134039	1.39	2.24	0.87	1.46	NR2F2	nuclear receptor subfamily 2, group F, member 2	chr15	protein_coding
ENSG00000140519.11	2.42924167	-1.7465894	0.00095666	0.0031536	0.04	0.01	0.23	0.19	RHCG	Rh family, C glycoprotein	chr15	protein_coding
ENSG00000168300.12	1.73355959	0.61179061	5.18E-06	2.66E-05	0.12	0.08	0.24	0.63	PCMTD1	protein-L-isoaspartate (D-aspartate) O-methyltransferase domain containing 1	chr8	protein_coding
ENSG00000135655.12	3.00616683	0.97529626	5.33E-14	7.64E-13	0.02	0.02	0.15	0.28	USP15	ubiquitin specific peptidase 15	chr12	protein_coding
ENSG00000224189.5	3.78027946	0.32225477	5.08E-15	8.13E-14	0.05	0	0.45	0.51	HAGLR	HOXD antisense growth-associated long non-coding RNA	chr2	antisense
ENSG00000179454.12	1.5770205	-0.5222906	0.00111944	0.0036441	0.02	0.06	0.03	0.26	KLHL28	kelch-like family member 28	chr14	protein_coding
ENSG00000223573.5	6.06981307	4.14408741	4.00E-54	7.11E-52	0.08	0.14	9.8	10.19	TINCR	tissue differentiation-inducing non-protein coding RNA	chr19	lincRNA
ENSG00000018625.13	3.62864353	0.57771855	1.93E-15	3.21E-14	0.01	0.05	0.53	0.49	ATP1A2	ATPase, Na+/K+ transporting, alpha 2 polypeptide	chr1	protein_coding
ENSG00000064933.15	2.55932554	1.07540018	1.33E-11	1.47E-10	0.1	0.09	0.53	1.01	PMS1	PMS1 postmeiotic segregation increased 1 (S. cerevisiae)	chr2	protein_coding
ENSG00000138172.9	3.06562514	0.29489794	1.44E-11	1.59E-10	0.14	0.06	0.92	1.32	CALHM2	calcium homeostasis modulator 2	chr10	protein_coding
ENSG00000102038.14	-1.0999869	1.02977366	0.00116271	0.00376756	0.52	0.57	0.16	0.51	SMARCA1	SWI/SNF related, matrix associated, actin dependent regulator of chromatin, subfamily a, member 1	chrX	protein_coding
ENSG00000168487.16	-2.4610628	7.49906044	2.31E-16	4.13E-15	52.09	51.66	13.37	11.69	BMP1	bone morphogenetic protein 1	chr8	protein_coding
ENSG00000206760.1	-1.3682829	0.02235226	0.00059739	0.00206259	8.68	6.38	3.61	4.15	SNORA6	small nucleolar RNA, H/ACA box 6	chr3	snoRNA
ENSG00000104763.16	1.32658987	4.03275206	7.86E-06	3.90E-05	2.37	2.74	6.68	10.49	ASAH1	N-acylsphingosine amidohydrolase (acid ceramidase) 1	chr8	protein_coding
ENSG00000184702.16	-1.9580723	3.36859915	1.99E-10	1.90E-09	5.9	4.9	1.88	1.83	5-Sep	septin 5	chr22	protein_coding
ENSG00000171488.13	1.31393378	1.27786959	0.00013452	0.00052728	0.18	0.12	0.44	0.55	LRRC8C	leucine rich repeat containing 8 family, member C	chr1	protein_coding
ENSG00000265386.2	-2.4933034	-2.7153812	0.00943894	0.02469931	0.36	0.57	0	0.18	RN7SL219P	RNA, 7SL, cytoplasmic 219, pseudogene	chr16	misc_RNA
ENSG00000103479.13	1.50995068	2.60087593	1.34E-06	7.55E-06	0.44	0.56	1.16	2.66	RBL2	retinoblastoma-like 2	chr16	protein_coding
ENSG00000198722.11	-1.2435912	4.89326856	2.03E-05	9.32E-05	5.12	5.28	2.91	2.94	UNC13B	unc-13 homolog B (C. elegans)	chr9	protein_coding
ENSG00000150201.13	-5.2072905	-3.0363253	0.00538165	0.01488851	0.19	0.07	0	0	FXYD4	FXYD domain containing ion transport regulator 4	chr10	protein_coding
ENSG00000154127.8	1.81510743	2.72467294	8.45E-09	6.55E-08	0.3	0.37	1.22	1.95	UBASH3B	ubiquitin associated and SH3 domain containing B	chr11	protein_coding
ENSG00000019505.6	-5.2072905	-3.0363253	0.00538165	0.01488851	0.03	0.01	0	0	SYT13	synaptotagmin XIII	chr11	protein_coding
ENSG00000152229.17	-1.8062524	2.12655785	1.93E-08	1.41E-07	2.12	1.52	0.61	0.78	PSTPIP2	proline-serine-threonine phosphatase interacting protein 2	chr18	protein_coding
ENSG00000270022.3	2.68299912	-1.3146876	2.93E-05	0.00013052	0.2	0	1.16	0.7	RNU12	RNA, U12 small nuclear	chr22	lincRNA
ENSG00000279417.1	6.8062165	0.35668565	8.33E-23	2.45E-21	0.01	0	0.46	0.81	RP11-382B1!	--	chr15	TEC
ENSG00000261353.1	-2.3645285	-1.6917055	0.00110238	0.00359298	1.17	0.91	0.56	0	CTA-14H9.5	--	chr6	lincRNA
ENSG00000157911.8	-1.1134795	5.77397564	0.00011858	0.00046931	20.39	21.22	13.49	12.09	PEX10	peroxisomal biogenesis factor 10	chr1	protein_coding
ENSG00000250986.1	2.90878529	0.15837515	1.07E-10	1.06E-09	0.08	0	0.4	0.46	AC141928.1	--	chr4	lincRNA
ENSG00000197603.12	3.66041373	2.83180154	1.90E-25	6.77E-24	0.06	0.09	1.02	1.48	C5orf42	chromosome 5 open reading frame 42	chr5	protein_coding
ENSG00000272906.1	-1.5484369	-0.2027256	0.00025876	0.00095857	1.19	0.83	0.42	0.5	RP11-533E1!	--	chr1	lincRNA
ENSG00000185825.14	-2.1446083	9.07516519	4.32E-13	5.61E-12	324.12	330.65	99.66	97.54	BCAP31	B-cell receptor-associated protein 31	chrX	protein_coding
ENSG00000151470.11	2.62380959	-1.9235986	0.00119227	0.00385709	0.01	0	0.09	0.02	C4orf33	chromosome 4 open reading frame 33	chr4	protein_coding
ENSG00000103355.11	-5.7793342	-2.7087353	0.00047761	0.00167191	0.1	0.05	0	0	PRSS33	protease, serine, 33	chr16	protein_coding
ENSG00000250132.5	-1.0608193	-0.4681869	0.01356448	0.03387516	0.2	0.2	0.04	0.21	RP11-359B1:	--	chr12	antisense
ENSG00000172260.12	-1.7677675	1.0263057	4.31E-07	2.63E-06	0.19	0.2	0.05	0.1	NEGR1	neuronal growth regulator 1	chr1	protein_coding
ENSG00000273387.1	1.78073159	-0.7191067	0.0003052	0.00110919	0.23	0.04	0.91	0.37	RP3-412A9.1	--	chr22	antisense
ENSG00000241563.3	-3.5758925	-2.637383	0.003237	0.00945156	0.14	0.15	0.03	0	CORT	cortistatin	chr1	protein_coding
ENSG00000235162.7	-1.605839	3.08700708	1.46E-07	9.54E-07	5.61	5.68	2.06	2.88	C12orf75	chromosome 12 open reading frame 75	chr12	protein_coding
ENSG00000213066.10	1.82945242	2.29590828	1.55E-08	1.15E-07	0.11	0.13	0.47	0.68	FGFR1OP	FGFR1 oncogene partner	chr6	protein_coding
ENSG00000067208.13	3.0822372	-0.1296932	3.86E-10	3.58E-09	0.03	0.01	0.12	0.31	EVI5	ecotropic viral integration site 5	chr1	protein_coding
ENSG00000274367.1	3.32455569	-2.4783616	0.00190233	0.00584723	0.06	0	0.54	0.52	LA16c-380H!	--	chr16	lincRNA
ENSG00000196466.9	3.20048317	-1.169104	1.08E-06	6.15E-06	0	0.03	0.14	0.28	ZNF799	zinc finger protein 799	chr19	protein_coding
ENSG00000105982.15	2.18622113	-1.120259	0.00011365	0.00045167	0.06	0	0.21	0.16	RNF32	ring finger protein 32	chr7	protein_coding
ENSG00000280303.2	1.10221751	1.99725503	0.0005911	0.00204264	0.99	1.02	2.94	2.8	ERICD	E2F1-regulated inhibitor of cell death (non-protein coding)	chr8	lincRNA
ENSG00000162494.5	5.9655835	-2.4200372	8.36E-05	0.00034052	0	0	0.13	0.1	LRRC38	leucine rich repeat containing 38	chr1	protein_coding
ENSG00000235989.3	1.50377745	-1.197762	0.0105162	0.02693027	0.03	0.08	0.15	0.27	MORC2-AS1	MORC2 antisense RNA 1	chr22	antisense
ENSG00000281501.1	1.2291578	-0.1959086	0.00424613	0.01210611	0.1	0.08	0.39	0.17	SEPSECS-AS	SEPSECS antisense RNA 1 (head to head)	chr4	antisense
ENSG00000243008.2	5.21752153	-2.8860363	0.00538165	0.01488851	0	0	0.11	0.06	RP11-556N2	--	chr13	processed_transcript
ENSG00000264885.1	1.33419669	-0.5566838	0.00460971	0.01304958	0.09	0.22	0.3	0.71	RP11-815I9.4	--	chr17	sense_intronic
ENSG00000146802.11	1.37853039	1.30471272	5.24E-05	0.00022269	0.18	0.1	0.39	0.59	TMEM168	transmembrane protein 168	chr7	protein_coding
ENSG00000151468.10	-2.7900986	3.97792284	7.58E-19	1.67E-17	6.03	6.07	1.19	1.14	CCDC3	coiled-coil domain containing 3	chr10	protein_coding
ENSG00000276248.1	1.90513443	-1.172344	0.00081646	0.00274429	0.07	0	0.07	0.3	RP11-230F1!	--	chr13	sense_intronic
ENSG00000144668.10	-5.9939092	-2.5705707	0.00014842	0.00057423	0.05	0	0	0	ITGA9	integrin, alpha 9	chr3	protein_coding
ENSG00000182141.8	2.19368771	-0.3028916	4.17E-06	2.17E-05	0	0.09	0.08	0.46	ZNF708	zinc finger protein 708	chr19	protein_coding
ENSG00000151458.10	1.77500348	0.14094824	1.86E-05	8.57E-05	0.06	0.02	0.16	0.23	ANKRD50	ankyrin repeat domain 50	chr4	protein_coding
ENSG00000139618.13	3.42492582	-0.4917018	1.79E-09	1.51E-08	0	0.01	0.05	0.15	BRCA2	breast cancer 2, early onset	chr13	protein_coding
ENSG00000072401.13	3.71111087	0.53595396	5.58E-16	9.69E-15	0.03	0.09	0.83	1.2	UBE2D1	ubiquitin-conjugating enzyme E2D 1	chr10	protein_coding
ENSG00000093000.17	1.18214304	4.33653942	5.93E-05	0.00024989	1.69	1.91	4.51	6.42	NUP50	nucleoporin 50kDa	chr22	protein_coding
ENSG00000277450.1	4.25789382	-1.8312363	4.25E-06	2.21E-05	0.09	0	1.25	1.51	CTB-96E2.10	--	chr17	sense_intronic
ENSG00000126895.12	-7.3491377	-1.5427978	7.05E-10	6.26E-09	0.24	0.29	0	0	AVPR2	arginine vasopressin receptor 2	chrX	protein_coding
ENSG00000172262.10	1.93188427	3.57222742	2.96E-10	2.77E-09	0.93	1.29	3.57	7.81	ZNF131	zinc finger protein 131	chr5	protein_coding
ENSG00000140274.12	-7.5673393	-1.3546917	3.23E-11	3.41E-10	0.38	0.27	0	0	DUOXA2	dual oxidase maturation factor 2	chr15	protein_coding
ENSG00000158636.15	1.40138058	1.77119227	2.11E-05	9.63E-05	0.16	0.23	0.61	0.76	C11orf30	chromosome 11 open reading frame 30	chr11	protein_coding
ENSG00000174021.9	-1.137248	4.65076318	9.76E-05	0.00039366	30.28	30.18	17.61	19.04	GNG5	guanine nucleotide binding protein (G protein), gamma 5	chr1	protein_coding
ENSG00000177830.16	1.21324907	5.85427841	2.85E-05	0.00012716	6.88	5.92	20.95	18.53	CHID1	chitinase domain containing 1	chr11	protein_coding
ENSG00000163331.9	-1.3158805	-0.1500852	0.00158222	0.00496128	0.46	0.87	0.36	0.34	DAPL1	death associated protein-like 1	chr2	protein_coding
ENSG00000121064.11	1.13552702	5.84177603	8.73E-05	0.00035398	15.42	14.43	41.9	45.63	SCPEP1	serine carboxypeptidase 1	chr17	protein_coding
ENSG00000250424.4	-3.6980412	-1.5118514	1.03E-06	5.93E-06	0.24	0.37	0.06	0	RP5-877J2.1	--	chr7	protein_coding
ENSG00000198039.10	3.38620755	-1.7962564	0.00010631	0.0004256	0	0.03	0.16	0.26	ZNF273	zinc finger protein 273	chr7	protein_coding
ENSG00000116675.14	1.58755953	0.89875455	1.29E-05	6.13E-05	0.11	0.11	0.36	0.56	DNAJC6	DnaJ (Hsp40) homolog, subfamily C, member 6	chr1	protein_coding
ENSG00000181291.5	7.94252606	-0.8507782	1.08E-12	1.34E-11	0	0	0.17	0.29	TMEM132E	transmembrane protein 132E	chr17	protein_coding
ENSG00000275994.1	-2.8599289	-1.0021835	6.90E-07	4.07E-06	5.51	4.54	0.98	0.83	SNORA24	small nucleolar RNA, H/ACA box 24	chr4	snoRNA
ENSG00000125735.9	2.40511309	-0.4464684	1.14E-06	6.48E-06	0.07	0	0.21	0.28	TNFSF14	tumor necrosis factor (ligand) superfamily, member 14	chr19	protein_coding
ENSG00000090612.19	2.87364418	0.03468016	4.84E-10	4.40E-09	0.02	0	0.1	0.16	ZNF268	zinc finger protein 268	chr12	protein_coding
ENSG00000165997.4	2.68221727	-0.586667	4.91E-07	2.97E-06	0.01	0.03	0.11	0.19	ARL5B	ADP-ribosylation factor-like 5B	chr10	protein_coding
ENSG00000146950.11	-2.7776064	3.04487463	9.17E-18	1.85E-16	1.47	1.6	0.27	0.33	SHROOM2	shroom family member 2	chrX	protein_coding
ENSG00000100077.13	2.9983898	0.41621464	6.39E-12	7.33E-11	0.02	0.02	0.26	0.25	ADRBK2	adrenergic, beta, receptor kinase 2	chr22	protein_coding
ENSG00000261229.4	5.44525159	-2.7521026	0.00158008	0.00495521	0	0	0.01	0.13	MTHFS	5,10-methenyltetrahydrofolate synthetase (5-formyltetrahydrofolate cyclo-ligase)	chr15	lincRNA
ENSG00000135338.12	5.84069036	-2.4999492	0.00026535	0.0009747	0	0	0	0.07	LCA5	Leber congenital amaurosis 5	chr6	protein_coding
ENSG00000001617.10	-4.0014445	6.38361369	4.03E-35	2.37E-33	33.83	28.05	2.72	2.43	SEMA3F	sema domain, immunoglobulin domain (Ig), short basic domain, secreted, (semaphorin) 3F	chr3	protein_coding
ENSG00000111348.7	-1.3793438	-1.3159019	0.01764505	0.04292107	0.3	0.24	0.23	0.05	ARHGDIB	Rho GDP dissociation inhibitor (GDI) beta	chr12	protein_coding
ENSG00000141562.16	1.25106537	7.40375054	1.47E-05	6.95E-05	19.37	16.22	55.08	57.96	NARF	nuclear prelamin A recognition factor	chr17	protein_coding
ENSG00000185305.9	3.12818781	-1.2302463	2.24E-06	1.22E-05	0.02	0.02	0.18	0.22	ARL15	ADP-ribosylation factor-like 15	chr5	protein_coding
ENSG00000260949.1	-1.2802775	-0.7733281	0.00624182	0.01703231	0.68	0.68	0.35	0.39	KB-1836B5.1	--	chr8	sense_overlapping
ENSG00000166024.12	1.27885662	2.48161378	4.12E-05	0.00017959	0.83	0.62	2.13	2.59	R3HCC1L	R3H domain and coiled-coil containing 1-like	chr10	protein_coding
ENSG00000272896.1	1.18280942	1.57701802	0.00034283	0.00123769	1.67	2.24	5.93	5.89	RP11-216L1:	--	chr9	protein_coding
ENSG00000185168.5	-2.0233022	0.17476344	4.02E-07	2.47E-06	0.74	0.67	0.17	0.28	LINC00482	long intergenic non-protein coding RNA 482	chr17	lincRNA
ENSG00000066382.15	-4.5677563	-1.2702477	3.63E-10	3.38E-09	0.08	0.09	0	0	MPPED2	metallophosphoesterase domain containing 2	chr11	protein_coding
ENSG00000171444.16	1.89561519	2.04300798	9.32E-09	7.17E-08	0.14	0.14	0.54	0.87	MCC	mutated in colorectal cancers	chr5	protein_coding
ENSG00000196659.9	1.58977359	-0.8408844	0.00217107	0.00661139	0.05	0.04	0.06	0.32	TTC30B	tetratricopeptide repeat domain 30B	chr2	protein_coding
ENSG00000204128.5	2.76281305	0.51349077	3.27E-11	3.44E-10	0.1	0.05	0.81	0.57	C2orf72	chromosome 2 open reading frame 72	chr2	protein_coding
ENSG00000254560.4	6.98683394	-1.6581702	1.87E-08	1.38E-07	0	0	0.37	0.42	BBOX1-AS1	BBOX1 antisense RNA 1	chr11	antisense
ENSG00000268163.1	-5.2118379	-3.0342168	0.00538165	0.01488851	0.04	0.3	0	0	AC004076.9	--	chr19	protein_coding
ENSG00000142149.7	10.9454643	1.94516386	3.32E-44	3.25E-42	0	0	1.11	1.07	HUNK	hormonally up-regulated Neu-associated kinase	chr21	protein_coding
ENSG00000159082.16	1.42429678	0.75655297	0.00010541	0.00042303	0.08	0.11	0.28	0.41	SYNJ1	synaptojanin 1	chr21	protein_coding
ENSG00000213077.5	6.17755099	-2.2703402	2.70E-05	0.00012094	0	0	0.08	0.17	FAM106A	family with sequence similarity 106, member A	chr17	protein_coding
ENSG00000222222.1	7.70424182	-1.0646725	2.65E-12	3.17E-11	0	0	5.23	3.73	RNU2-17P	RNA, U2 small nuclear 17, pseudogene	chr1	snRNA
ENSG00000230408.3	4.92065931	-3.045493	0.0100455	0.02582742	0	0	0.29	0	AC007163.6	--	chr2	antisense
ENSG00000159713.9	-7.5725697	-1.3515762	3.23E-11	3.41E-10	0.18	0.32	0	0	TPPP3	tubulin polymerization-promoting protein family member 3	chr16	protein_coding
ENSG00000083454.20	-2.1151486	3.45516471	8.16E-12	9.24E-11	5.57	4.67	1.8	1.35	P2RX5	purinergic receptor P2X, ligand gated ion channel, 5	chr17	protein_coding
ENSG00000166881.8	2.03051505	3.34853925	5.70E-11	5.87E-10	0.58	0.57	2.24	4.04	TMEM194A	transmembrane protein 194A	chr12	protein_coding
ENSG00000119787.12	2.02921342	2.36943907	4.22E-10	3.89E-09	0.42	0.42	1.63	2.94	ATL2	atlastin GTPase 2	chr2	protein_coding
ENSG00000072609.16	-1.0491623	4.44247132	0.00032659	0.00118191	2.04	2.13	1.22	1.47	CHFR	checkpoint with forkhead and ring finger domains, E3 ubiquitin protein ligase	chr12	protein_coding
ENSG00000167315.16	-7.5742824	5.4543836	4.94E-77	2.89E-74	18.57	19.98	0.07	0.19	ACAA2	acetyl-CoA acyltransferase 2	chr18	protein_coding
ENSG00000120738.7	3.2774148	3.02611393	2.65E-22	7.60E-21	0.34	0.43	5.44	4.55	EGR1	early growth response 1	chr5	protein_coding
ENSG00000153956.14	-2.5022183	-1.0110728	3.68E-06	1.94E-05	0.08	0.08	0.01	0.03	CACNA2D1	calcium channel, voltage-dependent, alpha 2/delta subunit 1	chr7	protein_coding
ENSG00000131015.4	2.33924884	2.90158642	2.54E-13	3.38E-12	1.63	1.28	8.69	11.02	ULBP2	UL16 binding protein 2	chr6	protein_coding
ENSG00000103507.12	-1.0985499	5.42289674	0.00015027	0.00058073	18.02	17.46	11.76	10.28	BCKDK	branched chain ketoacid dehydrogenase kinase	chr16	protein_coding
ENSG00000233184.5	-2.2642624	-0.2837915	4.31E-07	2.63E-06	0.53	0.63	0.1	0.21	RP11-421L21	--	chr1	antisense
ENSG00000196284.12	-2.8079097	1.92832054	5.65E-16	9.81E-15	1.13	1.16	0.24	0.2	SUPT3H	suppressor of Ty 3 homolog (S. cerevisiae)	chr6	protein_coding
ENSG00000267751.4	-4.2633841	0.89907942	3.10E-21	8.25E-20	3.84	2.76	0.41	0.06	AC009005.2	--	chr19	antisense
ENSG00000139324.10	5.34924395	0.32099221	2.93E-20	7.17E-19	0.01	0	0.24	0.42	TMTC3	transmembrane and tetratricopeptide repeat containing 3	chr12	protein_coding
ENSG00000136169.15	1.998371	-0.0802941	4.64E-06	2.40E-05	0.02	0.07	0.16	0.31	SETDB2	SET domain, bifurcated 2	chr13	protein_coding
ENSG00000051128.17	-1.5726519	5.78477715	7.57E-08	5.16E-07	25.12	25.66	10.92	11.84	HOMER3	homer scaffolding protein 3	chr19	protein_coding

ENSG00000175110.10	1.6980455	4.7293732	1.08E-08	8.20E-08	5.75	6.1	20.96	30.51	MRPS22	mitochondrial ribosomal protein S22	chr3	protein_coding
ENSG00000073150.12	-2.3377176	4.85643643	1.22E-14	1.88E-13	13.61	12.05	3.15	3.62	PANX2	pannexin 2	chr22	protein_coding
ENSG00000227214.2	-1.5267345	-1.3596799	0.0065433	0.01777607	0.36	0.44	0.06	0.29	HCG15	HLA complex group 15 (non-protein coding)	chr6	antisense
ENSG00000140350.14	-1.4836219	7.76250478	3.18E-07	1.98E-06	95.46	115.98	45.71	55.17	ANP32A	acidic (leucine-rich) nuclear phosphoprotein 32 family, member A	chr15	protein_coding
ENSG00000138100.12	-1.957123	-0.498281	1.83E-05	8.48E-05	0.52	0.3	0.14	0.14	TRIM54	tripartite motif containing 54	chr2	protein_coding
ENSG00000119862.11	1.77942785	1.34841891	2.73E-07	1.71E-06	0.25	0.2	0.9	1.19	LGALSL	lectin, galactoside-binding-like	chr2	protein_coding
ENSG00000143369.13	-2.6013173	3.38891831	2.15E-16	3.88E-15	6.75	6.69	1.55	1.4	ECM1	extracellular matrix protein 1	chr1	protein_coding
ENSG00000269893.5	-2.2275114	4.33435049	2.28E-13	3.04E-12	45.73	34.24	9.87	12.97	SNHG8	small nucleolar RNA host gene 8	chr4	lincRNA
ENSG00000170017.11	-2.9228739	2.0098033	2.34E-17	4.58E-16	1.09	1.35	0.14	0.28	ALCAM	activated leukocyte cell adhesion molecule	chr3	protein_coding
ENSG00000279107.1	1.87165054	-1.6741001	0.00486779	0.01371541	0.14	0	0.39	0.33	RP11-823P9.	--	chr5	TEC
ENSG00000143153.11	1.19810308	6.43264711	3.41E-05	0.00015037	14.53	16.85	40.05	56.24	ATP1B1	ATPase, Na+/K+ transporting, beta 1 polypeptide	chr1	protein_coding
ENSG00000123636.16	3.73396363	0.55401616	9.66E-16	1.64E-14	0	0.03	0.36	0.3	BAZ2B	bromodomain adjacent to zinc finger domain, 2B	chr2	protein_coding
ENSG00000258101.2	2.5936897	-1.6269967	0.00044263	0.00156293	0.04	0.04	0.2	0.43	RP11-977B1(	--	chr12	antisense
ENSG00000155868.7	1.41492849	0.77304424	0.0001206	0.00047706	0.3	0.21	0.6	1.24	MED7	mediator complex subunit 7	chr5	protein_coding
ENSG00000089177.16	1.5888352	1.33226555	3.71E-06	1.95E-05	0.14	0.18	0.58	0.68	KIF16B	kinesin family member 16B	chr20	protein_coding
ENSG00000162777.15	3.06182111	3.52204397	4.96E-21	1.30E-19	0.96	0.83	8.92	11.12	DENND2D	DENN/MADD domain containing 2D	chr1	protein_coding
ENSG00000105750.13	-7.0898943	-1.7590881	1.16E-08	8.76E-08	0.08	0.15	0	0	ZNF85	zinc finger protein 85	chr19	protein_coding
ENSG00000177888.7	3.458031	-1.7417278	6.90E-05	0.00028699	0.01	0	0.02	0.09	ZBTB41	zinc finger and BTB domain containing 41	chr1	protein_coding
ENSG00000179909.14	-2.8567001	-2.5079329	0.00737015	0.01977584	0.04	0.02	0.01	0	ZNF154	zinc finger protein 154	chr19	protein_coding
ENSG00000124302.11	5.06203518	-2.9716376	0.0100455	0.02582742	0	0	0.04	0.05	CHST8	carbohydrate (N-acetylgalactosamine 4-0) sulfotransferase 8	chr19	protein_coding
ENSG00000258819.1	-8.345054	-0.6531458	1.75E-16	3.17E-15	0.5	0.59	0	0	RP11-7F17.3	--	chr14	lincRNA
ENSG00000108381.9	4.87957143	-1.3399184	3.66E-08	2.59E-07	0	0.01	0.1	0.14	ASPA	aspartoacylase	chr17	protein_coding
ENSG00000278107.1	-5.0252555	-3.1285772	0.0100455	0.02582742	0	0.34	0	0	RP11-162A1.	--	chr18	lincRNA
ENSG00000197782.13	1.2892708	-0.2486936	0.00275034	0.008227	0.06	0.04	0.08	0.22	ZNF780A	zinc finger protein 780A	chr19	protein_coding
ENSG00000135454.12	-3.258306	3.45993915	2.72E-23	8.33E-22	4.02	3.58	0.55	0.51	B4GALNT1	beta-1,4-N-acetyl-galactosaminyl transferase 1	chr12	protein_coding
ENSG00000007314.10	-4.8742027	0.12892033	6.93E-20	1.66E-18	0.2	0.23	0	0.01	SCN4A	sodium channel, voltage gated, type IV alpha subunit	chr17	protein_coding
ENSG00000196668.3	-6.3572647	-2.3216041	8.95E-06	4.38E-05	0.15	0.16	0	0	LINC00173	long intergenic non-protein coding RNA 173	chr12	processed_transcript
ENSG00000241860.5	4.72703895	-3.141721	0.01889873	0.04519557	0	0	0.04	0.01	RP11-34P13.	--	chr1	processed_transcript
ENSG00000119547.5	-6.4080403	-0.3947938	7.61E-19	1.68E-17	0.04	0.11	0	0	ONECUT2	one cut homeobox 2	chr18	protein_coding
ENSG00000234880.1	2.34012448	1.60552899	2.94E-11	3.12E-10	0.43	0.29	2.32	2.6	LINC00163	long intergenic non-protein coding RNA 163	chr21	lincRNA
ENSG00000241945.6	-6.2250093	1.90275511	2.79E-39	2.06E-37	1.72	1.92	0.03	0.03	PWP2	PWP2 periodic tryptophan protein homolog (yeast)	chr21	protein_coding
ENSG00000187840.4	-3.0603069	8.7920923	2.14E-23	6.57E-22	626.89	629.94	108.47	91.81	EIF4EBP1	eukaryotic translation initiation factor 4E binding protein 1	chr8	protein_coding
ENSG00000164187.6	2.13383493	0.88928475	1.72E-08	1.27E-07	0.04	0.09	0.26	0.49	LMBRD2	LMBR1 domain containing 2	chr5	protein_coding
ENSG00000256043.2	3.35544692	-1.3773648	4.23E-06	2.20E-05	0	0.03	0.09	0.31	CTSO	cathepsin O	chr4	protein_coding
ENSG00000167460.13	-1.9223565	9.26946035	5.96E-11	6.12E-10	226.63	297.03	82.58	101.74	TPM4	tropomyosin 4	chr19	protein_coding
ENSG00000183979.7	-1.9364757	-1.6702601	0.00345484	0.01003143	0.16	0.16	0.03	0.08	NPB	neuropeptide B	chr17	protein_coding
ENSG00000246740.2	5.47433081	-2.7360128	0.00158008	0.00495521	0	0	0.09	0.05	PLA2G4E-AS	PLA2G4E antisense RNA 1	chr15	antisense
ENSG00000006042.10	6.02887888	3.52790894	1.19E-49	1.59E-47	0.05	0.07	4.86	5.48	TMEM98	transmembrane protein 98	chr17	protein_coding
ENSG00000131023.11	2.1781696	0.95447493	4.80E-09	3.84E-08	0.08	0.06	0.24	0.6	LATS1	large tumor suppressor kinase 1	chr6	protein_coding
ENSG00000269313.4	-1.3556157	1.04028415	9.74E-05	0.00039306	0.52	0.61	0.31	0.28	MAGIX	MAGI family member, X-linked	chrX	protein_coding
ENSG00000122592.7	1.65437113	4.98183986	2.07E-08	1.52E-07	4.77	7.44	26.27	24.8	HOXA7	homeobox A7	chr7	protein_coding
ENSG00000268895.4	-5.1754454	0.11245303	6.56E-21	1.68E-19	0.35	0.86	0	0.04	A1BG-AS1	A1BG antisense RNA 1	chr19	antisense
ENSG00000163617.9	2.04599638	-1.3723639	0.00068945	0.00234732	0.01	0.04	0.14	0.14	KIAA1407	KIAA1407	chr3	protein_coding
ENSG00000259001.3	2.58928971	3.28904956	5.02E-16	8.75E-15	3.44	3.55	43.28	12.12	RPPH1	ribonuclease P RNA component H1	chr14	antisense
ENSG00000258704.4	1.70500002	-0.597212	0.00033978	0.00122705	0.23	0.14	0.95	0.69	SRP54-AS1	SRP54 antisense RNA 1 (head to head)	chr14	processed_transcript
ENSG00000266278.1	-4.5669136	0.79497878	9.75E-23	2.86E-21	1.31	0.72	0.06	0.05	RP11-41O4.2	--	chr18	lincRNA
ENSG00000132676.14	-1.0005603	6.906352	0.00049964	0.00174495	47.56	46.42	30.44	32.19	DAP3	death associated protein 3	chr1	protein_coding
ENSG00000146072.6	-2.1251674	4.20858579	2.47E-12	2.98E-11	6.71	6.83	1.99	2.15	TNFRSF21	tumor necrosis factor receptor superfamily, member 21	chr6	protein_coding
ENSG00000139597.15	6.89851681	-1.7278451	4.98E-08	3.47E-07	0	0	0.13	0.17	N4BP2L1	NEDD4 binding protein 2-like 1	chr13	protein_coding
ENSG00000182175.12	1.9883716	-1.1166076	0.00032876	0.00118885	0.01	0.01	0.06	0.05	RGMA	repulsive guidance molecule family member a	chr15	protein_coding
ENSG00000197093.9	1.63150425	-1.4978137	0.00678619	0.01837345	0.02	0.07	0.26	0.11	GAL3ST4	galactose-3-O-sulfotransferase 4	chr7	protein_coding
ENSG00000125657.4	2.03551768	4.74536388	1.26E-11	1.39E-10	5.86	4.31	29.86	25.69	TNFSF9	tumor necrosis factor (ligand) superfamily, member 9	chr19	protein_coding
ENSG00000146263.10	2.12260199	0.30721656	2.76E-07	1.73E-06	0.04	0.04	0.16	0.31	MMS22L	MMS22-like, DNA repair protein	chr6	protein_coding
ENSG00000156966.6	1.7188329	-0.5734202	0.00044579	0.00157293	0.01	0.11	0.1	0.42	B3GNT7	UDP-GlcNAc:betaGal beta-1,3-N-acetylglucosaminyltransferase 7	chr2	protein_coding
ENSG00000106948.15	-1.764199	3.76166534	5.24E-09	4.18E-08	1.78	2.27	0.8	0.78	AKNA	AT-hook transcription factor	chr9	protein_coding
ENSG00000115756.11	-2.0132346	7.25635321	9.12E-12	1.03E-10	82.67	62.1	25.22	22.56	HPCAL1	hippocalcin-like 1	chr2	protein_coding
ENSG00000054148.16	-1.1615634	7.82846209	5.48E-05	0.00023199	279.39	227.73	163.84	137.79	PHPT1	phosphohistidine phosphatase 1	chr9	protein_coding
ENSG00000130433.6	-6.3890664	0.40636684	1.99E-25	7.09E-24	1	1.28	0.03	0	CACNG6	calcium channel, voltage-dependent, gamma subunit 6	chr19	protein_coding
ENSG00000144488.13	-3.5831961	2.61071413	5.36E-25	1.86E-23	2.1	1.57	0.15	0.26	ESPNL	espin-like	chr2	protein_coding
ENSG00000160588.8	4.45218895	0.15924278	5.08E-16	8.84E-15	0.02	0.01	0.4	0.66	MPZL3	myelin protein zero-like 3	chr11	protein_coding
ENSG00000270316.1	-4.8961988	0.60570537	4.26E-23	1.28E-21	1.04	0.73	0.02	0.05	C10orf32-AS	C10orf32-ASMT readthrough (NMD candidate)	chr10	protein_coding
ENSG00000226800.5	-5.7556012	-0.1975239	4.66E-19	1.05E-17	0.87	0.55	0.03	0	CACTIN-AS1	CACTIN antisense RNA 1	chr19	antisense
ENSG00000120068.6	1.76288915	0.13826278	1.86E-05	8.57E-05	0.13	0.28	0.95	0.91	HOXB8	homeobox B8	chr17	protein_coding
ENSG00000145002.11	-2.2103643	0.09794785	8.82E-08	5.94E-07	1.16	0.87	0.28	0.3	FAM86B2	family with sequence similarity 86, member B2	chr8	protein_coding
ENSG00000205413.6	2.68417763	-0.8934084	1.94E-06	1.07E-05	0	0.03	0.04	0.21	SAMD9	sterile alpha motif domain containing 9	chr7	protein_coding
ENSG00000253352.7	1.97603014	3.24525267	1.88E-10	1.81E-09	0.4	0.5	1.87	2.9	TUG1	taurine up-regulated 1 (non-protein coding)	chr22	antisense
ENSG00000162852.12	1.78312157	1.06530523	7.09E-07	4.18E-06	0.17	0.09	0.56	0.66	CNST	consortin, connexin sorting protein	chr1	protein_coding
ENSG00000224066.1	1.95686908	-1.8174398	0.00675696	0.01829845	0.04	0.06	0.28	0.26	RP4-622L5.7	--	chr1	antisense
ENSG00000135587.7	-1.2410148	3.72022016	3.08E-05	0.00013662	9.77	8.41	5.39	4.85	SMPD2	sphingomyelin phosphodiesterase 2, neutral membrane (neutral sphingomyelinase)	chr6	protein_coding
ENSG00000129757.11	-1.5478303	6.43451291	1.09E-07	7.30E-07	53.6	48.1	18.66	27.94	CDKN1C	cyclin-dependent kinase inhibitor 1C (p57, Kip2)	chr11	protein_coding
ENSG00000241635.6	4.87917439	-3.0678699	0.01889873	0.04519557	0	0	0	0.05	UGT1A1	UDP glucuronosyltransferase 1 family, polypeptide A1	chr2	protein_coding
ENSG00000130725.6	-1.1142045	8.11008612	0.00010673	0.00042707	215.84	189.73	136.12	113.03	UBE2M	ubiquitin-conjugating enzyme E2M	chr19	protein_coding
ENSG00000180638.16	5.06203518	-2.9716376	0.0100455	0.02582742	0	0	0.05	0.06	SLC47A2	solute carrier family 47 (multidrug and toxin extrusion), member 2	chr17	protein_coding
ENSG00000274021.1	1.74557532	-1.7542385	0.01347572	0.03367454	0.07	0	0.11	0.22	RP11-823E8.	--	chr12	lincRNA
ENSG00000108846.14	-1.0755358	7.72956355	0.00018282	0.00069522	38.22	34.38	24.06	21.8	ABCC3	ATP-binding cassette, sub-family C (CFTR/MRP), member 3	chr17	protein_coding
ENSG00000143797.10	1.31230141	1.13454457	0.00015453	0.00059567	0.1	0.15	0.39	0.45	MBOAT2	membrane bound O-acyltransferase domain containing 2	chr2	protein_coding
ENSG00000124440.14	-7.7024893	-1.2375484	6.01E-12	6.93E-11	0.08	0.12	0	0	HIF3A	hypoxia inducible factor 3, alpha subunit	chr19	protein_coding
ENSG00000143847.14	1.06183804	2.55081877	0.00058229	0.0020148	0.52	0.27	0.97	1.27	PPFIA4	protein tyrosine phosphatase, receptor type, f polypeptide (PTPRF), interacting protein (liprin), alpha 4	chr1	protein_coding
ENSG00000004848.6	5.35410067	-2.8076728	0.00290522	0.00856312	0	0	0.08	0.04	ARX	aristaless related homeobox	chrX	protein_coding
ENSG00000105963.12	1.15837207	3.46915238	0.00011036	0.00043952	1.99	1.94	6.55	5.1	ADAP1	ArfGAP with dual PH domains 1	chr7	protein_coding
ENSG00000166479.8	1.76962889	2.05622869	6.67E-08	4.59E-07	0.25	0.29	0.85	1.62	TMX3	thioredoxin-related transmembrane protein 3	chr18	protein_coding
ENSG00000176845.11	-1.3573855	5.44943017	3.19E-06	1.70E-05	23.92	16.68	10.35	10.81	METRNL	meteorin, glial cell differentiation regulator-like	chr17	protein_coding
ENSG00000147257.12	10.870622	1.87394468	3.41E-43	3.14E-41	0	0	2.58	3.22	GPC3	glypican 3	chrX	protein_coding
ENSG00000234961.1	-6.0013451	-2.5669109	0.00014842	0.00057423	0.19	0.36	0	0	RP11-124N1	--	chr10	antisense
ENSG00000138778.10	5.02374123	0.91831805	1.37E-23	4.30E-22	0.02	0	0.17	0.69	CENPE	centromere protein E, 312kDa	chr4	protein_coding
ENSG00000143742.11	1.22631838	4.11377296	3.38E-05	0.00014895	4.16	5.51	8.4	22.05	SRP9	signal recognition particle 9kDa	chr1	protein_coding
ENSG00000004455.15	-1.4968617	8.50708274	2.47E-07	1.56E-06	98.19	96.02	43.85	47.94	AK2	adenylate kinase 2	chr1	protein_coding
ENSG00000185246.16	3.6165106	1.62037434	1.04E-20	2.65E-19	0.15	0.1	0.99	3.14	PRPF39	pre-mRNA processing factor 39	chr14	protein_coding
ENSG00000279199.1	6.08024817	-2.335817	4.74E-05	0.00020301	0	0	0	0.23	RP11-749I16	--	chr17	TEC
ENSG00000132357.12	-1.2904677	1.72832147	6.92E-05	0.00028789	0.8	0.91	0.41	0.53	CARD6	caspase recruitment domain family, member 6	chr5	protein_coding
ENSG00000248677.1	6.49539722	-2.0380788	3.03E-06	1.62E-05	0	0	0.08	0.14	CTD-2044J1!	--	chr5	lincRNA
ENSG00000155099.6	-1.6675686	0.84896655	4.13E-06	2.15E-05	0.85	0.58	0.31	0.3	TMEM55A	transmembrane protein 55A	chr8	protein_coding
ENSG00000081320.9	2.52858902	-0.1356742	8.72E-08	5.89E-07	0.01	0.06	0.13	0.41	STK17B	serine/threonine kinase 17b	chr2	protein_coding
ENSG00000129071.8	1.55897508	3.21036691	3.33E-07	2.07E-06	1.02	1.52	4.02	5.94	MBD4	methyl-CpG binding domain protein 4	chr3	protein_coding
ENSG00000135976.15	5.21008573	-0.2969625	5.61E-15	8.96E-14	0.01	0	0.13	0.3	ANKRD36	ankyrin repeat domain 36	chr2	protein_coding
ENSG00000168646.11	-2.9484323	0.52876413	5.70E-13	7.34E-12	0.51	0.37	0.06	0.09	AXIN2	axin 2	chr17	protein_coding
ENSG00000197121.13	1.4123737	-0.4421905	0.00205333	0.00628319	0.02	0.03	0.05	0.12	PGAP1	post-GPI attachment to proteins 1	chr2	protein_coding
ENSG00000123191.12	1.18764683	1.8971156	0.00022669	0.0008491	0.2	0.35	0.79	0.85	ATP7B	ATPase, Cu++ transporting, beta polypeptide	chr13	protein_coding
ENSG00000163701.17	-2.6173105	-1.2007826	3.48E-06	1.84E-05	0.08	0.32	0.02	0.06	IL17RE	interleukin 17 receptor E	chr3	protein_coding
ENSG00000013374.14	1.43086389	6.48606411	8.56E-07	4.98E-06	10.19	14.2	36.21	51.67	NUB1	negative regulator of ubiquitin-like proteins 1	chr7	protein_coding
ENSG00000276851.1	1.68492594	-0.1907736	0.00011053	0.00043999	0.32	0.73	2.25	2.24	RP5-875H18	--	chr17	lincRNA
ENSG00000133104.11	2.32122993	2.70800176	7.42E-13	9.44E-12	0.19	0.47	1.38	2.96	SPG20	spastic paraplegia 20 (Troyer syndrome)	chr13	protein_coding
ENSG00000156853.10	-1.005212	4.32626855	0.0005866	0.00202884	6.08	6.13	4.17	3.92	ZNF689	zinc finger protein 689	chr16	protein_coding
ENSG00000073861.2	6.3477803	-2.1526603	5.19E-06	2.66E-05	0	0	0.19	0.07	TBX21	T-box 21	chr17	protein_coding
ENSG00000153395.8	-1.3522451	8.31724031	2.90E-06	1.56E-05	95.37	91.74	46.22	51.57	LPCAT1	lysophosphatidylcholine acyltransferase 1	chr5	protein_coding
ENSG00000184428.11	-1.0090682	7.32147488	0.00044328	0.00156475	73.09	72.28	48.29	47.93	TOP1MT	topoisomerase (DNA) I, mitochondrial	chr8	protein_coding
ENSG00000101596.13	2.6367206	2.0411735	2.10E-14	3.15E-13	0.1	0.11	0.58	1.11	SMCHD1	structural maintenance of chromosomes flexible hinge domain containing 1	chr18	protein_coding
ENSG00000196782.11	1.04346272	0.51393928	0.0047011	0.01328791	0.14	0.06	0.29	0.27	MAML3	mastermind-like 3 (Drosophila)	chr4	protein_coding
ENSG00000173715.14	-1.8772344	3.63247654	6.70E-10	5.98E-09	7.4	7.26	2.76	2.56	C11orf80	chromosome 11 open reading frame 80	chr11	protein_coding
ENSG00000281100.1	4.87917439	-3.0678699	0.01889873	0.04519557	0	0	0	0.11	RP11-640L9.	--	chr3	TEC
ENSG00000247556.5	1.12900521	3.89221742	0.00013691	0.00053592	0.62	0.59	1.54	2.01	OIP5-AS1	OIP5 antisense RNA 1	chr15	processed_transcript
ENSG00000154229.10	-2.6597176	4.95633152	4.59E-18	9.49E-17	4.74	5.18	0.97	1.12	PRKCA	protein kinase C, alpha	chr17	protein_coding
ENSG00000182816.7	5.58503228	-2.6679206	0.00086564	0.00288128	0	0	0.18	0.13	KRTAP13-2	keratin associated protein 13-2	chr21	protein_coding
ENSG00000140948.10	1.13663181	4.75937148	9.85E-05	0.00039736	1.71	2.14	5.21	6.09	ZCCHC14	zinc finger, CCHC domain containing 14	chr16	protein_coding
ENSG00000149557.11	6.5897802	-1.973179	1.05E-06	6.02E-06	0	0	0.22	0.08	FEZ1	fasciculation and elongation protein zeta 1 (zygin I)	chr11	protein_coding
ENSG00000119711.11	1.16919567	2.19215571	0.00021858	0.00082063	0.34	0.43	1.16	1.16	ALDH6A1	aldehyde dehydrogenase 6 family, member A1	chr14	protein_coding
ENSG00000100290.2	2.94691265	0.97986239	1.06E-13	1.47E-12	0.22	0.51	3.87	3.63	BIK	BCL2-interacting killer (apoptosis-inducing)	chr22	protein_coding
ENSG00000261427.5	-2.2408315	-0.7514665	7.83E-06	3.89E-05	0.73	1.12	0.32	0.2	CTD-2349B8	--	chr16	protein_coding
ENSG00000198899.2	1.46530816	11.4711145	4.19E-07	2.57E-06	1274.75	2325.05	7068.27	6109.34	MT-ATP6	mitochondrially encoded ATP synthase 6	chrM	protein_coding
ENSG00000128253.12	6.83488342	-1.782808	8.18E-08	5.55E-07	0	0	0.23	0.12	RFPL2	ret finger protein-like 2	chr22	protein_coding
ENSG00000228288.5	4.33172096	0.47922685	5.36E-18	1.10E-16	0.15	0.06	3.74	2.23	PCAT6	prostate cancer associated transcript 6 (non-protein coding)	chr1	antisense
ENSG00000132664.10	2.12222064	1.10912812	5.69E-09	4.52E-08	0.25	0.26	0.82	2.18	POLR3F	polymerase (RNA) III (DNA directed) polypeptide F, 39 kDa	chr20	protein_coding
ENSG00000261706.1	2.02248228	4.76315271	1.66E-11	1.82E-10	10	11.01	57.38	56.25	LINC00165	long intergenic non-protein coding RNA 165	chr21	lincRNA
ENSG00000124006.13	-1.0493957	7.08166369	0.00026354	0.00097455	22.06	19.84	13.48	13.51	OBSL1	obscurin-like 1	chr2	protein_coding
ENSG00000169609.12	1.55241717	5.43096317	1.19E-07	7.90E-07	1.55	1.5	5.61	6.33	C15orf40	chromosome 15 open reading frame 40	chr15	protein_coding
ENSG00000117114.18	-4.4567516	-1.3556717	1.07E-08	8.19E-08	0.08	0.08	0.01	0	LPHN2	--	chr1	protein_coding
ENSG00000138587.5	4.71091412	0.83959367	6.33E-22	1.76E-20	0	0.1	0.9	2.54	MNS1	meiosis-specific nuclear structural 1	chr15	protein_coding
ENSG00000271079.1	6.15179698	-2.2852162	4.74E-05	0.00020301	0	0	0	0.21	CTAGE15	CTAGE family, member 15	chr7	protein_coding
ENSG00000011426.9	1.68285073	3.68632294	2.78E-08	2.00E-07	0.92	1.13	2.42	6.45	ANLN	anillin, actin binding protein	chr7	protein_coding
ENSG00000152763.15	2.96221508	-2.0923973	0.00228047	0.00692082	0	0.01	0	0.12	WDR78	WD repeat domain 78	chr1	protein_coding
ENSG00000213742.5	1.74518725	-0.4160808	0.00020204	0.00076235	0.07	0.03	0.2	0.22	ZNF337-AS1	ZNF337 antisense RNA 1	chr20	antisense
ENSG00000160472.4	-5.0206302	-3.1306986	0.0100455	0.02582742	0.23	0.1	0	0	TMEM190	transmembrane protein 190	chr19	protein_coding
ENSG00000122778.8	-1.8389626	4.86275438	6.39E-10	5.72E-09	2.91	3.02	1.06	1.15	KIAA1549	KIAA1549	chr7	protein_coding
ENSG00000272205.1	-1.3339006	-1.040036	0.00917505	0.0241697	0.75	0.75	0.52	0.28	RP11-277B1!	--	chr1	lincRNA
ENSG00000153060.6	5.20747907	-2.8915189	0.00538165	0.01488851	0	0	0.08	0.1	TEKT5	tektin 5	chr16	protein_coding
ENSG00000151640.11	8.40234089	2.7086466	2.72E-51	3.95E-49	0.01	0.01	5.28	4.81	DPYSL4	dihydropyrimidinase-like 4	chr10	protein_coding
ENSG00000125999.9	5.93857659	-2.4353816	0.00014842	0.00057423	0	0	0.06	0.22	BPIFB1	BPI fold containing family B, member 1	chr20	protein_coding
ENSG00000254812.1	-2.6368358	-2.2766964	0.00174589	0.00541921	0.27	0.36	0	0.12	RP11-661A1.	--	chr8	antisense
ENSG00000168269.8	-5.8957624	-2.6355558	0.00026535	0.0009747	0.06	0.1	0	0	FOXI1	forkhead box I1	chr5	protein_coding
ENSG00000275645.1	1.39644559	0.01153092	0.00069622	0.00236937	1.09	0.88	3.91	3.06	RP11-817O1	--	chr15	antisense
ENSG00000223617.1	-5.2109276	-3.0346387	0.00538165	0.01488851	0.08	0.24	0	0	LINC00370	long intergenic non-protein coding RNA 370	chr13	lincRNA
ENSG00000099785.9	-3.5016946	1.8723045	9.77E-22	2.70E-20	2.9	3.1	0.25	0.45	2-Mar	membrane-associated ring finger (C3HC4) 2, E3 ubiquitin protein ligase	chr19	protein_coding
ENSG00000165795.19	1.63077209	3.65014071	6.96E-08	4.77E-07	1.13	1.41	5.5	4.98	NDRG2	NDRG family member 2	chr14	protein_coding
ENSG00000165949.11	-2.2494933	-2.2240887	0.00550654	0.01520944	0.23	0.31	0.04	0.1	IFI27	interferon, alpha-inducible protein 27	chr14	protein_coding
ENSG00000136244.10	-1.5959384	1.64396937	1.42E-06	8.00E-06	1.69	2.73	1.07	0.87	IL6	interleukin 6	chr7	protein_coding
ENSG00000166780.9	-6.1913931	4.83941879	1.28E-59	3.01E-57	17.9	16.09	0.36	0.26	C16orf45	chromosome 16 open reading frame 45	chr16	protein_coding
ENSG00000142178.7	-2.3132378	3.59341931	8.33E-14	1.17E-12	3.75	3.15	0.8	1.06	SIK1	salt-inducible kinase 1	chr21	protein_coding
ENSG00000137285.9	-3.5483107	0.57971612	1.90E-16	3.43E-15	1.19	1	0.1	0.15	TUBB2B	tubulin, beta 2B class IIb	chr6	protein_coding
ENSG00000121057.11	-1.1728949	6.38697491	4.89E-05	0.00020923	17.88	20.45	10.24	12.45	AKAP1	A kinase (PRKA) anchor protein 1	chr17	protein_coding
ENSG00000132801.6	1.13280177	2.51840781	0.00024797	0.00092345	1.08	0.98	2.86	3.17	ZSWIM3	zinc finger, SWIM-type containing 3	chr20	protein_coding
ENSG00000277152.1	3.7729392	-2.178289	0.00023419	0.00087473	0	0	0.01	0.07	RP11-622C2.	--	chr15	lincRNA
ENSG00000168569.7	-1.1750826	5.01357589	5.43E-05	0.00023017	21.1	21.39	12.93	12.12	TMEM223	transmembrane protein 223	chr11	protein_coding
ENSG00000166596.13	3.40938898	-2.4241991	0.00190233	0.00584723	0.01	0	0.08	0.12	CFAP52	cilia and flagella associated protein 52	chr17	protein_coding
ENSG00000271092.4	2.40654075	-0.5450647	1.94E-06	1.07E-05	0.04	0.17	0.43	1.02	TMEM56-RV	TMEM56-RWDD3 readthrough	chr1	protein_coding
ENSG00000101972.17	3.00216603	2.68129485	6.22E-19	1.39E-17	0.18	0.16	1.28	2.38	STAG2	stromal antigen 2	chrX	protein_coding
ENSG00000159337.6	5.58023914	-2.6705827	0.00086564	0.00288128	0	0	0.05	0.05	PLA2G4D	phospholipase A2, group IVD (cytosolic)	chr15	protein_coding
ENSG00000279236.1	5.06713725	-2.9688687	0.0100455	0.02582742	0	0	0.12	0.08	RP11-120K1!	--	chr18	TEC
ENSG00000198826.9	1.27443878	3.17314688	2.57E-05	0.0001158	0.65	0.7	2.13	2.22	ARHGAP11A	Rho GTPase activating protein 11A	chr15	protein_coding
ENSG00000144681.9	-3.7363742	3.1421524	9.56E-28	3.92E-26	3.6	4.66	0.43	0.4	STAC	SH3 and cysteine rich domain	chr3	protein_coding
ENSG00000115107.18	-1.4378199	6.90954878	7.31E-07	4.30E-06	30.56	31.77	15.29	15.36	STEAP3	STEAP family member 3, metalloreductase	chr2	protein_coding
ENSG00000088881.19	3.08636517	0.98193685	1.73E-14	2.62E-13	0.09	0.12	1.36	1.05	EBF4	early B-cell factor 4	chr20	protein_coding
ENSG00000205246.7	-1.1137647	4.94111388	0.00012892	0.00050731	27.88	20.71	14.74	15.22	RPSAP58	ribosomal protein SA pseudogene 58	chr19	protein_coding
ENSG00000164118.11	1.12644848	-0.4606457	0.01200175	0.03032314	0.05	0.05	0.1	0.2	CEP44	centrosomal protein 44kDa	chr4	protein_coding
ENSG00000103647.11	-3.1965795	-1.5917007	3.56E-06	1.88E-05	0.13	0.09	0	0.03	CORO2B	coronin, actin binding protein, 2B	chr15	protein_coding
ENSG00000169297.7	-6.3518111	-2.3243835	1.55E-05	7.26E-05	0.19	0.03	0	0	NR0B1	nuclear receptor subfamily 0, group B, member 1	chrX	protein_coding
ENSG00000113763.9	6.71262576	-1.8733651	3.73E-07	2.30E-06	0	0	0.09	0.13	UNC5A	unc-5 homolog A (C. elegans)	chr5	protein_coding
ENSG00000261386.2	4.99467843	2.22397204	5.04E-33	2.70E-31	0.07	0.5	12.16	11.67	CTD-2012K1	--	chr16	lincRNA
ENSG00000221818.7	4.89980841	-3.0567113	0.01889873	0.04519557	0	0	0.02	0.02	EBF2	early B-cell factor 2	chr8	protein_coding
ENSG00000197147.11	2.87865528	-0.294911	7.67E-09	5.97E-08	0.01	0.03	0.14	0.22	LRRC8B	leucine rich repeat containing 8 family, member B	chr1	protein_coding
ENSG00000149548.13	1.29517777	-1.1350368	0.01979609	0.04704122	0.06	0.03	0.16	0.14	CCDC15	coiled-coil domain containing 15	chr11	protein_coding
ENSG00000151532.12	1.01205107	3.5475177	0.00067014	0.00228645	0.95	1.45	2.94	3.52	VTI1A	vesicle transport through interaction with t-SNAREs 1A	chr10	protein_coding
ENSG00000102710.18	1.28634243	2.01889248	6.28E-05	0.00026353	0.41	0.64	1.19	2.23	SUPT20H	suppressor of Ty 20 homolog (S. cerevisiae)	chr13	protein_coding
ENSG00000245293.2	5.96106449	-2.422601	8.36E-05	0.00034052	0	0	0.08	0.08	RP11-286E1.	--	chr4	antisense
ENSG00000251003.6	-5.5205905	-2.8639386	0.00158008	0.00495521	0.11	0.03	0	0	ZFPM2-AS1	ZFPM2 antisense RNA 1	chr8	processed_transcript
ENSG00000196653.10	7.32308793	-1.3787208	4.49E-10	4.11E-09	0	0	0.12	0.27	ZNF502	zinc finger protein 502	chr3	protein_coding
ENSG00000089692.7	1.42809447	-0.87102	0.00438177	0.01246521	0.12	0.07	0.44	0.25	LAG3	lymphocyte-activation gene 3	chr12	protein_coding
ENSG00000200259.1	-1.2630219	-0.4777373	0.00359918	0.01041519	8.4	9.56	4.84	5.07	SNORD35A	small nucleolar RNA, C/D box 35A	chr19	snoRNA
ENSG00000210194.1	2.51893726	-0.7179391	3.53E-06	1.87E-05	1.16	2.46	13	15	MT-TE	mitochondrially encoded tRNA glutamic acid	chrM	Mt_tRNA
ENSG00000133710.14	8.98410897	2.43494494	1.19E-49	1.59E-47	0.01	0	1.94	4.14	SPINK5	serine peptidase inhibitor, Kazal type 5	chr5	protein_coding
ENSG00000081041.8	-3.9069324	2.99497225	5.60E-29	2.46E-27	10.3	9.59	0.87	0.89	CXCL2	chemokine (C-X-C motif) ligand 2	chr4	protein_coding
ENSG00000163359.14	-1.5124961	0.65031461	4.34E-05	0.00018836	0.2	0.14	0.11	0.05	COL6A3	collagen, type VI, alpha 3	chr2	protein_coding

ENSG00000157764.11	1.15136451	1.01779057	0.0009889	0.00325251	0.44	0.34	1.28	1.05	BRAF	B-Raf proto-oncogene, serine/threonine kinase	chr7	protein_coding
ENSG00000197050.9	1.86093383	-0.5663365	9.76E-05	0.00039366	0.05	0.06	0.33	0.24	ZNF420	zinc finger protein 420	chr19	protein_coding
ENSG00000027644.4	-3.5817465	-2.6400807	0.003237	0.00945156	0.03	0.03	0	0.01	INSRR	insulin receptor-related receptor	chr1	protein_coding
ENSG00000185009.11	1.2024844	3.13604188	7.40E-05	0.00030587	0.65	0.98	2.31	2.69	AP3M1	adaptor-related protein complex 3, mu 1 subunit	chr10	protein_coding
ENSG00000133687.14	-1.793757	5.20919345	1.26E-09	1.08E-08	4.25	5.61	1.82	1.96	TMTC1	transmembrane and tetratricopeptide repeat containing 1	chr12	protein_coding
ENSG00000189042.12	3.97139922	0.17552234	1.07E-14	1.66E-13	0.01	0.04	0.24	0.75	ZNF567	zinc finger protein 567	chr19	protein_coding
ENSG00000242114.4	-1.615986	0.89070931	6.25E-06	3.15E-05	1.76	1.42	0.8	0.58	MTFP1	mitochondrial fission process 1	chr22	protein_coding
ENSG00000206611.1	4.92065931	-3.045493	0.0100455	0.02582742	0	0	3.42	0	SNORD24	small nucleolar RNA, C/D box 24	chr9	snoRNA
ENSG00000137337.13	-2.068783	6.76900377	2.80E-12	3.34E-11	16.36	20.38	6.13	5.51	MDC1	mediator of DNA-damage checkpoint 1	chr6	protein_coding
ENSG00000280152.1	1.99490369	-0.560653	3.32E-05	0.0001467	0.08	0.01	0.27	0.24	RP11-331F4.	--	chr16	TEC
ENSG00000124140.11	1.99473584	2.01698515	1.82E-09	1.53E-08	0.19	0.19	0.97	1.03	SLC12A5	solute carrier family 12 (potassium/chloride transporter), member 5	chr20	protein_coding
ENSG00000103811.14	3.69070257	6.41345232	5.65E-31	2.72E-29	3.92	5.14	79.01	76.55	CTSH	cathepsin H	chr15	protein_coding
ENSG00000277938.1	-1.367626	0.28162953	0.0003073	0.0011156	0.46	0.53	0.17	0.33	RP5-965G21	--	chr20	lincRNA
ENSG00000155016.16	1.19435896	1.99204253	0.00020352	0.00076736	0.36	0.38	0.83	1.45	CYP2U1	cytochrome P450, family 2, subfamily U, polypeptide 1	chr4	protein_coding
ENSG00000227838.1	-5.7793342	-2.7087353	0.00047761	0.00167191	0.26	0.14	0	0	RP1-213J1P_	--	chr22	lincRNA
ENSG00000204498.9	-1.4634242	6.5149762	4.85E-07	2.94E-06	70.3	76.79	36.45	34.53	NFKBIL1	nuclear factor of kappa light polypeptide gene enhancer in B-cells inhibitor-like 1	chr6	protein_coding
ENSG00000275764.1	4.66554413	-0.7689815	5.75E-11	5.92E-10	0	0.03	0.31	0.79	RP11-582E3.	--	chr12	lincRNA
ENSG00000117595.9	10.3775446	1.39643148	9.02E-37	5.71E-35	0	0	1.24	1.16	IRF6	interferon regulatory factor 6	chr1	protein_coding
ENSG00000175592.7	-1.2710208	6.4142199	1.13E-05	5.43E-05	48.71	57.48	30.38	28.13	FOSL1	FOS-like antigen 1	chr11	protein_coding
ENSG00000179546.4	-3.7490563	2.3902218	2.73E-25	9.62E-24	2.75	2.82	0.37	0.18	HTR1D	5-hydroxytryptamine (serotonin) receptor 1D, G protein-coupled	chr1	protein_coding
ENSG00000084070.10	-1.0776268	4.96528978	0.00021034	0.00079194	13.47	12.29	8.93	7.3	SMAP2	small ArfGAP2	chr1	protein_coding
ENSG00000152818.17	2.38529995	2.43438212	4.60E-13	5.97E-12	0.11	0.1	0.72	0.79	UTRN	utrophin	chr6	protein_coding
ENSG00000210127.1	3.68532085	2.24800653	9.05E-24	2.87E-22	5.04	10.68	145.31	123.13	MT-TA	mitochondrially encoded tRNA alanine	chrM	Mt_tRNA
ENSG00000251359.4	2.07839419	-0.4920494	1.59E-05	7.44E-05	0.07	0.1	0.47	0.54	WWC2-AS2	WWC2 antisense RNA 2	chr4	lincRNA
ENSG00000168939.9	-5.1891546	-0.7348457	3.08E-14	4.53E-13	0.09	0.11	0	0.01	SPRY3	sprouty homolog 3 (Drosophila)	chrX	protein_coding
ENSG00000281195.1	6.42571756	-2.0976964	3.03E-06	1.62E-05	0	0	0.38	0.07	ZNF638-IT1	ZNF638 intronic transcript 1	chr2	processed_transcript
ENSG00000149564.10	1.15470035	-0.0295552	0.00431545	0.01229353	0.38	0.11	0.81	0.67	ESAM	endothelial cell adhesion molecule	chr11	protein_coding
ENSG00000182183.13	6.04467832	-2.3656018	4.74E-05	0.00020301	0	0	0.39	0.33	FAM159A	family with sequence similarity 159, member A	chr1	protein_coding
ENSG00000103037.10	1.17235795	4.36041481	6.68E-05	0.00027899	2.5	3.11	7.29	9.59	SETD6	SET domain containing 6	chr16	protein_coding
ENSG00000135341.16	1.58619865	1.95937423	1.13E-06	6.41E-06	0.19	0.29	0.67	1.25	MAP3K7	mitogen-activated protein kinase kinase kinase 7	chr6	protein_coding
ENSG00000083099.9	1.40811195	1.09625472	5.52E-05	0.00023355	0.18	0.11	0.33	0.7	LYRM2	LYR motif containing 2	chr6	protein_coding
ENSG00000182010.9	2.98551298	-1.3398833	1.43E-05	6.79E-05	0.01	0	0.04	0.08	RTKN2	rhotekin 2	chr10	protein_coding
ENSG00000116761.10	-1.8478846	3.12159434	2.00E-09	1.68E-08	4.74	5.56	1.89	1.92	CTH	cystathionine gamma-lyase	chr1	protein_coding
ENSG00000183401.10	1.33548778	2.00333303	3.44E-05	0.00015143	1.39	1.17	4.82	3.77	CCDC159	coiled-coil domain containing 159	chr19	protein_coding
ENSG00000176022.4	-1.1308979	6.05153466	9.09E-05	0.00036785	26.7	26.38	15.33	17	B3GALT6	UDP-Gal:betaGal beta 1,3-galactosyltransferase polypeptide 6	chr1	protein_coding
ENSG00000270885.1	-1.0612962	3.53496753	0.00036146	0.00129947	4.3	3.59	2.74	2.29	RASL10B	RAS-like, family 10, member B	chr17	protein_coding
ENSG00000253220.1	5.56116133	-2.6812008	0.00086564	0.00288128	0	0	0.13	0.61	RP11-103H7	--	chr8	lincRNA
ENSG00000109189.11	1.30248464	-0.3967544	0.00351339	0.01018787	0.03	0.05	0.08	0.17	USP46	ubiquitin specific peptidase 46	chr4	protein_coding
ENSG00000067141.15	6.93228084	6.30381051	2.32E-73	1.14E-70	0.12	0.17	24.08	21.82	NEO1	neogenin 1	chr15	protein_coding
ENSG00000232010.1	4.57135599	1.54104434	4.02E-26	1.49E-24	0.1	0.6	9.74	12.39	AP001059.5	--	chr21	antisense
ENSG00000168765.14	-10.434476	4.41661914	1.89E-82	1.38E-79	7.44	6.91	0	0.01	GSTM4	glutathione S-transferase mu 4	chr1	protein_coding
ENSG00000183426.14	-1.7051383	0.21183713	1.11E-05	5.36E-05	0.73	1.6	0.37	0.57	NPIPA1	nuclear pore complex interacting protein family, member A1	chr16	protein_coding
ENSG00000151208.15	1.63639196	7.26619811	2.04E-08	1.49E-07	8.38	7.96	34.45	33.24	DLG5	discs, large homolog 5 (Drosophila)	chr10	protein_coding
ENSG00000280325.1	-1.3513333	2.227216	1.86E-05	8.57E-05	5.6	6.15	3.61	2.51	AC074183.3	--	chr7	TEC
ENSG00000277324.1	5.37402558	-2.7967694	0.00158008	0.00495521	0	0	0.21	0	RP11-850A1	--	chr18	antisense
ENSG00000049860.12	1.00953315	6.44477228	0.00045522	0.00160336	14.62	17.93	35.59	52.08	HEXB	hexosaminidase B (beta polypeptide)	chr5	protein_coding
ENSG00000121390.16	1.33388276	5.4724939	4.71E-06	2.44E-05	7.04	9.23	22.37	32.44	PSPC1	paraspeckle component 1	chr13	protein_coding
ENSG00000247708.6	-1.1351753	0.13682159	0.00369859	0.01067581	0.1	0.29	0.14	0.1	STX18-AS1	STX18 antisense RNA 1 (head to head)	chr4	antisense
ENSG00000172292.13	1.69906242	2.37243134	1.05E-07	7.03E-07	0.33	0.21	0.92	1.4	CERS6	ceramide synthase 6	chr2	protein_coding
ENSG00000159184.7	1.95039483	4.3357974	1.00E-10	1.00E-09	1.98	1.87	10.05	9.8	HOXB13	homeobox B13	chr17	protein_coding
ENSG00000260401.1	-5.8632571	3.01819579	1.14E-46	1.32E-44	3.76	3.93	0.07	0.11	RP11-800A3	--	chr11	sense_overlapping
ENSG00000116704.7	1.83700644	3.08675733	3.28E-09	2.69E-08	0.51	0.45	1.89	2.72	SLC35D1	solute carrier family 35 (UDP-GlcA/UDP-GalNAc transporter), member D1	chr1	protein_coding
ENSG00000280378.1	1.89397405	-2.0956576	0.02035181	0.04824221	0.03	0.03	0.26	0.08	RP4-758J24.	--	chr1	TEC
ENSG00000175895.3	3.27106925	-0.4300822	1.80E-09	1.52E-08	0	0.06	0.4	0.42	PLEKHF2	pleckstrin homology domain containing, family F (with FYVE domain) member 2	chr8	protein_coding
ENSG00000149313.9	2.272154	2.48204113	3.18E-12	3.79E-11	0.4	0.63	2.48	4.17	AASDHPPT	aminoadipate-semialdehyde dehydrogenase-phosphopantetheinyl transferase	chr11	protein_coding
ENSG00000163687.12	2.2041172	-1.2653566	0.00025593	0.00094973	0.05	0.02	0.25	0.2	DNASE1L3	deoxyribonuclease I-like 3	chr3	protein_coding
ENSG00000095397.12	1.38822953	5.38764598	1.98E-06	1.09E-05	3.52	3.42	12.41	11.76	DFNB31	deafness, autosomal recessive 31	chr9	protein_coding
ENSG00000151025.9	7.73123256	-1.0369036	1.76E-12	2.16E-11	0	0	0.11	0.14	GPR158	G protein-coupled receptor 158	chr10	protein_coding
ENSG00000120662.14	2.04772914	0.13747159	1.03E-06	5.92E-06	0.12	0.12	0.46	0.84	MTRF1	mitochondrial translational release factor 1	chr13	protein_coding
ENSG00000113389.14	-2.9778007	3.93052058	6.50E-21	1.67E-19	2.42	2.61	0.33	0.52	NPR3	natriuretic peptide receptor 3	chr5	protein_coding
ENSG00000166450.11	1.90749812	-0.217663	2.12E-05	9.70E-05	0.01	0.03	0.09	0.13	PRTG	protogenin	chr15	protein_coding
ENSG00000139899.9	-1.9228234	0.95616254	7.40E-08	5.06E-07	0.76	1.16	0.29	0.38	CBLN3	cerebellin 3 precursor	chr14	protein_coding
ENSG00000095383.18	-1.9880866	6.20078512	1.79E-11	1.95E-10	28.27	23.36	8.75	8.6	TBC1D2	TBC1 domain family, member 2	chr9	protein_coding
ENSG00000204370.7	1.81944602	1.26433416	2.41E-07	1.53E-06	0.47	0.25	1.54	1.85	SDHD	succinate dehydrogenase complex, subunit D, integral membrane protein	chr11	protein_coding
ENSG00000259580.1	1.0987507	2.00055834	0.000563	0.00195285	7.57	5.08	16.92	19.35	RP11-37C7.1	--	chr15	processed_transcript
ENSG00000228716.5	1.05893275	5.16653557	0.00026426	0.0009747	4.96	4.64	10.38	16.44	DHFR	dihydrofolate reductase	chr5	protein_coding
ENSG00000111647.11	2.7891025	0.16202964	6.83E-10	6.10E-09	0.03	0.04	0.21	0.49	UHRF1BP1L	UHRF1 binding protein 1-like	chr12	protein_coding
ENSG00000275714.1	1.97000054	-1.8109004	0.00675696	0.01829845	0.2	0.14	1.17	0.66	HIST1H3A	histone cluster 1, H3a	chr6	protein_coding
ENSG00000068878.13	1.78646939	2.89790872	1.08E-08	8.22E-08	0.36	0.4	1.13	2.36	PSME4	proteasome (prosome, macropain) activator subunit 4	chr2	protein_coding
ENSG00000011114.13	1.59605778	2.36961802	4.83E-07	2.93E-06	0.19	0.24	0.85	0.86	BTBD7	BTB (POZ) domain containing 7	chr14	protein_coding
ENSG00000092200.11	6.24952512	-2.2192205	1.55E-05	7.26E-05	0	0	0.05	0.09	RPGRIP1	retinitis pigmentosa GTPase regulator interacting protein 1	chr14	protein_coding
ENSG00000257207.5	4.9050007	-3.0539124	0.01889873	0.04519557	0	0	0.05	0.03	AC108938.5	--	chr2	protein_coding
ENSG00000134775.14	-1.7983856	2.68837616	9.32E-09	7.17E-08	1.49	1.37	0.53	0.57	FHOD3	formin homology 2 domain containing 3	chr18	protein_coding
ENSG00000266469.1	2.92894135	-1.7007436	0.00023185	0.00086708	0.06	0.03	0.22	0.79	CTB-131K11.	--	chr17	antisense
ENSG00000276292.1	5.44525159	-2.7521026	0.00158008	0.00495521	0	0	0.15	1.41	RP11-33N14	--	chr12	antisense
ENSG00000138496.15	1.77621896	2.42911633	2.83E-08	2.04E-07	0.2	0.29	0.95	1.31	PARP9	poly (ADP-ribose) polymerase family, member 9	chr3	protein_coding
ENSG00000128310.2	6.7434818	-1.8548735	2.24E-07	1.42E-06	0	0	0.53	0.24	GALR3	galanin receptor 3	chr22	protein_coding
ENSG00000197555.8	2.10572425	5.17949207	2.22E-12	2.69E-11	1.16	1.41	7.14	7.62	SIPA1L1	signal-induced proliferation-associated 1 like 1	chr14	protein_coding
ENSG00000107651.11	1.54487664	3.48340672	3.32E-07	2.06E-06	0.66	0.62	2.1	2.89	SEC23IP	SEC23 interacting protein	chr10	protein_coding
ENSG00000140006.10	2.2024119	0.3156691	1.16E-07	7.73E-07	0.07	0.14	0.58	0.7	WDR89	WD repeat domain 89	chr14	protein_coding
ENSG00000241631.3	4.87917439	-3.0678699	0.01889873	0.04519557	0	0	0	0.8	RN7SL316P	RNA, 7SL, cytoplasmic 316, pseudogene	chr17	misc_RNA
ENSG00000138385.14	1.98283437	4.48872529	4.69E-11	4.88E-10	2.73	4.79	11.32	28.62	SSB	Sjogren syndrome antigen B (autoantigen La)	chr2	protein_coding
ENSG00000126003.6	1.65859646	4.62786486	2.37E-08	1.72E-07	1.56	1.81	7.33	6.8	PLAGL2	pleiomorphic adenoma gene-like 2	chr20	protein_coding
ENSG00000237187.7	-5.1555393	0.85364685	5.60E-26	2.05E-24	0.42	0.32	0.01	0.02	NR2F1-AS1	NR2F1 antisense RNA 1	chr5	antisense
ENSG00000180592.15	2.92771404	-0.2592367	4.62E-09	3.70E-08	0.02	0.03	0.21	0.22	SKIDA1	SKI/DACH domain containing 1	chr10	protein_coding
ENSG00000257184.3	-5.2081991	-3.0359038	0.00538165	0.01488851	0.17	0.11	0	0	HOXA10-HC	--	chr7	protein_coding
ENSG00000189229.10	-7.963838	-1.0051935	1.13E-13	1.56E-12	0.35	1.07	0	0	AC069277.2	--	chr3	lincRNA
ENSG00000236345.1	-2.8440775	-1.3629167	6.79E-06	3.41E-05	0.29	0.39	0.09	0.04	RP11-59D5_	--	chr6	antisense
