# Supplementary figures and images for: HIF-3α-Induced miR-630 Expression Promotes Cancer Hallmarks in Cervical Cancer Cells by Forming a Positive Feedback Loop
Source: J Immunol Res. 2022 Oct 13;2022:5262963. doi: 10.1155/2022/5262963 (PMC9584697; doi:10.1155/2022/5262963)

A

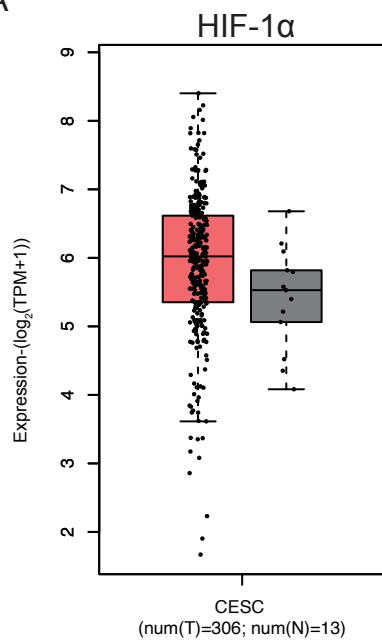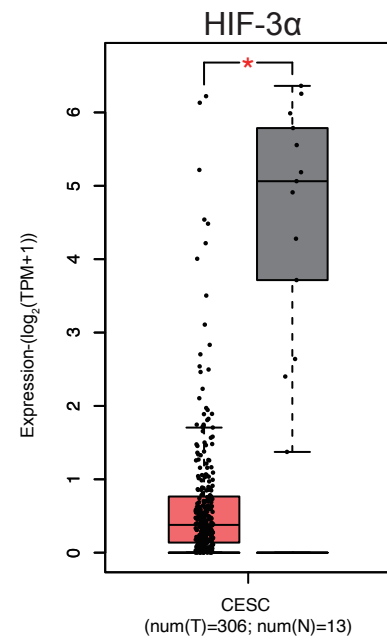

B

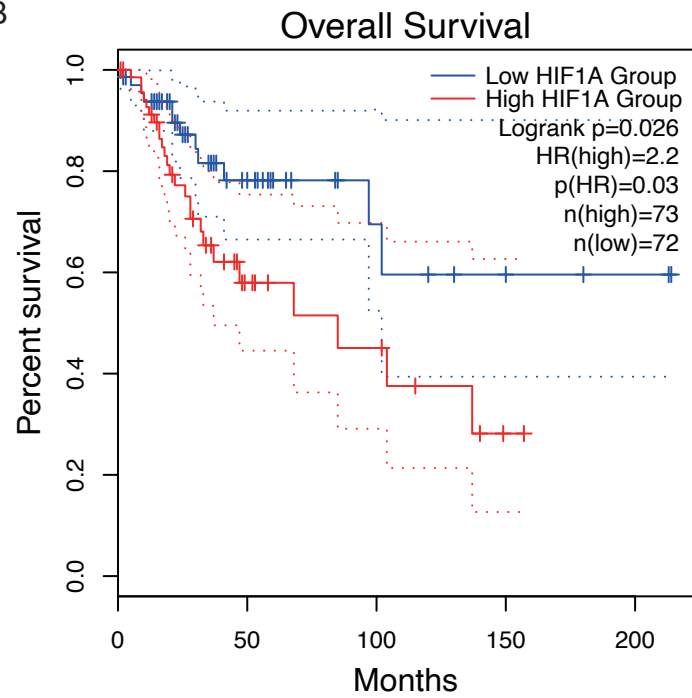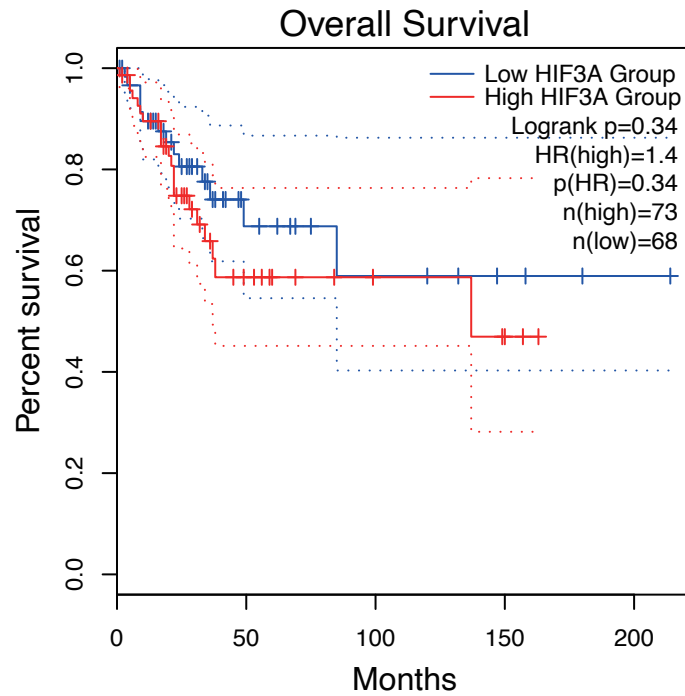

Supplement: Supplementary Materials — Figure S1: the expression level of HIF1A and HIF3A and their related survival time in cervical cancer patients and normal specimens. Figure S2: miR-630 enhances HeLa cell migration and invasion in vitro. Table S1: PCR primer table in this study. Table S2: differentially expressed genes (DEGs) between miR-630 and the control group. [file 5262963.f1.zip › Figure S1.pdf]

A

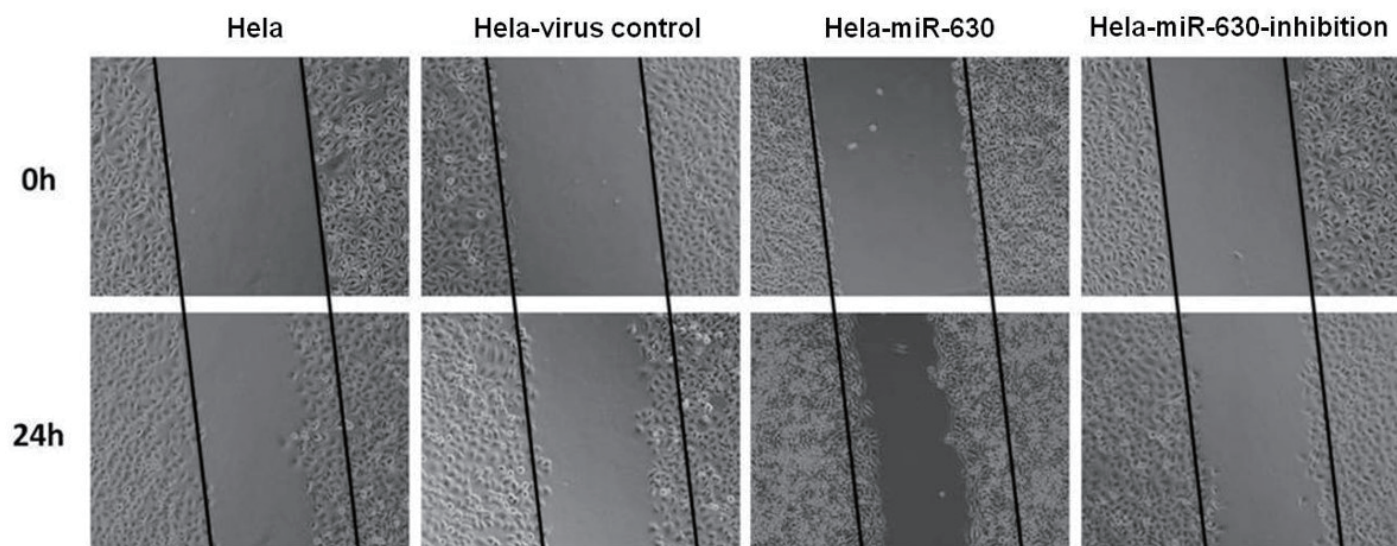

B

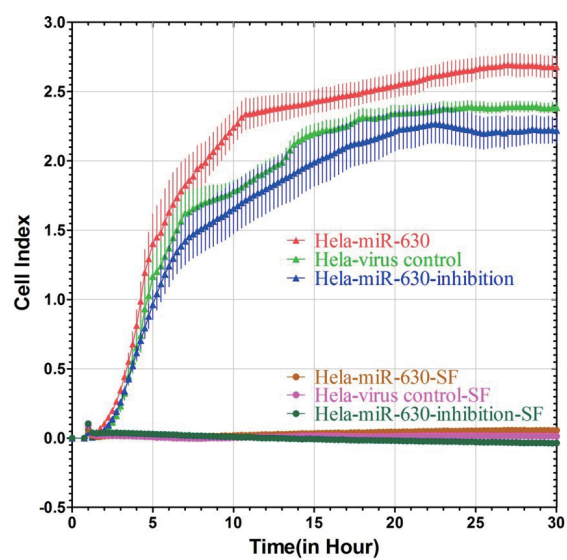

Supplement: Supplementary Materials — Figure S1: the expression level of HIF1A and HIF3A and their related survival time in cervical cancer patients and normal specimens. Figure S2: miR-630 enhances HeLa cell migration and invasion in vitro. Table S1: PCR primer table in this study. Table S2: differentially expressed genes (DEGs) between miR-630 and the control group. [file 5262963.f1.zip › Figure S2.pdf]
